# microRNAs associated with early neural crest development in *Xenopus laevis*

**DOI:** 10.1186/s12864-018-4436-0

**Published:** 2018-01-18

**Authors:** Nicole J. Ward, Darrell Green, Janet Higgins, Tamas Dalmay, Andrea Münsterberg, Simon Moxon, Grant N. Wheeler

**Affiliations:** 1 School of Biological Sciences, University of East Anglia, Norwich Research Park, Norwich, NR4 7TJ UK; 2 Norwich Medical School, University of East Anglia, Norwich Research Park, Norwich, NR4 7TJ UK; 3 Regulatory Genomics, Earlham Institute, Norwich Research Park, Norwich, NR4 7UZ UK

**Keywords:** Xenopus, microRNA, small RNA, Neural crest, Next generation sequencing

## Abstract

**Background:**

The neural crest (NC) is a class of transitory stem cell-like cells unique to vertebrate embryos. NC cells arise within the dorsal neural tube where they undergo an epithelial to mesenchymal transition in order to migrate and differentiate throughout the developing embryo. The derivative cell types give rise to multiple tissues, including the craniofacial skeleton, peripheral nervous system and skin pigment cells. Several well-studied gene regulatory networks underpin NC development, which when disrupted can lead to various neurocristopathies such as craniofrontonasal dysplasia, DiGeorge syndrome and some forms of cancer. Small RNAs, such as microRNAs (miRNAs) are non-coding RNA molecules important in post-transcriptional gene silencing and critical for cellular regulation of gene expression.

**Results:**

To uncover novel small RNAs in NC development we used high definition adapters and next generation sequencing of libraries derived from ectodermal explants of *Xenopus laevis* embryos induced to form neural and NC tissue. Ectodermal and blastula animal pole (blastula) stage tissues were also sequenced. We show that miR-427 is highly abundant in all four tissue types though in an isoform specific manner and we define a set of 11 miRNAs that are enriched in the NC. In addition, we show miR-301a and miR-338 are highly expressed in both the NC and blastula suggesting a role for these miRNAs in maintaining the stem cell-like phenotype of NC cells.

**Conclusion:**

We have characterised the miRNAs expressed in *Xenopus* embryonic explants treated to form ectoderm, neural or NC tissue. This has identified novel tissue specific miRNAs and highlighted differential expression of miR-427 isoforms.

**Electronic supplementary material:**

The online version of this article (10.1186/s12864-018-4436-0) contains supplementary material, which is available to authorized users.

## Background

The neural crest (NC) is a transient and multipotent cell population found exclusively in vertebrate embryos [1]. In the early stages of neurulation where the brain and central nervous system develops, several well-studied gene regulatory networks interact to establish a neural plate border (NPB) between the neural plate and non-neural ectoderm, and overlying the paraxial mesoderm [2]. Once the neural tube closes the NPB is competent to respond to intrinsic and extrinsic molecular cues specifying the generation of NC cells [3–5]. In the early migratory period NC cells lose cell-cell adhesion and undergo cytoskeletal rearrangements allowing them to delaminate and migrate from the neuroepithelium. After settling in diverse and sometimes distant sites in the developing embryo, NC cells differentiate into various cell types, which include melanophores, enteric ganglia, neuroendocrine cells, neurons and craniofacial chondrocytes [2, 3, 6]. This complex and multifaceted sequence of events has been extensively studied and reviewed in many model systems including *Xenopus* [2, 3, 7–9].

The genetics of NC development have been reported extensively, and there are a few transcriptomic studies, however, to date the complement of small RNAs (sRNAs) has not been characterized. sRNAs, 19–33 nucleotides (nt) in length, are a diverse class of non-coding RNA molecules that are key regulators of gene expression. sRNAs such as microRNAs (miRNAs) regulate the expression of >60% of protein coding genes in mammalian genomes via the complementary binding of messenger RNAs (mRNAs) and inhibition of their translation [10, 11]. In *Xenopus,* the expression of many miRNAs have been investigated, however, as of yet no miRNAs have been directly associated with NC development [12, 13]. Previous reports suggest an important role for miRNAs in NC development, with *Wnt1-Cre* mediated knockouts of Dicer, a protein involved in miRNA biogenesis, displaying phenotypes consisting of various NC related abnormalities such as impaired craniofacial development [14–16].

Genome wide identification of sRNAs by library construction prior to next generation sequencing is potentially biased for sequences that can readily anneal to adapters with a fixed sequence. sRNAs which have a lower annealing efficiency are less likely to be ligated to adapters and less likely to be sequenced. To overcome this limitation, we have used high definition (HD) adapters, which contain four degenerate assigned nucleotides at the ligating ends of HiSeq 2500 adapters. These were previously shown to increase the annealing efficiency between sRNAs and adapters [17, 18]. HD adapters were used to profile the sRNA population in ectodermal explants of *Xenopus laevis* embryos induced to form NC and neural tissue, as well as ectoderm and blastula tissues.

## Results

One cell stage embryos were injected with capped RNA for either *Wnt-1* and *Noggin* or *Noggin* alone. This resulted in induction of NC and neural tissue respectively. Animal caps were cut at stage 8 and either immediately flash frozen (blastula tissue) or left to develop until stage 15 (NC, neural and ectodermal tissue). Tissue induction was validated using qPCR and wholemount in situ hybridisation with relevant markers (Additional file 1).

### The sRNA population in NC, neural, ectoderm and blastula is enriched for 23 nt and 29 nt sequences

**Fig. 1:**
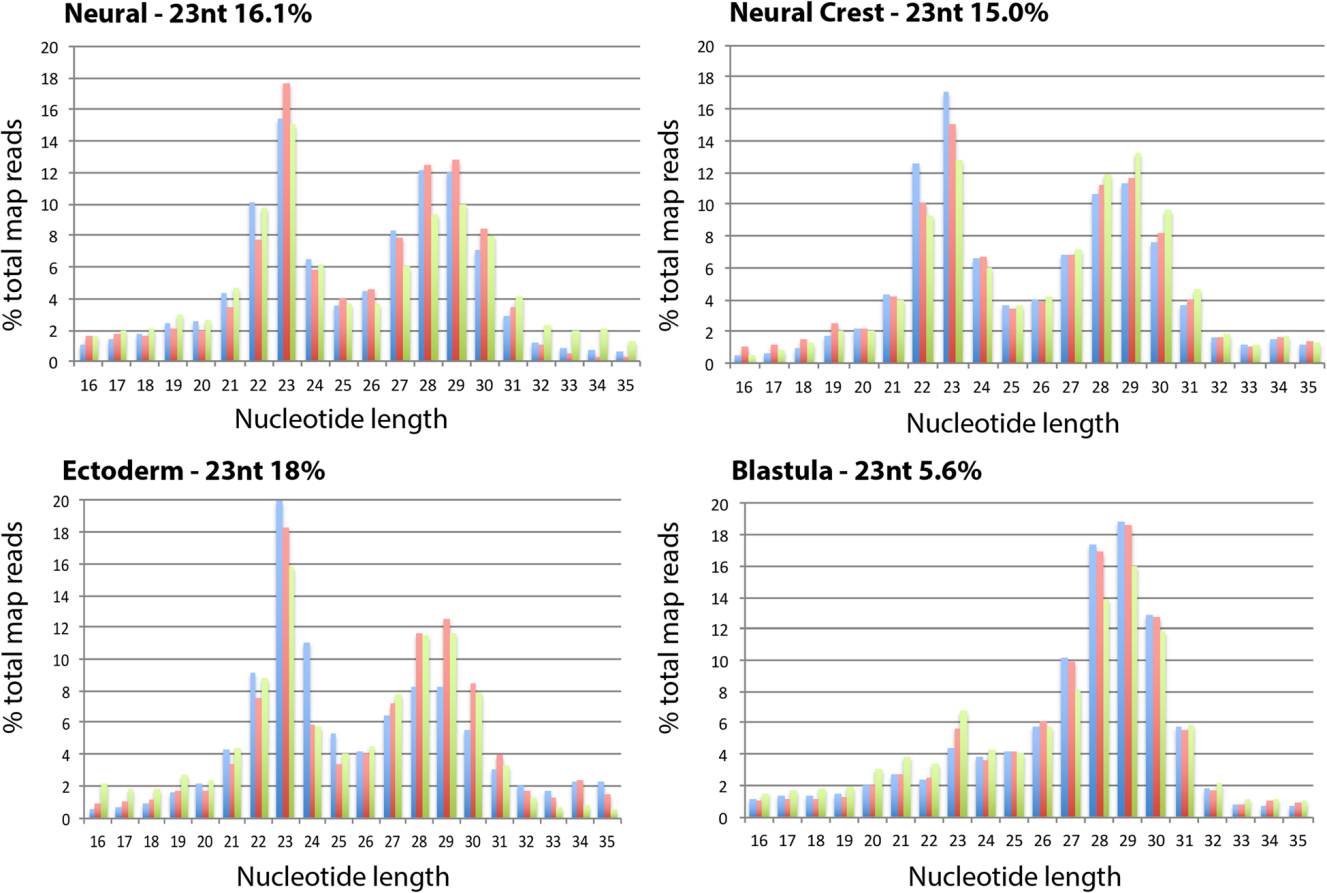
Size class distribution of small RNAs shows a bimodal distribution with peaks at 23 nt (miRNAs) and 29 nt (piRNAs and other sRNAs). In neural tissue 16.1% of reads are miRNA. In NC 15% of reads are miRNA. In ectoderm 18% of reads are miRNA. In blastula 5.6% of reads are miRNA

Sequencing reads matching to the *X. laevis* genome were normalised and revealed a bimodal size class distribution for the redundant reads at 23 nt and 29 nt in NC, neural and ectoderm tissue (Fig. 1). A unimodal size class distribution for the redundant reads was observed in the blastula at 29 nt (Fig. 1). Alignment against available annotations revealed the peak at 23 nt corresponded to miRNAs in NC (15%), neural (16.1%) and ectoderm (18%) confirming the presence of miRNAs (Fig. 1). The literature suggests the peak at 29 nt are piwi-interacting RNAs (piRNAs) [19]. Analysis of the 29 nt peak showed that of the four tissue types blastula had the least number of reads aligning to piRNAs (3.38%) whilst neural had the most at 6% suggesting that the peak observed at 29 nt included other classes of sRNA. Transcriptome analysis of the 29 nt peak showed that these sRNAs are not products of mRNA degradation (Additional file 2). In addition, no significant enrichment of rRNA or tRNA fragments in the blastula samples was observed. In total 525 mature miRNAs were identified (Additional file 3). Line plots showing their relative expression in the four tissue types are shown in Additional file 4. Sequences of all the hairpins identified and alignments of sequences to hairpins are shown in Additional files 5 and 6. 106 previously described miRNA families derived from 388 hairpins were expressed. Of these we identified 15 miRNAs yet to be annotated in the *Xenopus* genome (Additional file 7). In addition, we identified 102 novel miRNAs from 137 hairpins (Additional file 8). Four miRNAs were chosen to validate and confirm the sequencing data by qPCR (Additional file 9).

### Five isoforms of miR-427 are highly expressed in all four tissue types

**Fig. 2:**
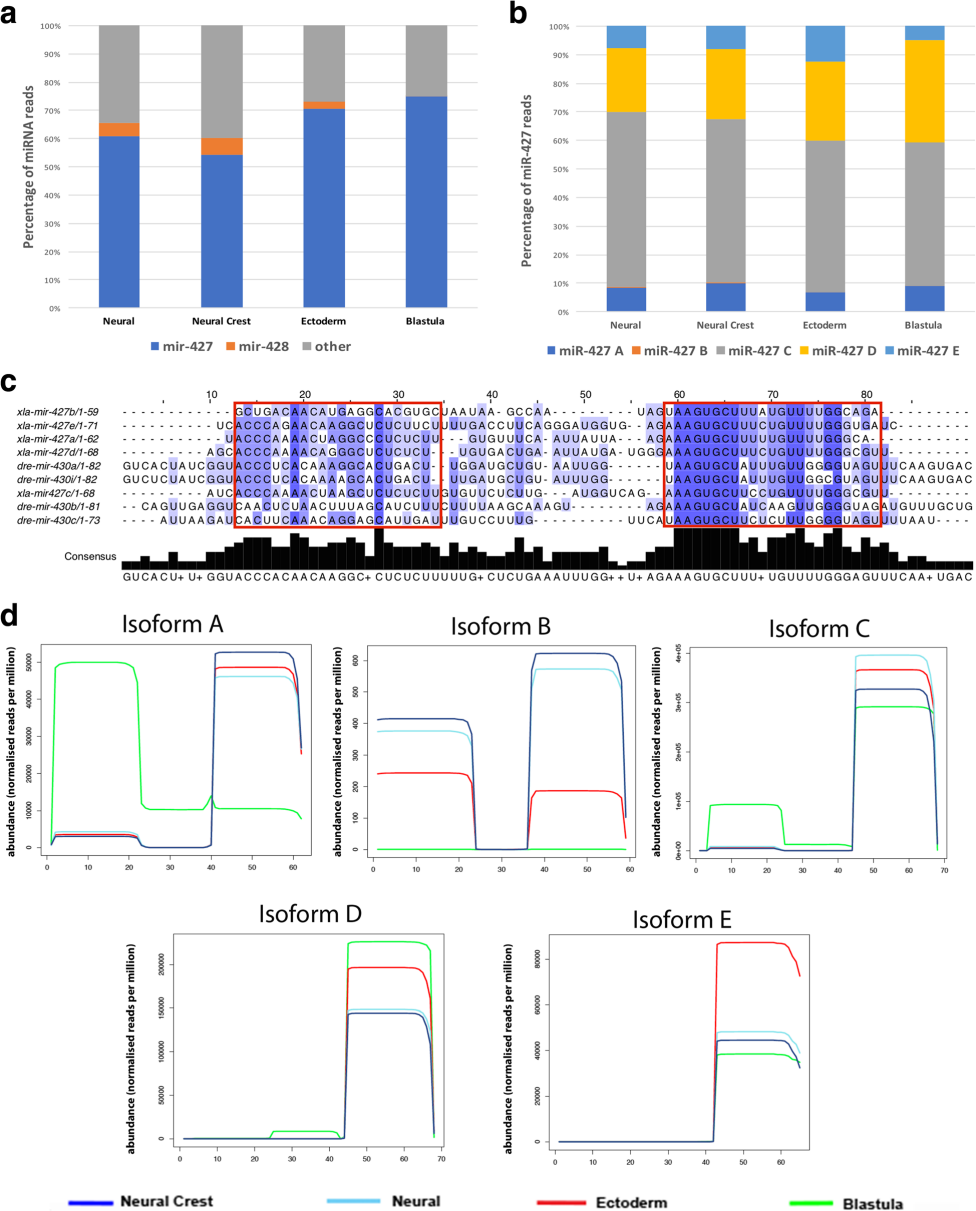
Analysis of miR-427 compared to other miRNAs across all tissue types shows miR-427 is the primary miRNA expressed. **a** miR-427 constitutes 60–75% of all miRNA reads in all four tissue types with miR-428 the second most prevalent miRNA in NC, neural and ectoderm only **b** Five isoforms of miR-427 were expressed all of which showed a similar hairpin distribution over the four tissue types with isoform C the most abundant **c** Alignments of the five isoforms of *Xenopus* miR-427 and Zebrafish miR-430 hairpins. The mature 5′ and 3′ sequences are indicated with red boxes. The 3′ strand has strong conservation whilst the 5′ strand does not **d** Abundance plots show the isoforms of miR-427 are largely derived from changes in the 5′ end of the miRNA except in the blastula where a miR-427 isoform is generated due to changes in the 3′ end of the miRNA

Annotation of the miRNAs across the four tissue types showed miR-427 constituted 67% of all miRNA sequences except in blastula where miR-427 constituted 74% of miRNA sequences (Fig. 2). miR-427 has been reported previously in early embryo development, specifically during the mid-blastula transition (MBT) where the 3′ mature strand has been shown to play a key role in the deadenylation and destabilisation of maternal transcripts [12, 20]. Five isoforms of miR-427 were annotated across the four samples. Analysis of the isoforms reveals that although the hairpins have a similar distribution across the tissue types the expression of mature 3′ and 5′ sequences differ greatly (Fig. 2b-d). Figure 2c shows that the 3′ strand of the isoforms have strong homology in their seed sequence both in *Xenopus* and Zebrafish. The 5′ strands show less homology. Isoform A has a clear tissue dependent switch between the 3′ and 5′ mature strand. The 5′ strand is abundant in blastula tissue and absent in neural, NC and ectoderm. The opposite is seen for the 3′ strand. Isoform C 5′ mature strand is also specifically abundant in the blastula tissue. Conversely, according to read number the most abundant 3′ mature strands in the blastula tissue were from isoform C and D (Fig. 2).

### MiRNA expression profile in NC, neural, ectoderm and blastula

**Fig. 3:**
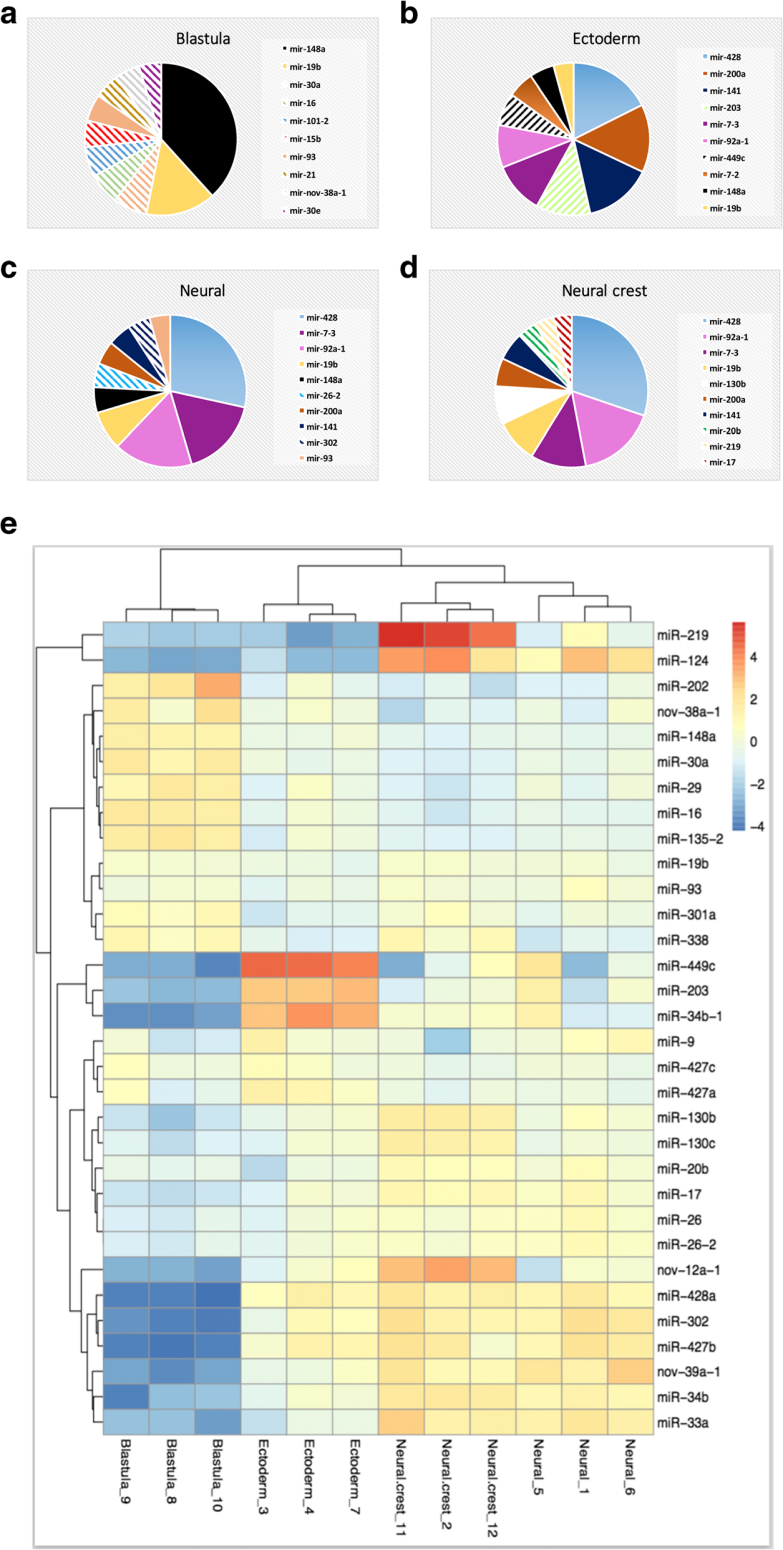
miRNA expression profiling shows a number of miRNAs are unique to each tissue type. Pie chart analysis shows the distribution of the top 10 most abundant miRNAs in each tissue type **a** Top 10 blastula miRNA **b** Top 10 ectoderm miRNAs **c** Top 10 neural miRNAs **d** Top 10 NC miRNAs **e** Heatmap of a selection of miRNAs

For each of the four tissue types we created a profile of the top 10 most highly expressed miRNAs (excluding miR-427) (Fig. 3a-d). We found that miR-7, miR-19b, miR-92a, miR-141, miR-200a and miR-428 were expressed in NC, neural and ectoderm tissue (Fig. 3b-d). miR-219 and miR-130b were detected specifically in NC (Fig. 3d) and miR-26, miR-93 and miR-302 were expressed specifically in neural tissue (Fig. 3c). Finally, miR-203 and miR-449 were shown to be expressed specifically in ectoderm (Fig. 3b).

There was a relatively low level of miRNA expression in the blastula when compared to the other tissue types. Of the miRNAs that were expressed in the blastula samples we identified a novel miRNA, miR-nov-38a-1. We observed that miR-428 was the second most prevalent miRNA in NC, neural and ectoderm tissue but was not expressed in blastula. The second most abundant miRNA in the blastula after miR-427 was miR-148a. Also highly expressed in the blastula were miR-16, miR-30a and miR-101 (Fig. 3a). Figure 3e shows a heatmap representation of the expression of these and other miRNAs averaged over the 3 biological replicates for each animal cap tissue type tested.

### Several miRNAs are unique to NC

**Fig. 4:**
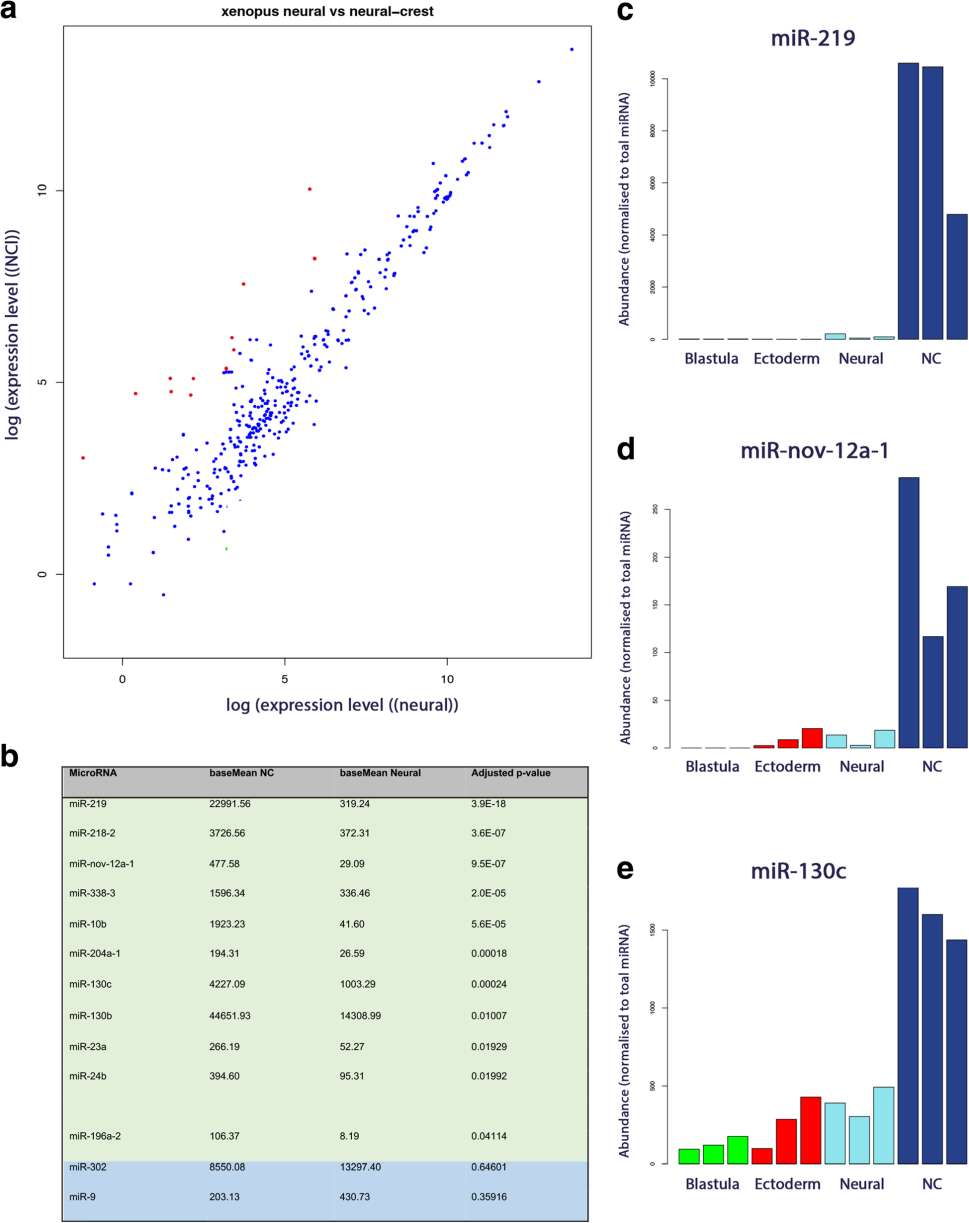
A number of miRNAs are upregulated in NC when compared to neural tissue. **a** Annotation shows 11 miRNAs (in green) are upregulated in NC when compared to neural tissue. Conversely 2 miRNAs are upregulated in neural tissue when compared to NC (in blue) **b** Scatter plot using DeSEQ2 shows a total number of miRNAs differentially expressed between NC and neural tissue (upregulated in NC in red, upregulated in neural in green) **c** Expression of miR-219 is highly expressed in NC with a low expression in neural tissue **d** Expression of a novel miRNA, miR-nov-12a-a, is highly expressed in NC with a low expression in neural and ectoderm tissue **e** Expression of 130c is highly expressed in NC with a lower expression in neural, ectoderm and blastula tissue. **c-e** were performed in triplicate for each tissue type

To identify miRNAs exclusively located in the NC differential expression analysis was performed on NC versus neural tissue. We observed 11 miRNAs that were significantly more highly expressed in the NC tissue compared to all other tissue types (Fig. 4a, b), this included miR-130b/c, miR-17, miR-20b, miR-196a, miR-10b and miR-219 (Fig. 4c, e). A novel miRNA, miR-nov-12a-1, was also highly expressed in NC (Fig. 4d). miR-9 and miR-302 were more highly expressed in the neural tissue when compared to NC (Fig. 4b).

### MiR-301a and miR-338 are expressed in NC and blastula tissue

**Fig. 5:**
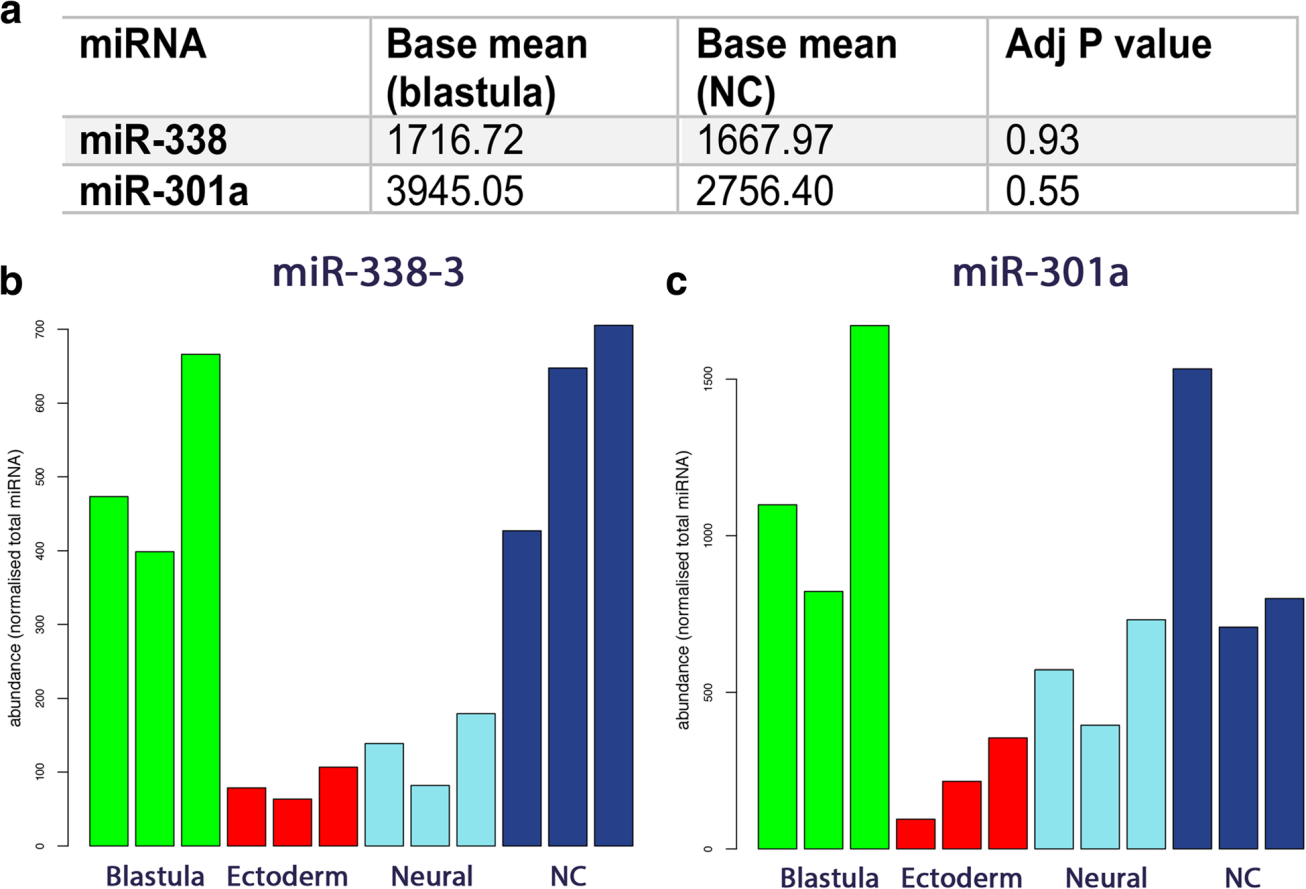
miR-301a and miR-338 are expressed in NC and blastula tissue suggesting a role for these miRNAs in maintaining multipotency of NC cells. **a** Mean read counts of miR-301a and miR-338 in NC and blastula **b** Enrichment of miR-338 across the four tissue types shows a high expression in NC and blastula **c** Enrichment of miR-301a across the four tissue types shows a high expression in NC and blastula

Based on work in *X. laevis*, the steps through which NC obtain their multipotency have recently been revised. Evidence now suggests that NC cells retain multipotency during their early development from the blastula stage, as opposed to re-acquiring multipotency as was previously believed [4, 21]. The exact mechanisms enabling NC to maintain their potency to generate multiple cell types are yet to be elucidated. To investigate possible miRNAs contributing to NC multipotency, we identified miRNAs enriched in both NC and blastula. We found that miR-301a and miR-338 had a high expression in both tissue types when compared to neural and ectoderm tissue (Fig. 5).

## Discussion

Molecular and computational analysis has been used to identify a set of miRNAs that are expressed in animal cap induced NC, neural, ectoderm and blastula tissue. We observed expression of another class of sRNA at 29 nt, which is consistent with previous data reporting this peak to be piRNAs [22]. piRNAs are not processed by Dicer during their biogenesis and are largely known for their roles in epigenetic silencing and targeting of transposable elements in germ cells [23]. ProTRAC analysis revealed the peak at 29 nt were not piRNAs with only a fraction of reads aligning to known piRNA genes (Additional file 2). We hypothesised that these may be products of RNA degradation as the embryo underwent mid blastula transition (MBT) in which maternal RNA products are broken down in preparation for the initiation of zygotic transcription [24]. We performed transcriptome analysis to investigate whether the peak at 29 nt was a consequence of degraded maternal transcripts. This analysis concluded that there is no significant increase in the abundance of degradation fragments from the *X. laevis* transcriptome in the blastula samples. A recent study described a new class of small RNAs in *Xenopus laevis* known as siteRNAs. These are remnants of transposable elements, which align in clusters to the introns of protein coding genes [25]. We found no enrichment of 29 nt sequences within introns in the blastula samples suggesting that the peak at 29 nt is not due to enrichment of siteRNAs. The simplest explanation for the 29 nt peak in the blastula samples is due to lower miRNA expression levels. This will have the effect of inflating the proportion of other, non-miRNA sequences therefore leading to a larger peak centred around 29 nt. This is a general problem with sequencing as it is only possible to compare proportions of reads rather than actual quantities without using synthetic spike-ins for normalisation [25].

### miR-427 is highly abundant in all tissue types, with five isoforms expressed in a tissue-dependent manner

One of the identified miRNAs, miR-427, was highly abundant in all four tissue types through the expression of its five isoforms. The high expression levels of miR-427 has been reported previously to be a result of the presence of hundreds of copies of a − 1.2 kb DNA repeat sequence containing all isoforms in the *X. laevis* genome [26]. MiR-427 expression has been reported previously in early embryo development [12, 26], specifically during MBT where the 3′ mature sequence has been shown to play a key role in the deadenylation and destabilisation of maternal transcripts [26, 27]. miR-427-3p has orthologues in both zebrafish and humans, termed miR-430 and miR-302 respectively [27, 28]. The seed sequence of the 3’ miRNA is conserved between frog miR-427, zebrafish miR-430 and human miR-302 [27, 29, 30] but the conservation of the 5’ miRNA is divergent between species and between different miR-427 isoforms in *Xenopus* (Fig. 2c). Our results show significant tissue specific differences in the expression of 5′ and 3′ miRNAs from different miR-427 isoforms. Specifically, we see high levels of expression of the 5′ miRNA of miR-427 isoform A and C in the blastula samples but almost no expression in the other tissues profiled (Fig. 2d). As the seed sequence of these blastula specific 5′ miRNAs is markedly different from that of the highly conserved 3’ miRNA it is clear that they have the potential to target a completely different set of transcripts. This leads to the possibility that miR-427-5p isoform A and C may have a specific function during early development that is not required in differentiated tissue. miR-427 has always been reported to play a role in early development, what it may be doing in later stages on development is unclear.

### miRNA diversity across NC, neural and ectoderm tissues is limited

We next compared the miRNA profiles of each tissue type. We discovered that miRNA diversity over the NC, neural and ectoderm tissues is remarkably limited. A few miRNAs were found to be abundantly expressed in these tissues (Fig. 3). The contribution of the top 10 miRNAs were found to make up 80% of the total miRNA reads observed in each tissue. This finding is in line with previous reports in that a small number of highly abundant miRNAs are dominant during *X. laevis* embryonic development [31]. After miR-427 the second most abundant miRNA in NC, neural and ectoderm was miR-428 (Fig. 3a). MiR-428 is specific to *X. laevis* and although its 5′ sequence has some similarity to mammalian miR-20 and miR-302 the function of miR-428 is yet to be elucidated [12].

### Tissue specific miRNAs were identified in the NC, neural and ectoderm animal caps

Despite the low complexity of miRNA expression, we identified several tissue specific miRNAs. Some of these tissue specific miRNAs were present at high abundance. We found miR-17, miR-20b, miR-130b and miR-219 were expressed in NC tissue (Fig. 3d and e). To our knowledge none of these miRNAs have previously been reported to be associated with NC development. Microarray analysis has shown that miR-17 and miR-20b are *Sox10* dependent miRNAs [32]. In addition, it has been shown that miR-17 is regulated by c-Myc, an essential regulator of NC development [33–35]. We found miR-93 and miR-302 are highly expressed in neural tissue (Fig. 3c and e). Previous in situ experiments have shown that miR-302 is highly expressed in early neuroepithelium and that mouse knockouts of miR-302 result in embryonic lethality characterised by an open neural tube defect [36, 37]. We also found miR-203 and miR-449 are expressed in the ectoderm (Fig. 3b and e). Both miR-203 and miR-449 have been implicated in epidermal development through targeting of the transcription factor p63, a p53 family member which is known to be critical in the development of stratifying epithelia in both human and mouse [38–40]. MiR-449 has been shown to play a vital role in cilliogenesis of epithelial tissue in both frogs and mice [41].

### The miRNA expression profile of the blastula animal pole is more distinct

While NC, neural and ectoderm tissue show some similarities in their miRNA expression profiles, blastula tissue is more distinct. Unlike the other tissue types, miR-428 is not one of the 10 most abundant miRNAs. The most abundant miRNA in the blastula after miR-427 was miR-148a (Fig. 3a and e). Also highly expressed in the blastula were miR-16, miR-30a and miR-101 (Fig. 3a and e). Previous data demonstrates that both miR-101 and miR-148a are abundant in frog oocytes so it is possible that expression of these two miRNAs is carried over from the oocyte [19]. Through targeting of Nodal receptors, miR-16 has been shown to be key in the development of Spemann’s organiser – a cluster of cells in the dorsal blastula that controls dorsal ventral patterning of the amphibian embryo [42]. It was therefore anticipated that this miRNA would be highly expressed in the blastula. A novel miRNA, miR-nov-38a-1, was present in blastula making up 2.5% of total miRNA reads (Fig. 3), such large read numbers indicate that miR-nov-38a-1 is likely to play a key role in early development.

### 11 miRNAs not previously associated with NC were upregulated in the NC tissue

The focus of this study was NC and we observed 11 miRNAs upregulated in the NC when compared to neural tissue (Fig. 4a, b). Of these the most abundant miRNA was miR-219 (Fig. 4c). Although there is no direct link between NC development and miR-219 in the literature, miR-219 has NC related targets. One of these targets is platelet derived growth factor receptor A (PDGFRα) [43], a receptor for the ligand platelet derived growth factor (PDGF), a marker for migrating NC cells [44]. Once bound the PDGFs exert their function by causing dimerisation and activation of the PDGFRα receptors, which activates a multitude of intracellular signalling cascades. The outcomes of these signalling events are diverse and include proliferation, migration, matrix deposition, survival and EMT [45]. PDGFRα has been demonstrated to be vital for initiation of NC cell migration through upregulation of matrix metalloproteinase 2 (MMP2) for matrix degradation prior to migration, and inhibition of apoptosis [46, 47]. Loss of function experiments for PDGFRα result in palatal defects in zebrafish, mice and humans [48–50]. It is possible that miR-219 functions in modulating PDGFRα expression in pre-migratory NC cells to prevent premature migration. In addition, our own target prediction using miRSystem [51] indicate that miR-219 targets multiple members of the Pax, Six, Eya, Dach (PSED) gene regulatory network. Multiple members of this network have been demonstrated to play roles in both NPB formation and NC development [52, 53].

**Fig. 6:**
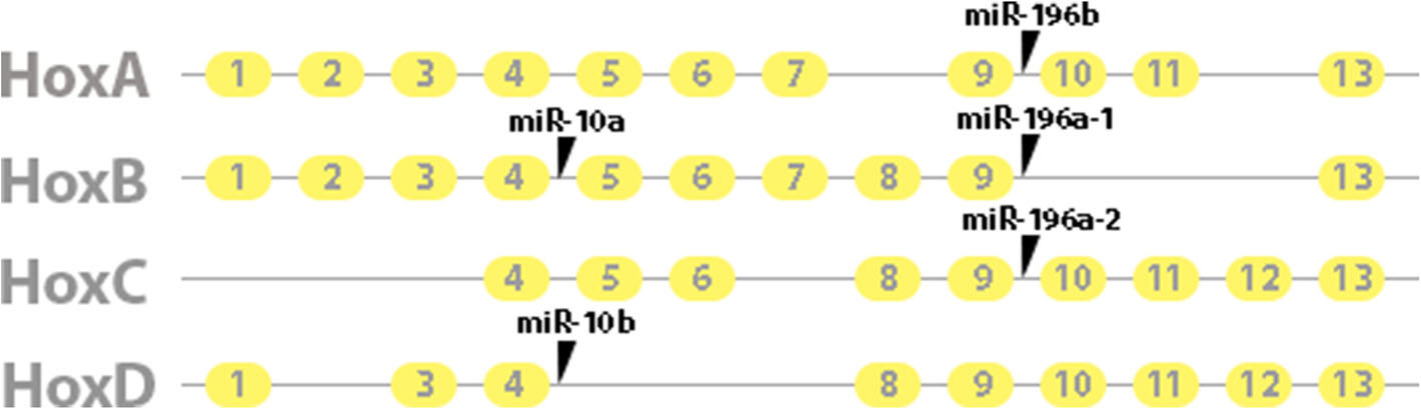
Schematic of the structure of the four mammalian HOX gene clusters, exons numbered in yellow circles, with the location of miR-10a, miR-10b, miR-196a-1, miR-196a-2 and miR-196b located in the introns (grey lines) (Data taken from [54])

Two of the miRNAs highly expressed in NC include miR-10b and miR-196a. Both of these miRNAs are located within a Hox cluster (Fig. 6) [54]. Both miR-10b and miR-196a have been shown to target multiple Hox genes some of which are implicated in NC development [55]. MiR-10b has been shown previously to target histone deacetylase 4 (HDAC4) [13]. HDAC4 is required for the generation of anterior facial structures in zebrafish by modulating the migration of cranial NC cells [56]. HDAC4 is also a key gene in successful embryonic bone development [57]. MiR-10b has also been shown to modulate the transforming growth factor beta (TGFβ) pathway by directly targeting TGFβ [58]. TGFβ ligands and their signalling intermediates have significant roles in embryonic patterning and specification of cranial NC cells [59].

MiR-nov-12a was the only novel miRNA to appear within the top 10 differentially expressed miRNAs in NC (Fig. 4a, b). Although the sequencing read numbers were low it is possible that this miRNA is playing a role in NC development. Two miRNAs that were upregulated in neural tissue when compared to NC were miR-9 and miR-302 (Fig. 4b). Both of these miRNAs have previously been implicated in neural development [60, 61].

### miR-301a and miR-338 are highly expressed in both NC and blastula animal pole suggesting a possible role for these miRNAs in the maintenance of NC pluripotency

Until recently, the model of NC development was controversial. Unlike the typical differentiation pathway of embryonic cell populations, NC were thought of as unique as they could selectively regain potency to increase their differentiation ability from ectodermal tissues to mesoderm (unlike their cellular neighbours). This seemed to contradict the model of Waddington that cells in the embryo are progressively restricted in their developmental potential [62]. In 2015, Buitrago-Delgado and colleagues published a paper that changed this working model of NC so it no longer defied the paradigm of progressive restriction in potential. This work provided evidence that instead of regaining pluripotency, NC cells selectively inherit it from the embryonic stem cells from which they are derived (blastula tissue) [21]. In order to identify potential miRNAs regulating the differentiation potency of NC cells we searched for miRNAs that were expressed in both NC and blastula. We found miR-301a and miR-338 were expressed in both tissues (Fig. 5). The literature confirms that both of these miRNAs have roles in maintaining pluripotency in the embryo [63–65]. MiR-301a has a strong link to the maintenance of pluripotency as it has been shown to contribute in a positive feedback loop essential for human pluripotent stem cell (hPSC) self-renewal and reprogramming [66]. This previous study demonstrated that miR-301a inhibits the translation of both serine/arginine-rich splicing factor 2 (SFRS2) and methyl-CpG-binding domain protein 2 (MBD2). SFRS2 is a splicing factor targeted by octamer-binding transcription factor 4 (OCT4) and is required for pluripotency. SFRS2 regulates alternative splicing of MBD2 whose isoforms play opposing roles in maintenance of and reprogramming to pluripotency. The study concluded that the miR-301 family independently regulates SFRS2 and MBD2 to “fine tune” the expression of MBD2 isoforms in favour of self-renewal [66].

MiR-338 is an intronic miRNA co-transcribed with a host gene encoding an apoptosis-associated tyrosine kinase [67]. It has been shown that miR-338 is regulated by *Sox10* via the direct regulation of its host gene therefore providing a link between miR-338 and NC development [32]. MiR-338 has also been shown in multiple biological contexts to be a negative regulator of cell differentiation. For example, miR-338 can inhibit the expression of osteoblast differentiation markers such as osterix (*Osx*), thus reducing osteoblast differentiation [65]. Finally, miR-338 is known to be upregulated by c-Myc in embryonic stem cells [63]. Myc proteins are known to have an important function in stem cell maintenance and c-Myc has previously been demonstrated to be key in early NC development [33, 34, 68].

## Conclusions

In conclusion, to determine sRNA expression and uncover novel small RNAs in NC development we used high definition adapters prior to next generation sequencing in four tissue types (induced NC and neural tissue, ectoderm and animal pole blastula) of *Xenopus laevis* embryos. We have shown that miR-427 is highly expressed in all four tissue types; five different isoforms or isomiRs exist and are differentially expressed, depending on the tissue type. We found a set of 11 miRNAs that are expressed explicitly in the NC, most of which were not previously linked to NC development and one of which is a novel miRNA. We report miR-301a and miR-338 are highly expressed in both the NC and blastula indicating a possible role for these miRNAs in maintaining the stem cell-like phenotype of NC cells.

## Methods

### Microinjection and microsurgery

All experiments were performed in compliance with the relevant laws and institutional guidelines at the University of East Anglia. The research was approved by the the UEA Animal Welfare and Ethical Review Body (AWERB) according to UK Home Office regulations. *X. laevis e*mbryos were obtained and prepared for microinjection as previously described [34]. Embryos were staged using the Nieuwkoop and Faber normal table of *X. laevis* development (Nieuwkoop, 1994). Capped RNA was synthesised using the Ambion mMessage mMachine in vitro transcription kit for *Wnt1* and *Noggin* (Thermo Fisher Scientific). Embryos were injected at the 2-cell stage and animal caps (approximately 80 per sample) were isolated manually when the embryos reached stage 8. Isolated animal caps were maintained in 1X MMR. Animal caps were left to develop until stage 15 as judged by sister controls (NC, neural and ectoderm) before snap freezing in liquid nitrogen.

### Next generation sequencing

Total RNA was extracted using the miRCURY RNA isolation kit (Exiqon) according to manufacturer’s instructions. RNA concentration and integrity was measured on the NanoDrop 8000 Spectrophotometer (Thermo Fisher Scientific). All 12 libraries (three replicates of four tissue types) were constructed using 1.5 μg of RNA which was ligated to 3′ and 5’ HD adapters [18]. Ligated RNA products were reverse transcribed to cDNA and amplified by qPCR. The cDNA products were selected and purified by 8% polyacrylamide gel electrophoresis and ethanol precipitation. Libraries were sequenced at the Earlham Institute, Norwich Research Park, on the HiSeq 2500 Ultra High Throughput Sequencing System (Illumina). The data was uploaded to NCBI accession PRJNA429716.

### Bioinformatics analysis

Fastq files were converted to fasta format. The 3′ adapter was trimmed using perfect sequence match to the first 8 nucleotides of the 3’ HiSeq 2500 adapter (TGGAATTC). The HD signatures (four assigned nucleotides at the ligating ends) of the reads were also trimmed [69]. Reads consisting 16–35 nt were kept for further analysis. sRNAs were mapped full length with no gaps or mismatches allowed to the *X. laevis* genome (v8) using PatMaN [70]. The latest set of *X. laevis* miRNAs were downloaded from miRBase (v21) [71] but as only 22 precursors we present in the annotation we conducted whole genome annotation of miRNAs. All animal miRNA precursors in miRBase were searched using BLASTN (E-value <1e-06) against the *Xenopus laevis* genome assembly 8.0 (removing any hits which have > = 30 blast hits or hairpin length < = 55). MiRNAs with a low abundance (<100 sequences mapping to the hairpin across all the samples) and miRNAs which did not have a typical hairpin structure, were removed from subsequent analyses (all hairpins were retained for a family if at least one member of the family had alignments).

For differential expression analysis read counts were obtained for all reads aligning to miRNA hairpins in each sample. These counts were used as input for DESeq2_1.8.2. We considered padj < 0.01 to call differential expression. Novel miRNAs were predicted using miRCat and miRdeep2 [72–75]. The intersect was taken for prediction from both methods and the longest hairpin sequence obtained. The hairpin secondary structure was obtained using RNAFold [76]. Each hairpin was checked manually and 137 hairpins which clustered into 70 groups (102 unique mature miRNAs) were selected which met our criterion, these were designated as novel *X. laevis* miRNAs. piRNA clustering and analysis we used probabilistic tracking and analysis of clusters (ProTRAC) [77].

### qPCR

Total RNA was quantified by agarose gel electrophoresis, density measurement and nanodrop and 10 ng of RNA was reverse transcribed to cDNA using the universal cDNA synthesis kit (Exiqon, Vedbæk, Denmark). cDNA was diluted 1:40. qPCR reactions were set up in triplicates with three biological replicates using the ABI one step detection system and SYBR green master mix (Exiqon) following the manufacturer’s instructions. The mature miRNA sequence used to synthesise custom miRNA locked nucleic acid (LNA) qPCR primer sets (Exiqon) were:

- Xtr-miR-196a 5’-UAGGUAGUUUCAUGUUGUUGG-3′,
- Ipu-miR-219a 5’-AGAAUUGUGCCUGGACAUCUGU-3′,
- Xtr-miR-302 5’-UAAGUGCUCCAAUGUUUUAGUGG-3′
- Dps-miR-219 5’-UGAUUGUCCAAACGCAAUUCUUG-3′.

U6 was used for normalisation.

### Statistical analysis

Evaluation of variability between sequencing libraries was conducted using scatter plots, size-split boxplots of the replicate-to-replicate differential expression, intersection and Jaccard similarity analyses [78]. The empirical differential expression analysis was confirmed by parametric (t-tests) and non-parametric (Mann-Whiney-U) tests. For the statistical tests we considered *p* < 0.05 as statistically significant. Bioinformatics analysis was conducted using custom-made Perl (5.24.0.1) and R (3.2.2) scripts. QPCR data is shown as mean ± SEM from three independent experiments.

## Additional files

Additional file 1: Figure S1. Validation of tissue induction (A) PCR on RNA extracted from stage 15 animal cap tissue induced to become either neural or NC showed that tissue was induced efficiently. The NC marker Snail2 was only expressed in the NC animal cap tissue whilst the neural marker Sox2 was enriched in the neural tissue and epidermal keratin was enriched in the ectoderm (Ecto) sample. Histone H4 was used as a positive control and Bracyury for a control of mesoderm contamination Whole embryos (WE) were used a positive controls for all genes. (B) WISH for the NC marker Sox10 on stage 15 induced animal caps. This is further confirmation of induction of NC tissue as expression is only evident in NC animal caps. (DOCX 7118 kb)

Additional file 2: Table S1. piRNA clustering and transcriptome analysis of the sRNA sequences. piRNA clustering and transcriptome analysis of the sRNA sequences using ProTRAC revealed that the 29 nt peak observed in blastula only contains a fraction of piRNAs (3.38%). Transcriptome analysis shows these are not degraded transcripts derived from mid blastula transition. The peak at 29 nt therefore contains an unidentified class of sRNA. (DOCX 92 kb)

Additional file 3 Table S2. Mature sequences for all miRNAs. (XLSX 59 kb)

Additional file 4: Figure S2. Line plots of miRNA expression levels across the different tissue types. (PDF 1374 kb)

Additional file 5: Figure S3. Hairpin sequences of all miRNAs. (TXT 67 kb)

Additional file 6: Figure S4. Alignment files of small RNAs to the hairpins. (TXT 1995 kb)

Additional file 7: Table S3. List of sequenced miRNAs that were not previously annotated in *Xenopus laevis*. (DOCX 48 kb)

Additional file 8: Figure S5. Hairpin structures of all novel miRNAs. (PDF 248 kb)

Additional file 9: Figure S6. qPCR validation of small RNA sequencing. NC miRNAs identified by sRNA sequencing were validated using qPCR. The same RNA was used to make both the sRNA libraries and for qPCR. (A) Abundance plots of miRNAs; miR-219, miR-196a, miR-302 and nov-12a-1 following sRNA sequencing on blastula and ectoderm animal cap tissue and animal caps induced to form NC and neural tissue (B) qPCR validation of the miRNAs identified from the sRNA sequencing in the same order as A. One way ANOVA with Tukey post-test statistical analyses were performed on the results of each qPCR. For significance, we considered *P* > 0.01*; *P* > 0.001**; *P* > 0.0001*** and *P* > 0.0001****. (DOCX 186 kb)

## Abbreviations

EMT: Epithelial mesenchymal transition
HD: High definition
HDAC4: Histone deacetylase 4
hPSC: Human pluripotent stem cells
LNA: Locked nucleic acid
MBT: Mid blastula transition
miRNA: micro RNA
mRNA: Messenger RNA
NC: Neural crest
nt: Nucleotide
PDGFRa: Platelet derived growth factor A
PiRNA: PIWI-interacting RNA
qPCR: Quantitative polymerase chain reaction
sRNA: small RNAs
TGFb: Transforming growth factor beta
*X.laevis*: *Xenopus laevis*
