# Supplementary material for: microRNAs associated with early neural crest development in Xenopus laevis
Source: BMC Genomics. 2018 Jan 18;19:59. doi: 10.1186/s12864-018-4436-0 (PMC5774138; doi:10.1186/s12864-018-4436-0)
Supplement: Supplementary file 1 — Validation of tissue induction (A) PCR on RNA extracted from stage 15 animal cap tissue induced to become either neural or NC showed that tissue was induced efficiently. The NC marker Snail2 was only expressed in the NC animal cap tissue whilst the neural marker Sox2 was enriched in the neural tissue and epidermal keratin was enriched in the ectoderm (Ecto) sample. Histone H4 was used as a positive control and Bracyury for a control of mesoderm contamination Whole embryos (WE) were used a positive controls for all genes. (B) WISH for the NC marker Sox10 on stage 15 induced animal caps. This is further confirmation of induction of NC tissue as expression is only evident in NC animal caps. (DOCX 7118 kb) [file 12864_2018_4436_MOESM1_ESM.docx]

**
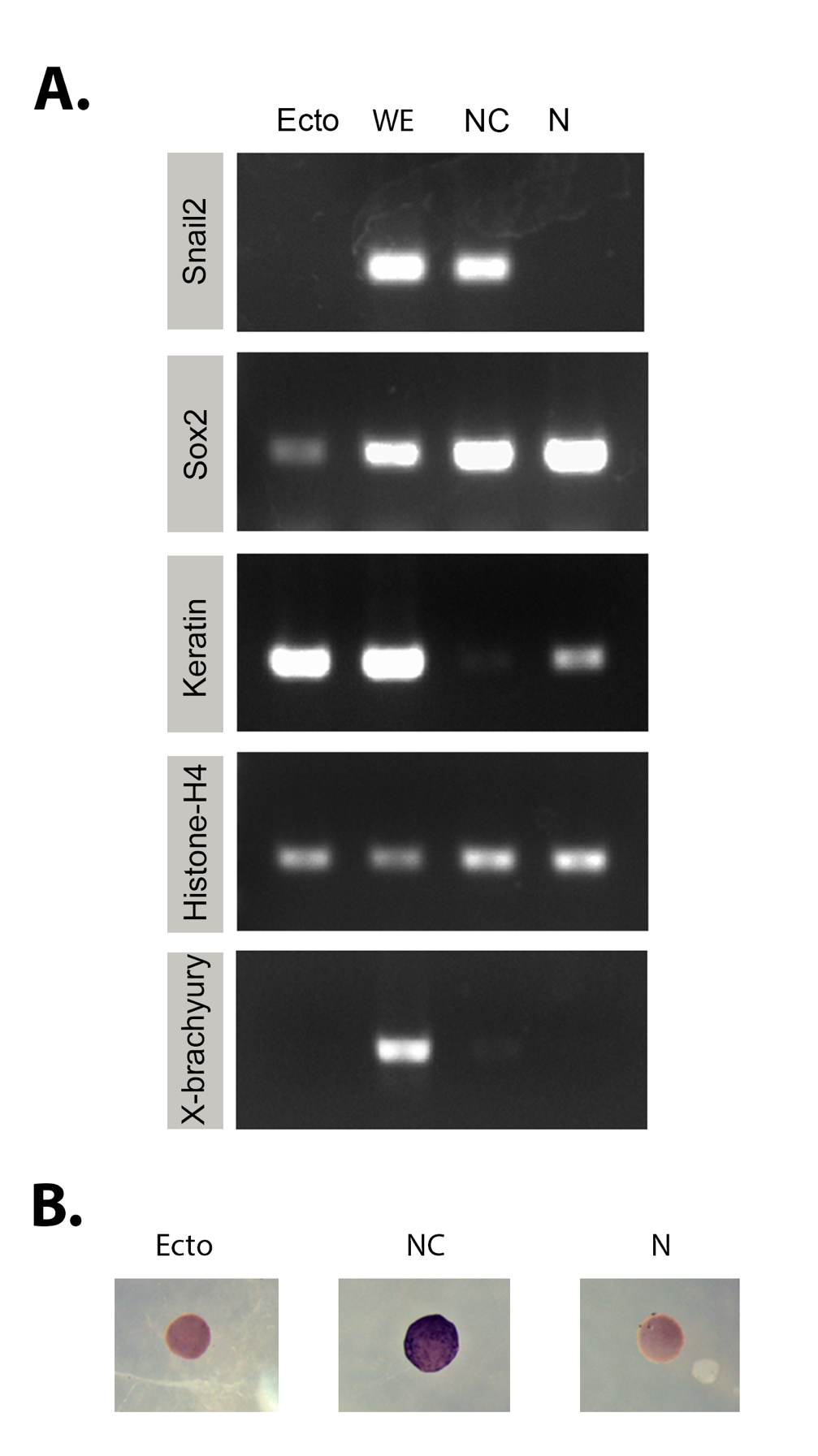
**

**SUPPLEMENTARY FIGURE 1: Validation of tissue induction** (**A**) PCR on RNA extracted from stage 15 animal cap tissue induced to become either neural or NC showed that tissue was induced efficiently. The NC marker Snail2 was only expressed in the NC animal cap tissue whilst the neural marker Sox2 was enriched in the neural tissue and epidermal keratin was enriched in the ectoderm (Ecto) sample. Histone H4 was used as a positive control and Brachyury for a control of mesoderm contamination Whole embryos (WE) were used as positive controls for all genes. (**B**) WISH for the NC marker Sox10 on stage 15 induced animal caps further confirm induction of NC tissue type with expression only being evident in NC animal caps.
