# Supplementary material for: microRNAs associated with early neural crest development in Xenopus laevis
Source: BMC Genomics. 2018 Jan 18;19:59. doi: 10.1186/s12864-018-4436-0 (PMC5774138; doi:10.1186/s12864-018-4436-0)
Supplement: Supplementary file 2 — piRNA clustering and transcriptome analysis of the sRNA sequences. piRNA clustering and transcriptome analysis of the sRNA sequences using ProTRAC revealed that the 29 nt peak observed in blastula only contains a fraction of piRNAs (3.38%). Transcriptome analysis shows these are not degraded transcripts derived from mid blastula transition. The peak at 29 nt therefore contains an unidentified class of sRNA. (DOCX 92 kb) [file 12864_2018_4436_MOESM2_ESM.docx]

**Additional file 2: Table S1 piRNA clustering and transcriptome analysis of the sRNA sequences.** piRNA clustering and transcriptome analysis of the sRNA sequences using ProTRAC revealed that the 29 nt peak observed in blastula only contains a fraction of piRNAs (3.38%). Transcriptome analysis shows these are not degraded transcripts derived from mid blastula transition. The peak at 29 nt therefore contains an unidentified class of sRNA.

|  |  | Ectoderm | Neural | Blastula | NC |
| --- | --- | --- | --- | --- | --- |
| **ProTRAC** | **Predicted piRNA clusters** | 59 | 74 | 56 | 59 |
|  | **Total size** | 0.02% | 0.03% | 0.02% | 0.02% |
|  | **Sequence reads that can be assigned to clusters** | 5.09% | 6.00% | 3.38% | 4.22% |
| **Transcriptome analysis** | **Total sequences aligned to transcriptome** | 1091358 | 1457080 | 978399 | 2074442 |
|  | **% aligning to genes** | 6.36% | 7.91% | 5.54% | 10.21% |
