# Supplementary material for: microRNAs associated with early neural crest development in Xenopus laevis
Source: BMC Genomics. 2018 Jan 18;19:59. doi: 10.1186/s12864-018-4436-0 (PMC5774138; doi:10.1186/s12864-018-4436-0)
Supplement: Supplementary file 4 — Line plots of miRNA expression levels across the different tissue types. (PDF 1374 kb) [file 12864_2018_4436_MOESM4_ESM.pdf]

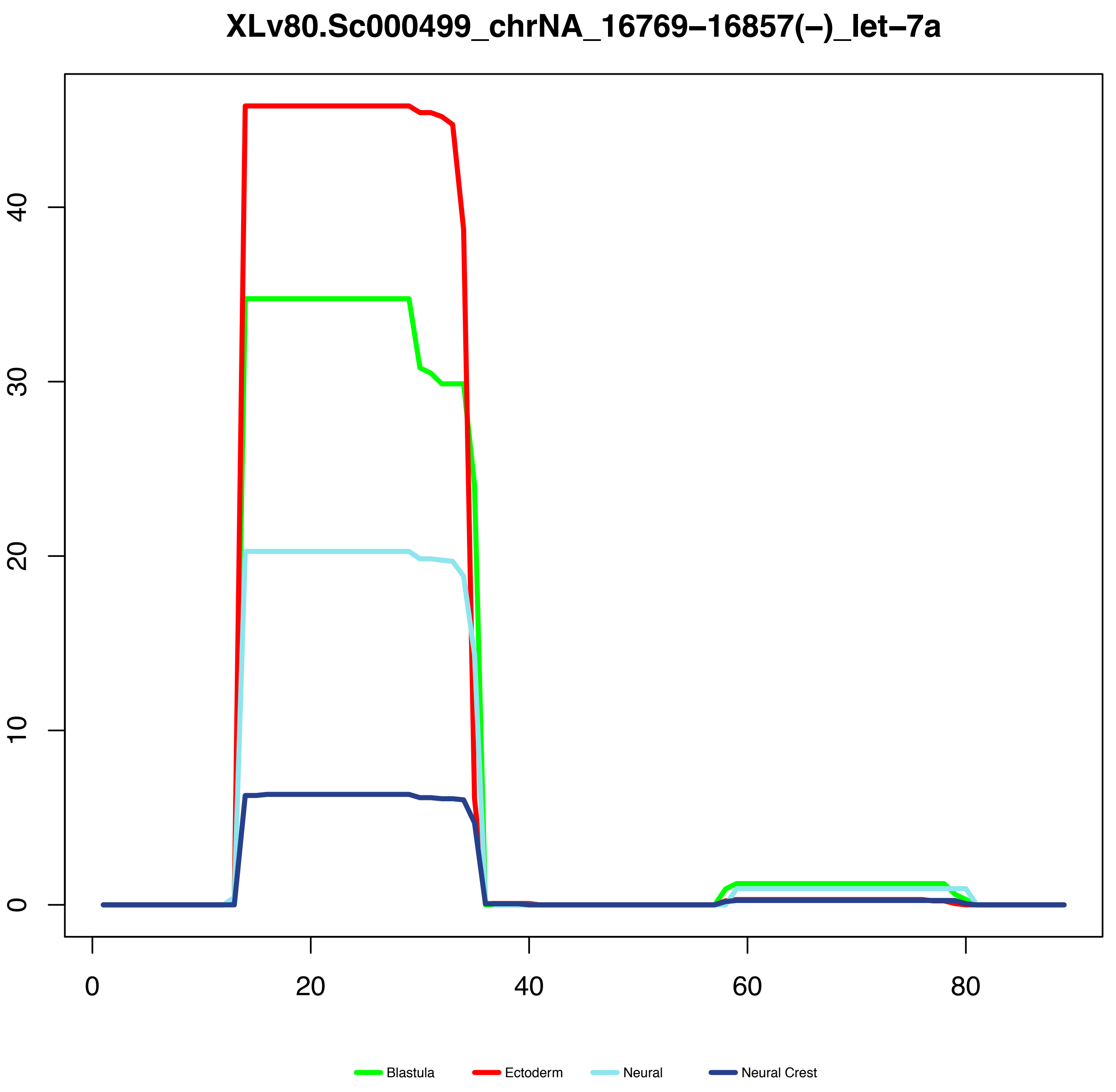

XLv80.Sc000499_chrNA_16769−16857(−)_let−7a
0
10
20
30
40
0
20
40
60
80
Blastula
Ectoderm
Neural
Neural Crest

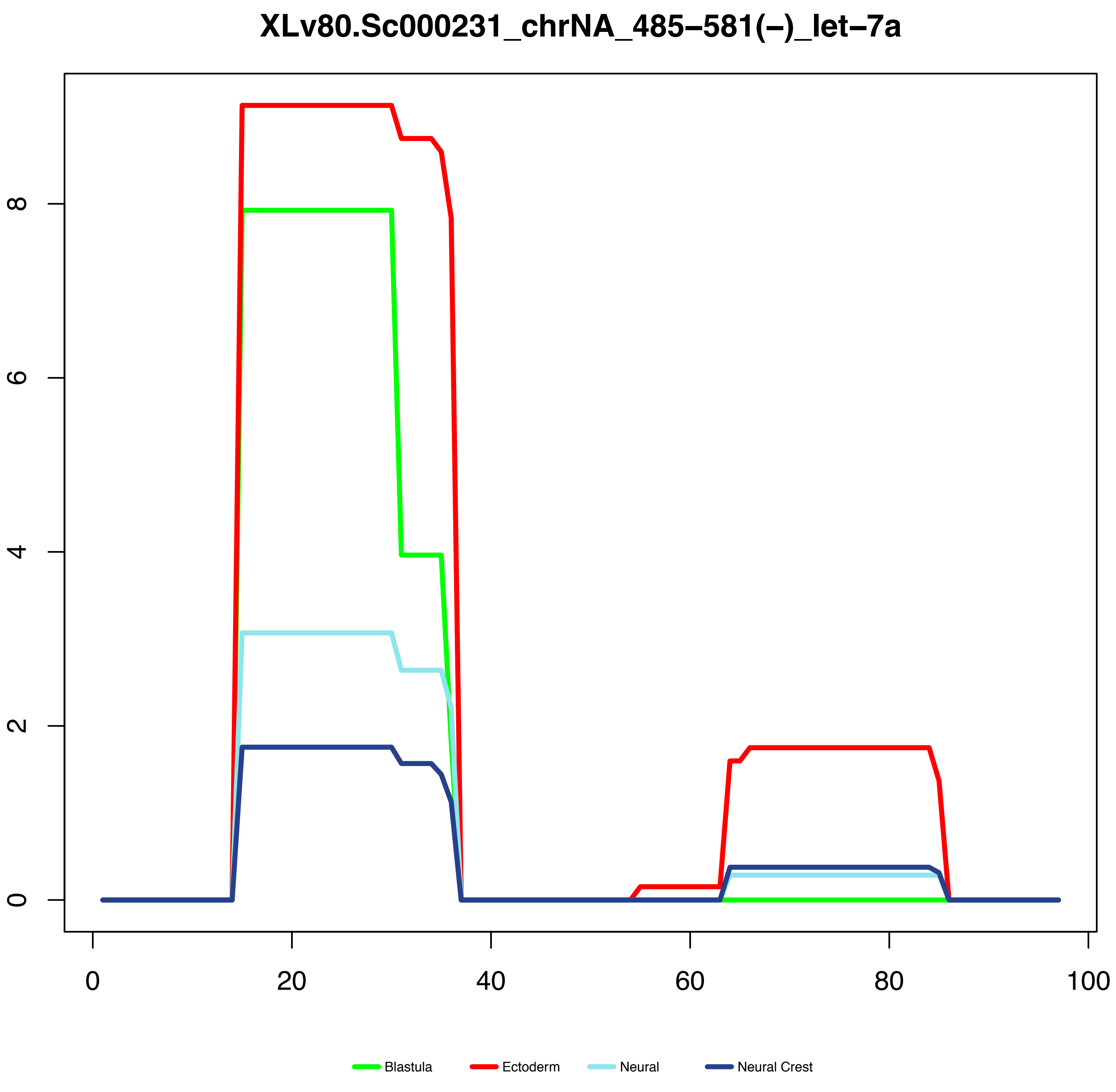
XLv80.Sc000231_chrNA_485–581(–)_let–7a
0
2
4
6
8
0
20
40
60
80
100
Blastula
Ectoderm
Neural
Neural Crest

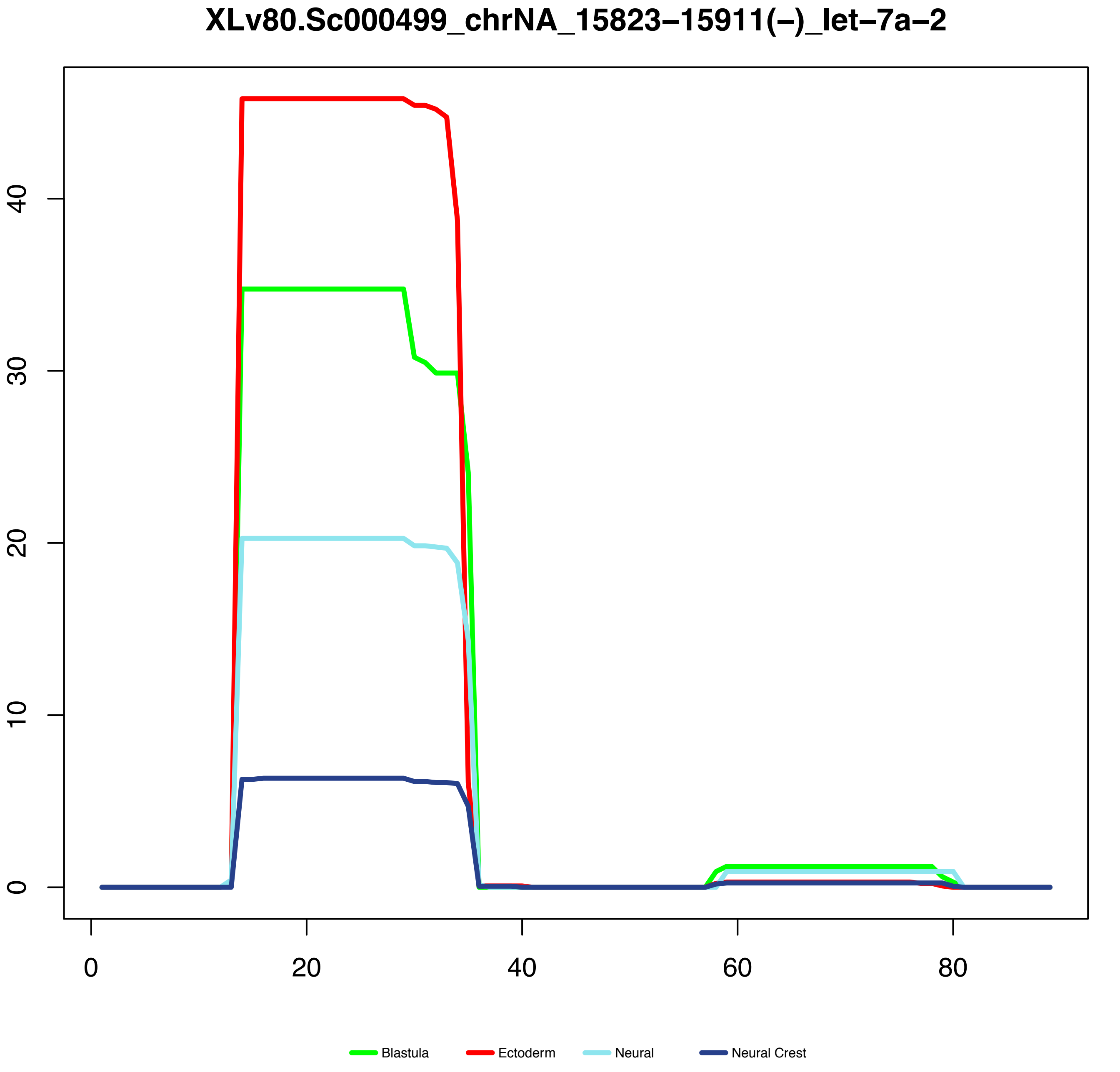
XLv80.Sc000499_chrNA_15823–15911(–)_let–7a–2
0
10
20
30
40
0
20
40
60
80
Blastula
Ectoderm
Neural
Neural Crest

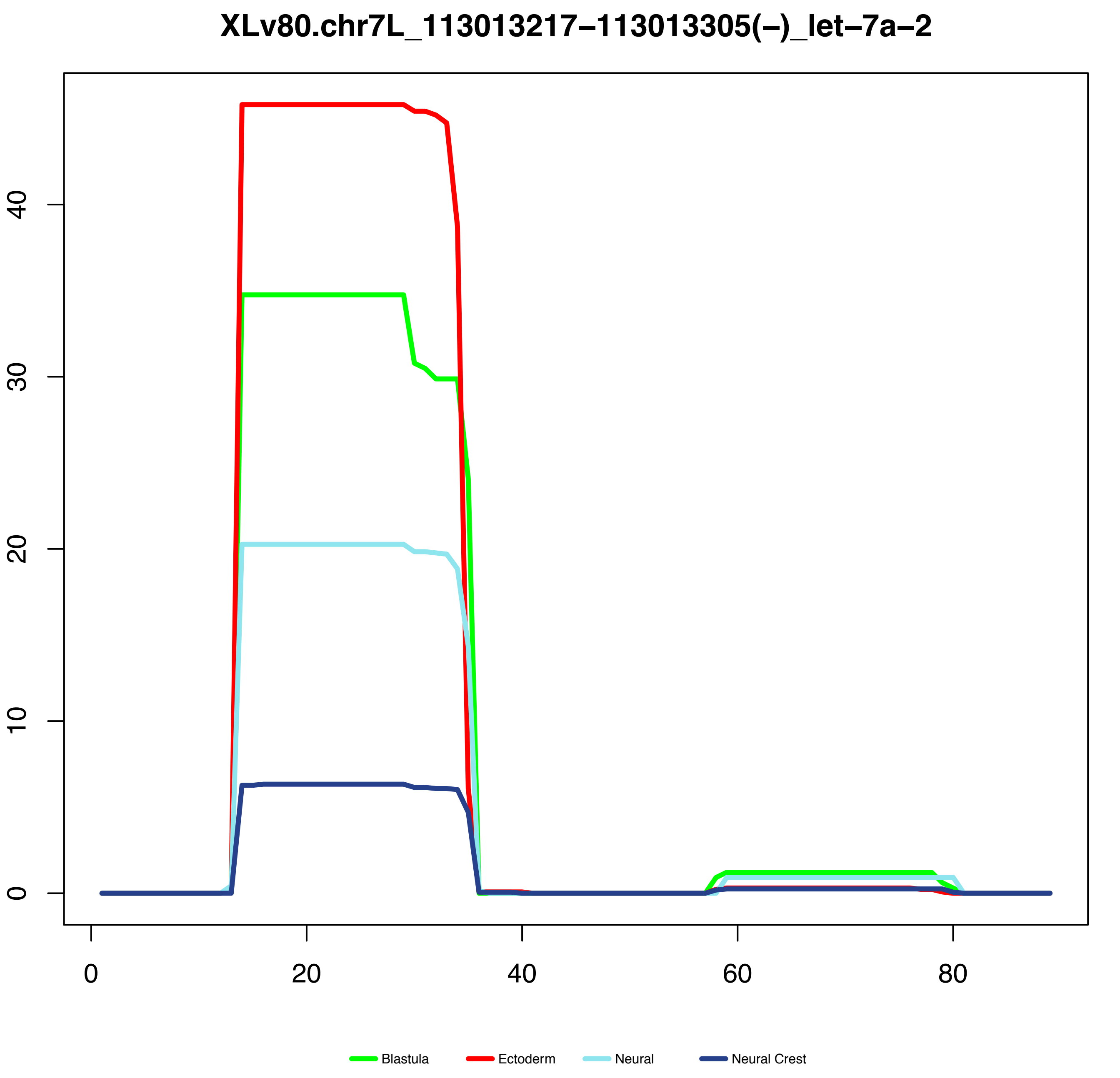
XLv80.chr7L_113013217−113013305(−)_let−7a−2
0
10
20
30
40
0
20
40
60
80
Blastula
Ectoderm
Neural
Neural Crest

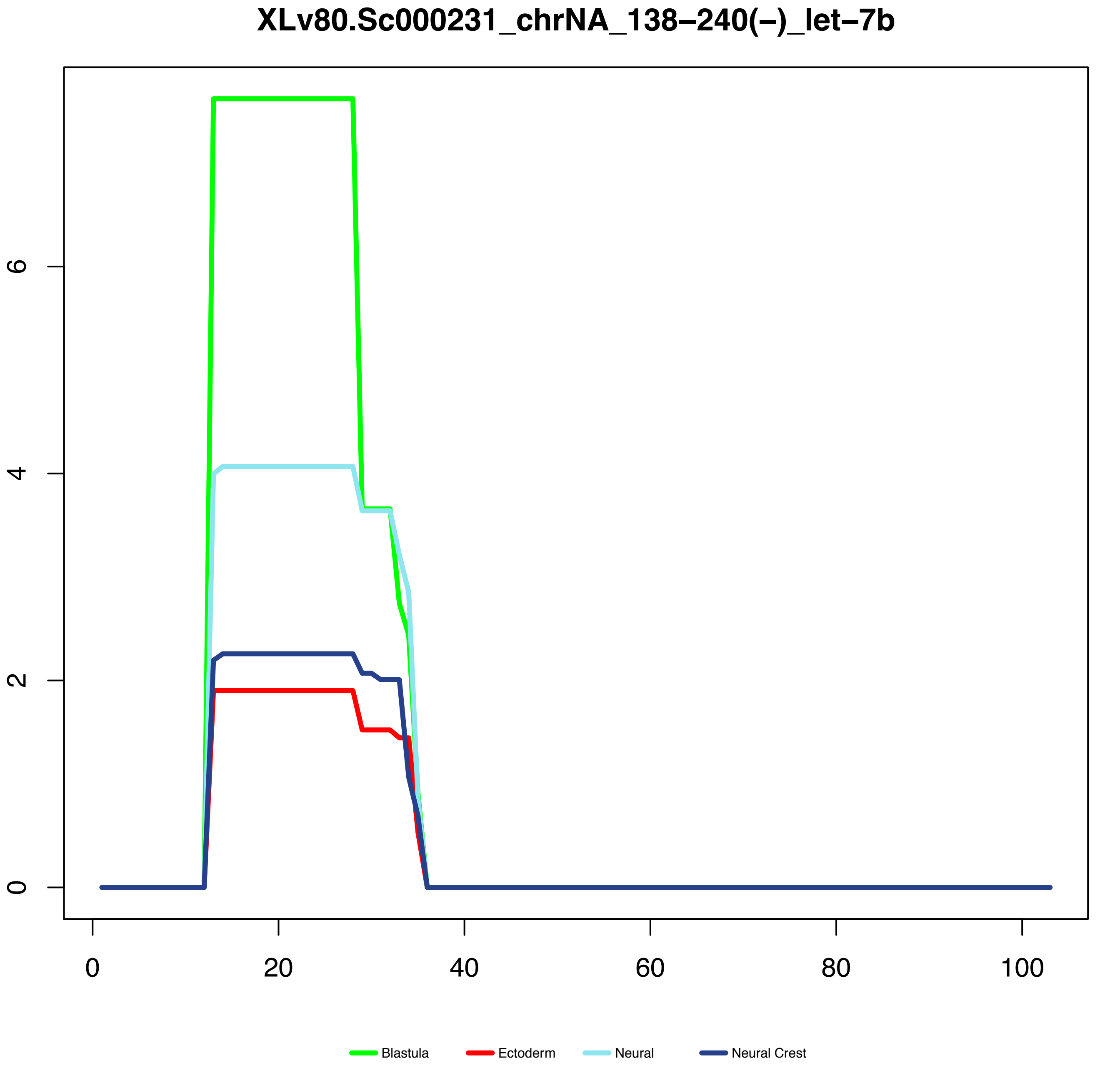

XLv80.Sc000231_chrNA_138−240(−)_let−7b
0
2
4
6
0
20
40
60
80
100
Blastula
Ectoderm
Neural
Neural Crest

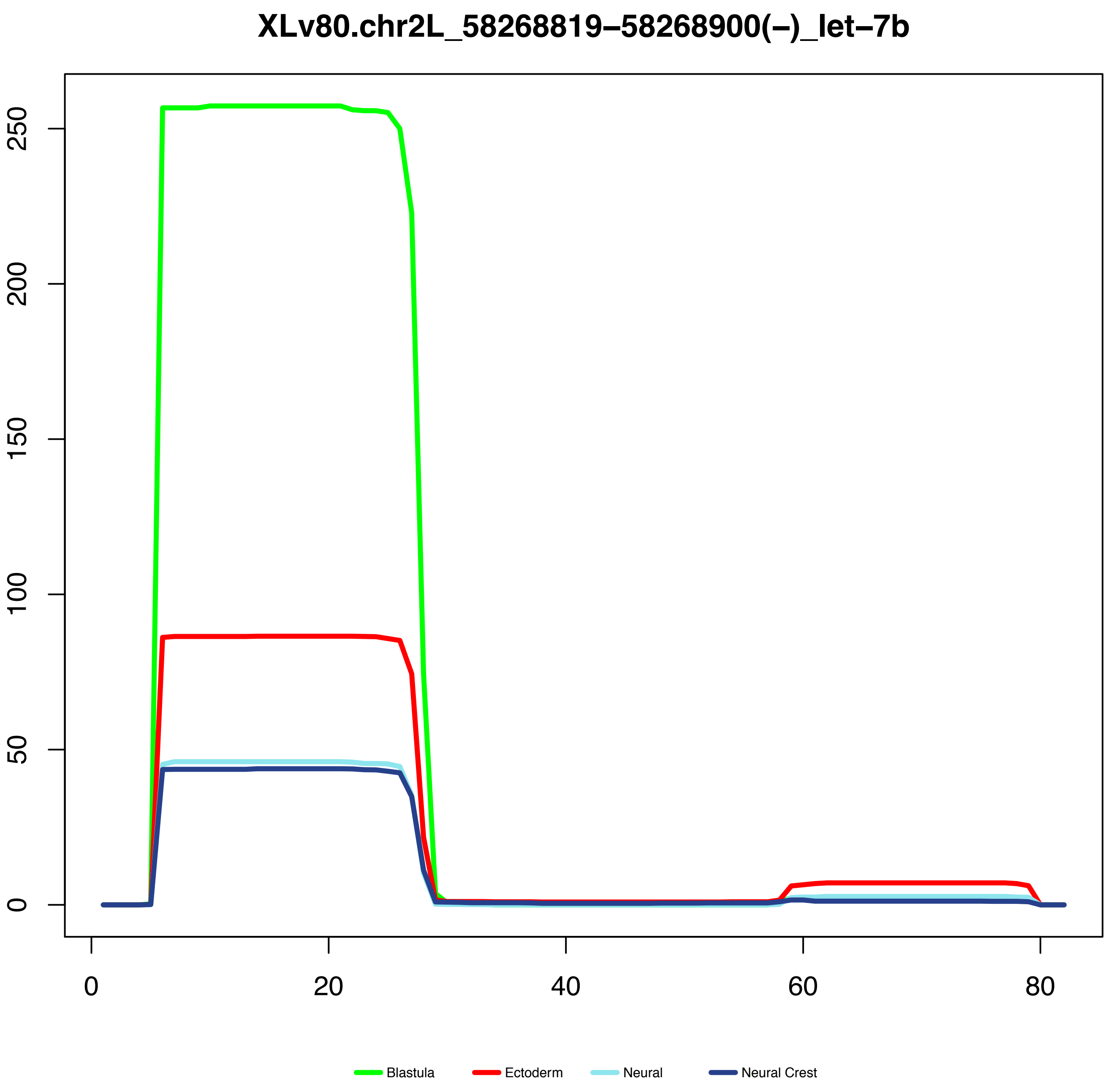
XLv80.chr2L_58268819–58268900(–)_let–7b
0
50
100
150
200
250
0
20
40
60
80
Blastula
Ectoderm
Neural
Neural Crest

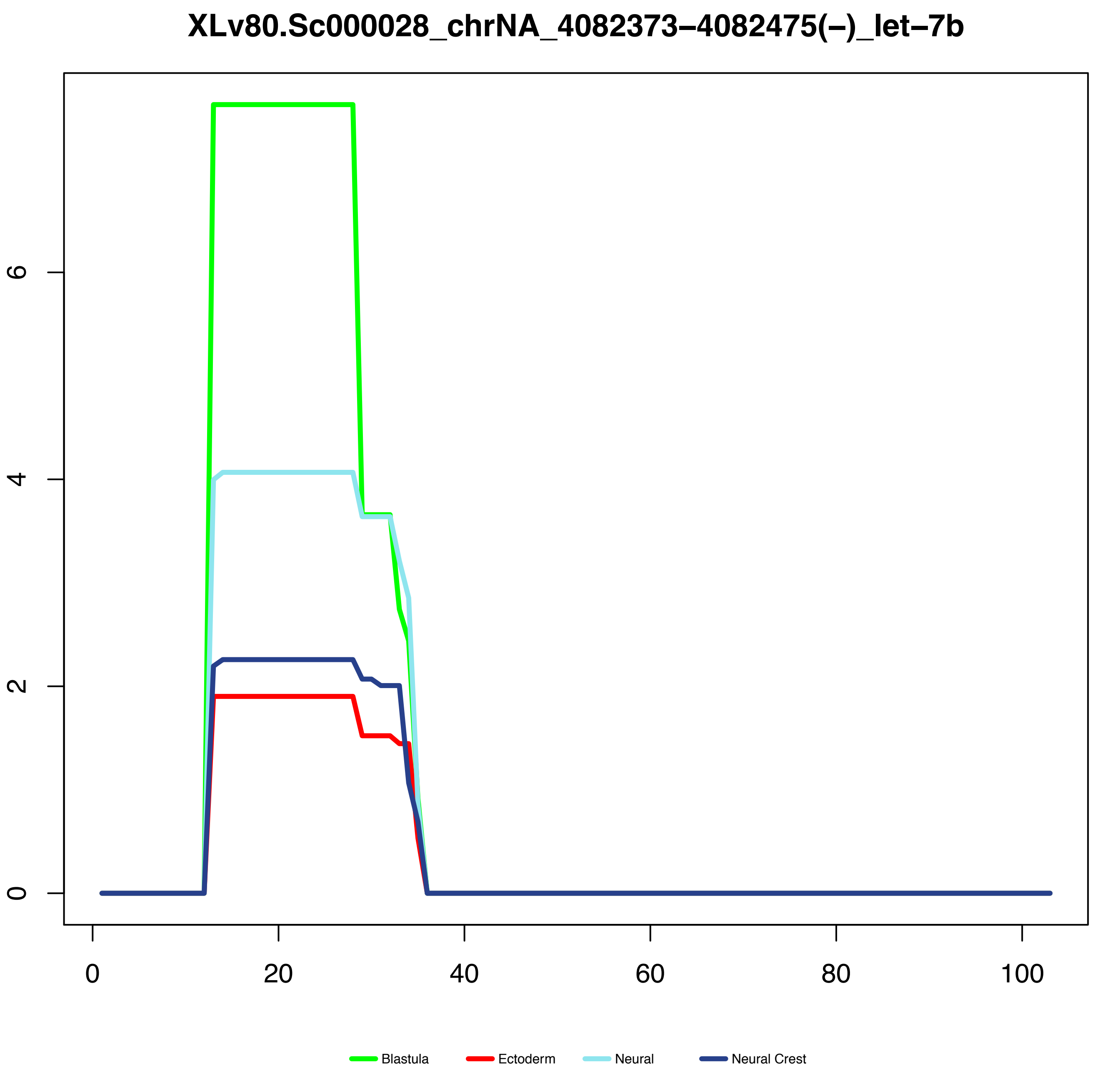

XLv80.Sc000028_chrNA_4082373−4082475(−)_let−7b
0
2
4
6
0
20
40
60
80
100
Blastula
Ectoderm
Neural
Neural Crest

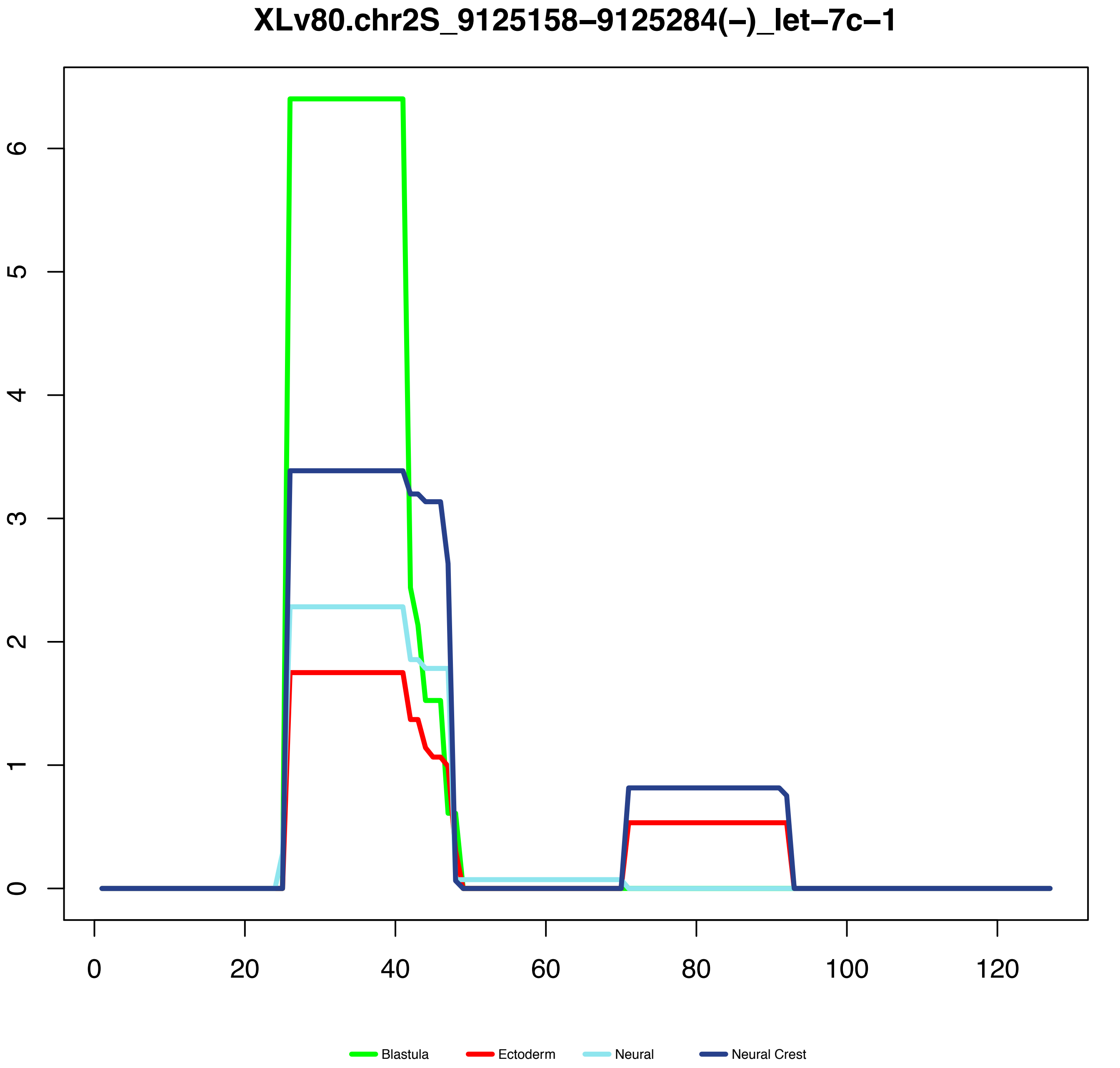
XLv80.chr2S_9125158−9125284(−)_let−7c−1
0
1
2
3
4
5
6
0
20
40
60
80
100
120
Blastula
Ectoderm
Neural
Neural Crest

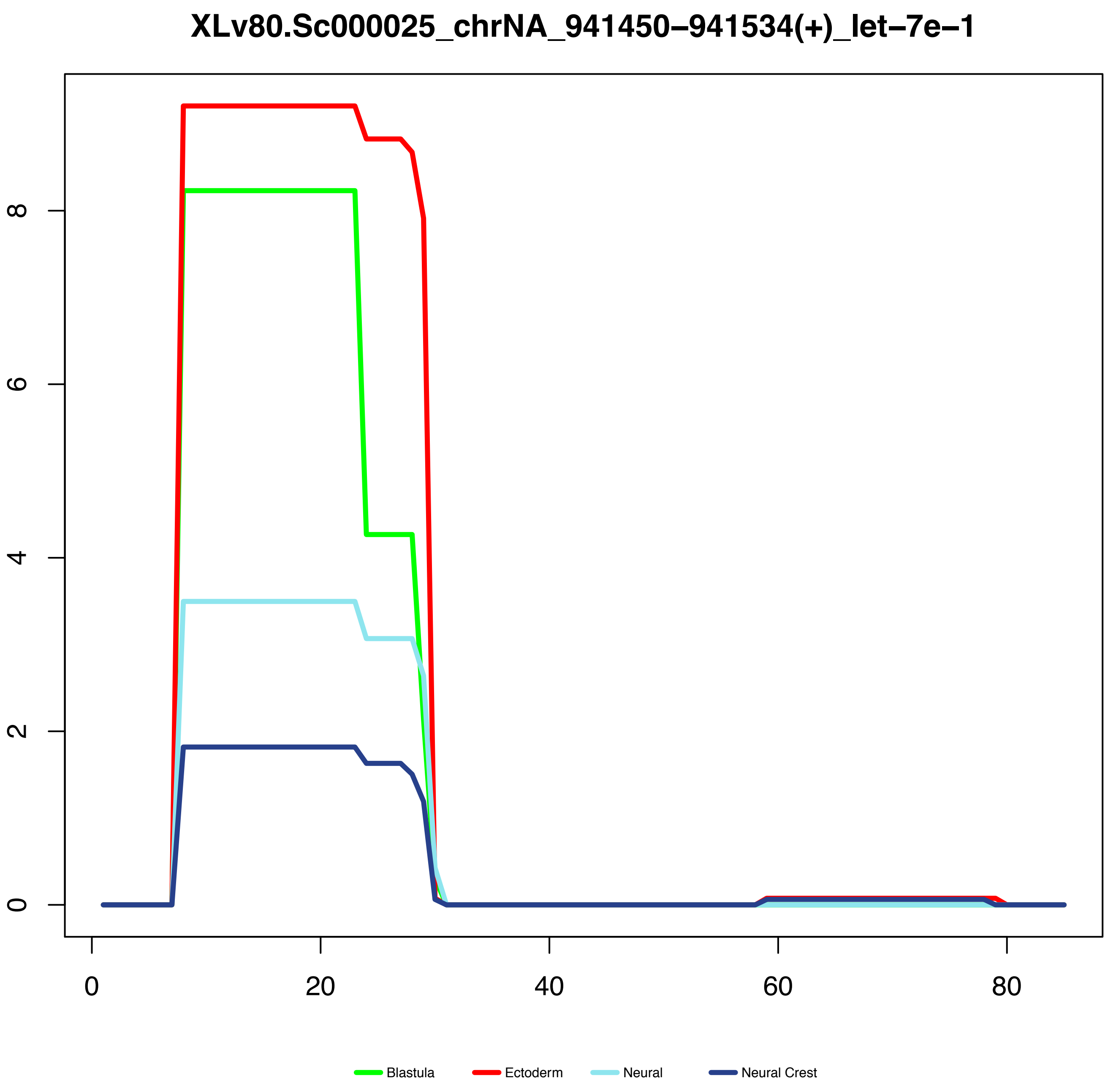
XLv80.Sc000025_chrNA_941450−941534(+)_let−7e−1
0
2
4
6
8
0
20
40
60
80
Blastula
Ectoderm
Neural
Neural Crest

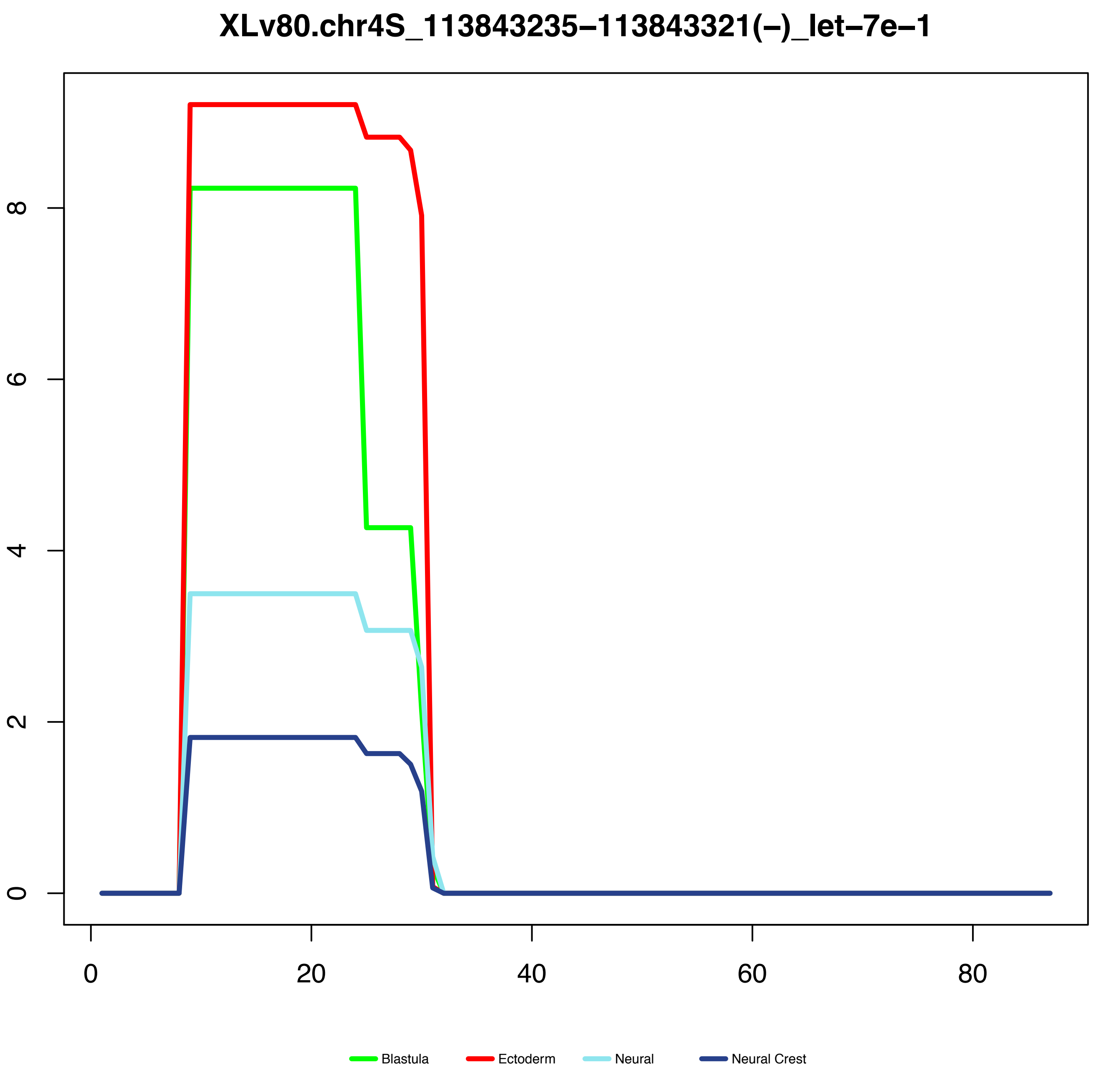
XLv80.chr4S_113843235−113843321(−)_let−7e−1
0
2
4
6
8
0
20
40
60
80
Blastula
Ectoderm
Neural
Neural Crest

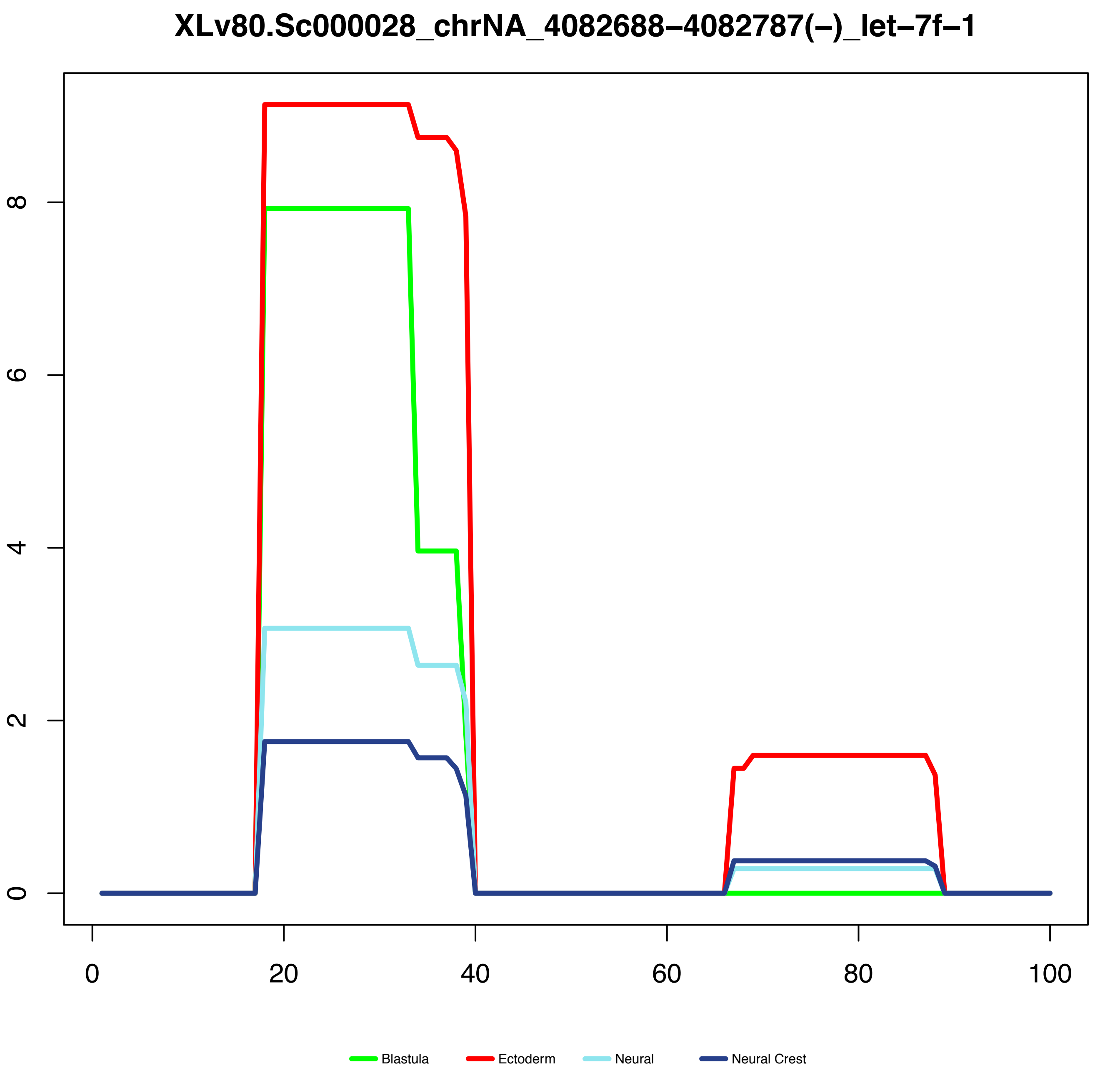
XLv80.Sc000028_chrNA_4082688−4082787(−)_let−7f−1
0
2
4
6
8
0
20
40
60
80
100
Blastula
Ectoderm
Neural
Neural Crest

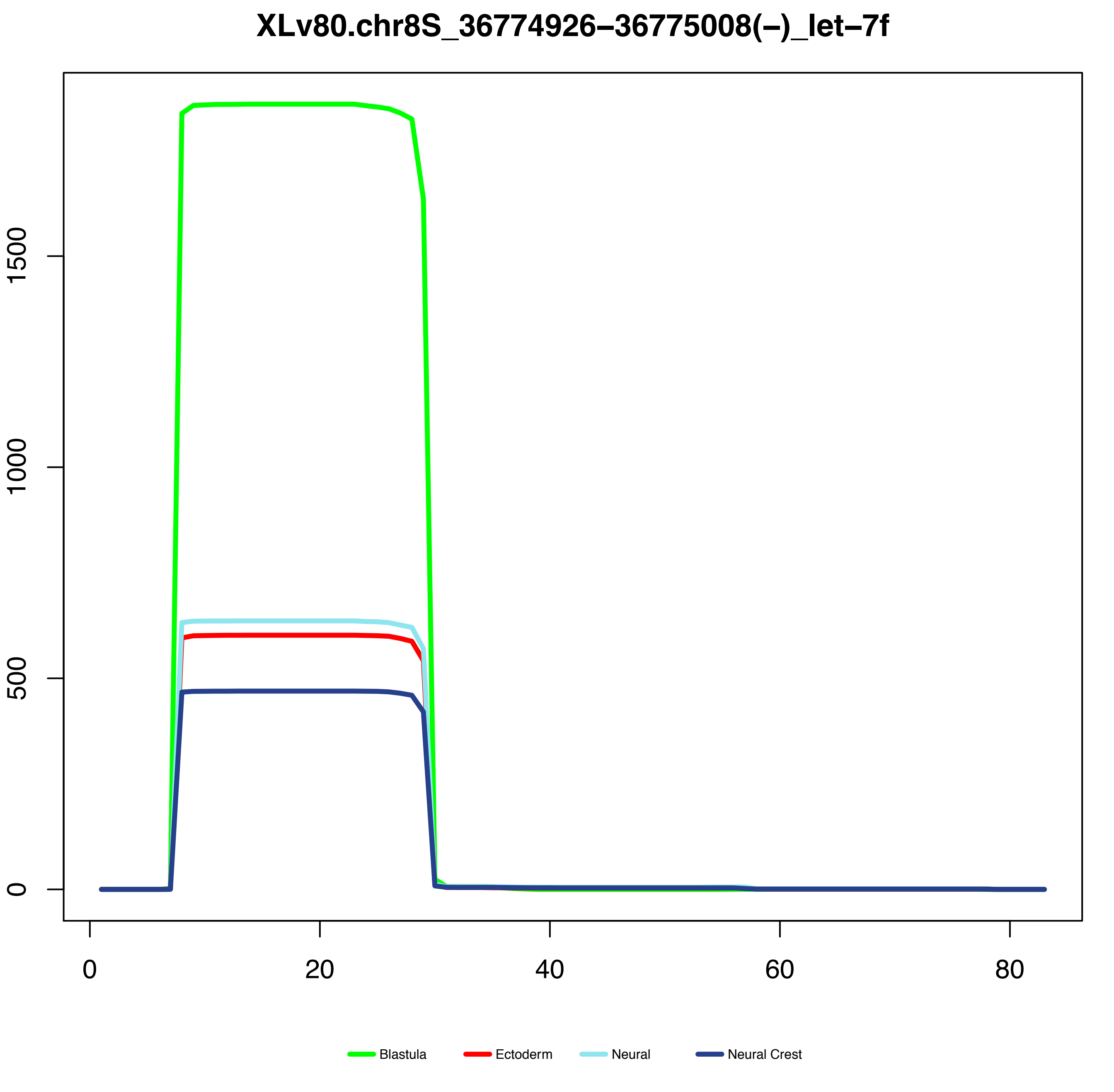
XLv80.chr8S_36774926–36775008(–)_let–7f
0
500
1000
1500
0
20
40
60
80
Blastula
Ectoderm
Neural
Neural Crest

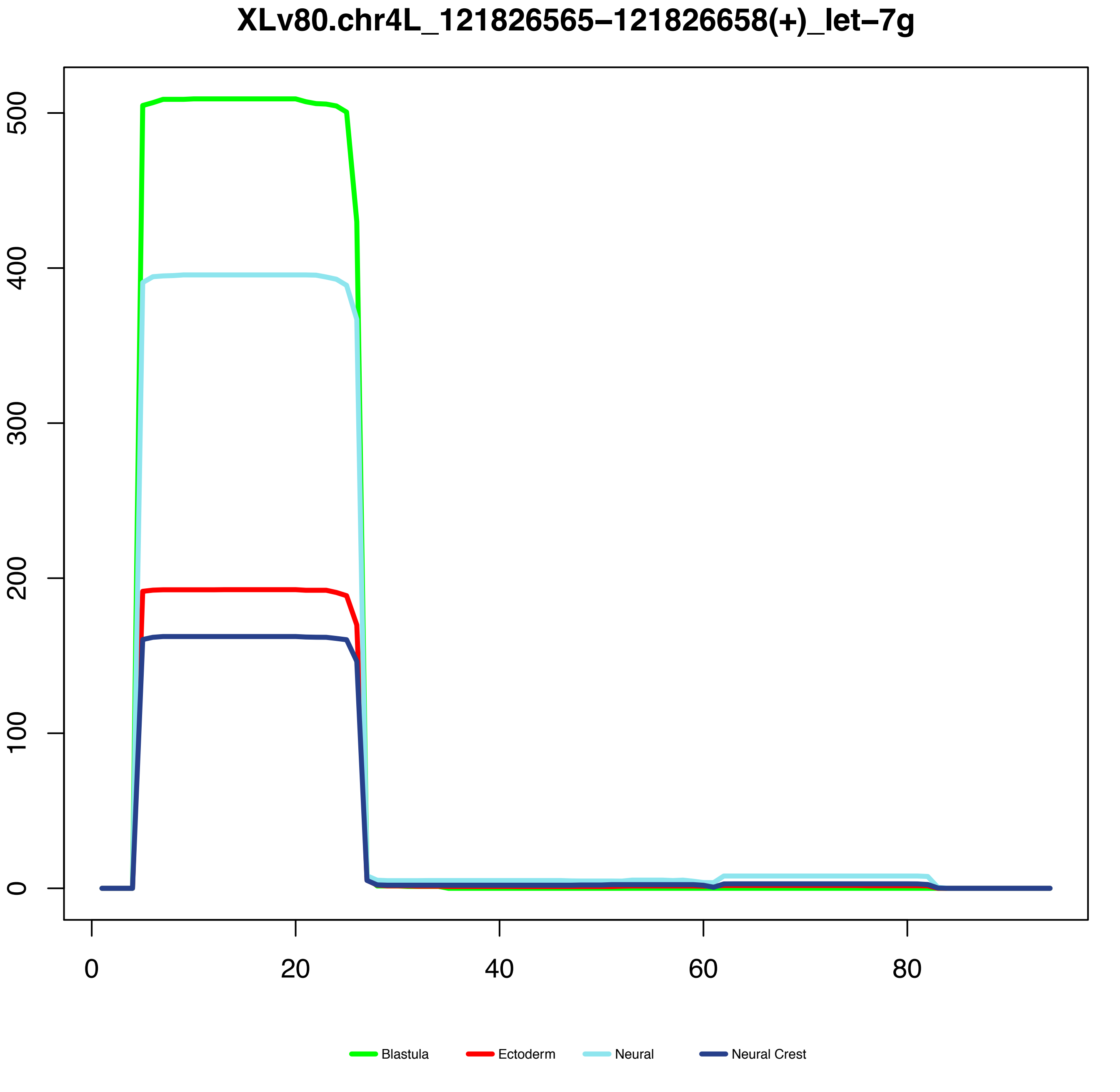

XLv80.chr4L_121826565−121826658(+)_let−7g
0
100
200
300
400
500
0
20
40
60
80
Blastula
Ectoderm
Neural
Neural Crest

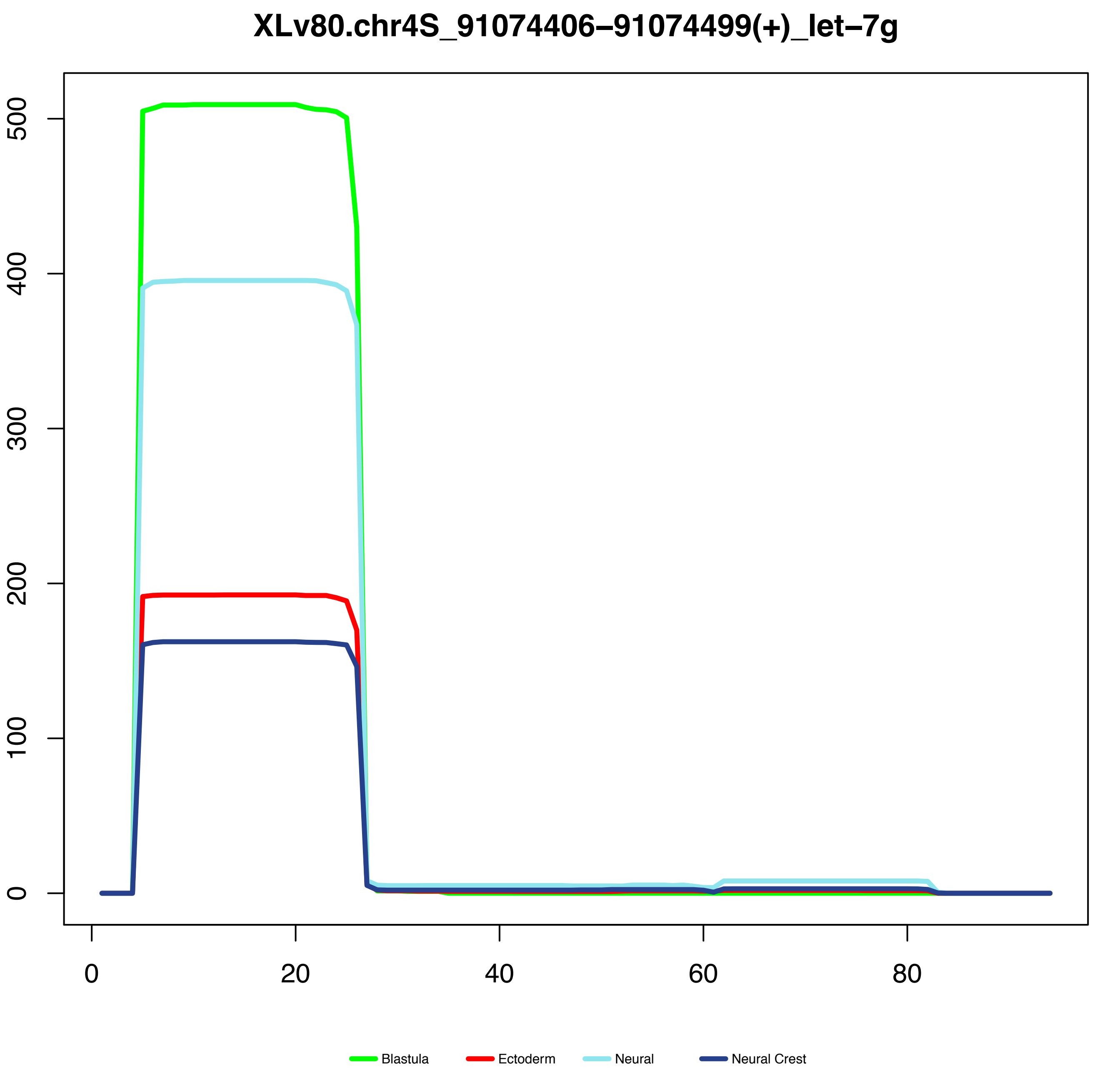

XLv80.chr4S_91074406–91074499(+)_let–7g
0
100
200
300
400
500
0
20
40
60
80
Blastula
Ectoderm
Neural
Neural Crest

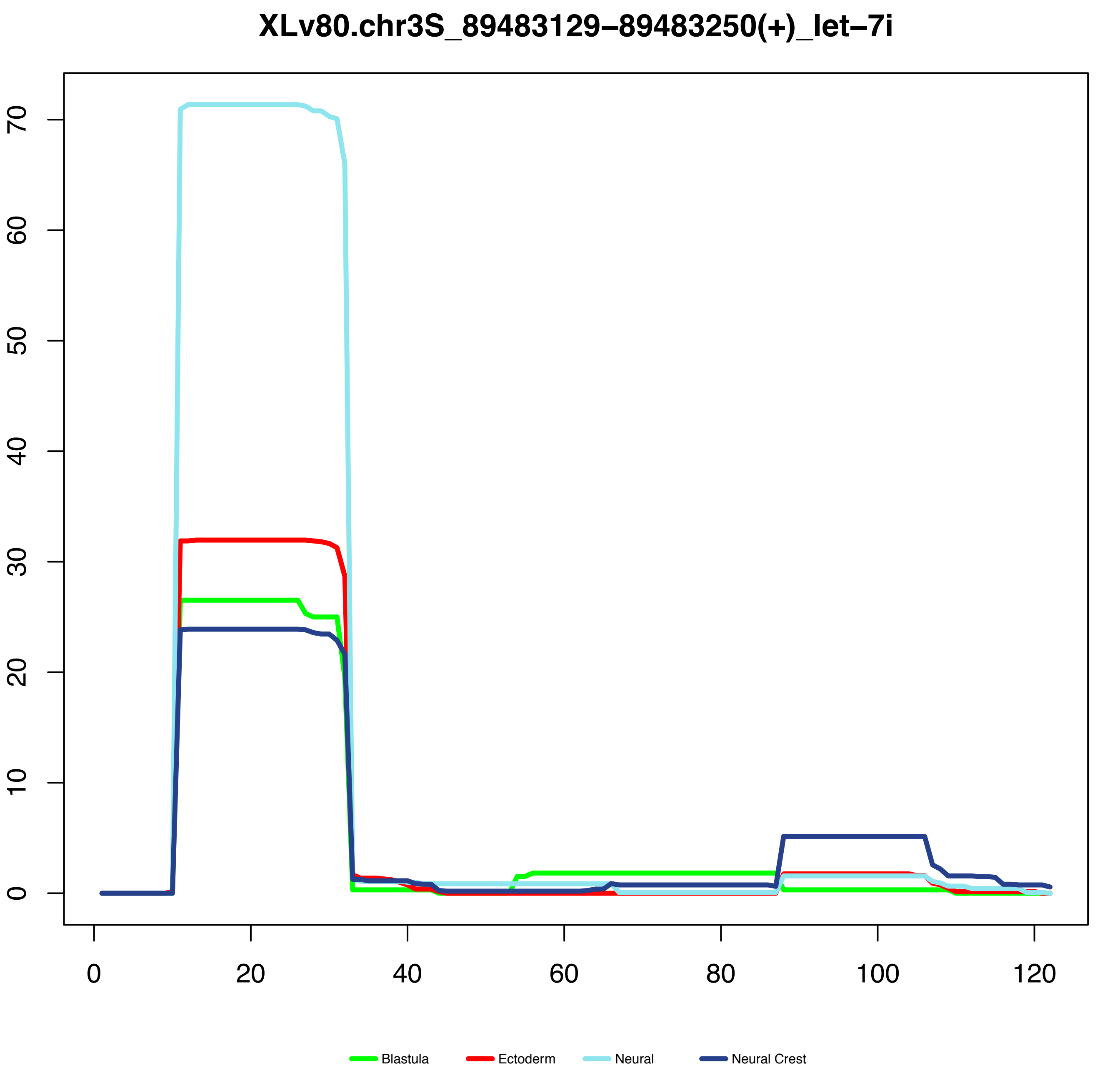
XLv80.chr3S_89483129−89483250(+)_let−7i
0
10
20
30
40
50
60
70
0
20
40
60
80
100
120
Blastula
Ectoderm
Neural
Neural Crest

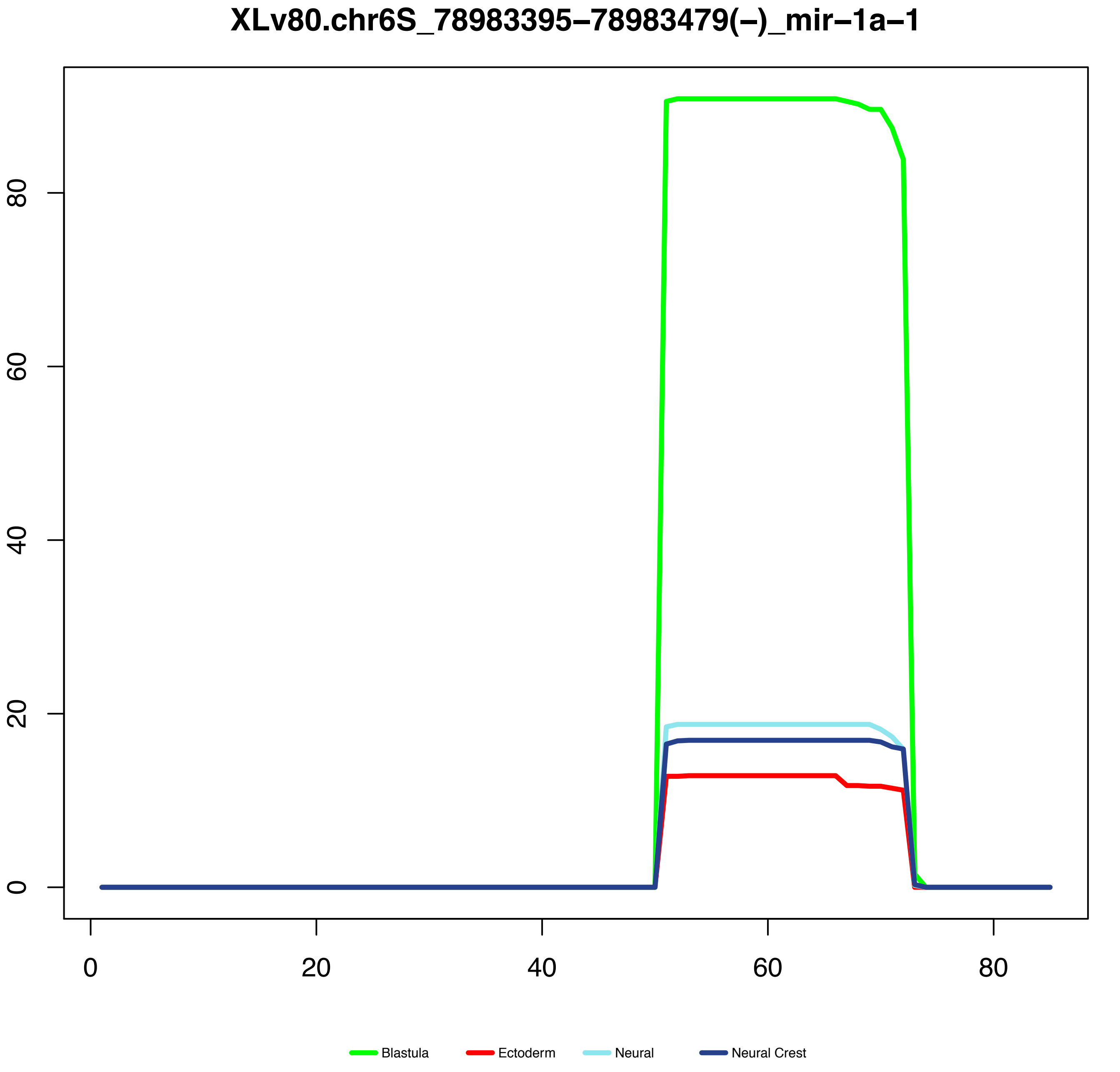

XLv80.chr6S_78983395−78983479(−)_mir−1a−1
0
20
40
60
80
0
20
40
60
80
Blastula
Ectoderm
Neural
Neural Crest

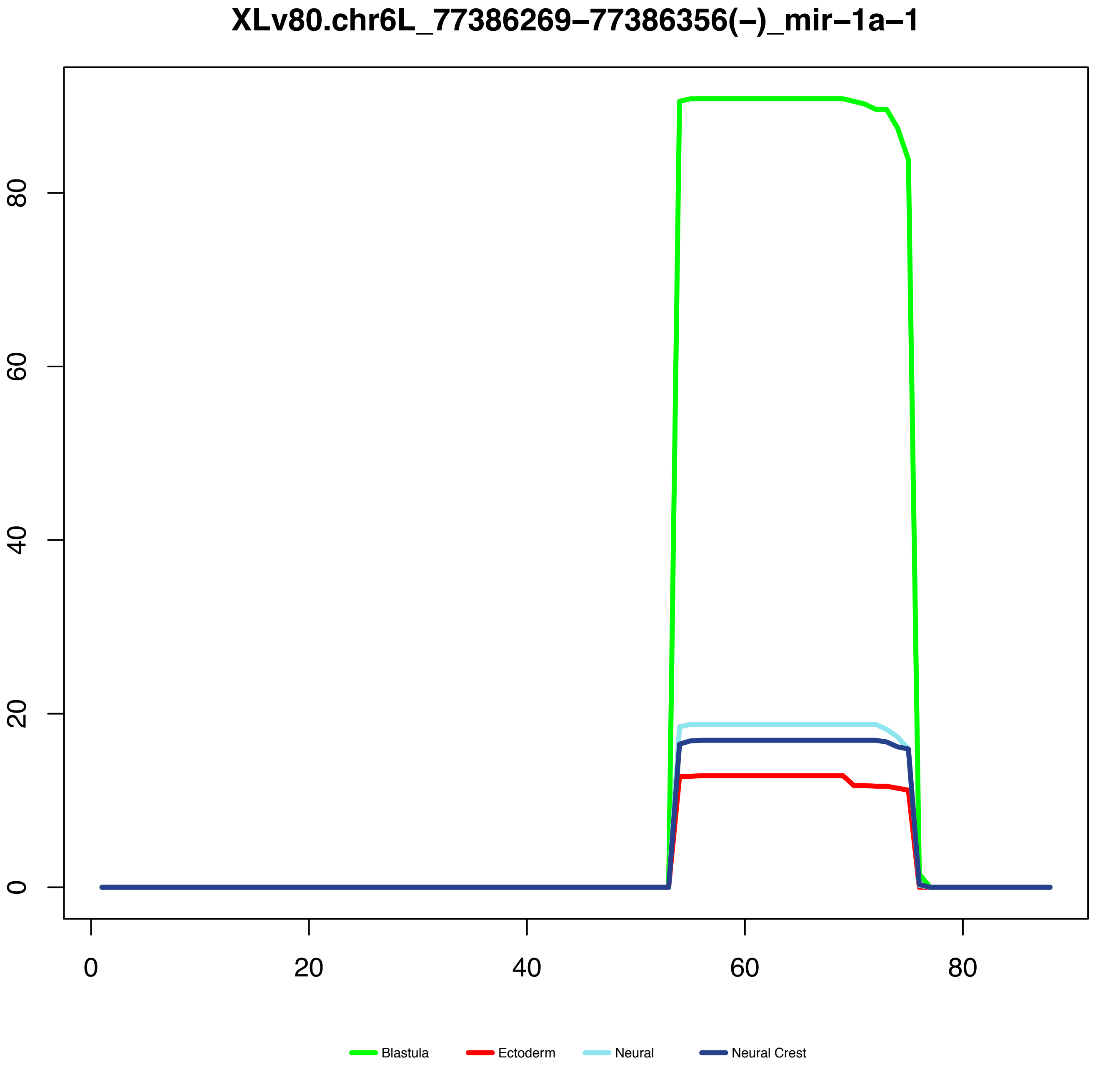
XLv80.chr6L_77386269−77386356(−)_mir−1a−1
0
20
40
60
80
0
20
40
60
80
Blastula
Ectoderm
Neural
Neural Crest

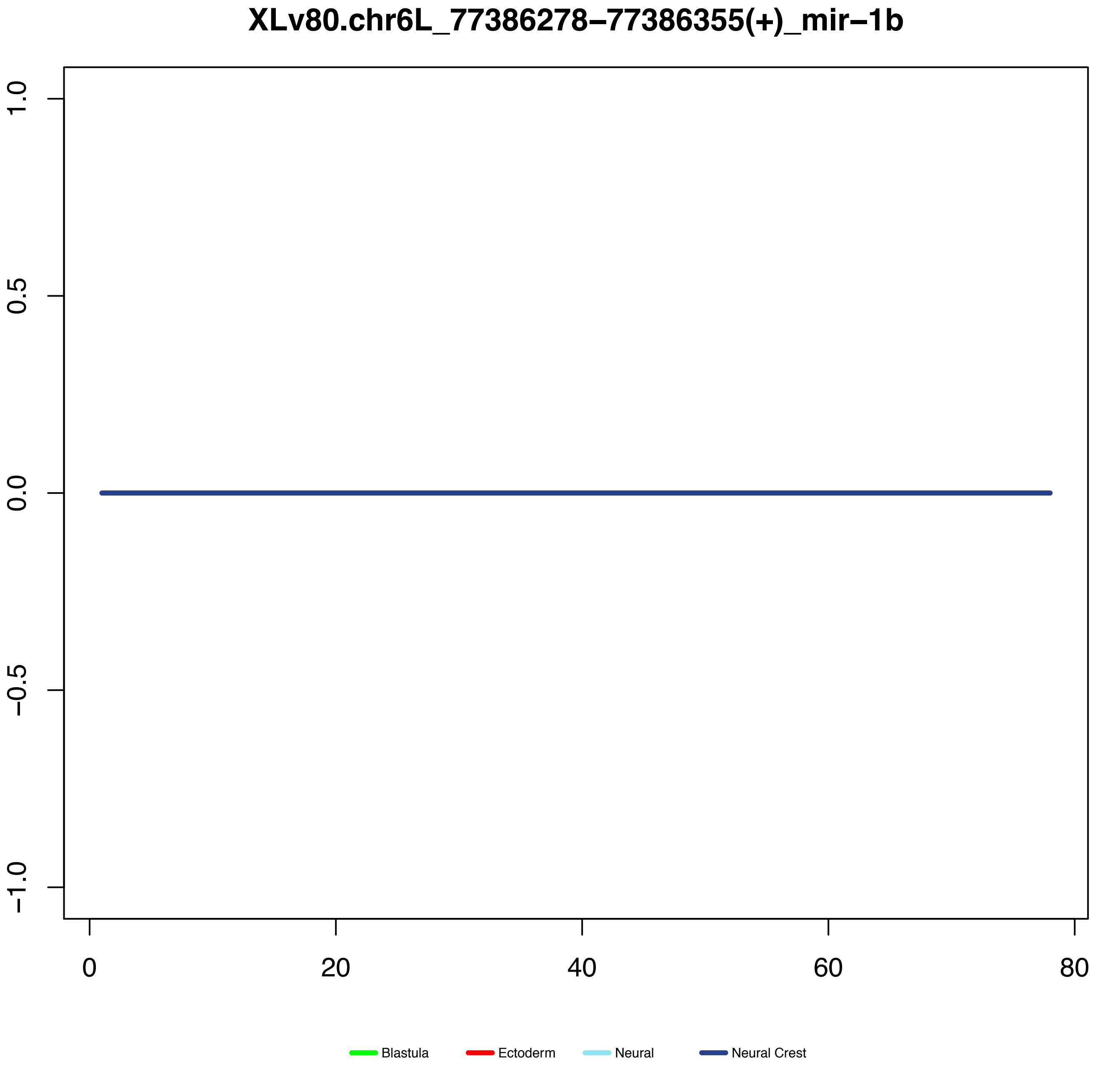

XLv80.chr6L_77386278−77386355(+)_mir−1b
1.0
0.5
0.0
−0.5
−1.0
0
20
40
60
80
Blastula
Ectoderm
Neural
Neural Crest

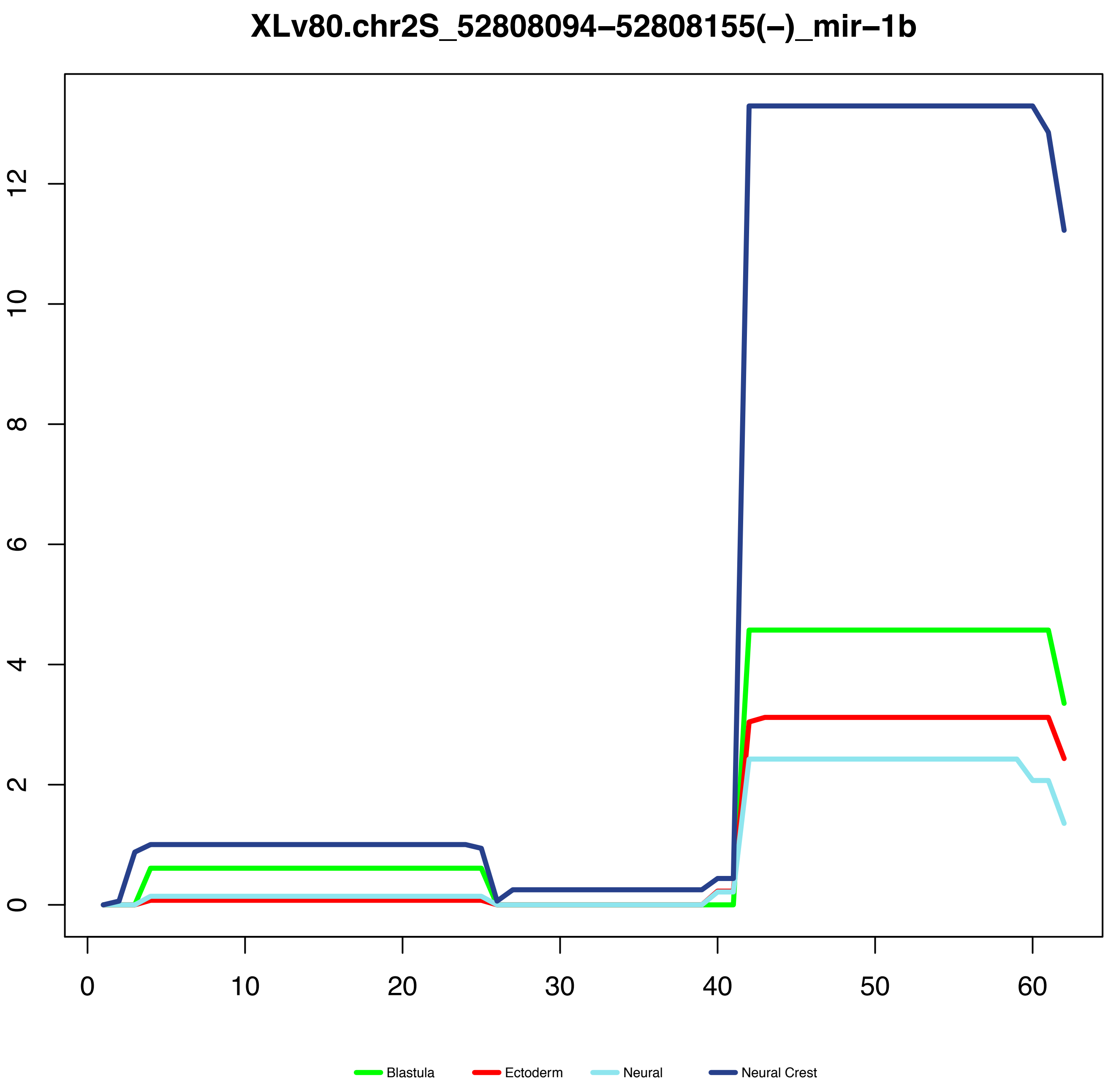

XLv80.chr2S_52808094−52808155(−)_mir−1b
0
2
4
6
8
10
12
0
10
20
30
40
50
60
Blastula
Ectoderm
Neural
Neural Crest

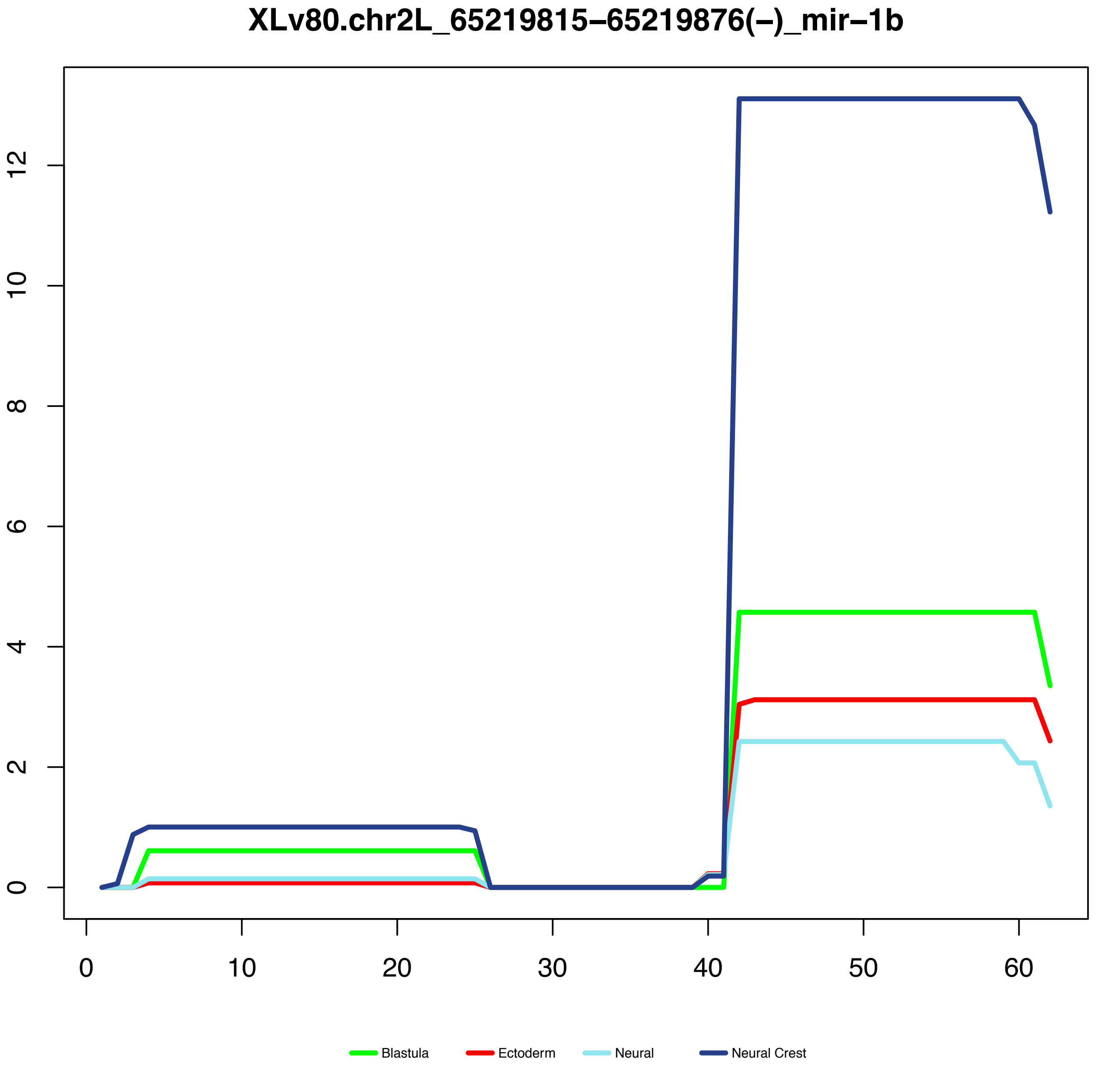
XLv80.chr2L_65219815−65219876(−)_mir−1b
0
2
4
6
8
10
12
0
10
20
30
40
50
60
Blastula
Ectoderm
Neural
Neural Crest

XLv80.chr6S_78983404−78983468(+)_mir−1b

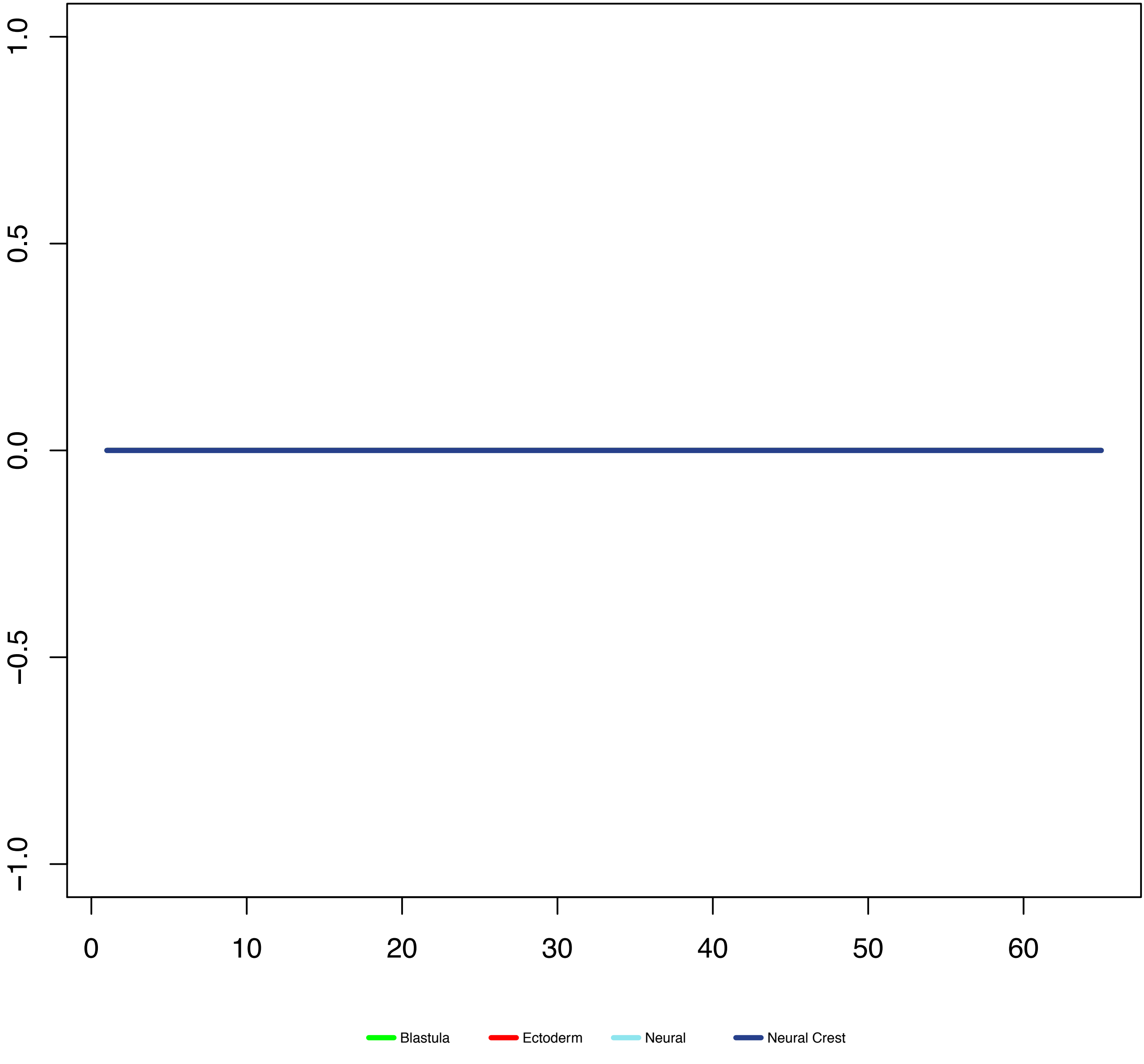

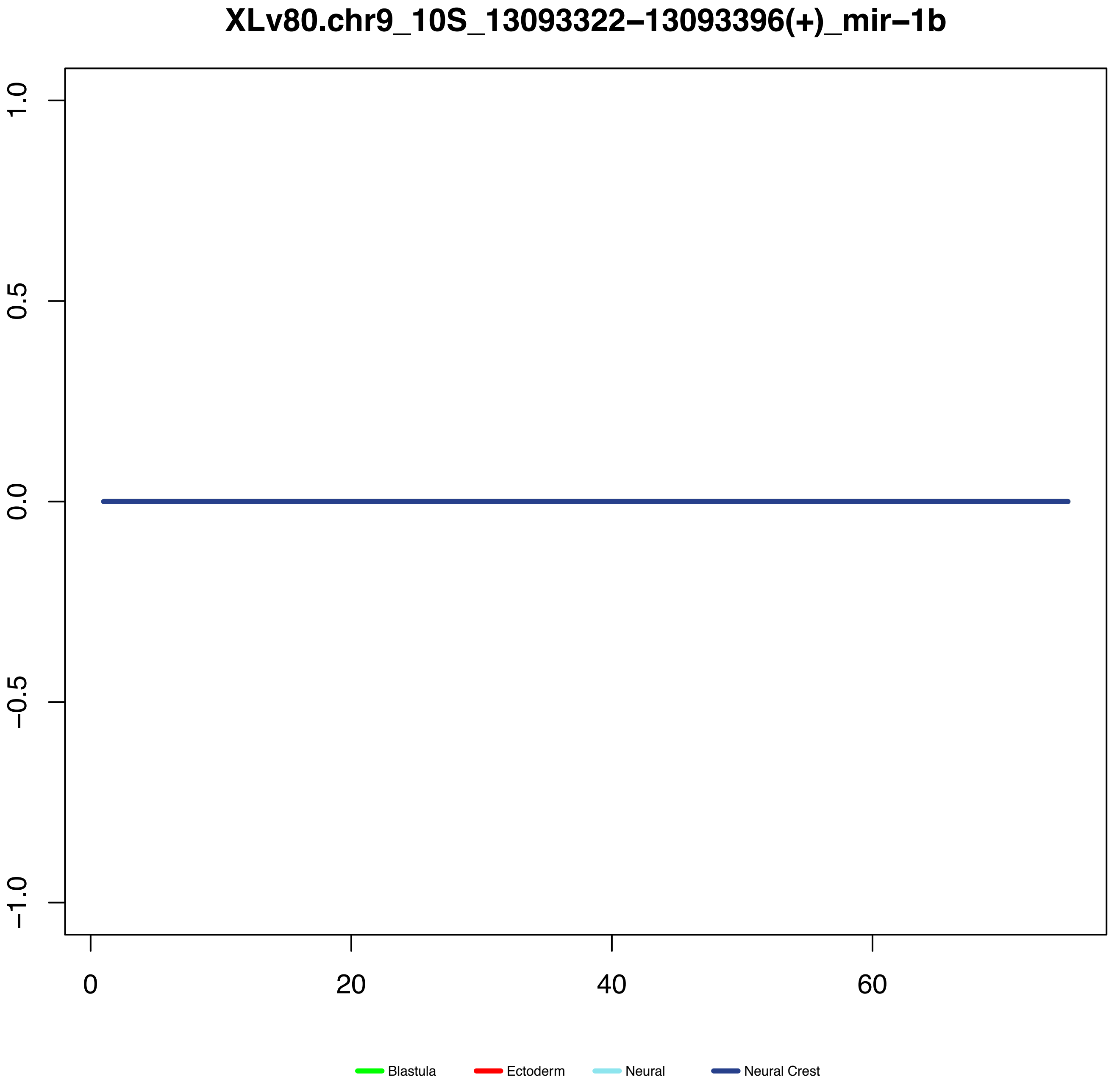
XLv80.chr9_10S_13093322−13093396(+)_mir−1b
1.0
0.5
0.0
−0.5
−1.0
0
20
40
60
Blastula
Ectoderm
Neural
Neural Crest

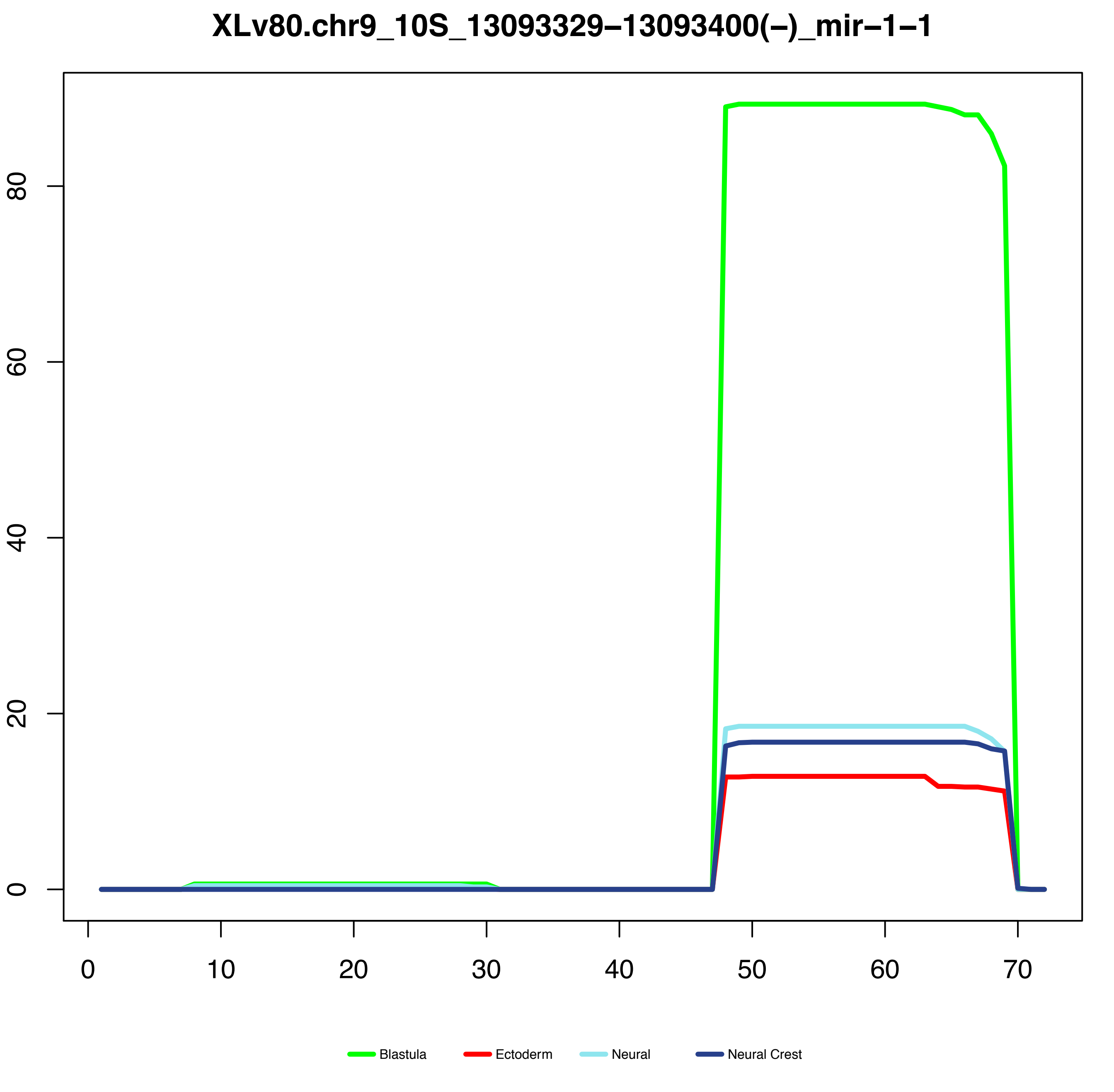
XLv80.chr9_10S_13093329−13093400(−)_mir−1−1
0
20
40
60
80
0
10
20
30
40
50
60
70
Blastula
Ectoderm
Neural
Neural Crest

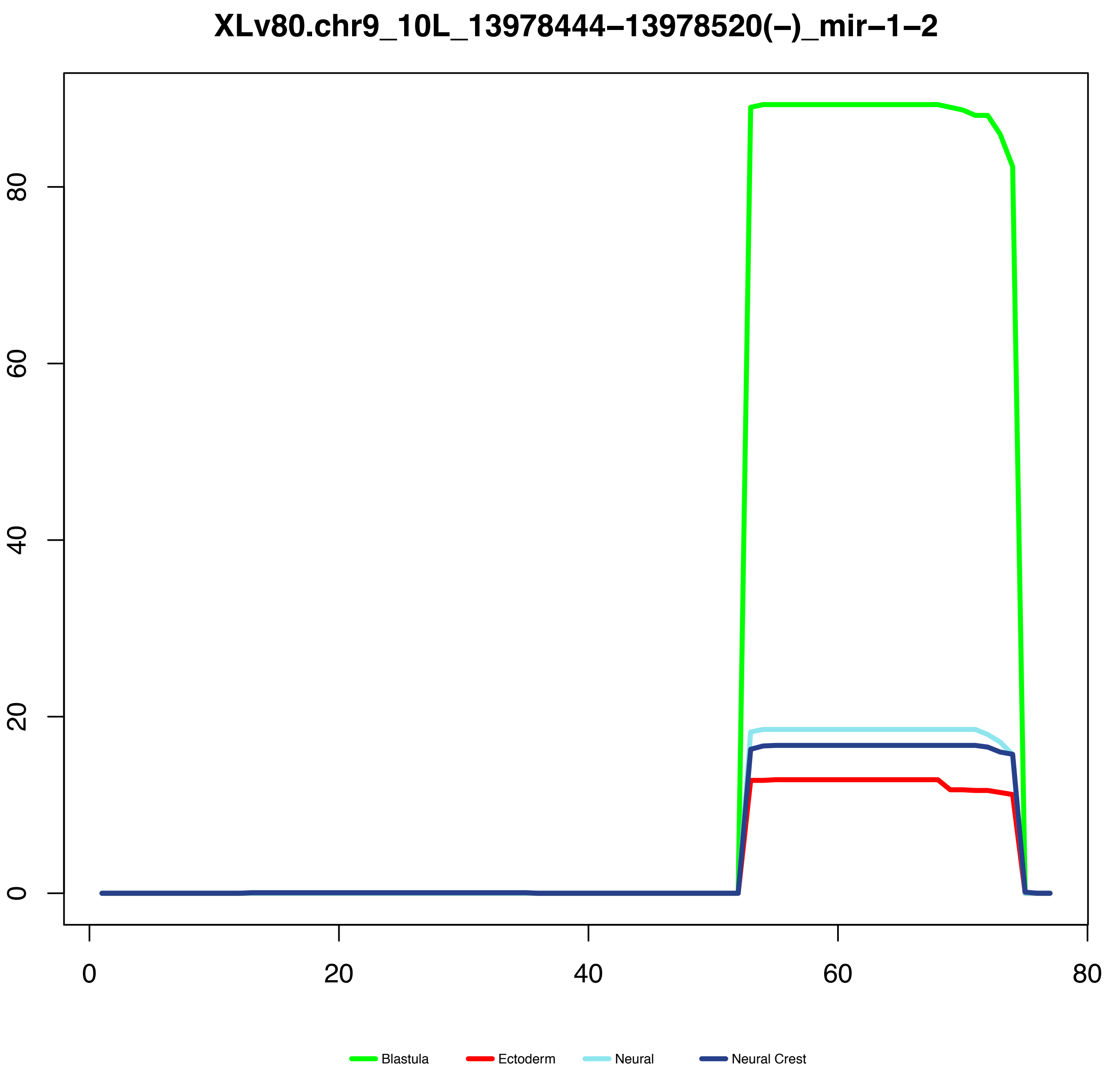

XLv80.chr9_10L_13978444−13978520(−)_mir−1−2
0
20
40
60
80
0
20
40
60
80
Blastula
Ectoderm
Neural
Neural Crest

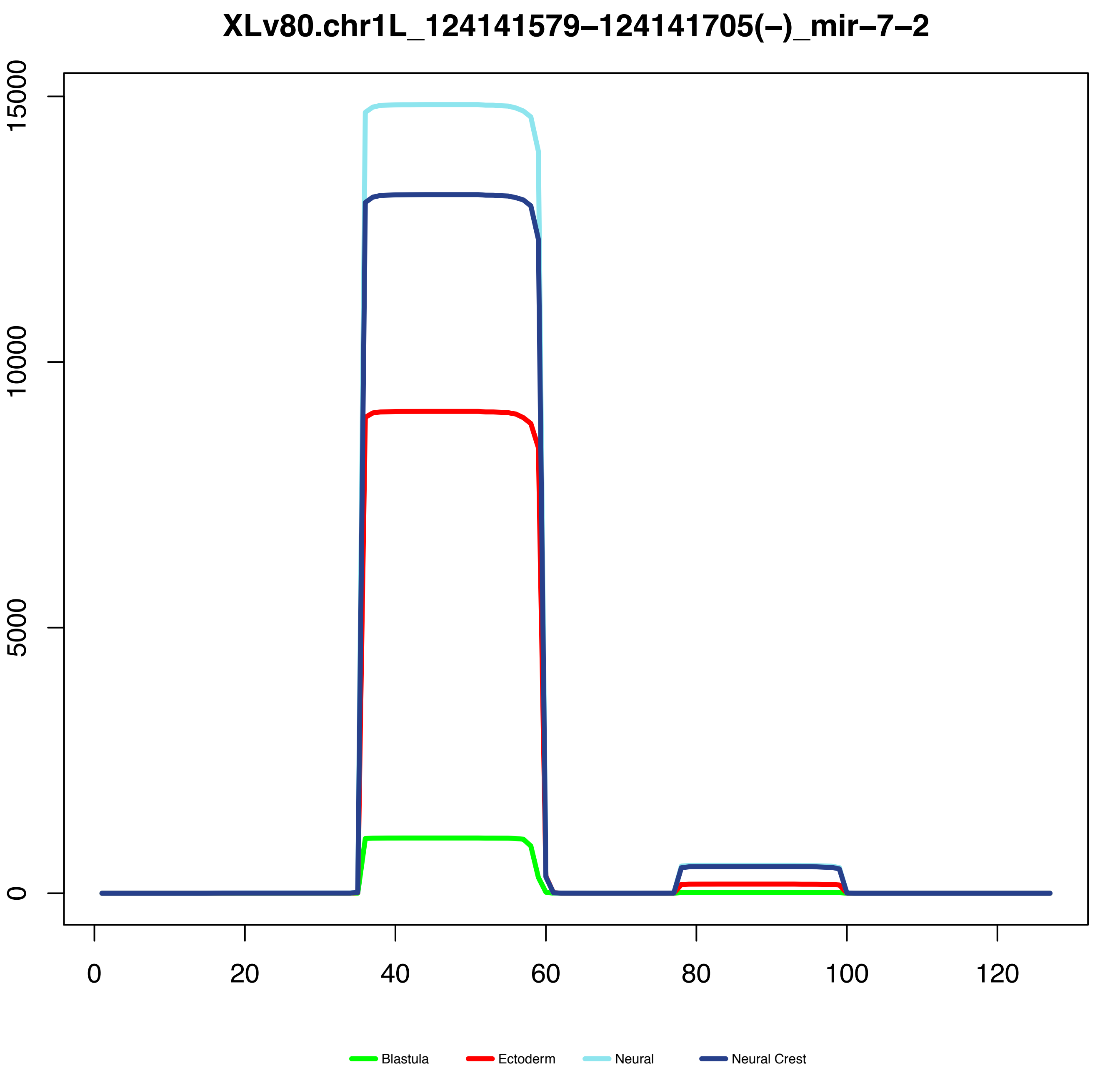
XLv80.chr1L_124141579−124141705(−)_mir−7−2
15000
10000
5000
0
0
20
40
60
80
100
120
Blastula
Ectoderm
Neural
Neural Crest

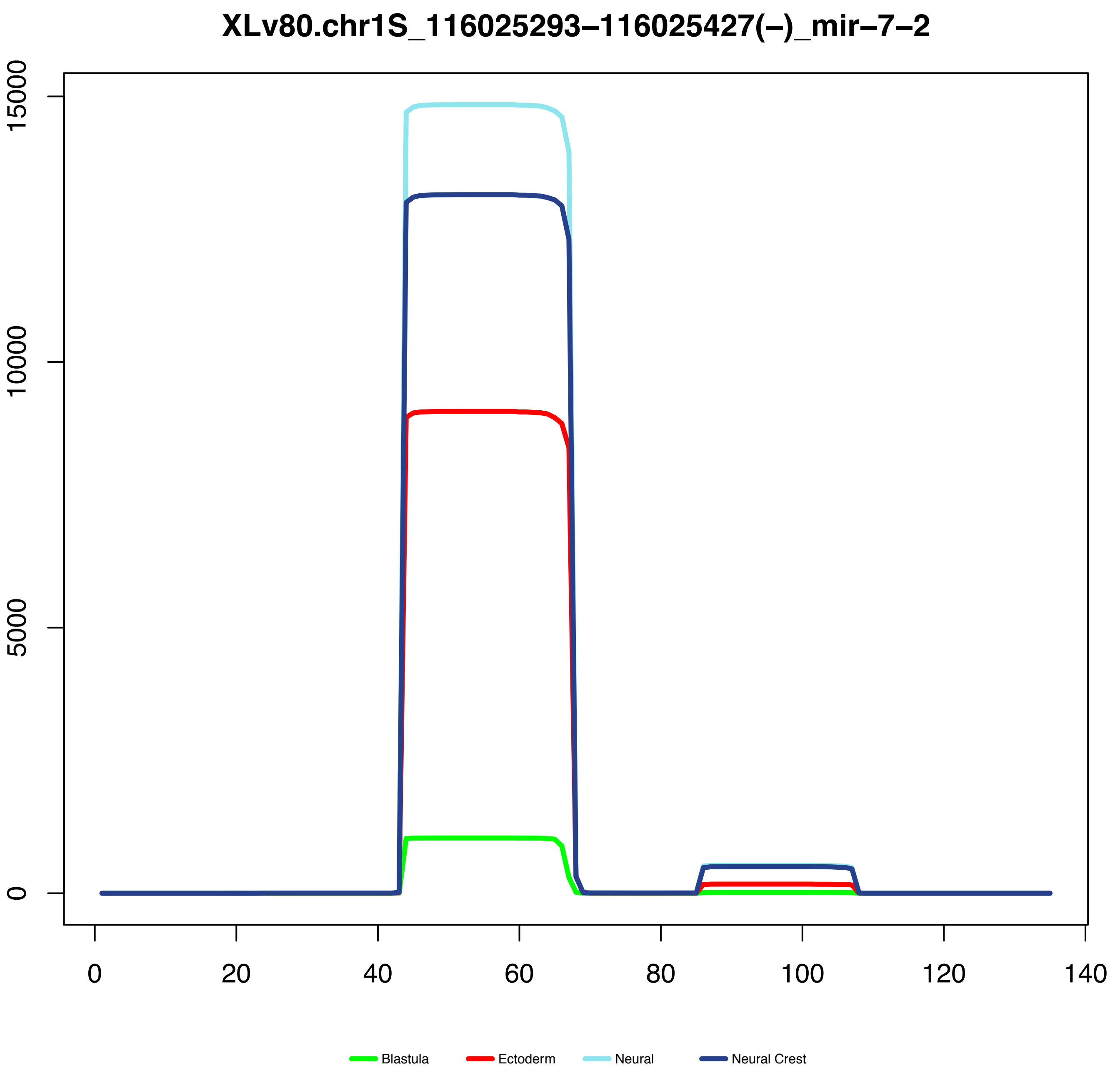
XLv80.chr1S_116025293−116025427(−)_mir−7−2
15000
10000
5000
0
0
20
40
60
80
100
120
140
Blastula
Ectoderm
Neural
Neural Crest

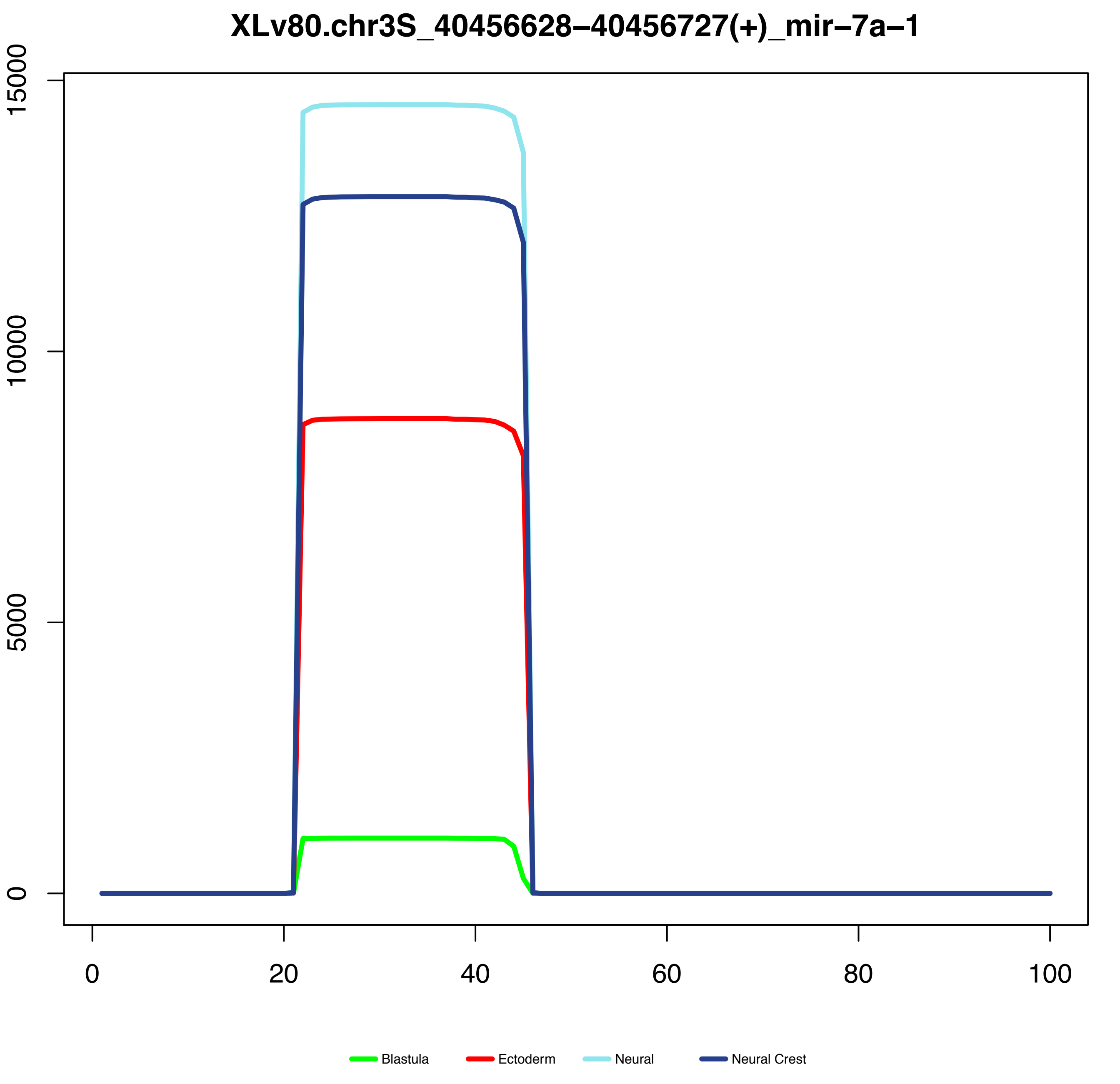
XLv80.chr3S_40456628−40456727(+)_mir−7a−1
15000
10000
5000
0
0
20
40
60
80
100
Blastula
Ectoderm
Neural
Neural Crest

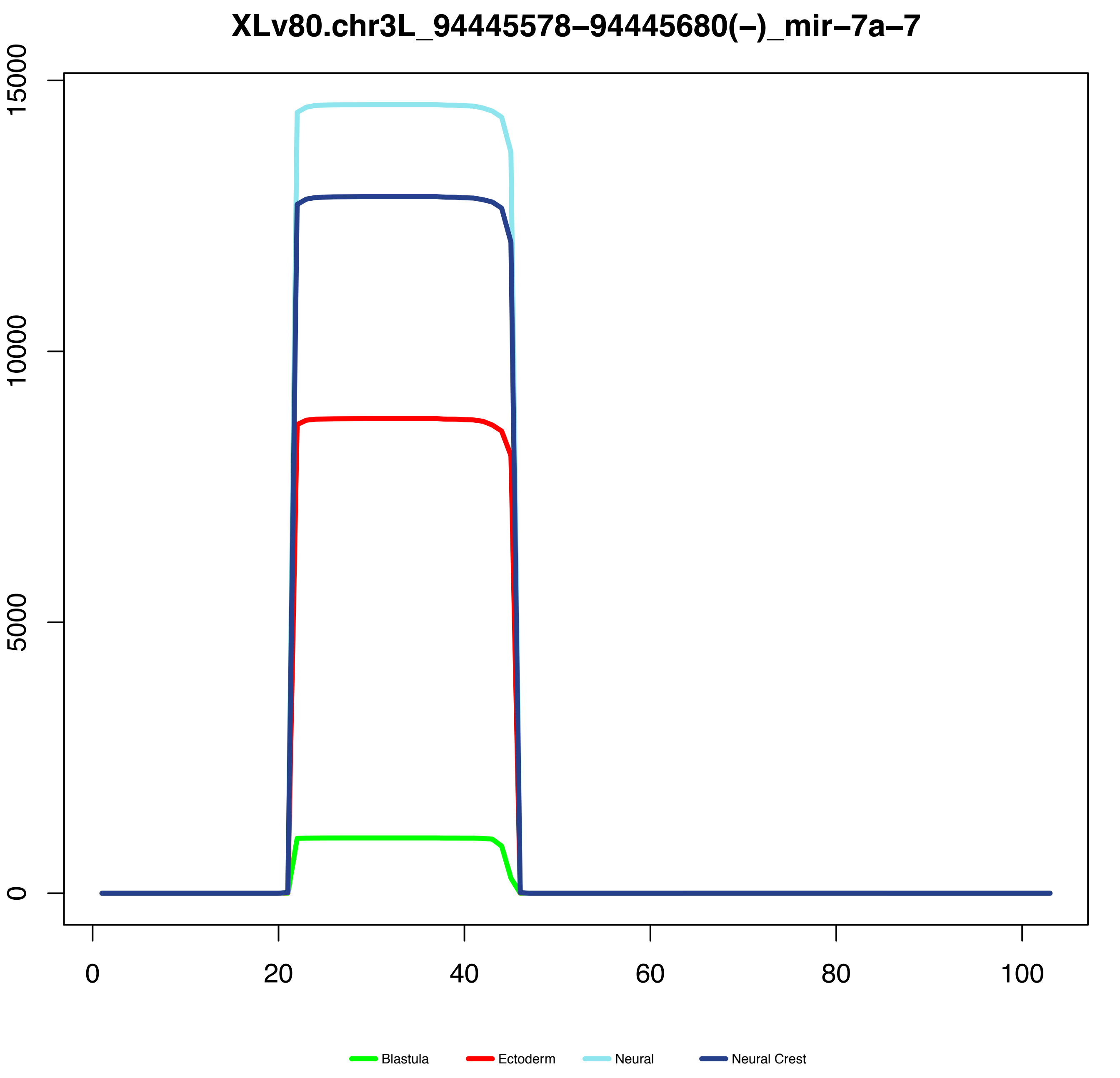
XLv80.chr3L_94445578−94445680(−)_mir−7a−7
15000
10000
5000
0
0
20
40
60
80
100
Blastula
Ectoderm
Neural
Neural Crest

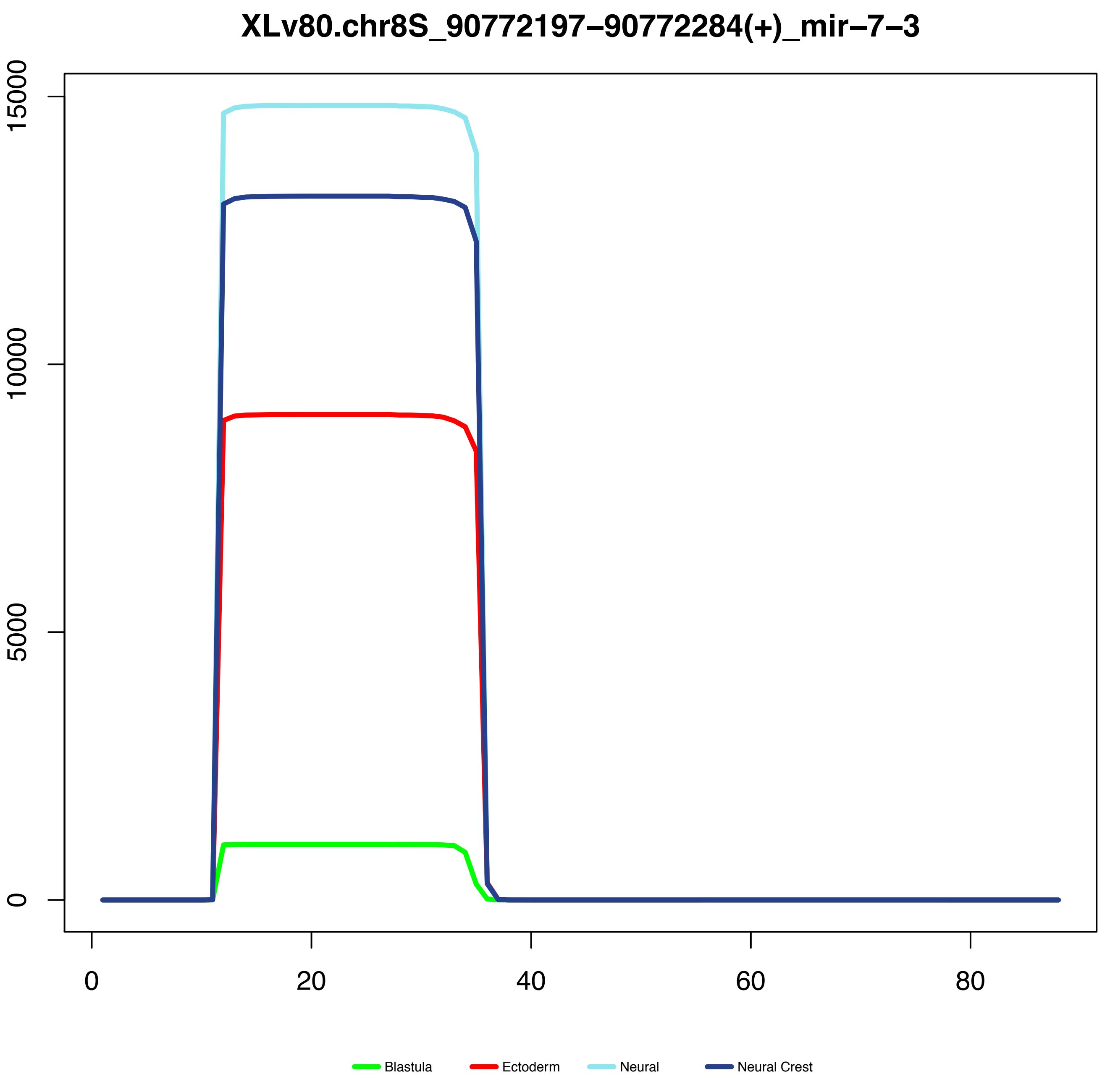
XLv80.chr8S_90772197−90772284(+)_mir−7−3
15000
10000
5000
0
0
20
40
60
80
Blastula
Ectoderm
Neural
Neural Crest

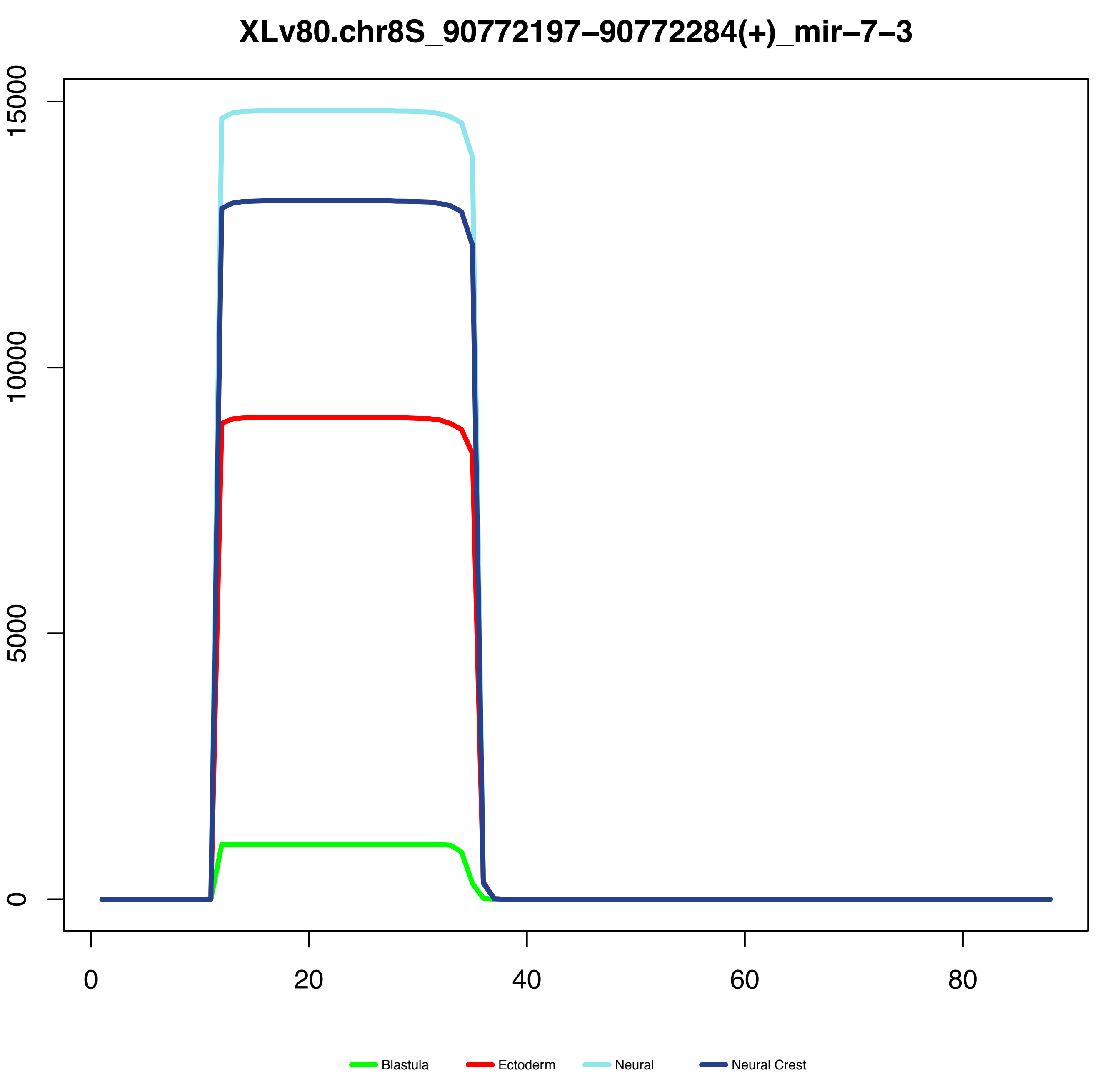
XLv80.chr8S_90772197−90772284(+)_mir−7−3
15000
10000
5000
0
0
20
40
60
80
Blastula
Ectoderm
Neural
Neural Crest

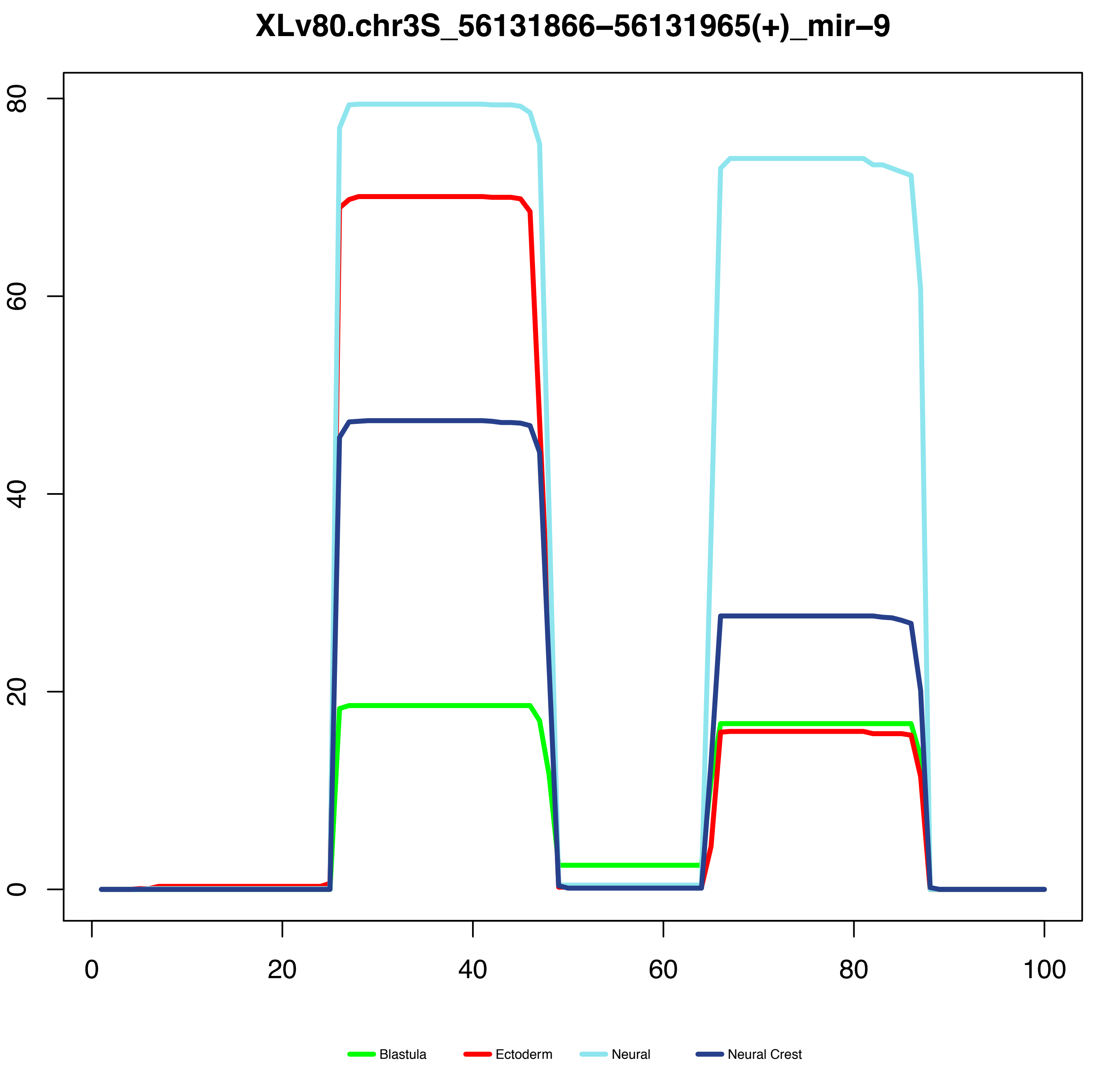

XLv80.chr3S_56131866–56131965(+)_mir–9
0
20
40
60
80
0
20
40
60
80
100
Blastula
Ectoderm
Neural
Neural Crest

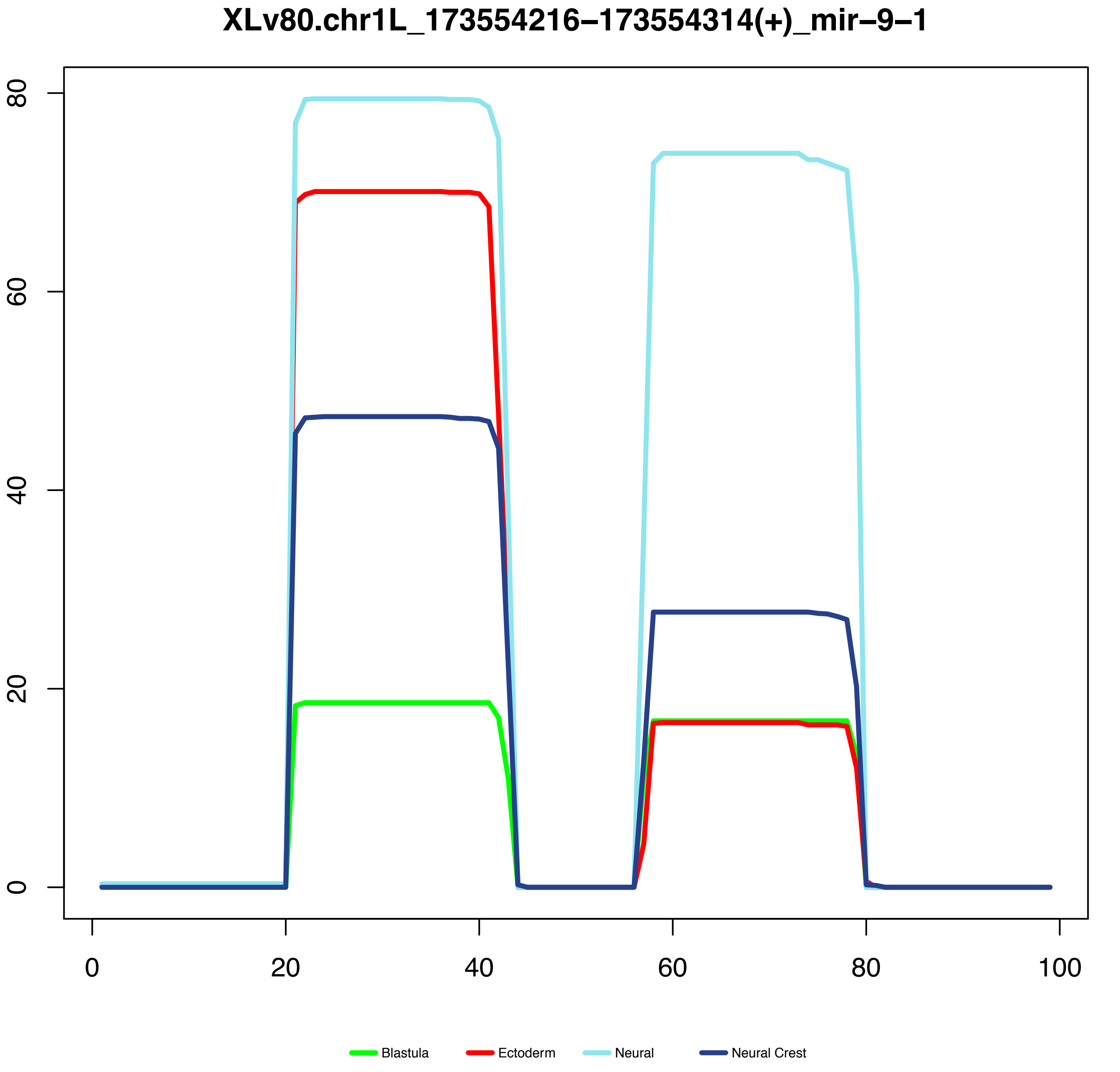

XLv80.chr1L_173554216−173554314(+)_mir−9−1
0
20
40
60
80
0
20
40
60
80
100
Blastula
Ectoderm
Neural
Neural Crest

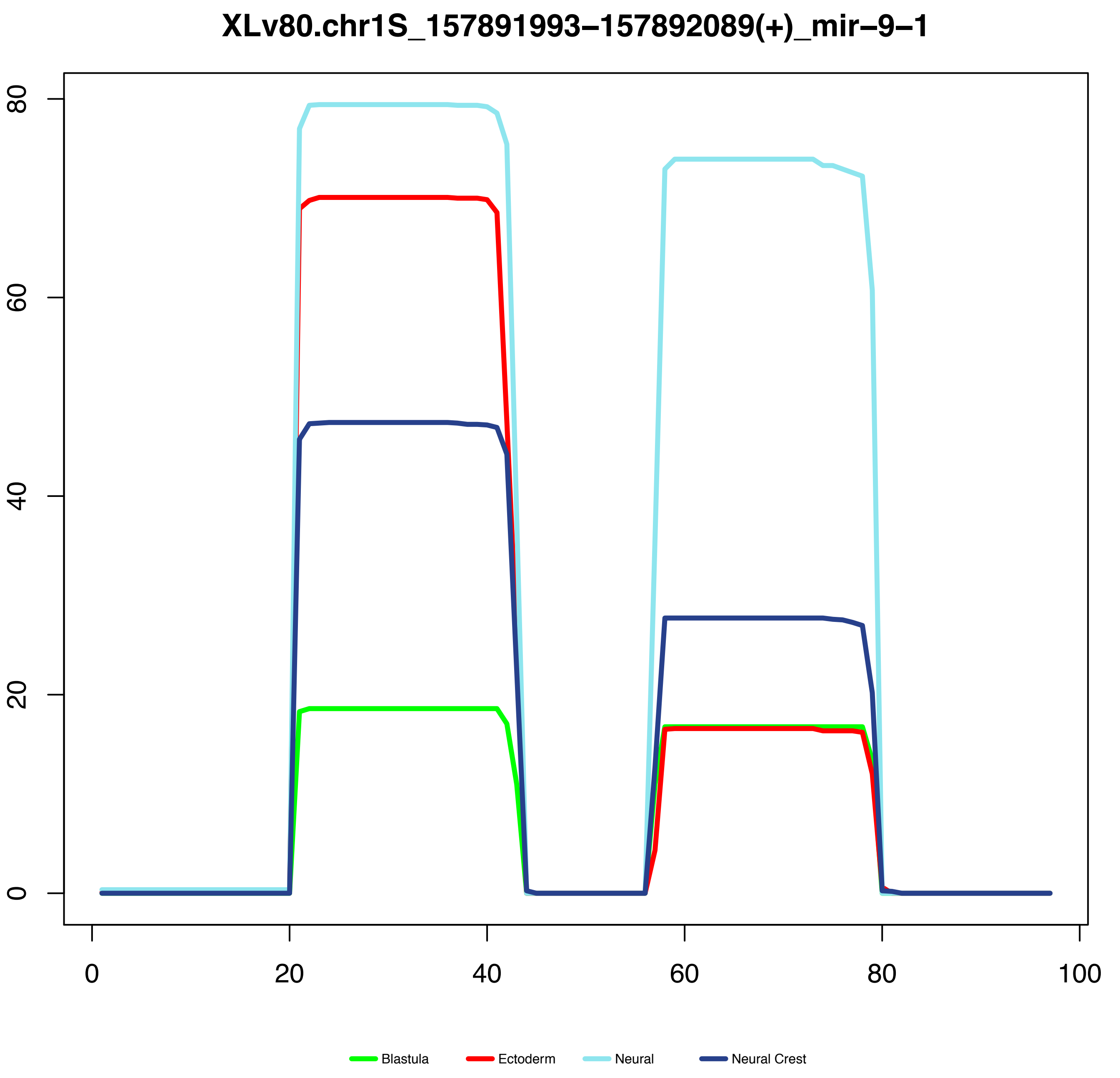

XLv80.chr1S_157891993−157892089(+)_mir−9−1
0
20
40
60
80
0
20
40
60
80
100
Blastula
Ectoderm
Neural
Neural Crest

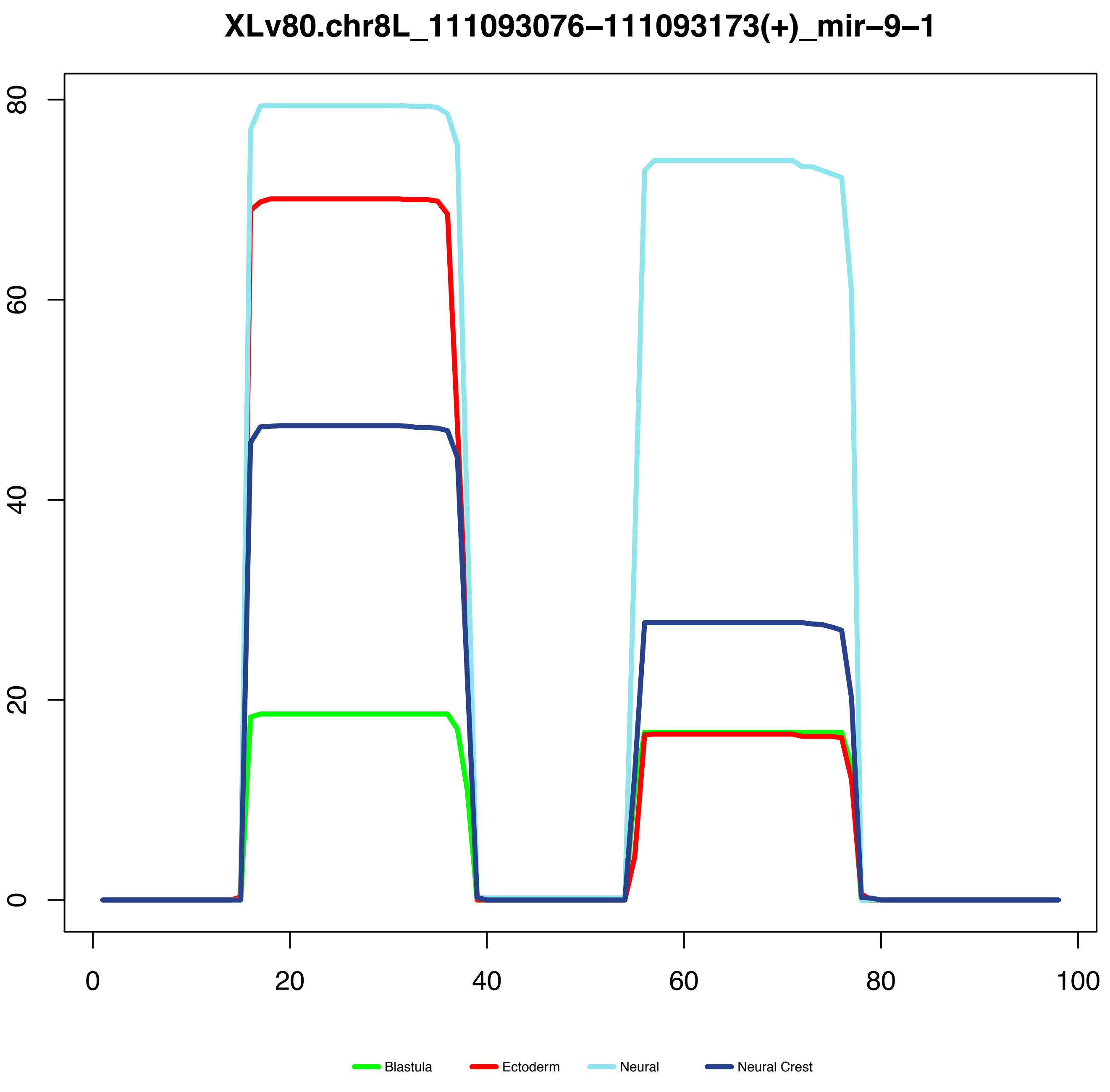

XLv80.chr8L_111093076−111093173(+)_mir−9−1
0
20
40
60
80
0
20
40
60
80
100
Blastula
Ectoderm
Neural
Neural Crest

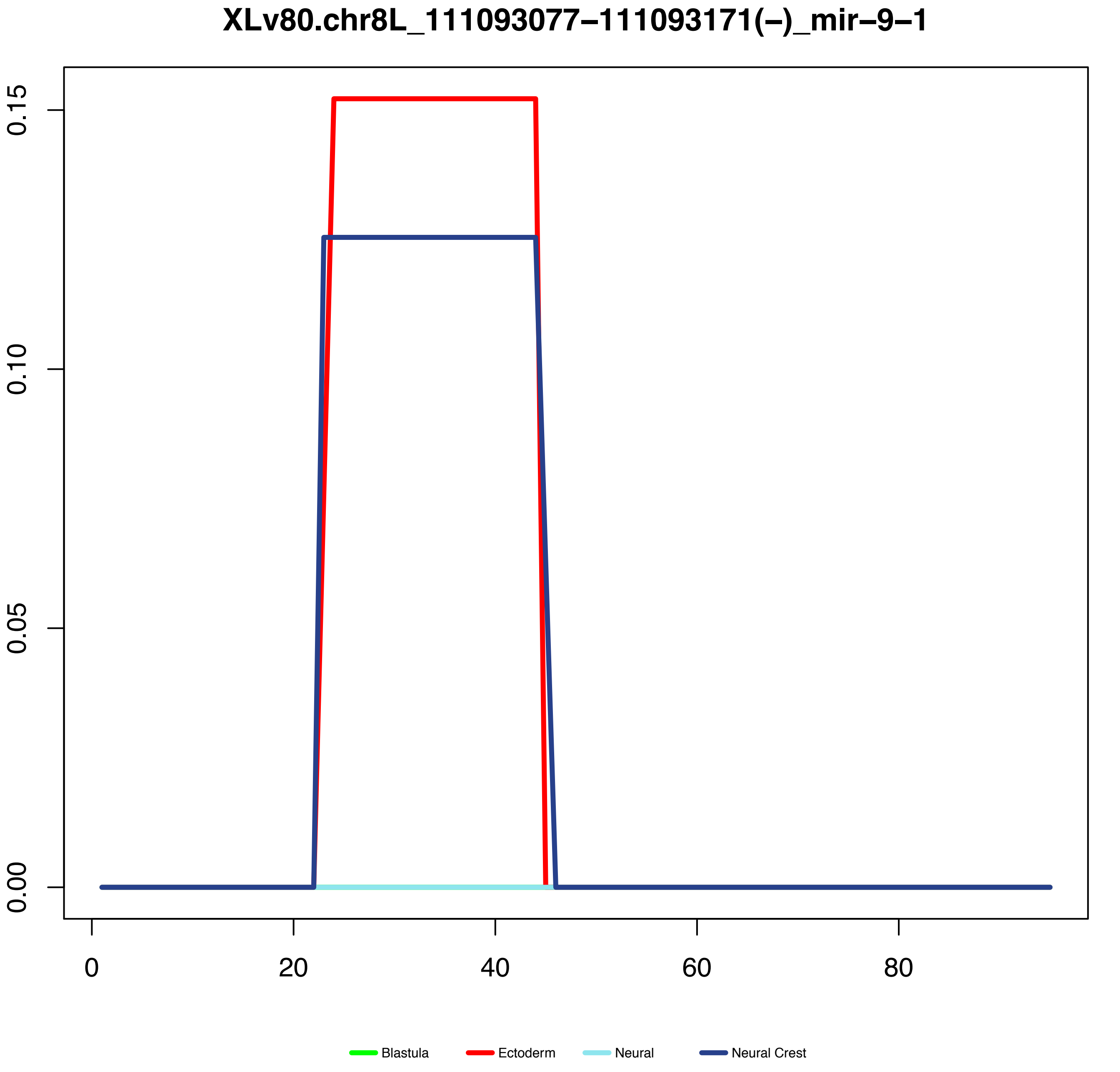
XLv80.chr8L_111093077−111093171(−)_mir−9−1
0.15
0.10
0.05
0.00
0
20
40
60
80
Blastula
Ectoderm
Neural
Neural Crest

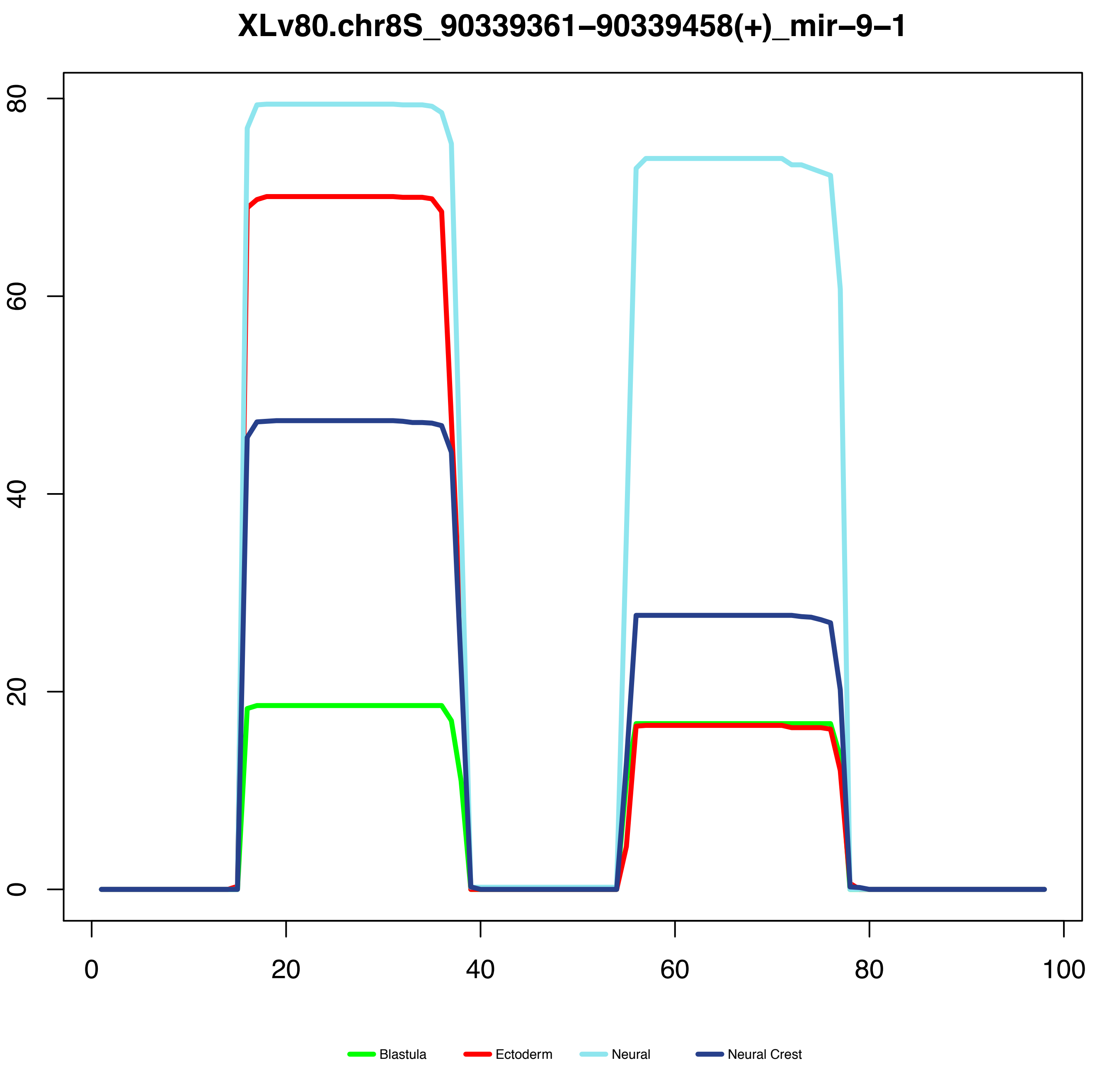

XLv80.chr8S_90339361−90339458(+)_mir−9−1
0
20
40
60
80
0
20
40
60
80
100
Blastula
Ectoderm
Neural
Neural Crest

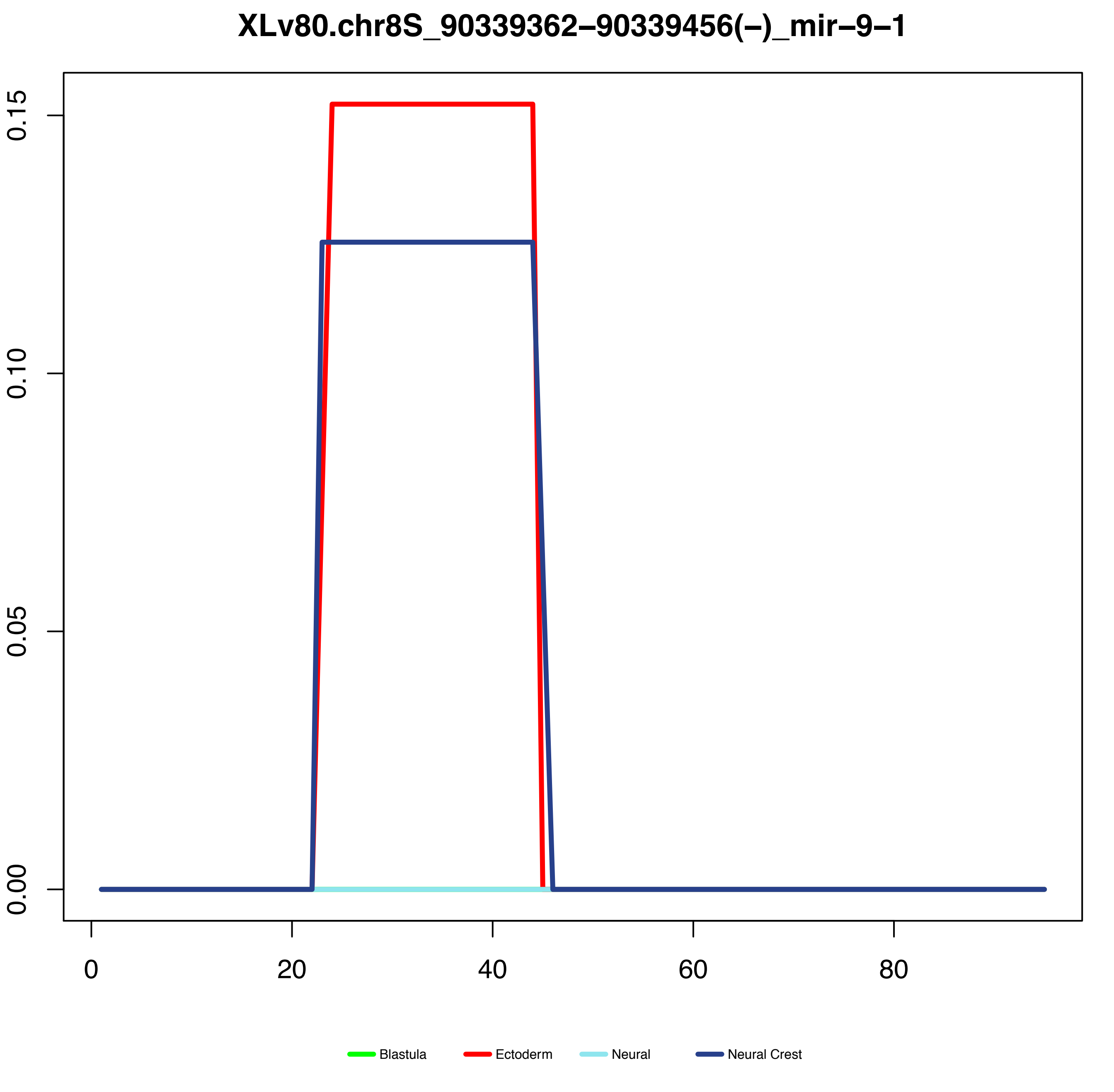
XLv80.chr8S_90339362−90339456(−)_mir−9−1
0.00
0.05
0.10
0.15
0
20
40
60
80
Blastula
Ectoderm
Neural
Neural Crest

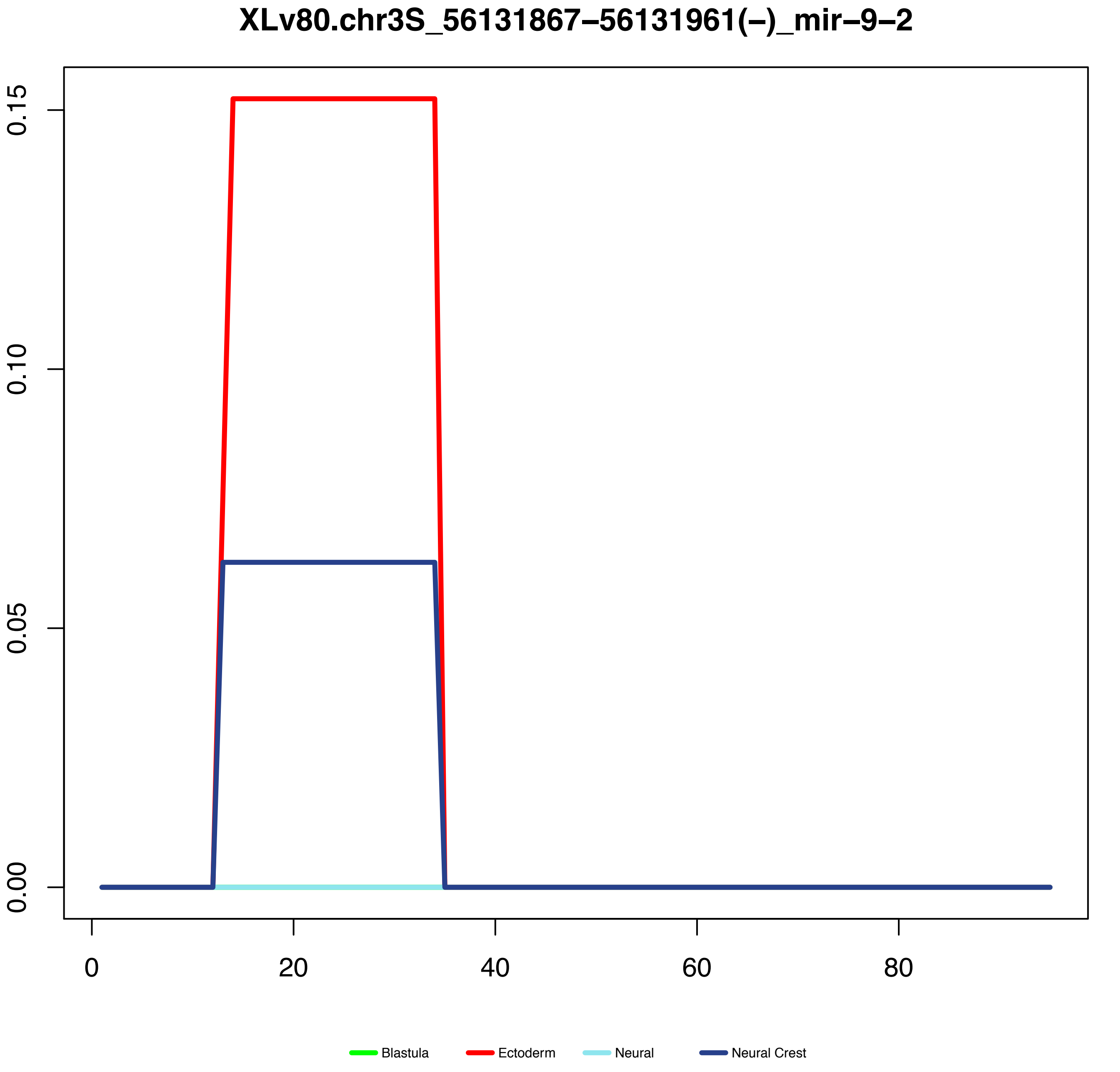
XLv80.chr3S_56131867–56131961(–)_mir–9–2
0.00
0.05
0.10
0.15
0
20
40
60
80
Blastula
Ectoderm
Neural
Neural Crest

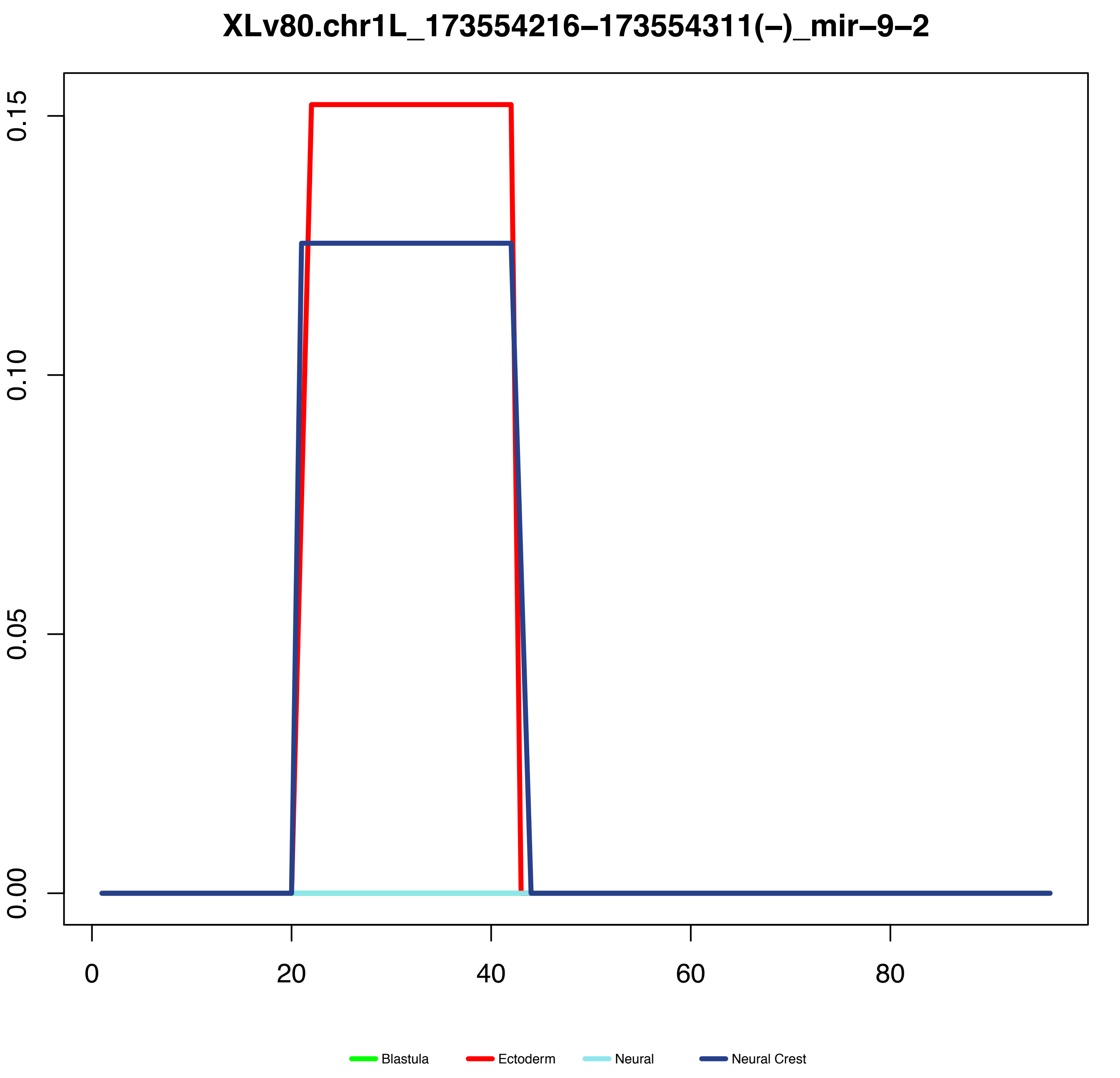
XLv80.chr1L_173554216−173554311(−)_mir−9−2
0.00
0.05
0.10
0.15
0
20
40
60
80
Blastula
Ectoderm
Neural
Neural Crest

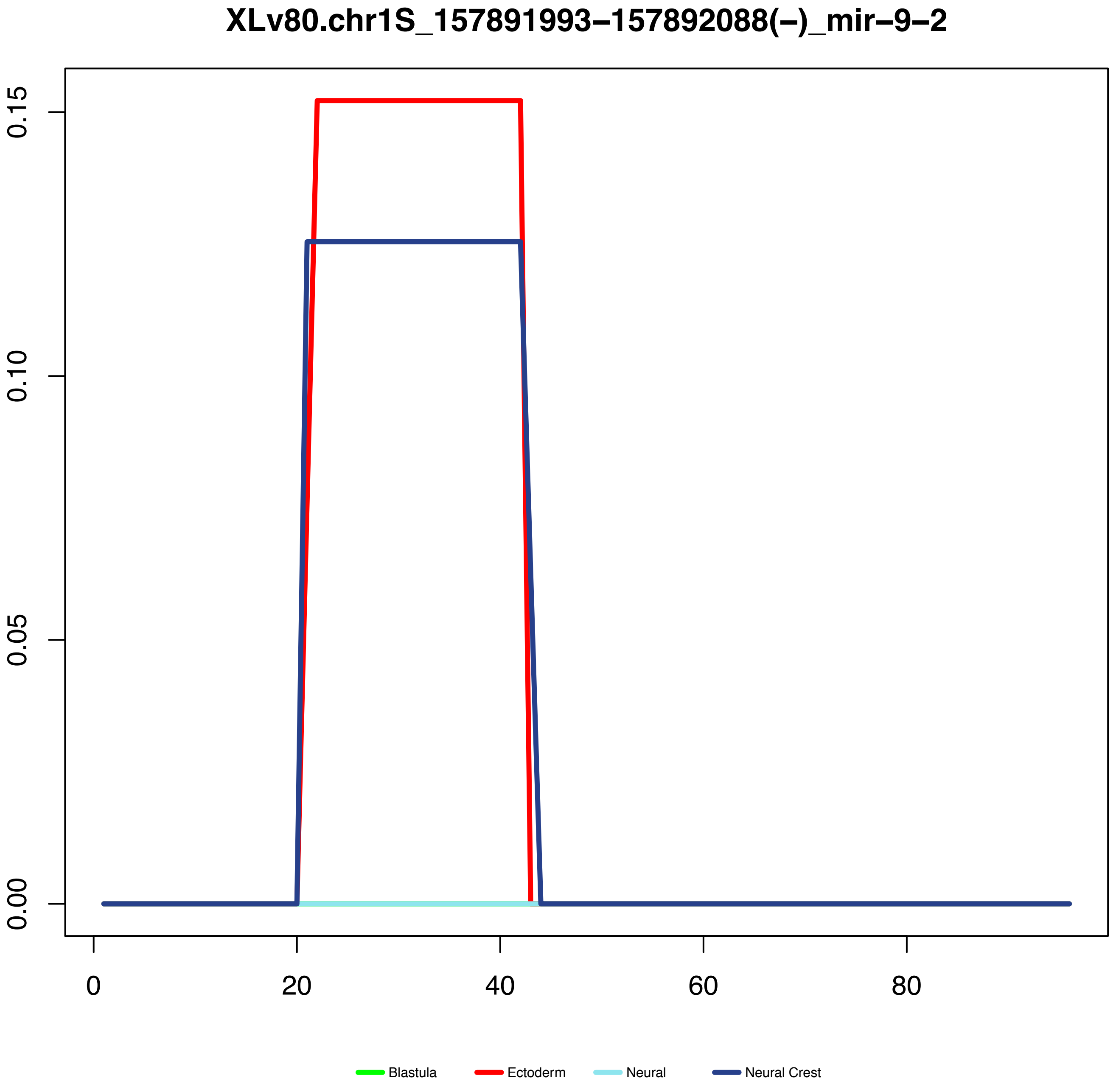
XLv80.chr1S_157891993−157892088(−)_mir−9−2
0.00
0.05
0.10
0.15
0
20
40
60
80
Blastula
Ectoderm
Neural
Neural Crest

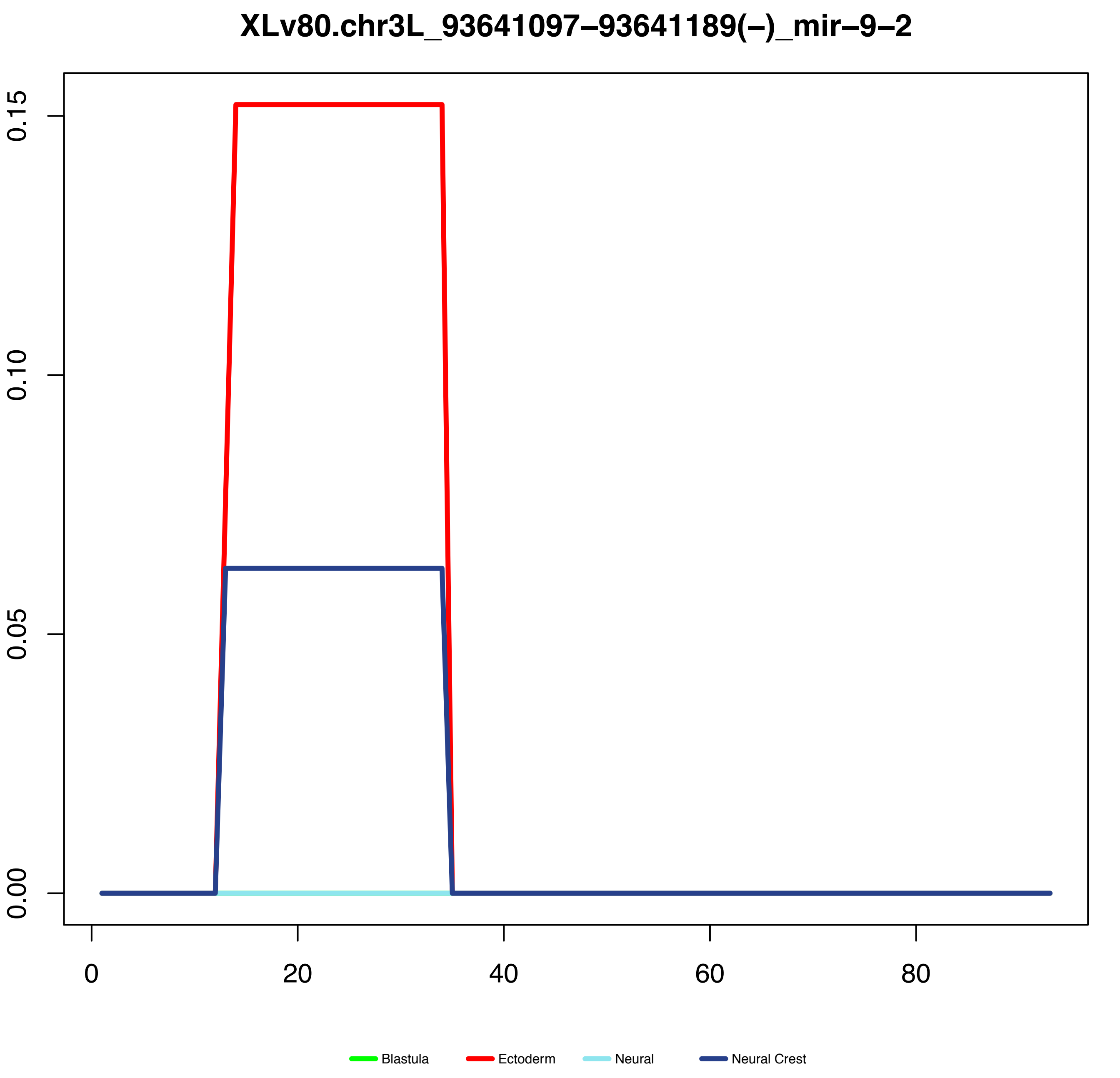

XLv80.chr3L_93641097−93641189(−)_mir−9−2
0.00
0.05
0.10
0.15
0
20
40
60
80
Blastula
Ectoderm
Neural
Neural Crest

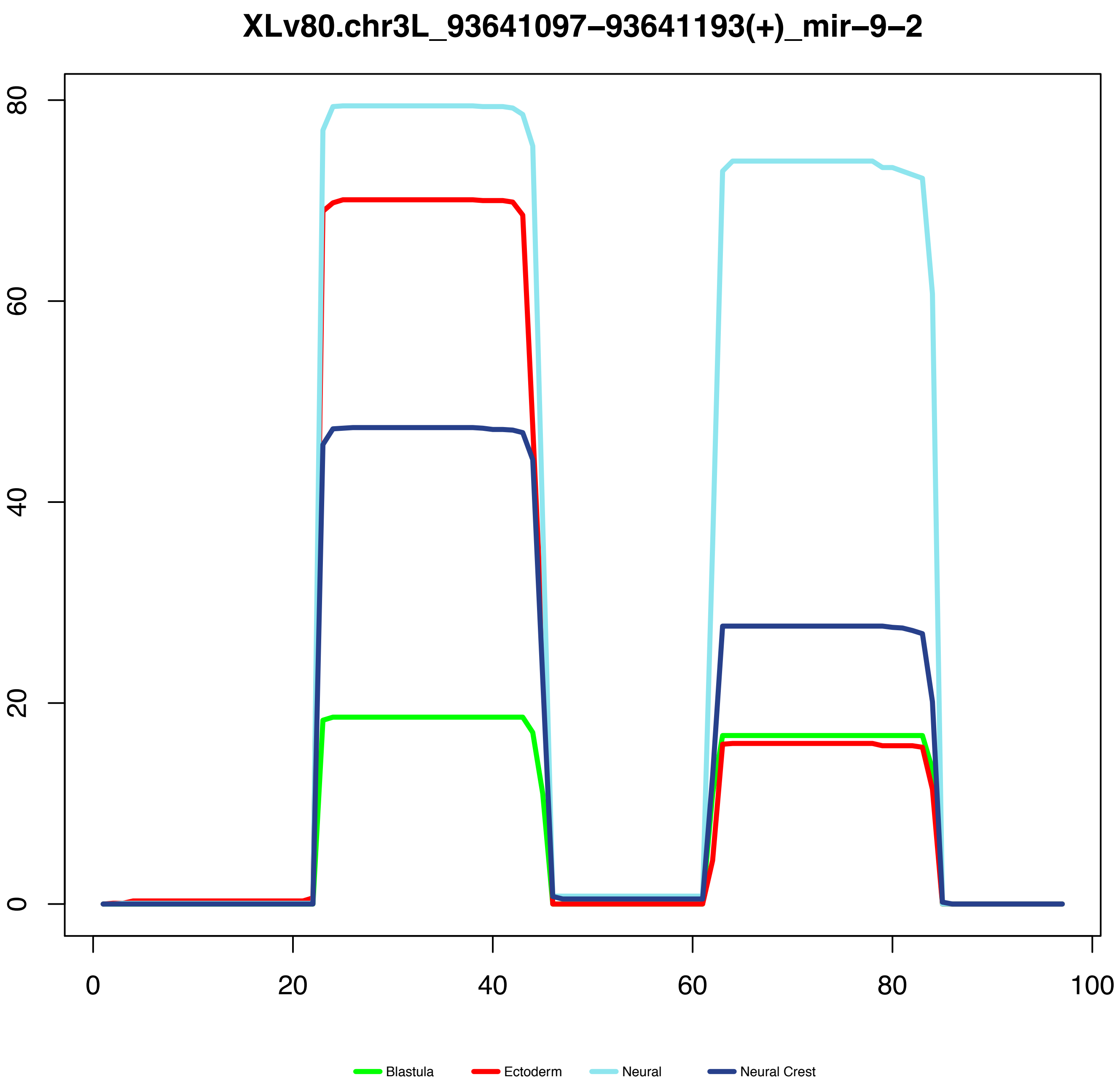

XLv80.chr3L_93641097−93641193(+)_mir−9−2
0
20
40
60
80
0
20
40
60
80
100
Blastula
Ectoderm
Neural
Neural Crest

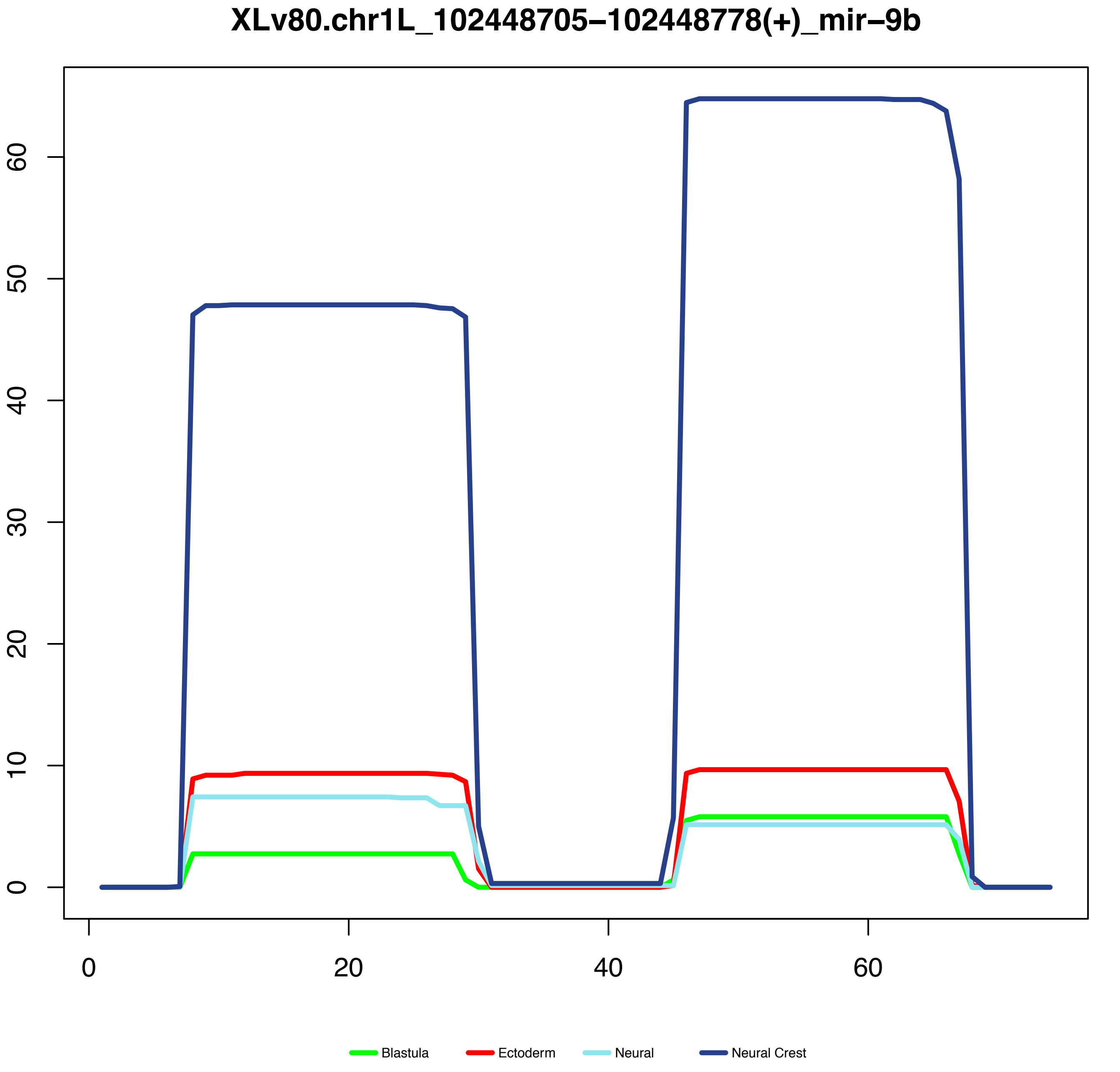
XLv80.chr1L_102448705−102448778(+)_mir−9b
0
10
20
30
40
50
60
0
20
40
60
Blastula
Ectoderm
Neural
Neural Crest

XLv80.chr1S_96896963−96897036(+)_mir−9b

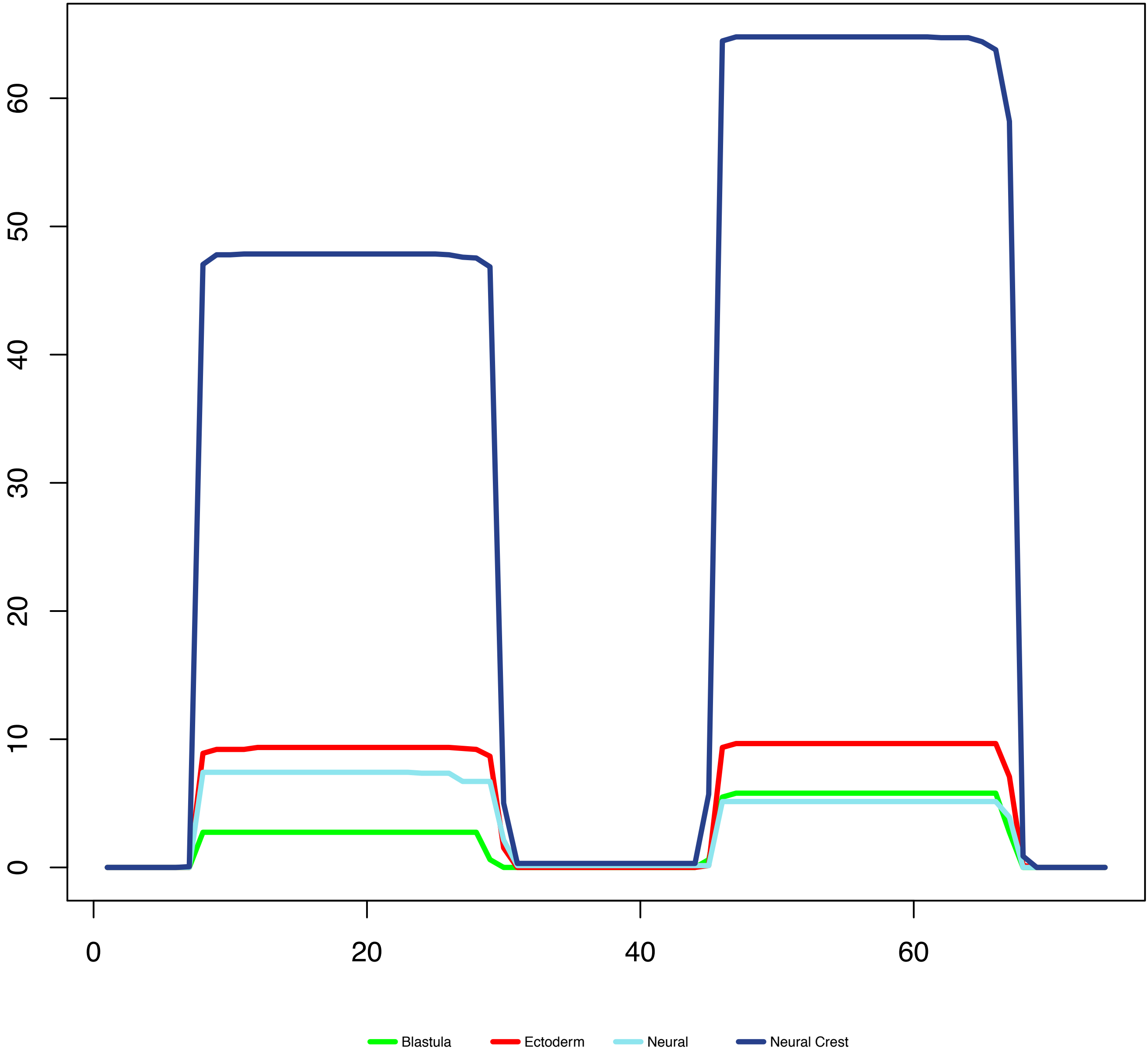

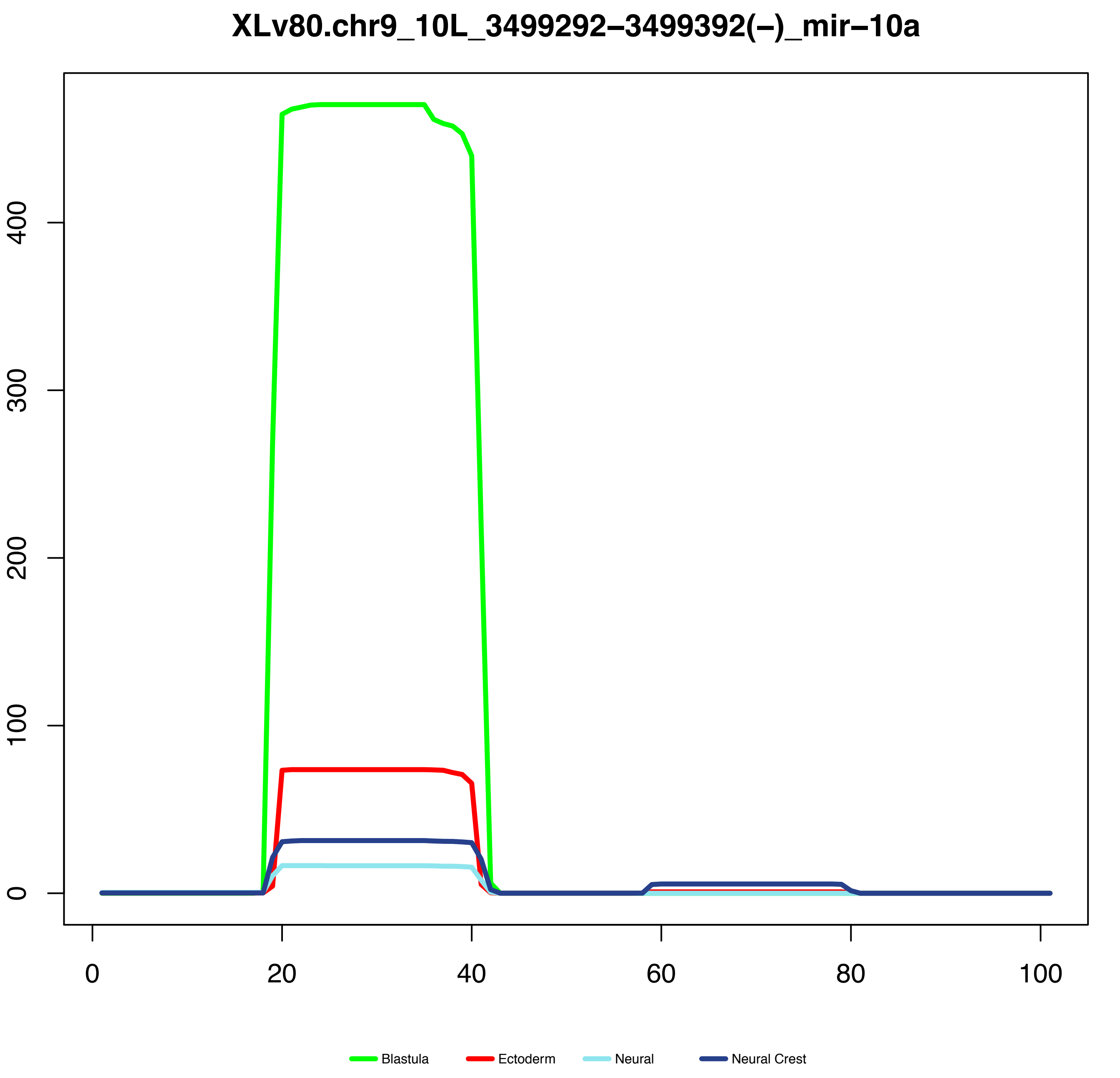

XLv80.chr9_10L_3499292–3499392(–)_mir–10a
0
100
200
300
400
0
20
40
60
80
100
Blastula
Ectoderm
Neural
Neural Crest

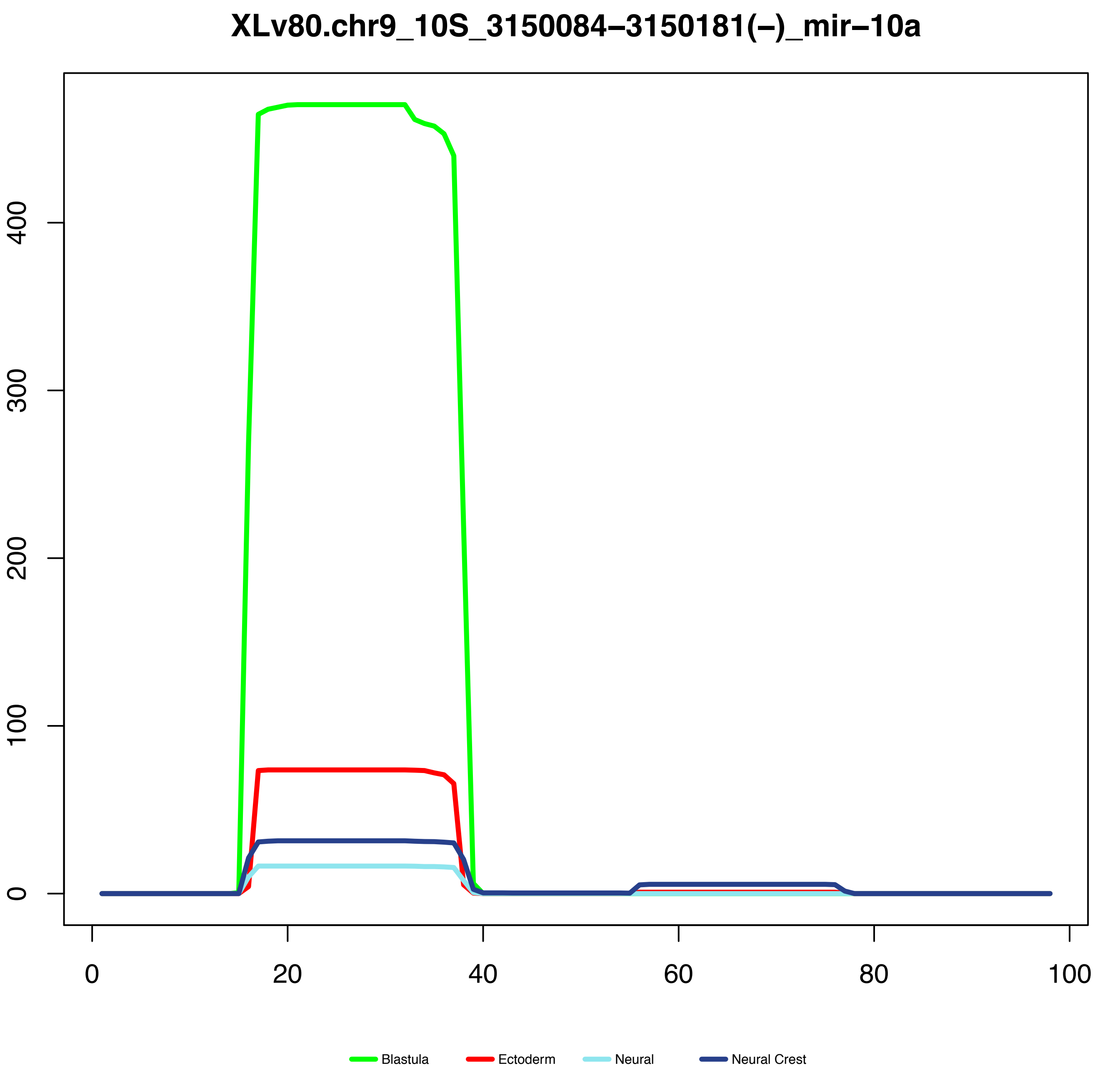

XLv80.chr9_10S_3150084−3150181(−)_mir−10a
0
100
200
300
400
0
20
40
60
80
100
Blastula
Ectoderm
Neural
Neural Crest

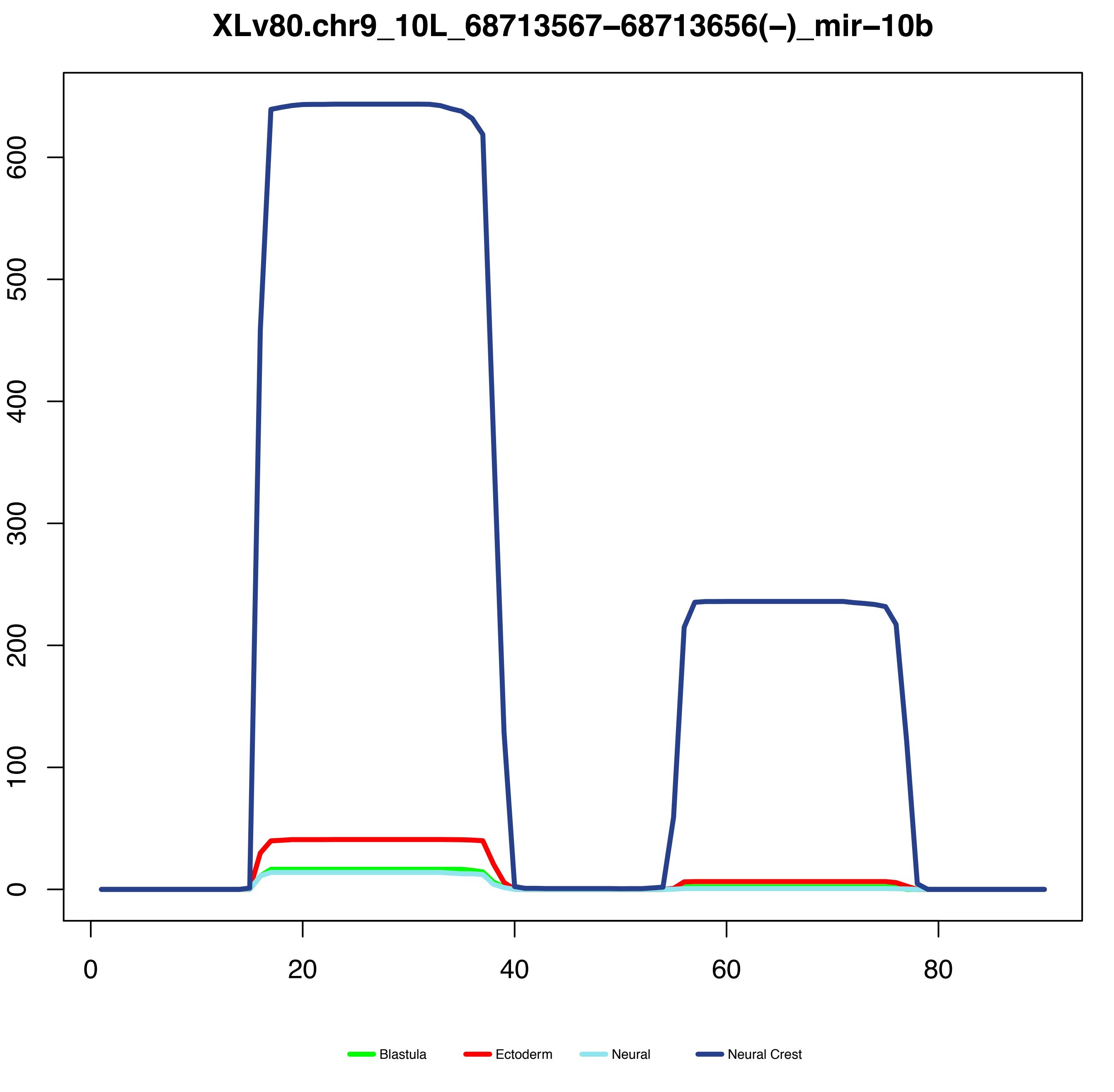

XLv80.chr9_10L_68713567−68713656(−)_mir−10b
0
100
200
300
400
500
600
0
20
40
60
80
Blastula
Ectoderm
Neural
Neural Crest

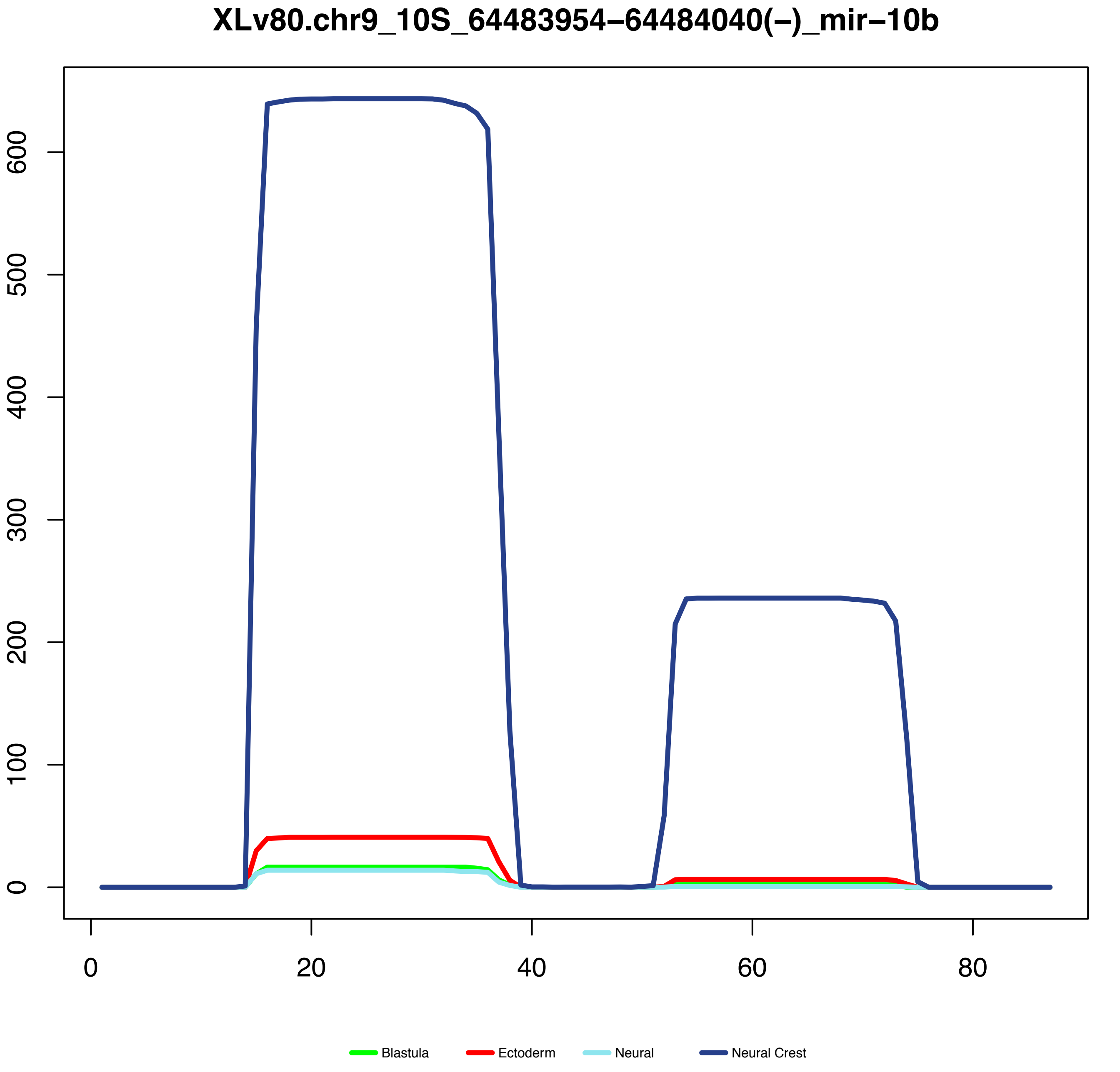

XLv80.chr9_10S_64483954−64484040(−)_mir−10b
0
100
200
300
400
500
600
0
20
40
60
80
Blastula
Ectoderm
Neural
Neural Crest

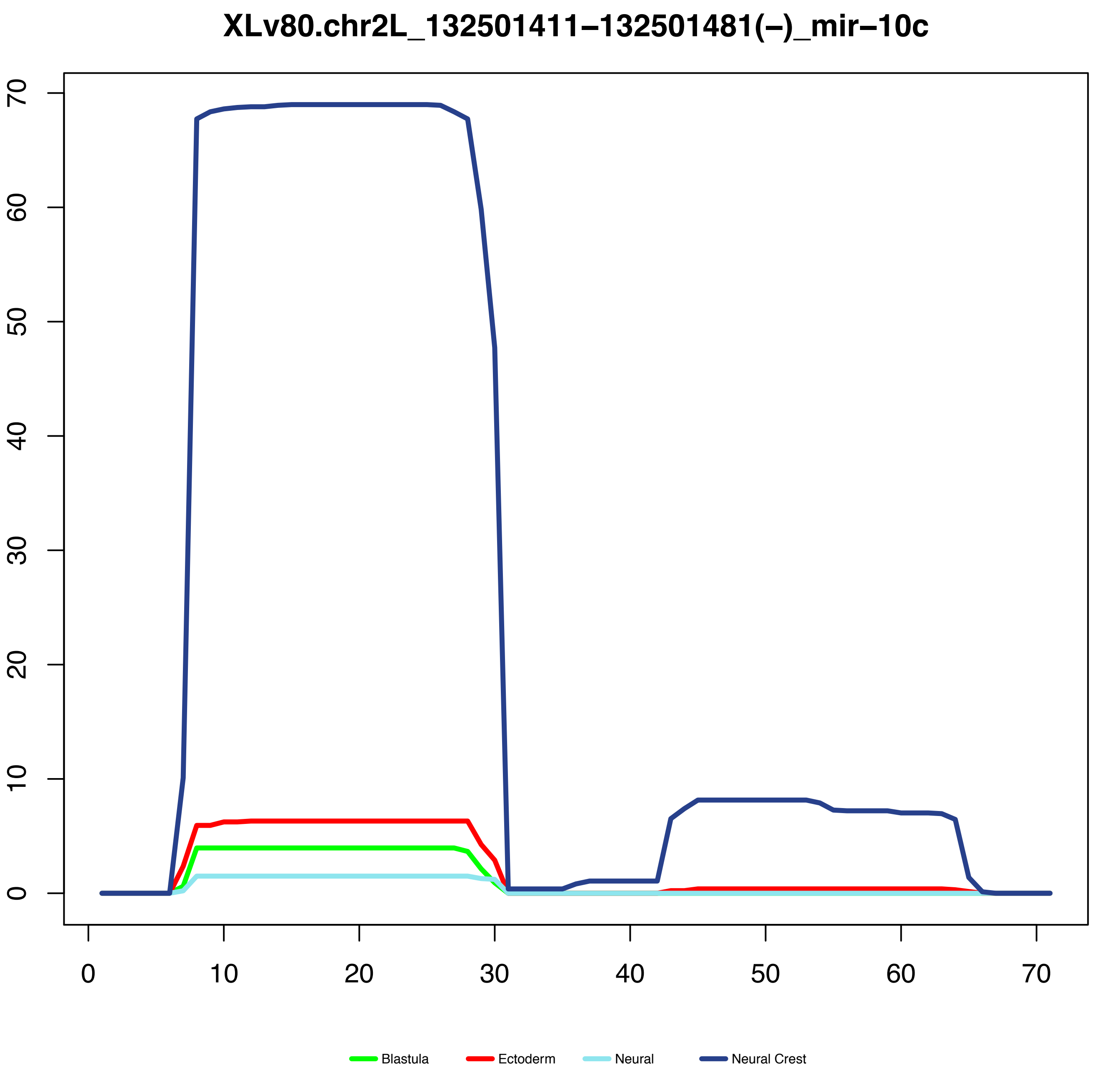
XLv80.chr2L_132501411−132501481(−)_mir−10c
0
10
20
30
40
50
60
70
Blastula
Ectoderm
Neural
Neural Crest

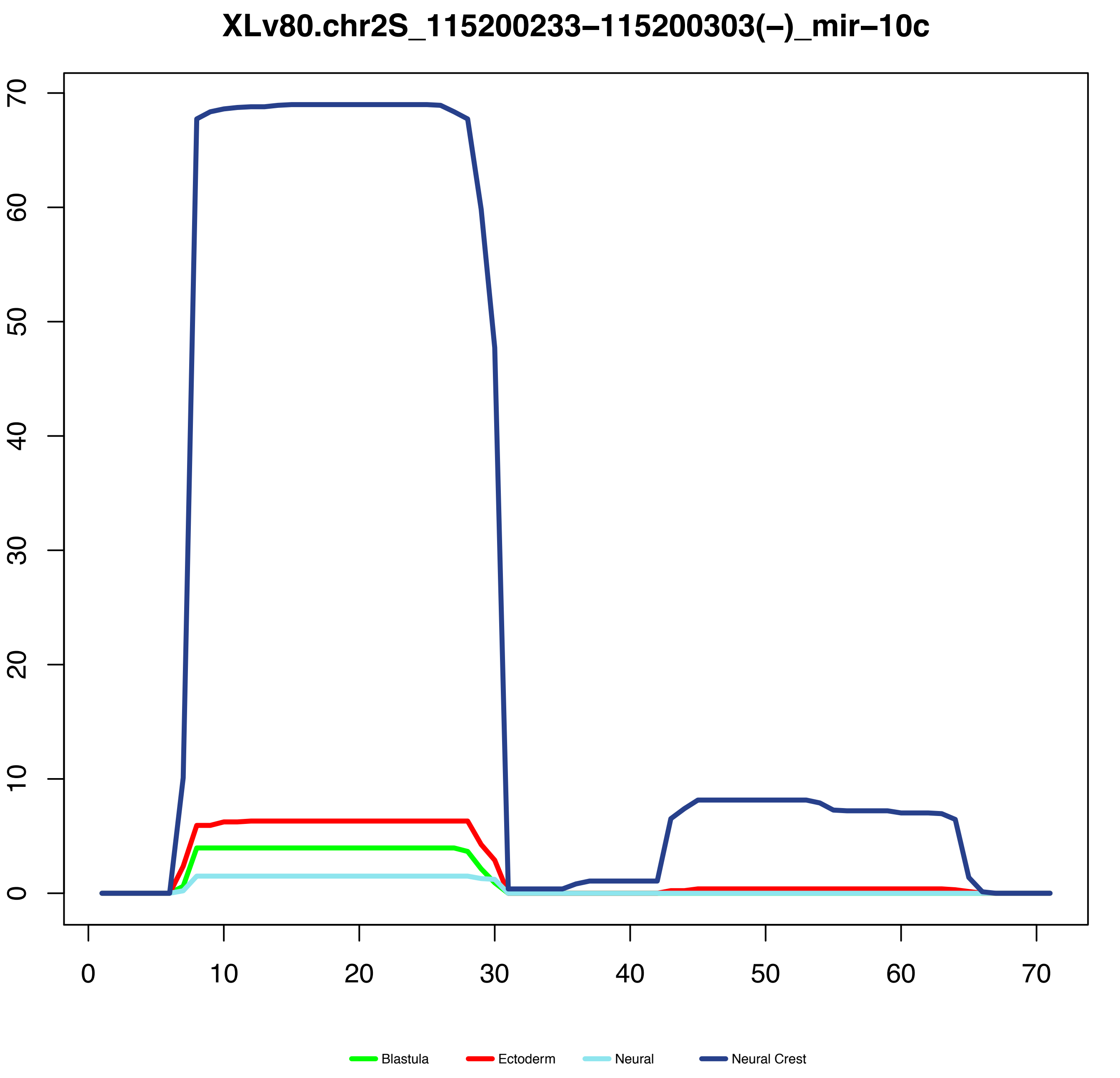
XLv80.chr2S_115200233−115200303(−)_mir−10c
0
10
20
30
40
50
60
70
0
10
20
30
40
50
60
70
Blastula
Ectoderm
Neural
Neural Crest

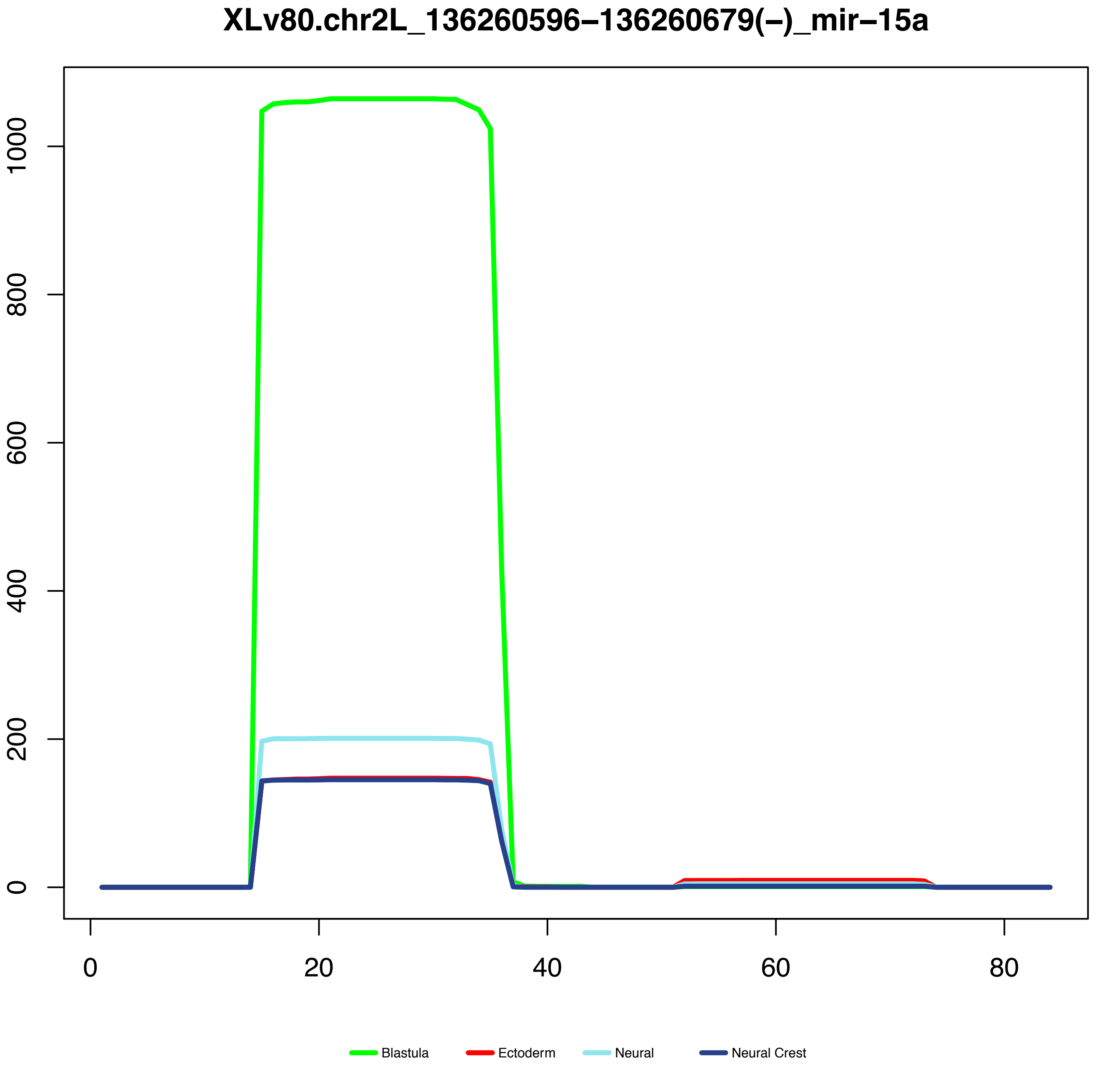
XLv80.chr2L_136260596–136260679(–)_mir–15a
0
200
400
600
800
1000
0
20
40
60
80
Blastula
Ectoderm
Neural
Neural Crest

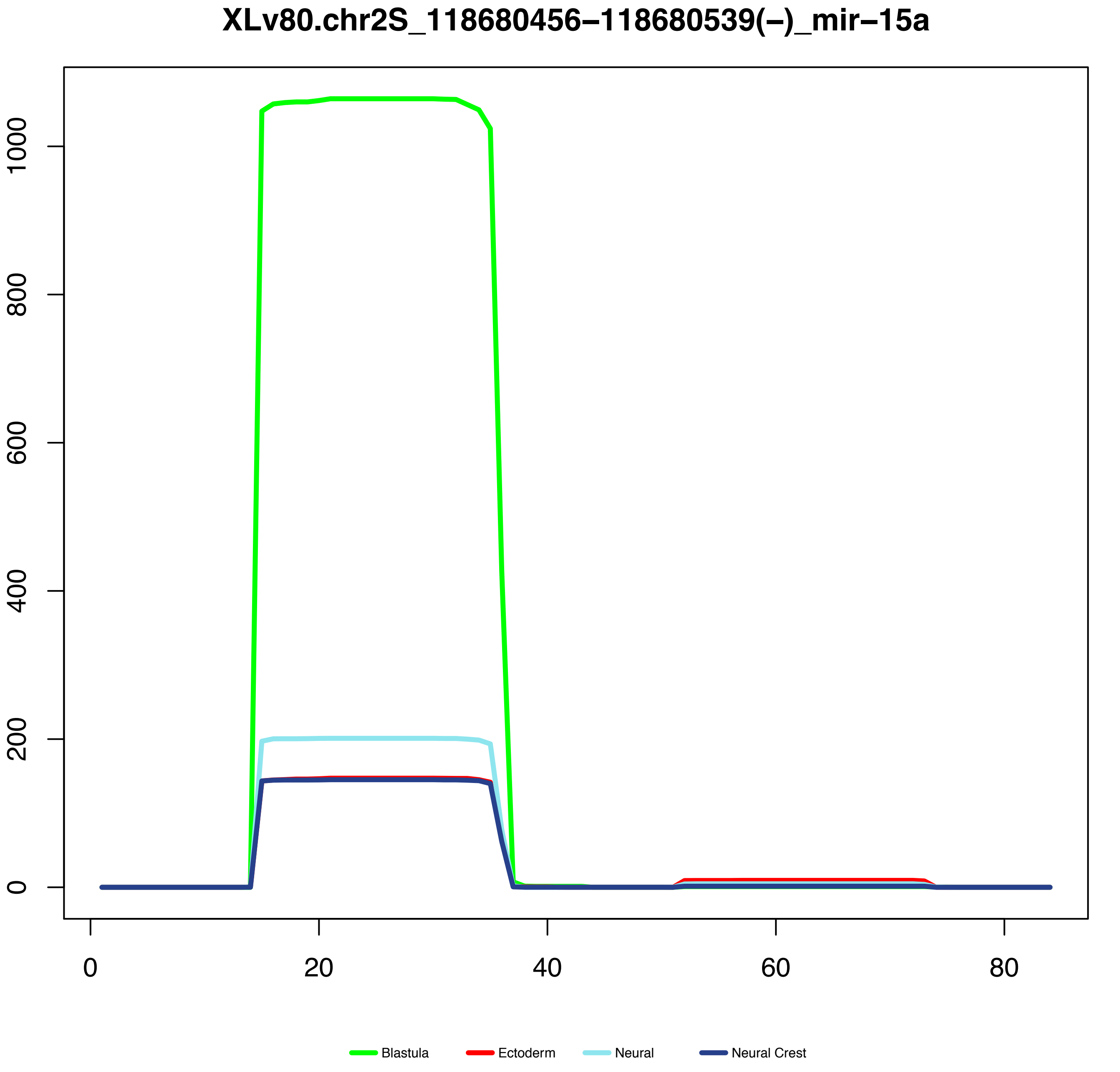
XLv80.chr2S_118680456−118680539(−)_mir−15a
0
200
400
600
800
1000
0
20
40
60
80
Blastula
Ectoderm
Neural
Neural Crest

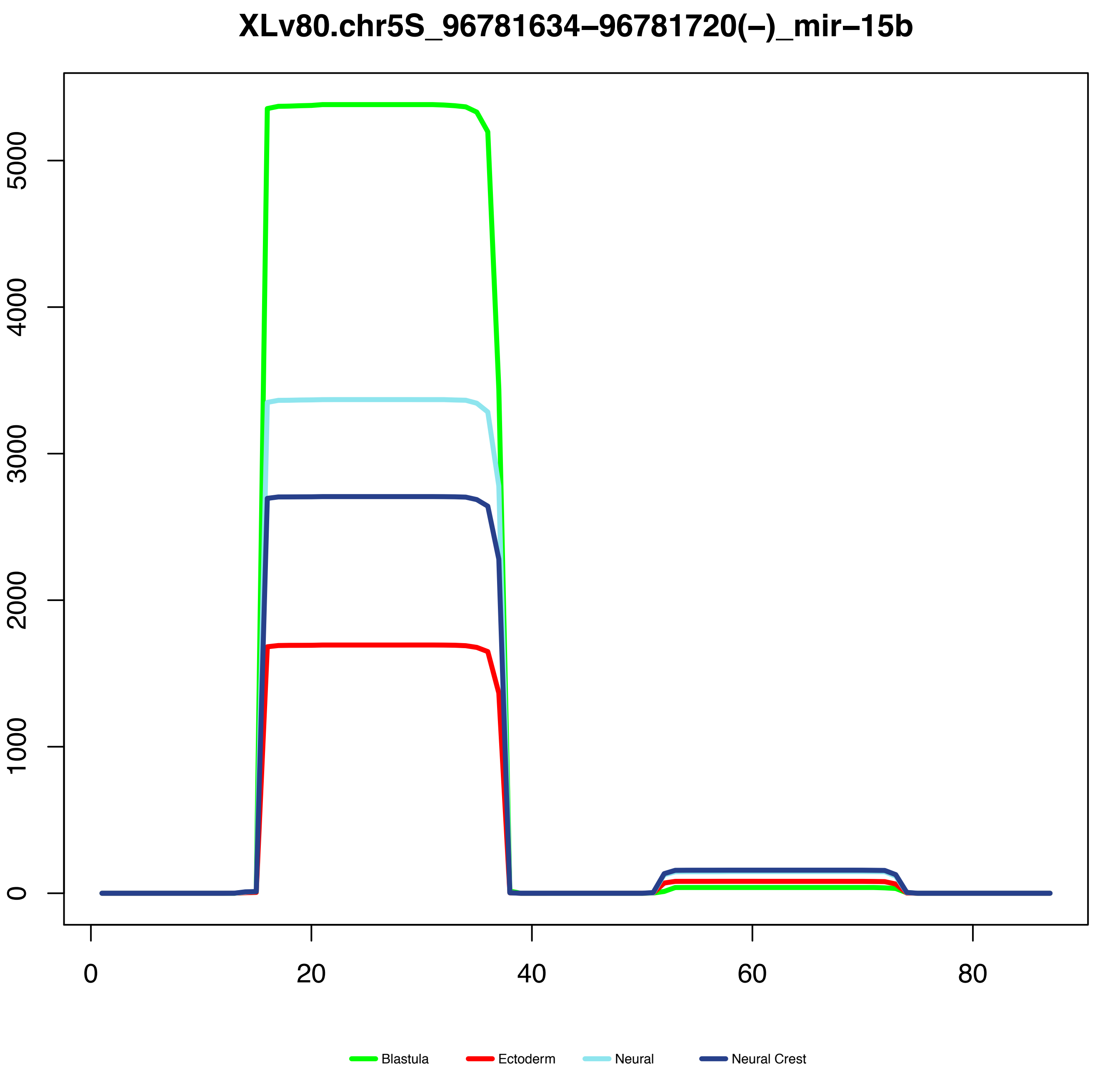
XLv80.chr5S_96781634−96781720(−)_mir−15b
0
1000
2000
3000
4000
5000
0
20
40
60
80
Blastula
Ectoderm
Neural
Neural Crest

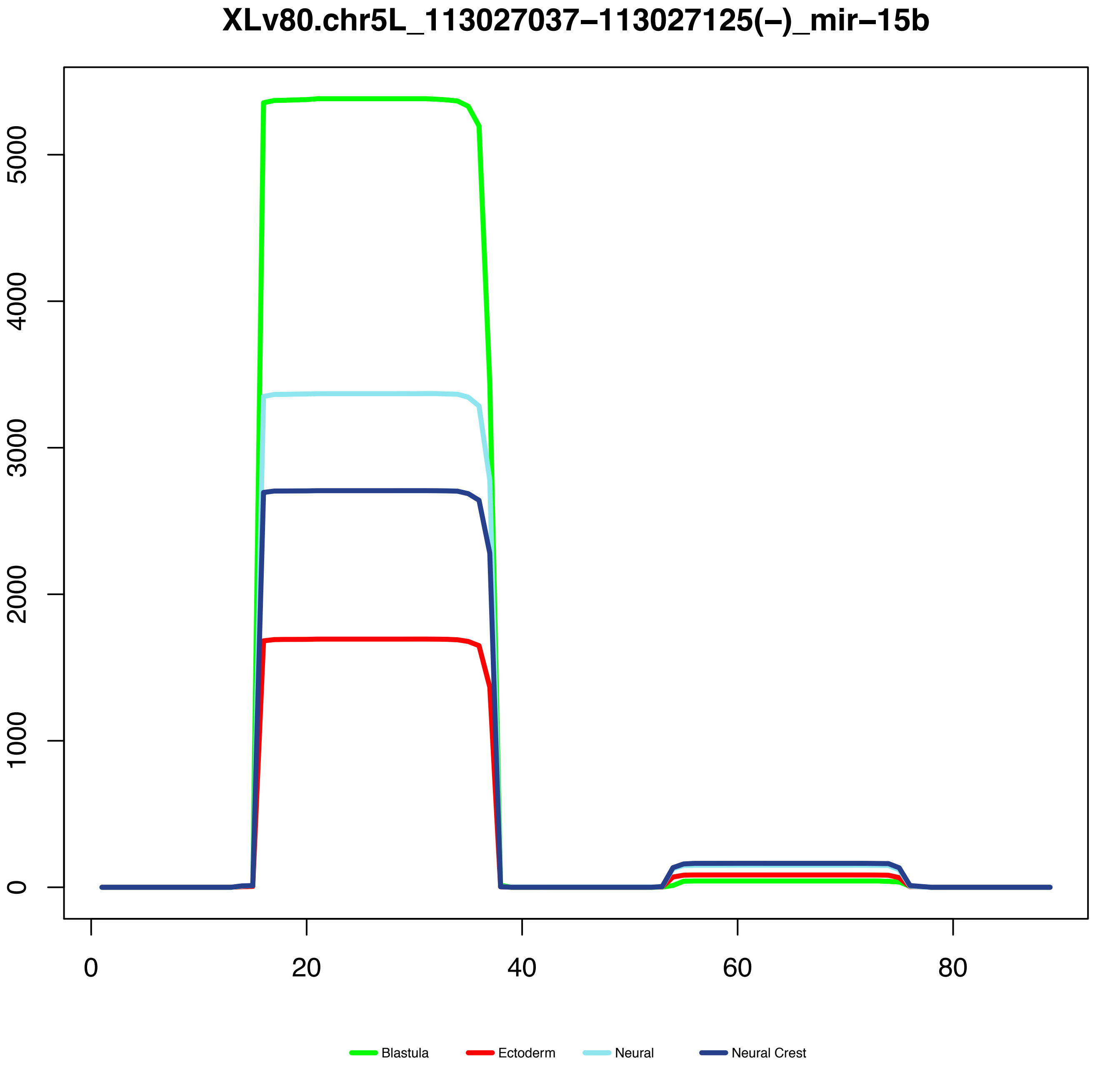
XLv80.chr5L_113027037−113027125(−)_mir−15b
0
1000
2000
3000
4000
5000
0
20
40
60
80
Blastula
Ectoderm
Neural
Neural Crest

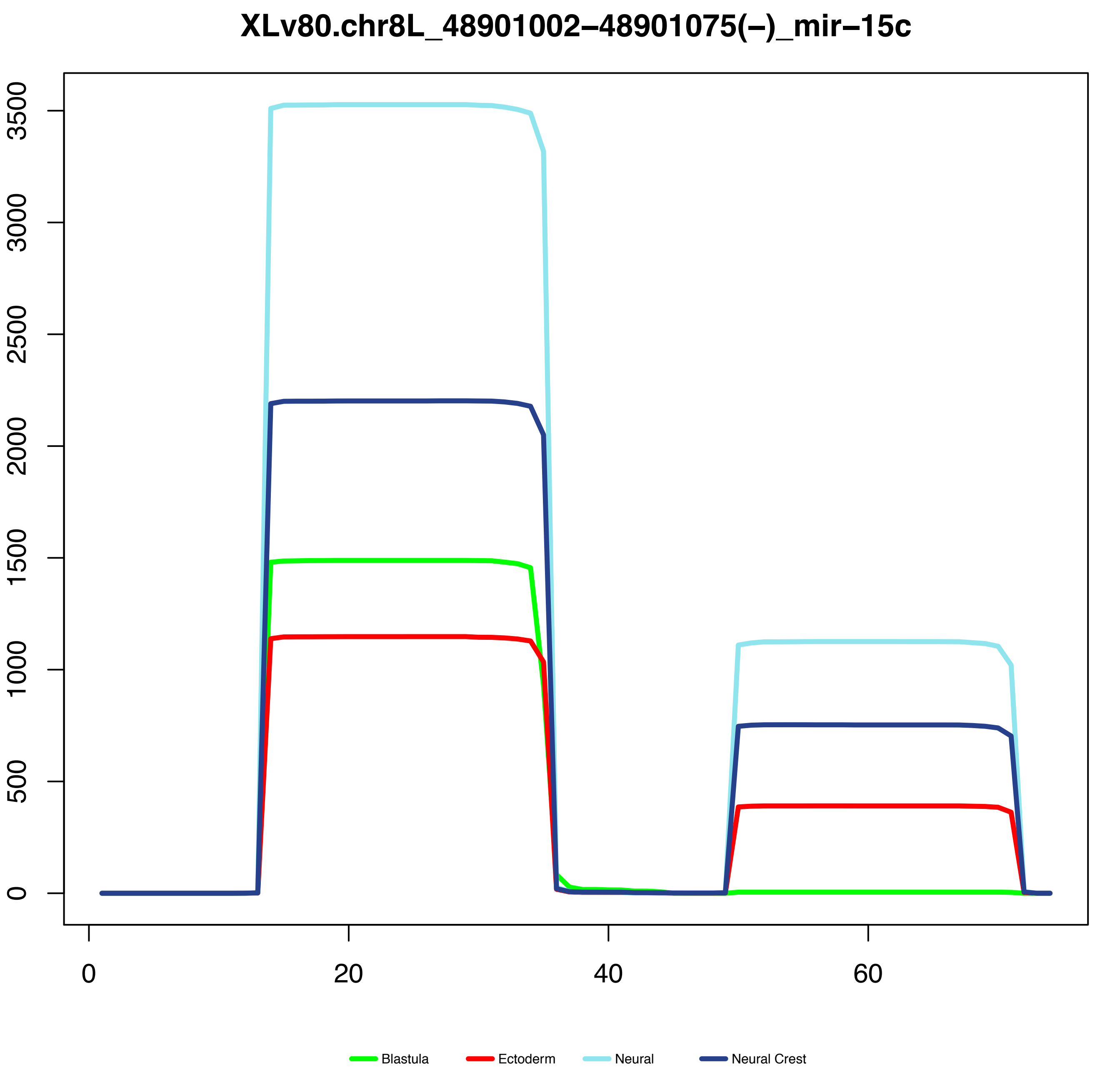
XLv80.chr8L_48901002−48901075(−)_mir−15c
0
500
1000
1500
2000
2500
3000
3500
0
20
40
60
Blastula
Ectoderm
Neural
Neural Crest

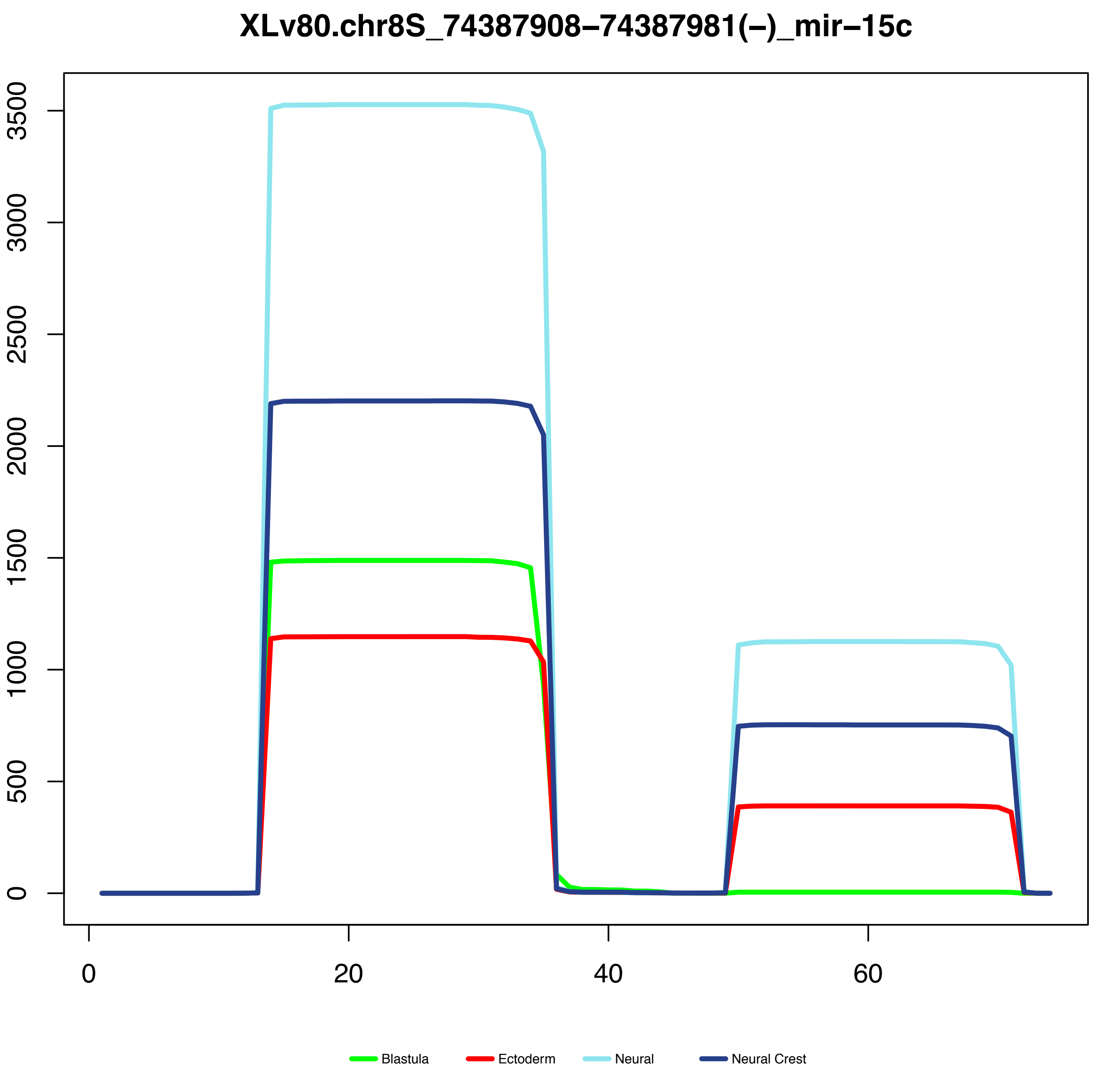
XLv80.chr8S_74387908−74387981(−)_mir−15c
0
500
1000
1500
2000
2500
3000
3500
0
20
40
60
Blastula
Ectoderm
Neural
Neural Crest

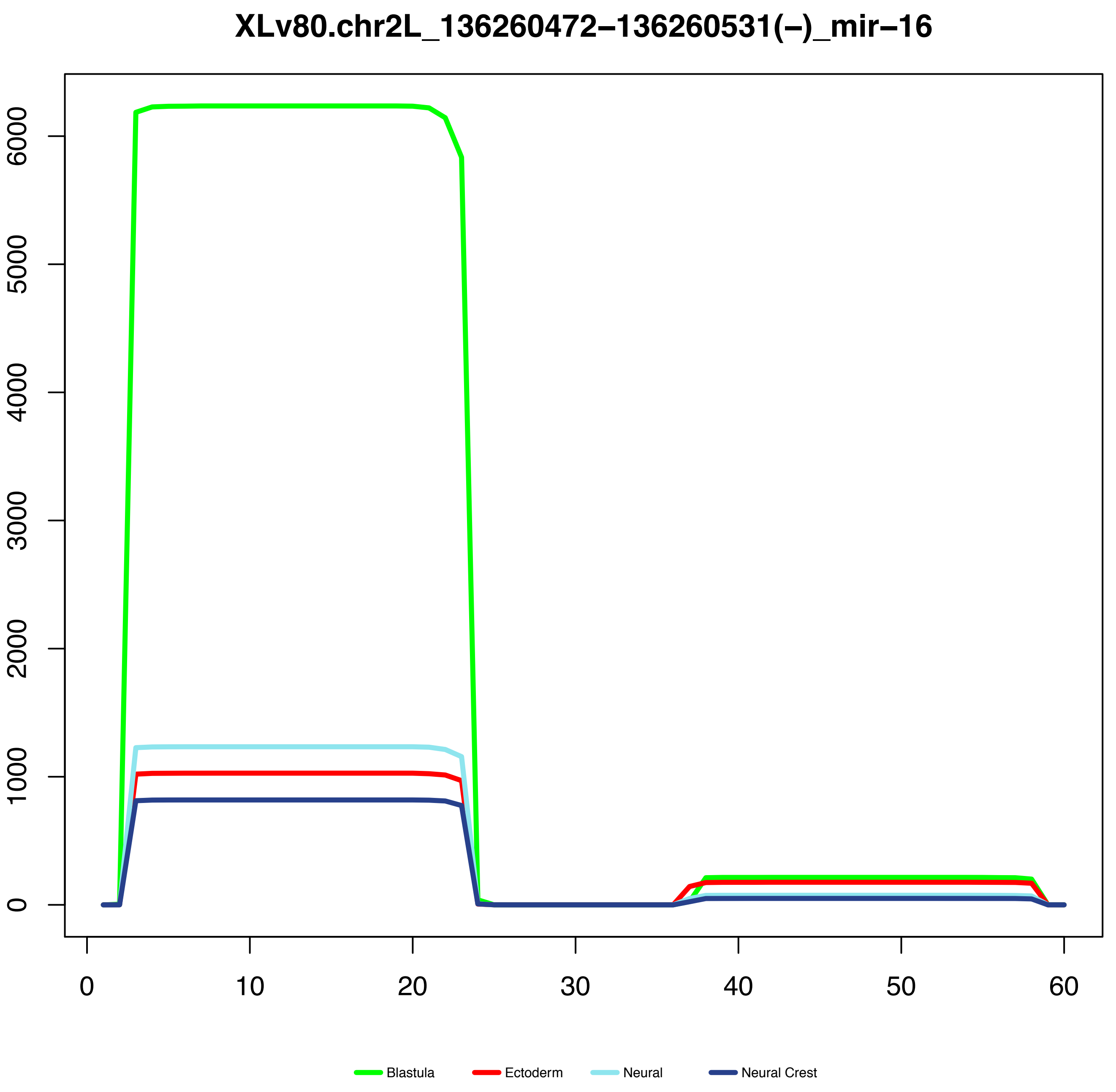
XLv80.chr2L_136260472−136260531(−)_mir−16
0
1000
2000
3000
4000
5000
6000
0
10
20
30
40
50
60
Blastula
Ectoderm
Neural
Neural Crest

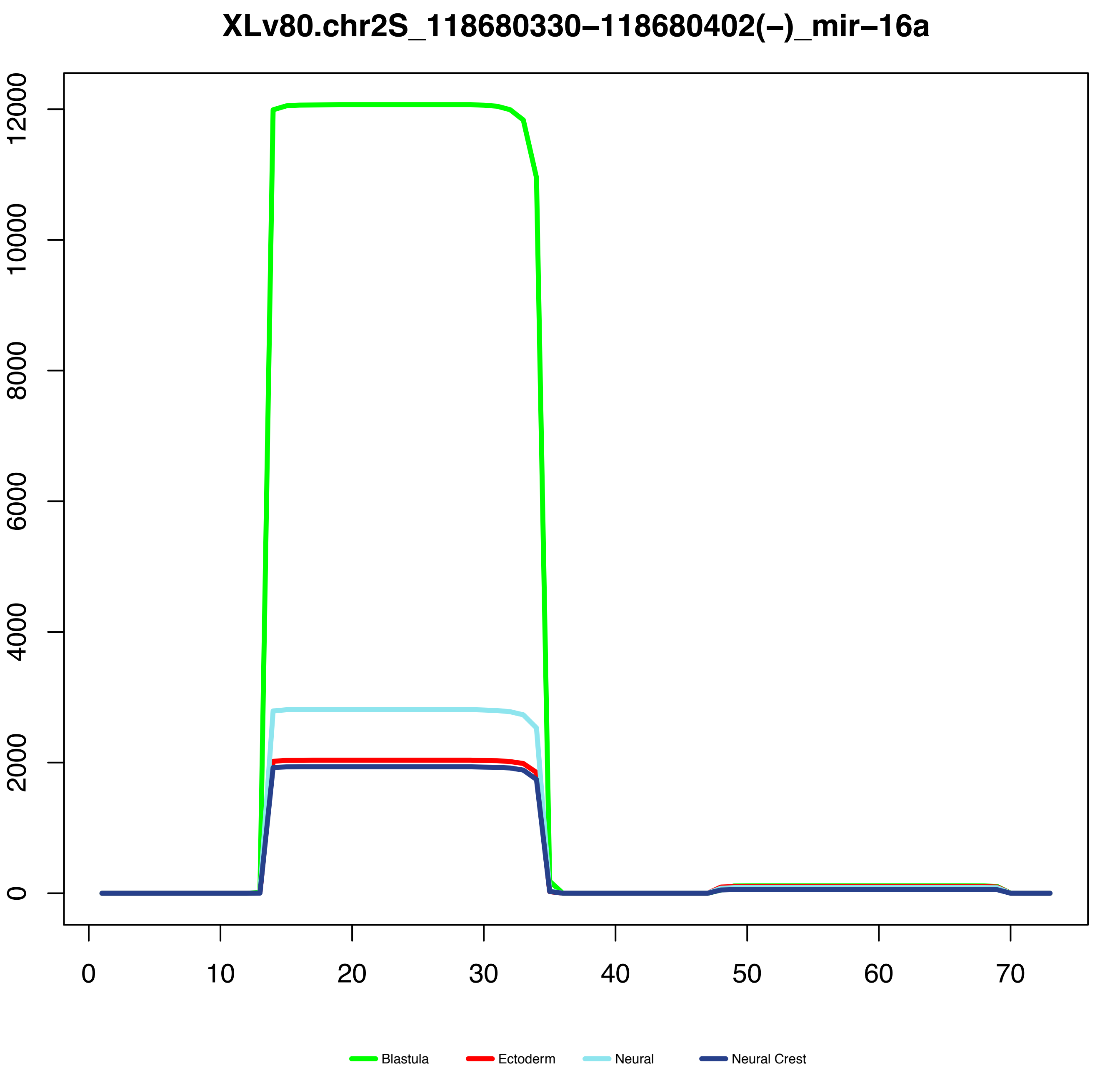
XLv80.chr2S_118680330−118680402(−)_mir−16a
0
2000
4000
6000
8000
10000
12000
0
10
20
30
40
50
60
70
Blastula
Ectoderm
Neural
Neural Crest

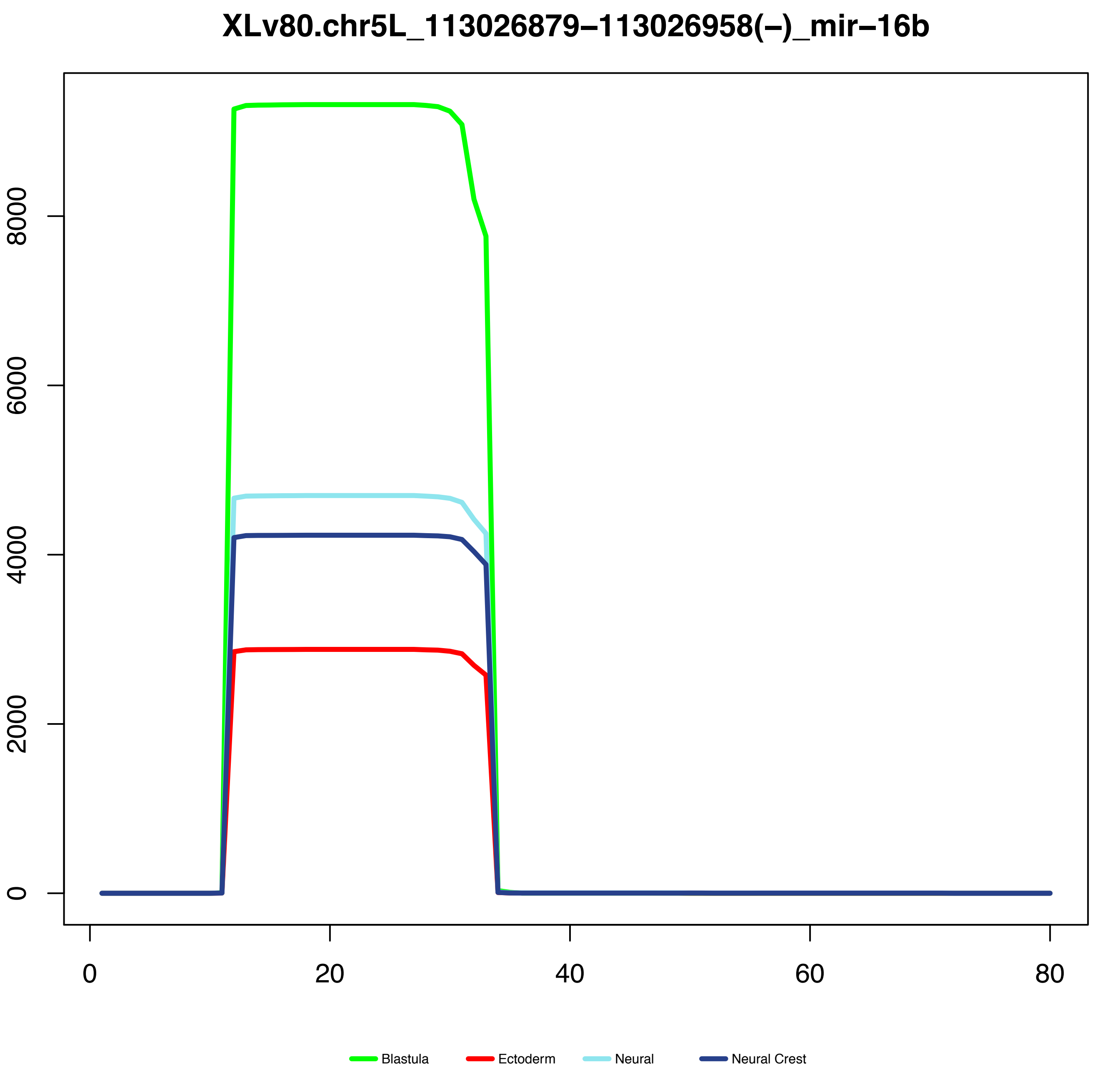

XLv80.chr5L_113026879−113026958(−)_mir−16b
0
2000
4000
6000
8000
0
20
40
60
80
Blastula
Ectoderm
Neural
Neural Crest

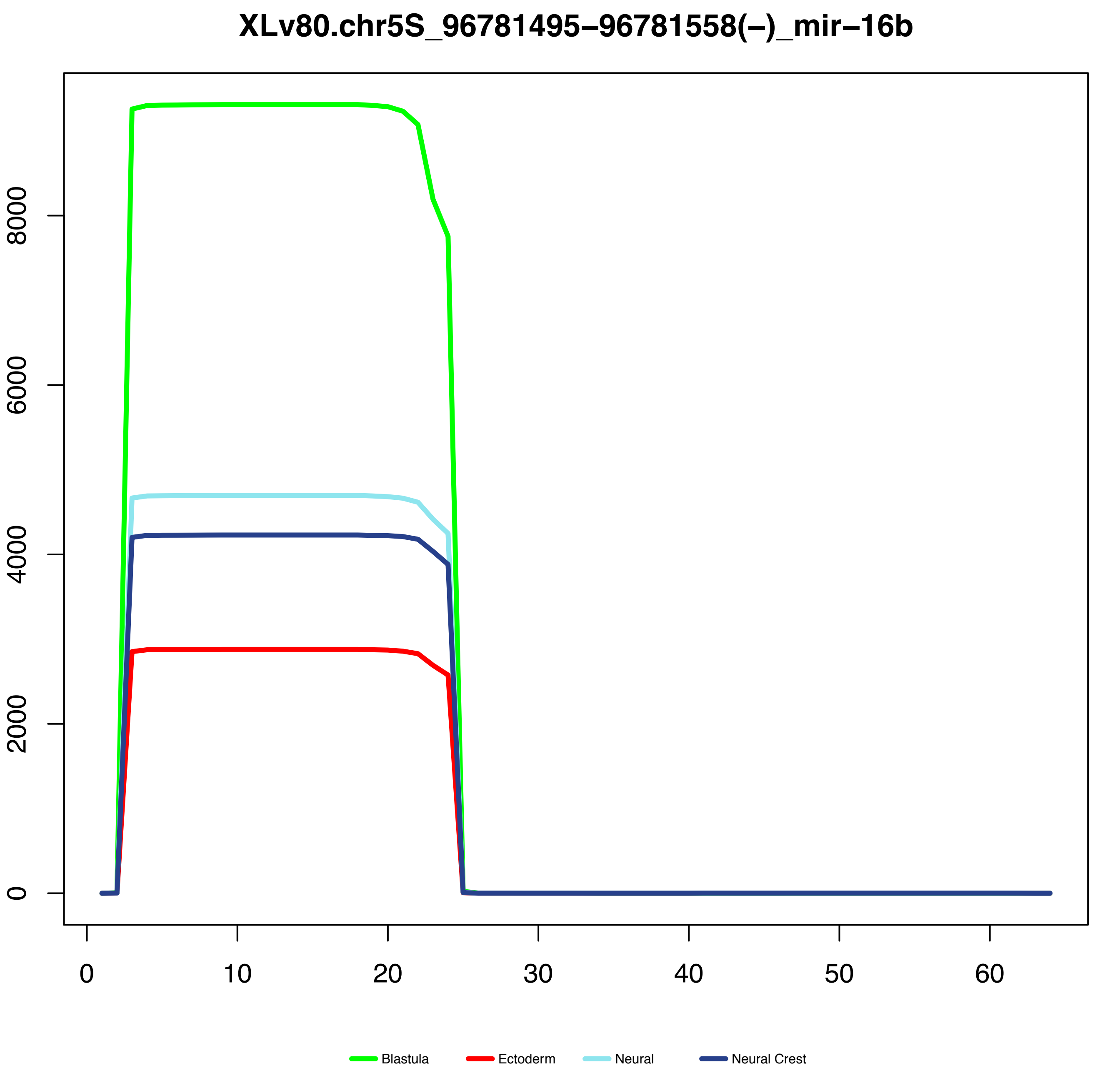

XLv80.chr5S_96781495−96781558(−)_mir−16b
0
2000
4000
6000
8000
0
10
20
30
40
50
60
Blastula
Ectoderm
Neural
Neural Crest

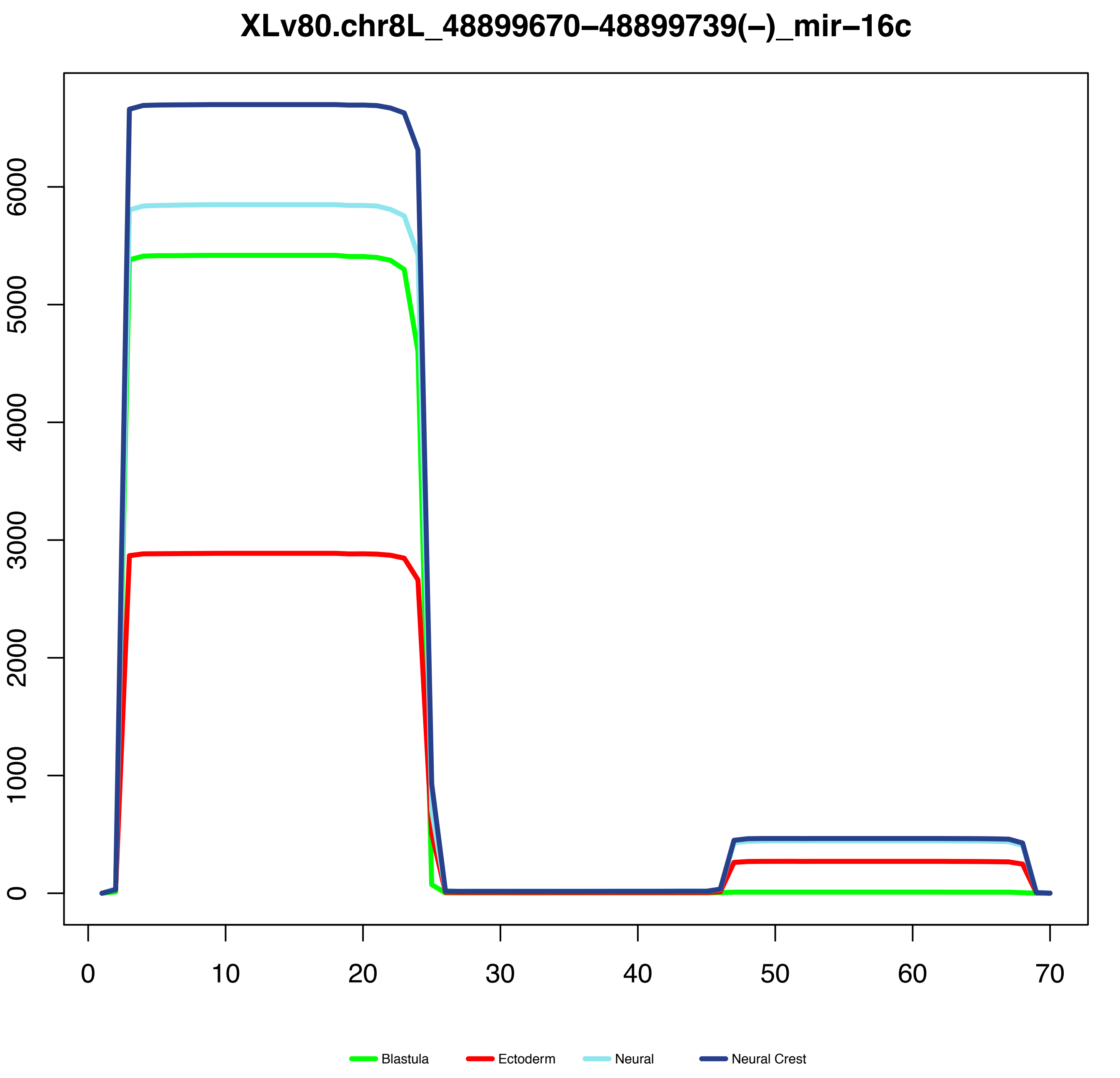
XLv80.chr8L_48899670−48899739(−)_mir−16c
0
1000
2000
3000
4000
5000
6000
0
10
20
30
40
50
60
70
Blastula
Ectoderm
Neural
Neural Crest

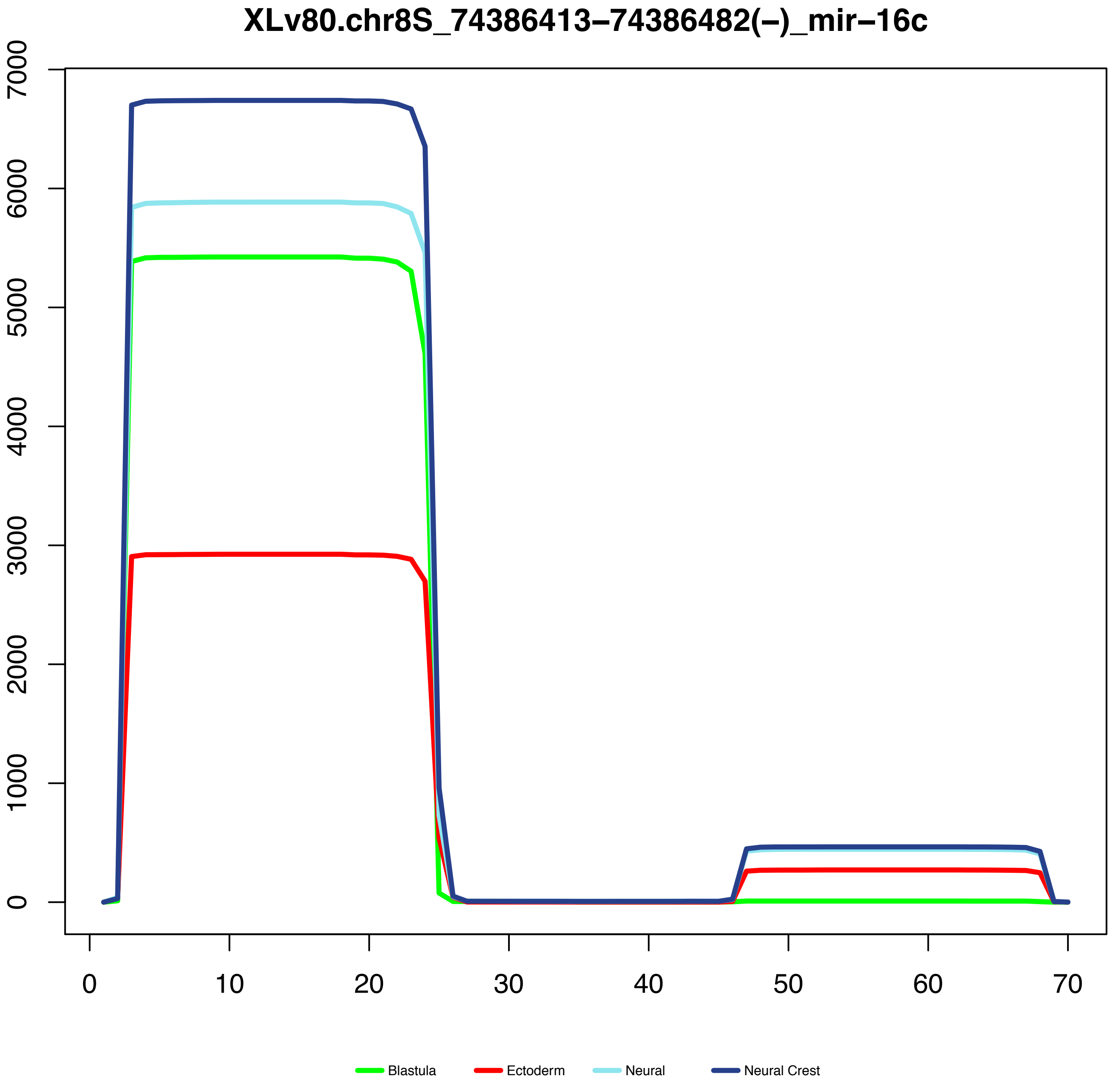

XLv80.chr8S_74386413−74386482(−)_mir−16c
0
1000
2000
3000
4000
5000
6000
7000
0
10
20
30
40
50
60
70
Blastula
Ectoderm
Neural
Neural Crest

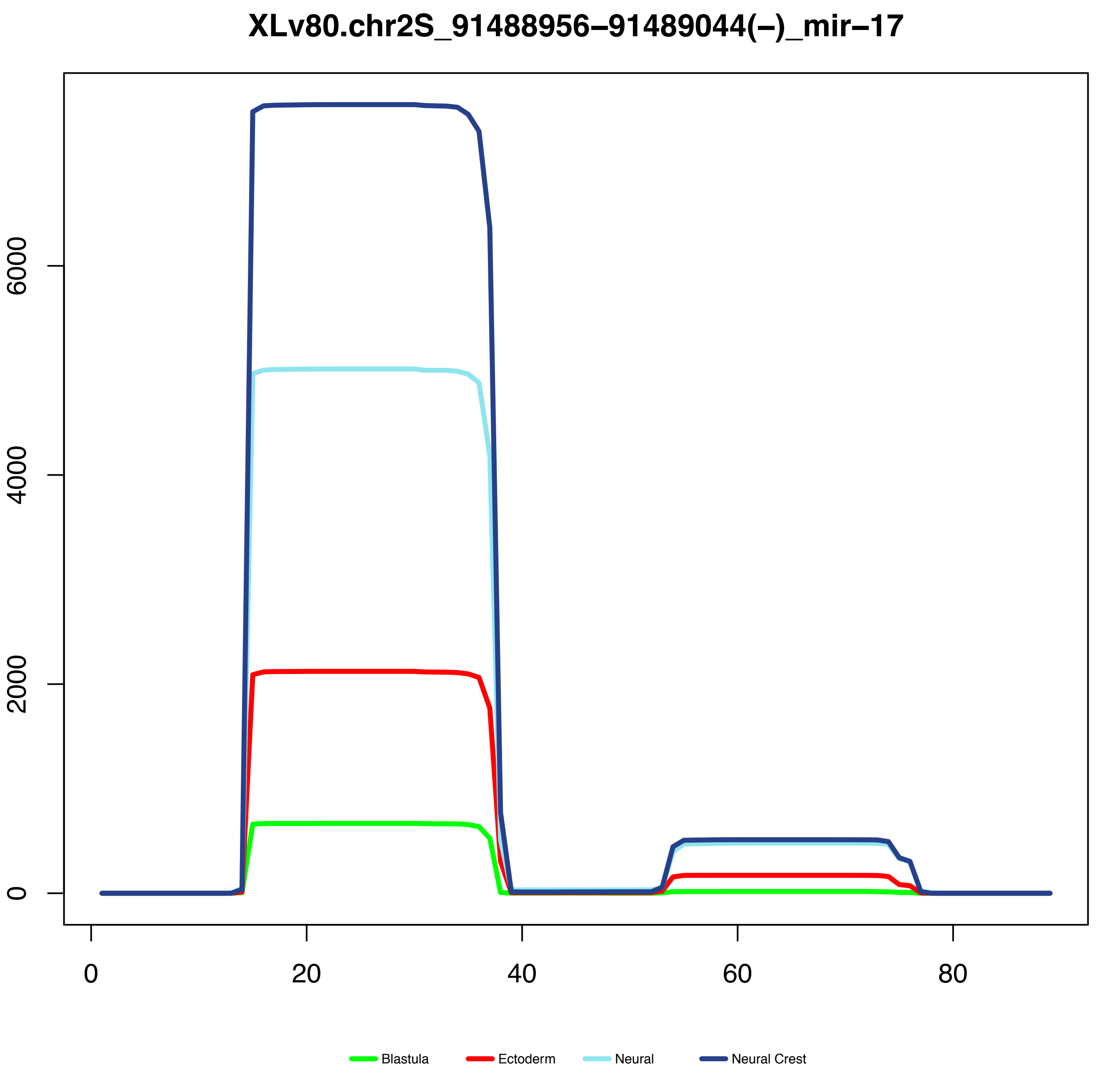
XLv80.chr2S_91488956−91489044(−)_mir−17
0
2000
4000
6000
0
20
40
60
80
Blastula
Ectoderm
Neural
Neural Crest

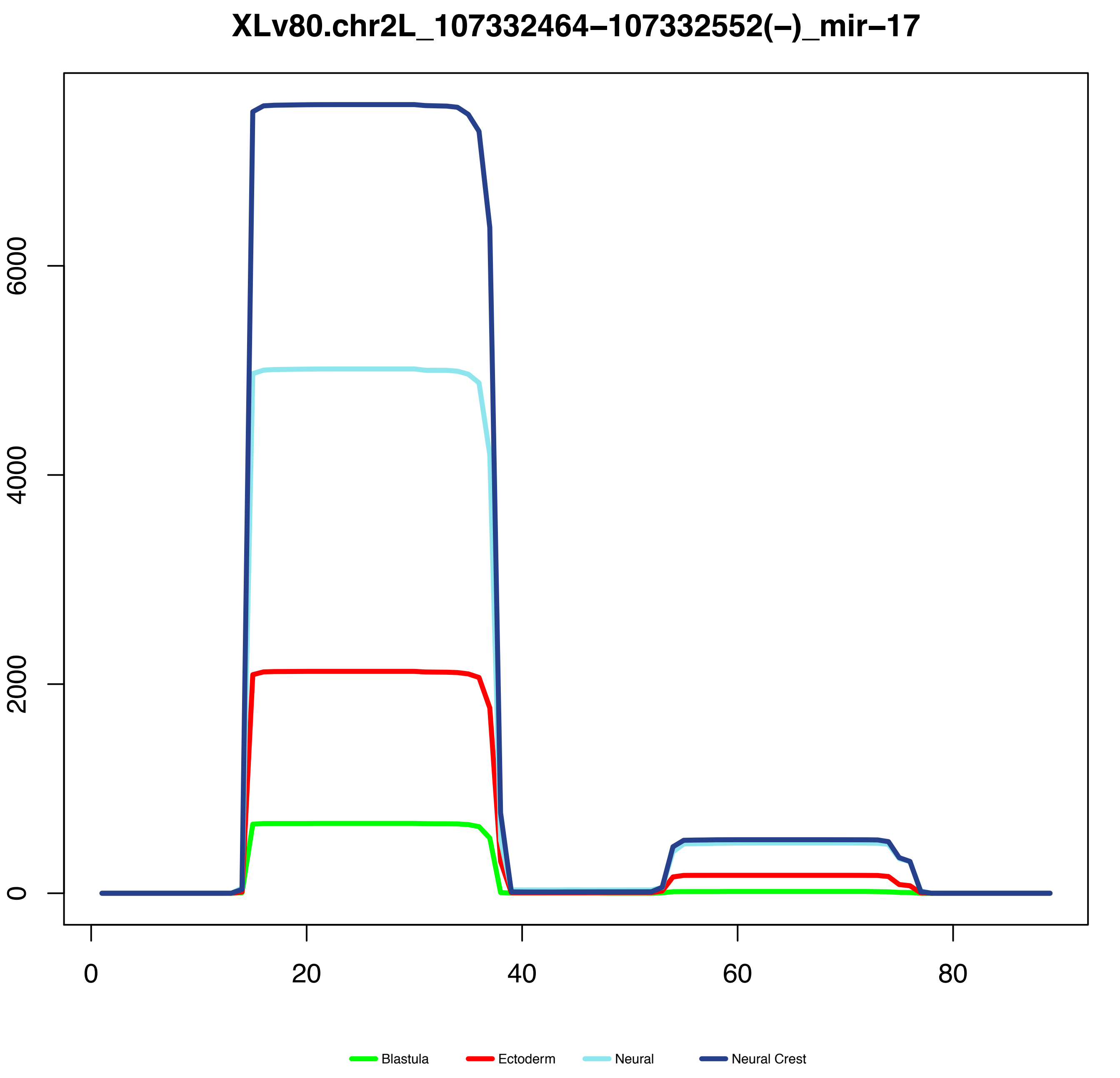
XLv80.chr2L_107332464−107332552(−)_mir−17
0
2000
4000
6000
0
20
40
60
80
Blastula
Ectoderm
Neural
Neural Crest

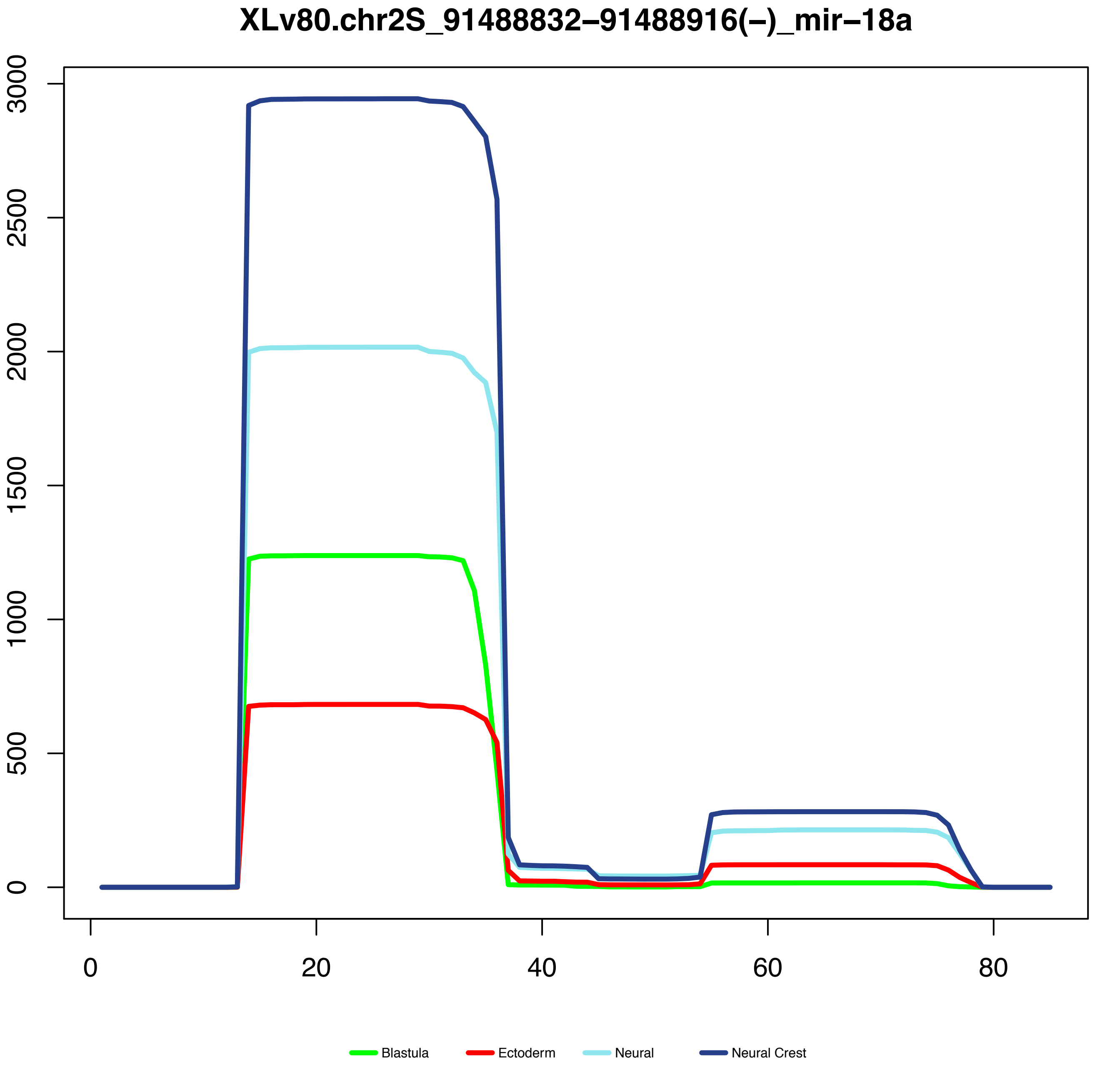

XLv80.chr2S_91488832−91488916(−)_mir−18a
0
500
1000
1500
2000
2500
3000
0
20
40
60
80
Blastula
Ectoderm
Neural
Neural Crest

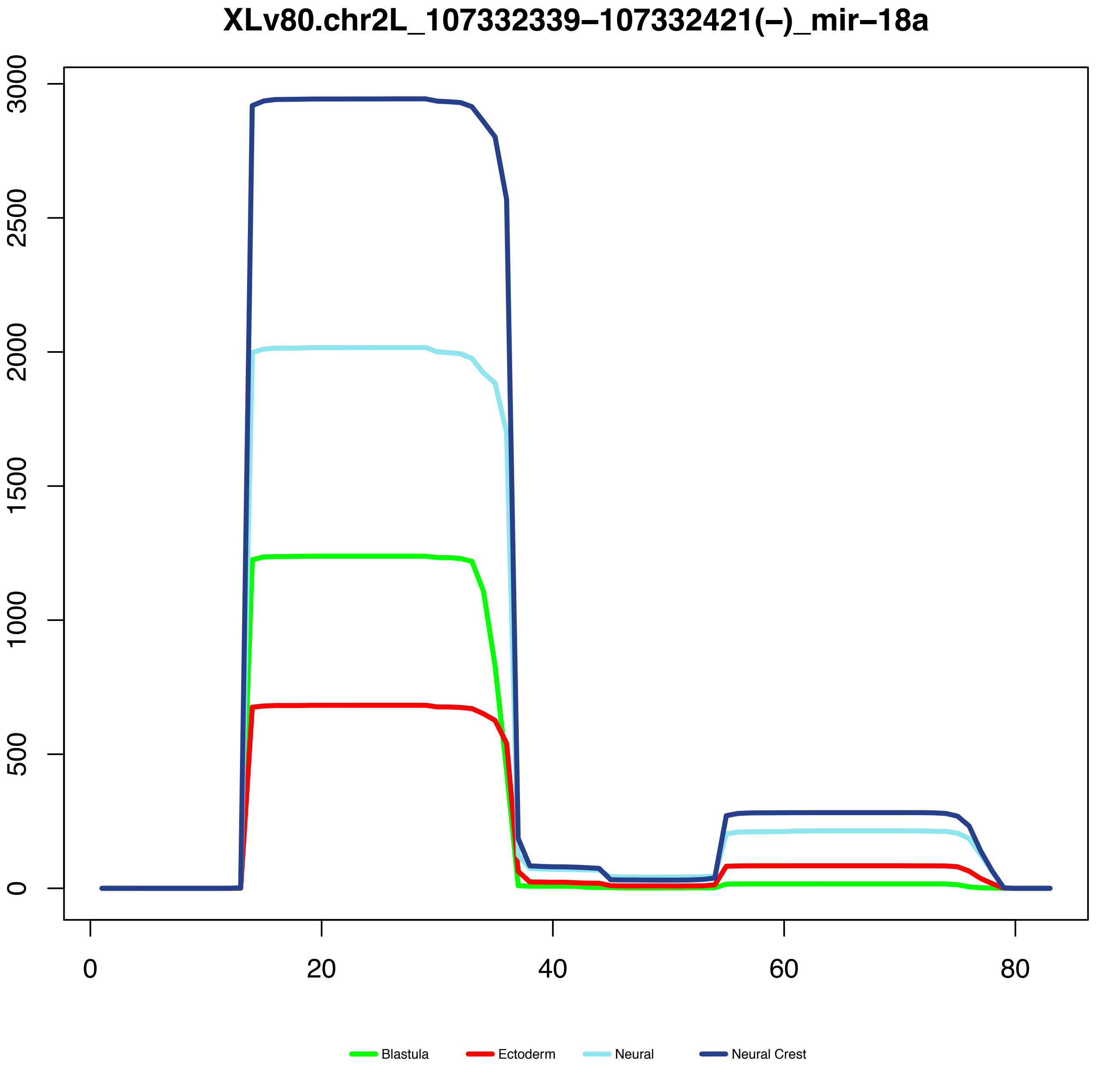

XLv80.chr2L_107332339−107332421(−)_mir−18a
3000
2500
2000
1500
1000
500
0
0
20
40
60
80
Blastula
Ectoderm
Neural
Neural Crest

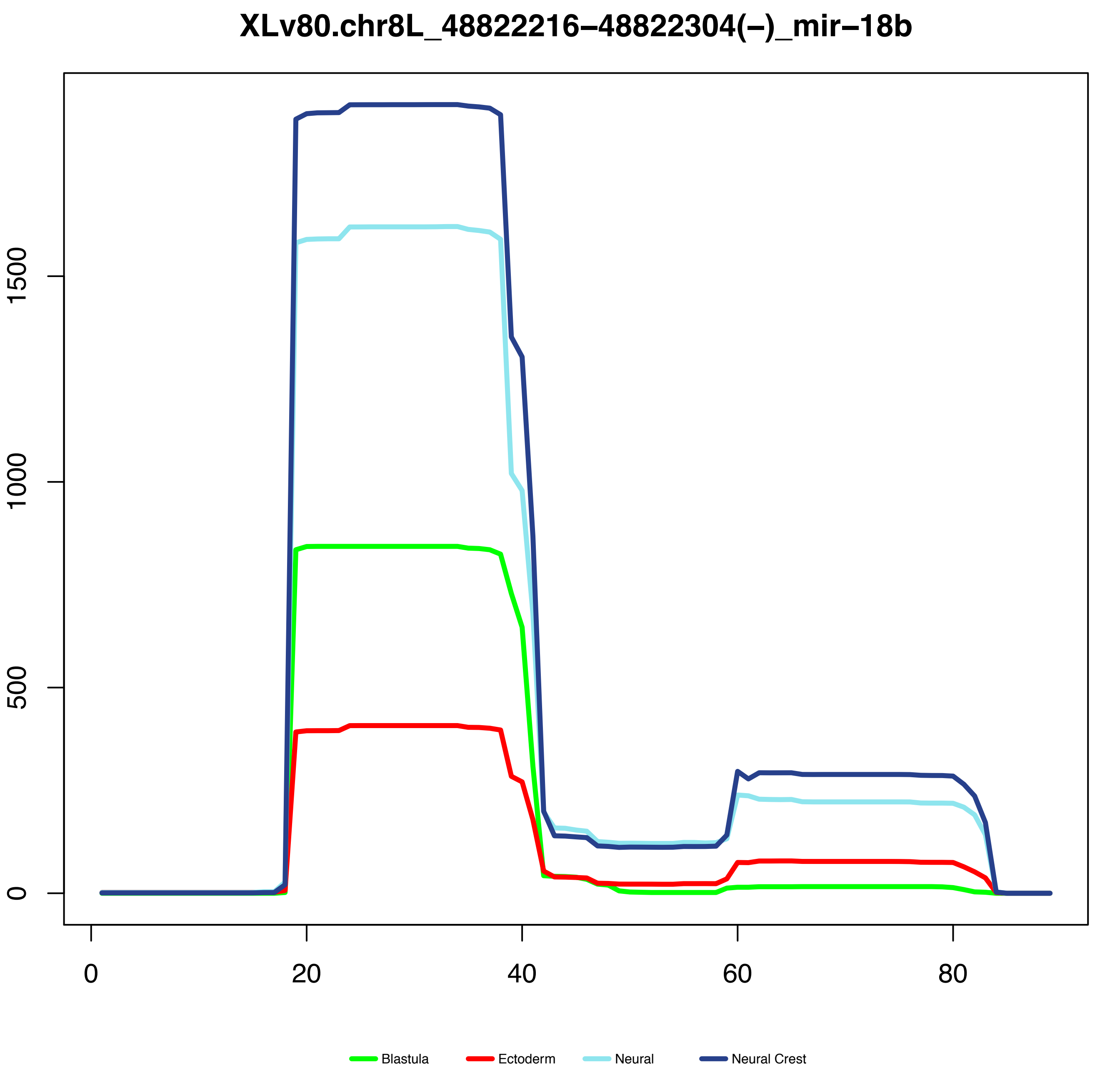
XLv80.chr8L_48822216–48822304(–)_mir–18b
0
500
1000
1500
0
20
40
60
80
Blastula
Ectoderm
Neural
Neural Crest

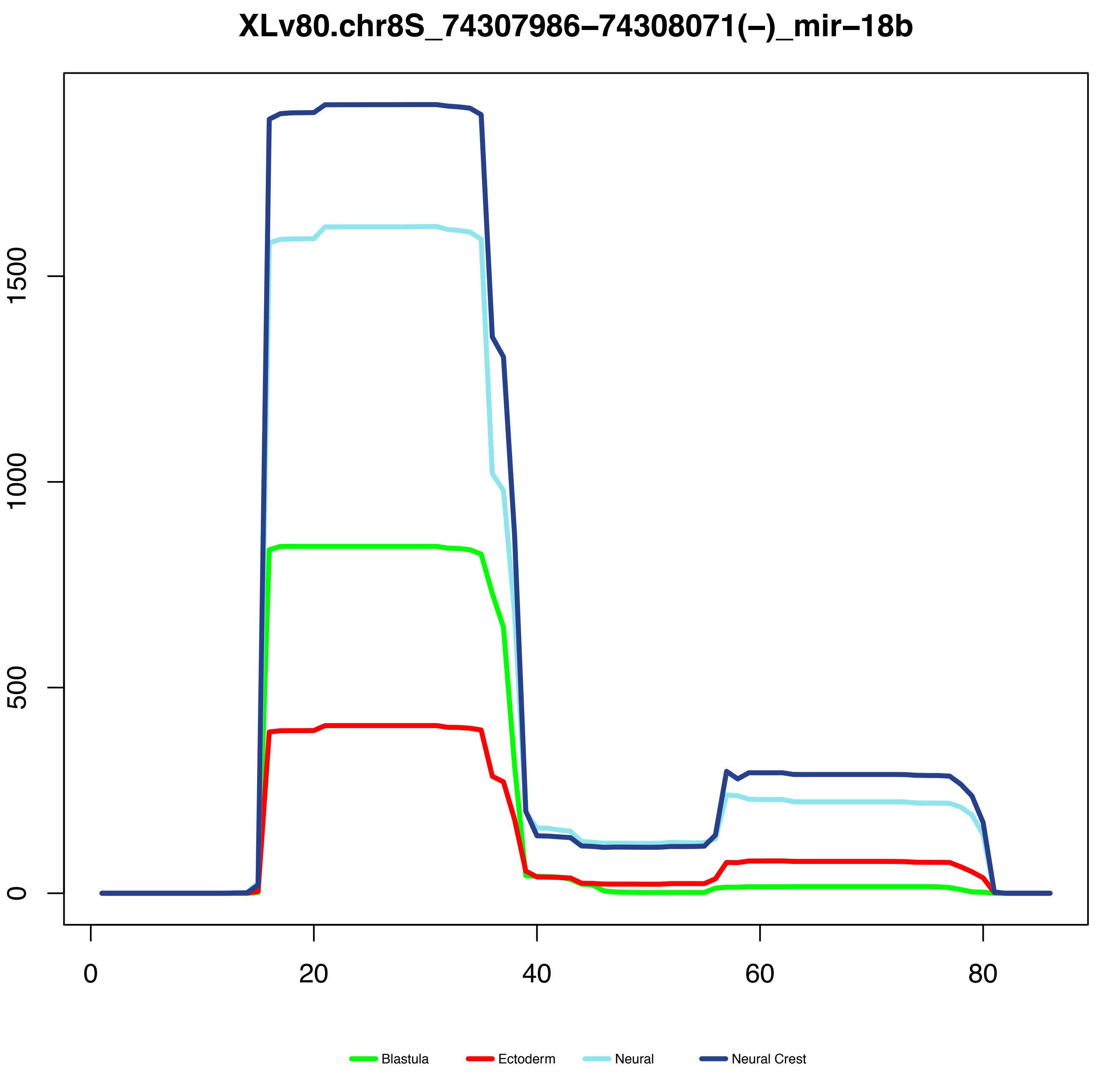
XLv80.chr8S_74307986−74308071(−)_mir−18b
0
500
1000
1500
0
20
40
60
80
Blastula
Ectoderm
Neural
Neural Crest

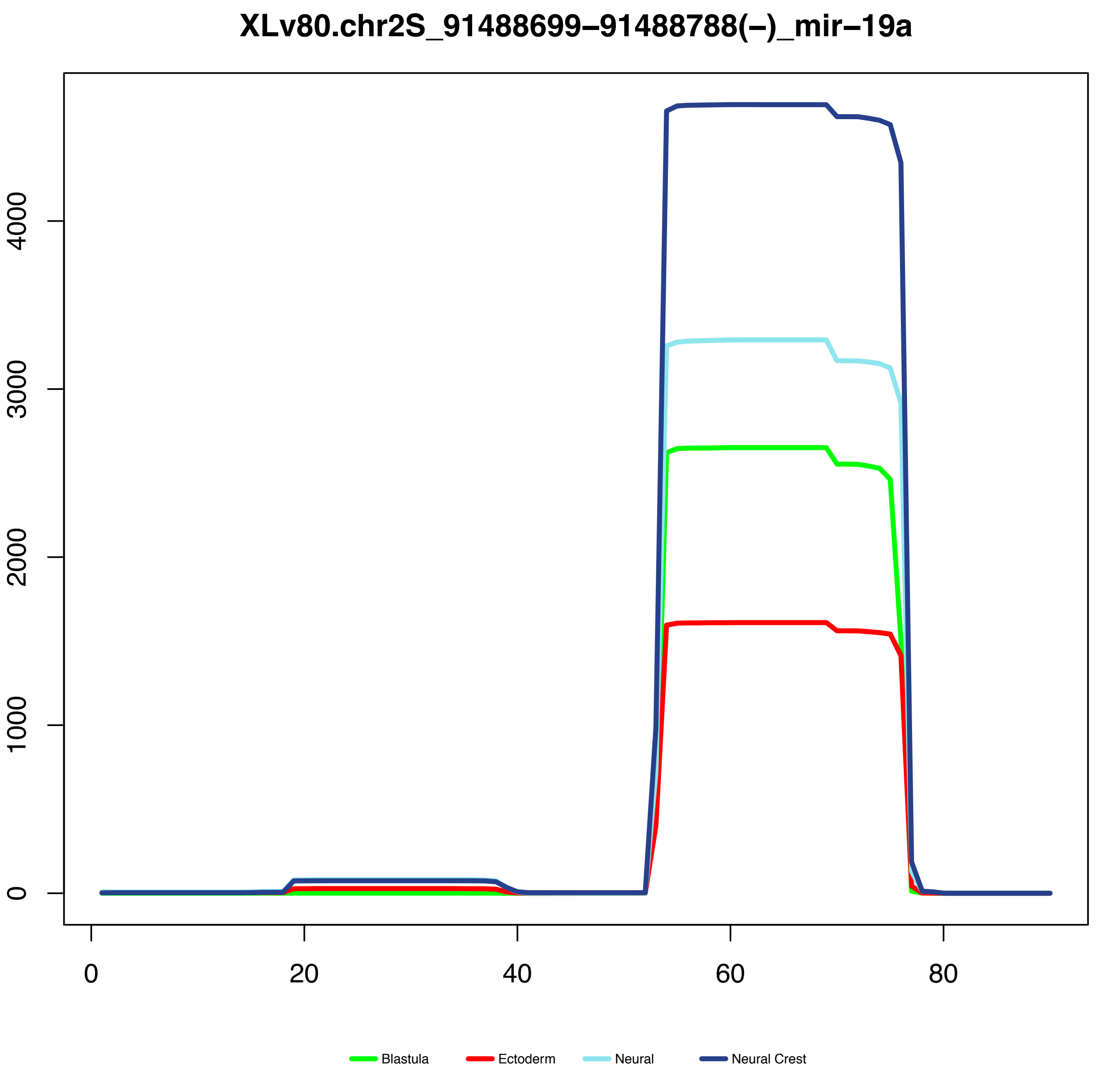

XLv80.chr2S_91488699−91488788(−)_mir−19a
0
1000
2000
3000
4000
0
20
40
60
80
Blastula
Ectoderm
Neural
Neural Crest

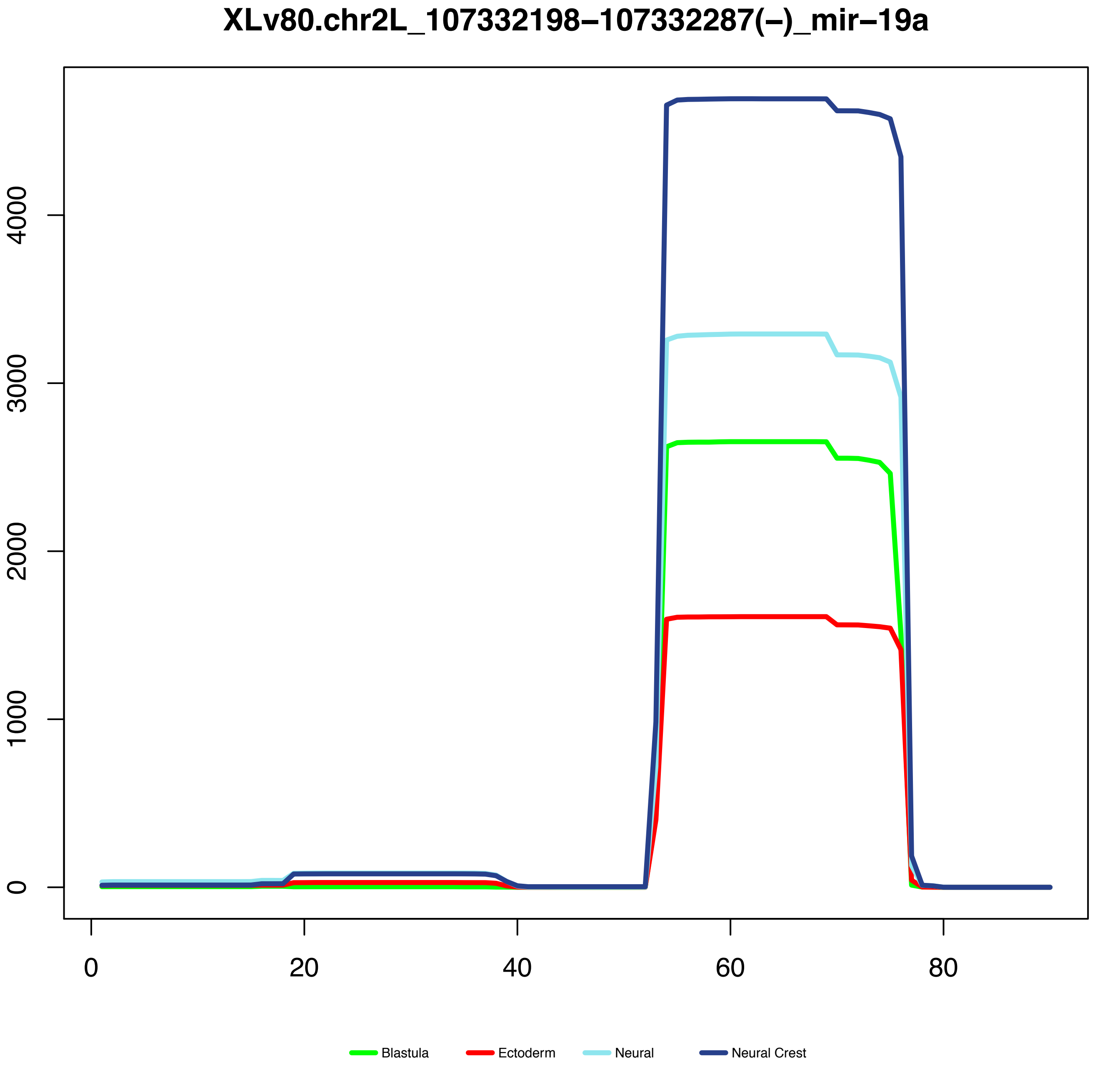

XLv80.chr2L_107332198−107332287(−)_mir−19a
0
1000
2000
3000
4000
0
20
40
60
80
Blastula
Ectoderm
Neural
Neural Crest

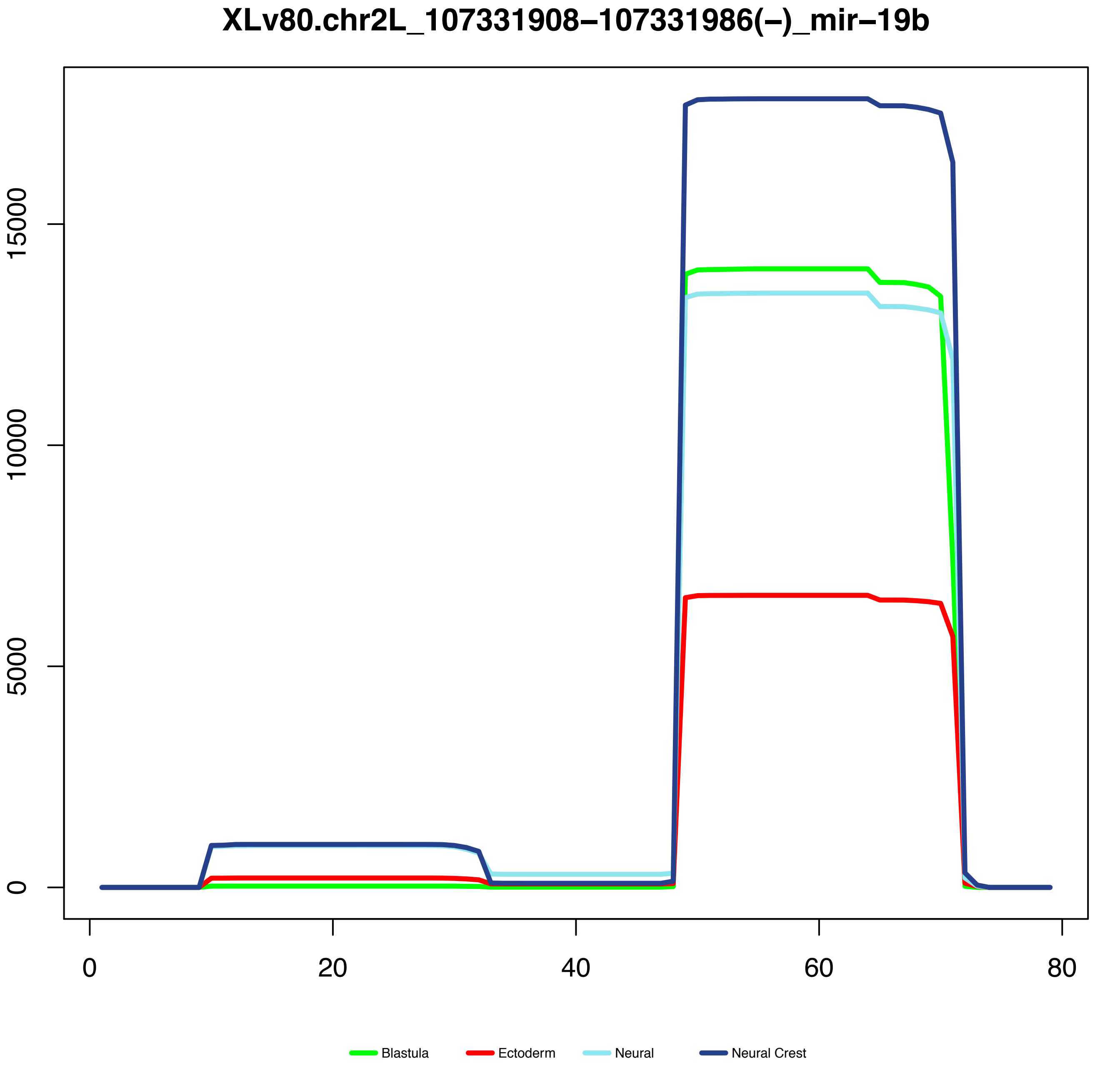
XLv80.chr2L_107331908−107331986(−)_mir−19b
0
5000
10000
15000
0
20
40
60
80
Blastula
Ectoderm
Neural
Neural Crest

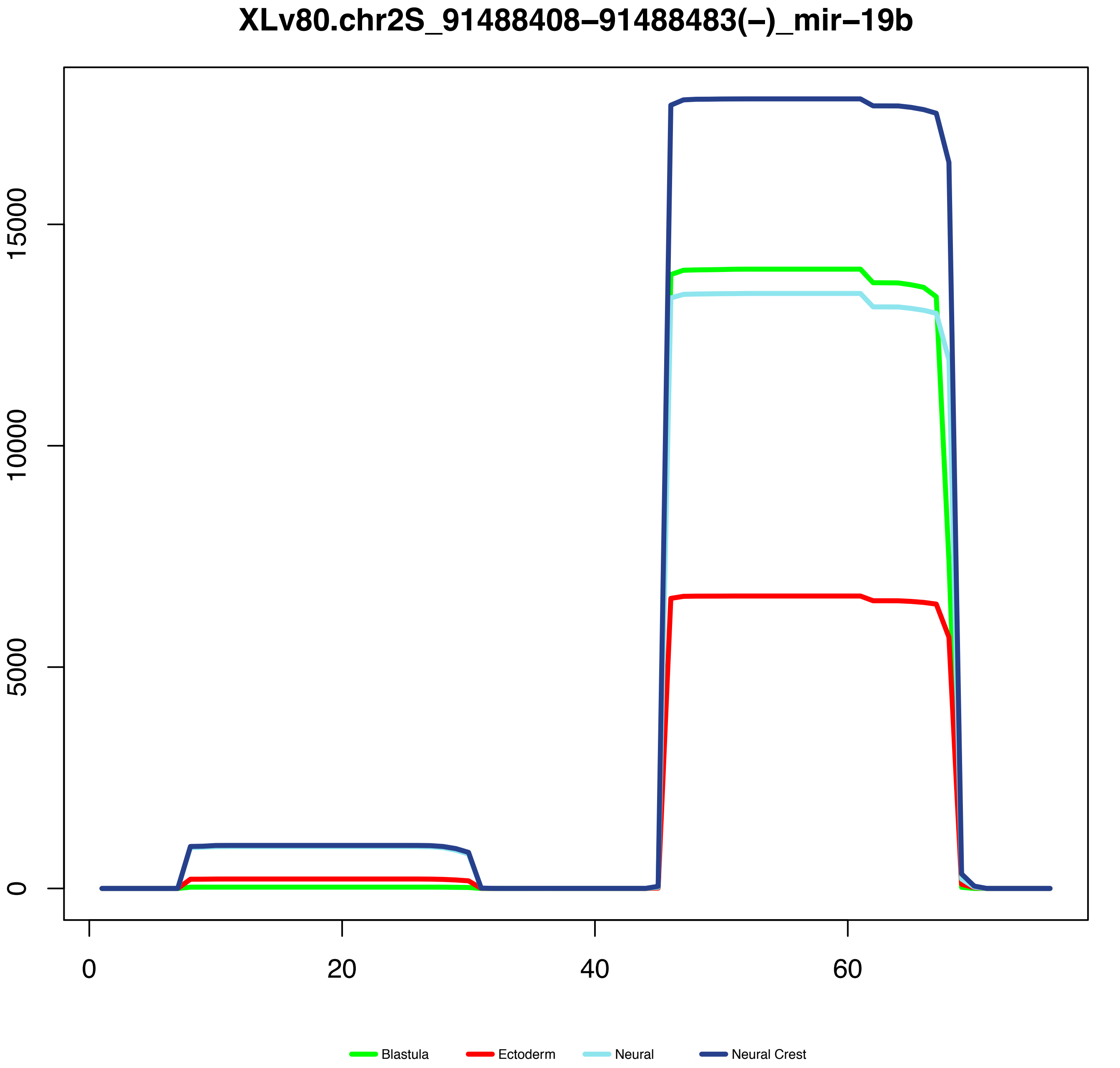
XLv80.chr2S_91488408−91488483(−)_mir−19b
0
5000
10000
15000
0
20
40
60
Blastula
Ectoderm
Neural
Neural Crest

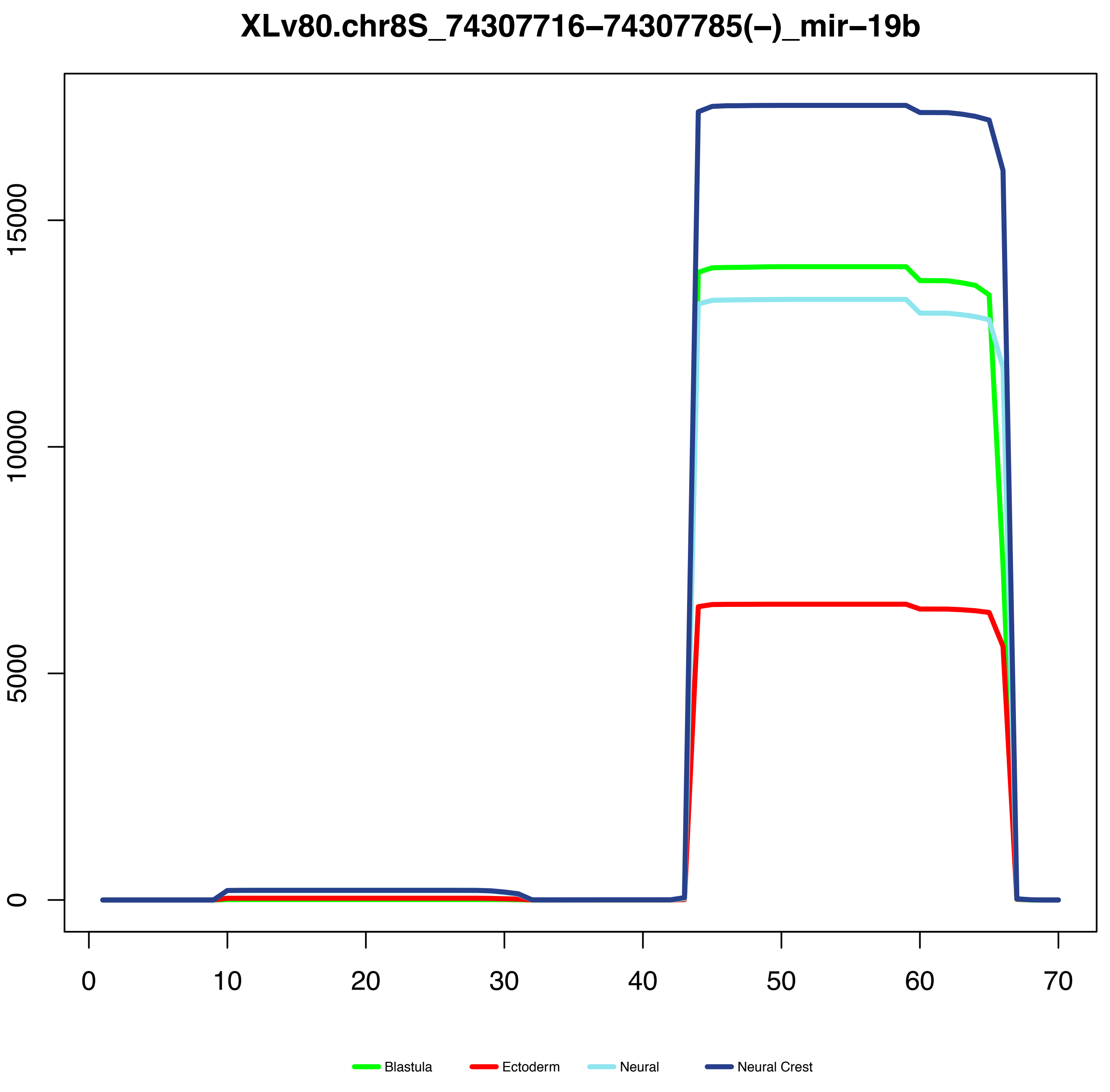
XLv80.chr8S_74307716−74307785(−)_mir−19b
0
5000
10000
15000
0
10
20
30
40
50
60
70
Blastula
Ectoderm
Neural
Neural Crest

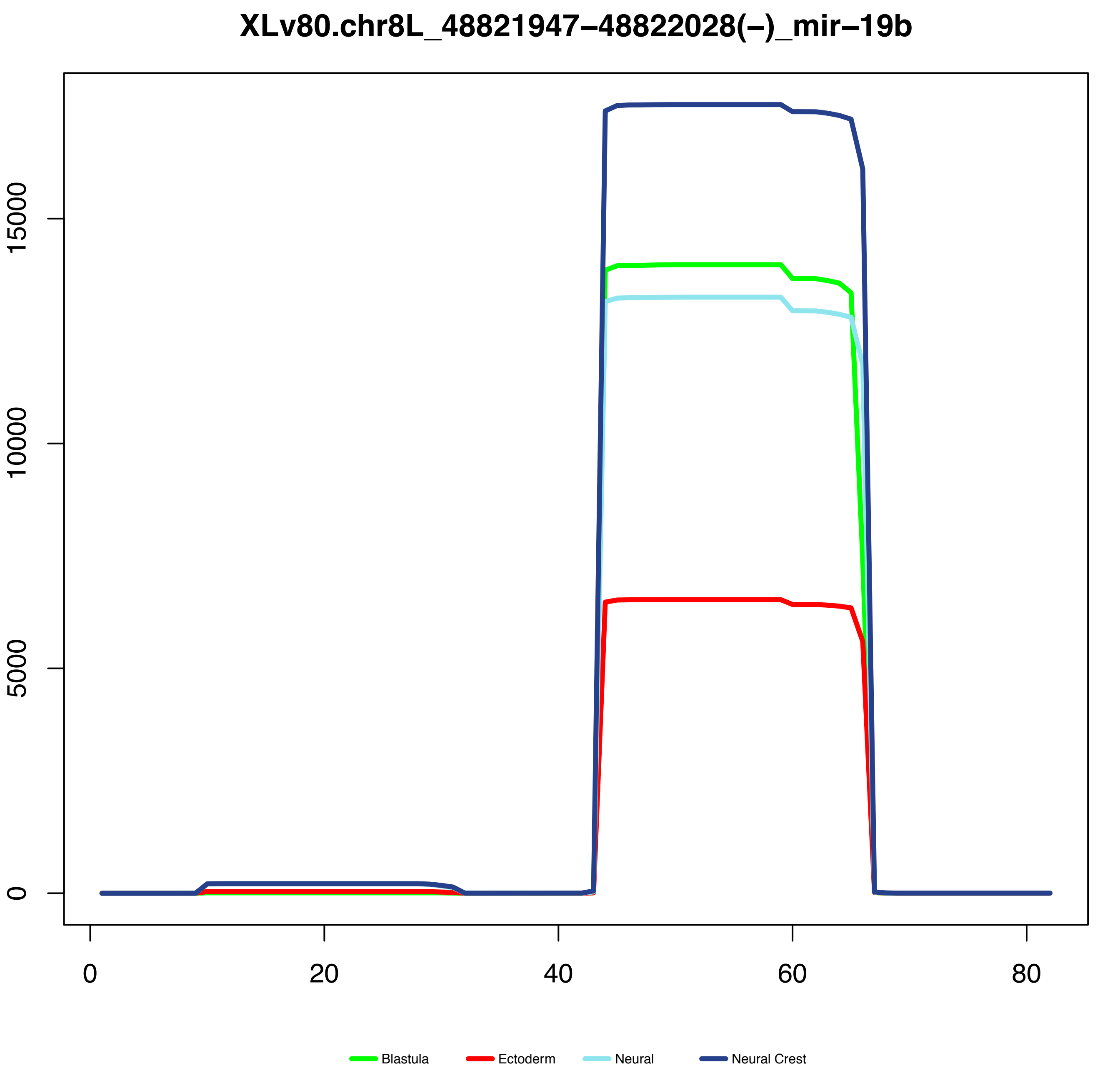
XLv80.chr8L_48821947−48822028(−)_mir−19b
0
5000
10000
15000
0
20
40
60
80
Blastula
Ectoderm
Neural
Neural Crest

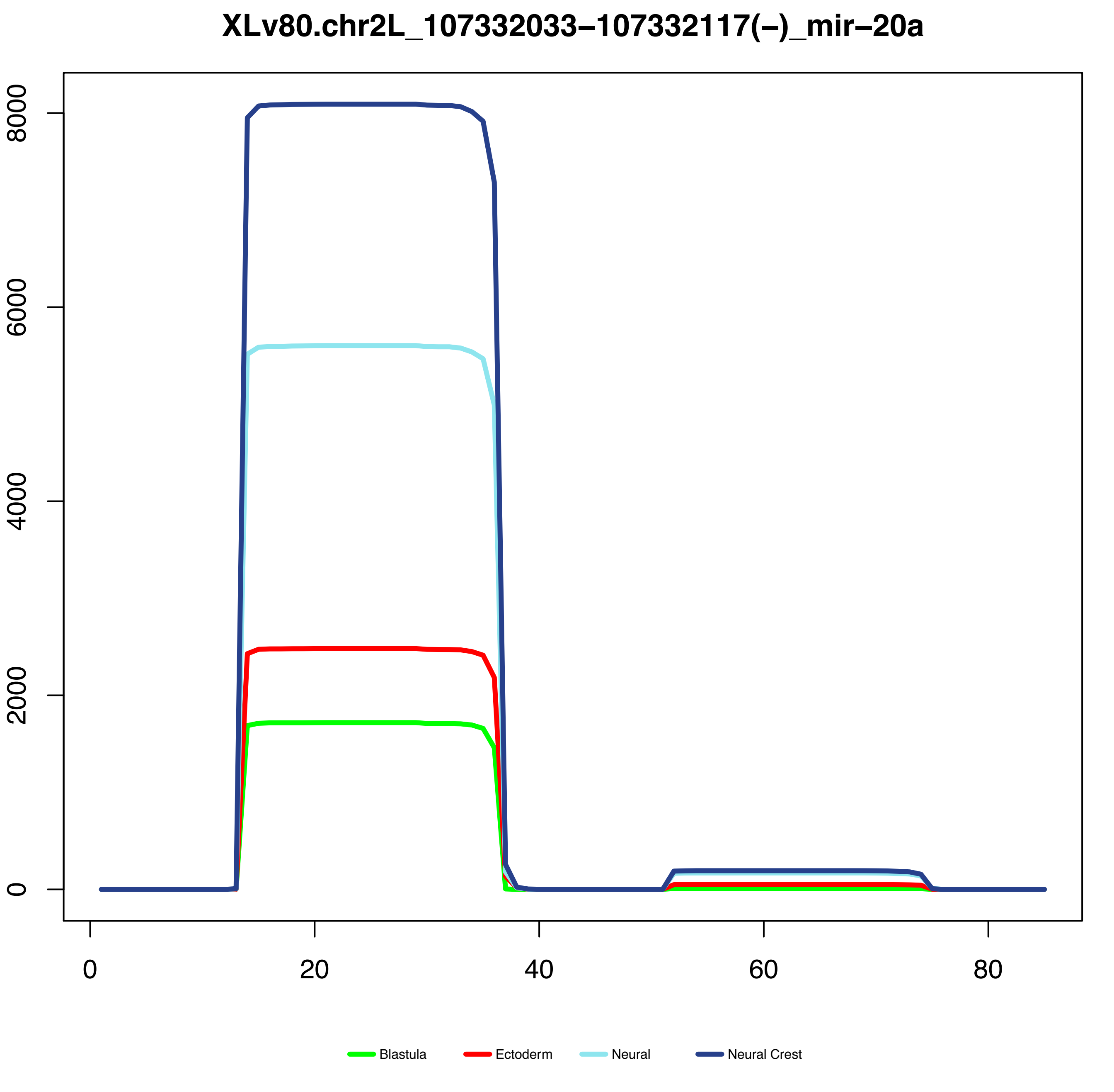

XLv80.chr2L_107332033−107332117(−)_mir−20a
0
2000
4000
6000
8000
0
20
40
60
80
Blastula
Ectoderm
Neural
Neural Crest

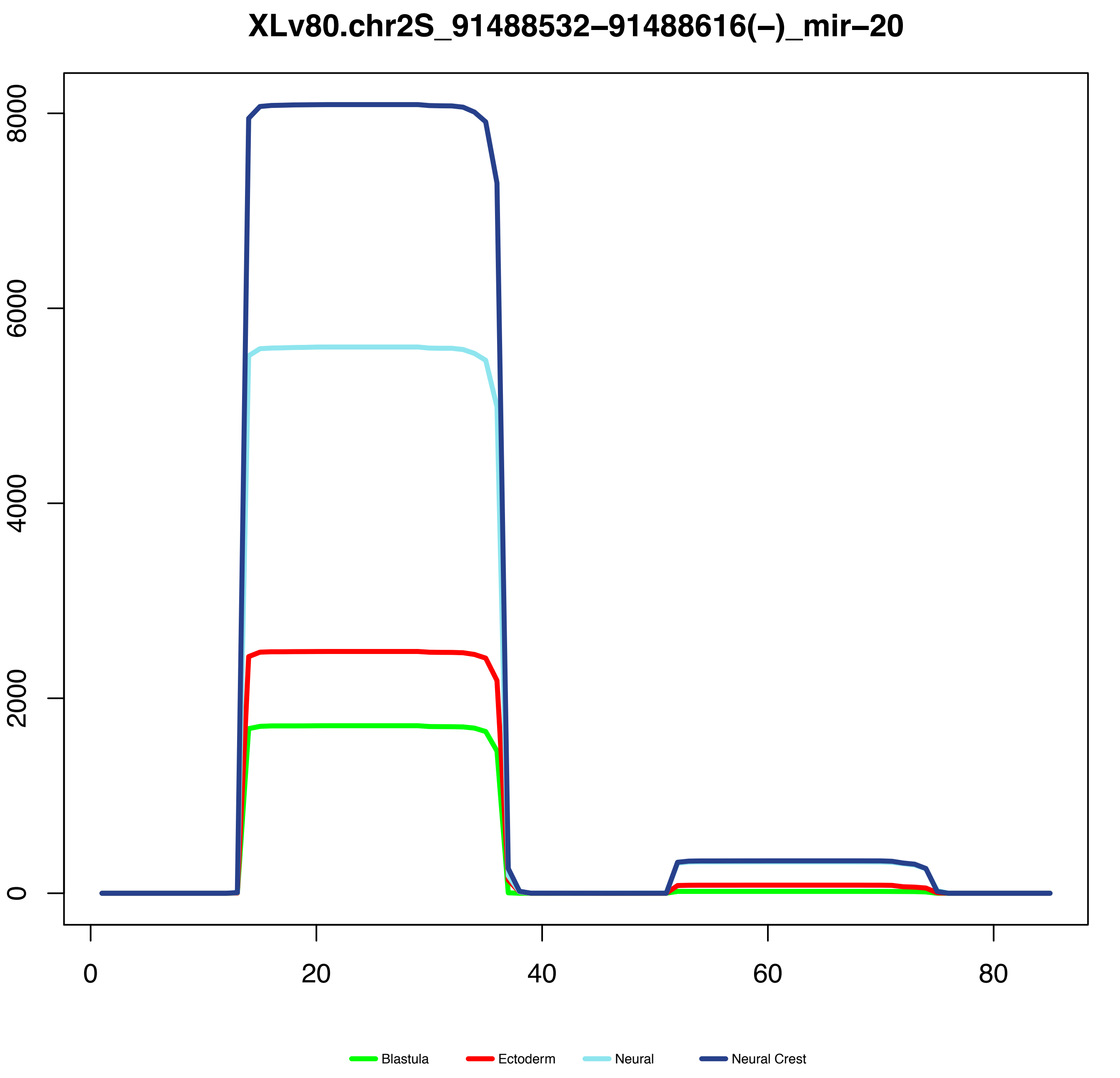
XLv80.chr2S_91488532–91488616(–)_mir–20
8000
6000
4000
2000
0
0
20
40
60
80
Blastula
Ectoderm
Neural
Neural Crest

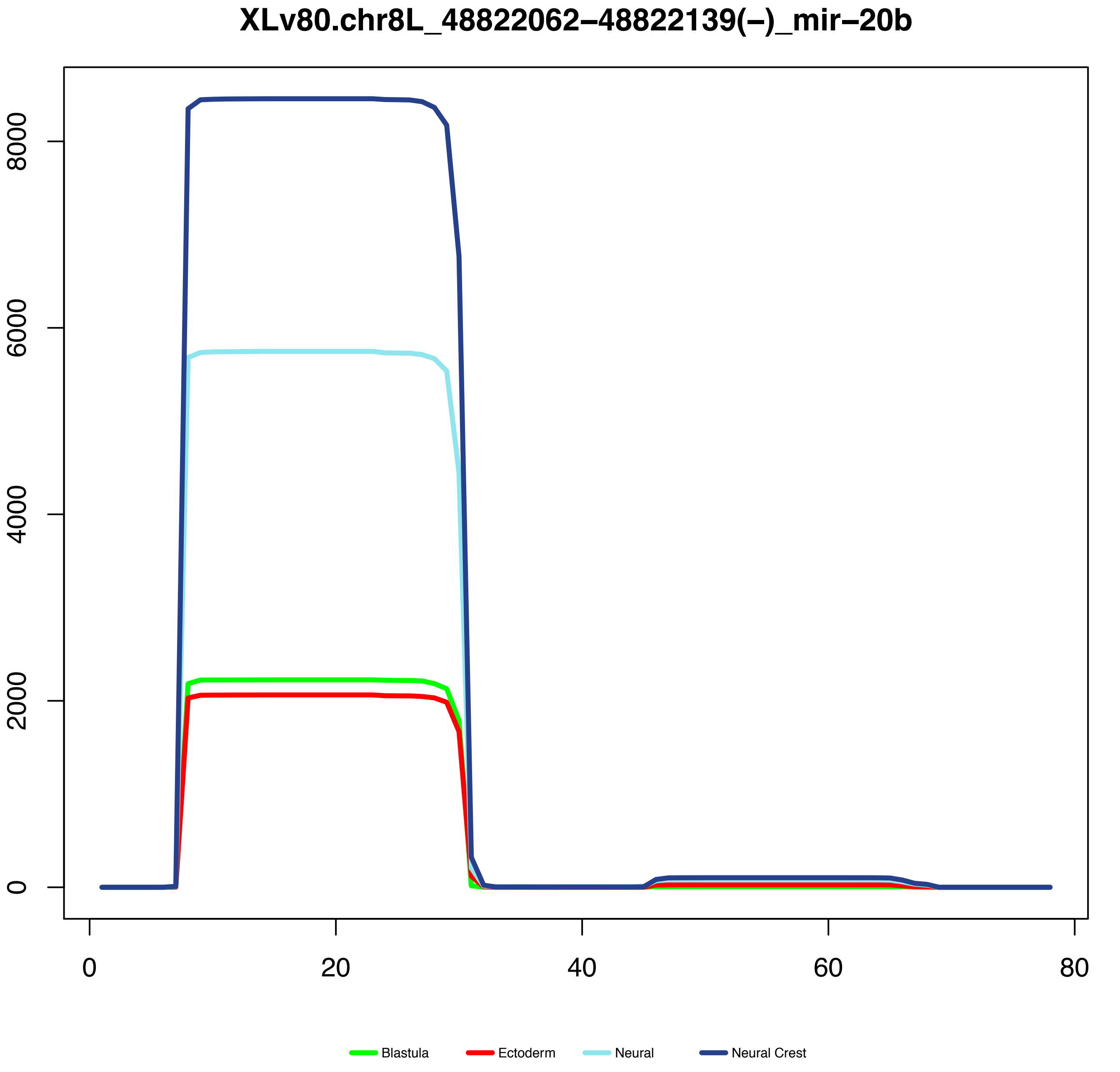
XLv80.chr8L_48822062−48822139(−)_mir−20b
0
2000
4000
6000
8000
0
20
40
60
80
Blastula
Ectoderm
Neural
Neural Crest

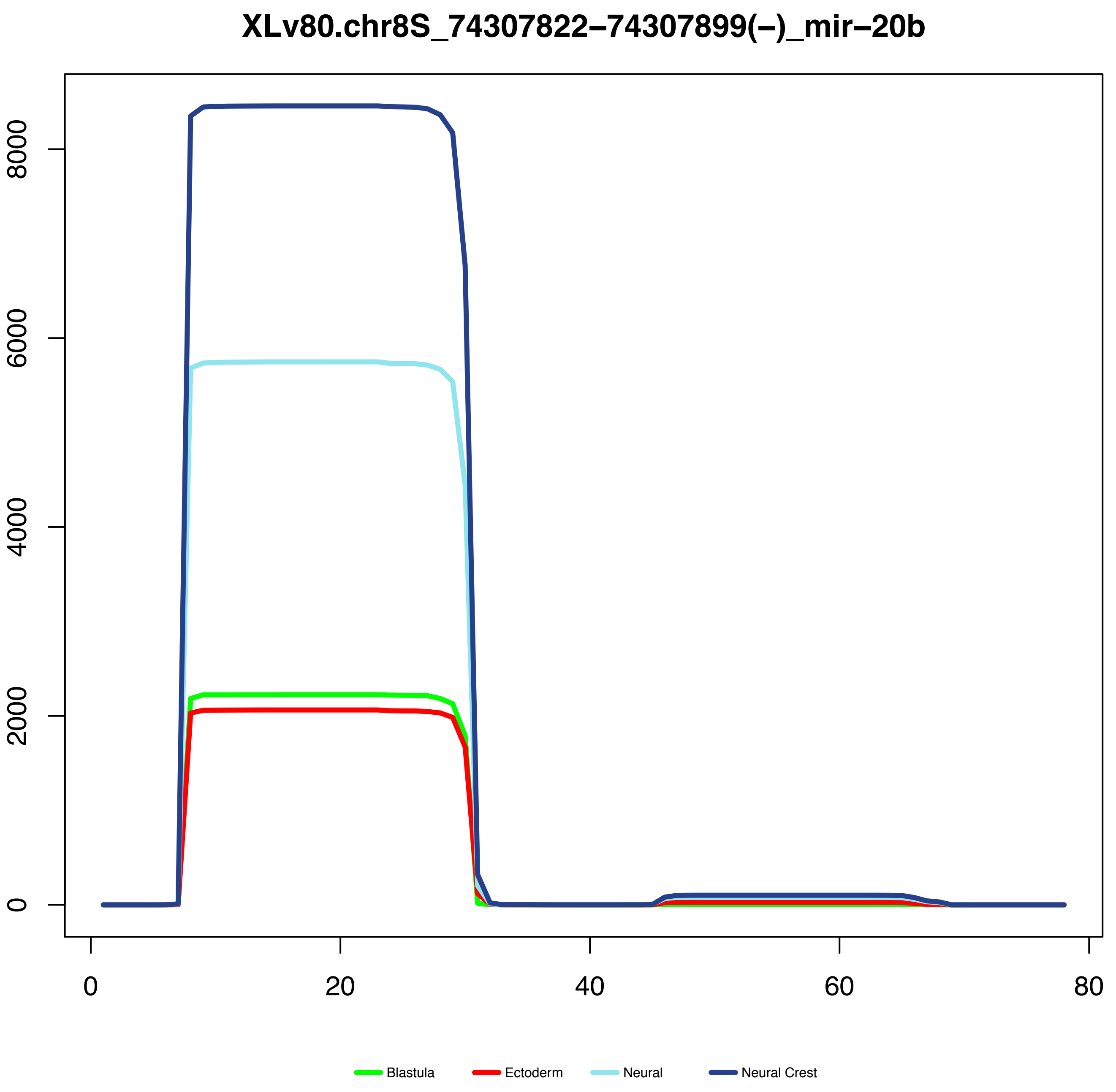
XLv80.chr8S_74307822−74307899(−)_mir−20b
0
2000
4000
6000
8000
0
20
40
60
80
Blastula
Ectoderm
Neural
Neural Crest

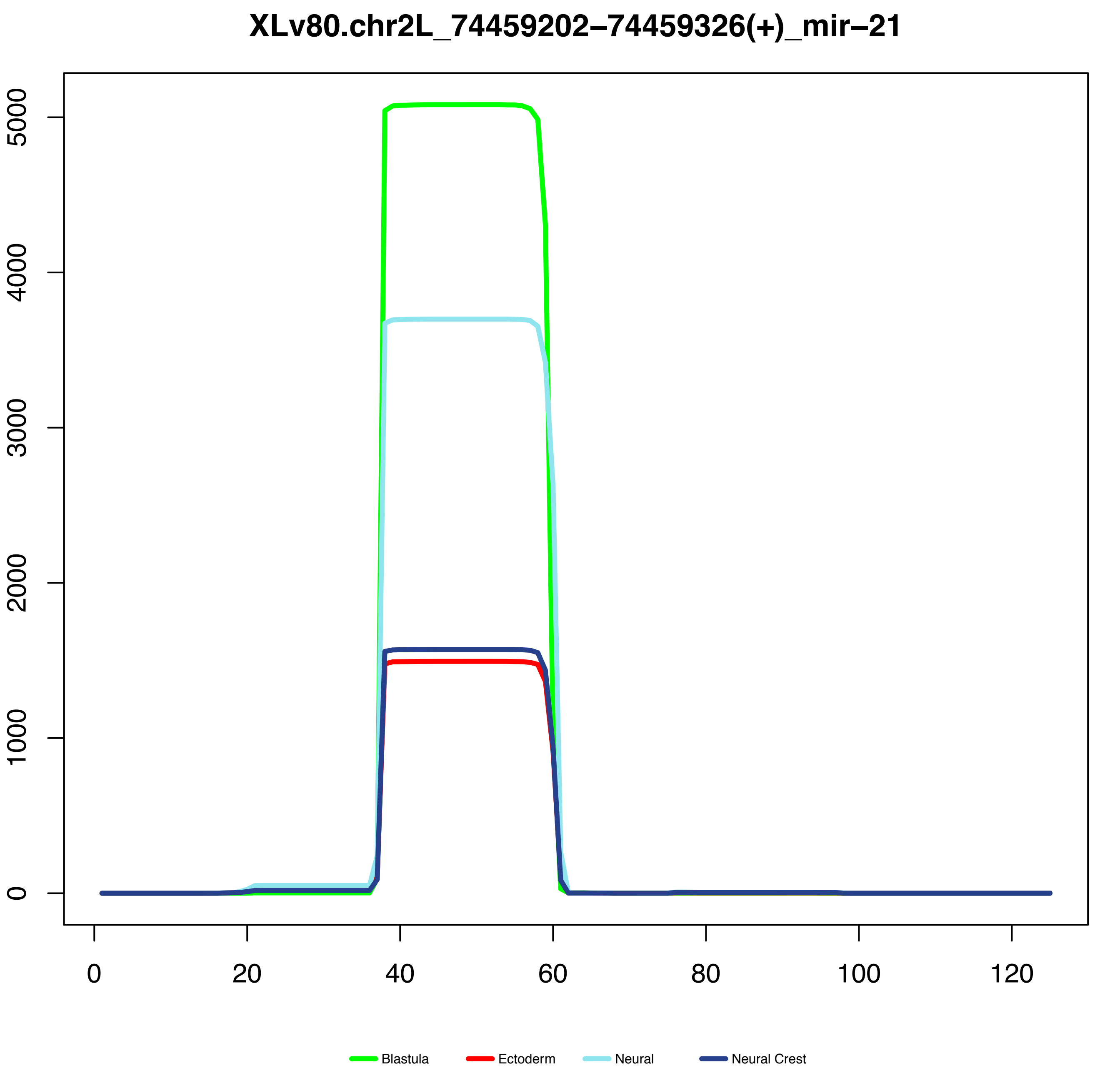

XLv80.chr2L_74459202−74459326(+)_mir−21
0
1000
2000
3000
4000
5000
0
20
40
60
80
100
120
Blastula
Ectoderm
Neural
Neural Crest

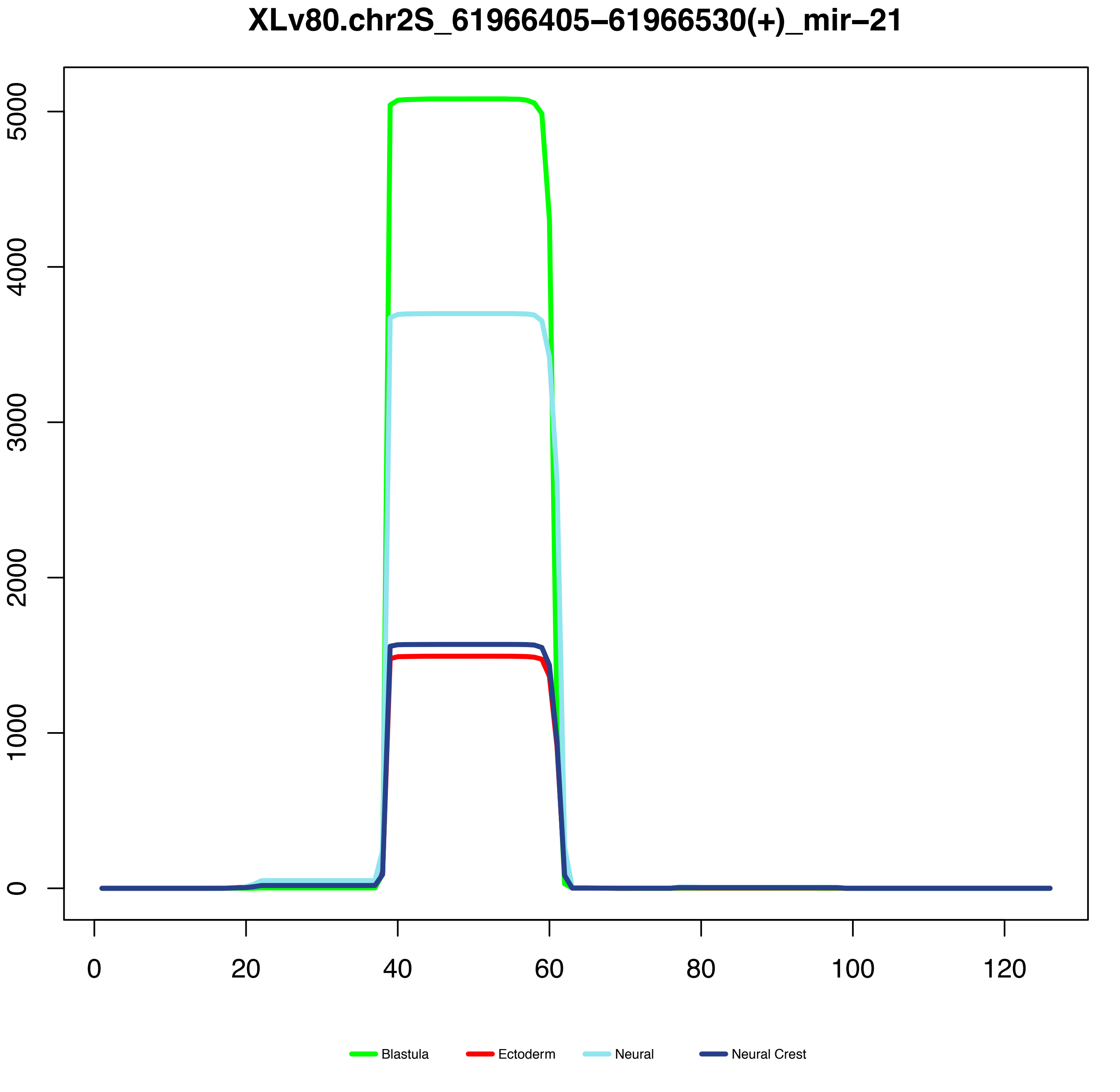
XLv80.chr2S_61966405−61966530(+)_mir−21
0
1000
2000
3000
4000
5000
0
20
40
60
80
100
120
Blastula
Ectoderm
Neural
Neural Crest

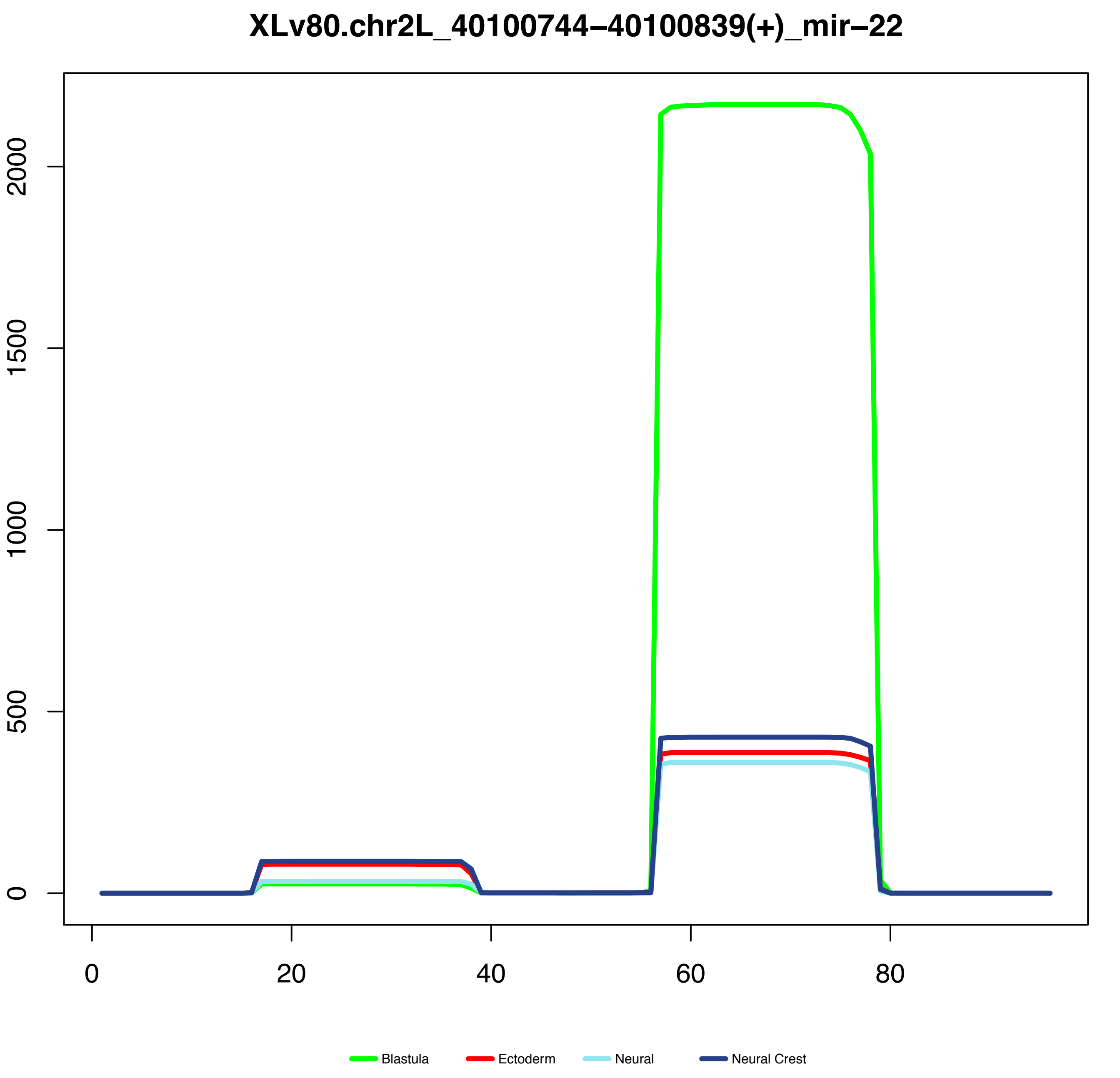
XLv80.chr2L_40100744−40100839(+)_mir−22
0
500
1000
1500
2000
0
20
40
60
80
Blastula
Ectoderm
Neural
Neural Crest

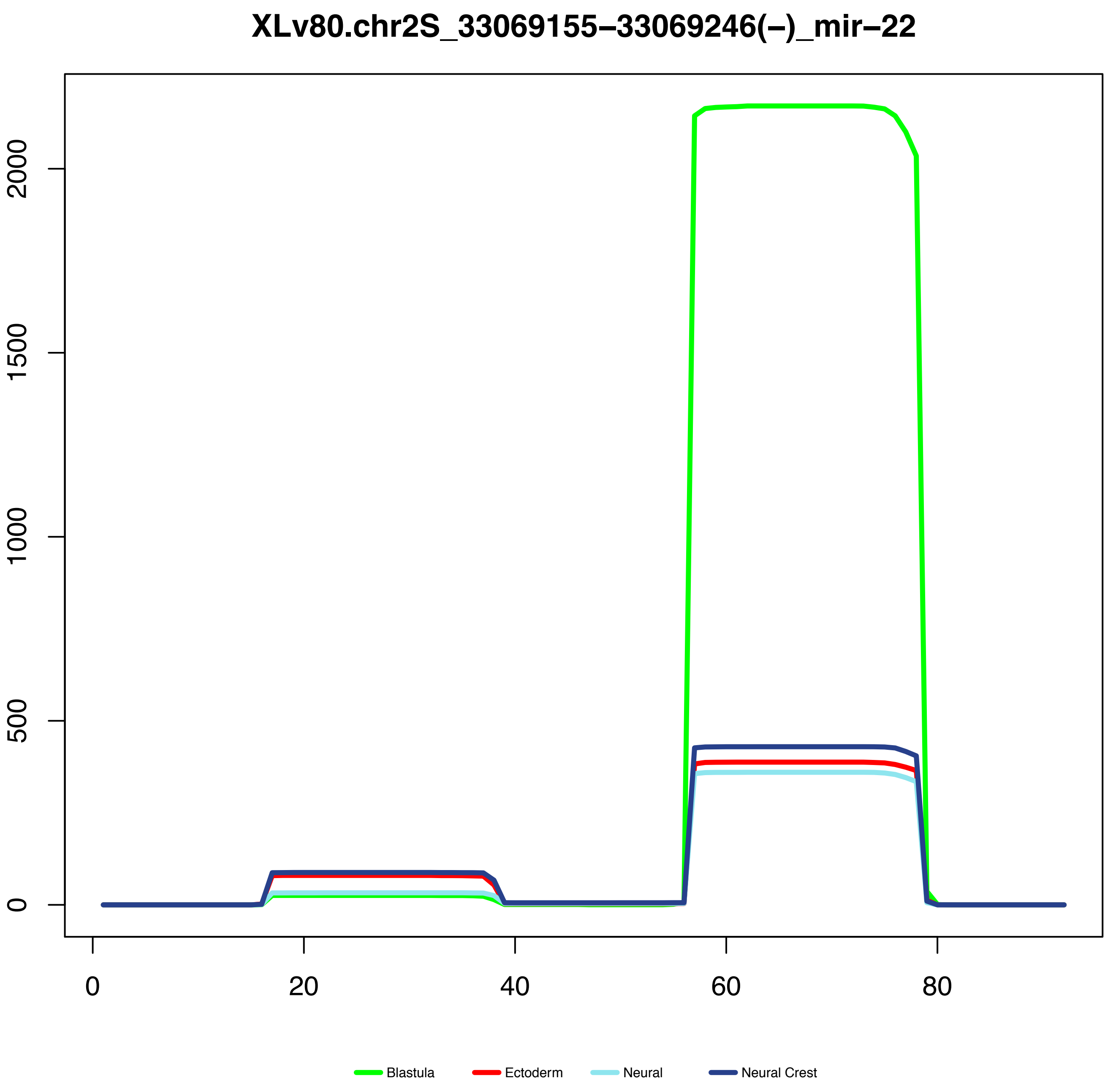
XLv80.chr2S_33069155–33069246(–)_mir–22
0
500
1000
1500
2000
0
20
40
60
80
Blastula
Ectoderm
Neural
Neural Crest

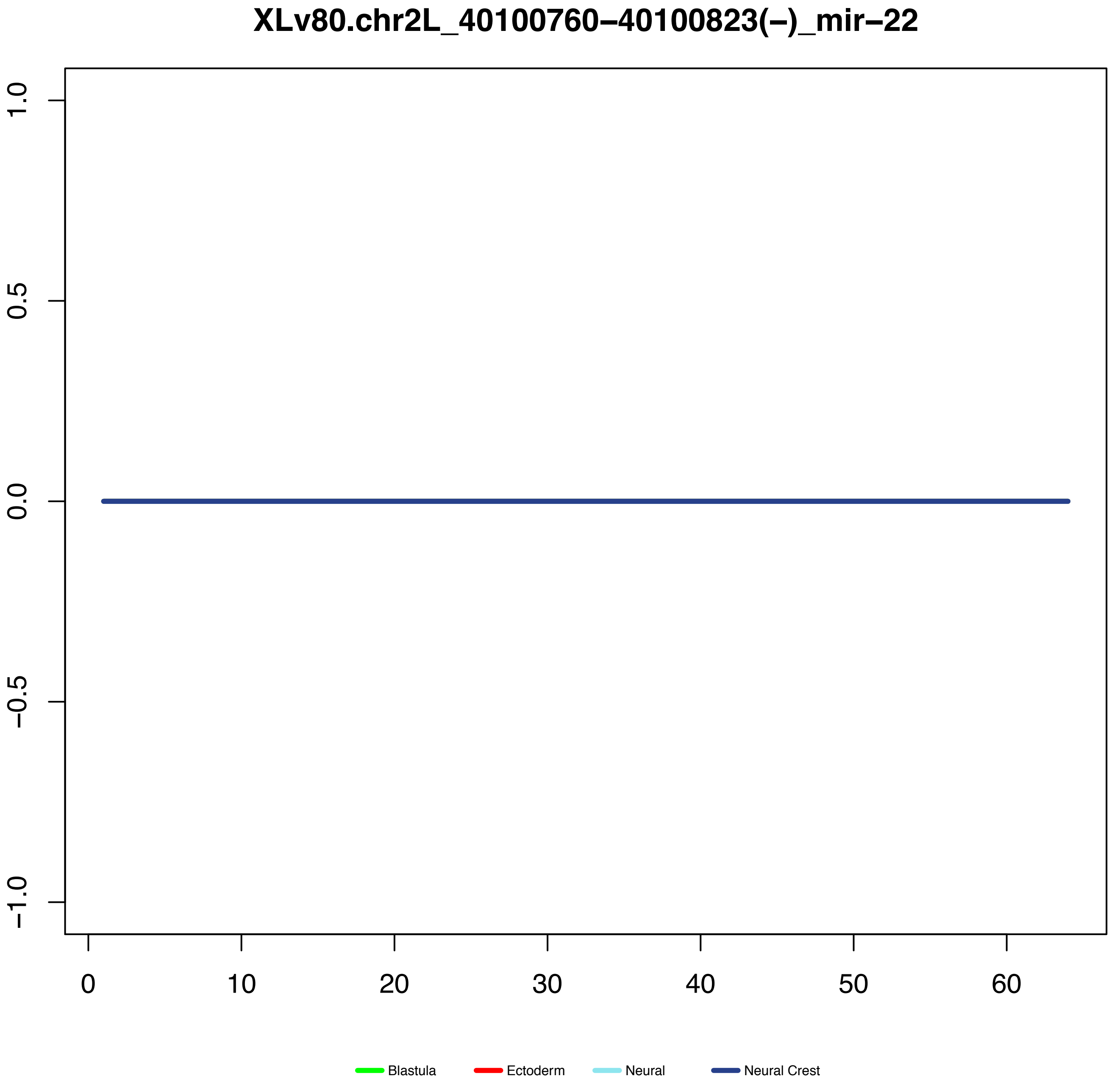

XLv80.chr2L_40100760−40100823(−)_mir−22
1.0
0.5
0.0
−0.5
−1.0
0
10
20
30
40
50
60
Blastula
Ectoderm
Neural
Neural Crest

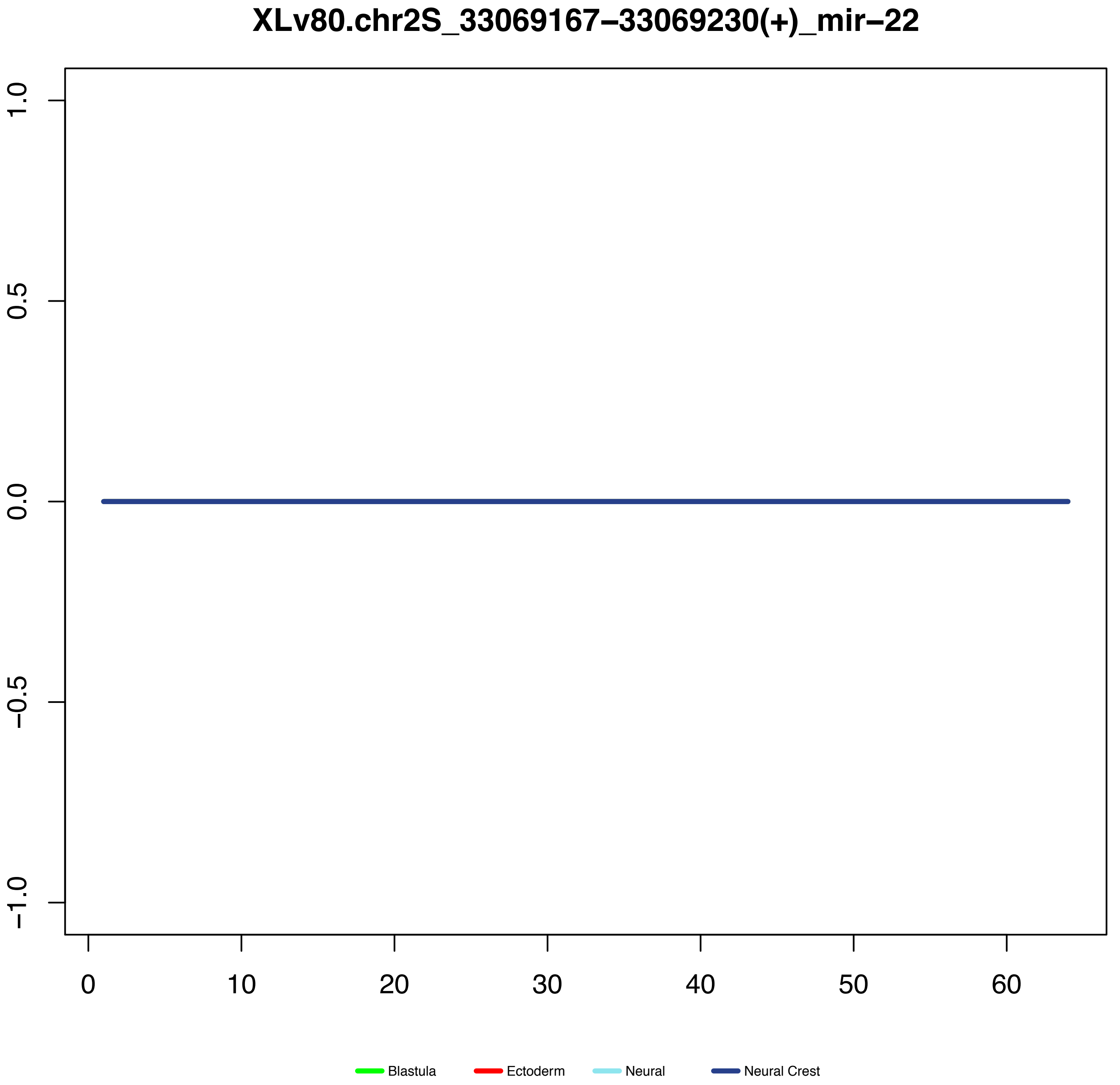
XLv80.chr2S_33069167−33069230(+)_mir−22
1.0
0.5
0.0
−0.5
−1.0
0
10
20
30
40
50
60
Blastula
Ectoderm
Neural
Neural Crest

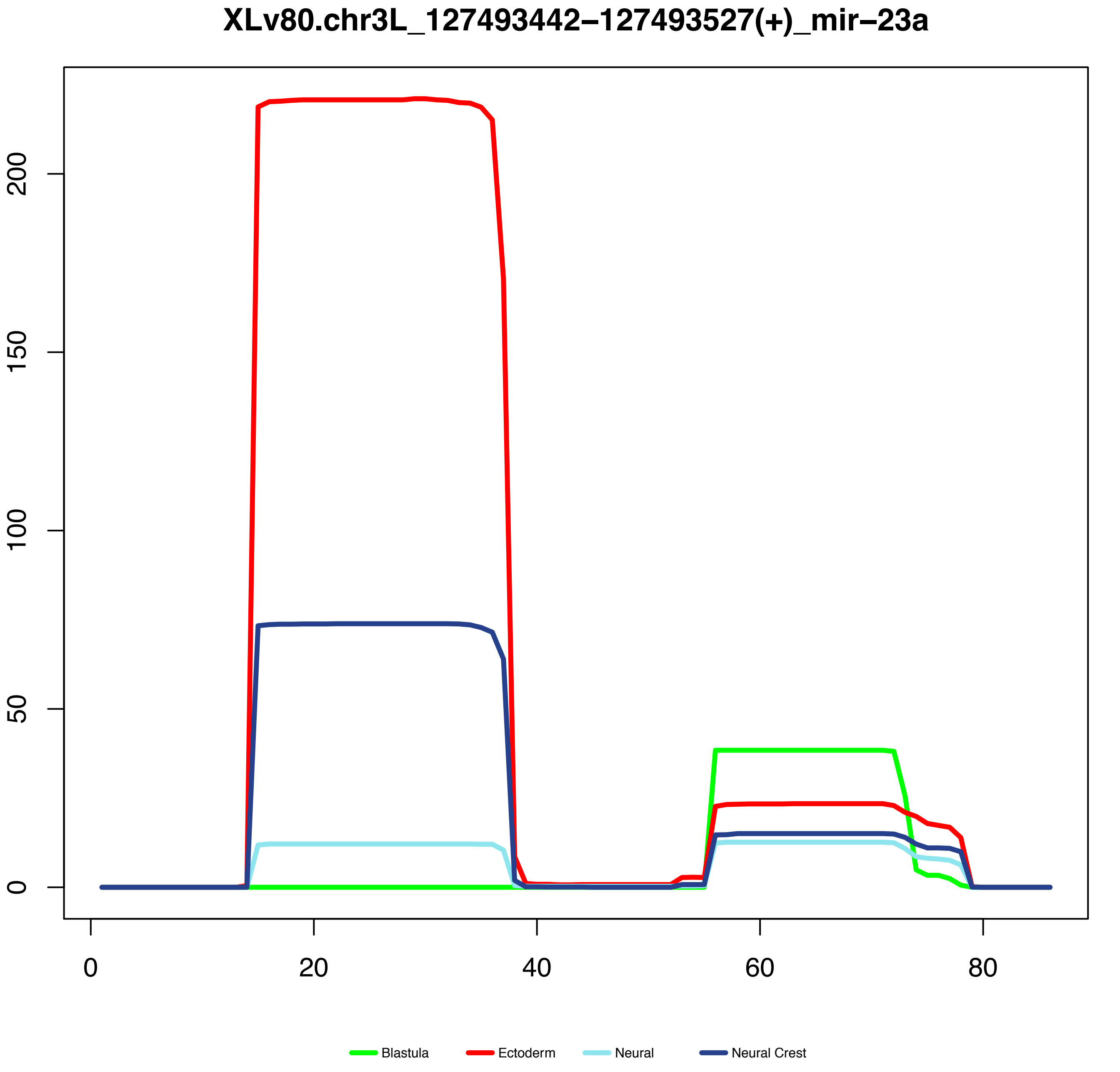

XLv80.chr3L_127493442−127493527(+)_mir−23a
0
50
100
150
200
0
20
40
60
80
Blastula
Ectoderm
Neural
Neural Crest

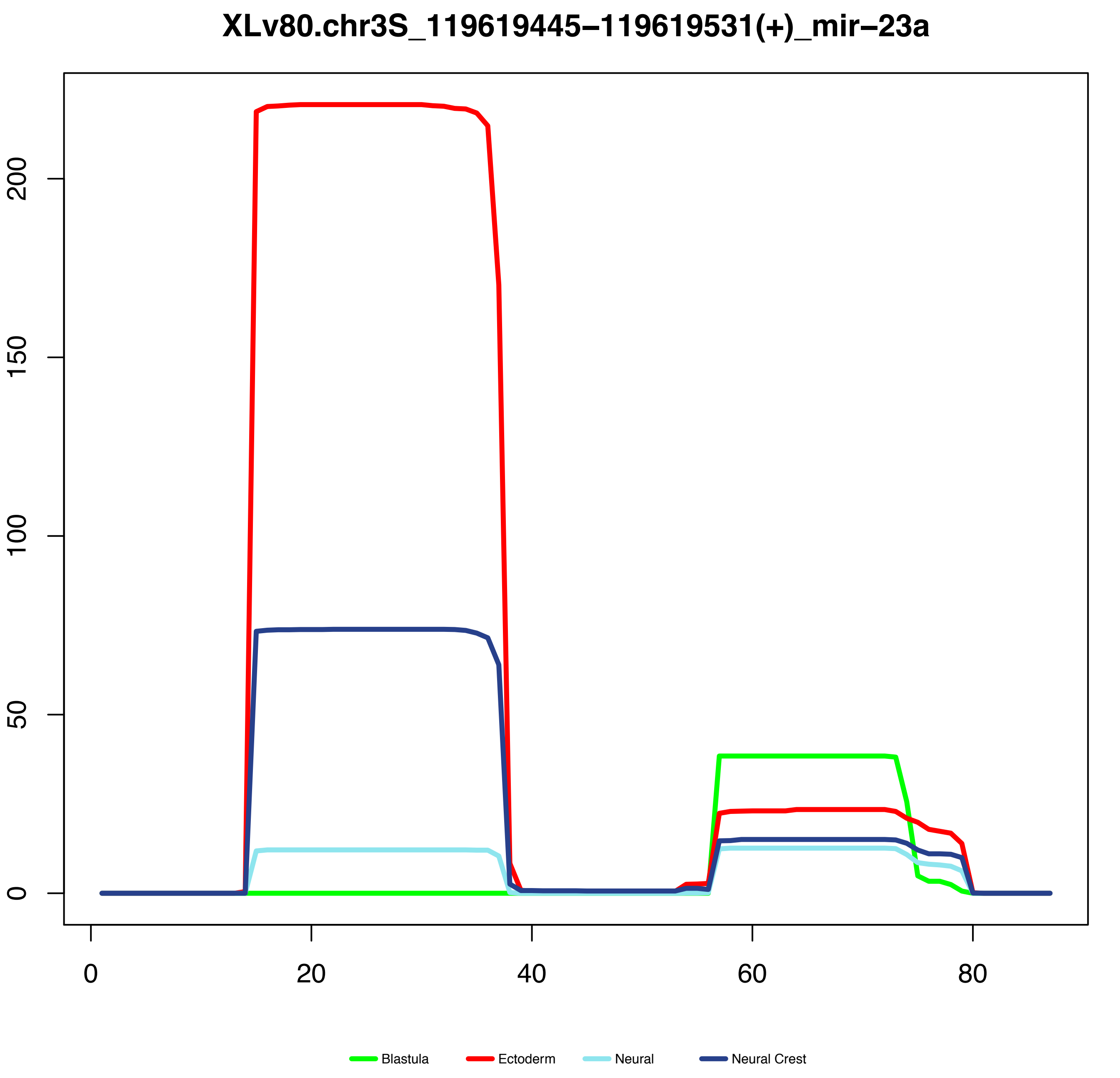
XLv80.chr3S_119619445−119619531(+)_mir−23a
0
50
100
150
200
0
20
40
60
80
Blastula
Ectoderm
Neural
Neural Crest

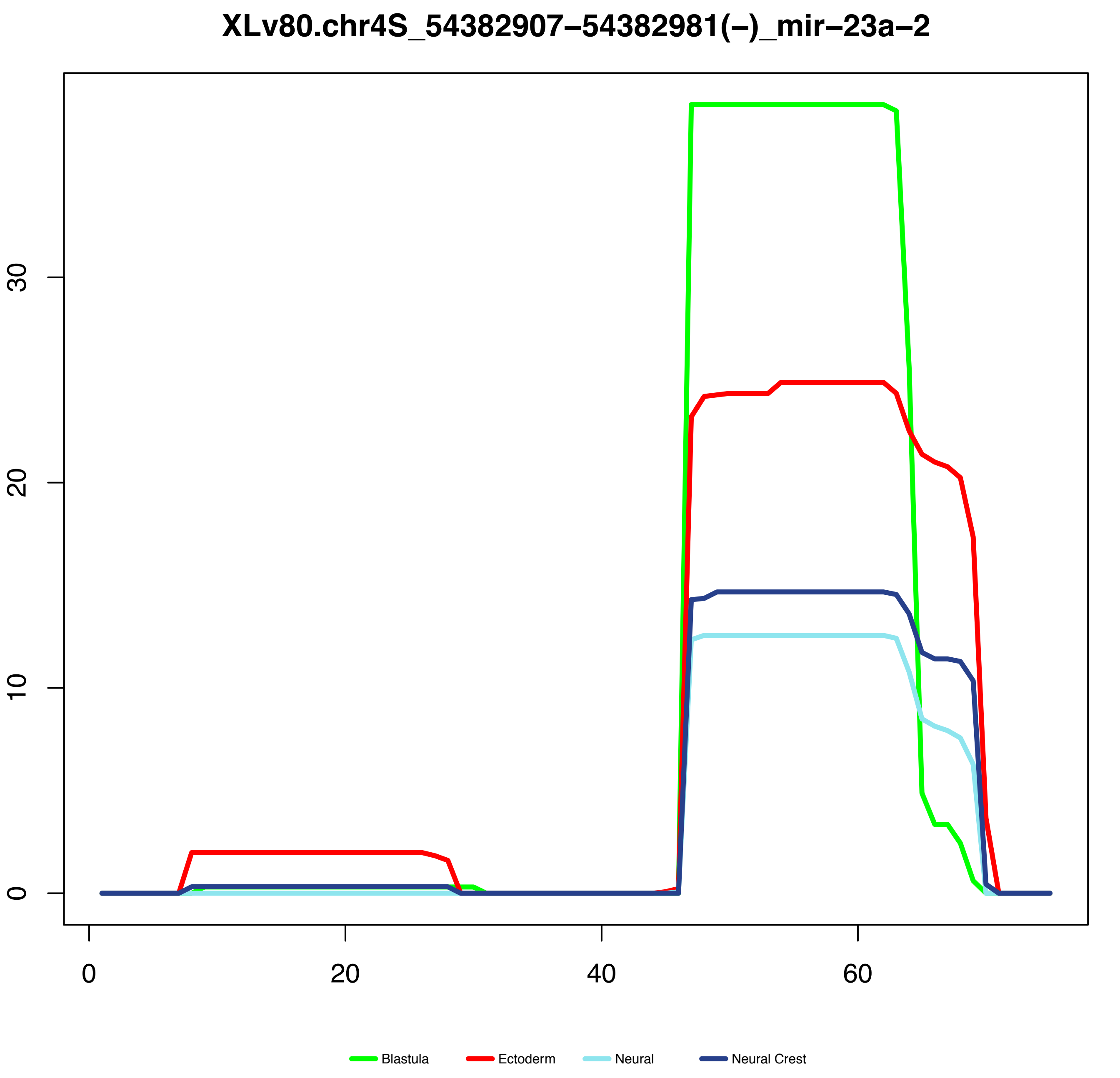
XLv80.chr4S_54382907−54382981(−)_mir−23a−2
0
10
20
30
0
20
40
60
Blastula
Ectoderm
Neural
Neural Crest

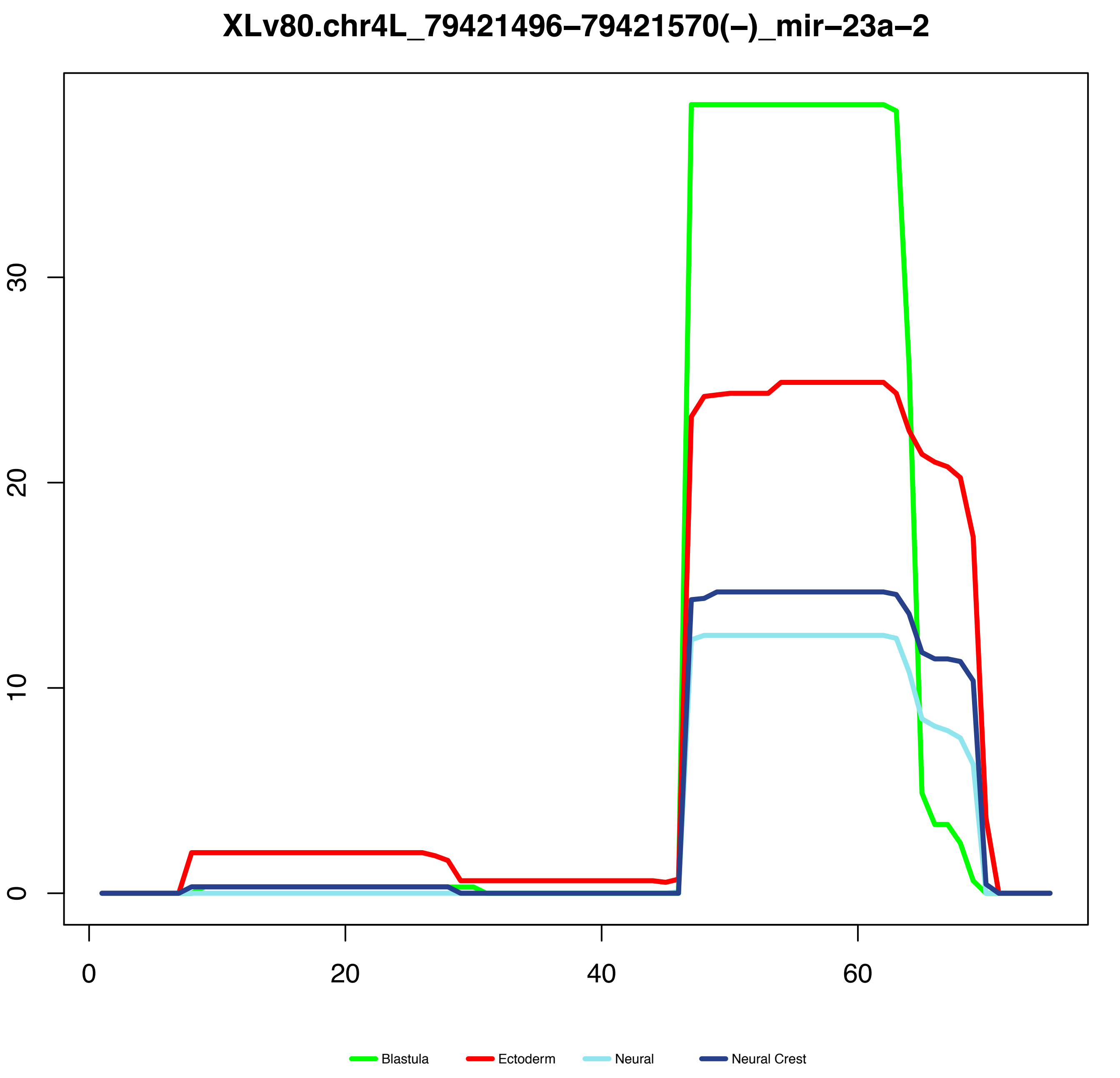
XLv80.chr4L_79421496−79421570(−)_mir−23a−2
0
10
20
30
0
20
40
60
Blastula
Ectoderm
Neural
Neural Crest

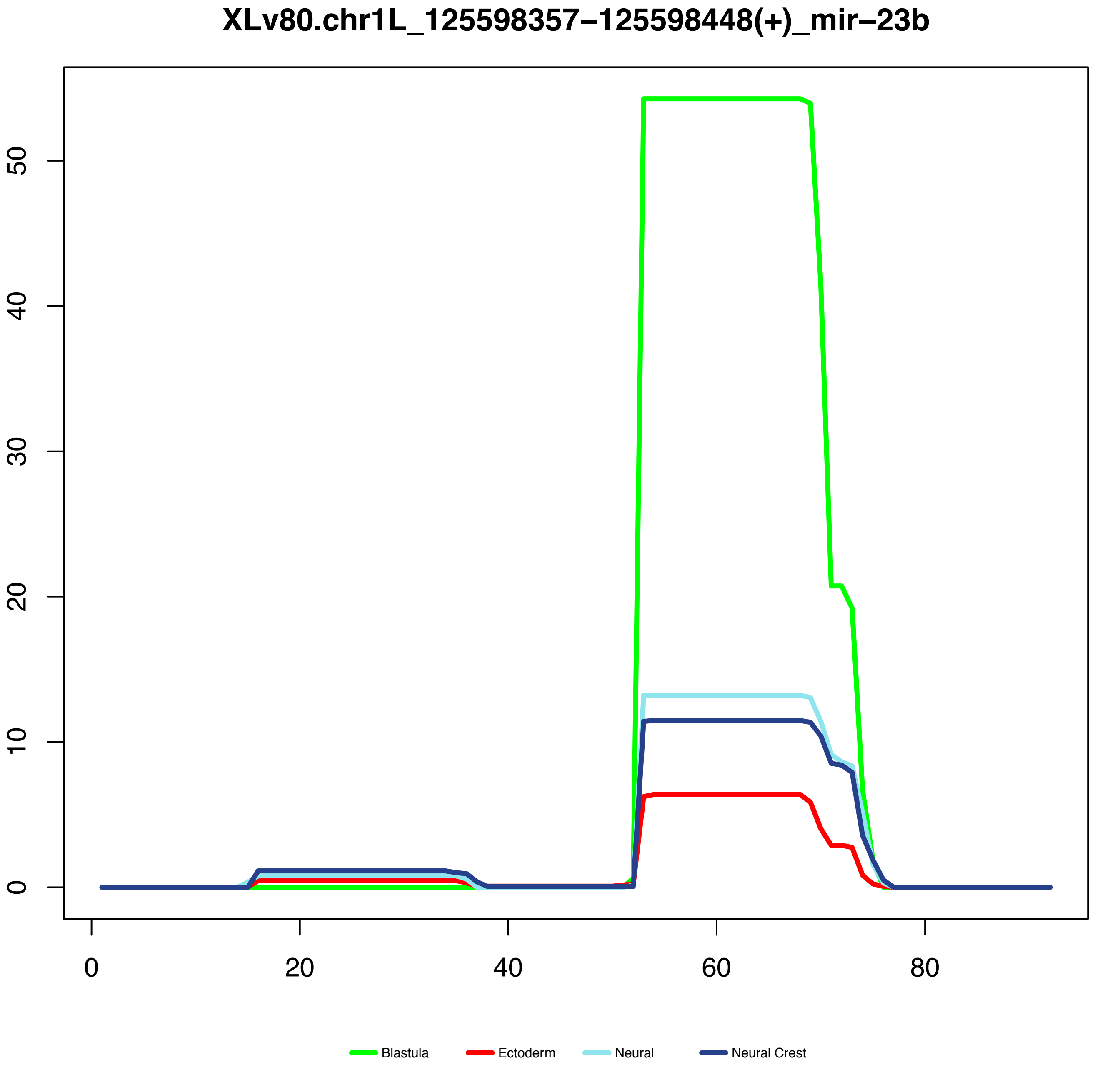

XLv80.chr1L_125598357−125598448(+)_mir−23b
0
10
20
30
40
50
0
20
40
60
80
Blastula
Ectoderm
Neural
Neural Crest

XLv80.chr1S_117534183−117534273(+)_mir−23b

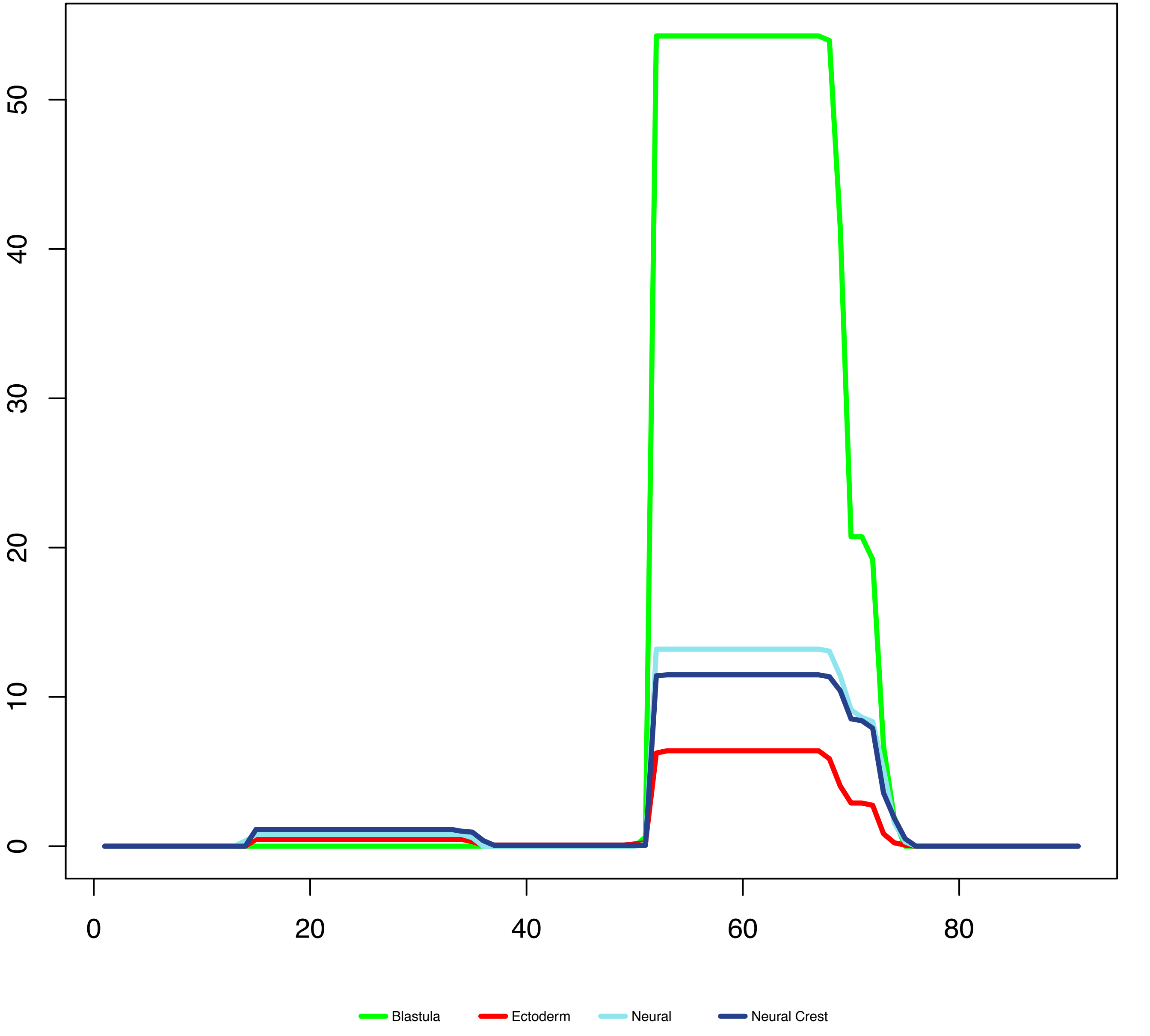

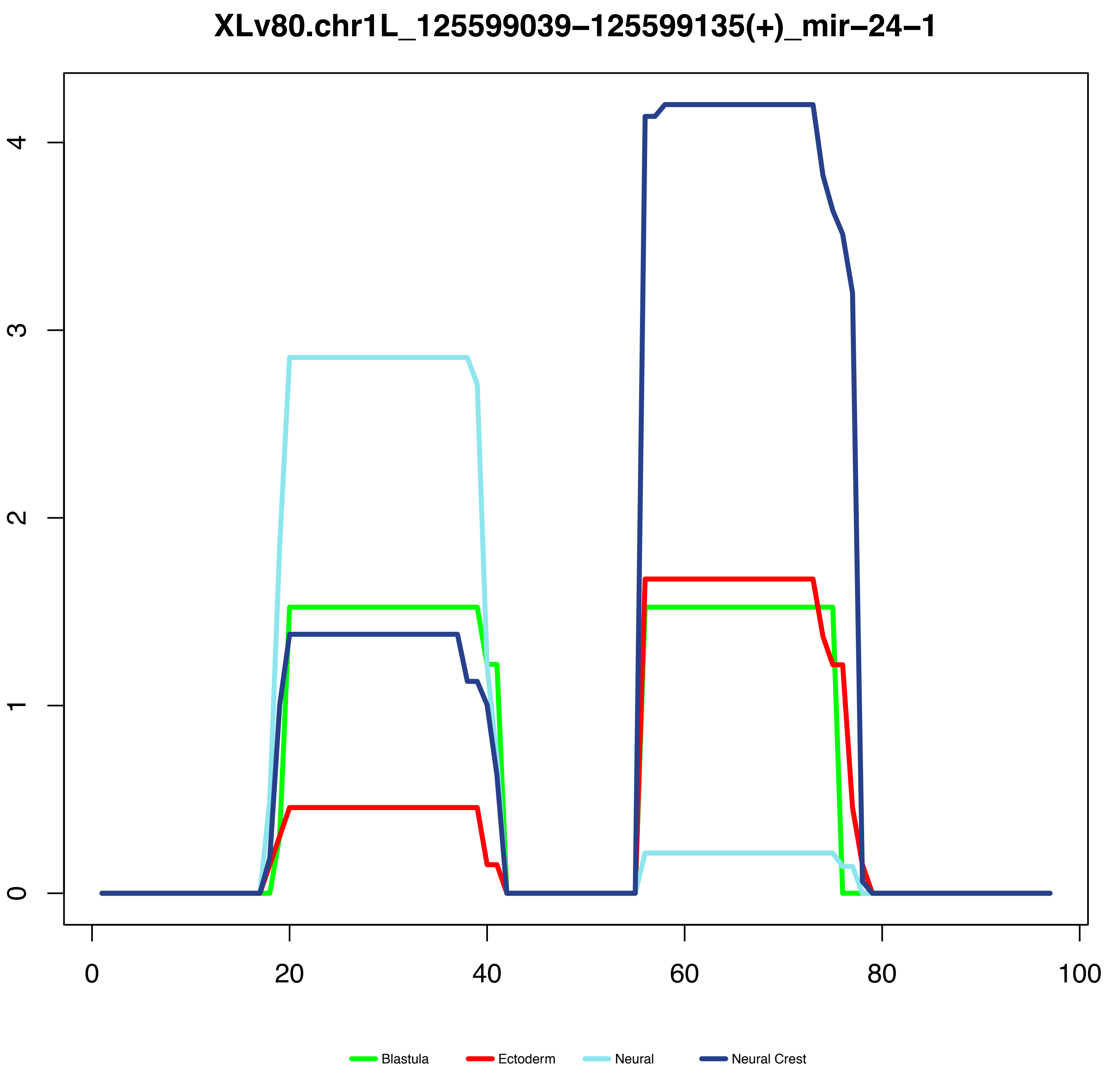
XLv80.chr1L_125599039–125599135(+)_mir–24–1
0
1
2
3
4
0
20
40
60
80
100
Blastula
Ectoderm
Neural
Neural Crest

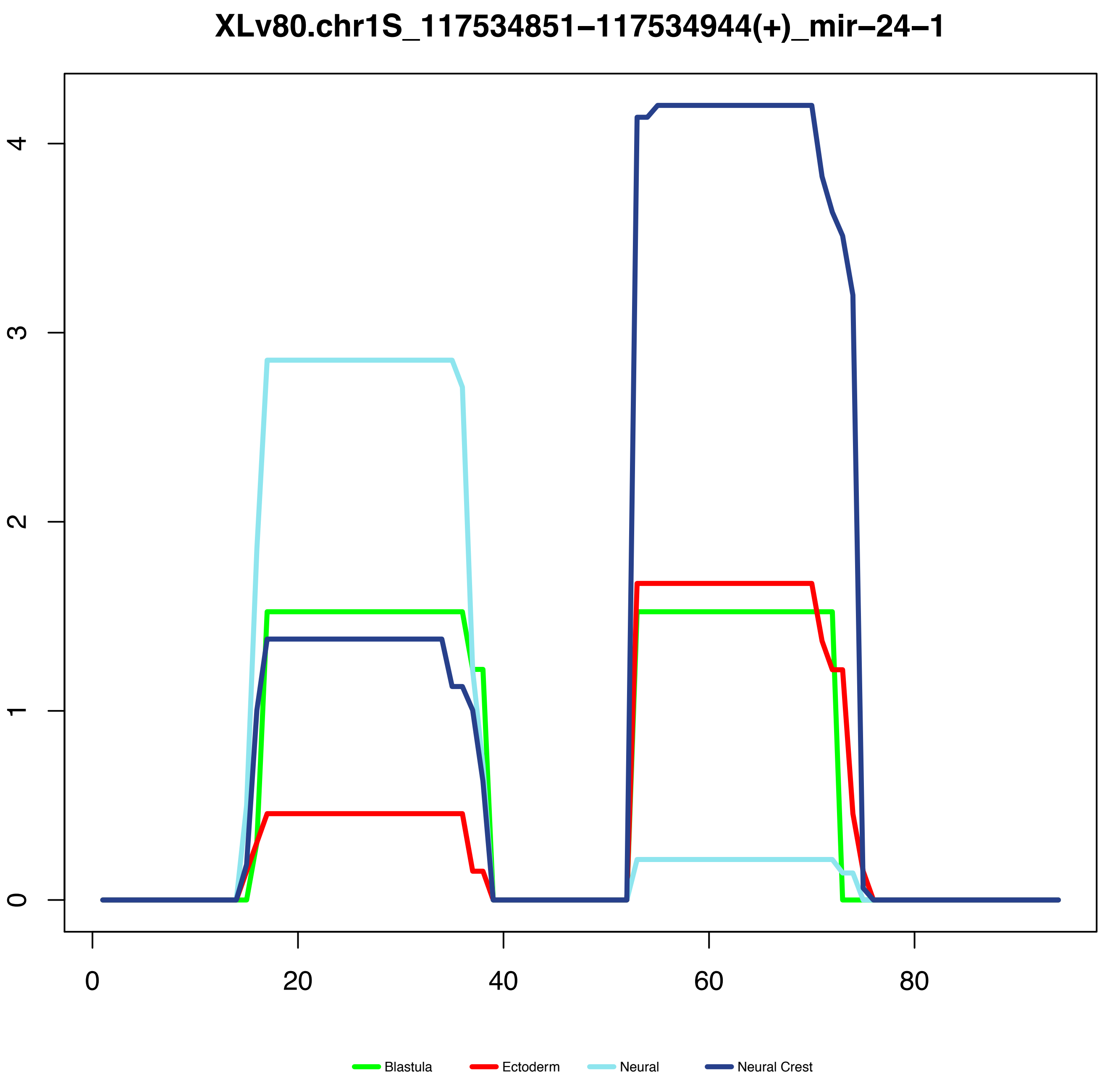
XLv80.chr1S_117534851−117534944(+)_mir−24−1
0
1
2
3
4
0
20
40
60
80
Blastula
Ectoderm
Neural
Neural Crest

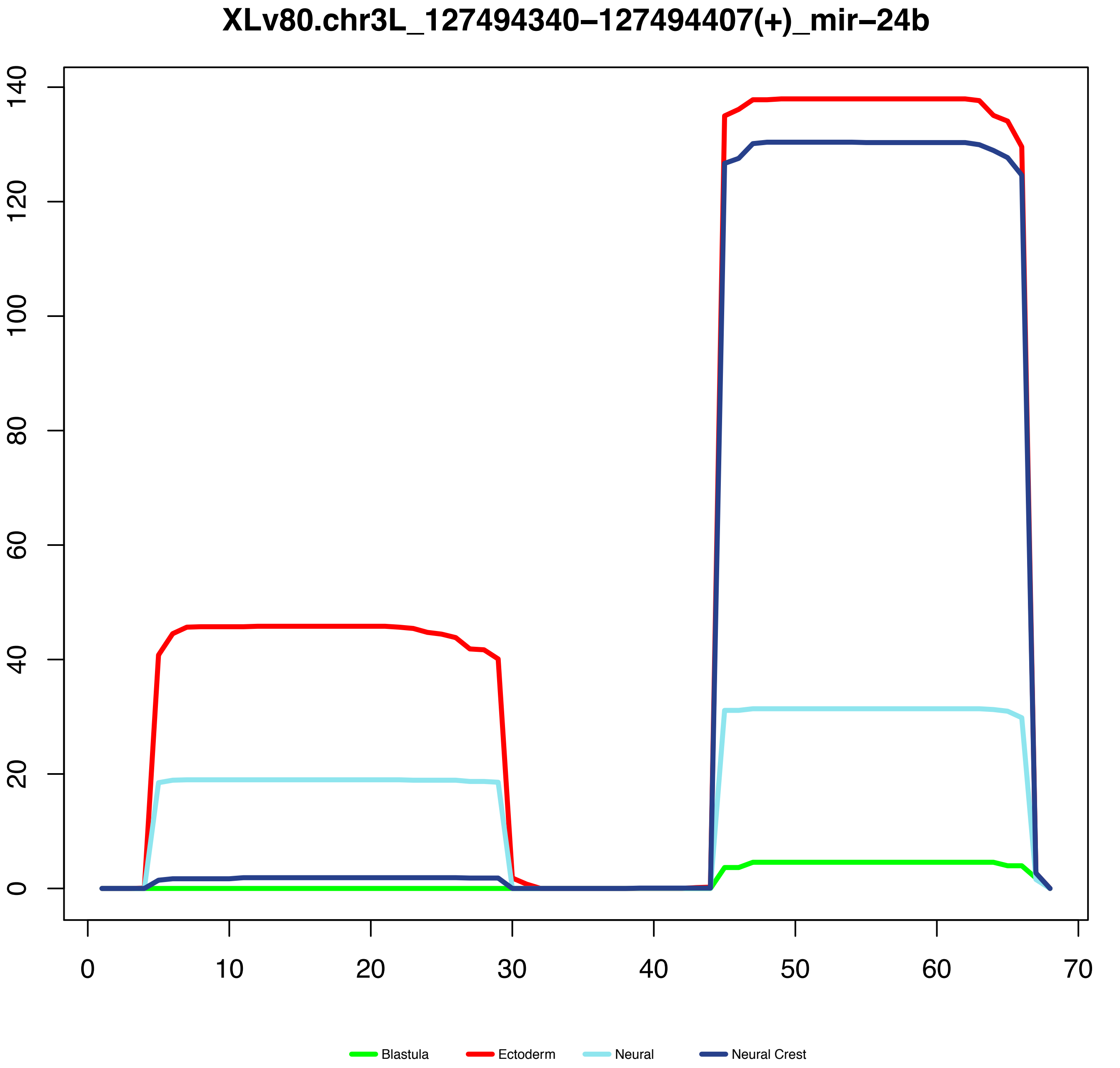
XLv80.chr3L_127494340−127494407(+)_mir−24b
0
20
40
60
80
100
120
140
0
10
20
30
40
50
60
70
Blastula
Ectoderm
Neural
Neural Crest

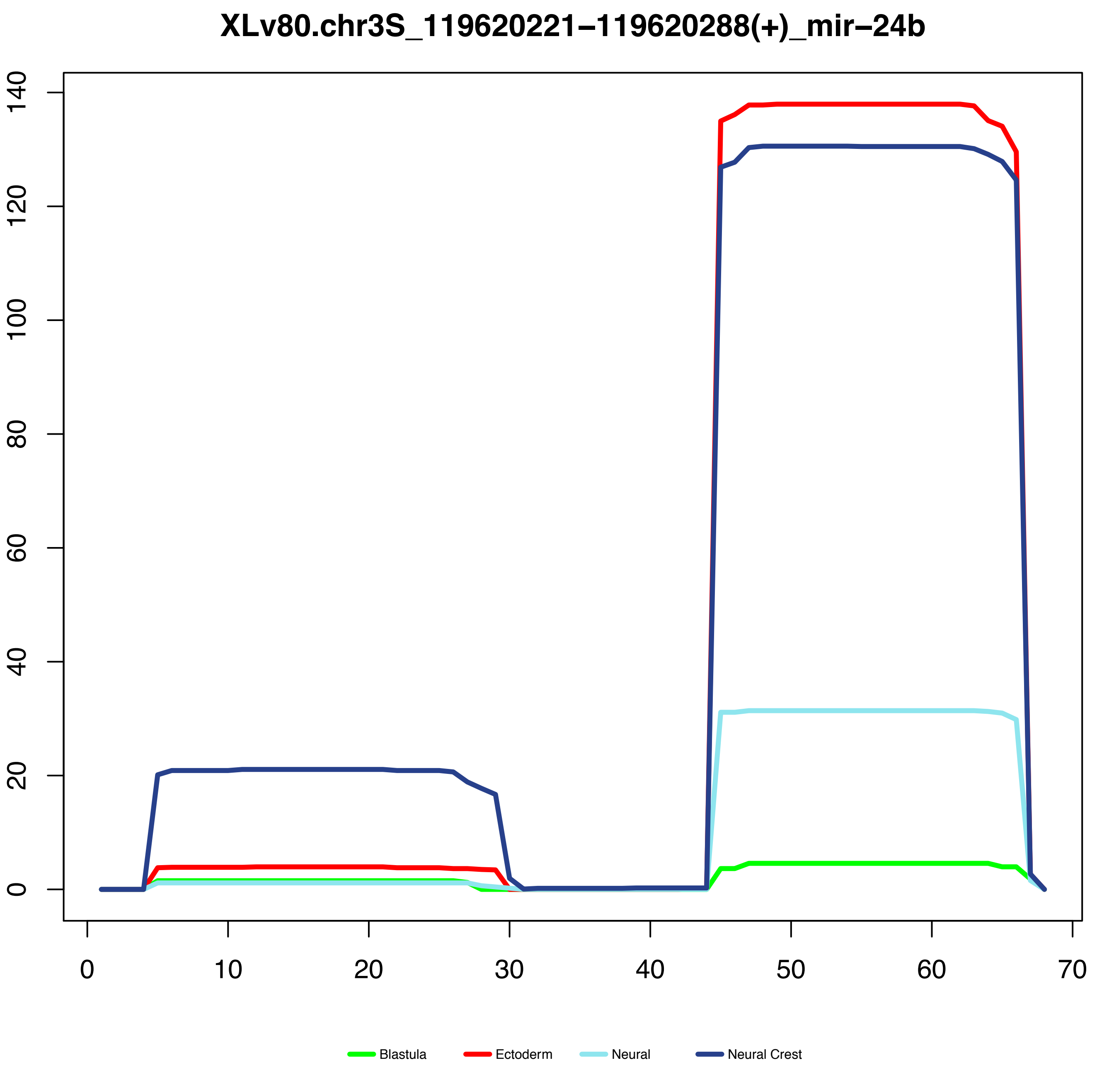
XLv80.chr3S_119620221−119620288(+)_mir−24b
0
20
40
60
80
100
120
140
0
10
20
30
40
50
60
70
Blastula
Ectoderm
Neural
Neural Crest

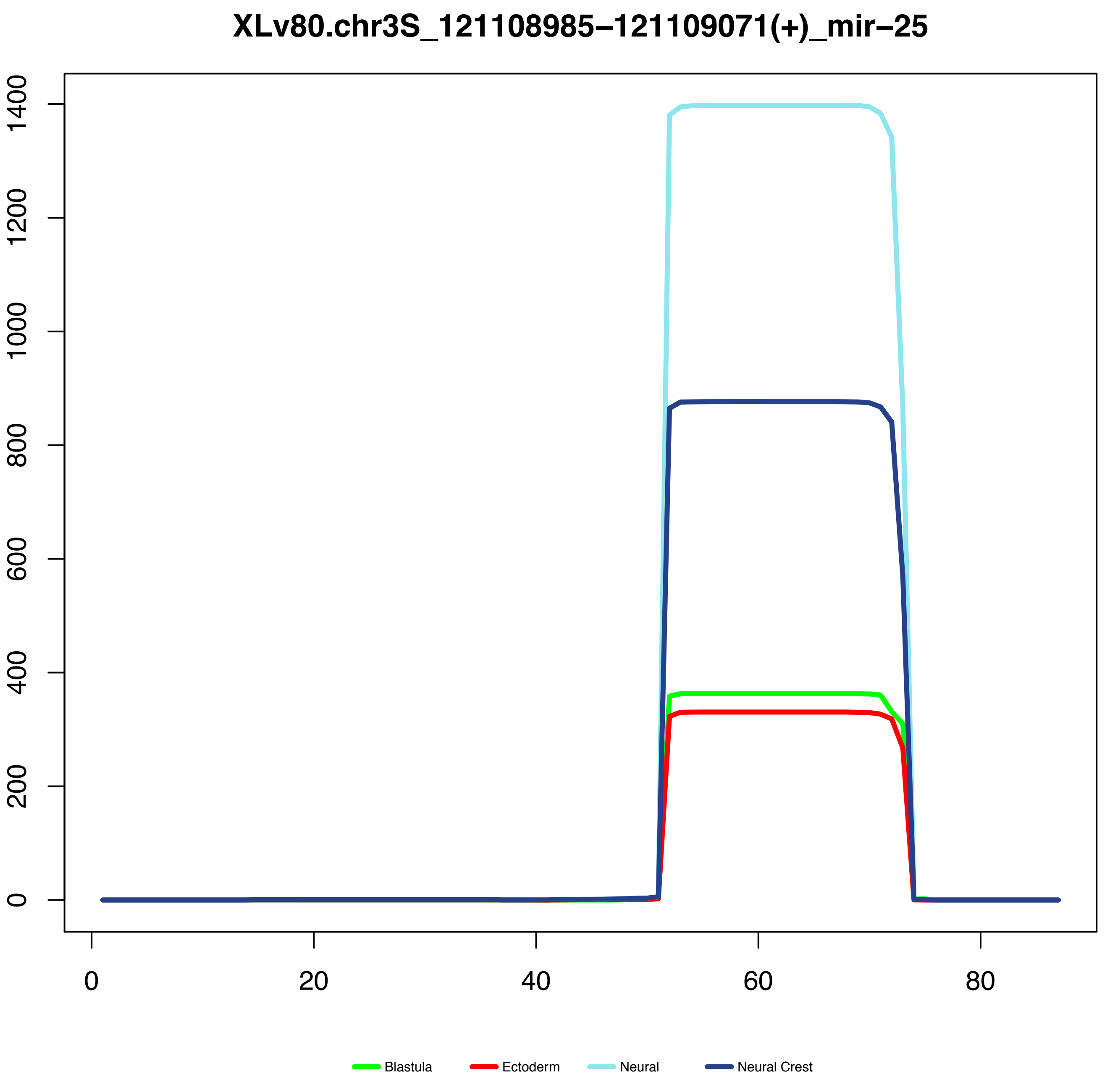

XLv80.chr3S_121108985−121109071(+)_mir−25
0
200
400
600
800
1000
1200
1400
0
20
40
60
80
Blastula
Ectoderm
Neural
Neural Crest

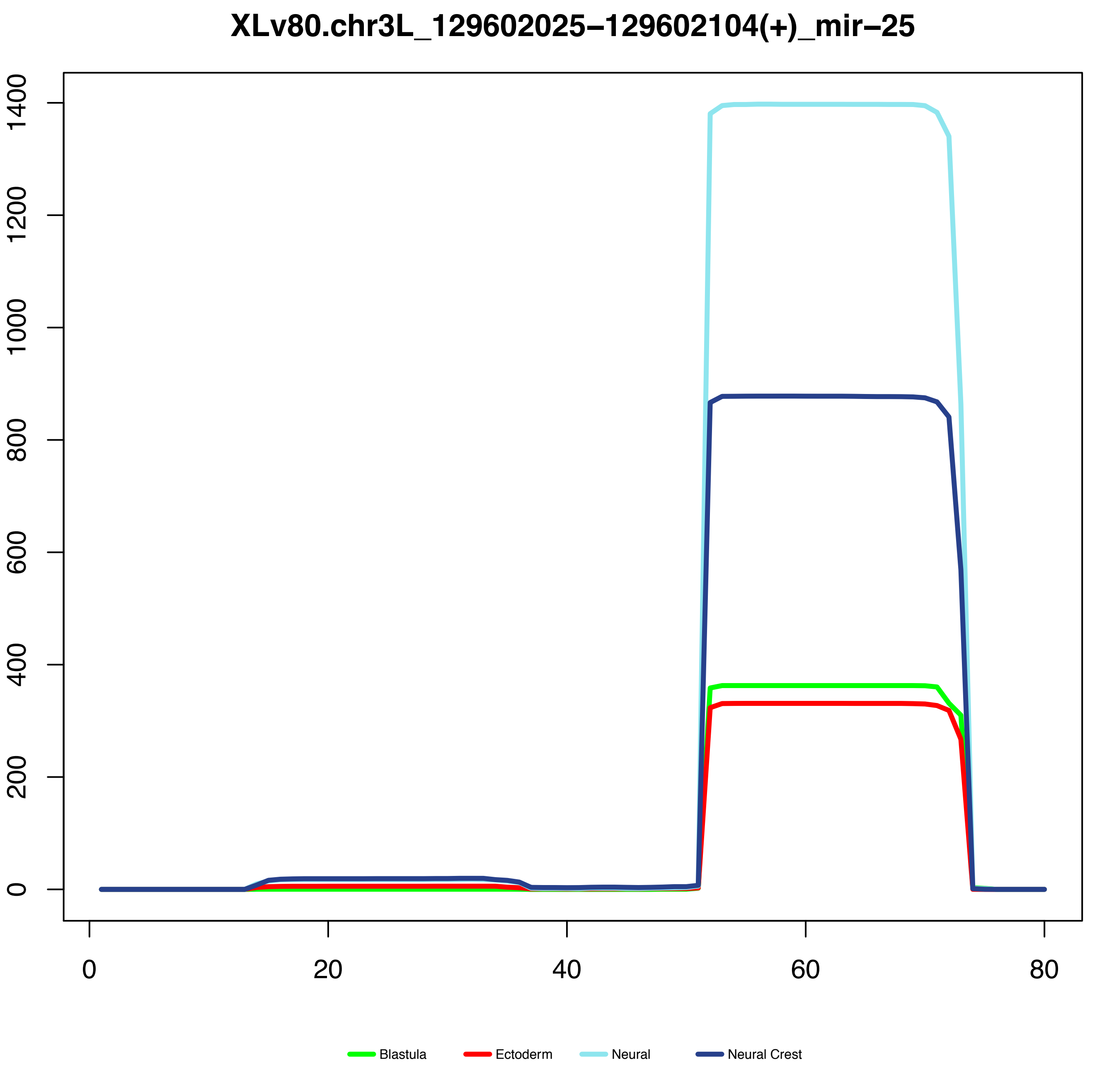

XLv80.chr3L_129602025−129602104(+)_mir−25
0
200
400
600
800
1000
1200
1400
0
20
40
60
80
Blastula
Ectoderm
Neural
Neural Crest

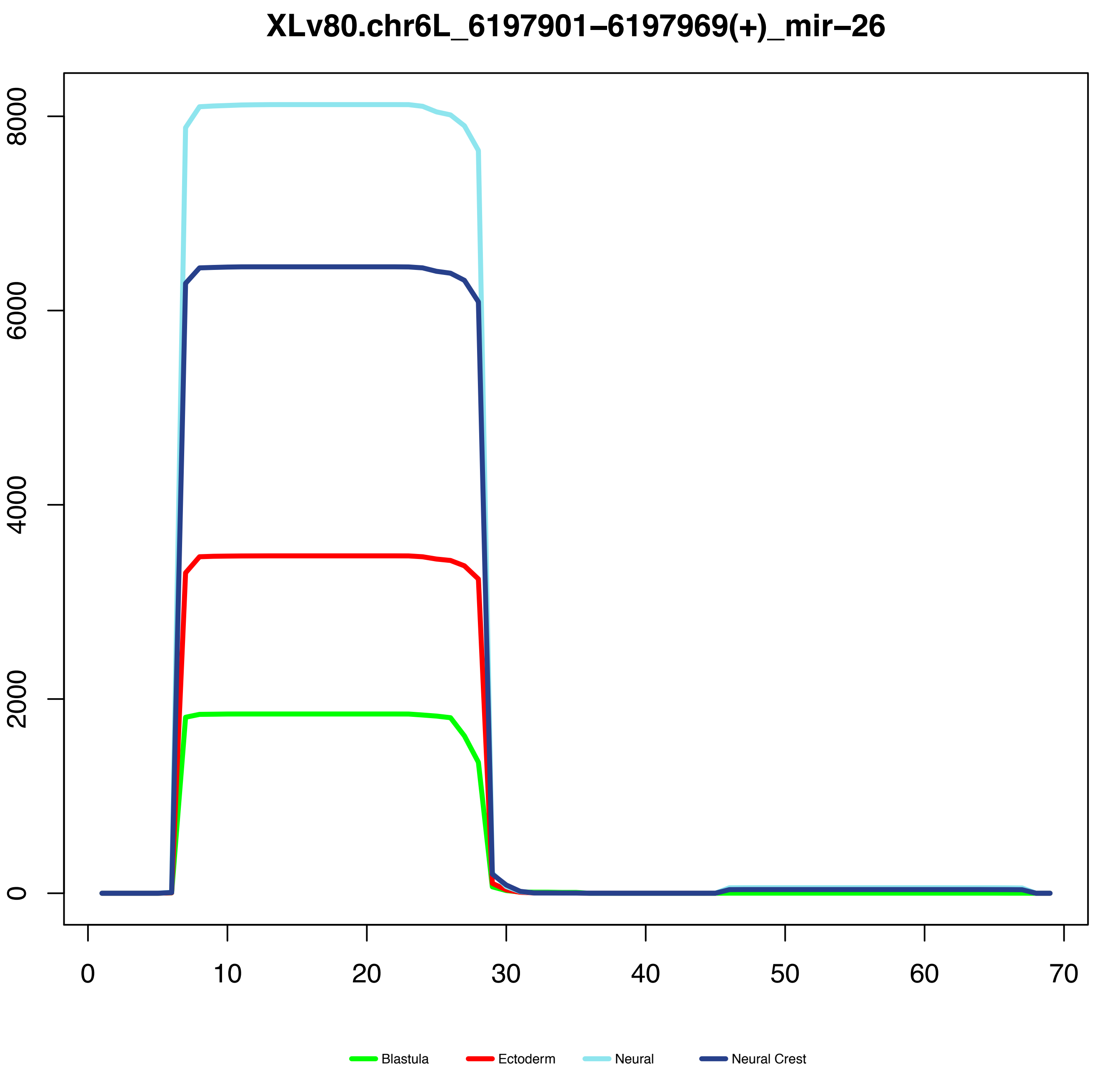

XLv80.chr6L_6197901−6197969(+)_mir−26
0
2000
4000
6000
8000
0
10
20
30
40
50
60
70
Blastula
Ectoderm
Neural
Neural Crest

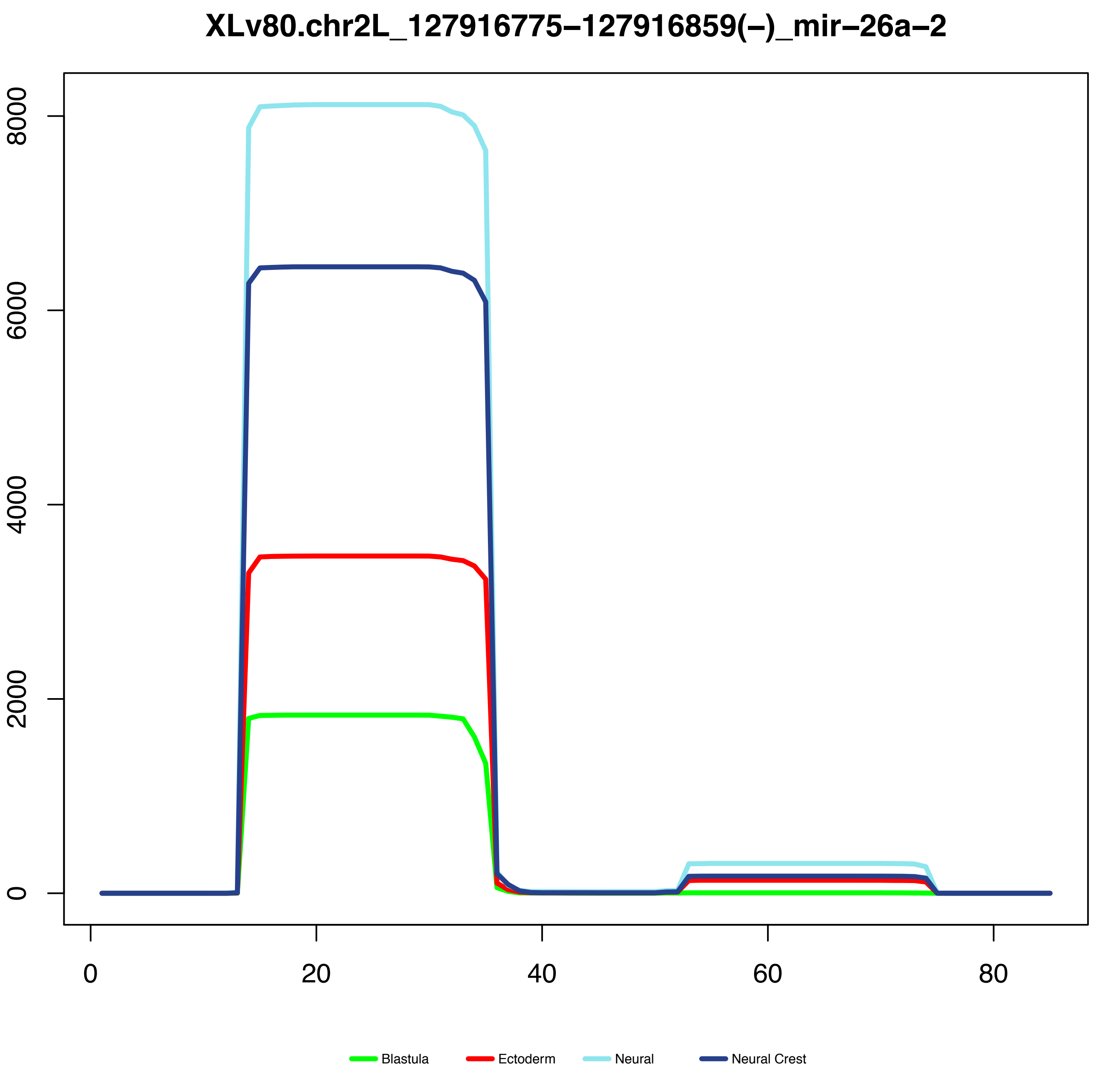
XLv80.chr2L_127916775−127916859(−)_mir−26a−2
0
2000
4000
6000
8000
0
20
40
60
80
Blastula
Ectoderm
Neural
Neural Crest

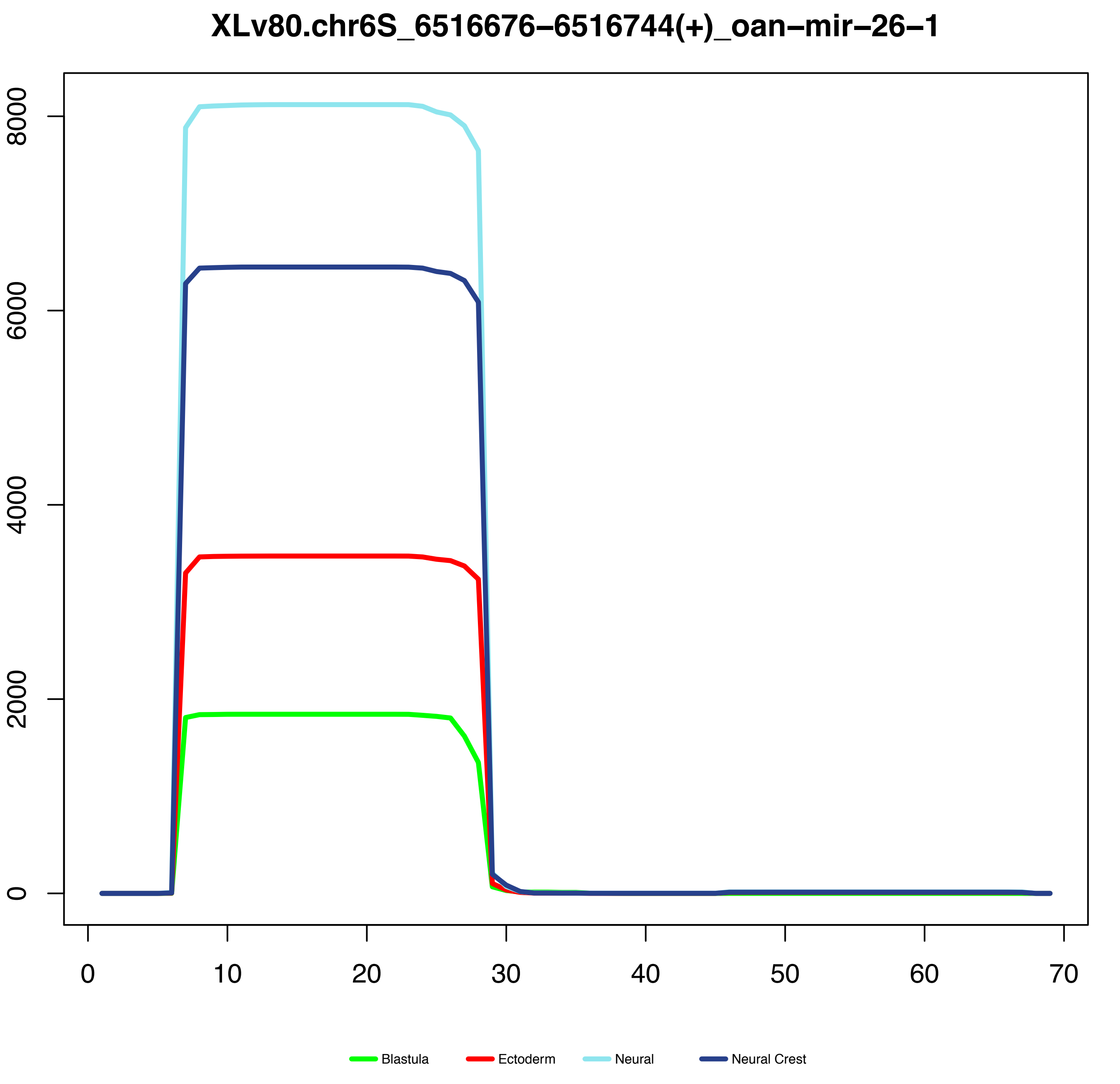
XLv80.chr6S_6516676–6516744(+)_oan–mir–26–1
0
2000
4000
6000
8000
0
10
20
30
40
50
60
70
Blastula
Ectoderm
Neural
Neural Crest

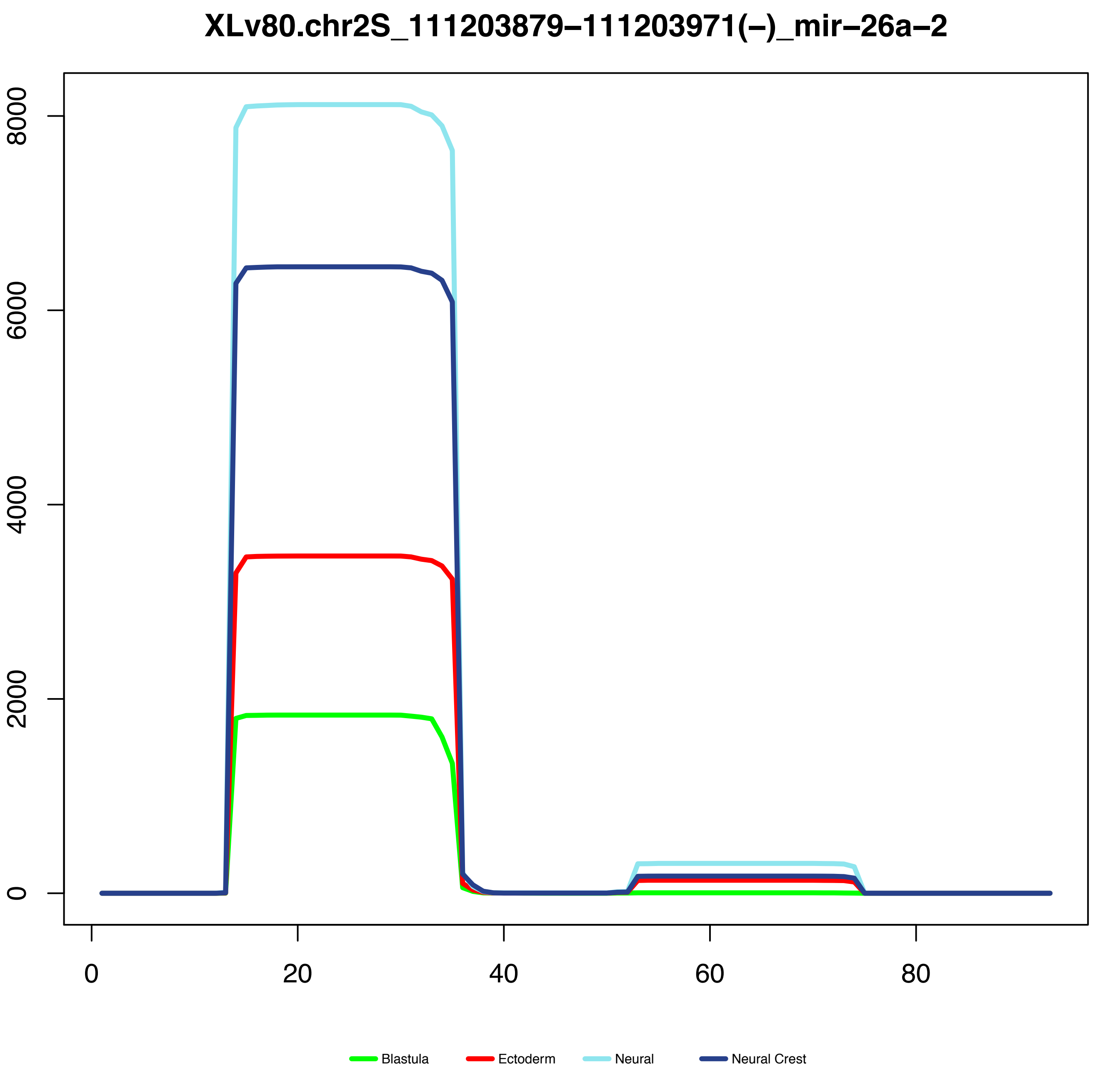

XLv80.chr2S_111203879−111203971(−)_mir−26a−2
0
2000
4000
6000
8000
0
20
40
60
80
Blastula
Ectoderm
Neural
Neural Crest

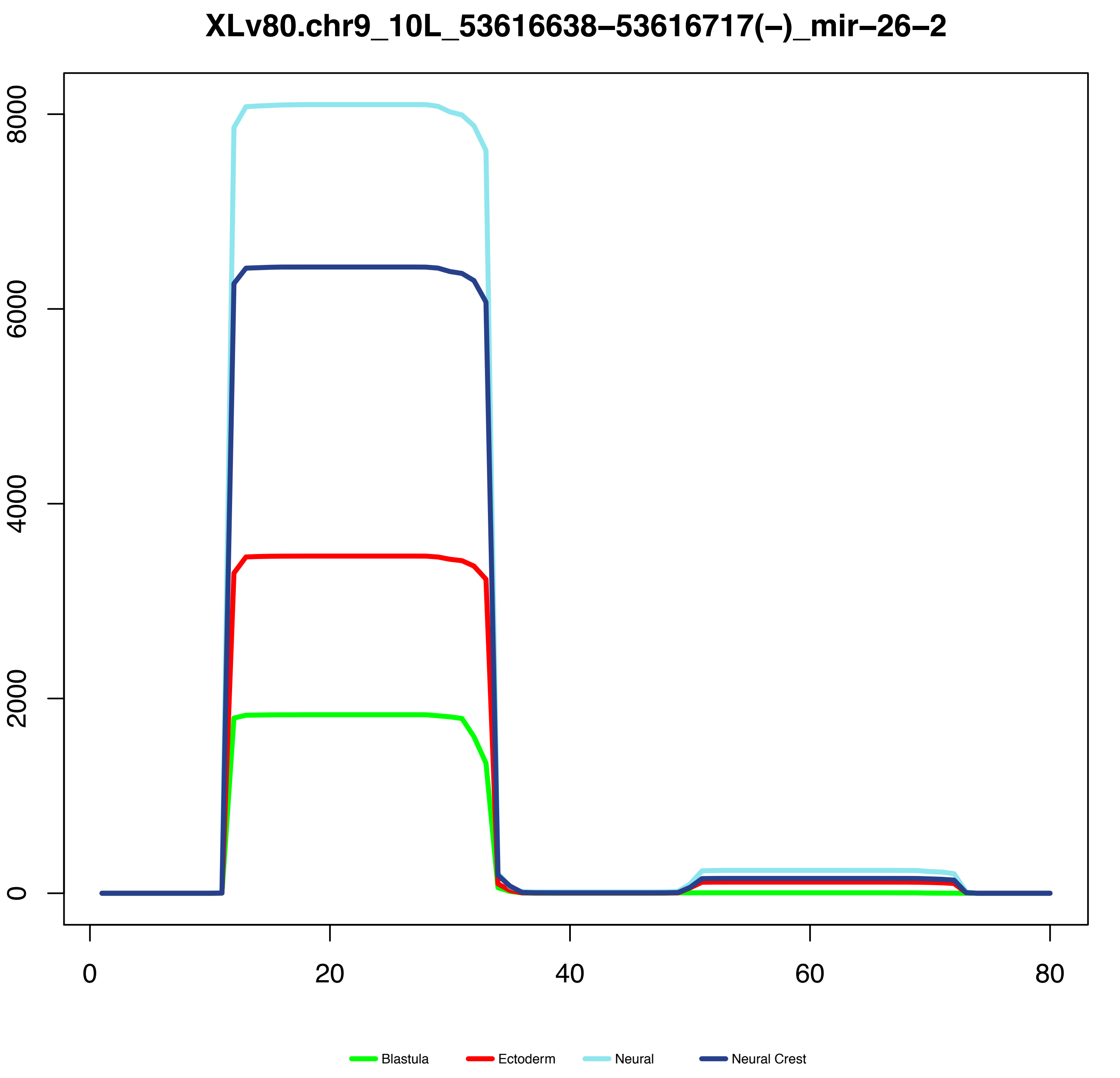

XLv80.chr9_10L_53616638−53616717(−)_mir−26−2
0
2000
4000
6000
8000
0
20
40
60
80
Blastula
Ectoderm
Neural
Neural Crest

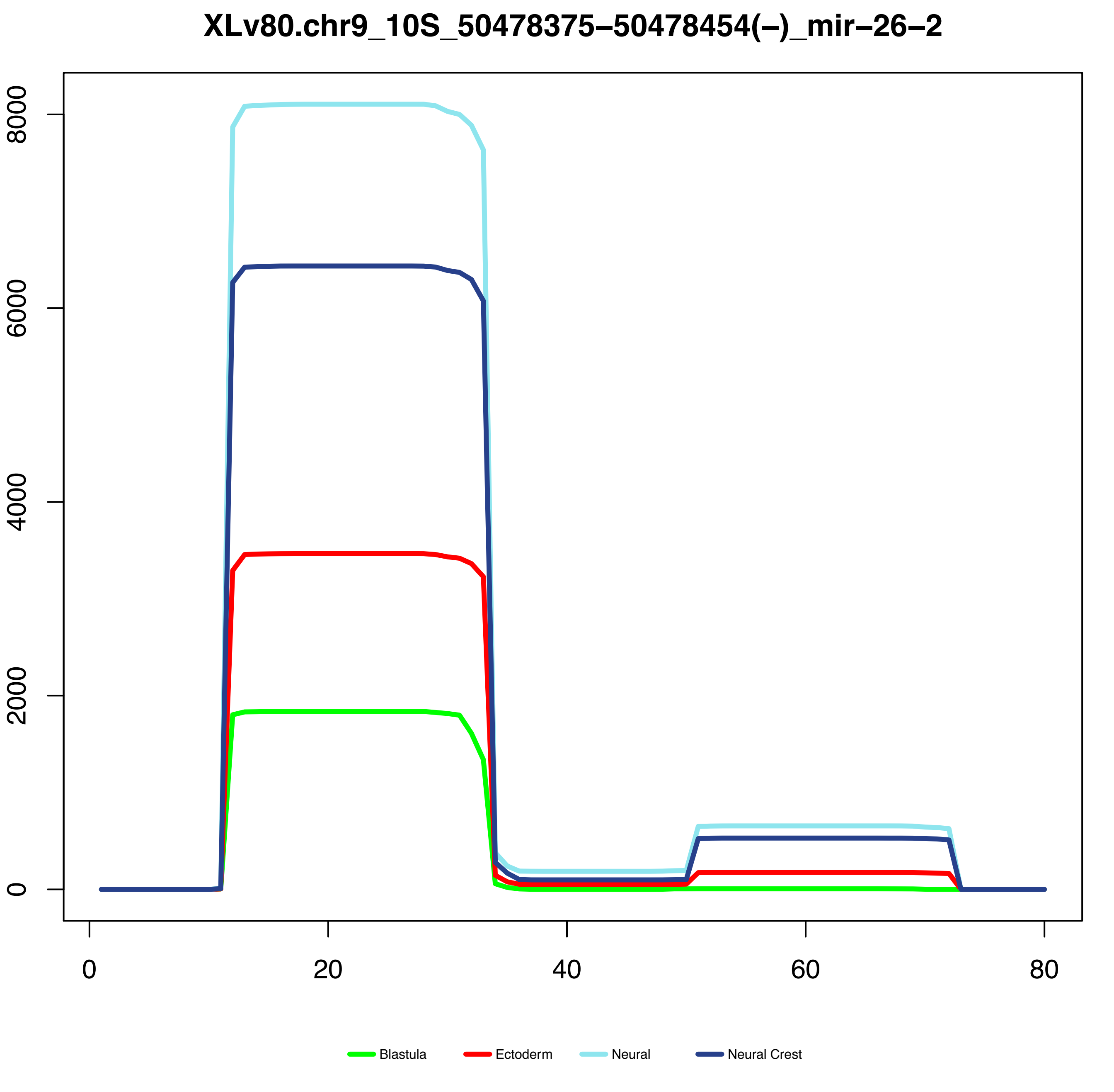
XLv80.chr9_10S_50478375−50478454(−)_mir−26−2
0
2000
4000
6000
8000
0
20
40
60
80
Blastula
Ectoderm
Neural
Neural Crest

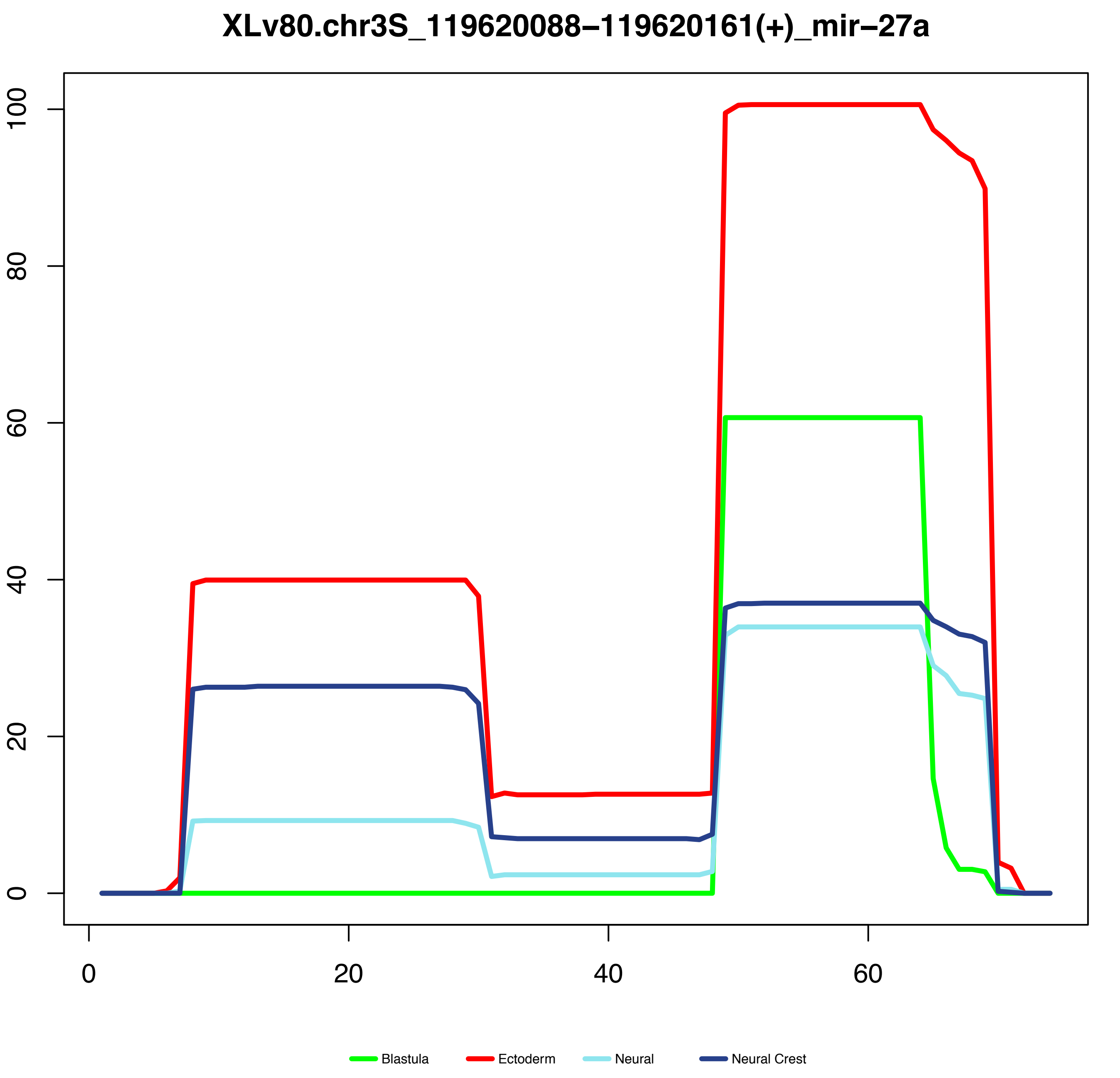
XLv80.chr3S_119620088−119620161(+)_mir−27a
0
20
40
60
80
100
0
20
40
60
Blastula
Ectoderm
Neural
Neural Crest

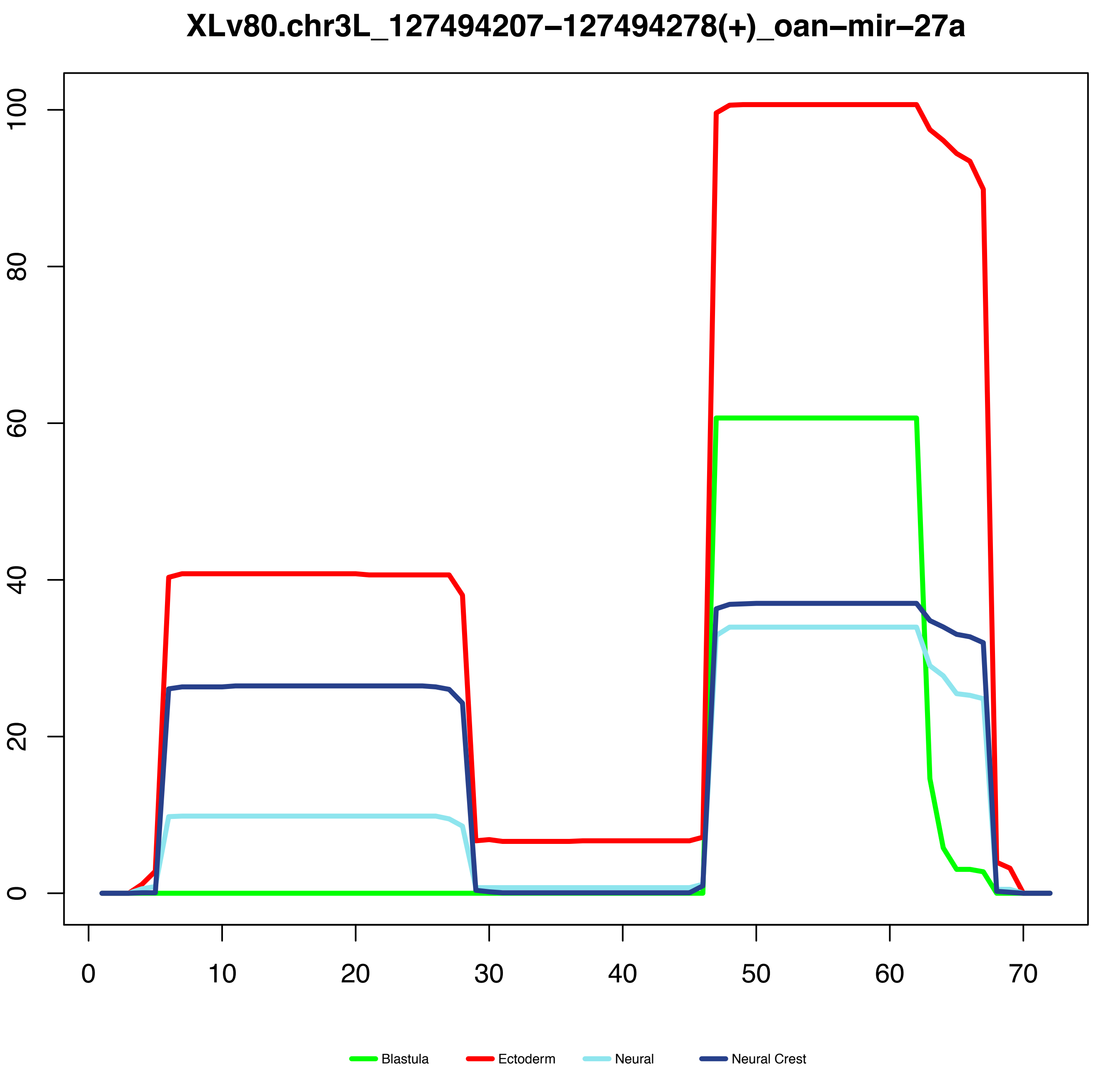
XLv80.chr3L_127494207−127494278(+)_oan−mir−27a
0
20
40
60
80
100
0
10
20
30
40
50
60
70
Blastula
Ectoderm
Neural
Neural Crest

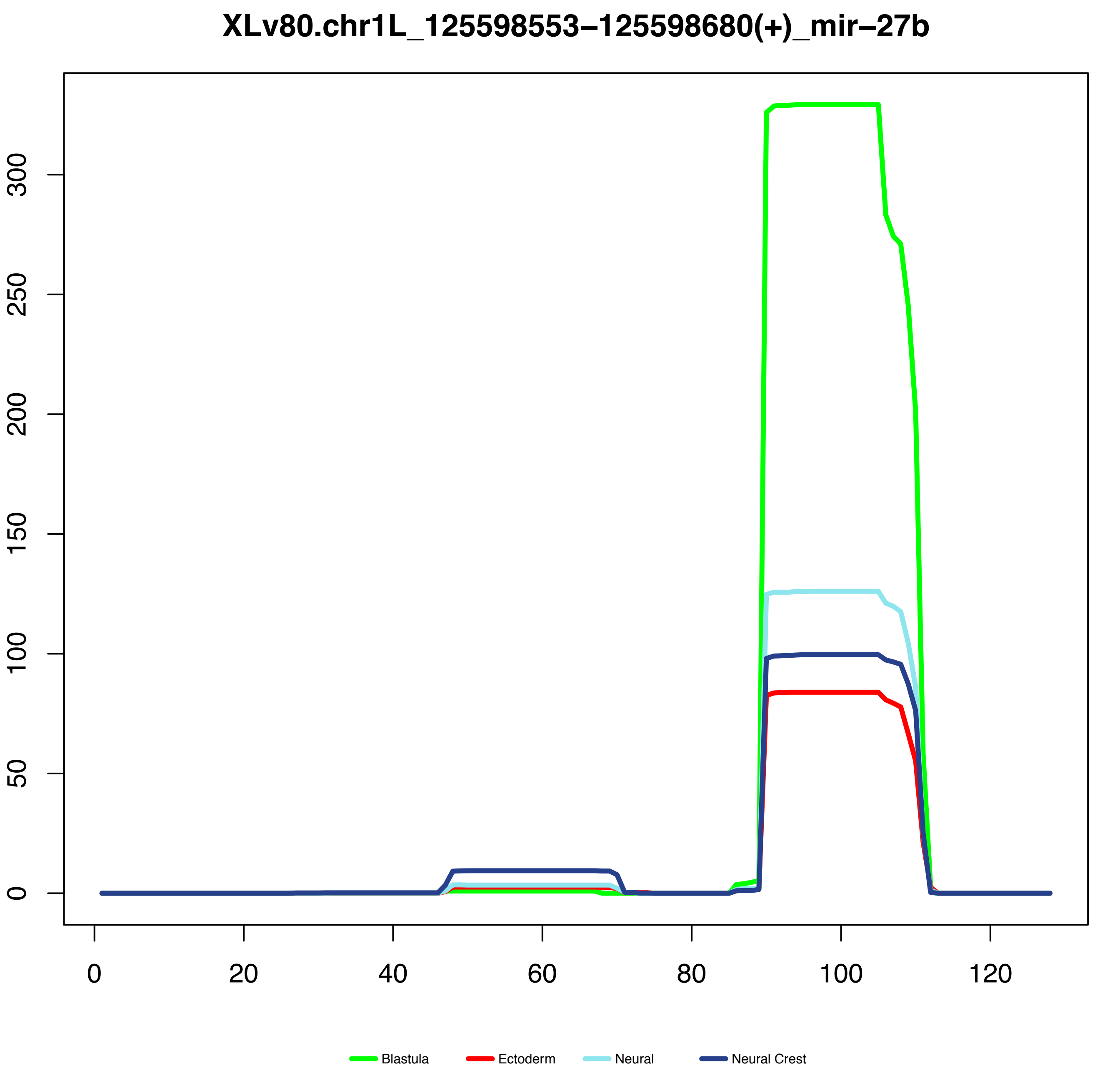

XLv80.chr1L_125598553−125598680(+)_mir−27b
0
50
100
150
200
250
300
0
20
40
60
80
100
120
Blastula
Ectoderm
Neural
Neural Crest

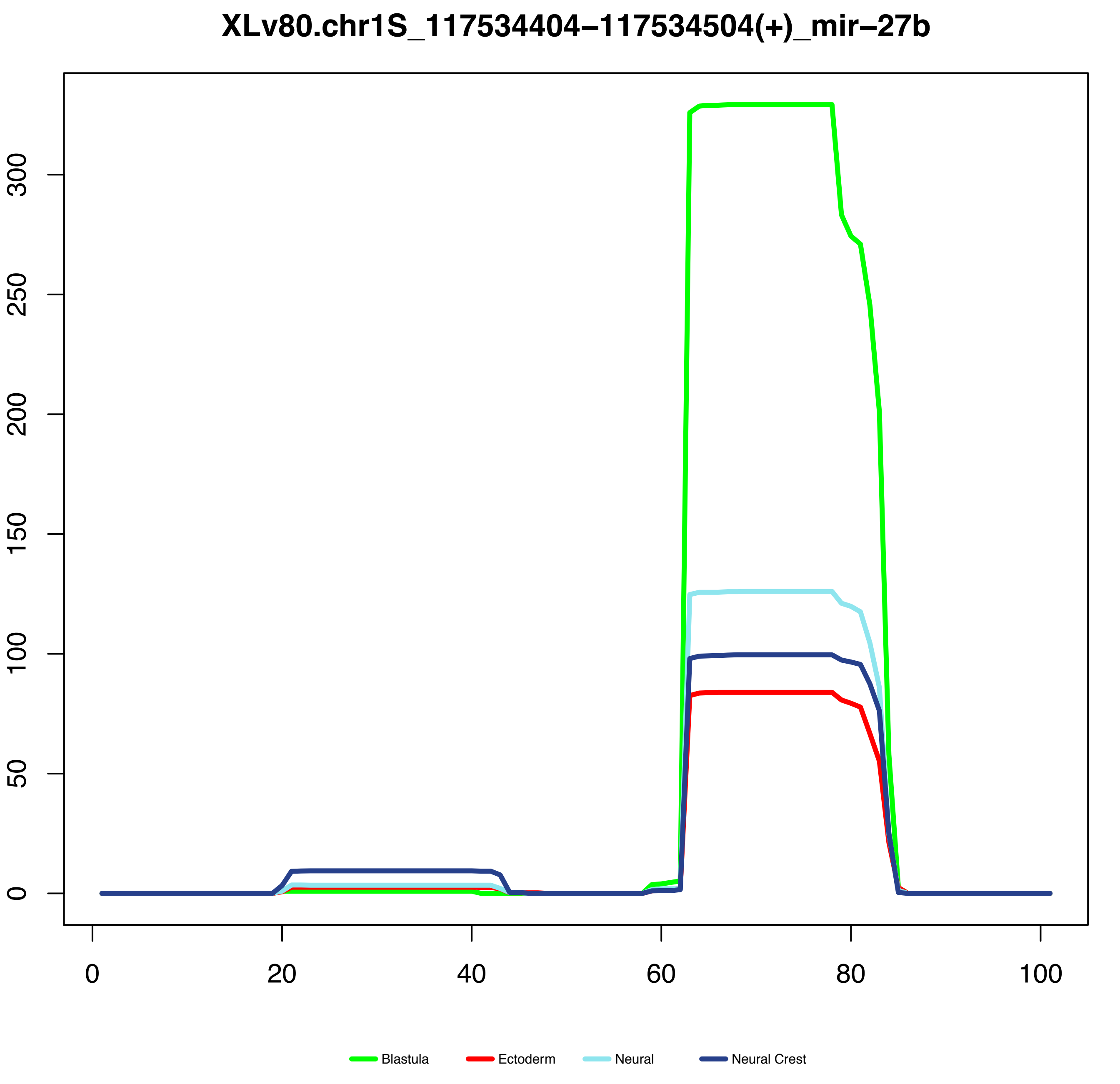

XLv80.chr1S_117534404−117534504(+)_mir−27b
0
50
100
150
200
250
300
0
20
40
60
80
100
Blastula
Ectoderm
Neural
Neural Crest

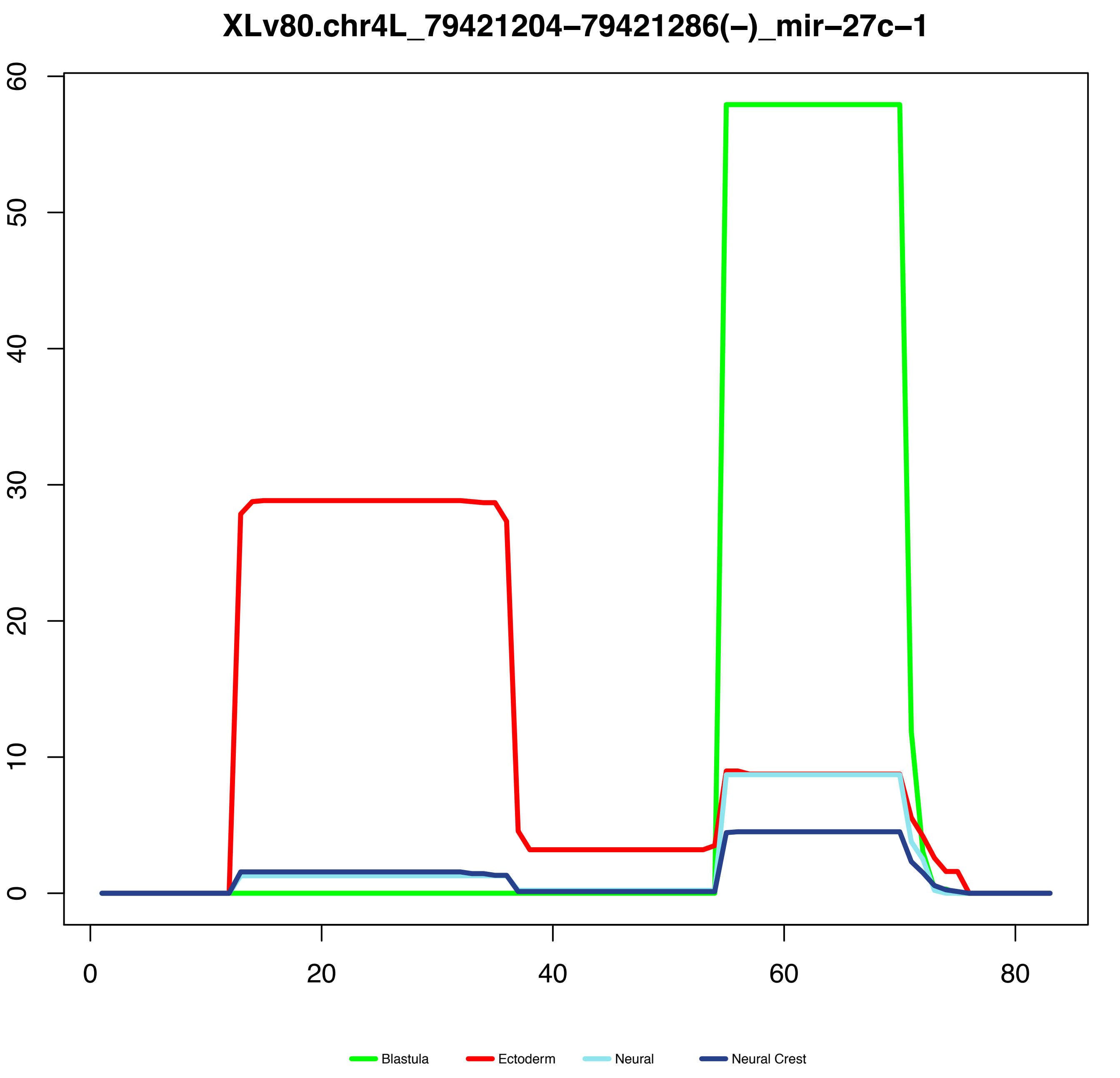
XLv80.chr4L_79421204−79421286(−)_mir−27c−1
0
10
20
30
40
50
60
0
20
40
60
80
Blastula
Ectoderm
Neural
Neural Crest

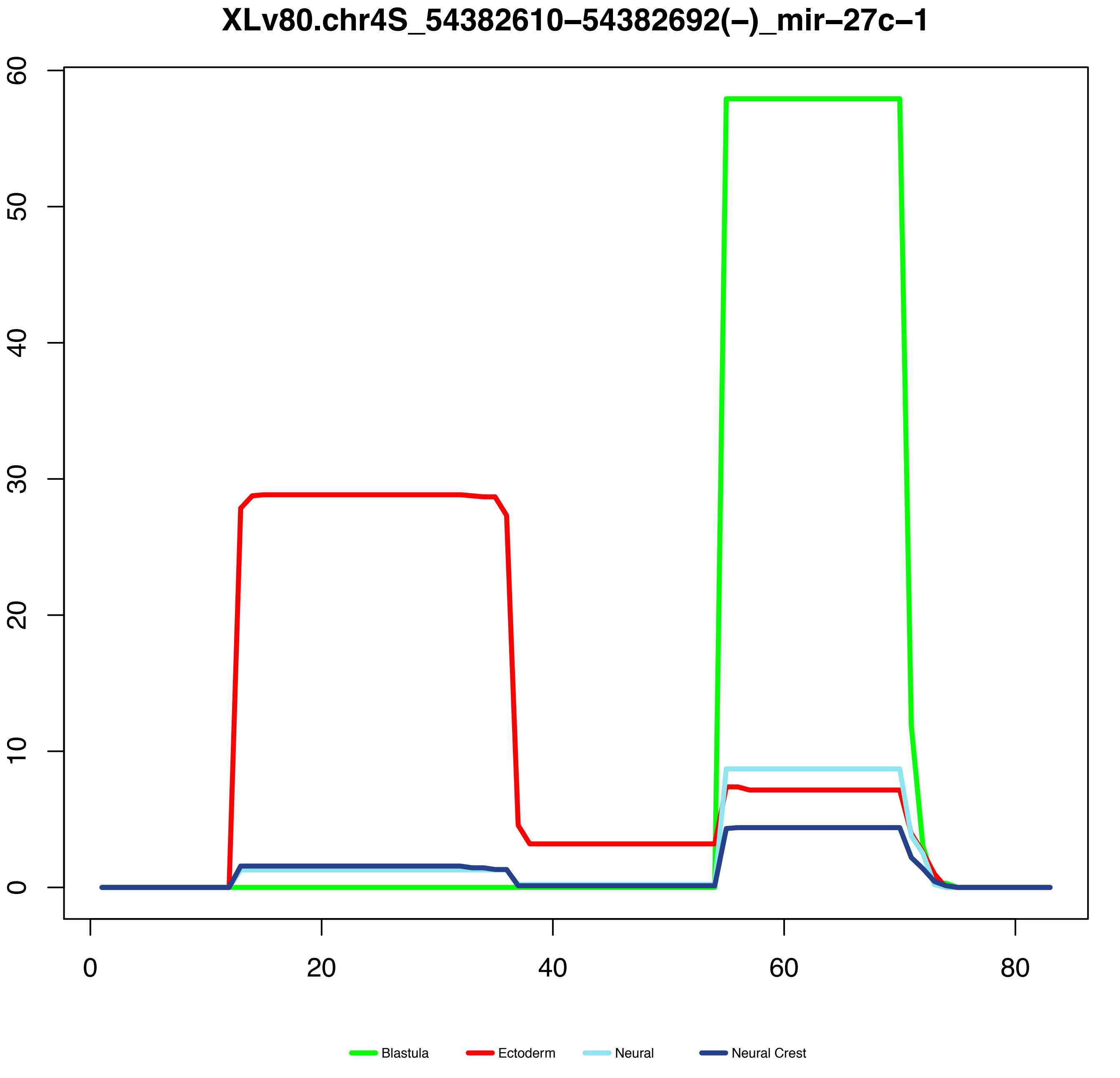
XLv80.chr4S_54382610−54382692(−)_mir−27c−1
0
10
20
30
40
50
60
0
20
40
60
80
Blastula
Ectoderm
Neural
Neural Crest

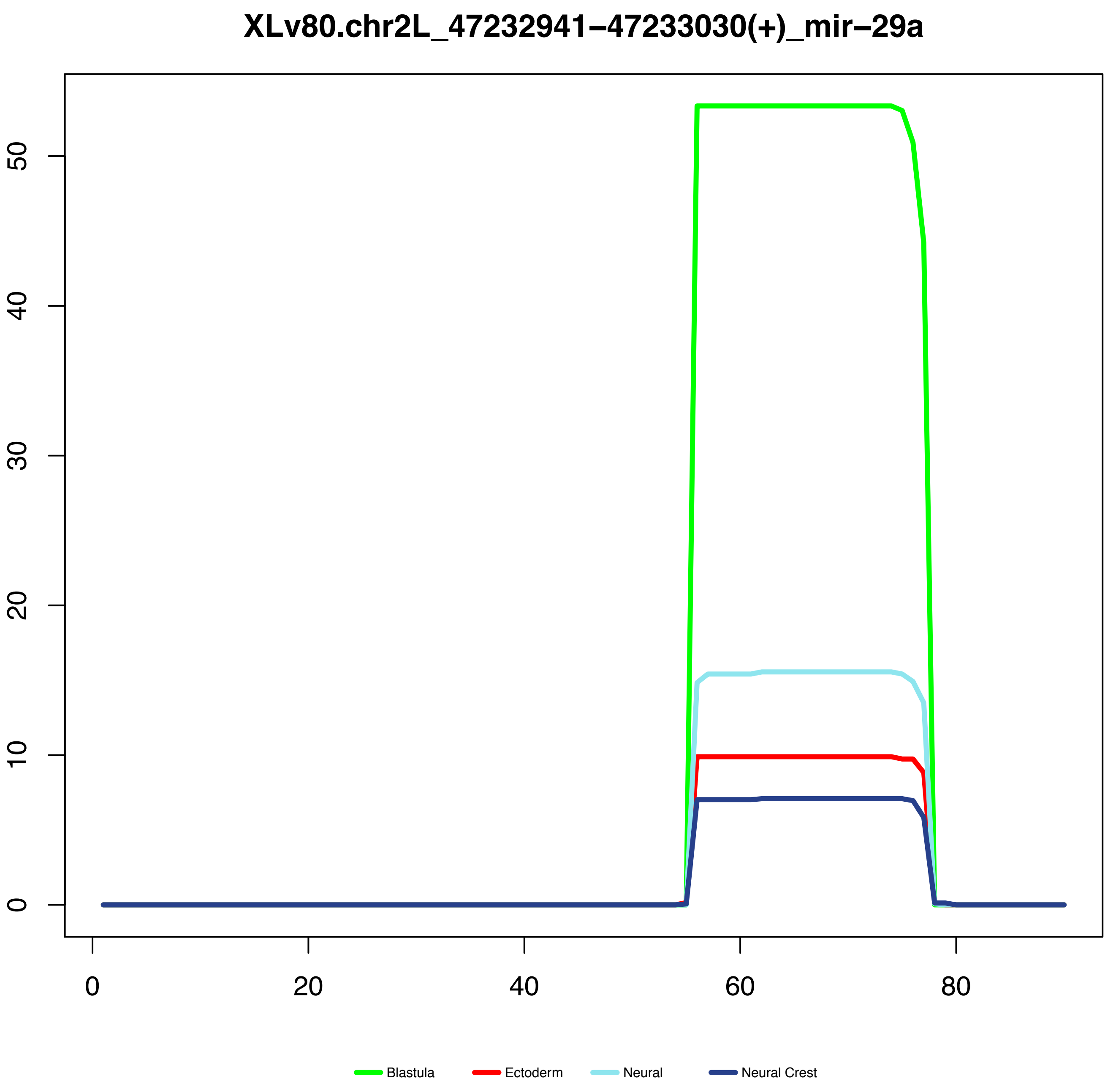

XLv80.chr2L_47232941−47233030(+)_mir−29a
0
10
20
30
40
50
0
20
40
60
80
Blastula
Ectoderm
Neural
Neural Crest

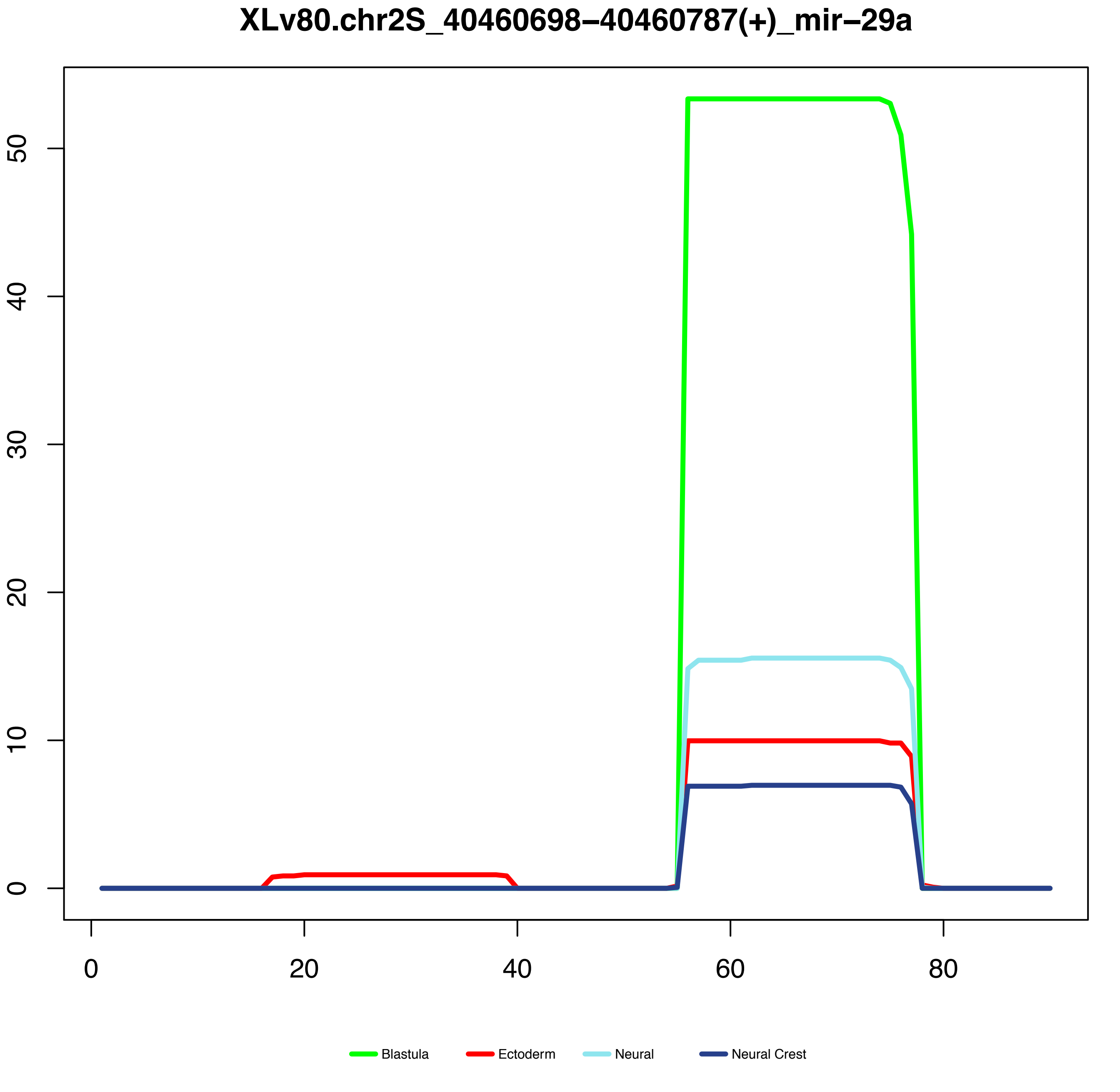
XLv80.chr2S_40460698−40460787(+)_mir−29a
0
10
20
30
40
50
0
20
40
60
80
Blastula
Ectoderm
Neural
Neural Crest

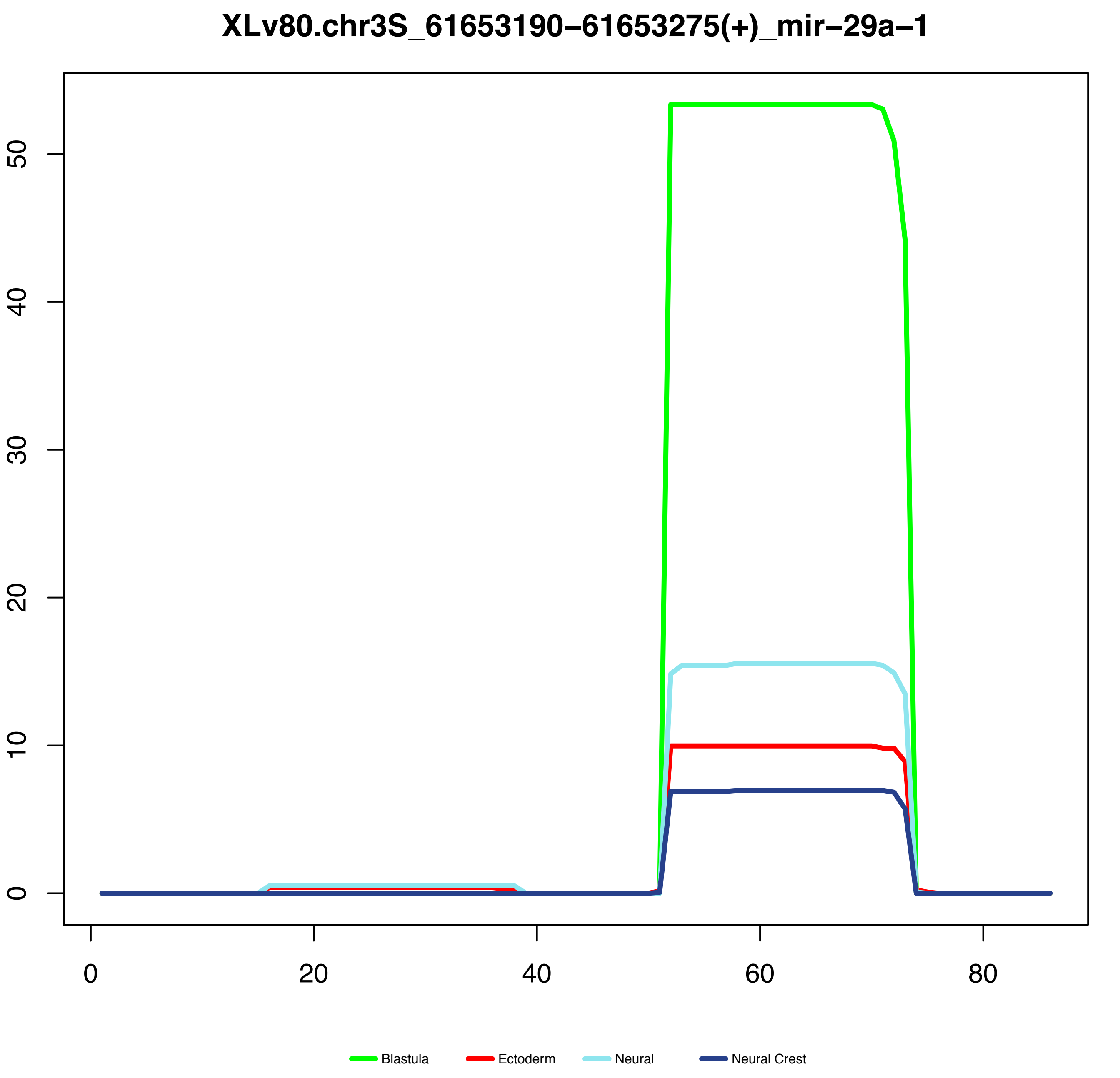
XLv80.chr3S_61653190−61653275(+)_mir−29a−1
0
10
20
30
40
50
0
20
40
60
80
Blastula
Ectoderm
Neural
Neural Crest

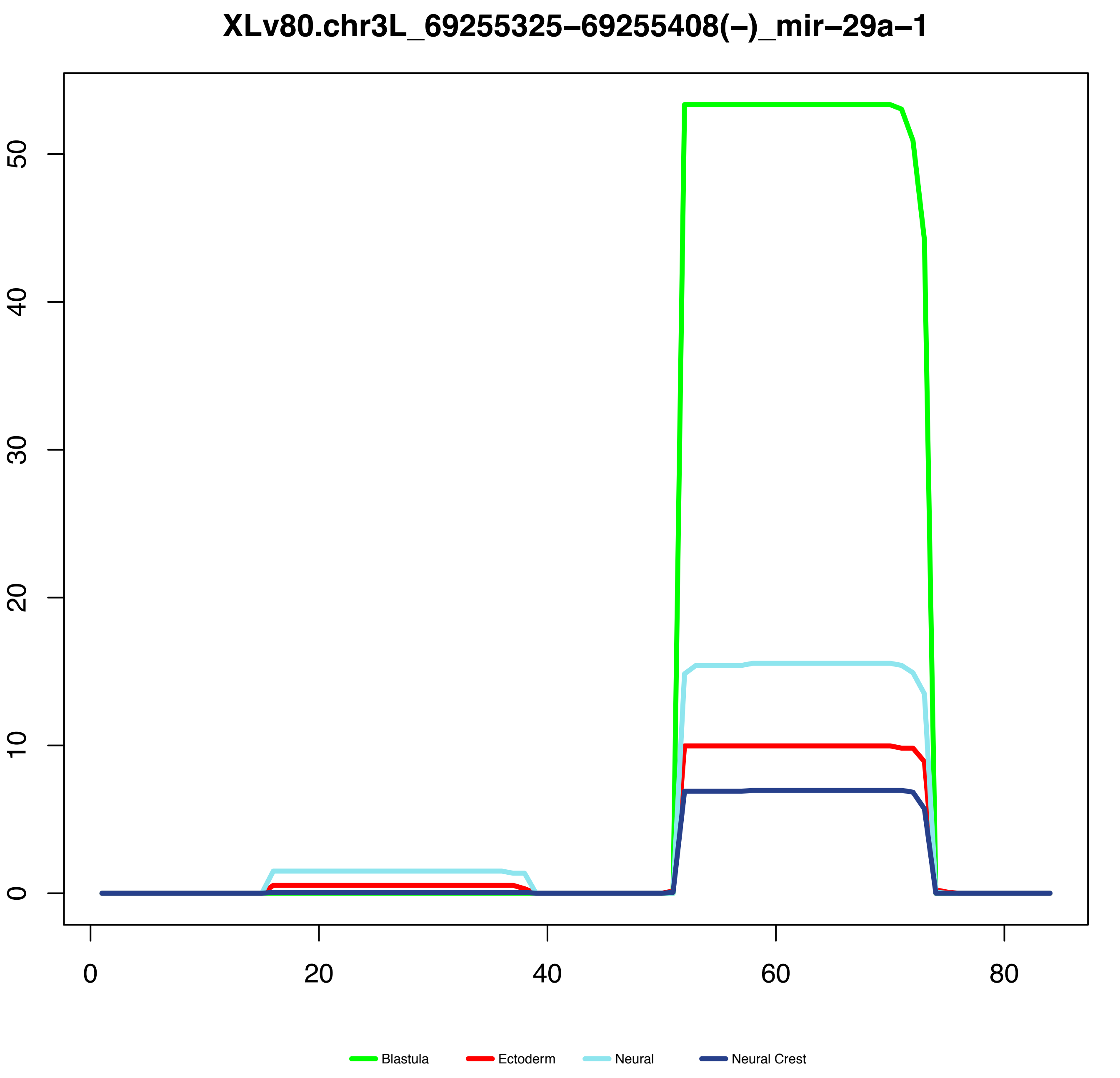
XLv80.chr3L_69255325−69255408(−)_mir−29a−1
0
10
20
30
40
50
0
20
40
60
80
Blastula
Ectoderm
Neural
Neural Crest

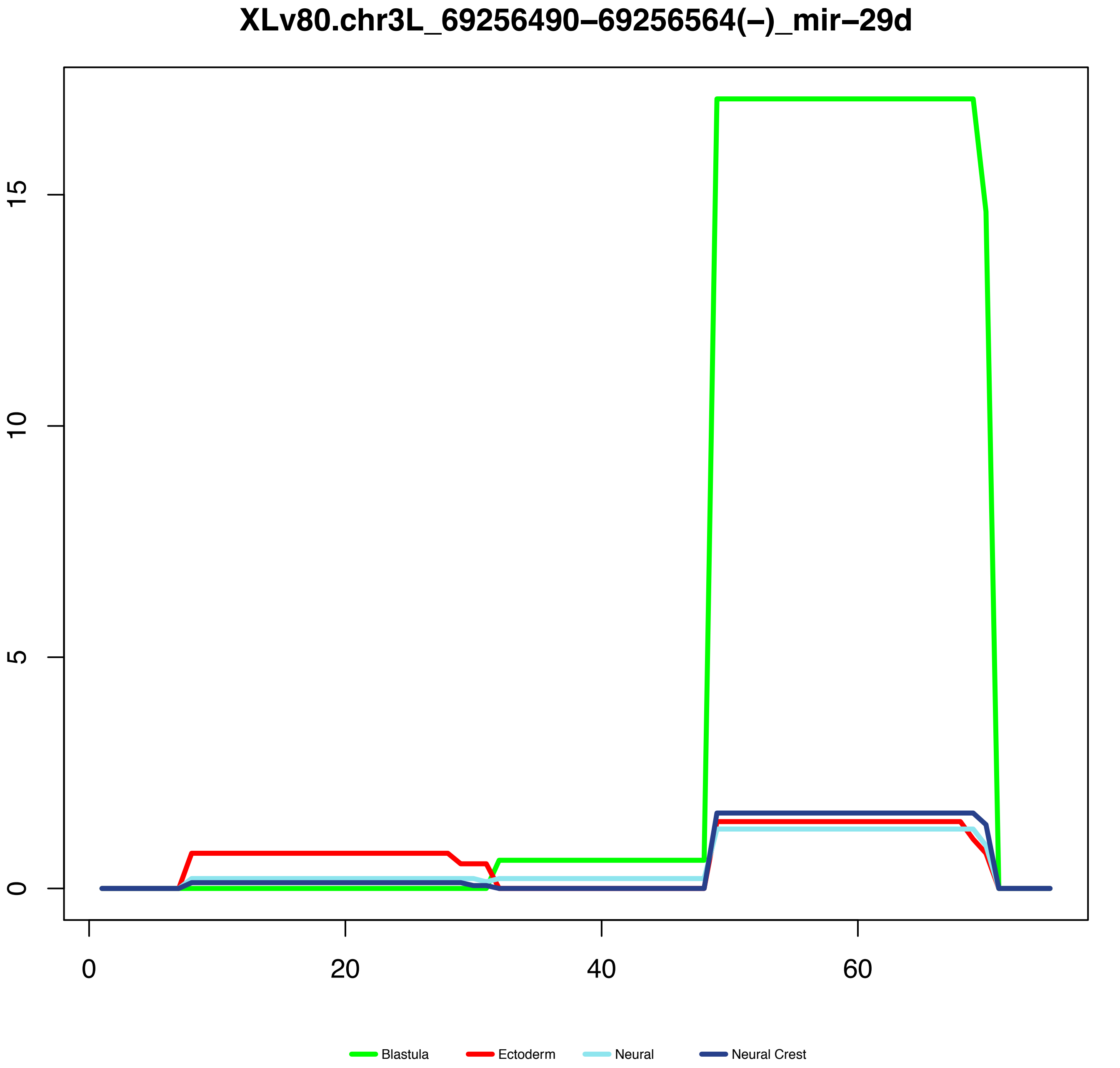

XLv80.chr3L_69256490−69256564(−)_mir−29d
0
5
10
15
0
20
40
60
Blastula
Ectoderm
Neural
Neural Crest

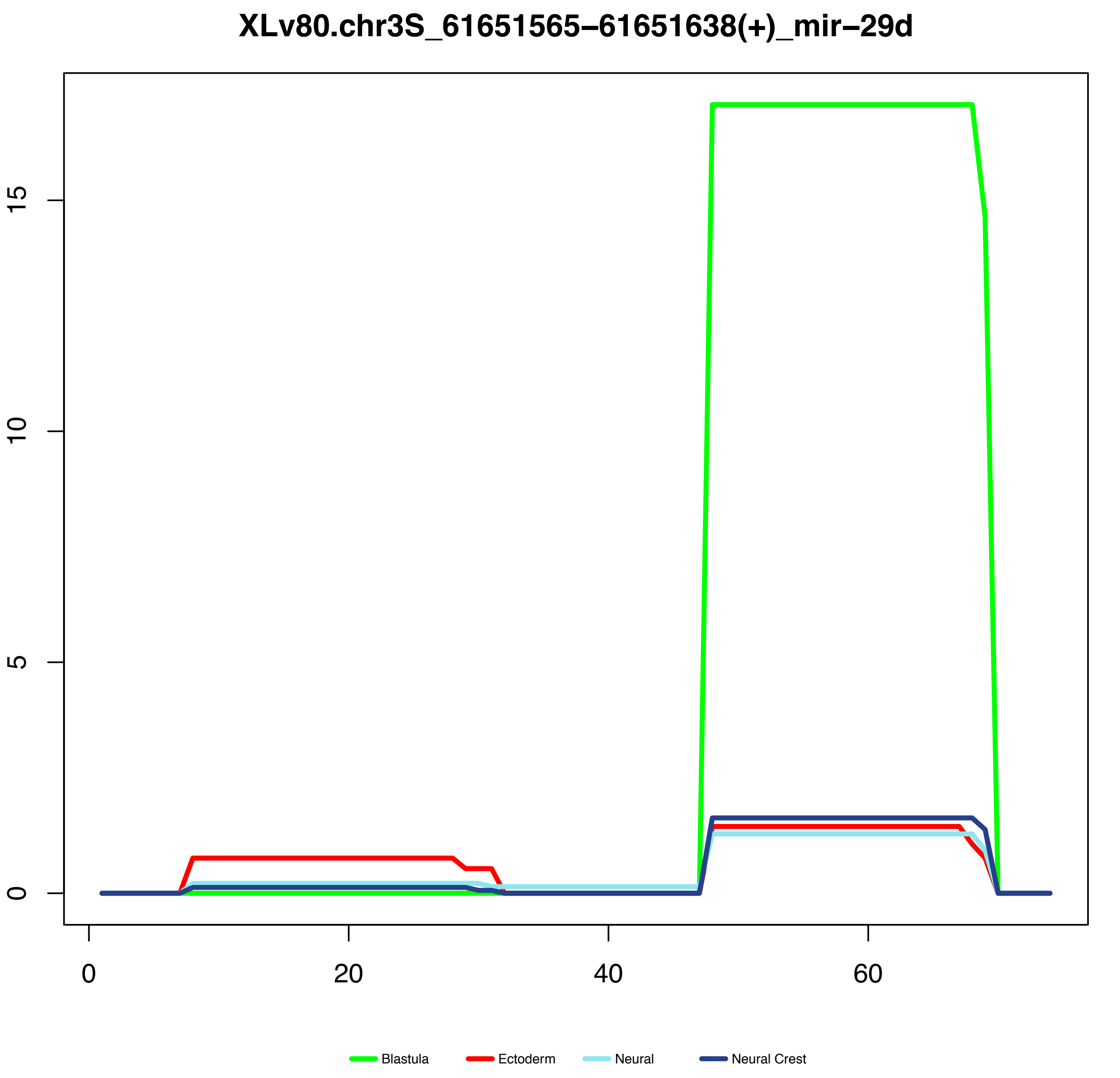

XLv80.chr3S_61651565−61651638(+)_mir−29d
0
5
10
15
0
20
40
60
Blastula
Ectoderm
Neural
Neural Crest

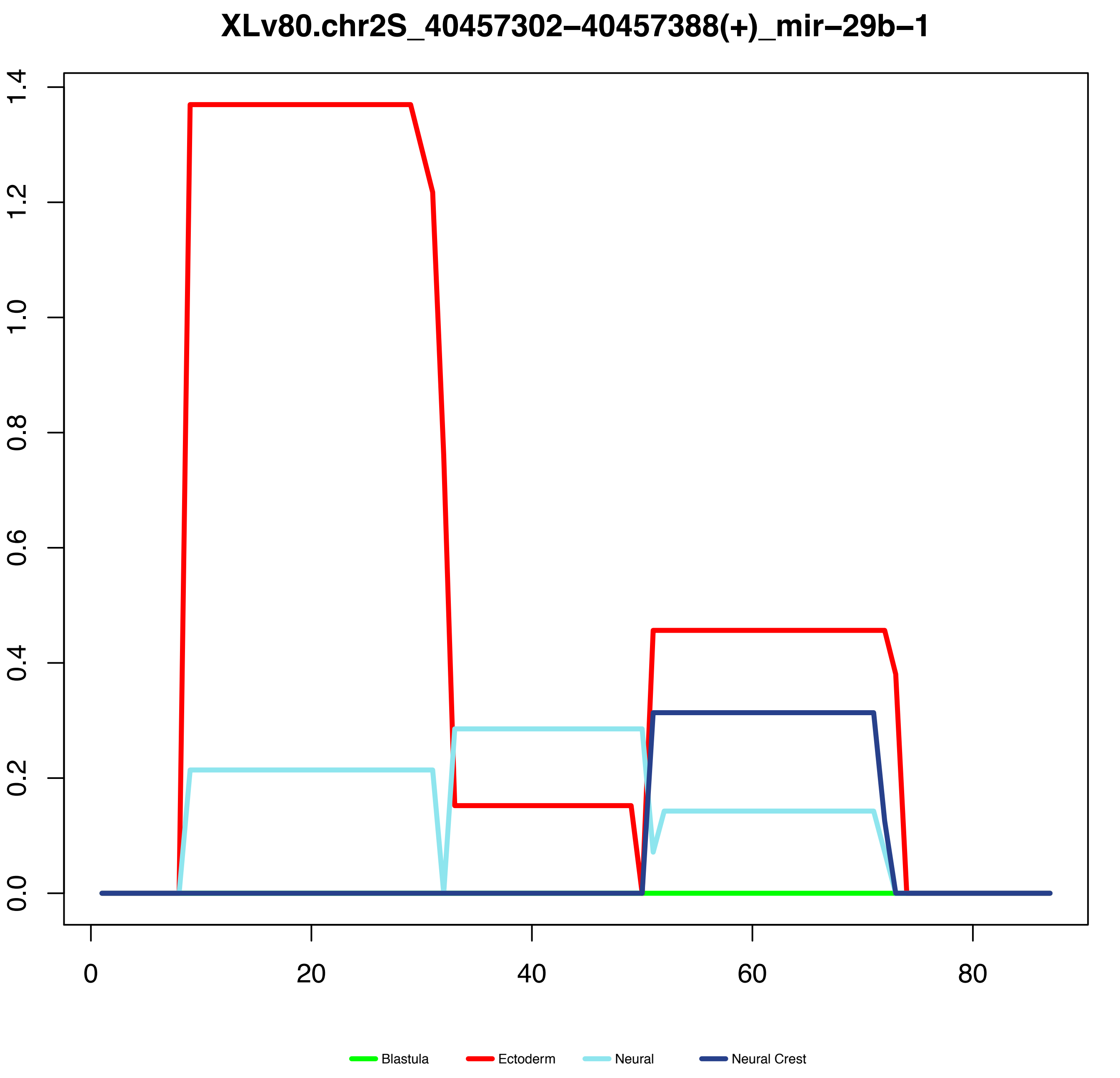
XLv80.chr2S_40457302−40457388(+)_mir−29b−1
0.0
0.2
0.4
0.6
0.8
1.0
1.2
1.4
0
20
40
60
80
Blastula
Ectoderm
Neural
Neural Crest

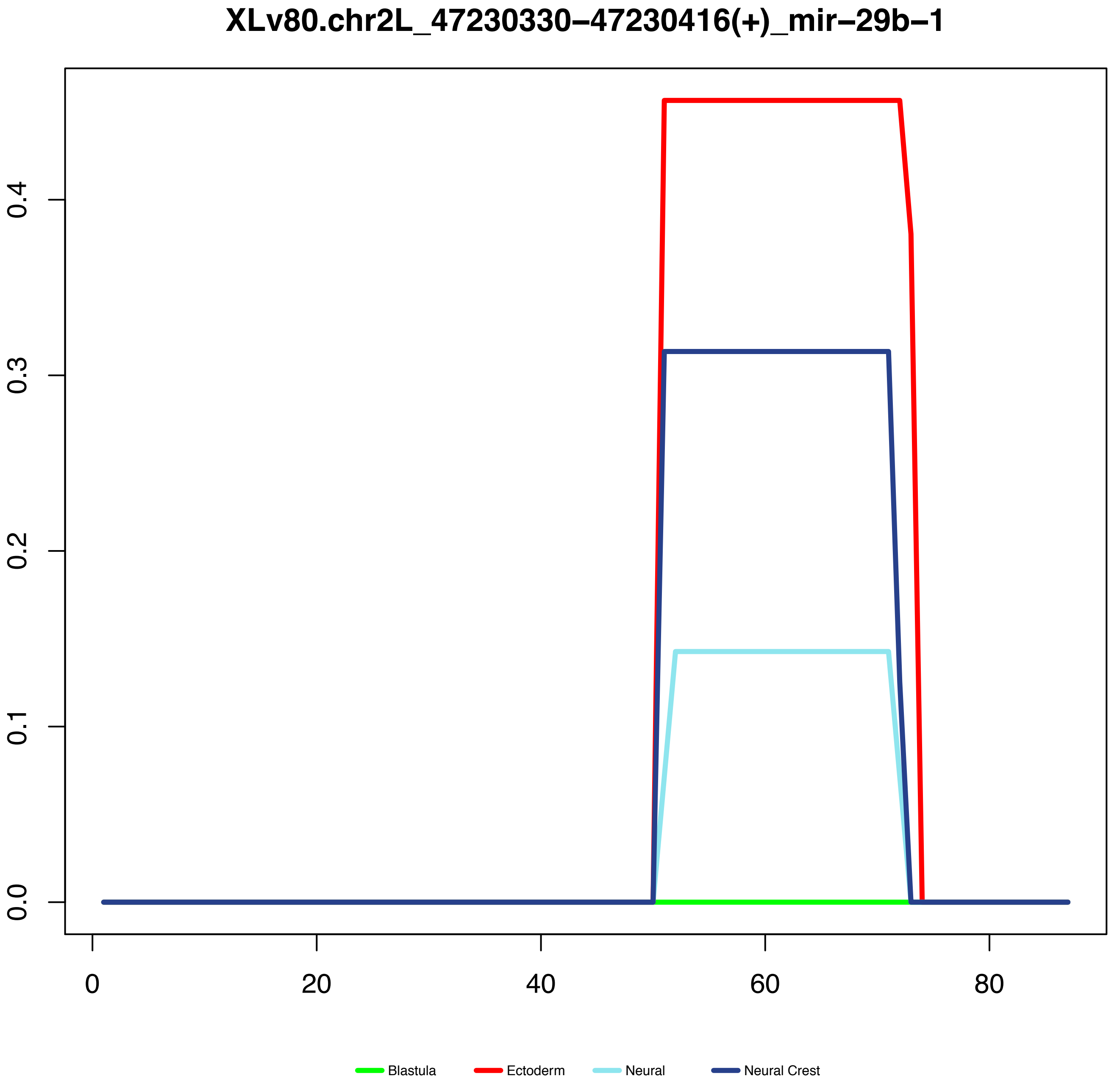
XLv80.chr2L_47230330−47230416(+)_mir−29b−1
0.0
0.1
0.2
0.3
0.4
0
20
40
60
80
Blastula
Ectoderm
Neural
Neural Crest

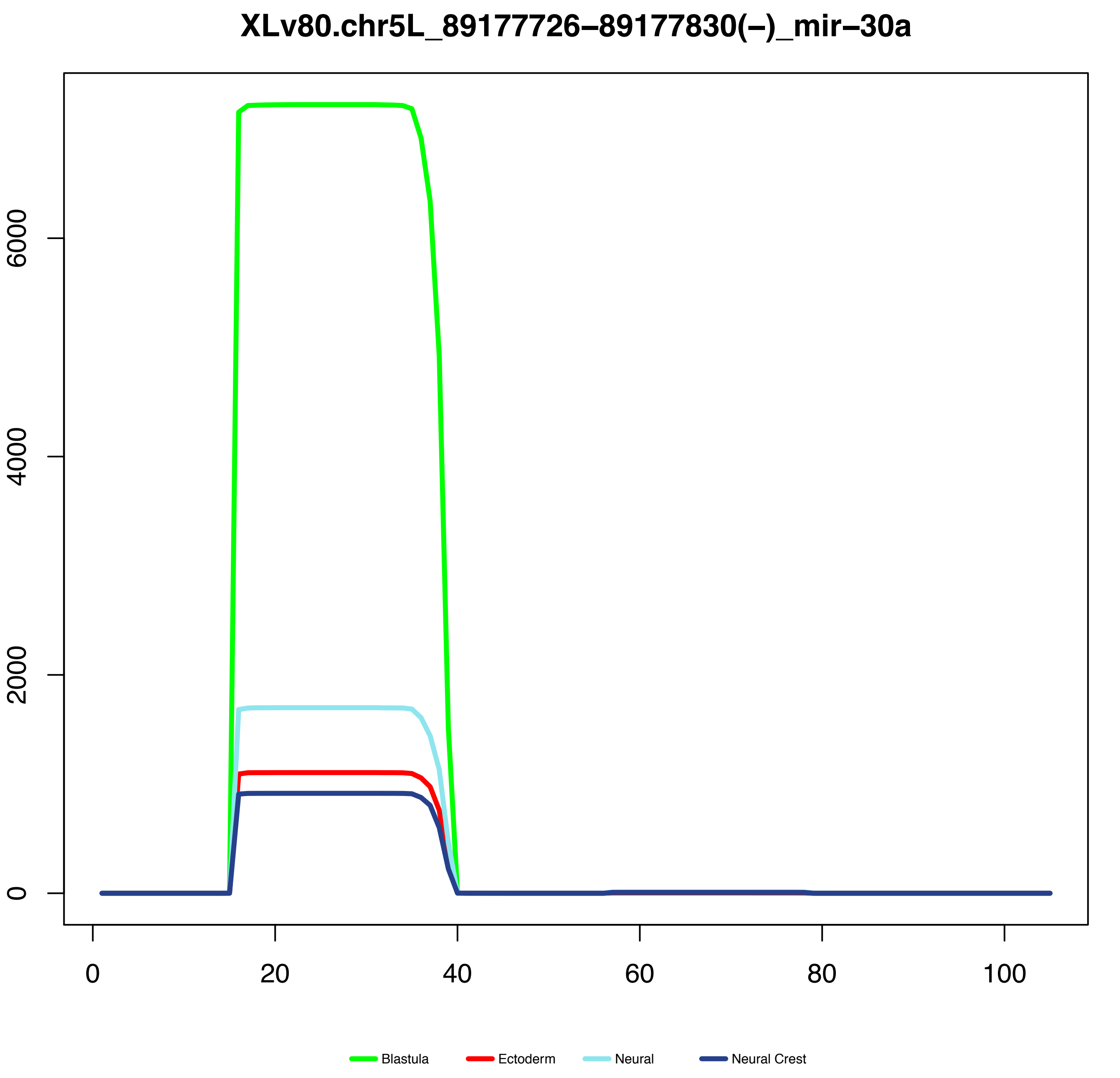
XLv80.chr5L_89177726−89177830(−)_mir−30a
0
2000
4000
6000
0
20
40
60
80
100
Blastula
Ectoderm
Neural
Neural Crest

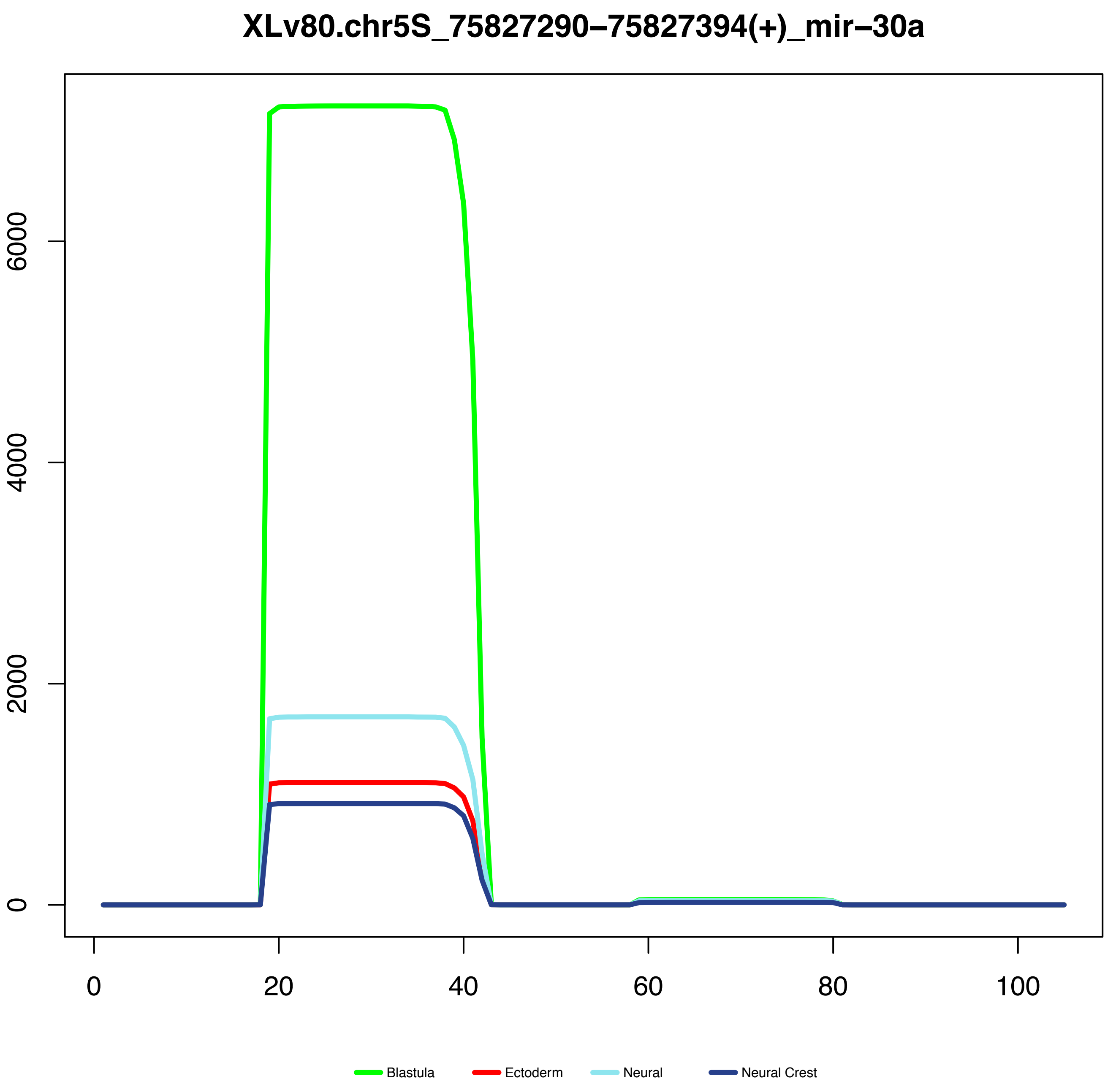

XLv80.chr5S_75827290−75827394(+)_mir−30a
0
2000
4000
6000
0
20
40
60
80
100
Blastula
Ectoderm
Neural
Neural Crest

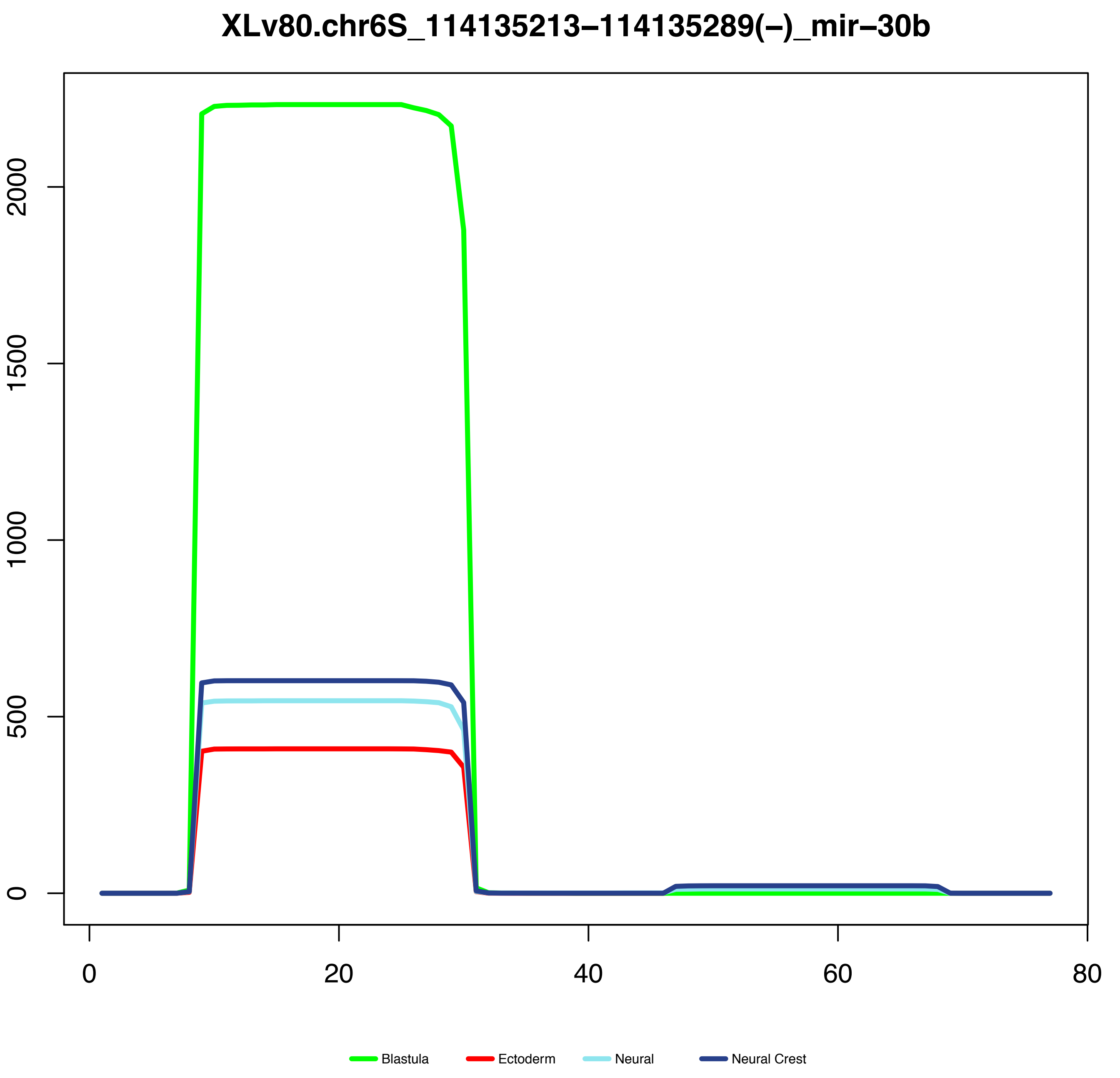

XLv80.chr6S_114135213−114135289(−)_mir−30b
0
500
1000
1500
2000
0
20
40
60
80
Blastula
Ectoderm
Neural
Neural Crest

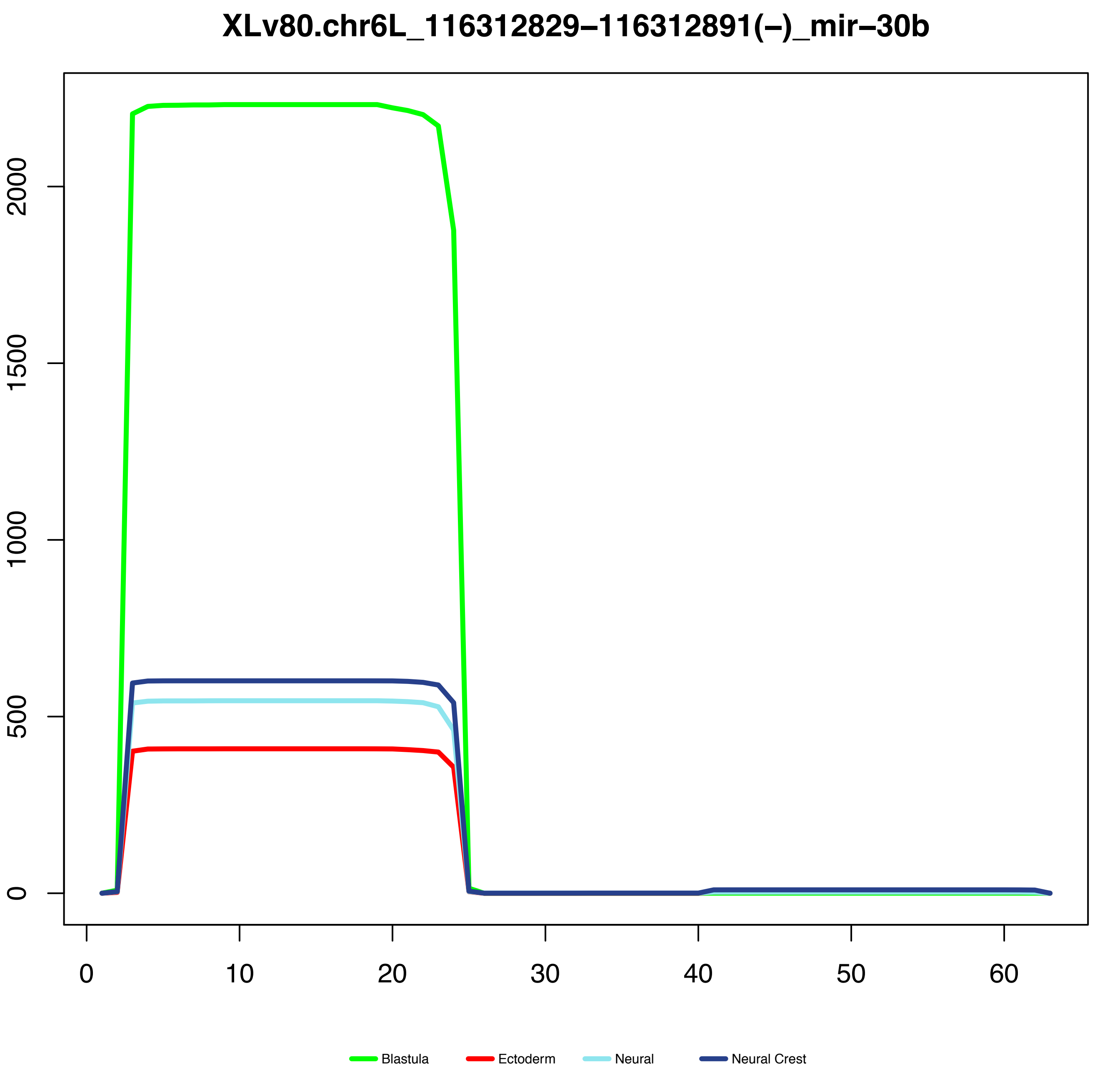
XLv80.chr6L_116312829−116312891(−)_mir−30b
0
500
1000
1500
2000
0
10
20
30
40
50
60
Blastula
Ectoderm
Neural
Neural Crest

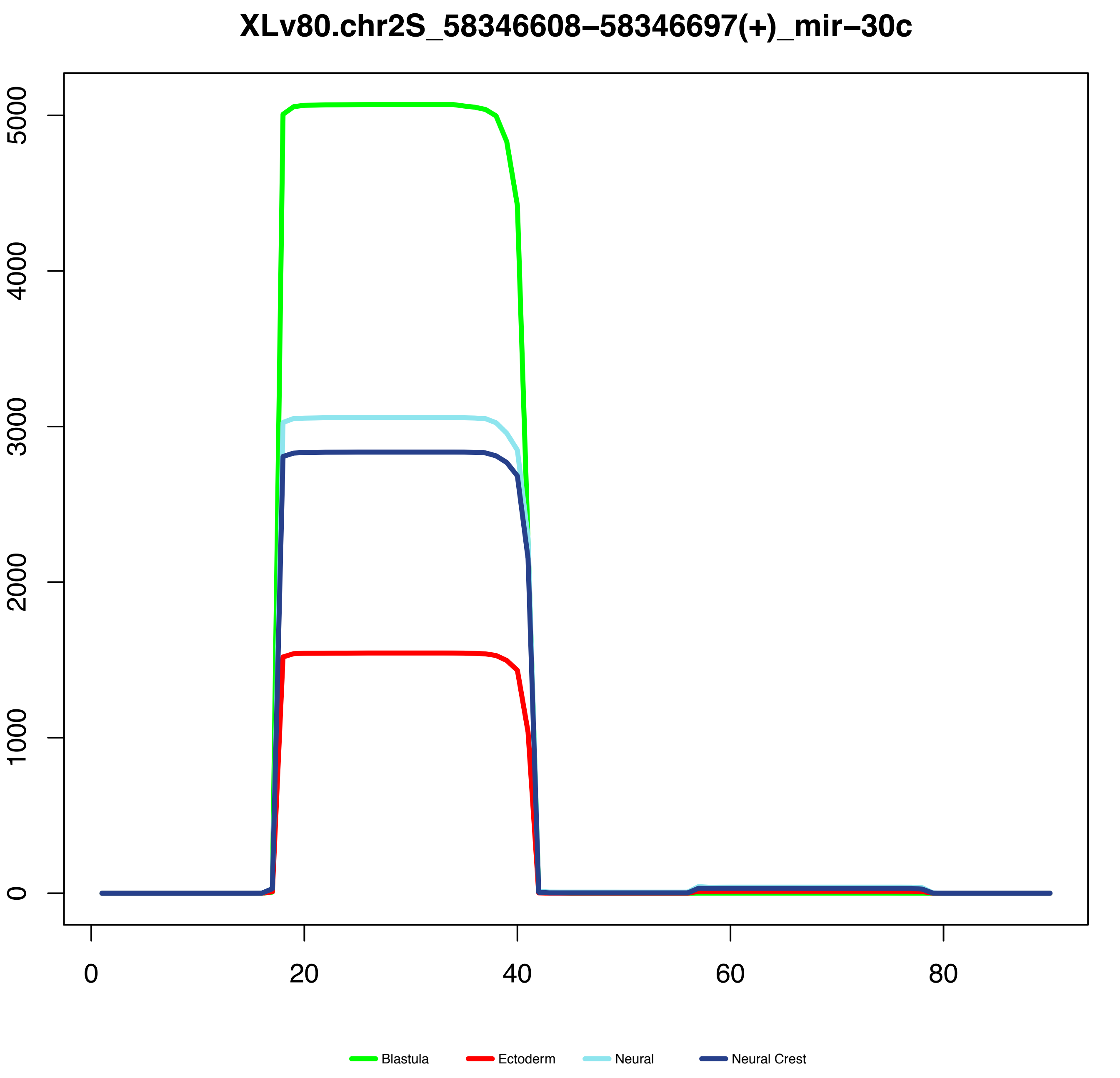

XLv80.chr2S_58346608−58346697(+)_mir−30c
0
1000
2000
3000
4000
5000
0
20
40
60
80
Blastula
Ectoderm
Neural
Neural Crest

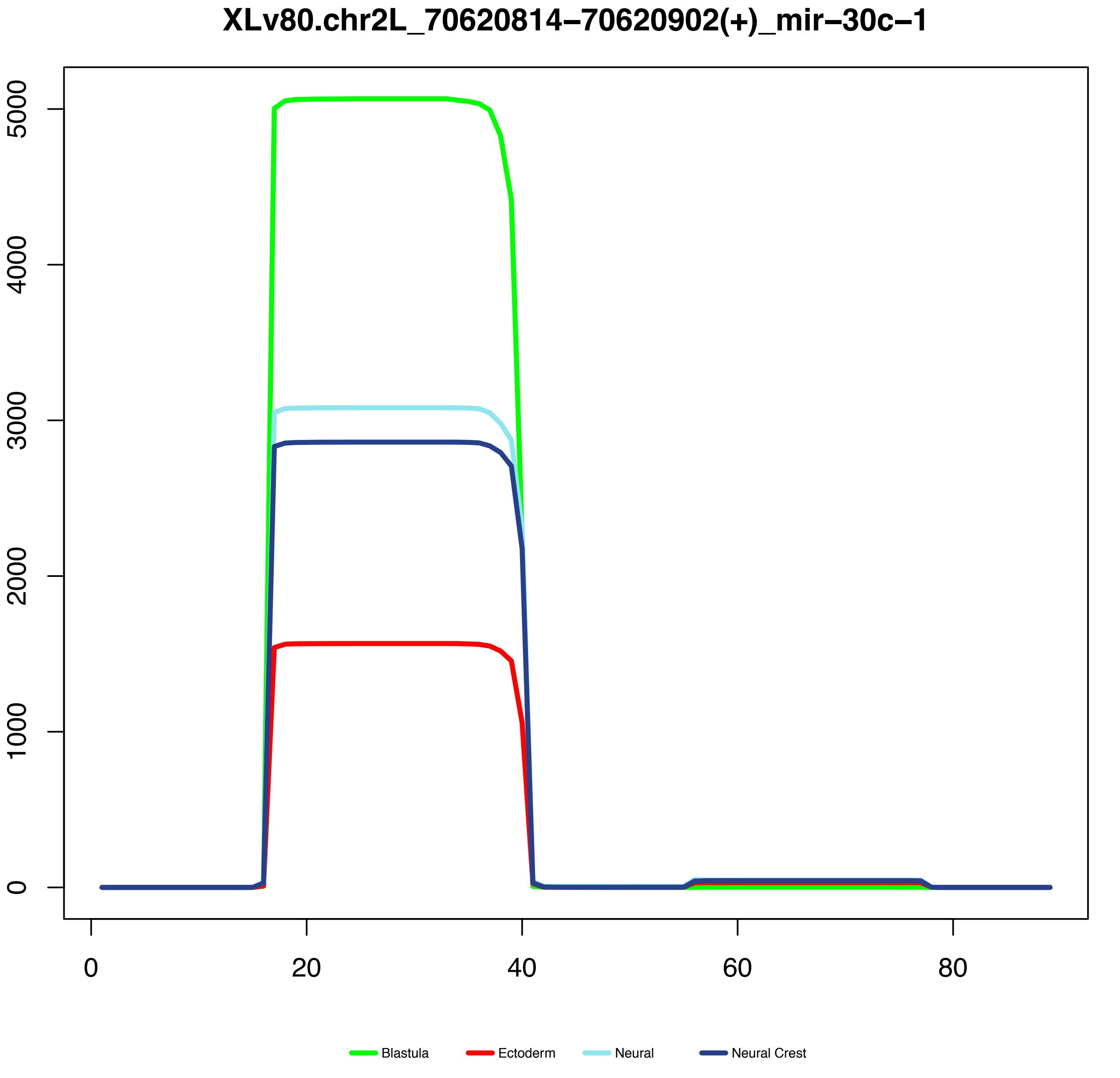

XLv80.chr2L_70620814−70620902(+)_mir−30c−1
0
1000
2000
3000
4000
5000
0
20
40
60
80
Blastula
Ectoderm
Neural
Neural Crest

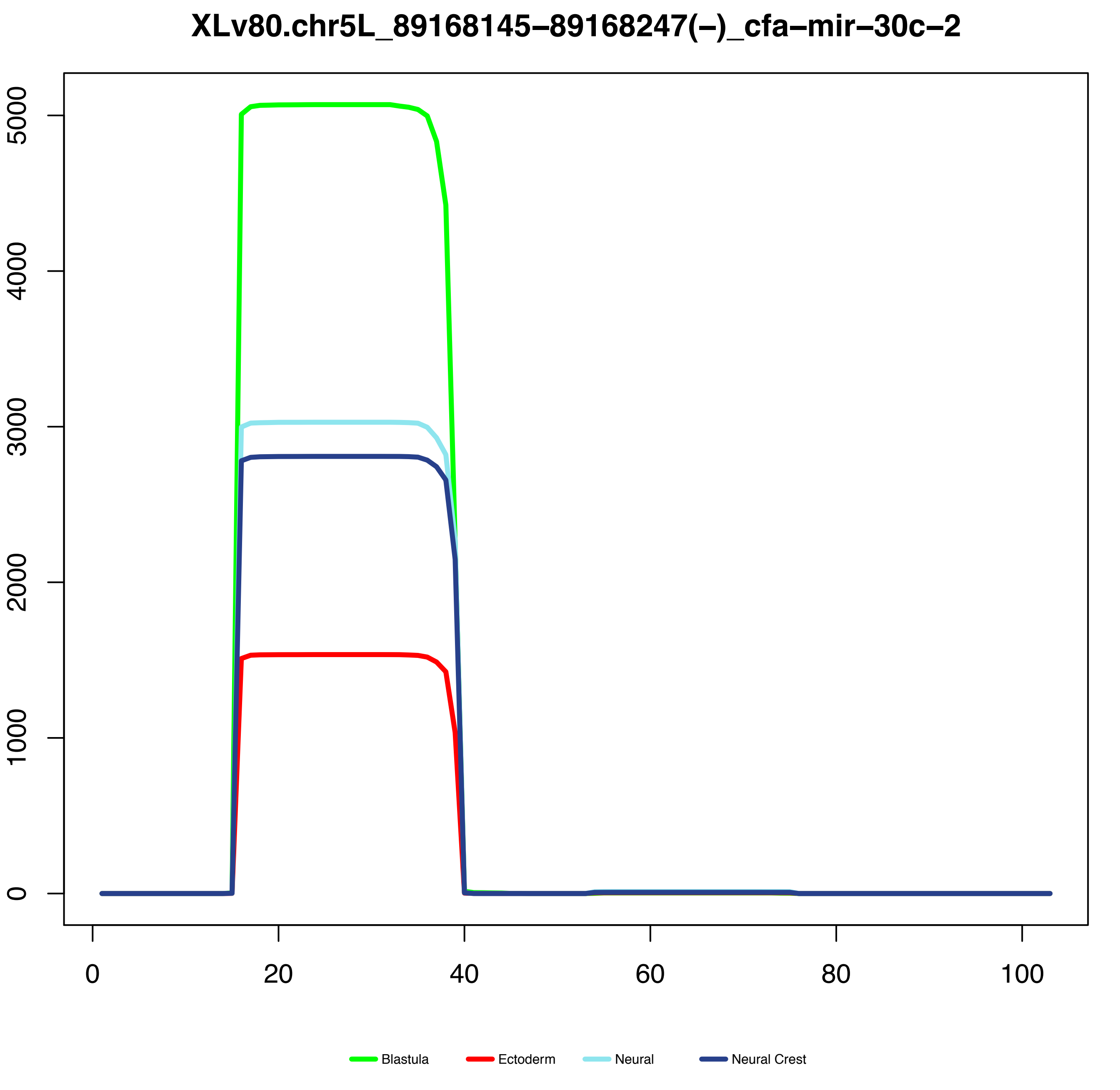
XLv80.chr5L_89168145–89168247(–)_cfa–mir–30c–2
0
1000
2000
3000
4000
5000
0
20
40
60
80
100
Blastula
Ectoderm
Neural
Neural Crest

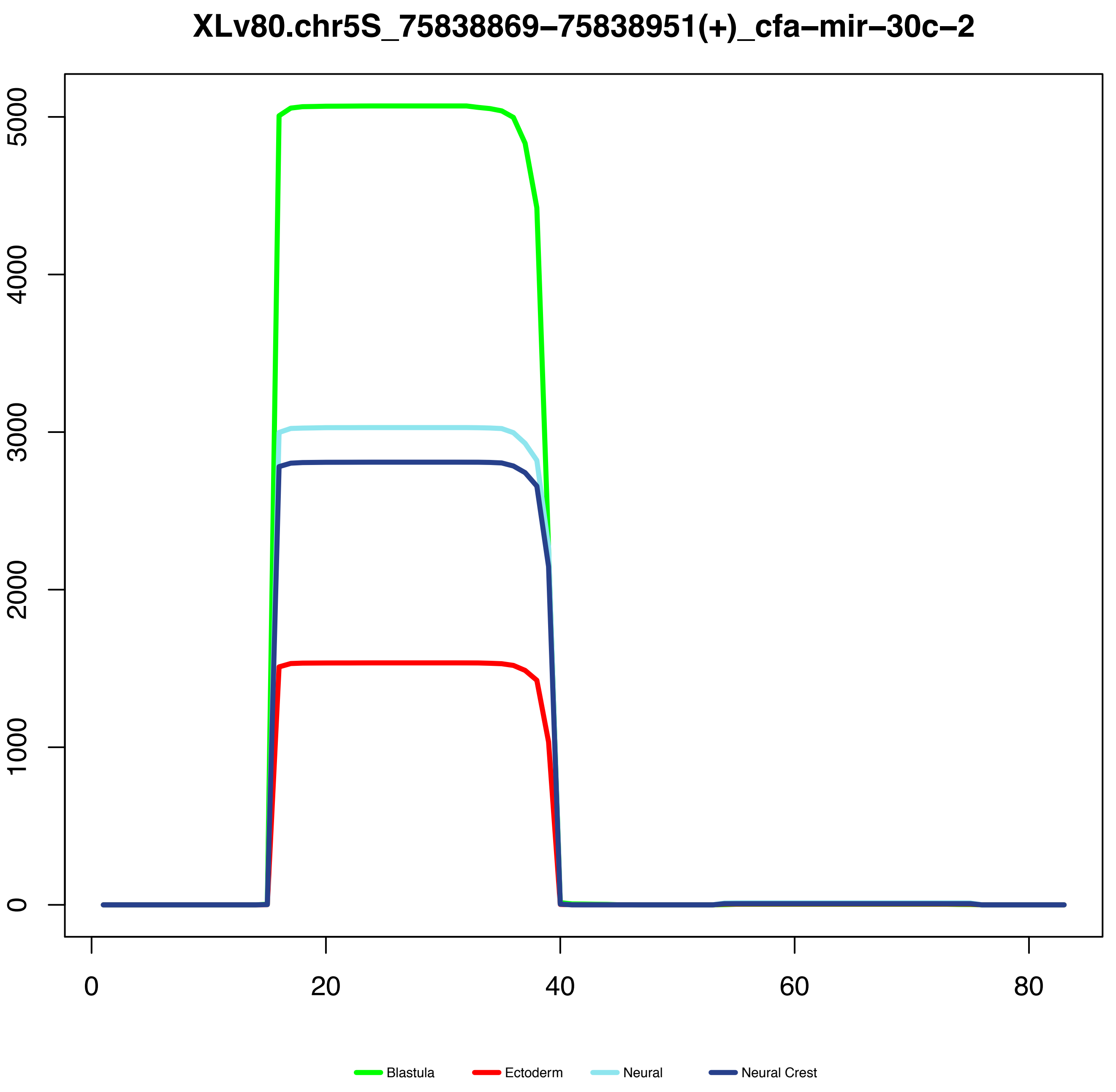

XLv80.chr5S_75838869−75838951(+)_cfa−mir−30c−2
0
1000
2000
3000
4000
5000
0
20
40
60
80
Blastula
Ectoderm
Neural
Neural Crest

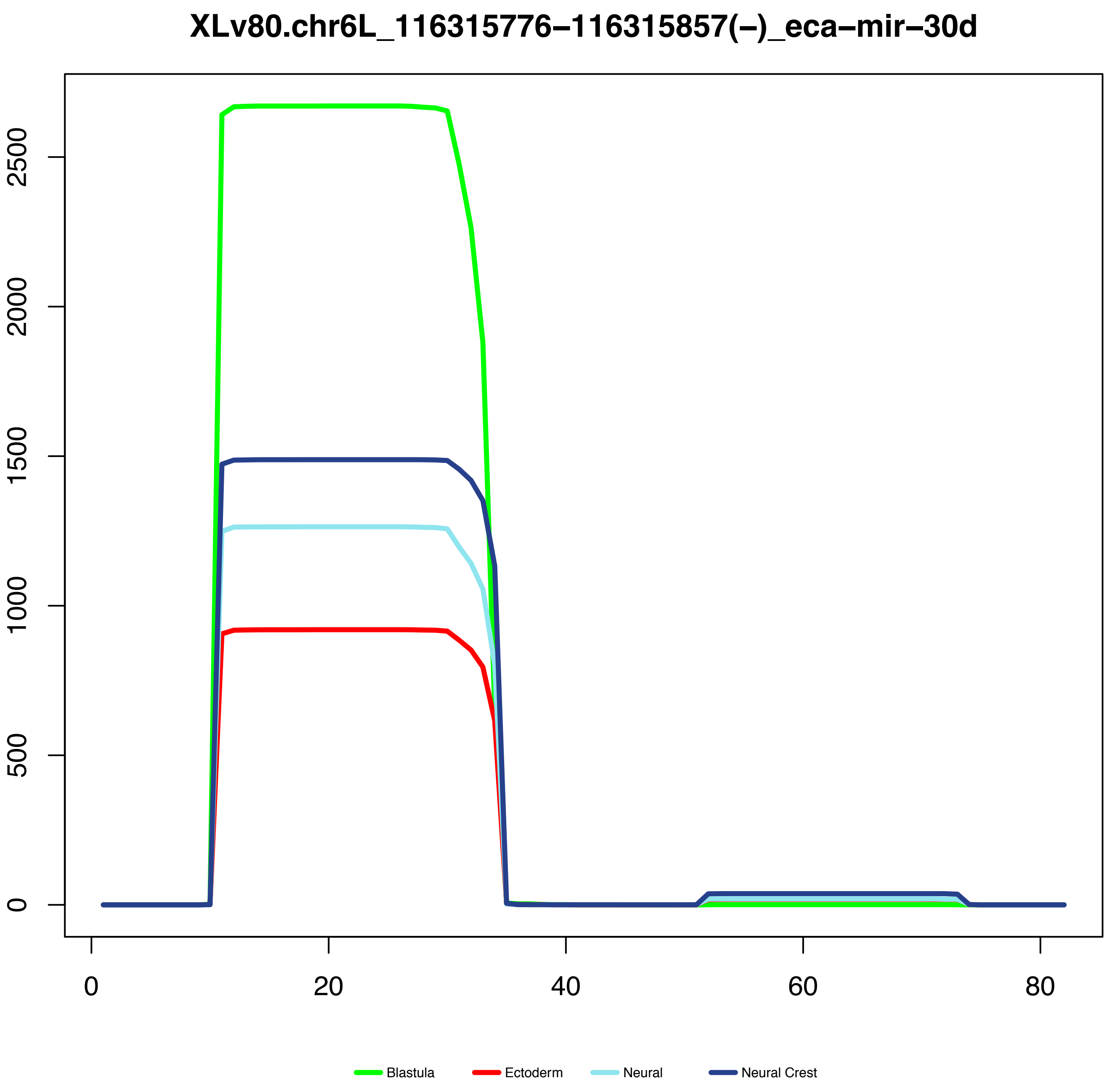

XLv80.chr6L_116315776−116315857(−)_eca−mir−30d
0
500
1000
1500
2000
2500
0
20
40
60
80
Blastula
Ectoderm
Neural
Neural Crest

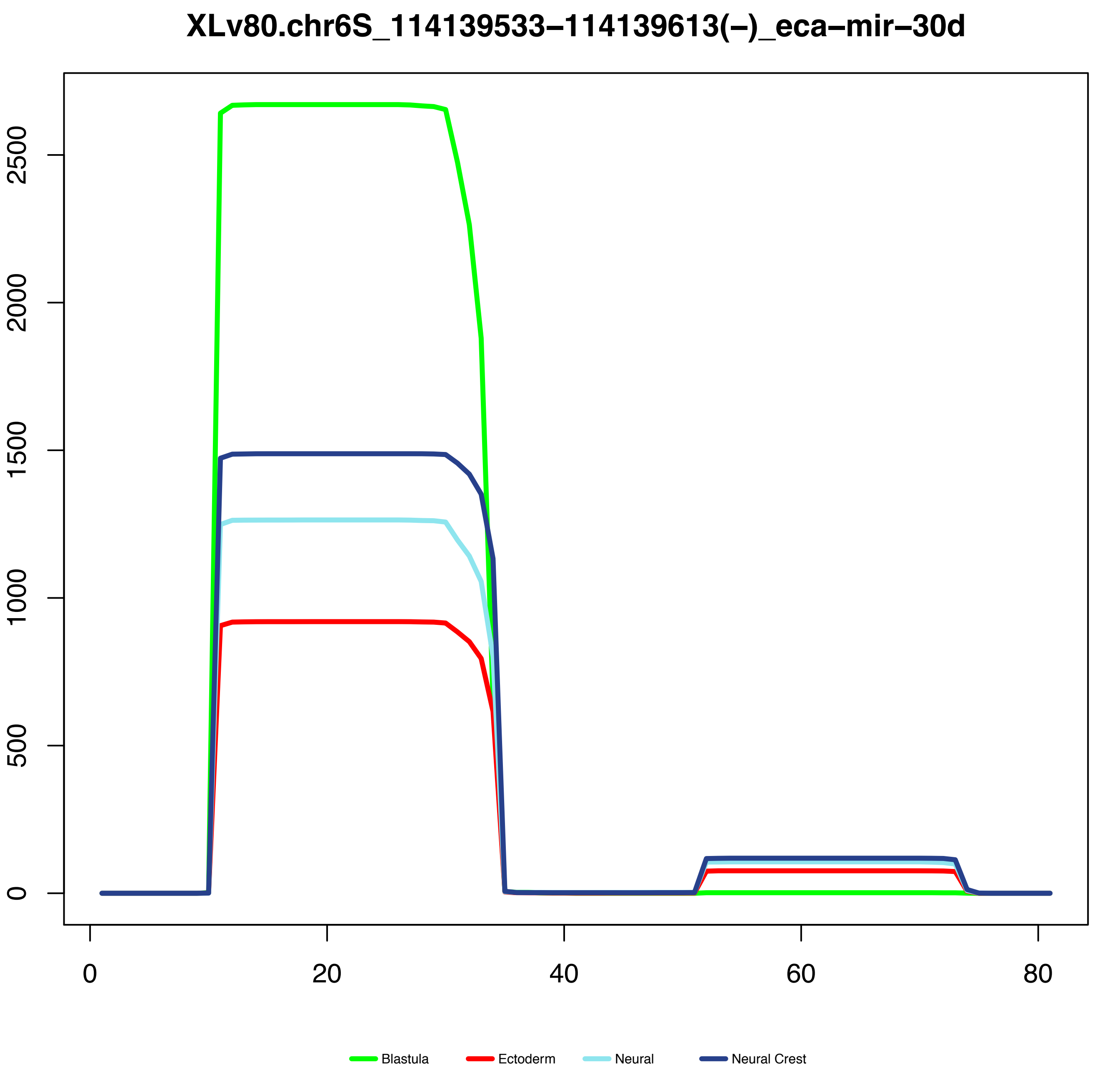
XLv80.chr6S_114139533–114139613(–)_eca–mir–30d
0
500
1000
1500
2000
2500
0
20
40
60
80
Blastula
Ectoderm
Neural
Neural Crest

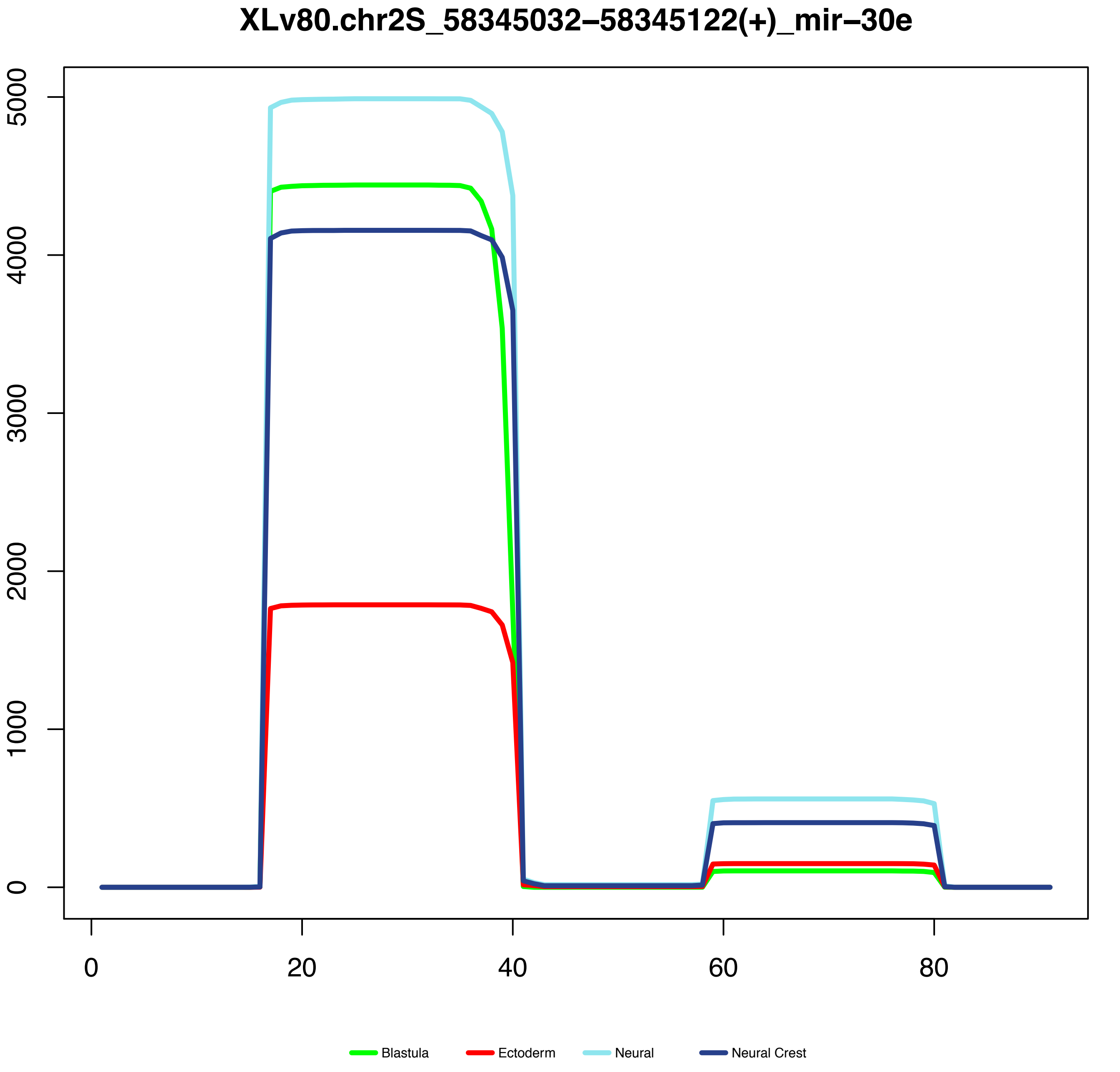
XLv80.chr2S_58345032−58345122(+)_mir−30e
5000
4000
3000
2000
1000
0
0
20
40
60
80
Blastula
Ectoderm
Neural
Neural Crest

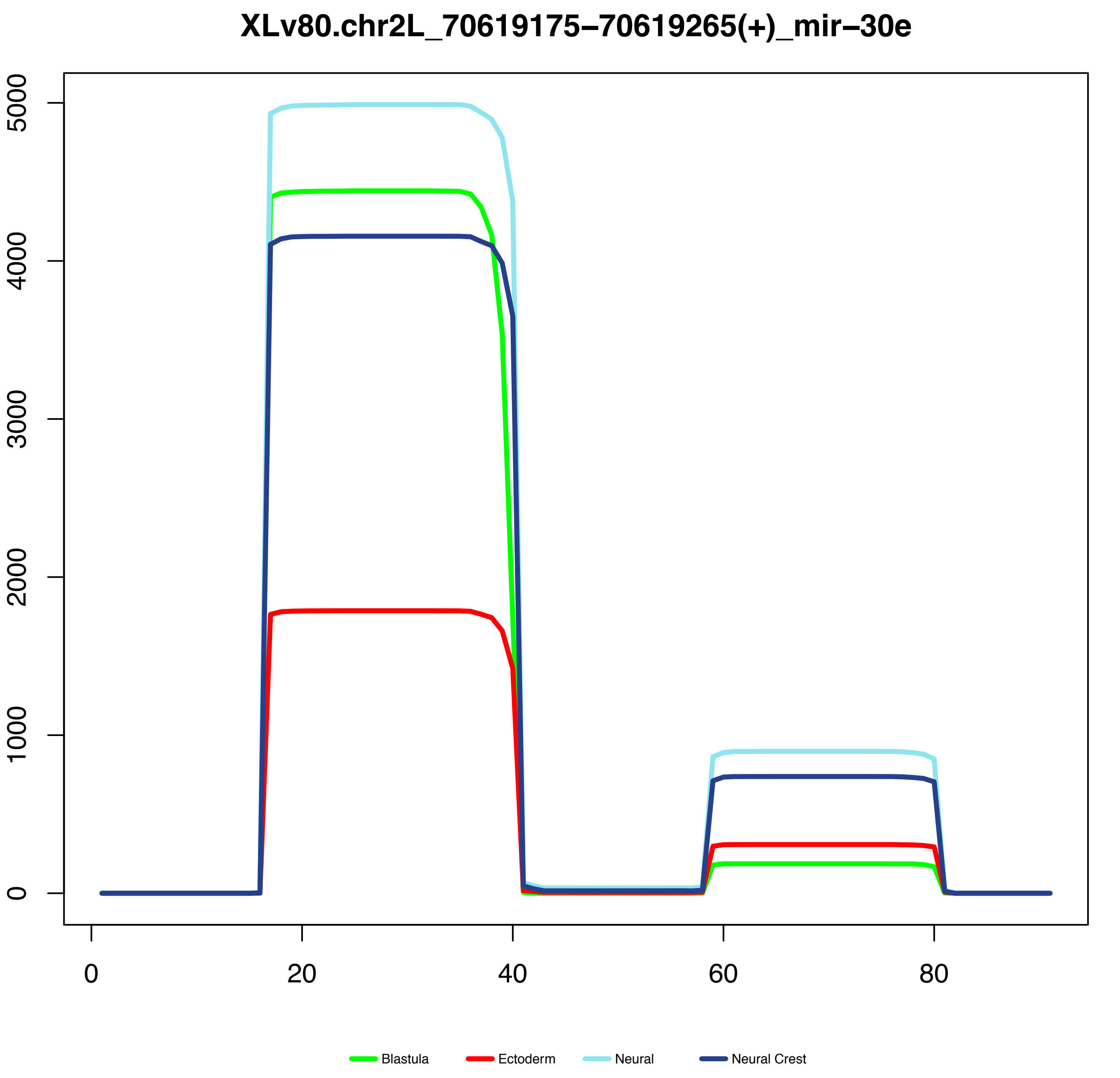

XLv80.chr2L_70619175−70619265(+)_mir−30e
0
1000
2000
3000
4000
5000
0
20
40
60
80
Blastula
Ectoderm
Neural
Neural Crest

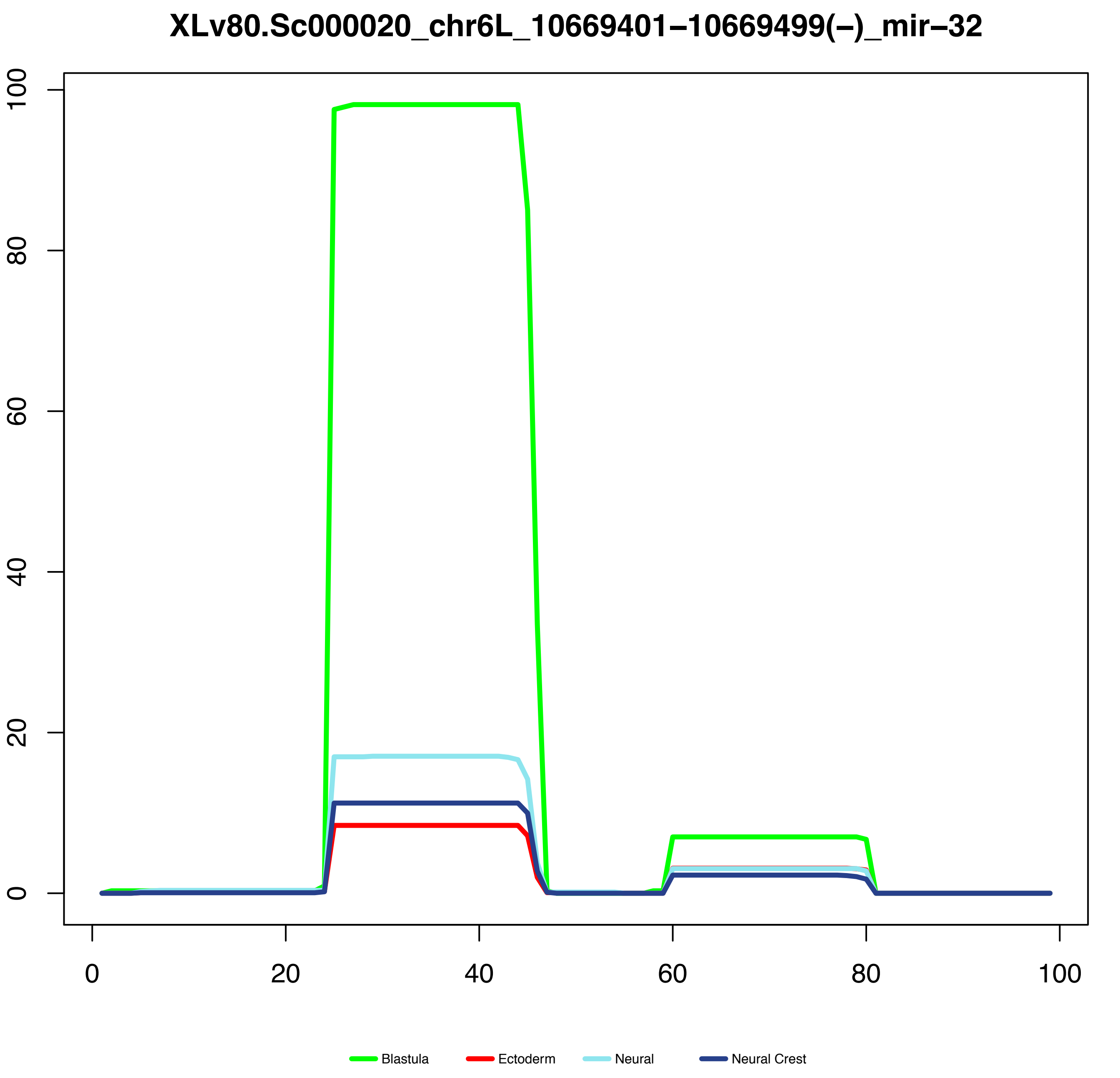
XLv80.Sc000020_chr6L_10669401−10669499(−)_mir−32
0
20
40
60
80
100
0
20
40
60
80
100
Blastula
Ectoderm
Neural
Neural Crest

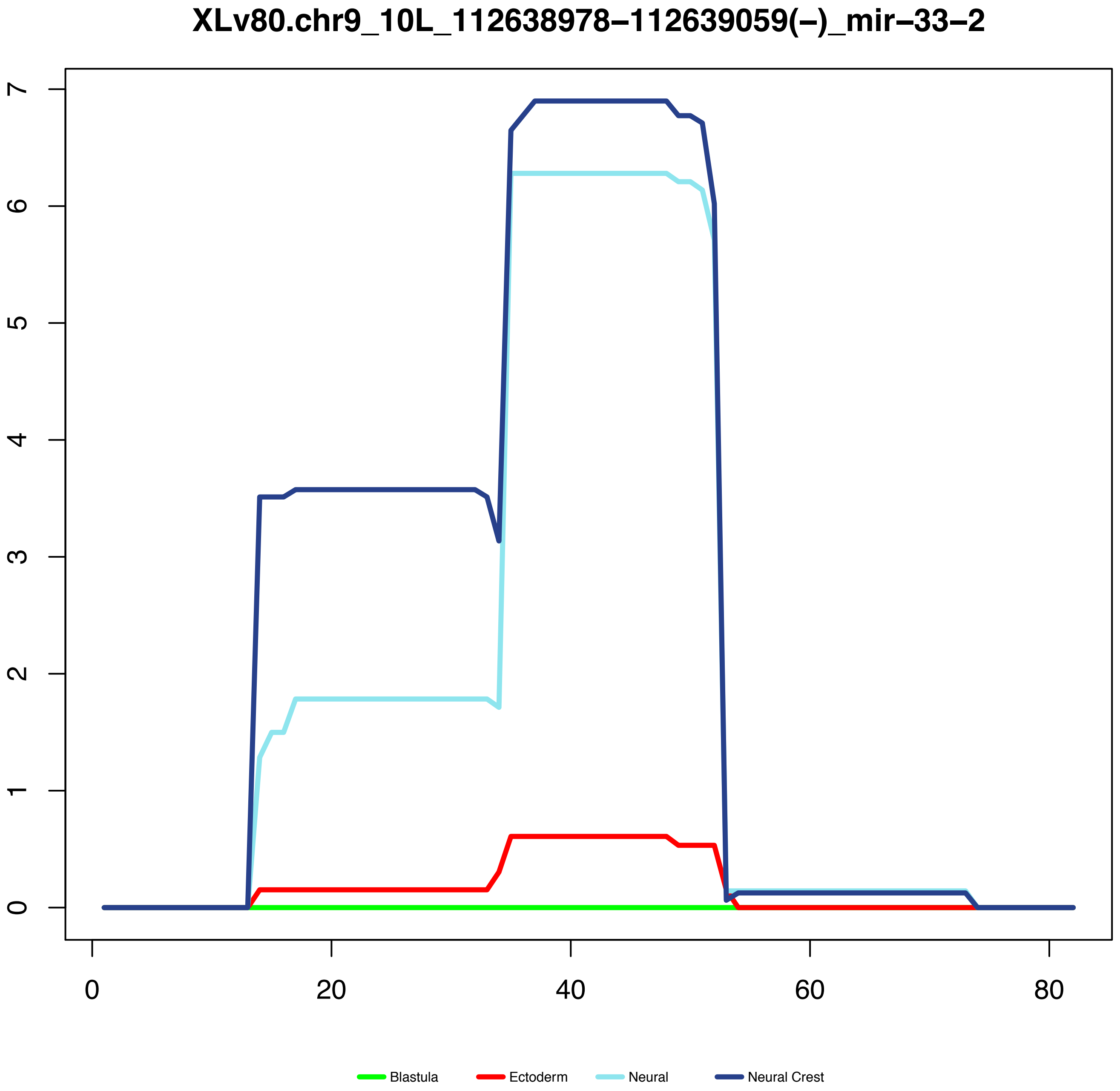

XLv80.chr9_10L_112638978−112639059(−)_mir−33−2
0
1
2
3
4
5
6
7
0
20
40
60
80
Blastula
Ectoderm
Neural
Neural Crest

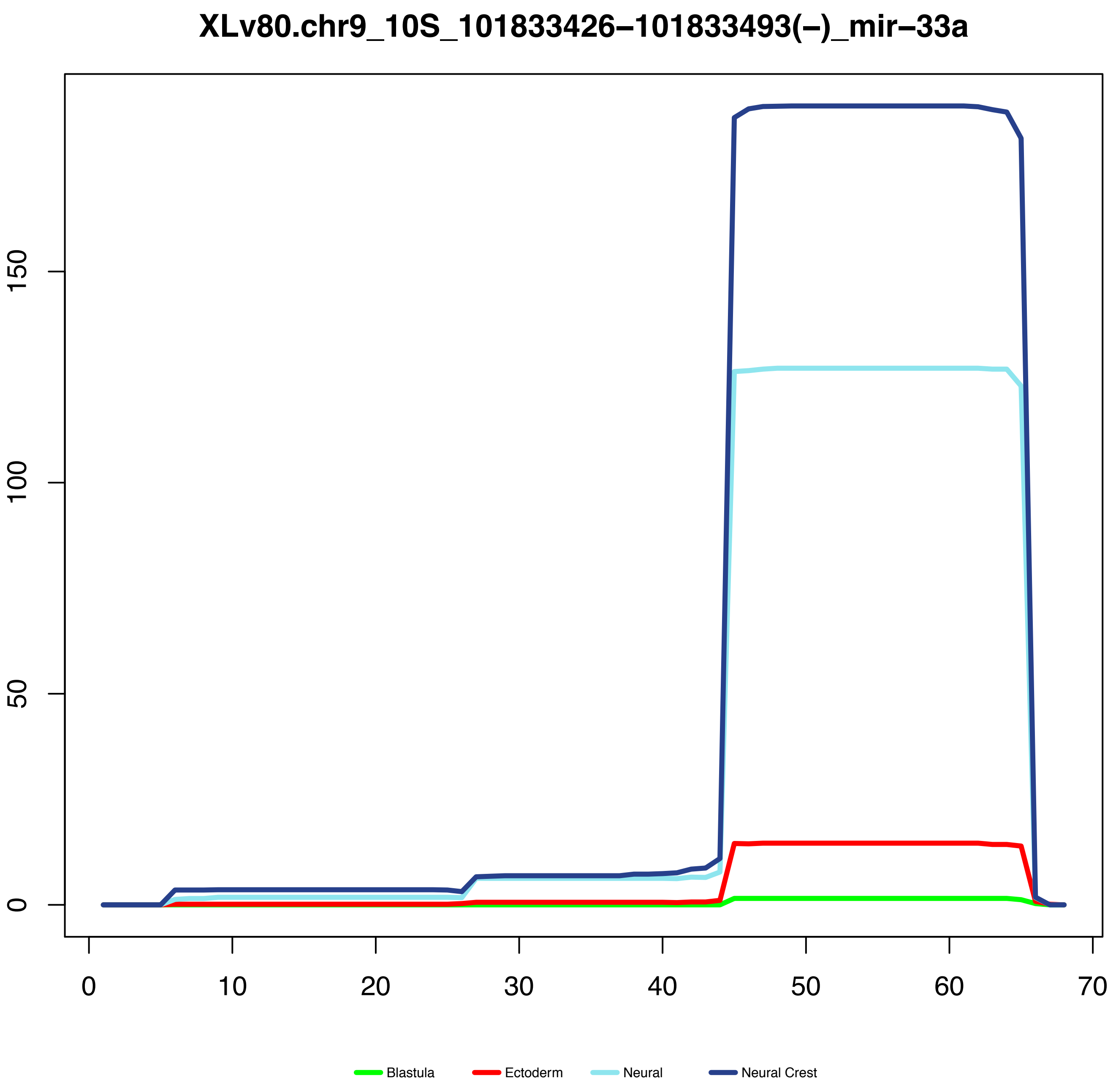
XLv80.chr9_10S_101833426−101833493(−)_mir−33a
0
50
100
150
0
10
20
30
40
50
60
70
Blastula
Ectoderm
Neural
Neural Crest

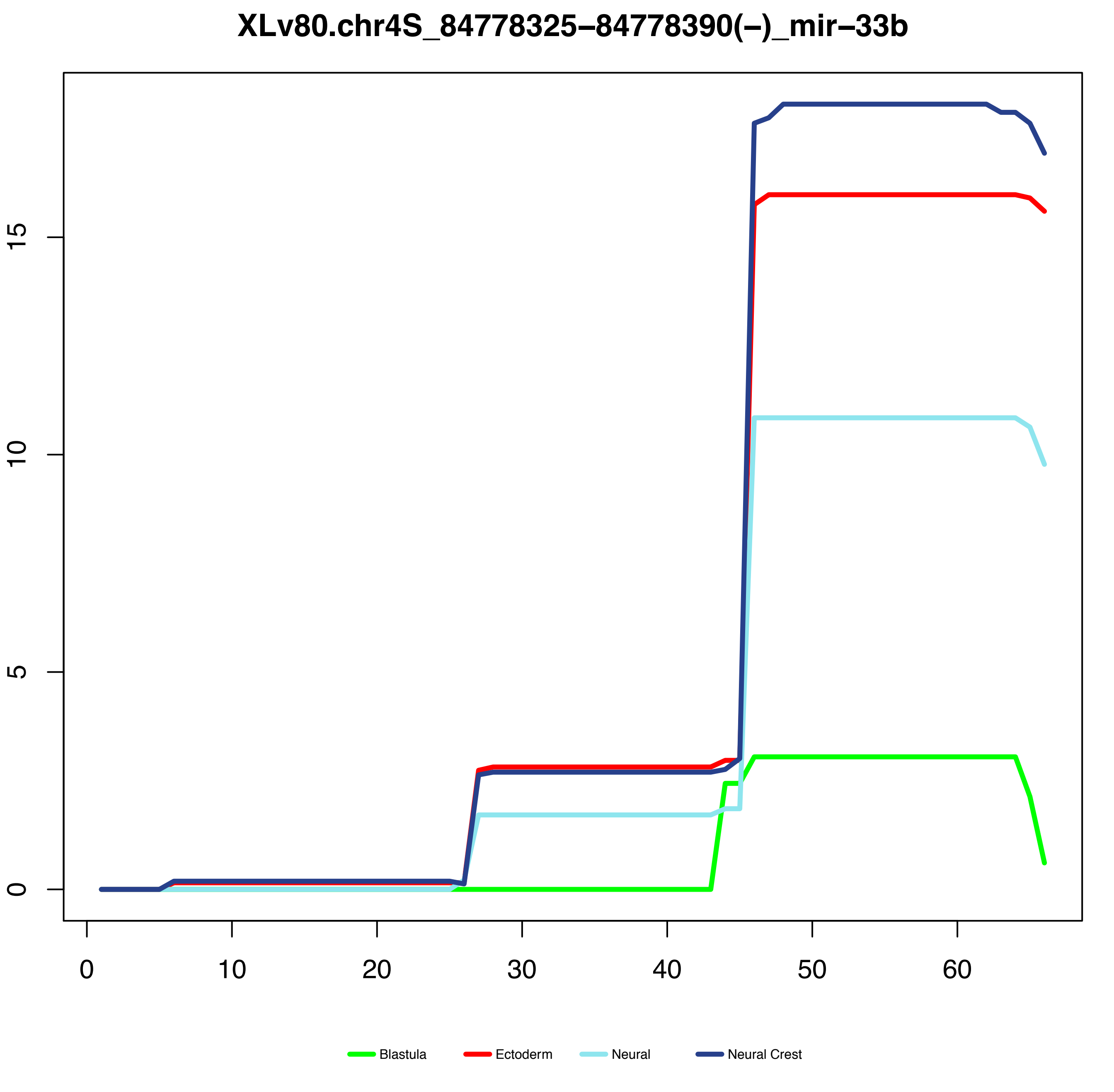
XLv80.chr4S_84778325–84778390(–)_mir–33b
0
5
10
15
0
10
20
30
40
50
60
Blastula
Ectoderm
Neural
Neural Crest

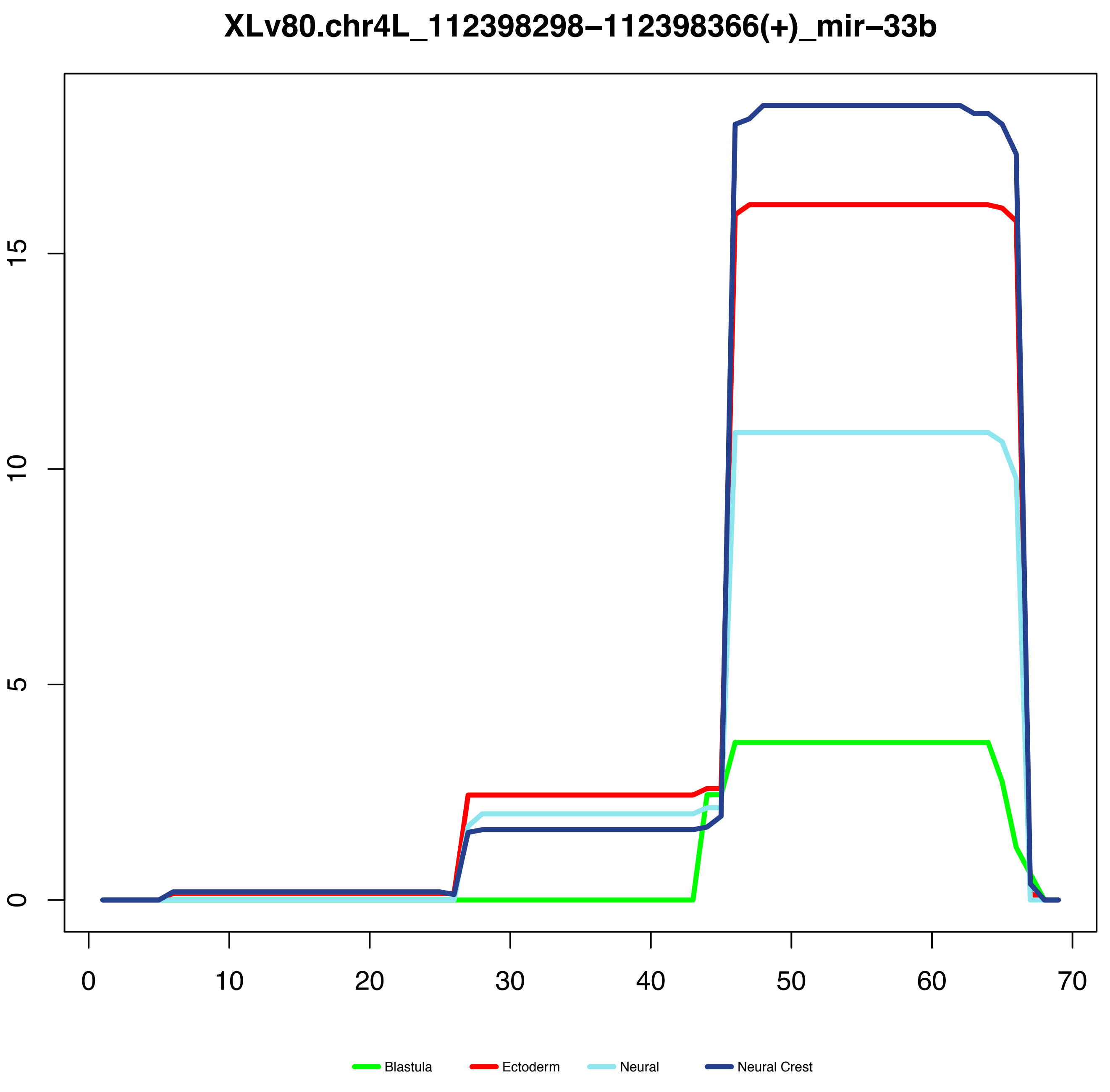

XLv80.chr4L_112398298−112398366(+)_mir−33b
0
5
10
15
0
10
20
30
40
50
60
70
Blastula
Ectoderm
Neural
Neural Crest

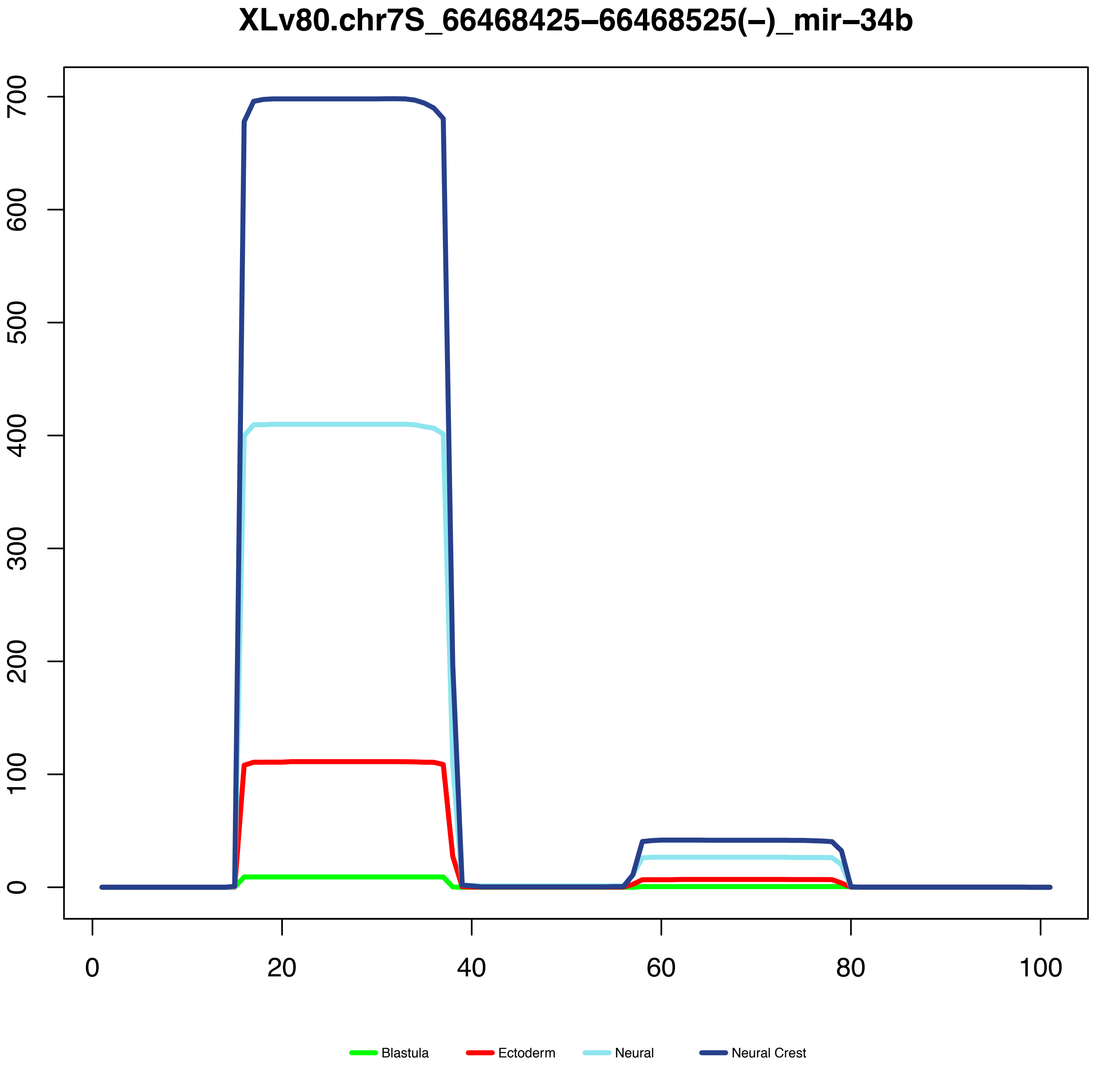
XLv80.chr7S_66468425−66468525(−)_mir−34b
0
100
200
300
400
500
600
700
0
20
40
60
80
100
Blastula
Ectoderm
Neural
Neural Crest

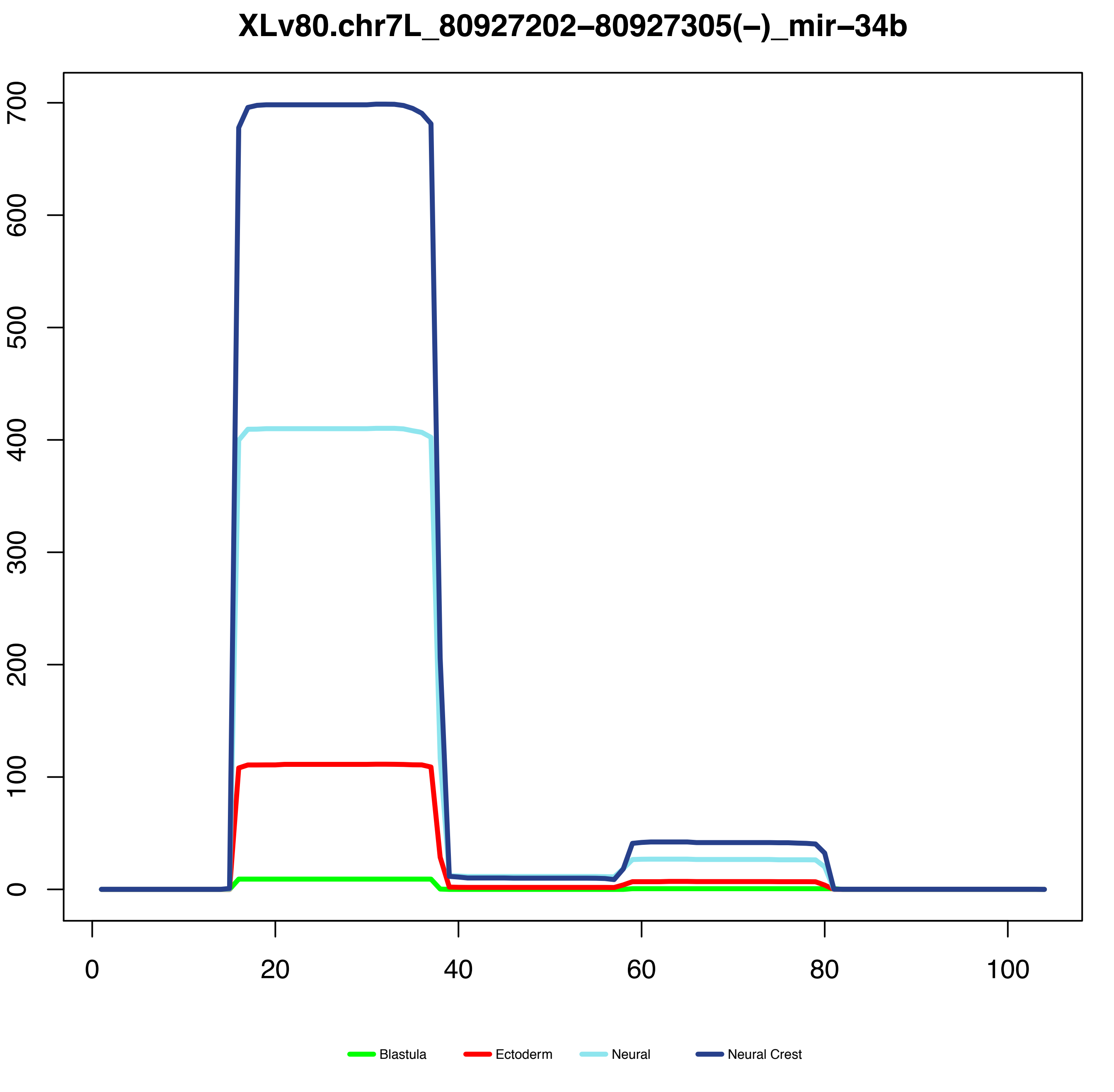

XLv80.chr7L_80927202−80927305(−)_mir−34b
0
100
200
300
400
500
600
700
0
20
40
60
80
100
Blastula
Ectoderm
Neural
Neural Crest

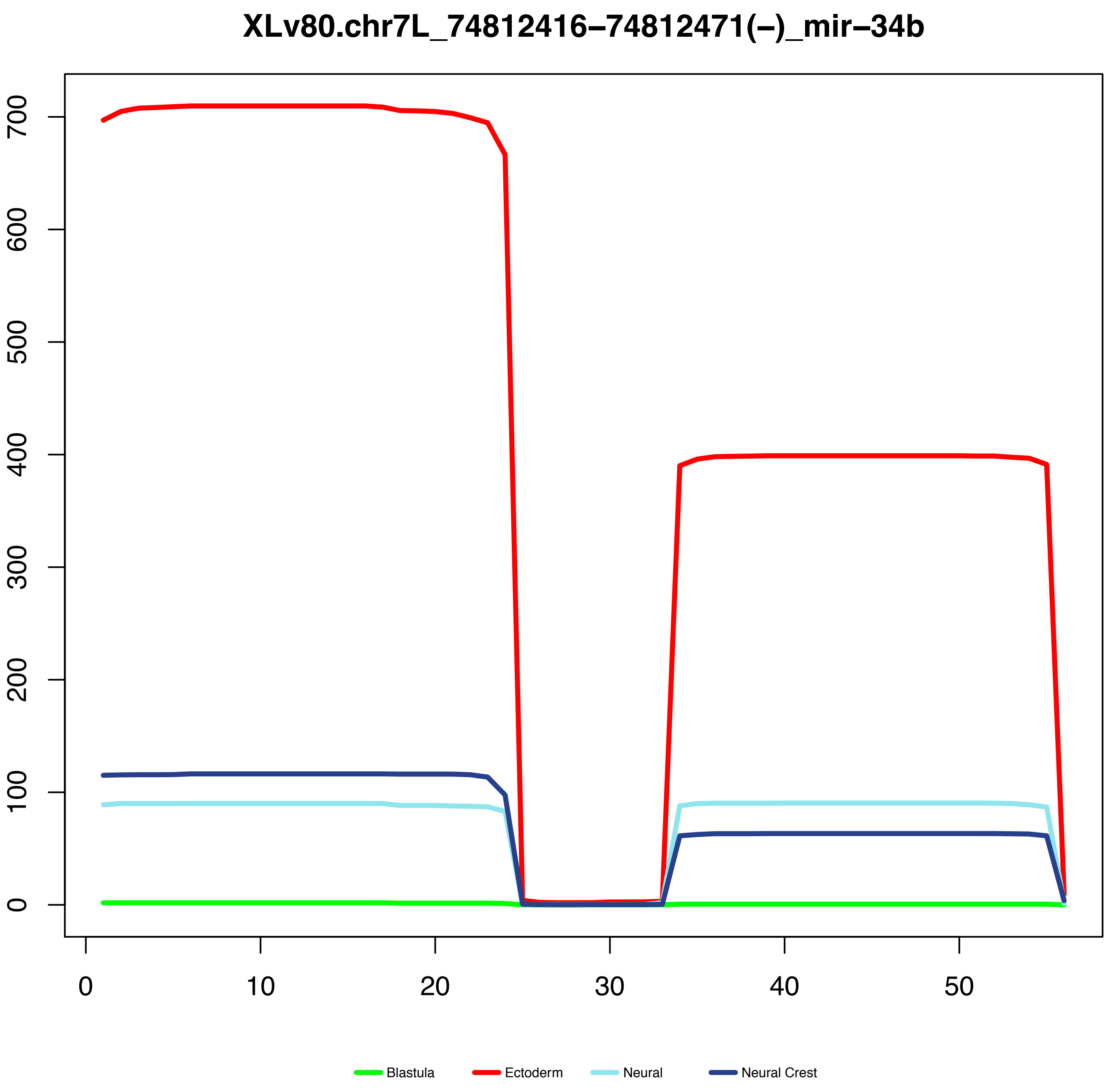
XLv80.chr7L_74812416–74812471(–)_mir–34b
0
100
200
300
400
500
600
700
0
10
20
30
40
50
Blastula
Ectoderm
Neural
Neural Crest

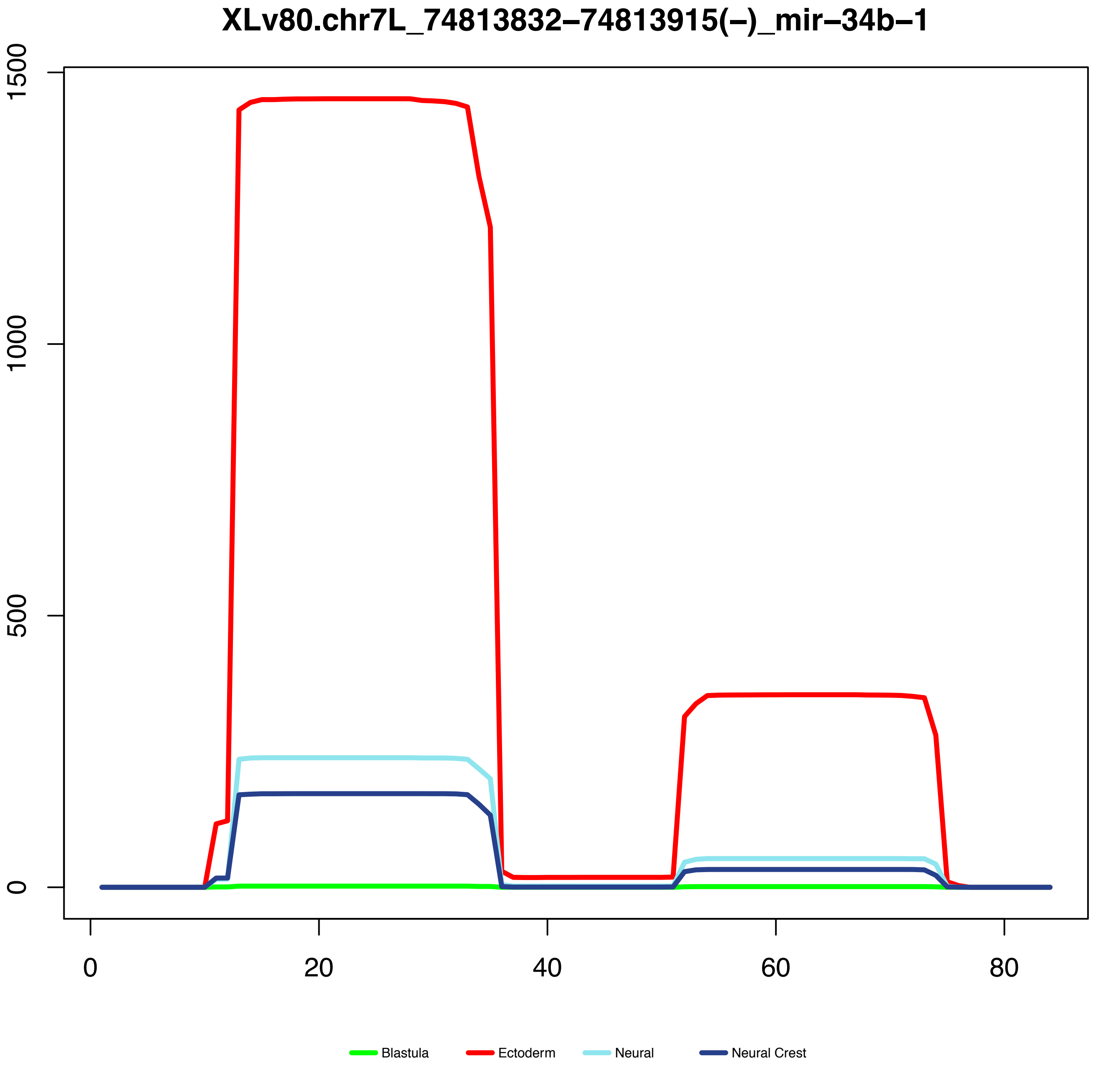
XLv80.chr7L_74813832−74813915(−)_mir−34b−1
1500
1000
500
0
0
20
40
60
80
Blastula
Ectoderm
Neural
Neural Crest

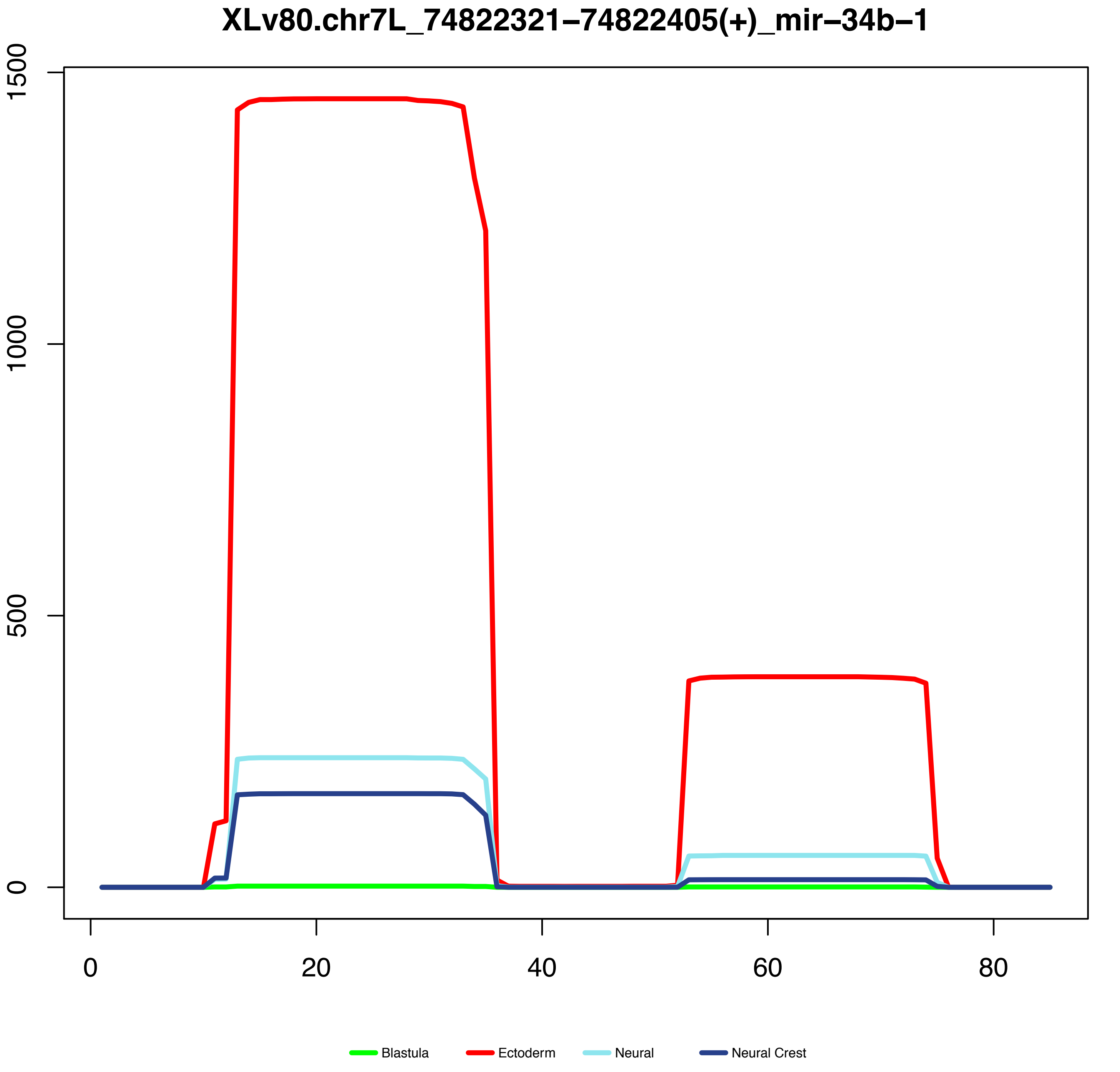
XLv80.chr7L_74822321−74822405(+)_mir−34b−1
0
500
1000
1500
0
20
40
60
80
Blastula
Ectoderm
Neural
Neural Crest

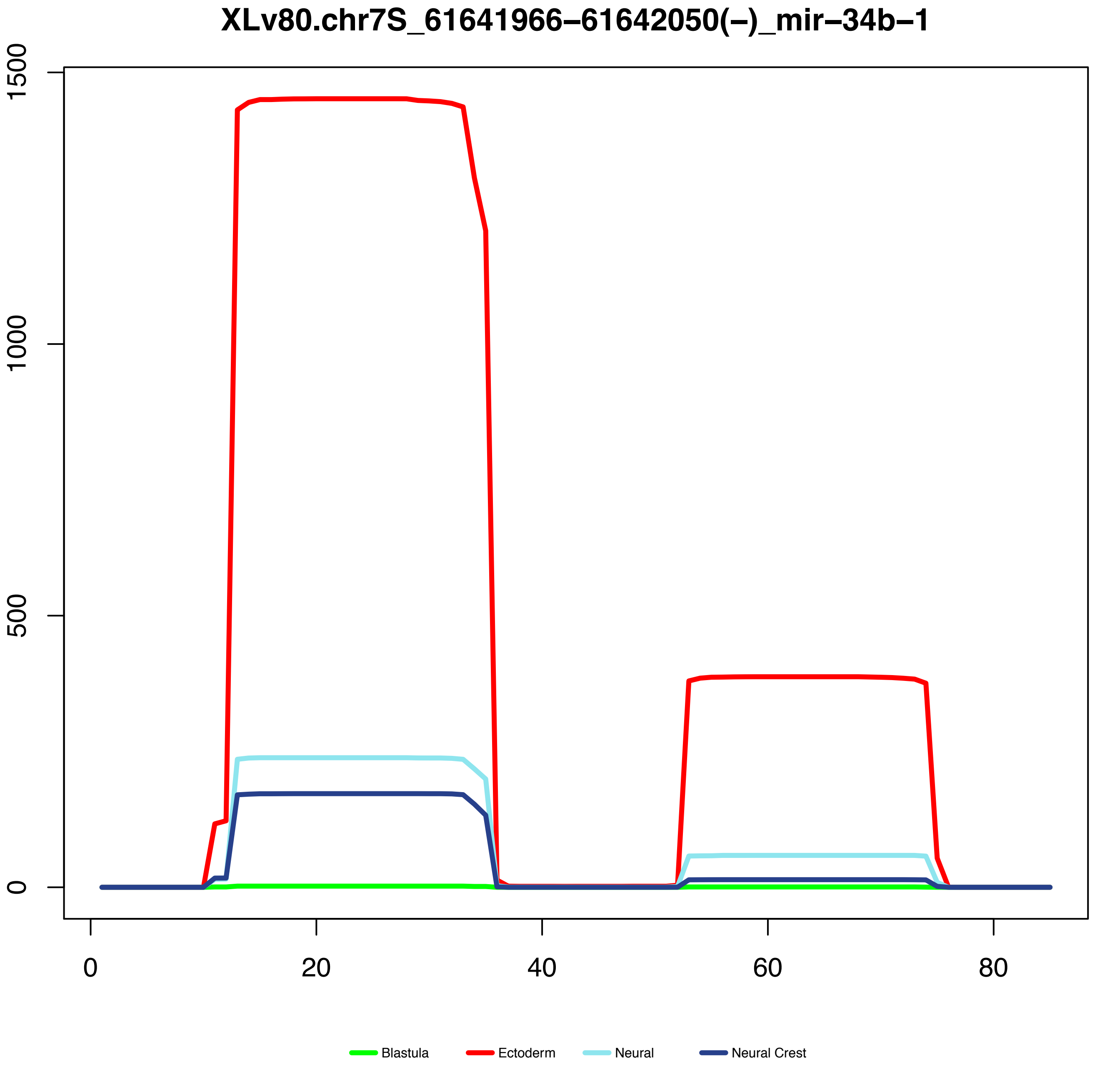

XLv80.chr7S_61641966–61642050(–)_mir–34b–1
1500
1000
500
0
0
20
40
60
80
Blastula
Ectoderm
Neural
Neural Crest

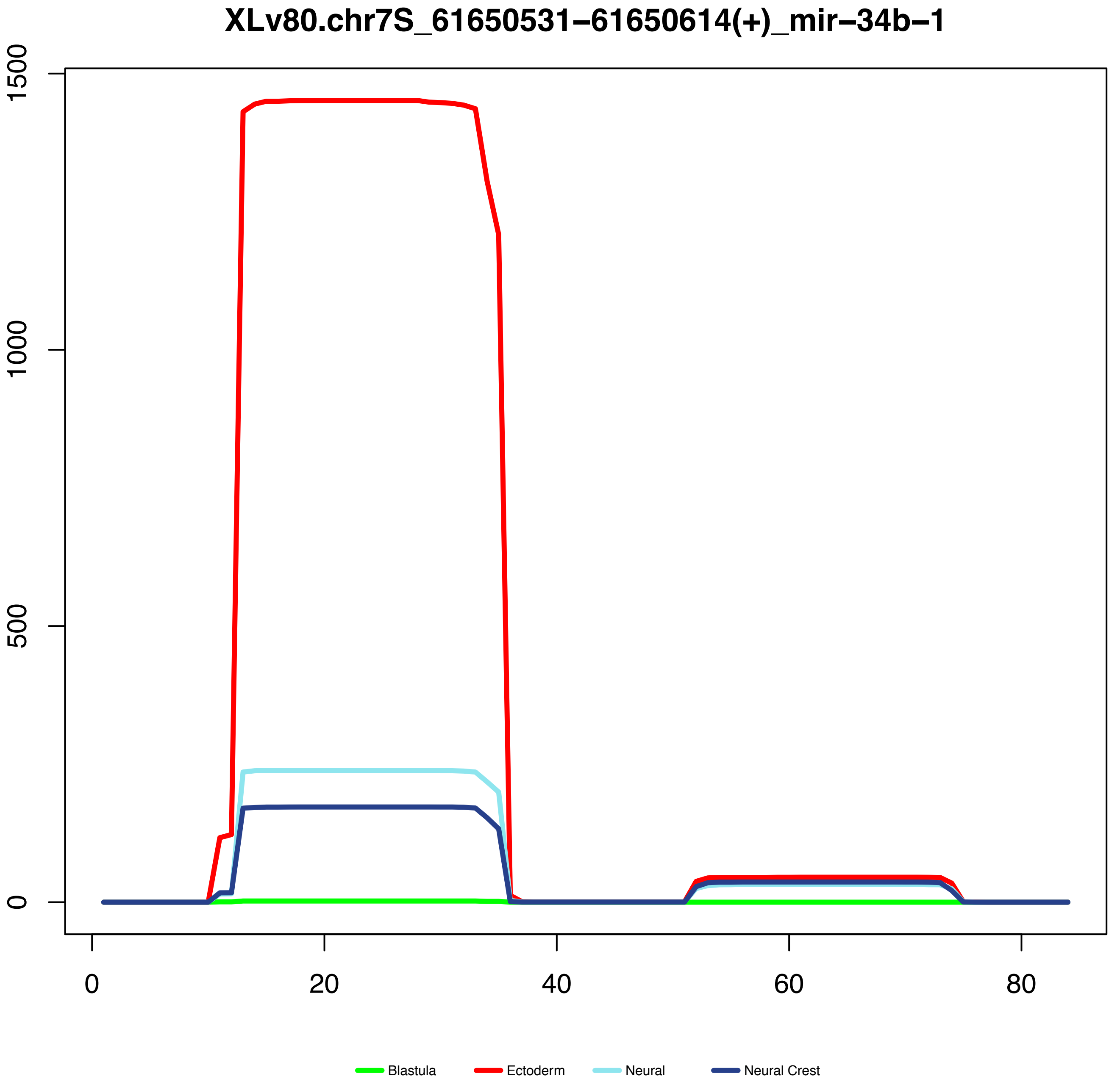
XLv80.chr7S_61650531−61650614(+)_mir−34b−1
1500
1000
500
0
0
20
40
60
80
Blastula
Ectoderm
Neural
Neural Crest

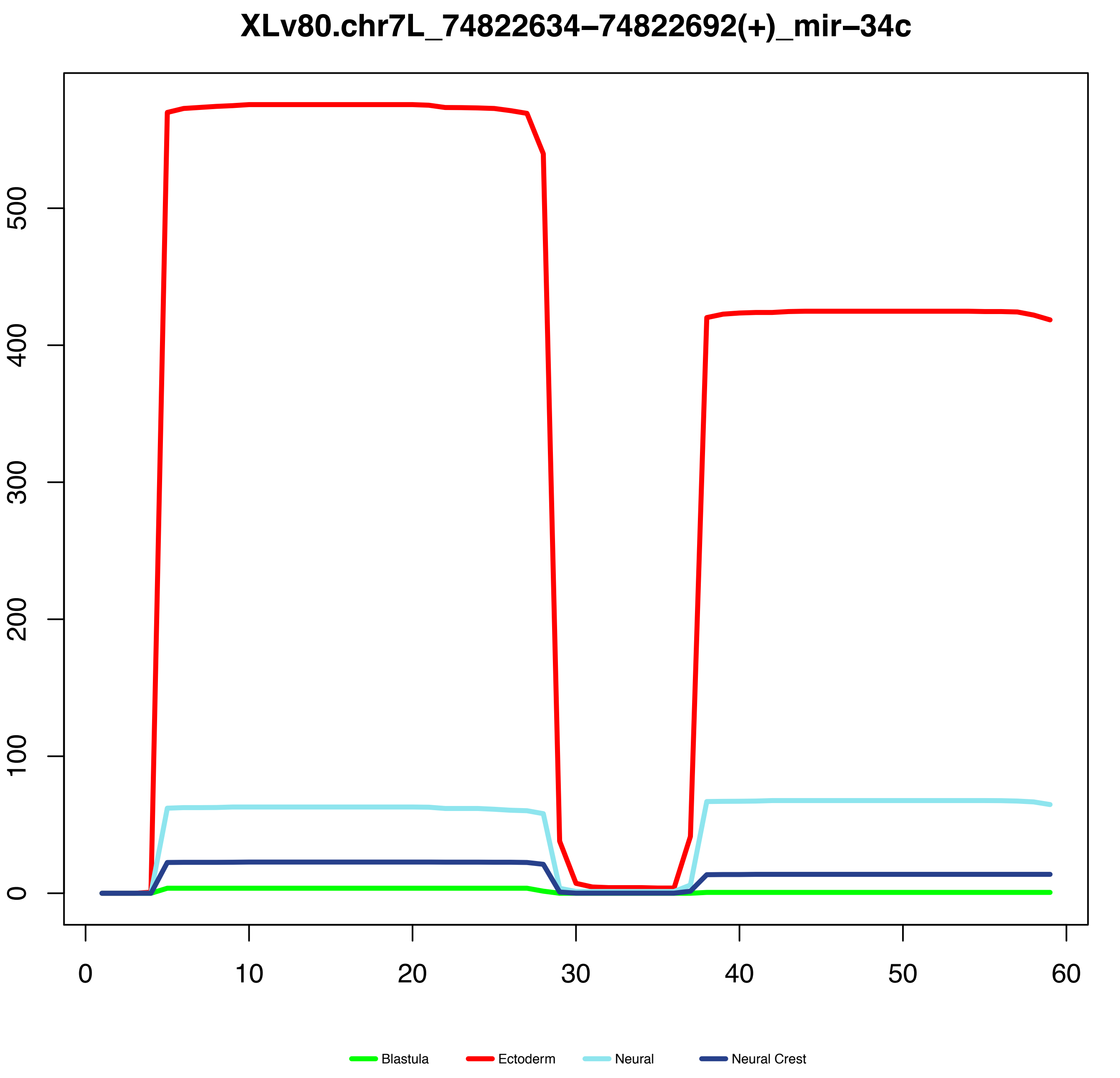

XLv80.chr7L_74822634−74822692(+)_mir−34c
0
100
200
300
400
500
0
10
20
30
40
50
60
Blastula
Ectoderm
Neural
Neural Crest

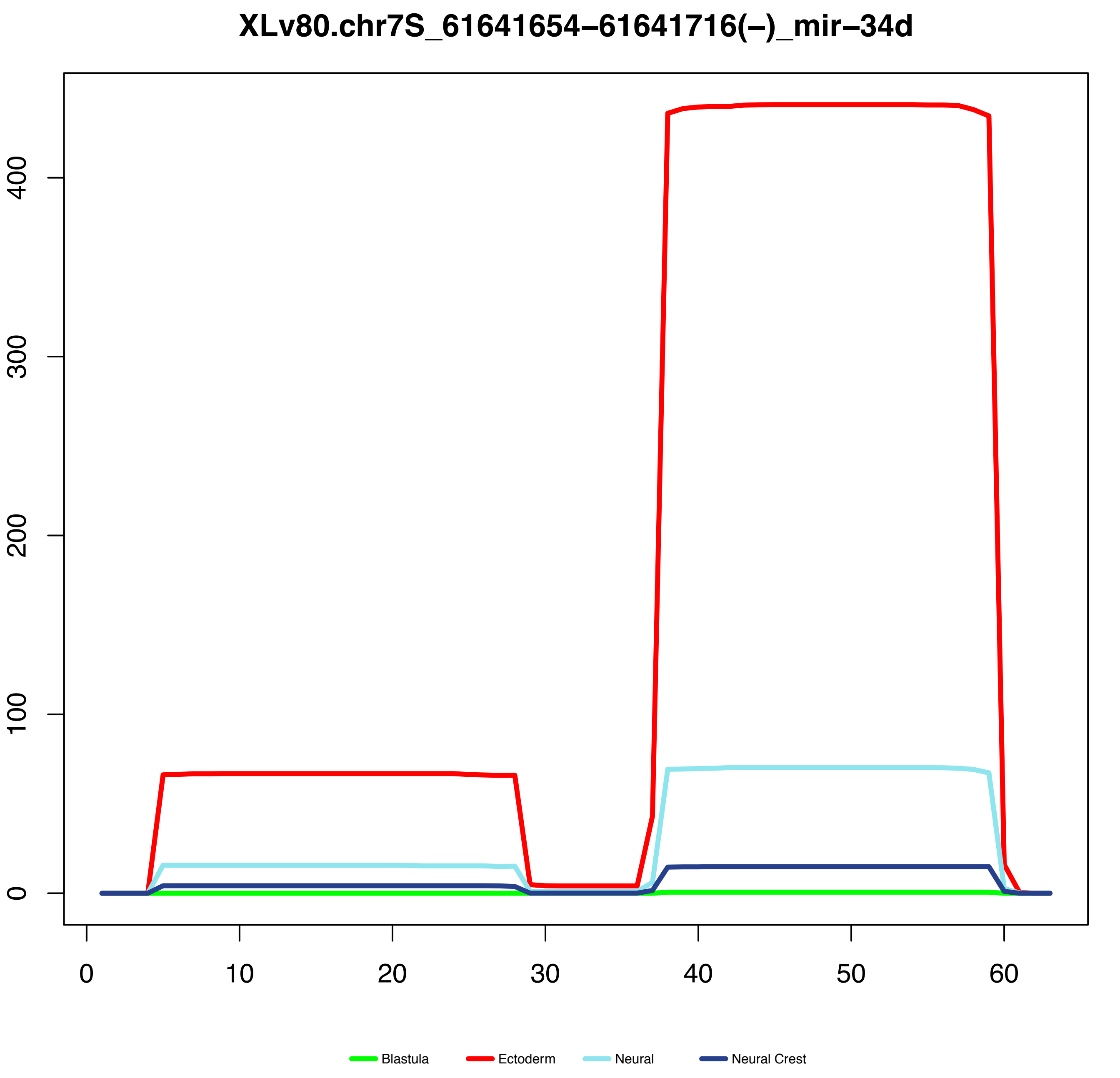

XLv80.chr7S_61641654−61641716(−)_mir−34d
0
100
200
300
400
0
10
20
30
40
50
60
Blastula
Ectoderm
Neural
Neural Crest

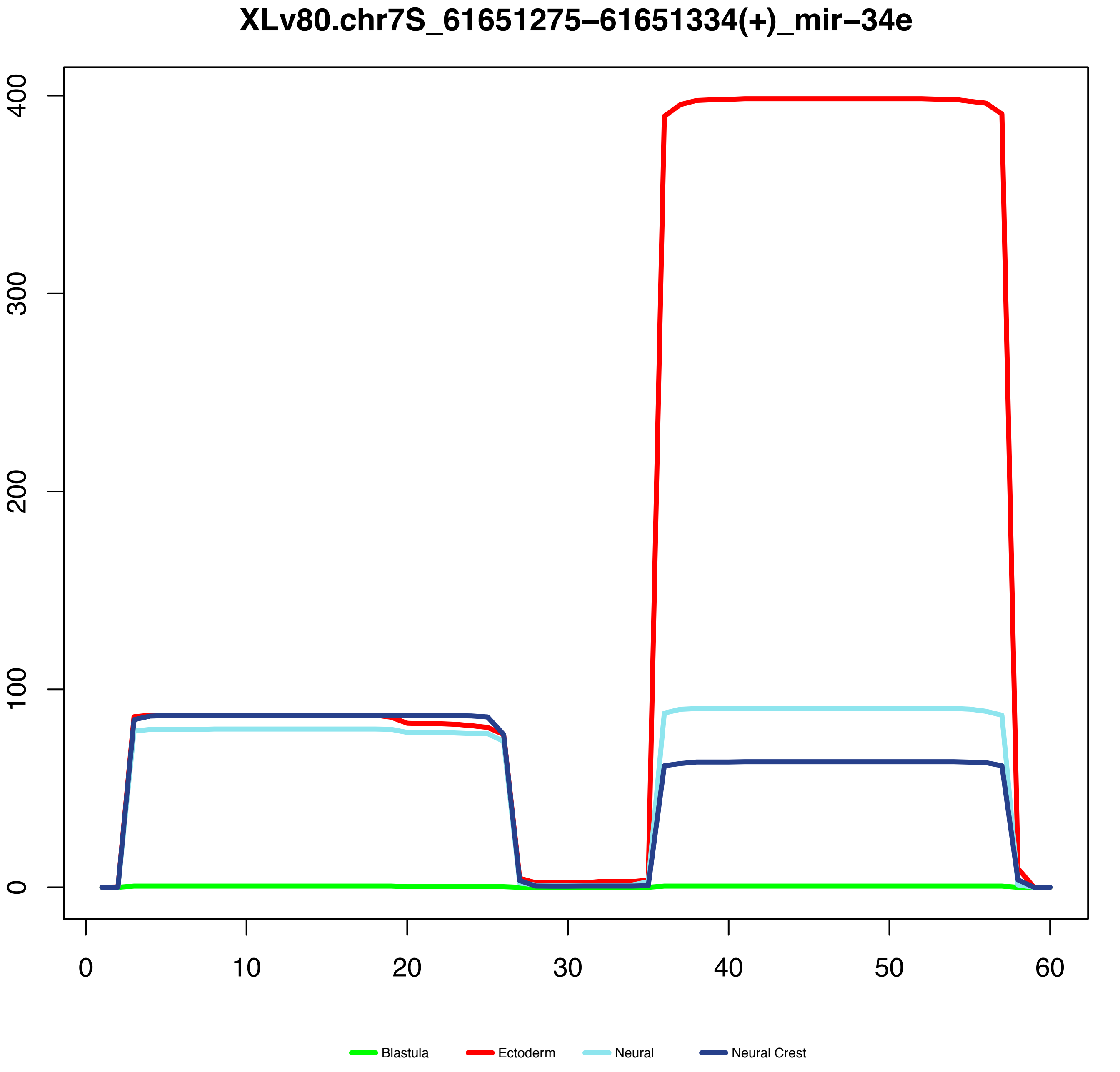
XLv80.chr7S_61651275−61651334(+)_mir−34e
0
100
200
300
400
0
10
20
30
40
50
60
Blastula
Ectoderm
Neural
Neural Crest

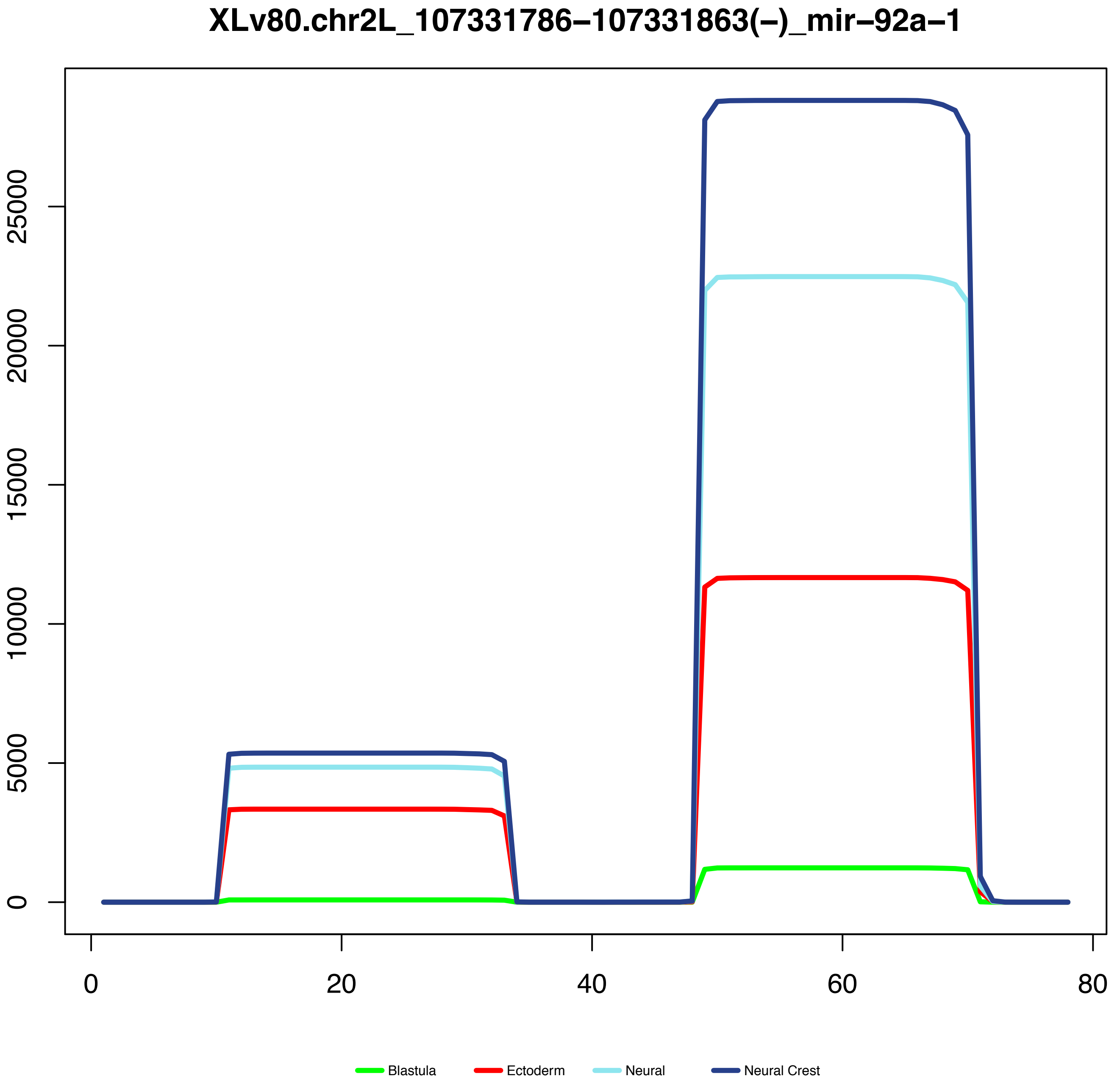

XLv80.chr2L_107331786–107331863(–)_mir–92a–1
0
5000
10000
15000
20000
25000
0
20
40
60
80
Blastula
Ectoderm
Neural
Neural Crest

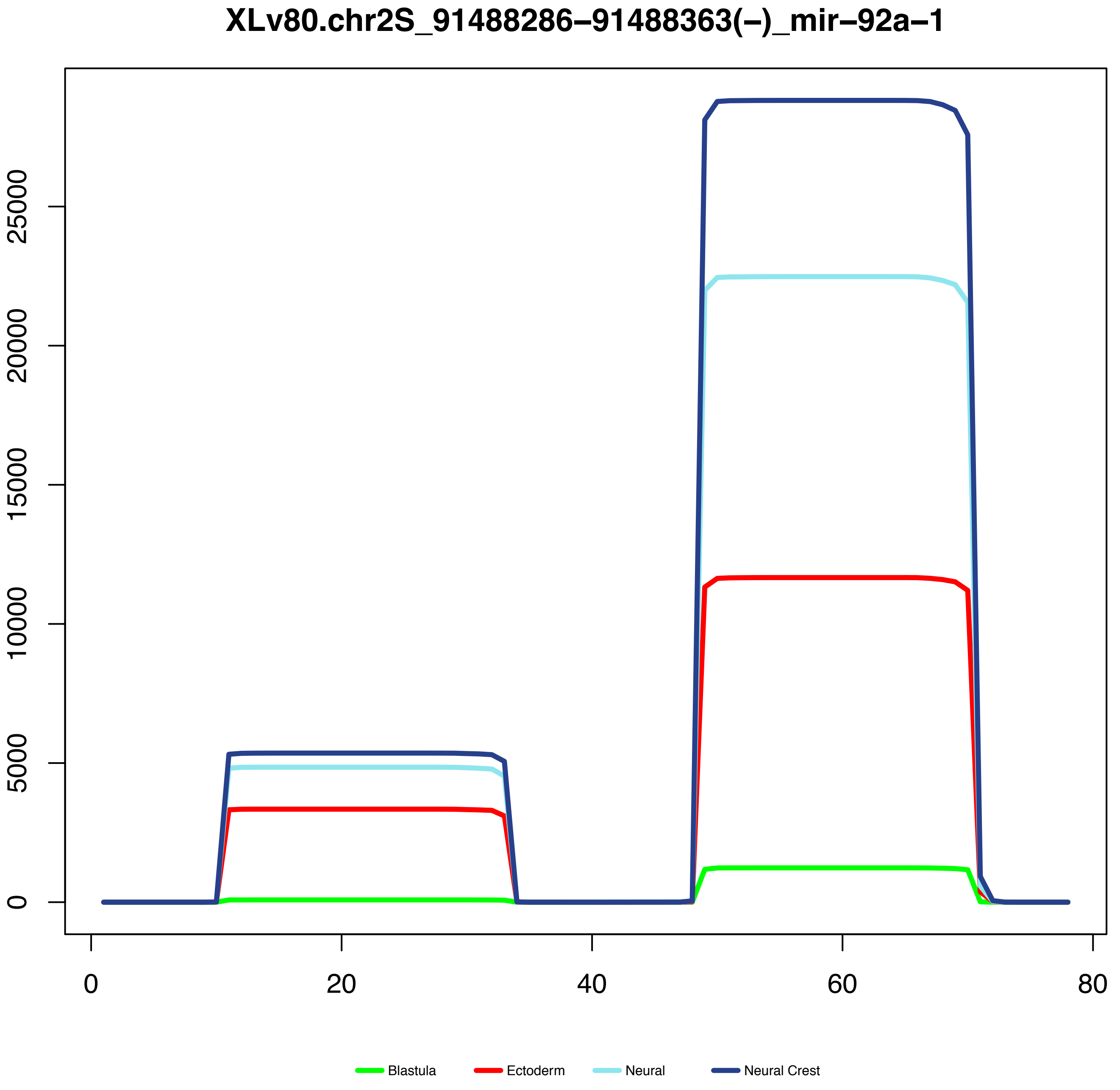
XLv80.chr2S_91488286−91488363(−)_mir−92a−1
0
5000
10000
15000
20000
25000
0
20
40
60
80
Blastula
Ectoderm
Neural
Neural Crest

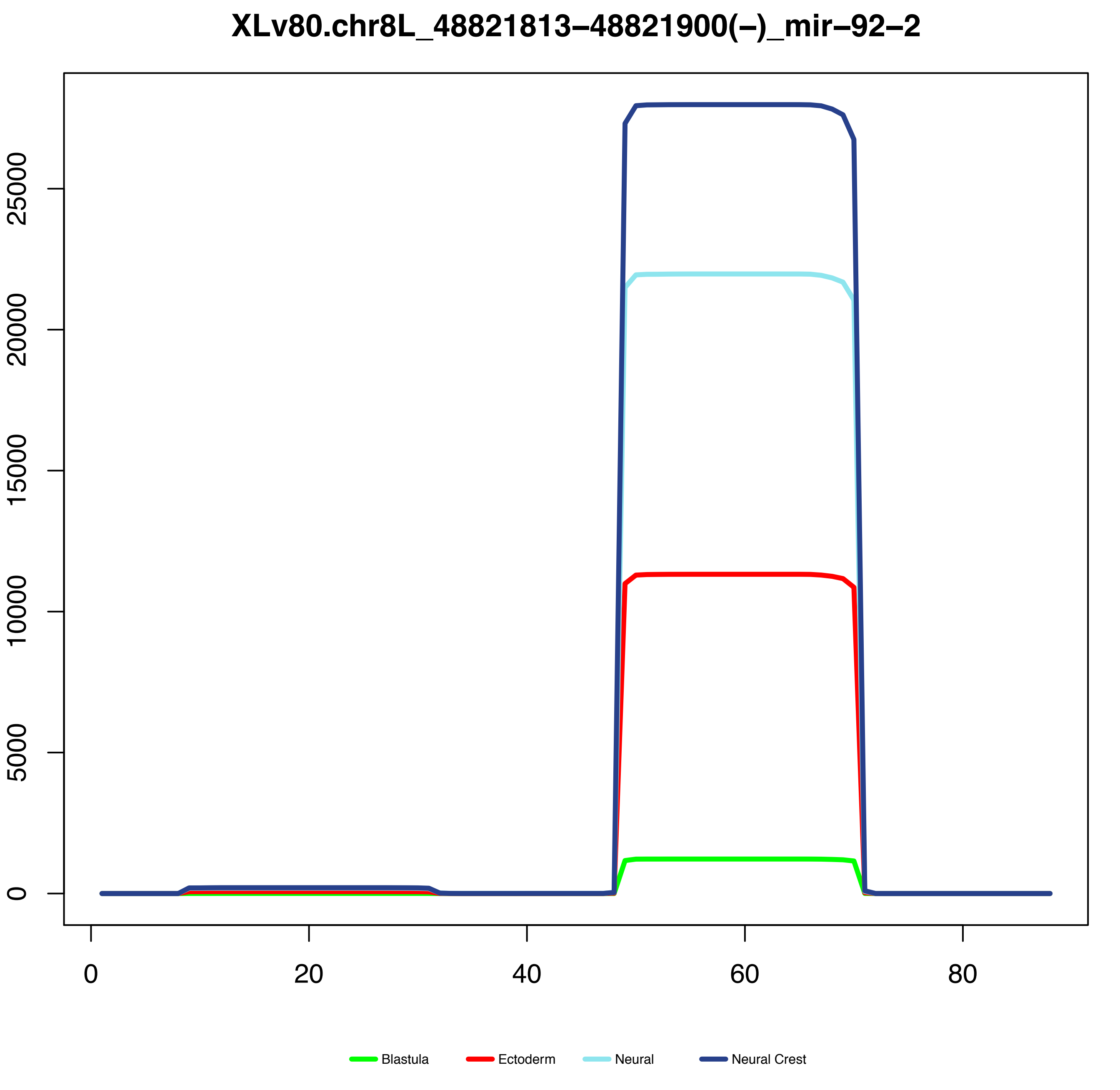

XLv80.chr8L_48821813−48821900(−)_mir−92−2
0
5000
10000
15000
20000
25000
0
20
40
60
80
Blastula
Ectoderm
Neural
Neural Crest

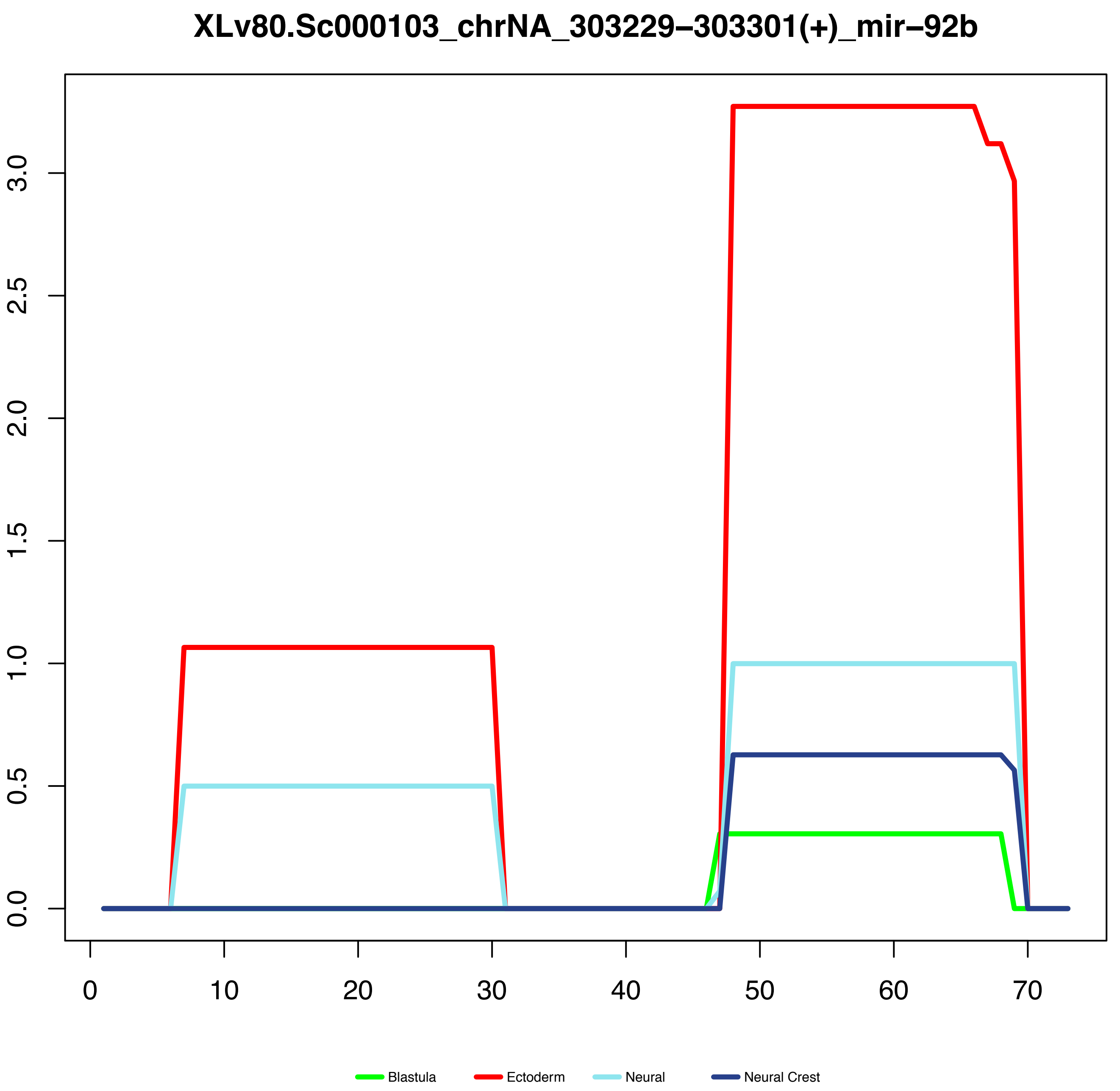
XLv80.Sc000103_chrNA_303229−303301(+)_mir−92b
0.0
0.5
1.0
1.5
2.0
2.5
3.0
0
10
20
30
40
50
60
70
Blastula
Ectoderm
Neural
Neural Crest

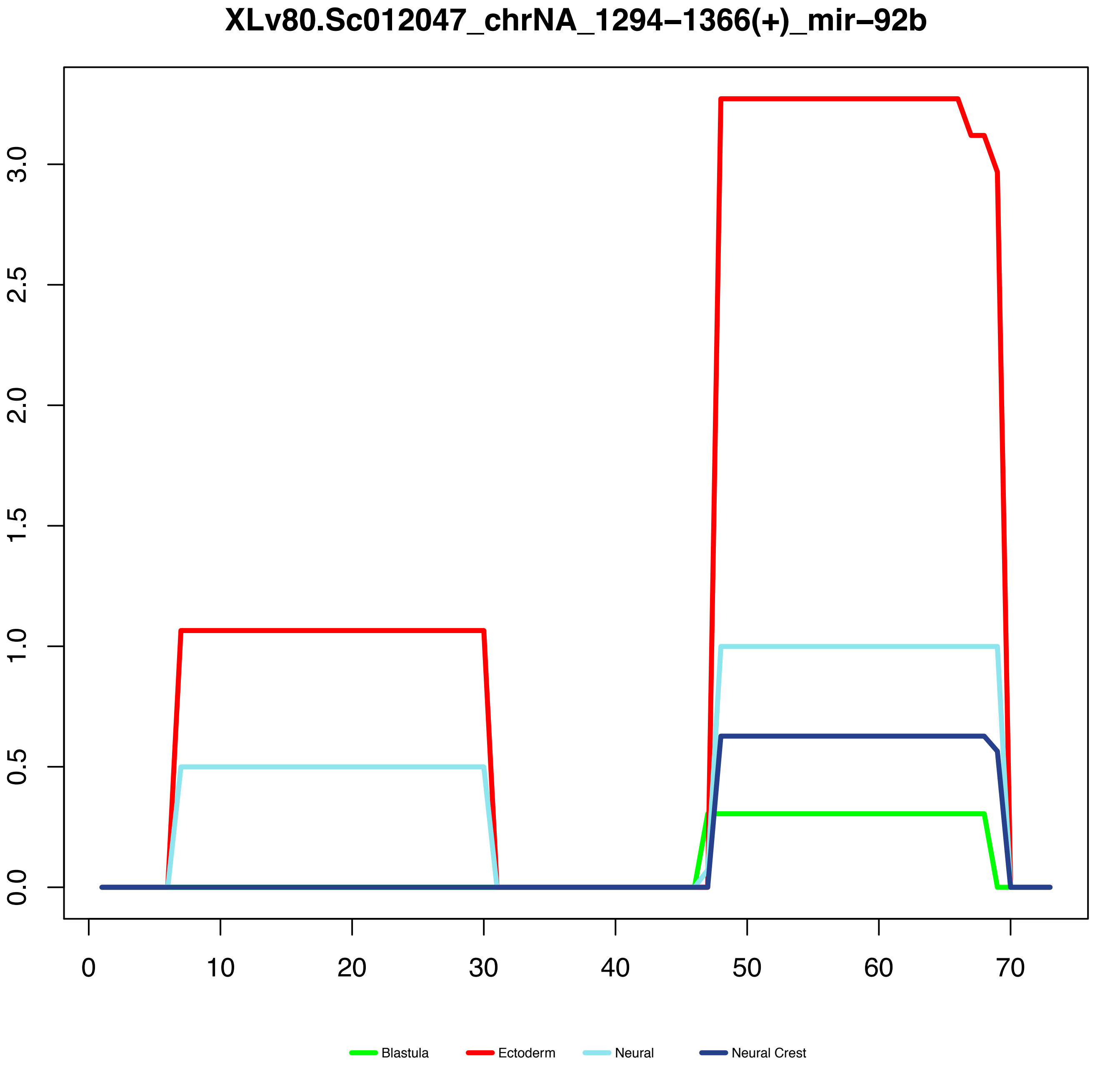
XLv80.Sc012047_chrNA_1294−1366(+)_mir−92b
0.0
0.5
1.0
1.5
2.0
2.5
3.0
0
10
20
30
40
50
60
70
Blastula
Ectoderm
Neural
Neural Crest

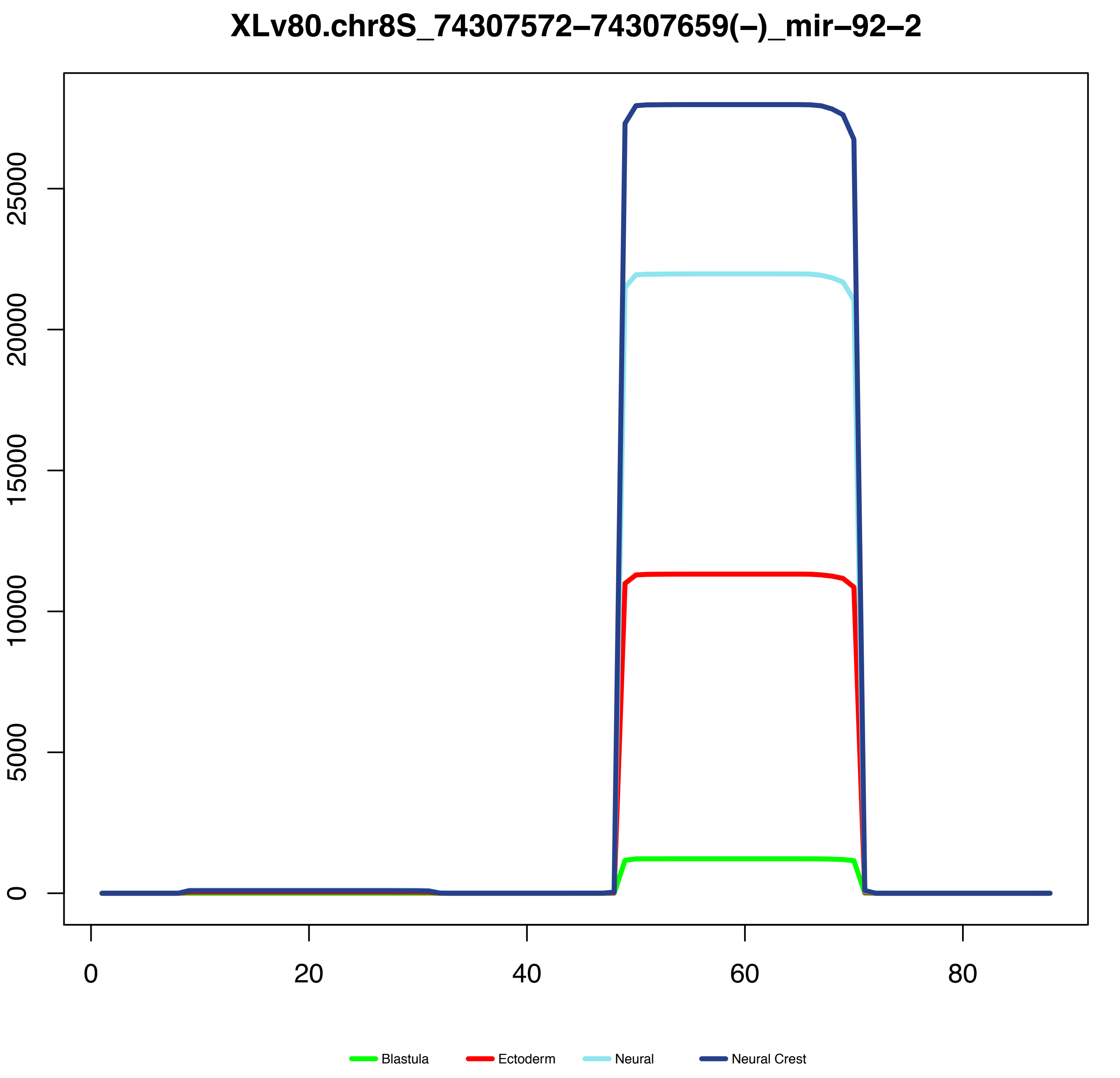

XLv80.chr8S_74307572−74307659(−)_mir−92−2
0
5000
10000
15000
20000
25000
0
20
40
60
80
Blastula
Ectoderm
Neural
Neural Crest

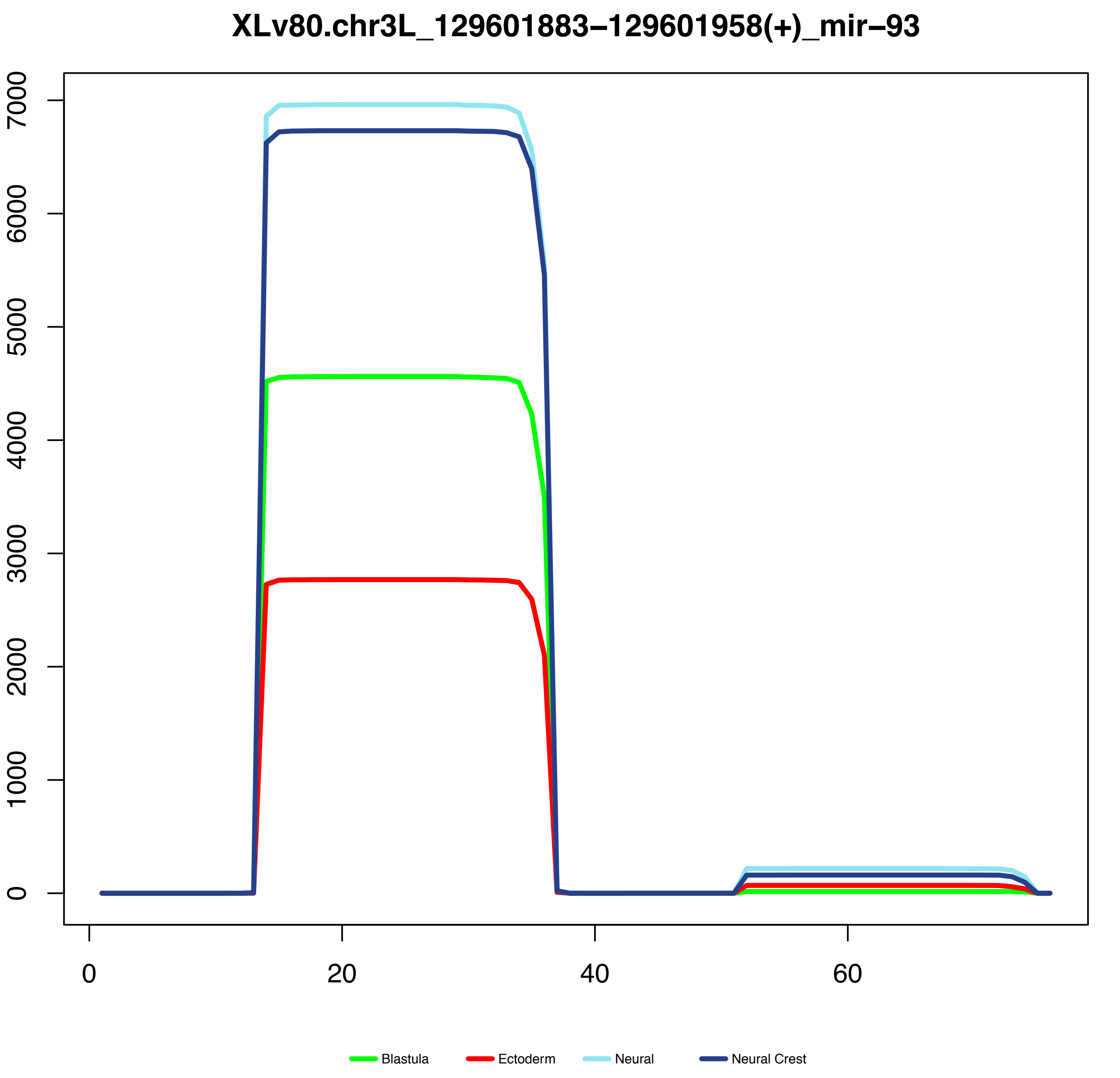
XLv80.chr3L_129601883−129601958(+)_mir−93
0
1000
2000
3000
4000
5000
6000
7000
0
20
40
60
Blastula
Ectoderm
Neural
Neural Crest

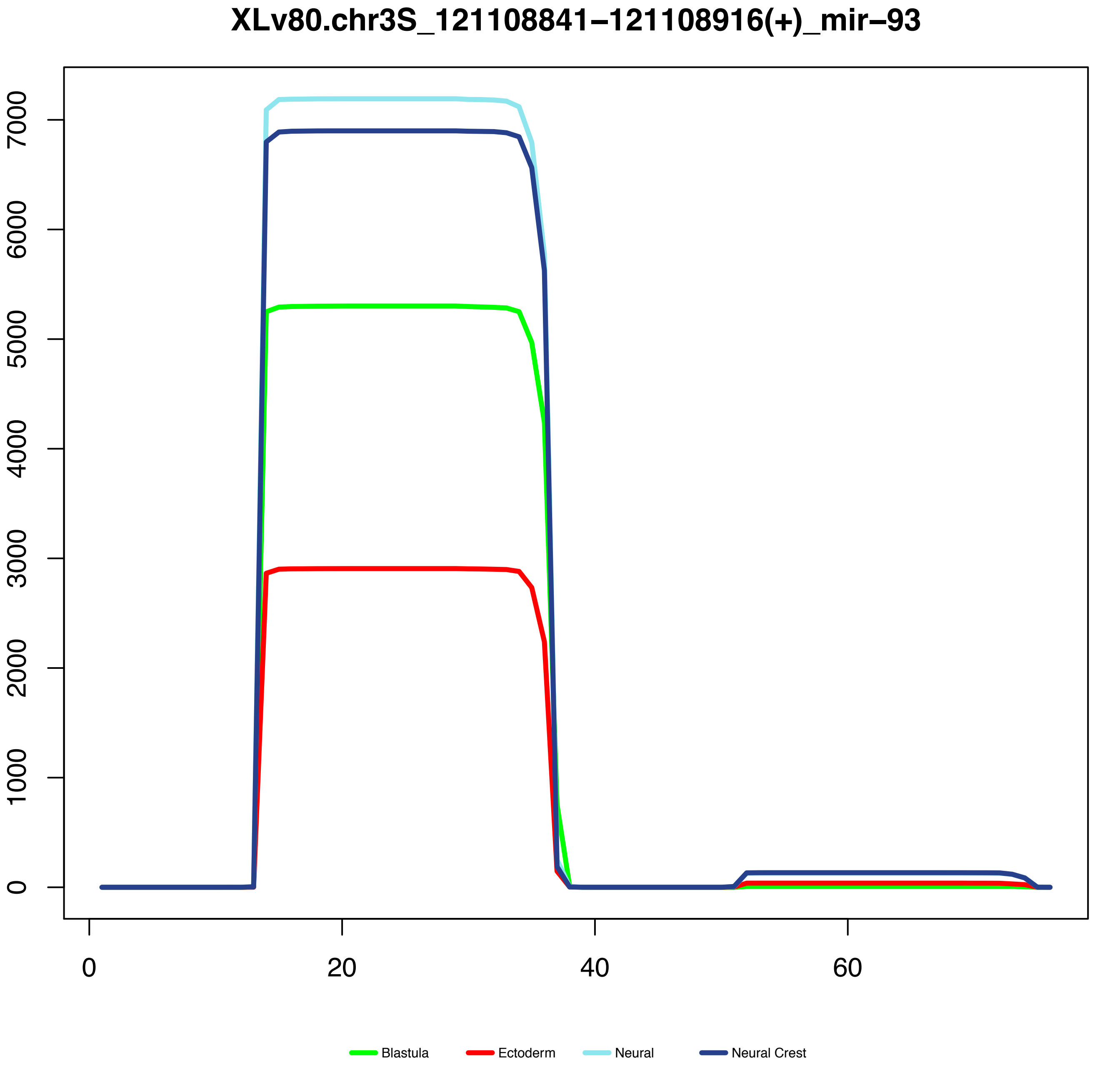
XLv80.chr3S_121108841−121108916(+)_mir−93
0
1000
2000
3000
4000
5000
6000
7000
0
20
40
60
Blastula
Ectoderm
Neural
Neural Crest

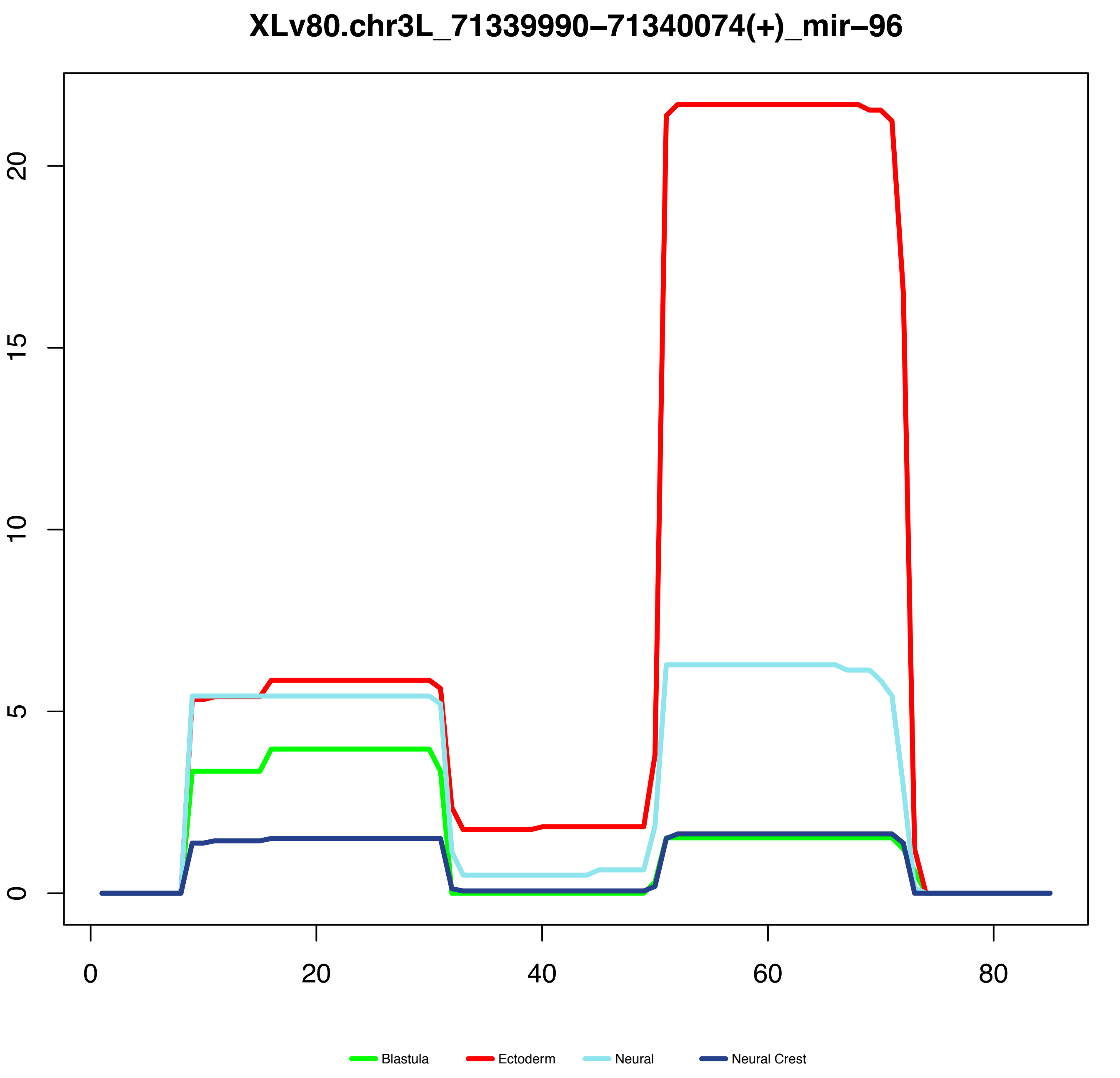
XLv80.chr3L_71339990−71340074(+)_mir−96
0
5
10
15
20
0
20
40
60
80
Blastula
Ectoderm
Neural
Neural Crest

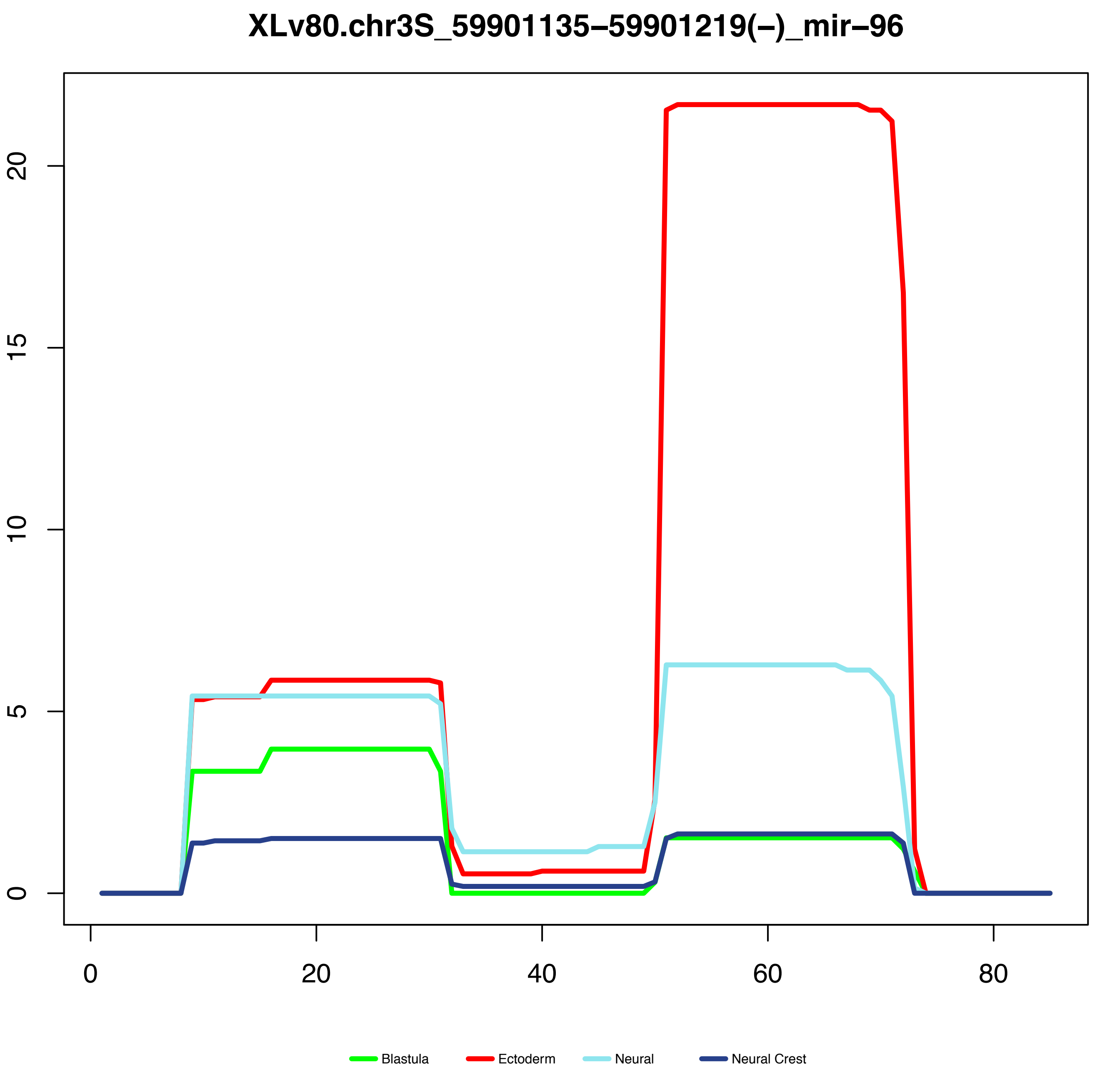
XLv80.chr3S_59901135–59901219(–)_mir–96
0
5
10
15
20
0
20
40
60
80
Blastula
Ectoderm
Neural
Neural Crest

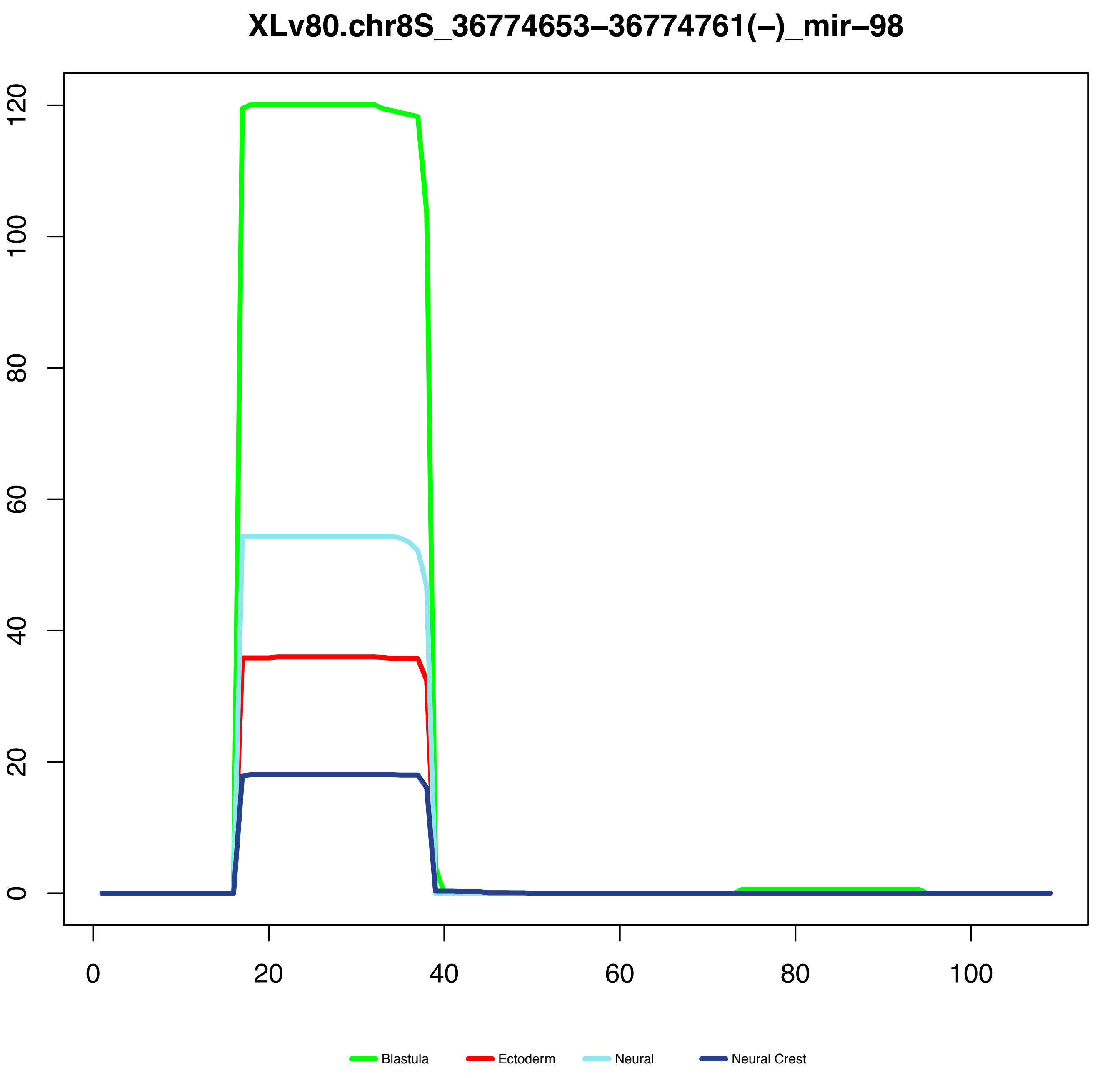
XLv80.chr8S_36774653–36774761(–)_mir–98
0
20
40
60
80
100
120
0
20
40
60
80
100
Blastula
Ectoderm
Neural
Neural Crest

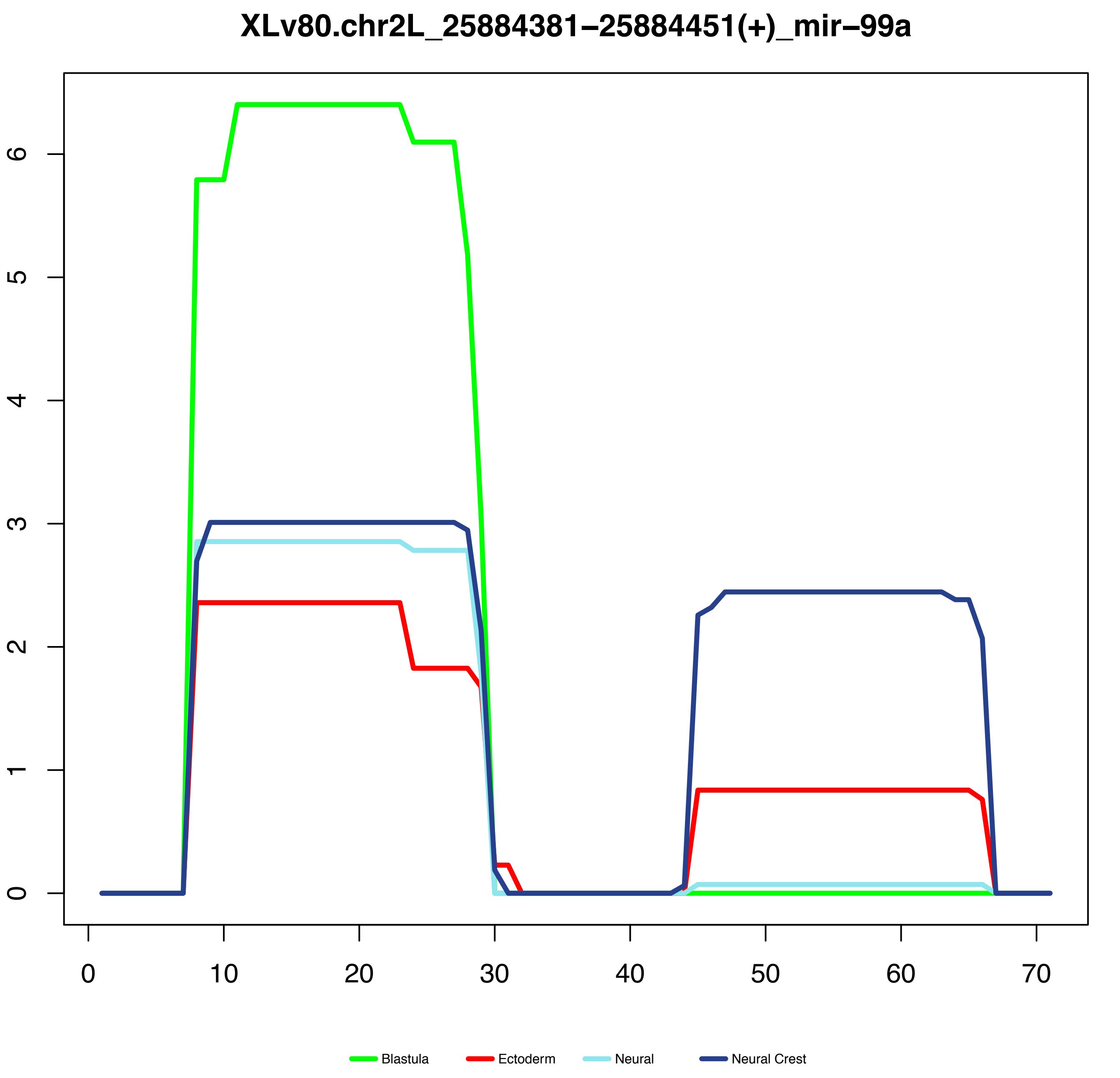
XLv80.chr2L_25884381−25884451(+)_mir−99a
0
1
2
3
4
5
6
0
10
20
30
40
50
60
70
Blastula
Ectoderm
Neural
Neural Crest

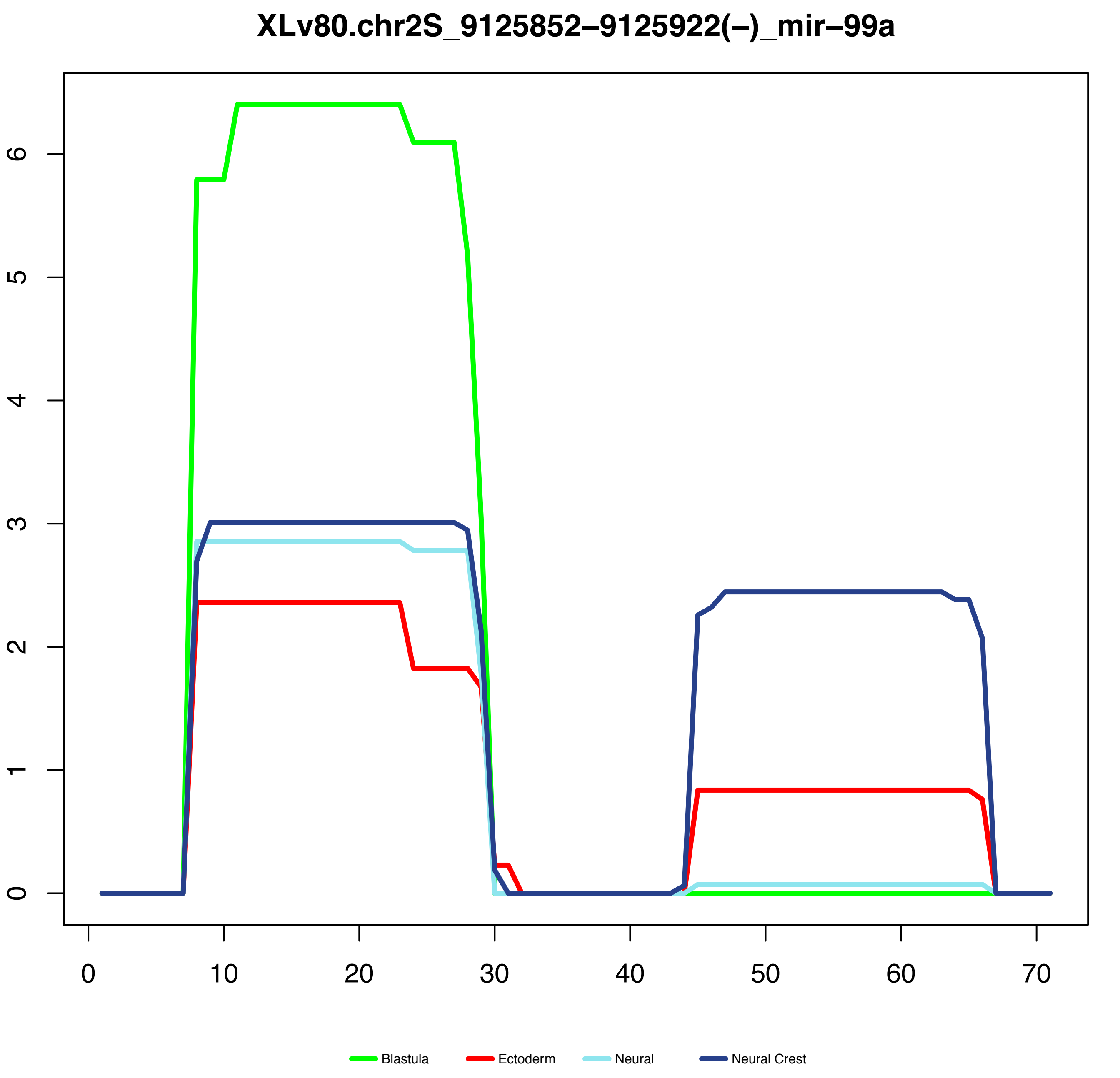

XLv80.chr2S_9125852−9125922(−)_mir−99a
0
1
2
3
4
5
6
0
10
20
30
40
50
60
70
Blastula
Ectoderm
Neural
Neural Crest

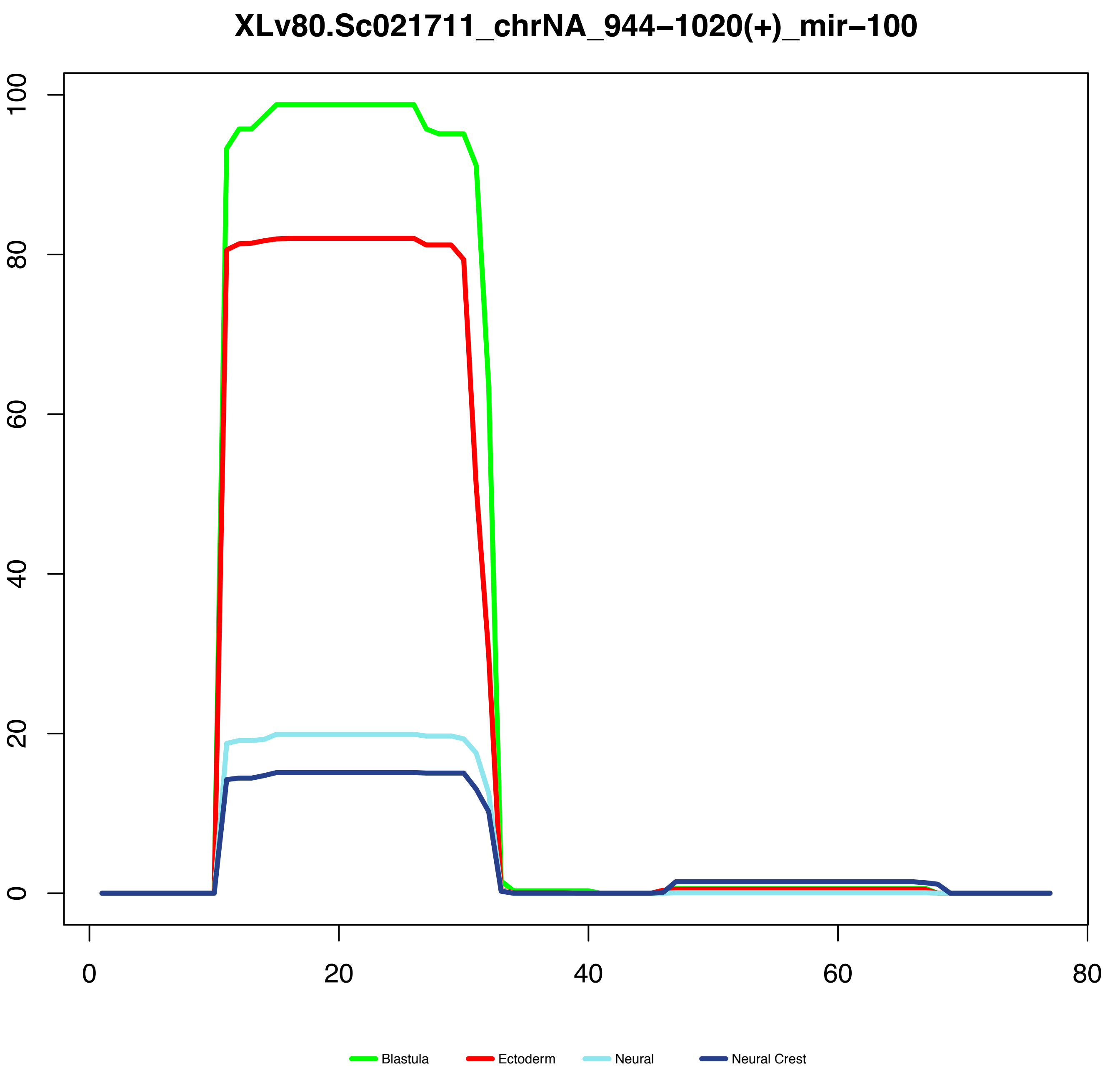
XLv80.Sc021711_chrNA_944−1020(+)_mir−100
0
20
40
60
80
100
0
20
40
60
80
Blastula
Ectoderm
Neural
Neural Crest

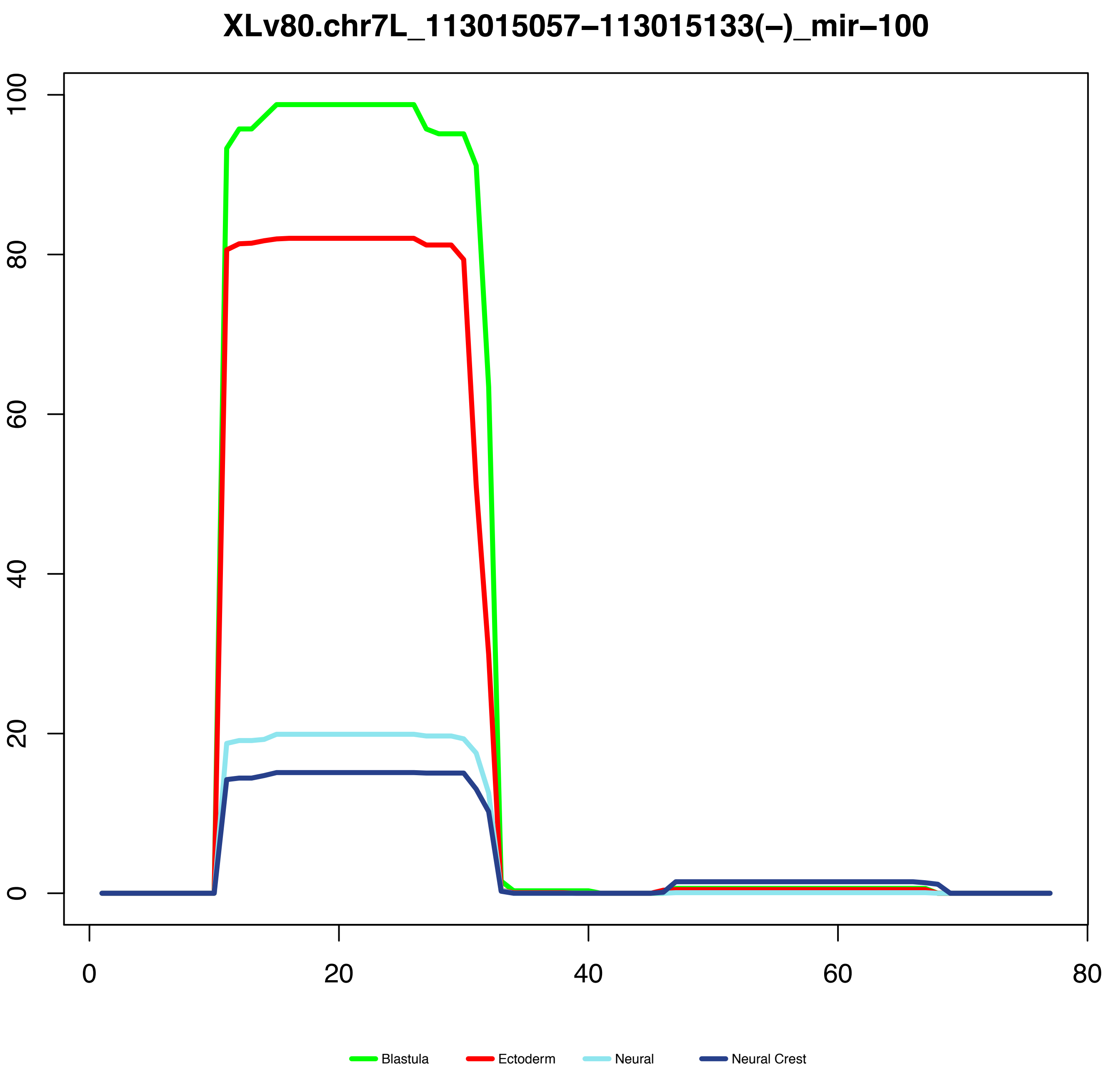
XLv80.chr7L_113015057−113015133(−)_mir−100
0
20
40
60
80
100
0
20
40
60
80
Blastula
Ectoderm
Neural
Neural Crest

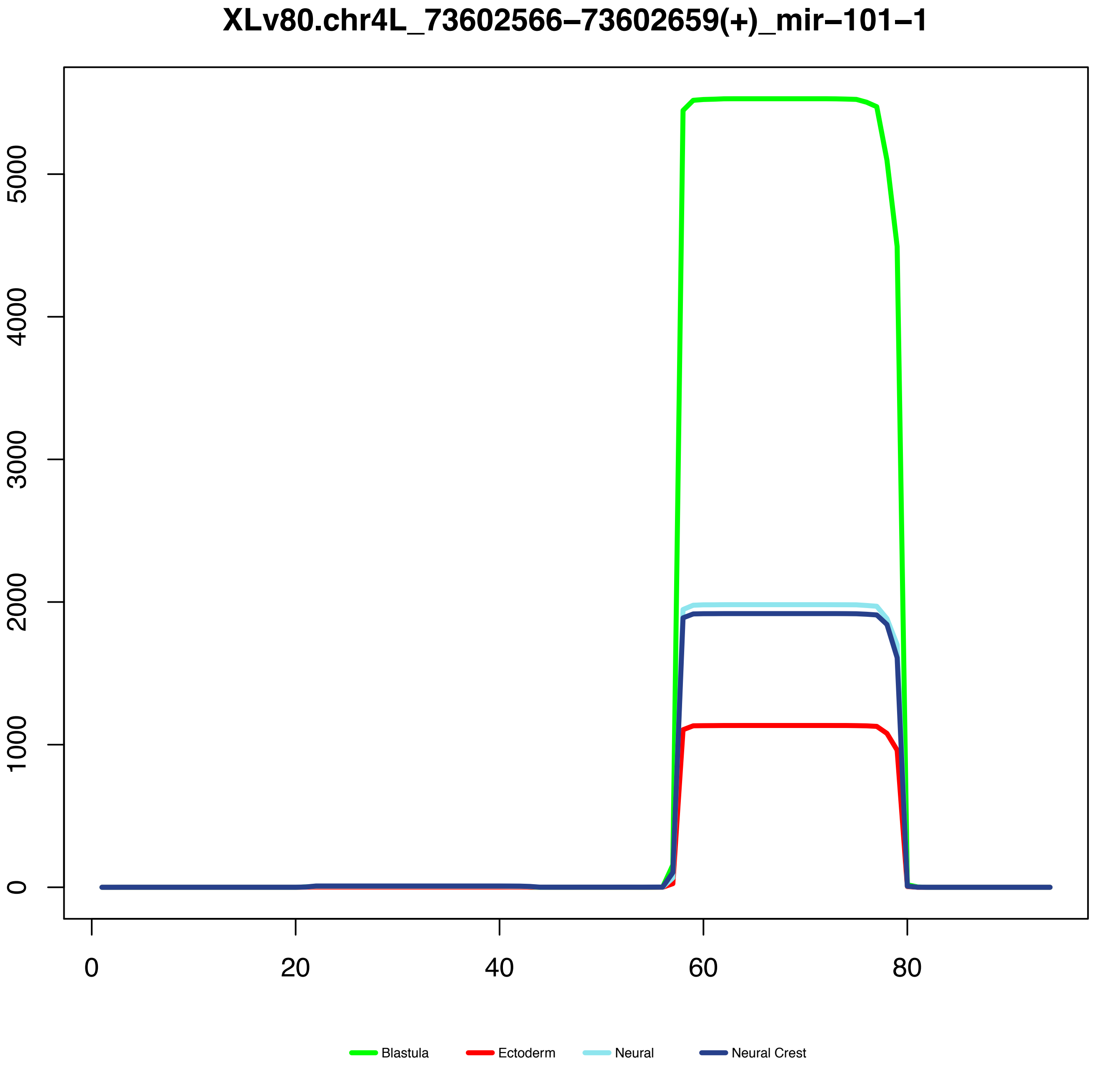

XLv80.chr4L_73602566−73602659(+)_mir−101−1
0
1000
2000
3000
4000
5000
0
20
40
60
80
Blastula
Ectoderm
Neural
Neural Crest

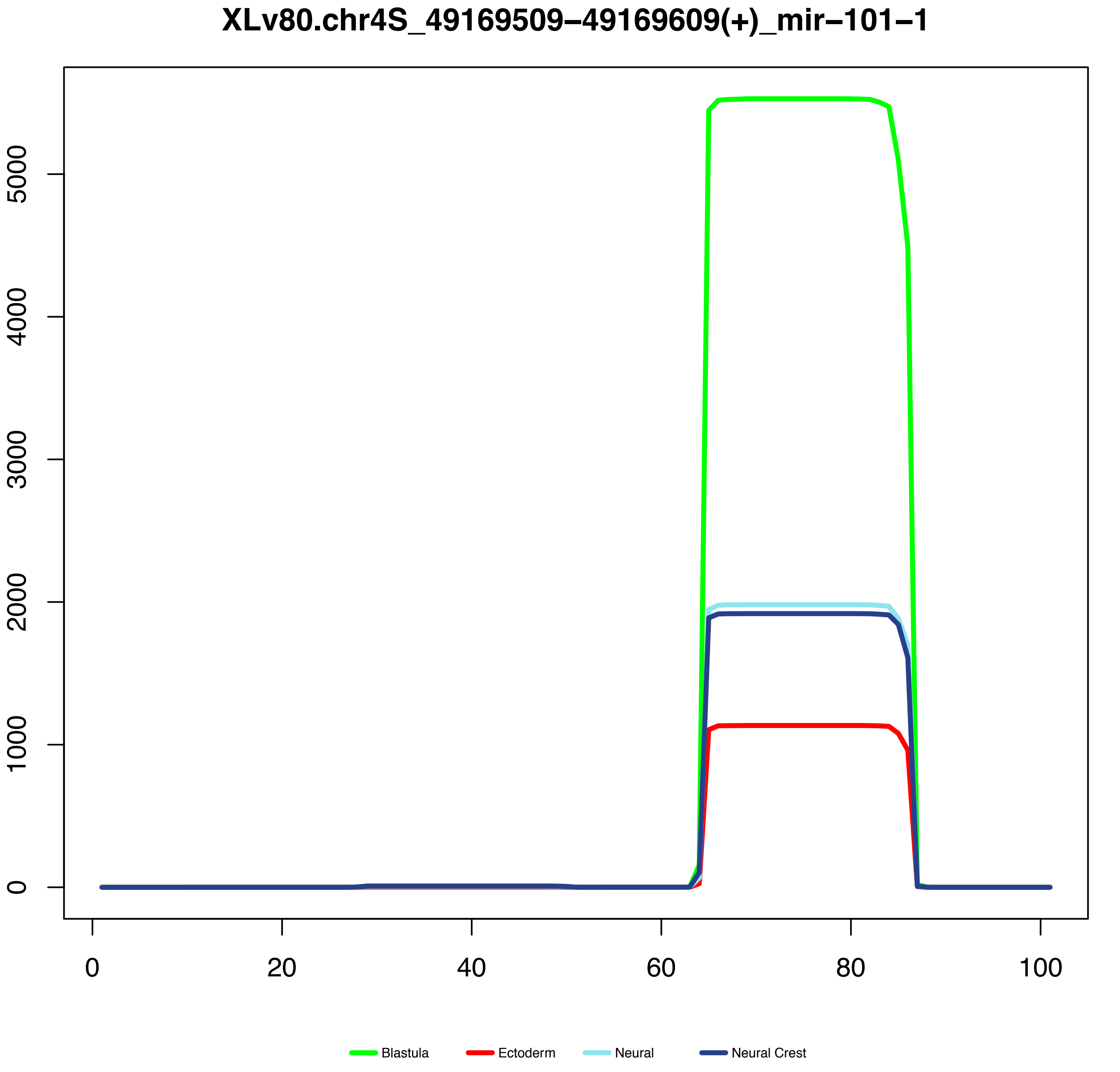
XLv80.chr4S_49169509−49169609(+)_mir−101−1
0
1000
2000
3000
4000
5000
0
20
40
60
80
100
Blastula
Ectoderm
Neural
Neural Crest

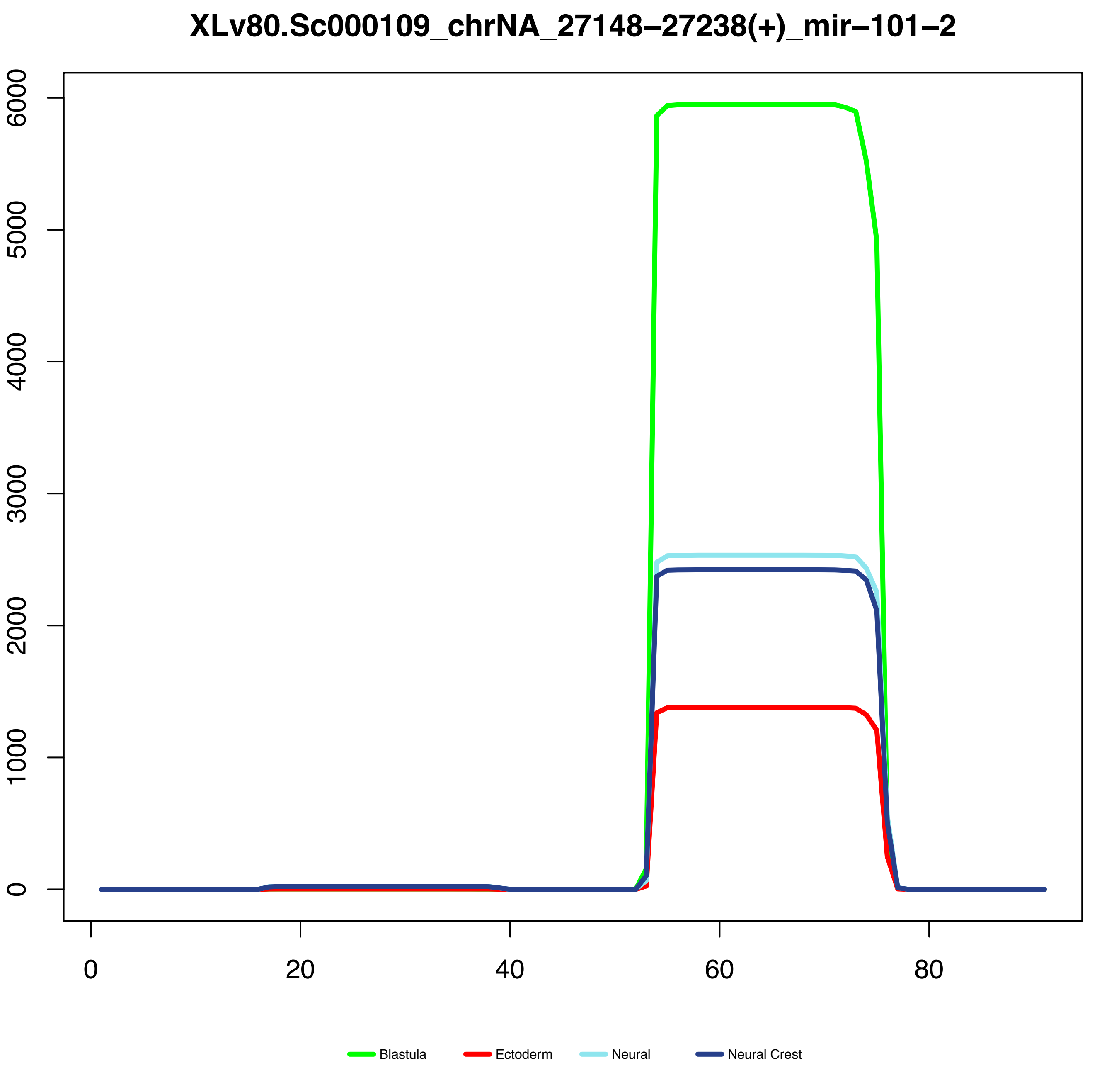
XLv80.Sc000109_chrNA_27148−27238(+)_mir−101−2
0
1000
2000
3000
4000
5000
6000
0
20
40
60
80
Blastula
Ectoderm
Neural
Neural Crest

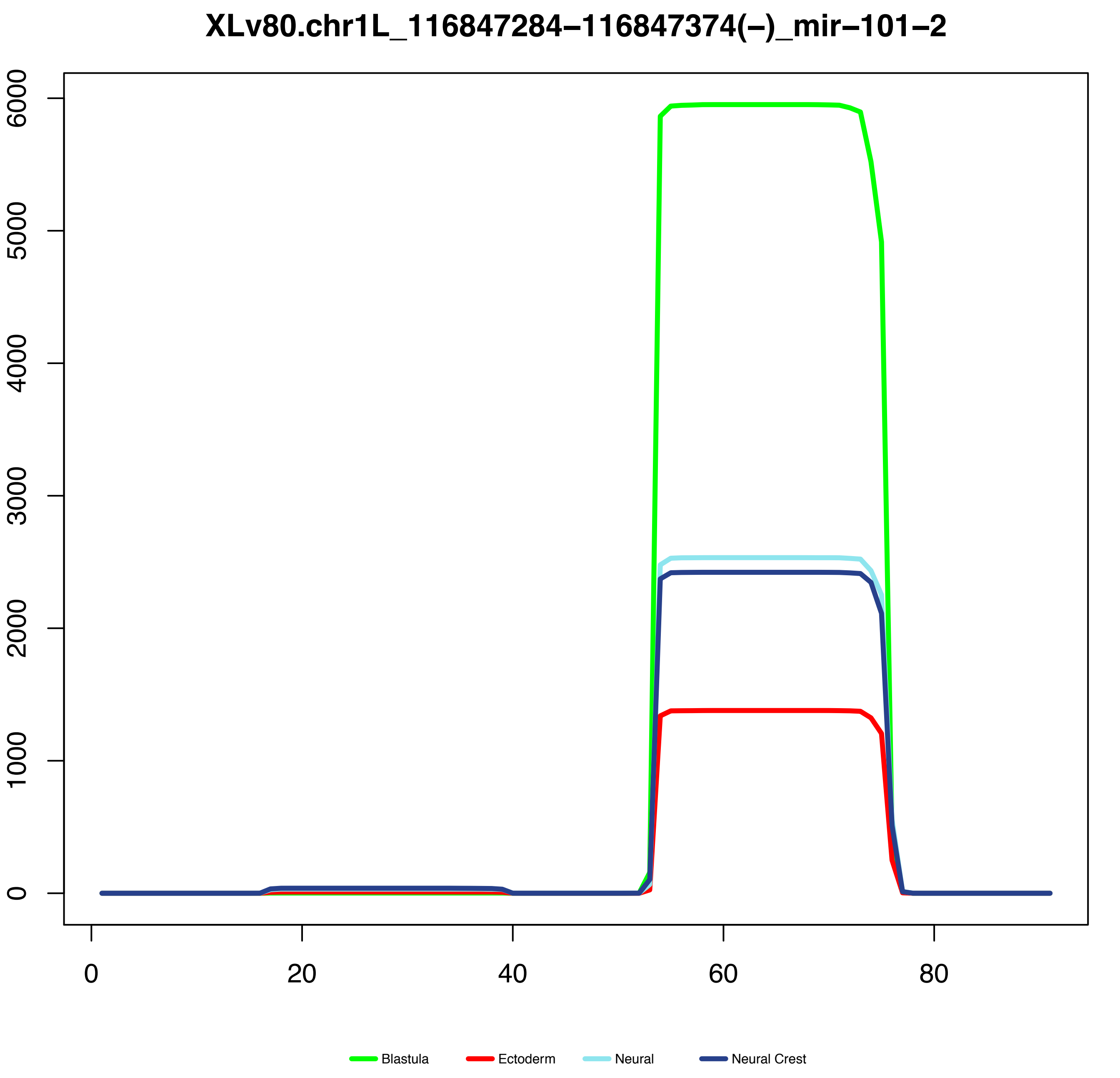
XLv80.chr1L_116847284−116847374(−)_mir−101−2
0
1000
2000
3000
4000
5000
6000
0
20
40
60
80
Blastula
Ectoderm
Neural
Neural Crest

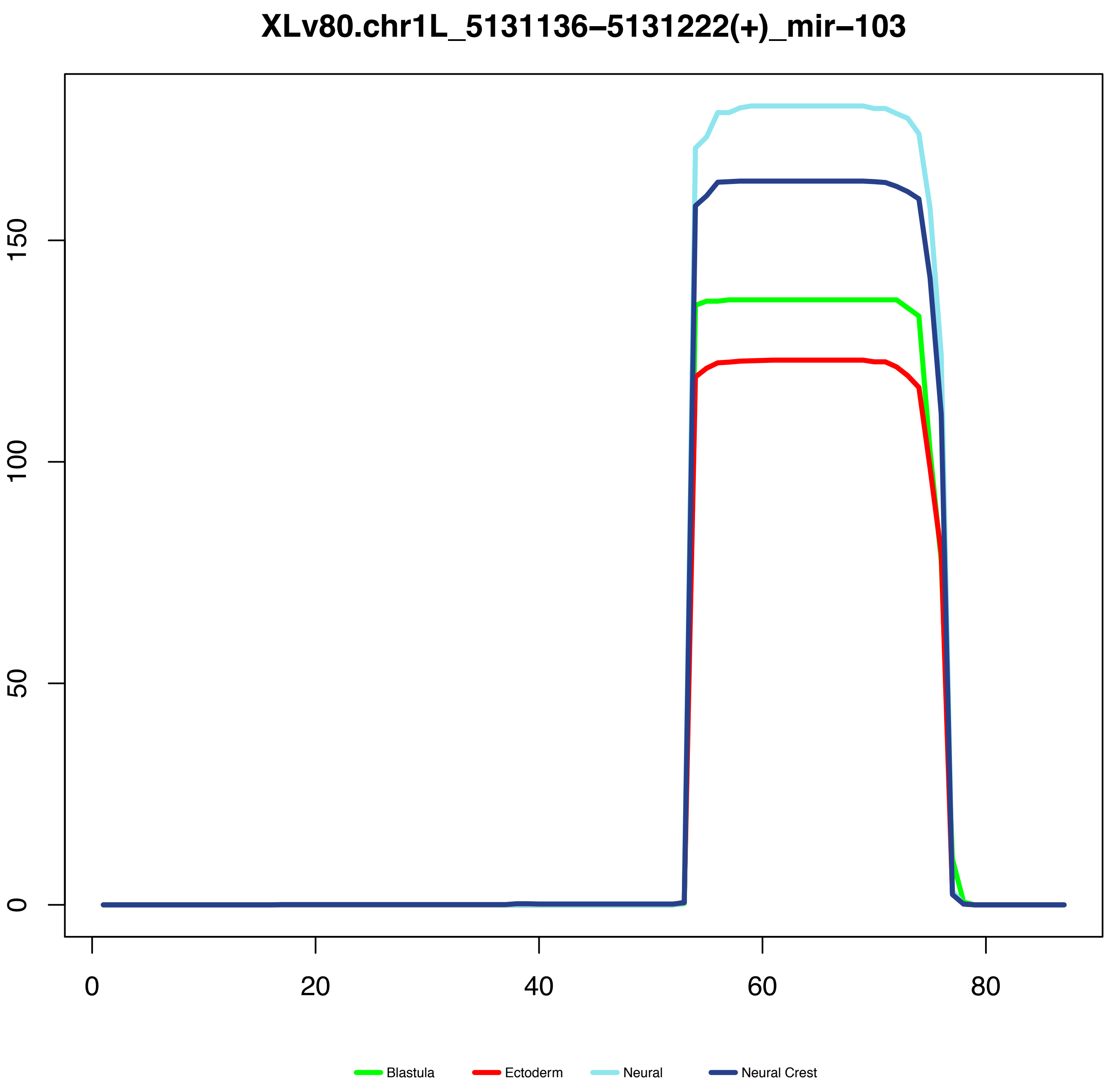

XLv80.chr1L_5131136−5131222(+)_mir−103
0
50
100
150
0
20
40
60
80
Blastula
Ectoderm
Neural
Neural Crest

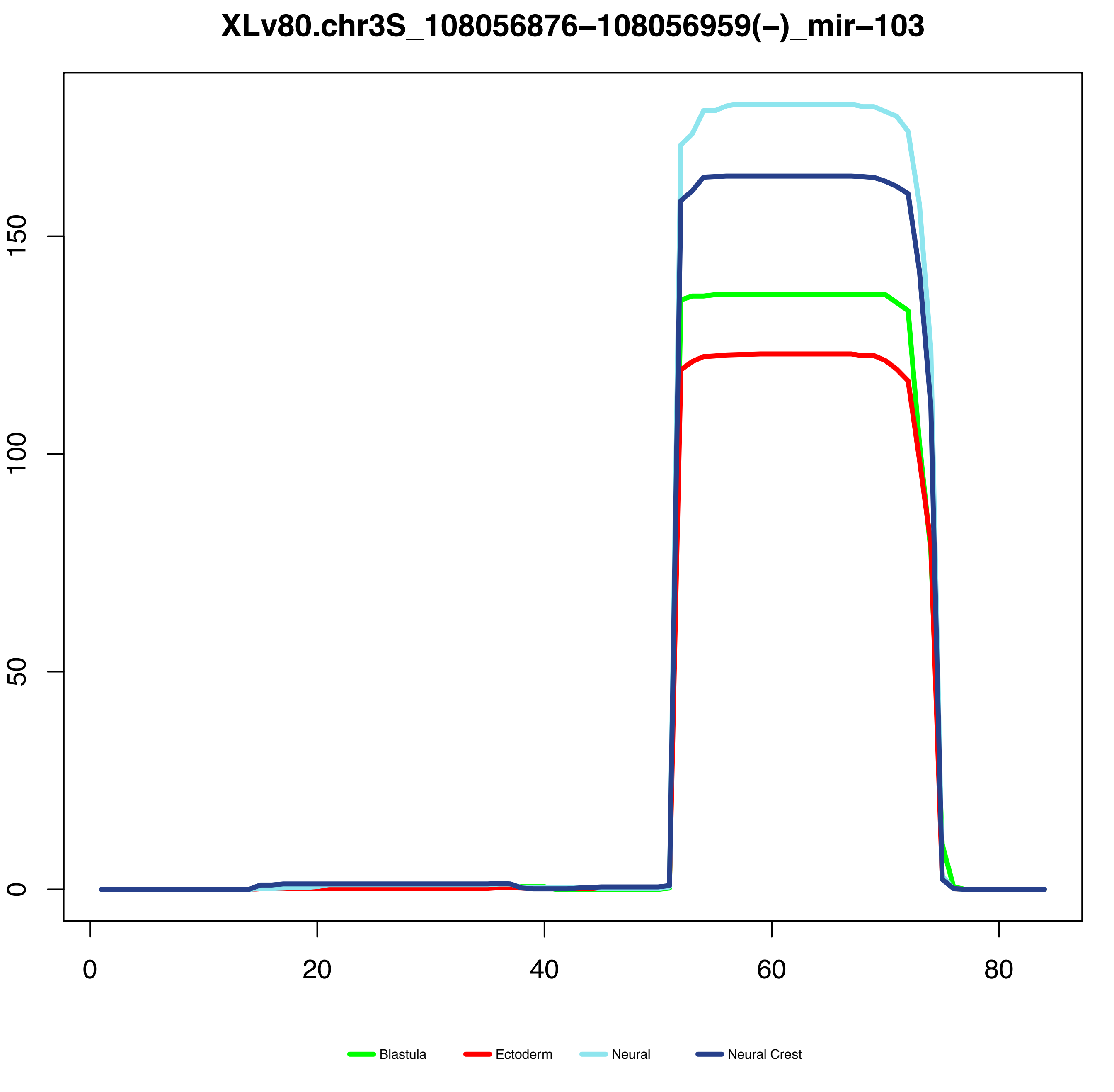
XLv80.chr3S_108056876−108056959(−)_mir−103
0
50
100
150
0
20
40
60
80
Blastula
Ectoderm
Neural
Neural Crest

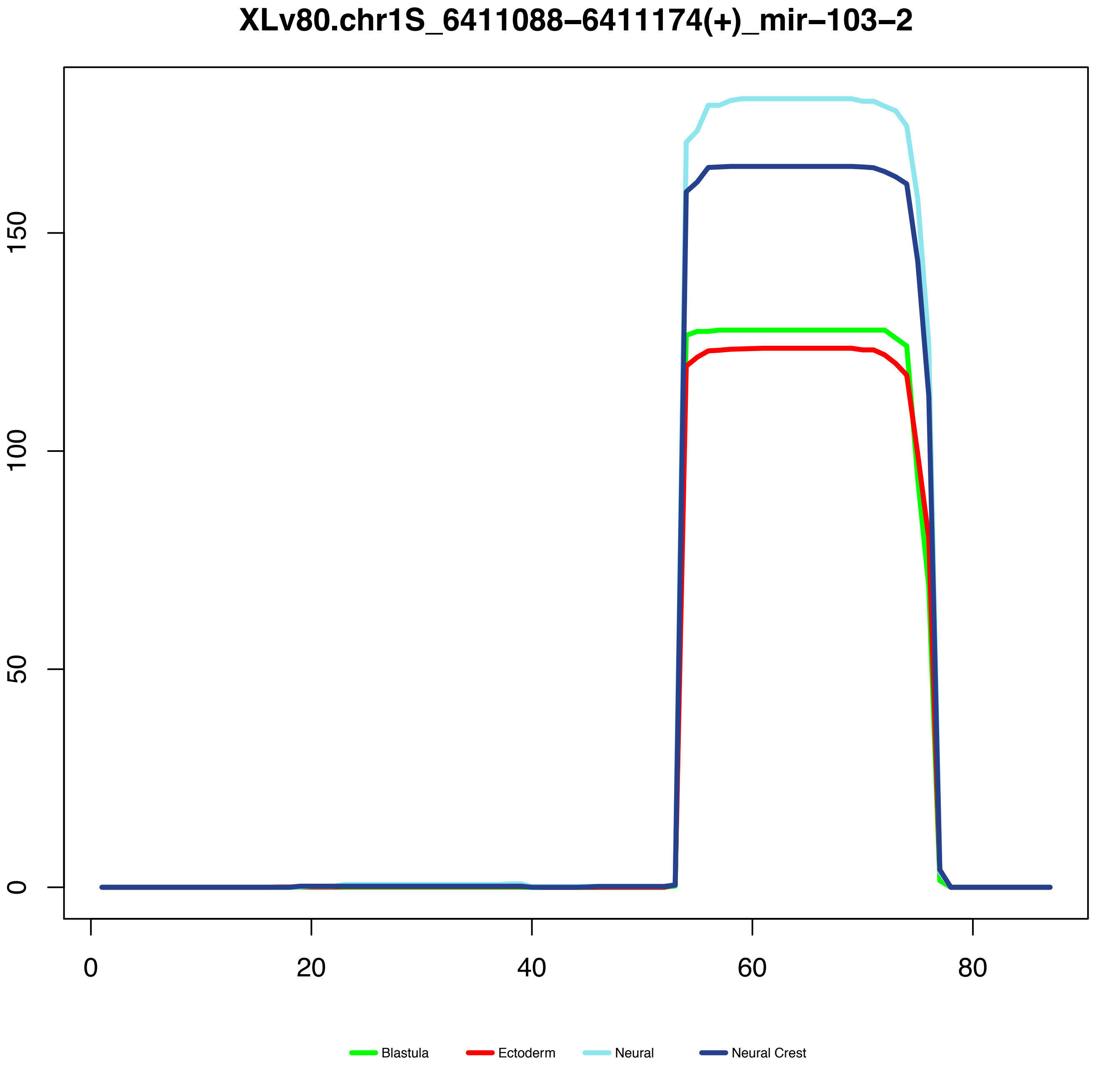
XLv80.chr1S_6411088−6411174(+)_mir−103−2
0
50
100
150
0
20
40
60
80
Blastula
Ectoderm
Neural
Neural Crest

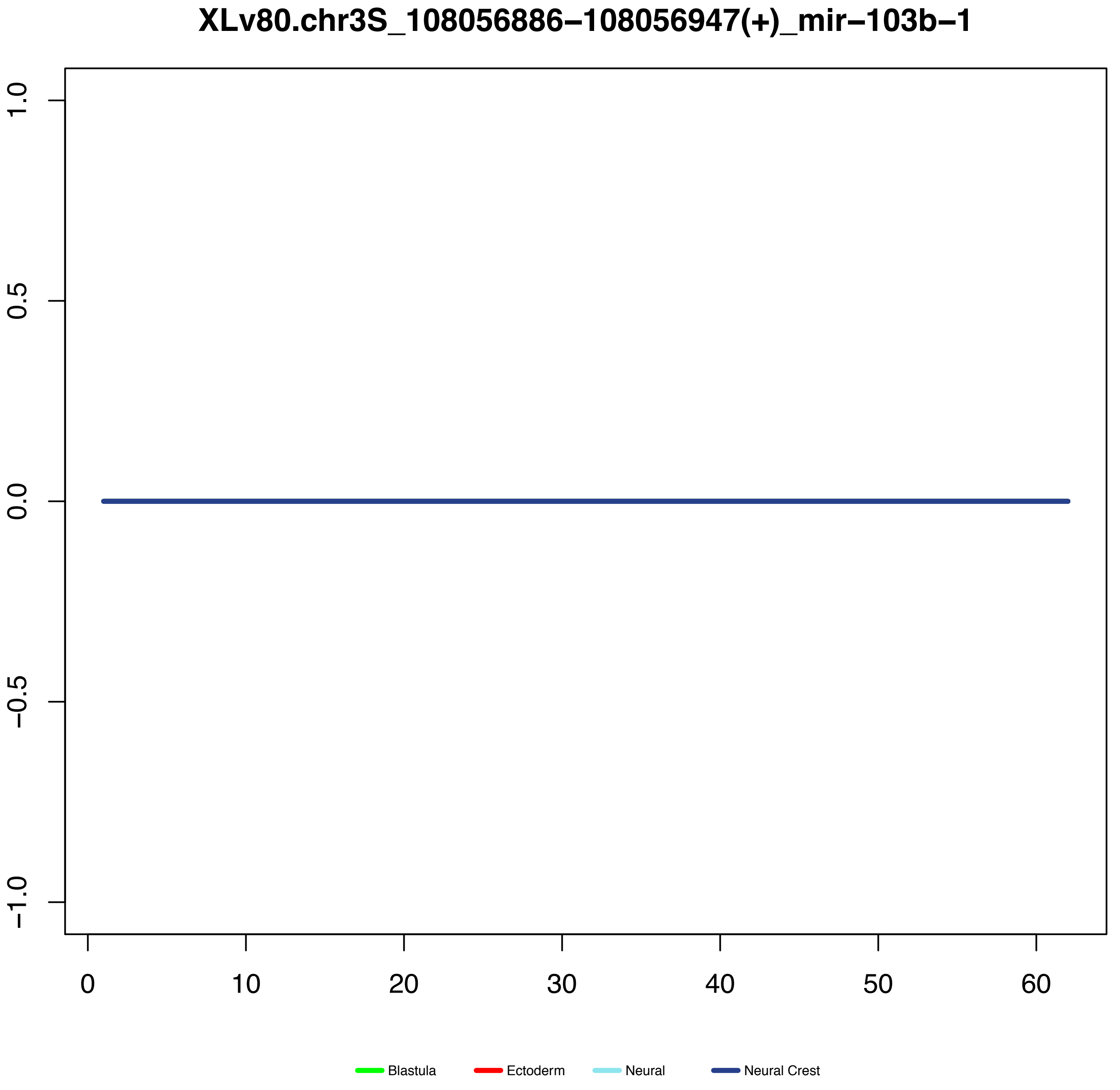
XLv80.chr3S_108056886−108056947(+)_mir−103b−1
1.0
0.5
0.0
−0.5
−1.0
0
10
20
30
40
50
60
Blastula
Ectoderm
Neural
Neural Crest

XLv80.chr8L_48822346−48822408(−)_mir−106

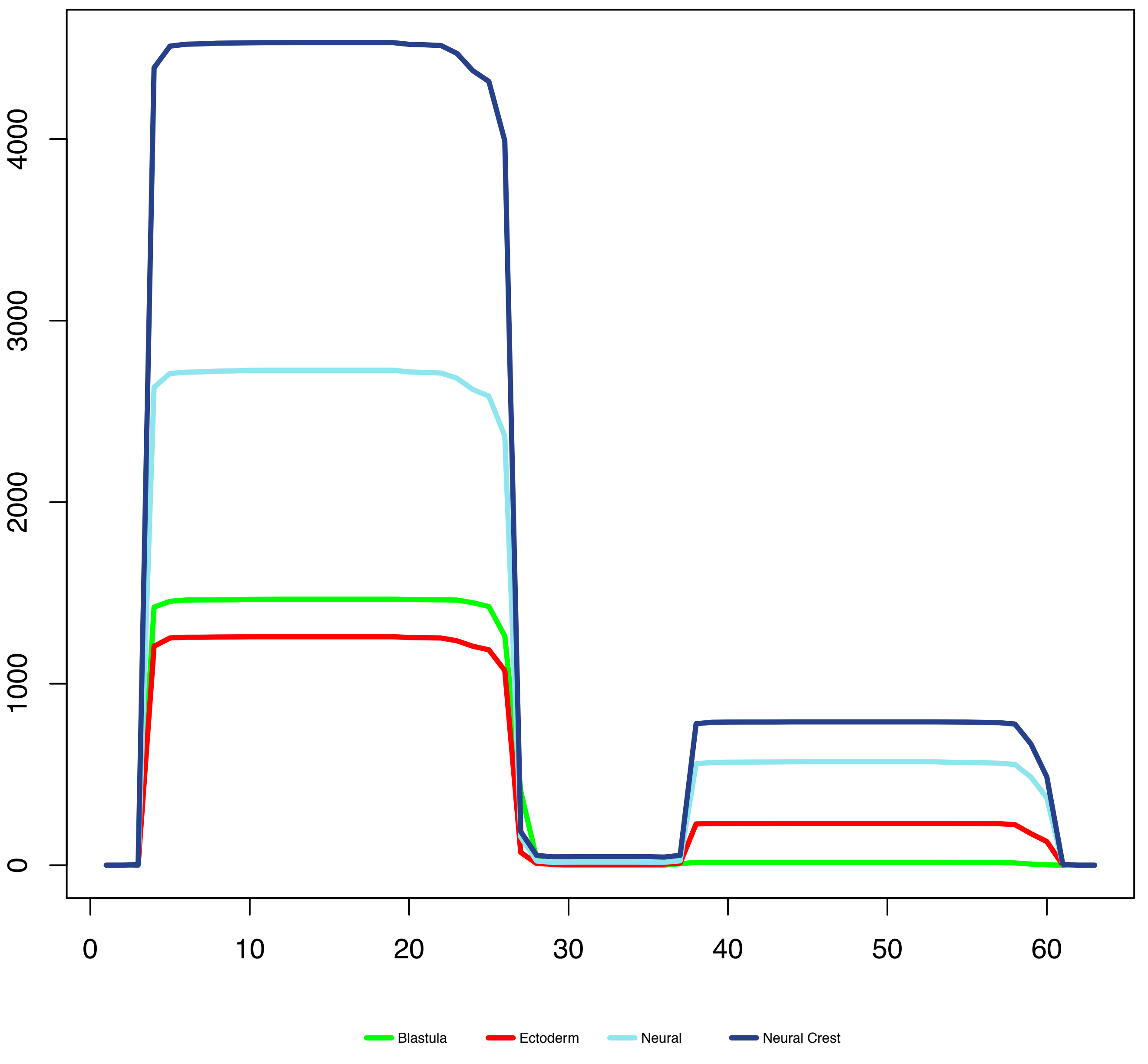

XLv80.chr7L_31729193–31729287(–)_mir–107

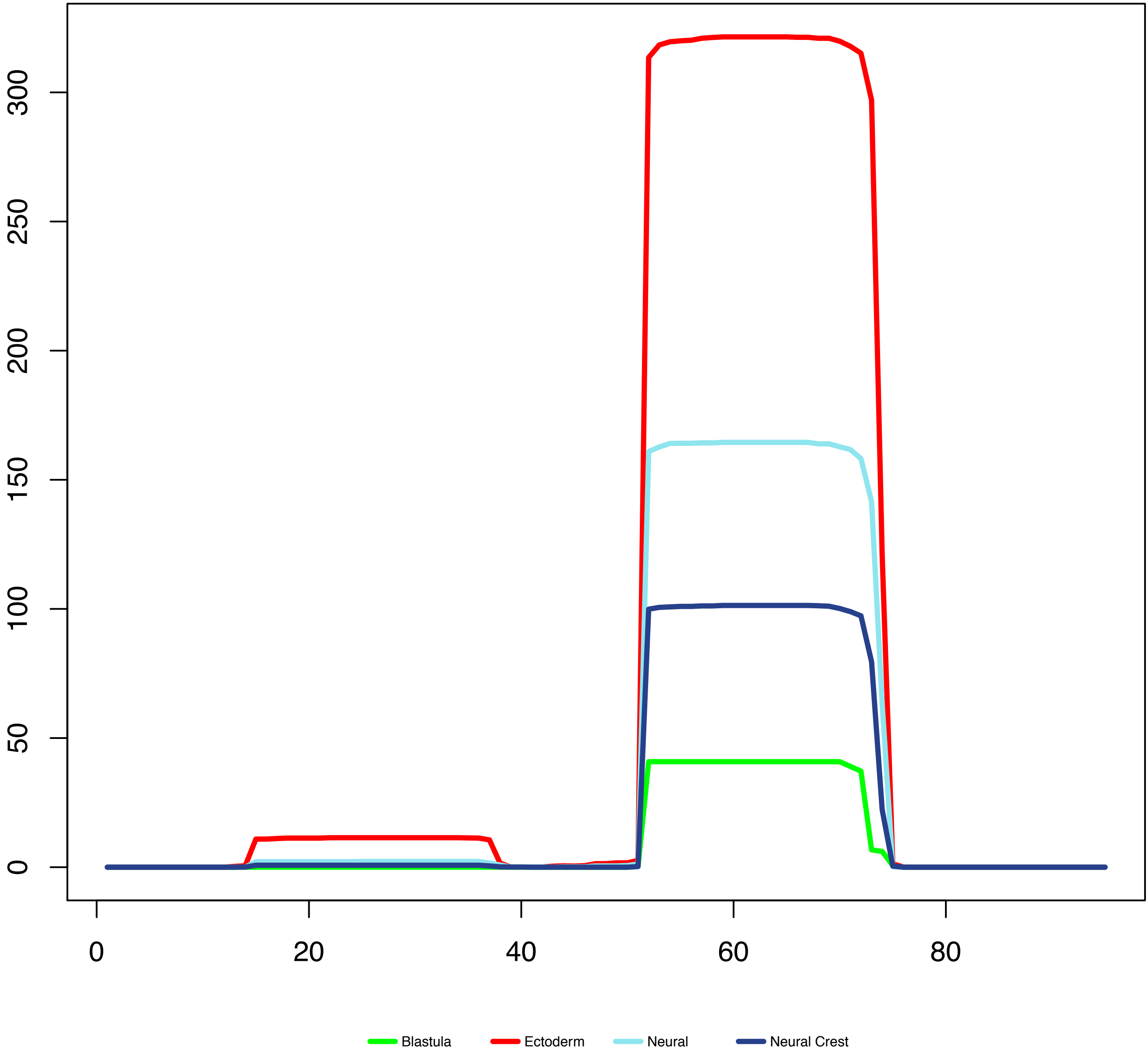

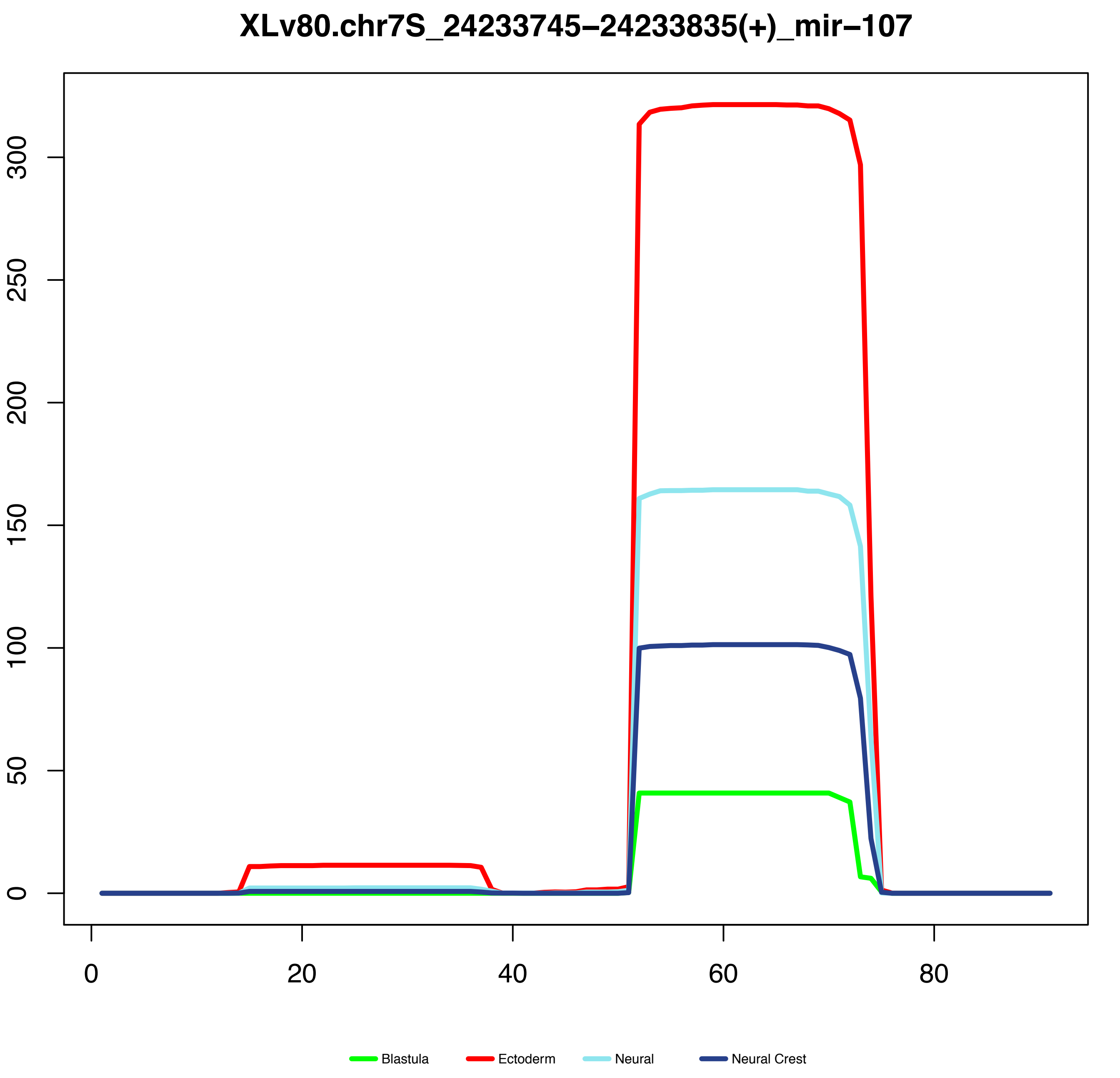
XLv80.chr7S_24233745−24233835(+)_mir−107
0
50
100
150
200
250
300
0
20
40
60
80
Blastula
Ectoderm
Neural
Neural Crest

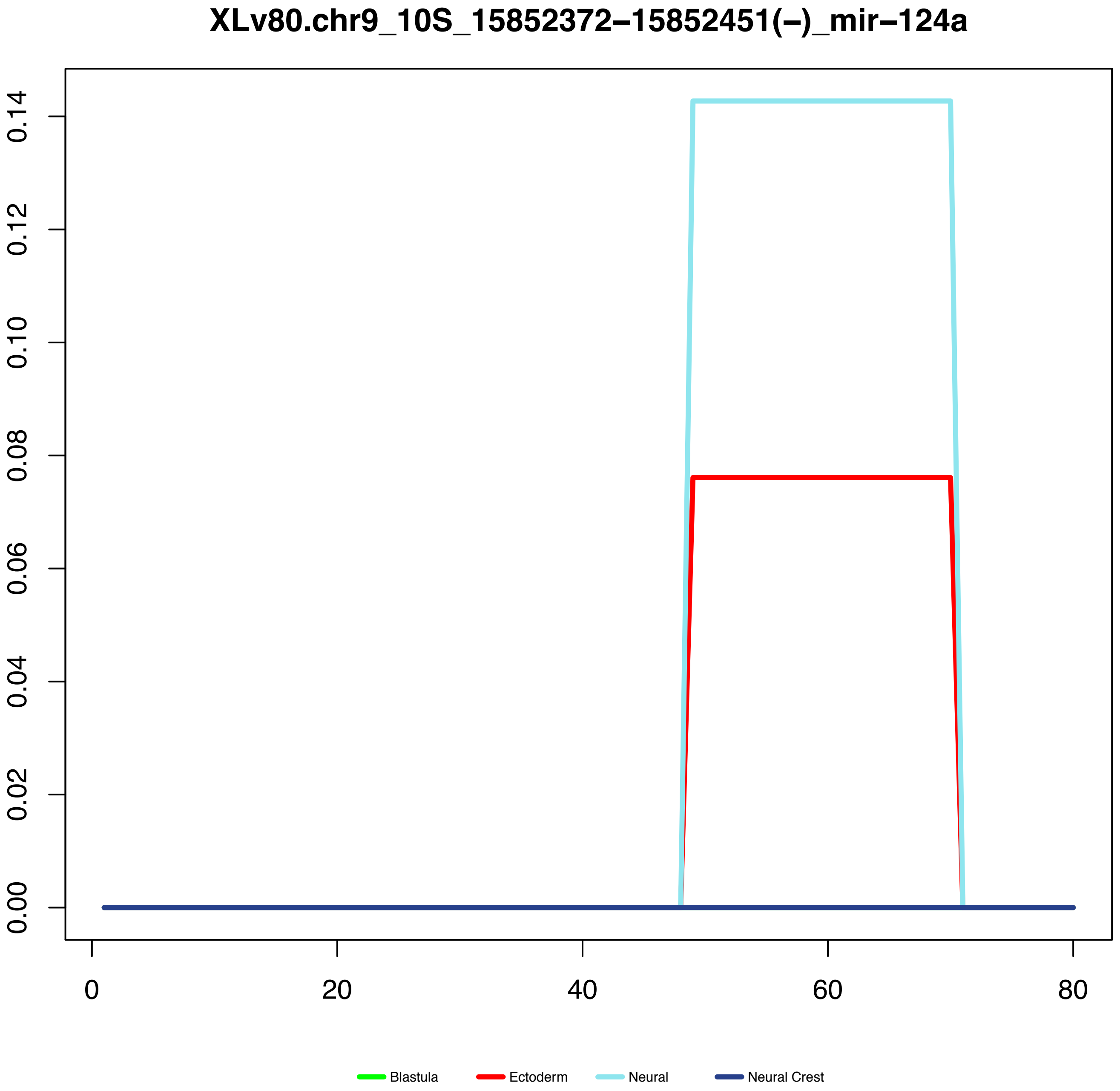
XLv80.chr9_10S_15852372–15852451(–)_mir–124a
0.00
0.02
0.04
0.06
0.08
0.10
0.12
0.14
0
20
40
60
80
Blastula
Ectoderm
Neural
Neural Crest

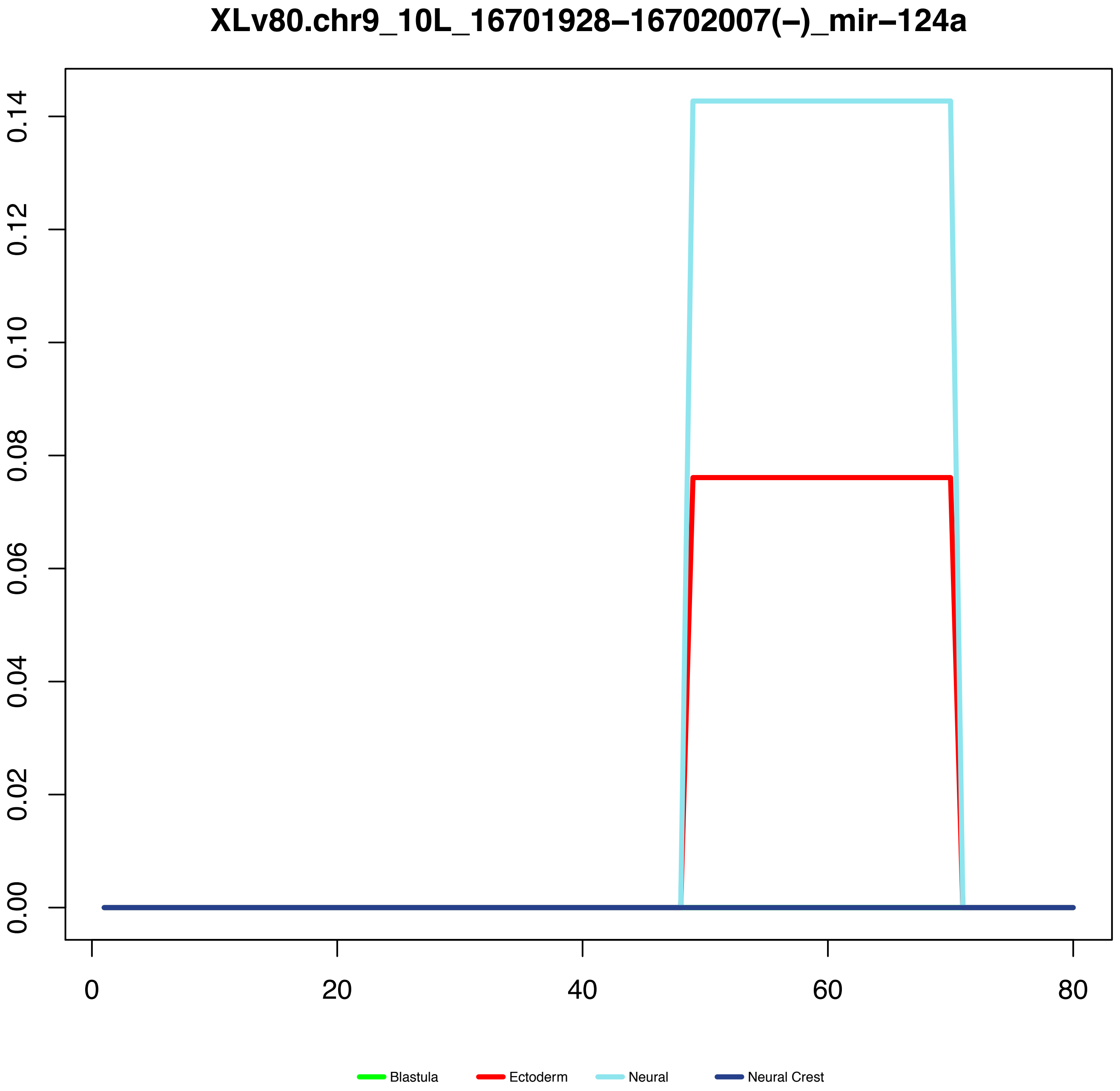
XLv80.chr9_10L_16701928−16702007(−)_mir−124a
0.00
0.02
0.04
0.06
0.08
0.10
0.12
0.14
0
20
40
60
80
Blastula
Ectoderm
Neural
Neural Crest

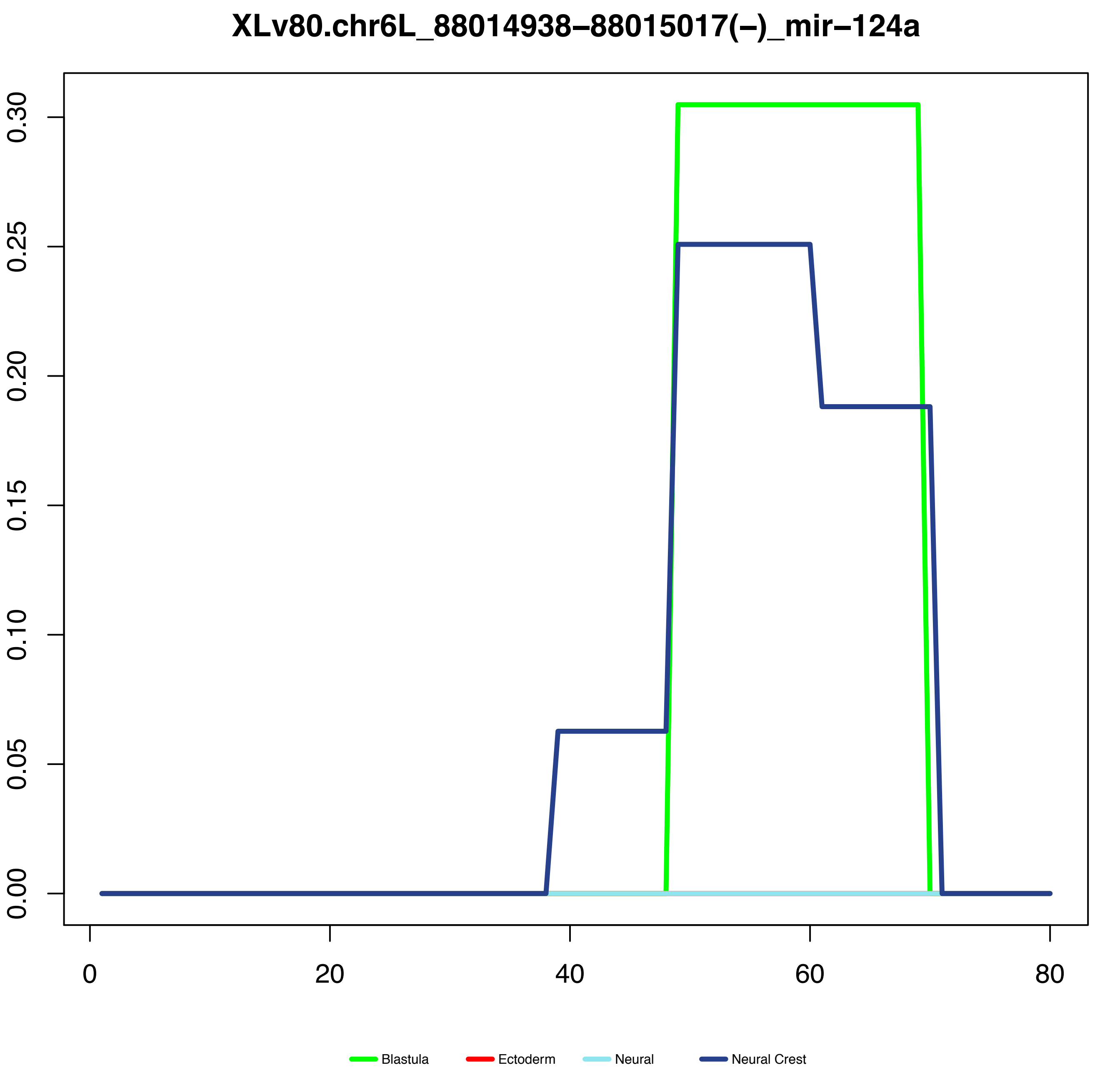
XLv80.chr6L_88014938−88015017(−)_mir−124a
0.00
0.05
0.10
0.15
0.20
0.25
0.30
0
20
40
60
80
Blastula
Ectoderm
Neural
Neural Crest

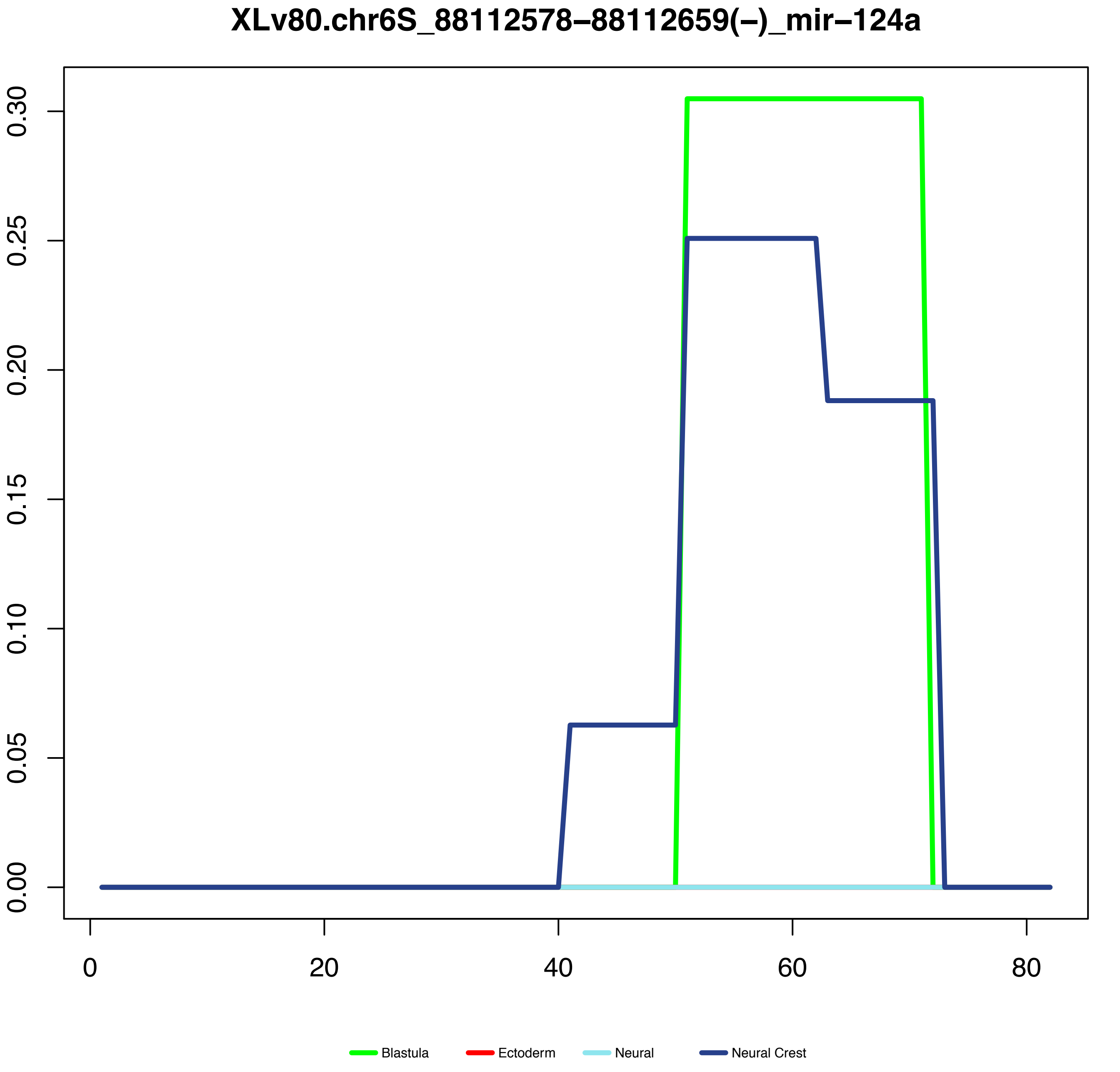
XLv80.chr6S_88112578−88112659(−)_mir−124a
0.00
0.05
0.10
0.15
0.20
0.25
0.30
0
20
40
60
80
Blastula
Ectoderm
Neural
Neural Crest

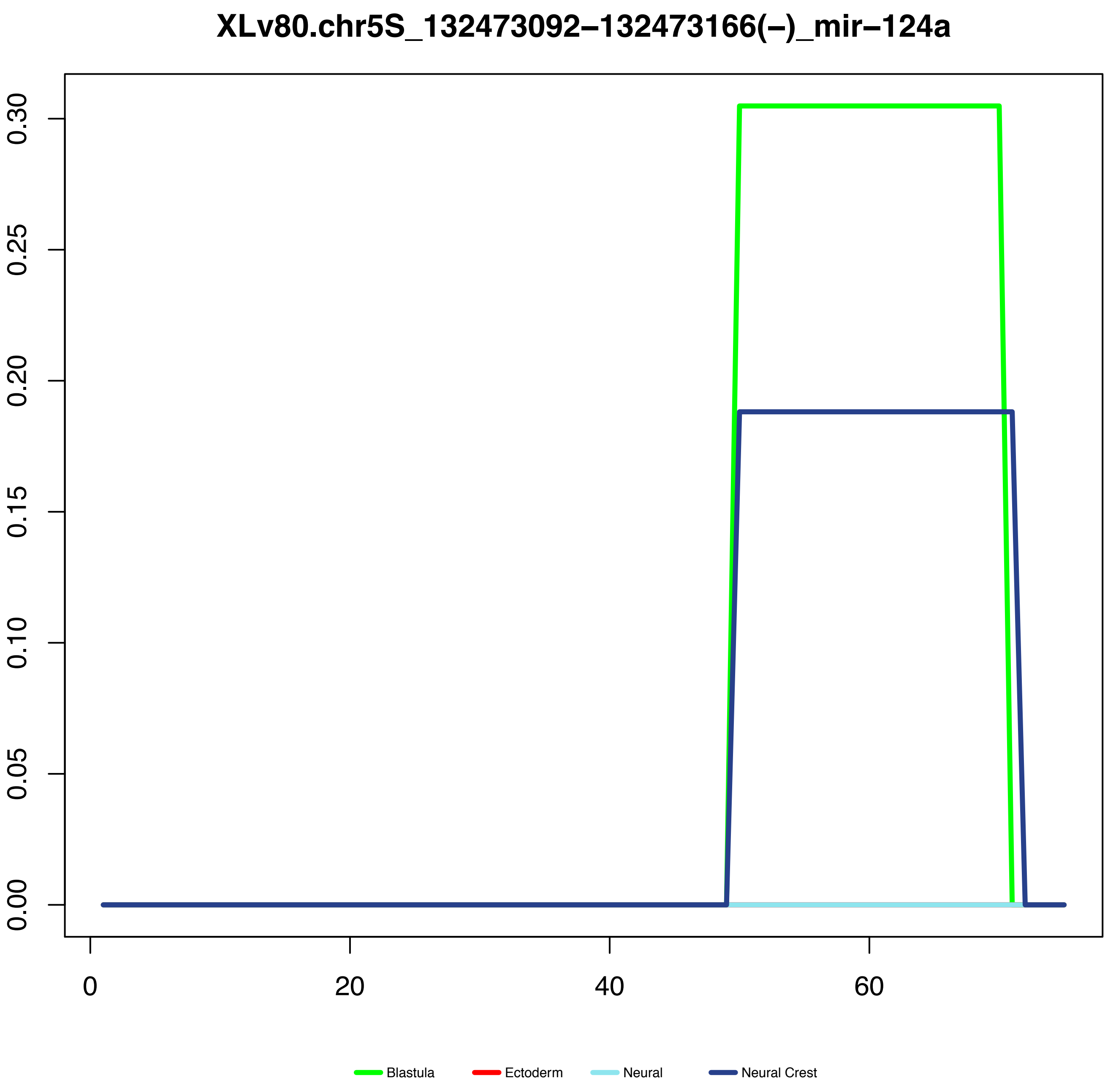
XLv80.chr5S_132473092−132473166(−)_mir−124a
0.00
0.05
0.10
0.15
0.20
0.25
0.30
0
20
40
60
Blastula
Ectoderm
Neural
Neural Crest

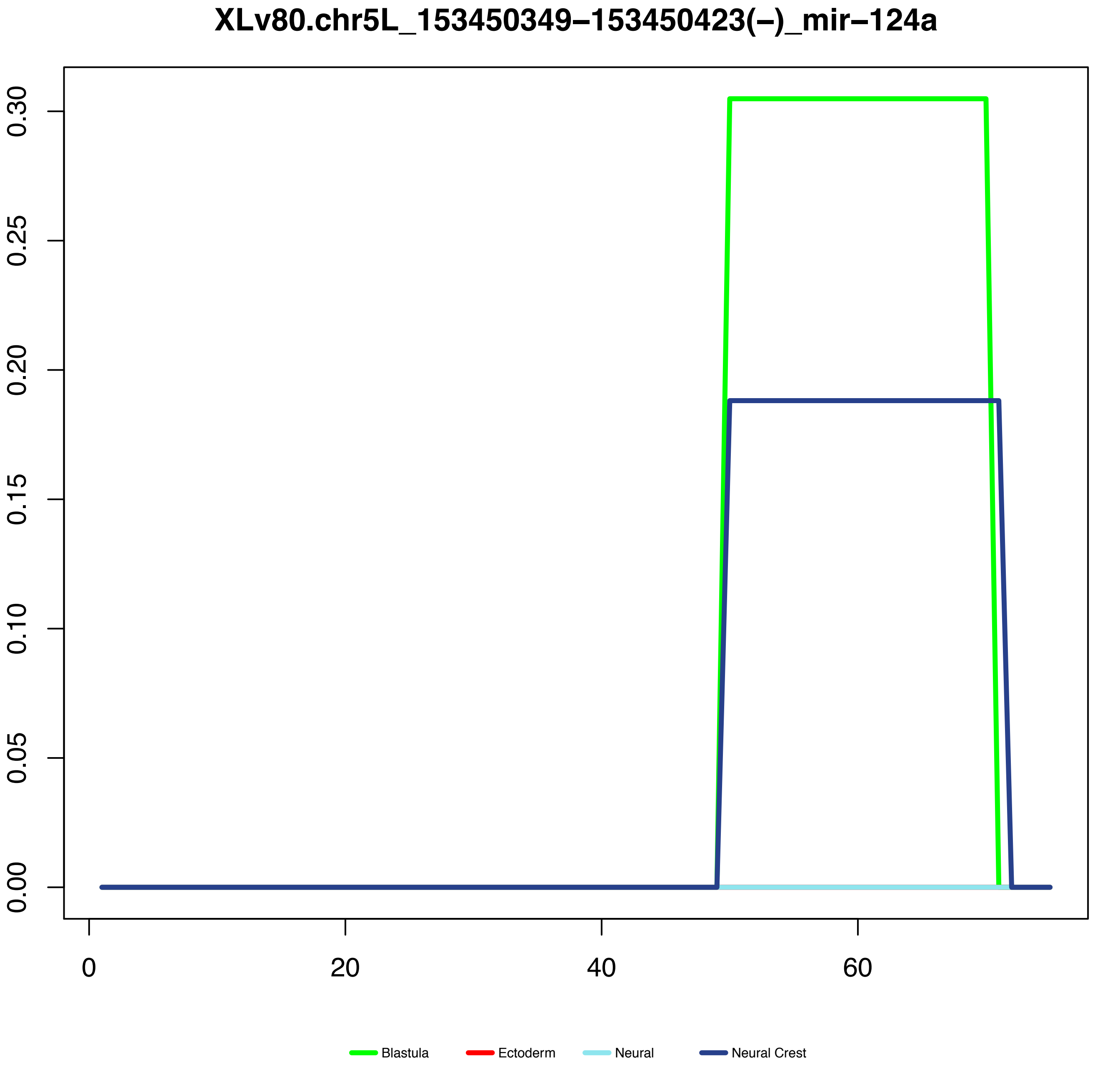
XLv80.chr5L_153450349−153450423(−)_mir−124a
0.30
0.25
0.20
0.15
0.10
0.05
0.00
0
20
40
60
Blastula
Ectoderm
Neural
Neural Crest

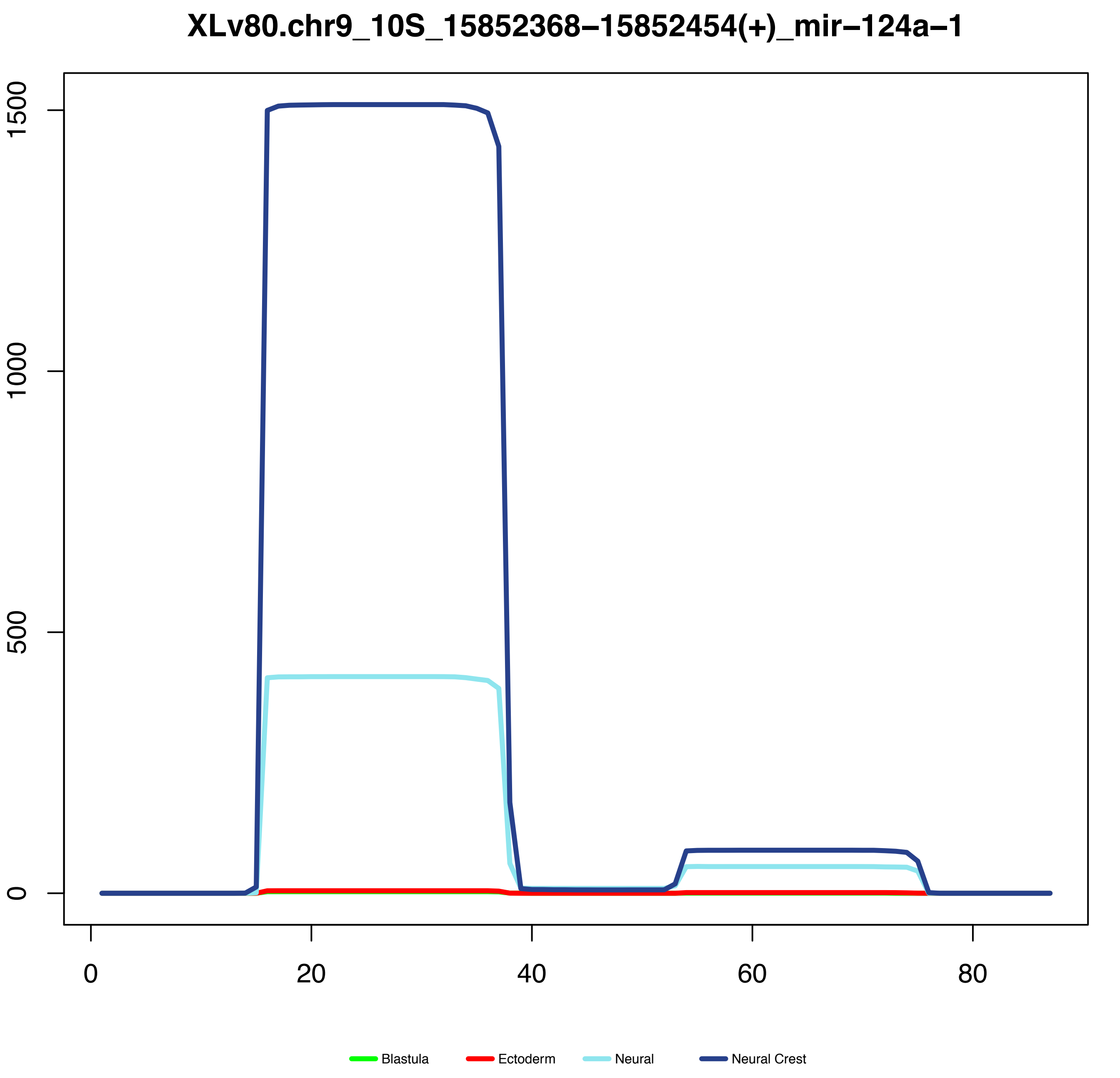

XLv80.chr9_10S_15852368−15852454(+)_mir−124a−1
0
500
1000
1500
0
20
40
60
80
Blastula
Ectoderm
Neural
Neural Crest

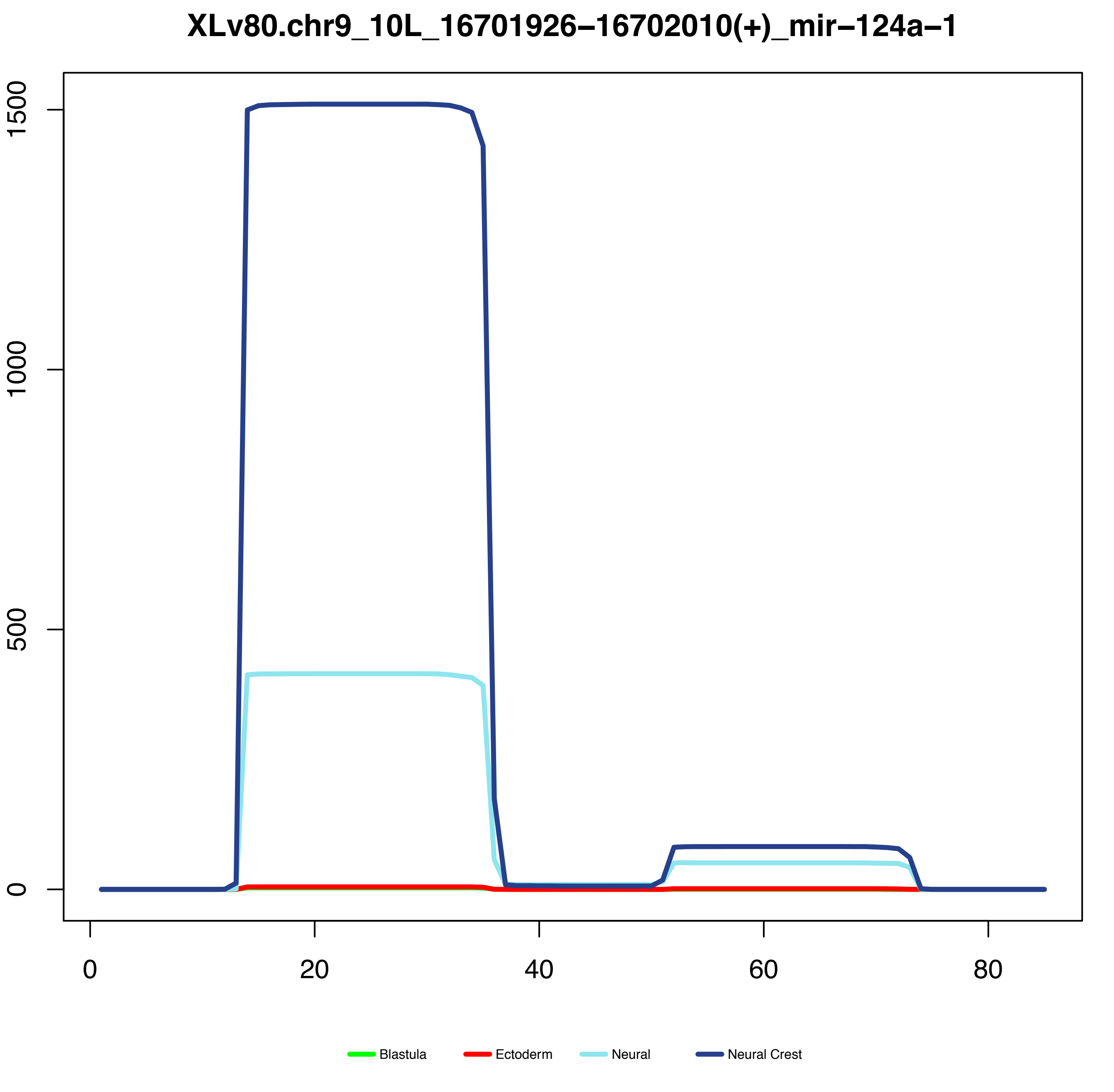

XLv80.chr9_10L_16701926−16702010(+)_mir−124a−1
1500
1000
500
0
0
20
40
60
80
Blastula
Ectoderm
Neural
Neural Crest

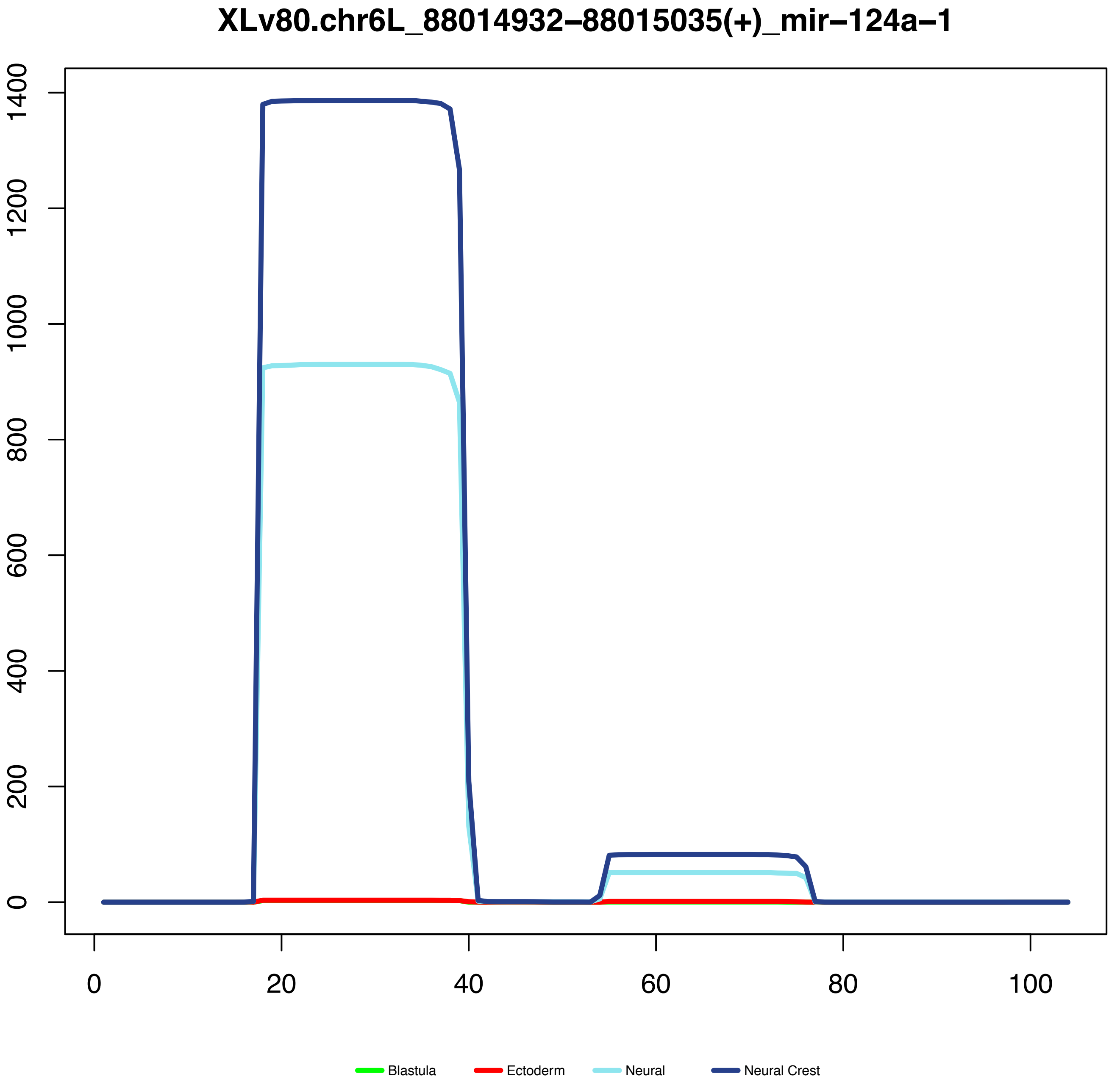
XLv80.chr6L_88014932−88015035(+)_mir−124a−1
0
200
400
600
800
1000
1200
1400
0
20
40
60
80
100
Blastula
Ectoderm
Neural
Neural Crest

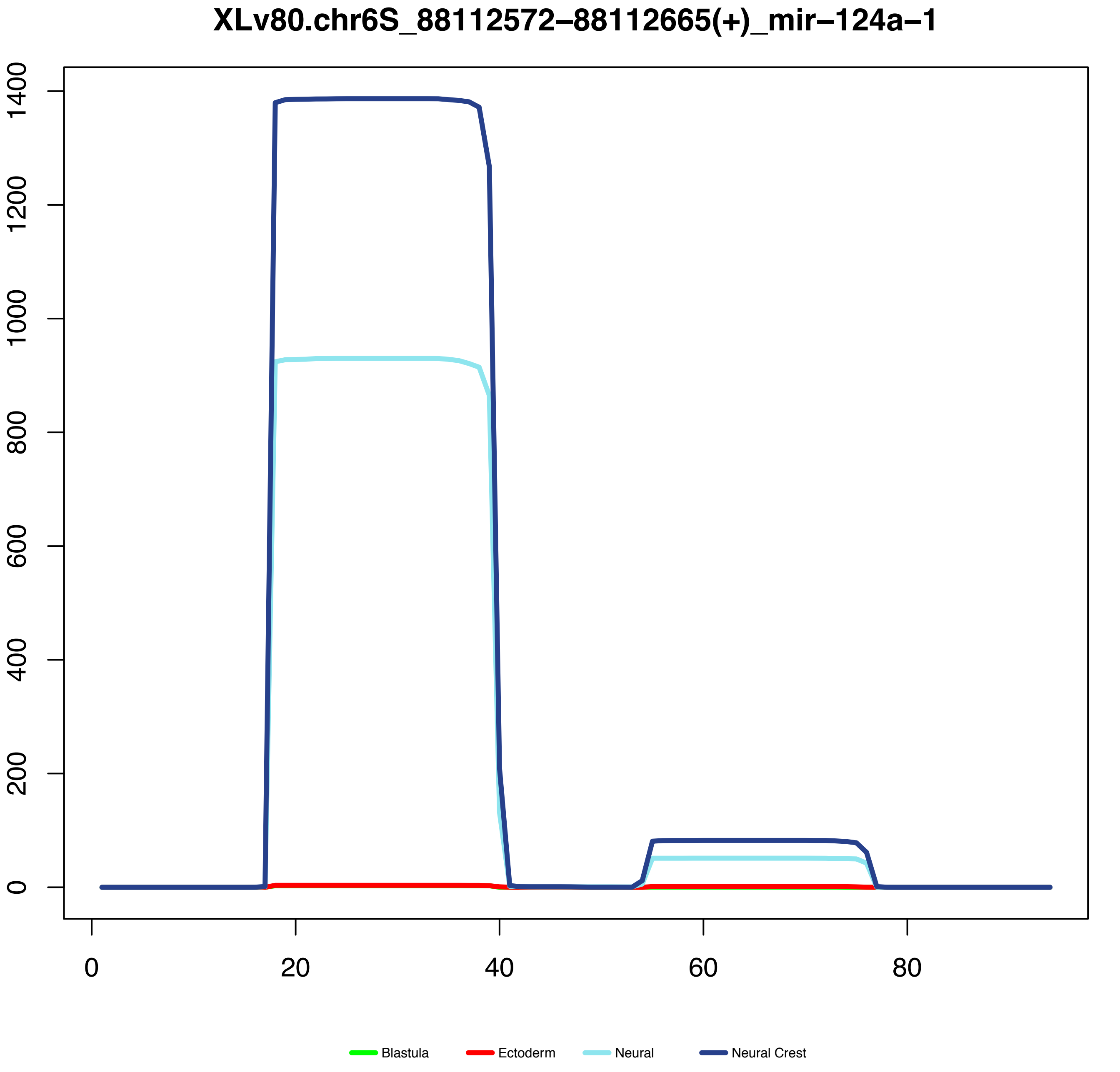

XLv80.chr6S_88112572−88112665(+)_mir−124a−1
0
200
400
600
800
1000
1200
1400
0
20
40
60
80
Blastula
Ectoderm
Neural
Neural Crest

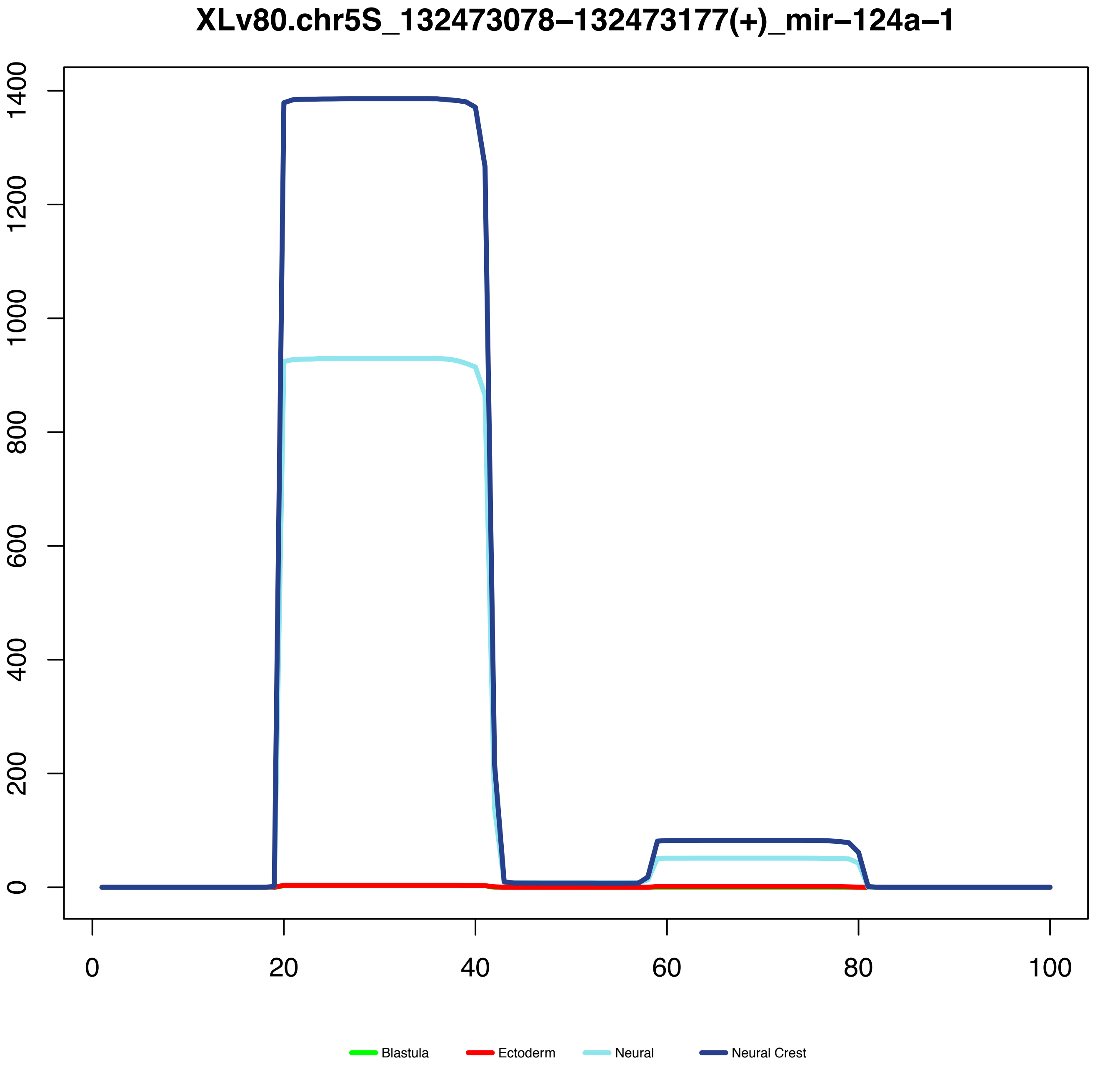
XLv80.chr5S_132473078−132473177(+)_mir−124a−1
1400
1200
1000
800
600
400
200
0
0
20
40
60
80
100
Blastula
Ectoderm
Neural
Neural Crest

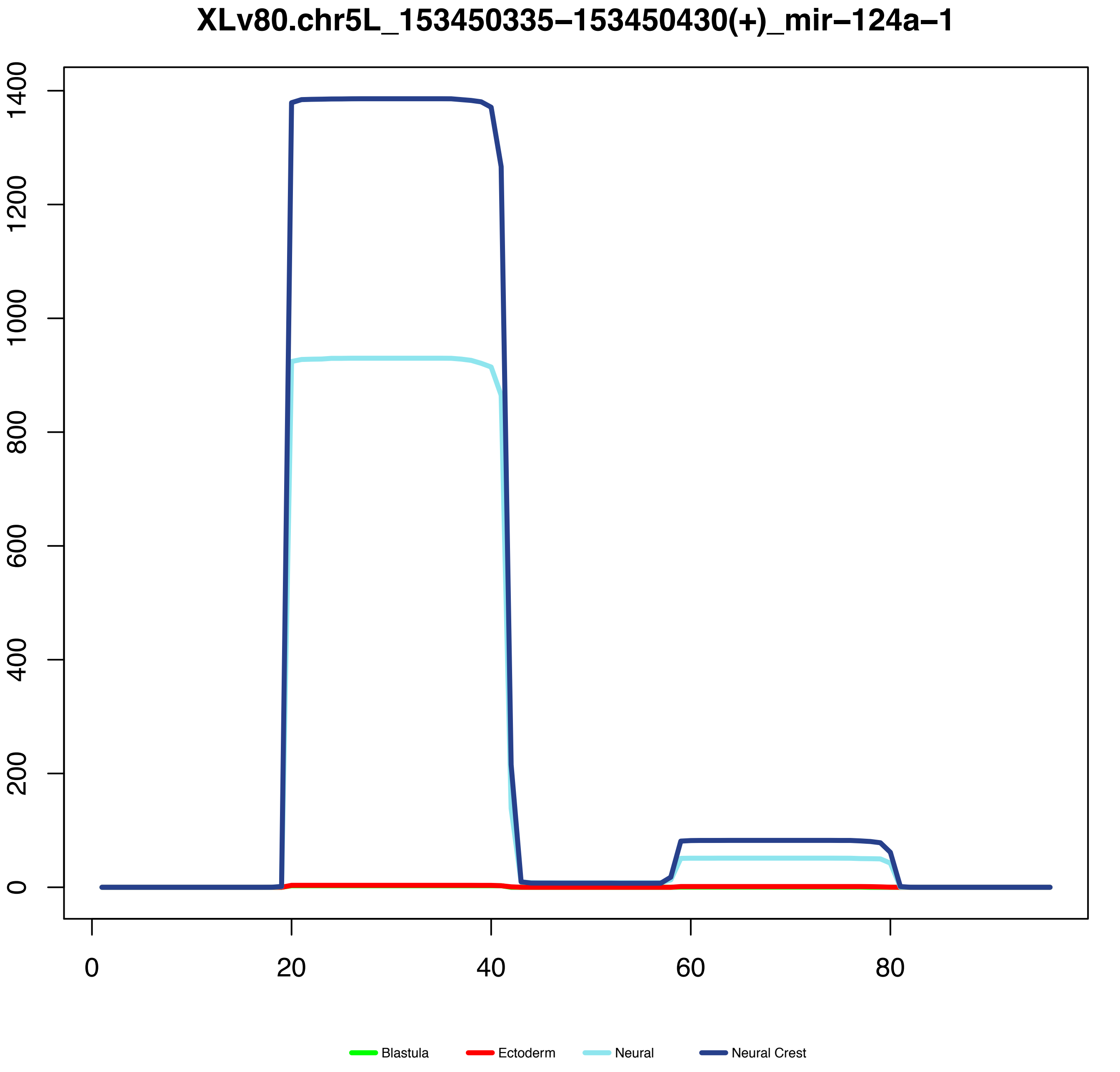
XLv80.chr5L_153450335−153450430(+)_mir−124a−1
0
200
400
600
800
1000
1200
1400
0
20
40
60
80
Blastula
Ectoderm
Neural
Neural Crest

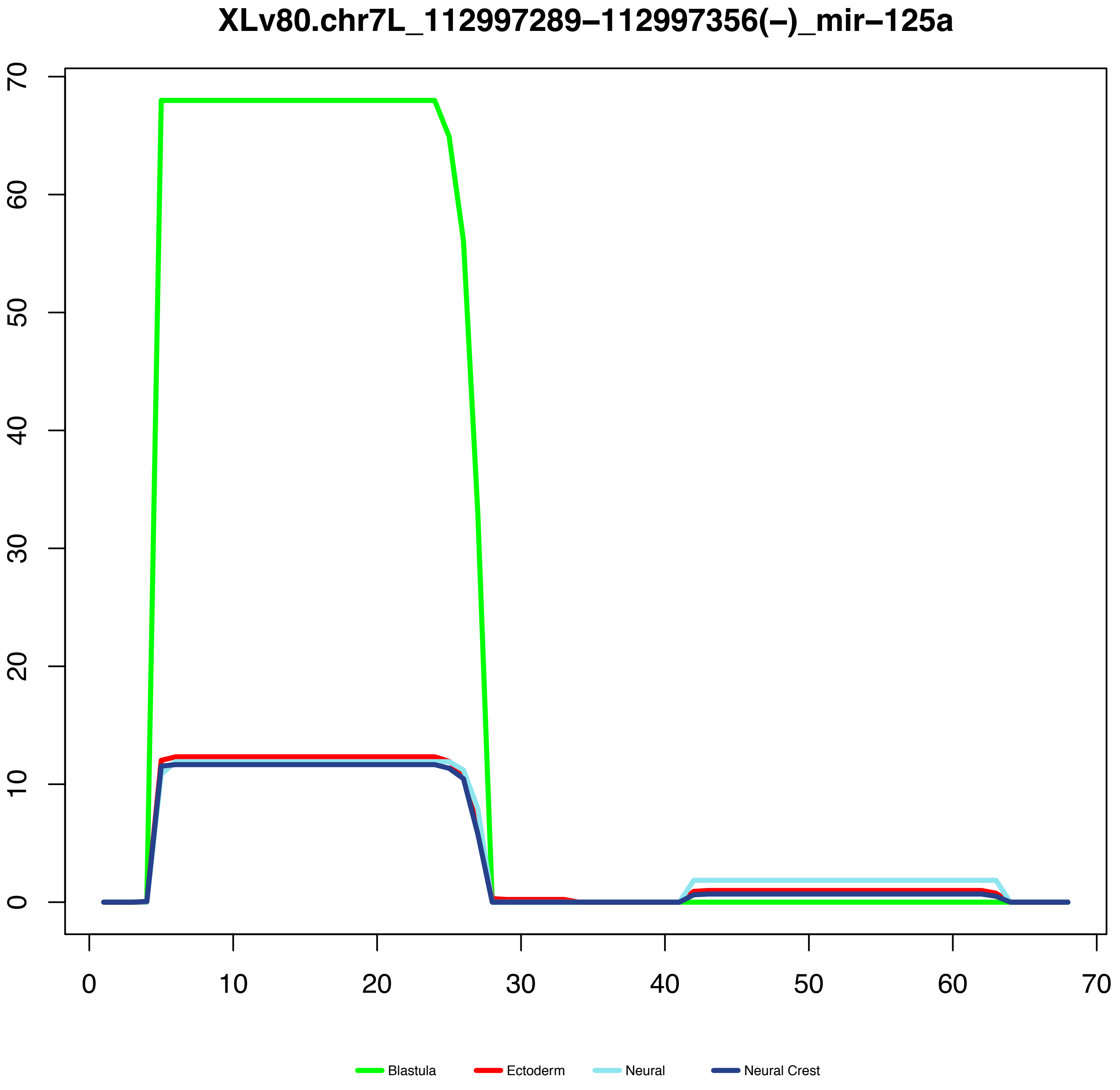

XLv80.chr7L_112997289−112997356(−)_mir−125a
0
10
20
30
40
50
60
70
0
10
20
30
40
50
60
70
Blastula
Ectoderm
Neural
Neural Crest

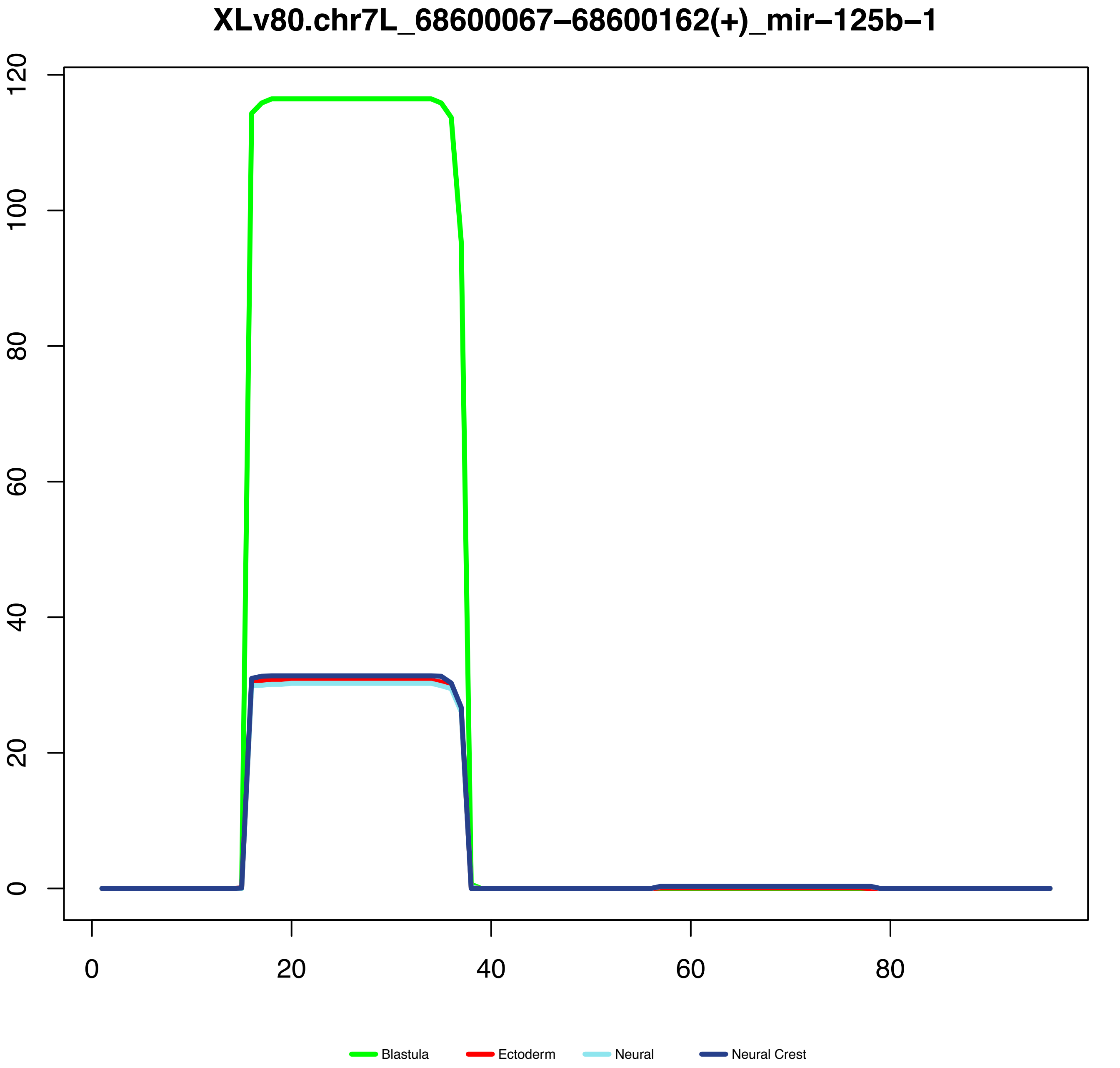
XLv80.chr7L_68600067−68600162(+)_mir−125b−1
0
20
40
60
80
100
120
0
20
40
60
80
Blastula
Ectoderm
Neural
Neural Crest

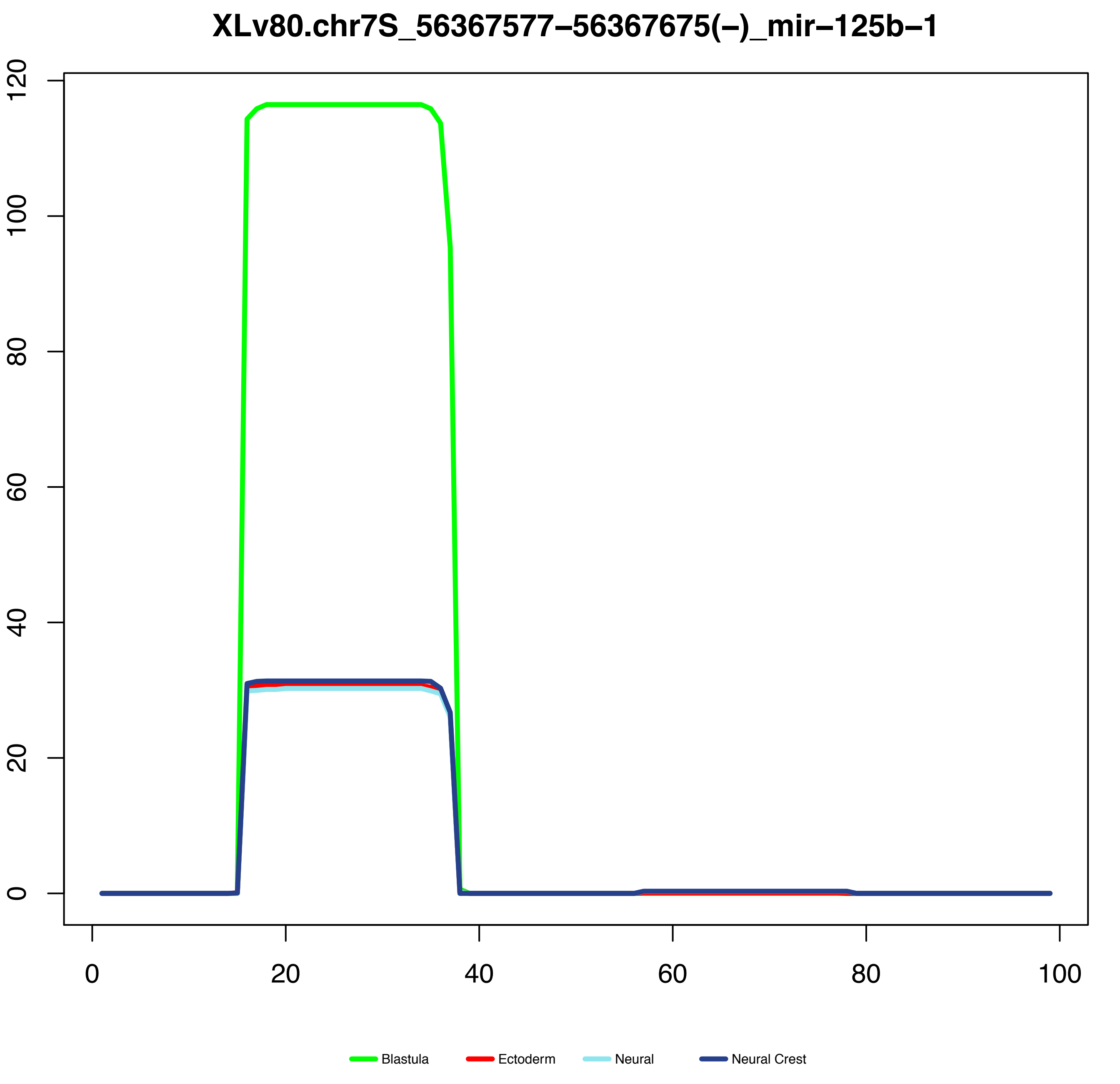
XLv80.chr7S_56367577−56367675(−)_mir−125b−1
0
20
40
60
80
100
120
Blastula
Ectoderm
Neural
Neural Crest

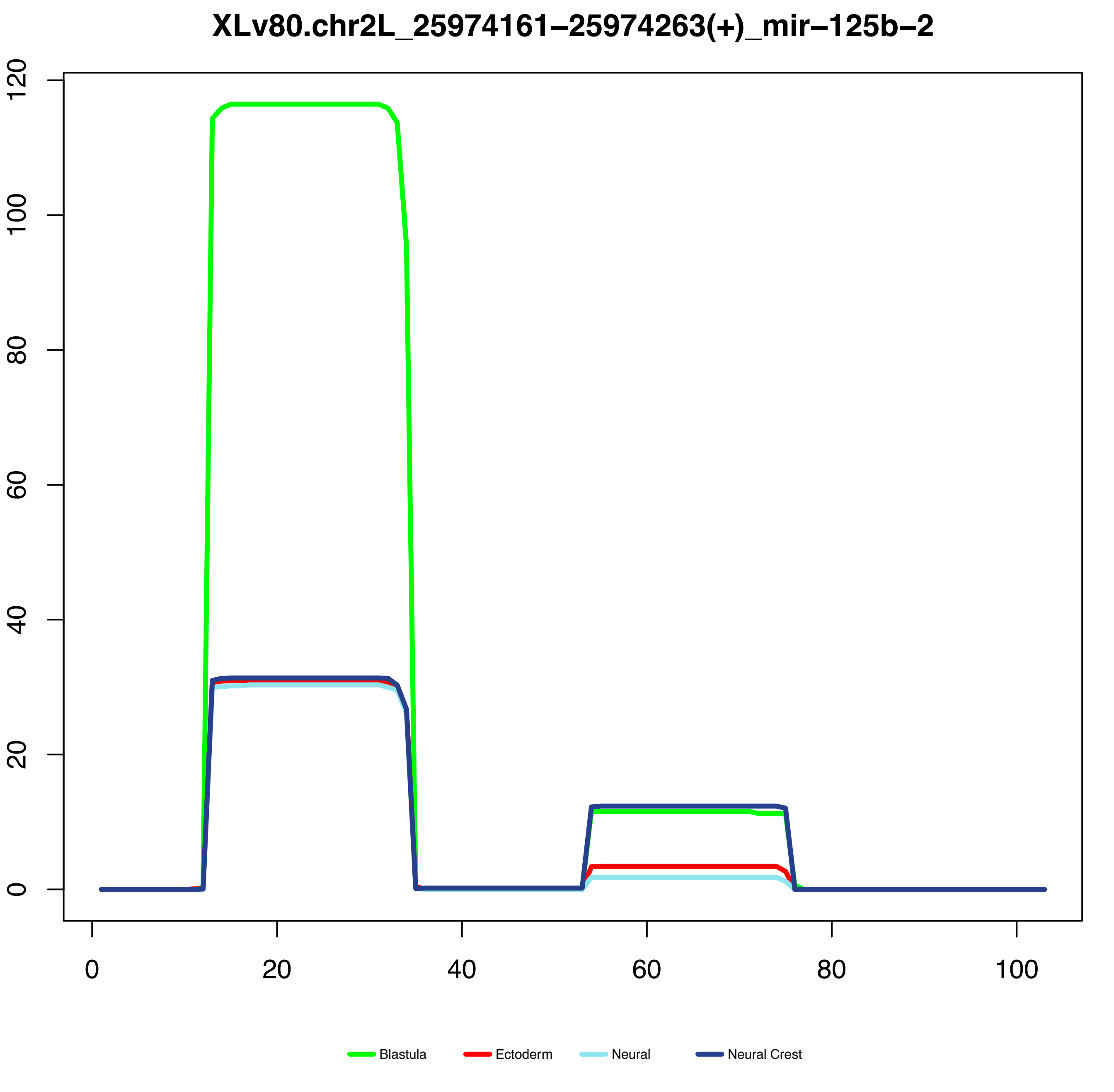
XLv80.chr2L_25974161−25974263(+)_mir−125b−2
0
20
40
60
80
100
120
0
20
40
60
80
100
Blastula
Ectoderm
Neural
Neural Crest

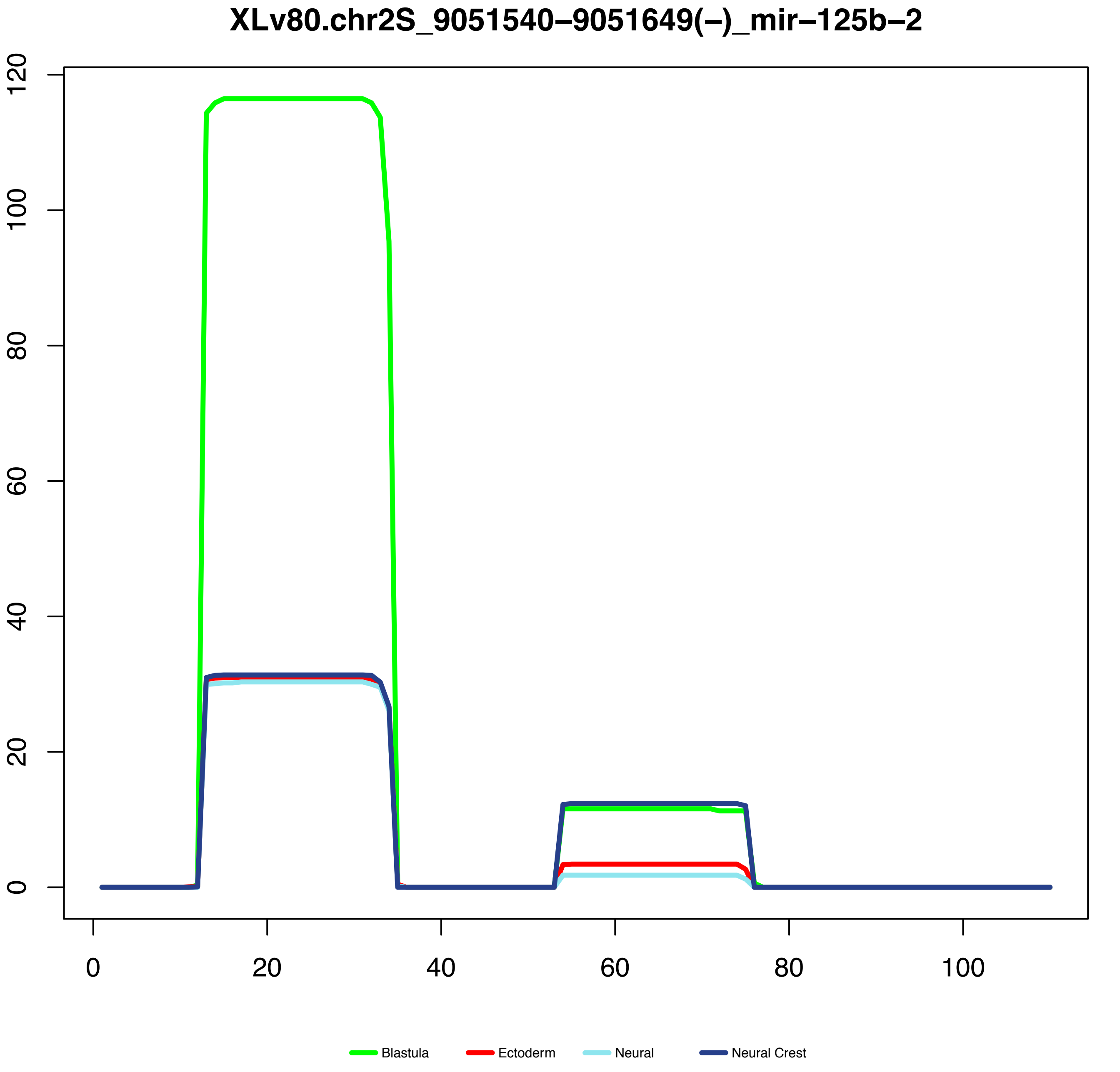

XLv80.chr2S_9051540−9051649(−)_mir−125b−2
0
20
40
60
80
100
120
0
20
40
60
80
100
Blastula
Ectoderm
Neural
Neural Crest

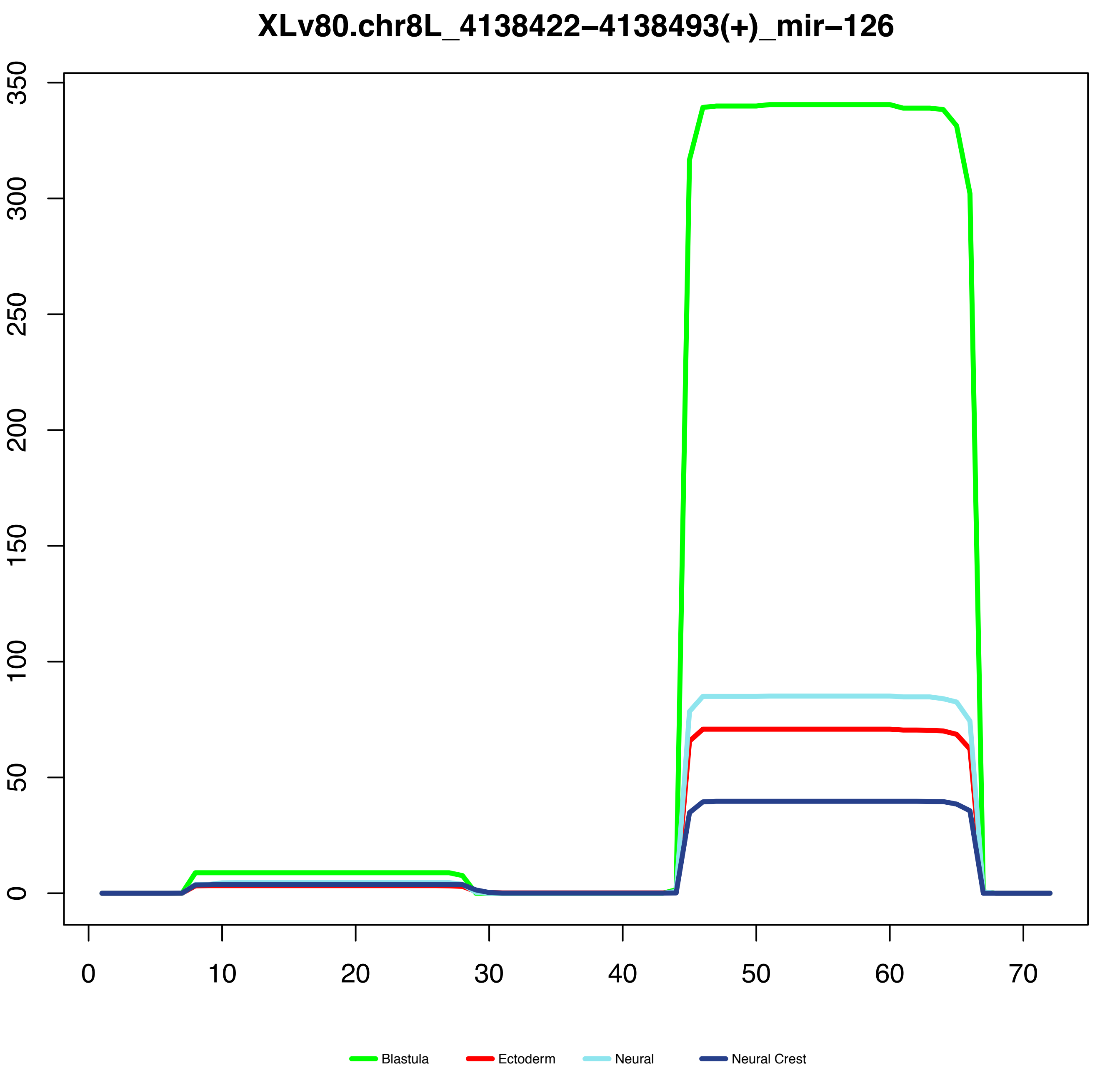

XLv80.chr8L_4138422–4138493(+)_mir–126
350
300
250
200
150
100
50
0
0
10
20
30
40
50
60
70
Blastula
Ectoderm
Neural
Neural Crest

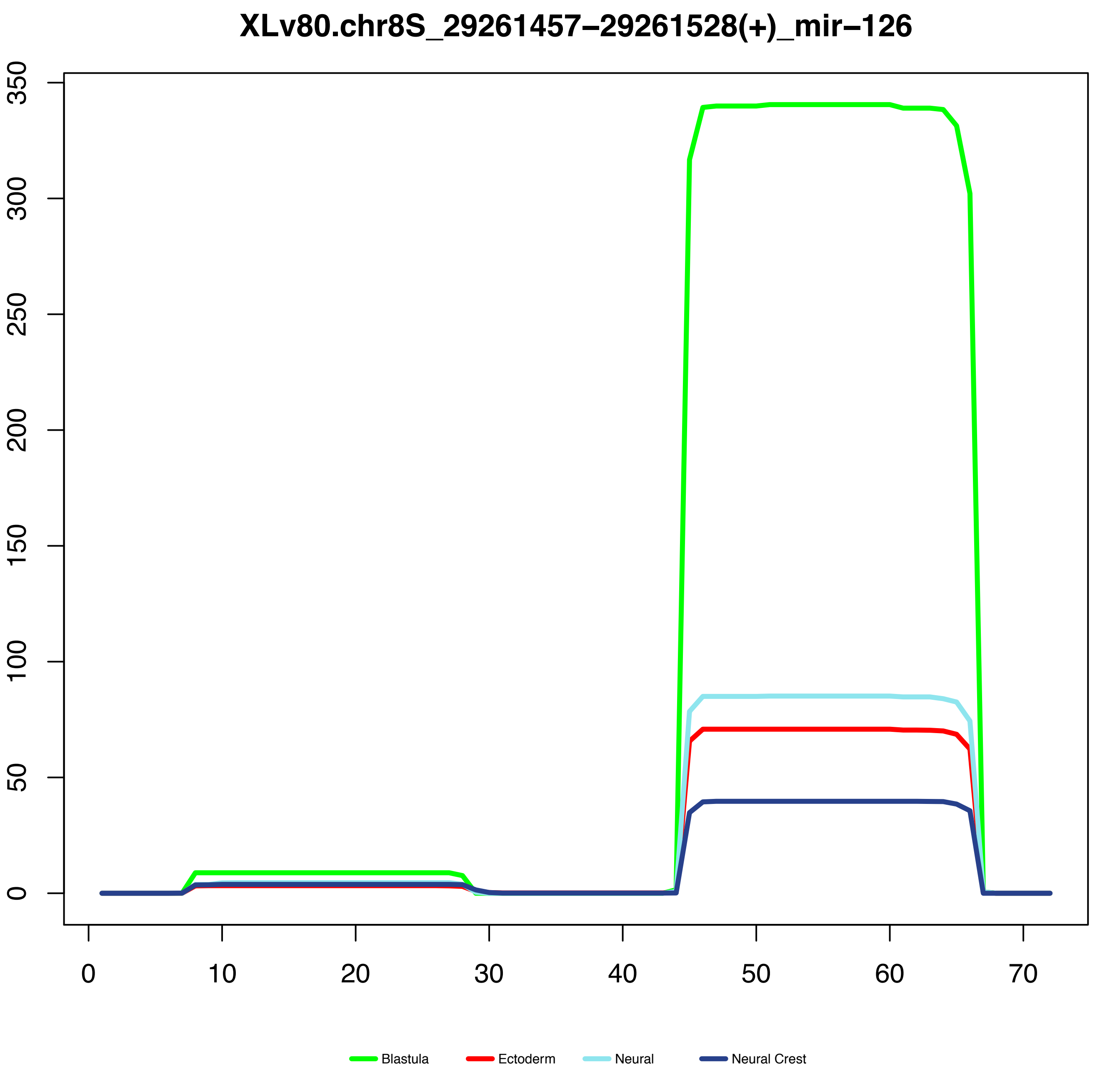

XLv80.chr8S_29261457−29261528(+)_mir−126
0
50
100
150
200
250
300
350
0
10
20
30
40
50
60
70
Blastula
Ectoderm
Neural
Neural Crest

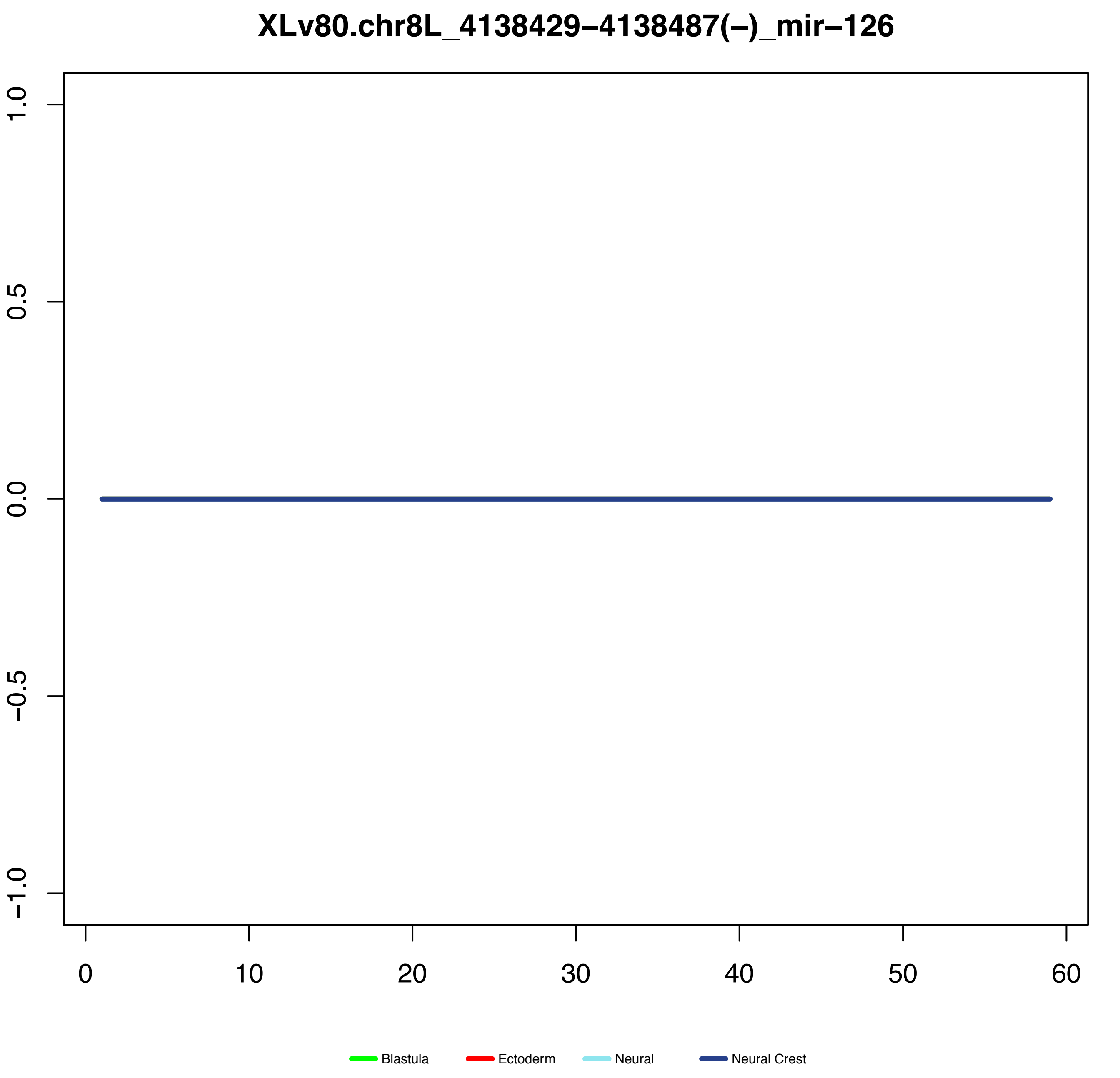
XLv80.chr8L_4138429−4138487(−)_mir−126
1.0
0.5
0.0
−0.5
−1.0
0
10
20
30
40
50
60
Blastula
Ectoderm
Neural
Neural Crest

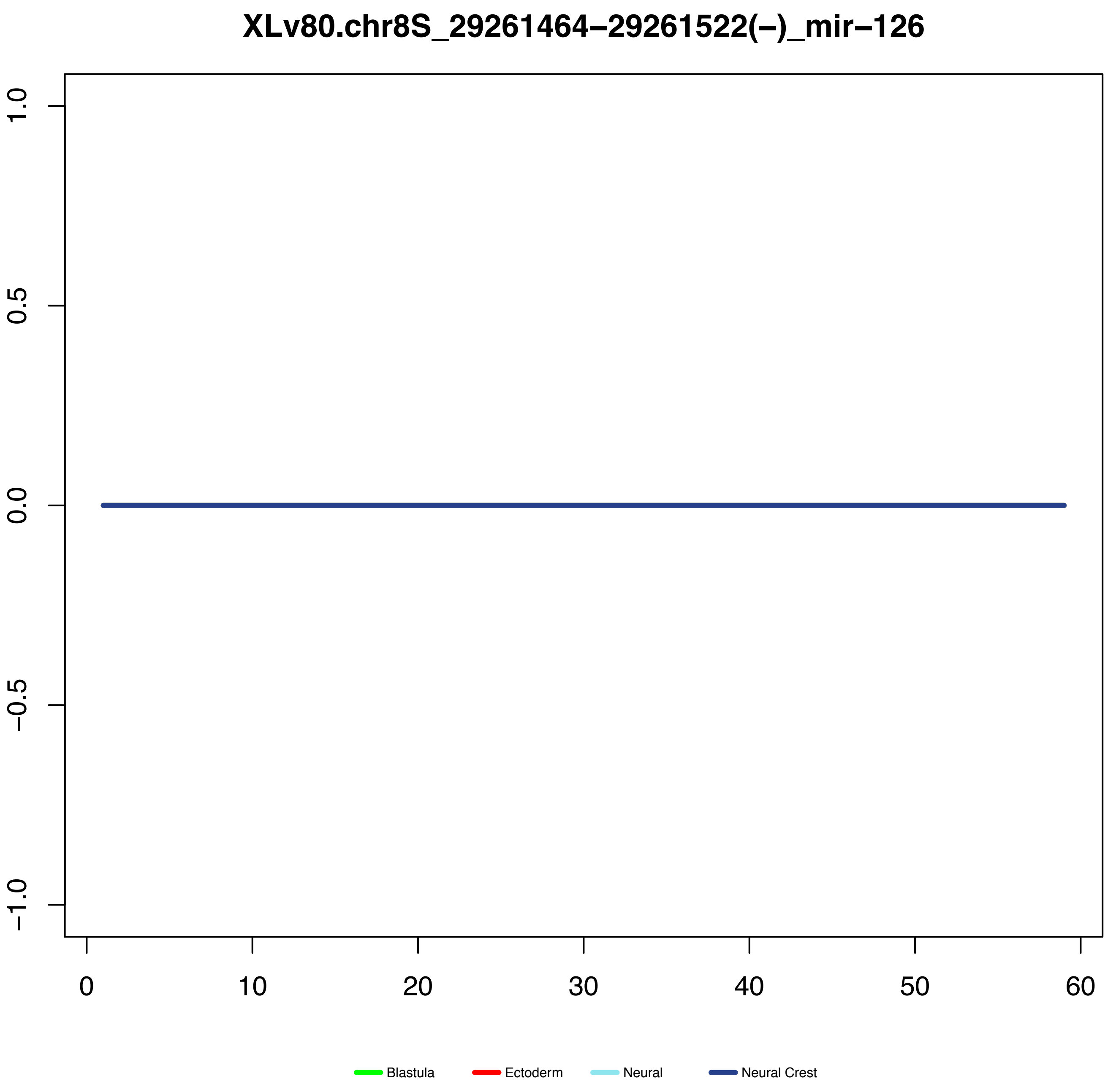

XLv80.chr8S_29261464−29261522(−)_mir−126
1.0
0.5
0.0
−0.5
−1.0
0
10
20
30
40
50
60
Blastula
Ectoderm
Neural
Neural Crest

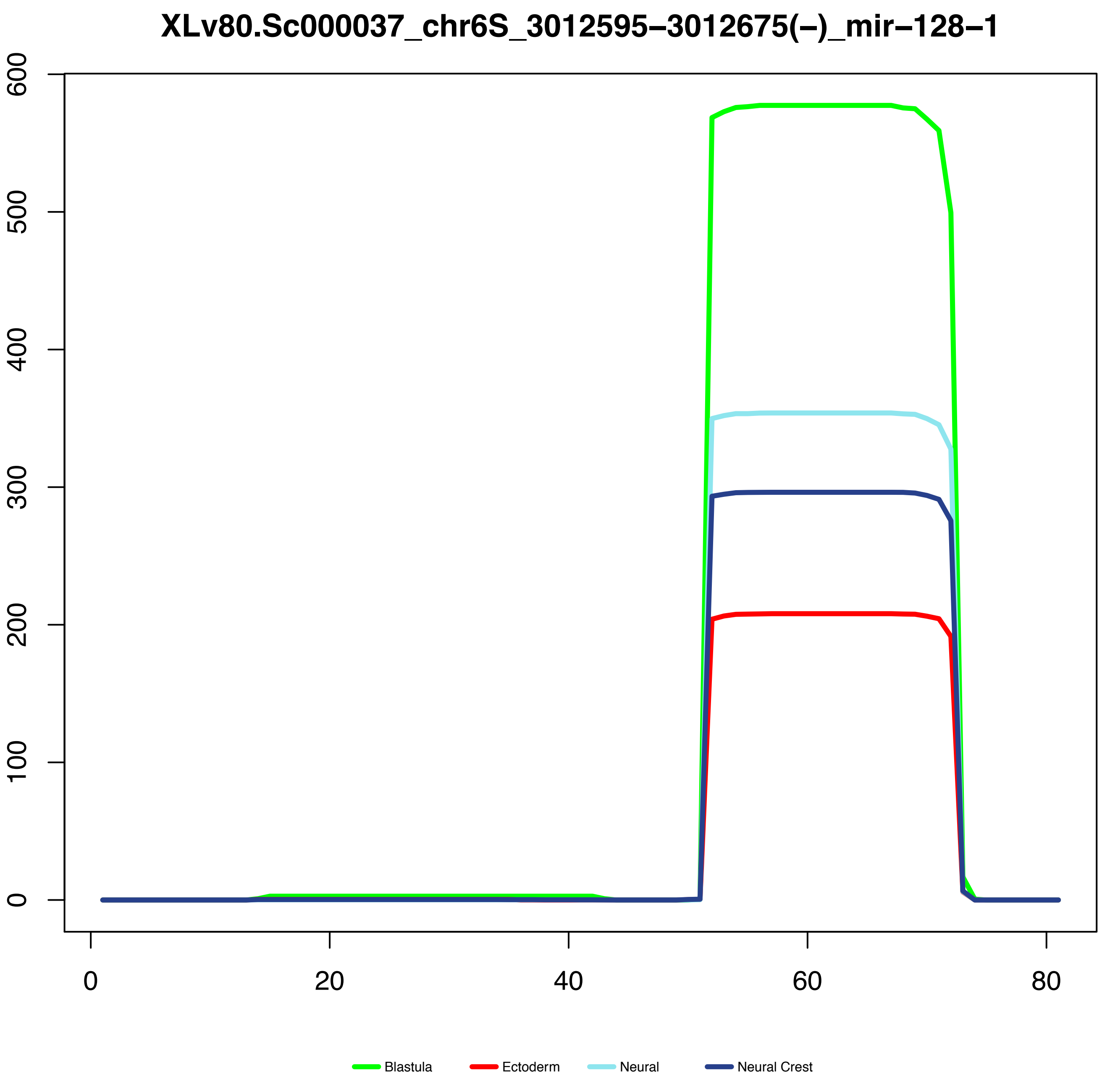

XLv80.Sc000037_chr6S_3012595−3012675(−)_mir−128−1
0
100
200
300
400
500
600
0
20
40
60
80
Blastula
Ectoderm
Neural
Neural Crest

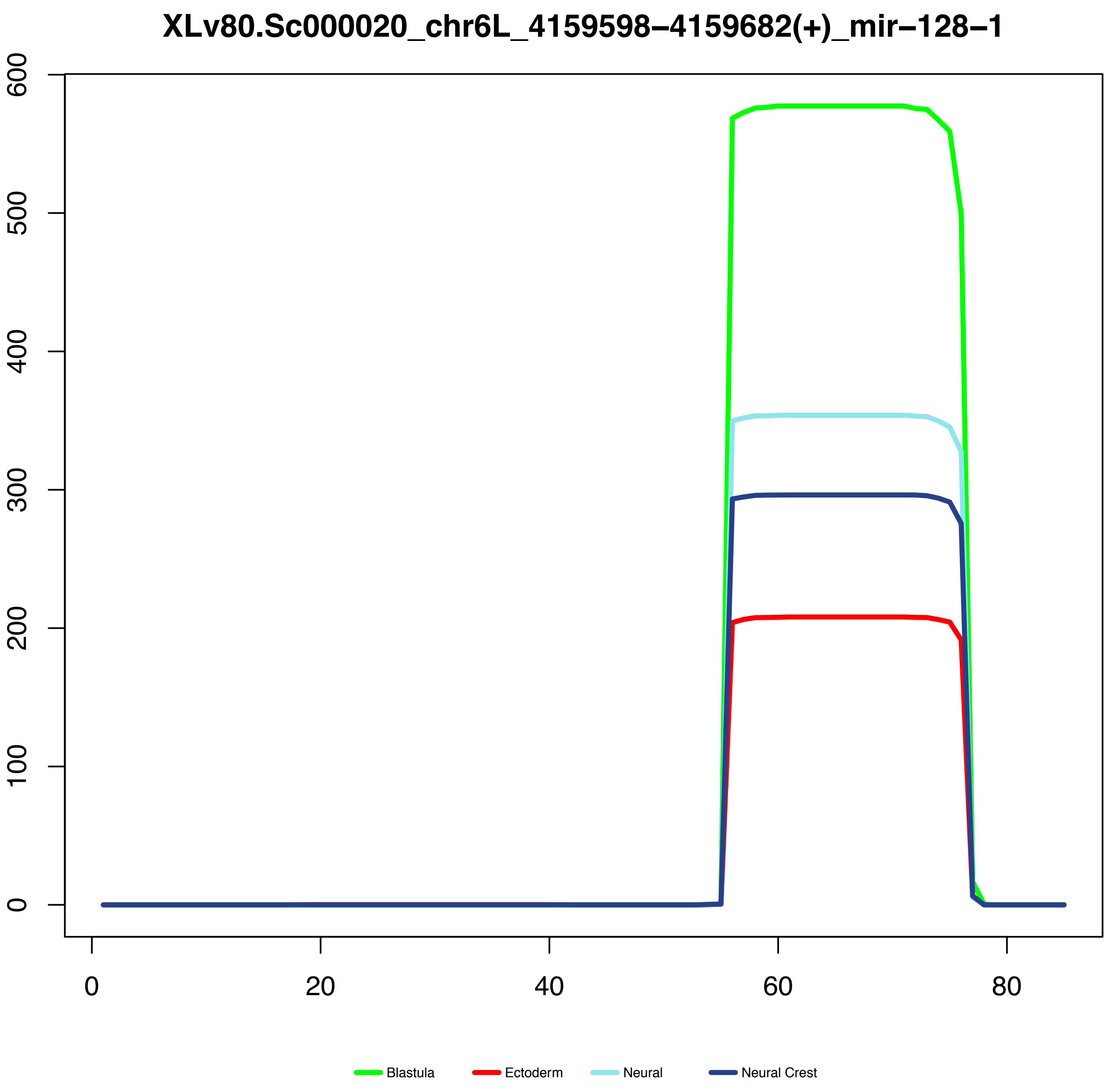

XLv80.Sc000020_chr6L_4159598−4159682(+)_mir−128−1
600
500
400
300
200
100
0
0
20
40
60
80
Blastula
Ectoderm
Neural
Neural Crest

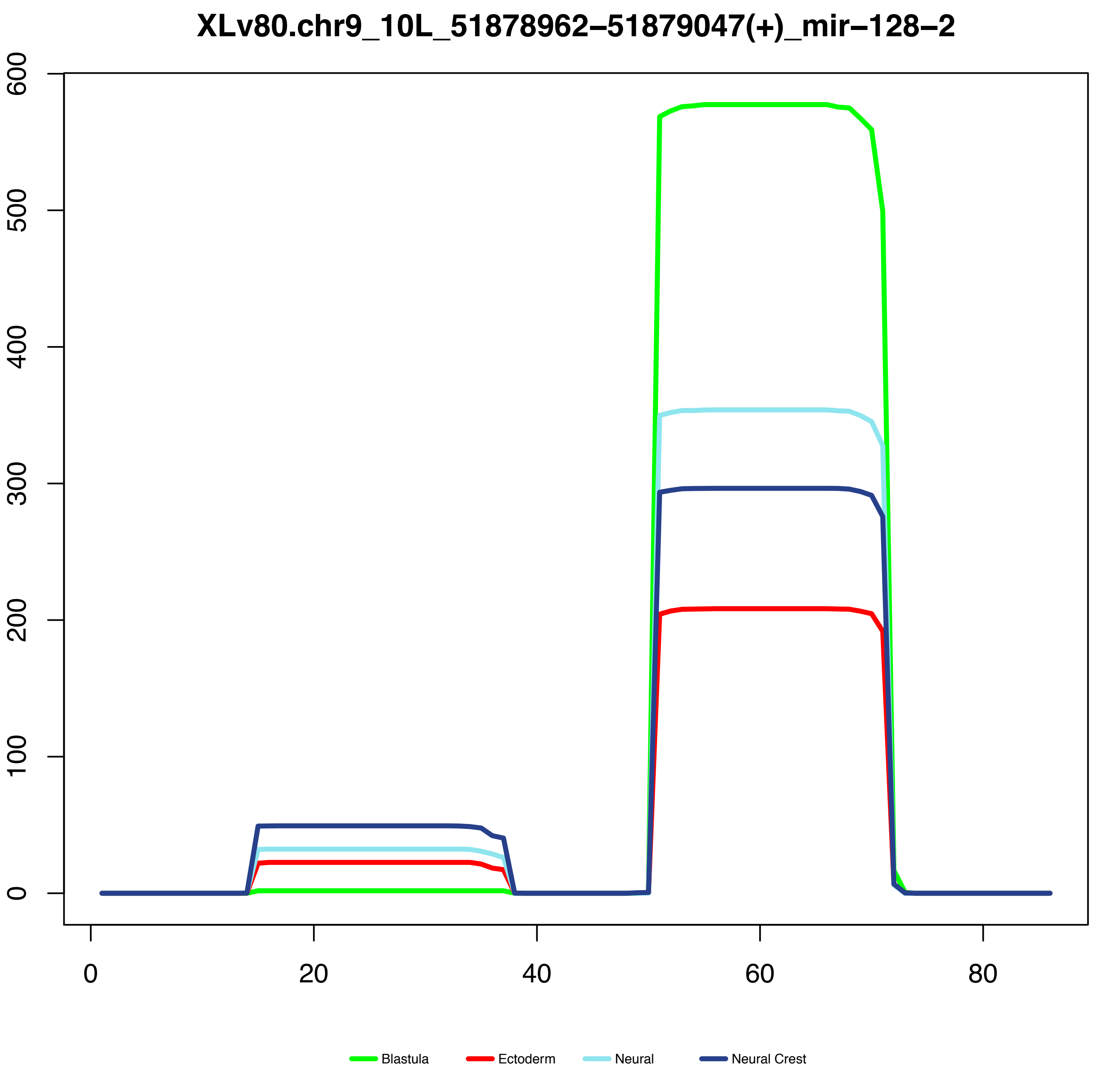
XLv80.chr9_10L_51878962−51879047(+)_mir−128−2
0
100
200
300
400
500
600
0
20
40
60
80
Blastula
Ectoderm
Neural
Neural Crest

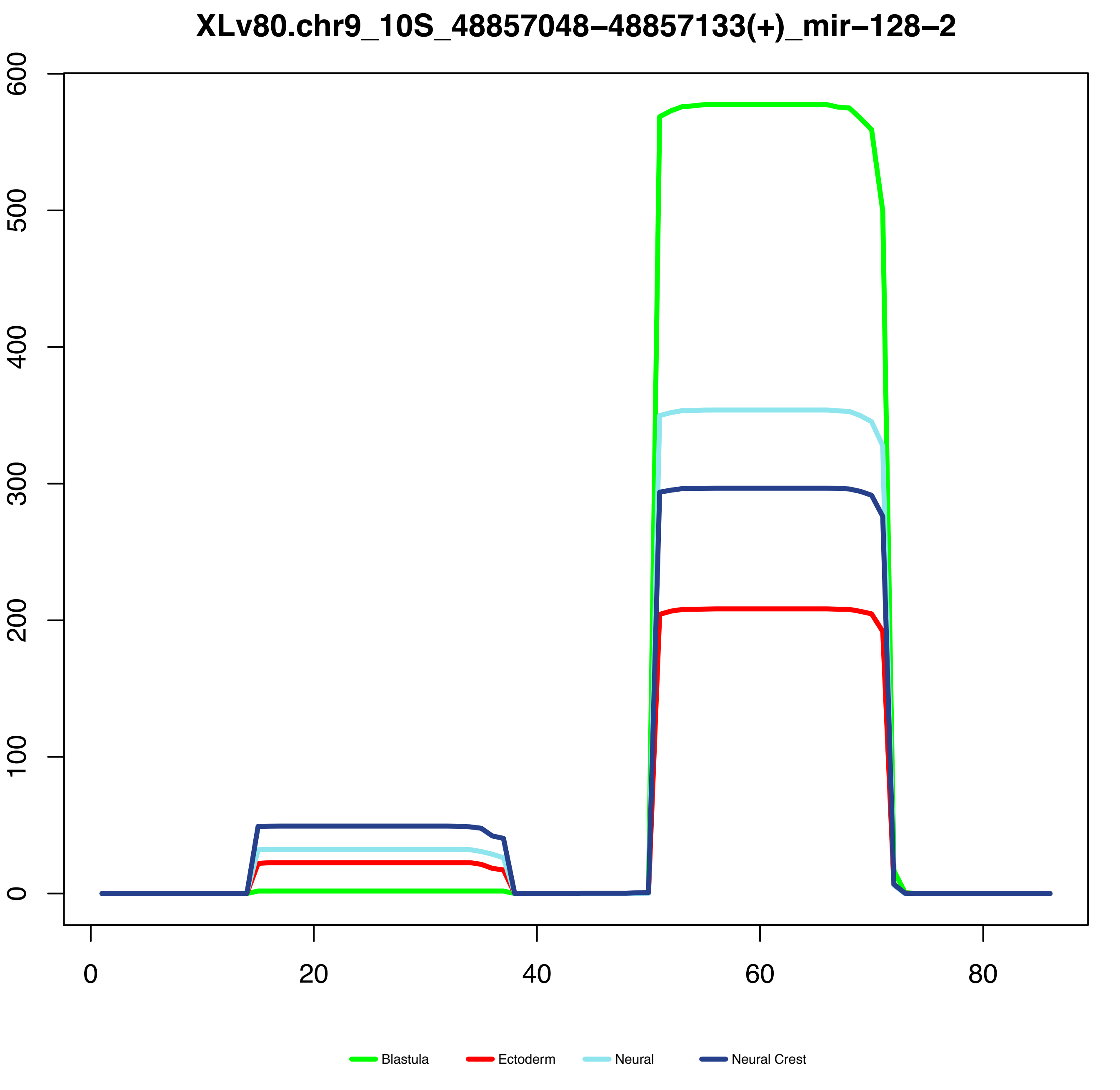

XLv80.chr9_10S_48857048−48857133(+)_mir−128−2
0
100
200
300
400
500
600
0
20
40
60
80
Blastula
Ectoderm
Neural
Neural Crest

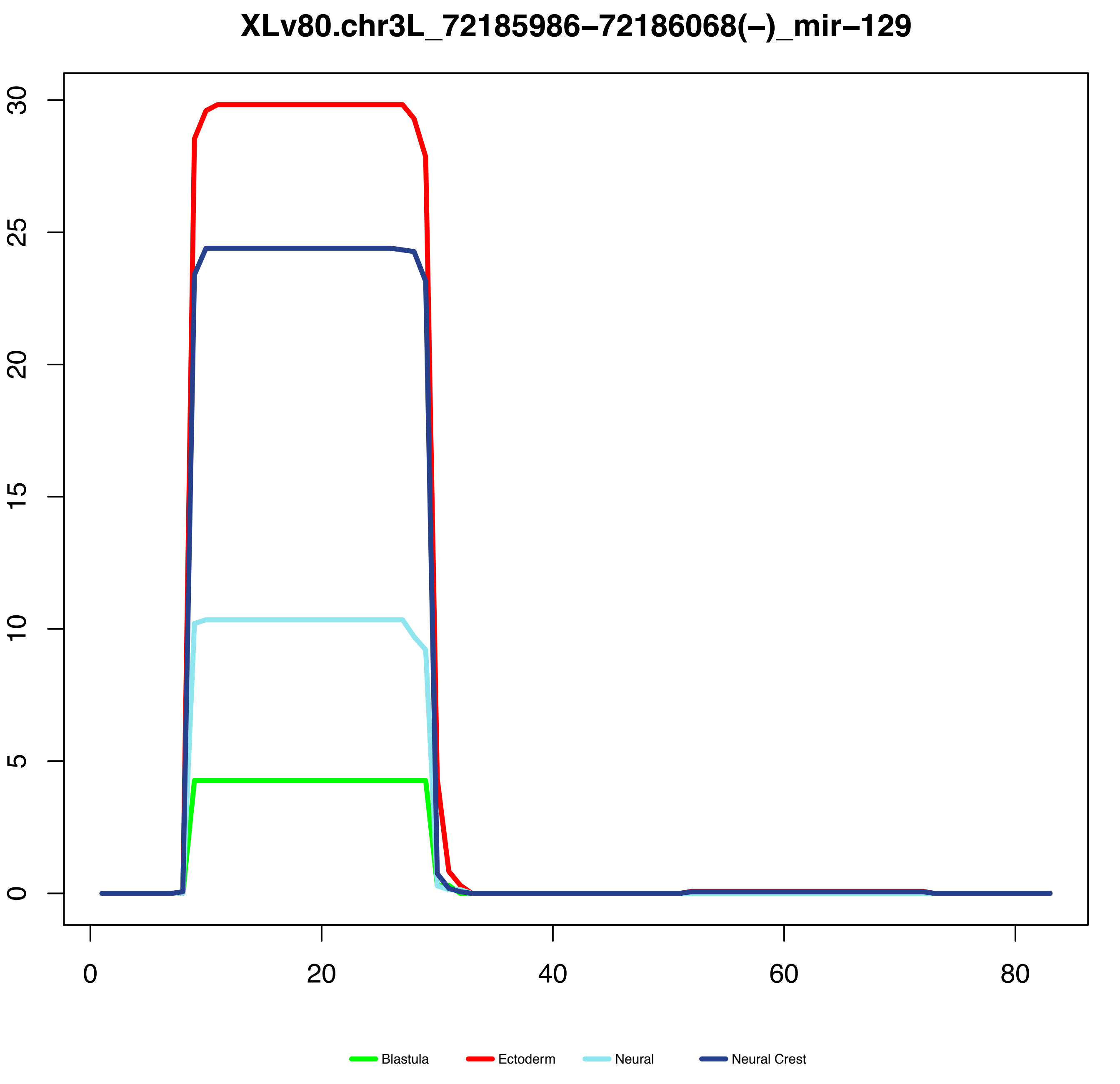
XLv80.chr3L_72185986−72186068(−)_mir−129
30
25
20
15
10
5
0
0
20
40
60
80
Blastula
Ectoderm
Neural
Neural Crest

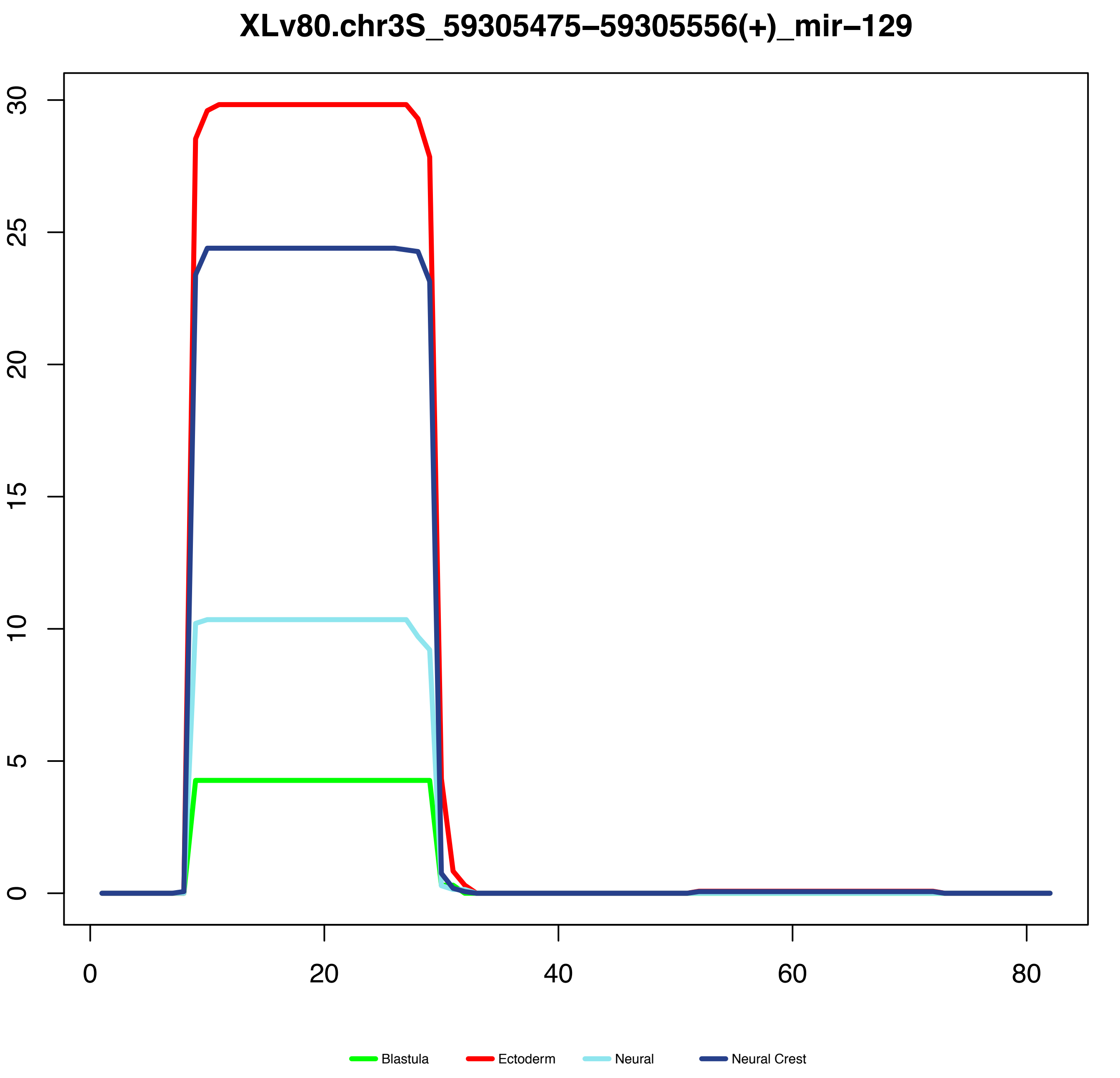
XLv80.chr3S_59305475−59305556(+)_mir−129
0
5
10
15
20
25
30
0
20
40
60
80
Blastula
Ectoderm
Neural
Neural Crest

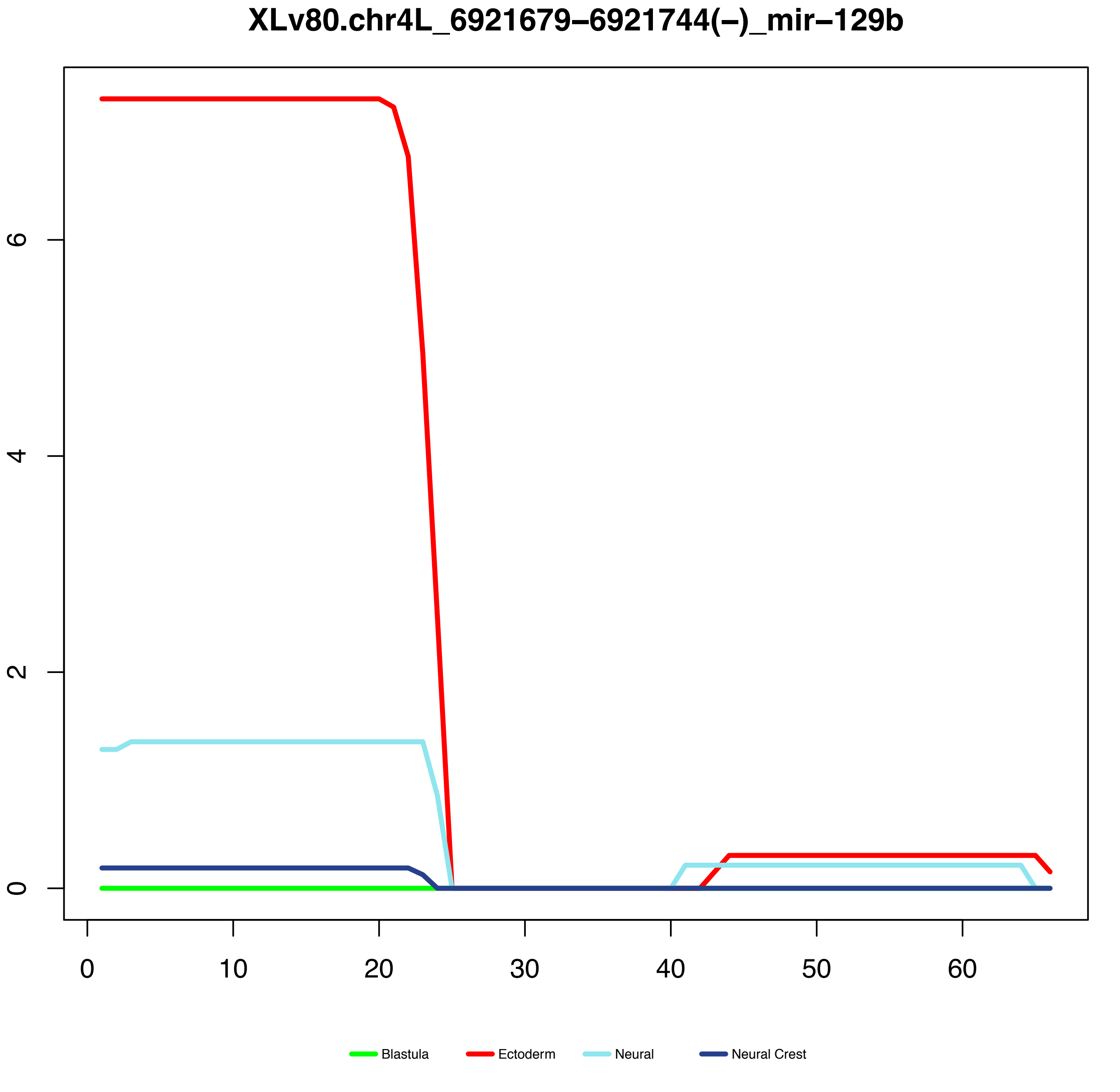

XLv80.chr4L_6921679−6921744(−)_mir−129b
0
2
4
6
0
10
20
30
40
50
60
Blastula
Ectoderm
Neural
Neural Crest

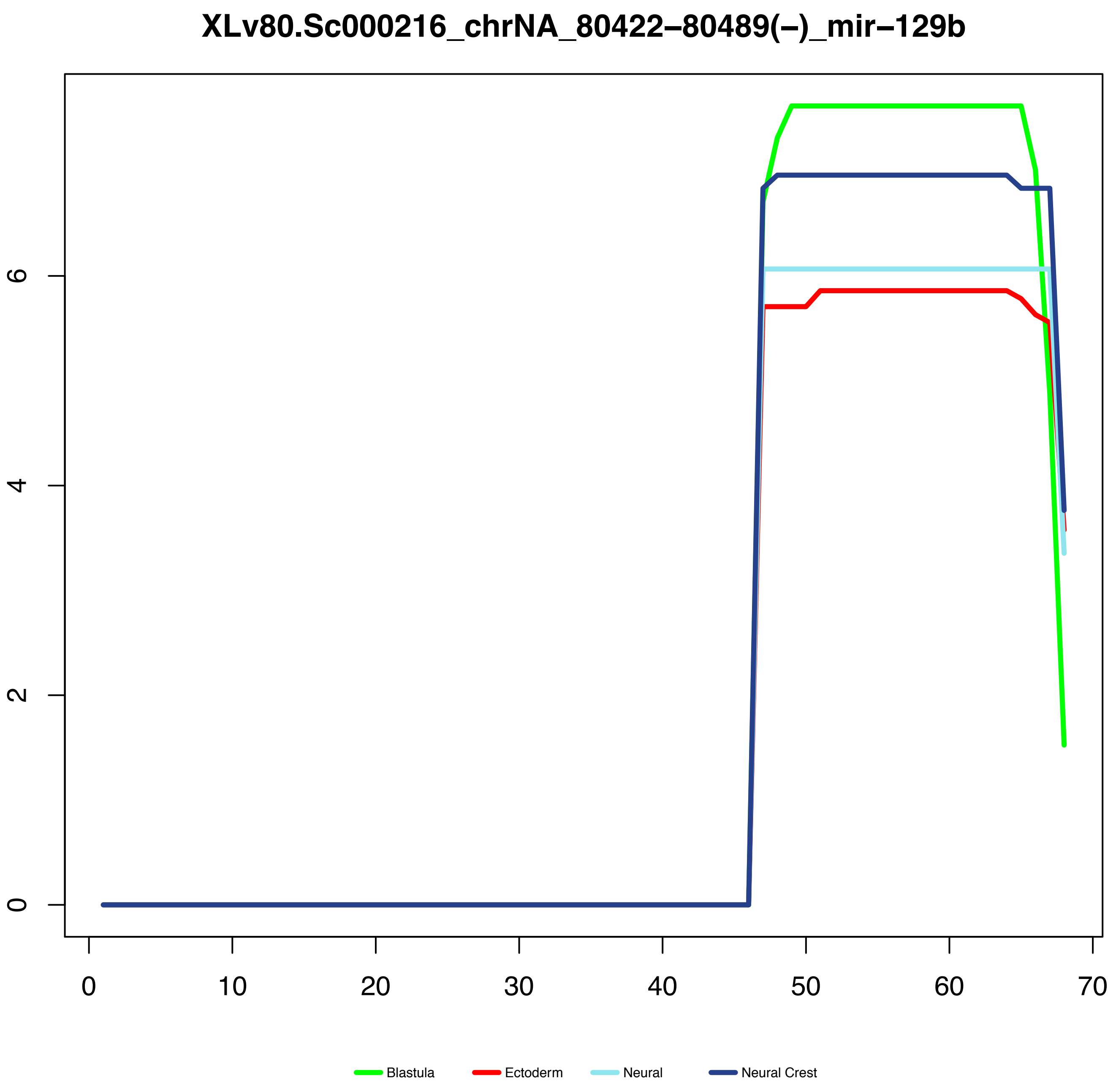

XLv80.Sc000216_chrNA_80422–80489(–)_mir–129b
0
2
4
6
0
10
20
30
40
50
60
70
Blastula
Ectoderm
Neural
Neural Crest

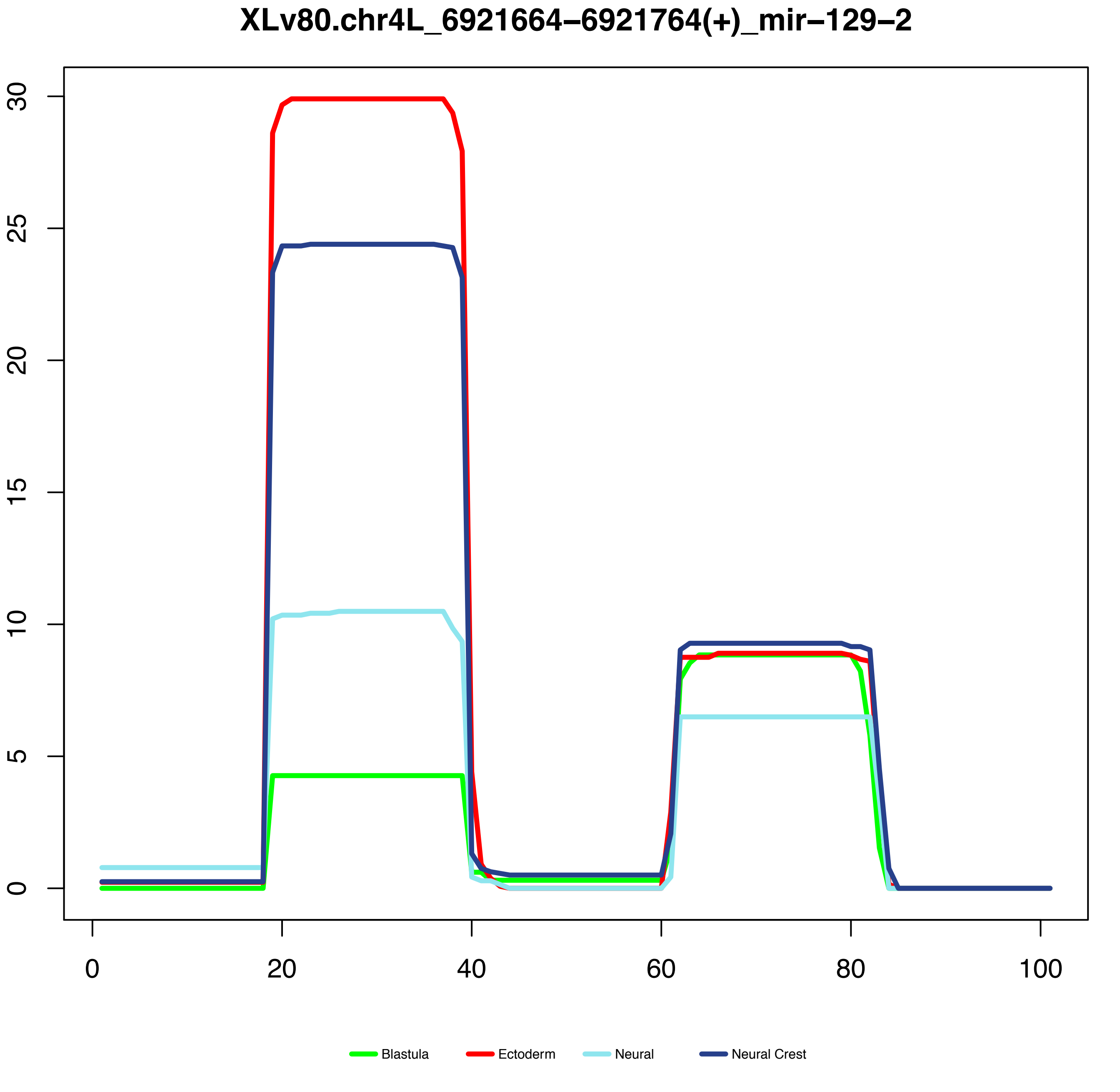
XLv80.chr4L_6921664–6921764(+)_mir–129–2
0
5
10
15
20
25
30
0
20
40
60
80
100
Blastula
Ectoderm
Neural
Neural Crest

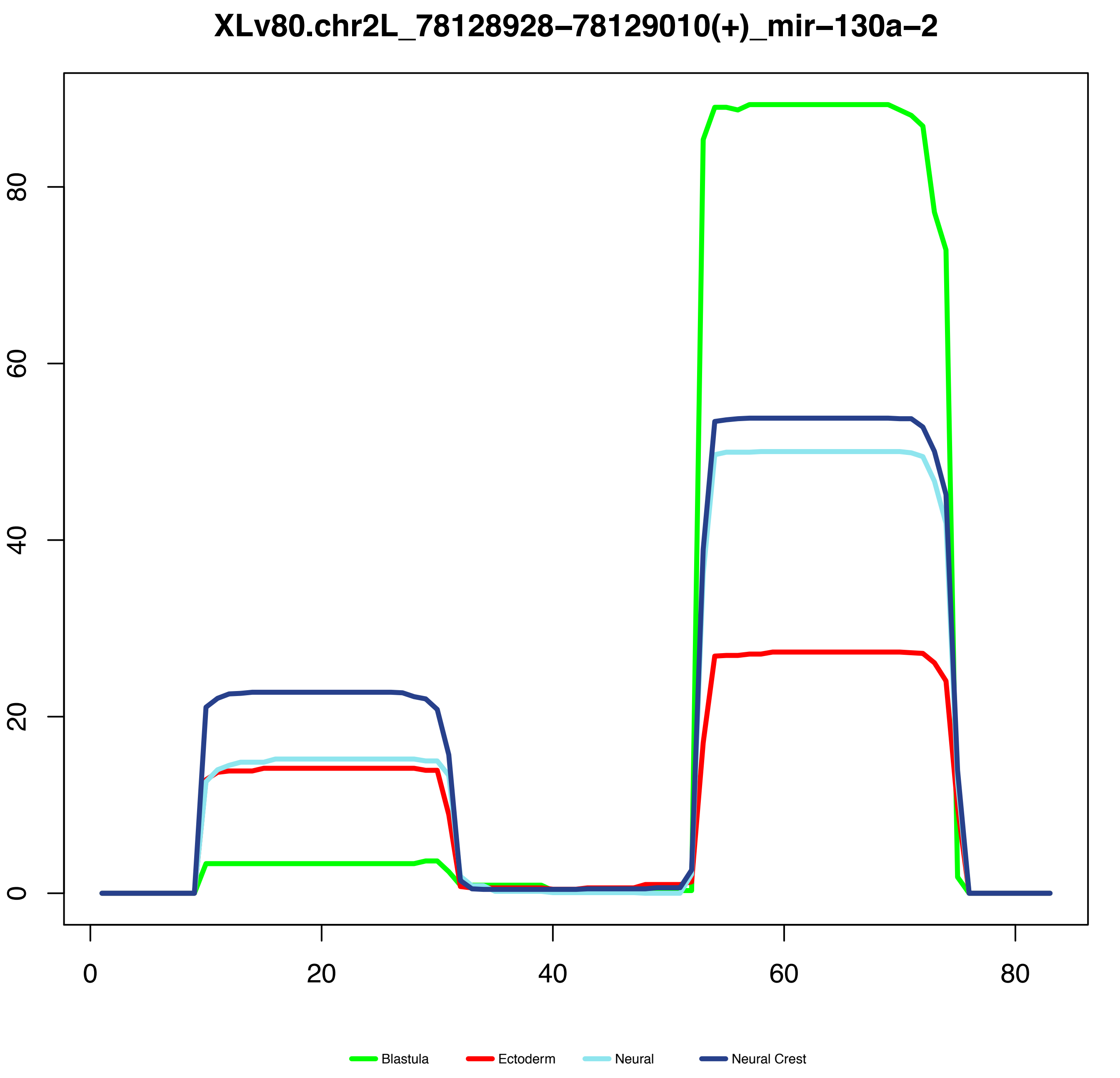
XLv80.chr2L_78128928−78129010(+)_mir−130a−2
0
20
40
60
80
0
20
40
60
80
Blastula
Ectoderm
Neural
Neural Crest

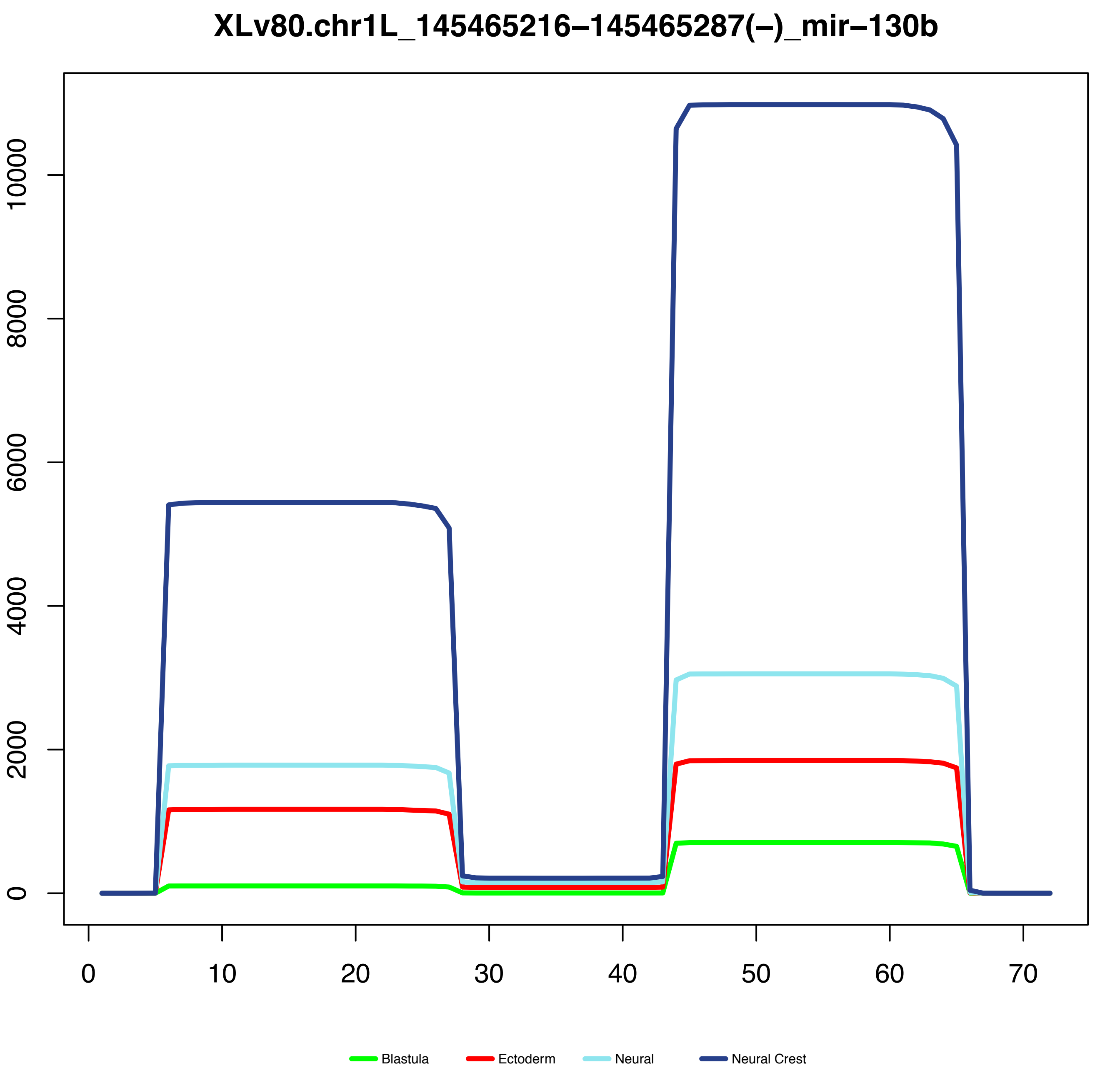
XLv80.chr1L_145465216−145465287(−)_mir−130b
0
2000
4000
6000
8000
10000
0
10
20
30
40
50
60
70
Blastula
Ectoderm
Neural
Neural Crest

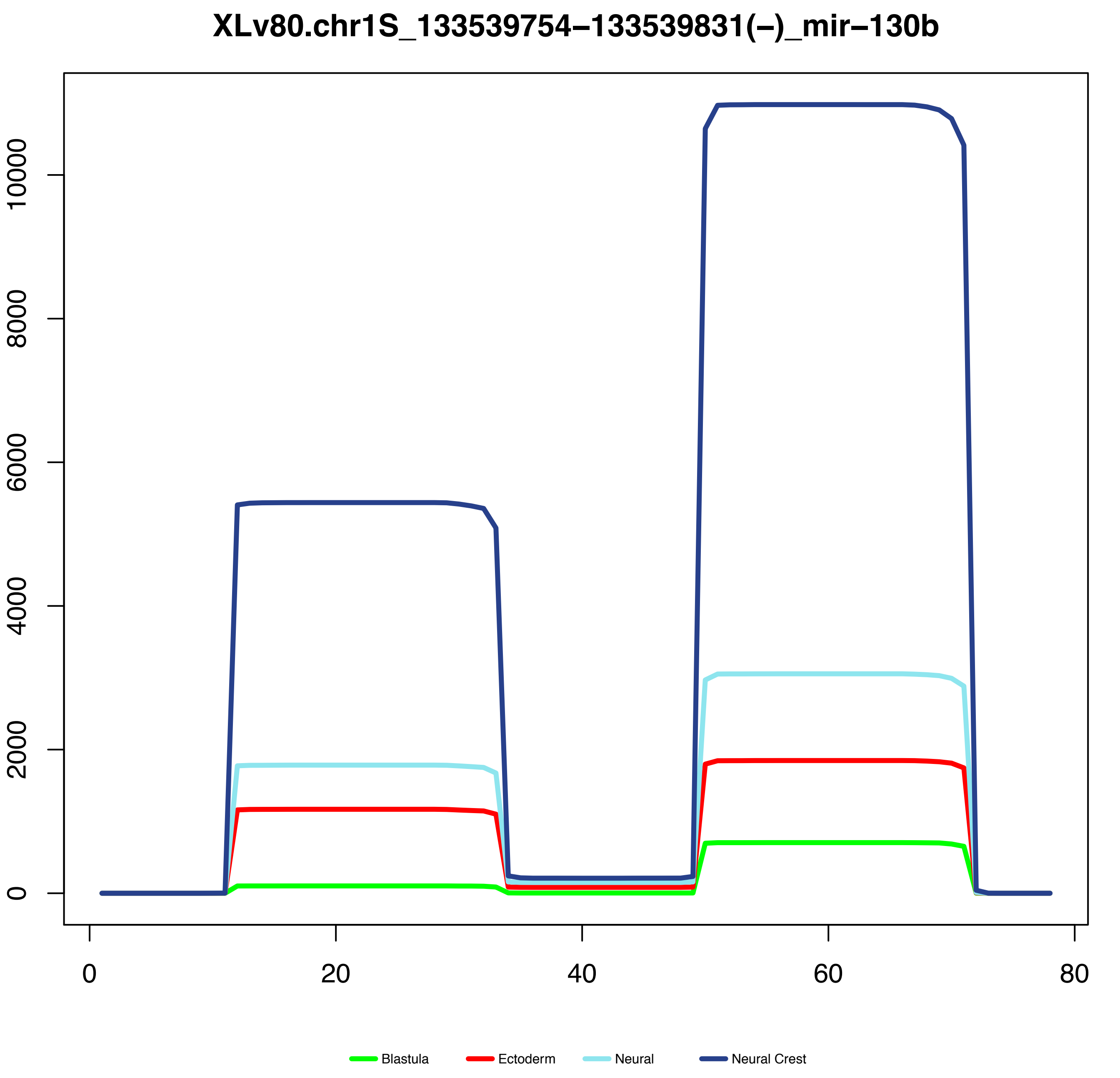
XLv80.chr1S_133539754−133539831(−)_mir−130b
0
2000
4000
6000
8000
10000
0
20
40
60
80
Blastula
Ectoderm
Neural
Neural Crest

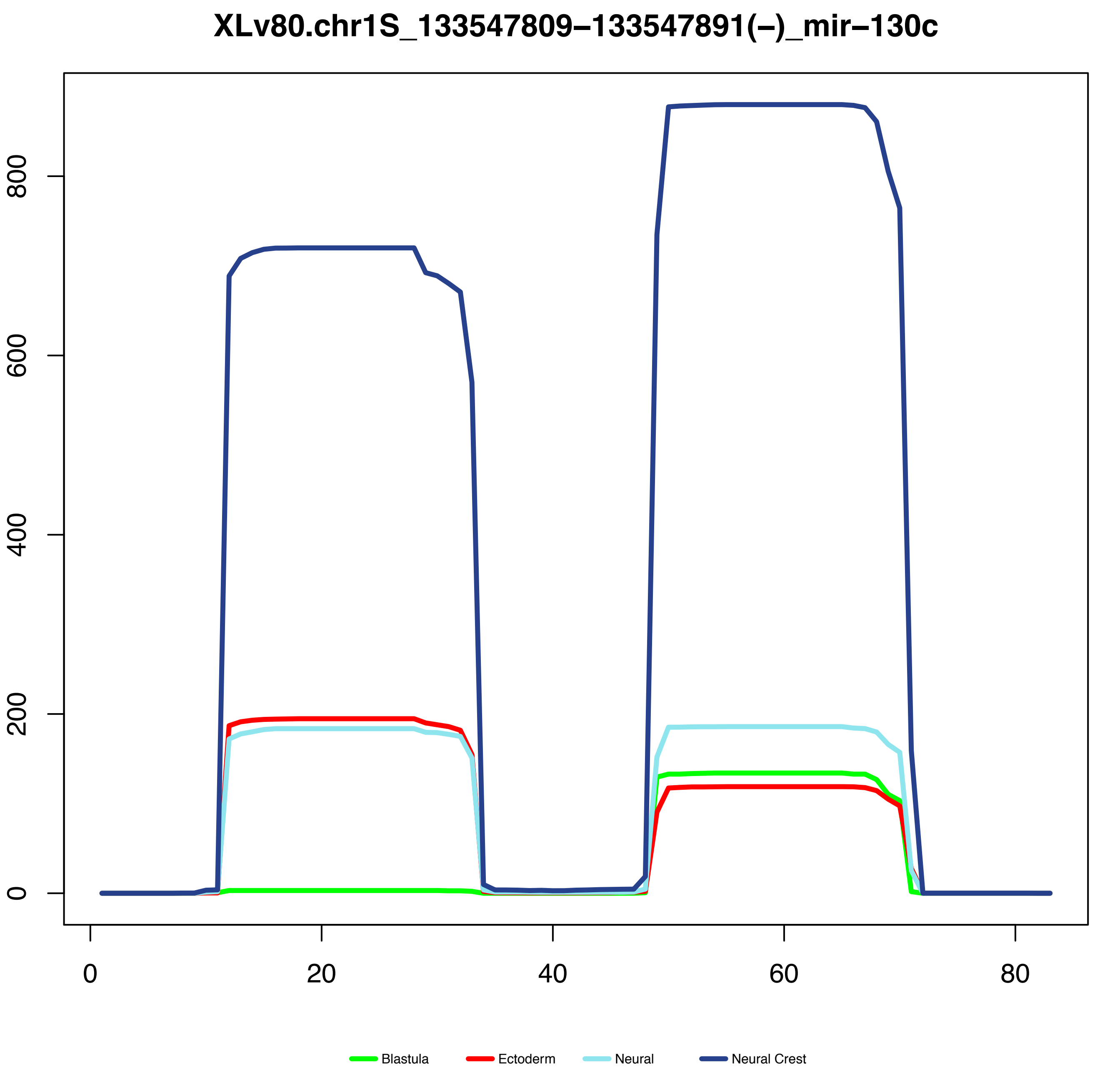

XLv80.chr1S_133547809−133547891(−)_mir−130c
0
200
400
600
800
0
20
40
60
80
Blastula
Ectoderm
Neural
Neural Crest

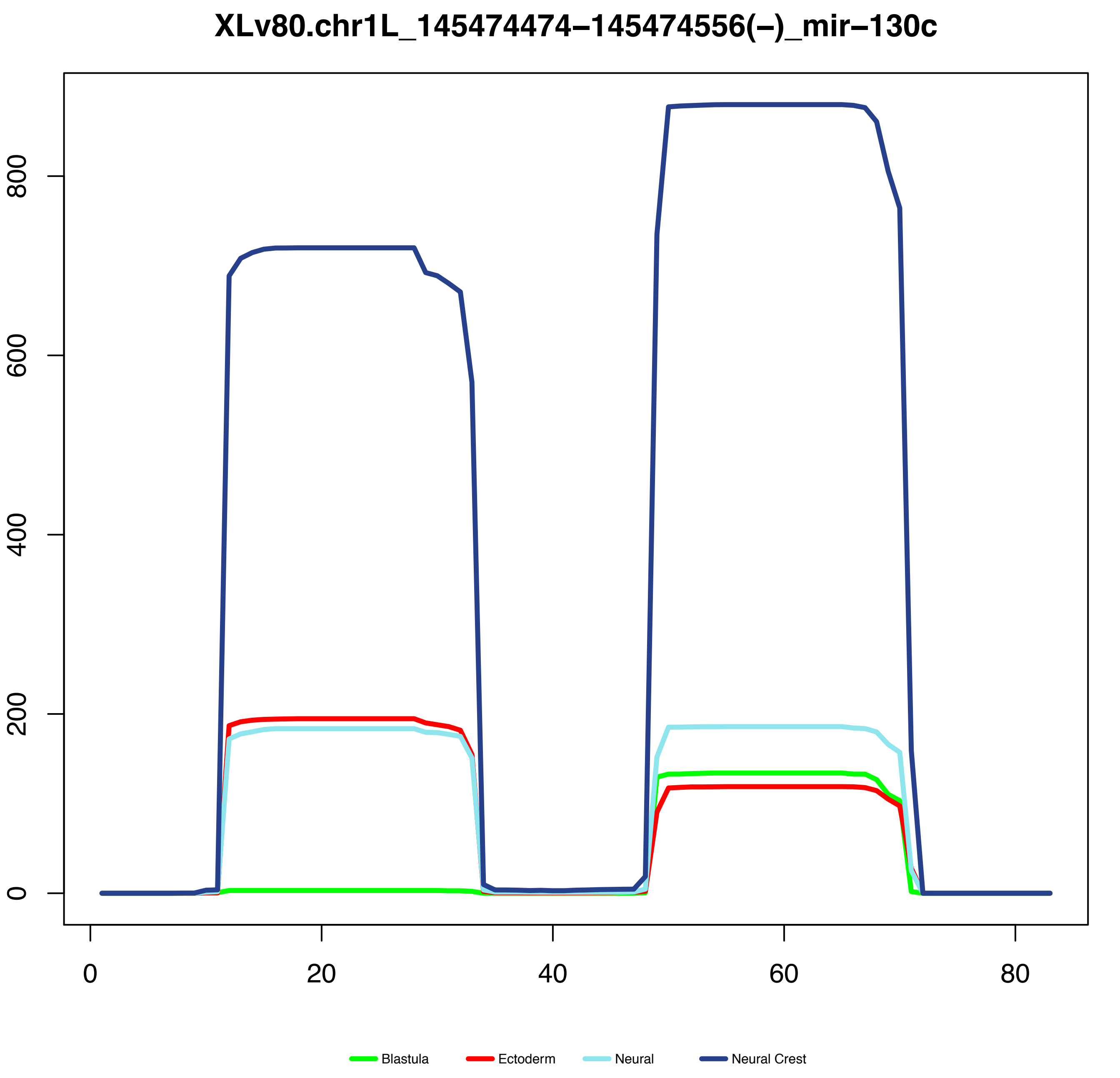
XLv80.chr1L_145474474−145474556(−)_mir−130c
0
200
400
600
800
0
20
40
60
80
Blastula
Ectoderm
Neural
Neural Crest

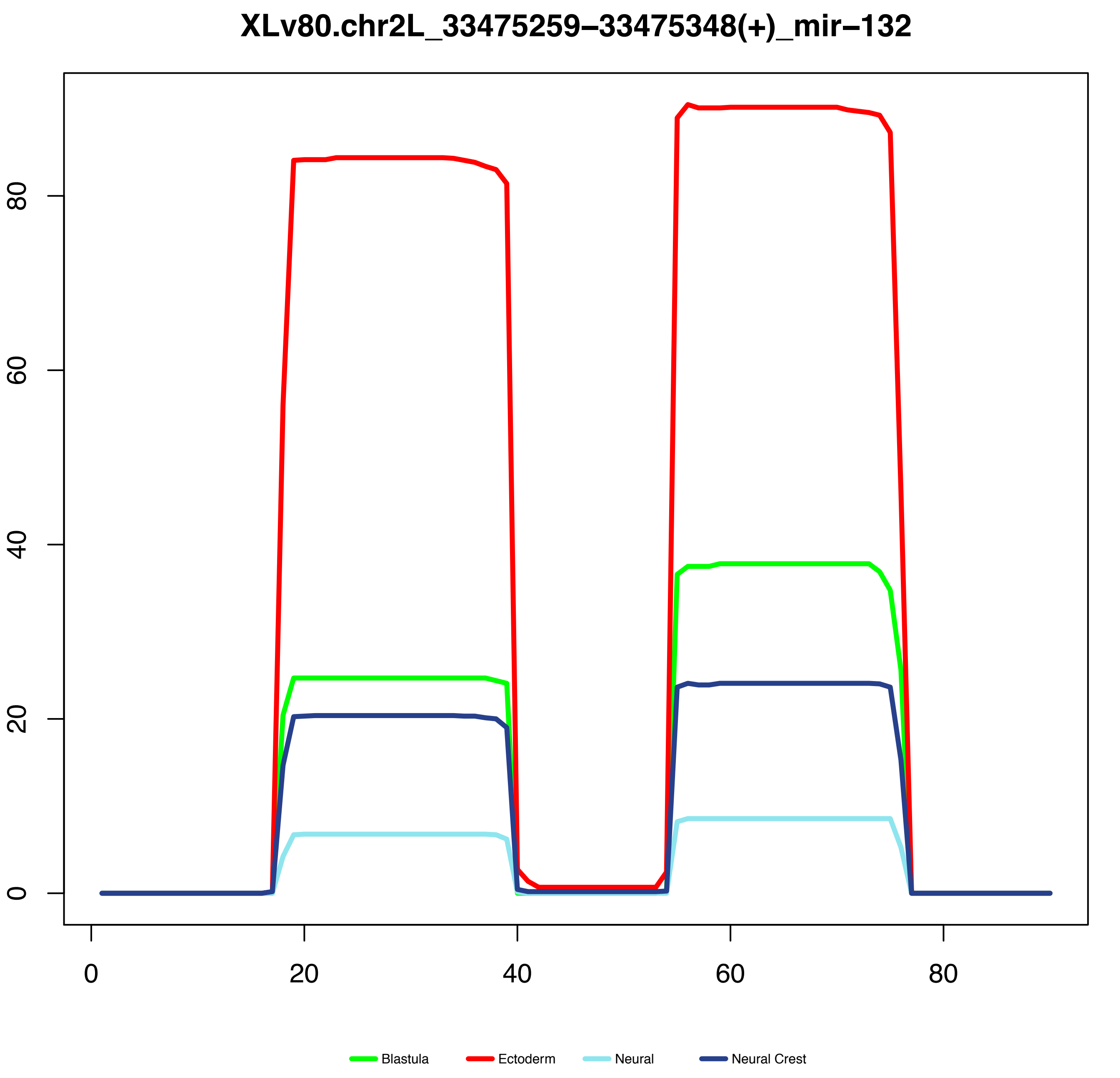

XLv80.chr2L_33475259−33475348(+)_mir−132
0
20
40
60
80
0
20
40
60
80
Blastula
Ectoderm
Neural
Neural Crest

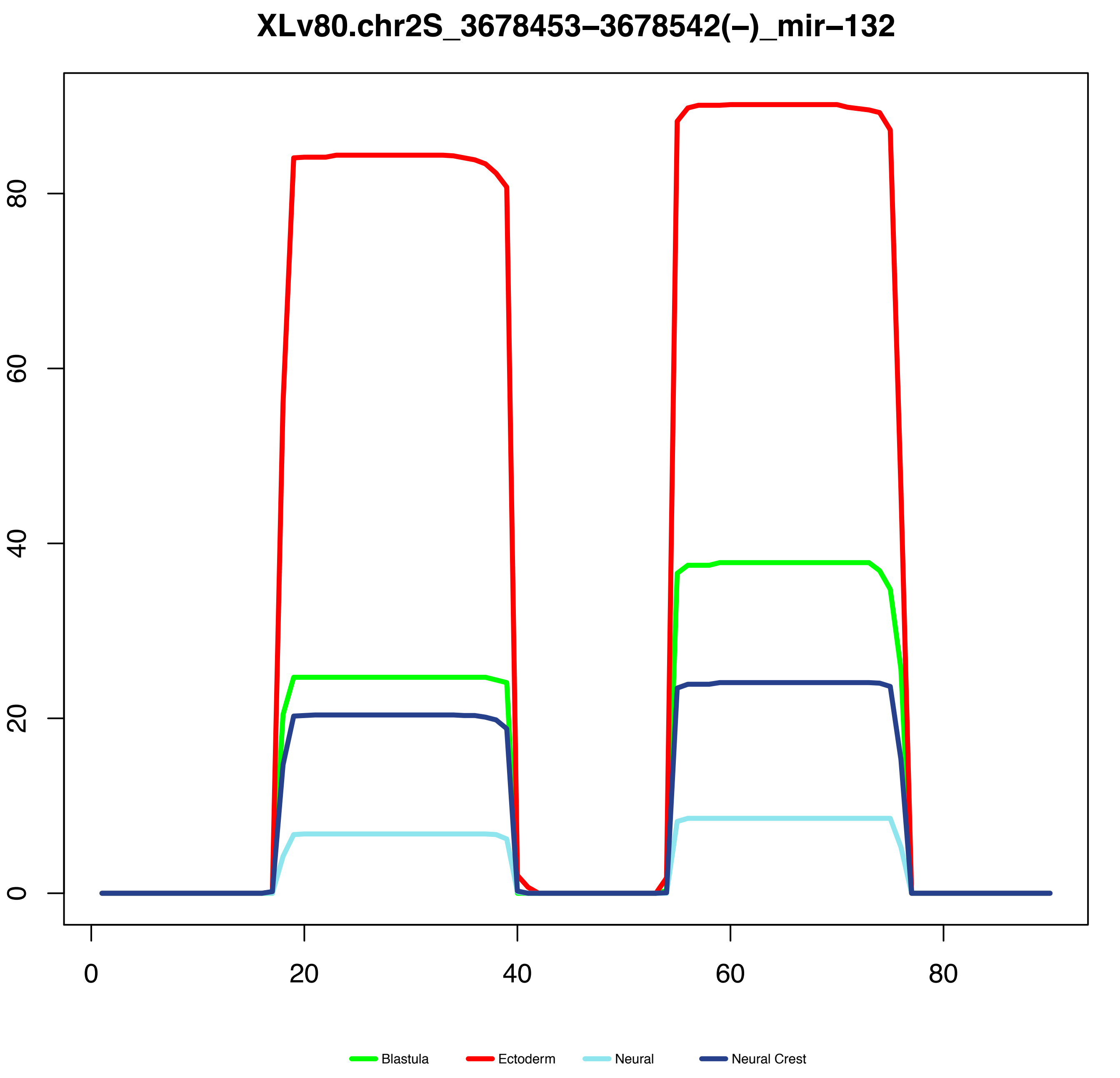
XLv80.chr2S_3678453−3678542(−)_mir−132
0
20
40
60
80
0
20
40
60
80
Blastula
Ectoderm
Neural
Neural Crest

XLv80.chr6L_77383854–77383950(–)_mir–133a–1

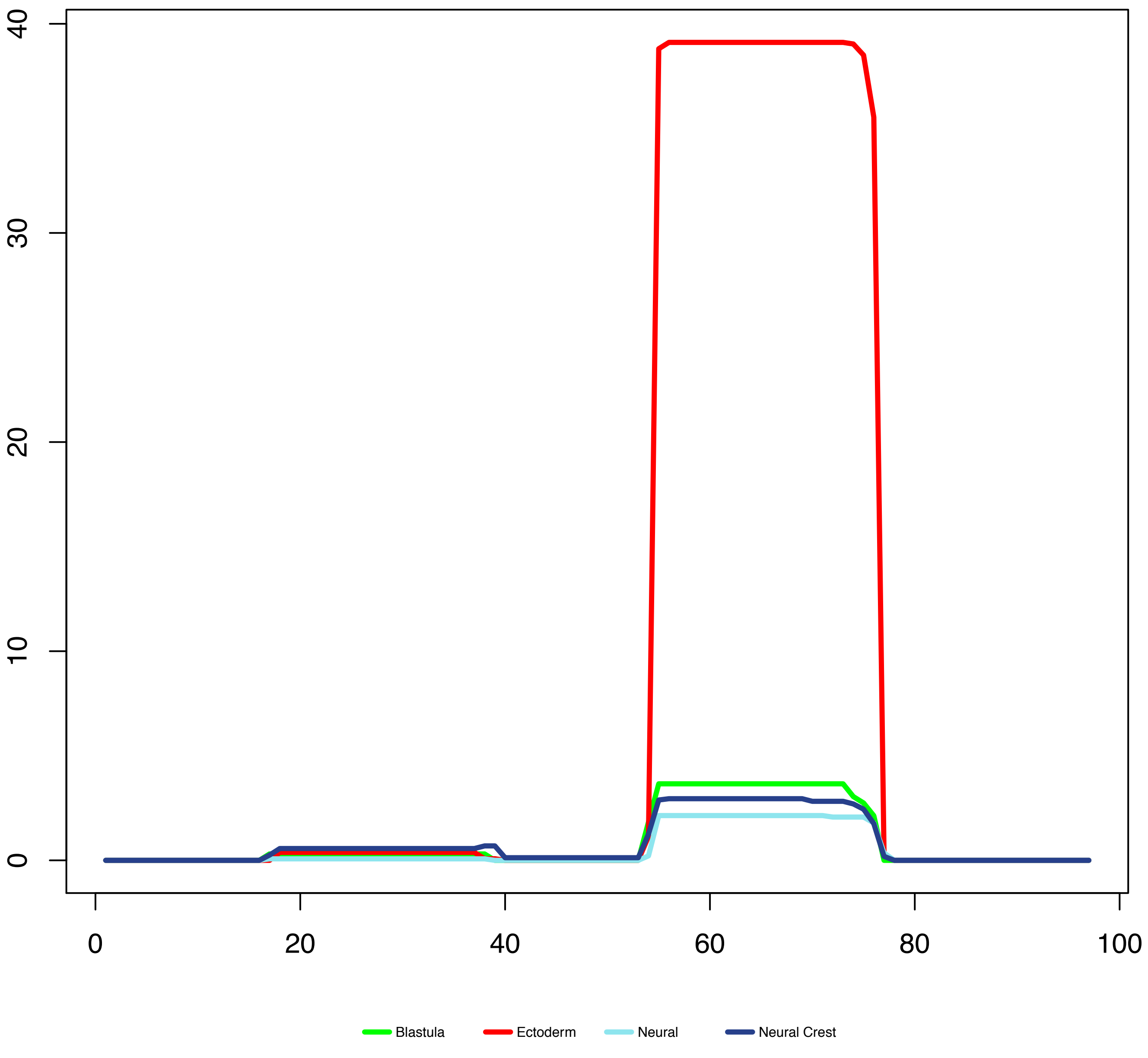

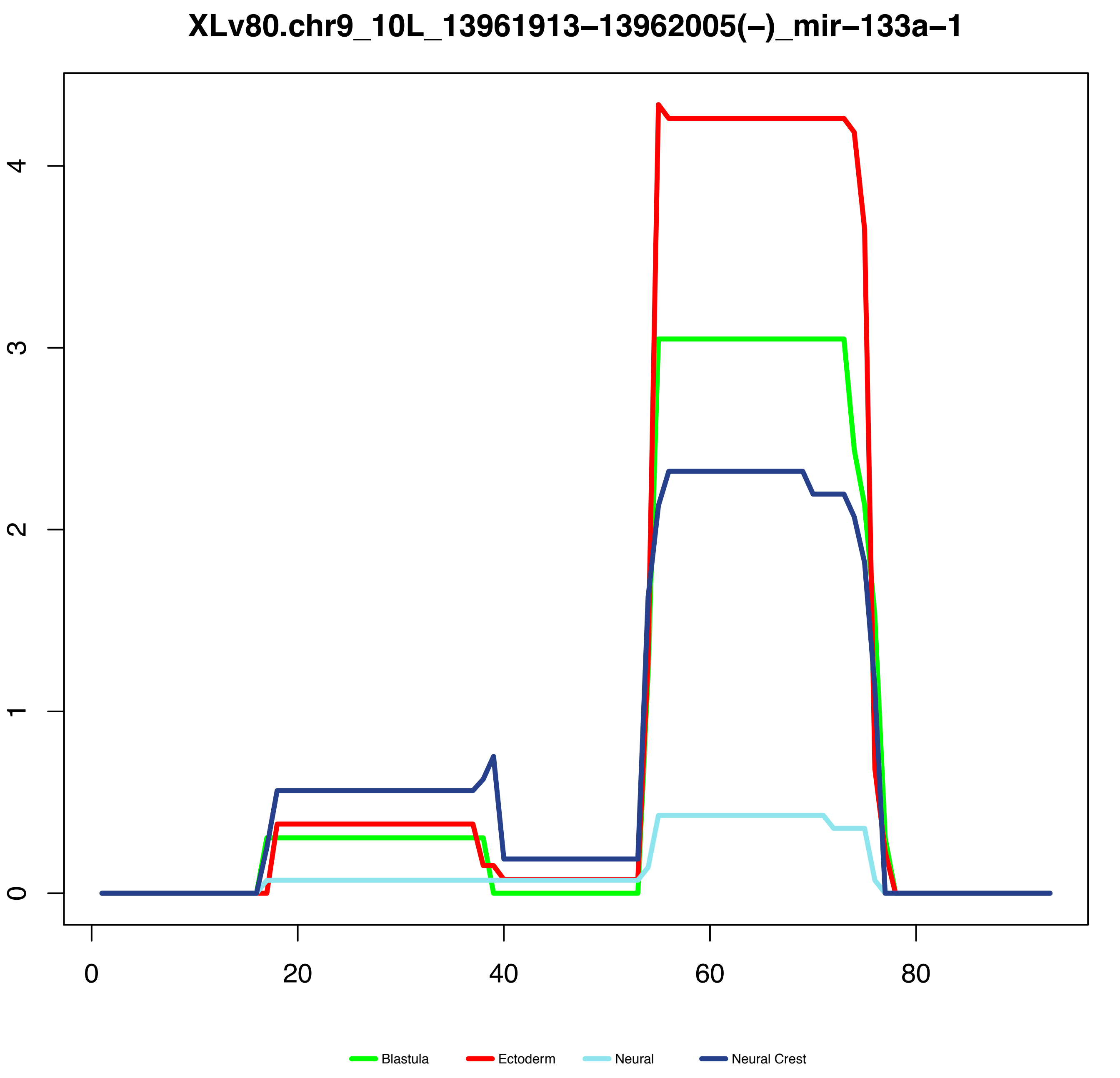
XLv80.chr9_10L_13961913−13962005(−)_mir−133a−1
0
1
2
3
4
0
20
40
60
80
Blastula
Ectoderm
Neural
Neural Crest

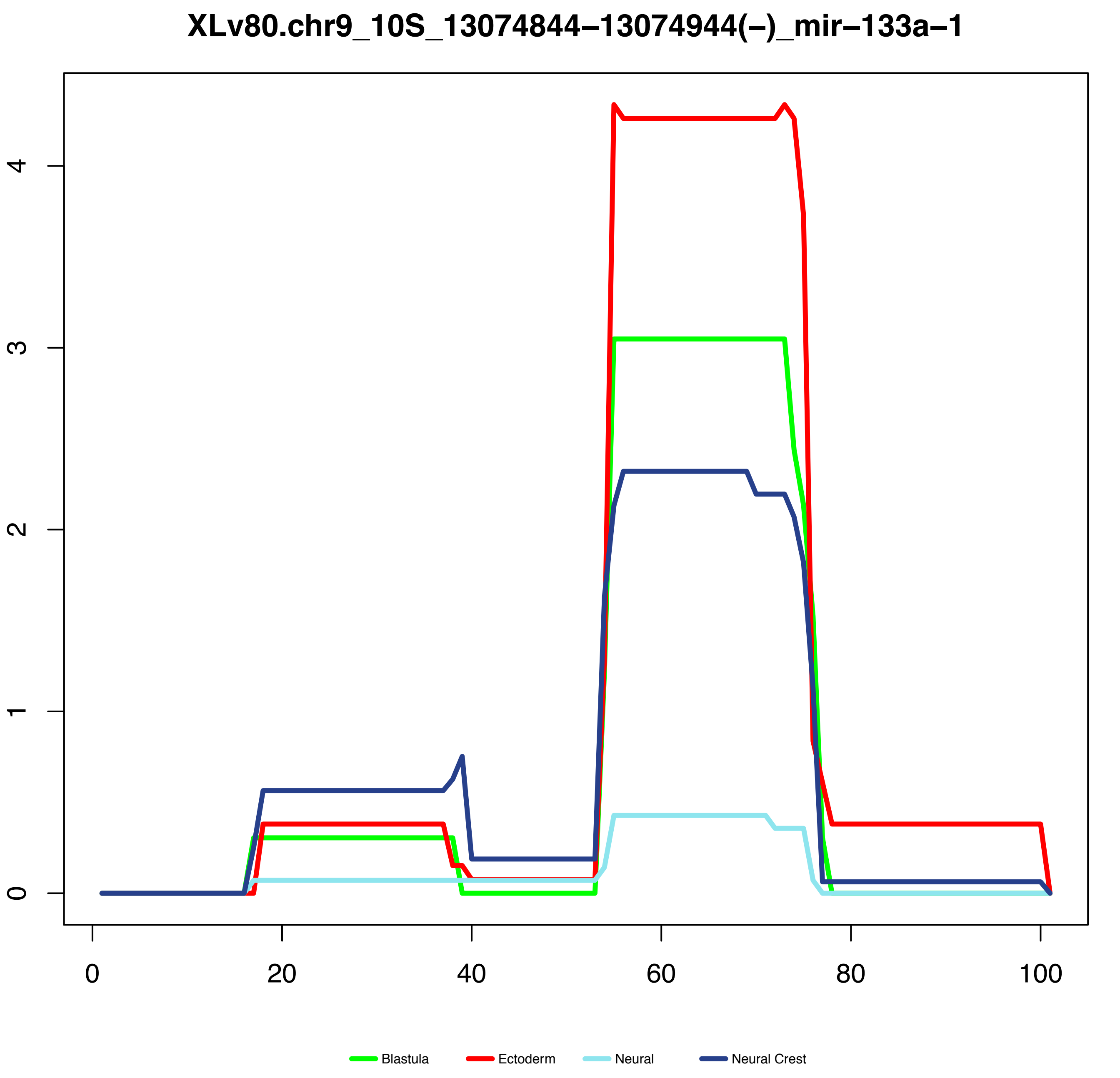
XLv80.chr9_10S_13074844–13074944(–)_mir–133a–1
0
1
2
3
4
0
20
40
60
80
100
Blastula
Ectoderm
Neural
Neural Crest

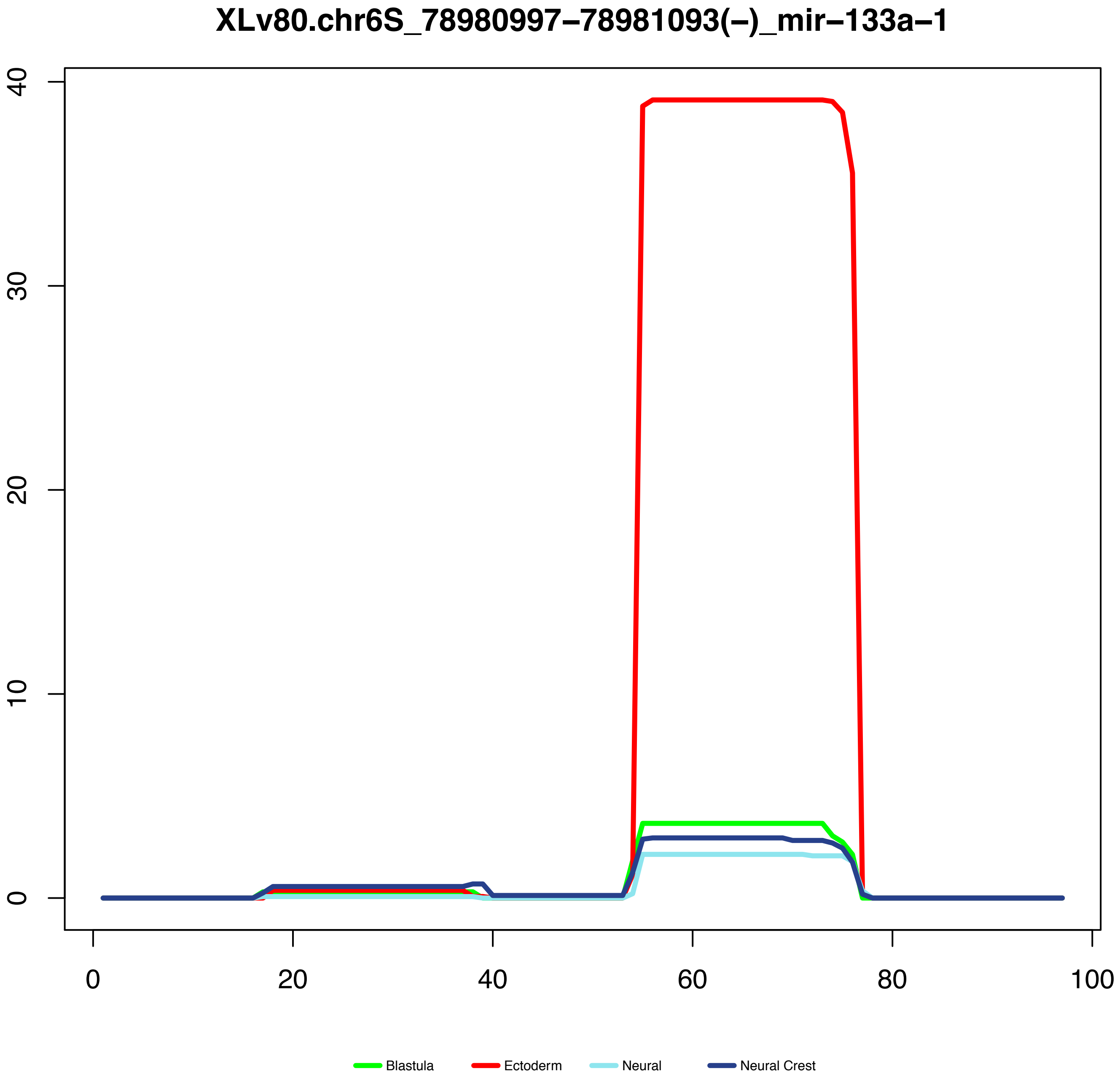
XLv80.chr6S_78980997−78981093(−)_mir−133a−1
0
10
20
30
40
0
20
40
60
80
100
Blastula
Ectoderm
Neural
Neural Crest

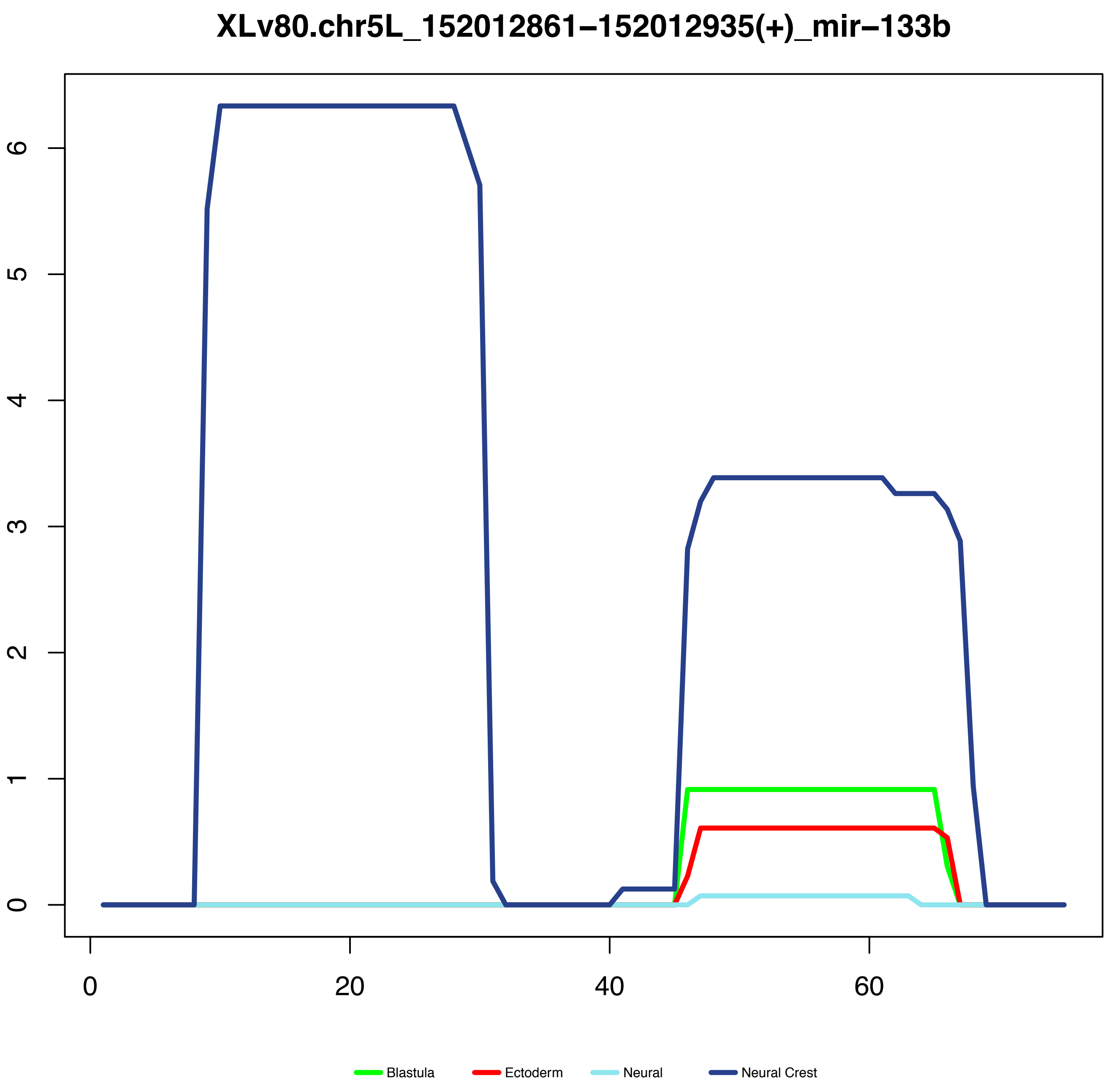

XLv80.chr5L_152012861−152012935(+)_mir−133b
0
1
2
3
4
5
6
0
20
40
60
Blastula
Ectoderm
Neural
Neural Crest

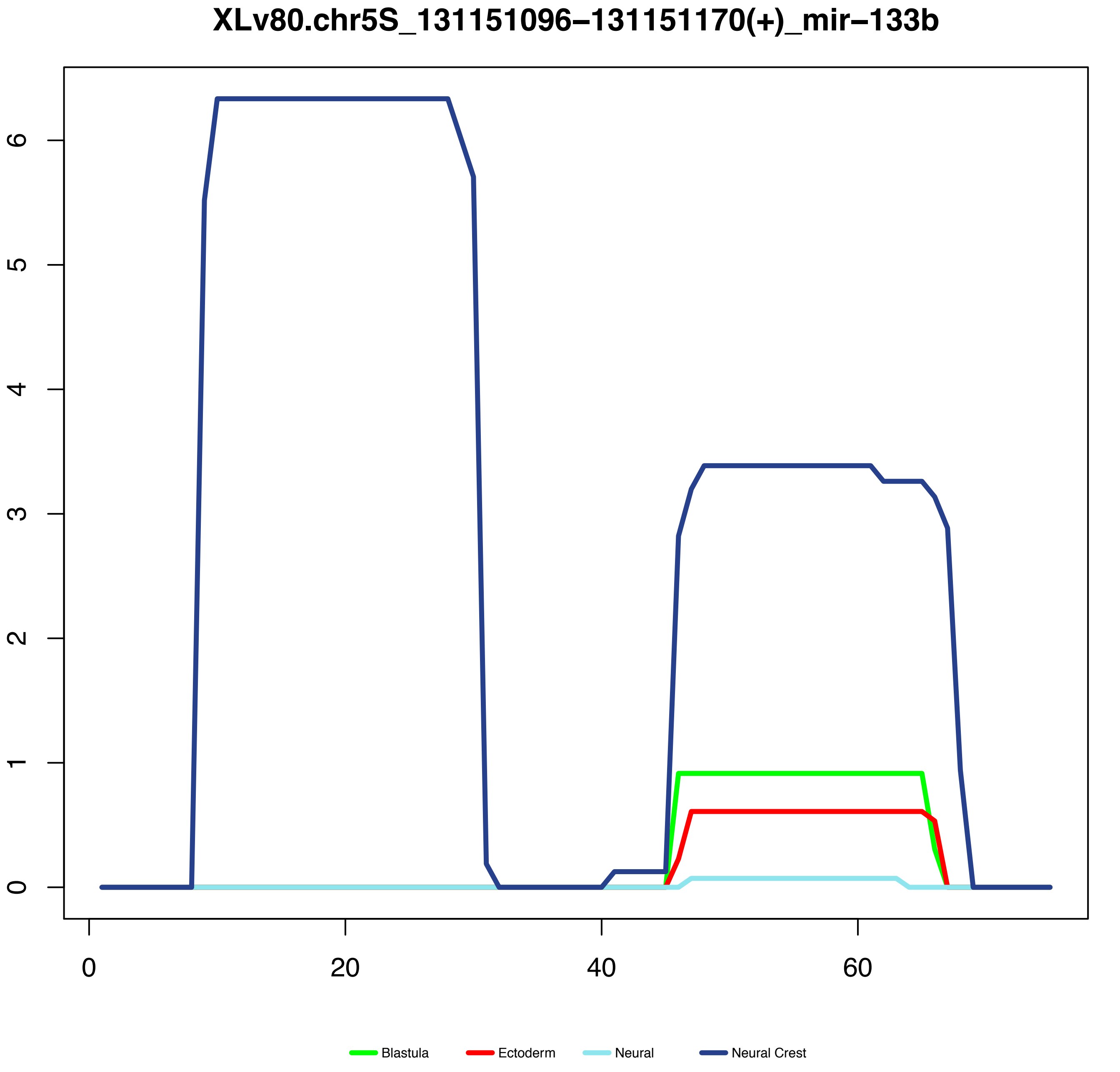

XLv80.chr5S_131151096−131151170(+)_mir−133b
0
1
2
3
4
5
6
0
20
40
60
Blastula
Ectoderm
Neural
Neural Crest

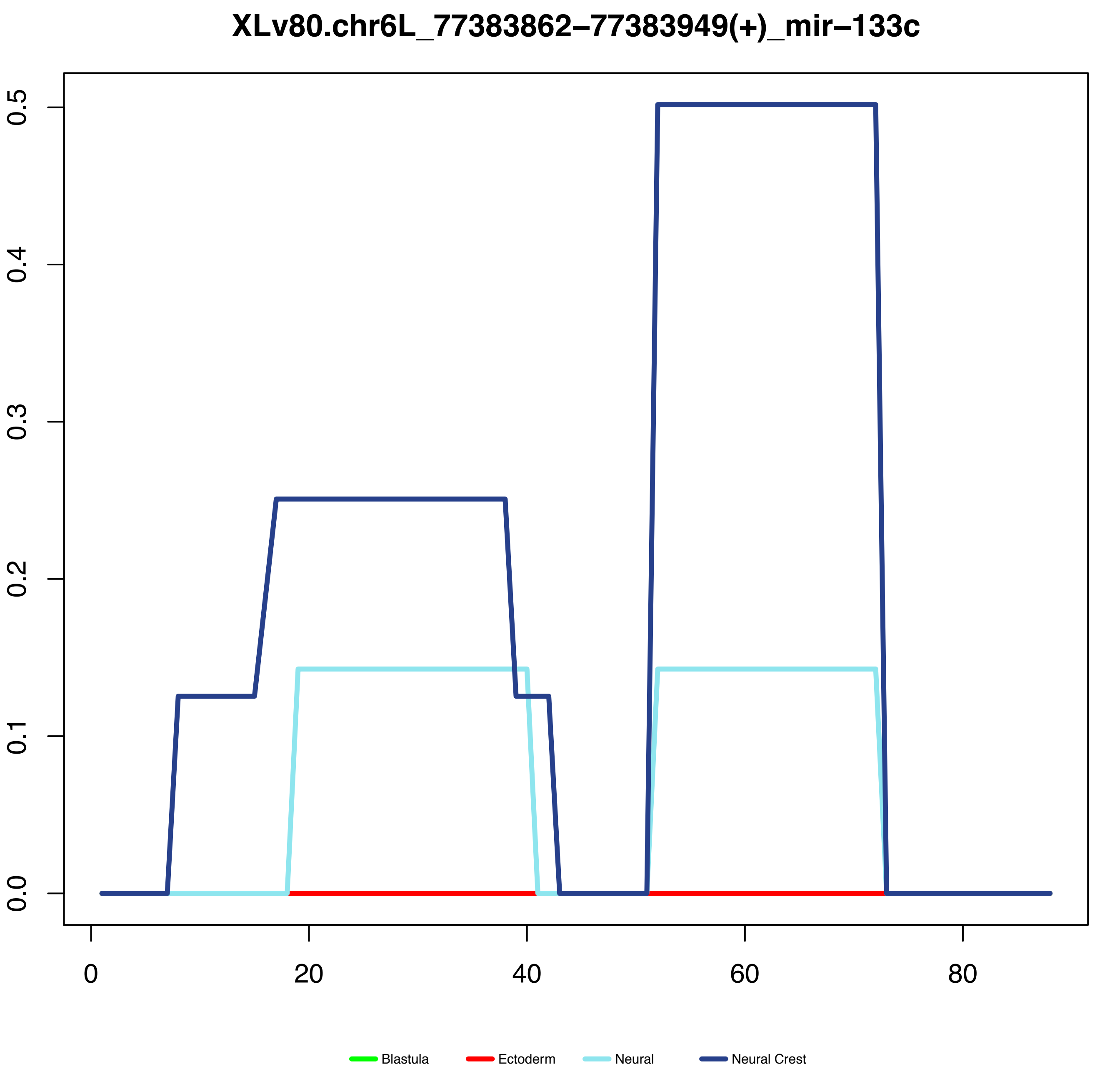
XLv80.chr6L_77383862−77383949(+)_mir−133c
0.0
0.1
0.2
0.3
0.4
0.5
0
20
40
60
80
Blastula
Ectoderm
Neural
Neural Crest

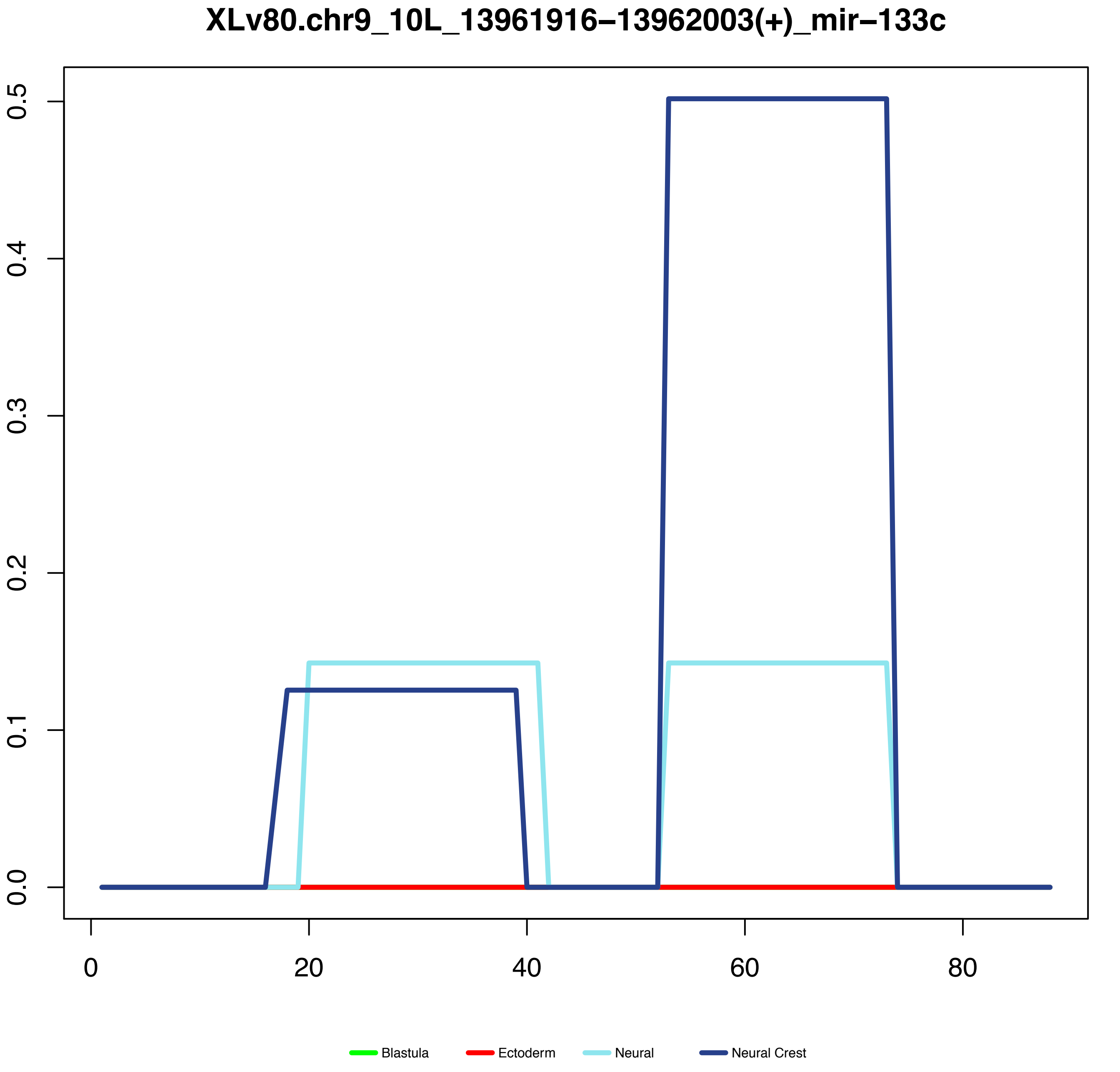
XLv80.chr9_10L_13961916−13962003(+)_mir−133c
0.0
0.1
0.2
0.3
0.4
0.5
0
20
40
60
80
Blastula
Ectoderm
Neural
Neural Crest

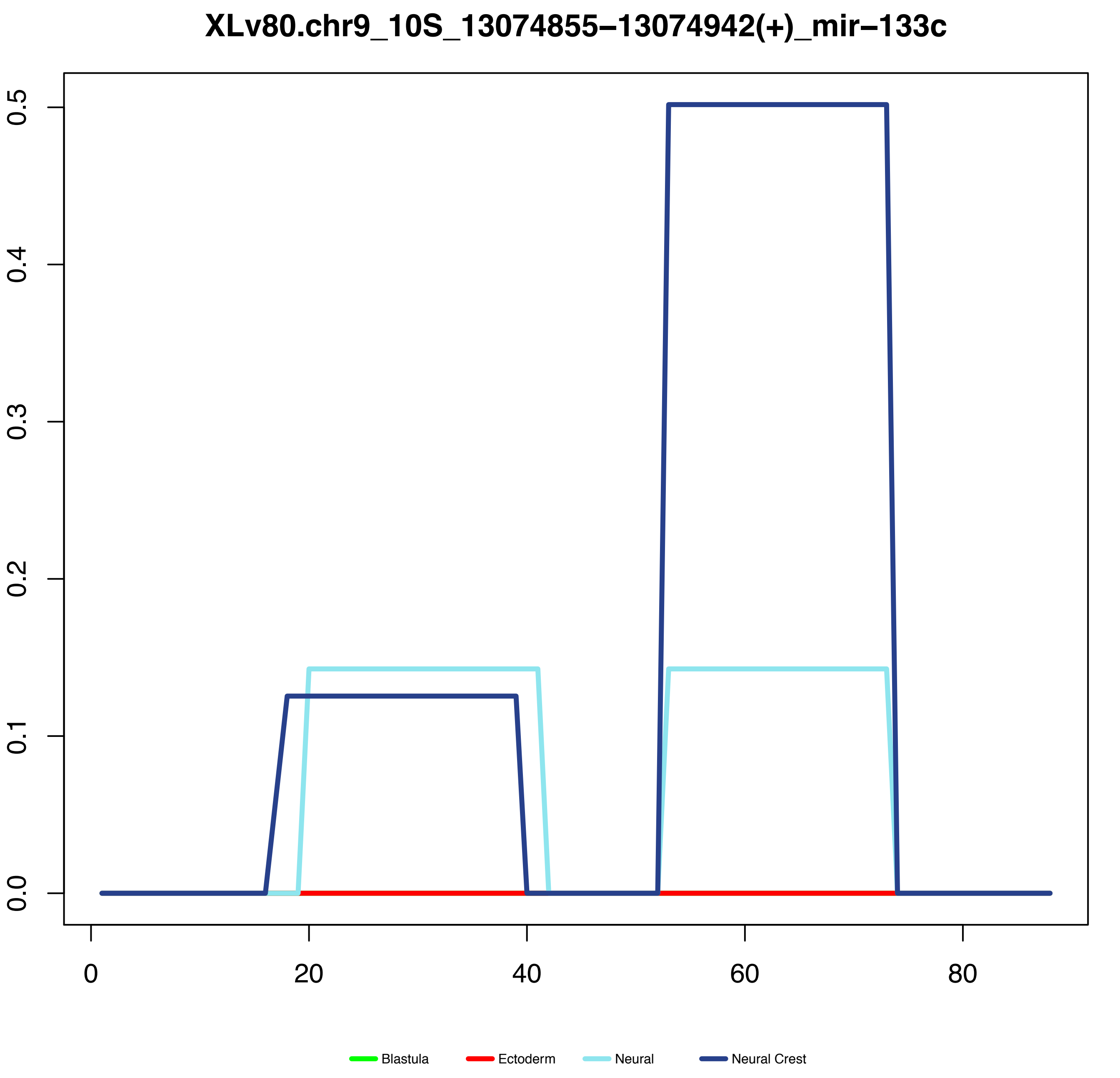
XLv80.chr9_10S_13074855−13074942(+)_mir−133c
0.0
0.1
0.2
0.3
0.4
0.5
0
20
40
60
80
Blastula
Ectoderm
Neural
Neural Crest

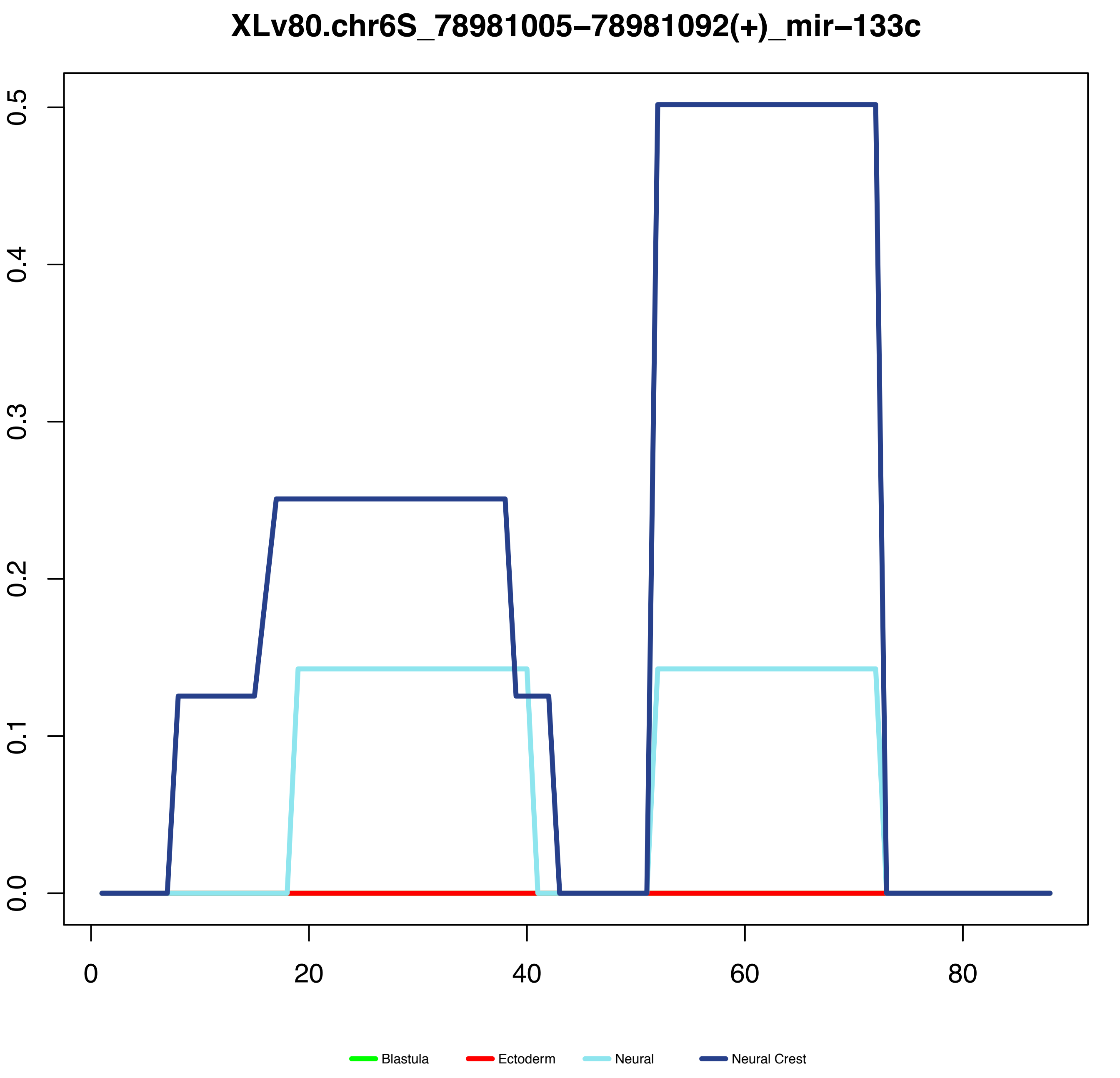

XLv80.chr6S_78981005−78981092(+)_mir−133c
0.0
0.1
0.2
0.3
0.4
0.5
0
20
40
60
80
Blastula
Ectoderm
Neural
Neural Crest

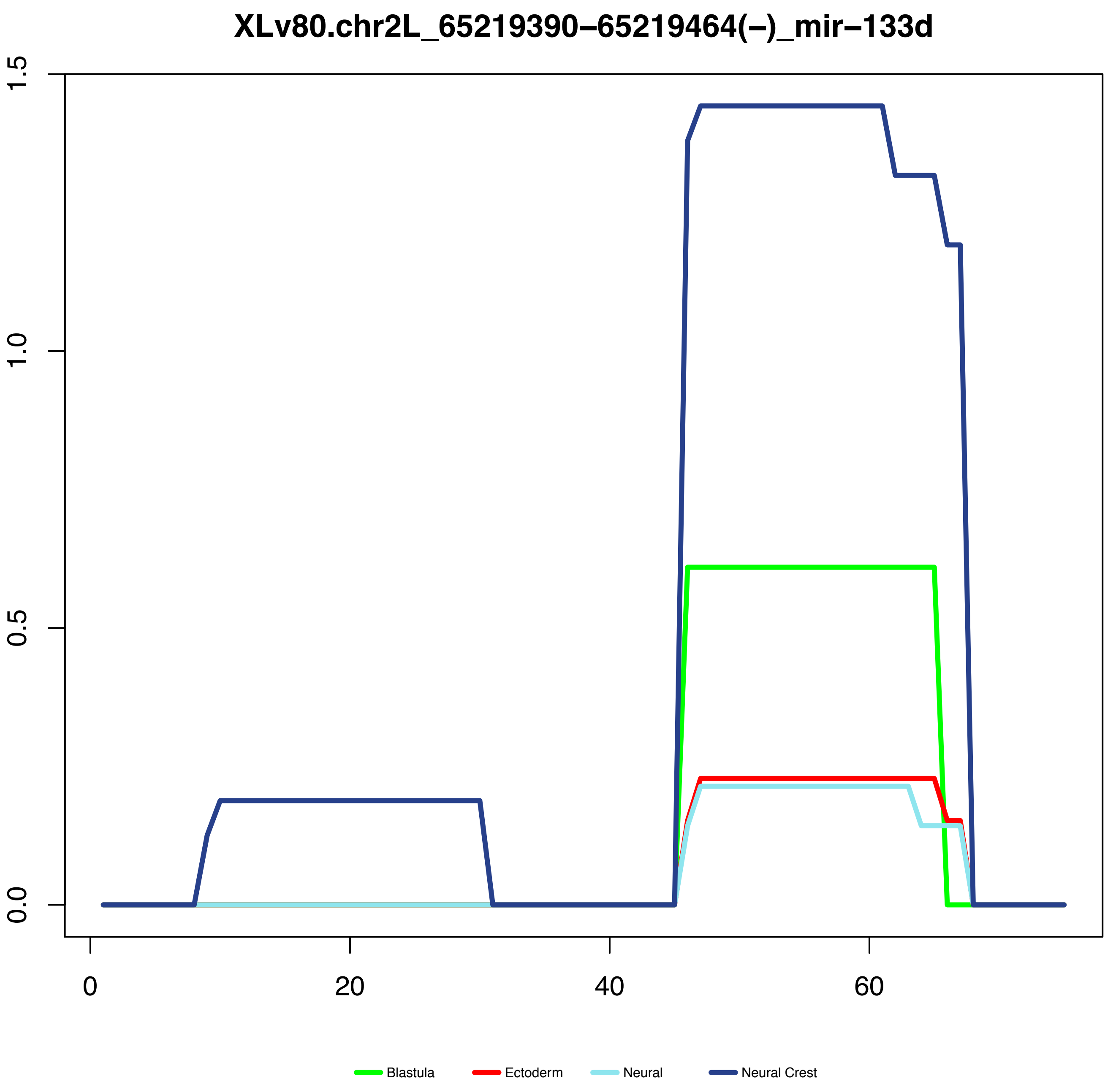
XLv80.chr2L_65219390−65219464(−)_mir−133d
0.0
0.5
1.0
1.5
0
20
40
60
Blastula
Ectoderm
Neural
Neural Crest

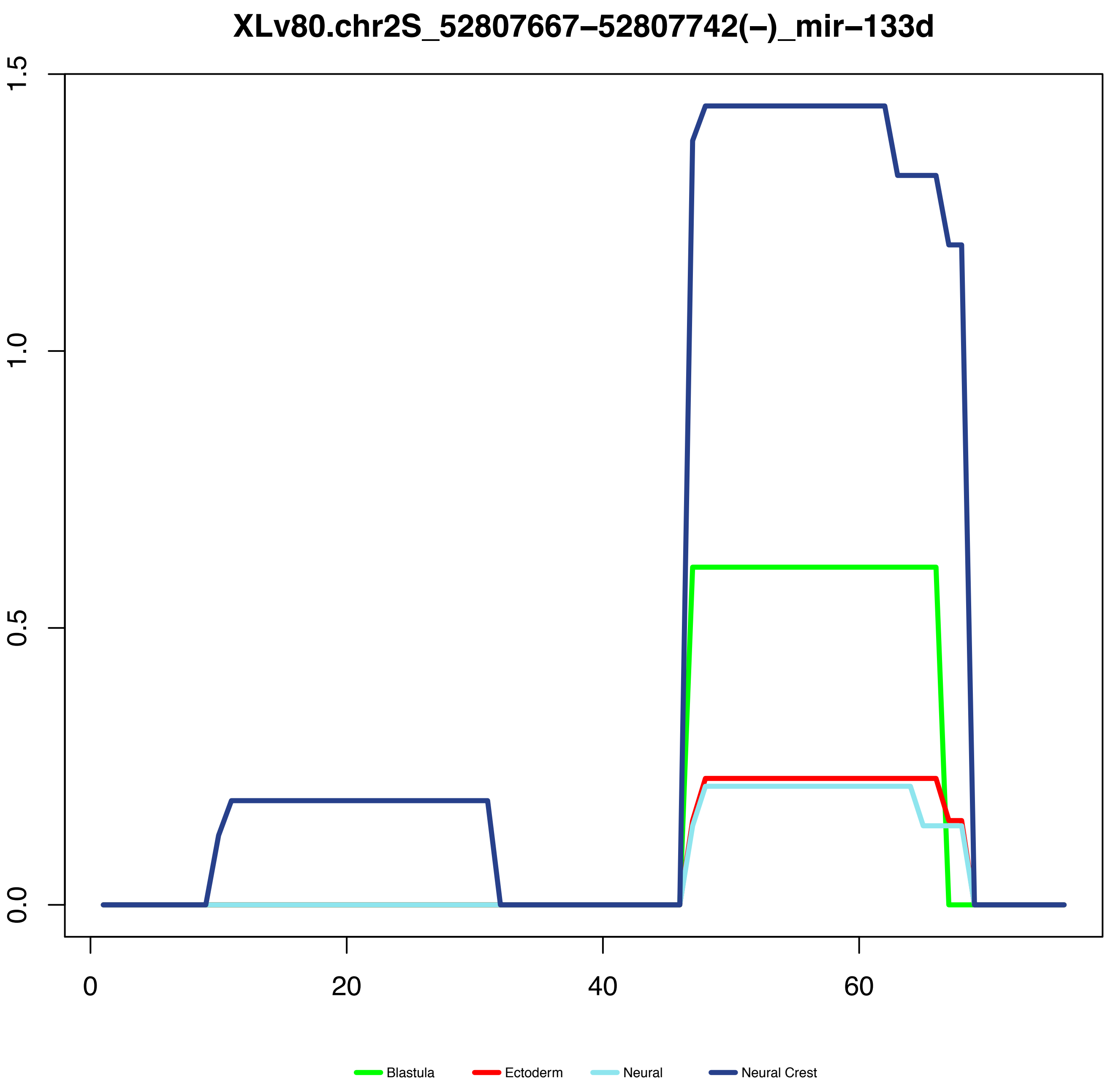

XLv80.chr2S_52807667−52807742(−)_mir−133d
0.0
0.5
1.0
1.5
0
20
40
60
Blastula
Ectoderm
Neural
Neural Crest

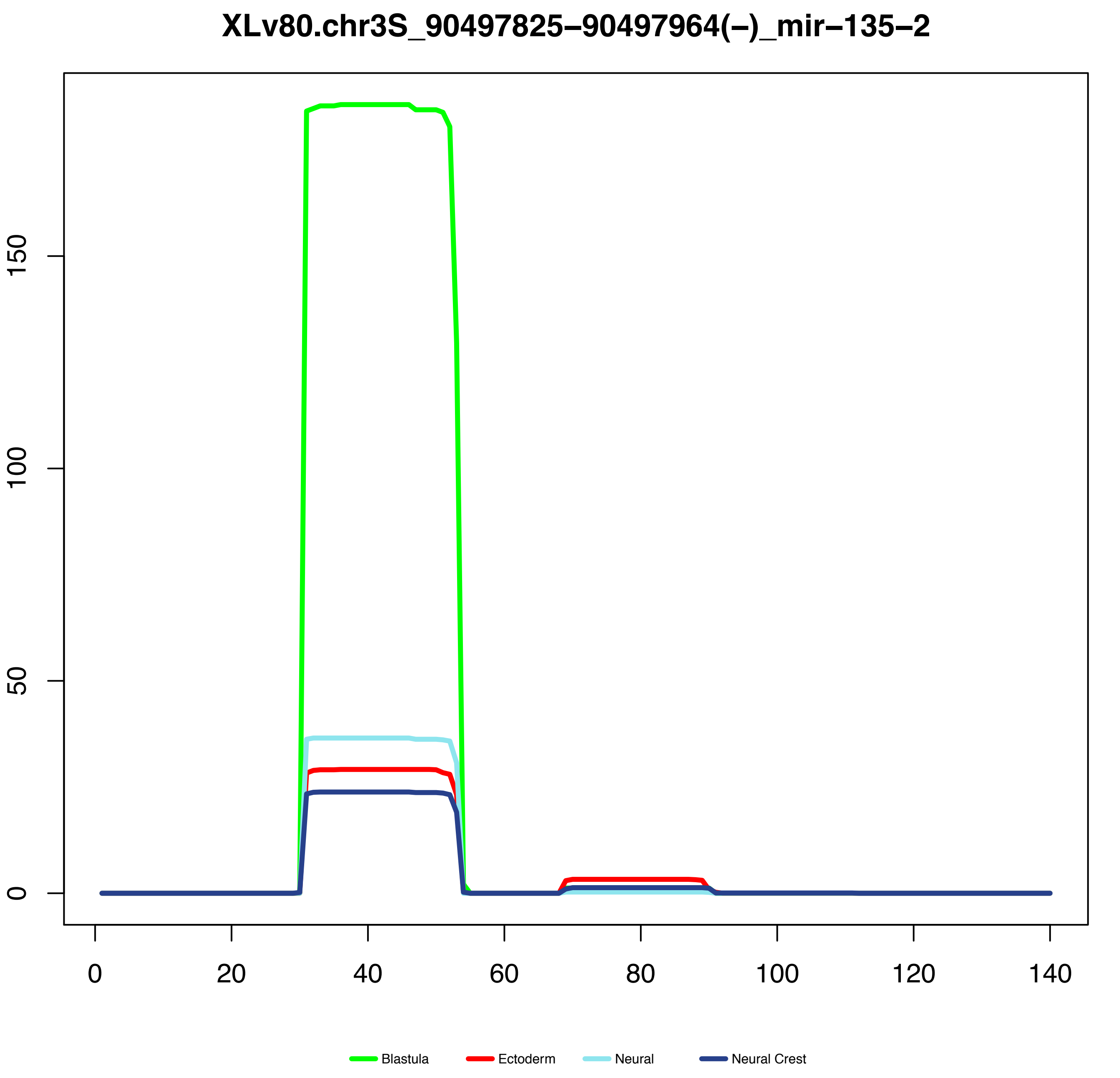

XLv80.chr3S_90497825−90497964(−)_mir−135−2
0
50
100
150
0
20
40
60
80
100
120
140
Blastula
Ectoderm
Neural
Neural Crest

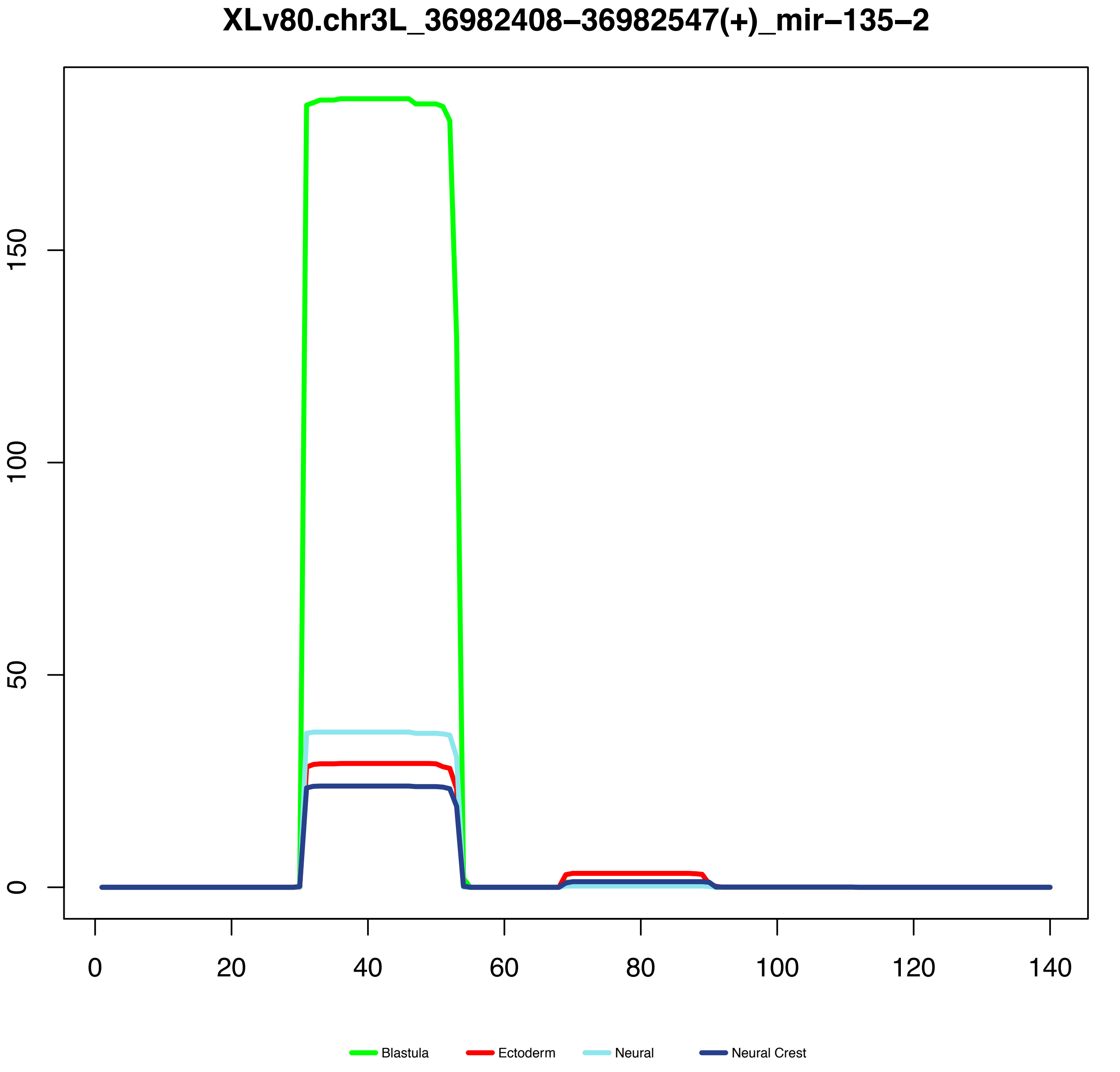
XLv80.chr3L_36982408−36982547(+)_mir−135−2
0
50
100
150
0
20
40
60
80
100
120
140
Blastula
Ectoderm
Neural
Neural Crest

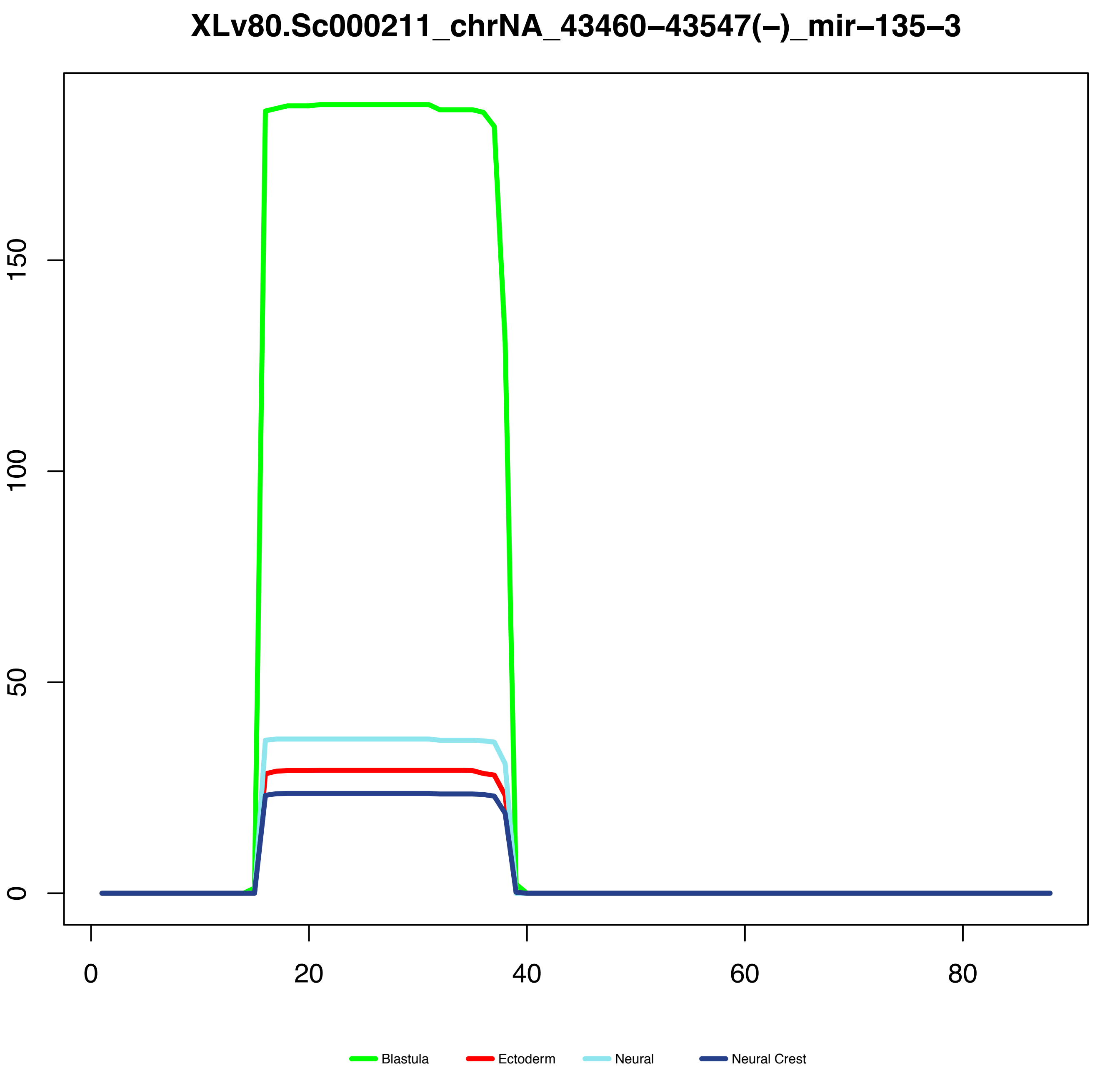
XLv80.Sc000211_chrNA_43460−43547(−)_mir−135−3
0
50
100
150
0
20
40
60
80
Blastula
Ectoderm
Neural
Neural Crest

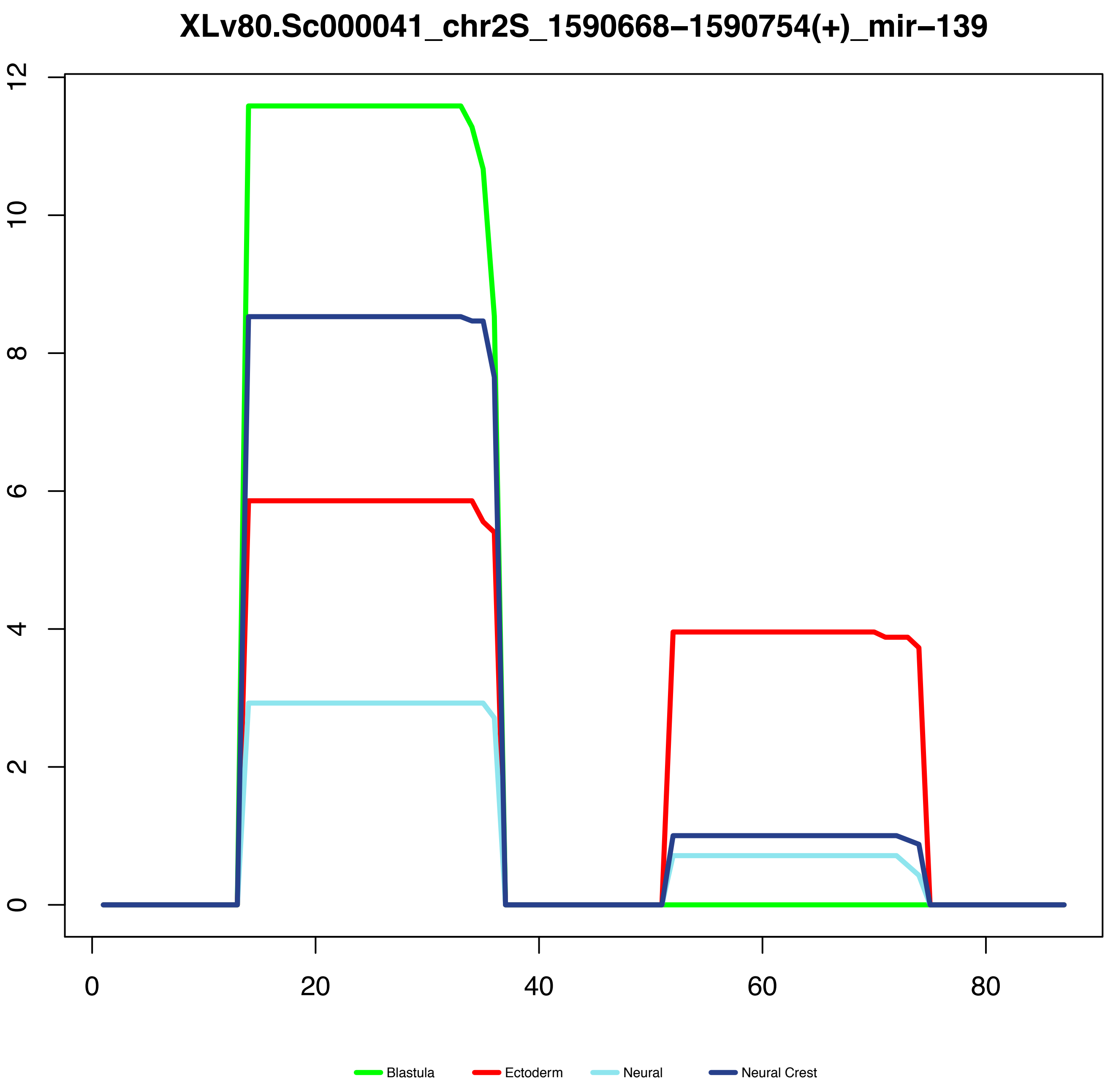

XLv80.Sc000041_chr2S_1590668−1590754(+)_mir−139
0
2
4
6
8
10
12
0
20
40
60
80
Blastula
Ectoderm
Neural
Neural Crest

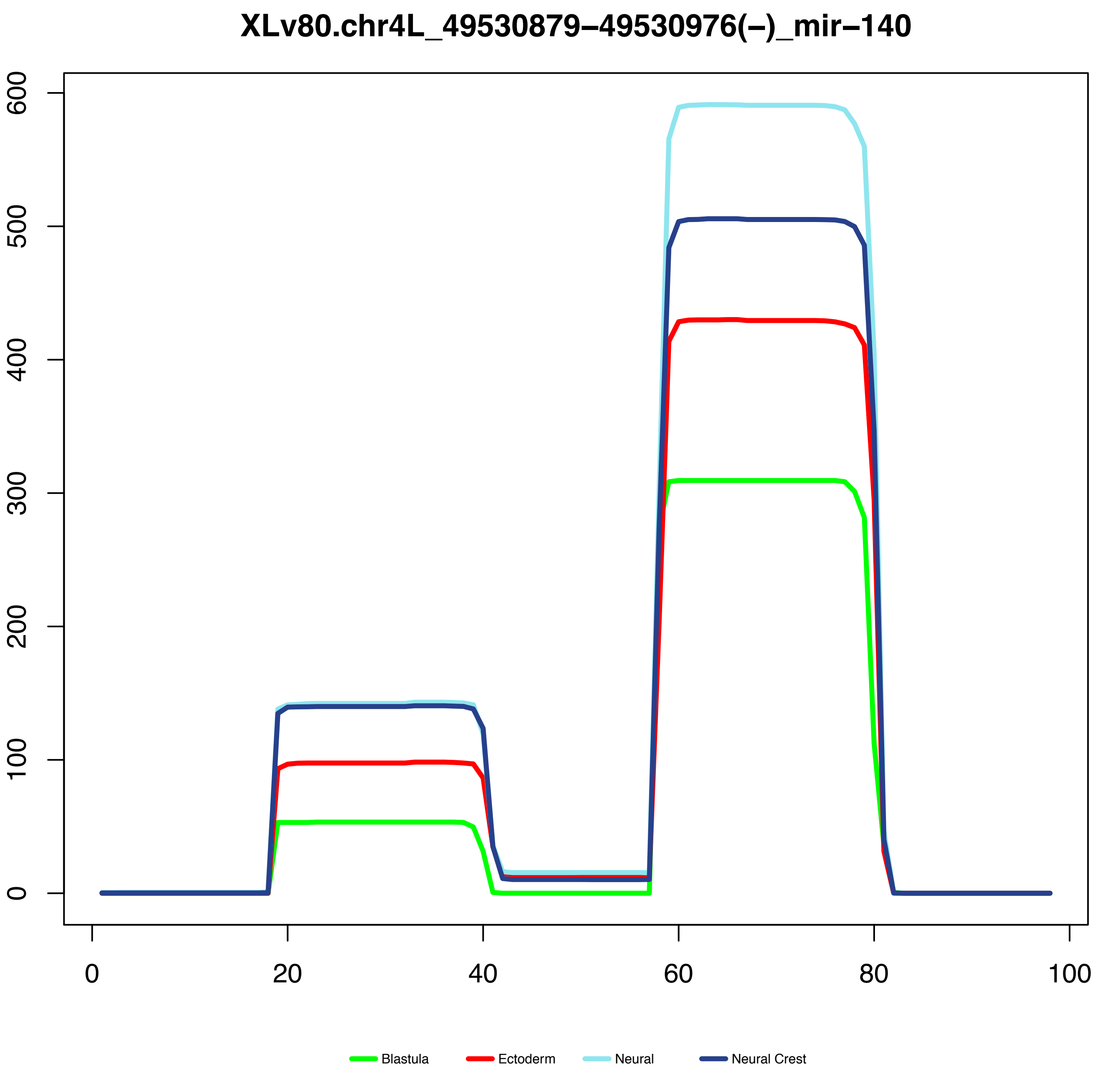
XLv80.chr4L_49530879−49530976(−)_mir−140
0
100
200
300
400
500
600
0
20
40
60
80
100
Blastula
Ectoderm
Neural
Neural Crest

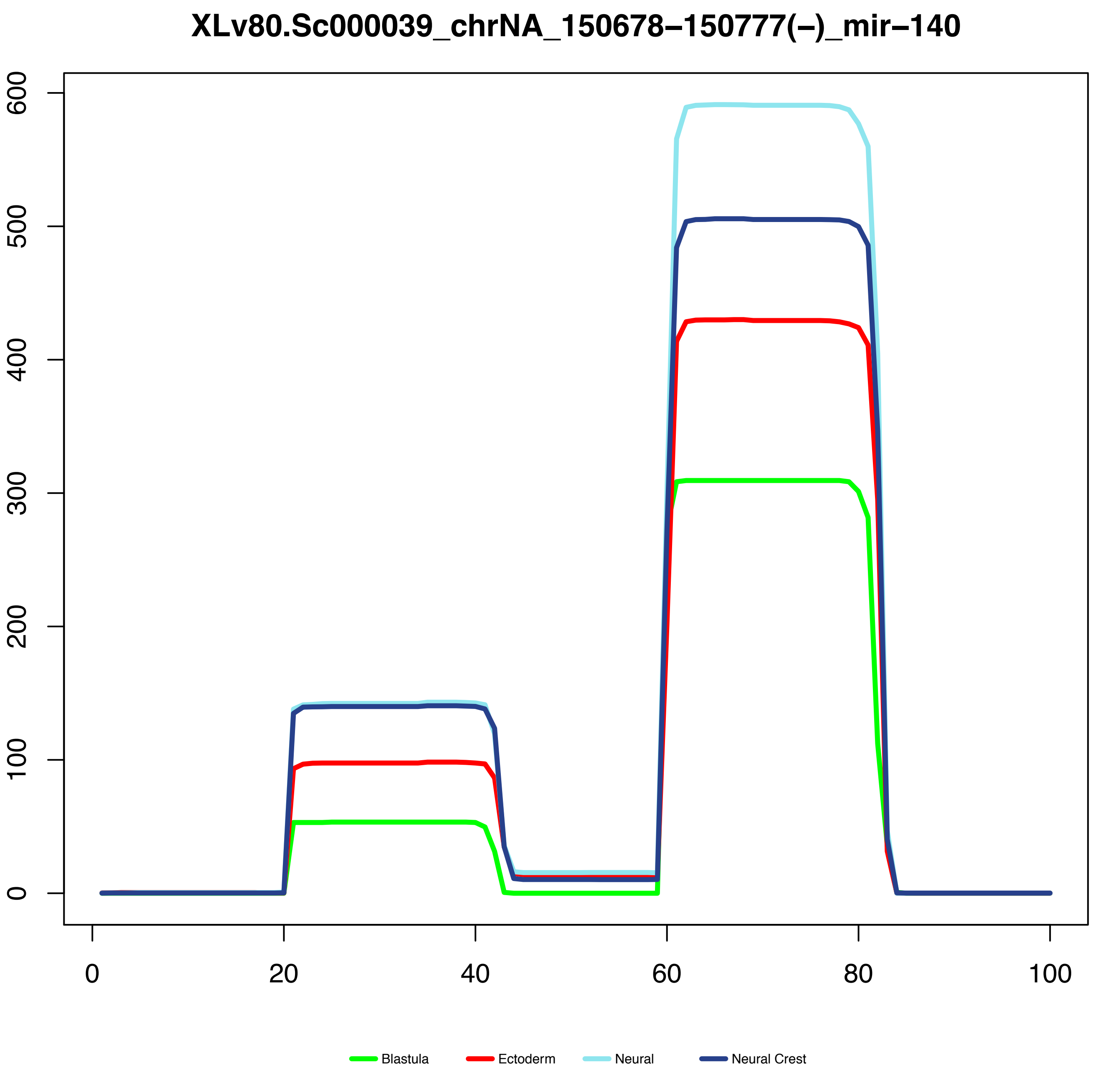
XLv80.Sc000039_chrNA_150678−150777(−)_mir−140
0
100
200
300
400
500
600
0
20
40
60
80
100
Blastula
Ectoderm
Neural
Neural Crest

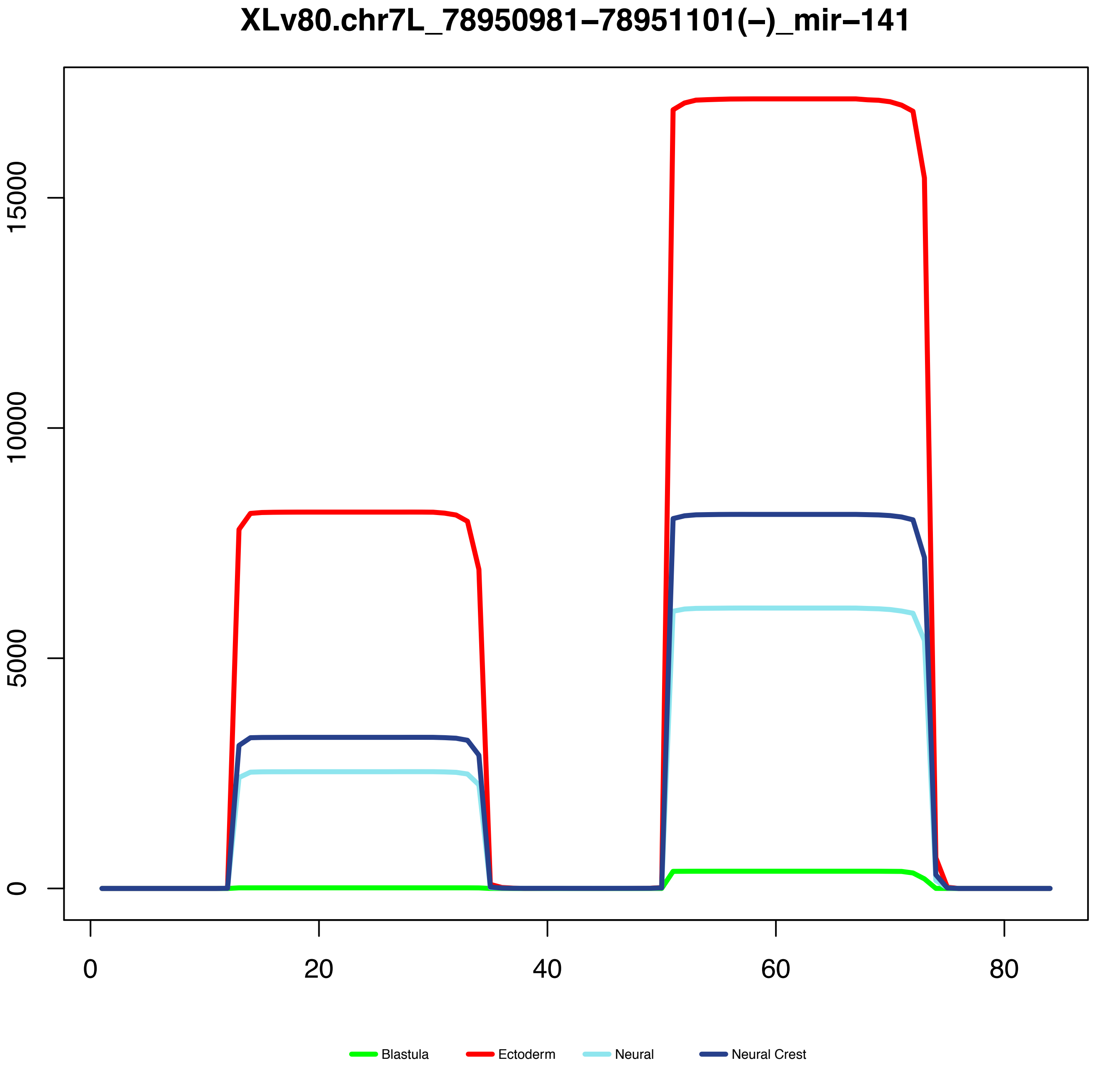

XLv80.chr7L_78950981−78951101(−)_mir−141
0
5000
10000
15000
0
20
40
60
80
Blastula
Ectoderm
Neural
Neural Crest

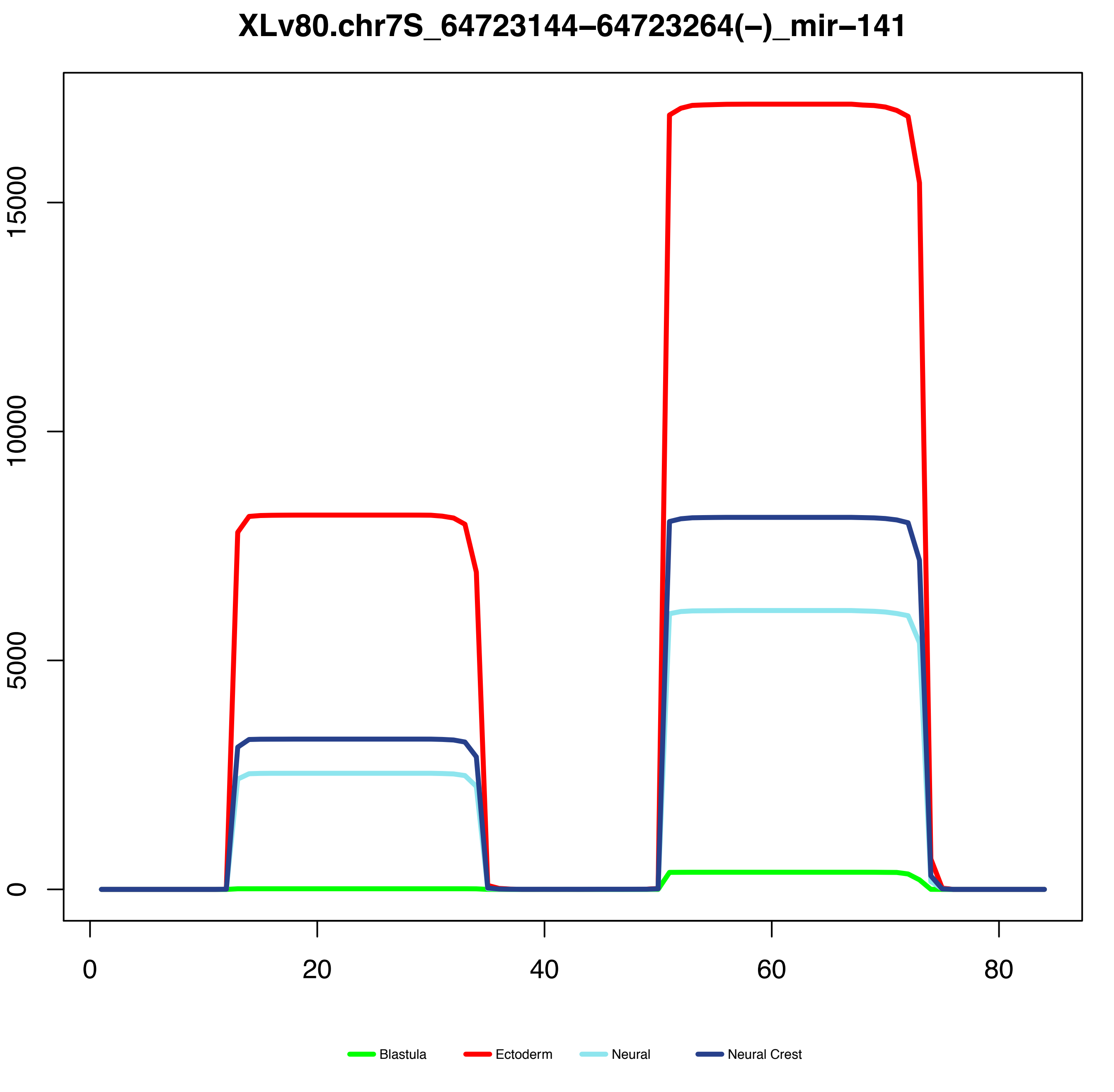

XLv80.chr7S_64723144−64723264(−)_mir−141
0
5000
10000
15000
0
20
40
60
80
Blastula
Ectoderm
Neural
Neural Crest

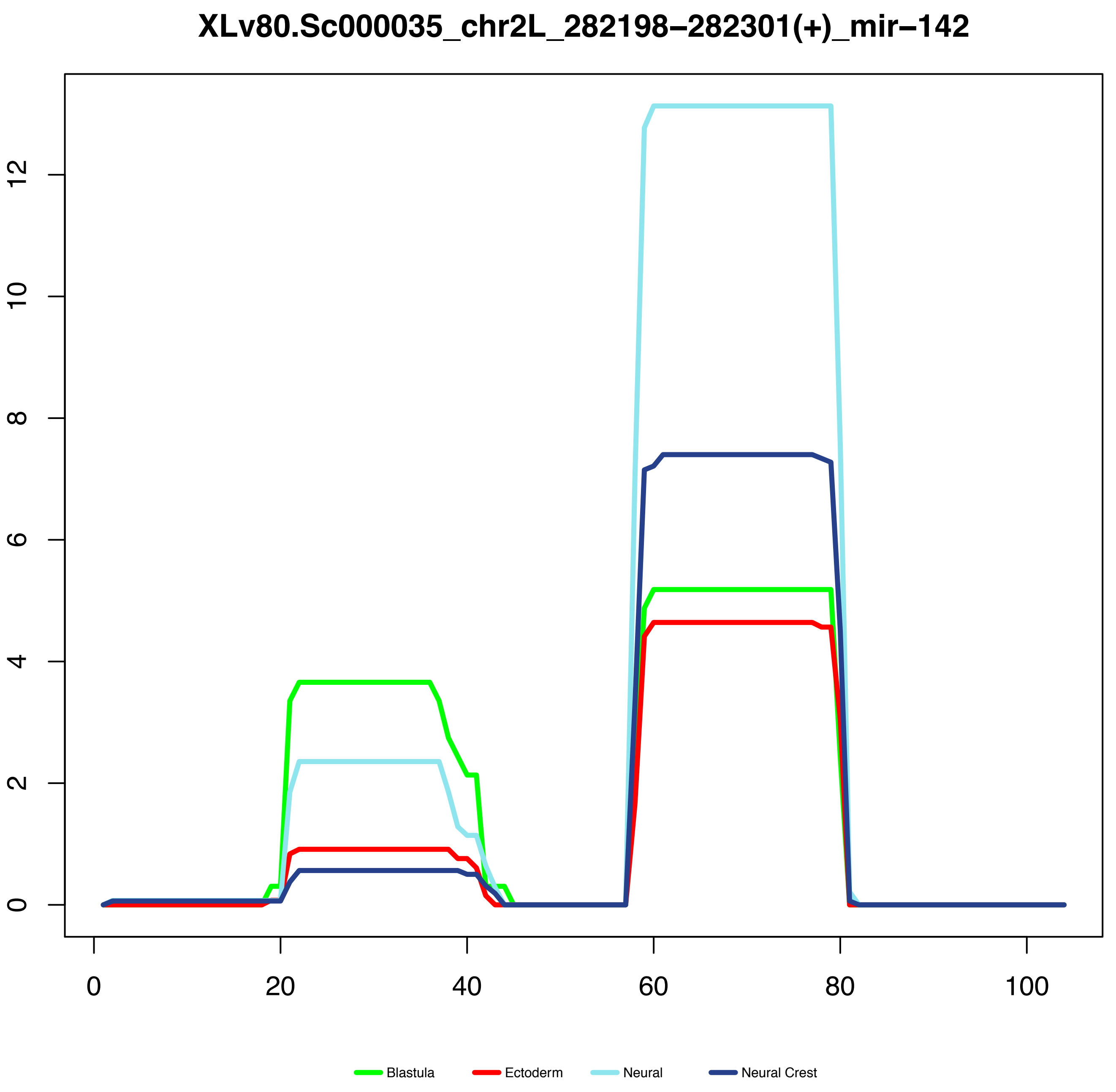
XLv80.Sc000035_chr2L_282198−282301(+)_mir−142
0
2
4
6
8
10
12
0
20
40
60
80
100
Blastula
Ectoderm
Neural
Neural Crest

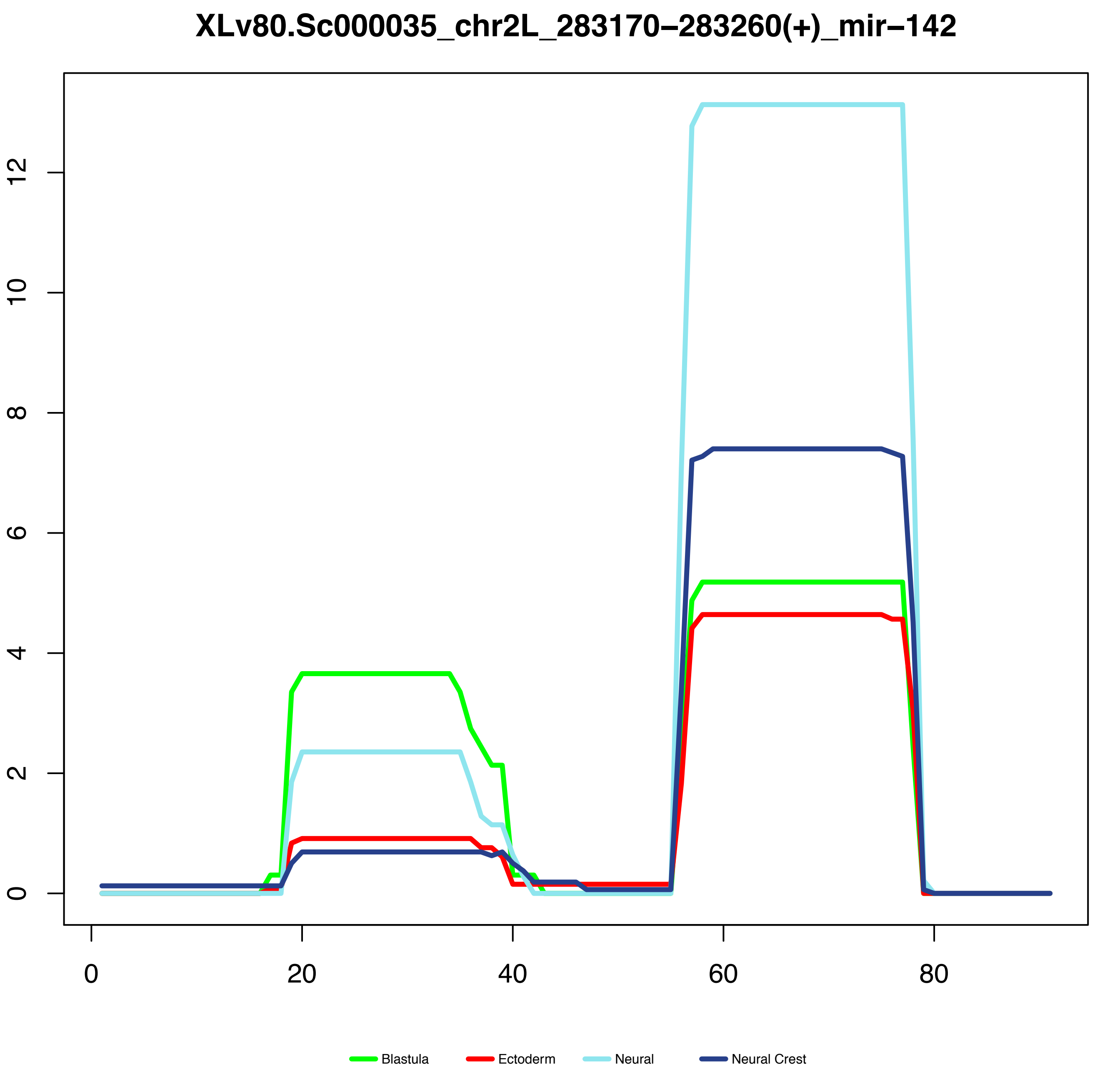

XLv80.Sc000035_chr2L_283170–283260(+)_mir–142
0
2
4
6
8
10
12
0
20
40
60
80
Blastula
Ectoderm
Neural
Neural Crest

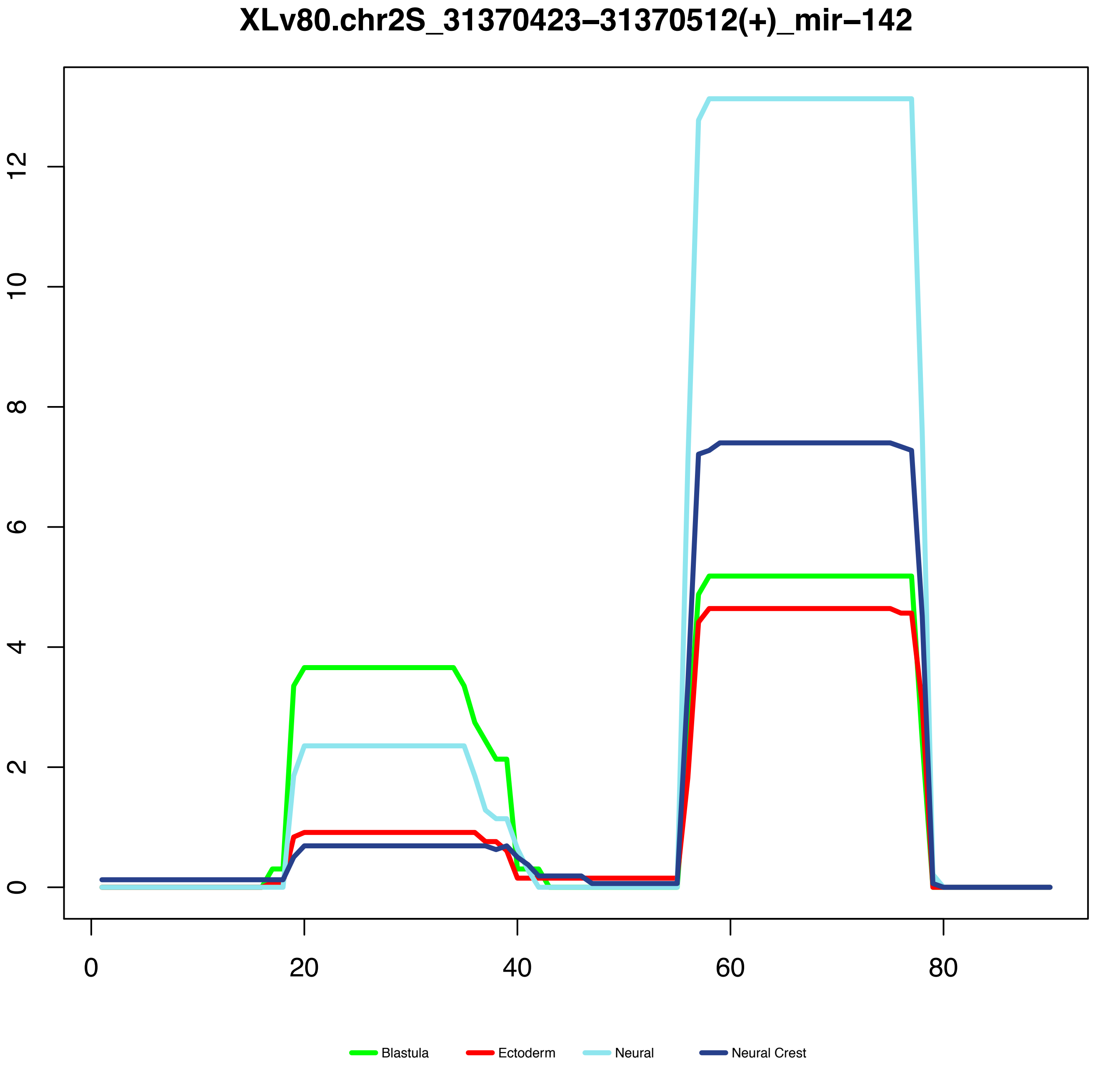

XLv80.chr2S_31370423−31370512(+)_mir−142
0
2
4
6
8
10
12
0
20
40
60
80
Blastula
Ectoderm
Neural
Neural Crest

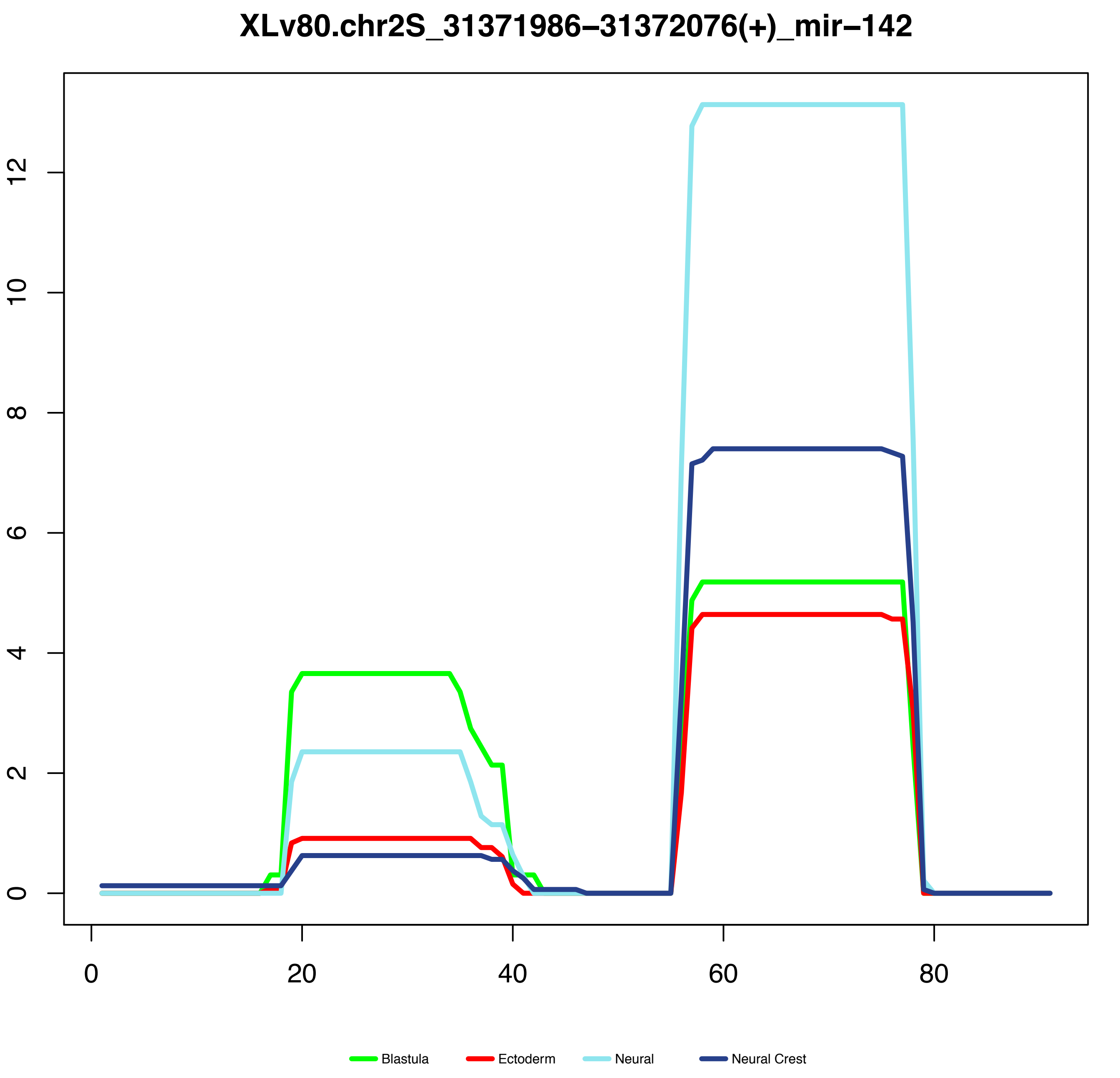

XLv80.chr2S_31371986−31372076(+)_mir−142
0
2
4
6
8
10
12
0
20
40
60
80
Blastula
Ectoderm
Neural
Neural Crest

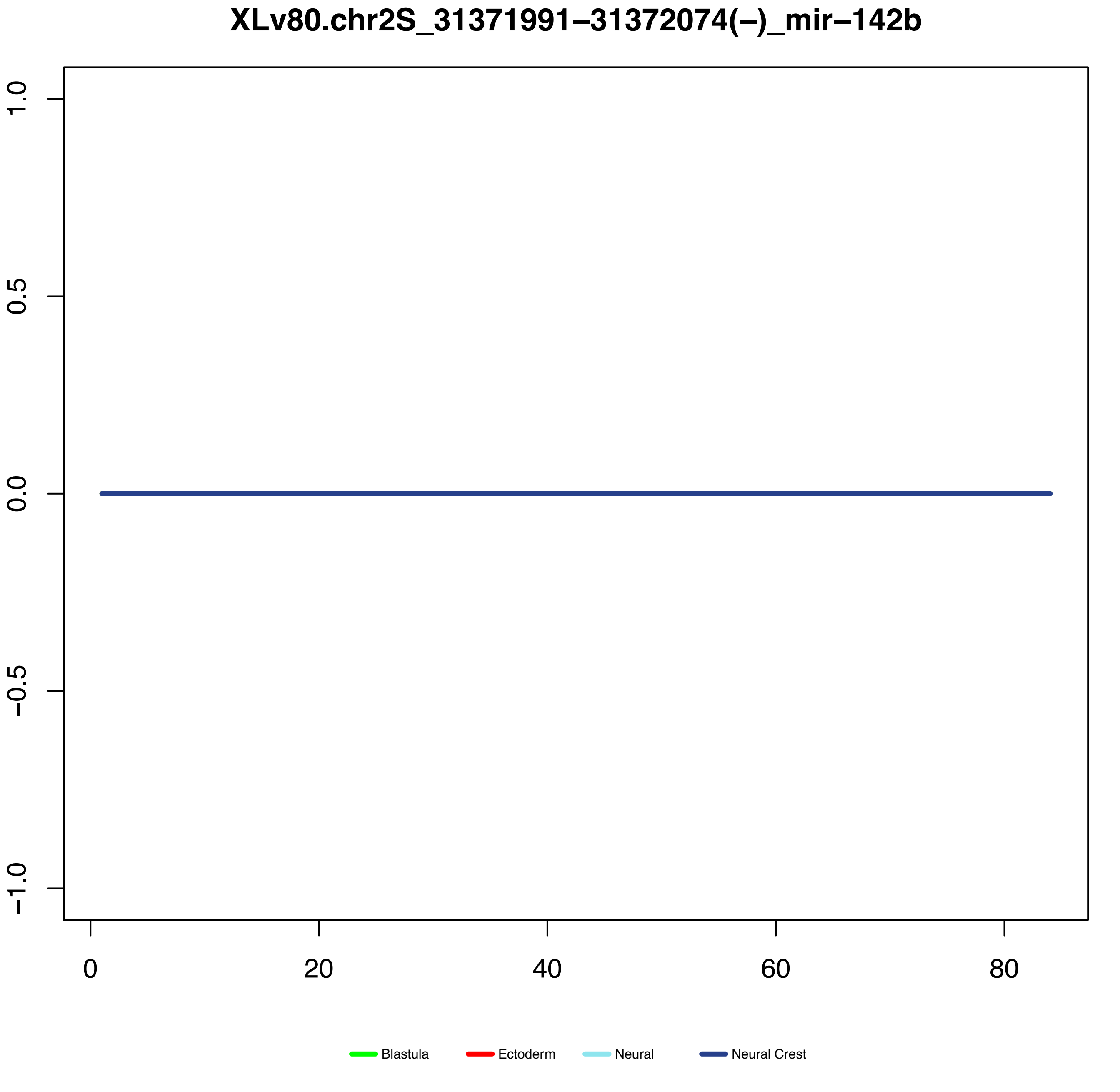
XLv80.chr2S_31371991−31372074(−)_mir−142b
1.0
0.5
0.0
−0.5
−1.0
0
20
40
60
80
Blastula
Ectoderm
Neural
Neural Crest

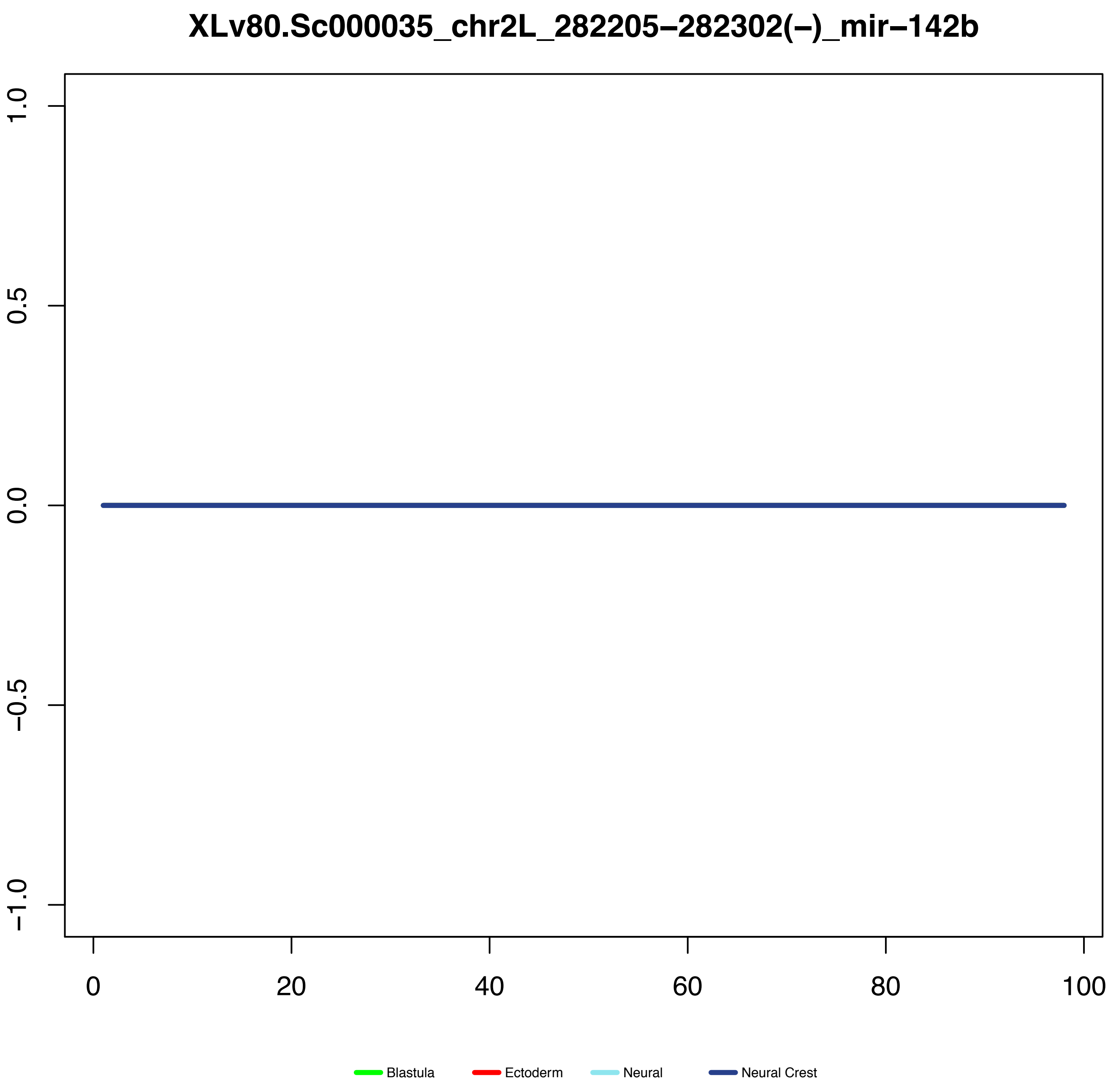
XLv80.Sc000035_chr2L_282205−282302(−)_mir−142b
1.0
0.5
0.0
−0.5
−1.0
0
20
40
60
80
100
Blastula
Ectoderm
Neural
Neural Crest

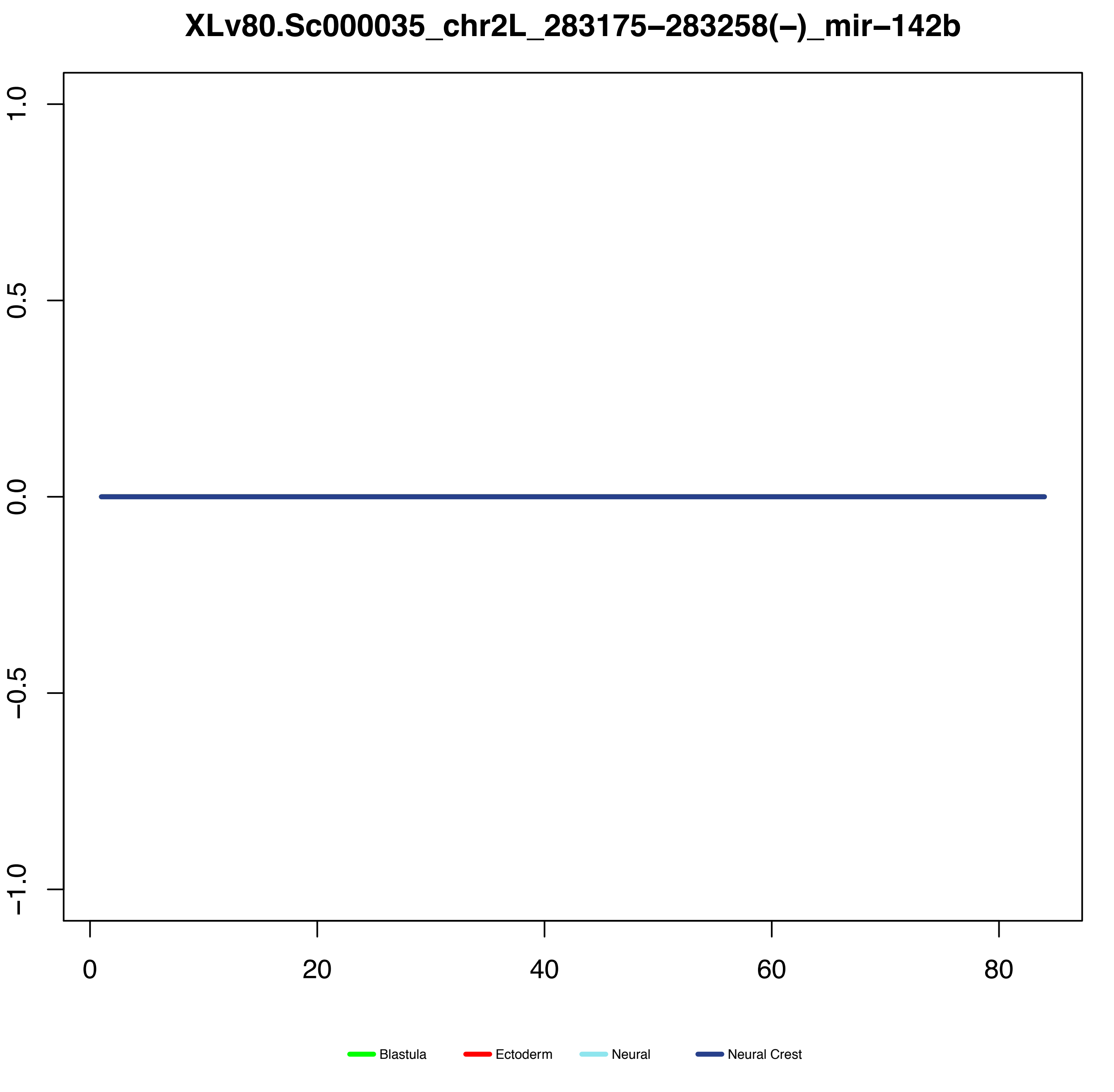
XLv80.Sc000035_chr2L_283175–283258(–)_mir–142b
1.0
0.5
0.0
–0.5
–1.0
0
20
40
60
80
Blastula
Ectoderm
Neural
Neural Crest

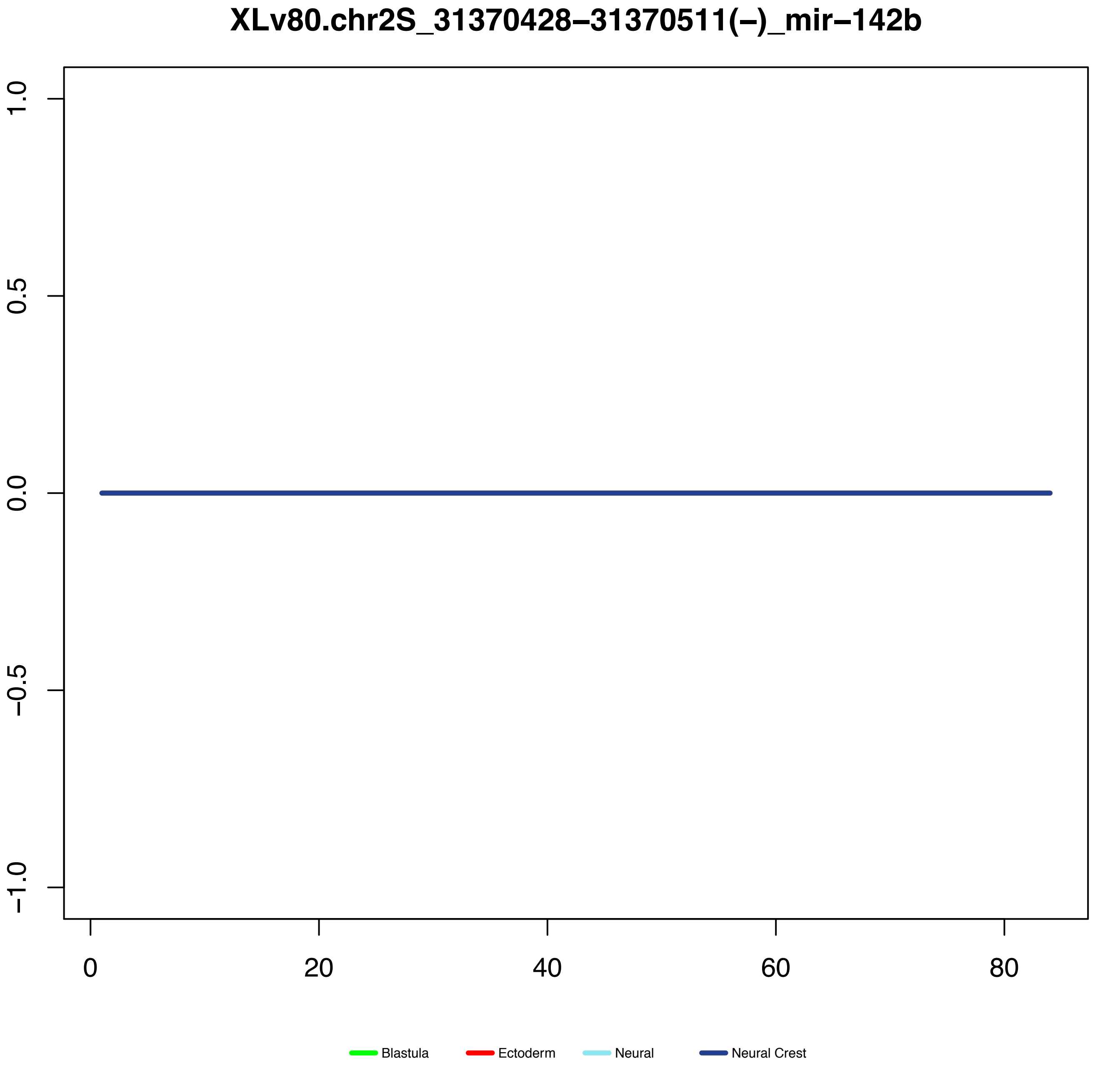

XLv80.chr2S_31370428−31370511(−)_mir−142b
1.0
0.5
0.0
−0.5
−1.0
0
20
40
60
80
Blastula
Ectoderm
Neural
Neural Crest

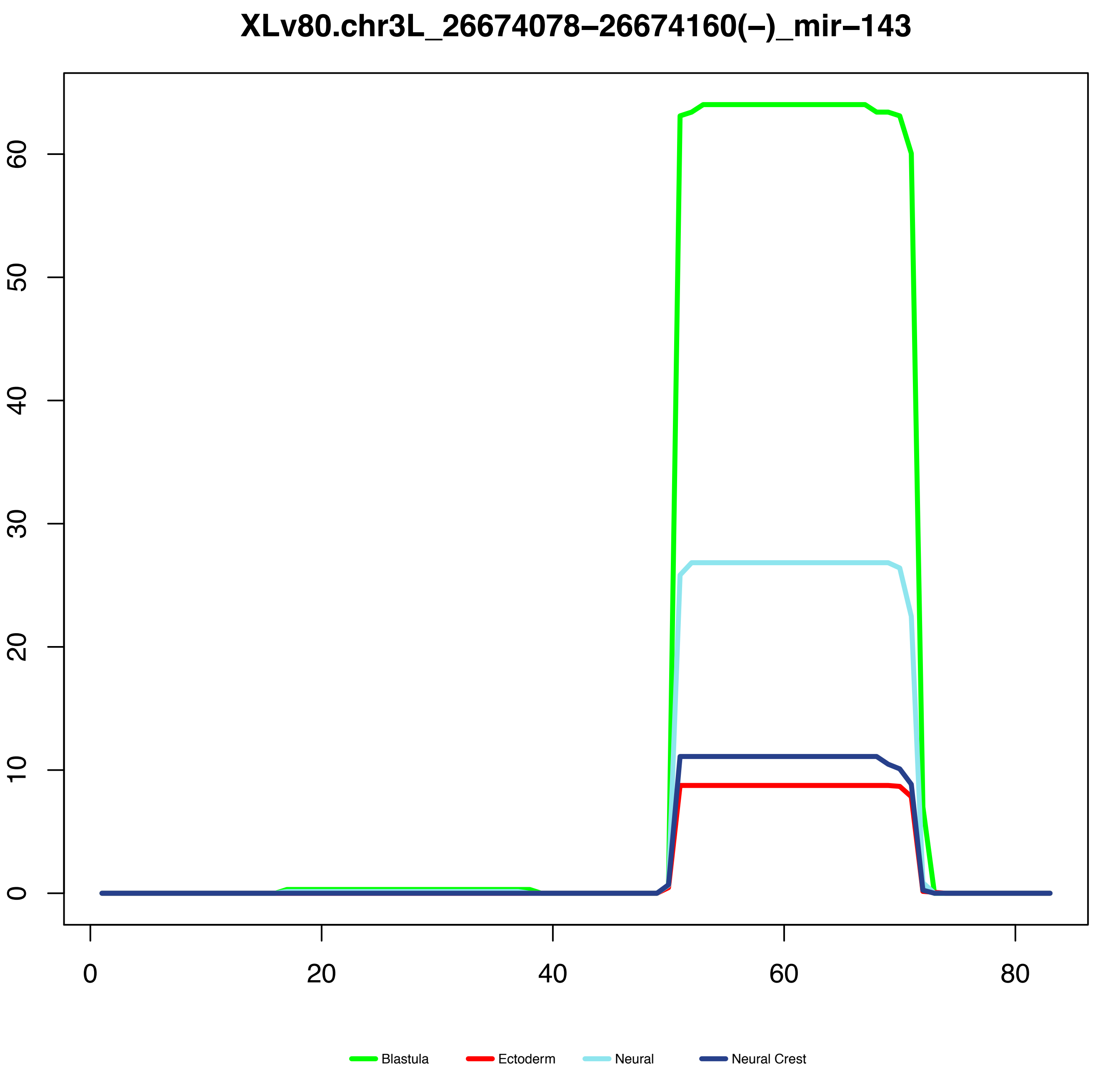
XLv80.chr3L_26674078−26674160(−)_mir−143
0
10
20
30
40
50
60
0
20
40
60
80
Blastula
Ectoderm
Neural
Neural Crest

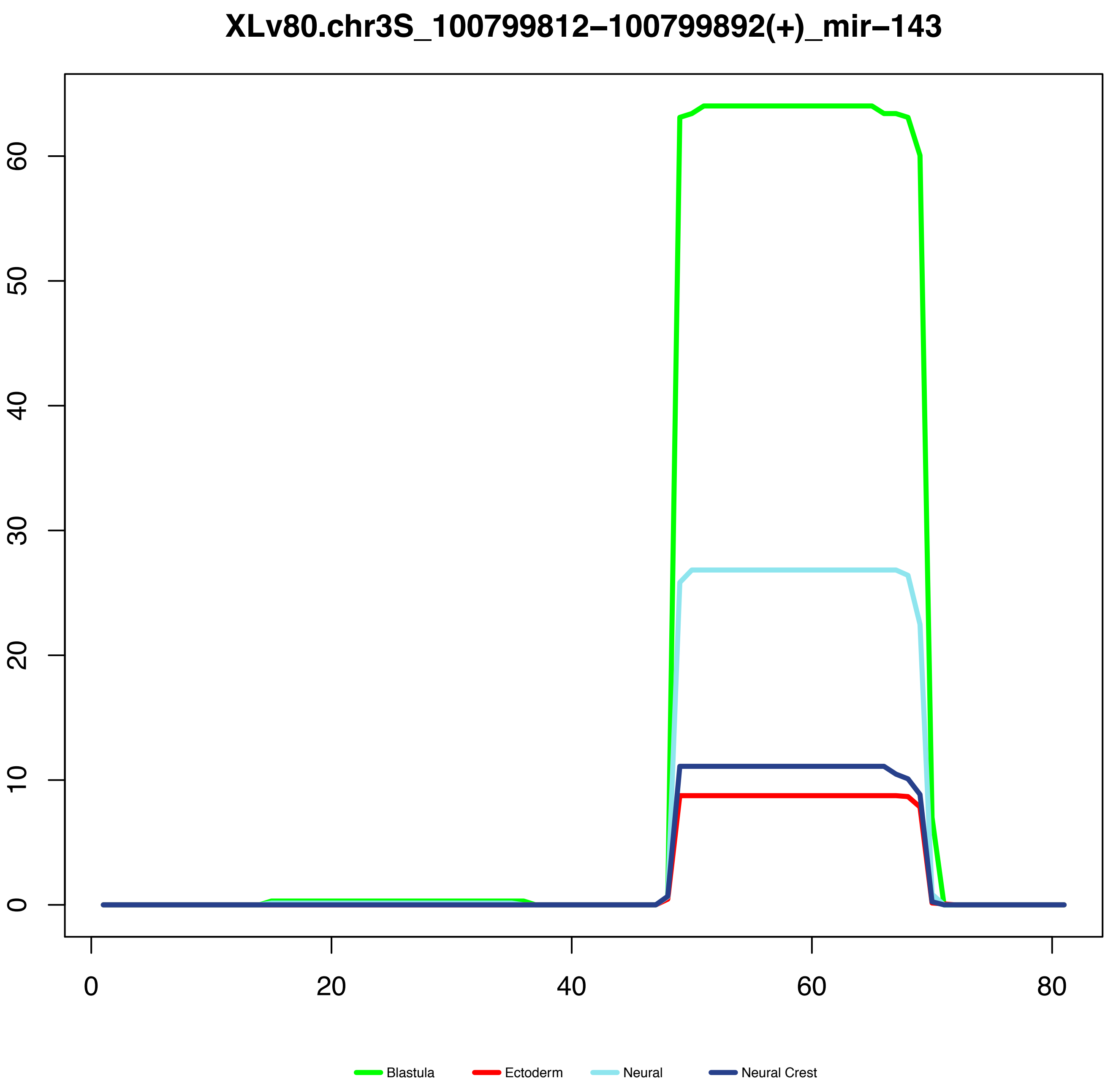

XLv80.chr3S_100799812−100799892(+)_mir−143
0
10
20
30
40
50
60
0
20
40
60
80
Blastula
Ectoderm
Neural
Neural Crest

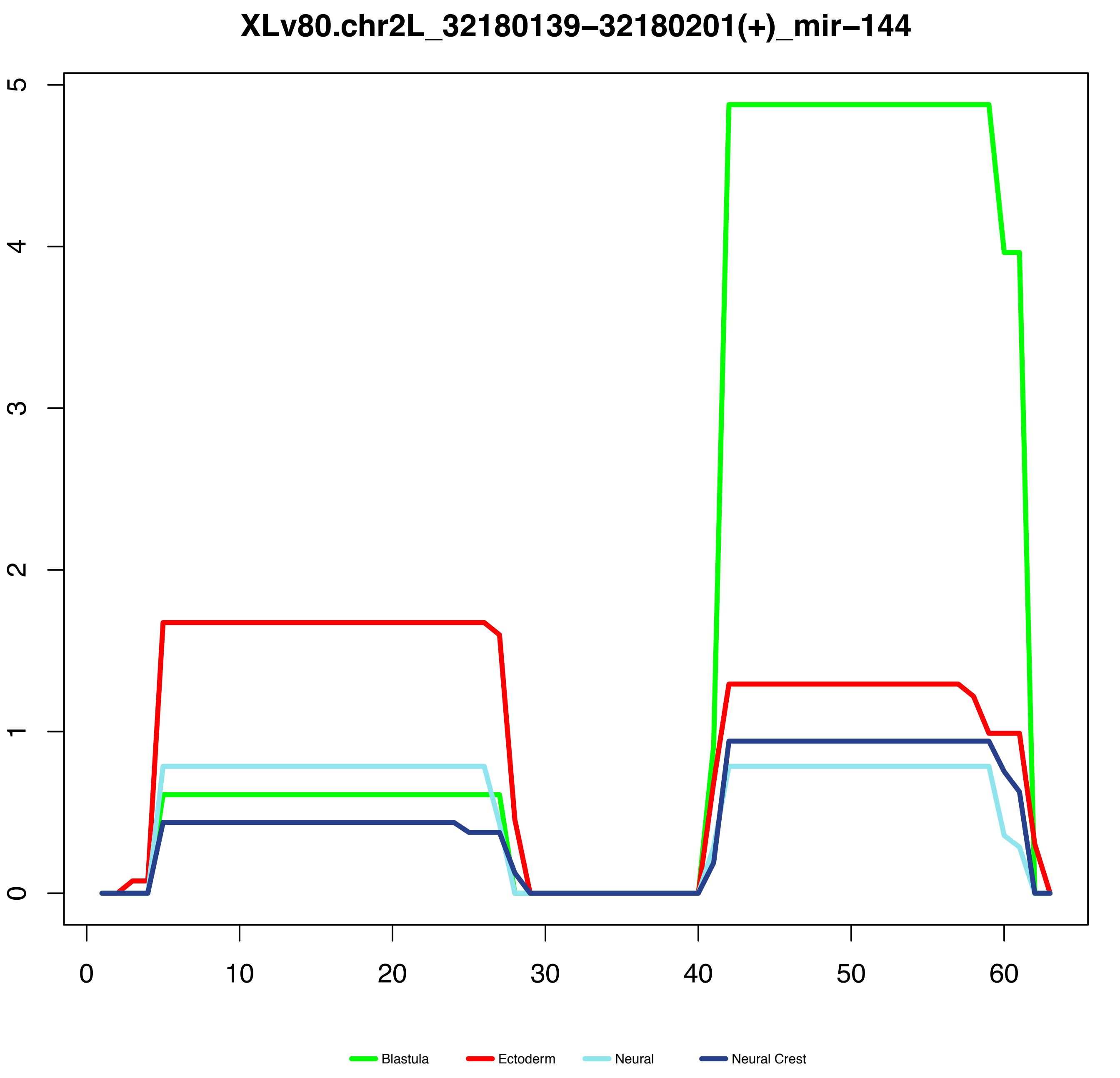

XLv80.chr2L_32180139−32180201(+)_mir−144
0
1
2
3
4
5
0
10
20
30
40
50
60
Blastula
Ectoderm
Neural
Neural Crest

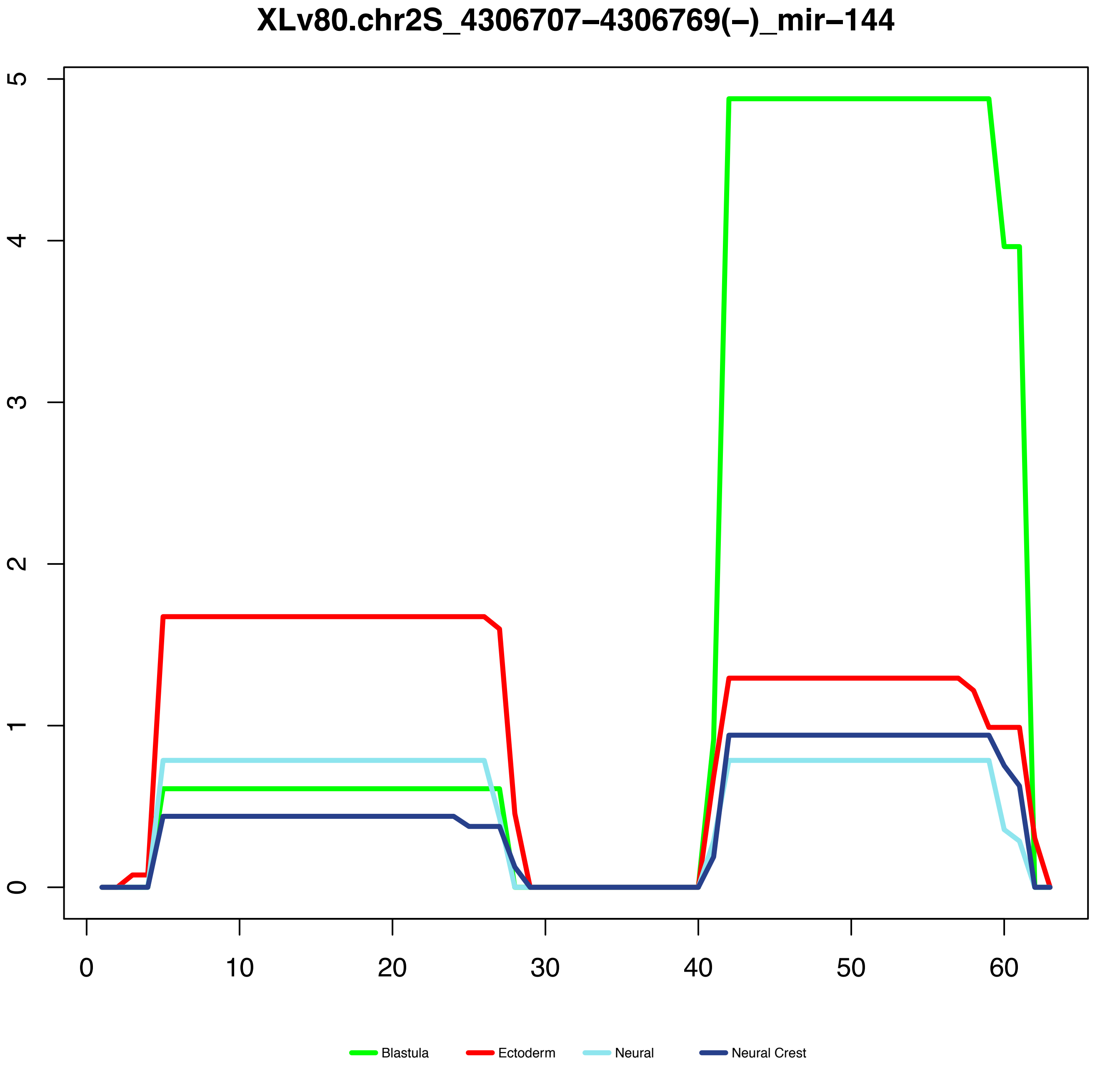
XLv80.chr2S_4306707−4306769(−)_mir−144
0
1
2
3
4
5
0
10
20
30
40
50
60
Blastula
Ectoderm
Neural
Neural Crest

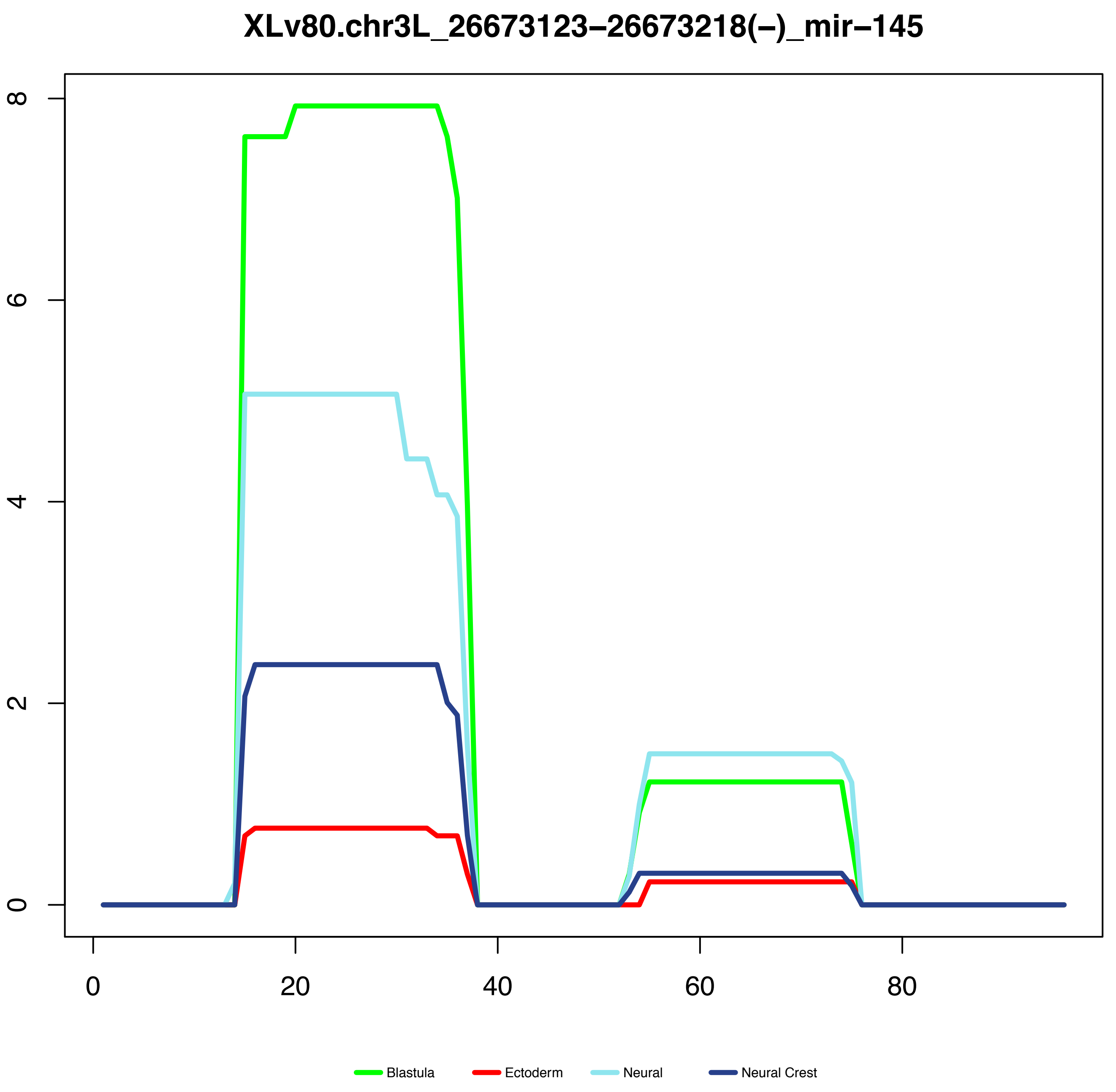

XLv80.chr3L_26673123−26673218(−)_mir−145
0
2
4
6
8
0
20
40
60
80
Blastula
Ectoderm
Neural
Neural Crest

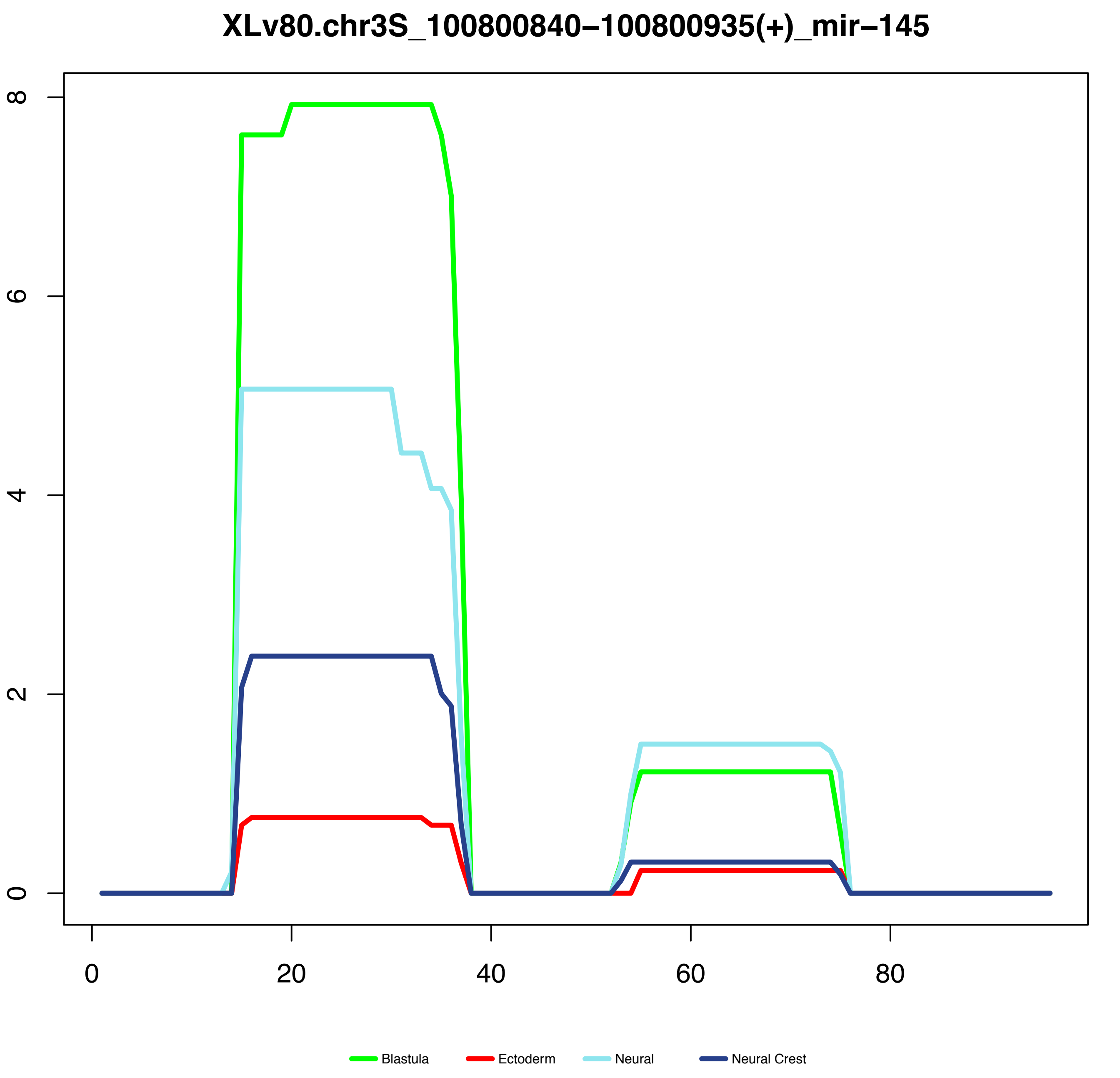
XLv80.chr3S_100800840−100800935(+)_mir−145
0
2
4
6
8
0
20
40
60
80
Blastula
Ectoderm
Neural
Neural Crest

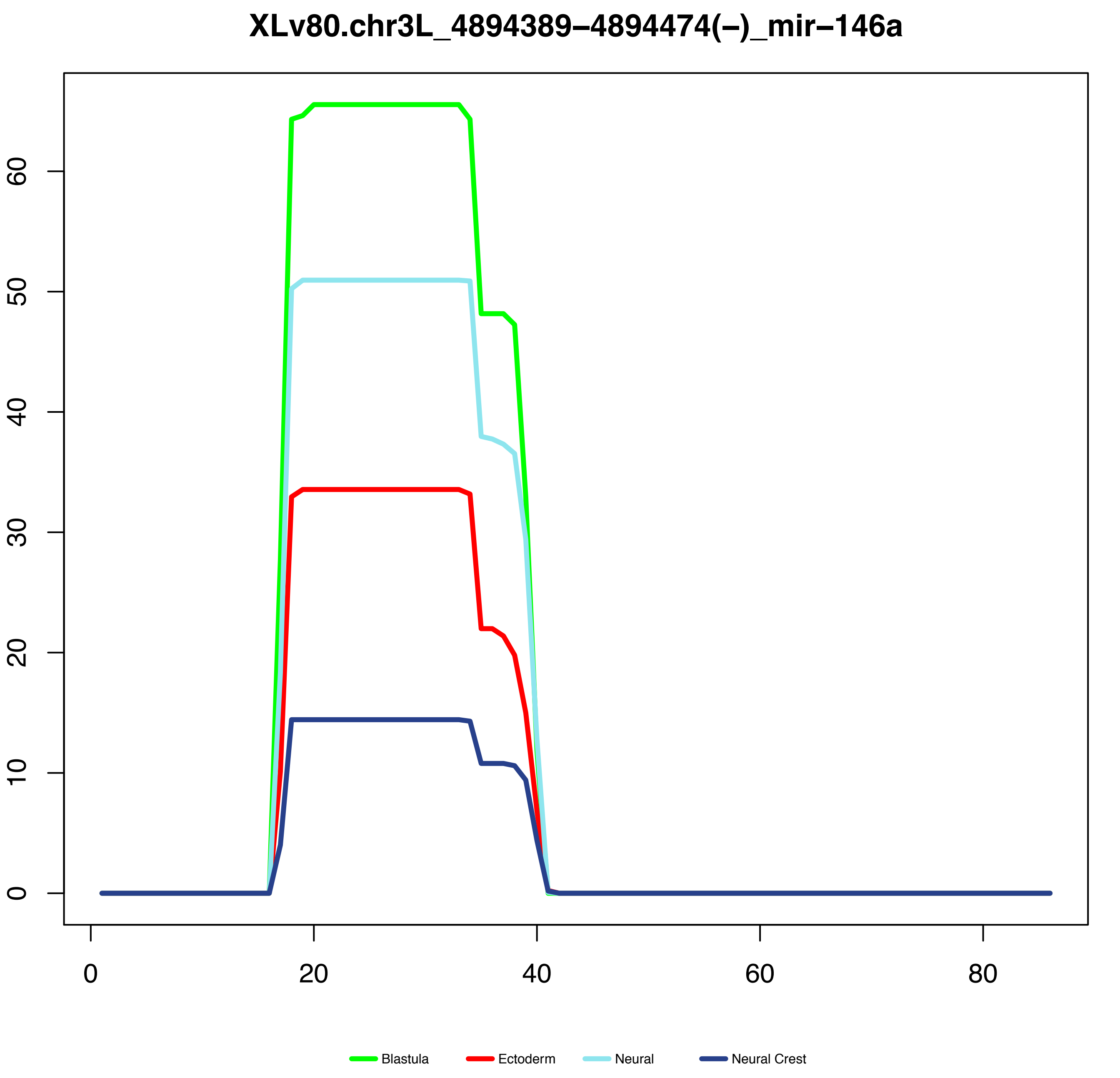

XLv80.chr3L_4894389−4894474(−)_mir−146a
0
10
20
30
40
50
60
0
20
40
60
80
Blastula
Ectoderm
Neural
Neural Crest

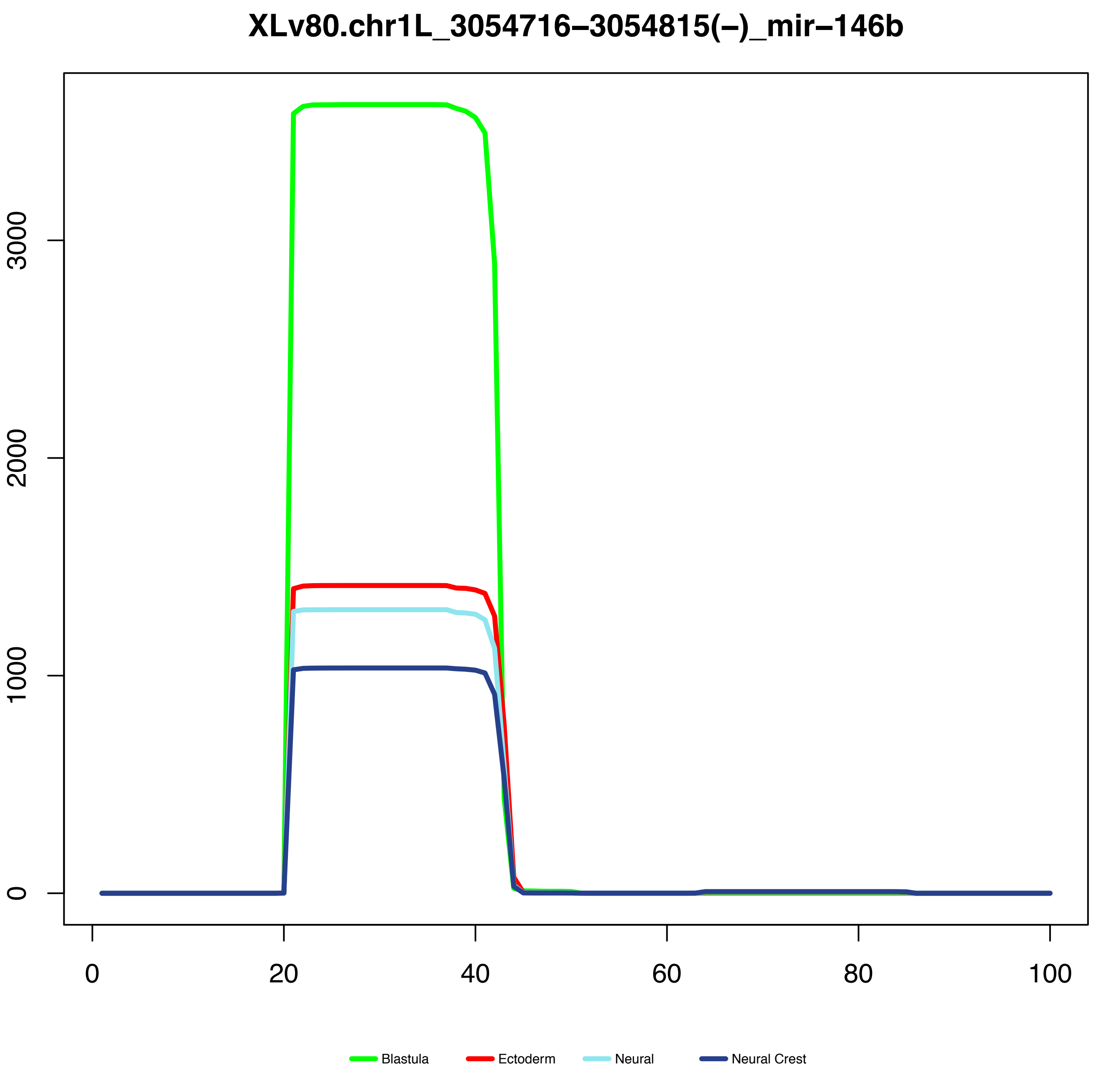
XLv80.chr1L_3054716–3054815(–)_mir–146b
0
1000
2000
3000
0
20
40
60
80
100
Blastula
Ectoderm
Neural
Neural Crest

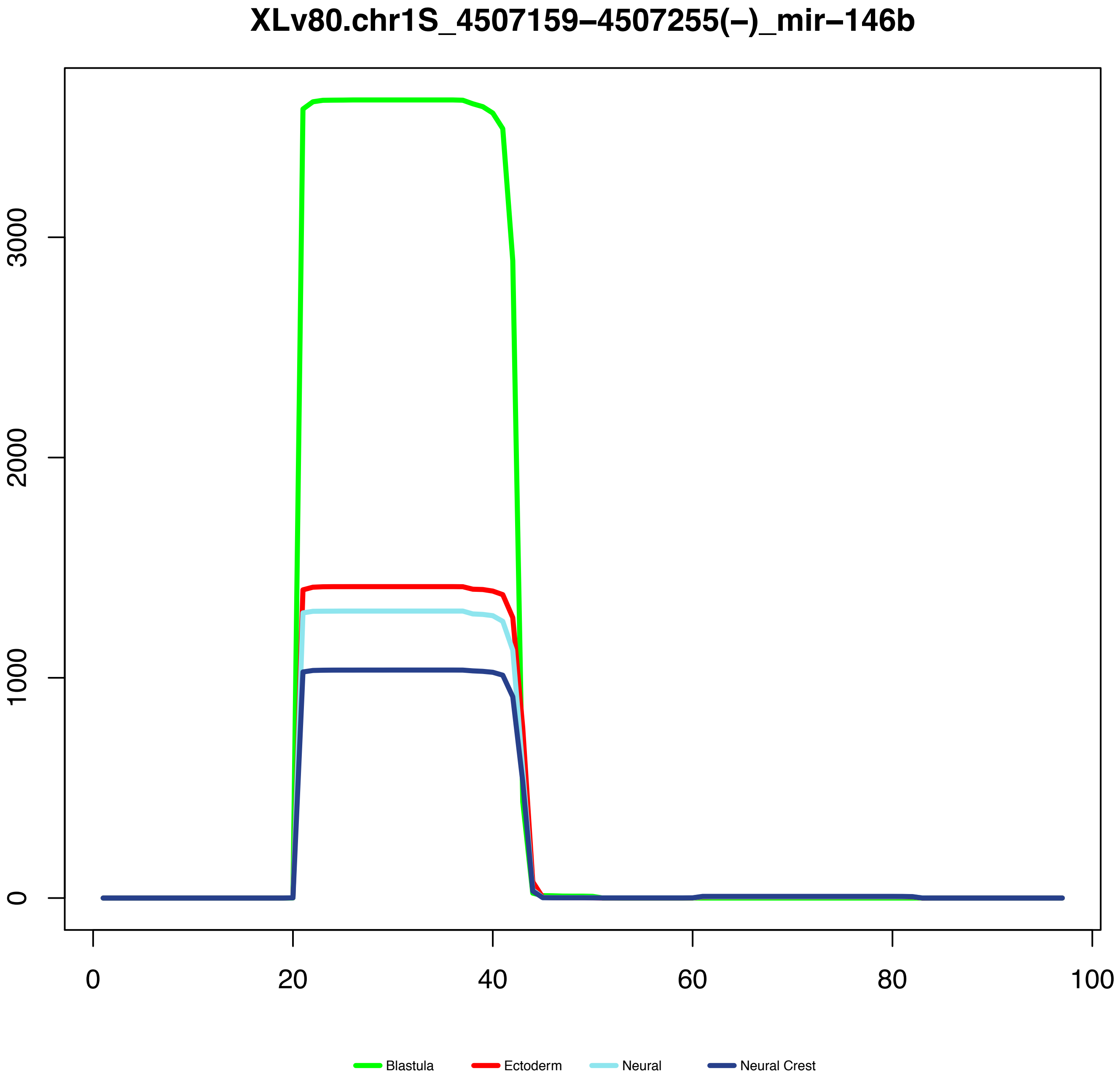
XLv80.chr1S_4507159−4507255(−)_mir−146b
0
1000
2000
3000
0
20
40
60
80
100
Blastula
Ectoderm
Neural
Neural Crest

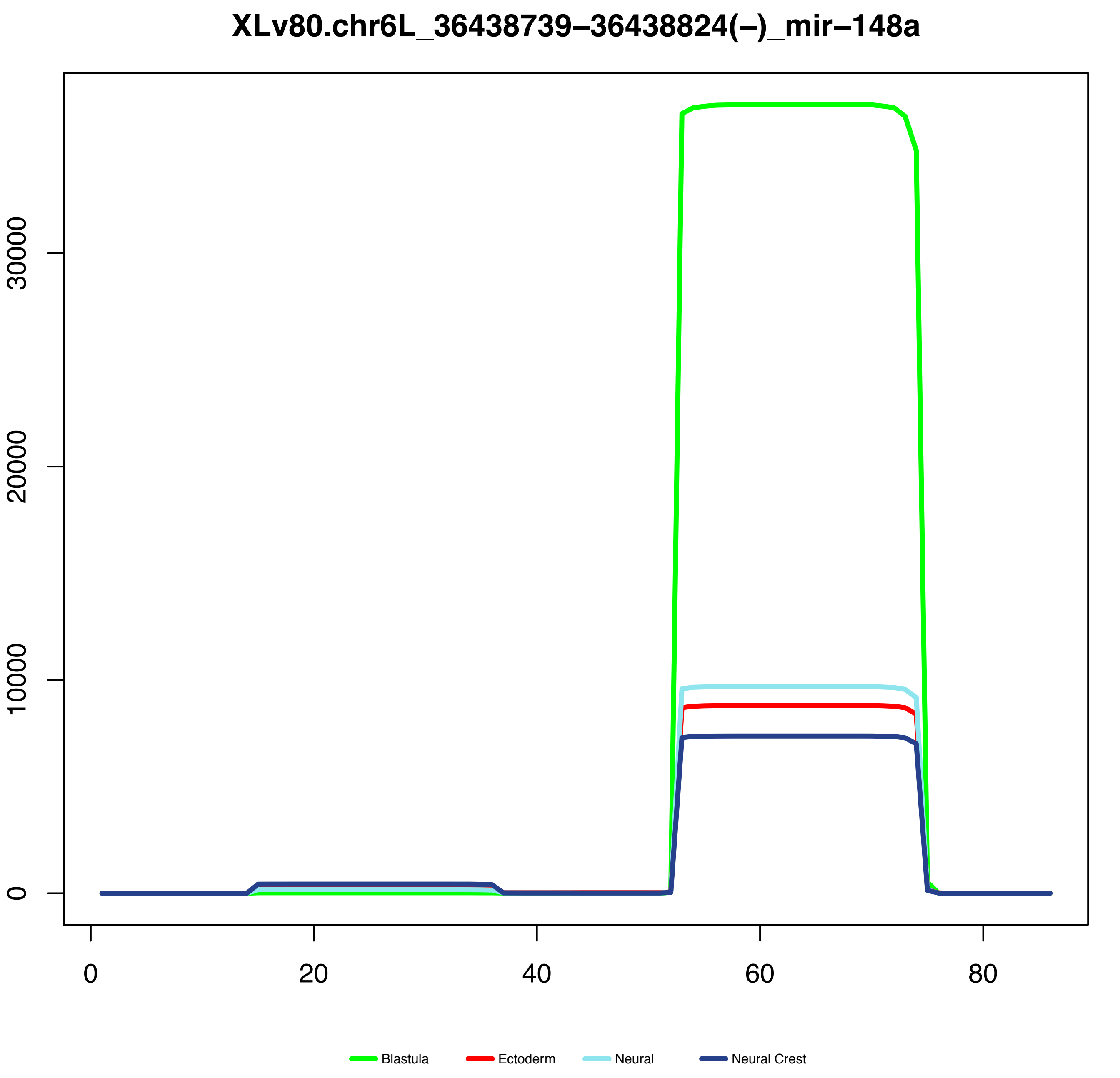
XLv80.chr6L_36438739–36438824(–)_mir–148a
0
10000
20000
30000
0
20
40
60
80
Blastula
Ectoderm
Neural
Neural Crest

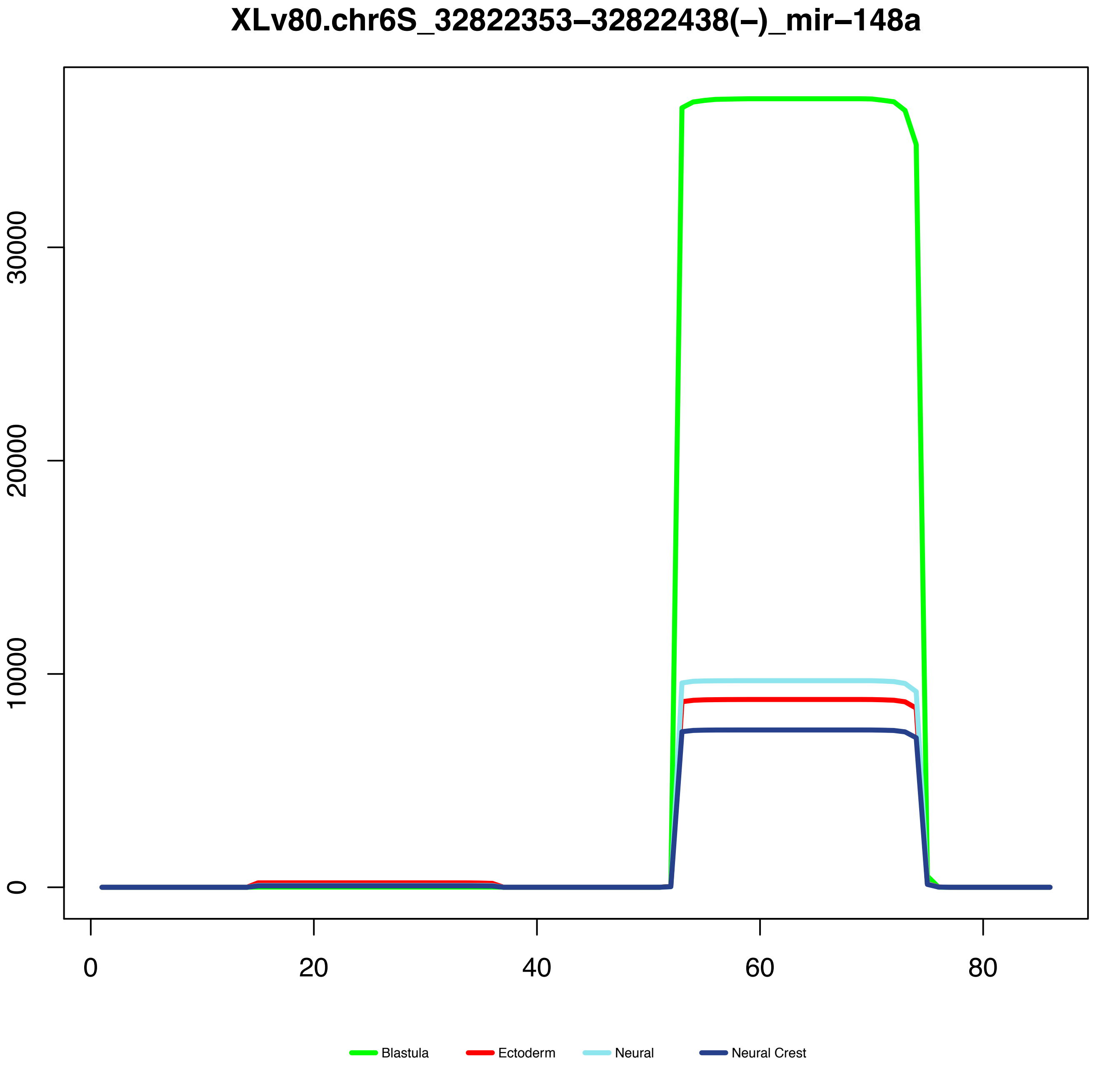
XLv80.chr6S_32822353−32822438(−)_mir−148a
0
10000
20000
30000
0
20
40
60
80
Blastula
Ectoderm
Neural
Neural Crest

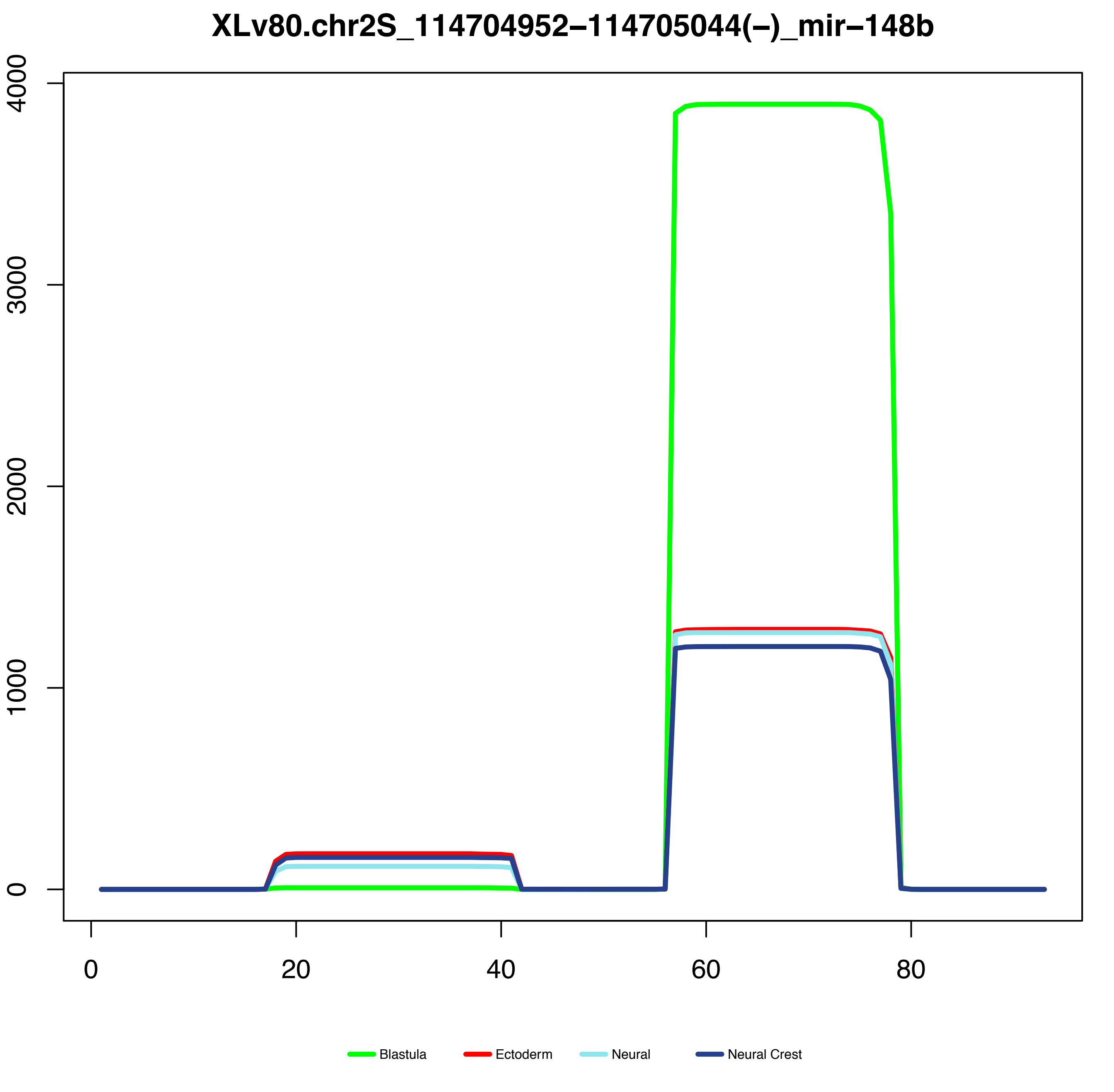
XLv80.chr2S_114704952−114705044(−)_mir−148b
0
1000
2000
3000
4000
0
20
40
60
80
Blastula
Ectoderm
Neural
Neural Crest

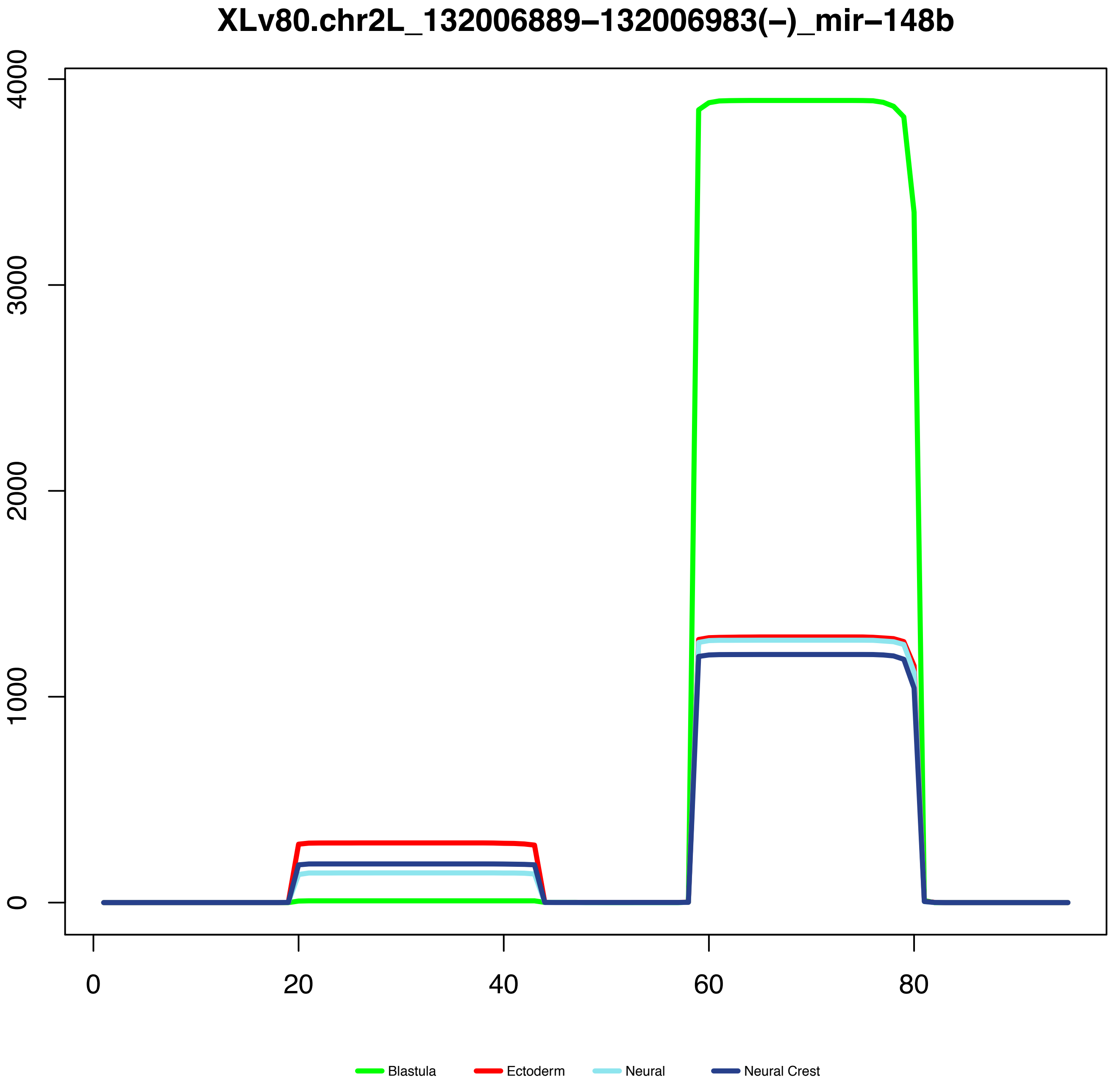

XLv80.chr2L_132006889−132006983(−)_mir−148b
0
1000
2000
3000
4000
0
20
40
60
80
Blastula
Ectoderm
Neural
Neural Crest

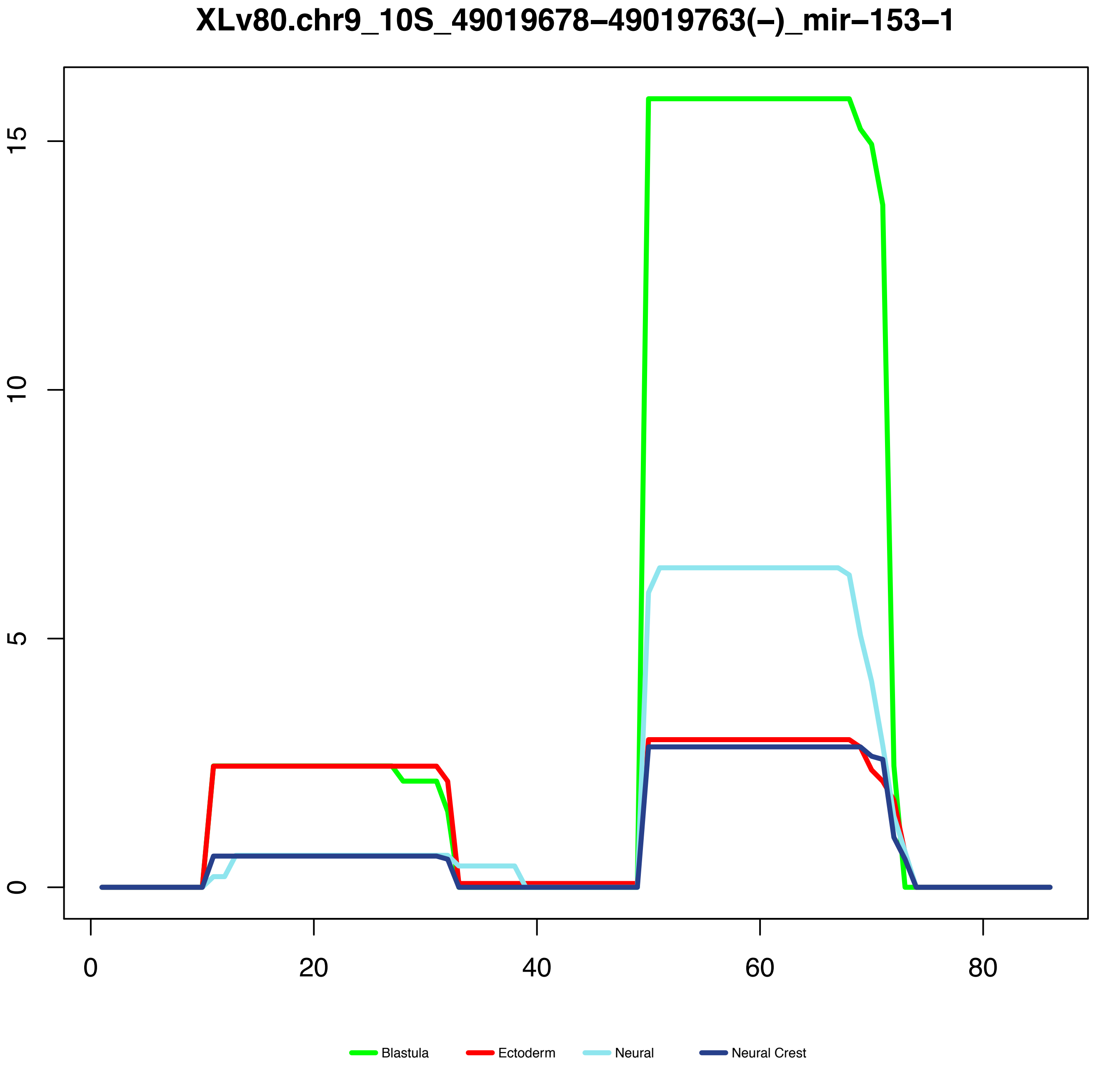
XLv80.chr9_10S_49019678−49019763(−)_mir−153−1
0
5
10
15
0
20
40
60
80
Blastula
Ectoderm
Neural
Neural Crest

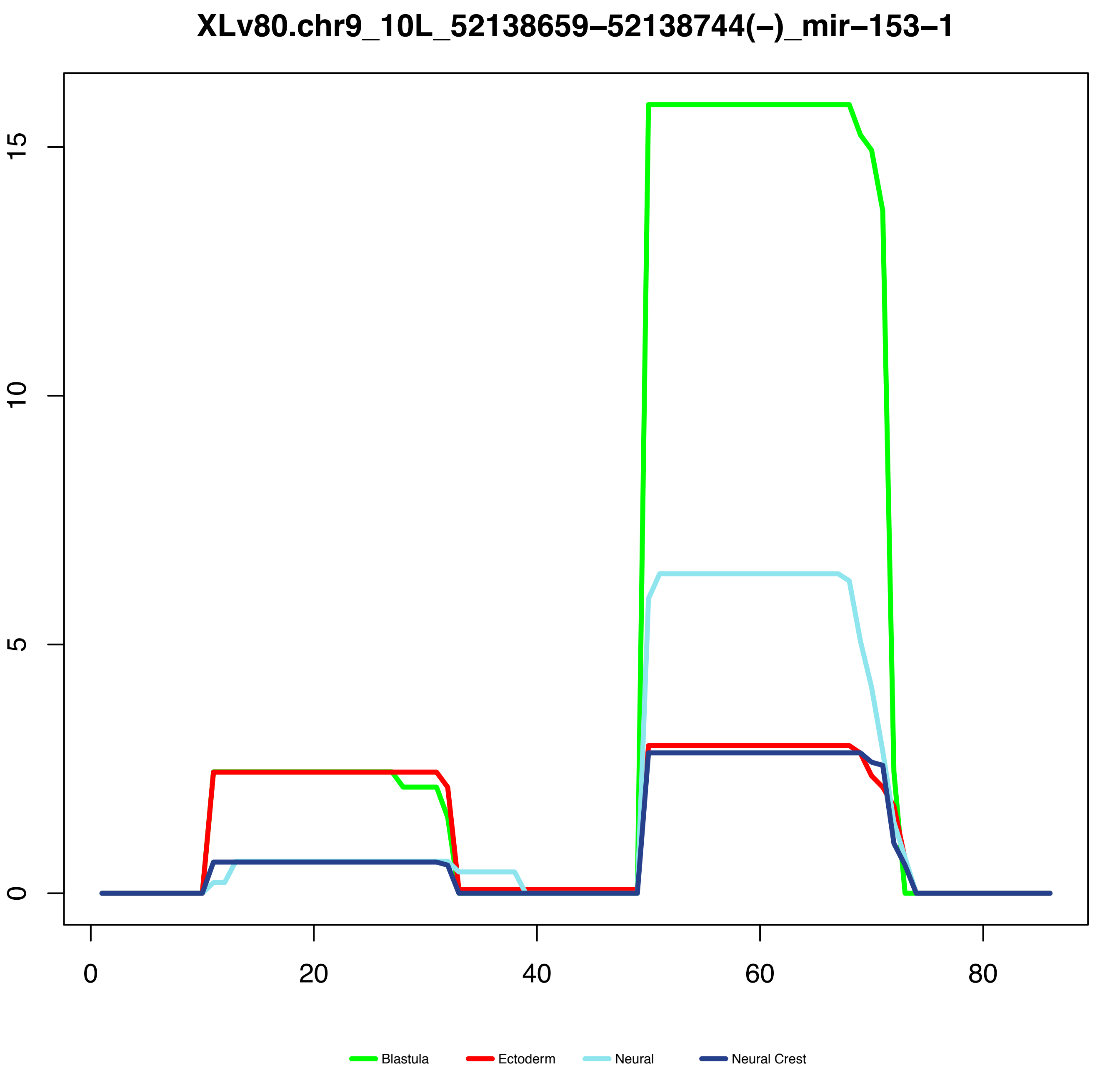
XLv80.chr9_10L_52138659−52138744(−)_mir−153−1
0
5
10
15
0
20
40
60
80
Blastula
Ectoderm
Neural
Neural Crest

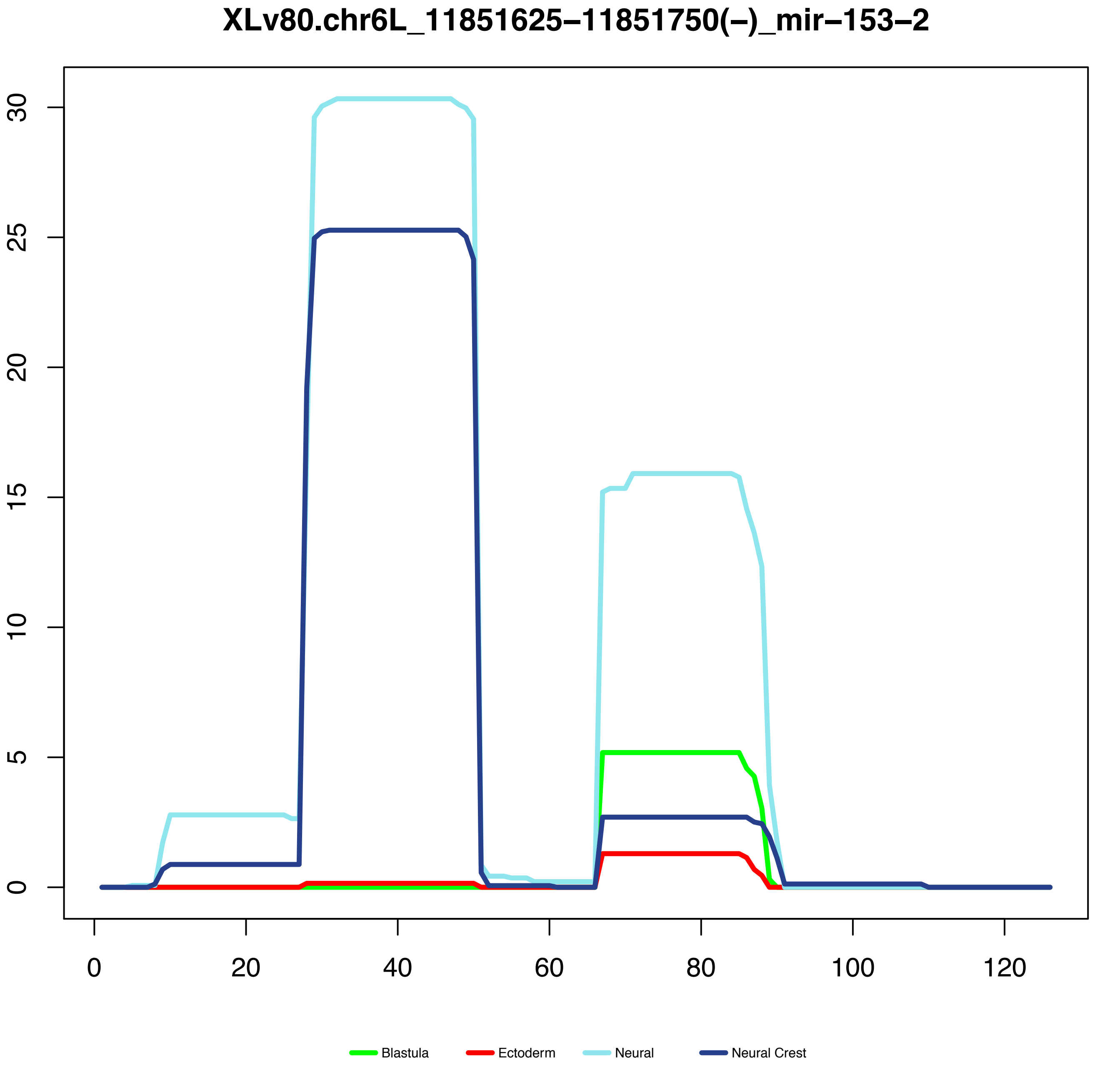
XLv80.chr6L_11851625−11851750(−)_mir−153−2
0
5
10
15
20
25
30
0
20
40
60
80
100
120
Blastula
Ectoderm
Neural
Neural Crest

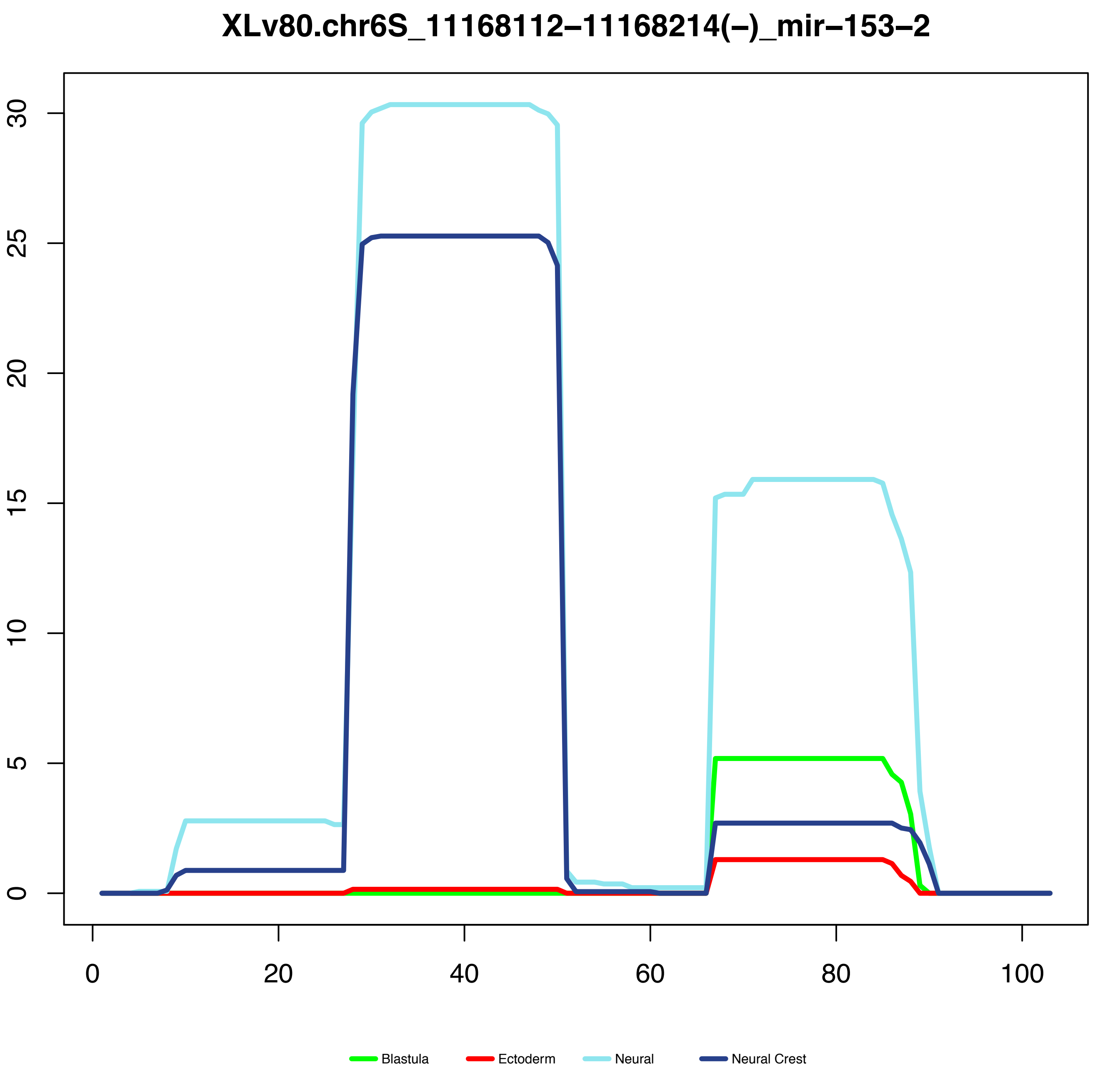
XLv80.chr6S_11168112−11168214(−)_mir−153−2
0
5
10
15
20
25
30
0
20
40
60
80
100
Blastula
Ectoderm
Neural
Neural Crest

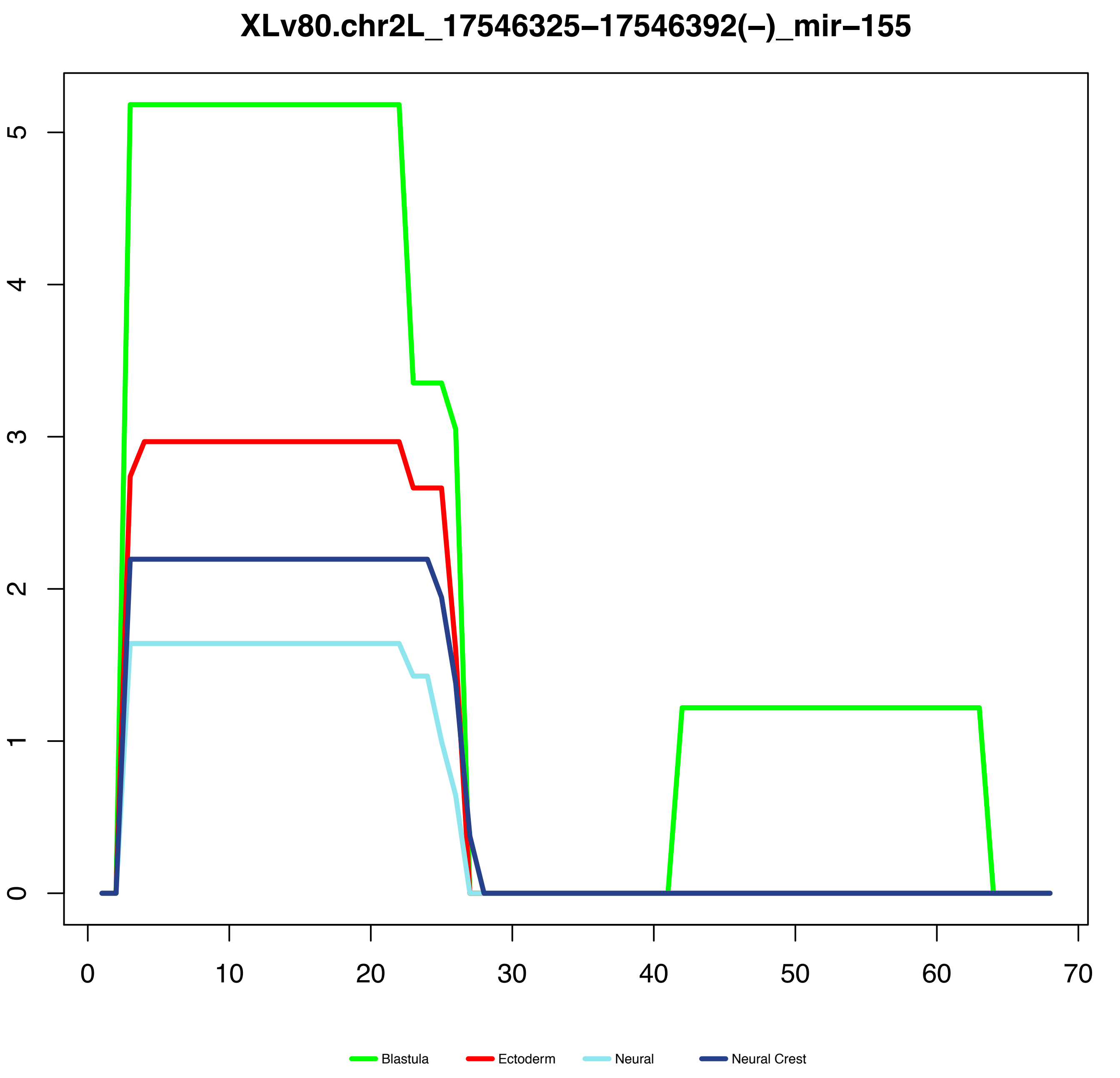
XLv80.chr2L_17546325−17546392(−)_mir−155
0
1
2
3
4
5
0
10
20
30
40
50
60
70
Blastula
Ectoderm
Neural
Neural Crest

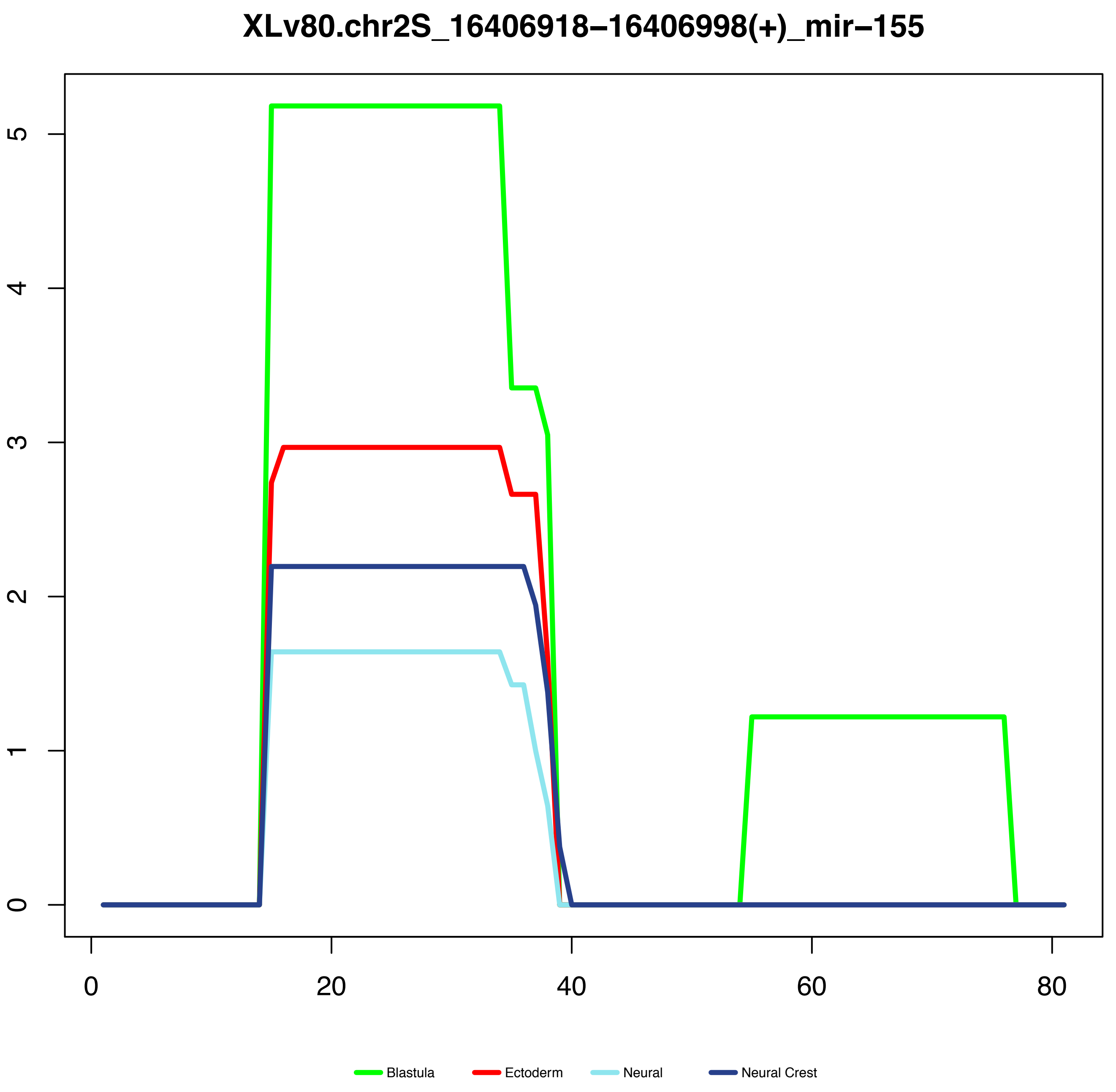
XLv80.chr2S_16406918−16406998(+)_mir−155
0
1
2
3
4
5
0
20
40
60
80
Blastula
Ectoderm
Neural
Neural Crest

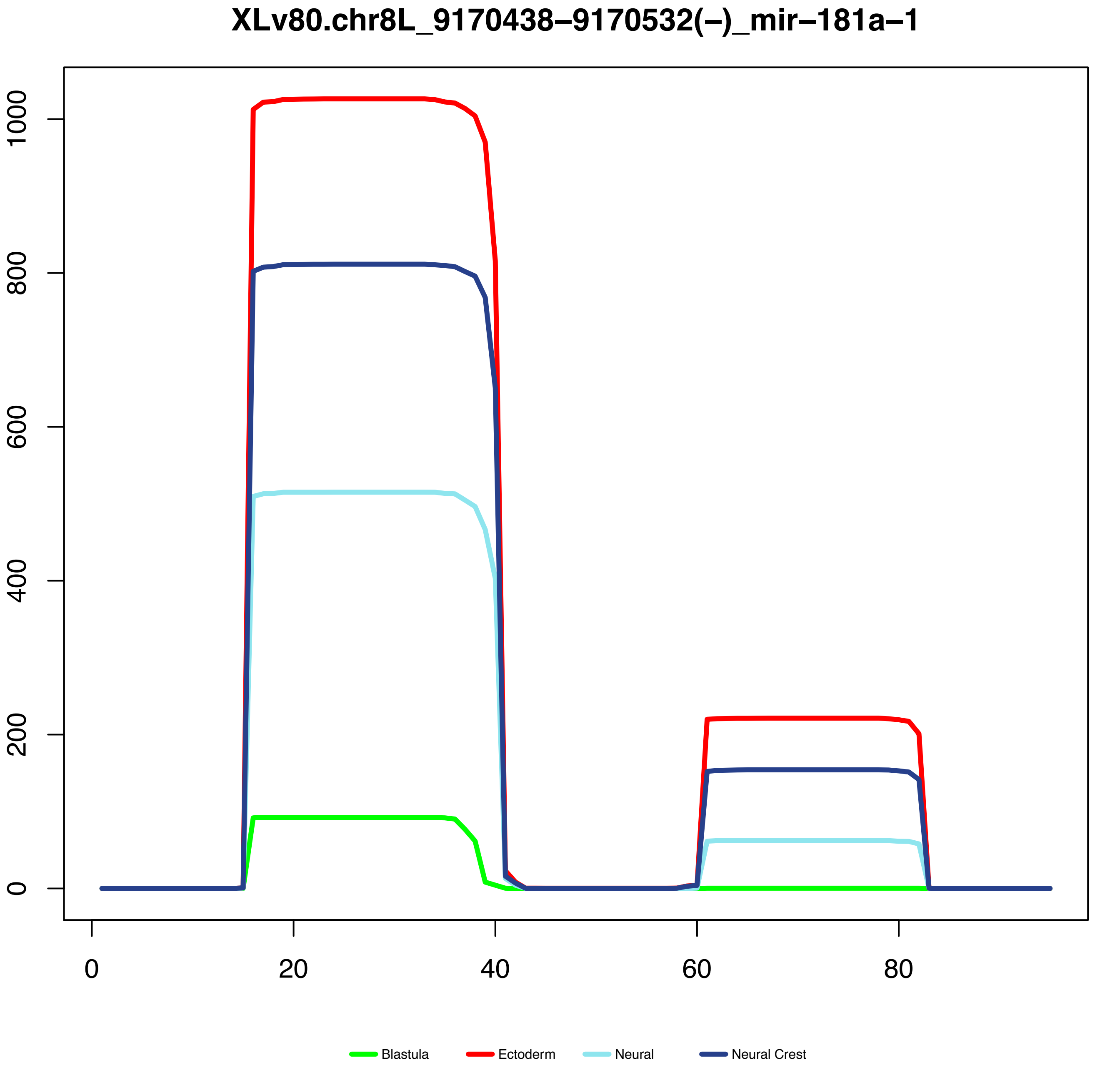

XLv80.chr8L_9170438–9170532(–)_mir–181a–1
0
200
400
600
800
1000
0
20
40
60
80
Blastula
Ectoderm
Neural
Neural Crest

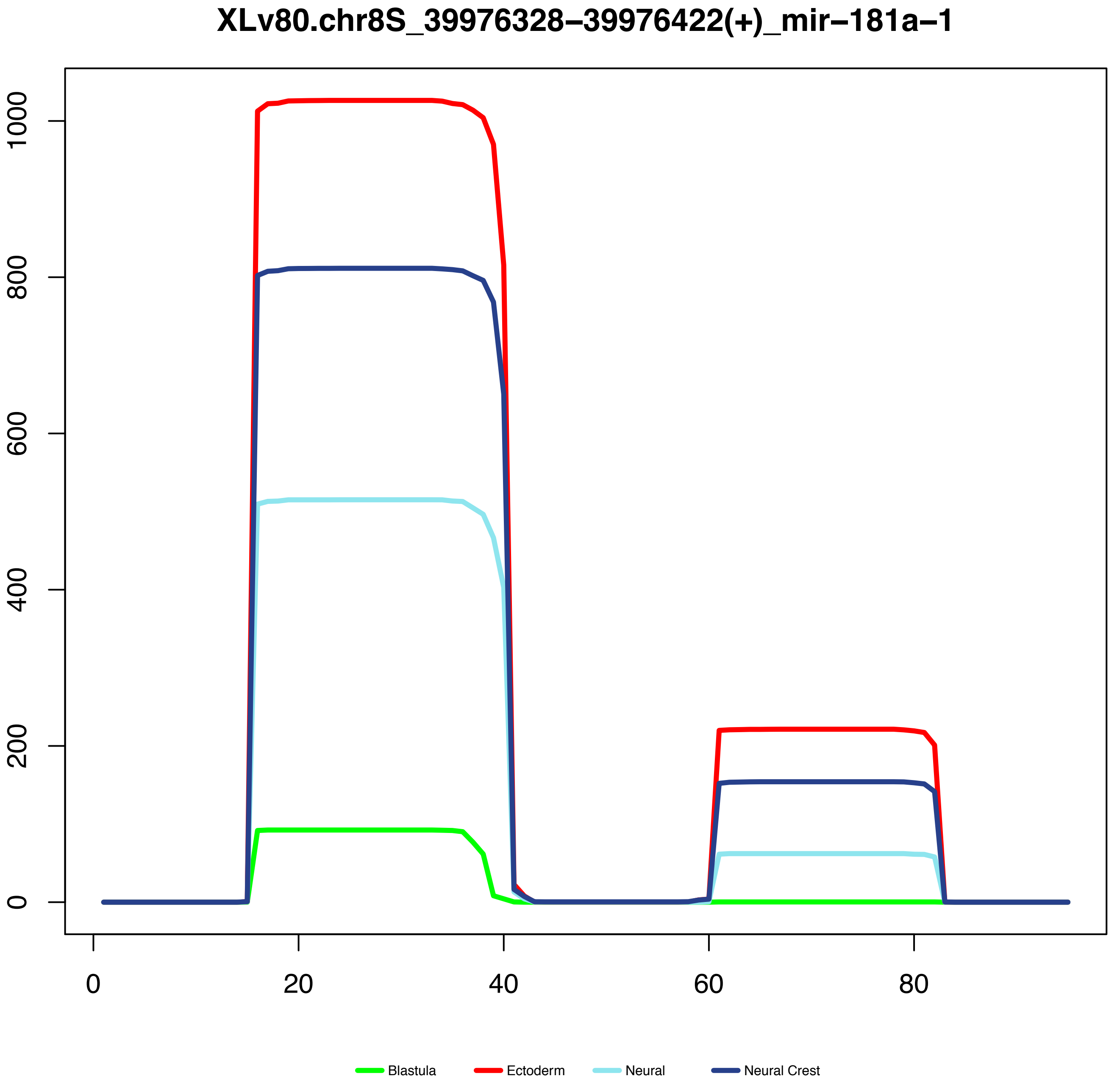

XLv80.chr8S_39976328−39976422(+)_mir−181a−1
0
200
400
600
800
1000
0
20
40
60
80
Blastula
Ectoderm
Neural
Neural Crest

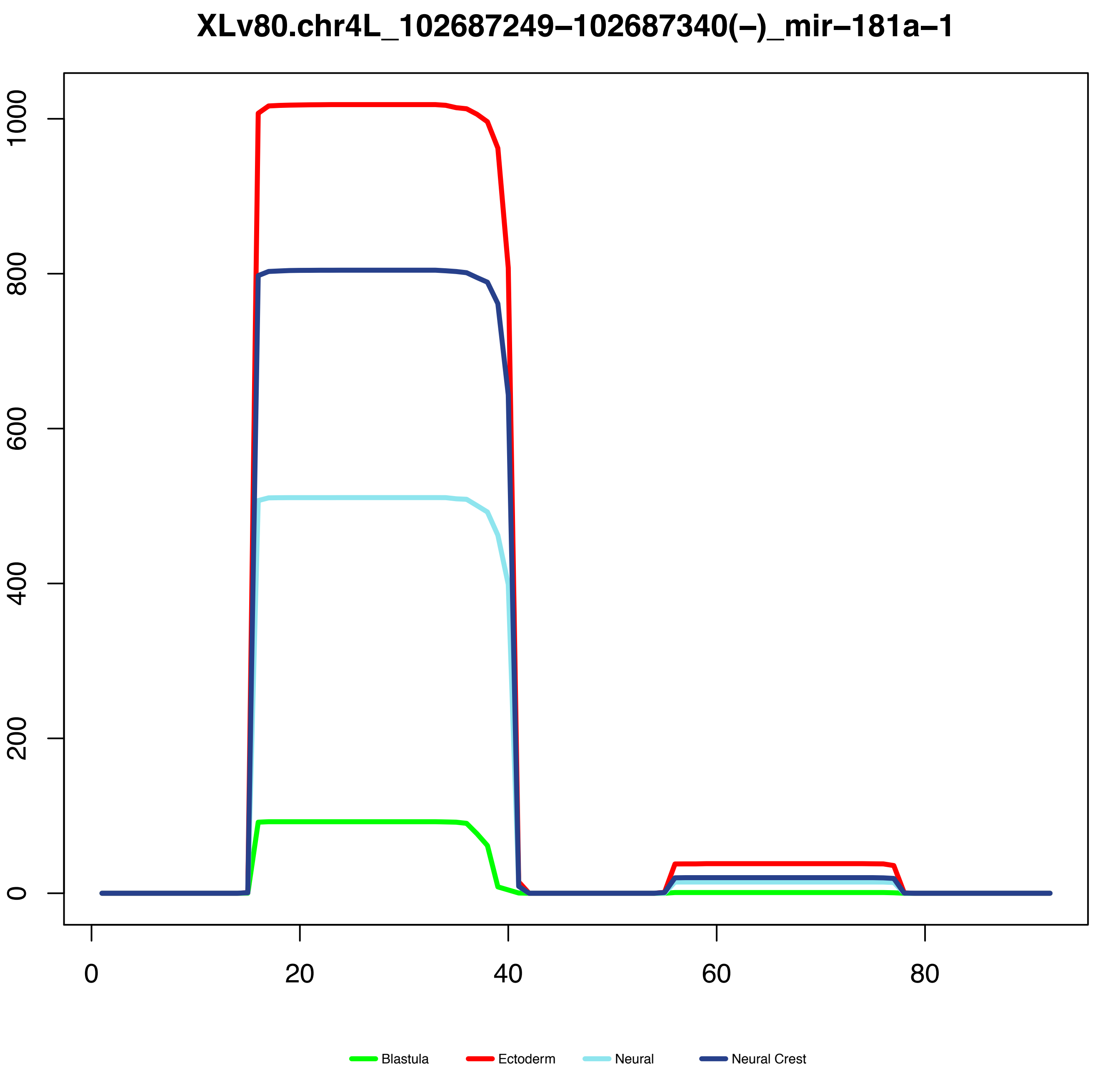

XLv80.chr4L_102687249−102687340(−)_mir−181a−1
0
200
400
600
800
1000
0
20
40
60
80
Blastula
Ectoderm
Neural
Neural Crest

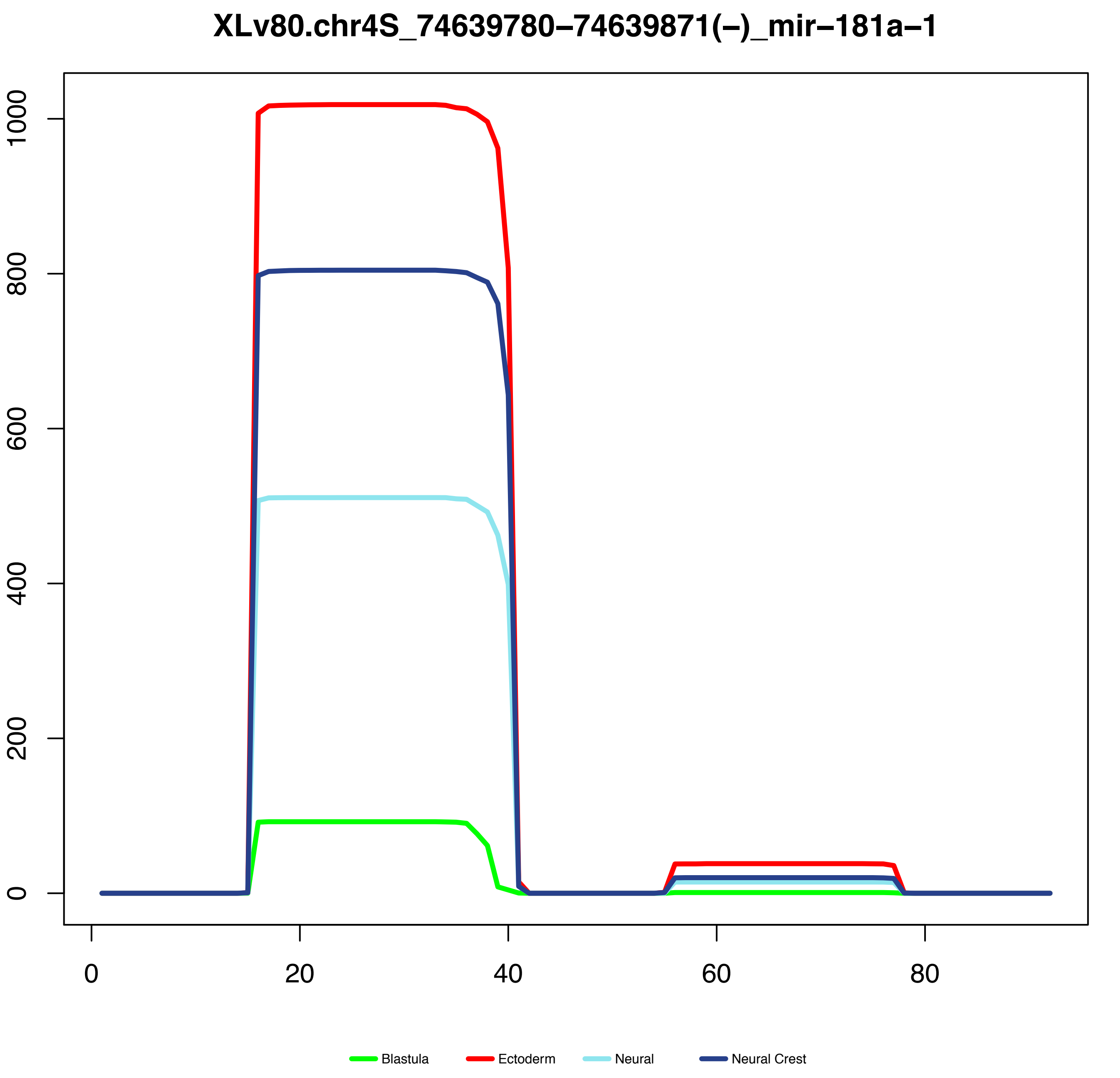

XLv80.chr4S_74639780−74639871(−)_mir−181a−1
0
200
400
600
800
1000
0
20
40
60
80
Blastula
Ectoderm
Neural
Neural Crest

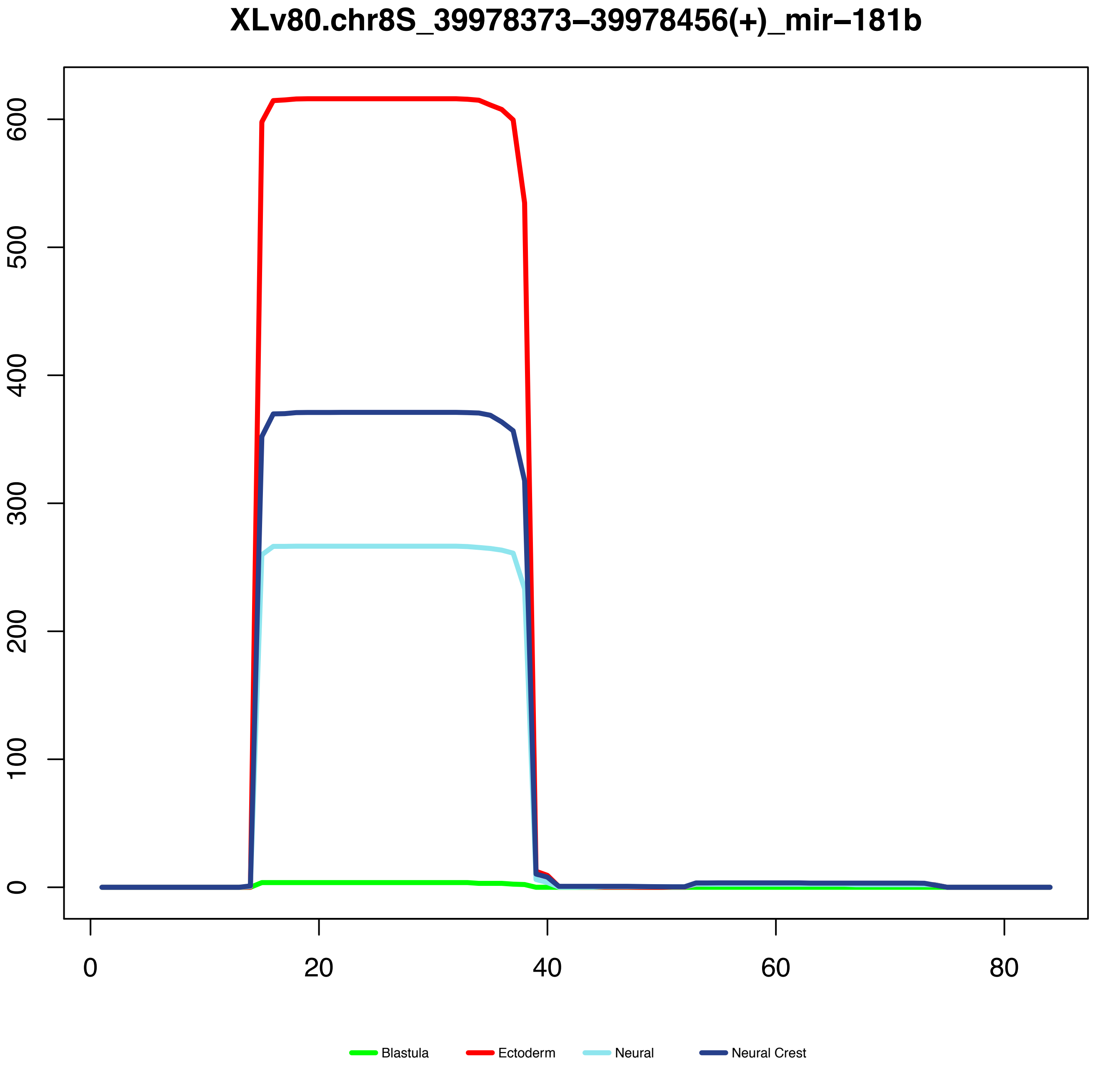
XLv80.chr8S_39978373−39978456(+)_mir−181b
0
100
200
300
400
500
600
0
20
40
60
80
Blastula
Ectoderm
Neural
Neural Crest

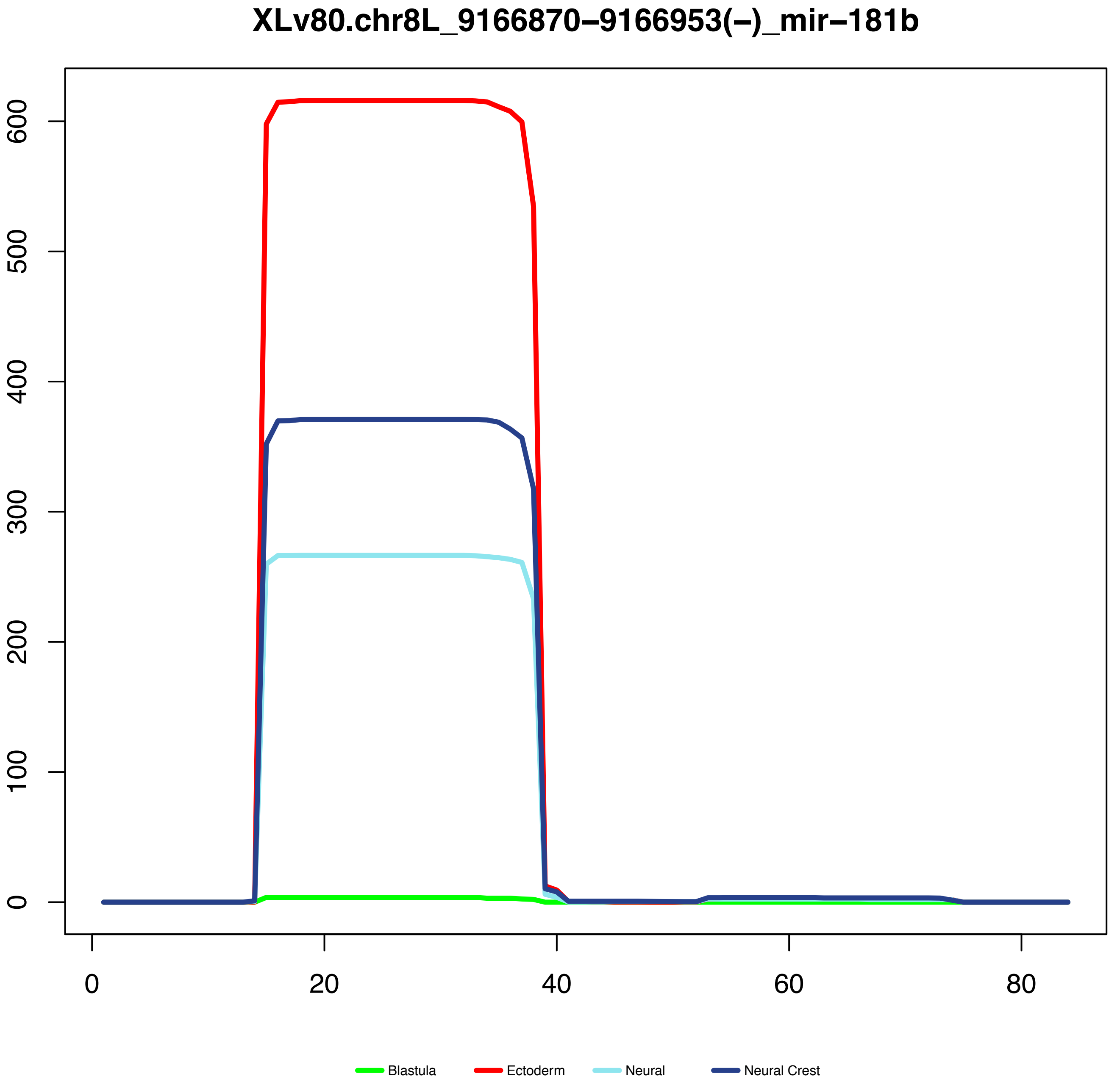
XLv80.chr8L_9166870−9166953(−)_mir−181b
0
100
200
300
400
500
600
0
20
40
60
80
Blastula
Ectoderm
Neural
Neural Crest

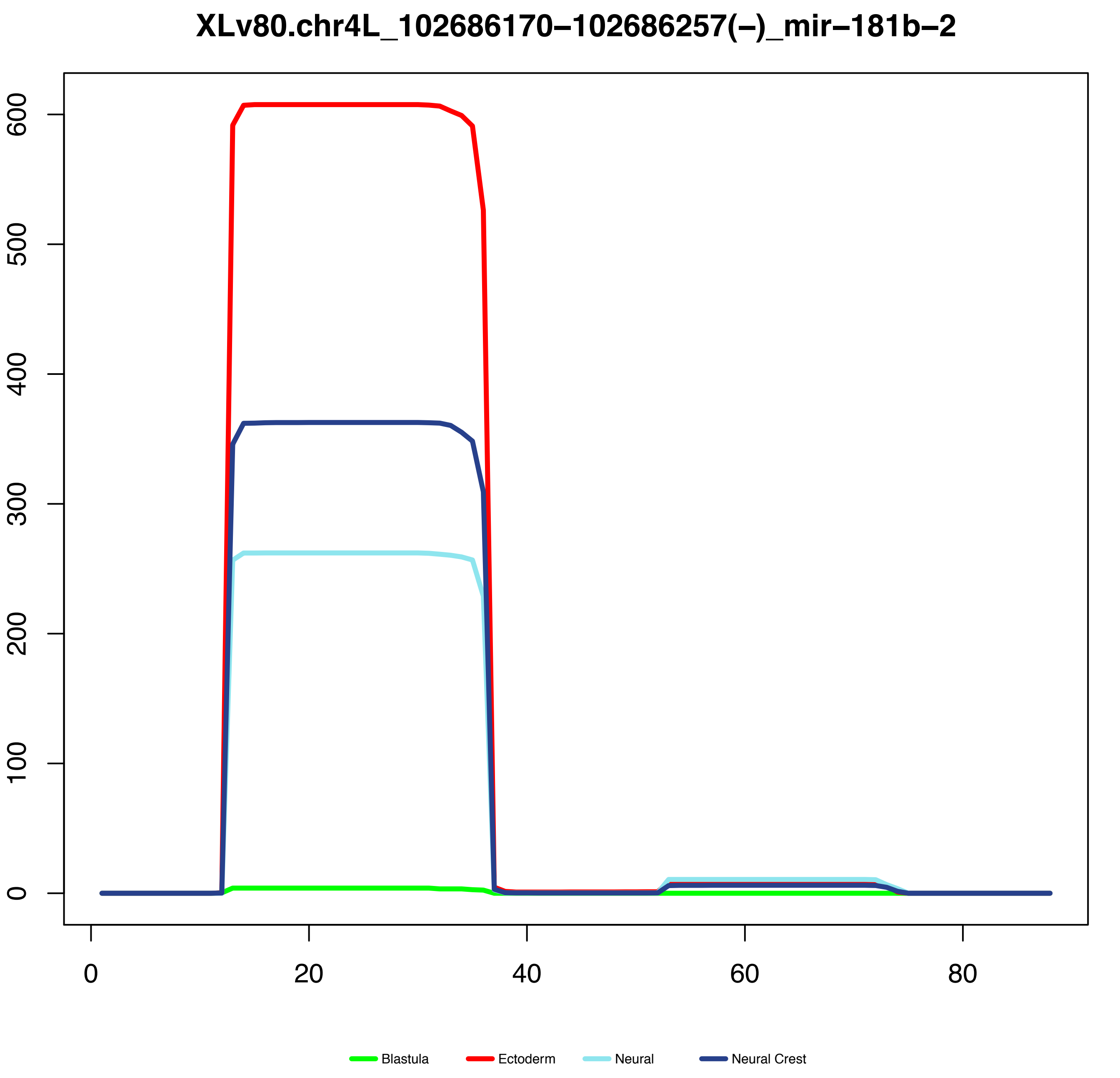
XLv80.chr4L_102686170−102686257(−)_mir−181b−2
0
100
200
300
400
500
600
0
20
40
60
80
Blastula
Ectoderm
Neural
Neural Crest

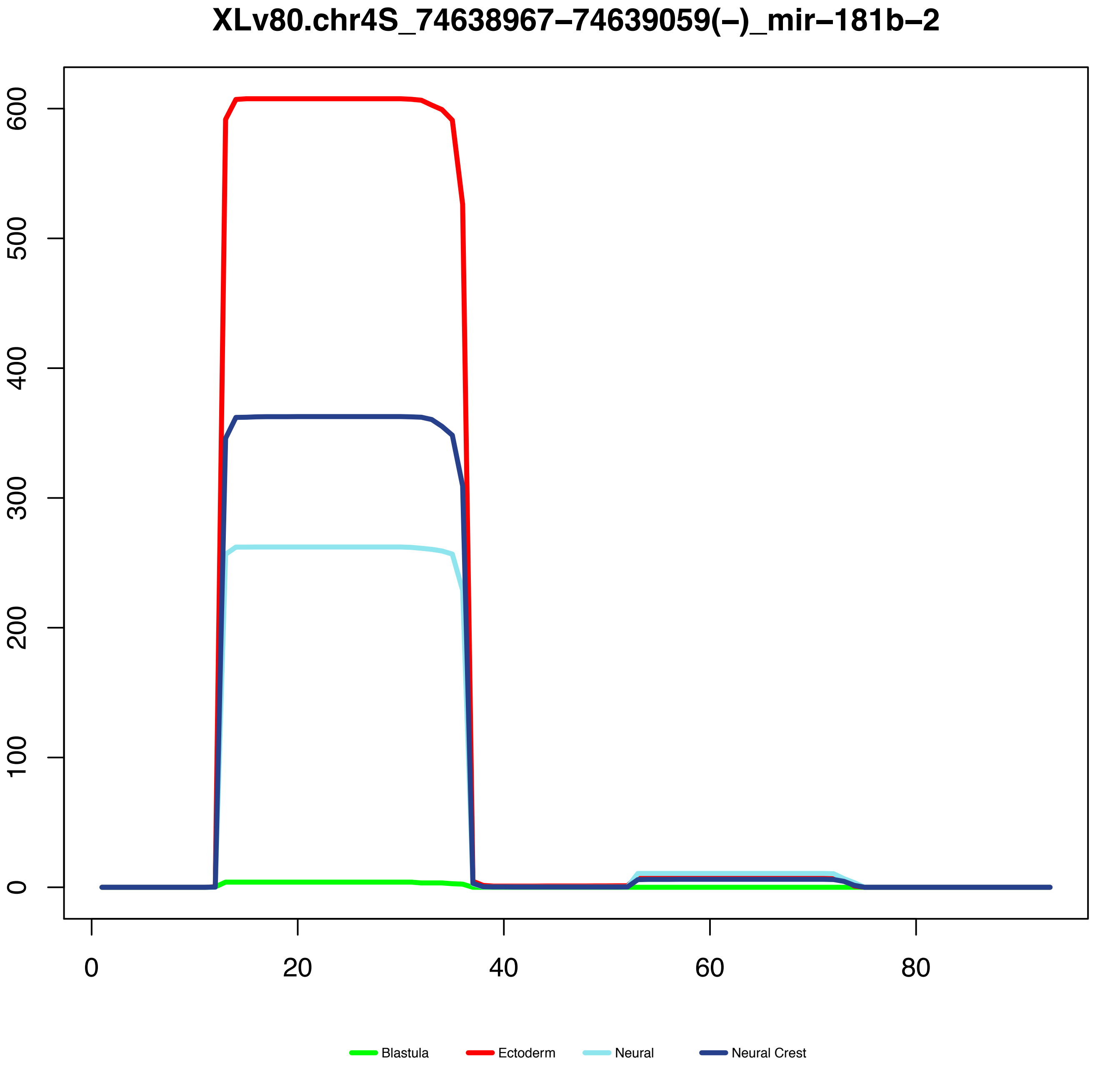
XLv80.chr4S_74638967−74639059(−)_mir−181b−2
0
100
200
300
400
500
600
0
20
40
60
80
Blastula
Ectoderm
Neural
Neural Crest

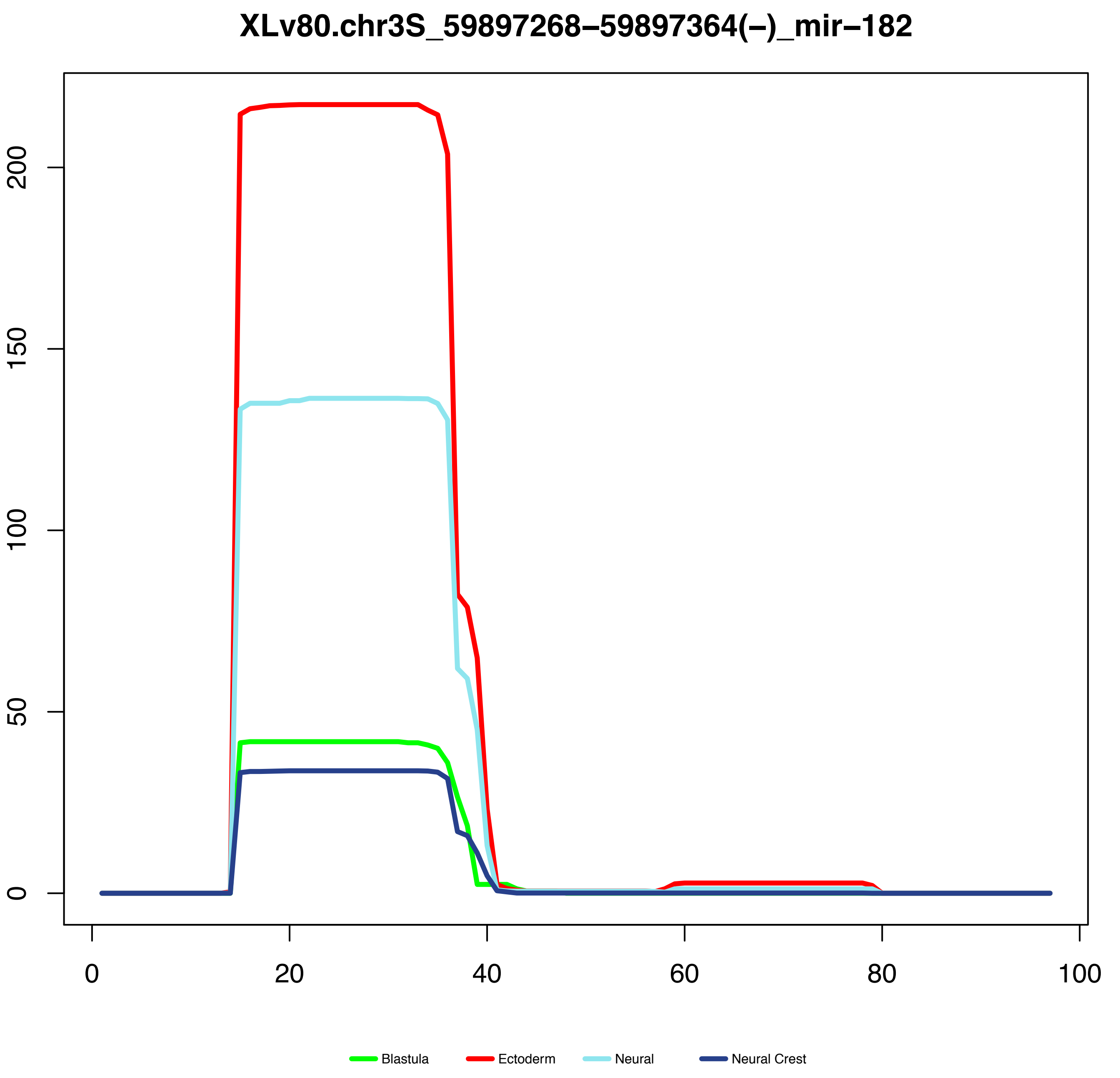

XLv80.chr3S_59897268−59897364(−)_mir−182
0
50
100
150
200
0
20
40
60
80
100
Blastula
Ectoderm
Neural
Neural Crest

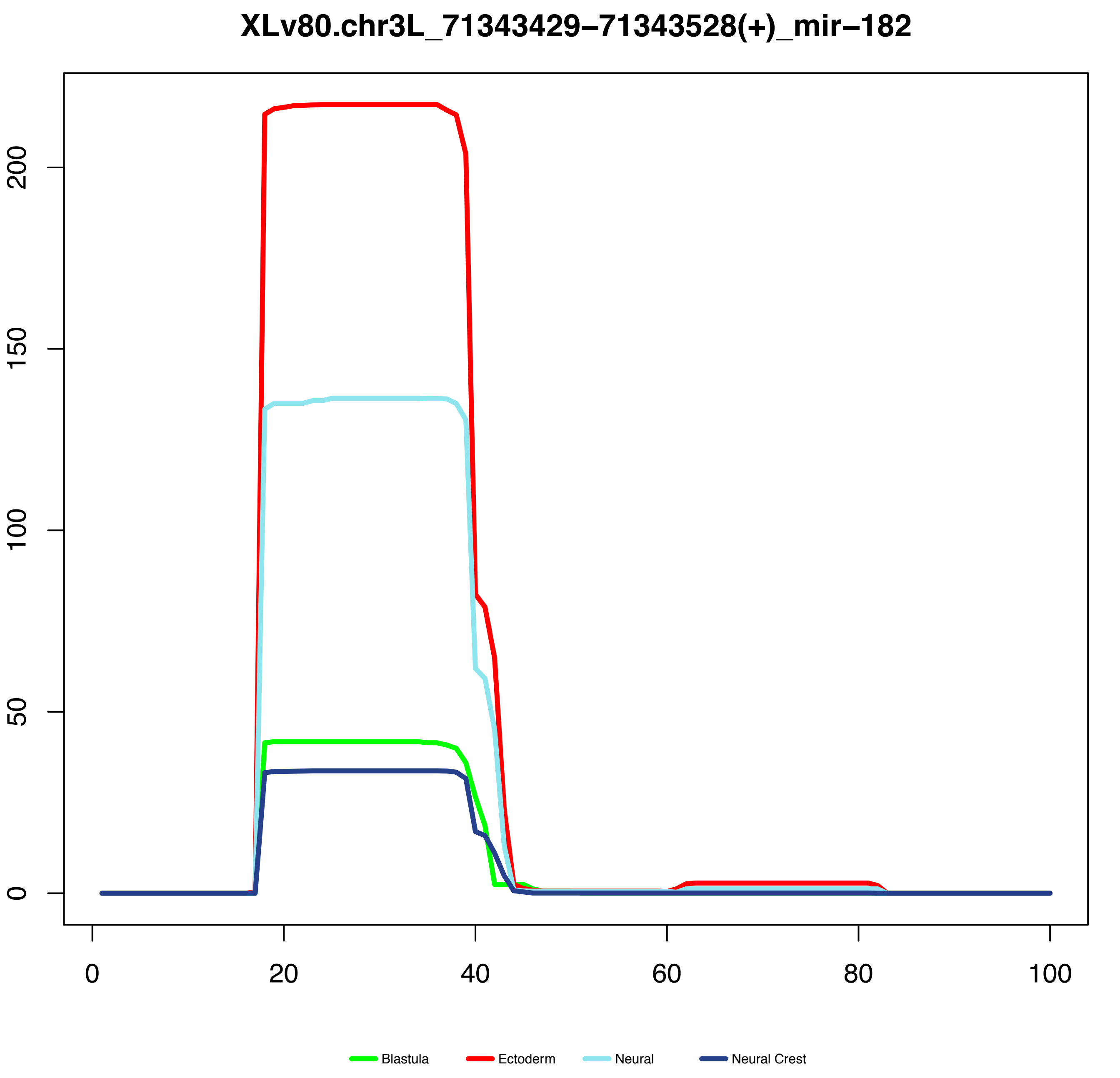

XLv80.chr3L_71343429−71343528(+)_mir−182
0
50
100
150
200
0
20
40
60
80
100
Blastula
Ectoderm
Neural
Neural Crest

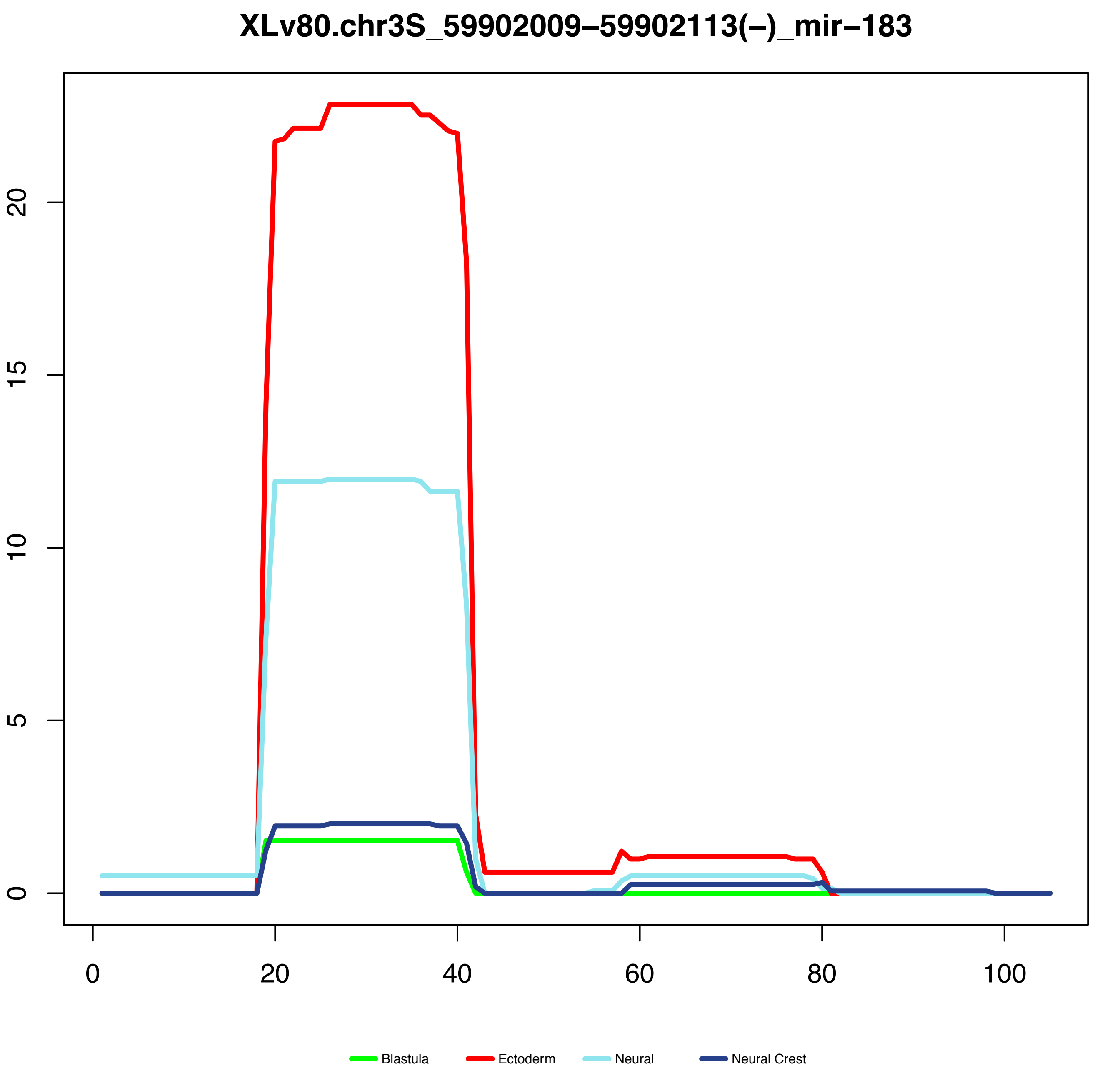
XLv80.chr3S_59902009−59902113(−)_mir−183
0
5
10
15
20
0
20
40
60
80
100
Blastula
Ectoderm
Neural
Neural Crest

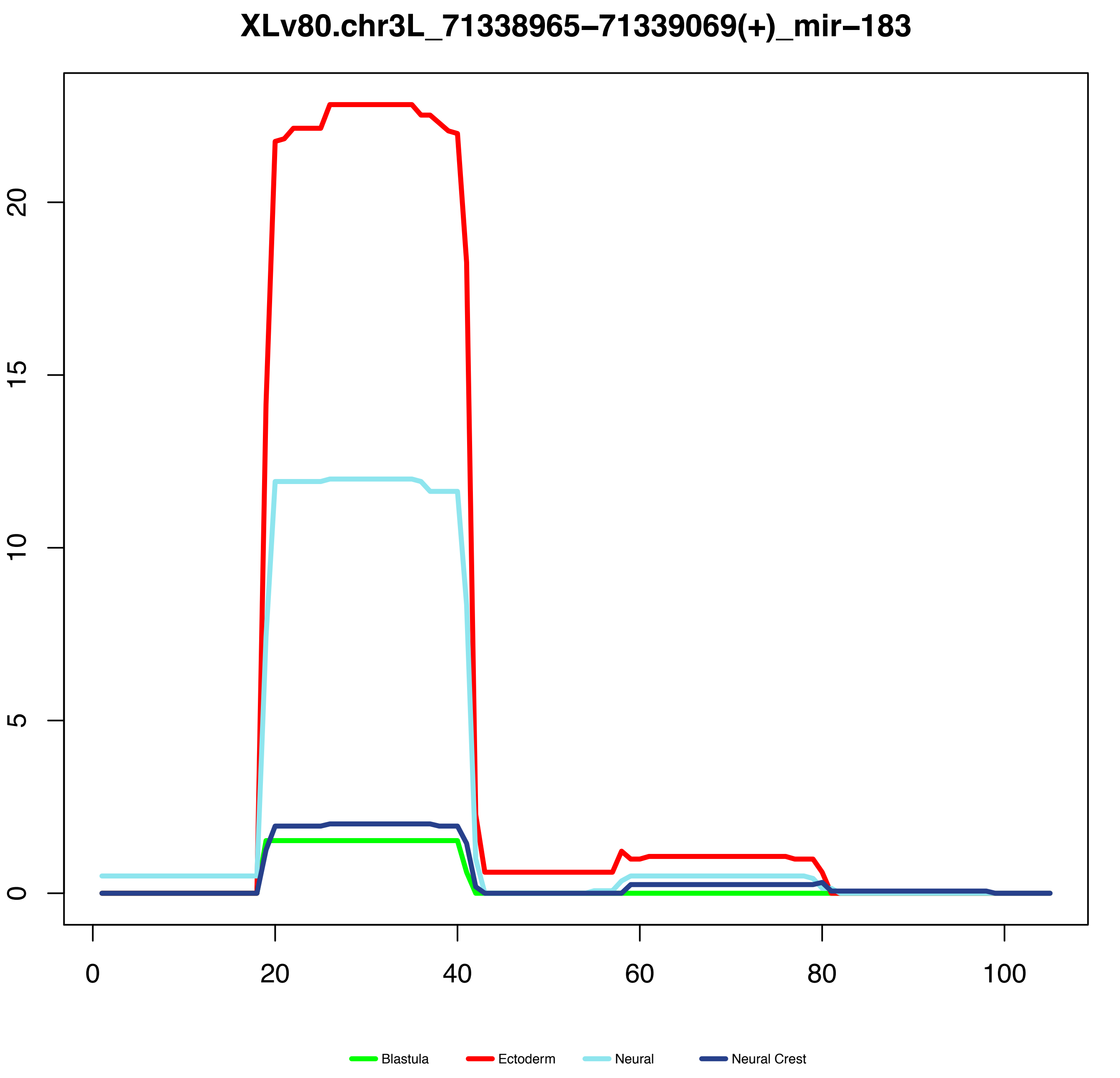
XLv80.chr3L_71338965−71339069(+)_mir−183
0
5
10
15
20
0
20
40
60
80
100
Blastula
Ectoderm
Neural
Neural Crest

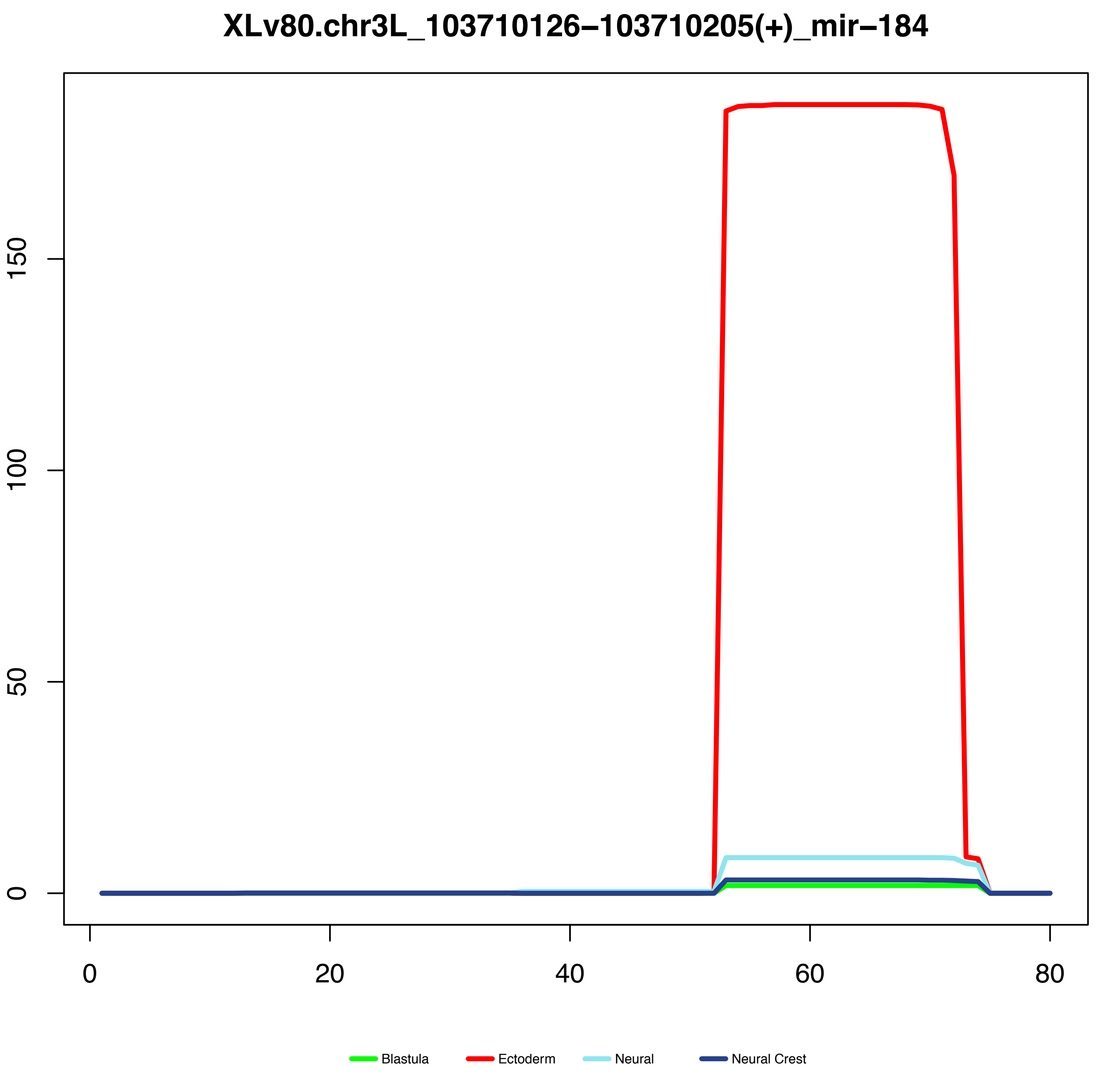
XLv80.chr3L_103710126−103710205(+)_mir−184
0
50
100
150
0
20
40
60
80
Blastula
Ectoderm
Neural
Neural Crest

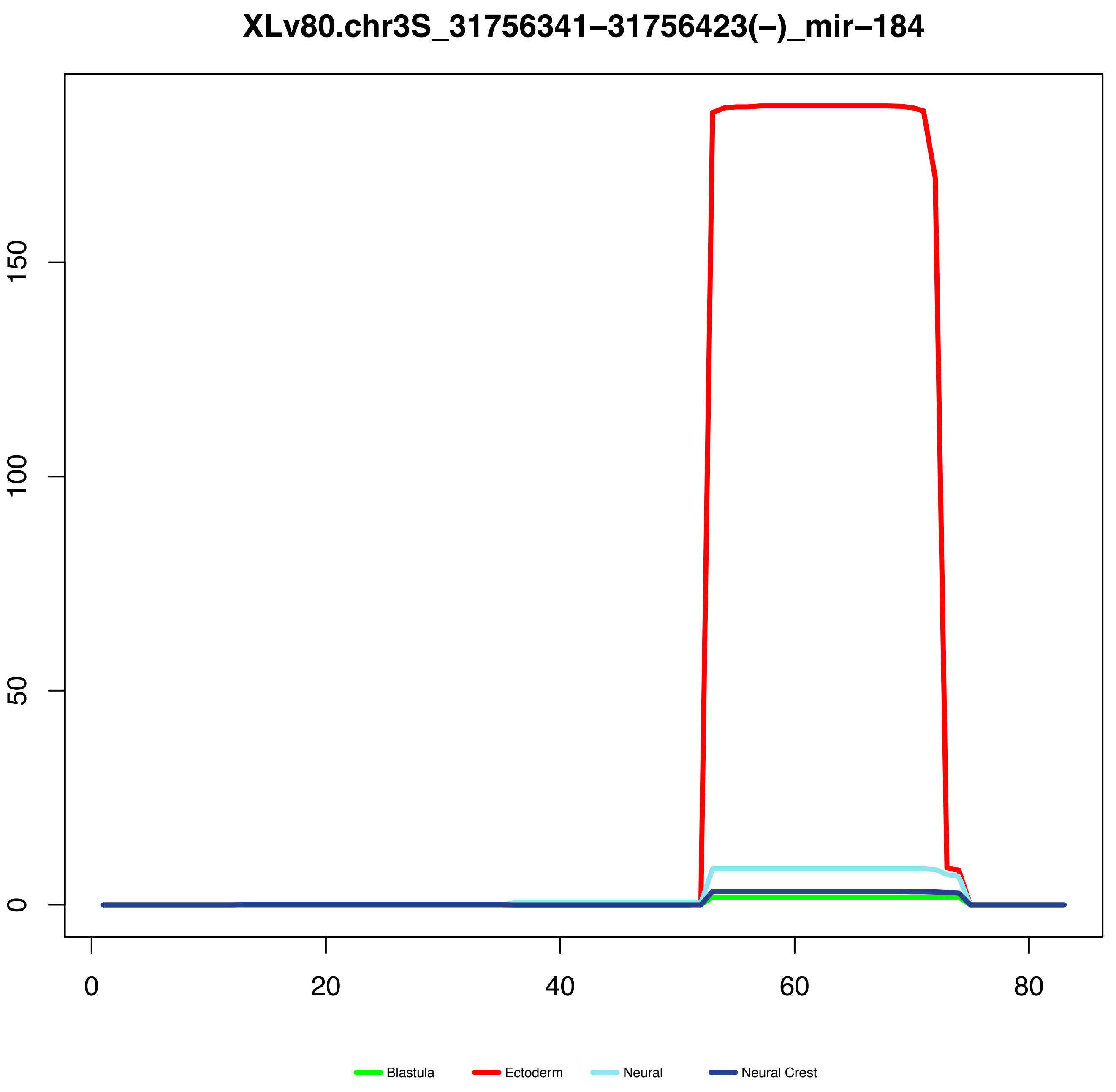
XLv80.chr3S_31756341–31756423(–)_mir–184
0
50
100
150
0
20
40
60
80
Blastula
Ectoderm
Neural
Neural Crest

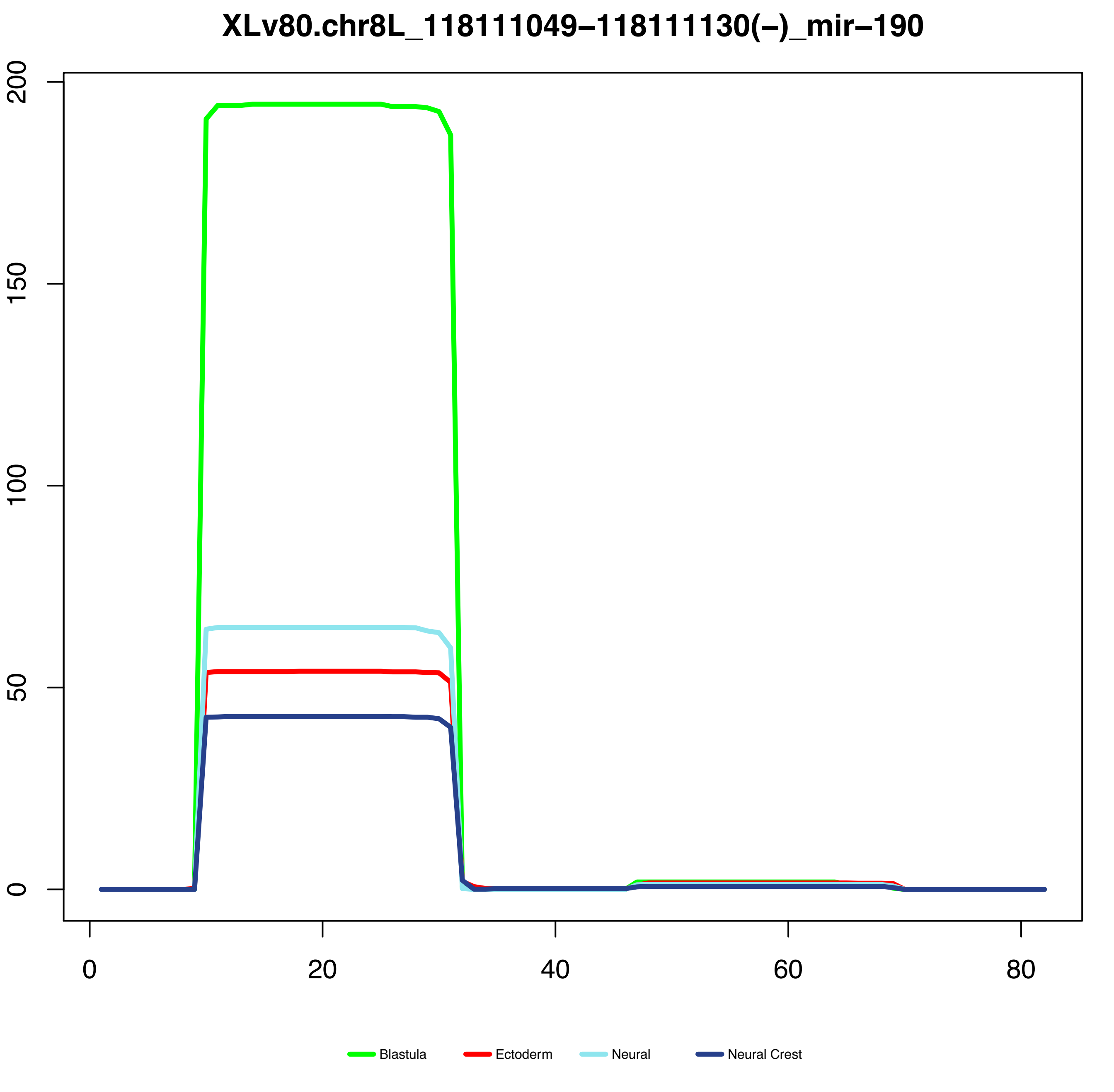
XLv80.chr8L_118111049−118111130(−)_mir−190
0
50
100
150
200
0
20
40
60
80
Blastula
Ectoderm
Neural
Neural Crest

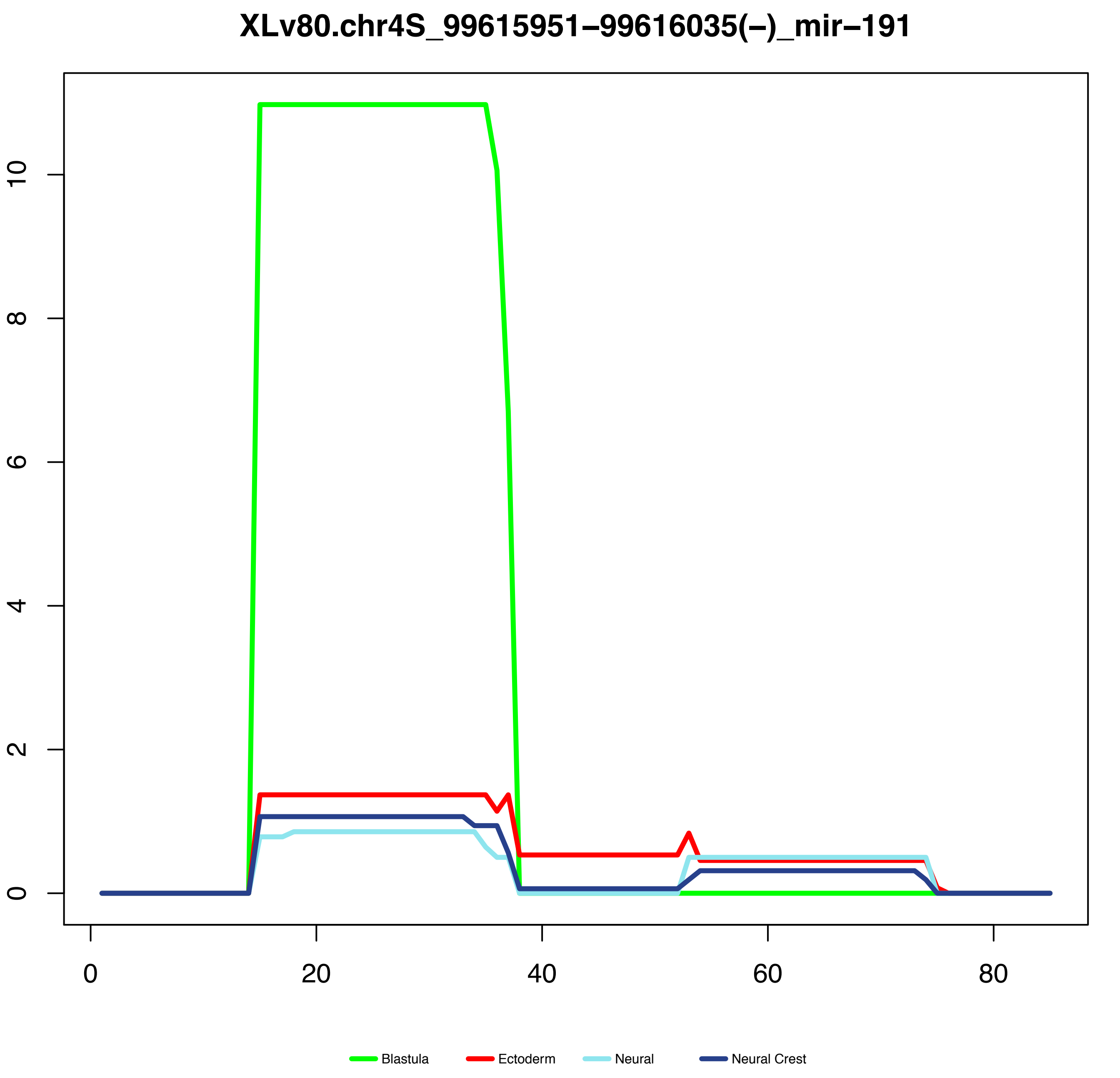
XLv80.chr4S_99615951−99616035(−)_mir−191
0
2
4
6
8
10
0
20
40
60
80
Blastula
Ectoderm
Neural
Neural Crest

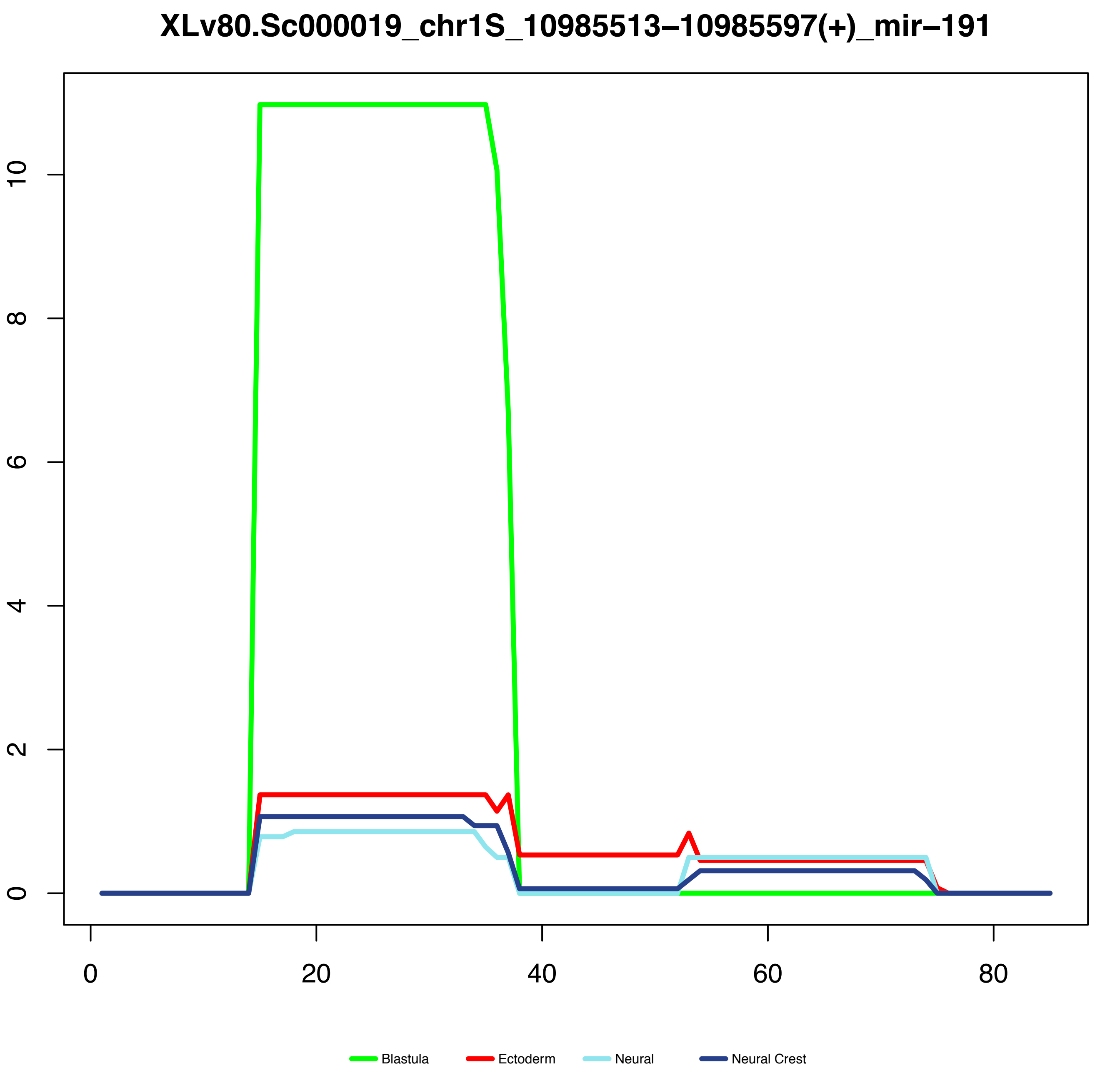

XLv80.Sc000019_chr1S_10985513−10985597(+)_mir−191
0
2
4
6
8
10
0
20
40
60
80
Blastula
Ectoderm
Neural
Neural Crest

XLv80.chr9_10L_90417008−90417091(−)_mir−193b

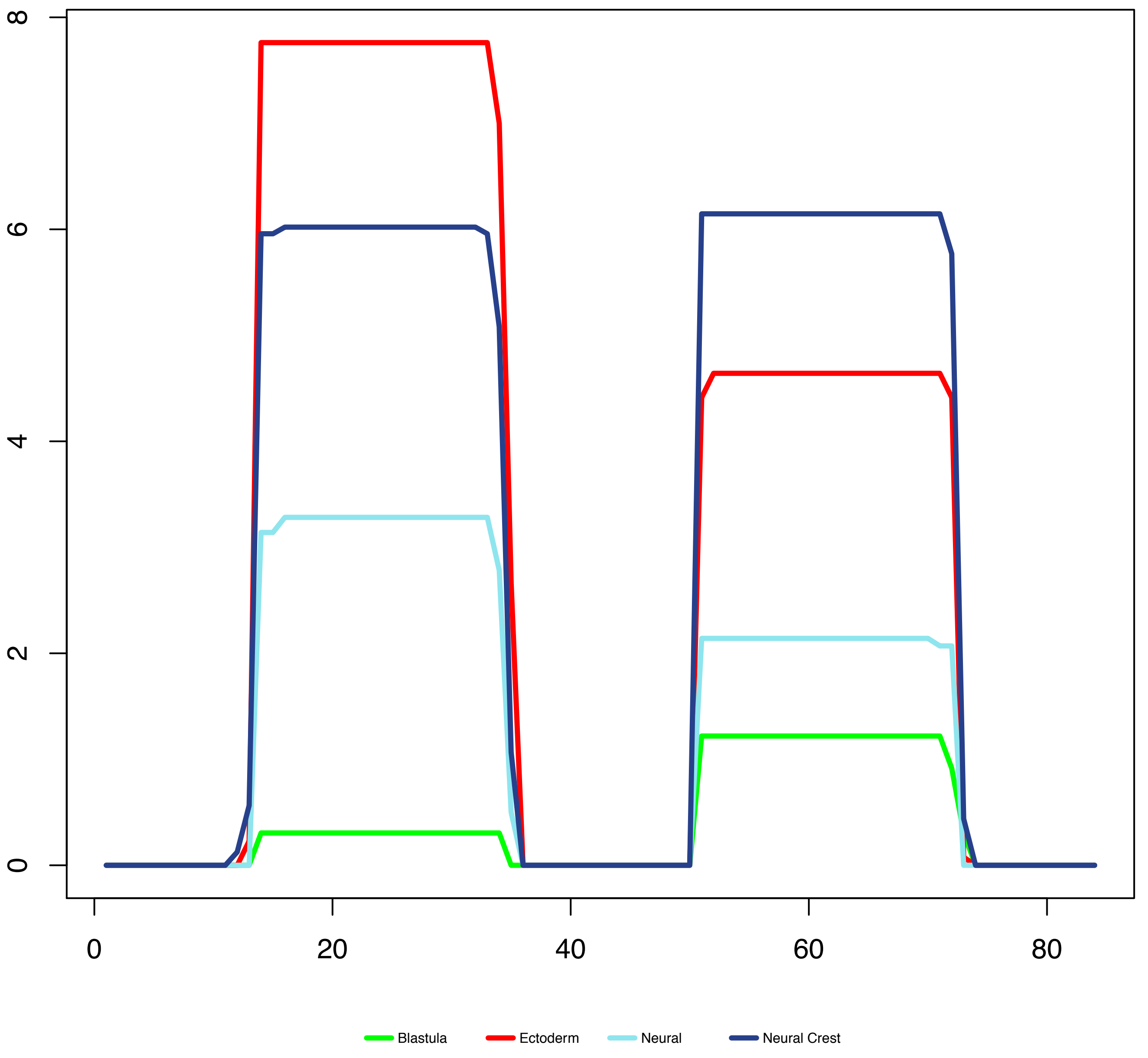

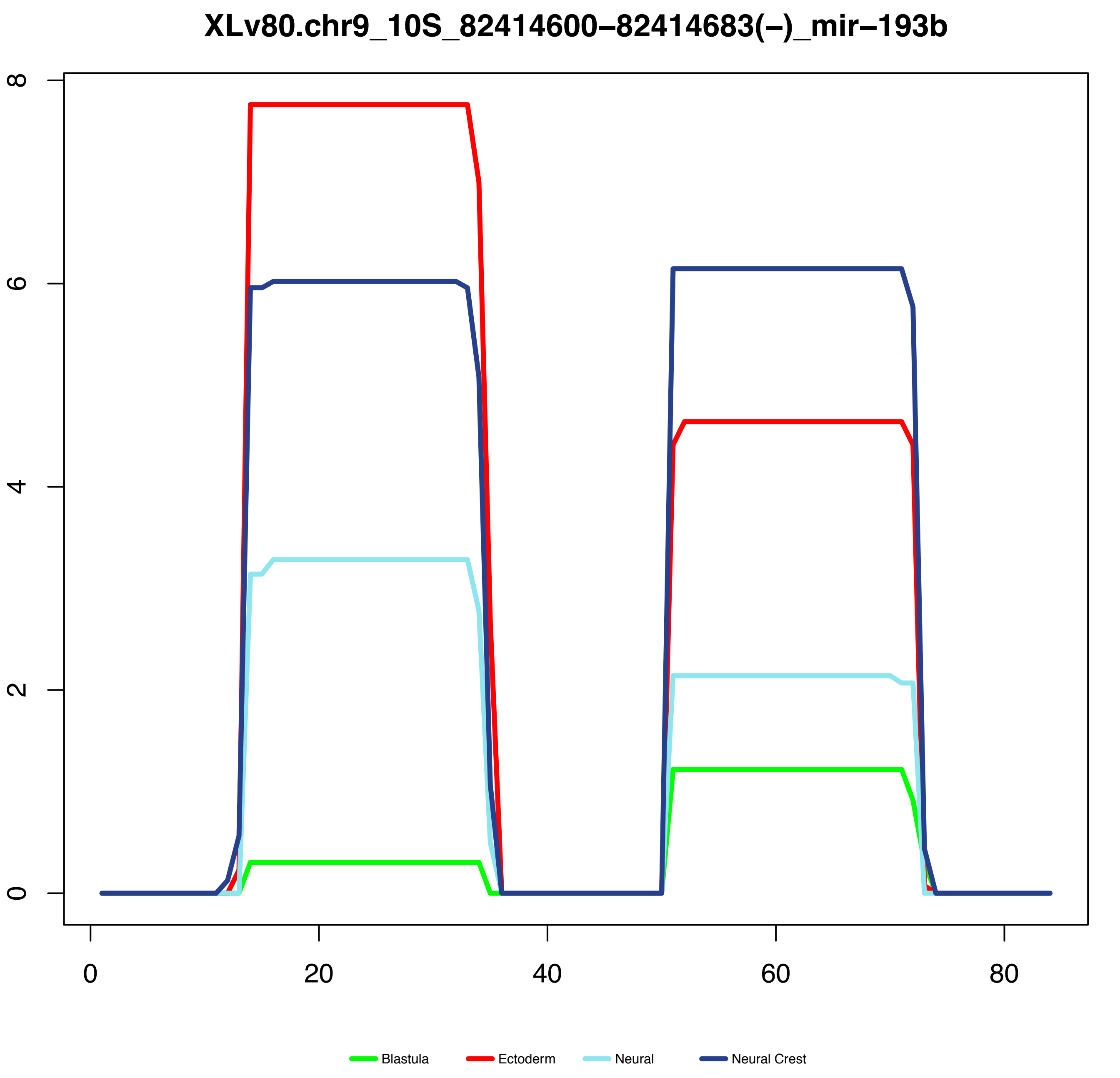
XLv80.chr9_10S_82414600–82414683(–)_mir–193b
0
2
4
6
8
0
20
40
60
80
Blastula
Ectoderm
Neural
Neural Crest

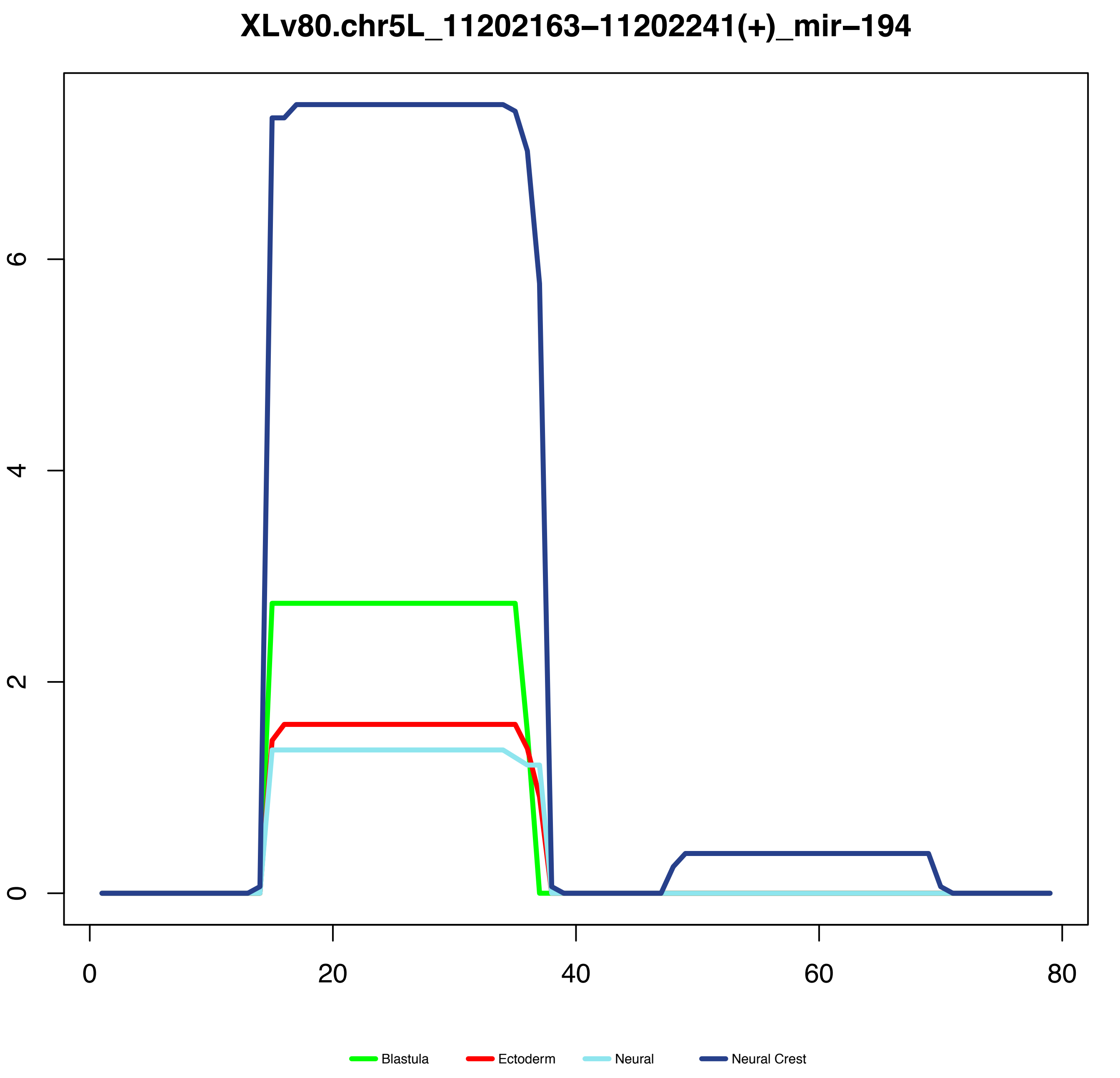
XLv80.chr5L_11202163−11202241(+)_mir−194
0
2
4
6
0
20
40
60
80
Blastula
Ectoderm
Neural
Neural Crest

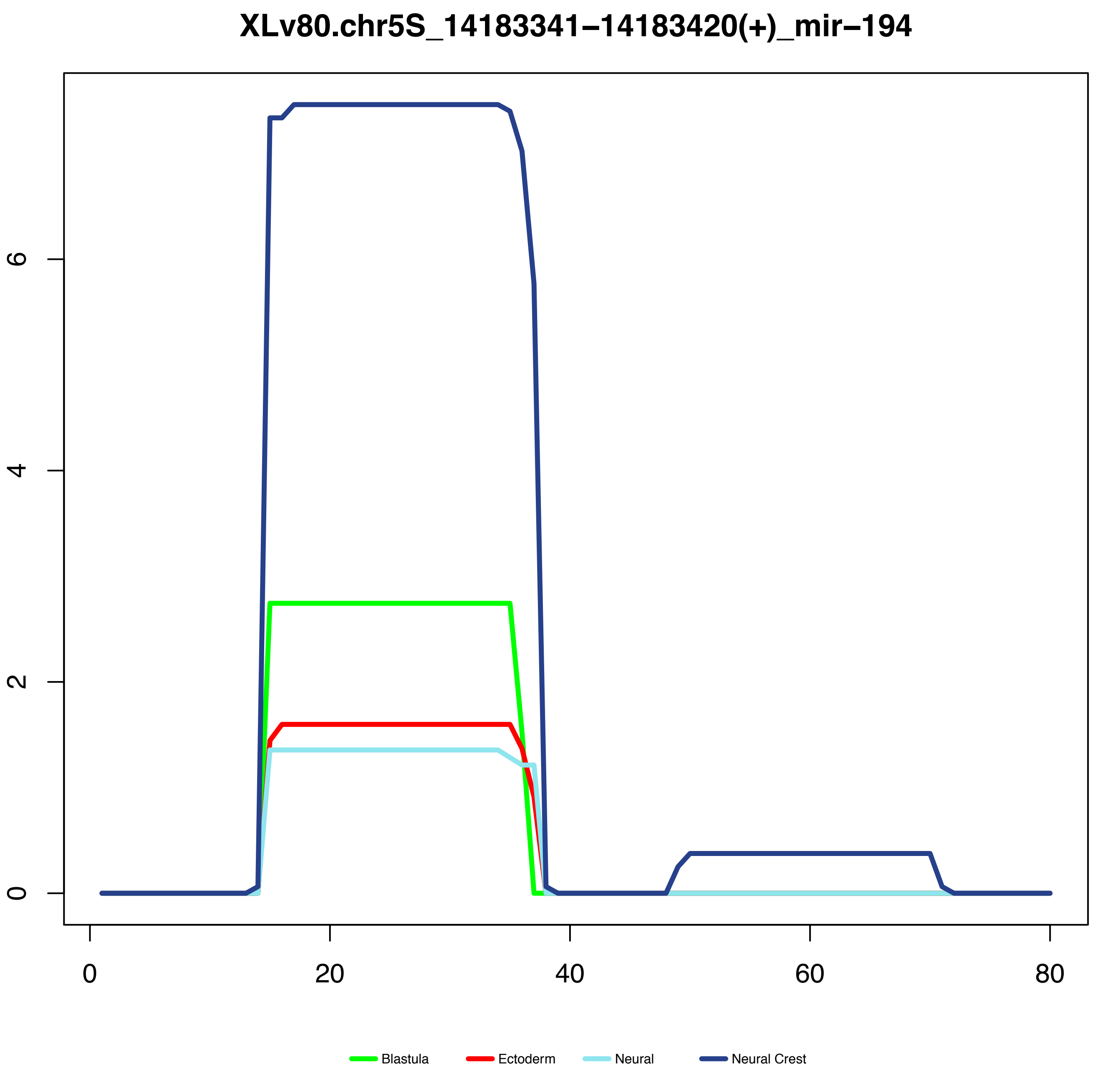

XLv80.chr5S_14183341−14183420(+)_mir−194
0
2
4
6
0
20
40
60
80
Blastula
Ectoderm
Neural
Neural Crest

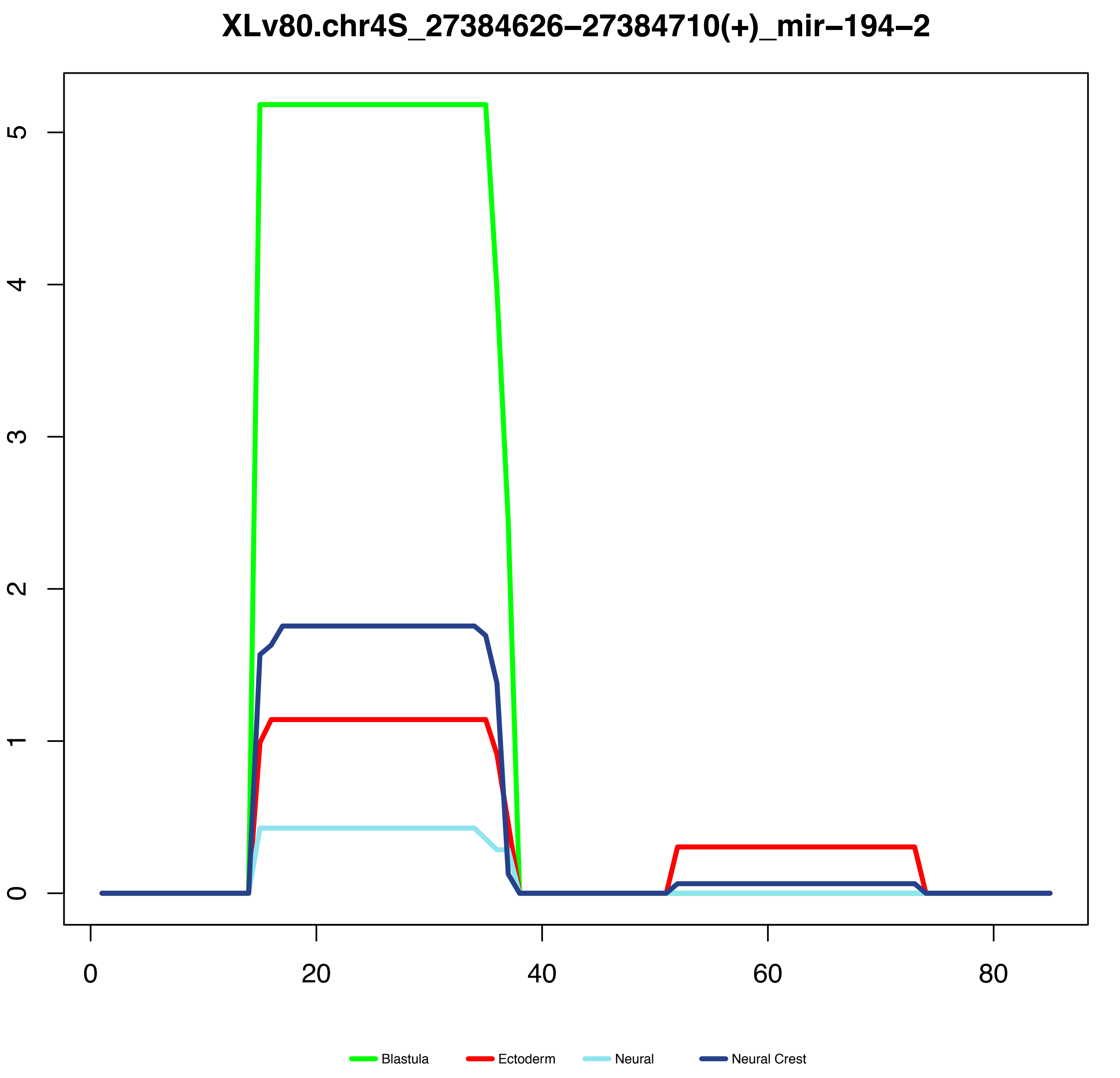
XLv80.chr4S_27384626-27384710(+)_mir-194-2
0
1
2
3
4
5
0
20
40
60
80
Blastula
Ectoderm
Neural
Neural Crest

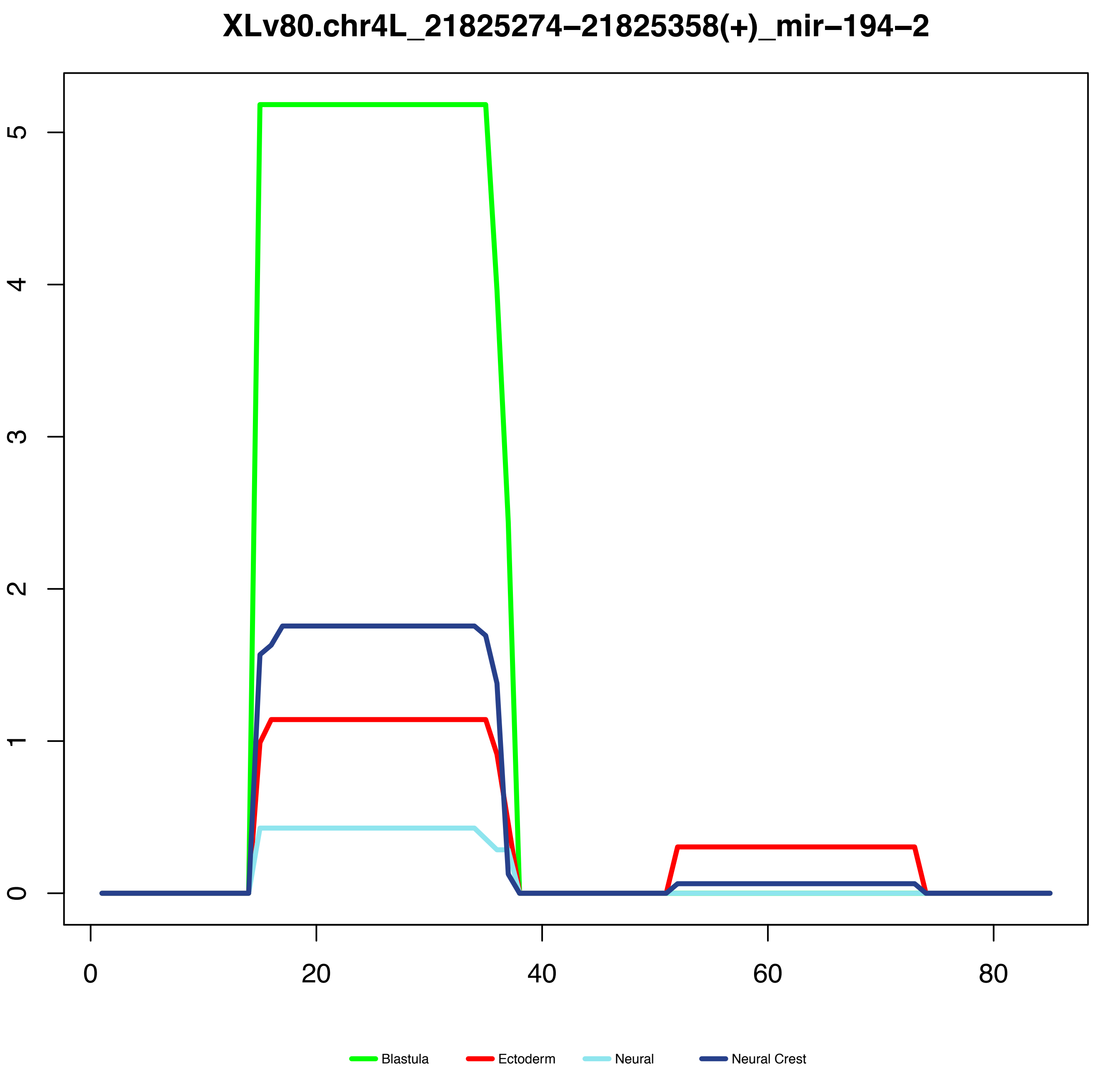
XLv80.chr4L_21825274−21825358(+)_mir−194−2
0
1
2
3
4
5
0
20
40
60
80
Blastula
Ectoderm
Neural
Neural Crest

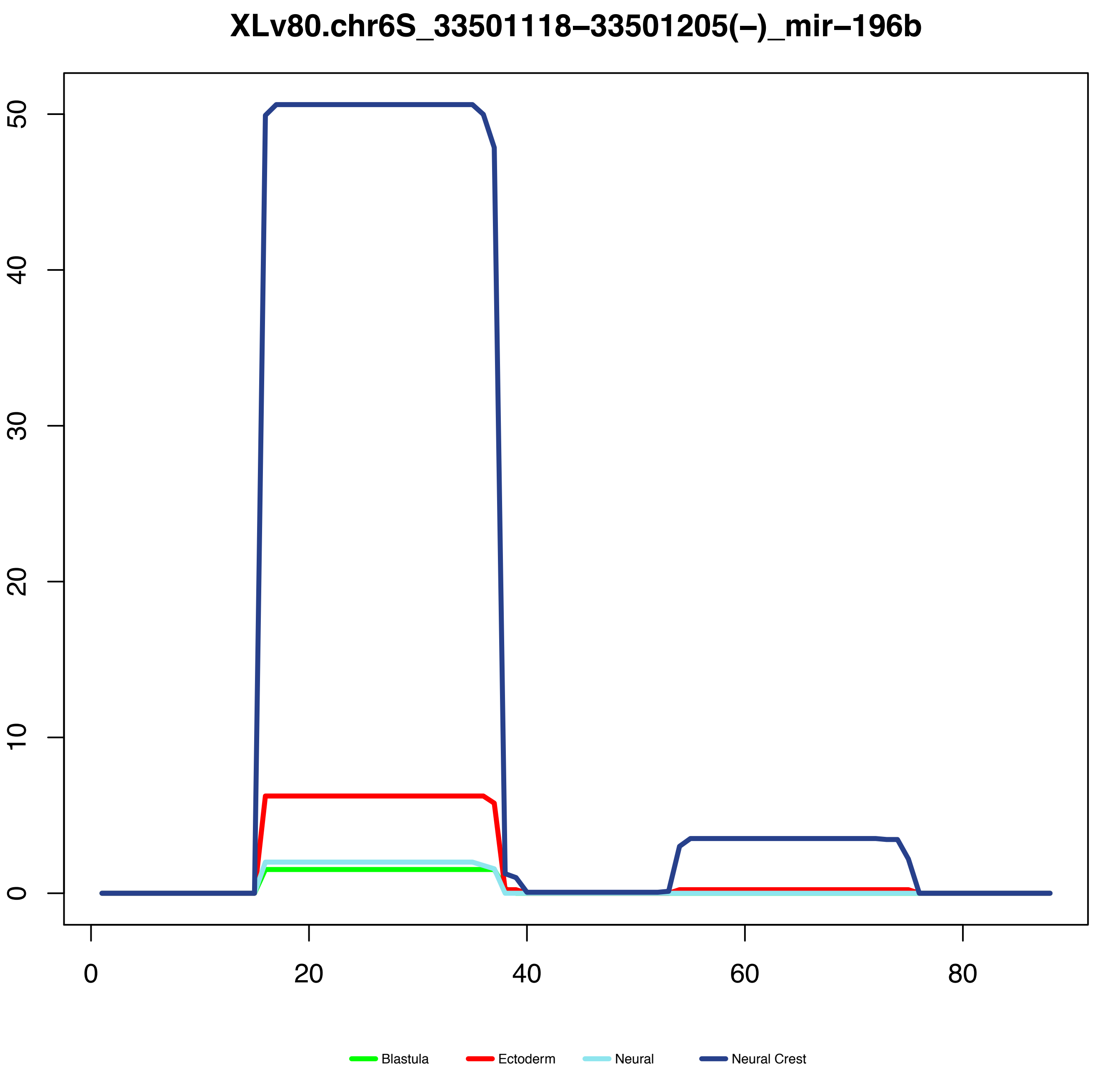

XLv80.chr6S_33501118−33501205(−)_mir−196b
0
10
20
30
40
50
0
20
40
60
80
Blastula
Ectoderm
Neural
Neural Crest

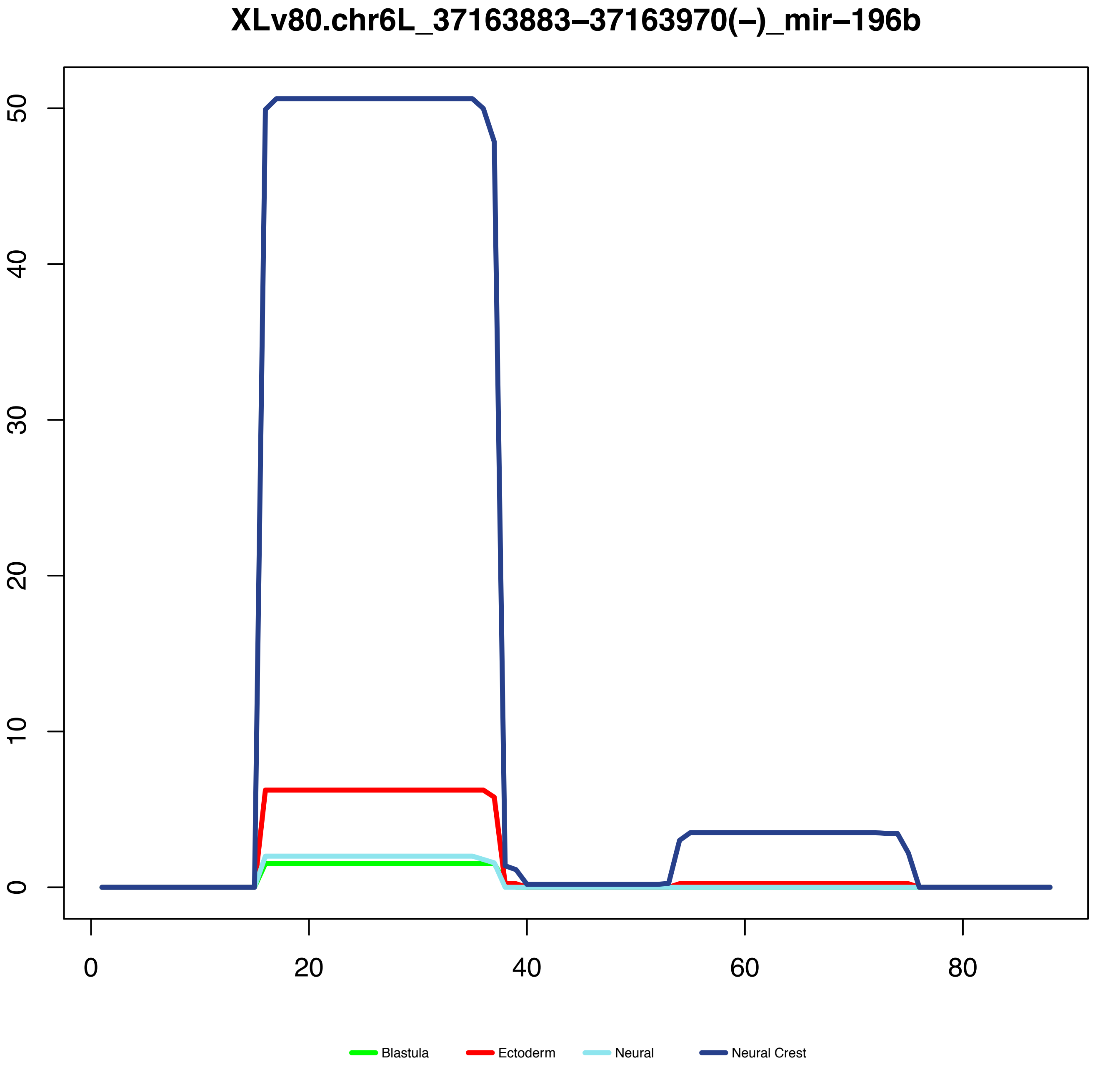

XLv80.chr6L_37163883−37163970(−)_mir−196b
0
10
20
30
40
50
0
20
40
60
80
Blastula
Ectoderm
Neural
Neural Crest

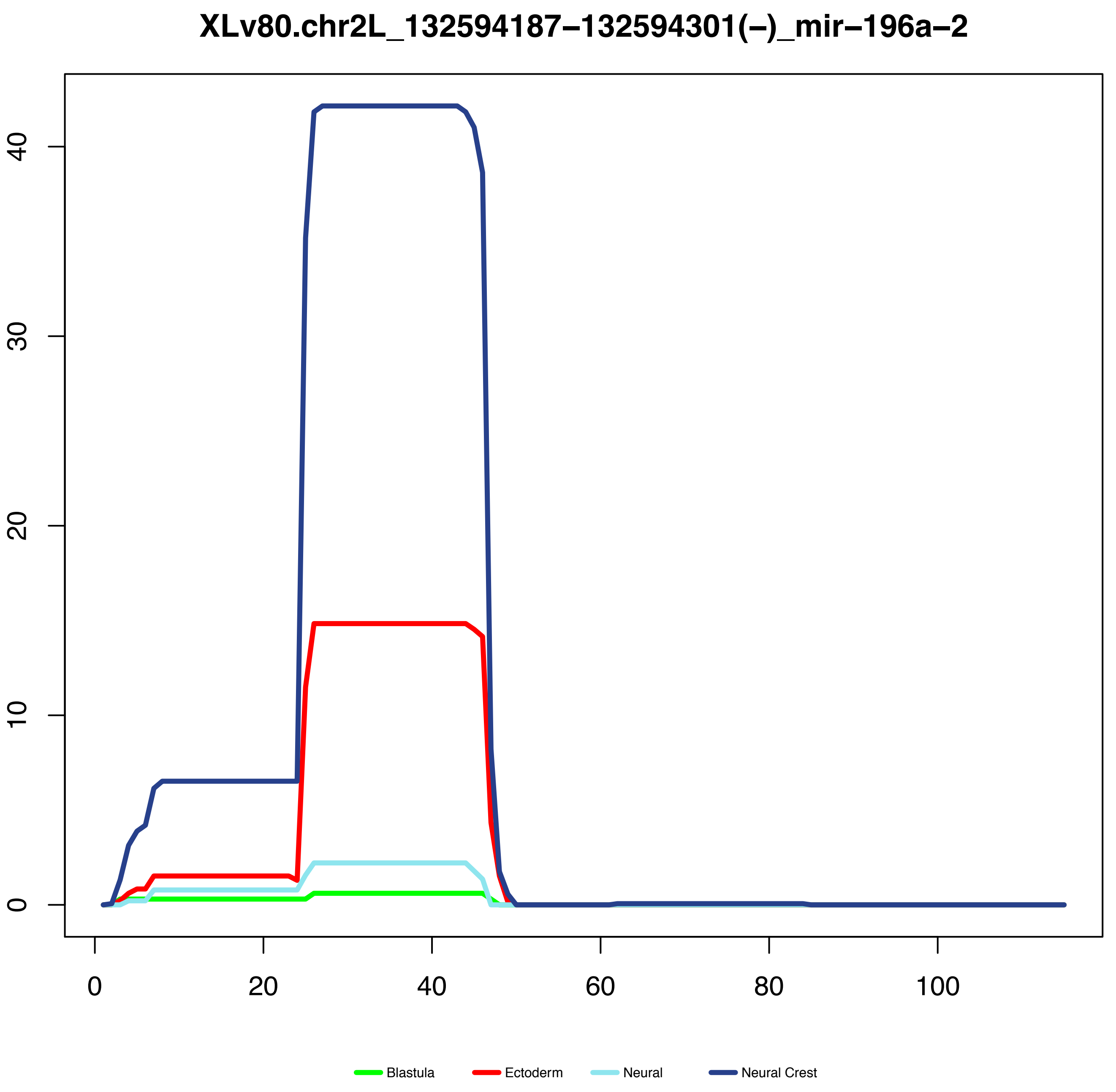
XLv80.chr2L_132594187−132594301(−)_mir−196a−2
0
10
20
30
40
0
20
40
60
80
100
Blastula
Ectoderm
Neural
Neural Crest

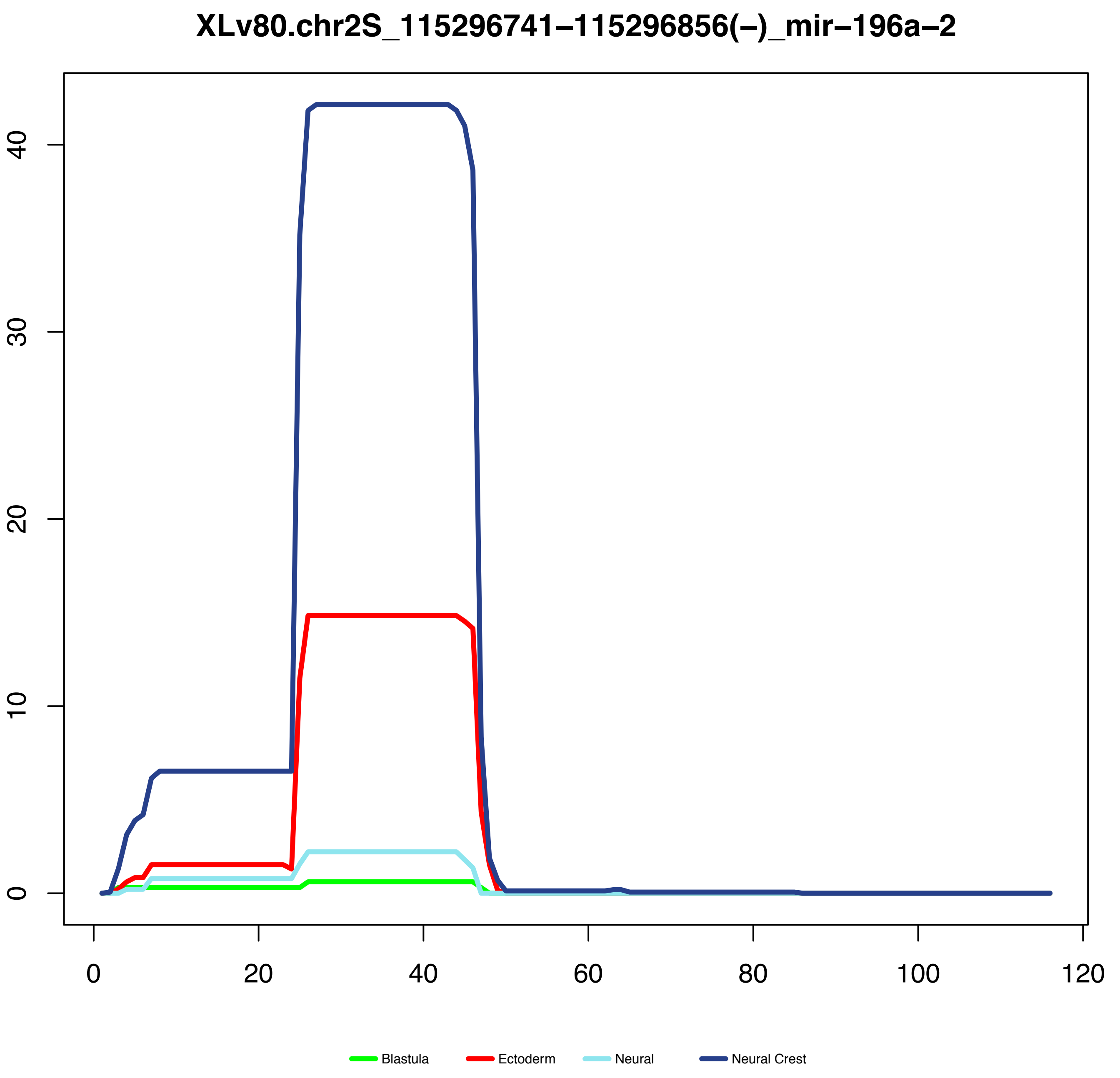
XLv80.chr2S_115296741−115296856(−)_mir−196a−2
0
10
20
30
40
0
20
40
60
80
100
120
Blastula
Ectoderm
Neural
Neural Crest

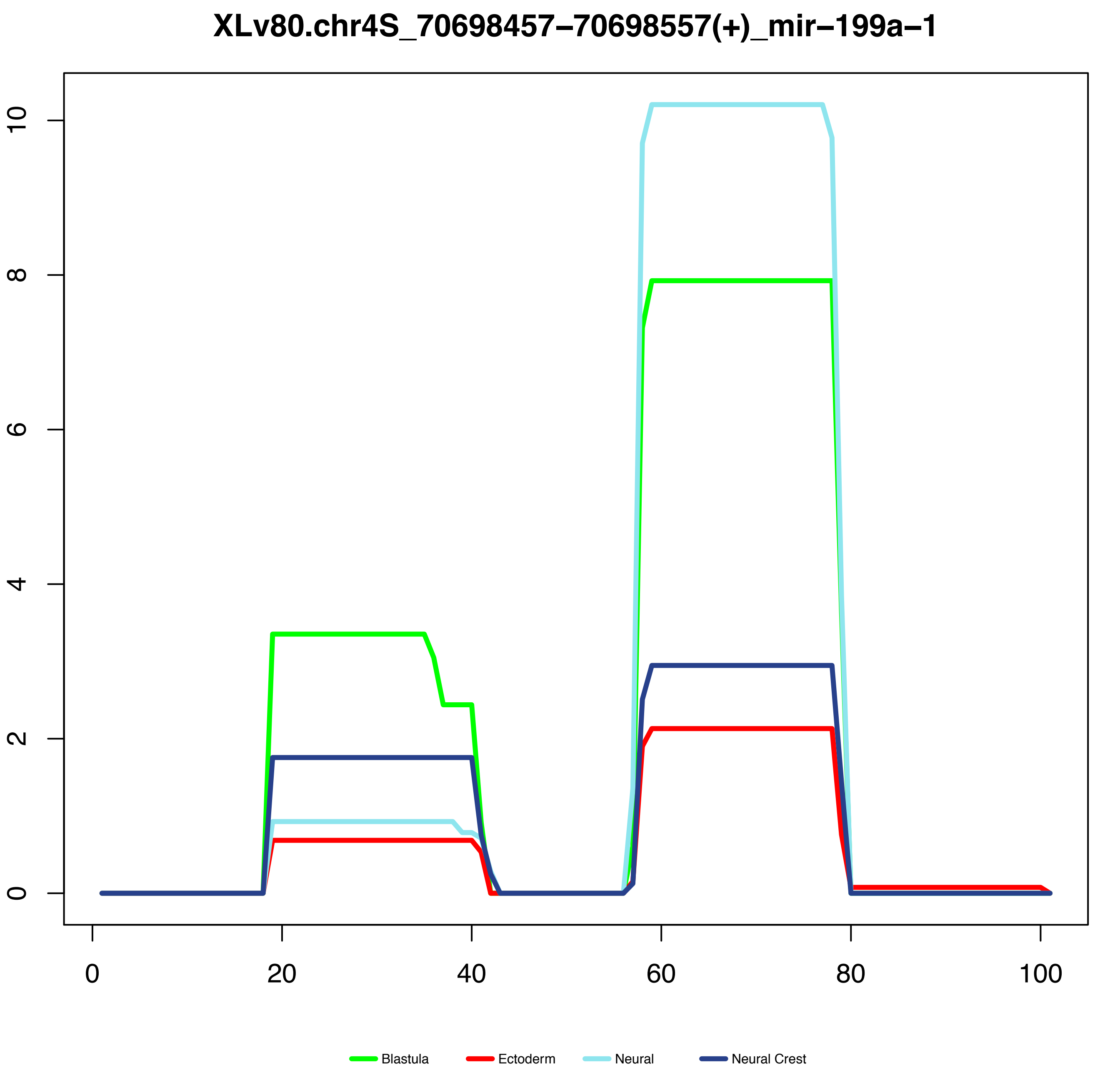
XLv80.chr4S_70698457−70698557(+)_mir−199a−1
0
2
4
6
8
10
0
20
40
60
80
100
Blastula
Ectoderm
Neural
Neural Crest

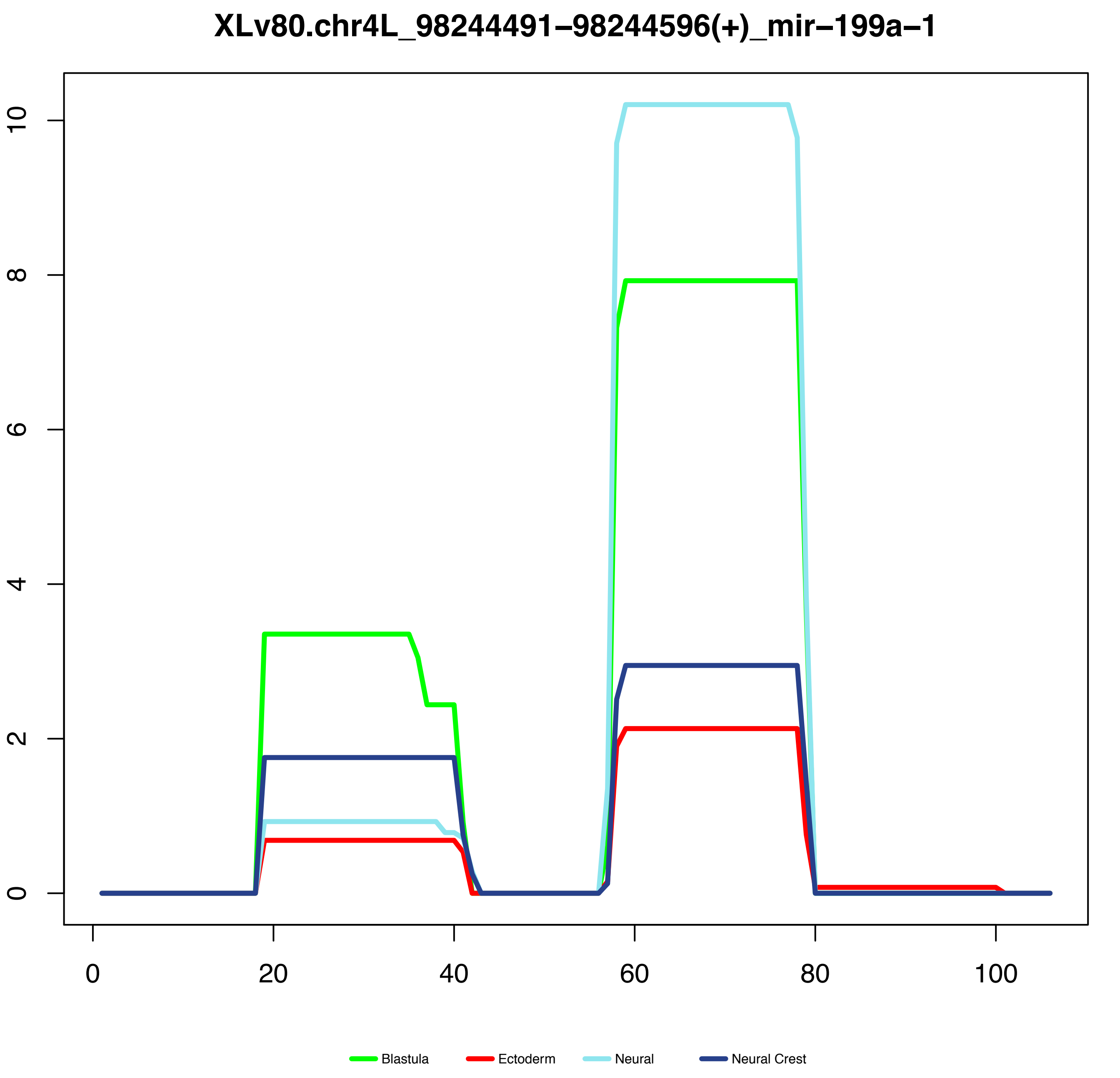
XLv80.chr4L_98244491−98244596(+)_mir−199a−1
0
2
4
6
8
10
0
20
40
60
80
100
Blastula
Ectoderm
Neural
Neural Crest

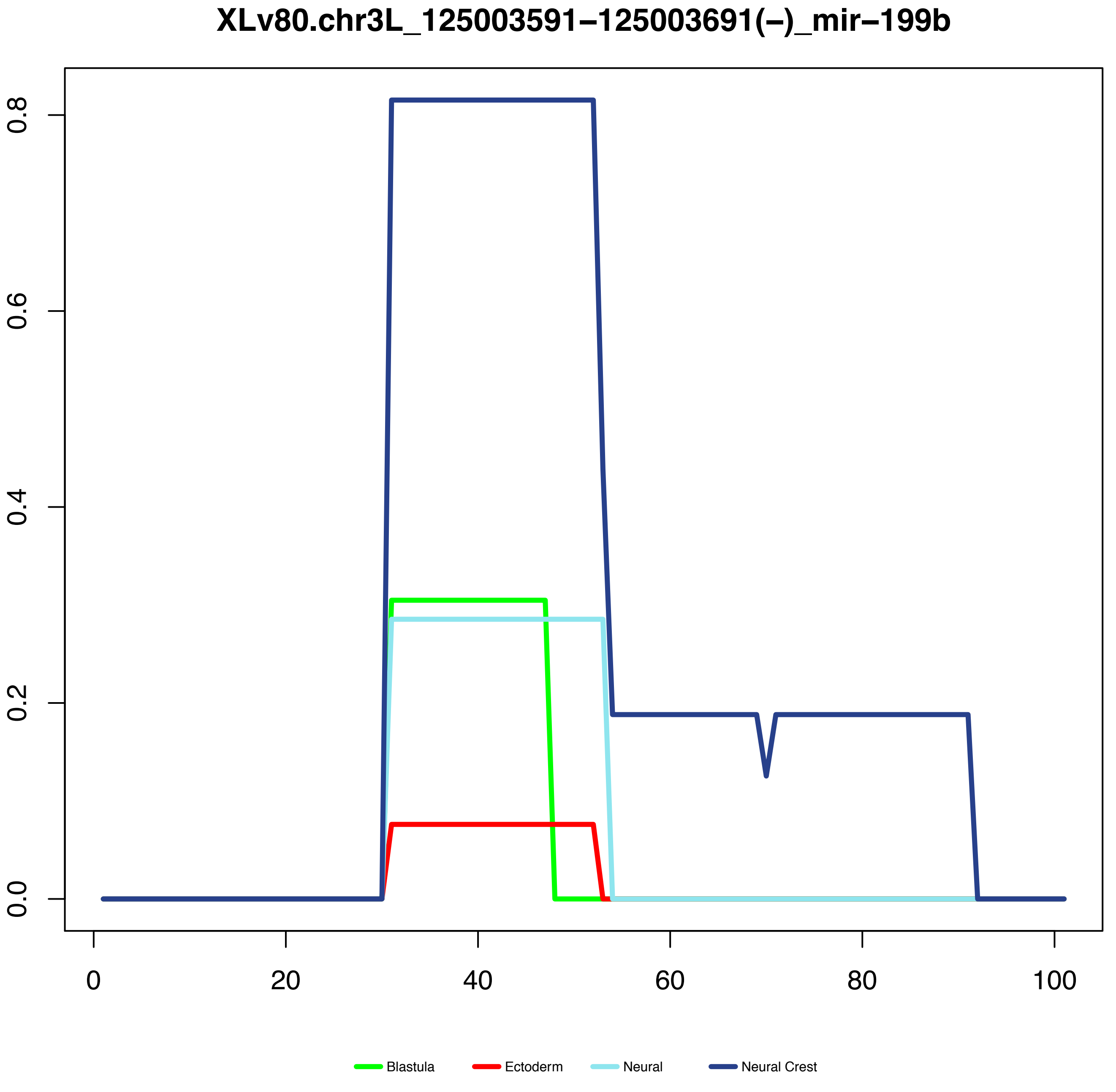

XLv80.chr3L_125003591−125003691(−)_mir−199b
0.0
0.2
0.4
0.6
0.8
0
20
40
60
80
100
Blastula
Ectoderm
Neural
Neural Crest

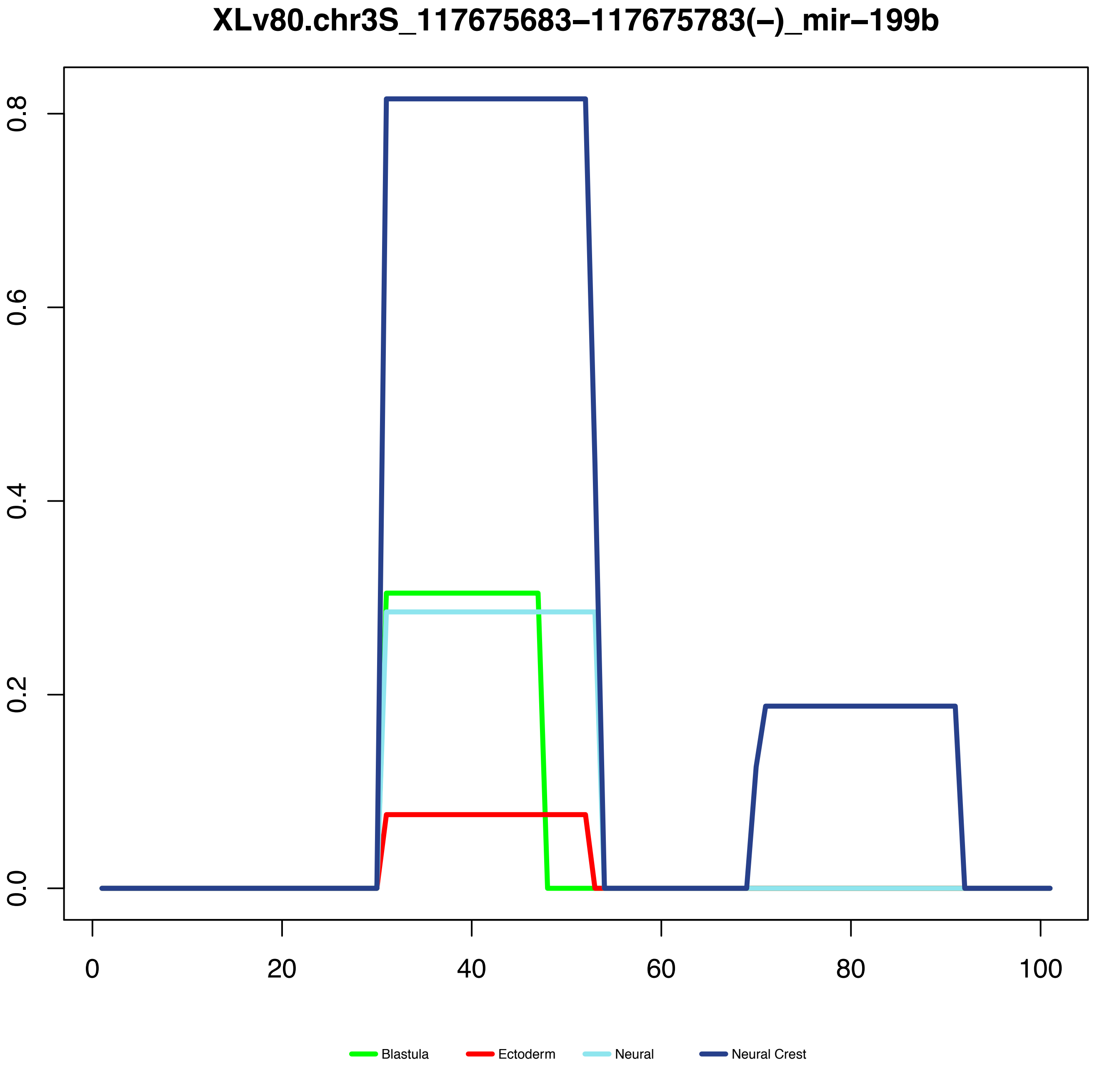

XLv80.chr3S_117675683−117675783(−)_mir−199b
0.0
0.2
0.4
0.6
0.8
0
20
40
60
80
100
Blastula
Ectoderm
Neural
Neural Crest

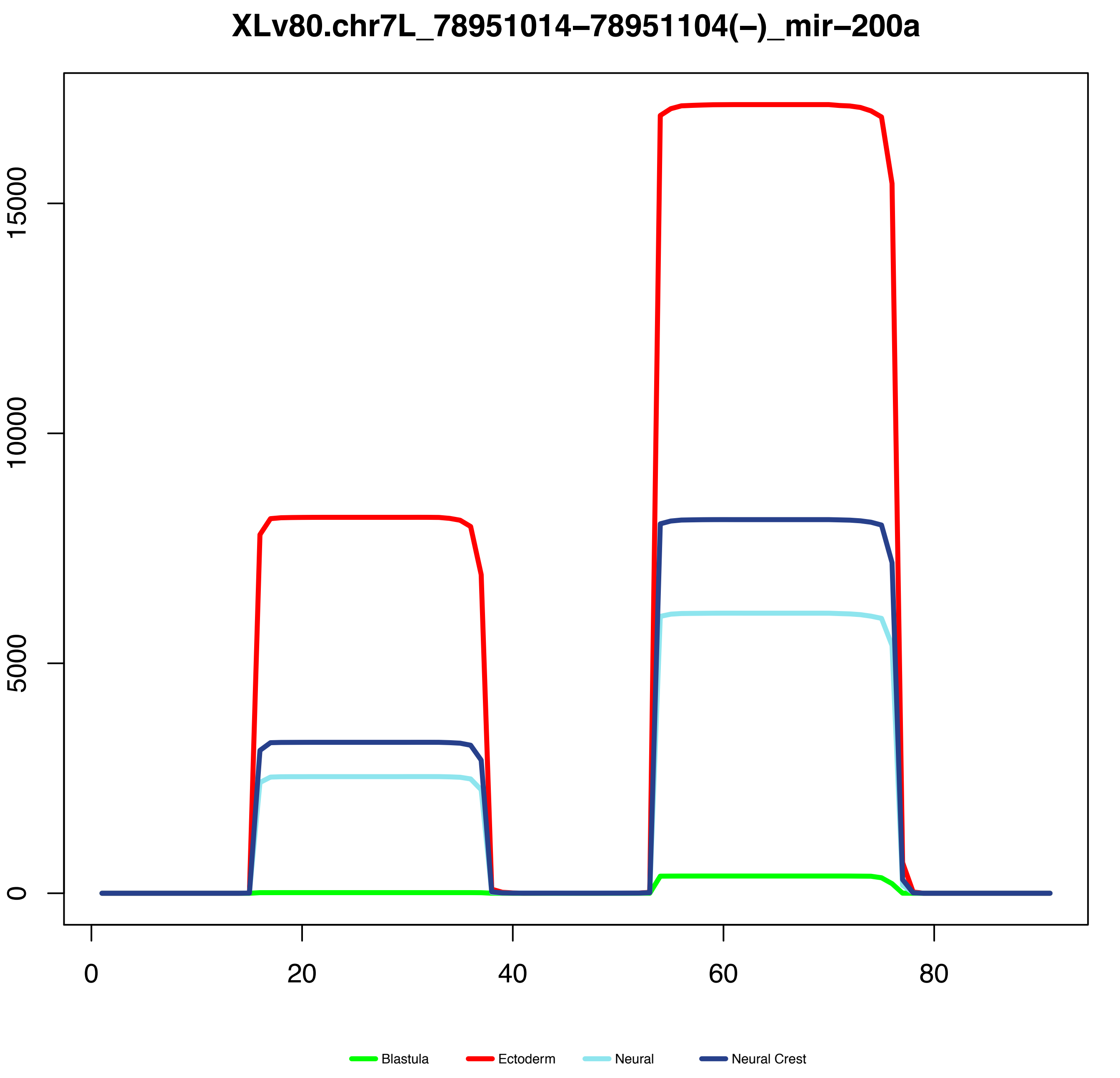
XLv80.chr7L_78951014−78951104(−)_mir−200a
0
5000
10000
15000
0
20
40
60
80
Blastula
Ectoderm
Neural
Neural Crest

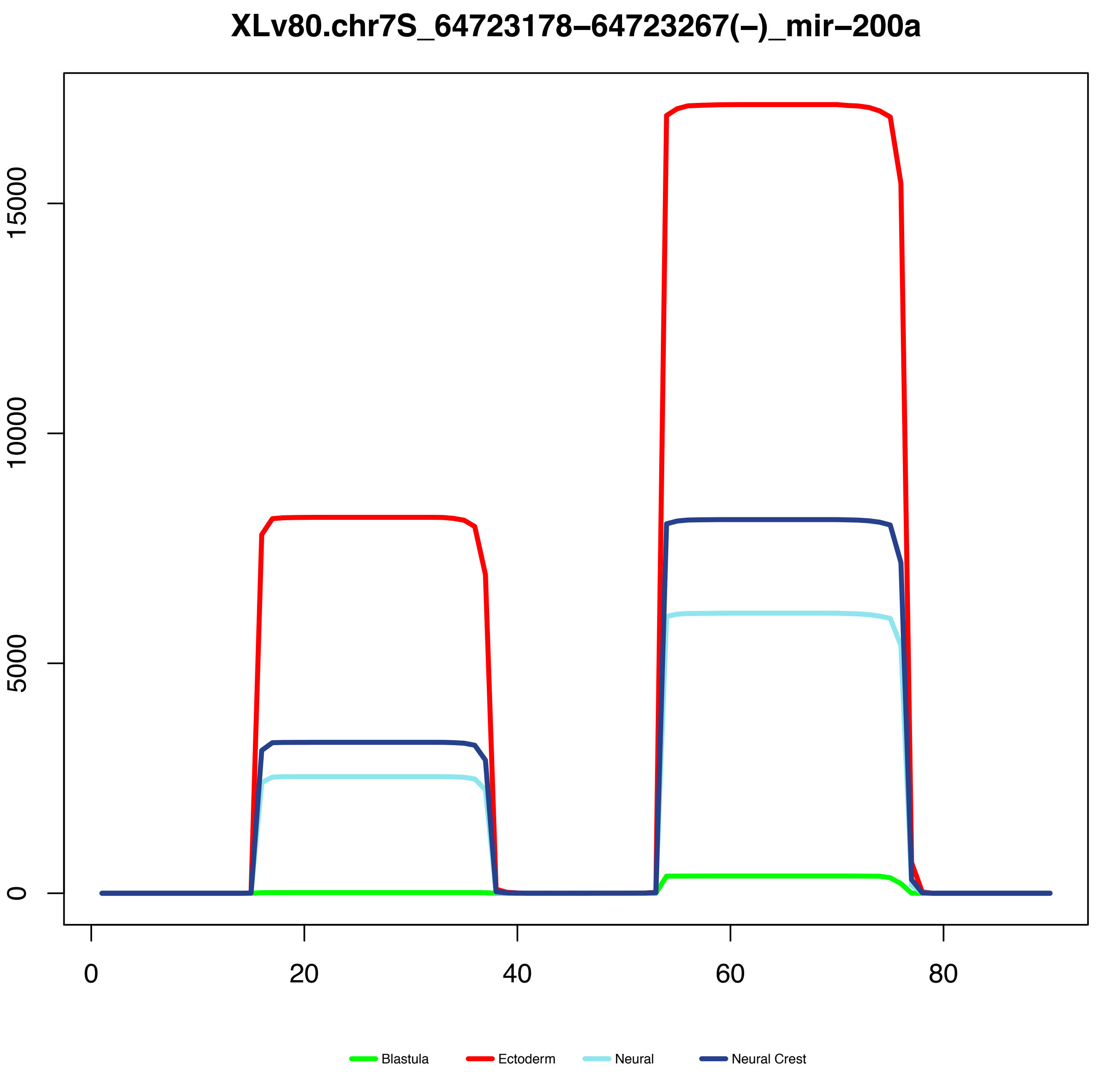
XLv80.chr7S_64723178–64723267(–)_mir–200a
0
5000
10000
15000
0
20
40
60
80
Blastula
Ectoderm
Neural
Neural Crest

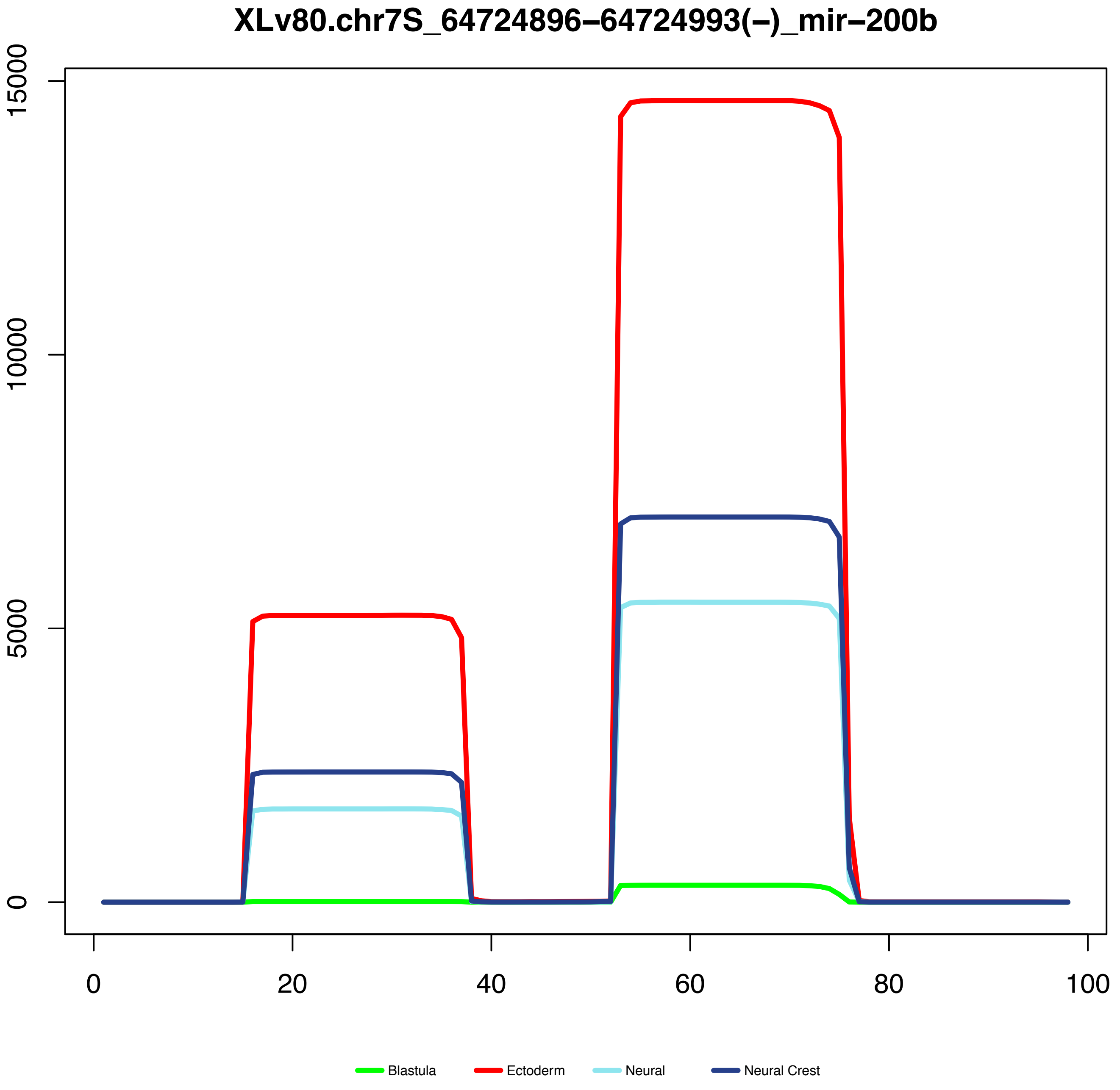
XLv80.chr7S_64724896−64724993(−)_mir−200b
0
5000
10000
15000
0
20
40
60
80
100
Blastula
Ectoderm
Neural
Neural Crest

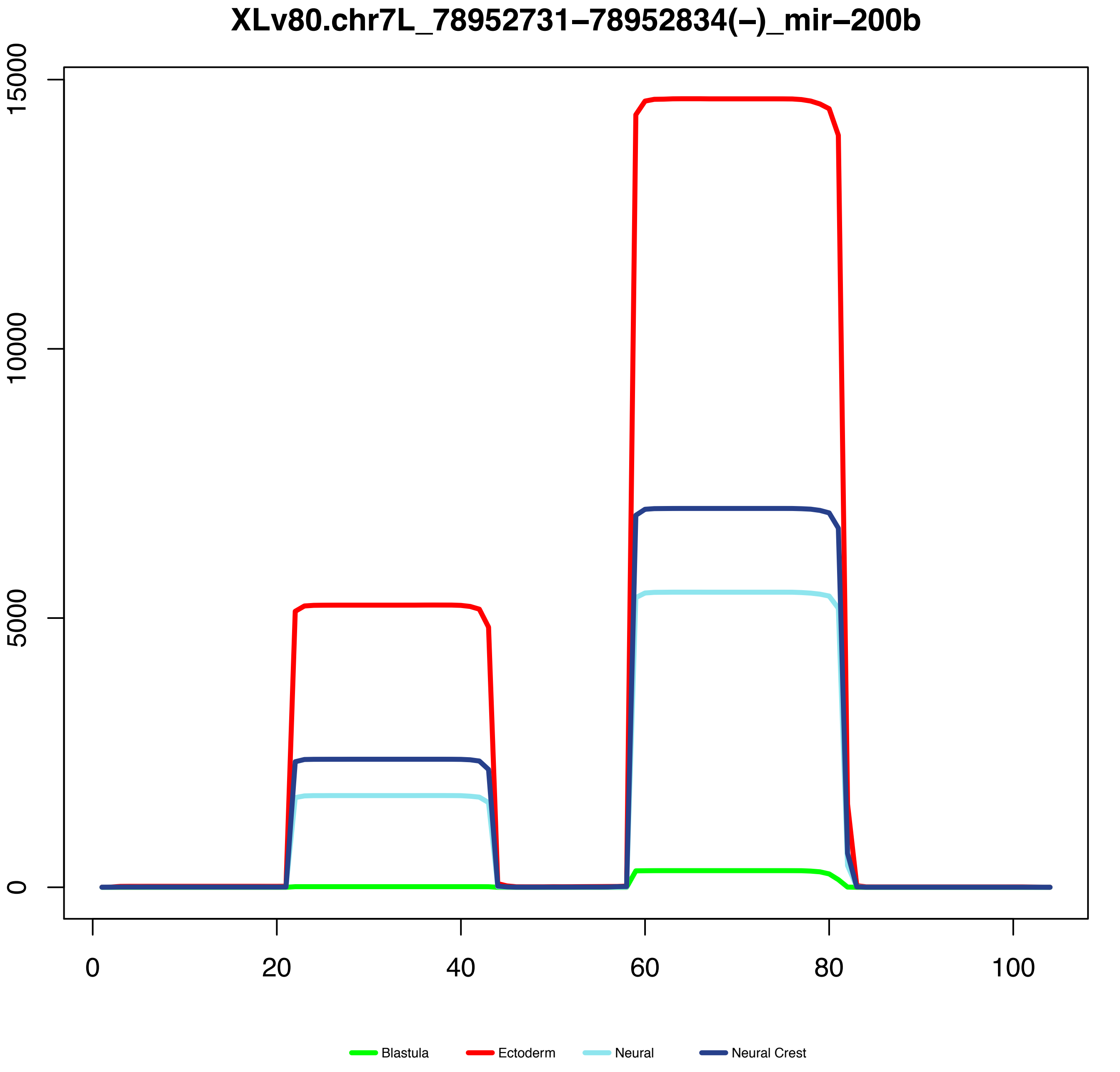
XLv80.chr7L_78952731−78952834(−)_mir−200b
15000
10000
5000
0
0
20
40
60
80
100
Blastula
Ectoderm
Neural
Neural Crest

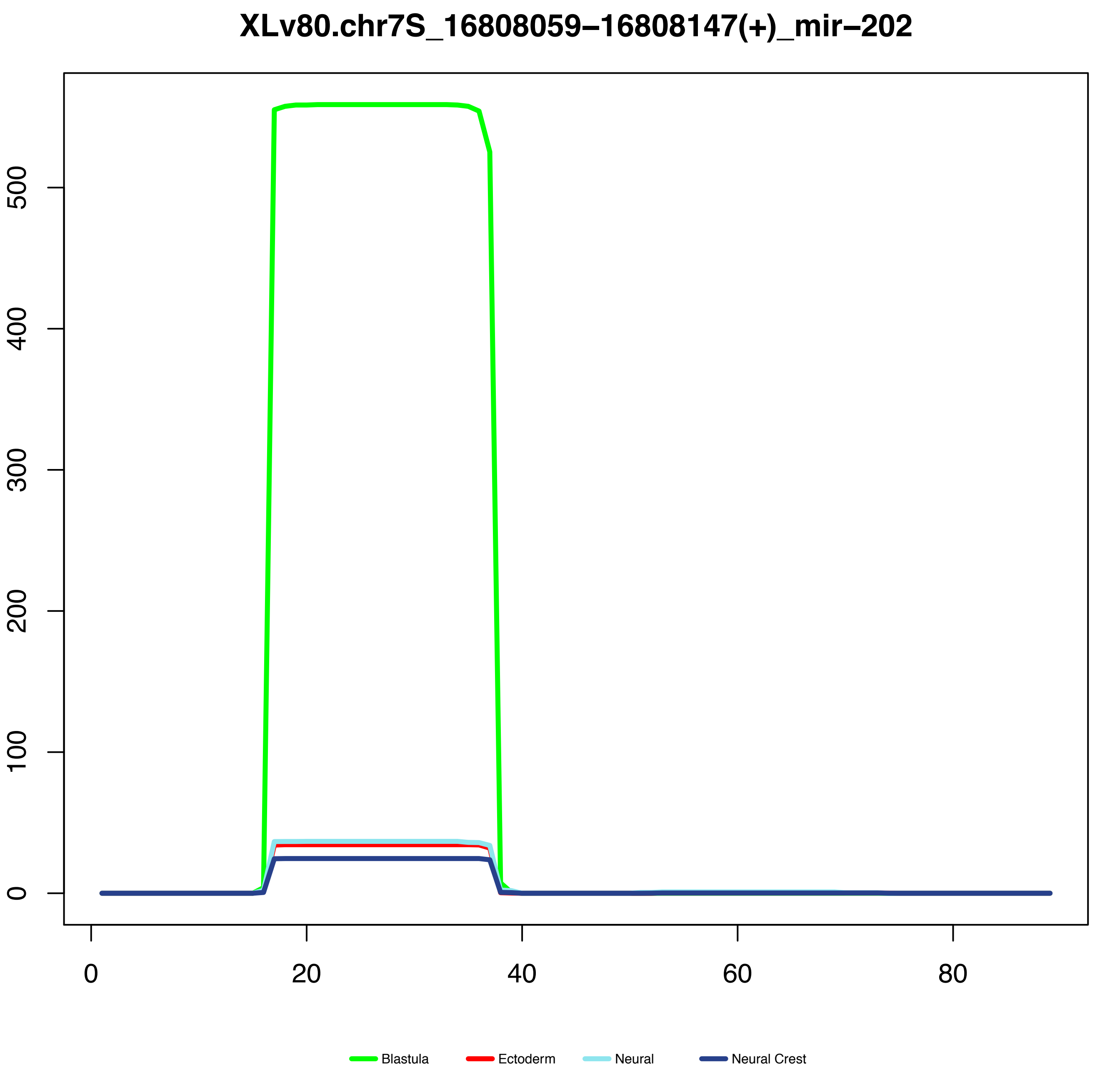
XLv80.chr7S_16808059−16808147(+)_mir−202
0
100
200
300
400
500
0
20
40
60
80
Blastula
Ectoderm
Neural
Neural Crest

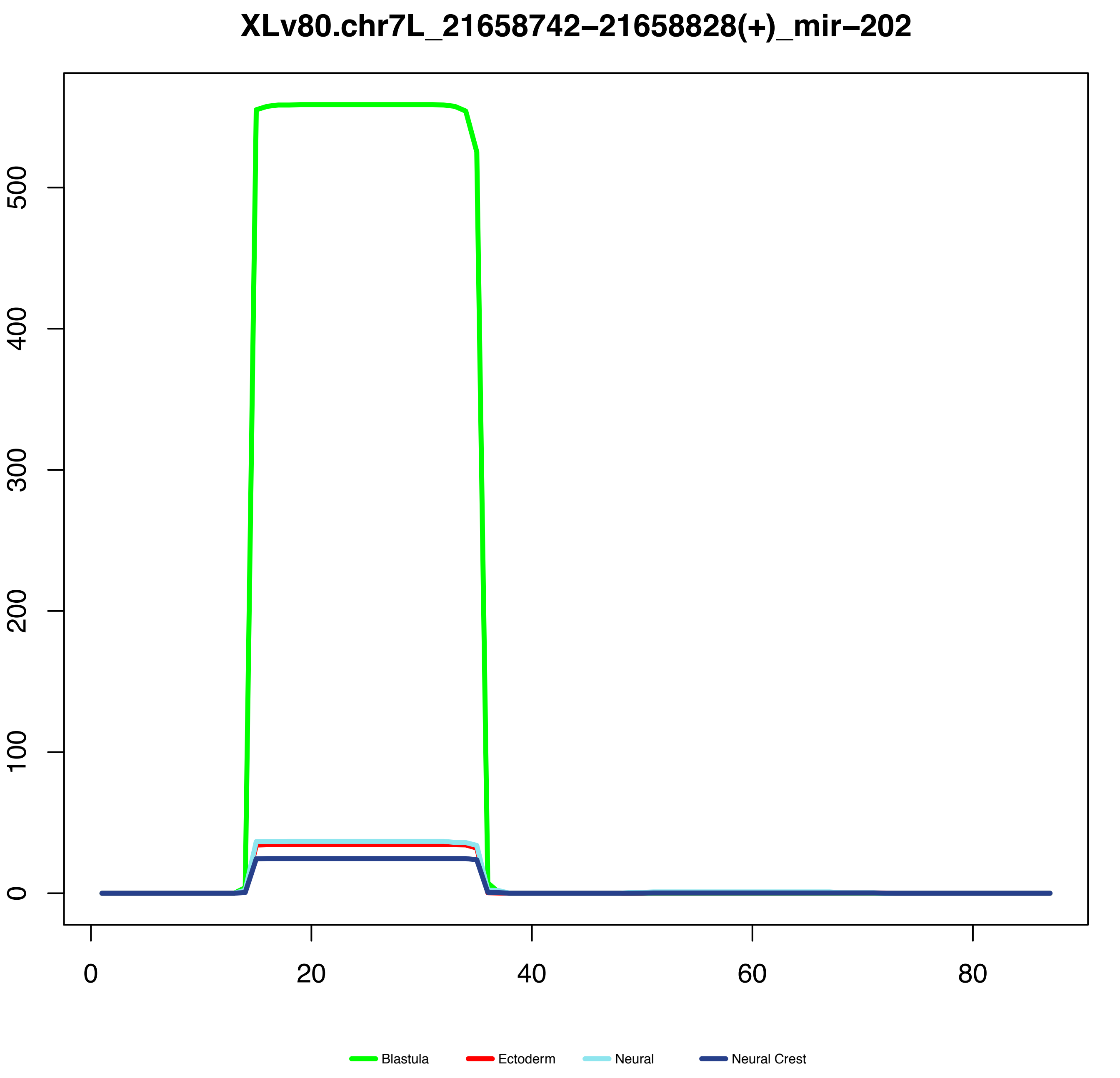
XLv80.chr7L_21658742−21658828(+)_mir−202
0
100
200
300
400
500
0
20
40
60
80
Blastula
Ectoderm
Neural
Neural Crest

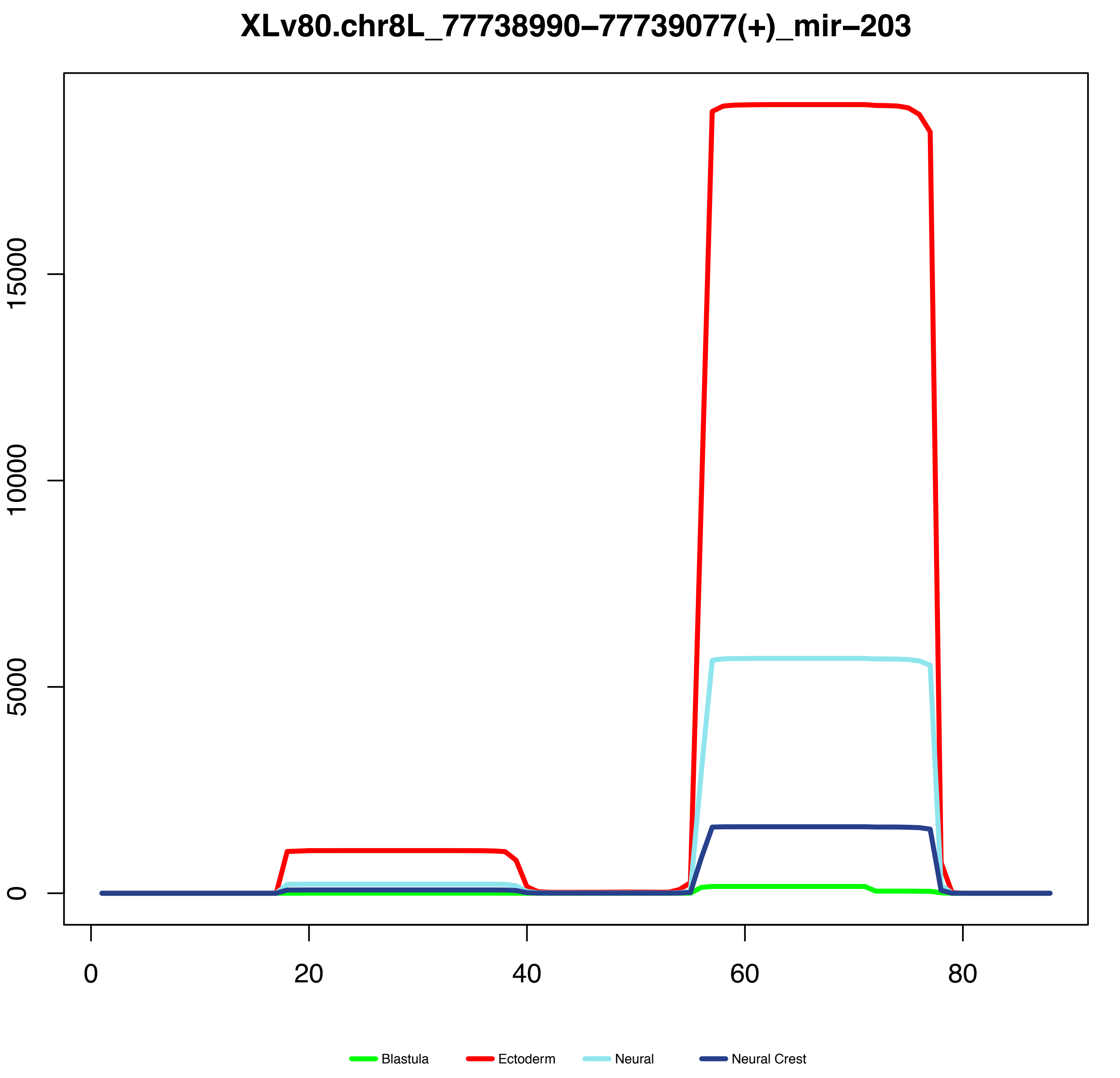
XLv80.chr8L_77738990−77739077(+)_mir−203
0
5000
10000
15000
0
20
40
60
80
Blastula
Ectoderm
Neural
Neural Crest

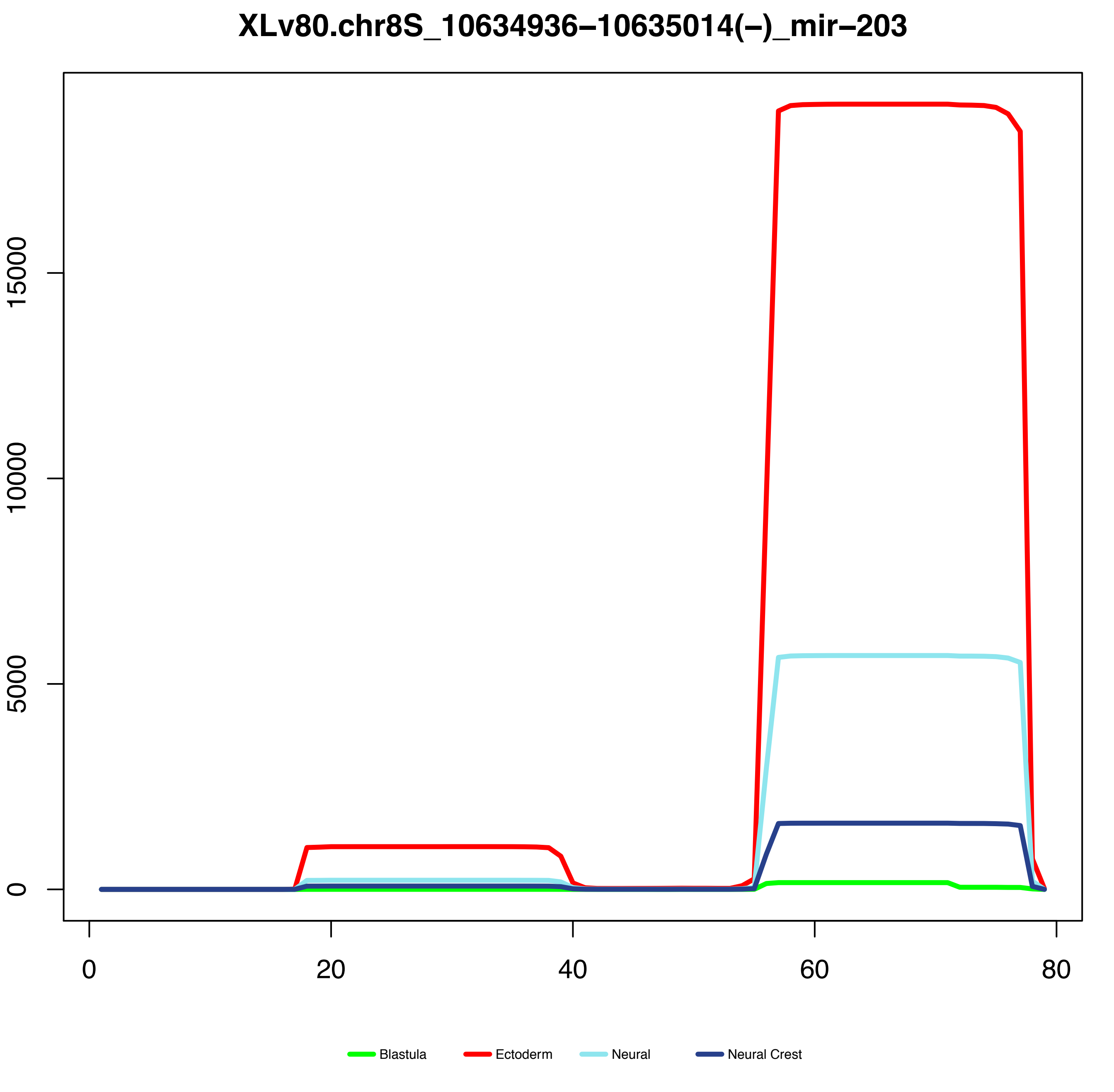
XLv80.chr8S_10634936−10635014(−)_mir−203
0
5000
10000
15000
0
20
40
60
80
Blastula
Ectoderm
Neural
Neural Crest

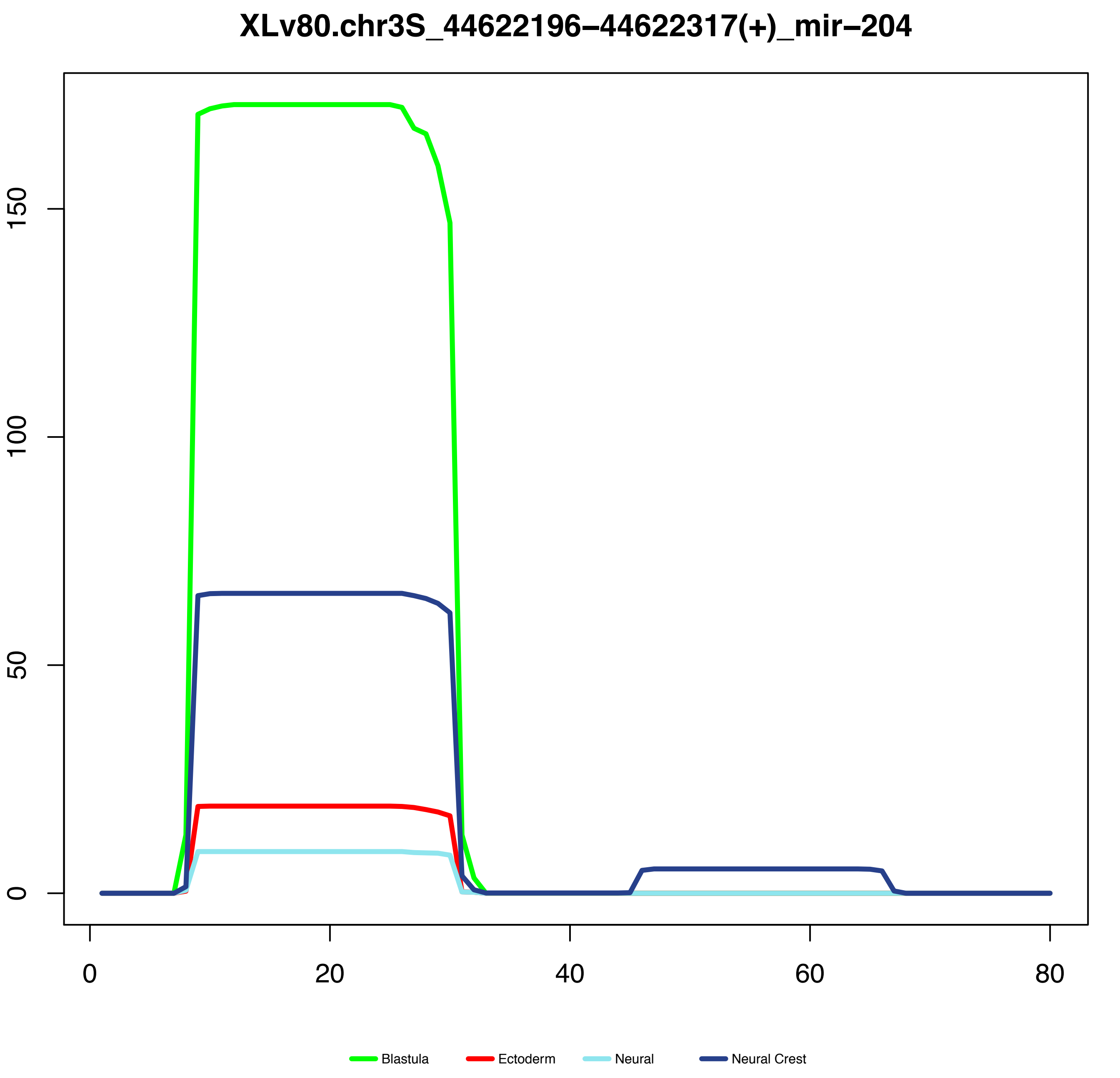
XLv80.chr3S_44622196−44622317(+)_mir−204
0
50
100
150
0
20
40
60
80
Blastula
Ectoderm
Neural
Neural Crest

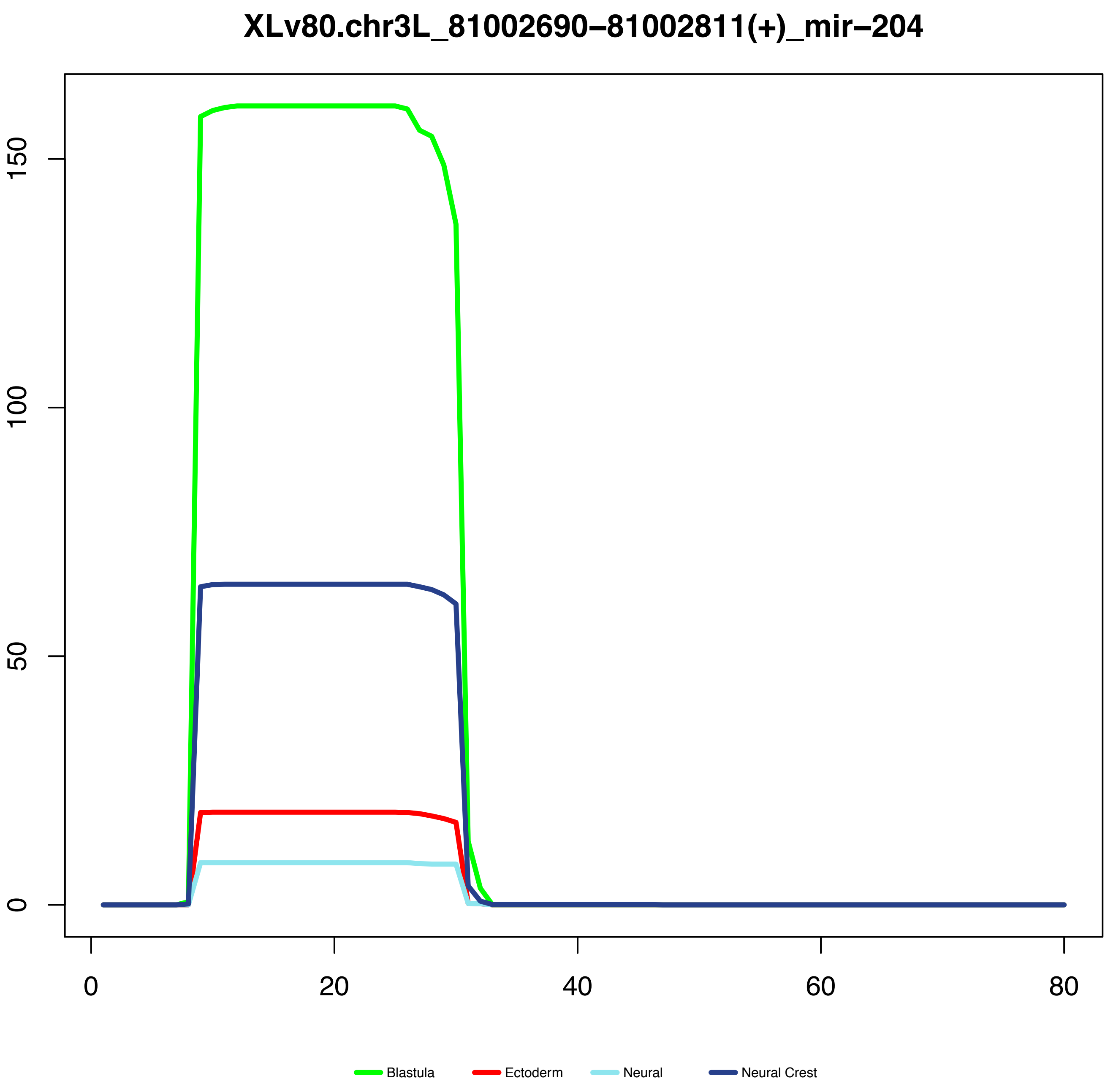
XLv80.chr3L_81002690−81002811(+)_mir−204
150
100
50
0
0
20
40
60
80
Blastula
Ectoderm
Neural
Neural Crest

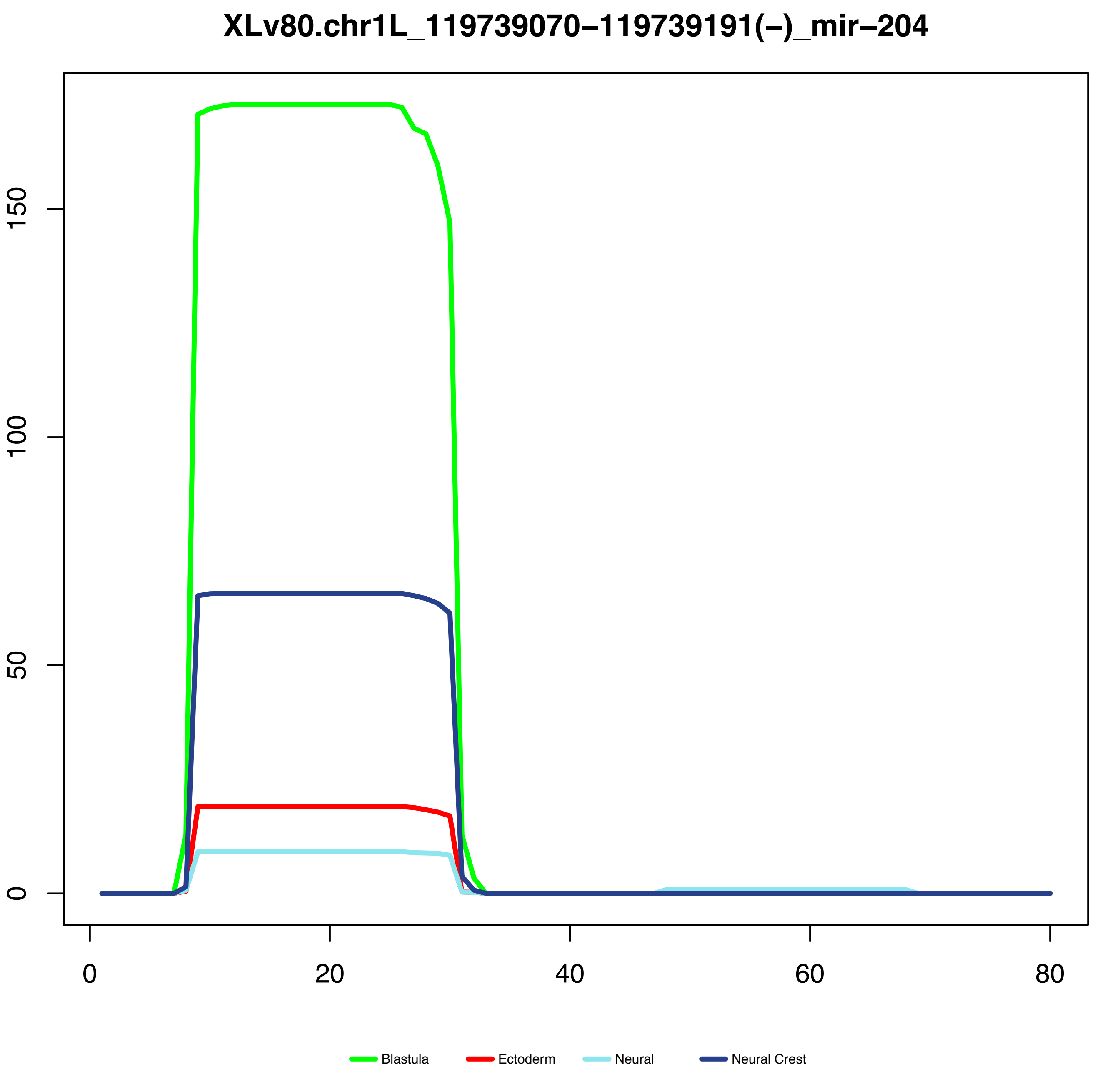

XLv80.chr1L_119739070−119739191(−)_mir−204
150
100
50
0
0
20
40
60
80
Blastula
Ectoderm
Neural
Neural Crest

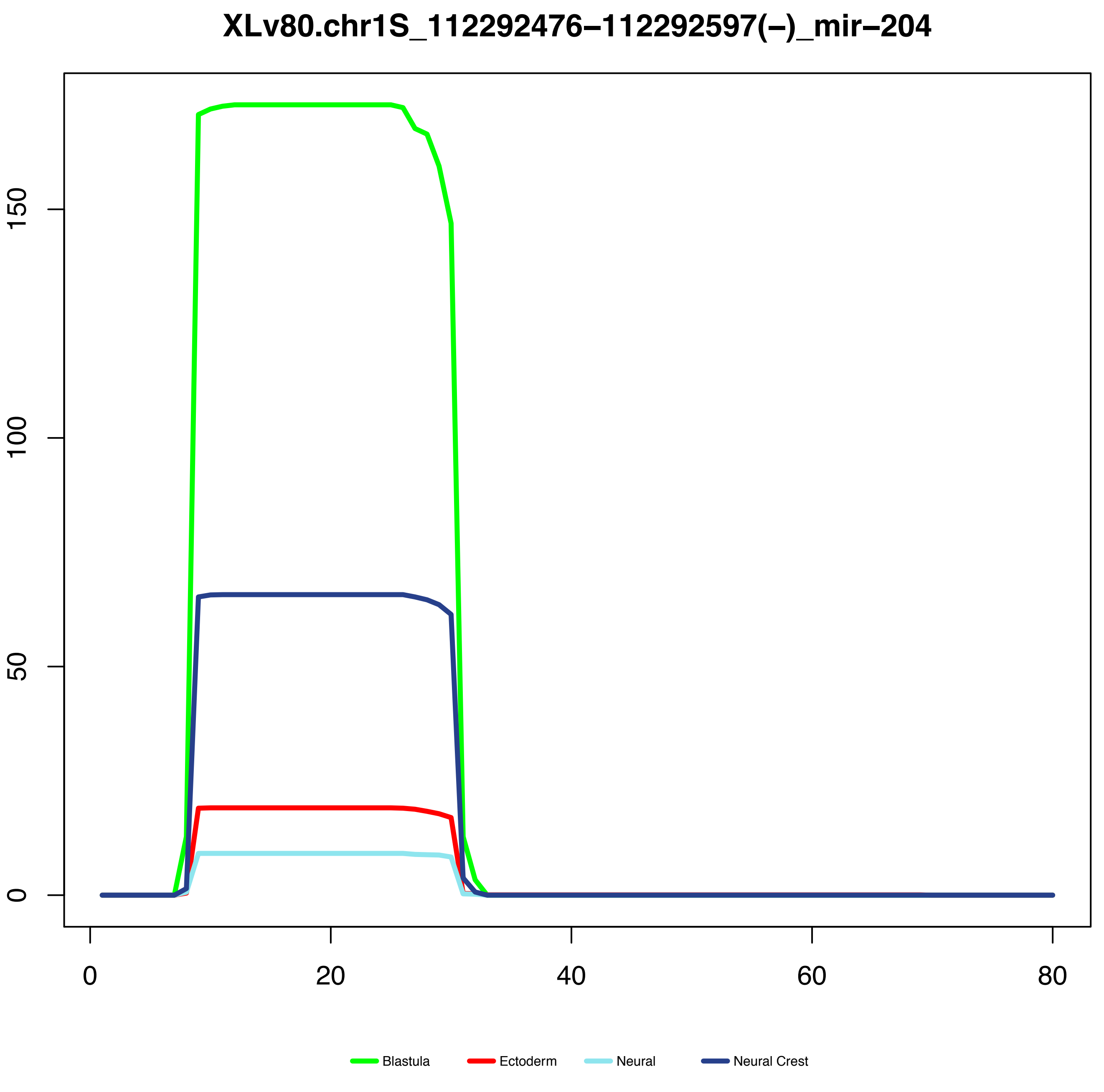

XLv80.chr1S_112292476–112292597(–)_mir–204
0
50
100
150
0
20
40
60
80
Blastula
Ectoderm
Neural
Neural Crest

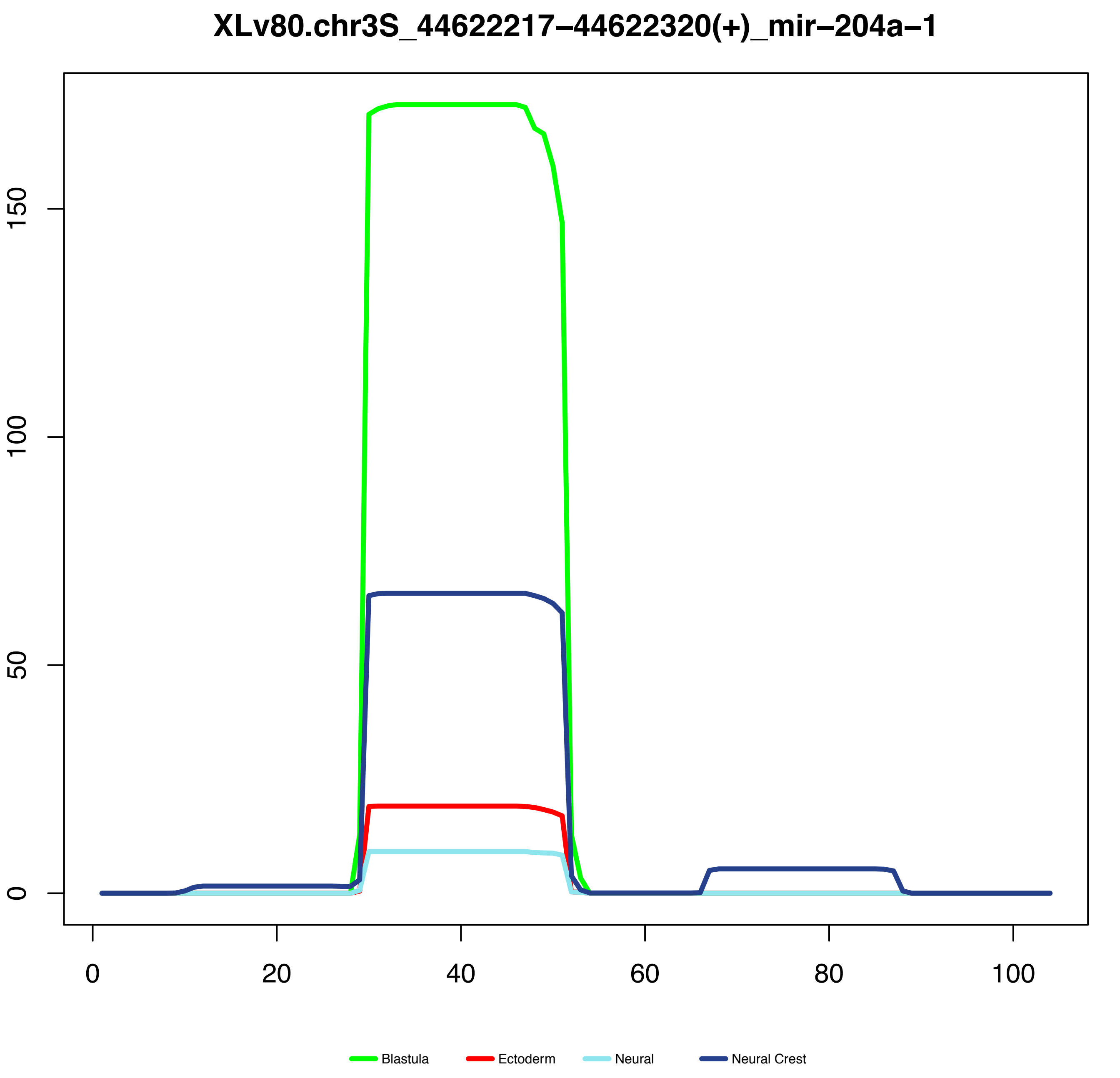

XLv80.chr3S_44622217−44622320(+)_mir−204a−1
150
100
50
0
0
20
40
60
80
100
Blastula
Ectoderm
Neural
Neural Crest

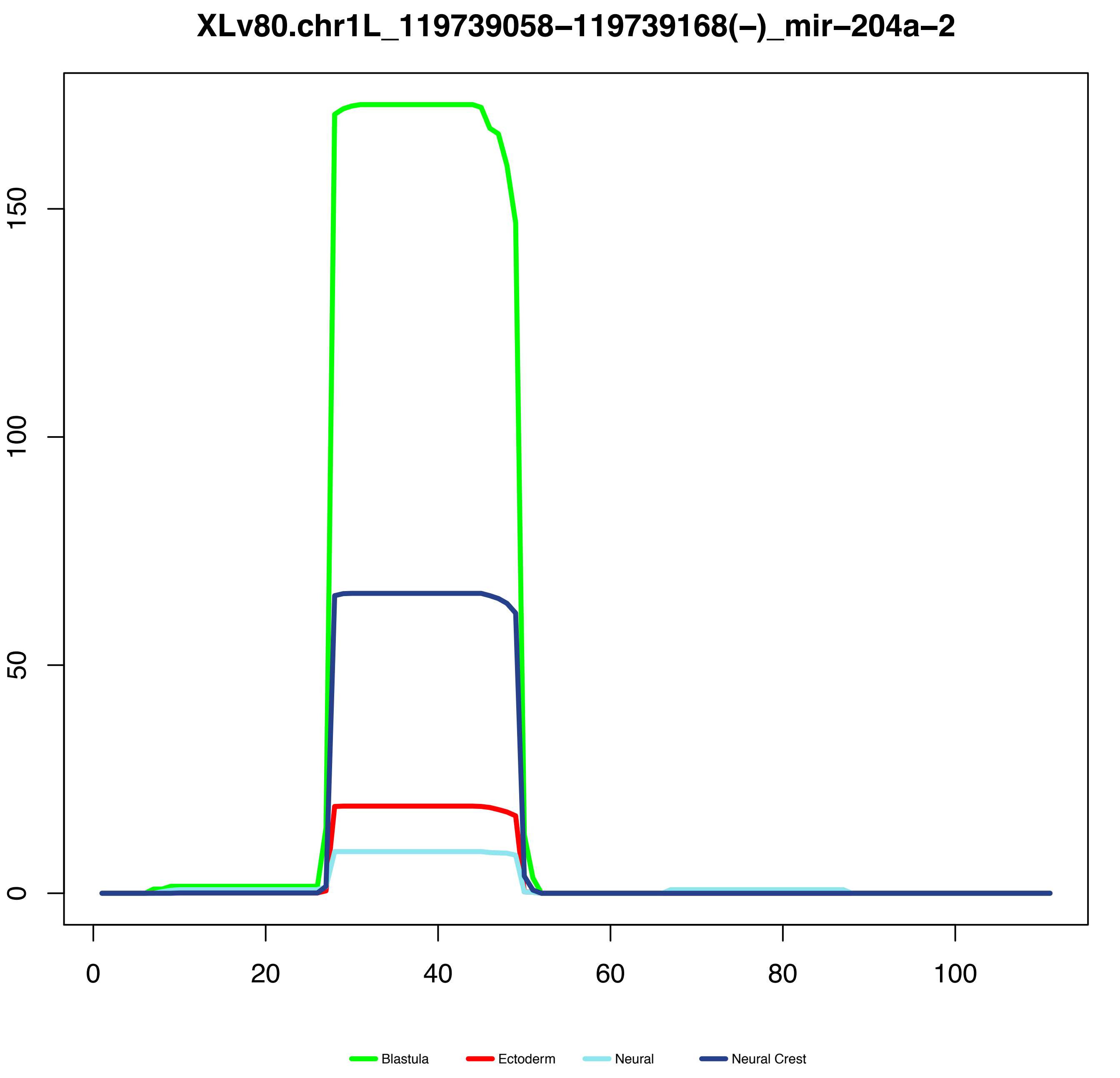
XLv80.chr1L_119739058−119739168(−)_mir−204a−2
0
50
100
150
0
20
40
60
80
100
Blastula
Ectoderm
Neural
Neural Crest

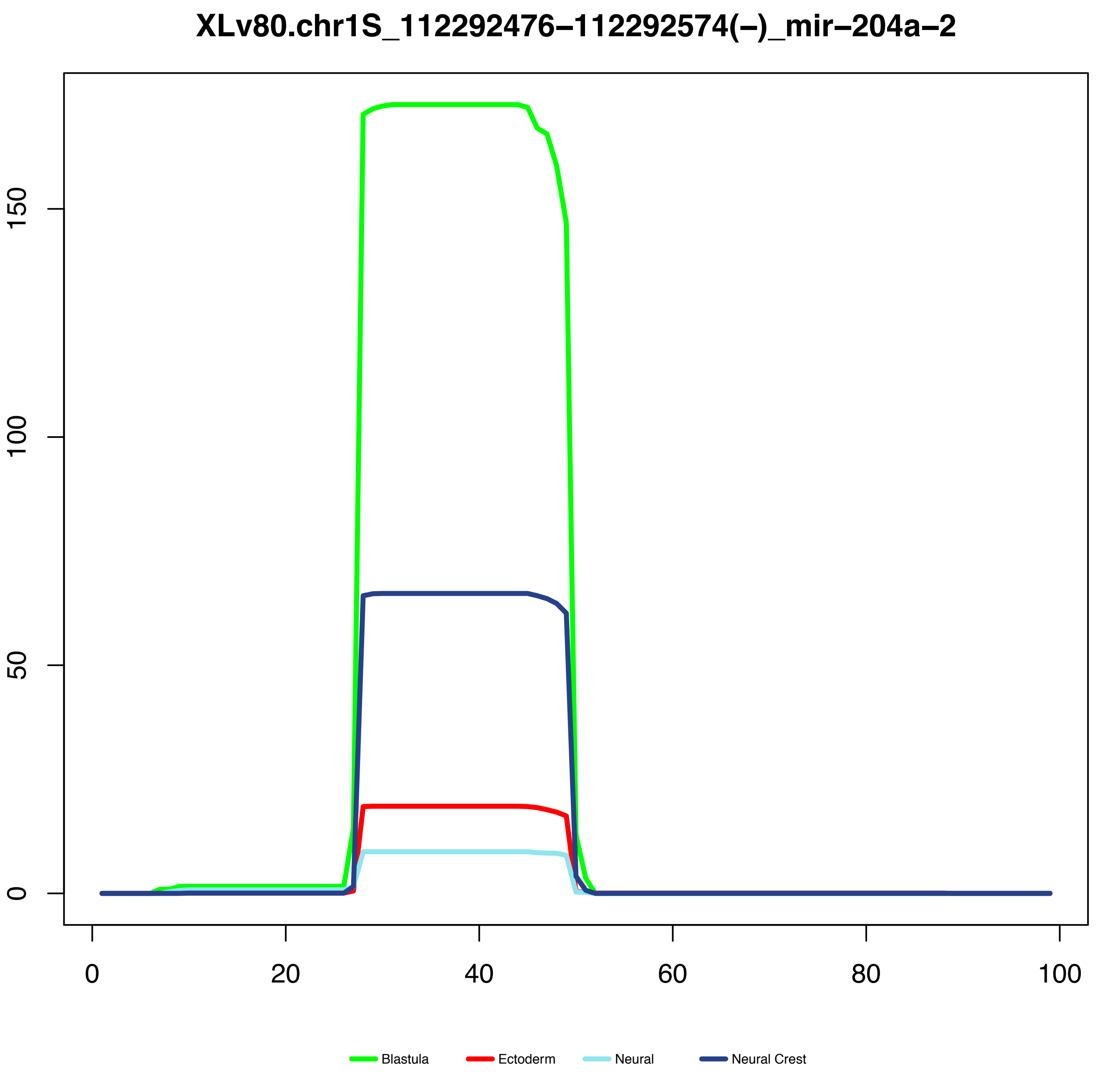
XLv80.chr1S_112292476−112292574(−)_mir−204a−2
0
50
100
150
0
20
40
60
80
100
Blastula
Ectoderm
Neural
Neural Crest

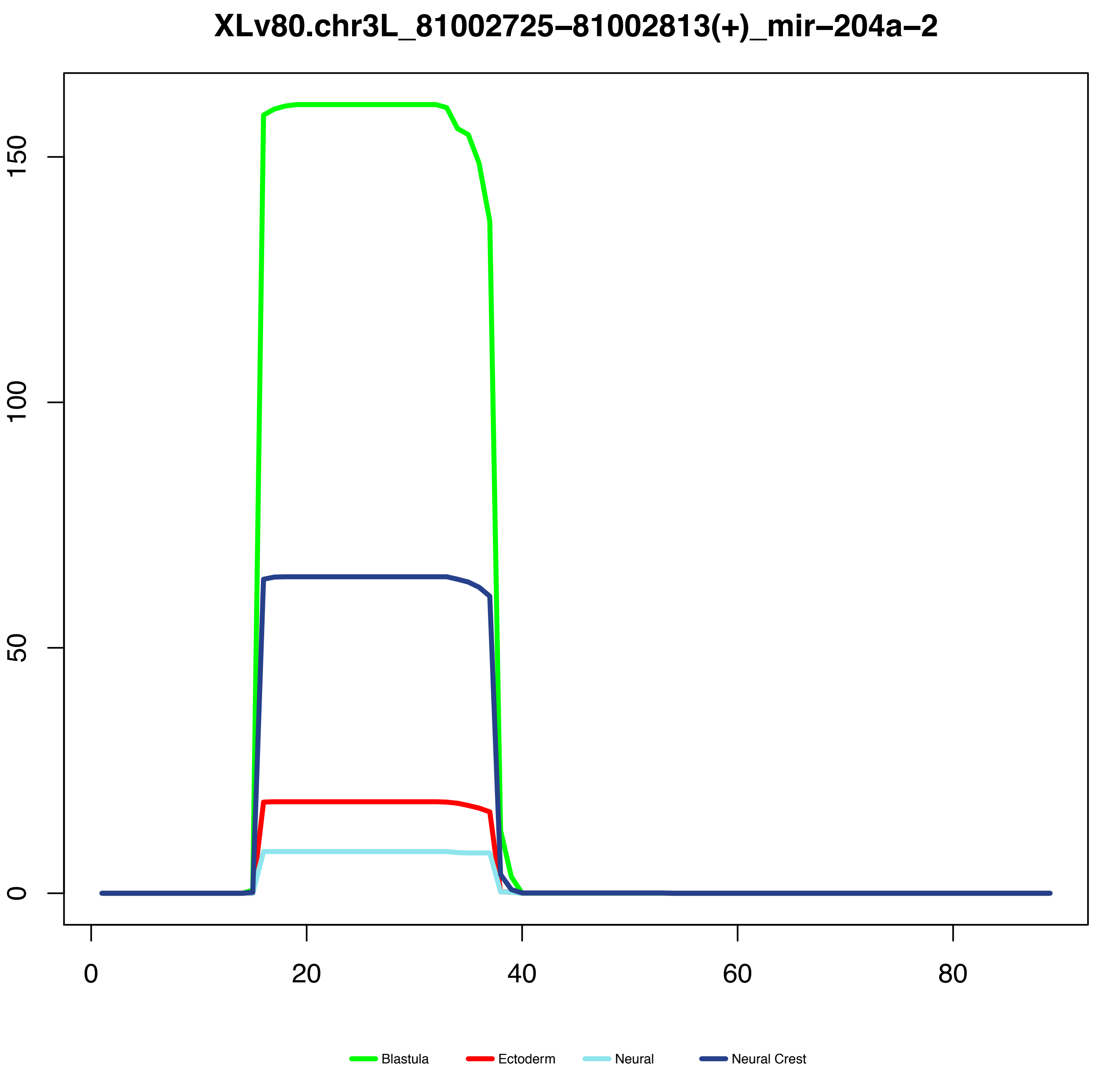

XLv80.chr3L_81002725−81002813(+)_mir−204a−2
0
50
100
150
0
20
40
60
80
Blastula
Ectoderm
Neural
Neural Crest

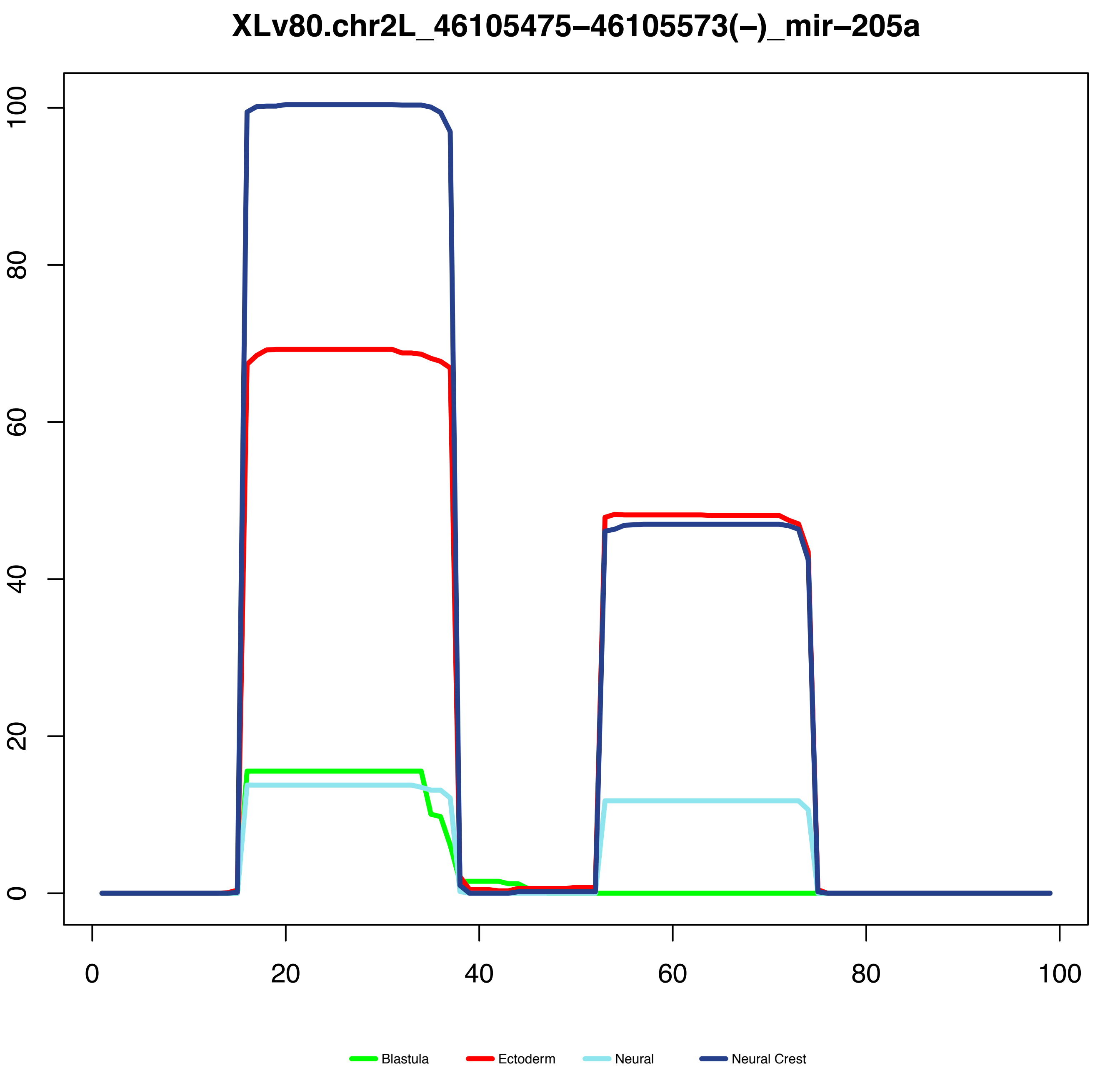
XLv80.chr2L_46105475−46105573(−)_mir−205a
0
20
40
60
80
100
0
20
40
60
80
100
Blastula
Ectoderm
Neural
Neural Crest

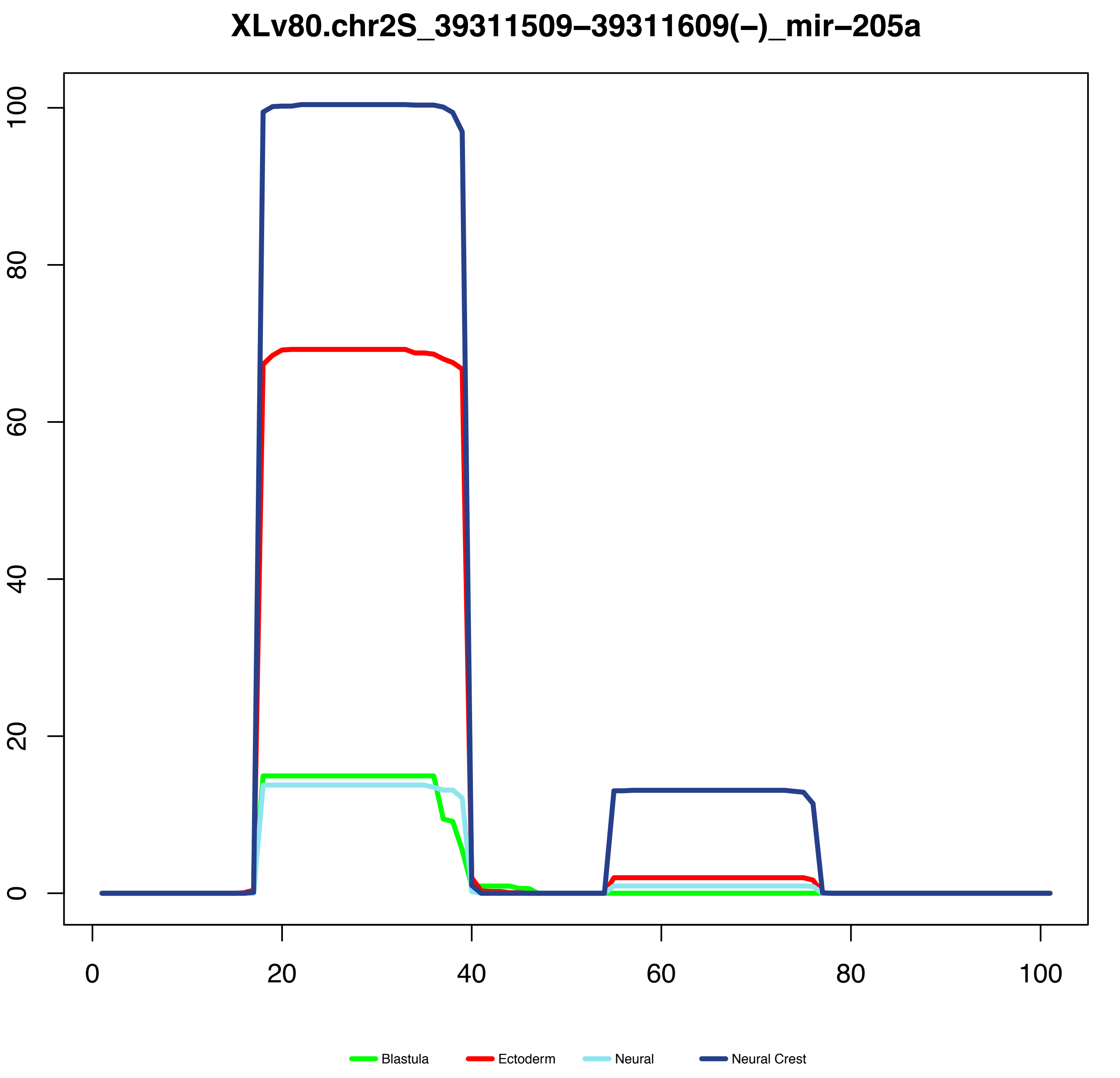
XLv80.chr2S_39311509−39311609(−)_mir−205a
0
20
40
60
80
100
0
20
40
60
80
100
Blastula
Ectoderm
Neural
Neural Crest

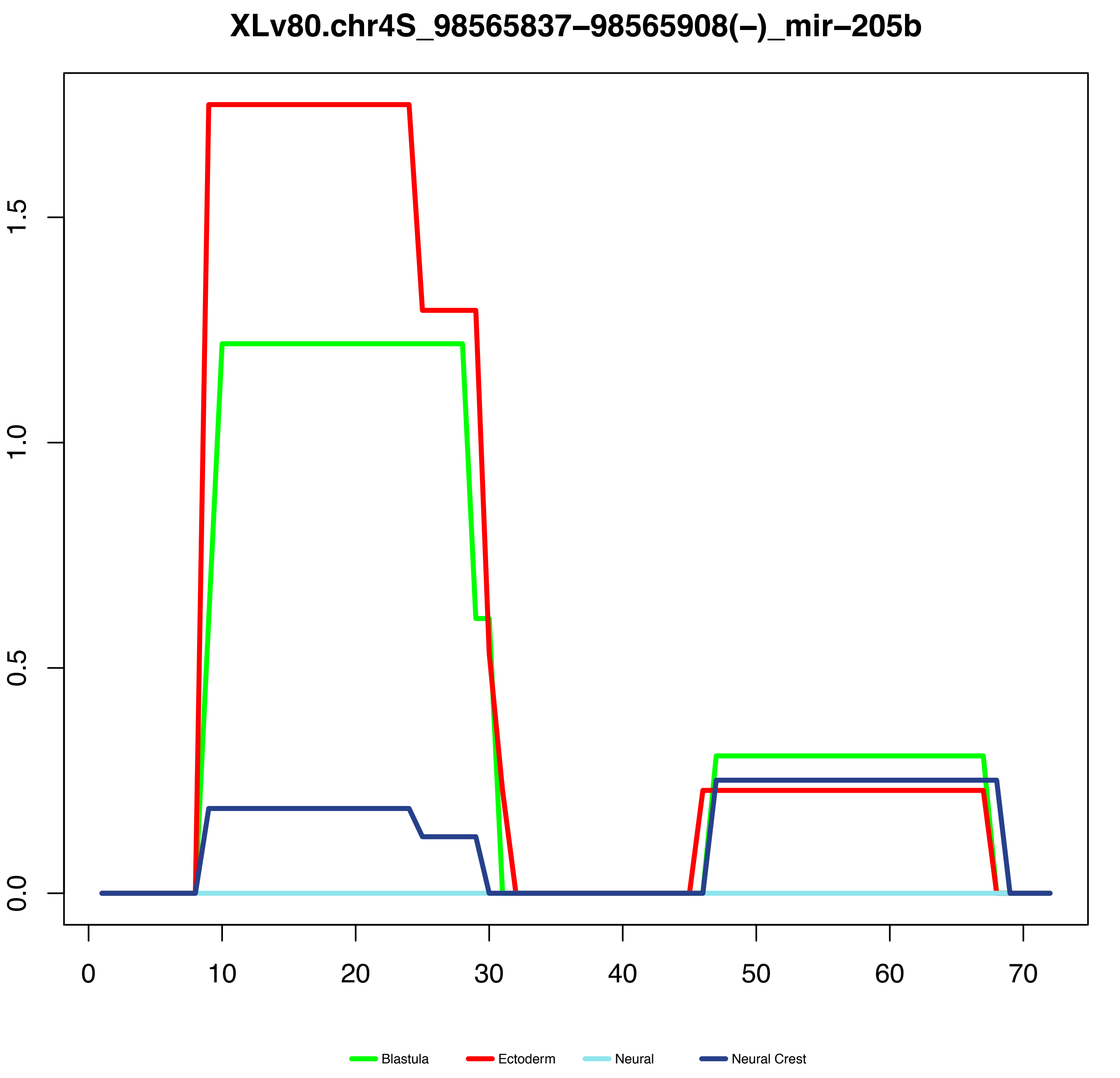
XLv80.chr4S_98565837−98565908(−)_mir−205b
0.0
0.5
1.0
1.5
0
10
20
30
40
50
60
70
Blastula
Ectoderm
Neural
Neural Crest

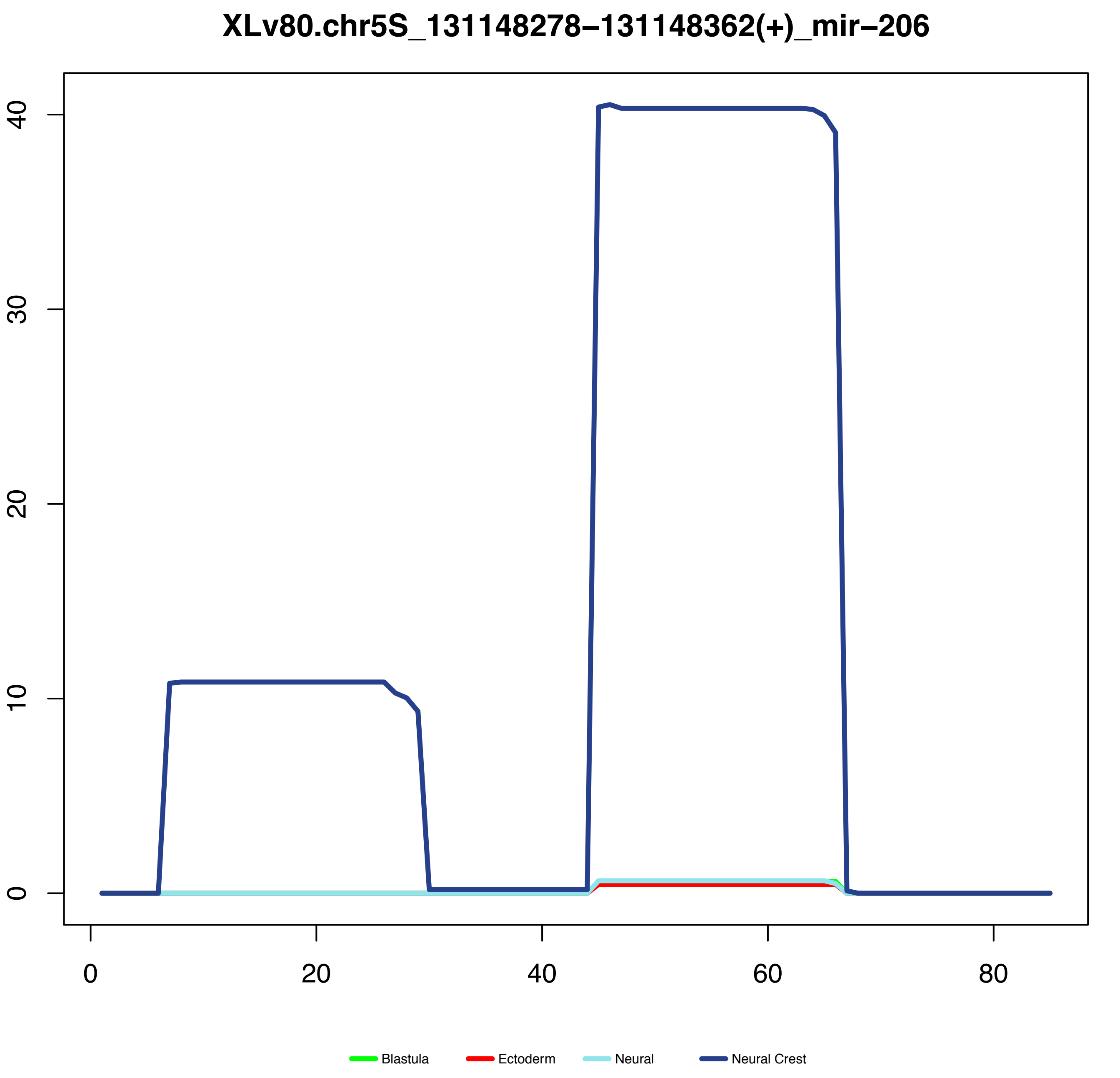
XLv80.chr5S_131148278−131148362(+)_mir−206
0
10
20
30
40
0
20
40
60
80
Blastula
Ectoderm
Neural
Neural Crest

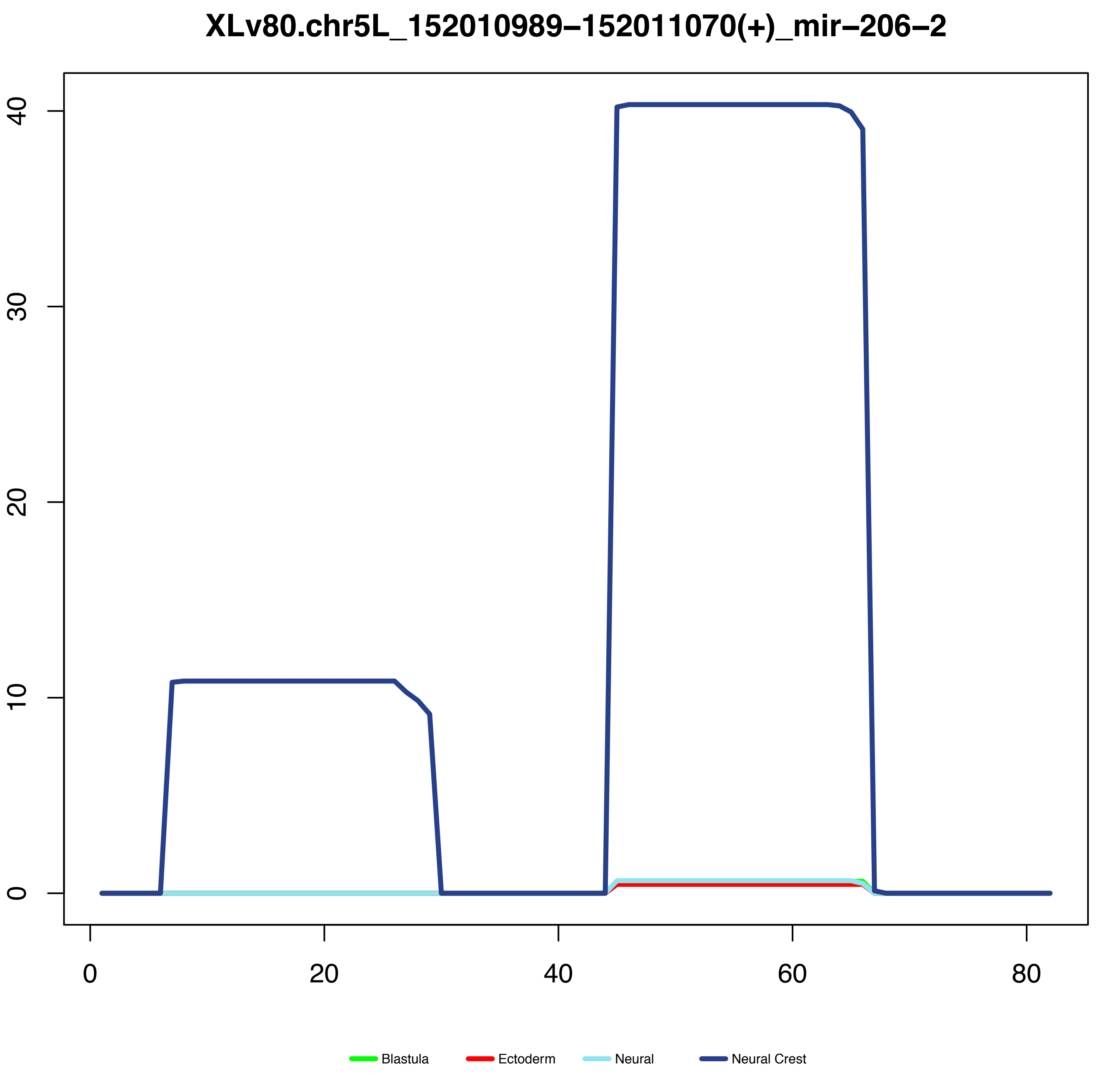
XLv80.chr5L_152010989−152011070(+)_mir−206−2
0
10
20
30
40
0
20
40
60
80
Blastula
Ectoderm
Neural
Neural Crest

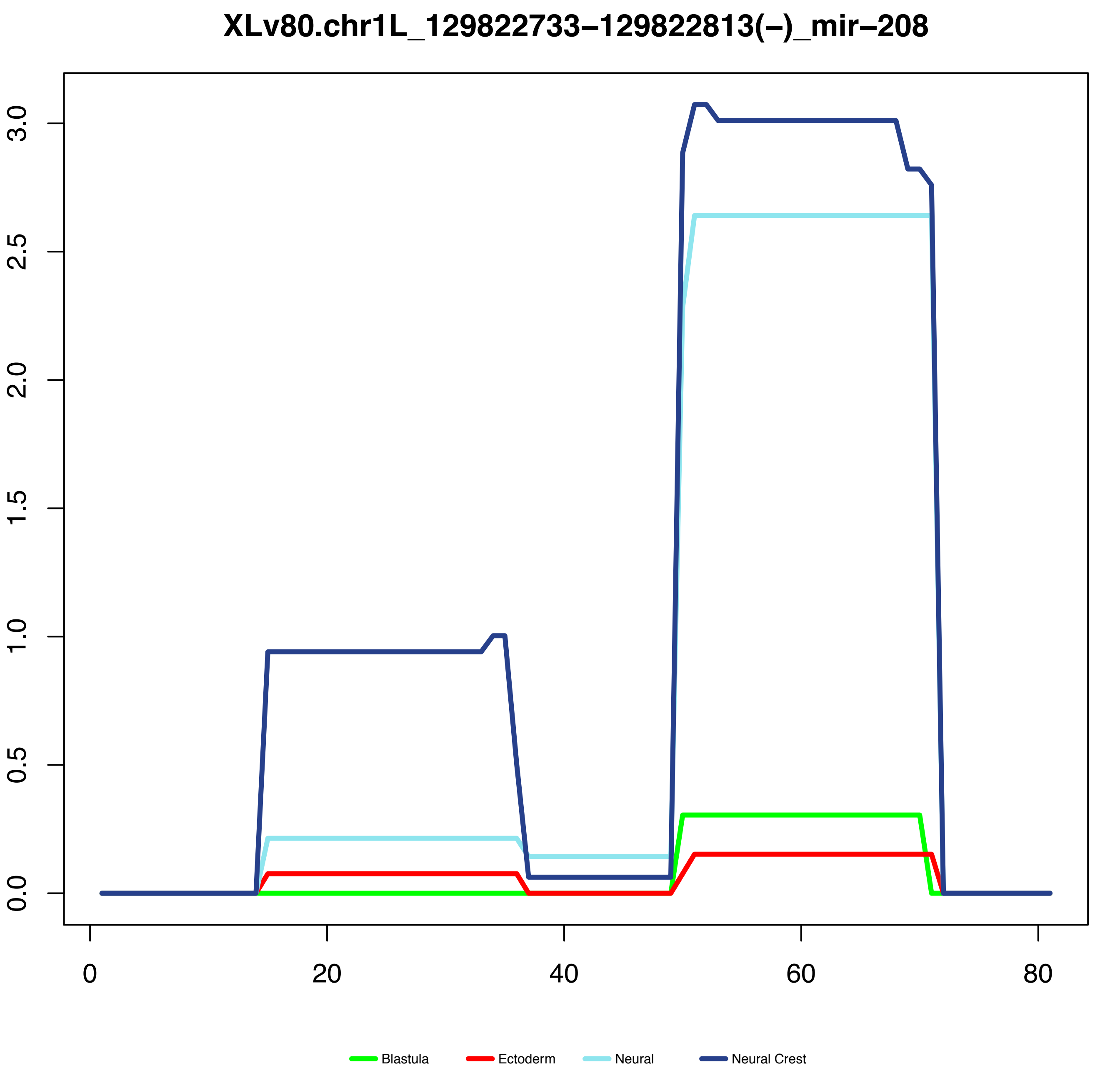

XLv80.chr1L_129822733−129822813(−)_mir−208
0.0
0.5
1.0
1.5
2.0
2.5
3.0
0
20
40
60
80
Blastula
Ectoderm
Neural
Neural Crest

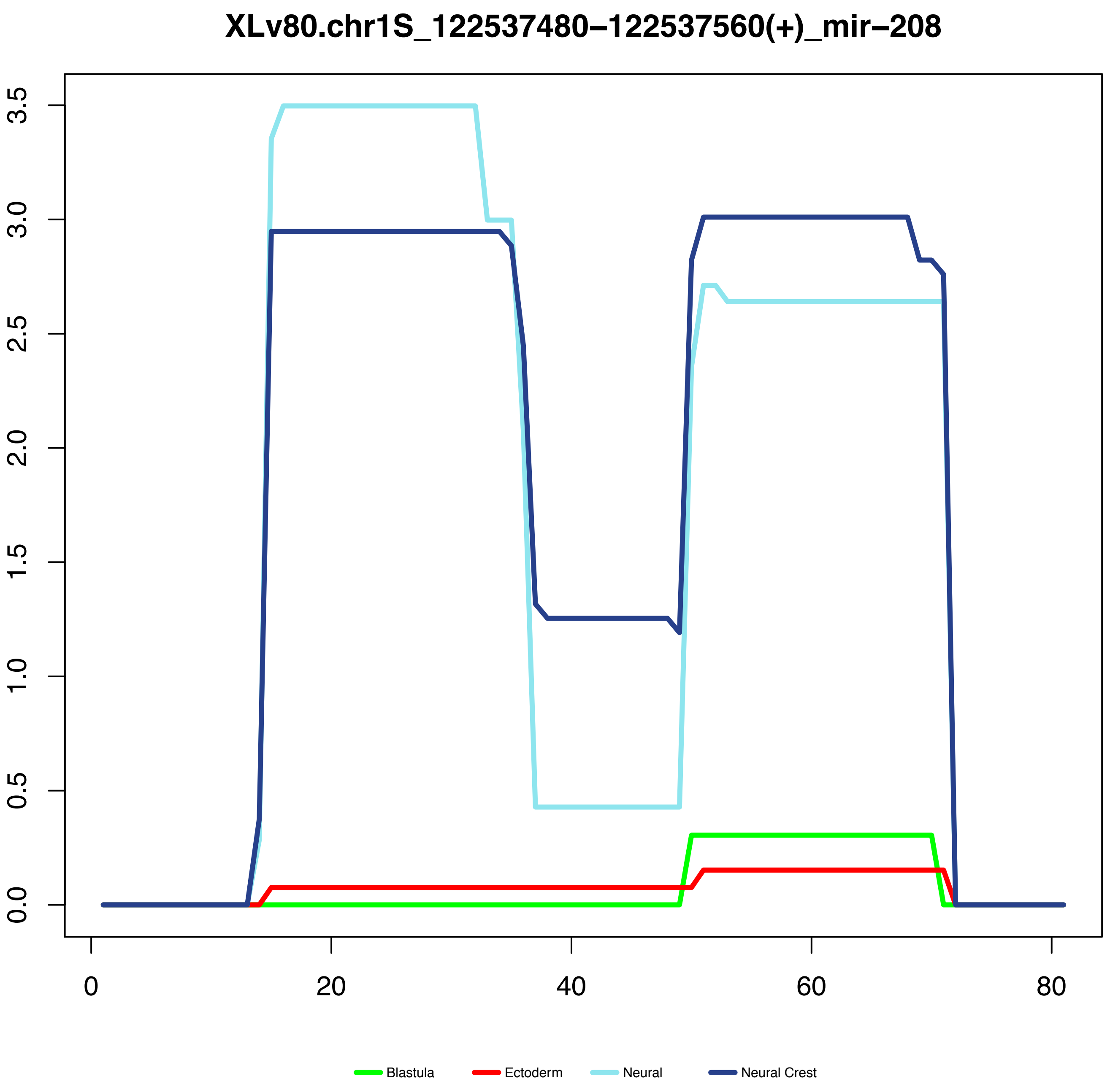
XLv80.chr1S_122537480−122537560(+)_mir−208
0.0
0.5
1.0
1.5
2.0
2.5
3.0
3.5
0
20
40
60
80
Blastula
Ectoderm
Neural
Neural Crest

XLv80.chr4L_22477257−22477347(−)_mir−210

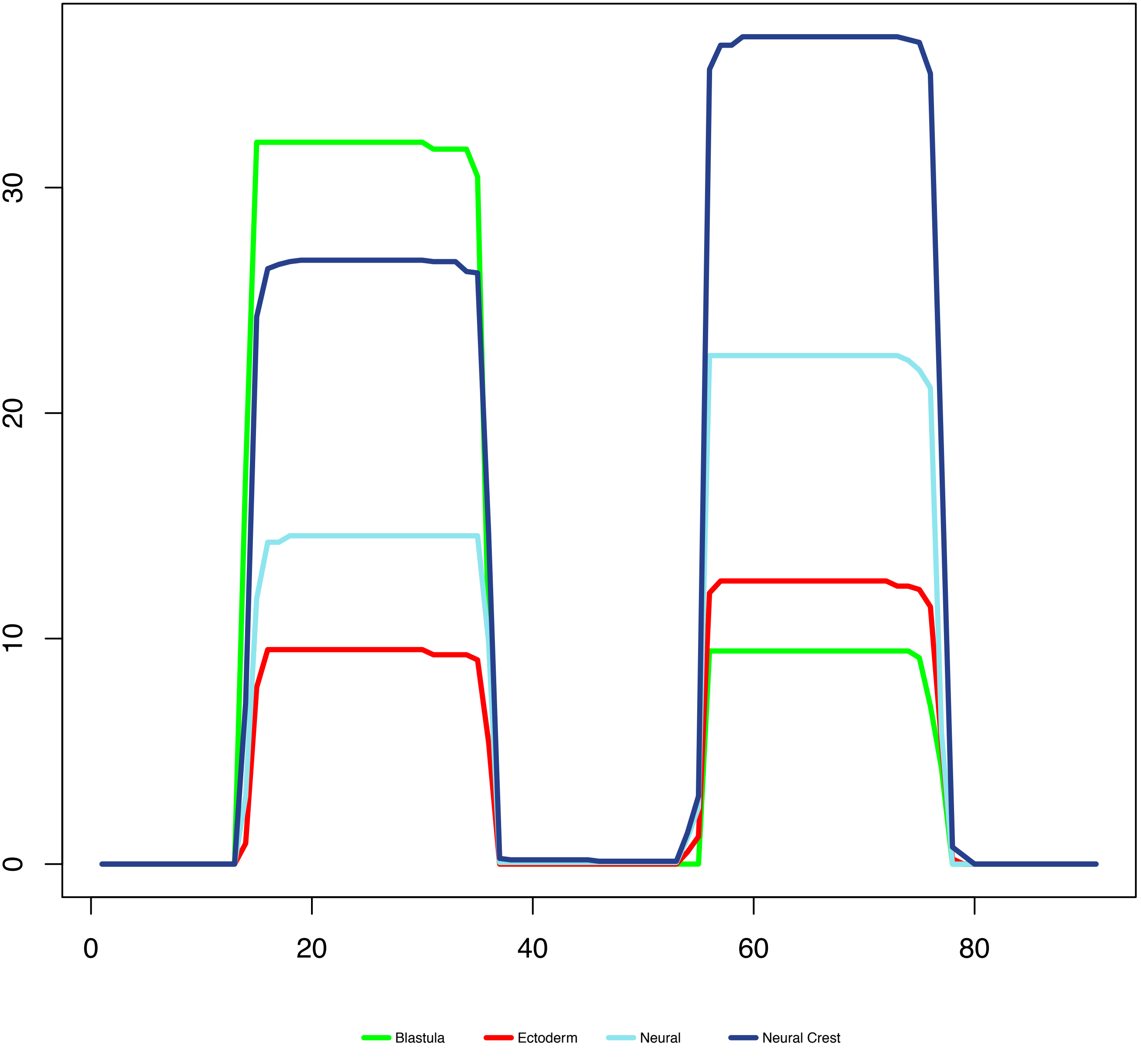

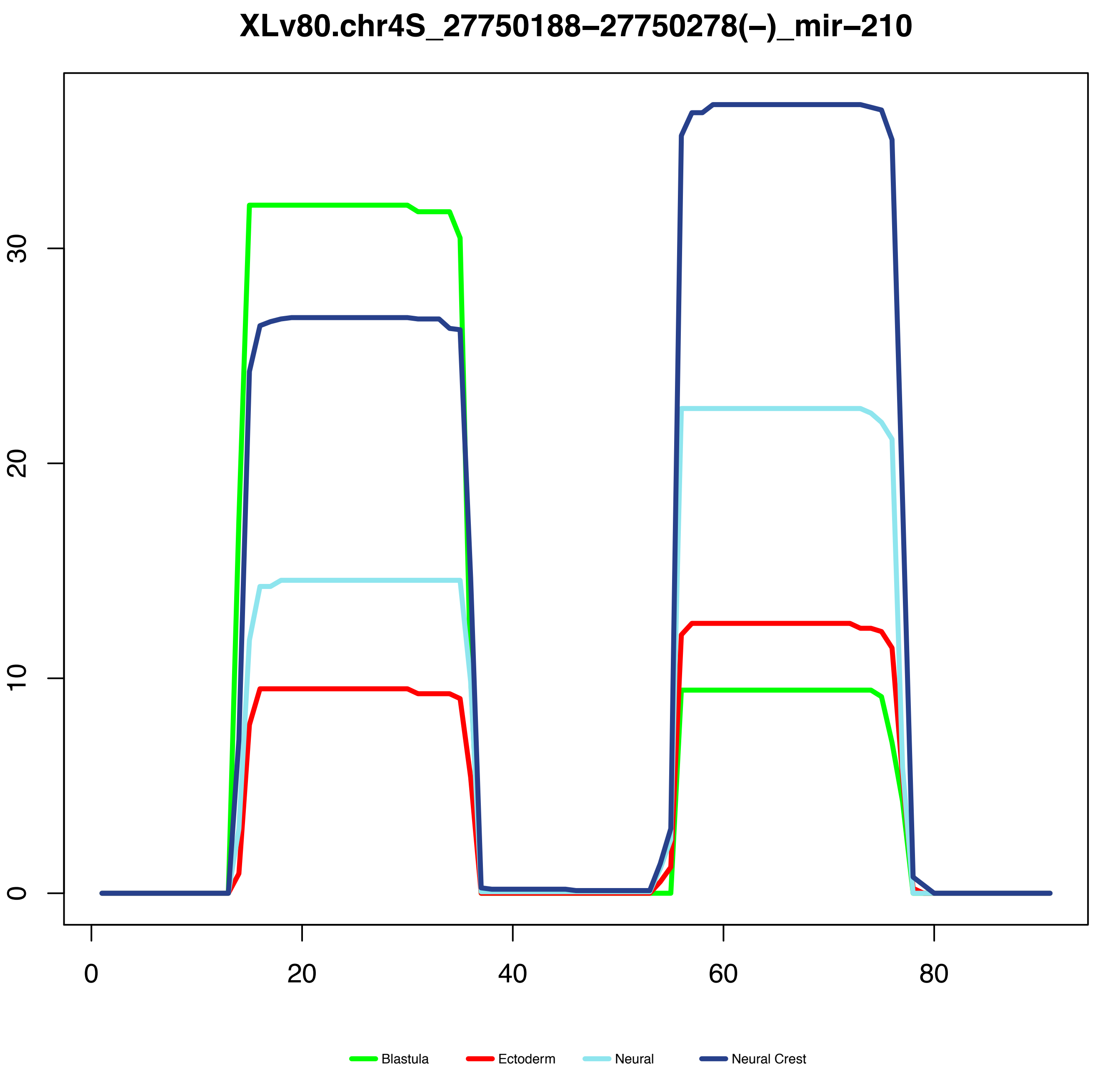

XLv80.chr4S_27750188−27750278(−)_mir−210
0
10
20
30
0
20
40
60
80
Blastula
Ectoderm
Neural
Neural Crest

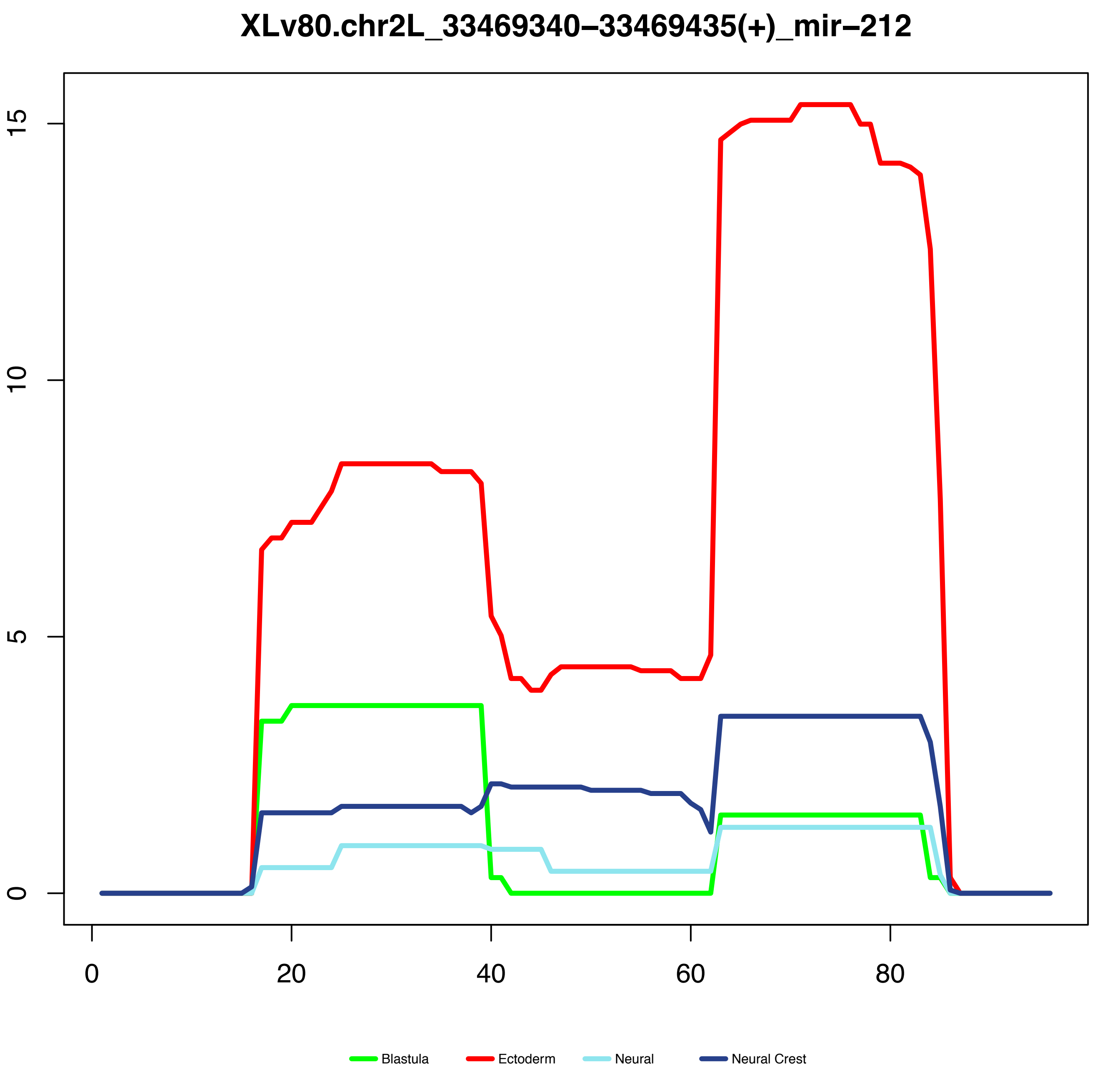

XLv80.chr2L_33469340−33469435(+)_mir−212
0
5
10
15
0
20
40
60
80
Blastula
Ectoderm
Neural
Neural Crest

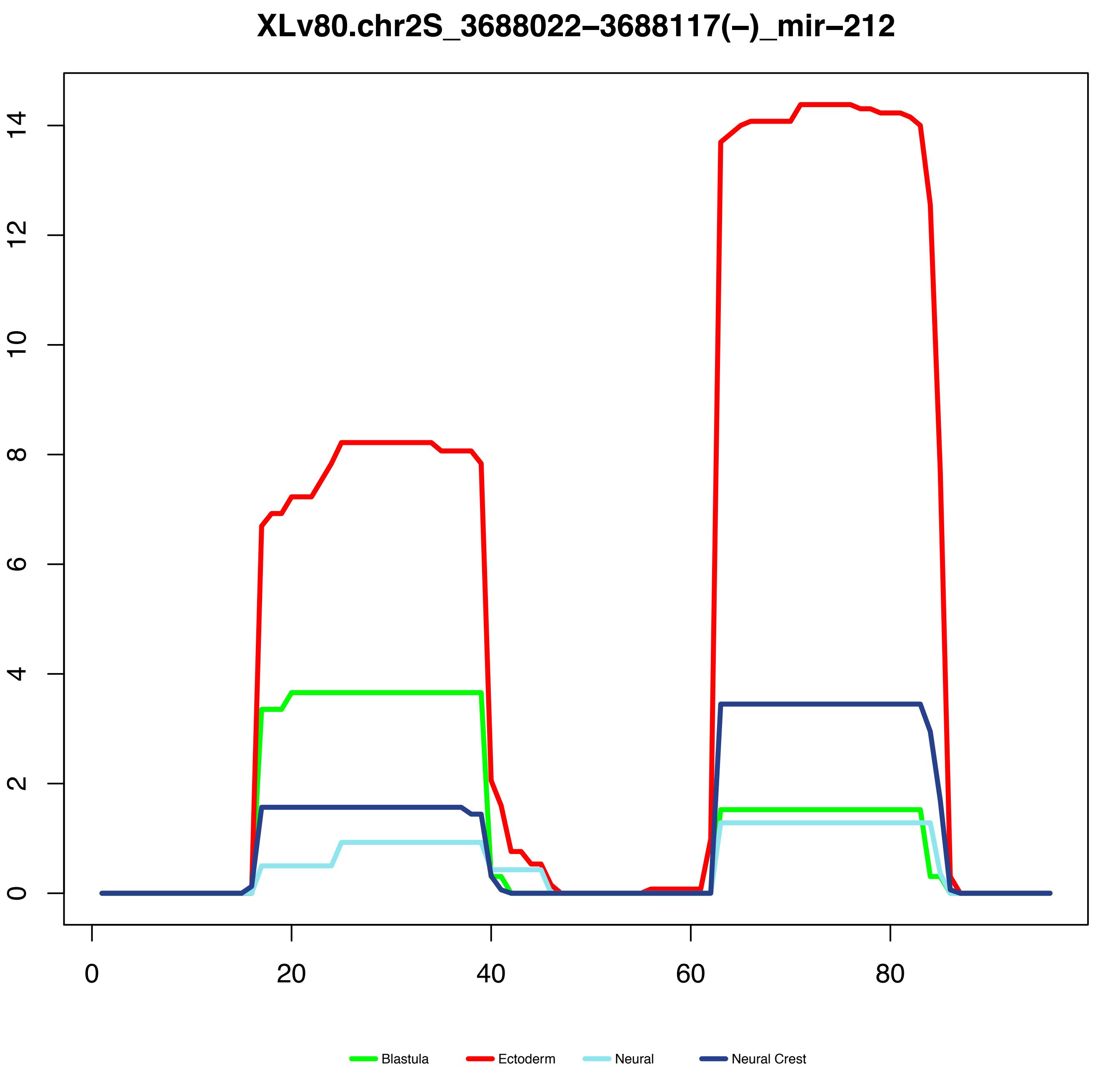
XLv80.chr2S_3688022−3688117(−)_mir−212
0
2
4
6
8
10
12
14
0
20
40
60
80
Blastula
Ectoderm
Neural
Neural Crest

XLv80.chr5S_14184512−14184608(+)_mir−215

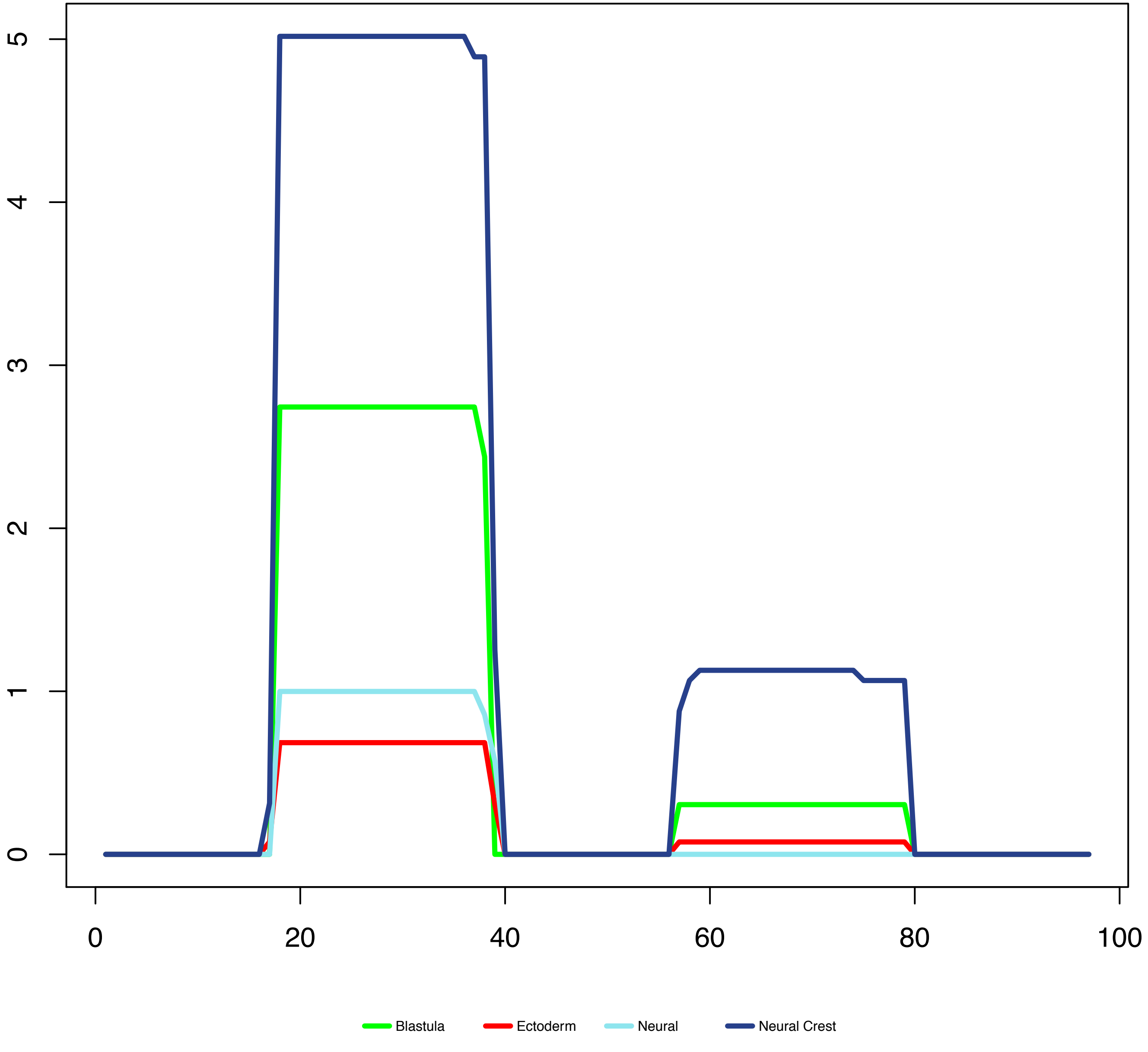

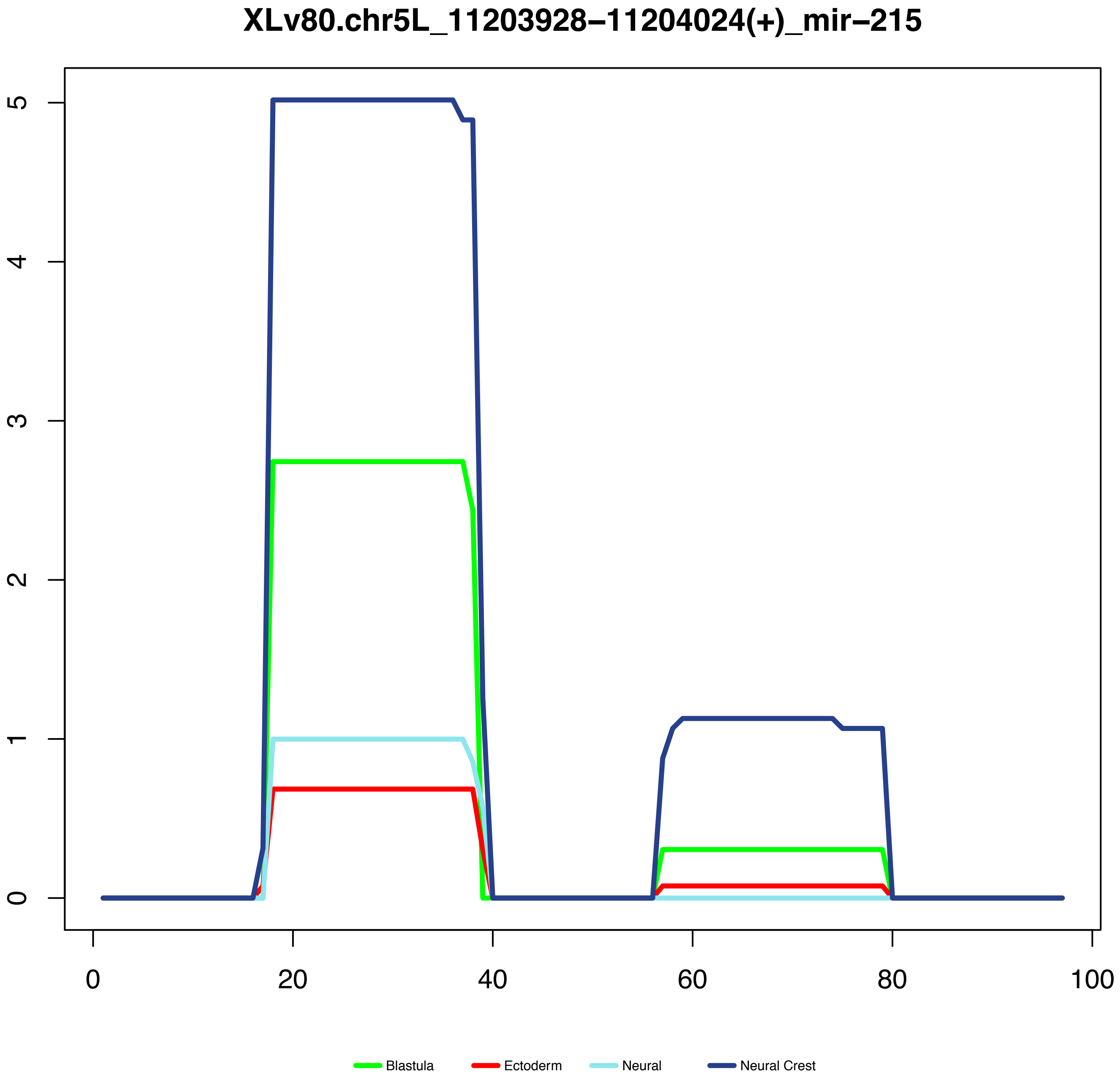

XLv80.chr5L_11203928−11204024(+)_mir−215
0
1
2
3
4
5
0
20
40
60
80
100
Blastula
Ectoderm
Neural
Neural Crest

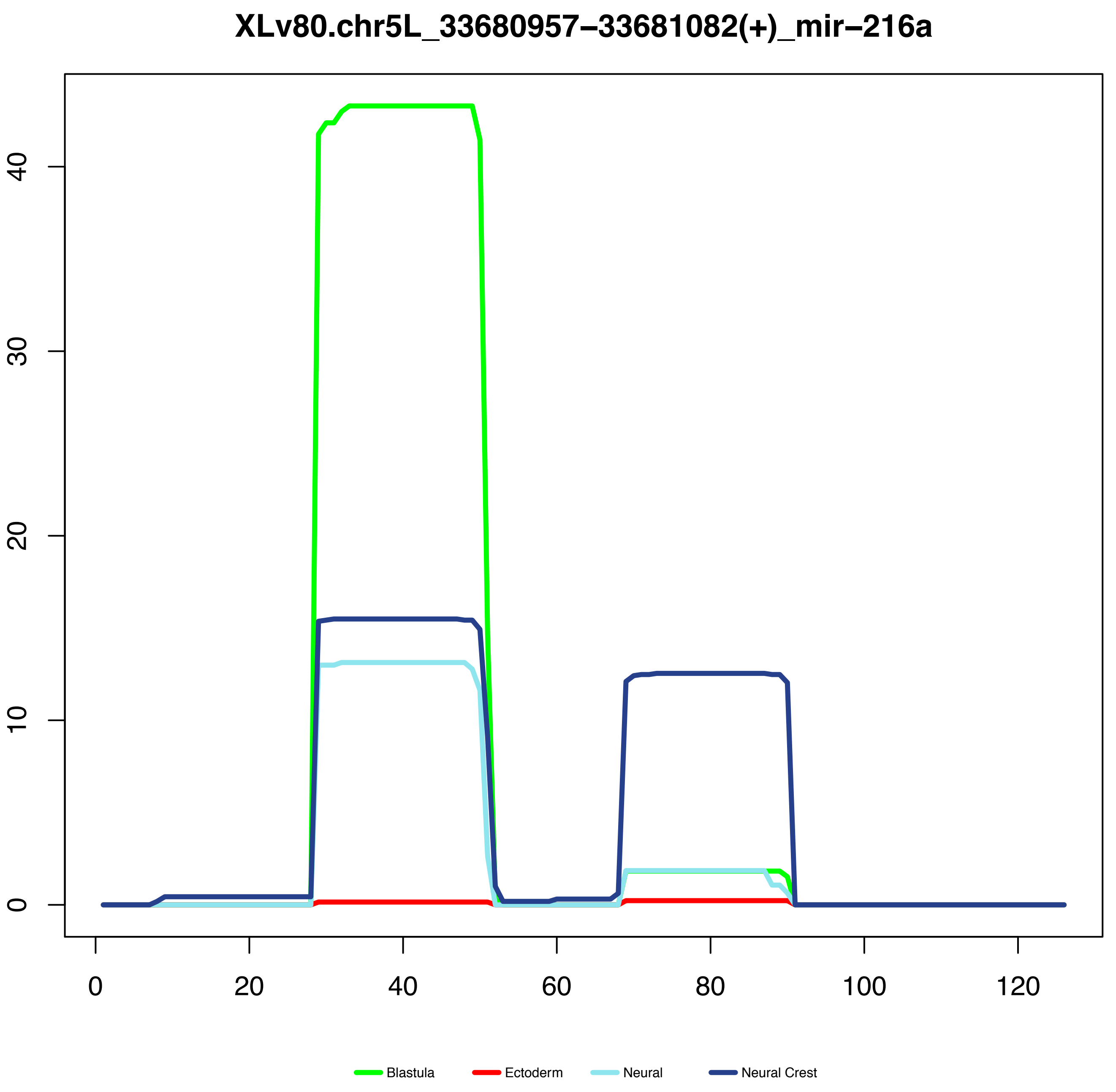
XLv80.chr5L_33680957−33681082(+)_mir−216a
0
10
20
30
40
0
20
40
60
80
100
120
Blastula
Ectoderm
Neural
Neural Crest

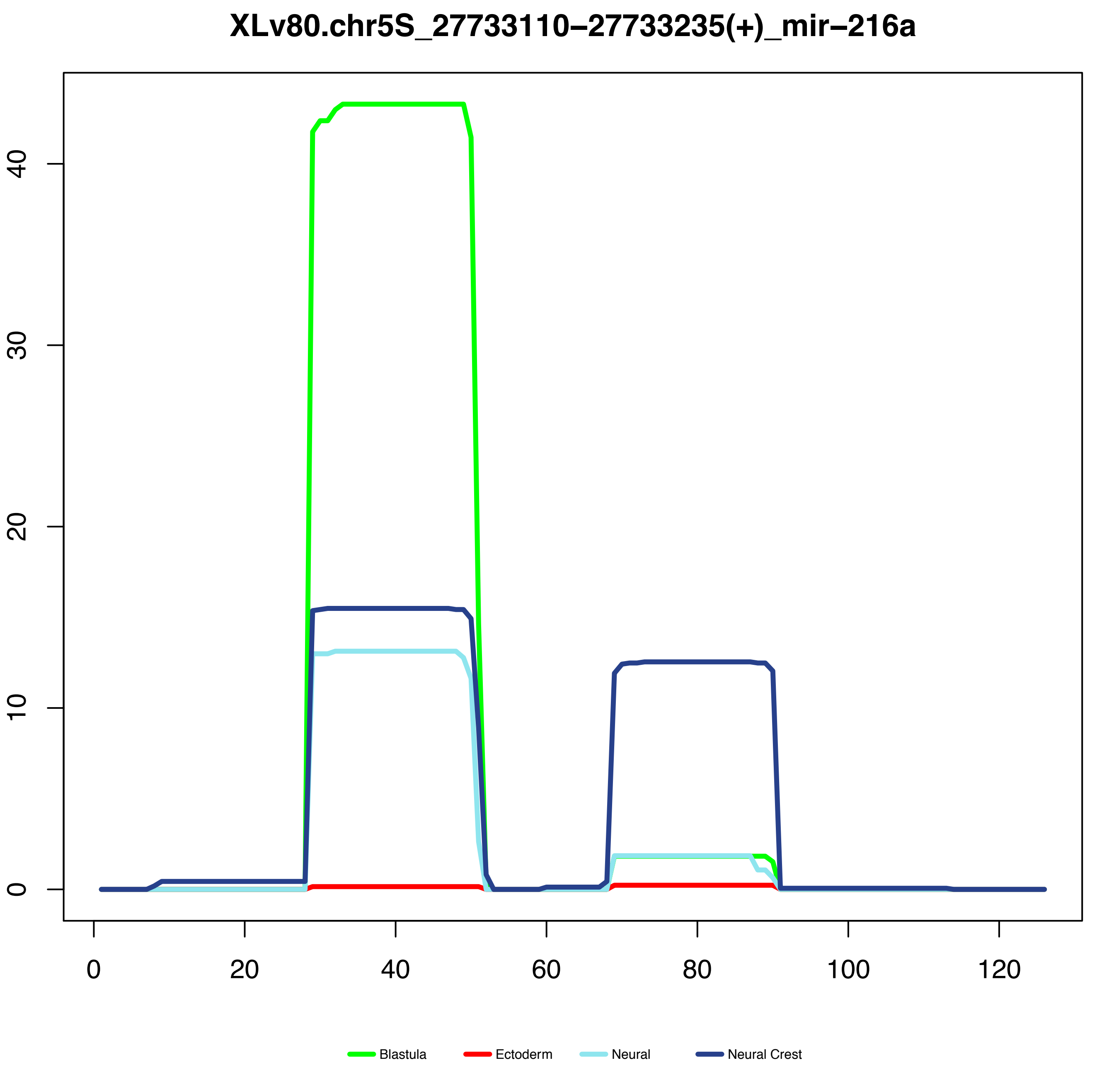
XLv80.chr5S_27733110−27733235(+)_mir−216a
0
10
20
30
40
0
20
40
60
80
100
120
Blastula
Ectoderm
Neural
Neural Crest

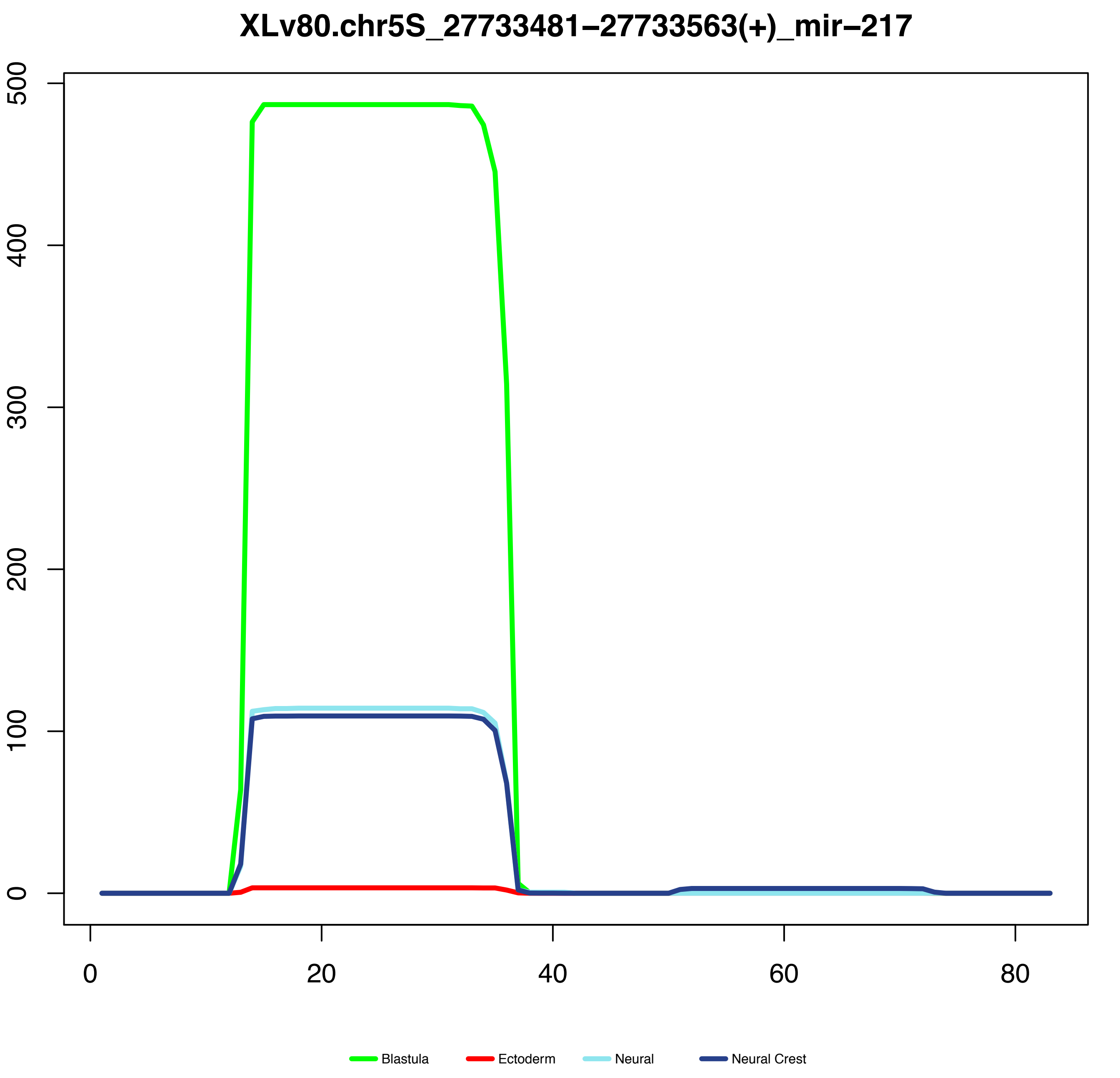
XLv80.chr5S_27733481−27733563(+)_mir−217
500
400
300
200
100
0
0
20
40
60
80
Blastula
Ectoderm
Neural
Neural Crest

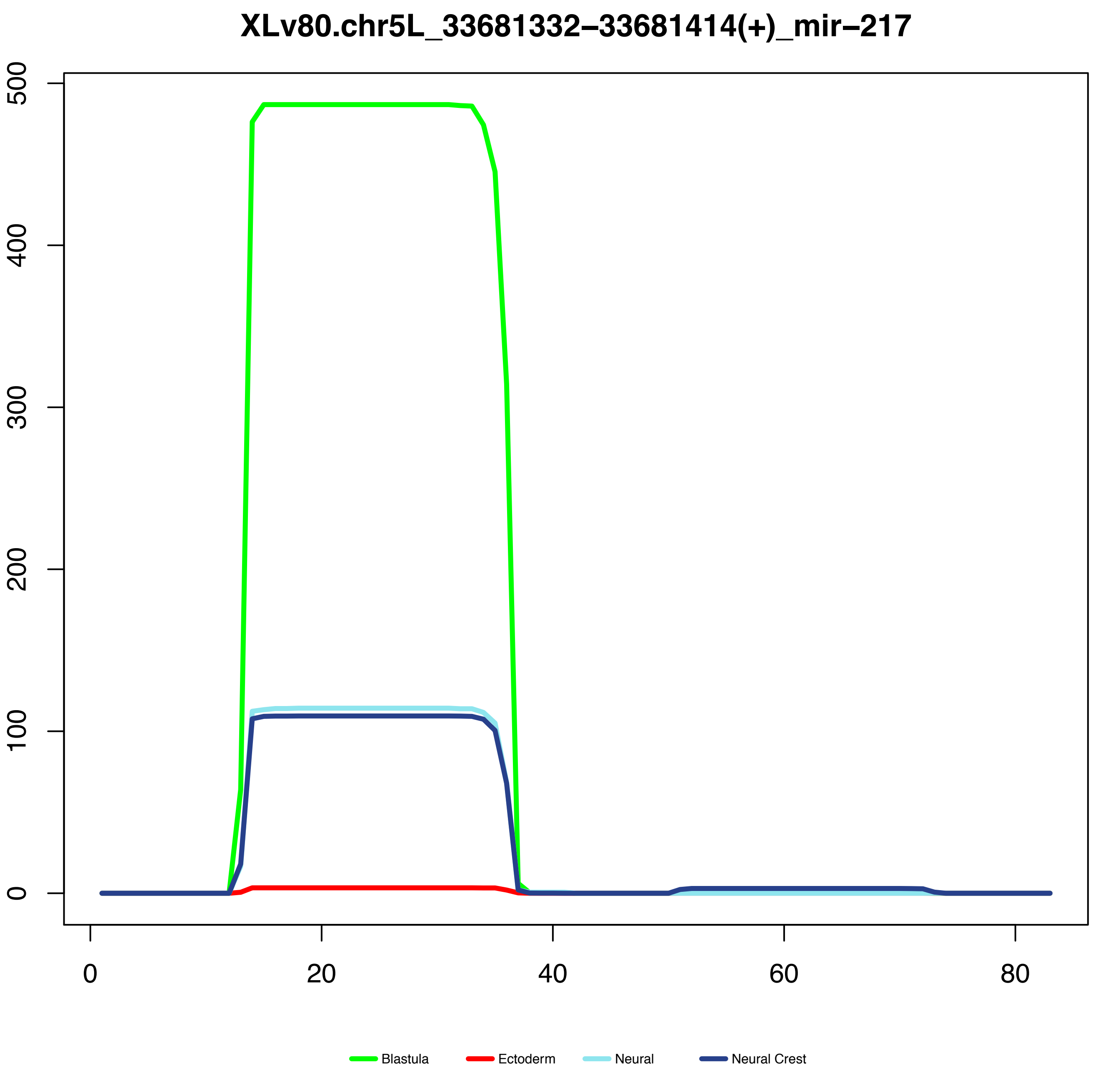

XLv80.chr5L_33681332−33681414(+)_mir−217
0
100
200
300
400
500
0
20
40
60
80
Blastula
Ectoderm
Neural
Neural Crest

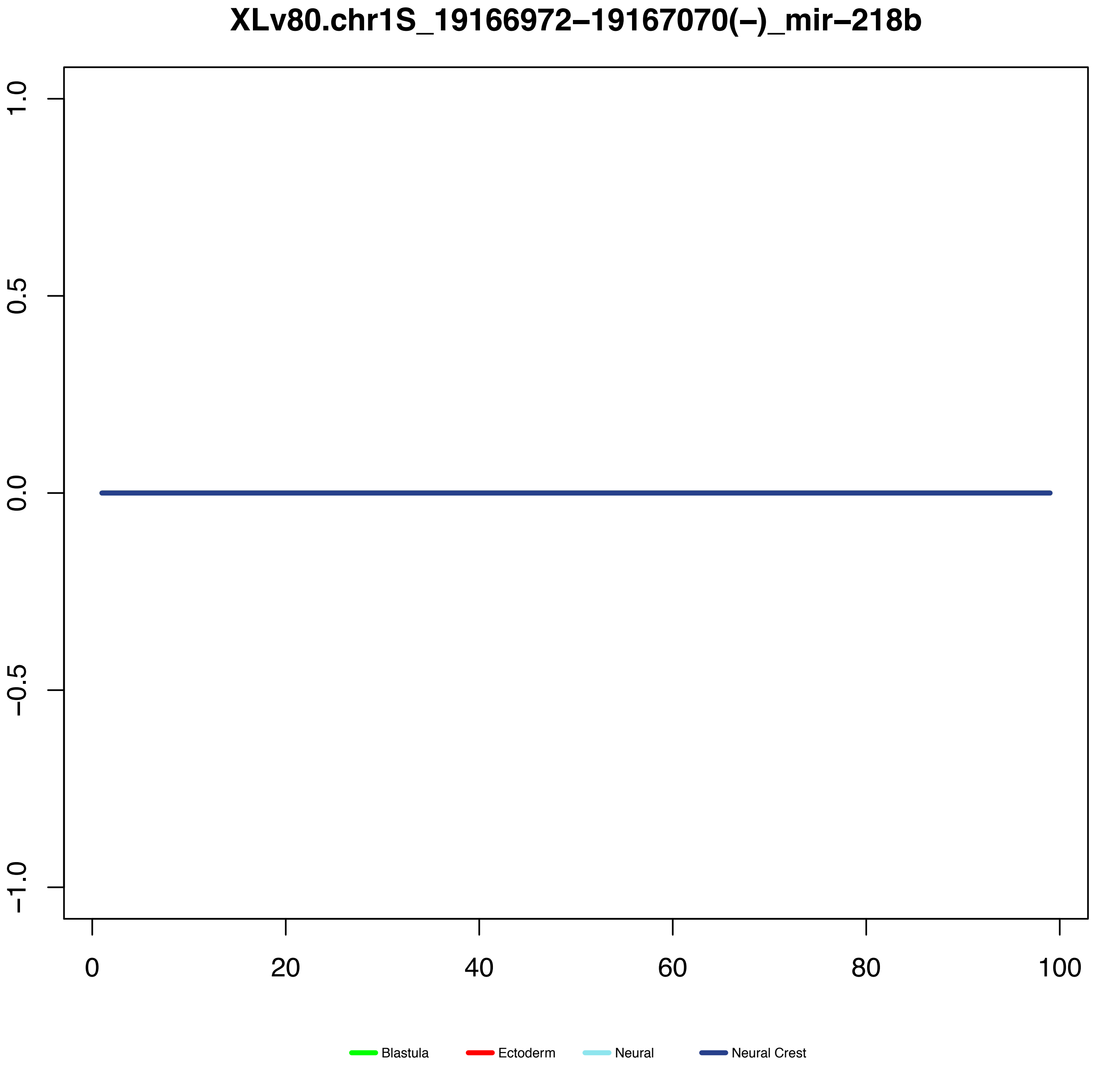
XLv80.chr1S_19166972−19167070(−)_mir−218b
1.0
0.5
0.0
−0.5
−1.0
0
20
40
60
80
100
Blastula
Ectoderm
Neural
Neural Crest

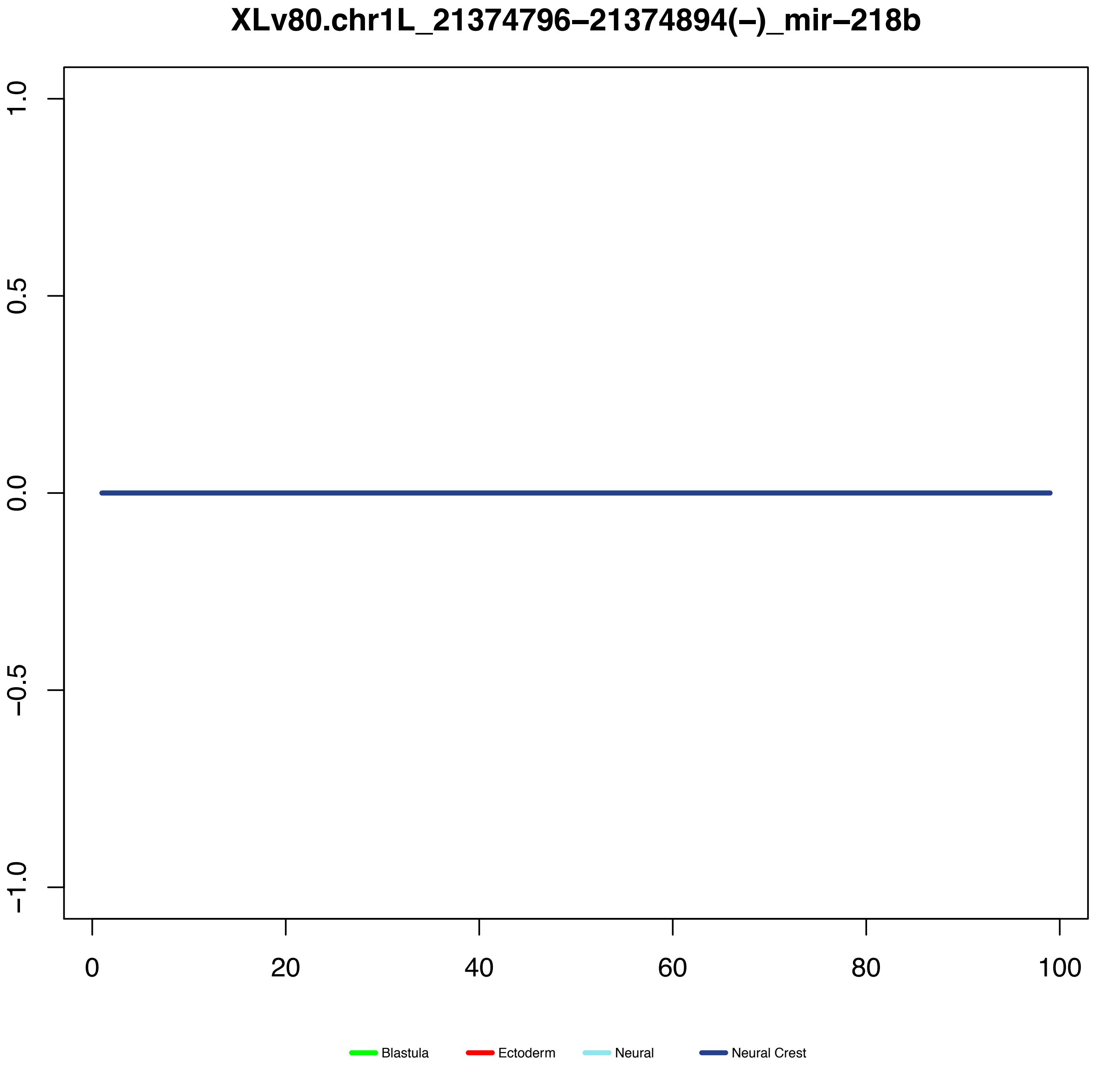

XLv80.chr1L_21374796–21374894(–)_mir–218b
1.0
0.5
0.0
–0.5
–1.0
0
20
40
60
80
100
Blastula
Ectoderm
Neural
Neural Crest

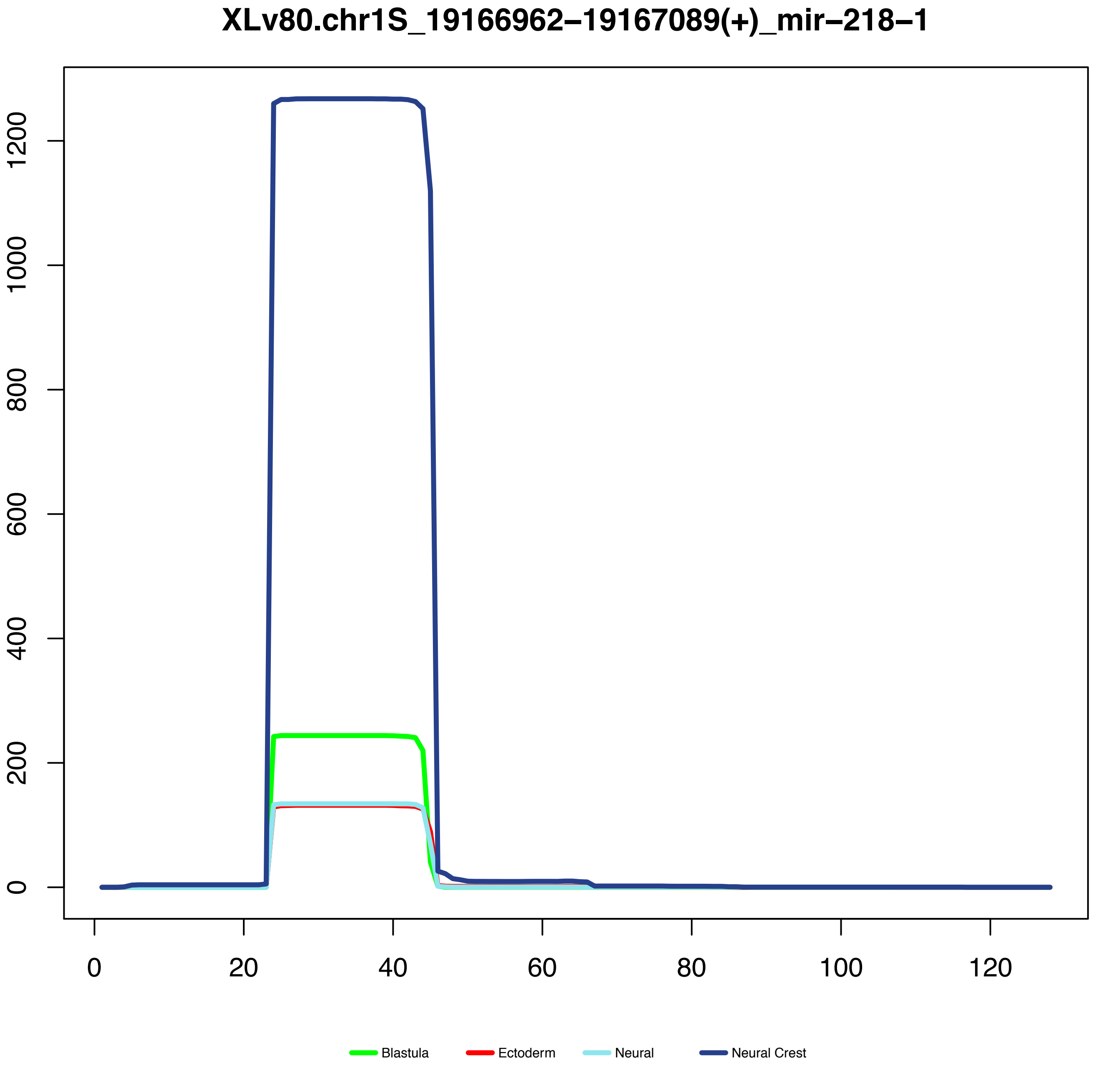

XLv80.chr1S_19166962−19167089(+)_mir−218−1
0
200
400
600
800
1000
1200
0
20
40
60
80
100
120
Blastula
Ectoderm
Neural
Neural Crest

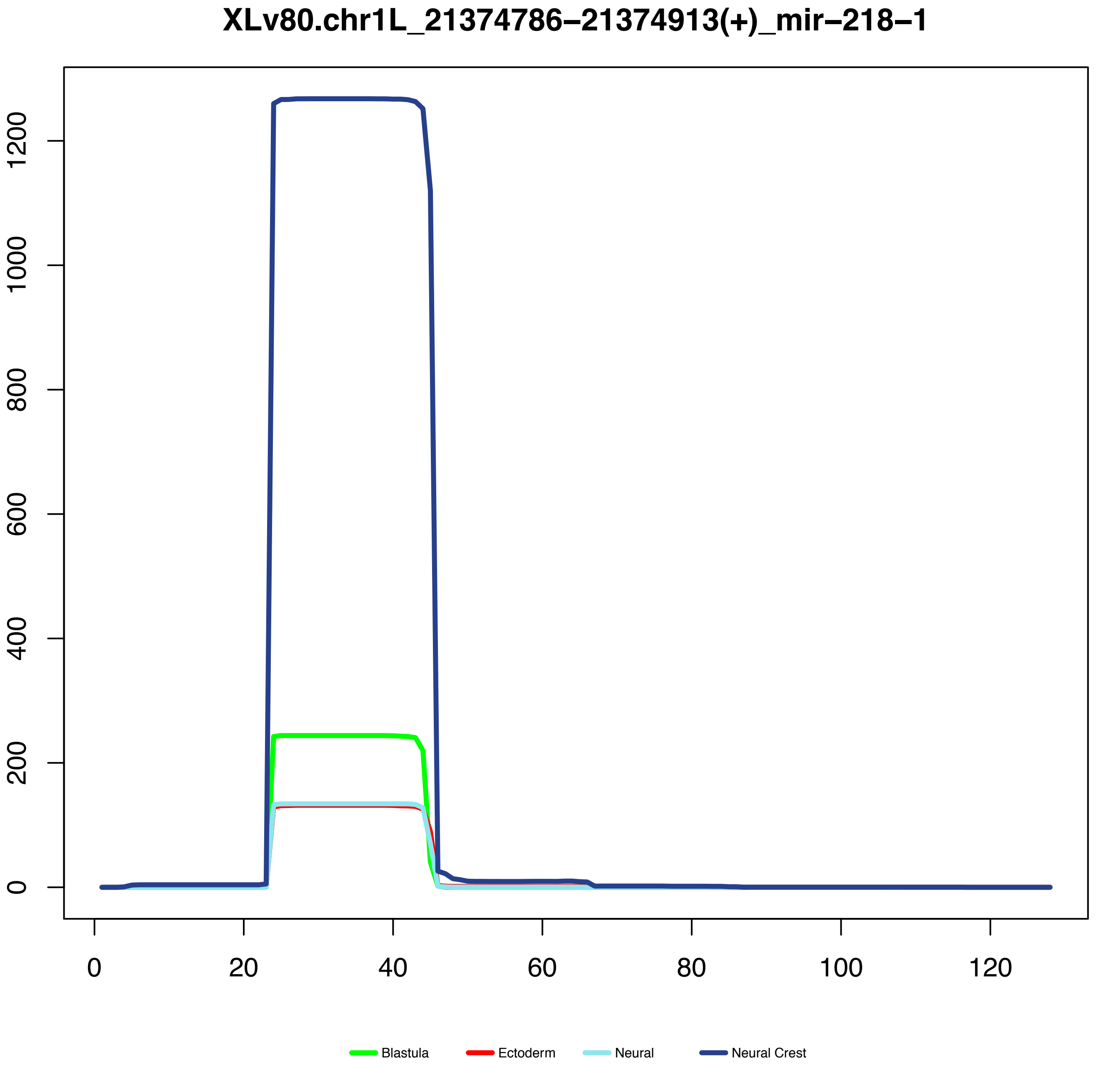

XLv80.chr1L_21374786−21374913(+)_mir−218−1
0
200
400
600
800
1000
1200
0
20
40
60
80
100
120
Blastula
Ectoderm
Neural
Neural Crest

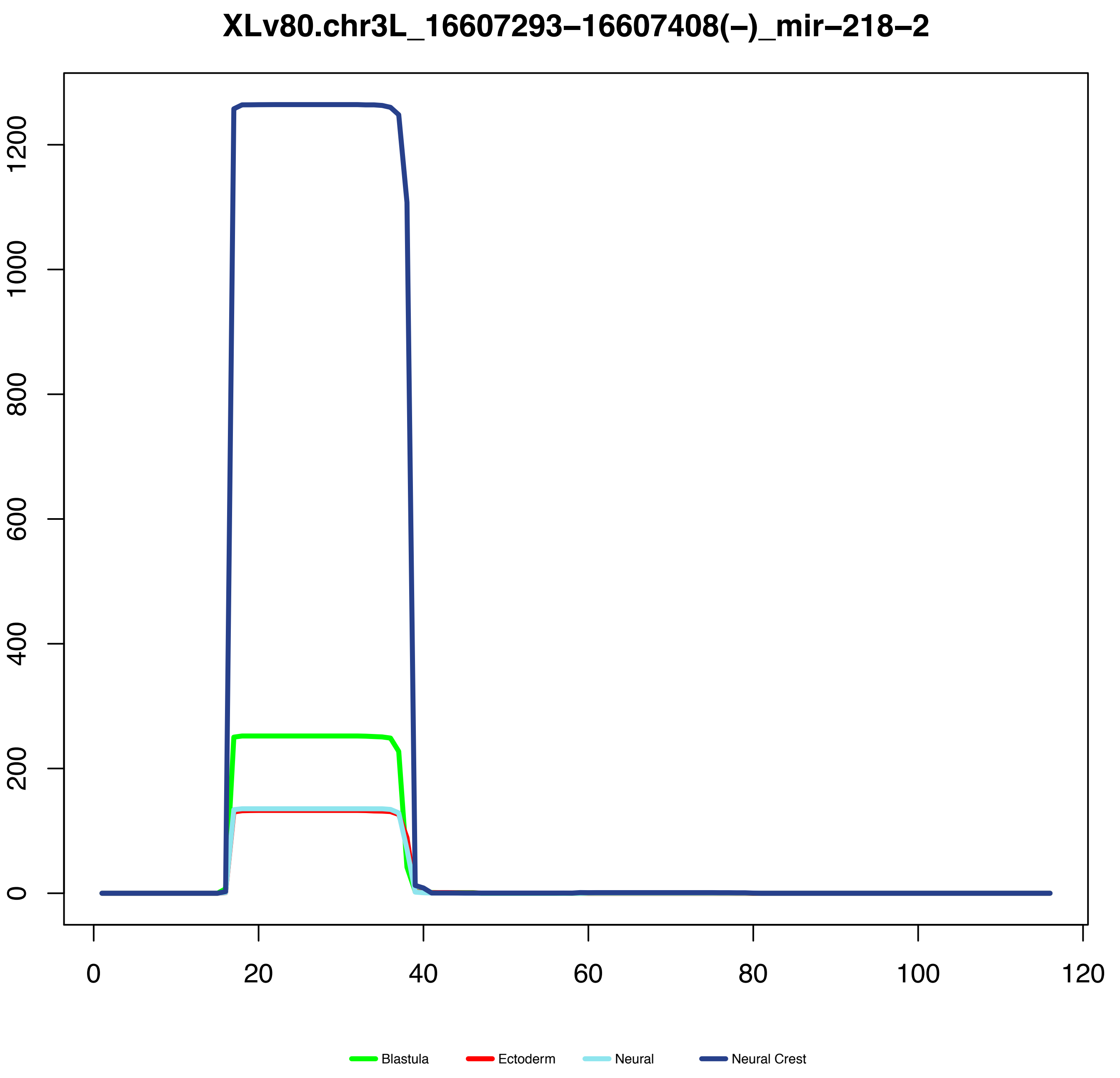
XLv80.chr3L_16607293−16607408(−)_mir−218−2
0
200
400
600
800
1000
1200
0
20
40
60
80
100
120
Blastula
Ectoderm
Neural
Neural Crest

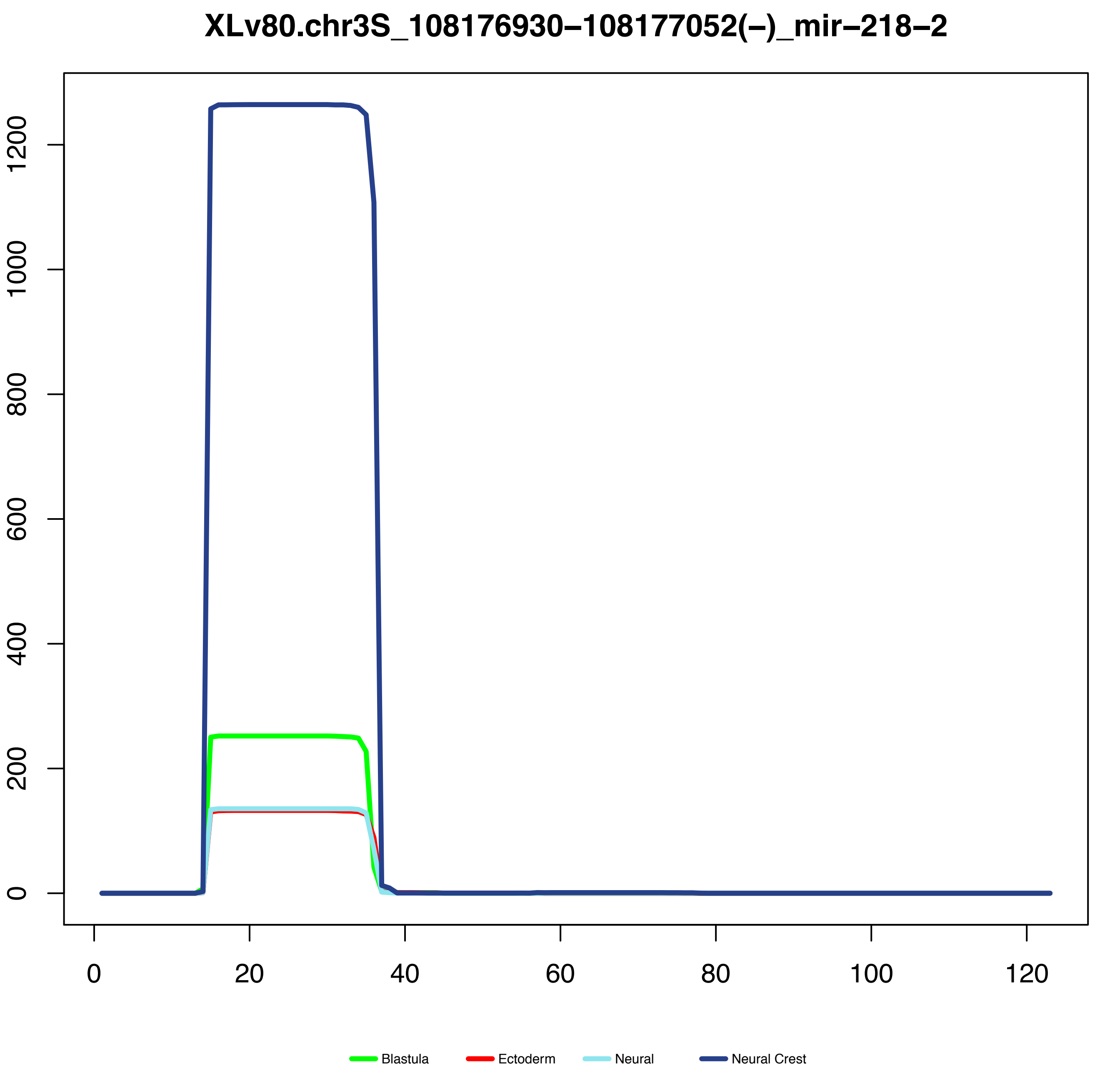

XLv80.chr3S_108176930−108177052(−)_mir−218−2
0
200
400
600
800
1000
1200
0
20
40
60
80
100
120
Blastula
Ectoderm
Neural
Neural Crest

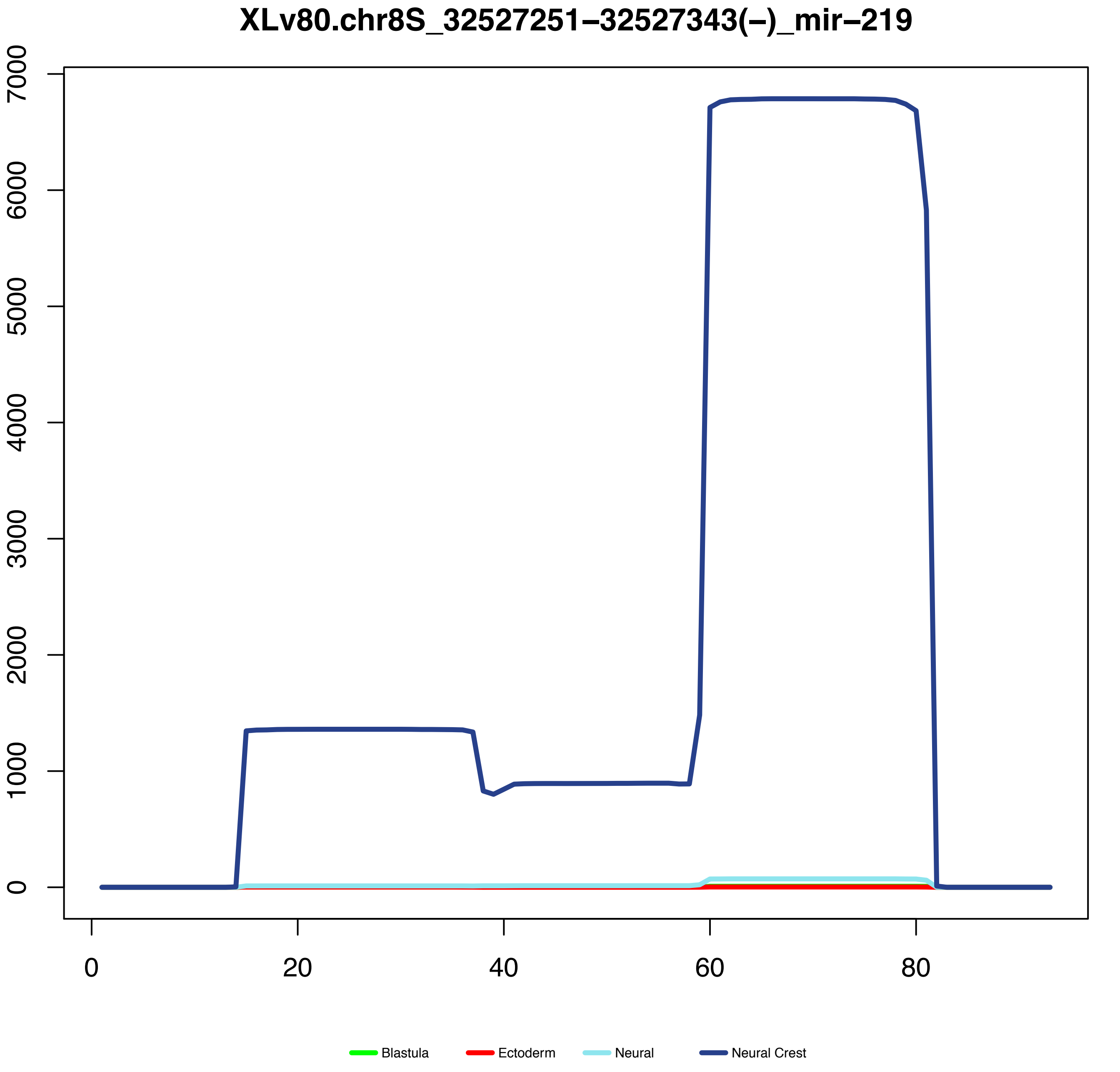
XLv80.chr8S_32527251−32527343(−)_mir−219
0
1000
2000
3000
4000
5000
6000
7000
0
20
40
60
80
Blastula
Ectoderm
Neural
Neural Crest

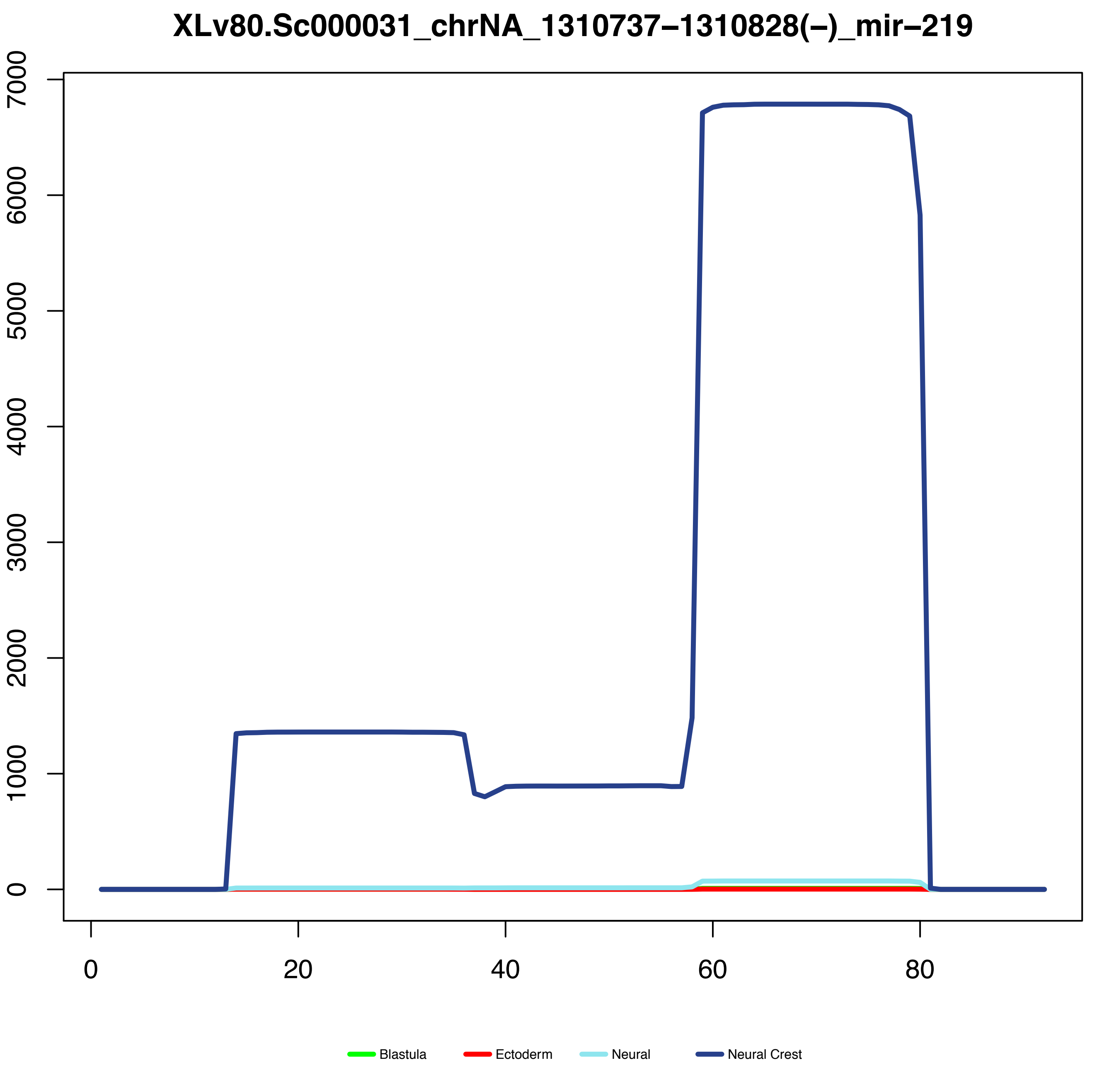

XLv80.Sc000031_chrNA_1310737−1310828(−)_mir−219
0
1000
2000
3000
4000
5000
6000
7000
0
20
40
60
80
Blastula
Ectoderm
Neural
Neural Crest

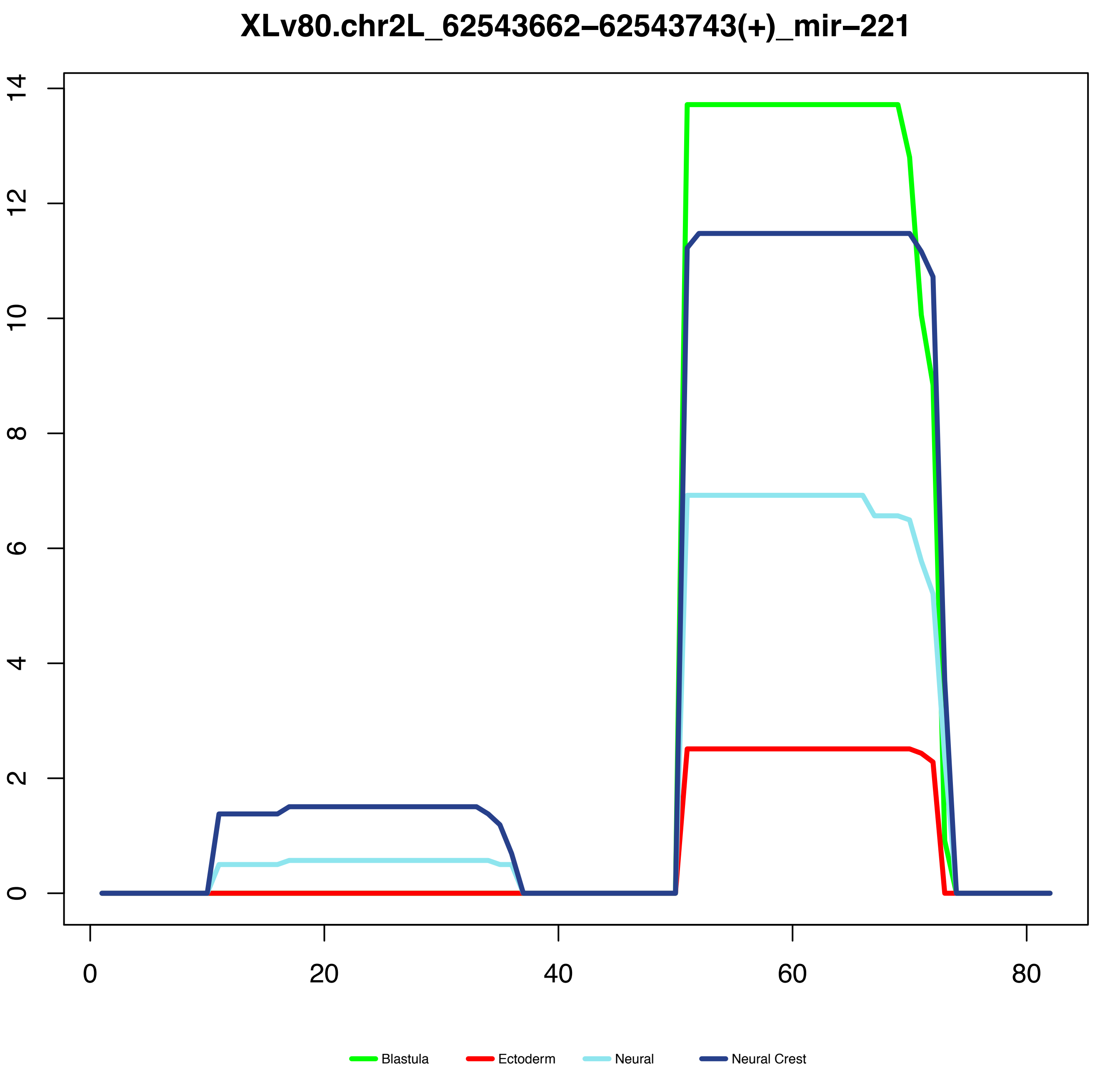

XLv80.chr2L_62543662−62543743(+)_mir−221
0
2
4
6
8
10
12
14
0
20
40
60
80
Blastula
Ectoderm
Neural
Neural Crest

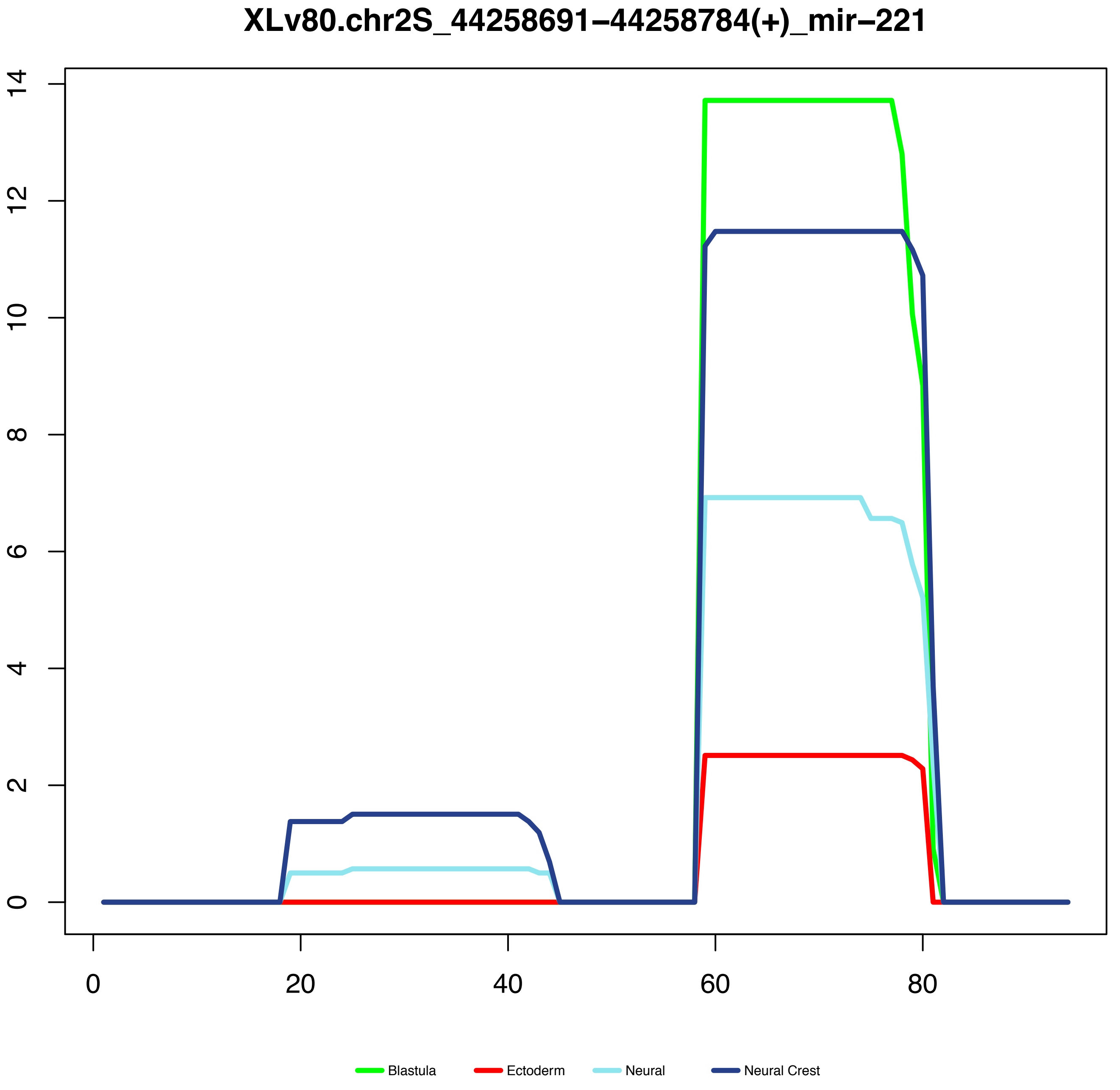
XLv80.chr2S_44258691−44258784(+)_mir−221
0
2
4
6
8
10
12
14
0
20
40
60
80
Blastula
Ectoderm
Neural
Neural Crest

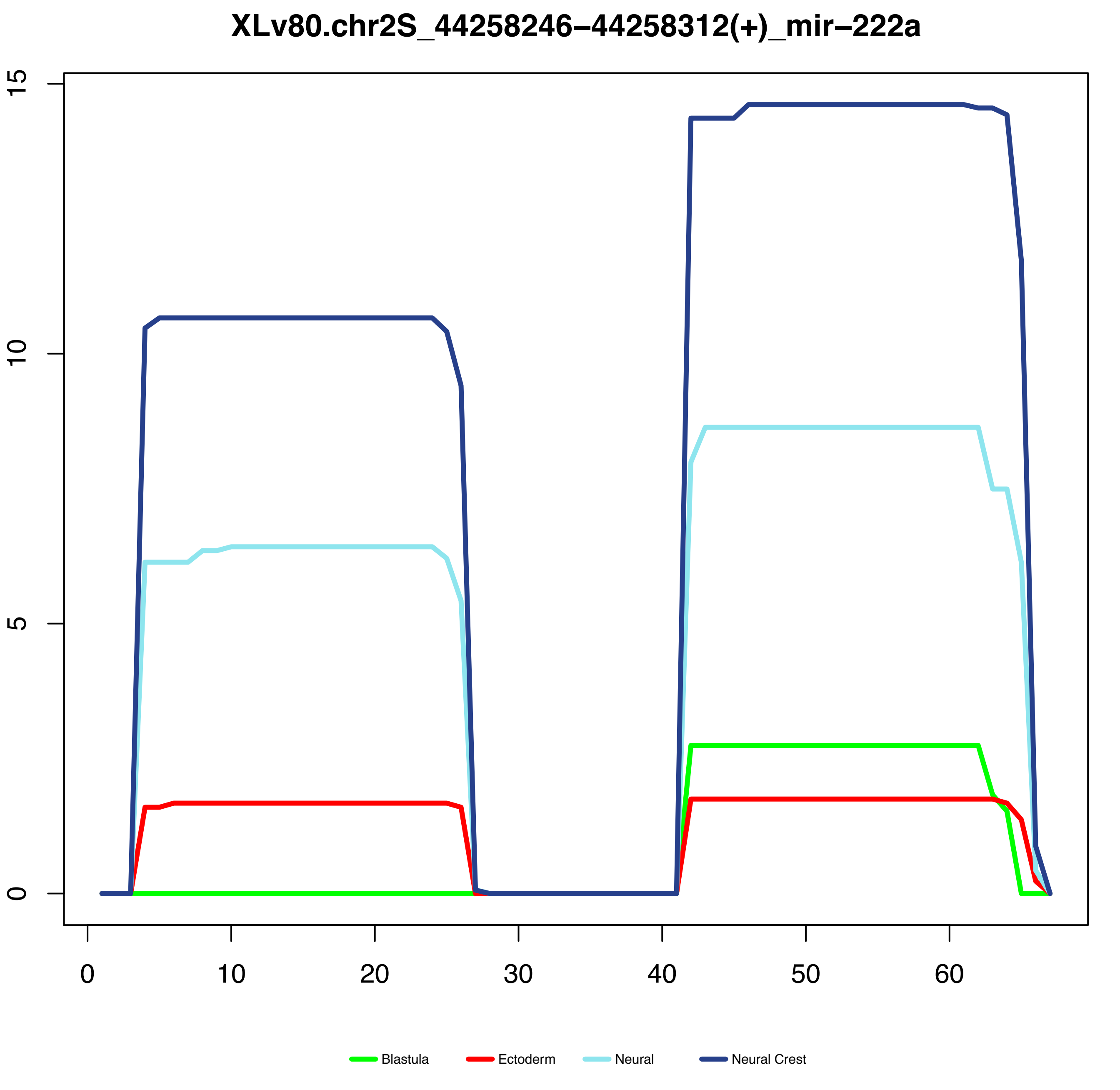

XLv80.chr2S_44258246−44258312(+)_mir−222a
15
10
5
0
0
10
20
30
40
50
60
Blastula
Ectoderm
Neural
Neural Crest

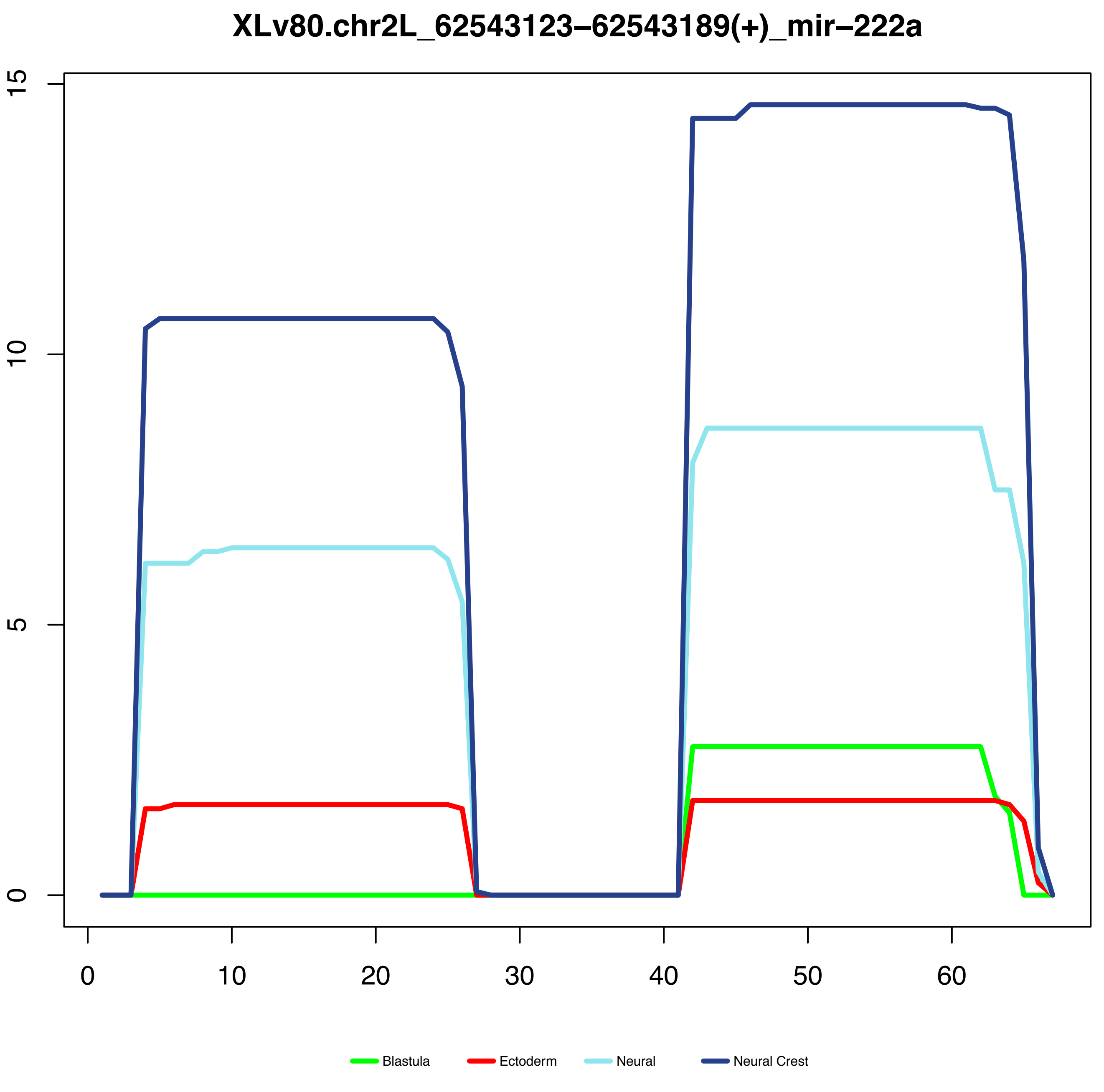
XLv80.chr2L_62543123−62543189(+)_mir−222a
0
5
10
15
0
10
20
30
40
50
60
Blastula
Ectoderm
Neural
Neural Crest

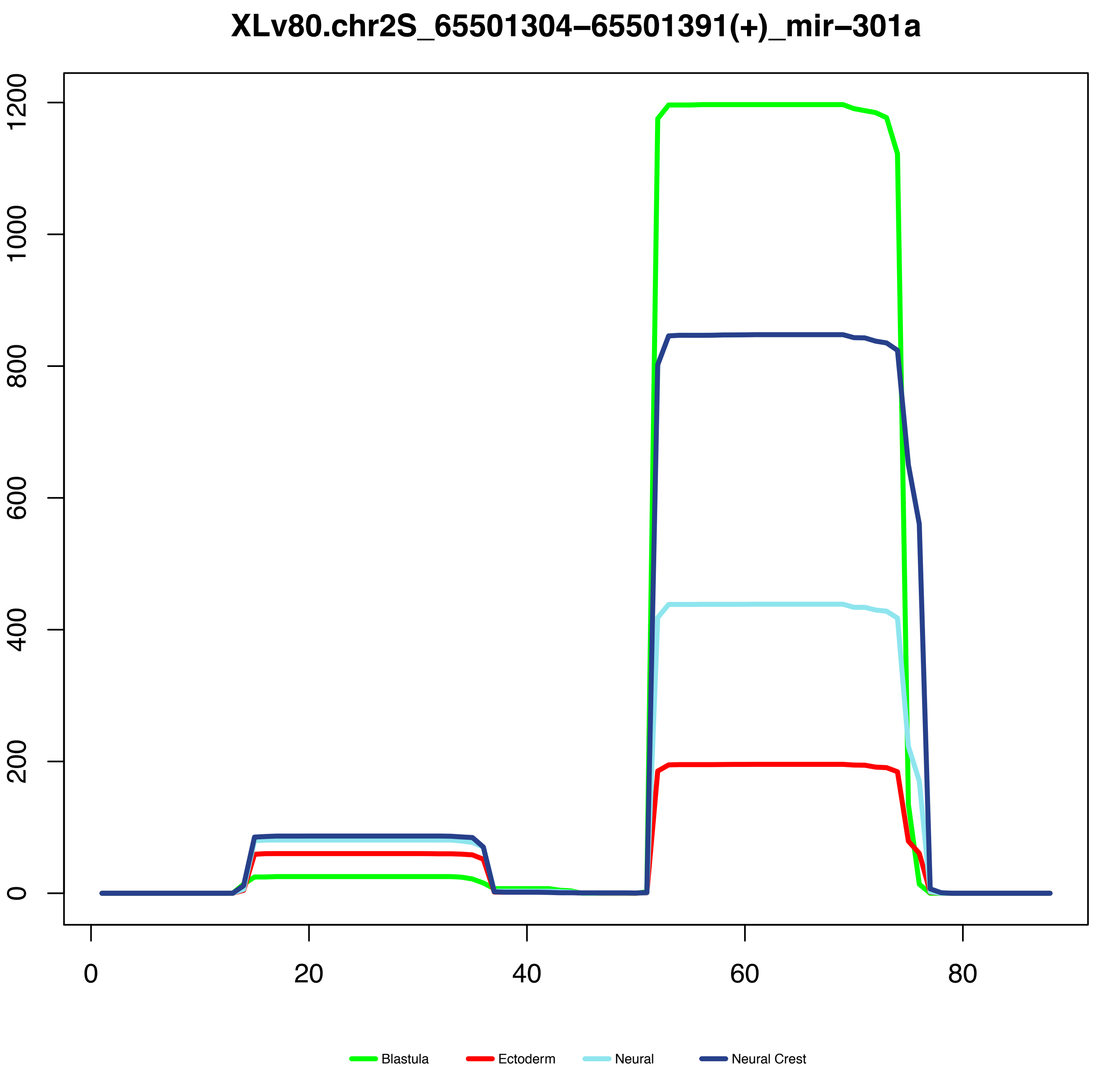

XLv80.chr2S_65501304−65501391(+)_mir−301a
0
200
400
600
800
1000
1200
0
20
40
60
80
Blastula
Ectoderm
Neural
Neural Crest

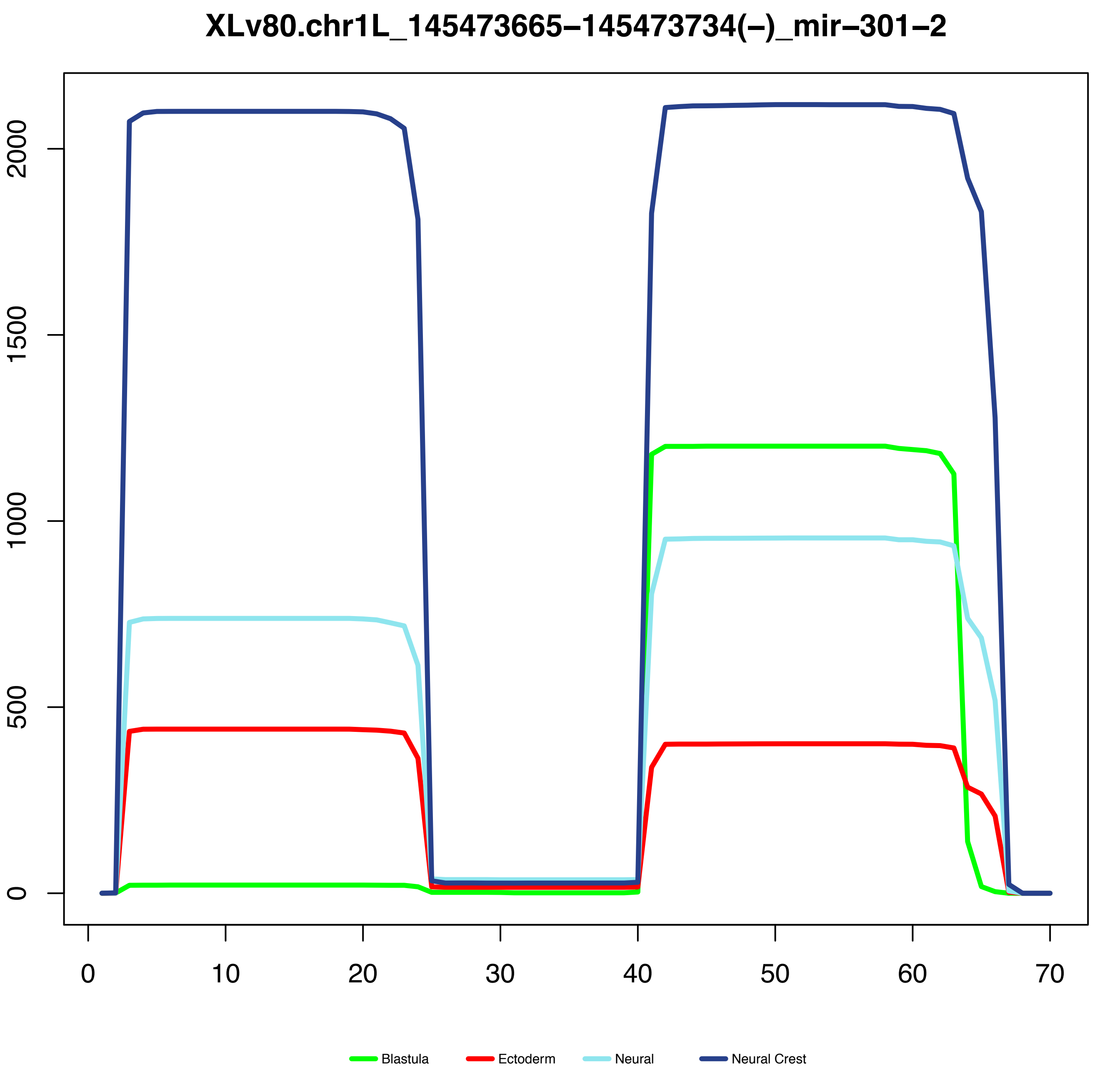

XLv80.chr1L_145473665−145473734(−)_mir−301−2
0
500
1000
1500
2000
0
10
20
30
40
50
60
70
Blastula
Ectoderm
Neural
Neural Crest

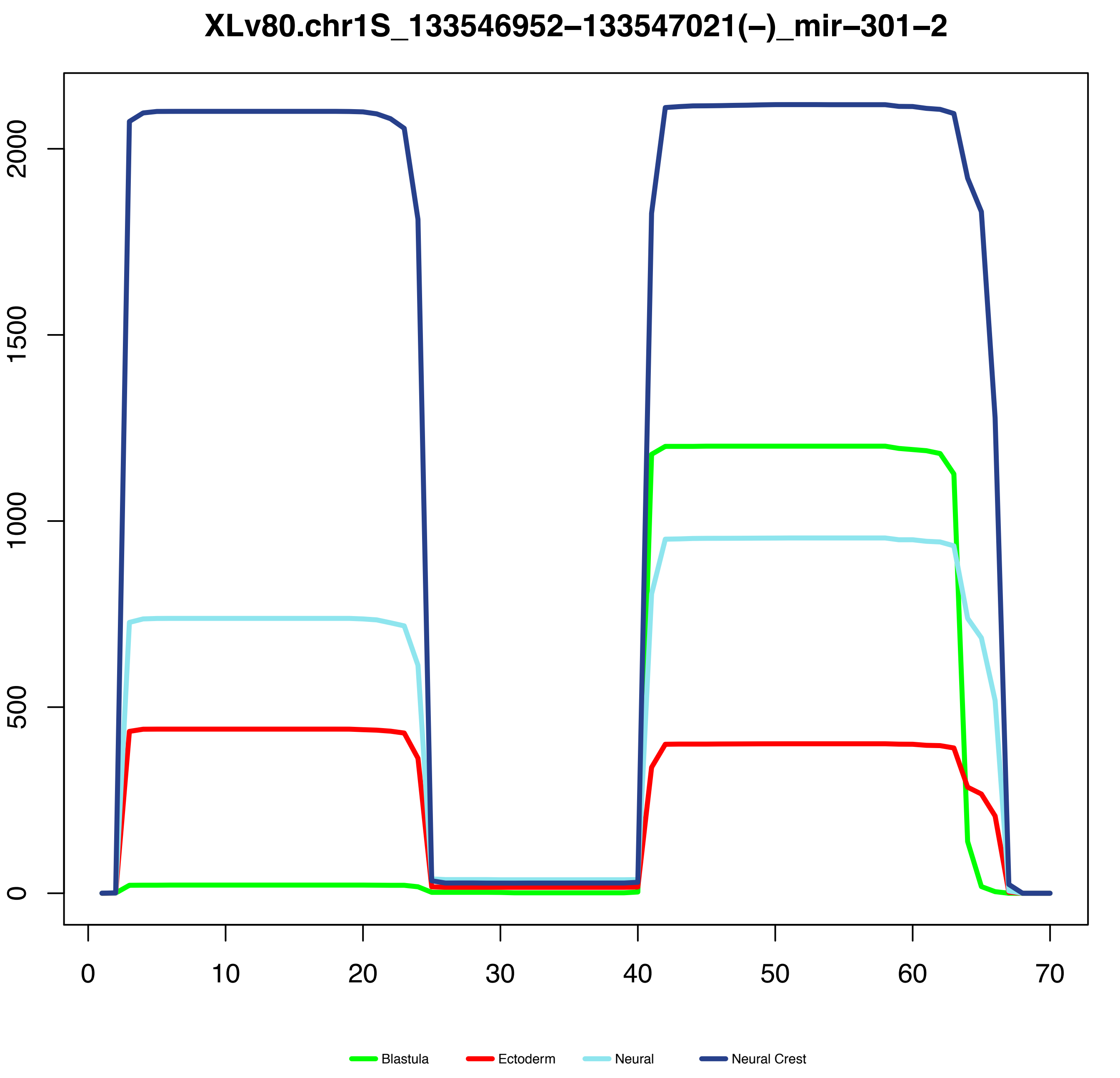
XLv80.chr1S_133546952−133547021(−)_mir−301−2
0
500
1000
1500
2000
0
10
20
30
40
50
60
70
Blastula
Ectoderm
Neural
Neural Crest

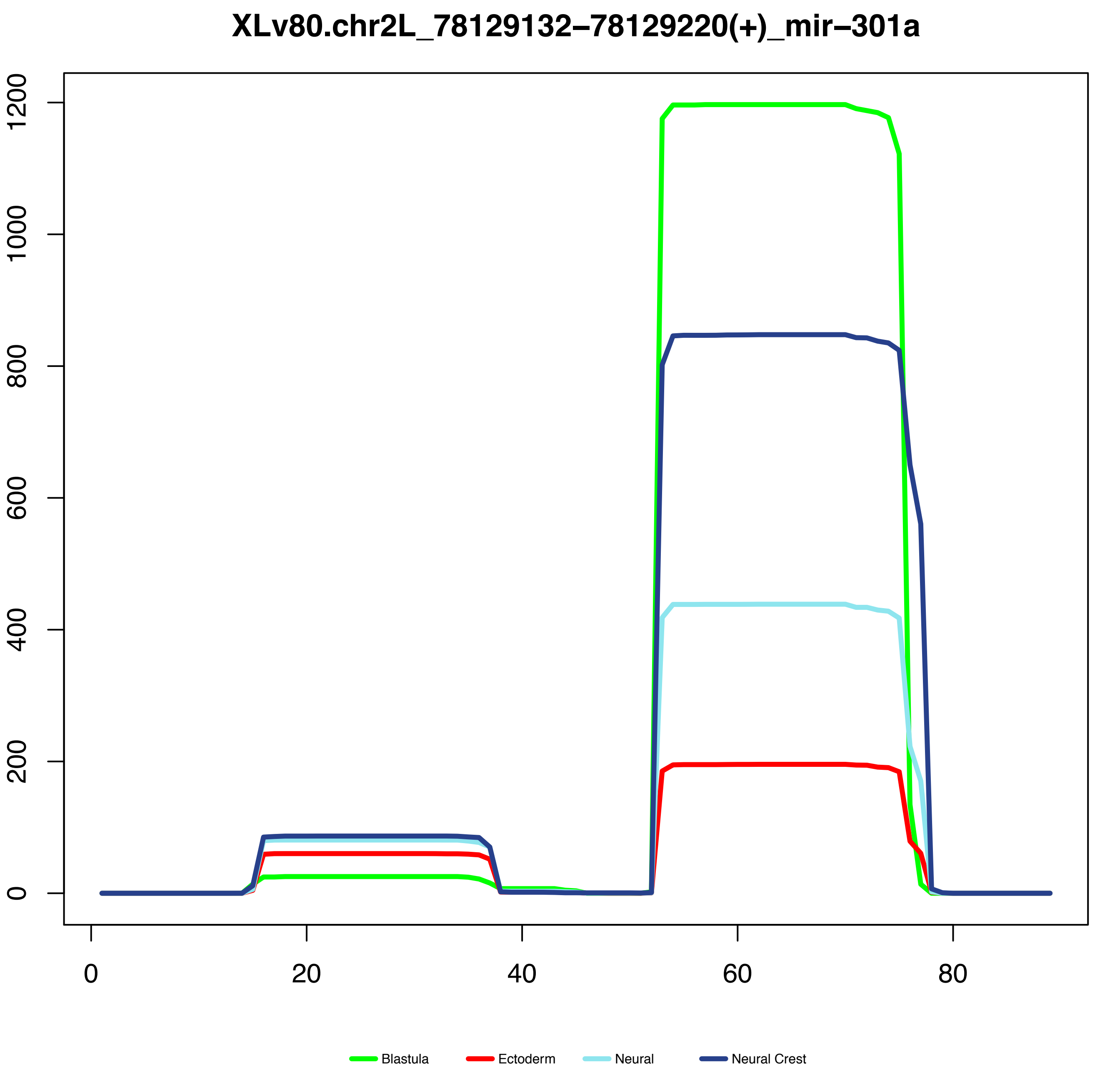
XLv80.chr2L_78129132−78129220(+)_mir−301a
0
200
400
600
800
1000
1200
0
20
40
60
80
Blastula
Ectoderm
Neural
Neural Crest

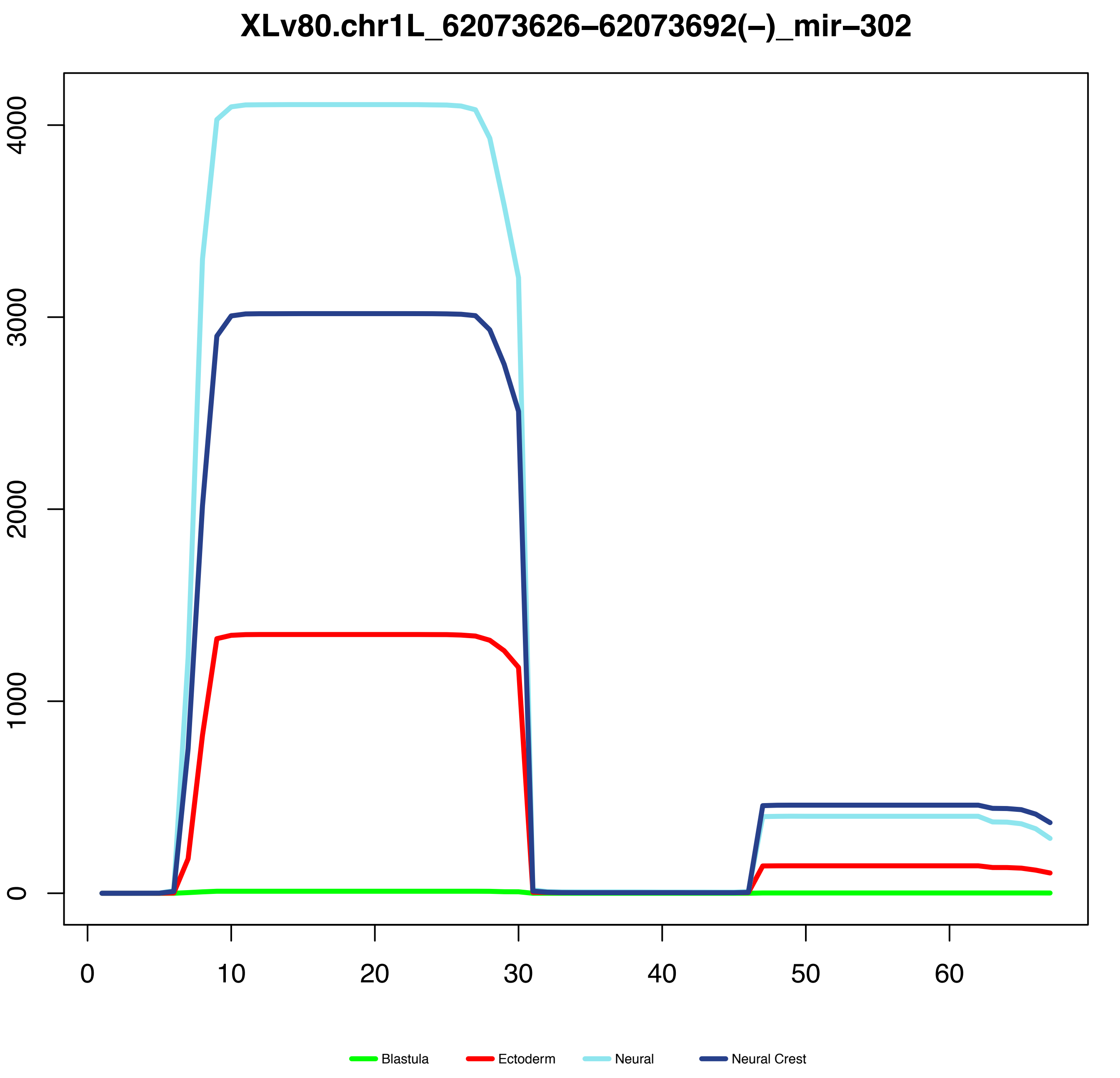
XLv80.chr1L_62073626–62073692(–)_mir–302
0
1000
2000
3000
4000
0
10
20
30
40
50
60
Blastula
Ectoderm
Neural
Neural Crest

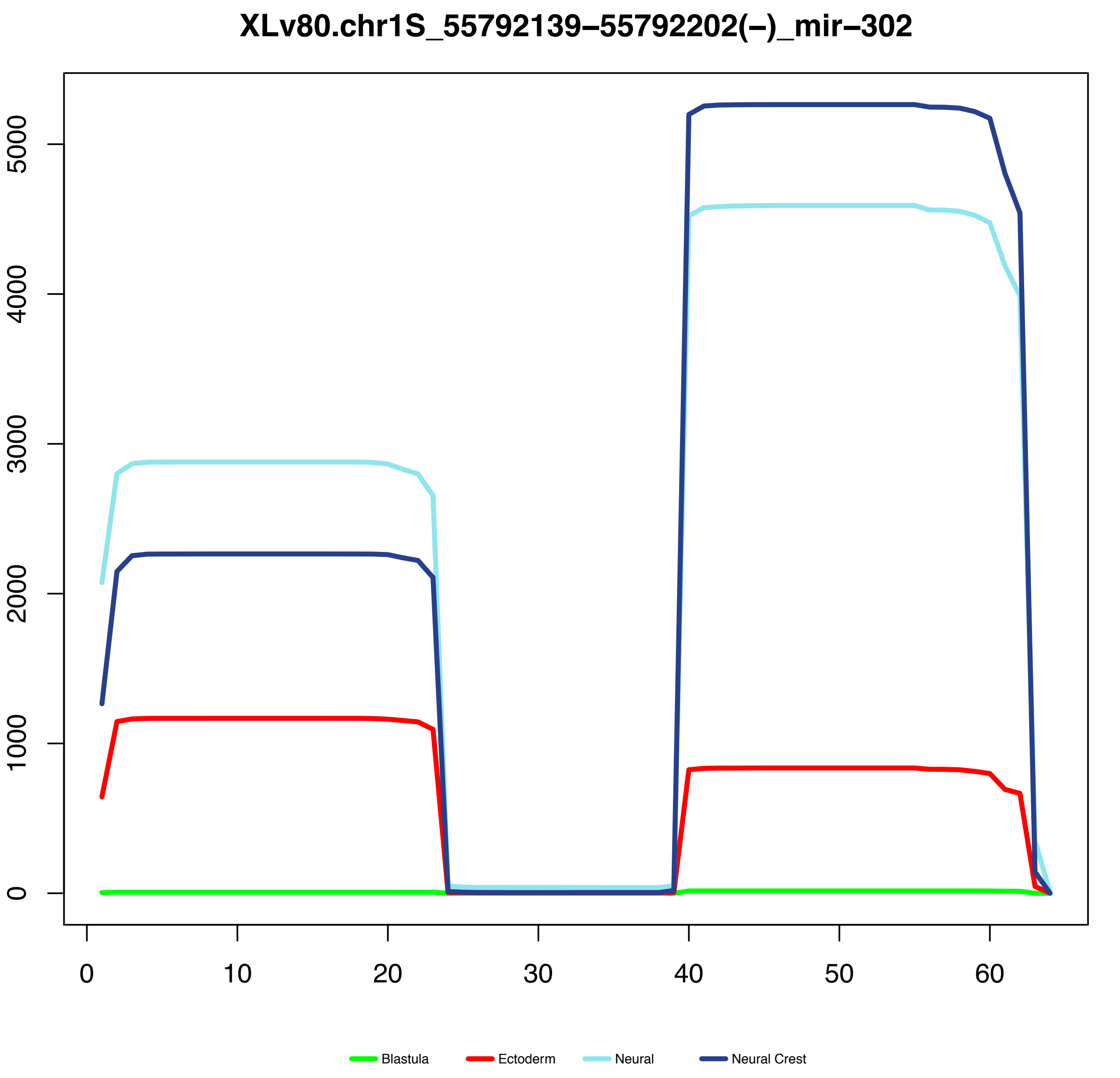

XLv80.chr1S_55792139−55792202(−)_mir−302
0
1000
2000
3000
4000
5000
0
10
20
30
40
50
60
Blastula
Ectoderm
Neural
Neural Crest

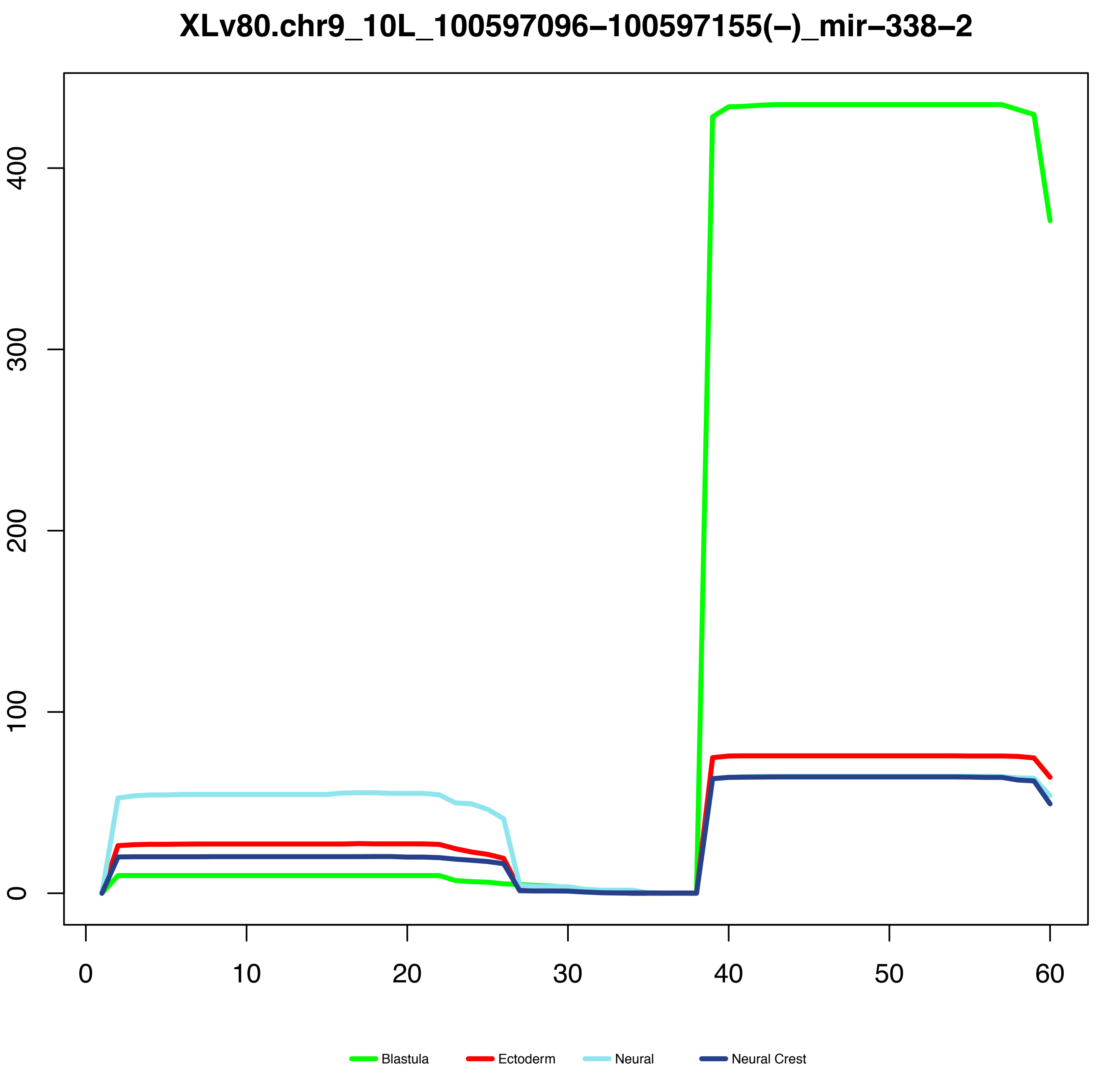
XLv80.chr9_10L_100597096−100597155(−)_mir−338−2
0
100
200
300
400
0
10
20
30
40
50
60
Blastula
Ectoderm
Neural
Neural Crest

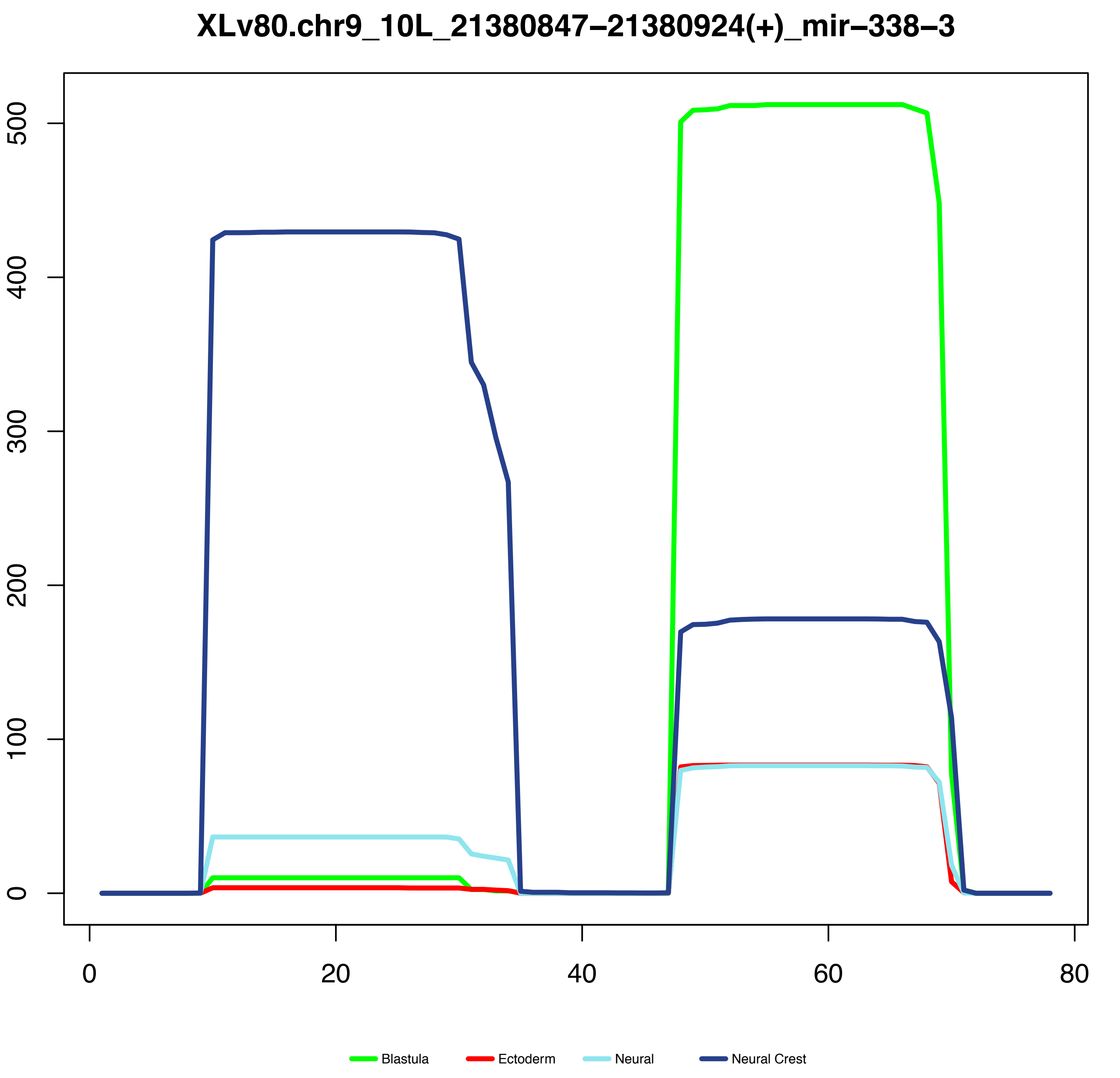

XLv80.chr9_10L_21380847−21380924(+)_mir−338−3
0
100
200
300
400
500
0
20
40
60
80
Blastula
Ectoderm
Neural
Neural Crest

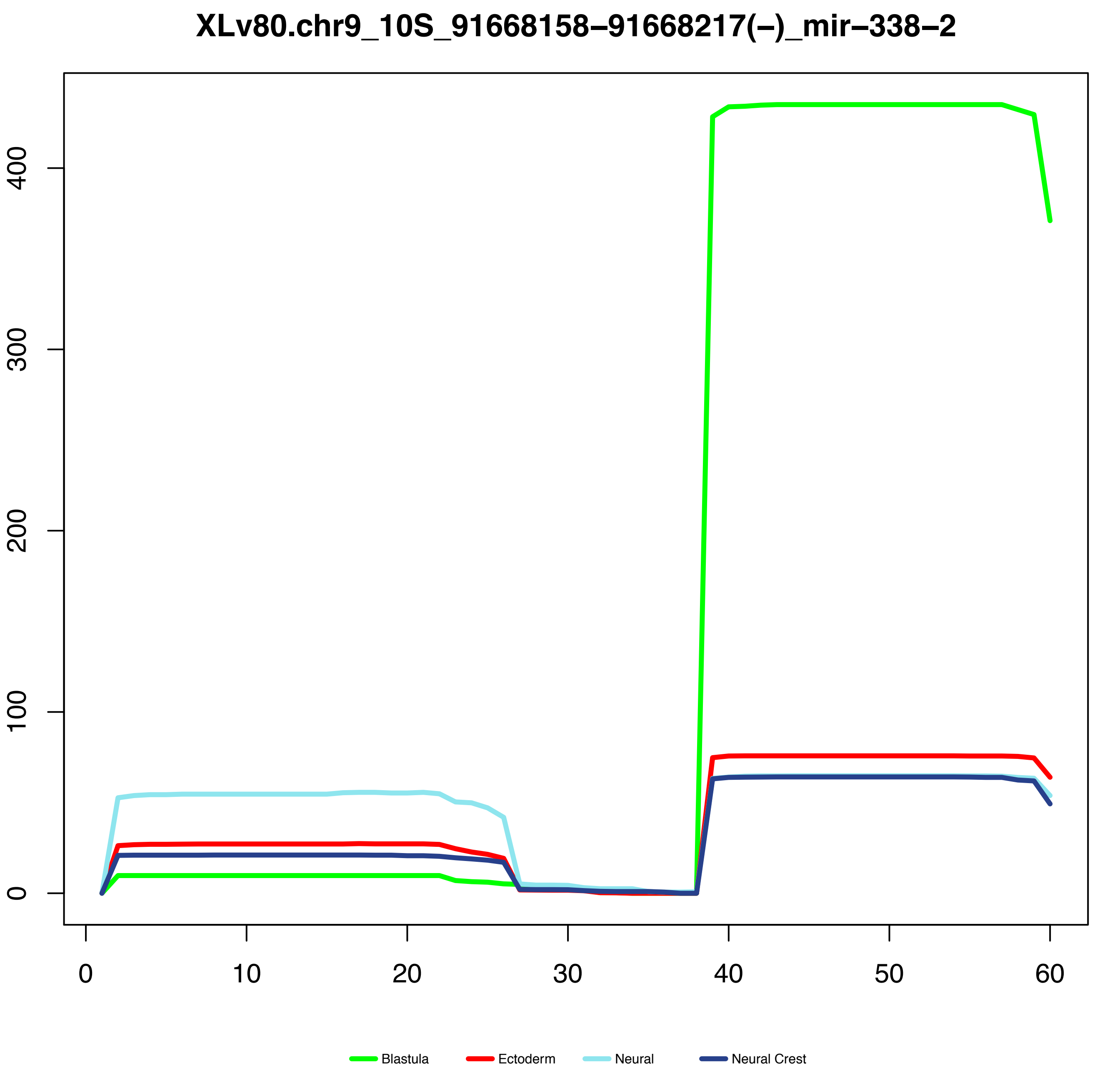
XLv80.chr9_10S_91668158−91668217(−)_mir−338−2
0
100
200
300
400
0
10
20
30
40
50
60
Blastula
Ectoderm
Neural
Neural Crest

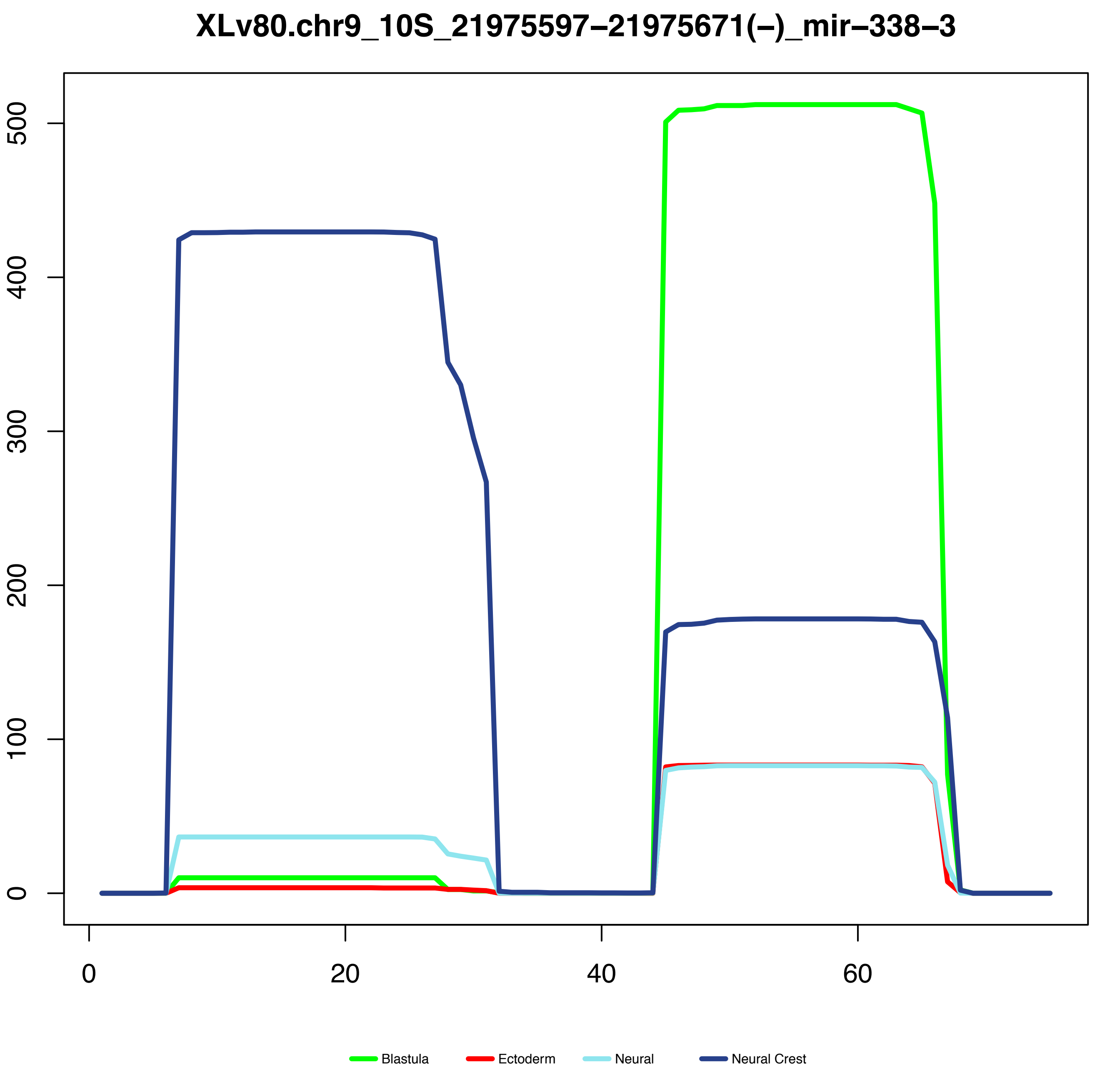
XLv80.chr9_10S_21975597−21975671(−)_mir−338−3
0
100
200
300
400
500
0
20
40
60
Blastula
Ectoderm
Neural
Neural Crest

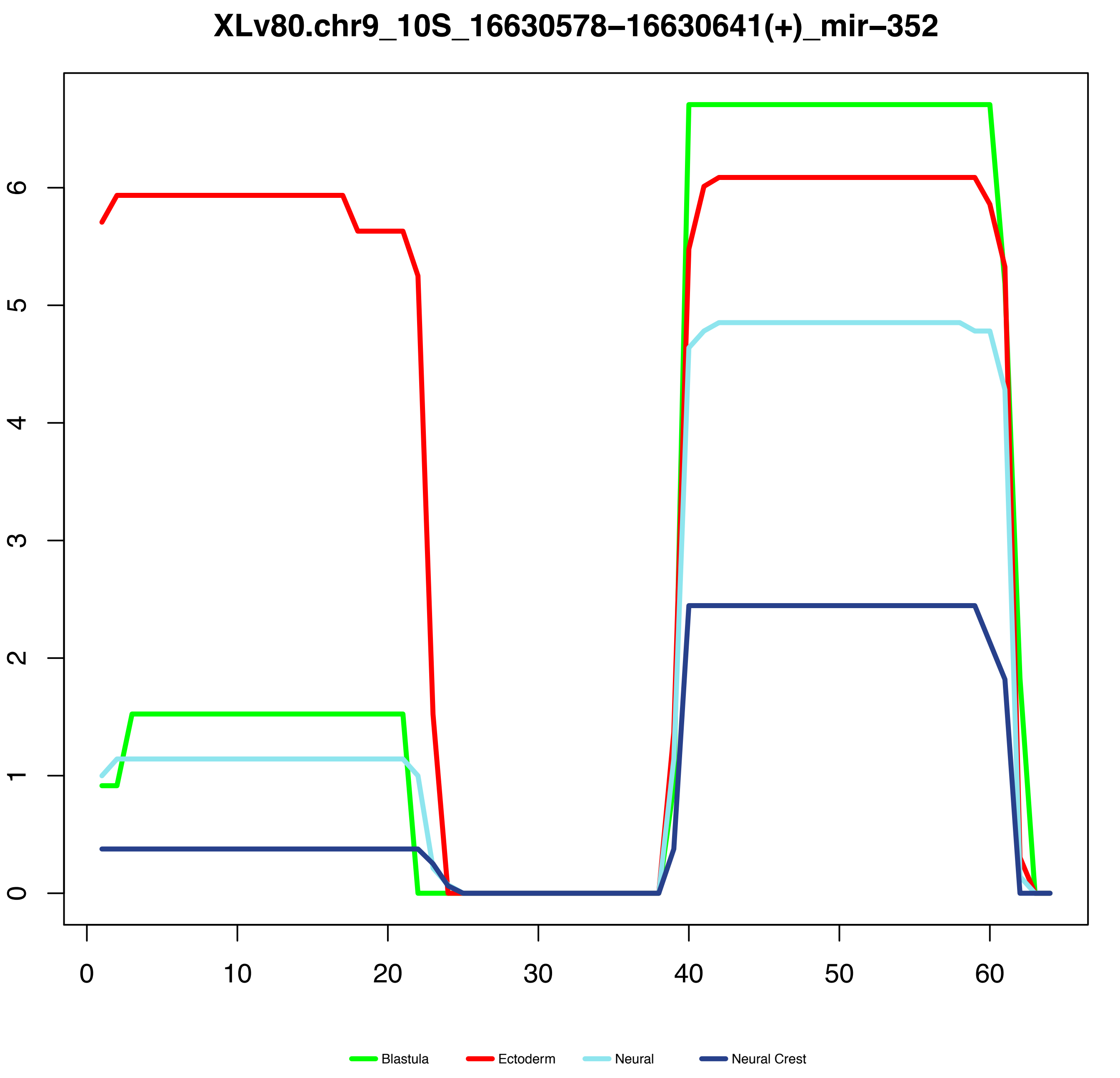
XLv80.chr9_10S_16630578−16630641(+)_mir−352
0
1
2
3
4
5
6
0
10
20
30
40
50
60
Blastula
Ectoderm
Neural
Neural Crest

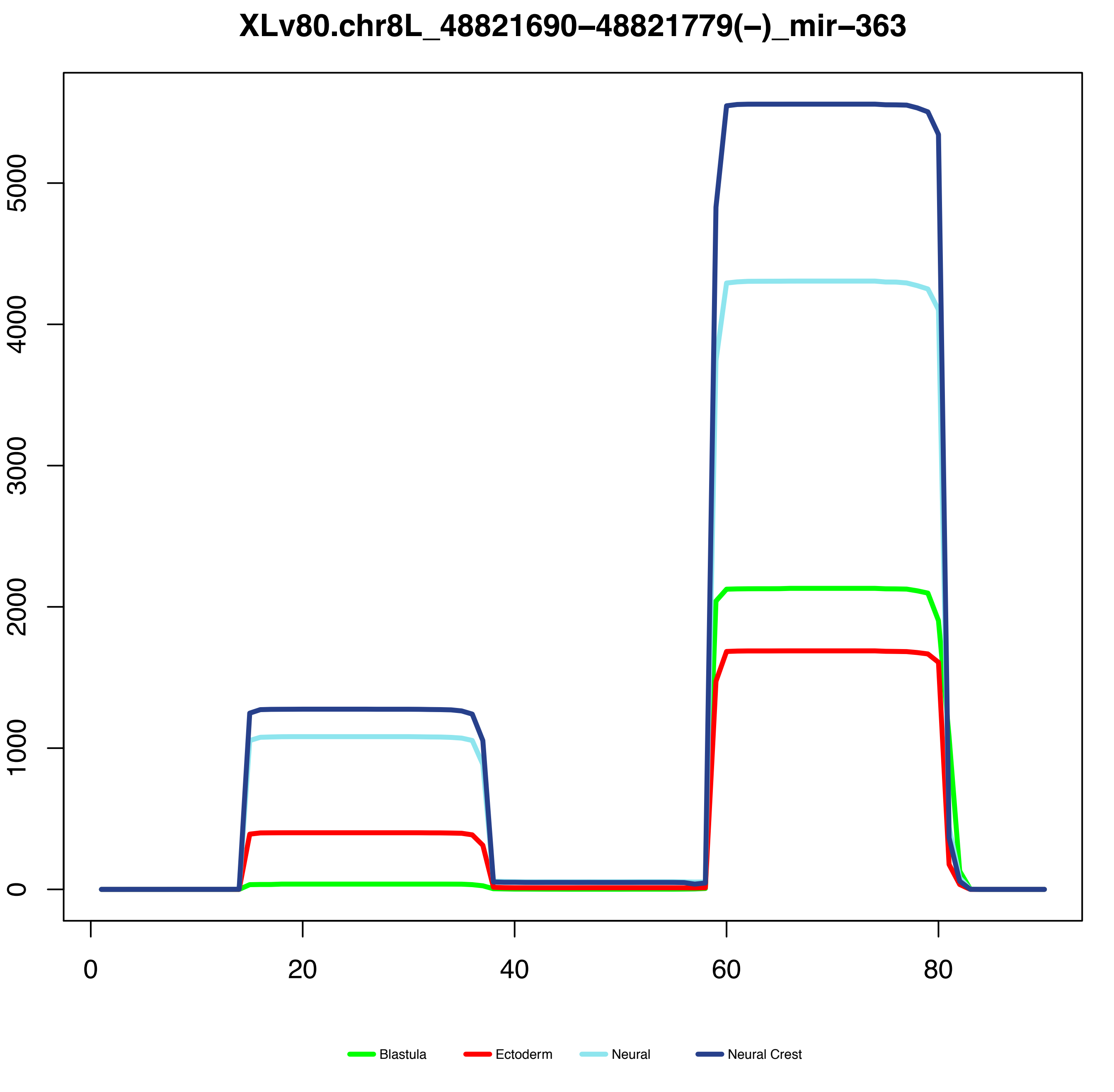

XLv80.chr8L_48821690−48821779(−)_mir−363
0
1000
2000
3000
4000
5000
0
20
40
60
80
Blastula
Ectoderm
Neural
Neural Crest

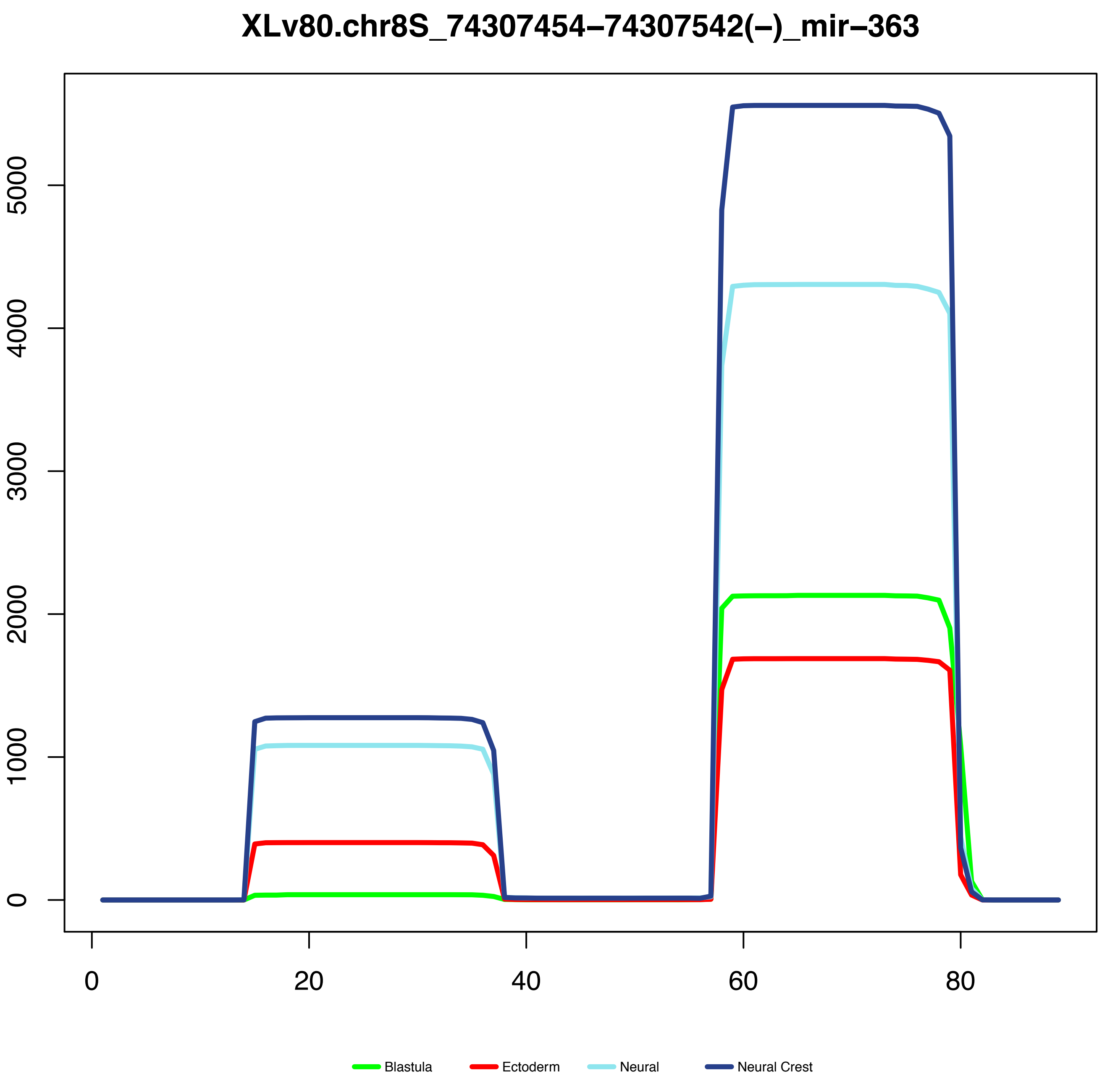
XLv80.chr8S_74307454−74307542(−)_mir−363
0
1000
2000
3000
4000
5000
0
20
40
60
80
Blastula
Ectoderm
Neural
Neural Crest

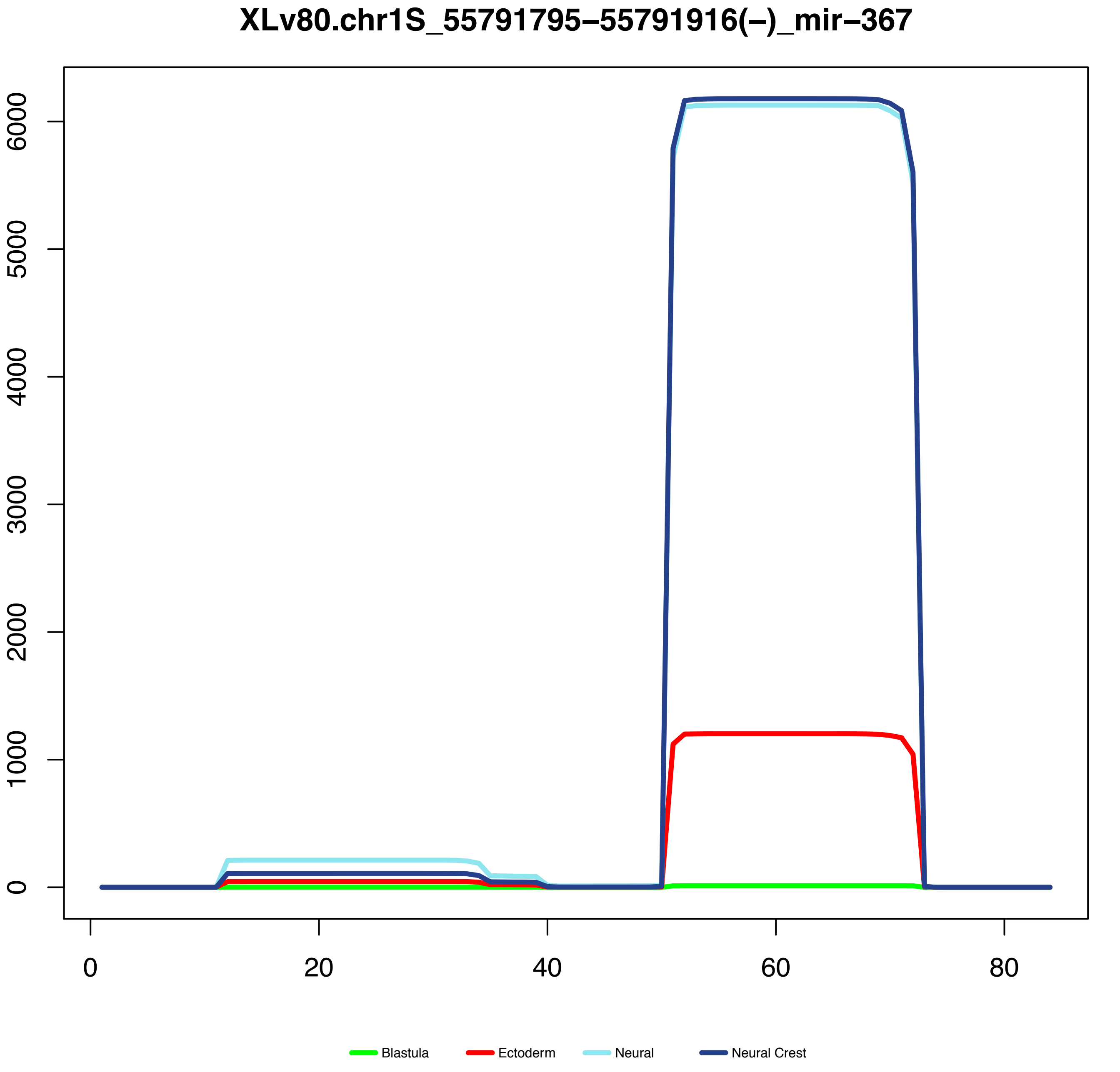

XLv80.chr1S_55791795−55791916(−)_mir−367
0
1000
2000
3000
4000
5000
6000
0
20
40
60
80
Blastula
Ectoderm
Neural
Neural Crest

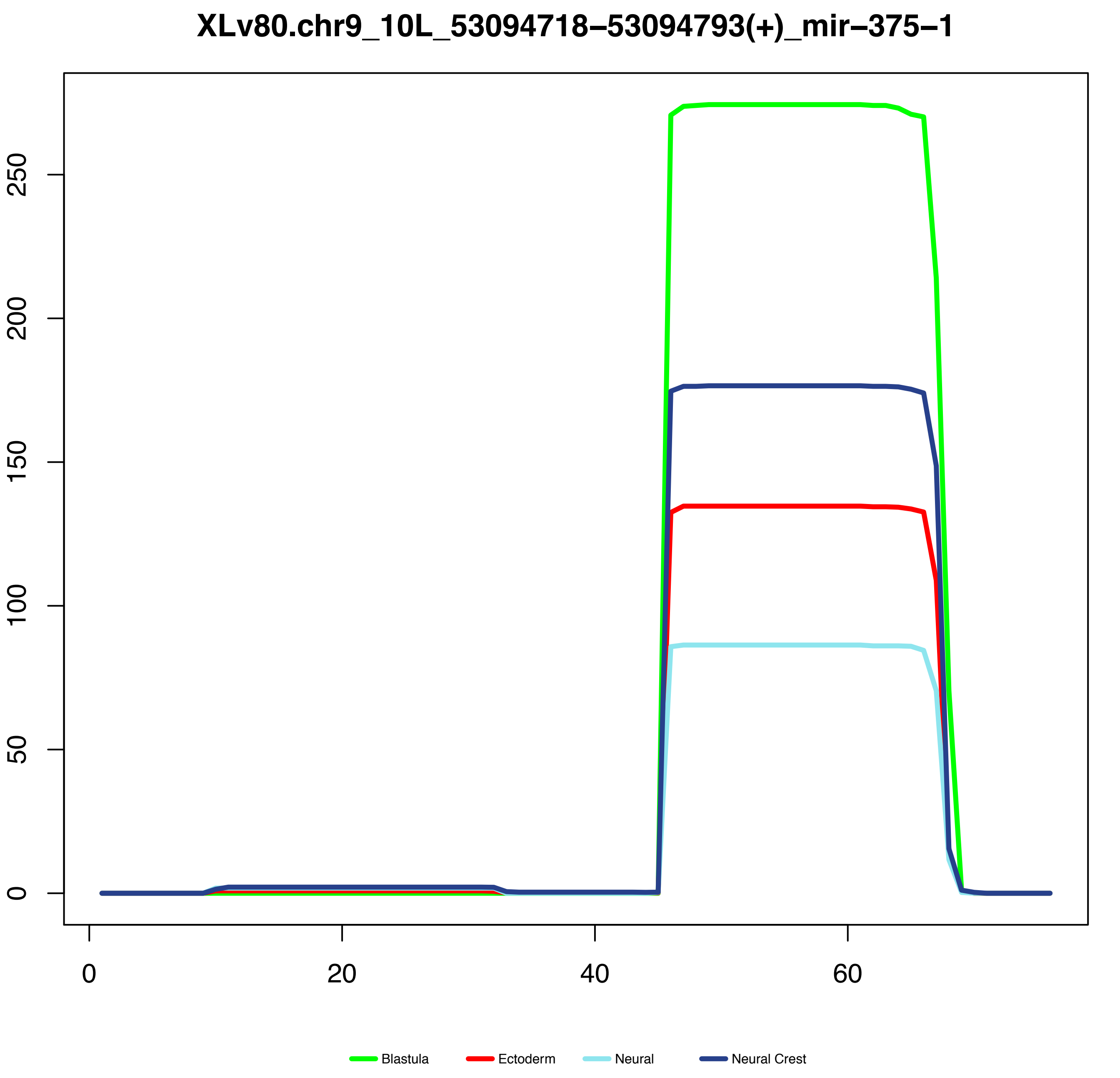

XLv80.chr9_10L_53094718–53094793(+)_mir–375–1
0
50
100
150
200
250
0
20
40
60
Blastula
Ectoderm
Neural
Neural Crest

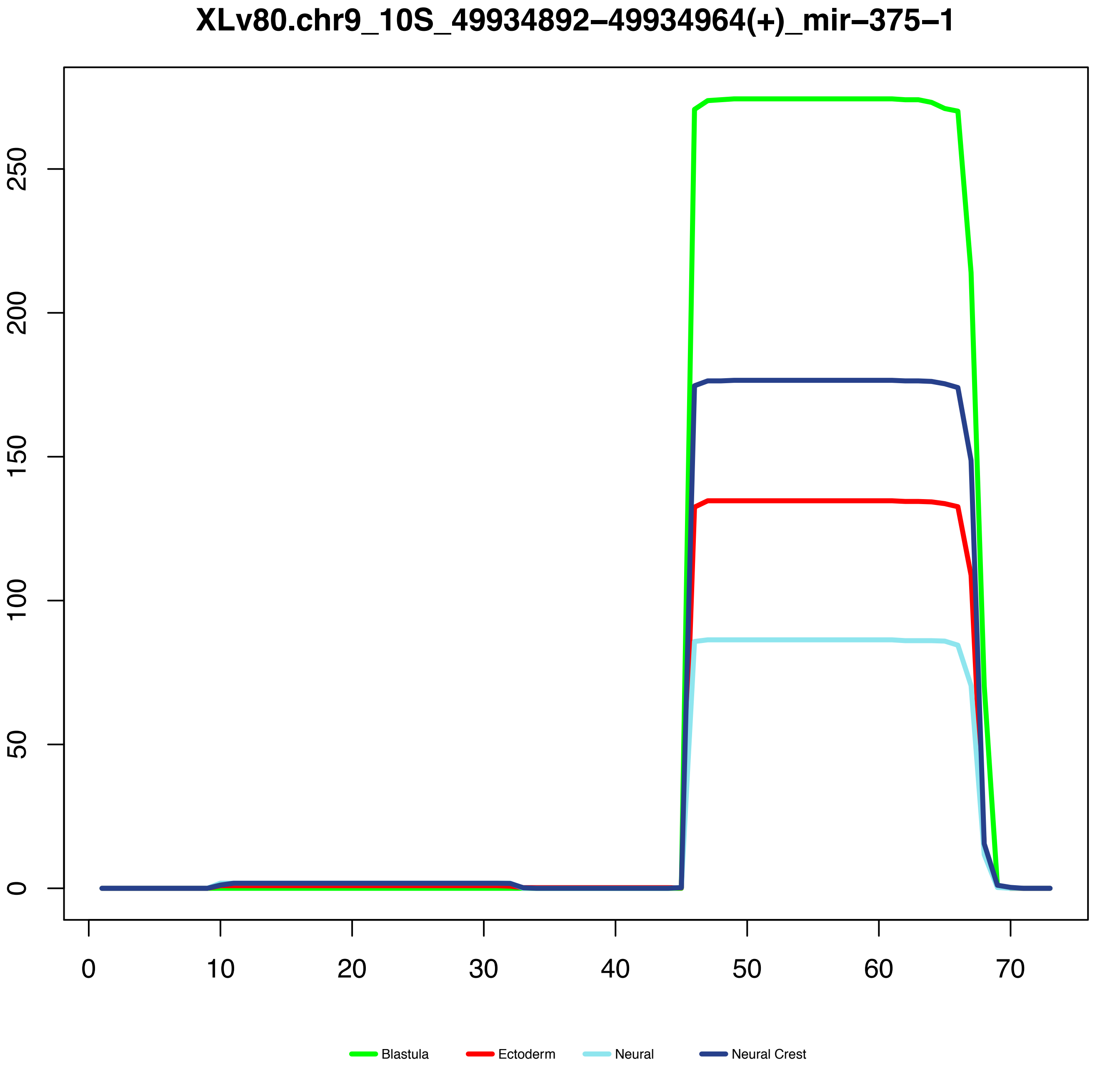
XLv80.chr9_10S_49934892−49934964(+)_mir−375−1
0
50
100
150
200
250
0
10
20
30
40
50
60
70
Blastula
Ectoderm
Neural
Neural Crest

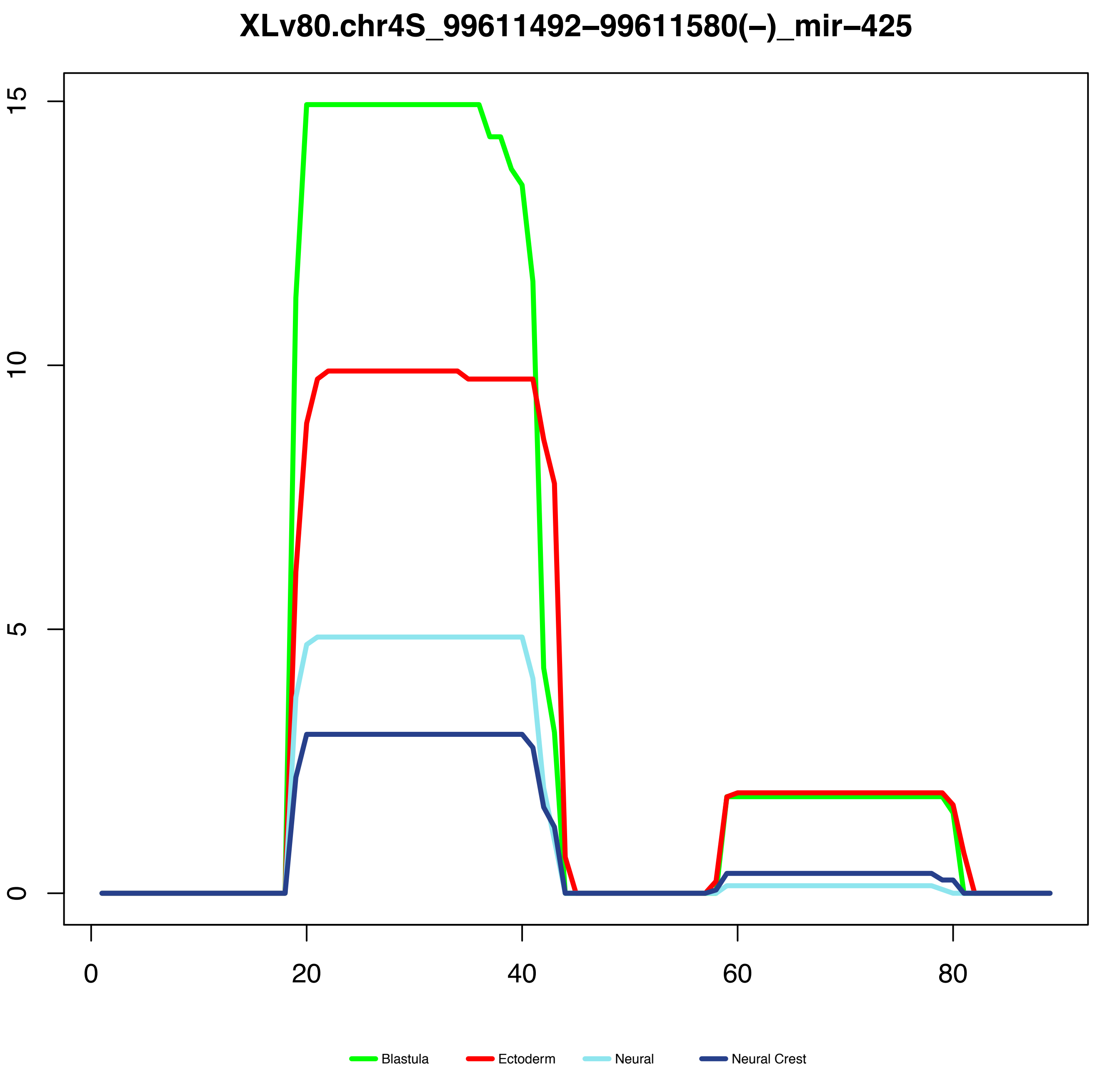

XLv80.chr4S_99611492−99611580(−)_mir−425
0
5
10
15
0
20
40
60
80
Blastula
Ectoderm
Neural
Neural Crest

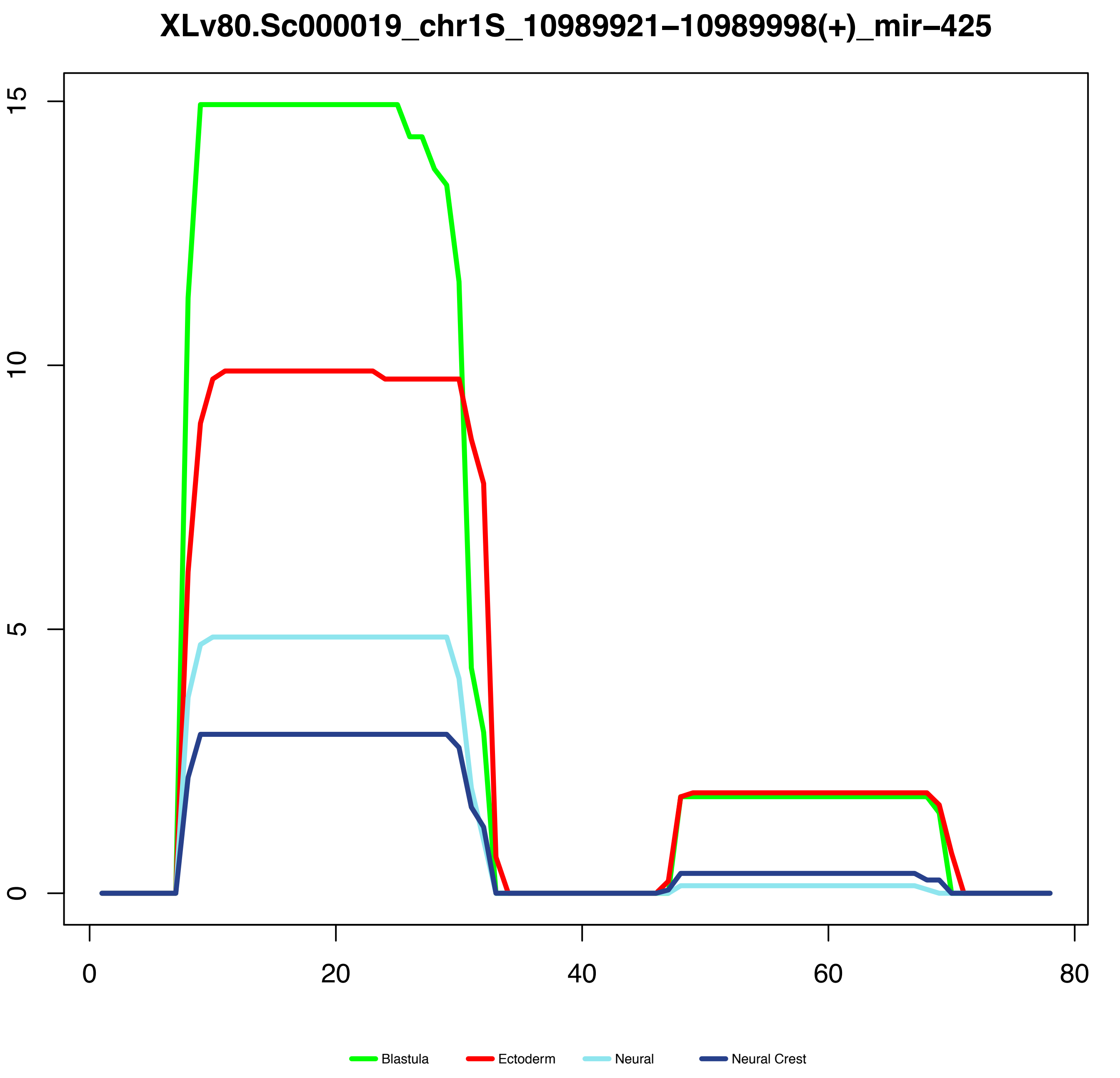
XLv80.Sc000019_chr1S_10989921−10989998(+)_mir−425
0
5
10
15
0
20
40
60
80
Blastula
Ectoderm
Neural
Neural Crest

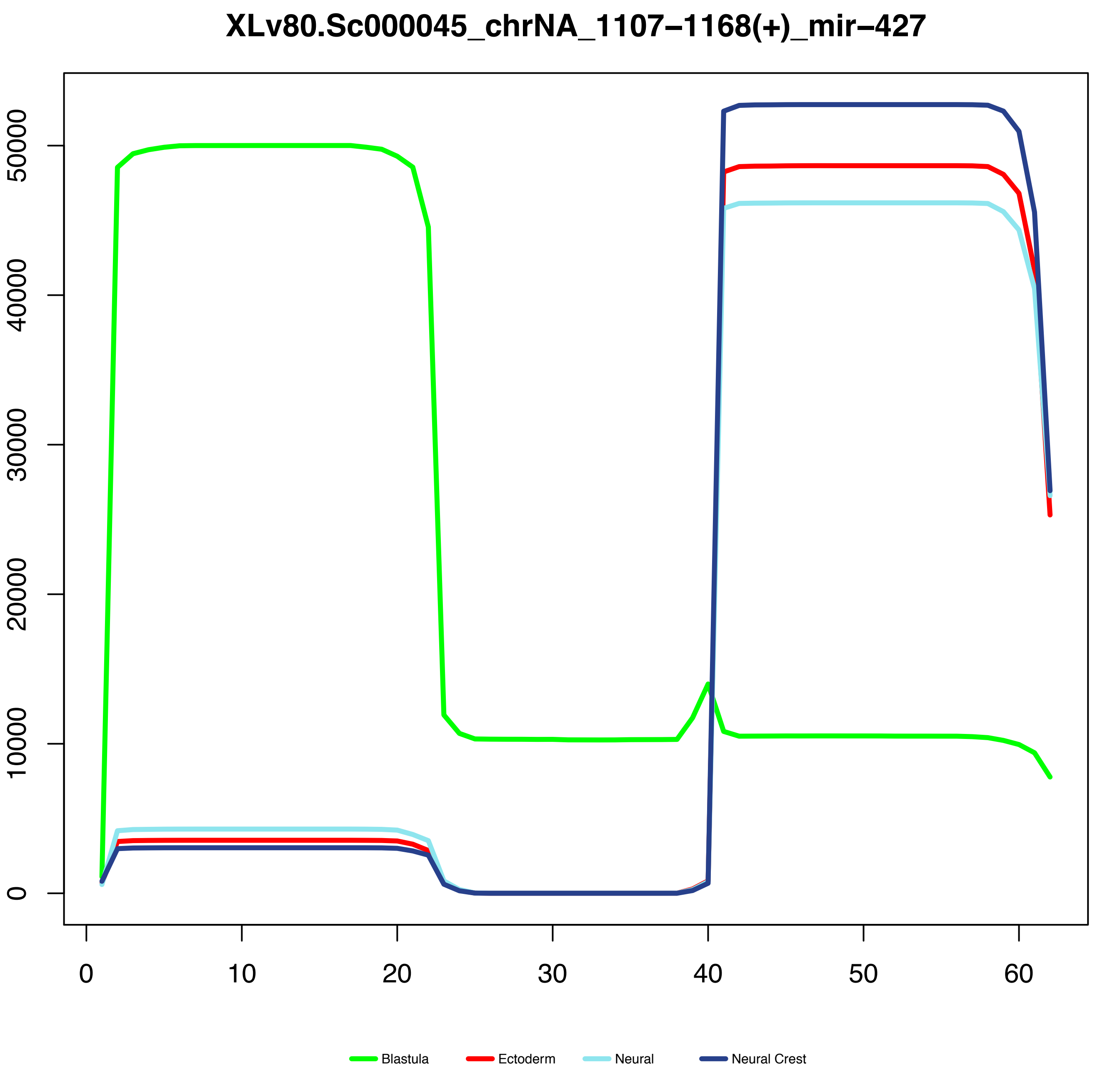
XLv80.Sc000045_chrNA_1107−1168(+)_mir−427
0
10000
20000
30000
40000
50000
0
10
20
30
40
50
60
Blastula
Ectoderm
Neural
Neural Crest

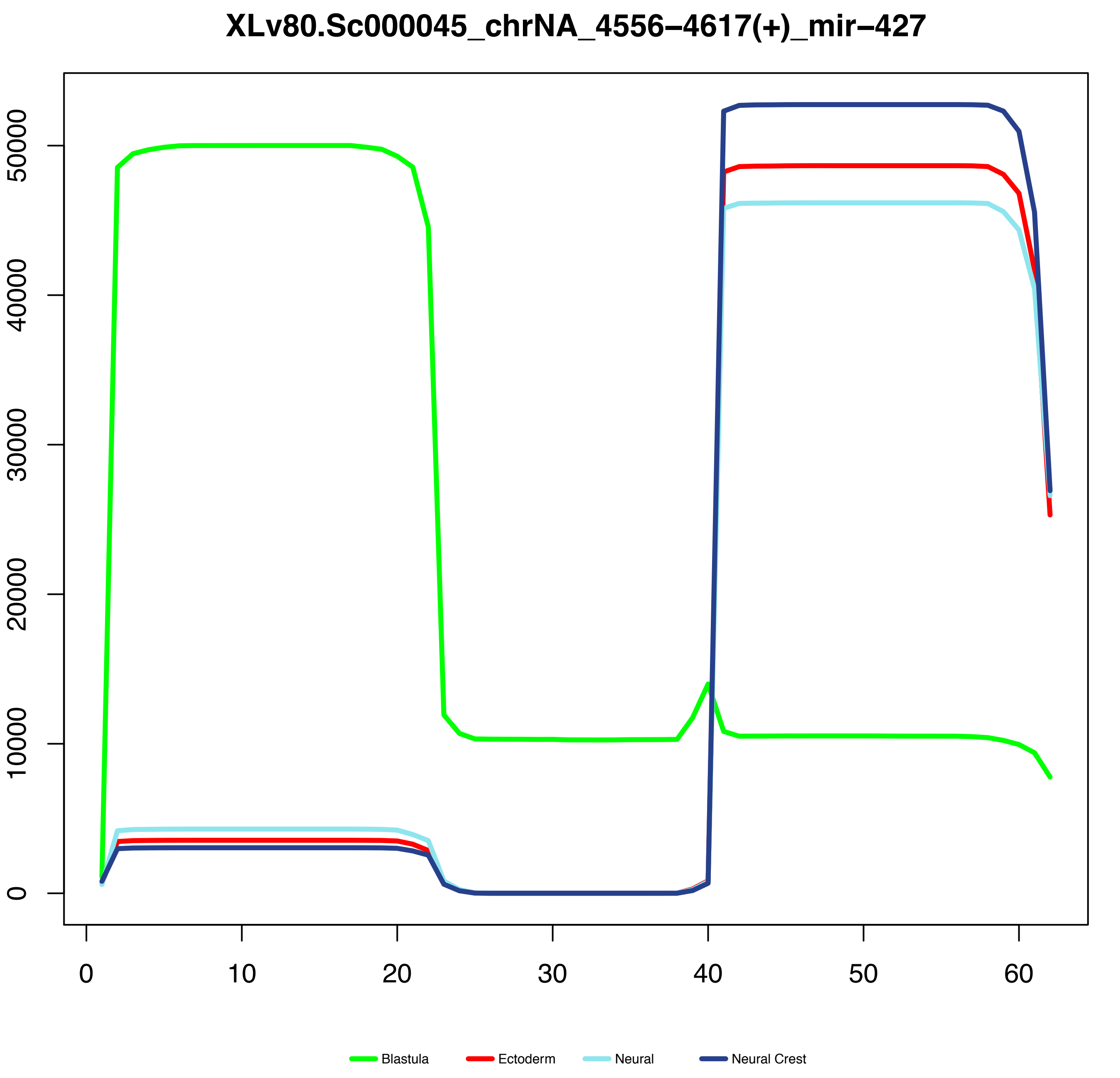
XLv80.Sc000045_chrNA_4556–4617(+)_mir–427
0
10000
20000
30000
40000
50000
0
10
20
30
40
50
60
Blastula
Ectoderm
Neural
Neural Crest

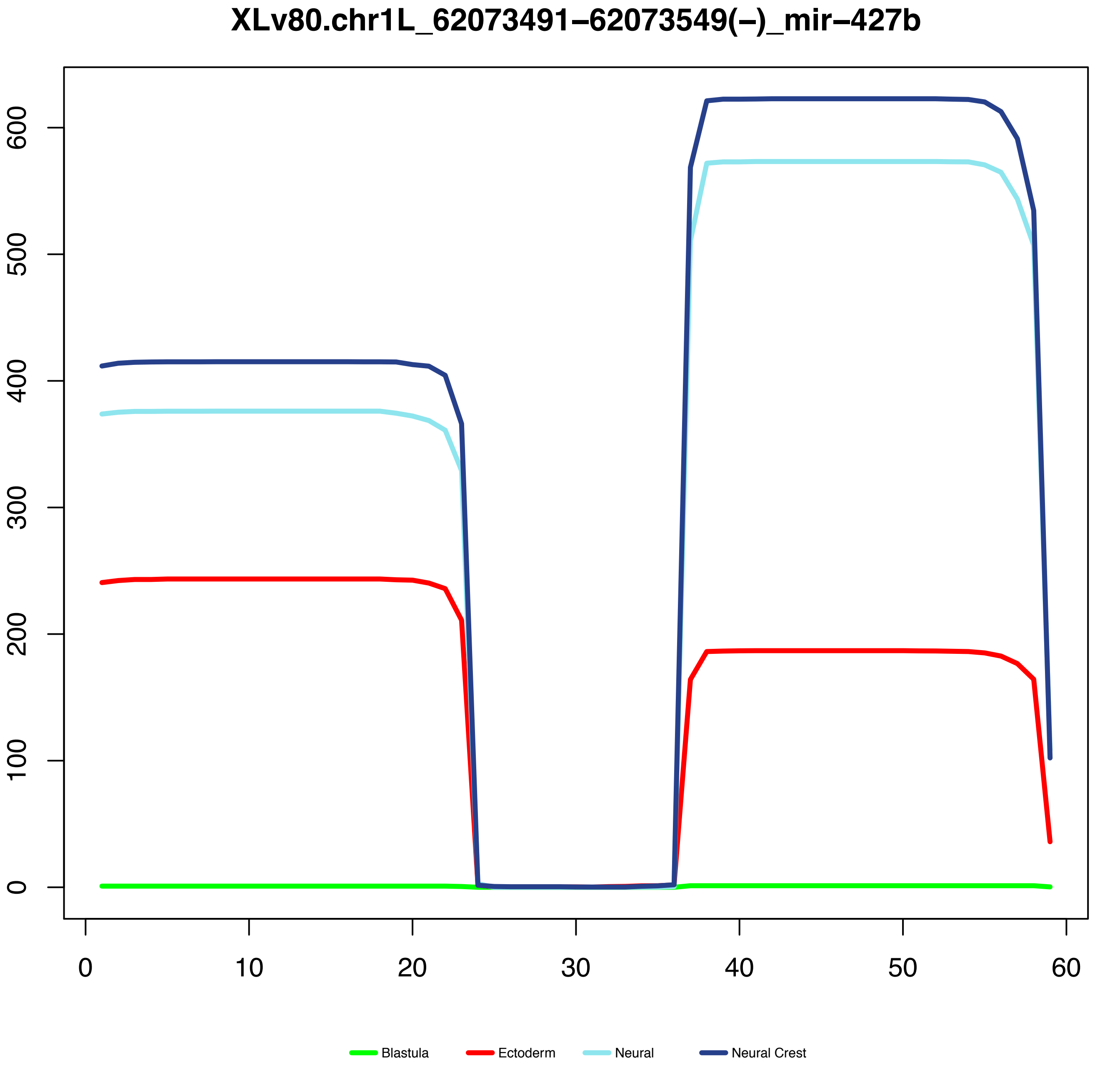

XLv80.chr1L_62073491−62073549(−)_mir−427b
0
100
200
300
400
500
600
0
10
20
30
40
50
60
Blastula
Ectoderm
Neural
Neural Crest

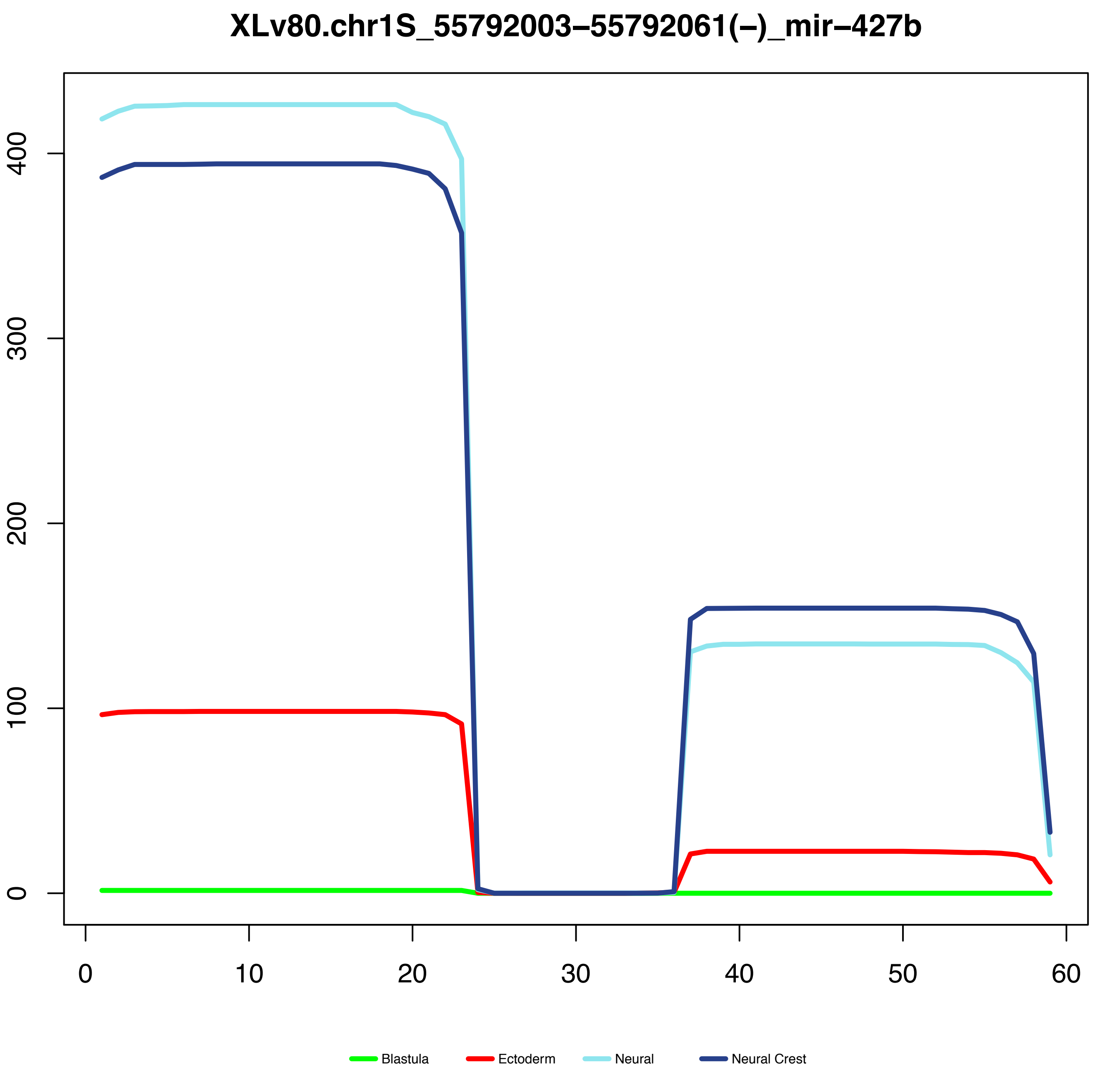

XLv80.chr1S_55792003−55792061(−)_mir−427b
0
100
200
300
400
0
10
20
30
40
50
60
Blastula
Ectoderm
Neural
Neural Crest

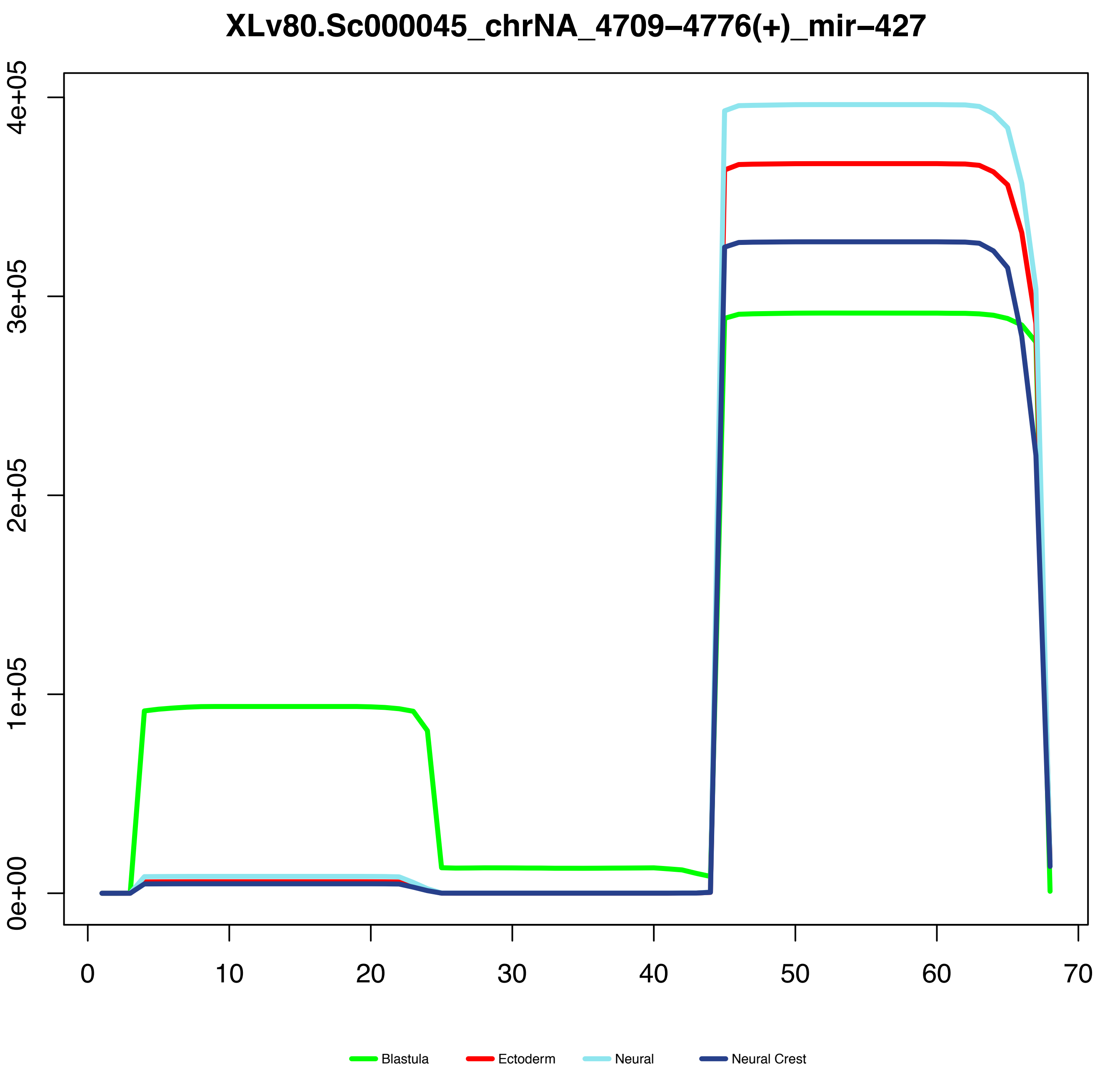
XLv80.Sc000045_chrNA_4709−4776(+)_mir−427
0e+00
1e+05
2e+05
3e+05
4e+05
0
10
20
30
40
50
60
70
Blastula
Ectoderm
Neural
Neural Crest

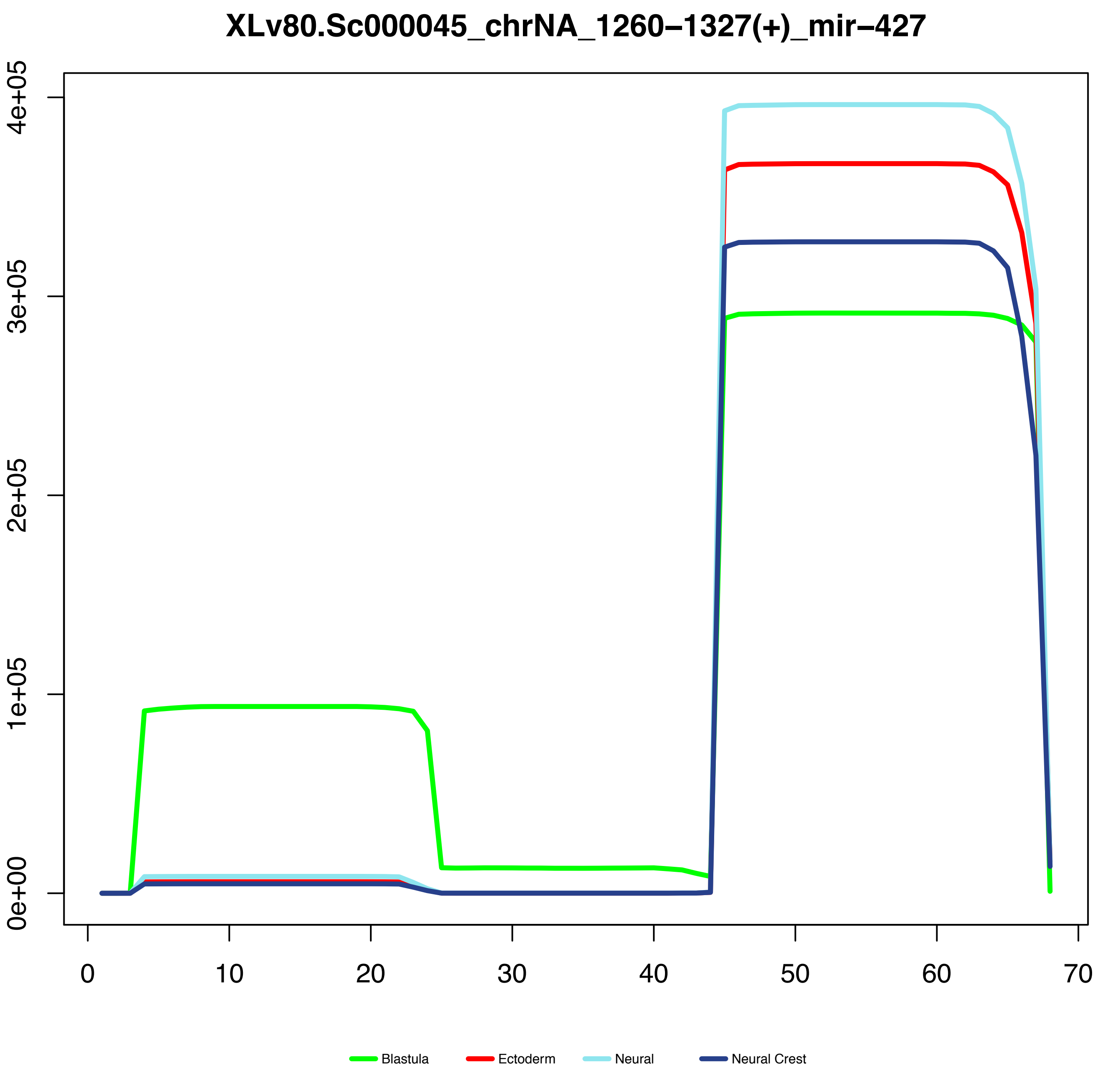
XLv80.Sc000045_chrNA_1260−1327(+)_mir−427
0e+00
1e+05
2e+05
3e+05
4e+05
0
10
20
30
40
50
60
70
Blastula
Ectoderm
Neural
Neural Crest

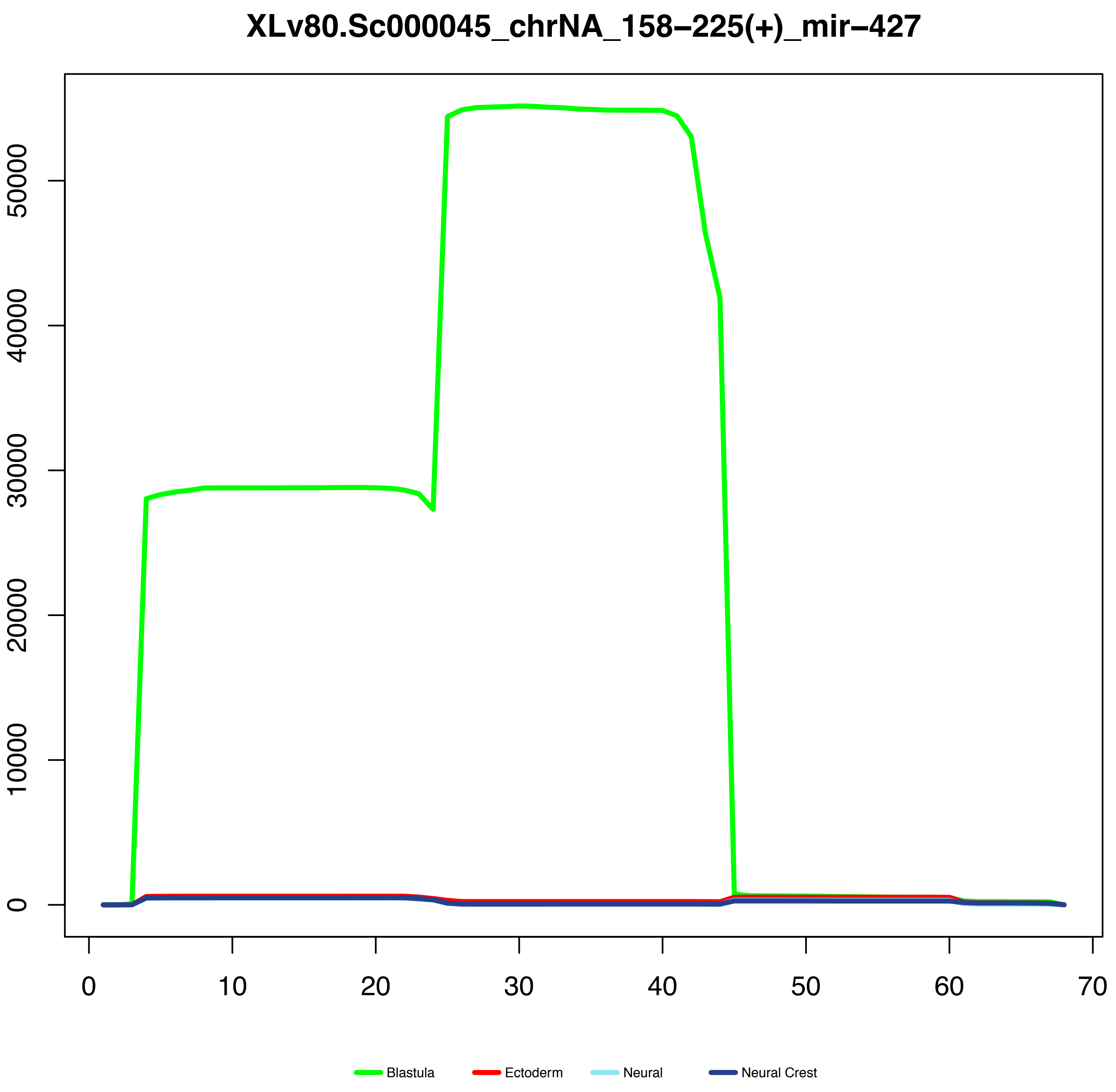
XLv80.Sc000045_chrNA_158−225(+)_mir−427
0
10000
20000
30000
40000
50000
0
10
20
30
40
50
60
70
Blastula
Ectoderm
Neural
Neural Crest

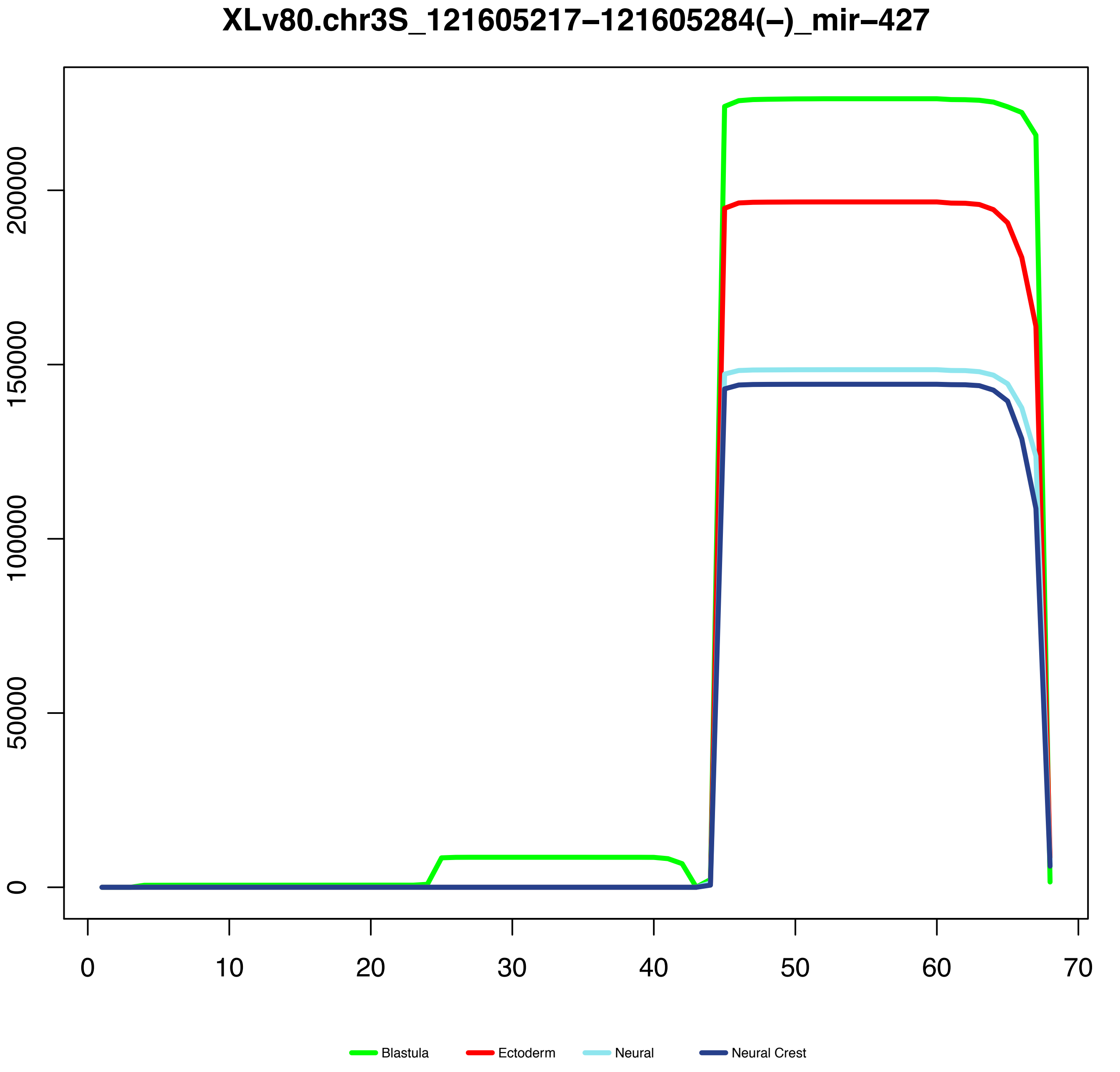

XLv80.chr3S_121605217−121605284(−)_mir−427
0
50000
100000
150000
200000
0
10
20
30
40
50
60
70
Blastula
Ectoderm
Neural
Neural Crest

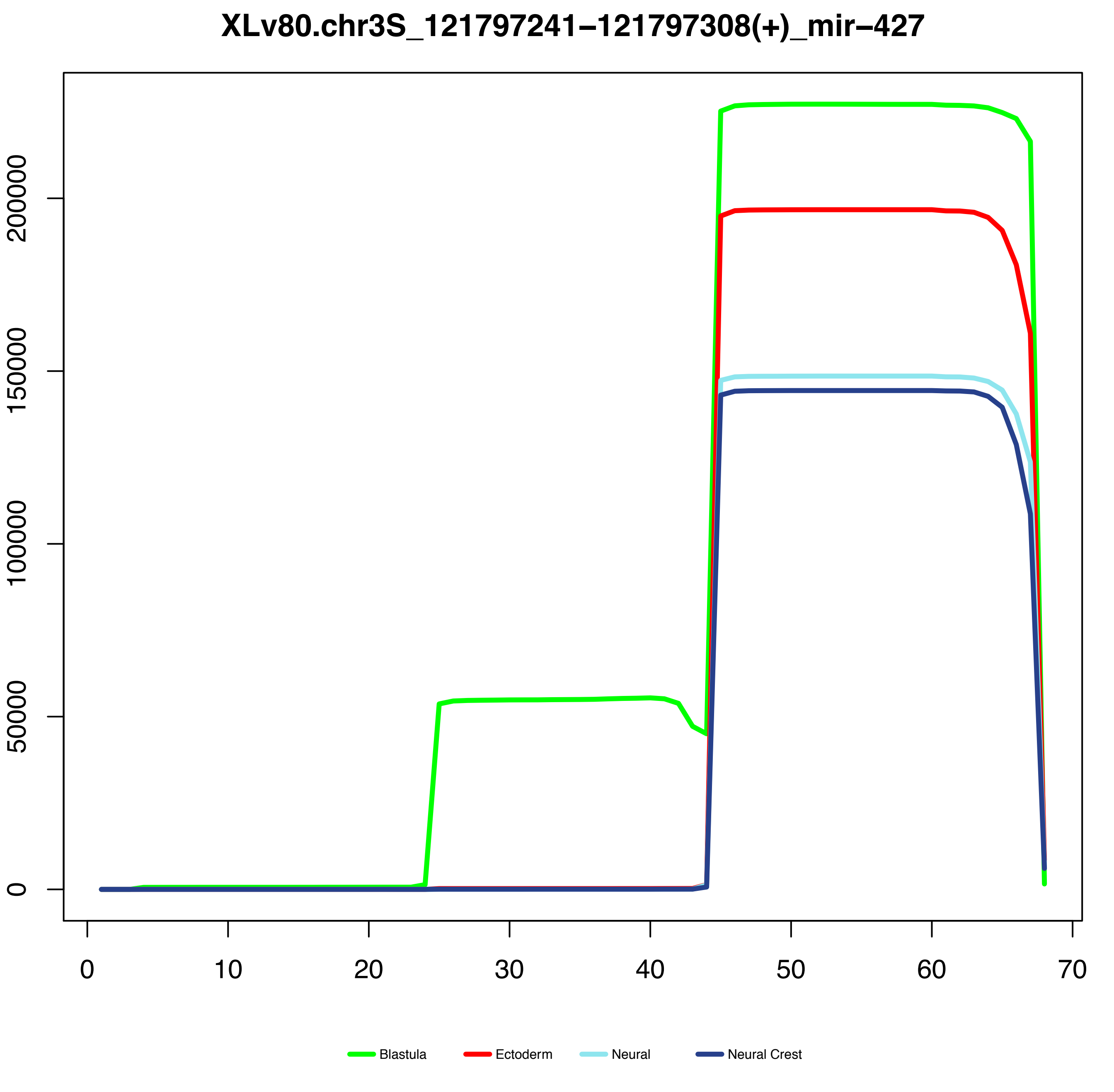
XLv80.chr3S_121797241−121797308(+)_mir−427
0
50000
100000
150000
200000
0
10
20
30
40
50
60
70
Blastula
Ectoderm
Neural
Neural Crest

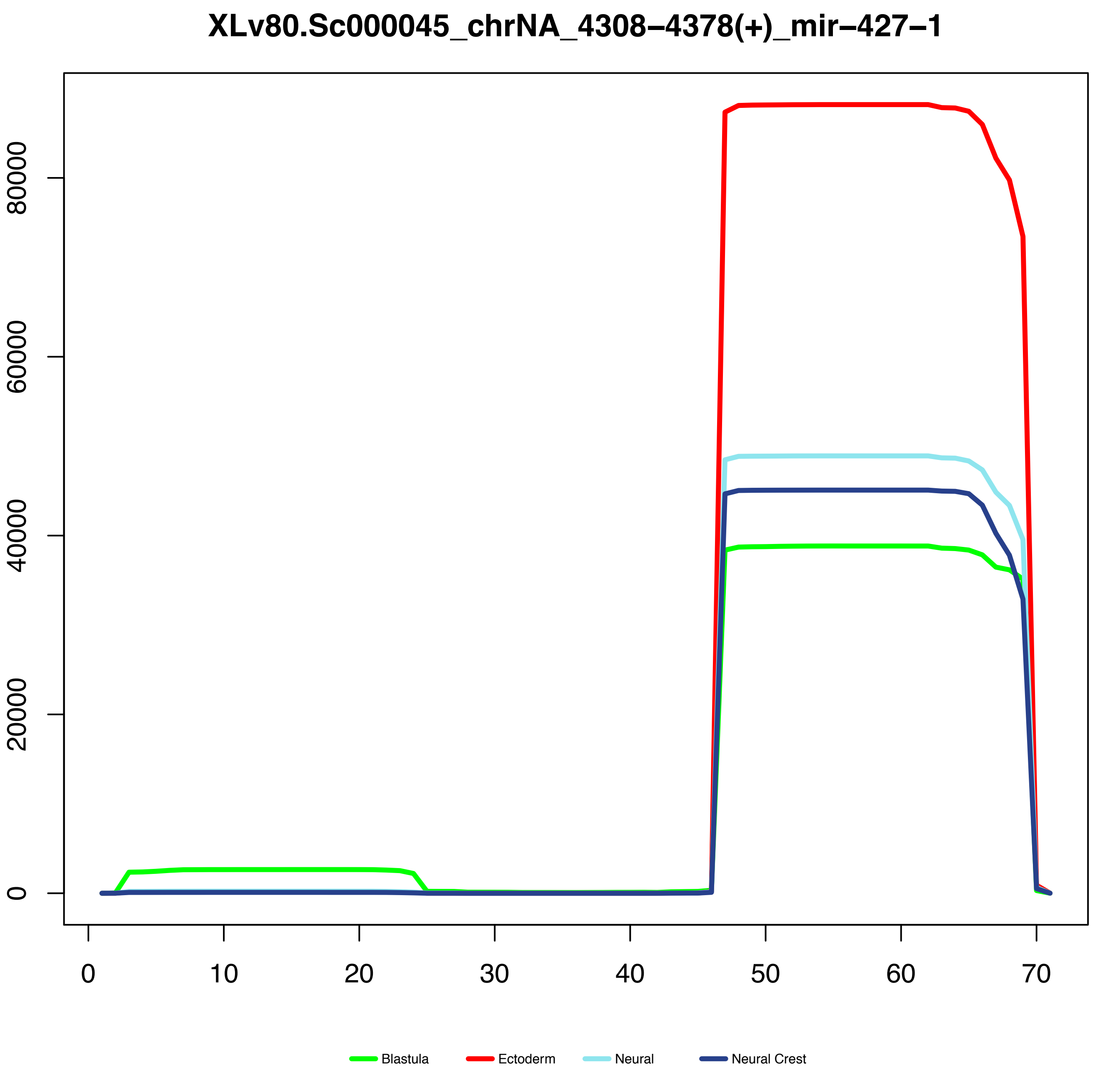

XLv80.Sc000045_chrNA_4308−4378(+)_mir−427−1
0
20000
40000
60000
80000
0
10
20
30
40
50
60
70
Blastula
Ectoderm
Neural
Neural Crest

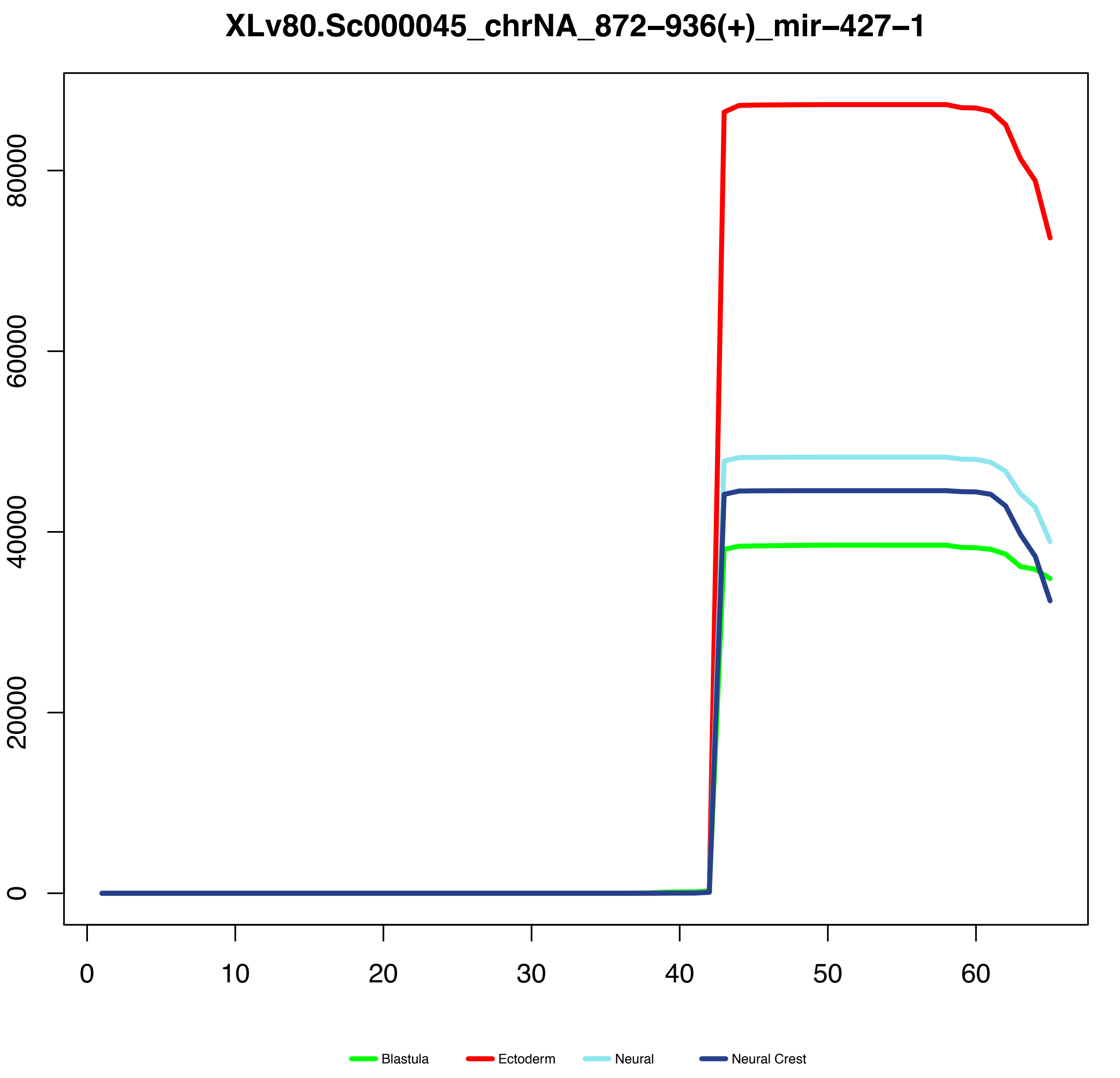
XLv80.Sc000045_chrNA_872−936(+)_mir−427−1
0
20000
40000
60000
80000
0
10
20
30
40
50
60
Blastula
Ectoderm
Neural
Neural Crest

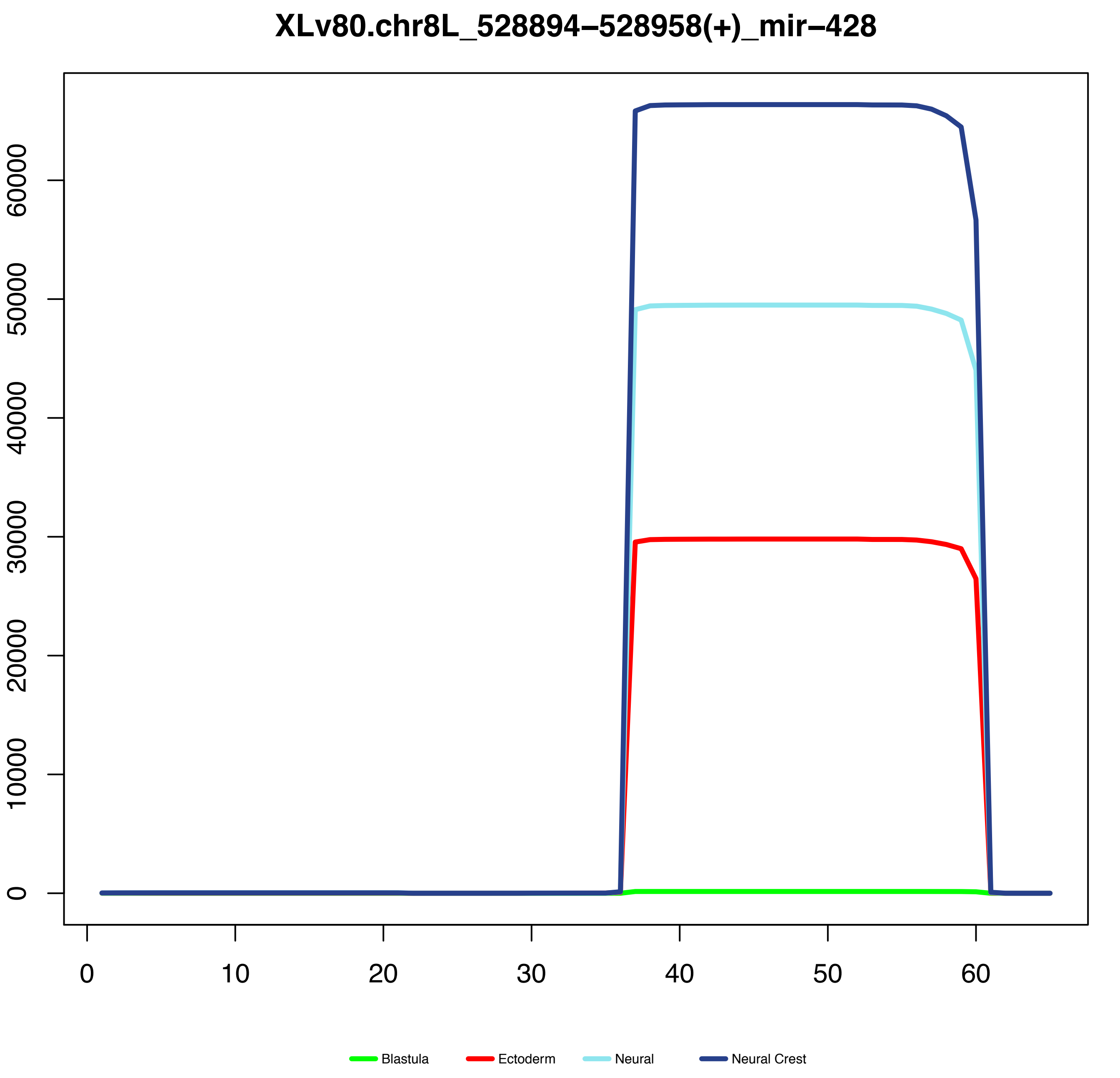

XLv80.chr8L_528894−528958(+)_mir−428
0
10000
20000
30000
40000
50000
60000
0
10
20
30
40
50
60
Blastula
Ectoderm
Neural
Neural Crest

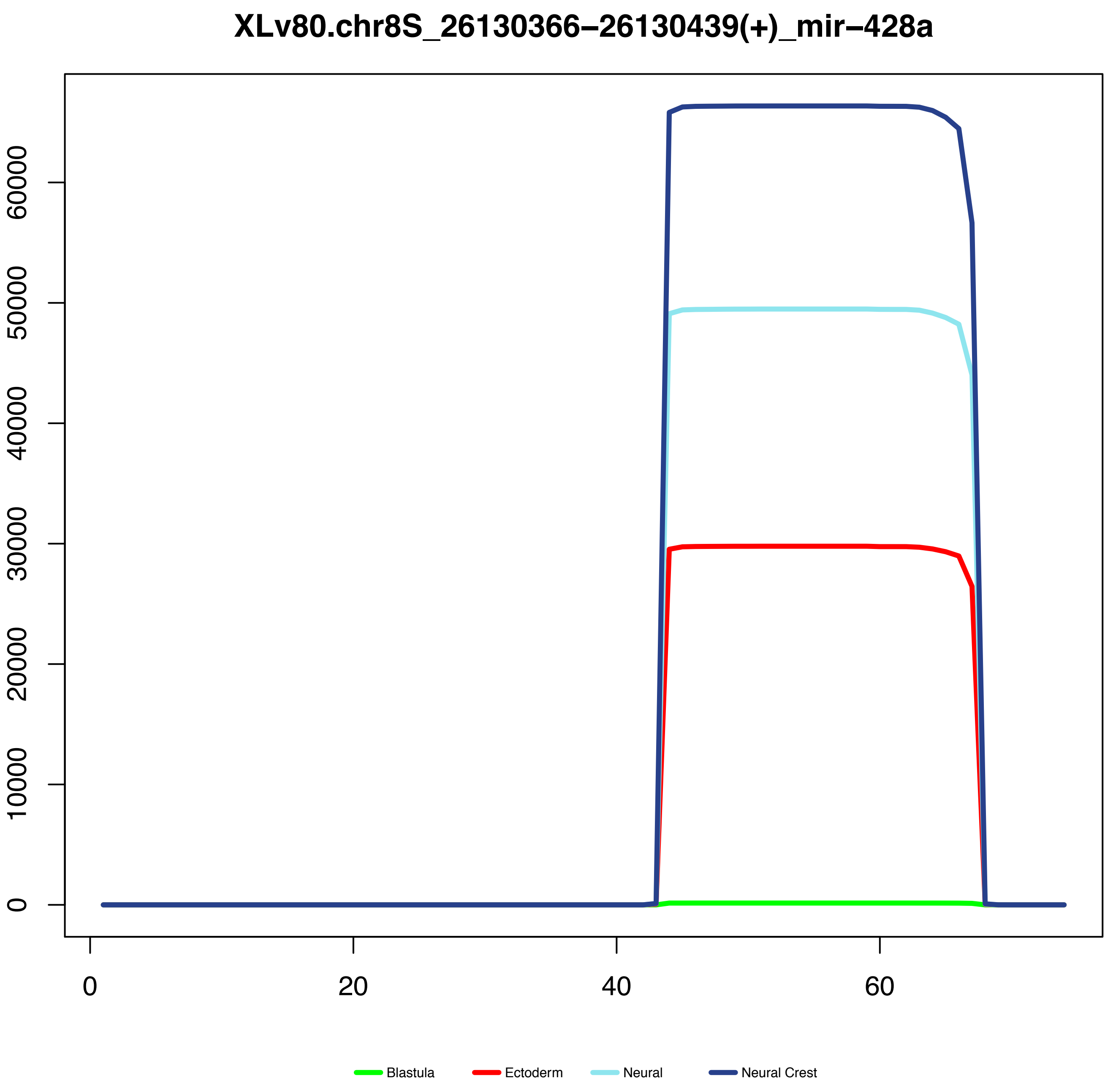
XLv80.chr8S_26130366−26130439(+)_mir−428a
60000
50000
40000
30000
20000
10000
0
0
20
40
60
Blastula
Ectoderm
Neural
Neural Crest

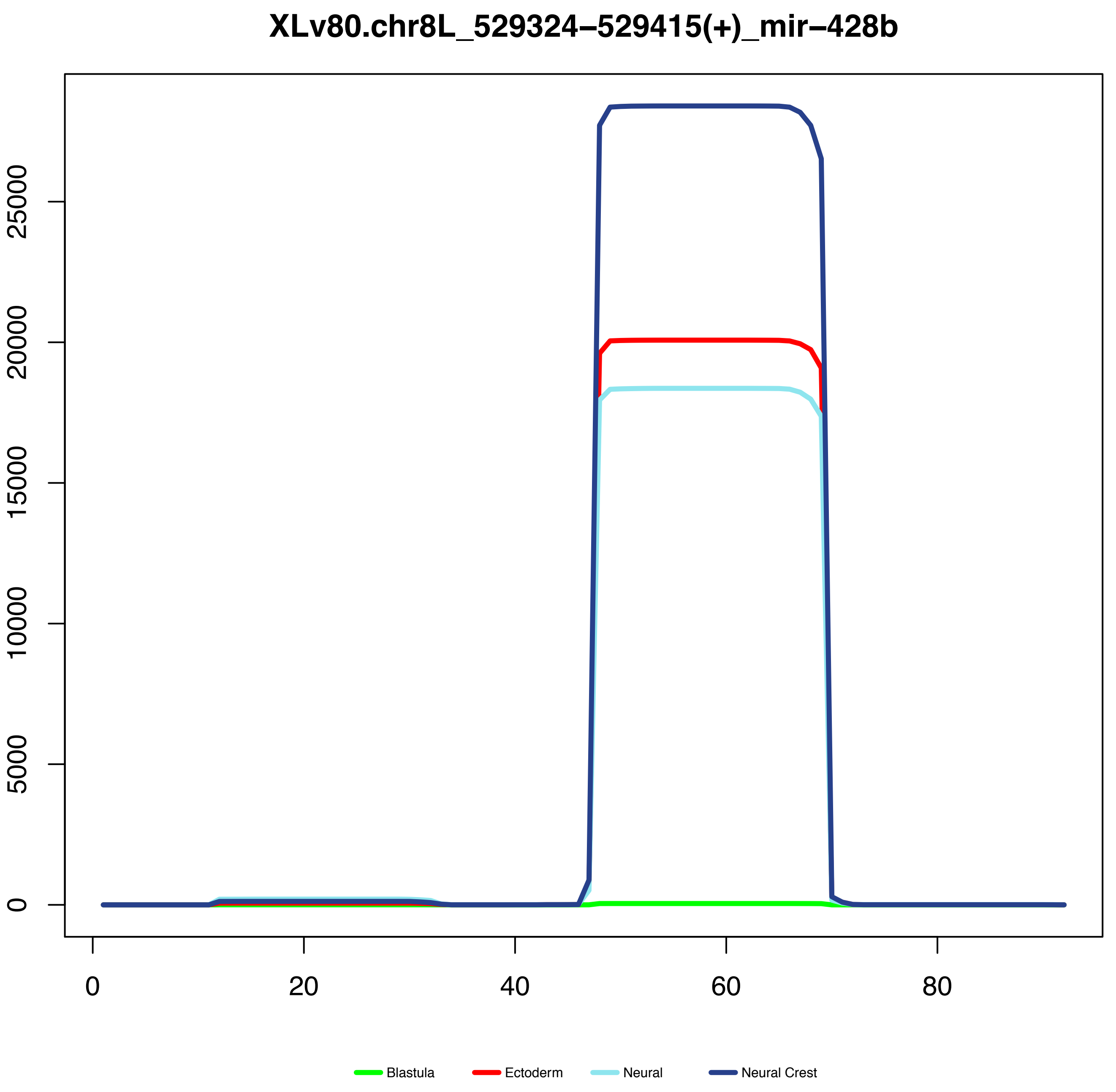

XLv80.chr8L_529324−529415(+)_mir−428b
0
5000
10000
15000
20000
25000
0
20
40
60
80
Blastula
Ectoderm
Neural
Neural Crest

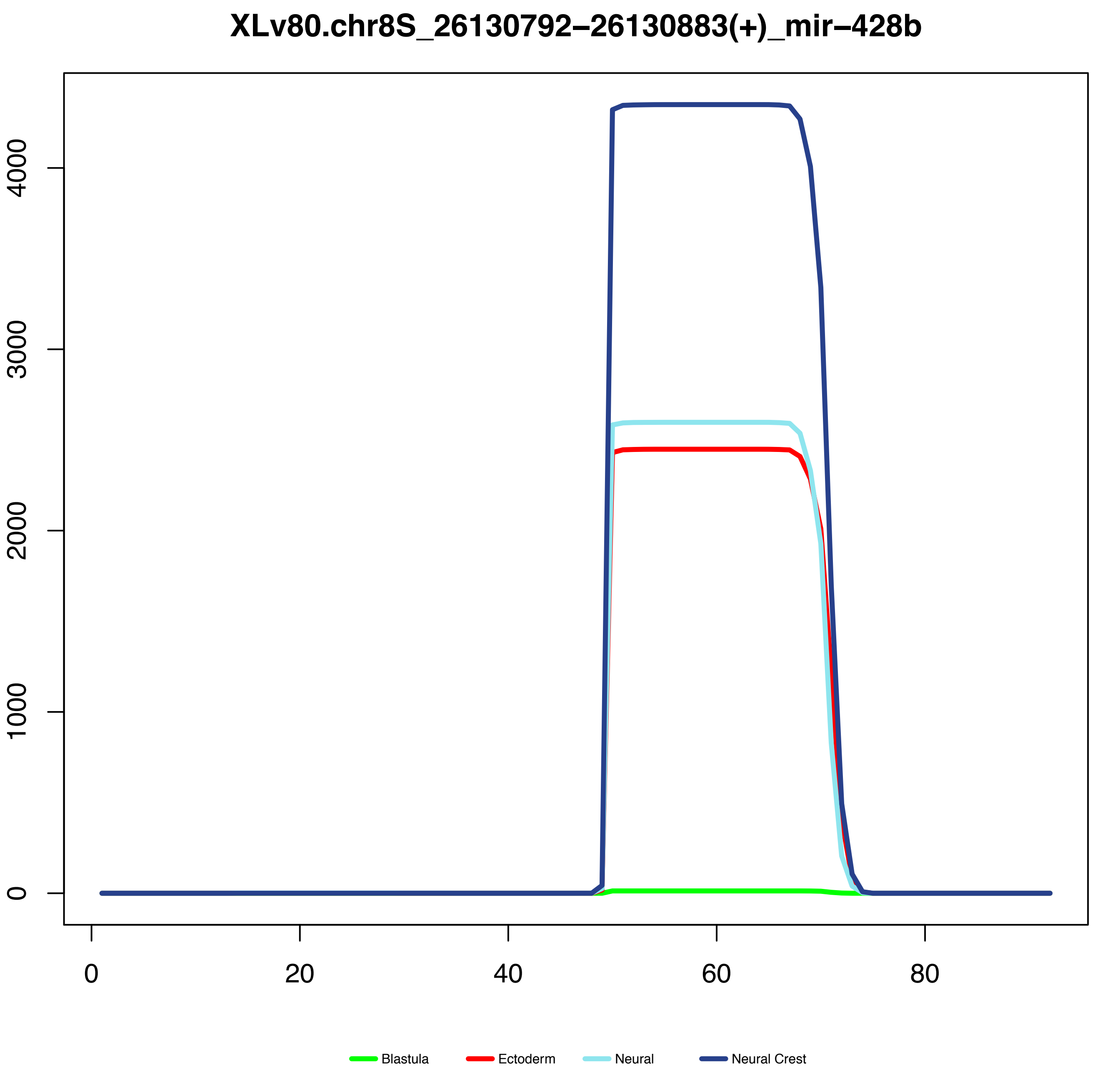

XLv80.chr8S_26130792−26130883(+)_mir−428b
0
1000
2000
3000
4000
0
20
40
60
80
Blastula
Ectoderm
Neural
Neural Crest

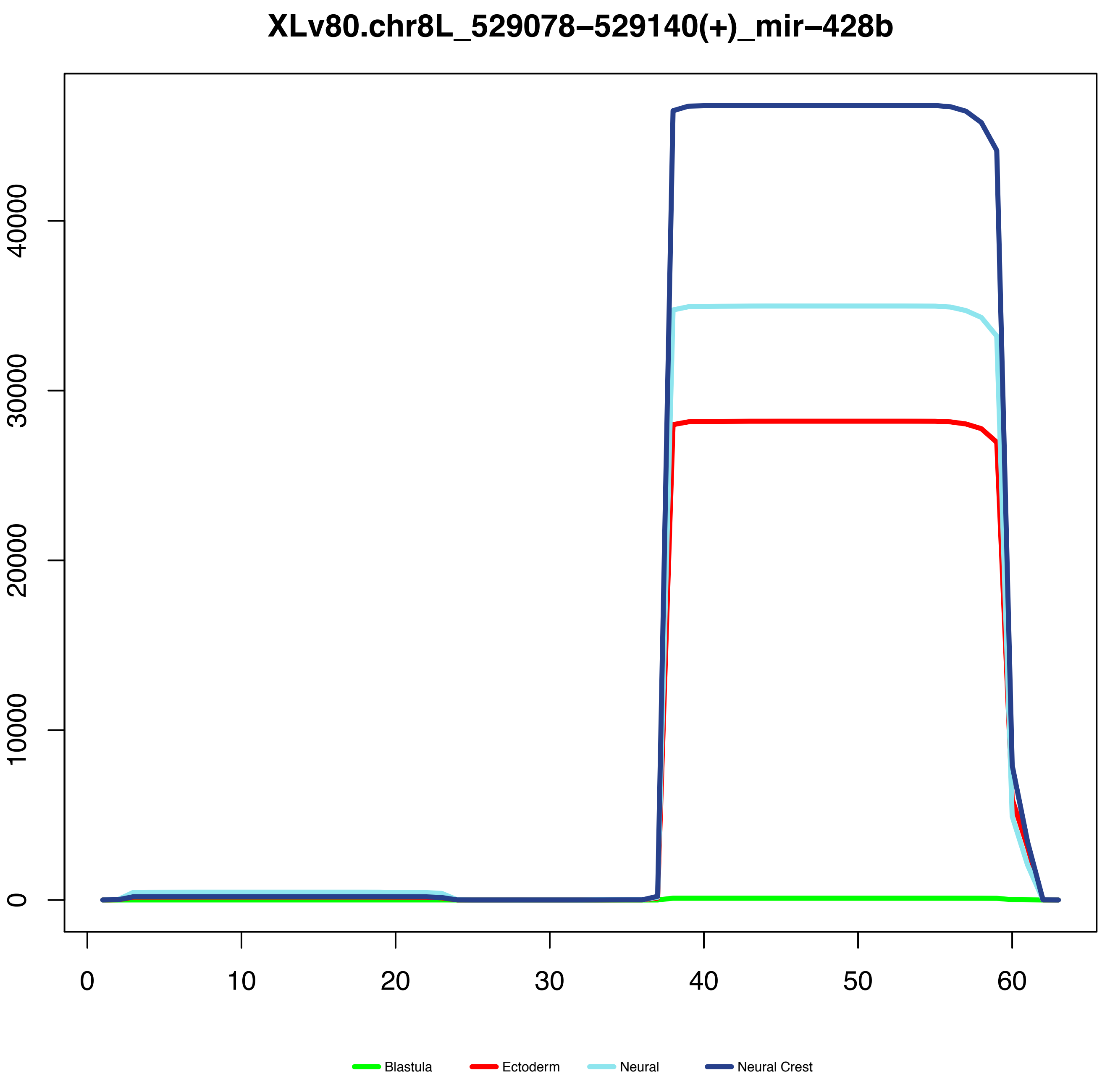
XLv80.chr8L_529078−529140(+)_mir−428b
0
10000
20000
30000
40000
0
10
20
30
40
50
60
Blastula
Ectoderm
Neural
Neural Crest

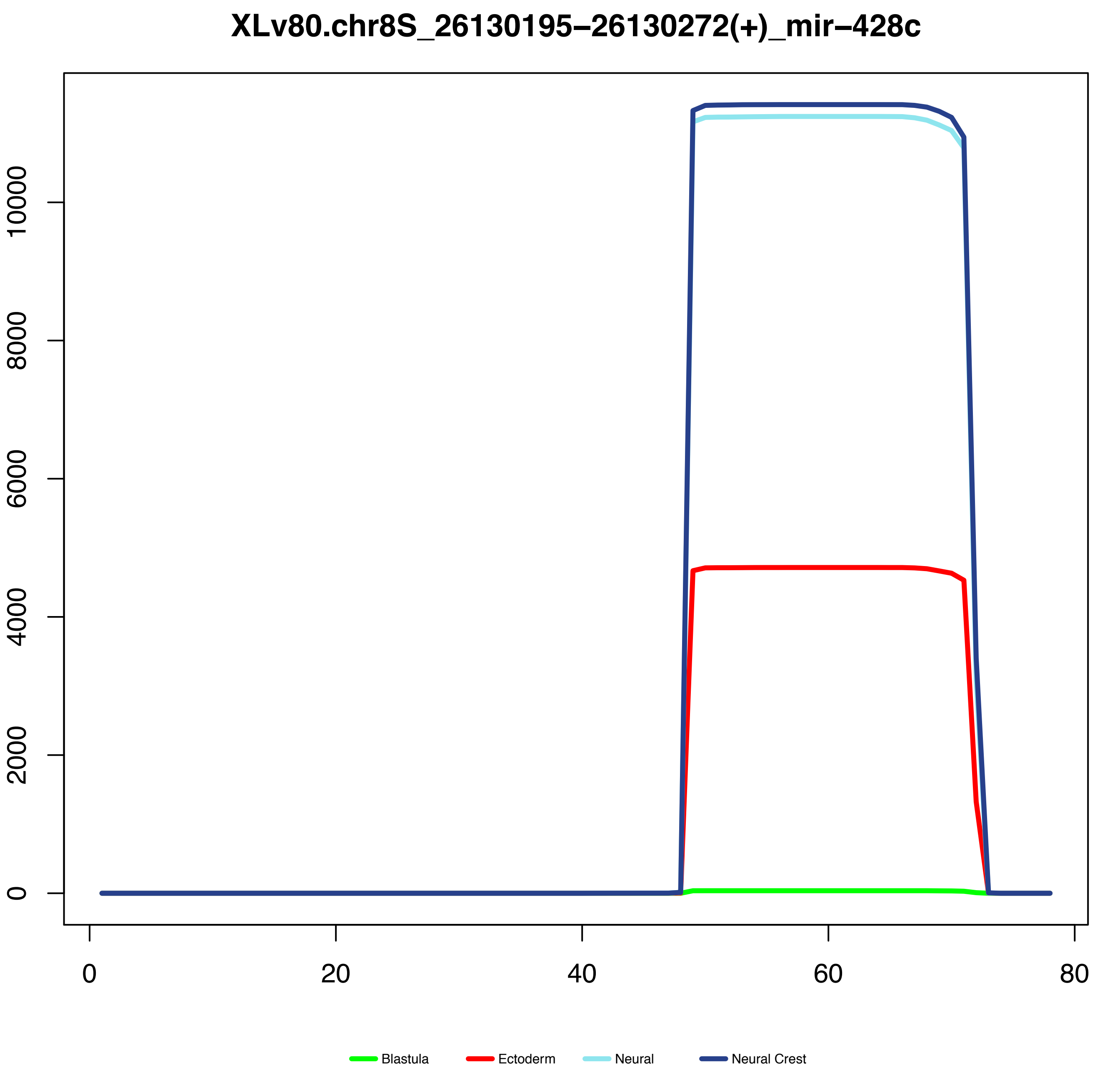

XLv80.chr8S_26130195−26130272(+)_mir−428c
0
2000
4000
6000
8000
10000
0
20
40
60
80
Blastula
Ectoderm
Neural
Neural Crest

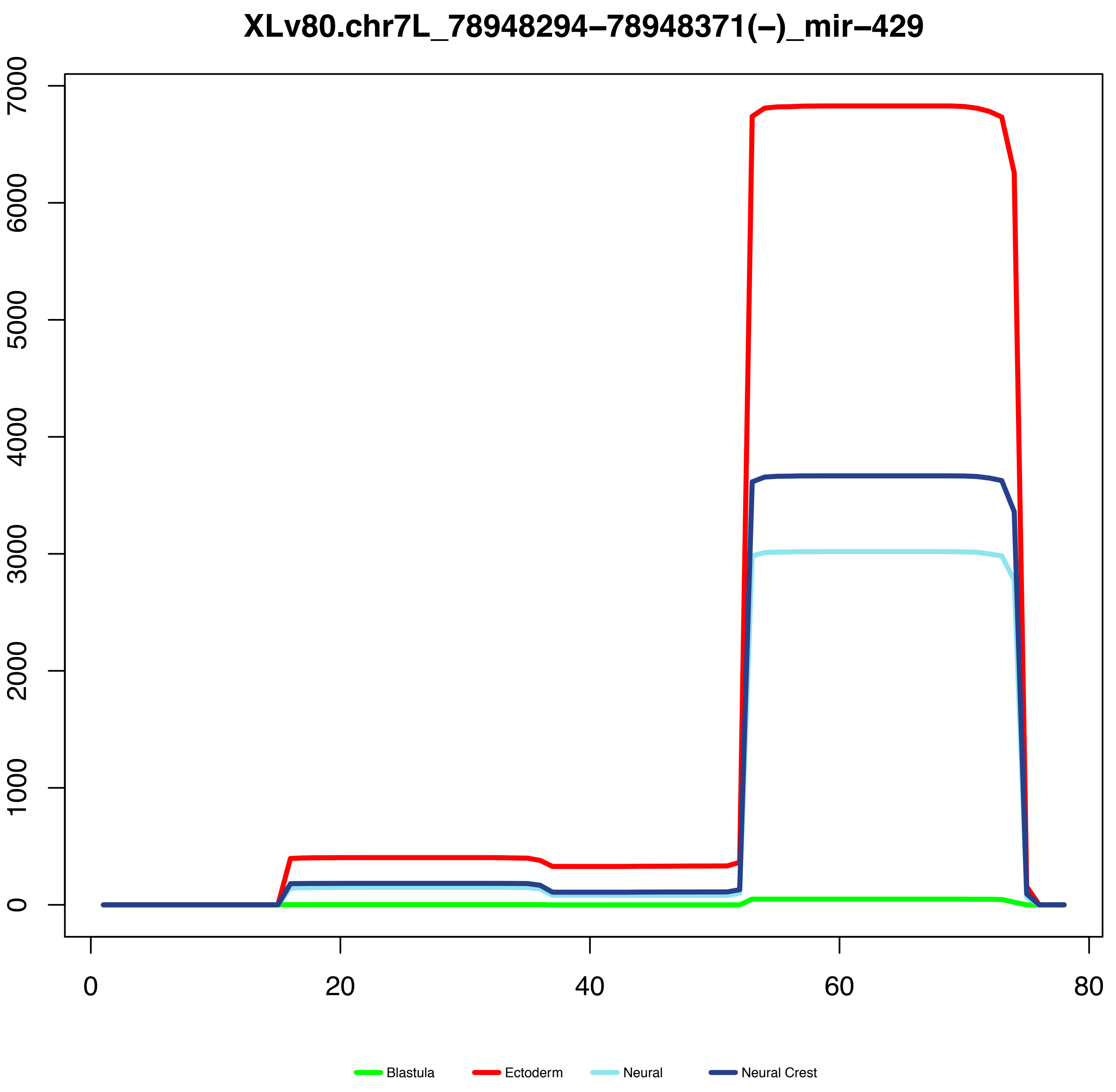
XLv80.chr7L_78948294−78948371(−)_mir−429
0
1000
2000
3000
4000
5000
6000
7000
0
20
40
60
80
Blastula
Ectoderm
Neural
Neural Crest

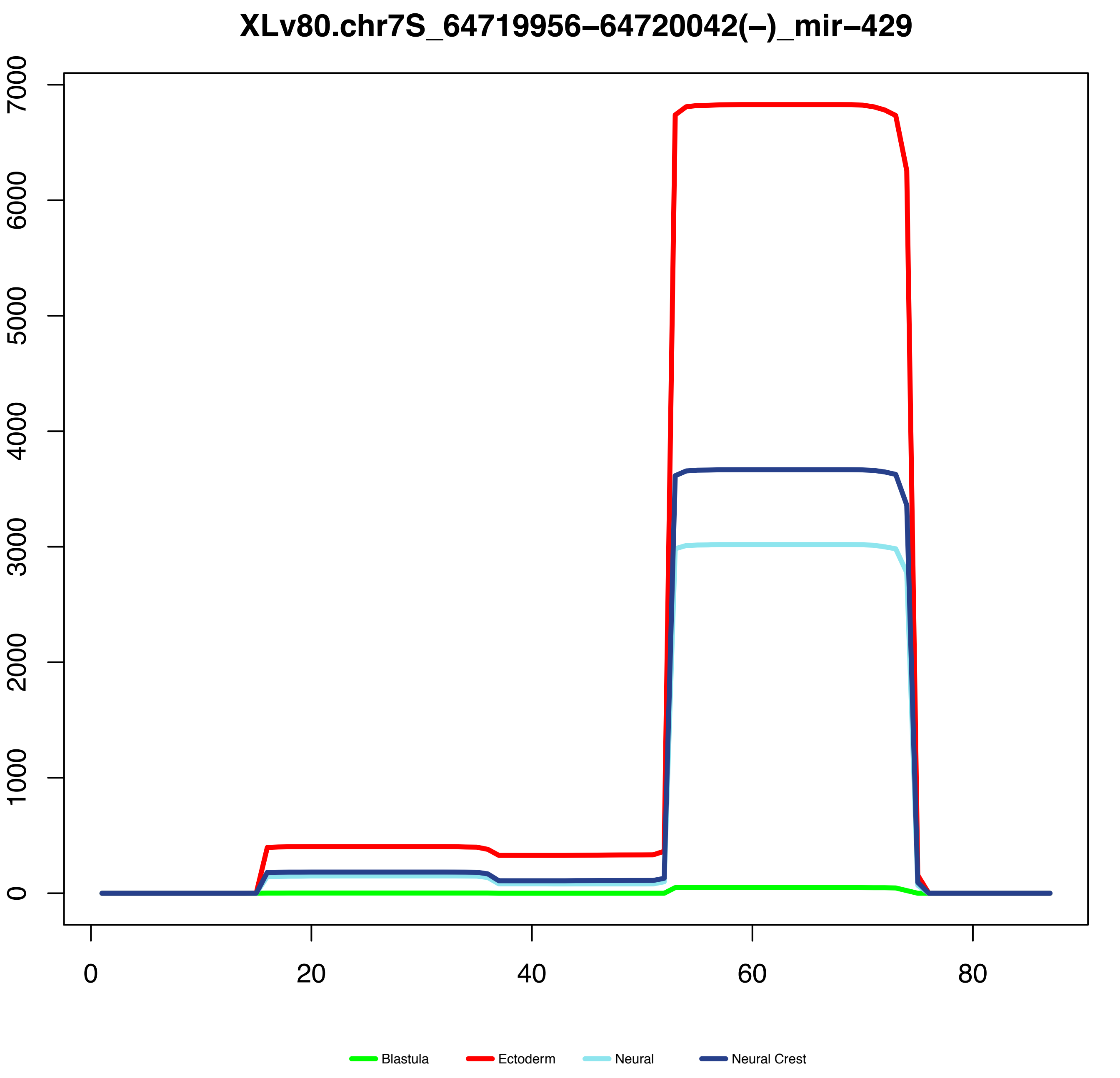

XLv80.chr7S_64719956−64720042(−)_mir−429
0
1000
2000
3000
4000
5000
6000
7000
0
20
40
60
80
Blastula
Ectoderm
Neural
Neural Crest

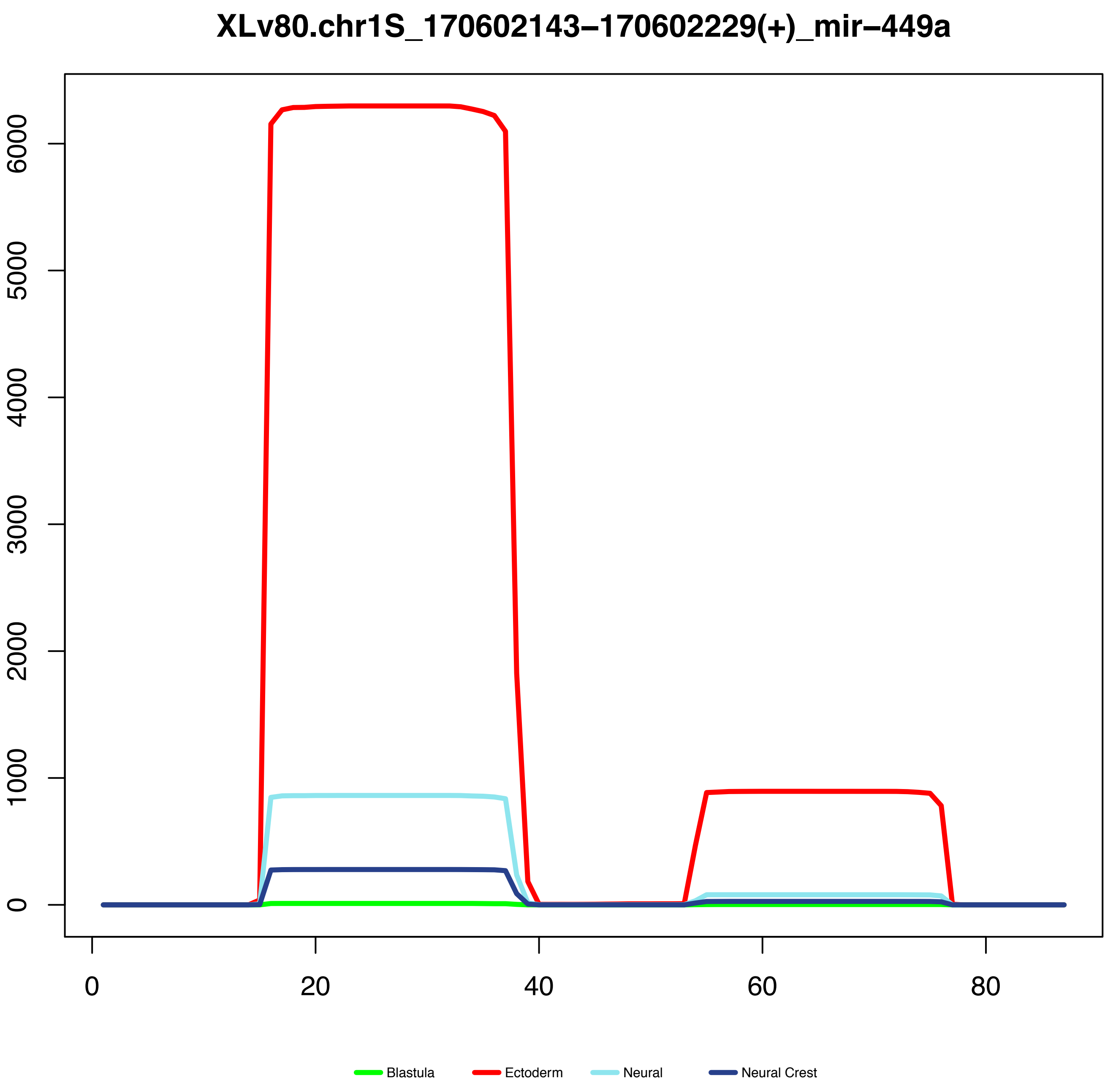

XLv80.chr1S_170602143−170602229(+)_mir−449a
0
1000
2000
3000
4000
5000
6000
0
20
40
60
80
Blastula
Ectoderm
Neural
Neural Crest

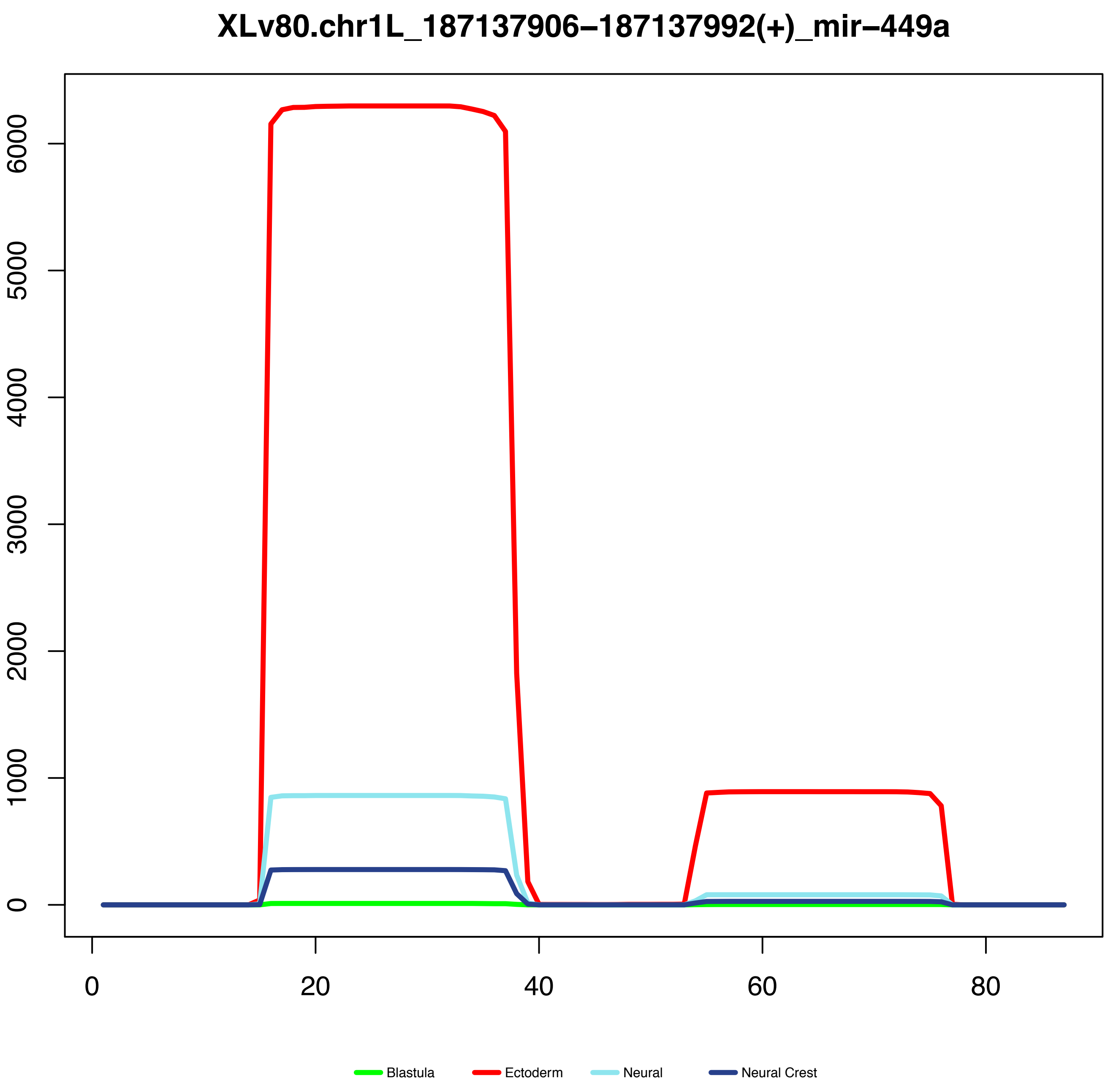

XLv80.chr1L_187137906−187137992(+)_mir−449a
0
1000
2000
3000
4000
5000
6000
0
20
40
60
80
Blastula
Ectoderm
Neural
Neural Crest

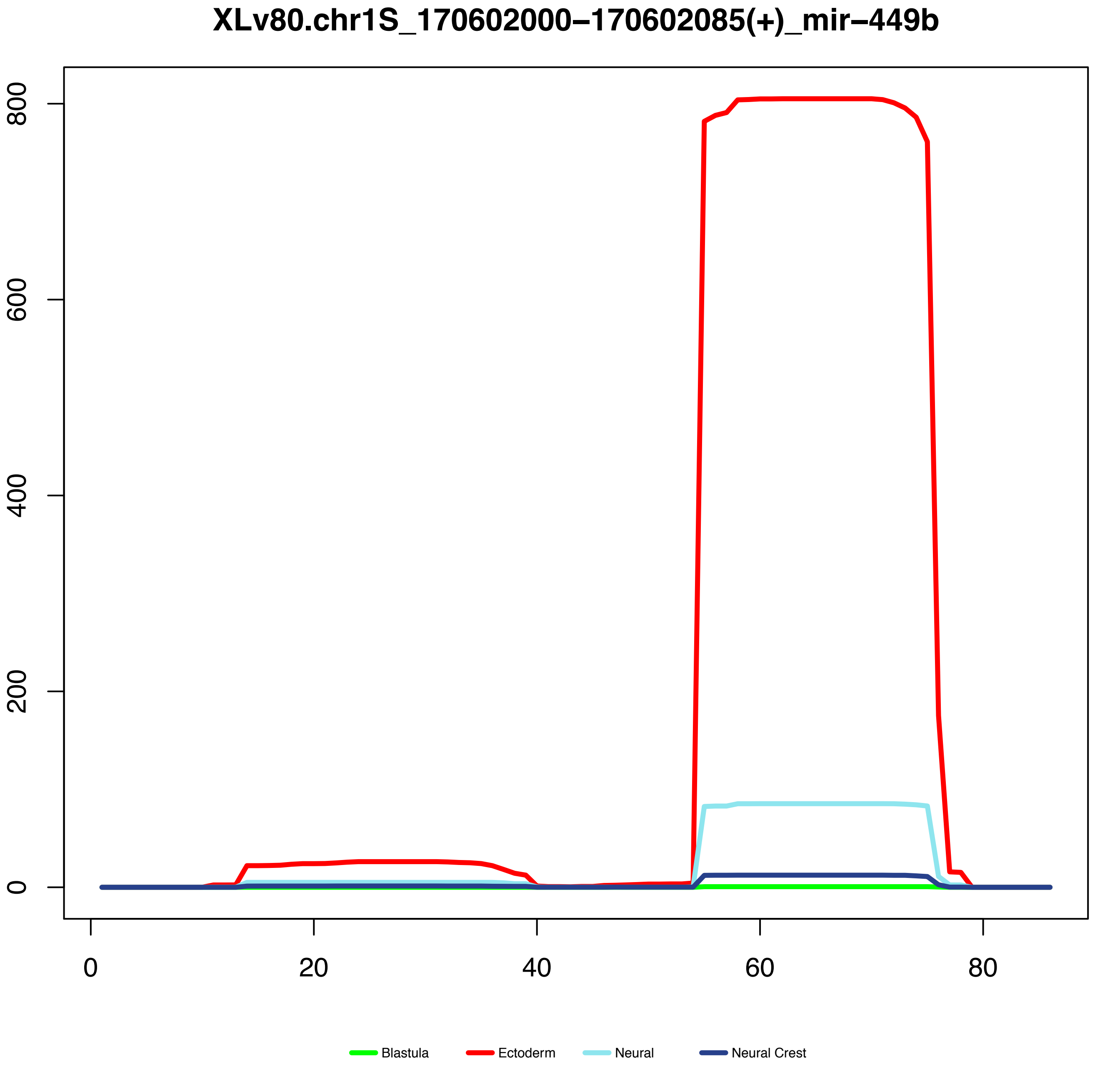

XLv80.chr1S_170602000−170602085(+)_mir−449b
0
200
400
600
800
0
20
40
60
80
Blastula
Ectoderm
Neural
Neural Crest

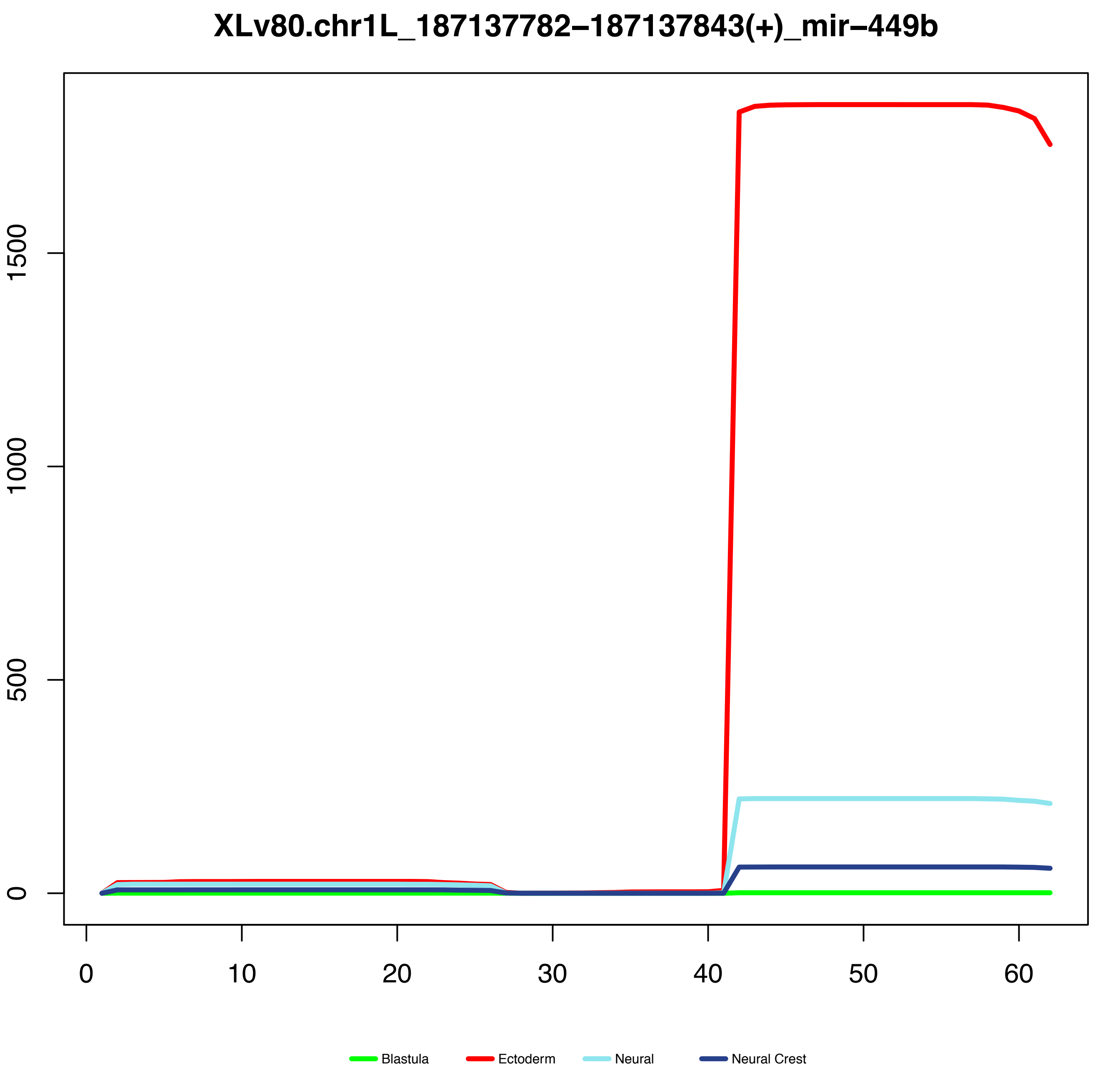
XLv80.chr1L_187137782−187137843(+)_mir−449b
0
500
1000
1500
0
10
20
30
40
50
60
Blastula
Ectoderm
Neural
Neural Crest

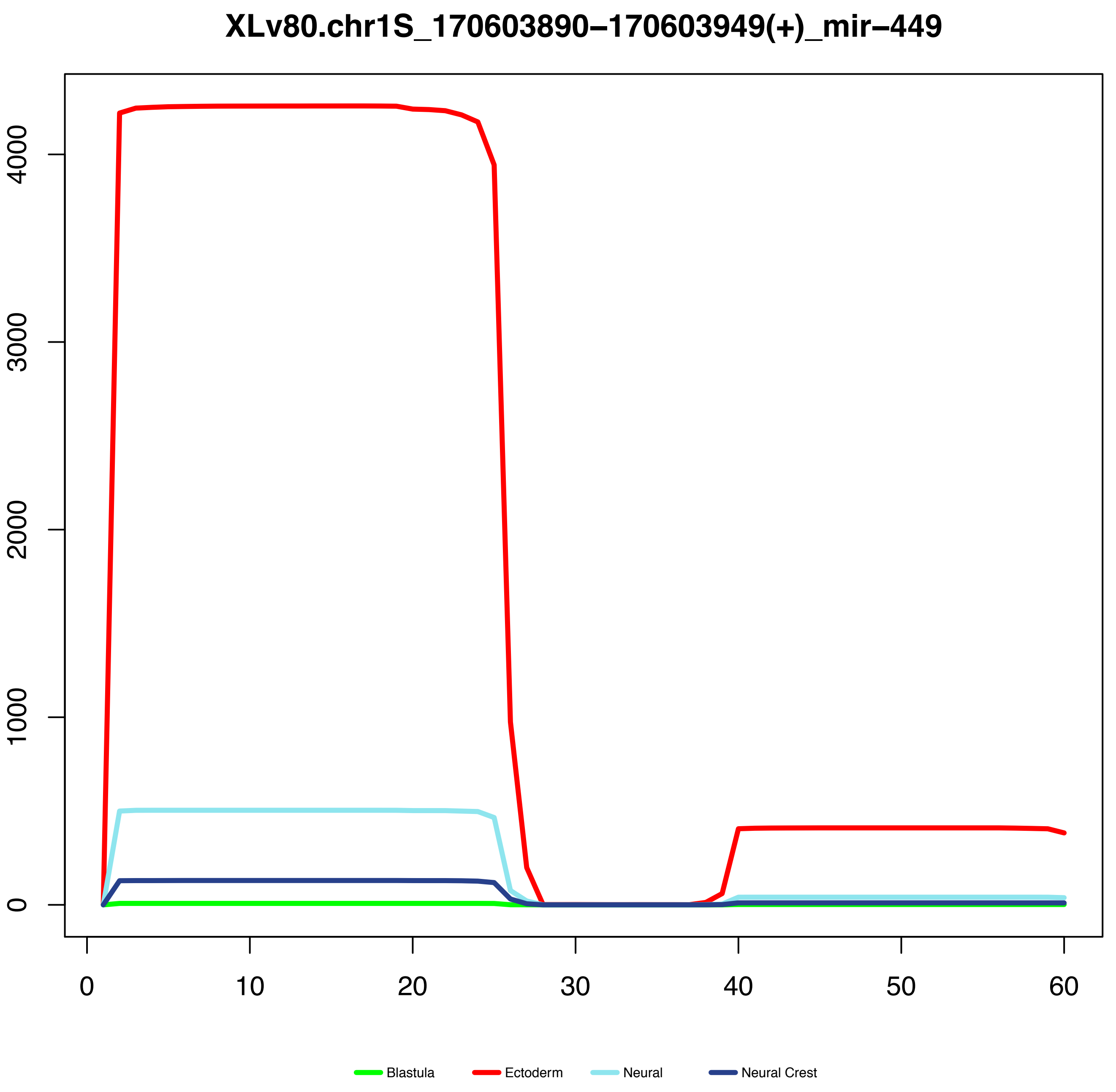
XLv80.chr1S_170603890−170603949(+)_mir−449
0
1000
2000
3000
4000
0
10
20
30
40
50
60
Blastula
Ectoderm
Neural
Neural Crest

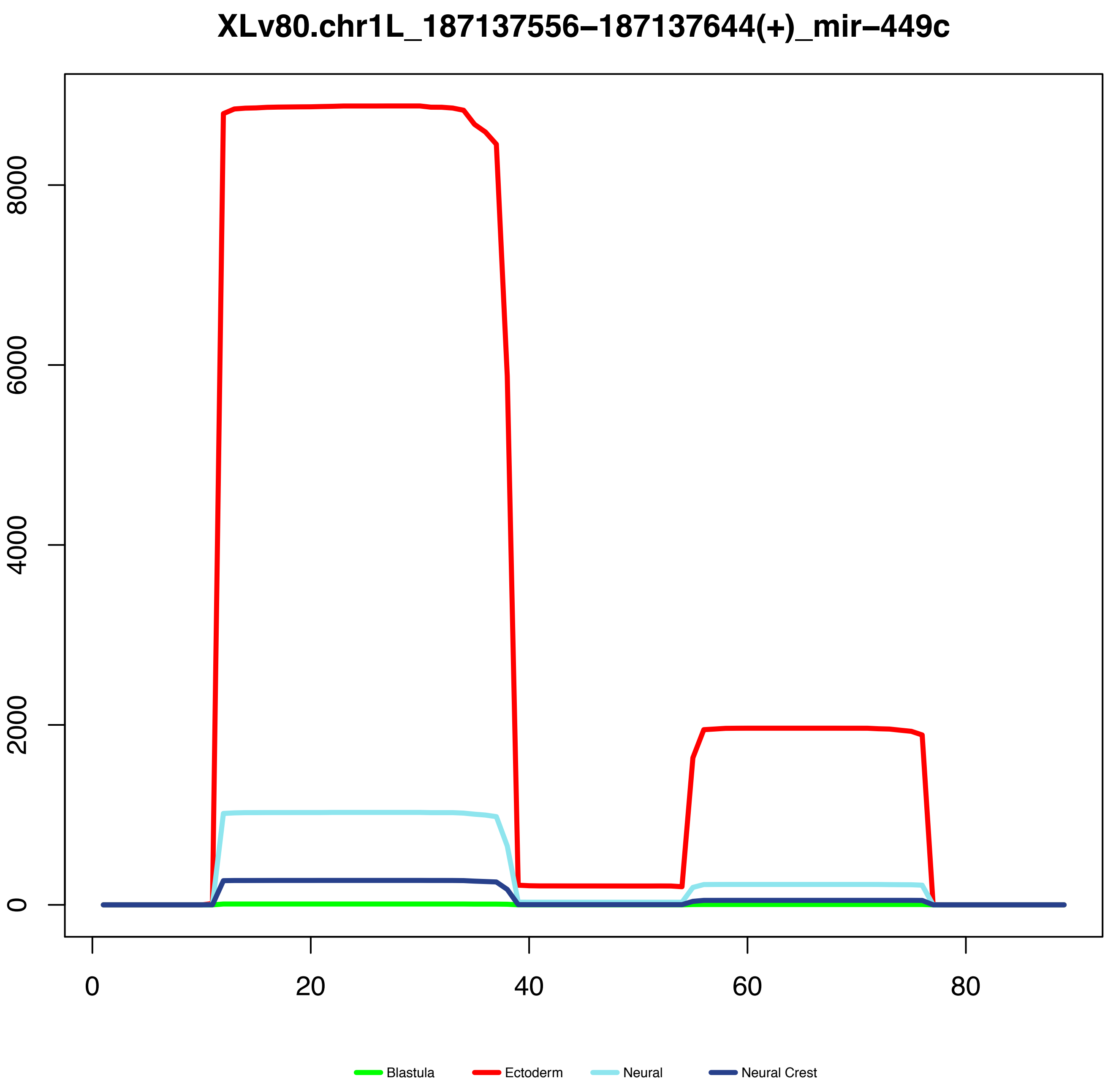

XLv80.chr1L_187137556−187137644(+)_mir−449c
0
2000
4000
6000
8000
0
20
40
60
80
Blastula
Ectoderm
Neural
Neural Crest

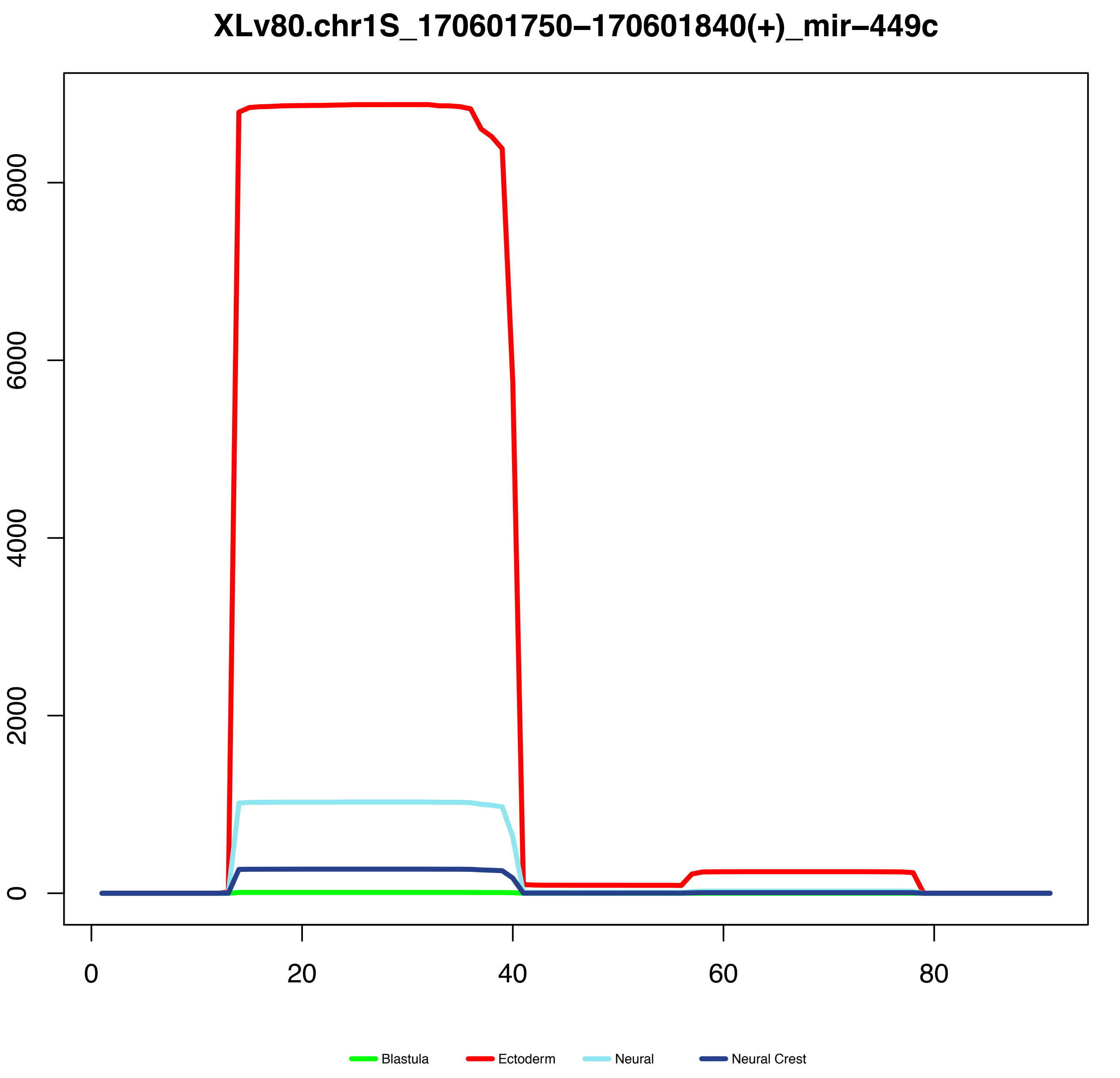

XLv80.chr1S_170601750−170601840(+)_mir−449c
0
2000
4000
6000
8000
0
20
40
60
80
Blastula
Ectoderm
Neural
Neural Crest

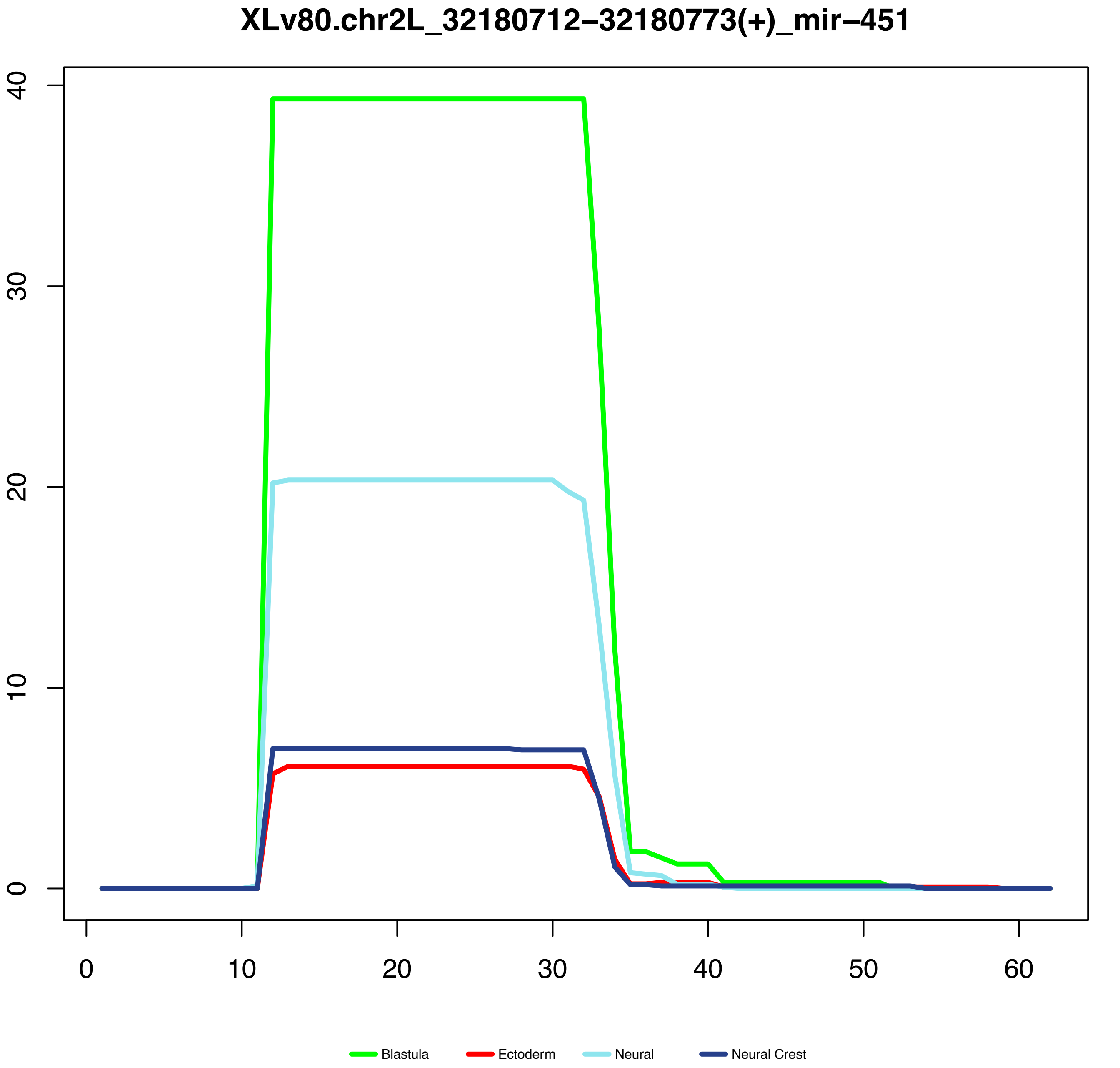

XLv80.chr2L_32180712−32180773(+)_mir−451
0
10
20
30
40
50
60
Blastula
Ectoderm
Neural
Neural Crest

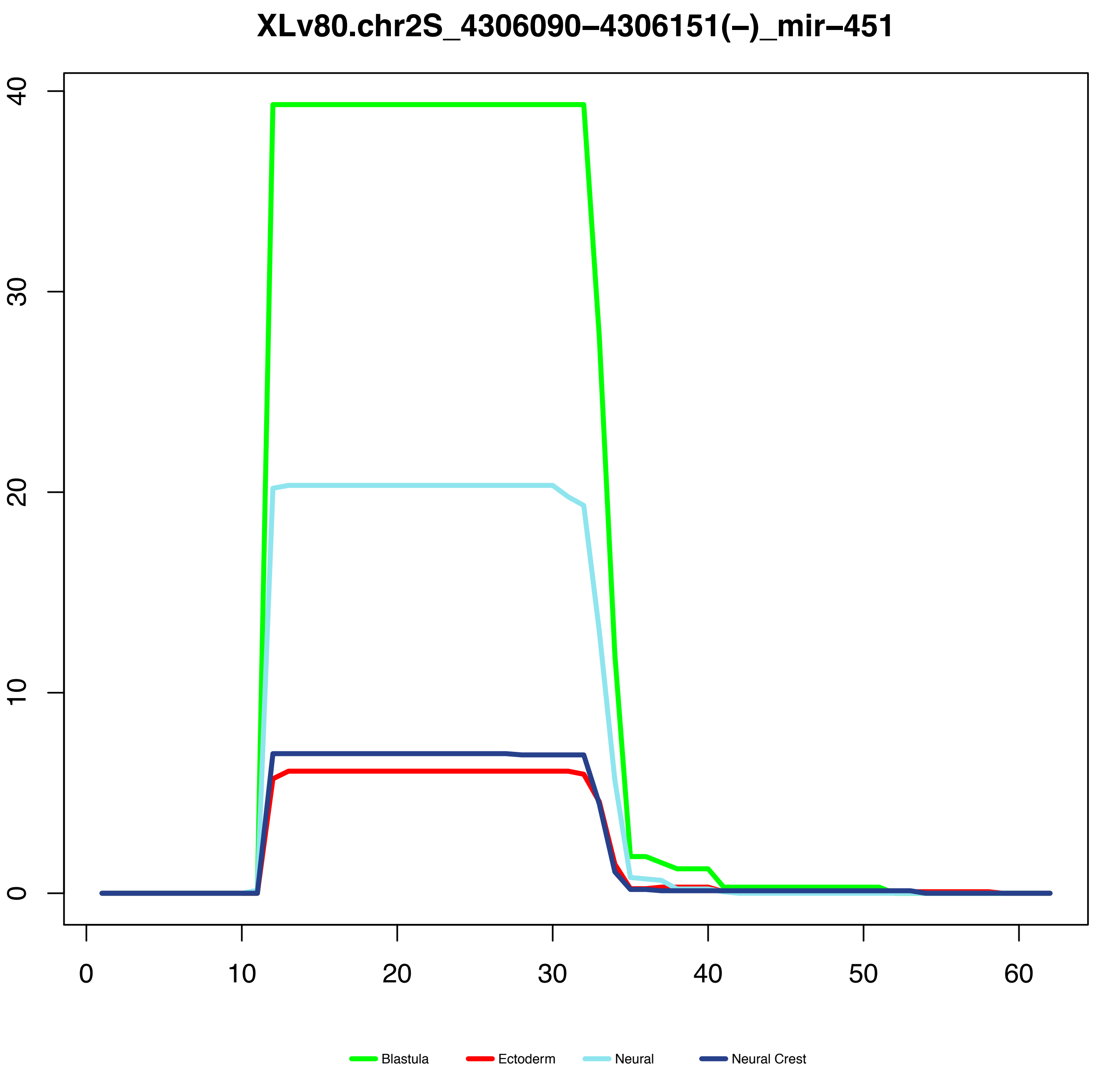
XLv80.chr2S_4306090−4306151(−)_mir−451
0
10
20
30
40
0
10
20
30
40
50
60
Blastula
Ectoderm
Neural
Neural Crest

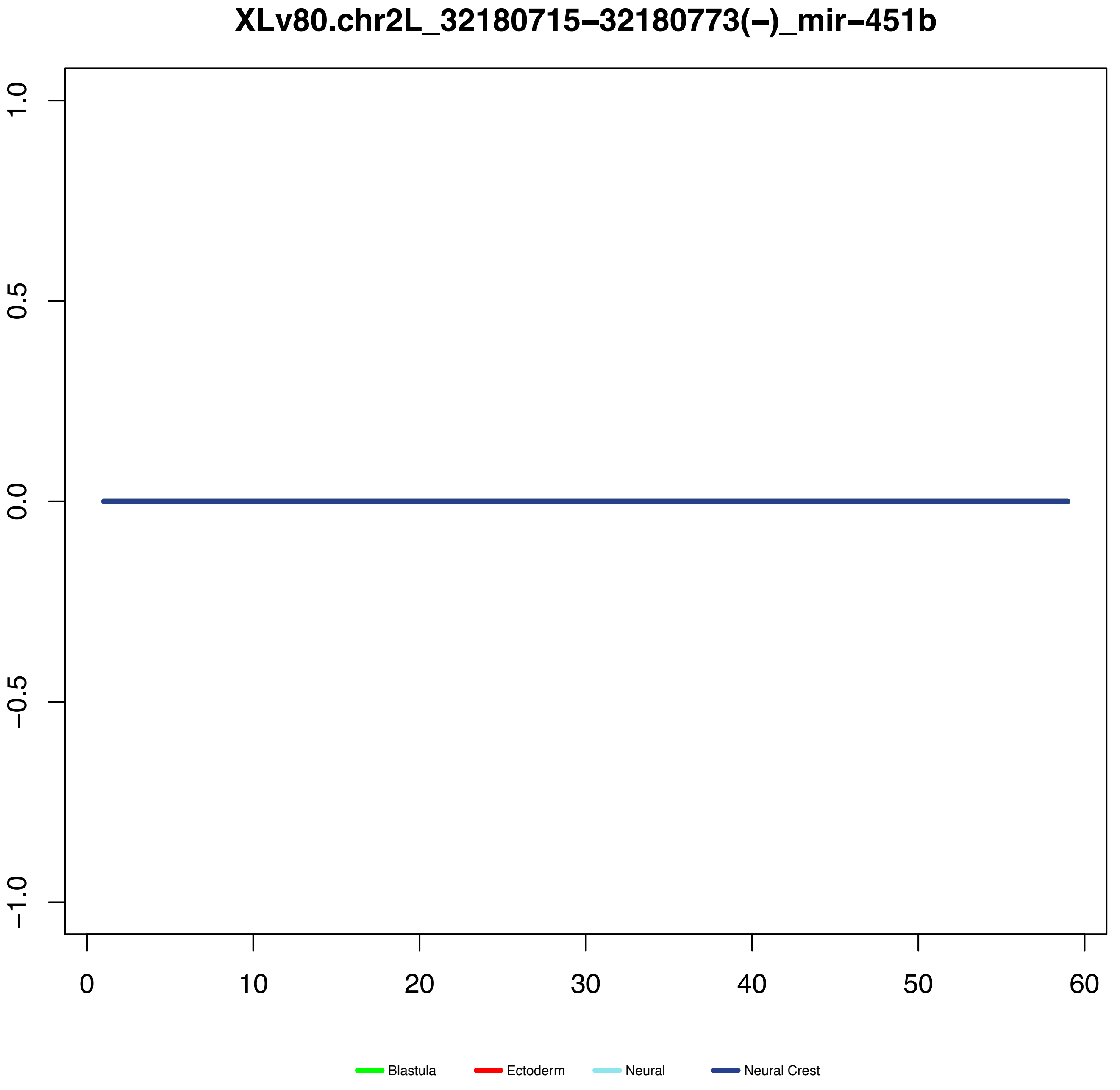

XLv80.chr2L_32180715−32180773(−)_mir−451b
1.0
0.5
0.0
−0.5
−1.0
0
10
20
30
40
50
60
Blastula
Ectoderm
Neural
Neural Crest

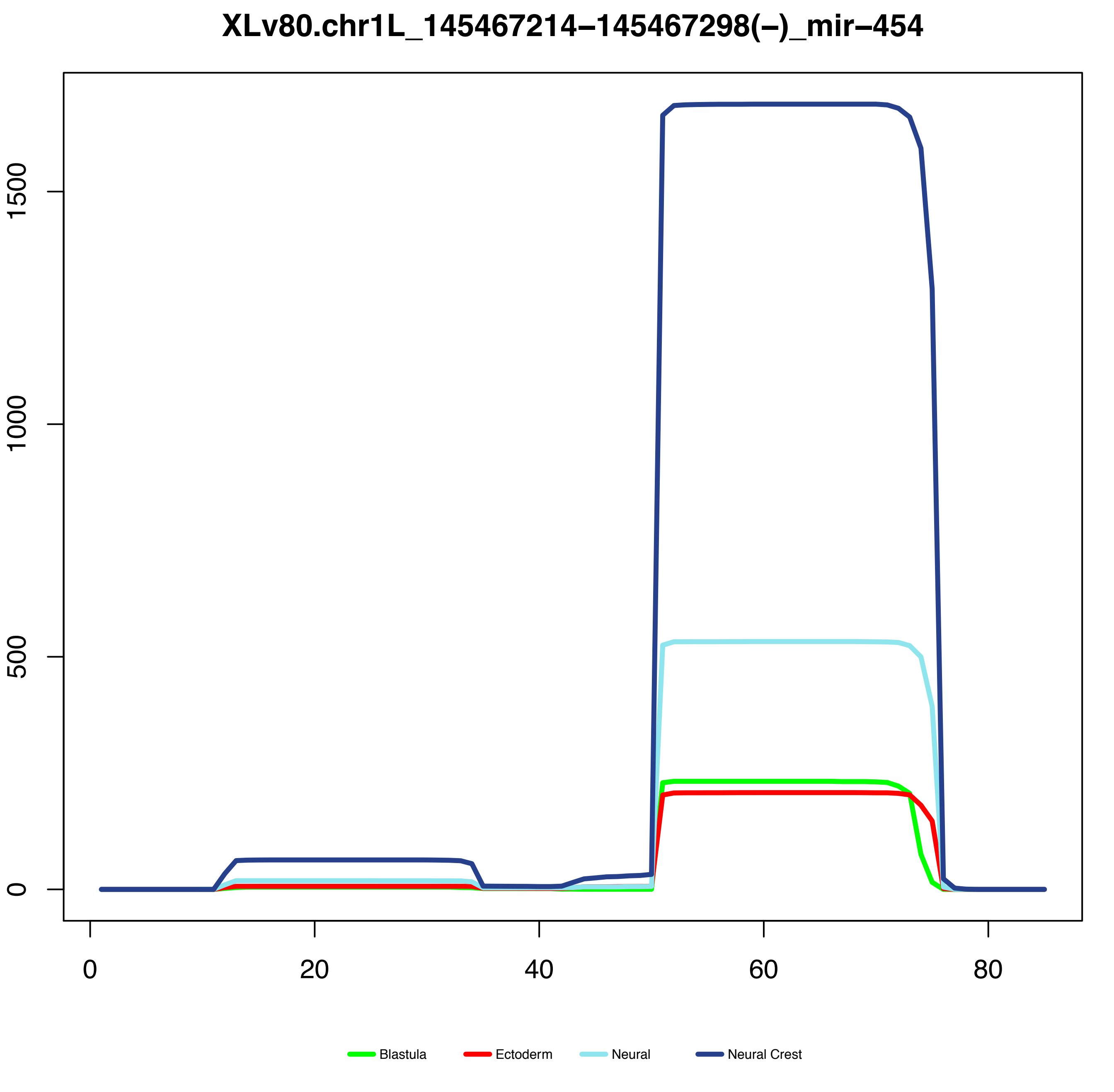
XLv80.chr1L_145467214−145467298(−)_mir−454
0
500
1000
1500
0
20
40
60
80
Blastula
Ectoderm
Neural
Neural Crest

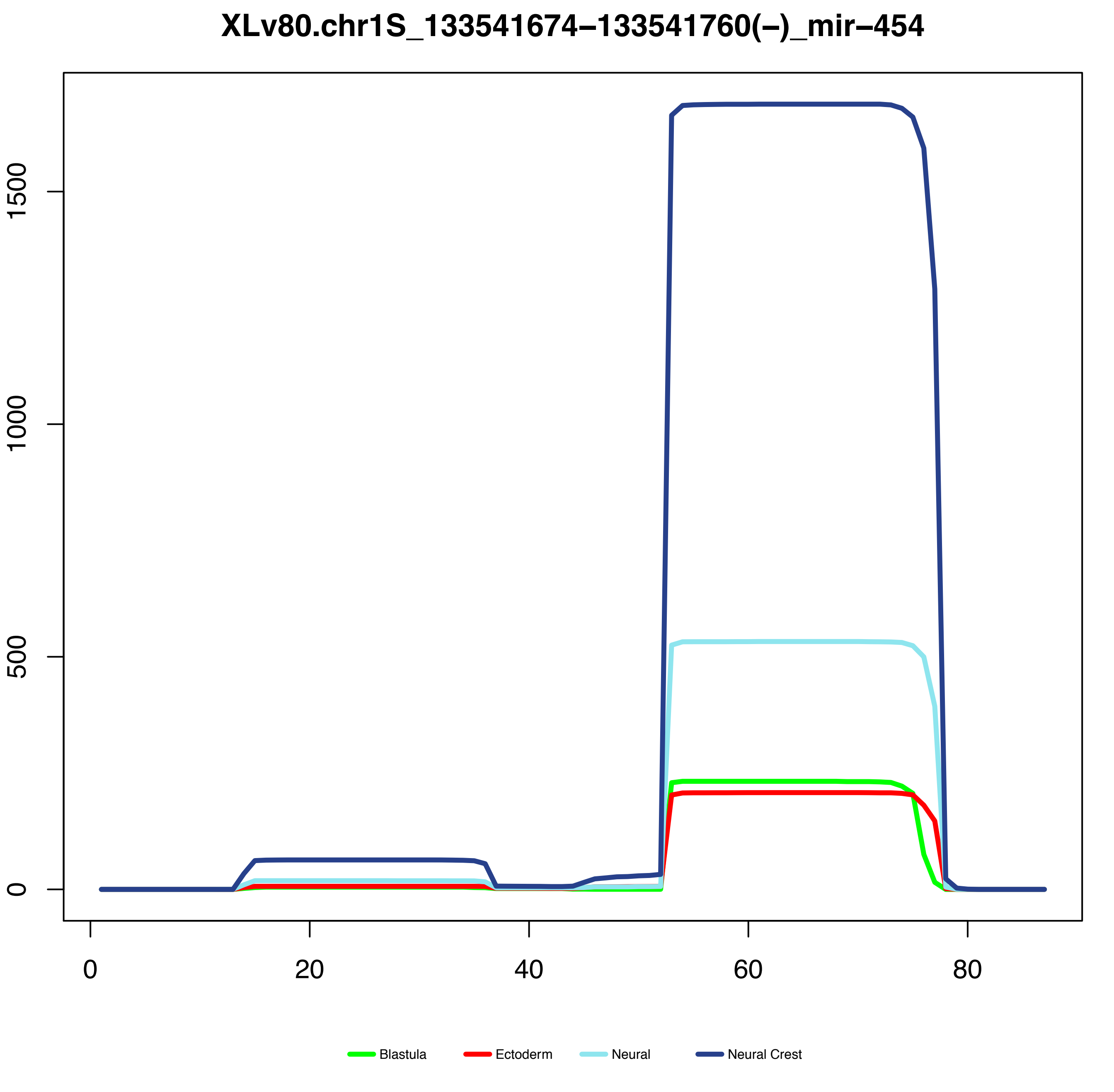
XLv80.chr1S_133541674−133541760(−)_mir−454
0
500
1000
1500
0
20
40
60
80
Blastula
Ectoderm
Neural
Neural Crest

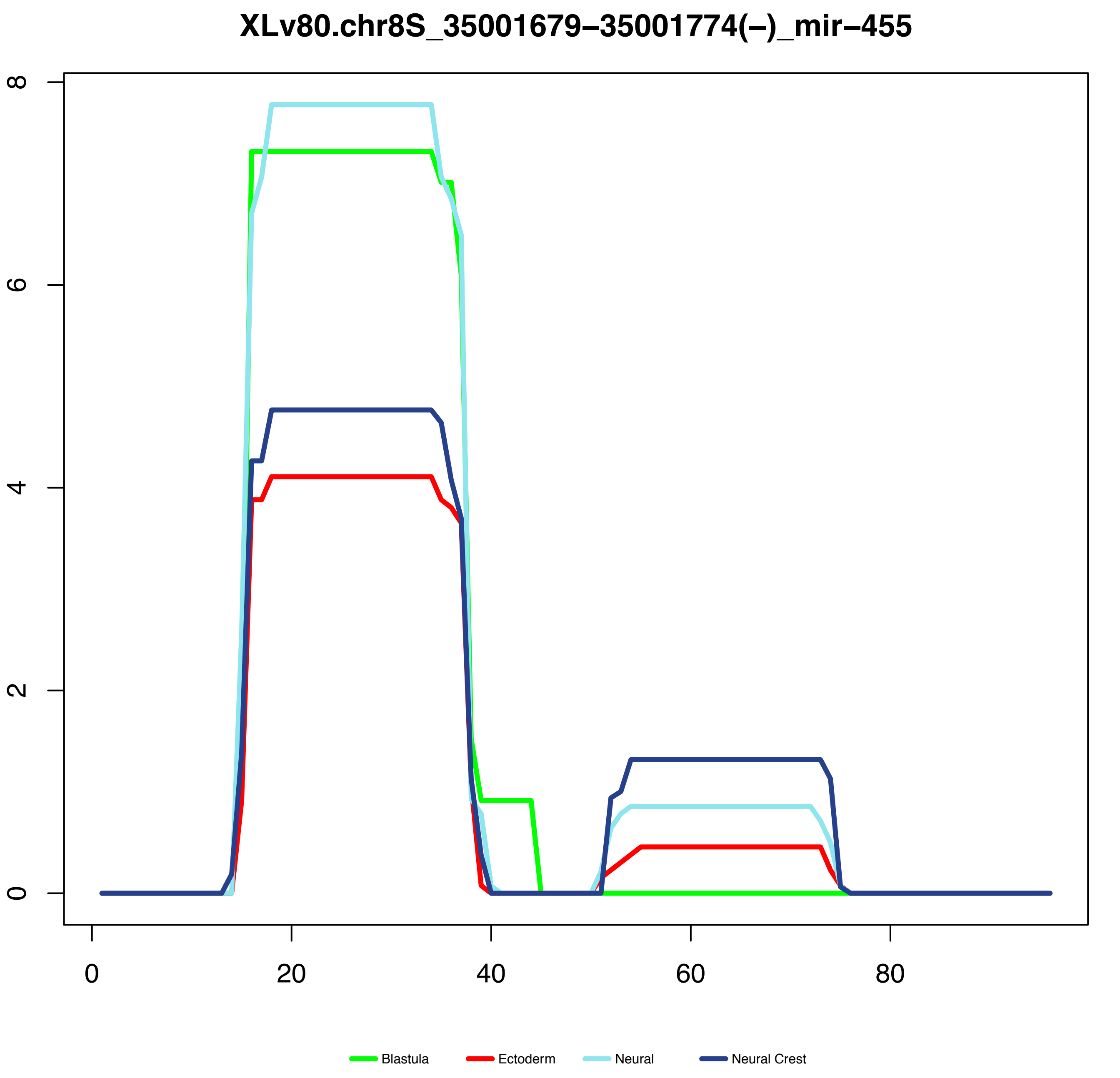
XLv80.chr8S_35001679–35001774(–)_mir–455
0
2
4
6
8
0
20
40
60
80
Blastula
Ectoderm
Neural
Neural Crest

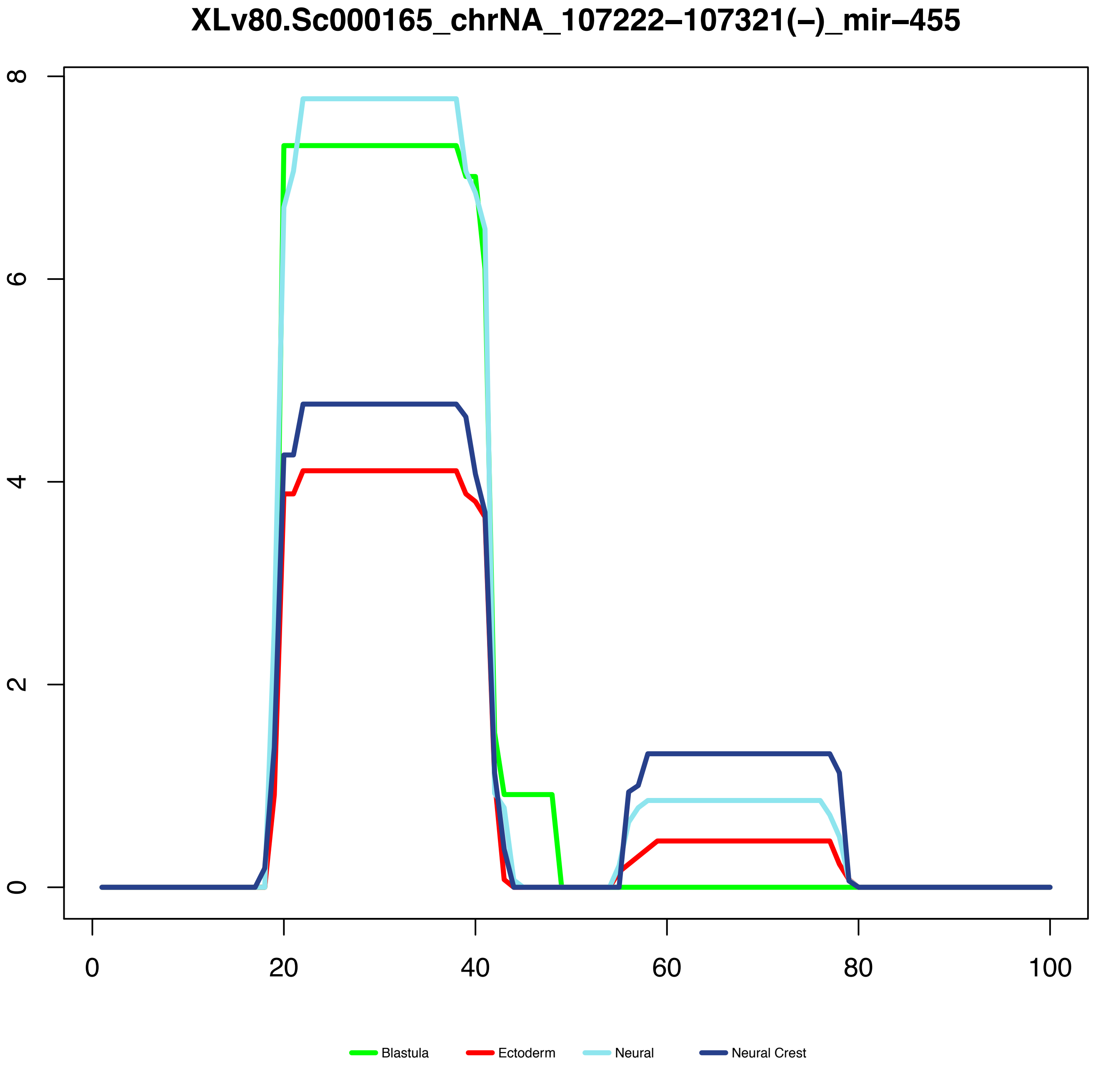

XLv80.Sc000165_chrNA_107222−107321(−)_mir−455
0
2
4
6
8
0
20
40
60
80
100
Blastula
Ectoderm
Neural
Neural Crest

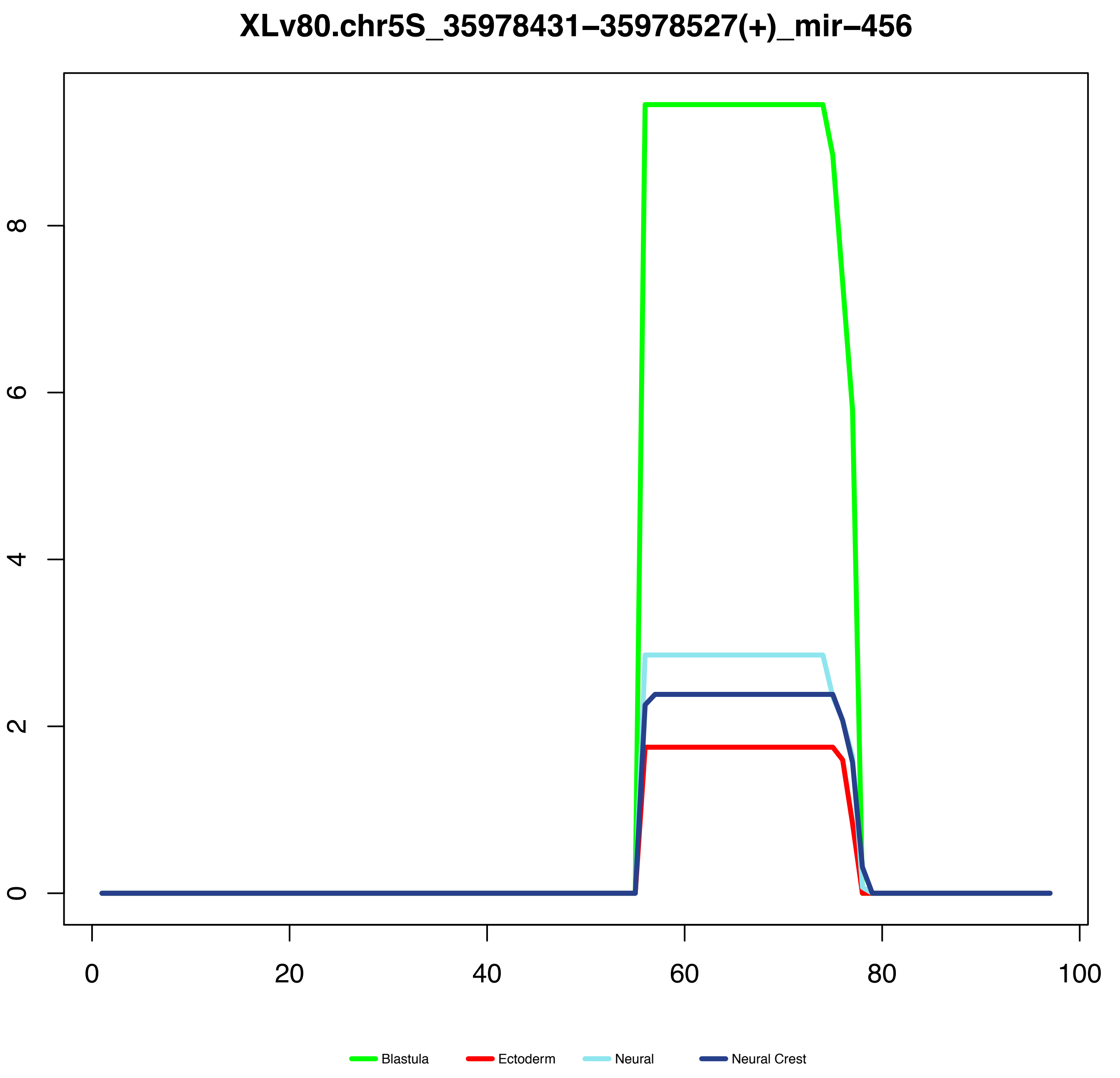

XLv80.chr5S_35978431−35978527(+)_mir−456
0
2
4
6
8
0
20
40
60
80
100
Blastula
Ectoderm
Neural
Neural Crest

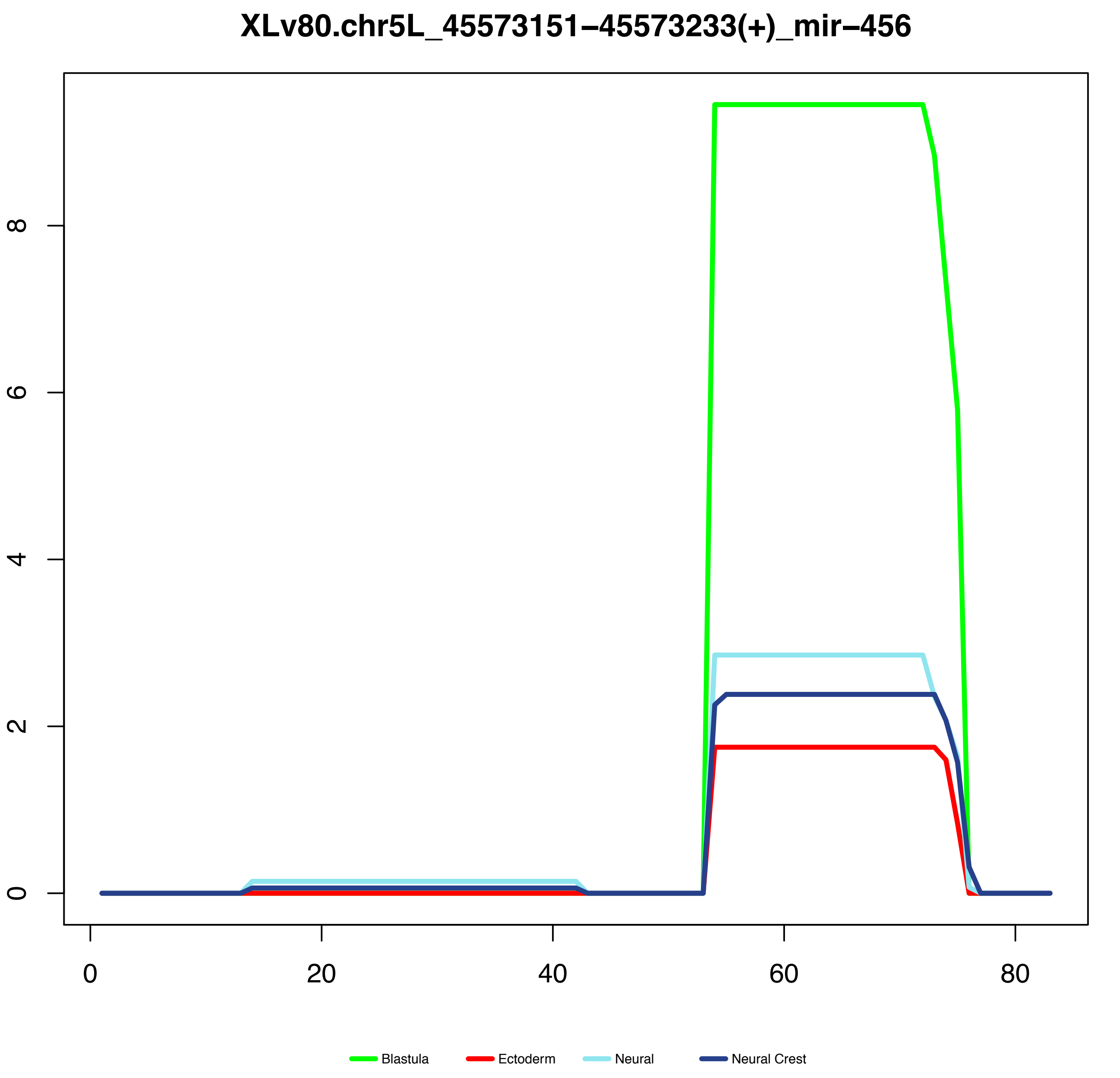
XLv80.chr5L_45573151−45573233(+)_mir−456
0
2
4
6
8
0
20
40
60
80
Blastula
Ectoderm
Neural
Neural Crest

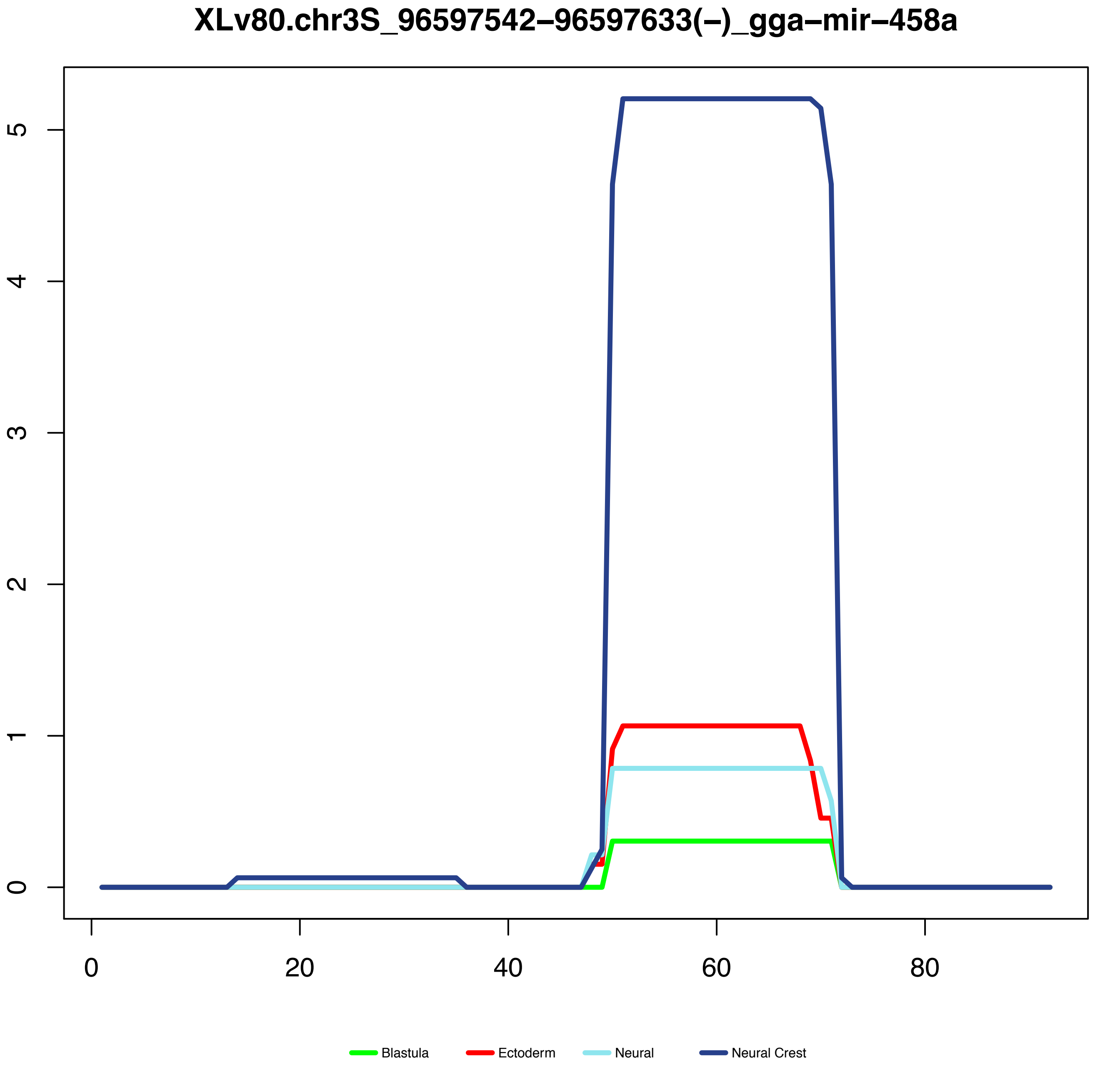
XLv80.chr3S_96597542−96597633(−)_gga−mir−458a
0
1
2
3
4
5
0
20
40
60
80
Blastula
Ectoderm
Neural
Neural Crest

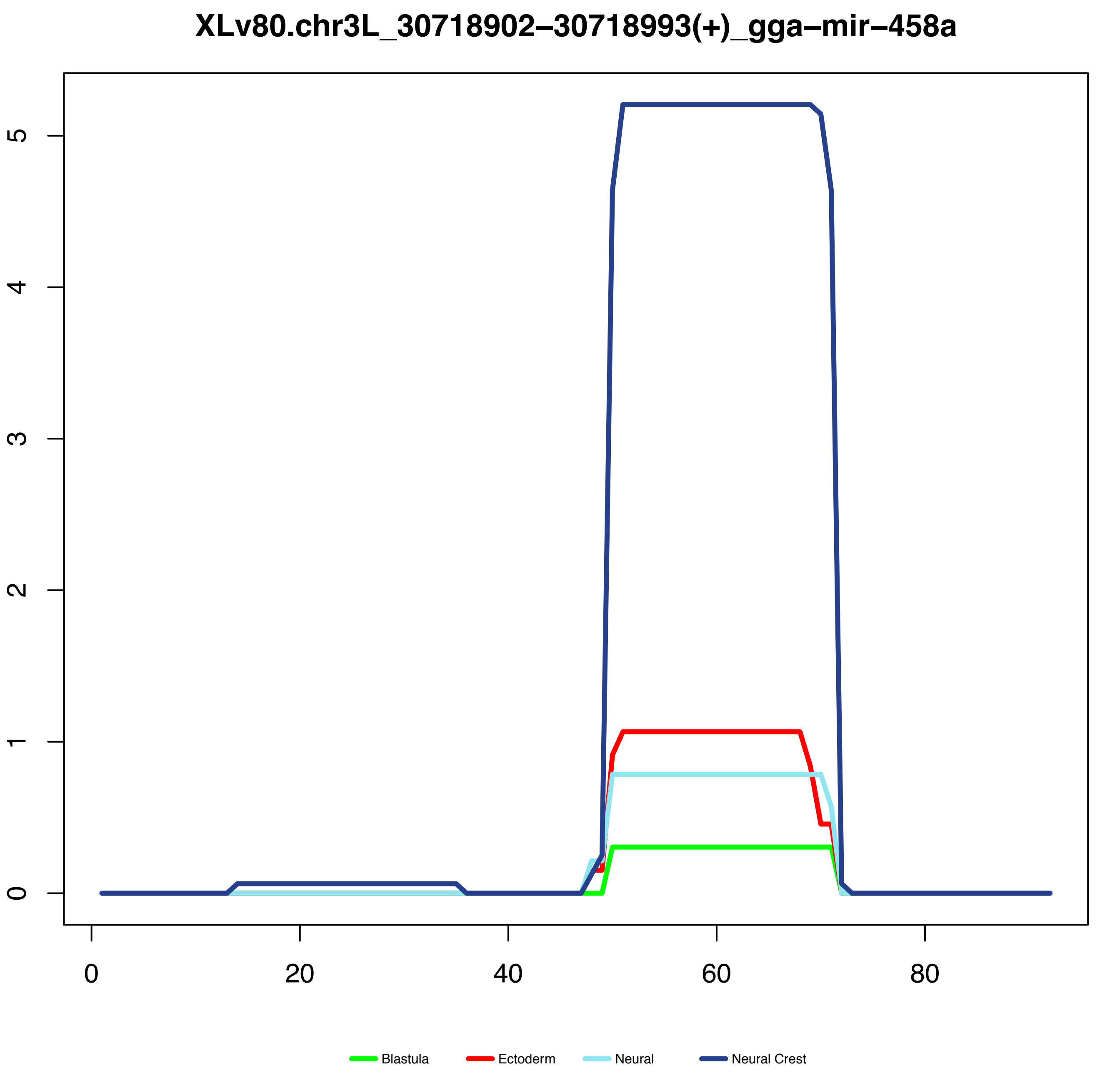

XLv80.chr3L_30718902−30718993(+)_gga−mir−458a
0
1
2
3
4
5
0
20
40
60
80
Blastula
Ectoderm
Neural
Neural Crest

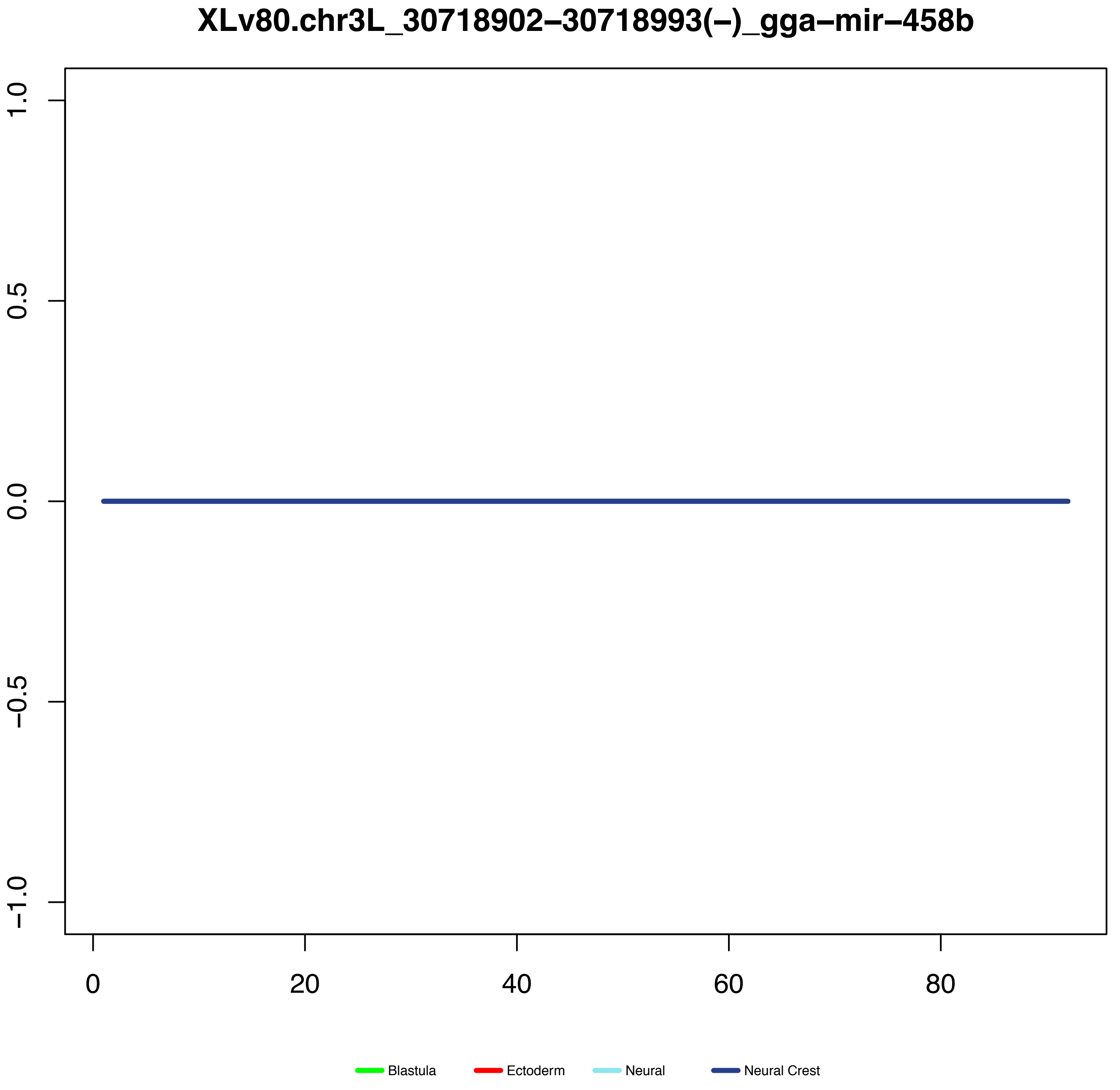

XLv80.chr3L_30718902–30718993(–)_gga–mir–458b
1.0
0.5
0.0
−0.5
−1.0
0
20
40
60
80
Blastula
Ectoderm
Neural
Neural Crest

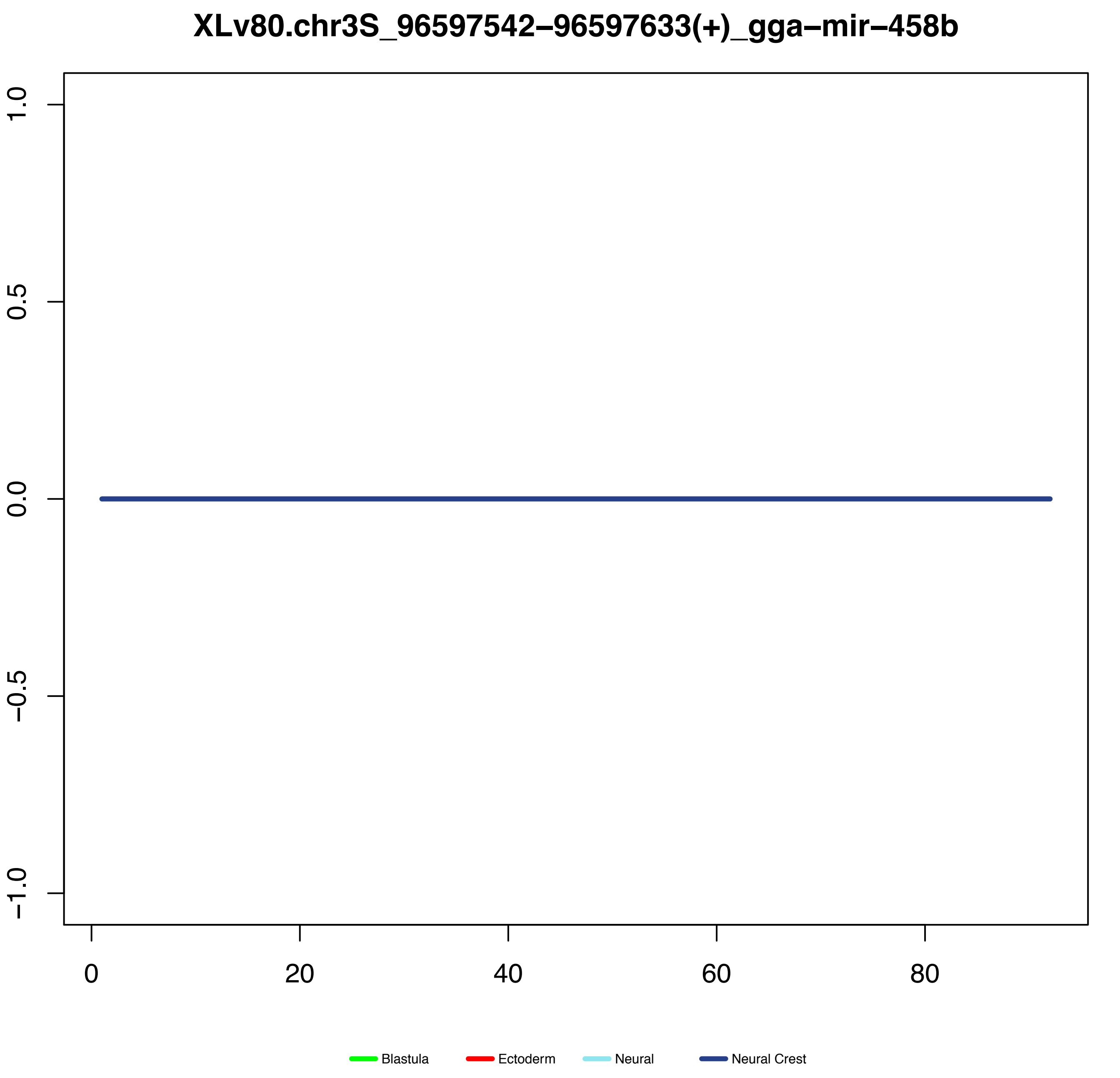
XLv80.chr3S_96597542−96597633(+)_gga−mir−458b
1.0
0.5
0.0
−0.5
−1.0
0
20
40
60
80
Blastula
Ectoderm
Neural
Neural Crest

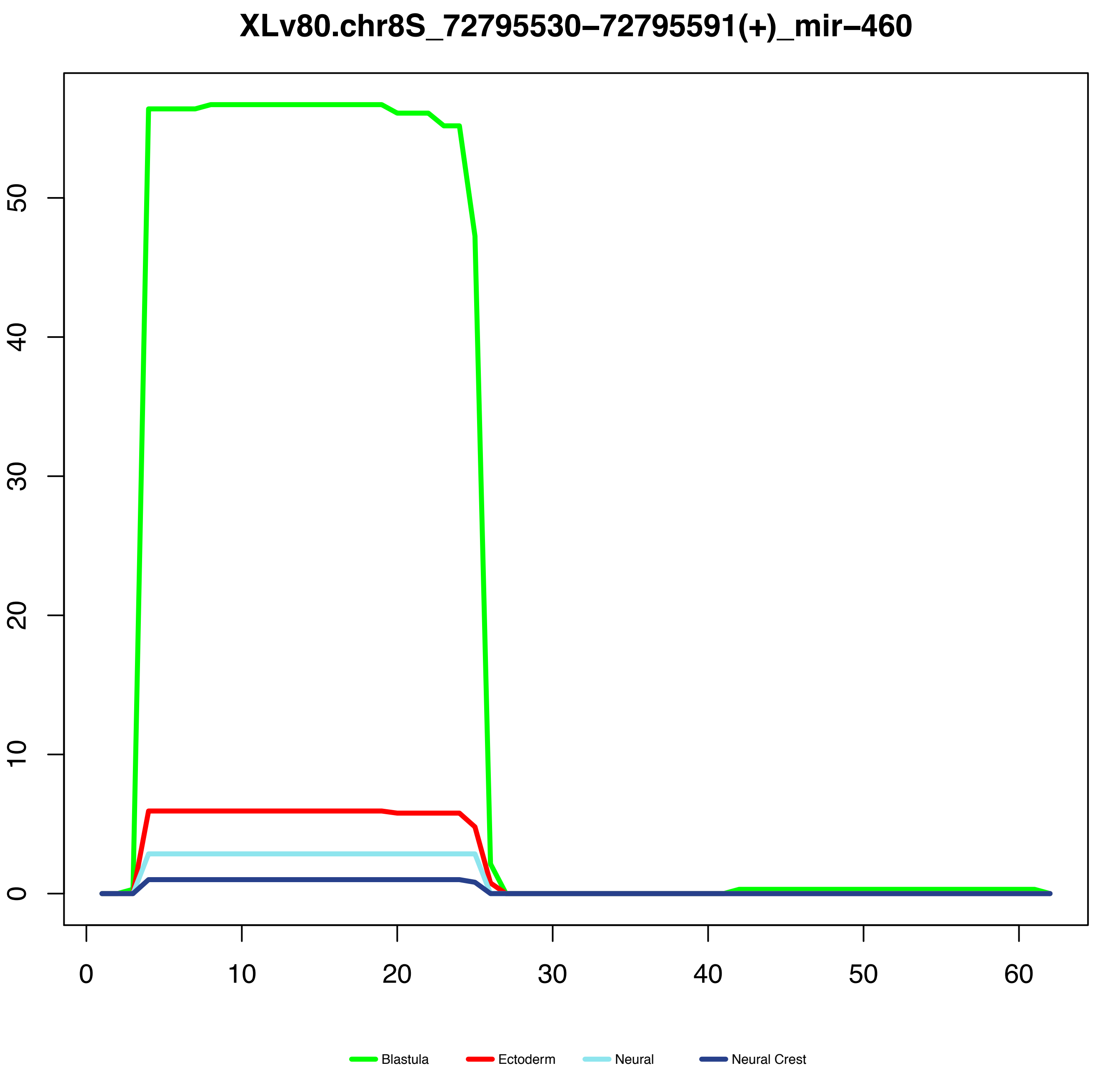
XLv80.chr8S_72795530–72795591(+)_mir–460
0
10
20
30
40
50
60
Blastula
Ectoderm
Neural
Neural Crest

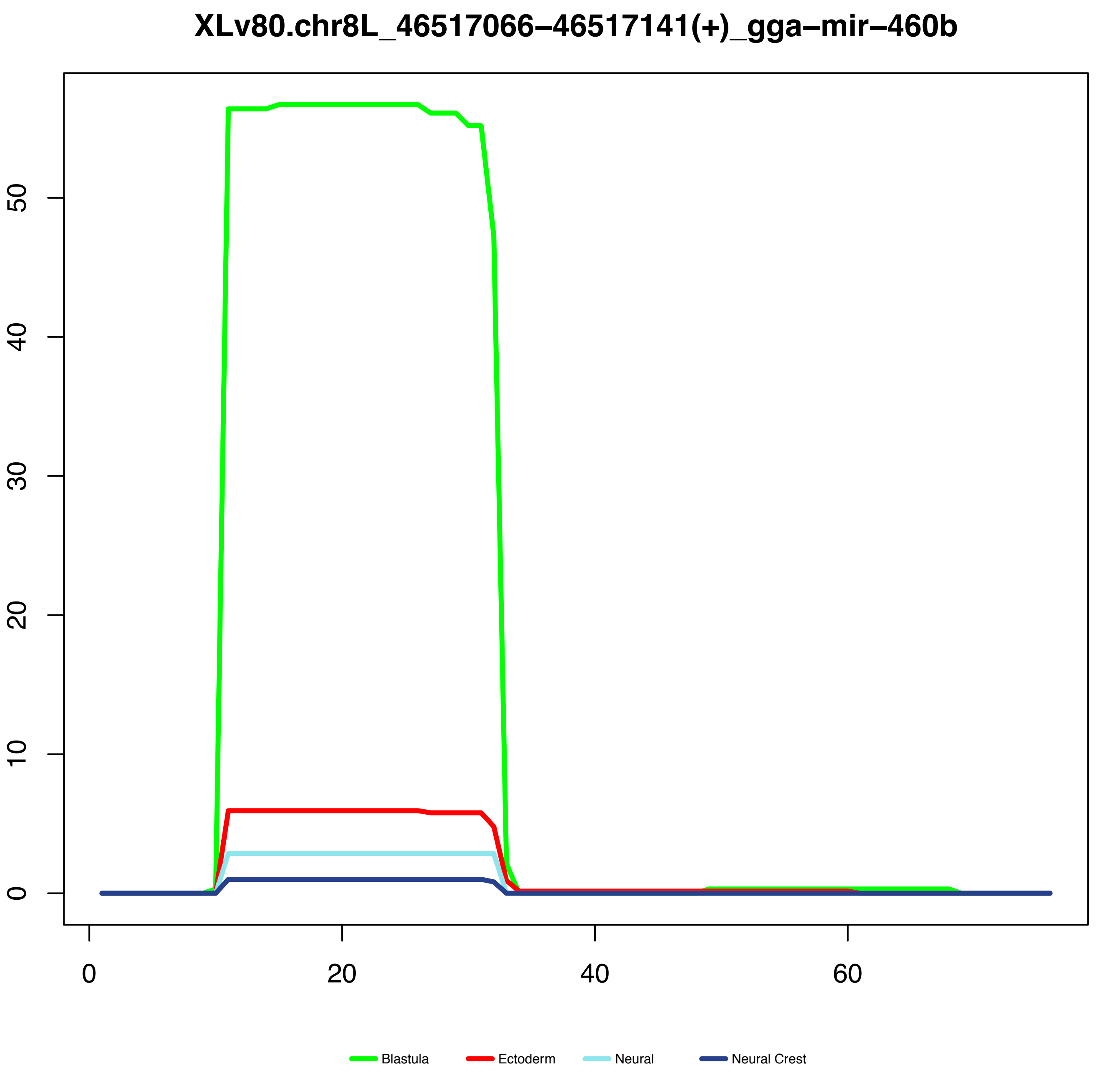
XLv80.chr8L_46517066–46517141(+)_gga–mir–460b
0
10
20
30
40
50
0
20
40
60
Blastula
Ectoderm
Neural
Neural Crest

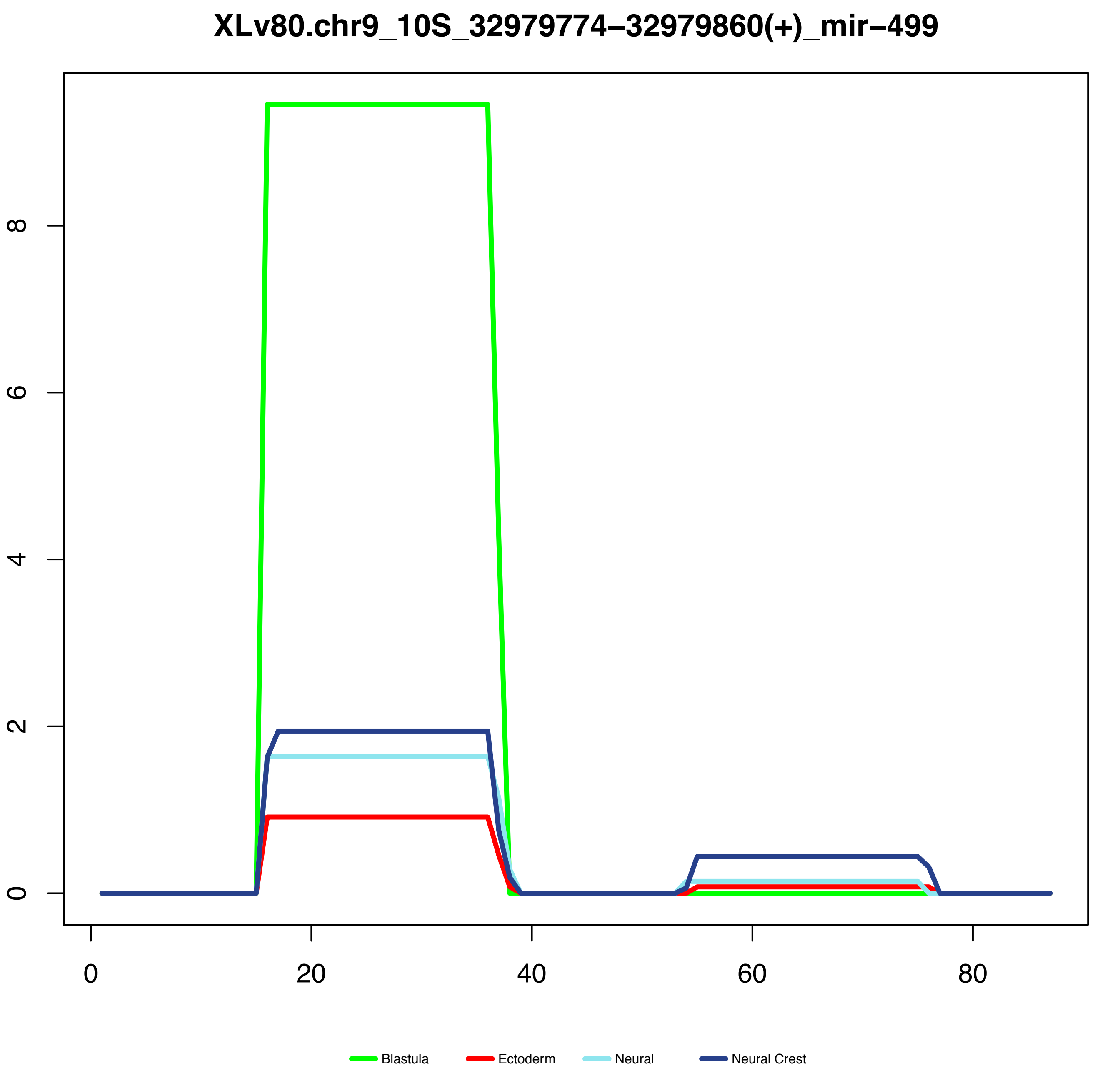

XLv80.chr9_10S_32979774–32979860(+)_mir–499
0
2
4
6
8
0
20
40
60
80
Blastula
Ectoderm
Neural
Neural Crest

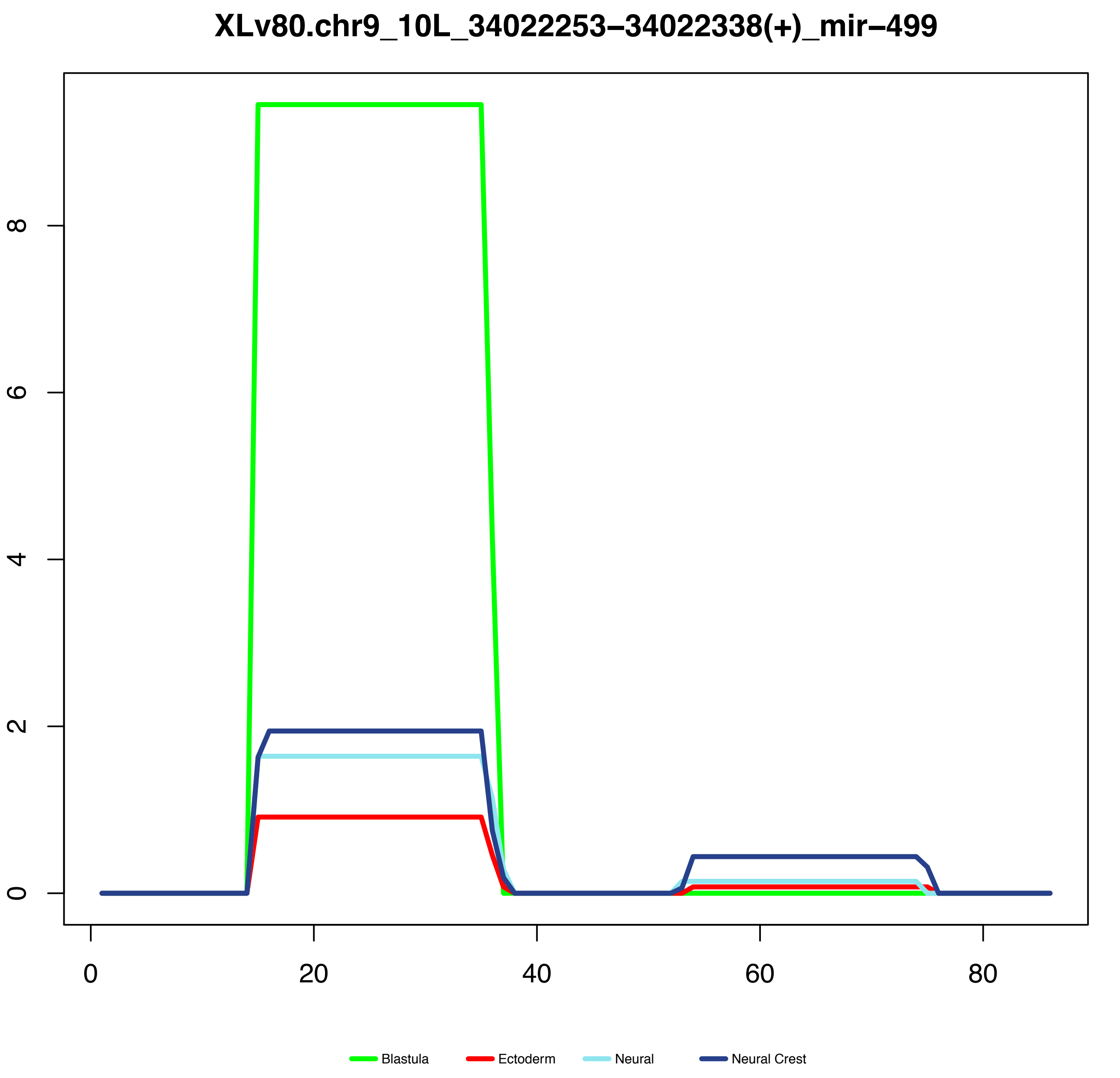
XLv80.chr9_10L_34022253−34022338(+)_mir−499
0
2
4
6
8
0
20
40
60
80
Blastula
Ectoderm
Neural
Neural Crest

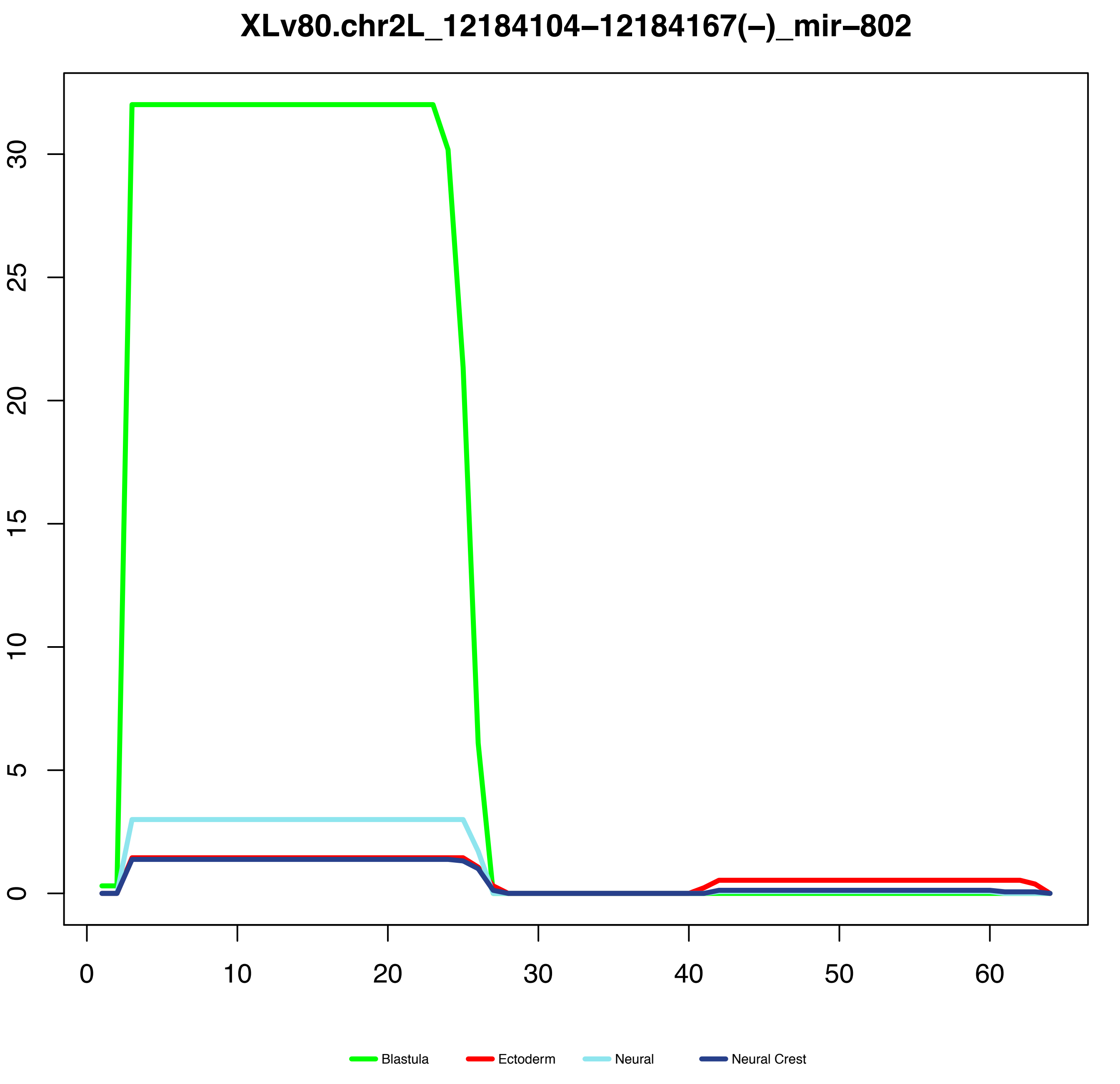
XLv80.chr2L_12184104−12184167(−)_mir−802
0
5
10
15
20
25
30
0
10
20
30
40
50
60
Blastula
Ectoderm
Neural
Neural Crest

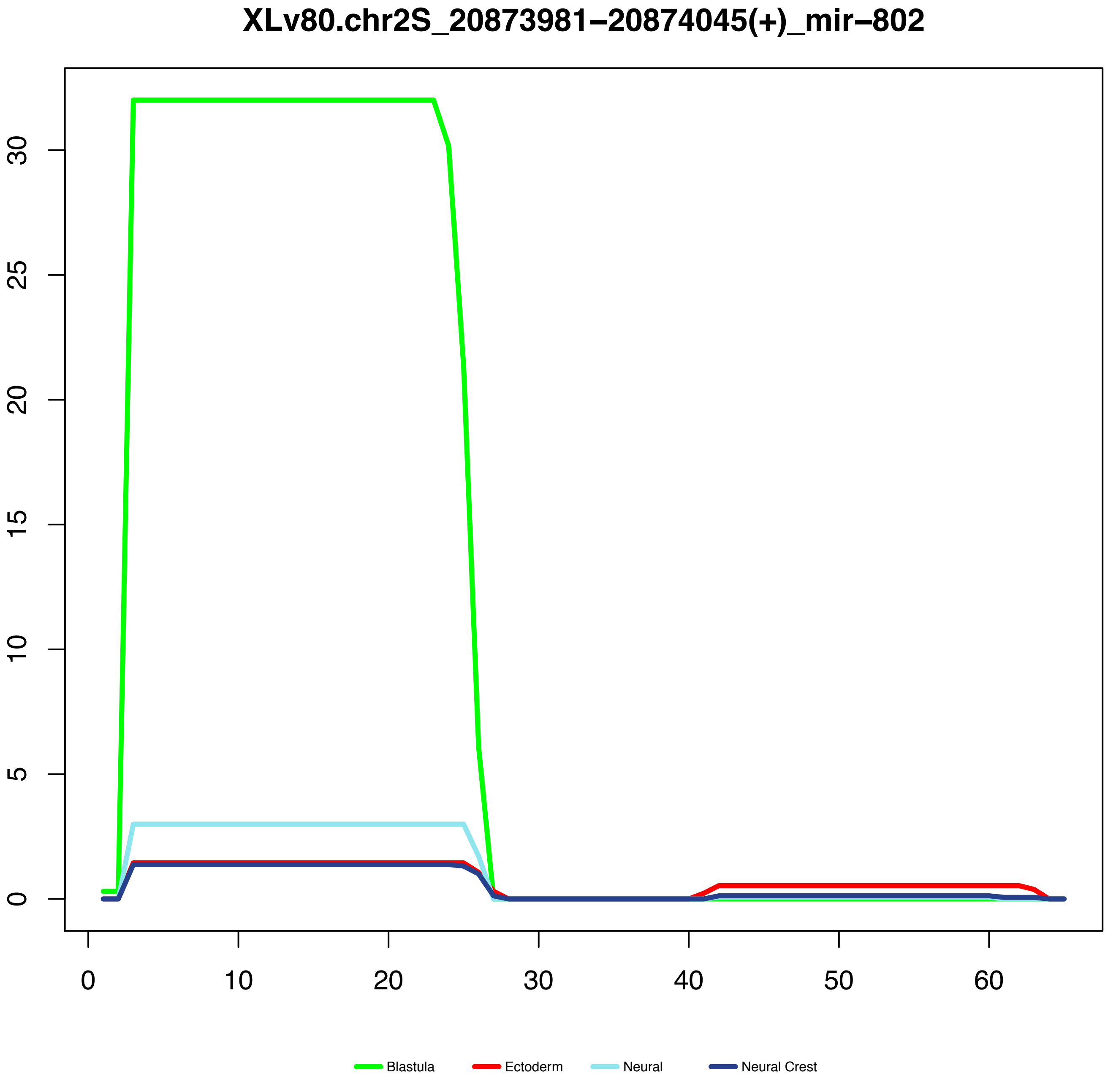

XLv80.chr2S_20873981−20874045(+)_mir−802
0
5
10
15
20
25
30
0
10
20
30
40
50
60
Blastula
Ectoderm
Neural
Neural Crest

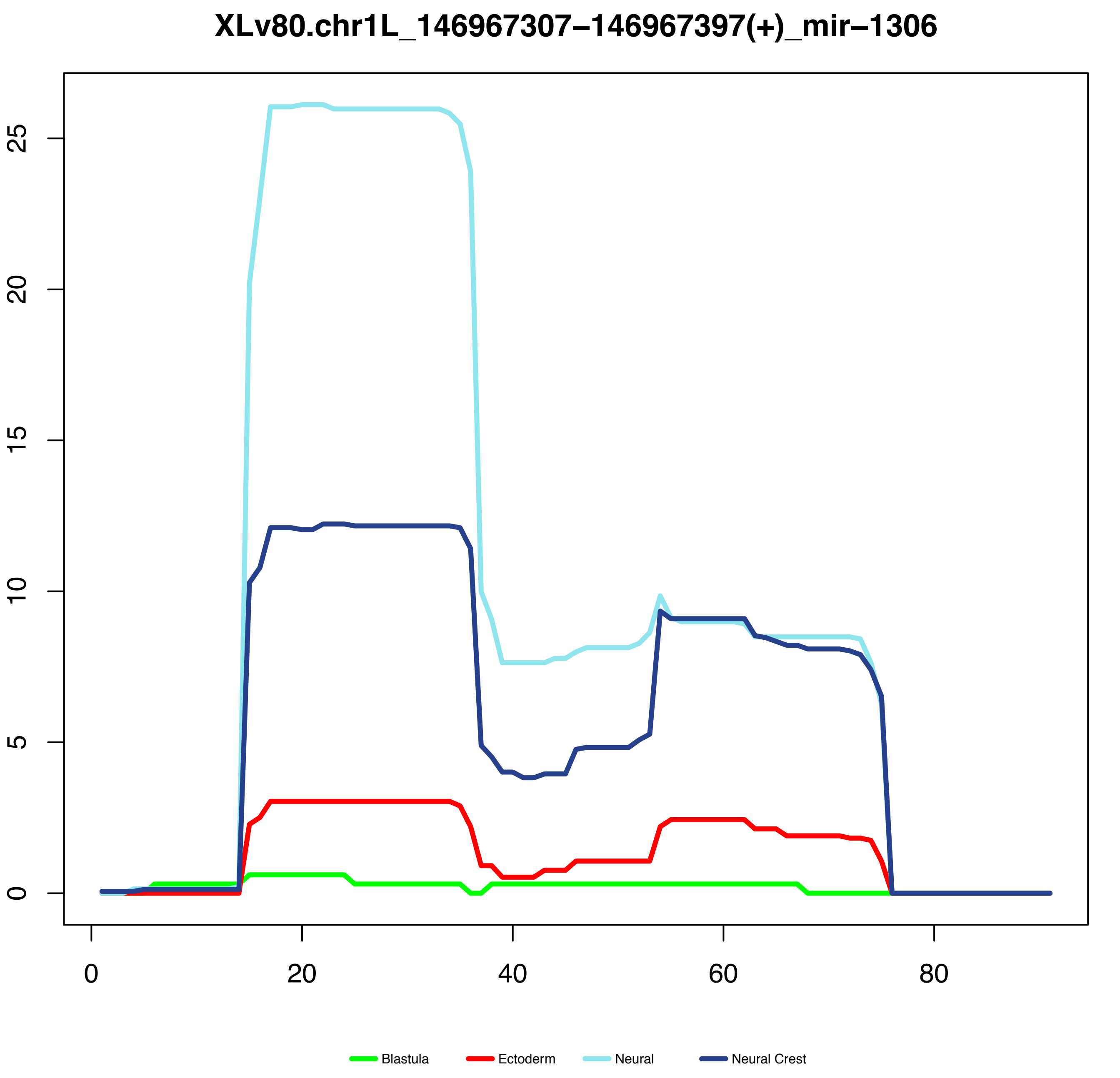

XLv80.chr1L_146967307−146967397(+)_mir−1306
0
5
10
15
20
25
0
20
40
60
80
Blastula
Ectoderm
Neural
Neural Crest

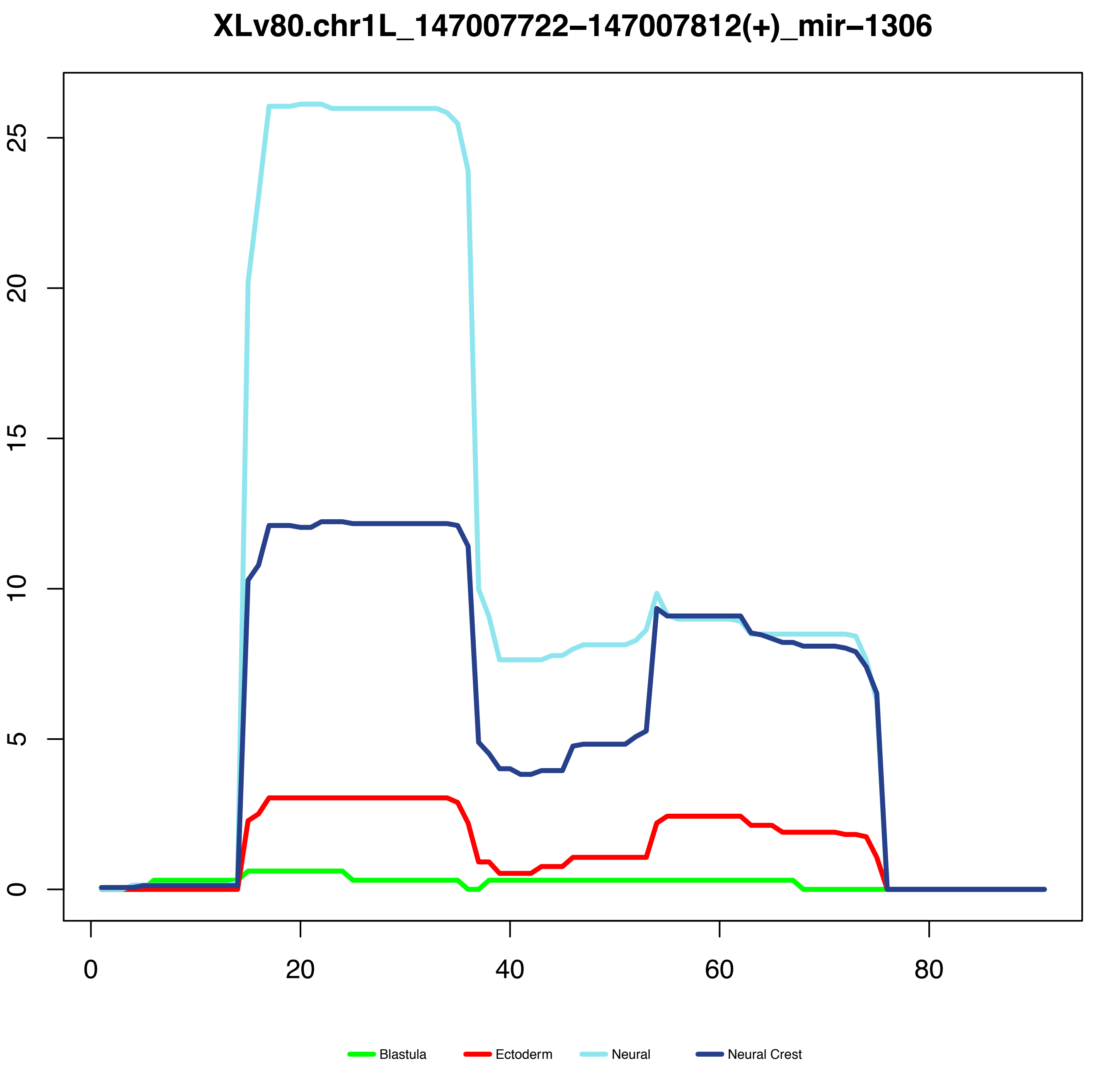

XLv80.chr1L_147007722−147007812(+)_mir−1306
0
5
10
15
20
25
0
20
40
60
80
Blastula
Ectoderm
Neural
Neural Crest

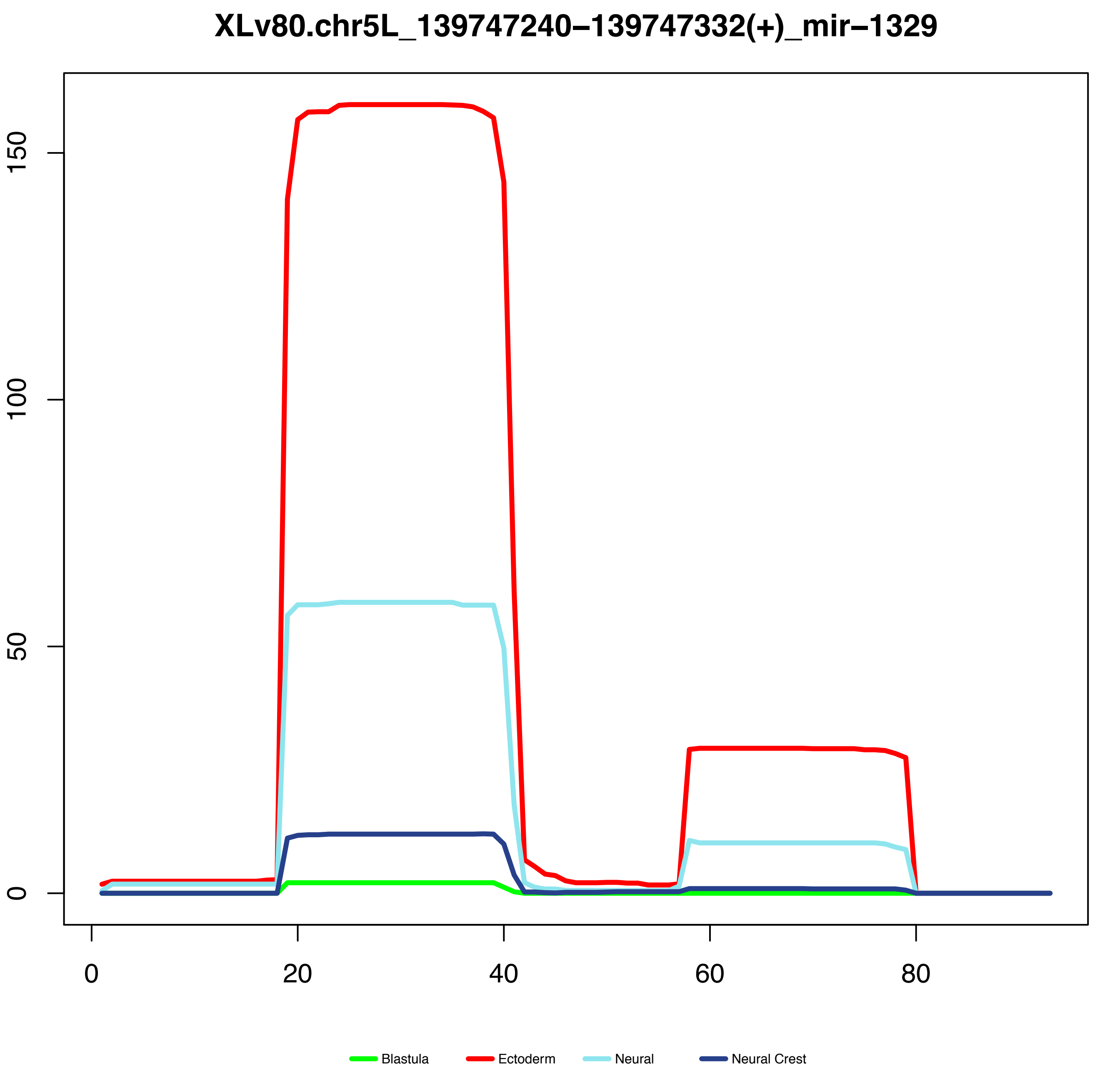

XLv80.chr5L_139747240−139747332(+)_mir−1329
0
50
100
150
0
20
40
60
80
Blastula
Ectoderm
Neural
Neural Crest

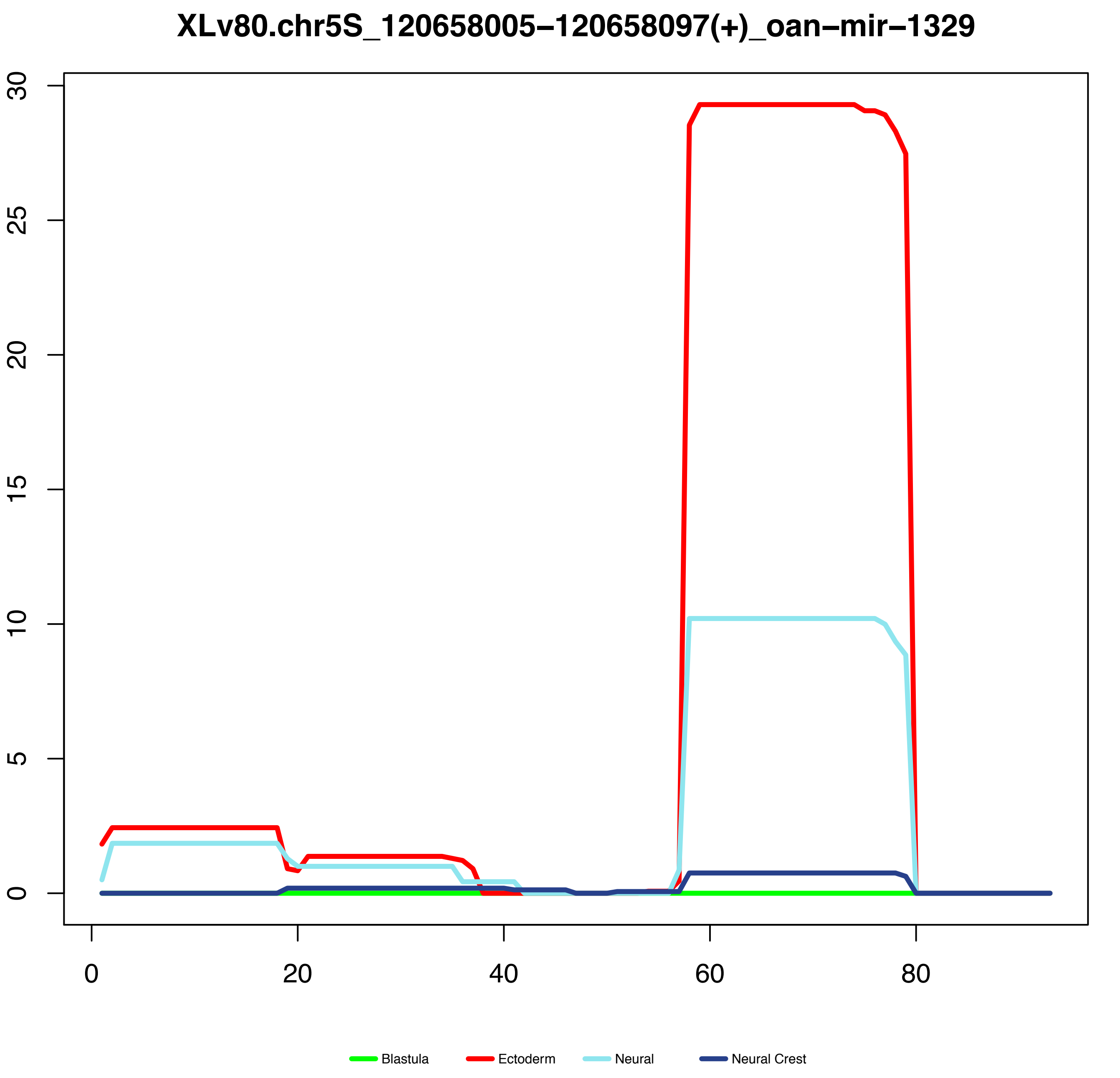

XLv80.chr5S_120658005−120658097(+)_oan−mir−1329
0
5
10
15
20
25
30
0
20
40
60
80
Blastula
Ectoderm
Neural
Neural Crest

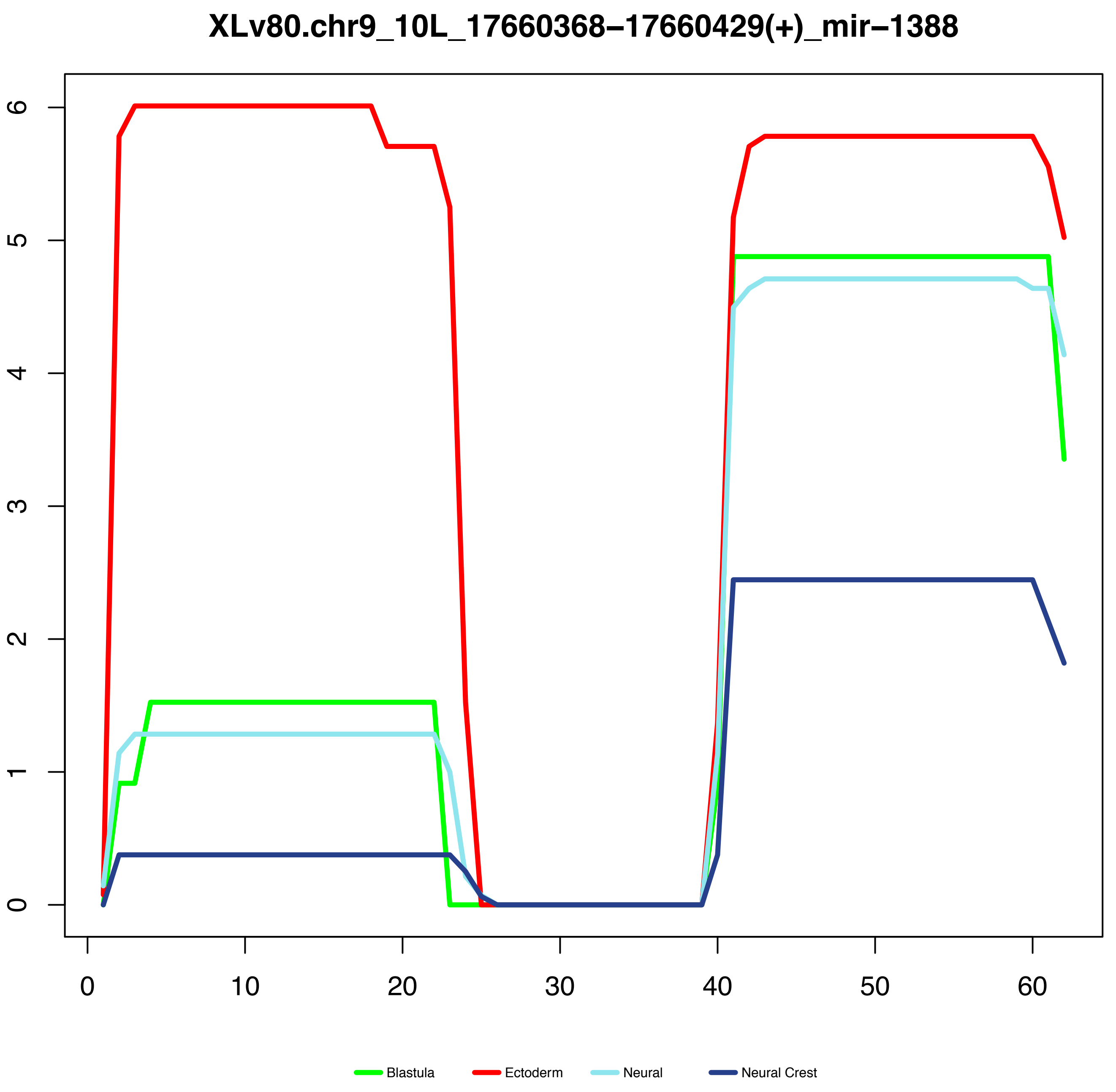

XLv80.chr9_10L_17660368−17660429(+)_mir−1388
0
1
2
3
4
5
6
0
10
20
30
40
50
60
Blastula
Ectoderm
Neural
Neural Crest

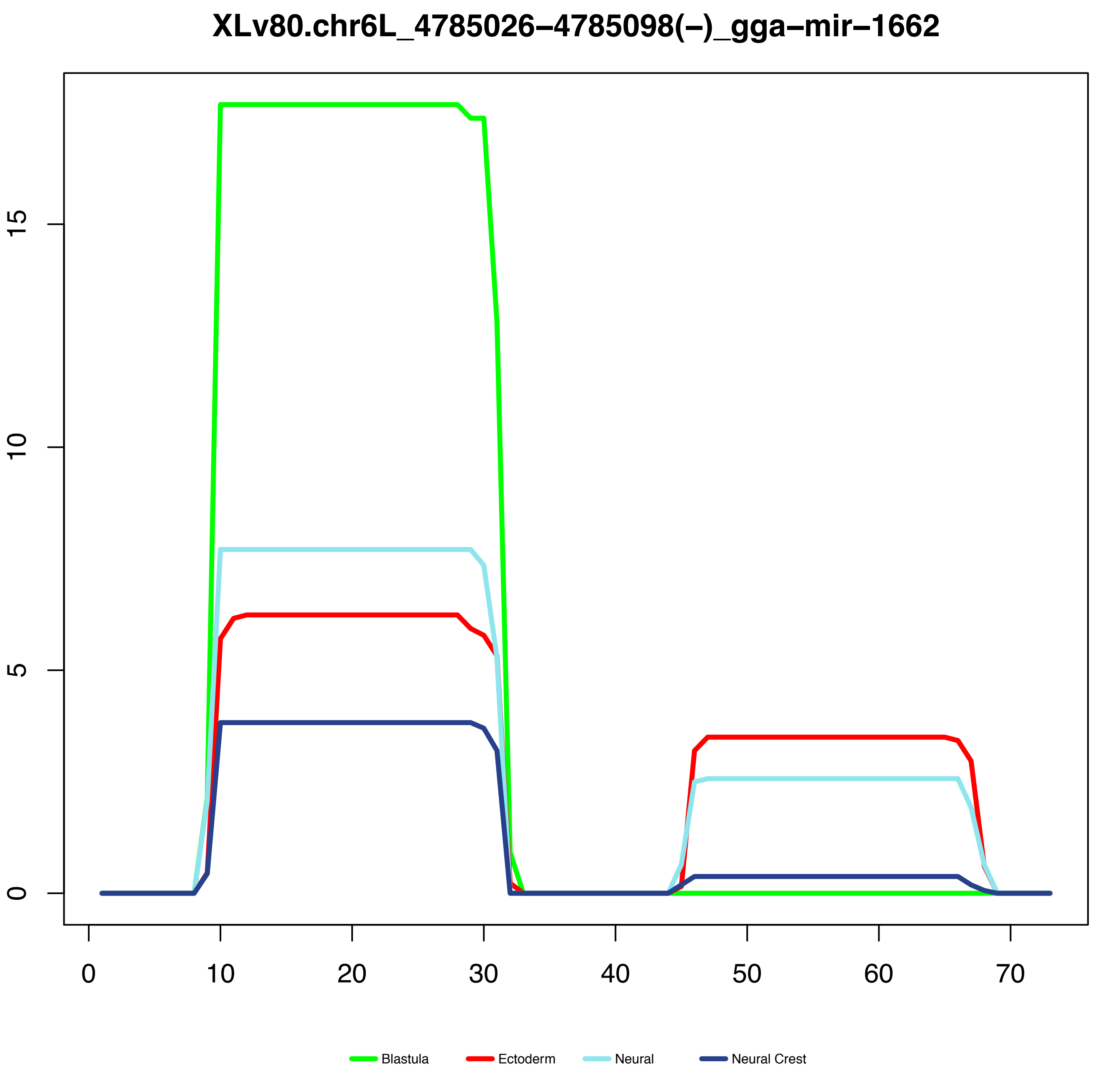
XLv80.chr6L_4785026–4785098(–)_gga–mir–1662
0
5
10
15
0
10
20
30
40
50
60
70
Blastula
Ectoderm
Neural
Neural Crest

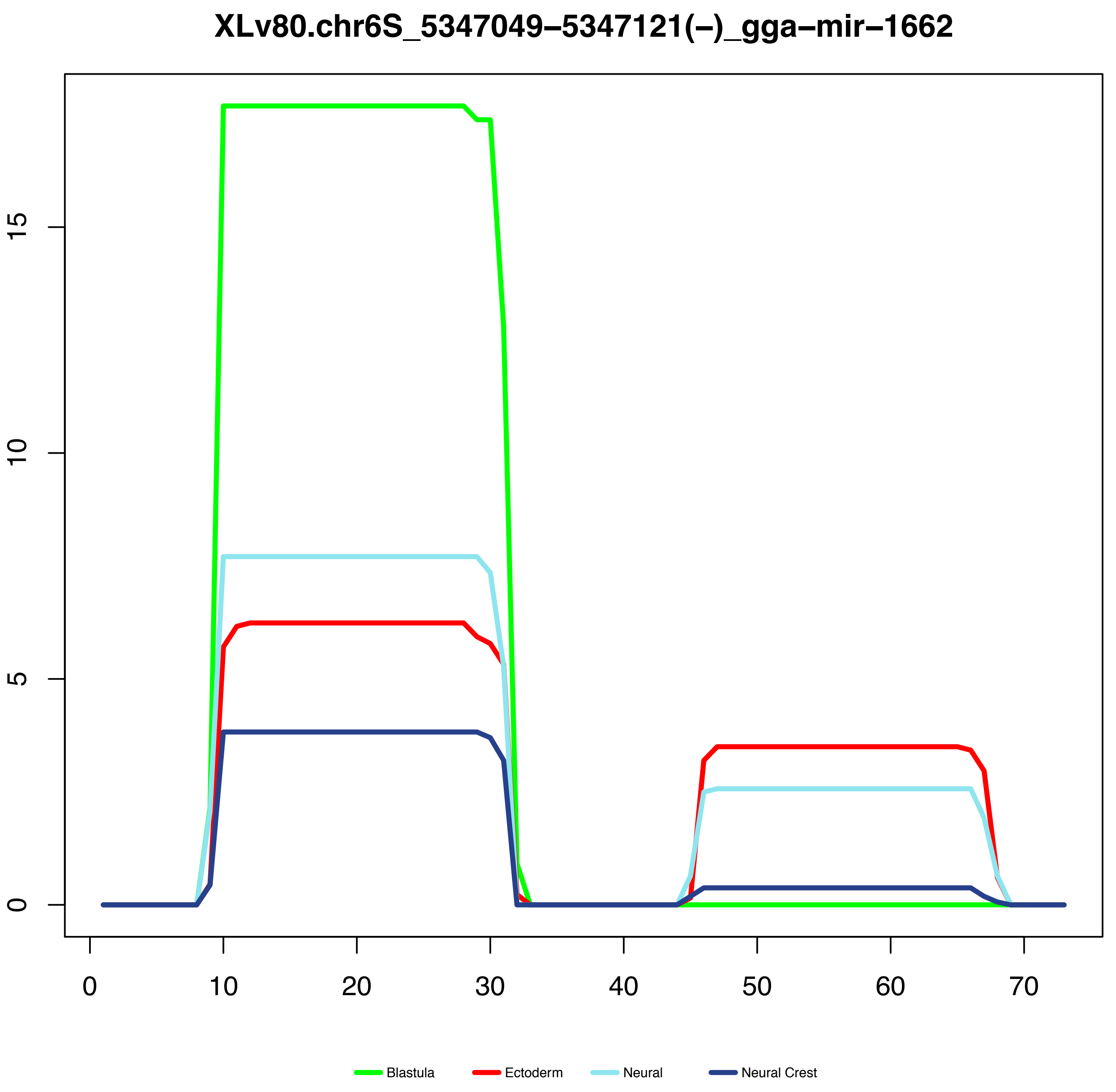

XLv80.chr6S_5347049–5347121(–)_gga–mir–1662
0
5
10
15
0
10
20
30
40
50
60
70
Blastula
Ectoderm
Neural
Neural Crest

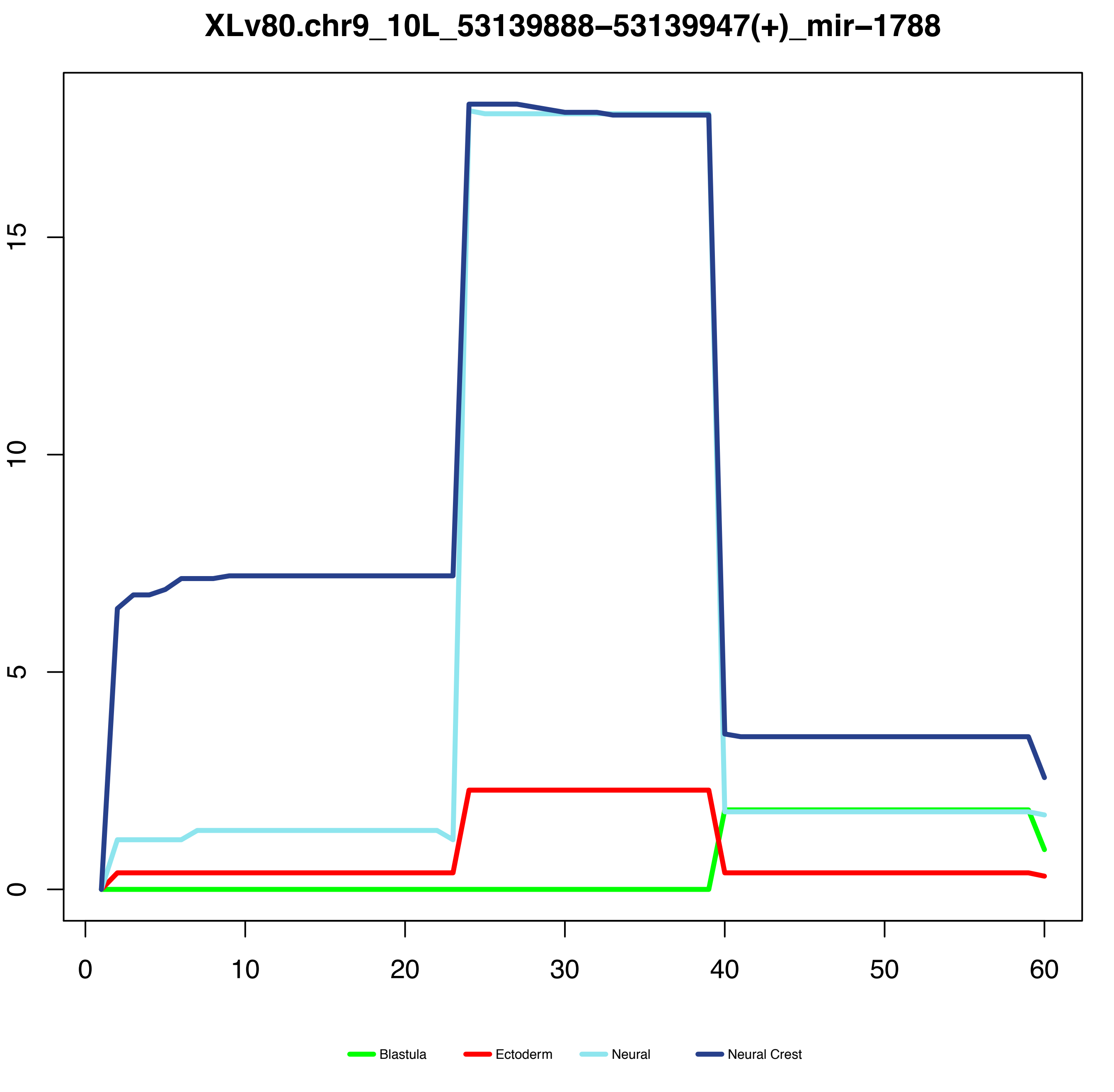

XLv80.chr9_10L_53139888−53139947(+)_mir−1788
0
5
10
15
0
10
20
30
40
50
60
Blastula
Ectoderm
Neural
Neural Crest

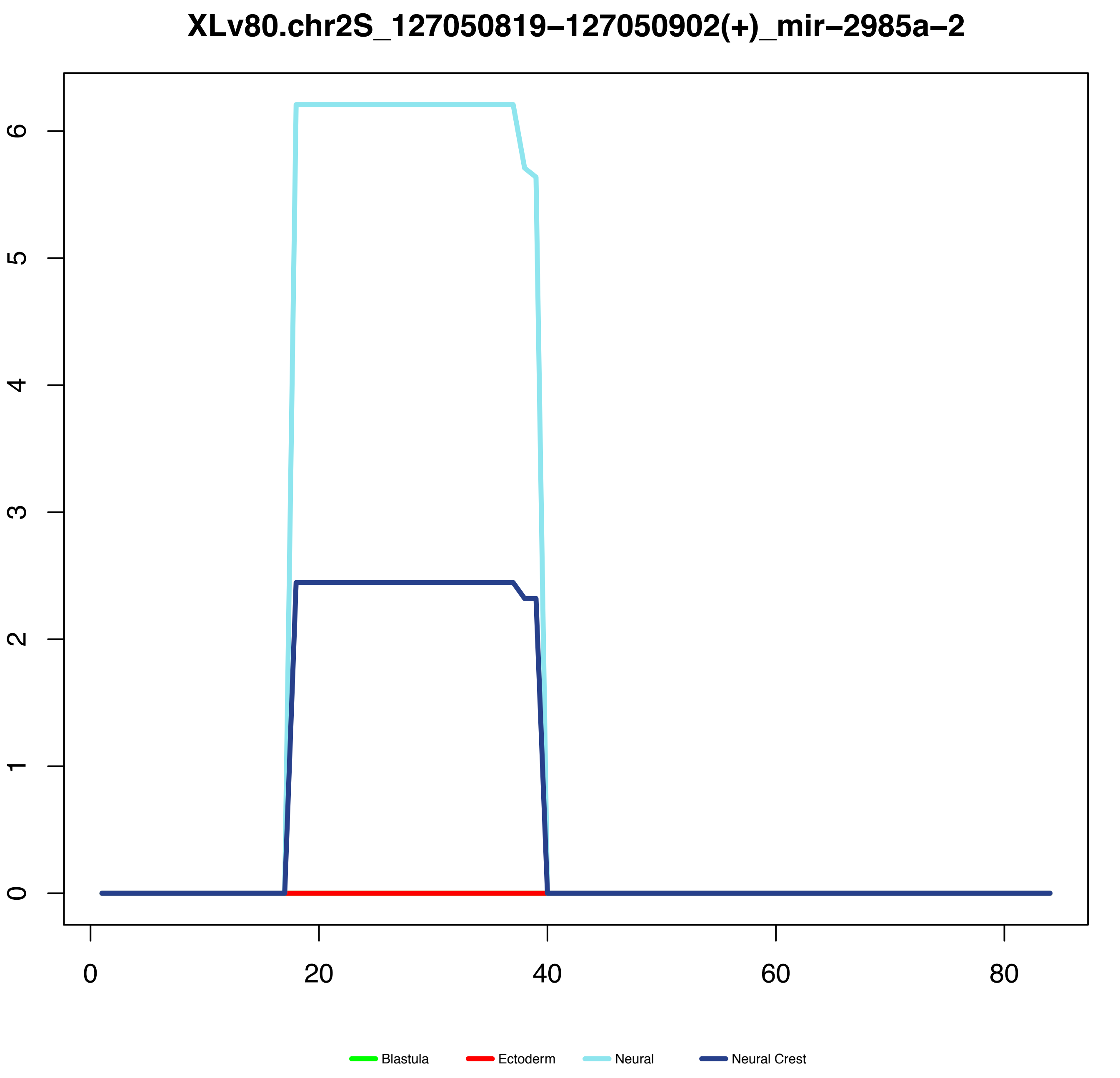

XLv80.chr2S_127050819–127050902(+)_mir–2985a–2
0
1
2
3
4
5
6
0
20
40
60
80
Blastula
Ectoderm
Neural
Neural Crest

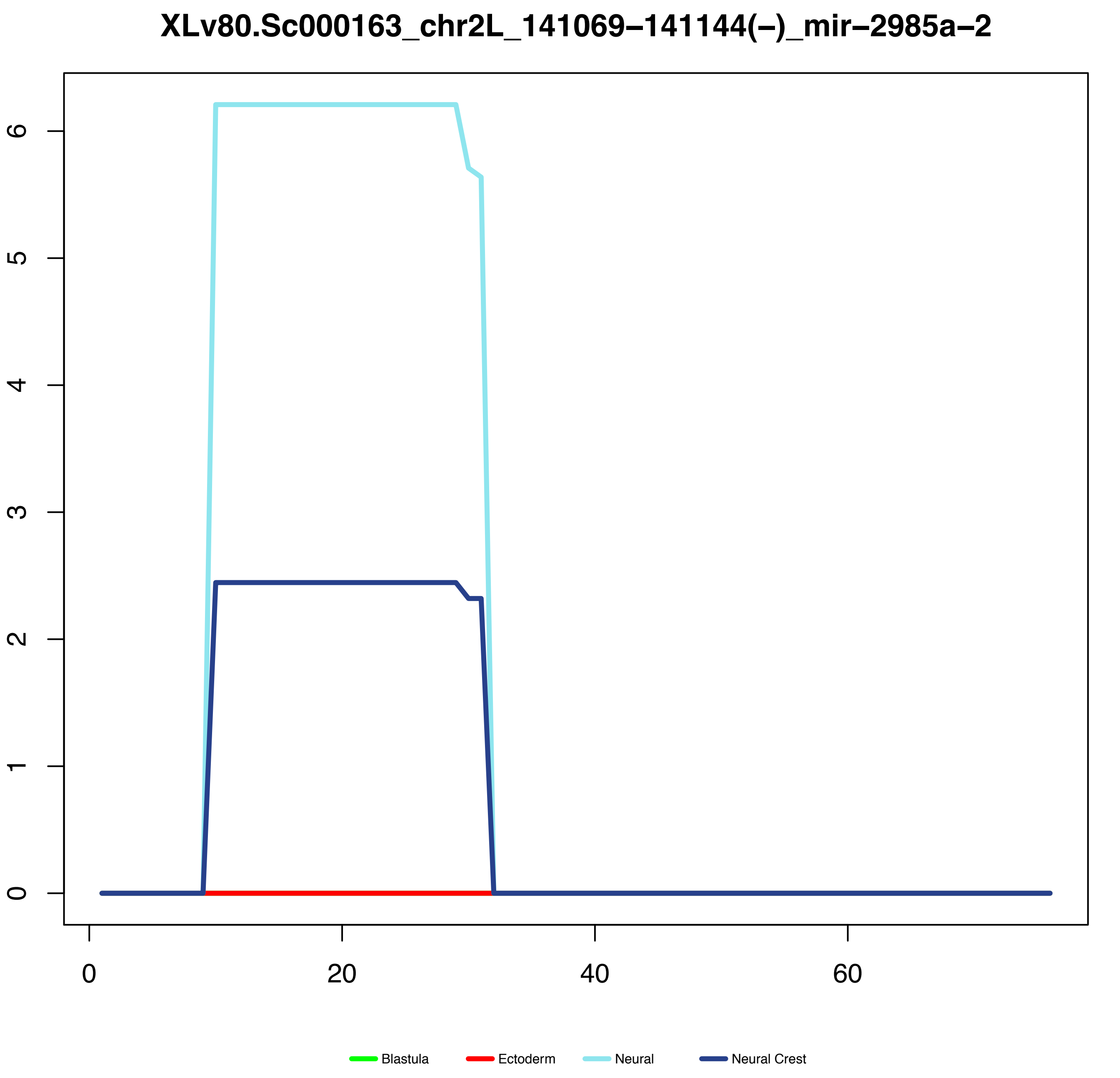
XLv80.Sc000163_chr2L_141069−141144(−)_mir−2985a−2
0
1
2
3
4
5
6
0
20
40
60
Blastula
Ectoderm
Neural
Neural Crest

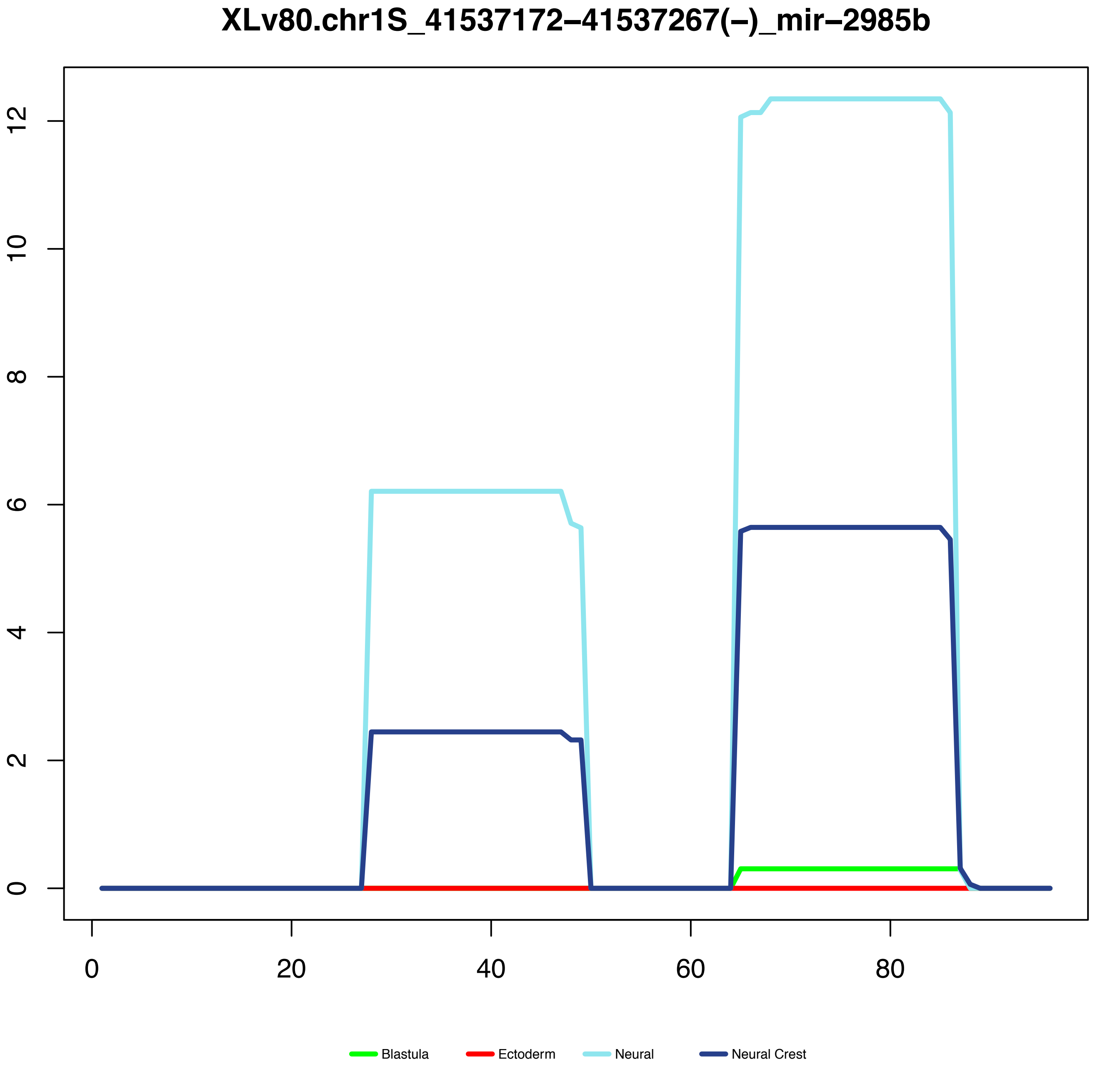

XLv80.chr1S_41537172−41537267(−)_mir−2985b
0
2
4
6
8
10
12
0
20
40
60
80
Blastula
Ectoderm
Neural
Neural Crest

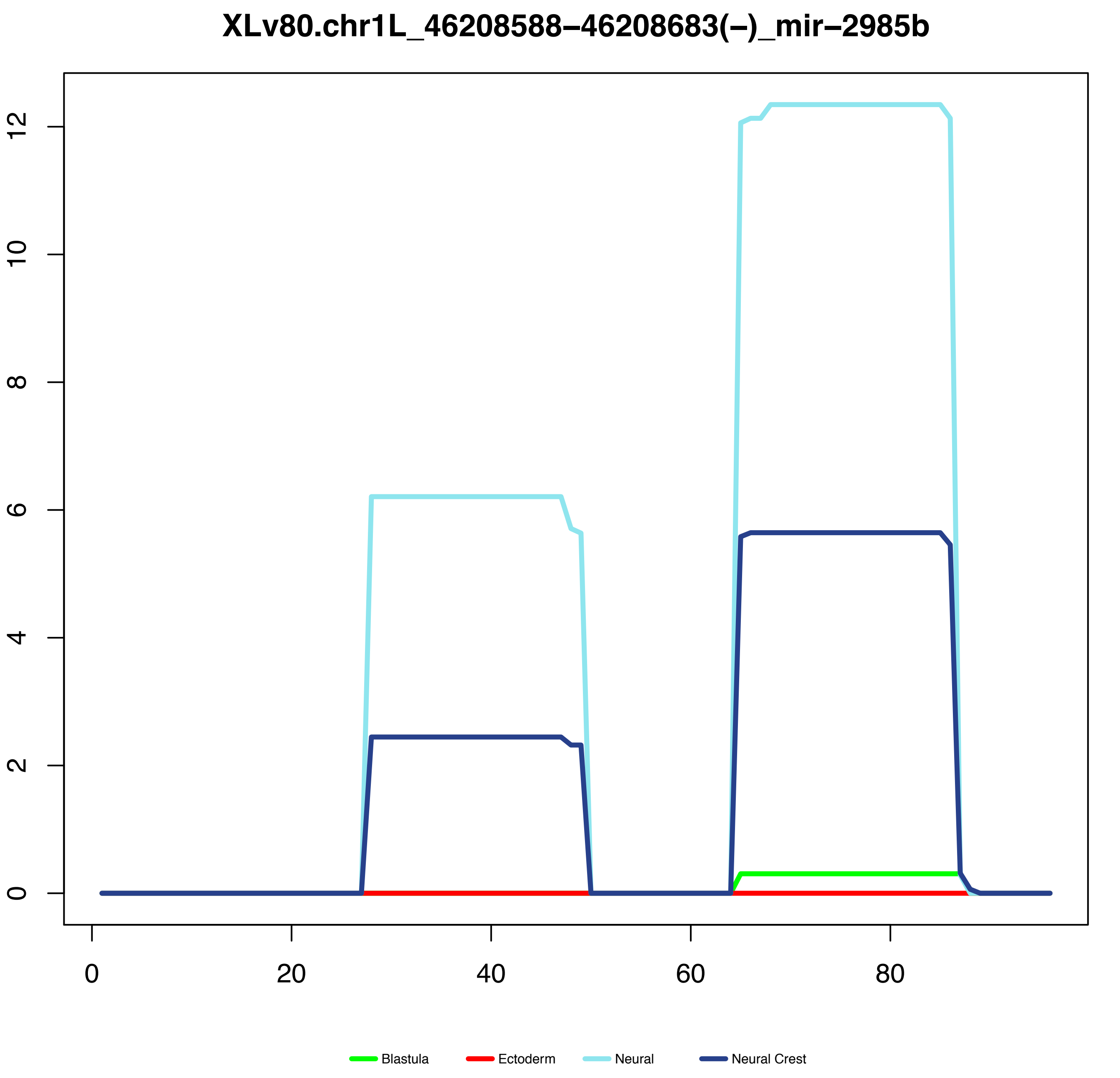
XLv80.chr1L_46208588−46208683(−)_mir−2985b
0
2
4
6
8
10
12
0
20
40
60
80
Blastula
Ectoderm
Neural
Neural Crest

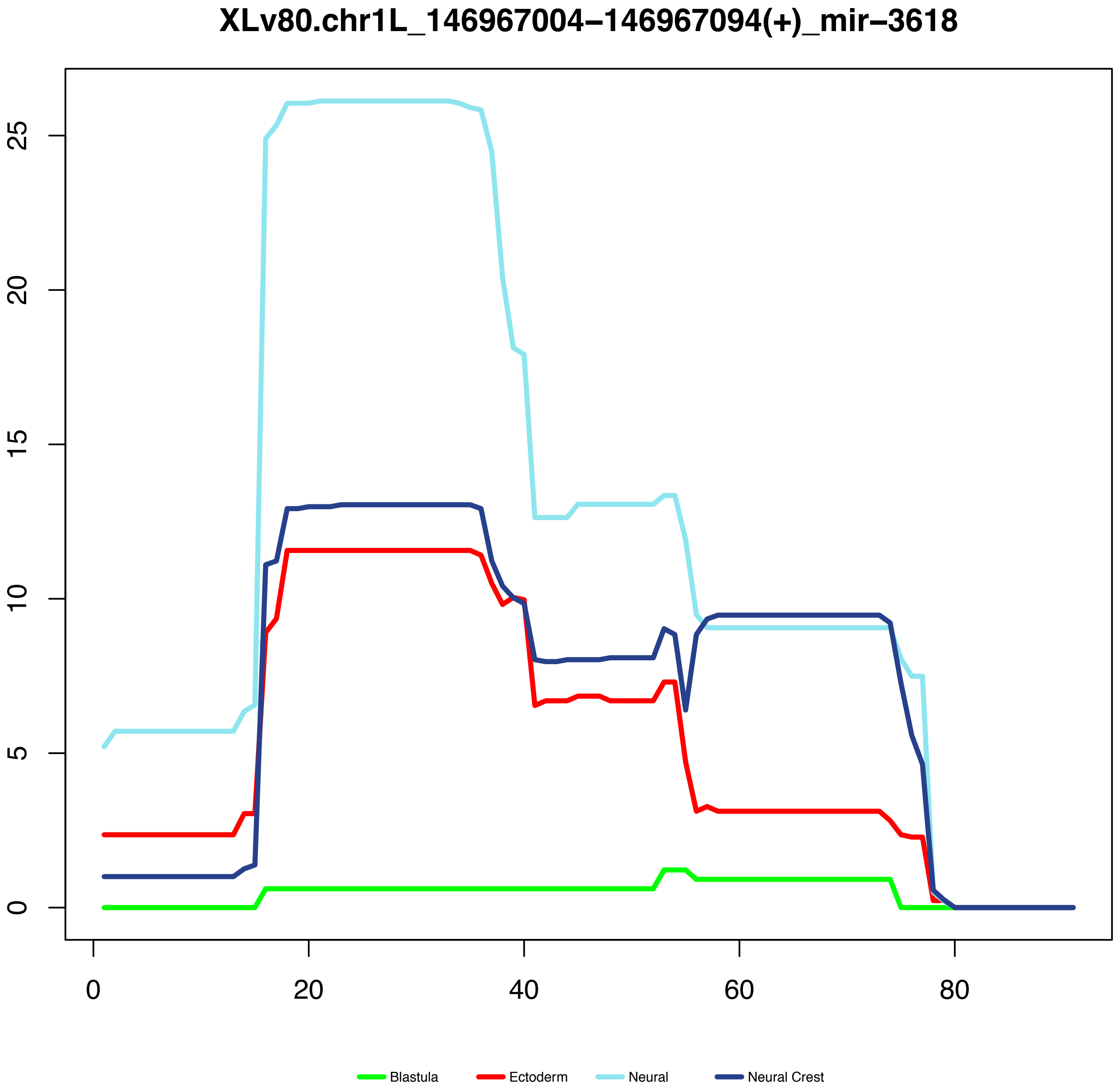

XLv80.chr1L_146967004−146967094(+)_mir−3618
0
5
10
15
20
25
0
20
40
60
80
Blastula
Ectoderm
Neural
Neural Crest

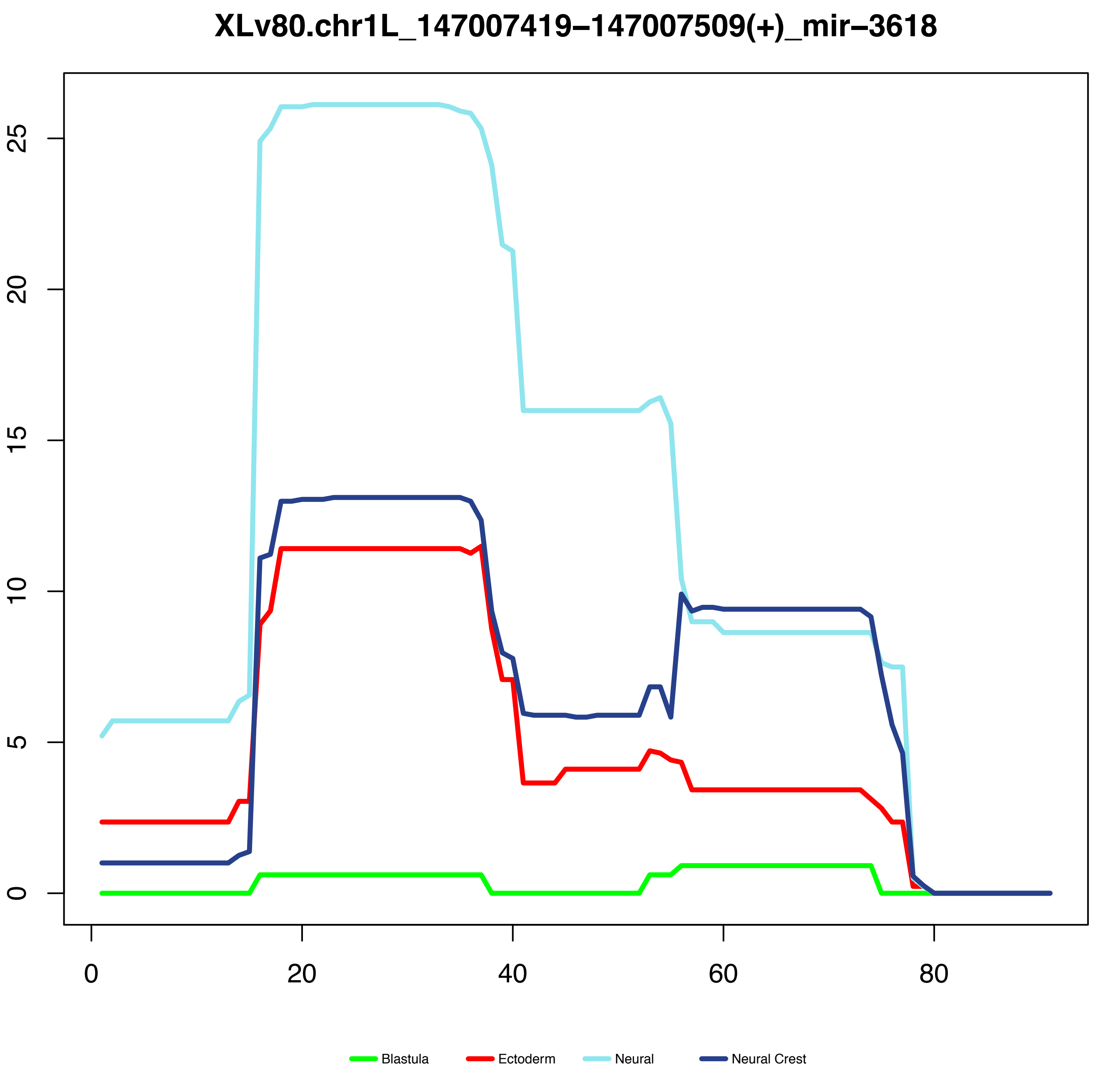

XLv80.chr1L_147007419−147007509(+)_mir−3618
0
5
10
15
20
25
0
20
40
60
80
Blastula
Ectoderm
Neural
Neural Crest

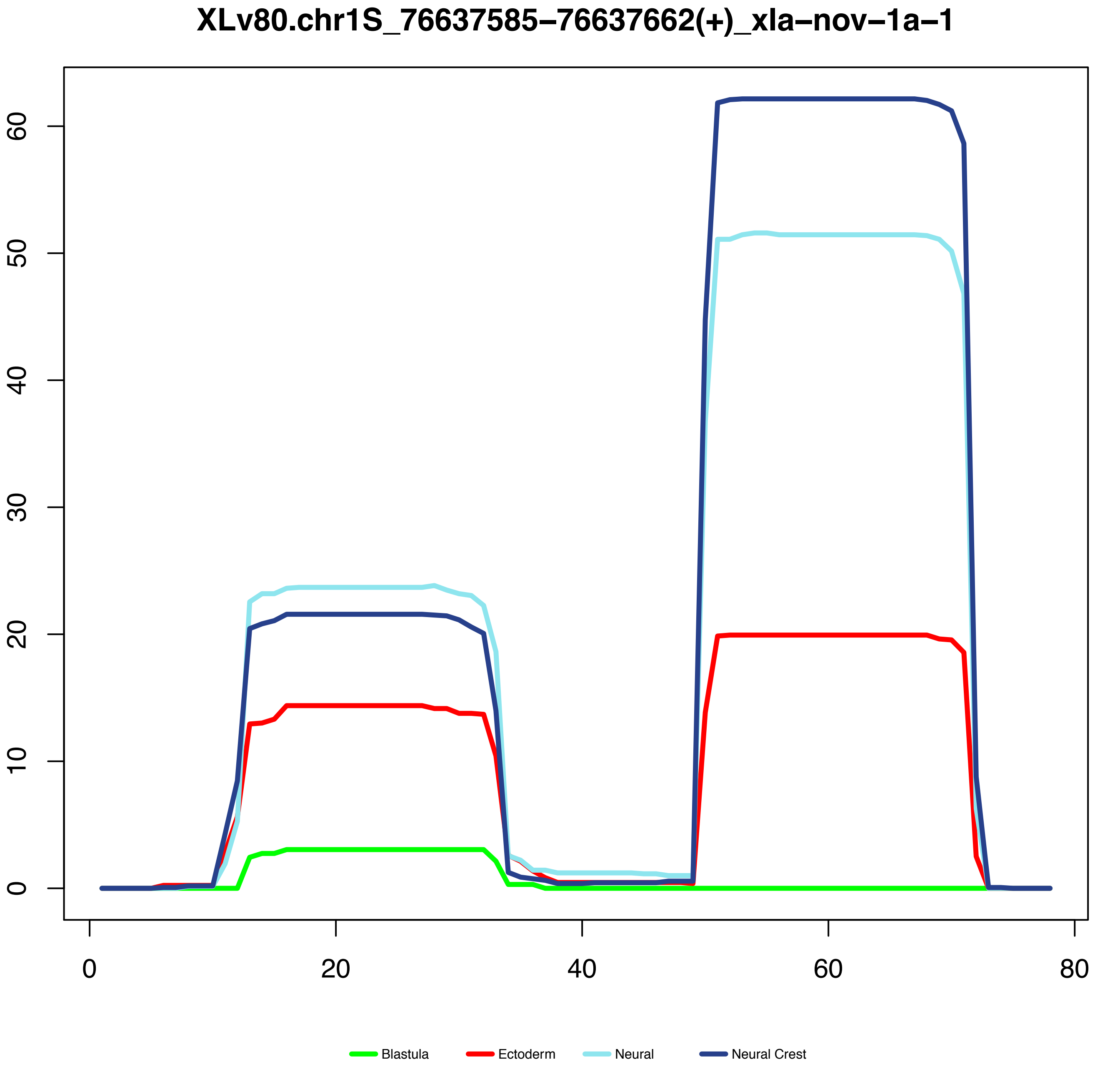

XLv80.chr1S_76637585−76637662(+)_xla−nov−1a−1
0
10
20
30
40
50
60
0
20
40
60
80
Blastula
Ectoderm
Neural
Neural Crest

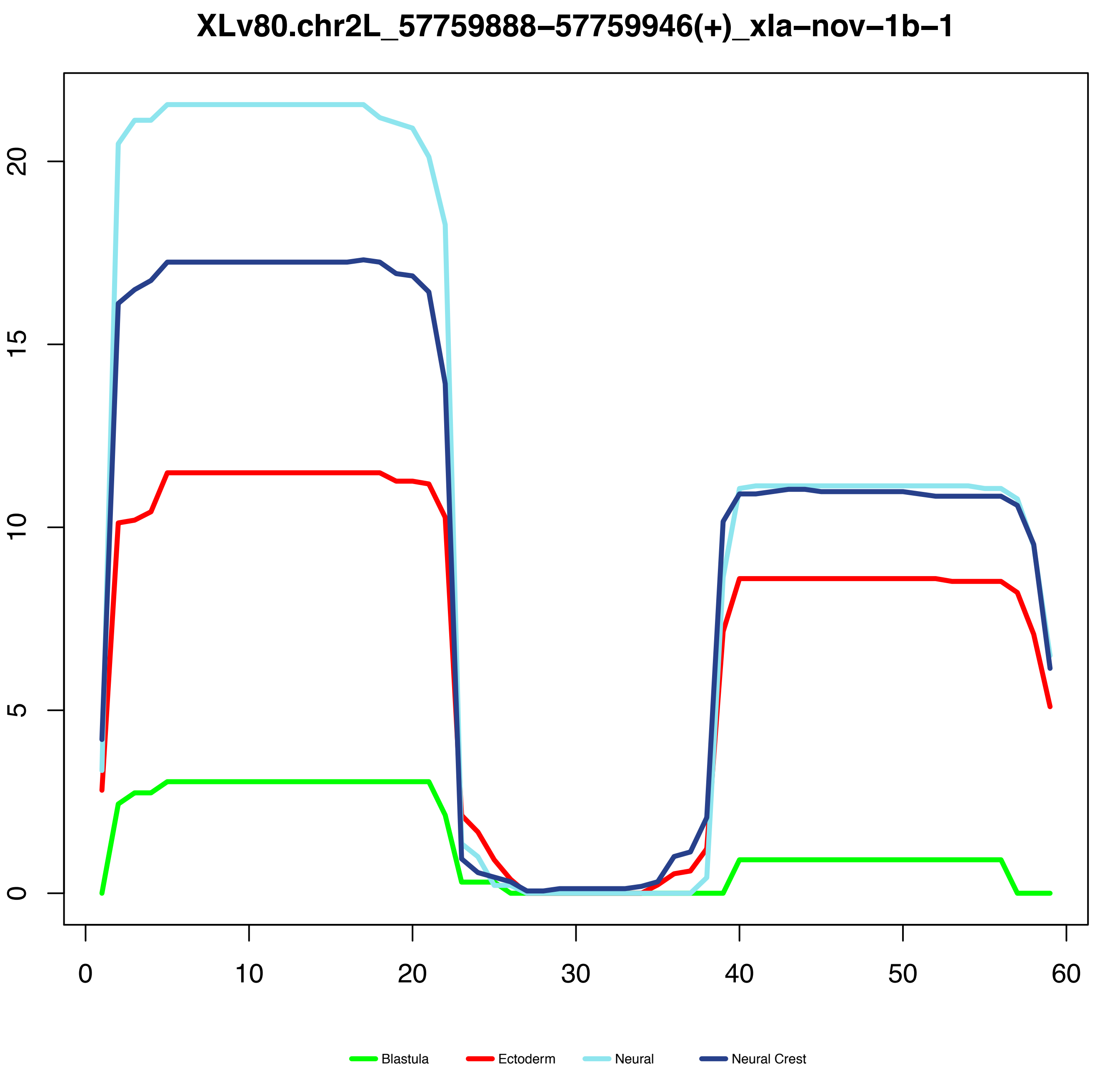
XLv80.chr2L_57759888−57759946(+)_xla−nov−1b−1
0
5
10
15
20
0
10
20
30
40
50
60
Blastula
Ectoderm
Neural
Neural Crest

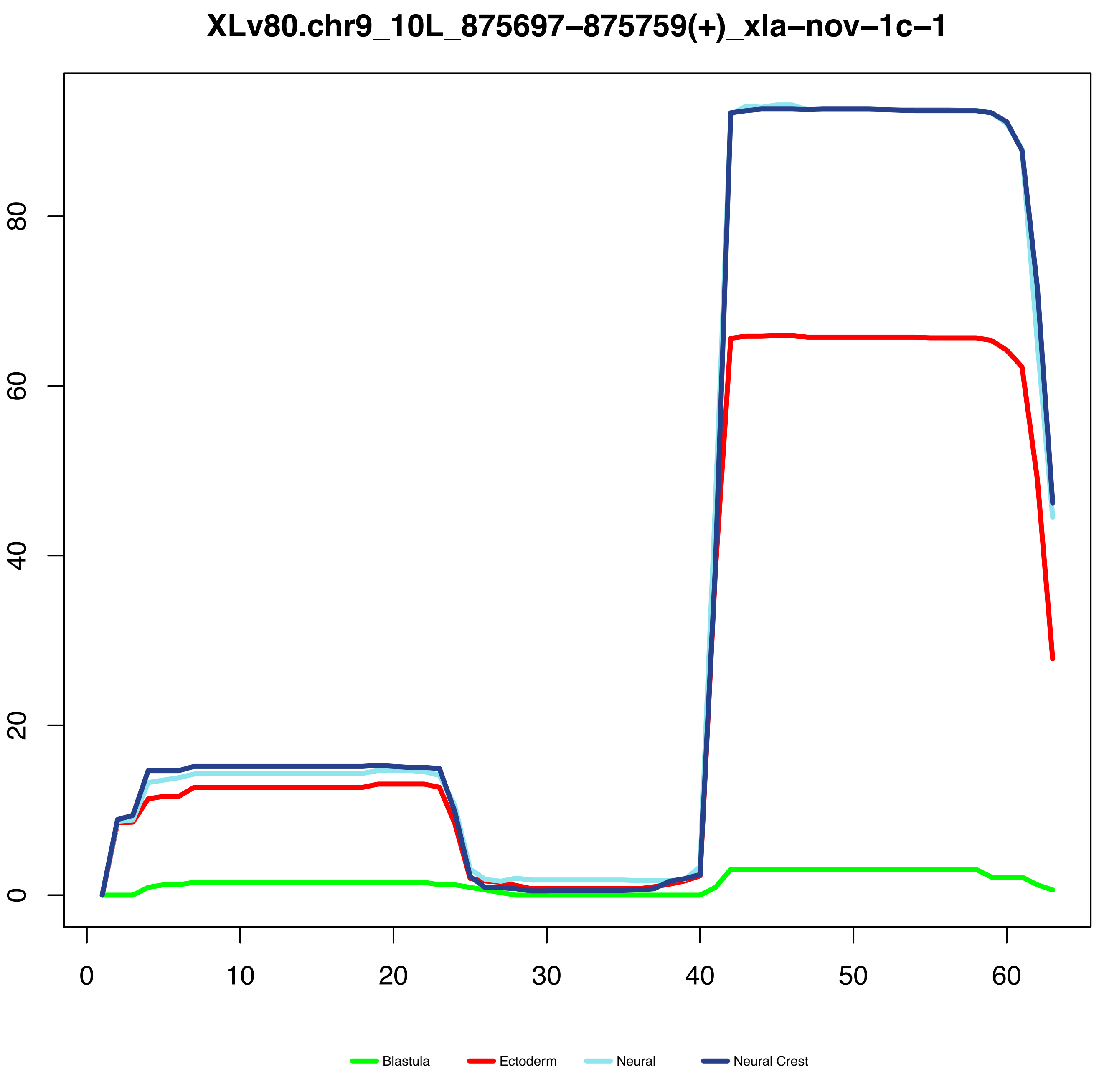
XLv80.chr9_10L_875697−875759(+)_xla−nov−1c−1
0
20
40
60
80
0
10
20
30
40
50
60
Blastula
Ectoderm
Neural
Neural Crest

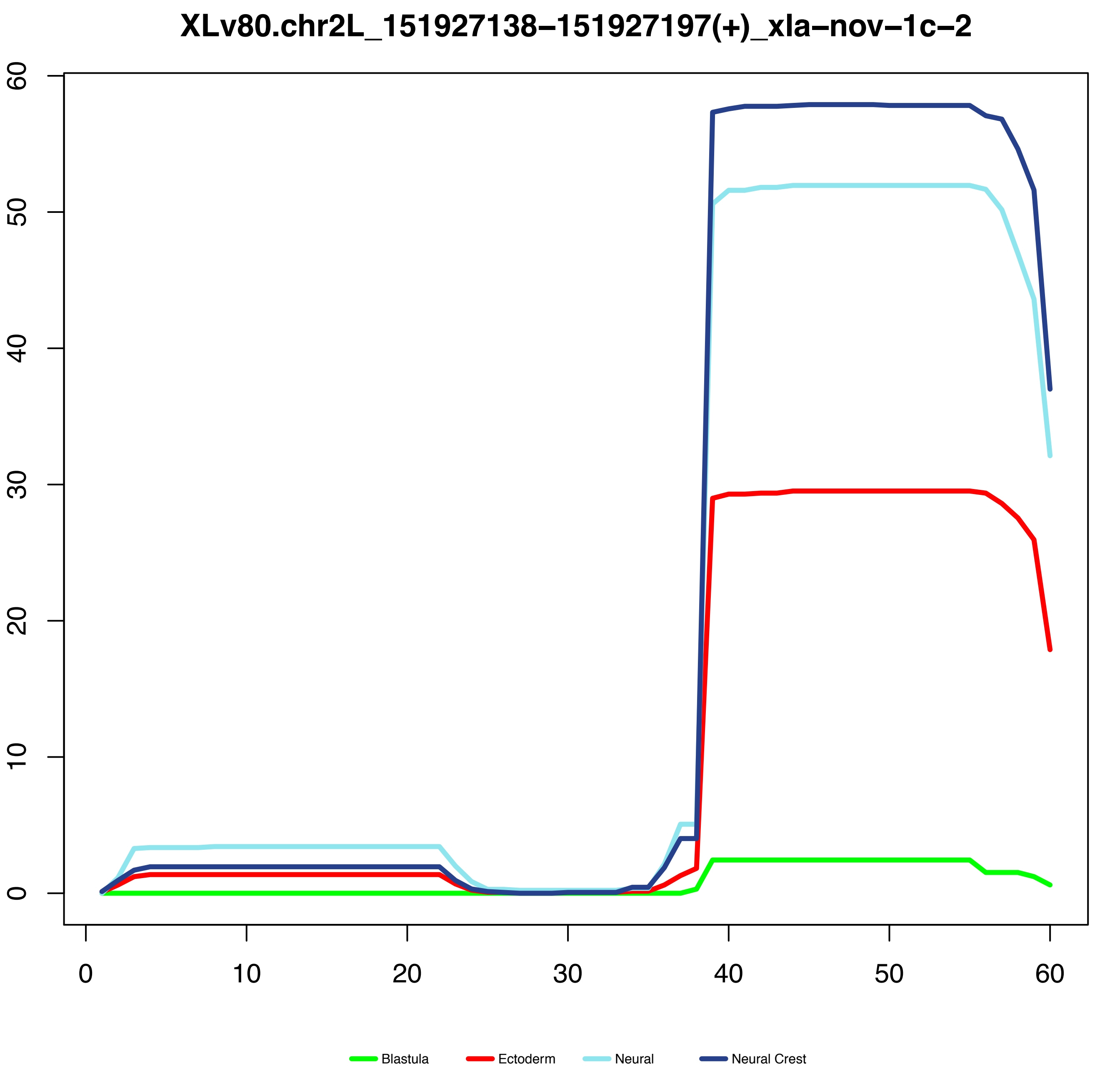

XLv80.chr2L_151927138−151927197(+)_xla−nov−1c−2
0
10
20
30
40
50
60
0
10
20
30
40
50
60
Blastula
Ectoderm
Neural
Neural Crest

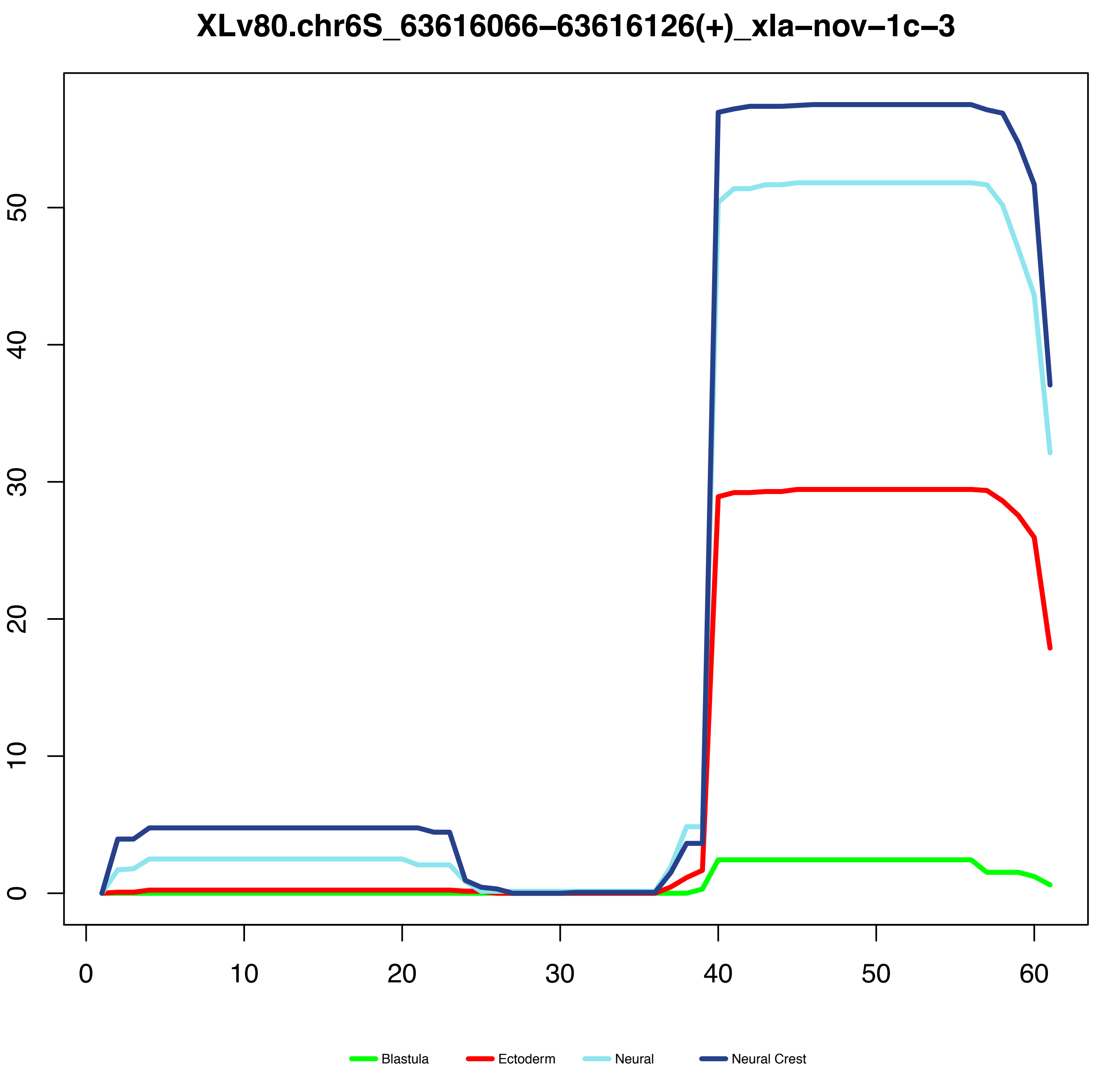

XLv80.chr6S_63616066–63616126(+)_xla–nov–1c–3
0
10
20
30
40
50
60
0
10
20
30
40
50
Blastula
Ectoderm
Neural
Neural Crest

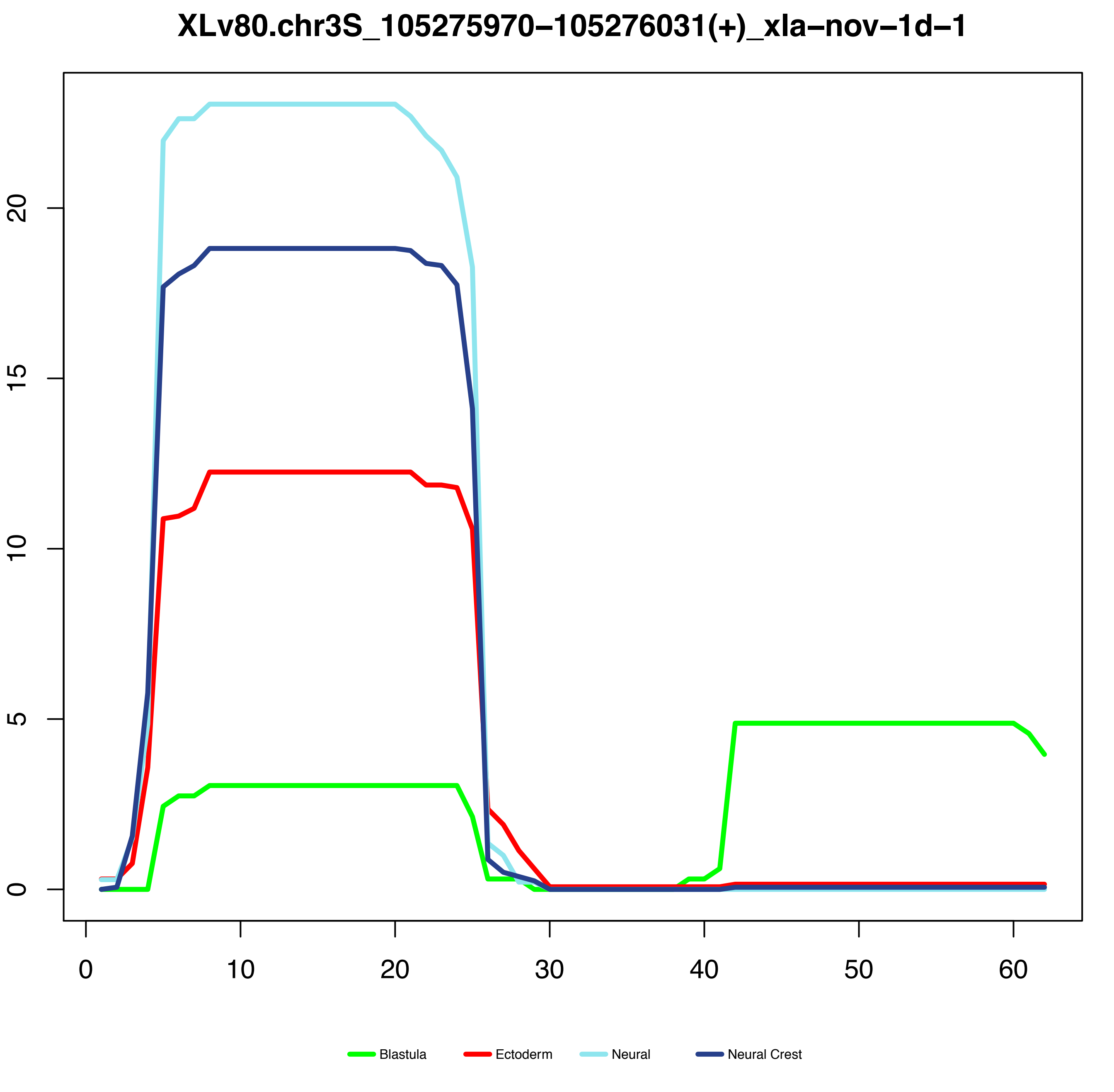
XLv80.chr3S_105275970−105276031(+)_xla−nov−1d−1
0
5
10
15
20
0
10
20
30
40
50
60
Blastula
Ectoderm
Neural
Neural Crest

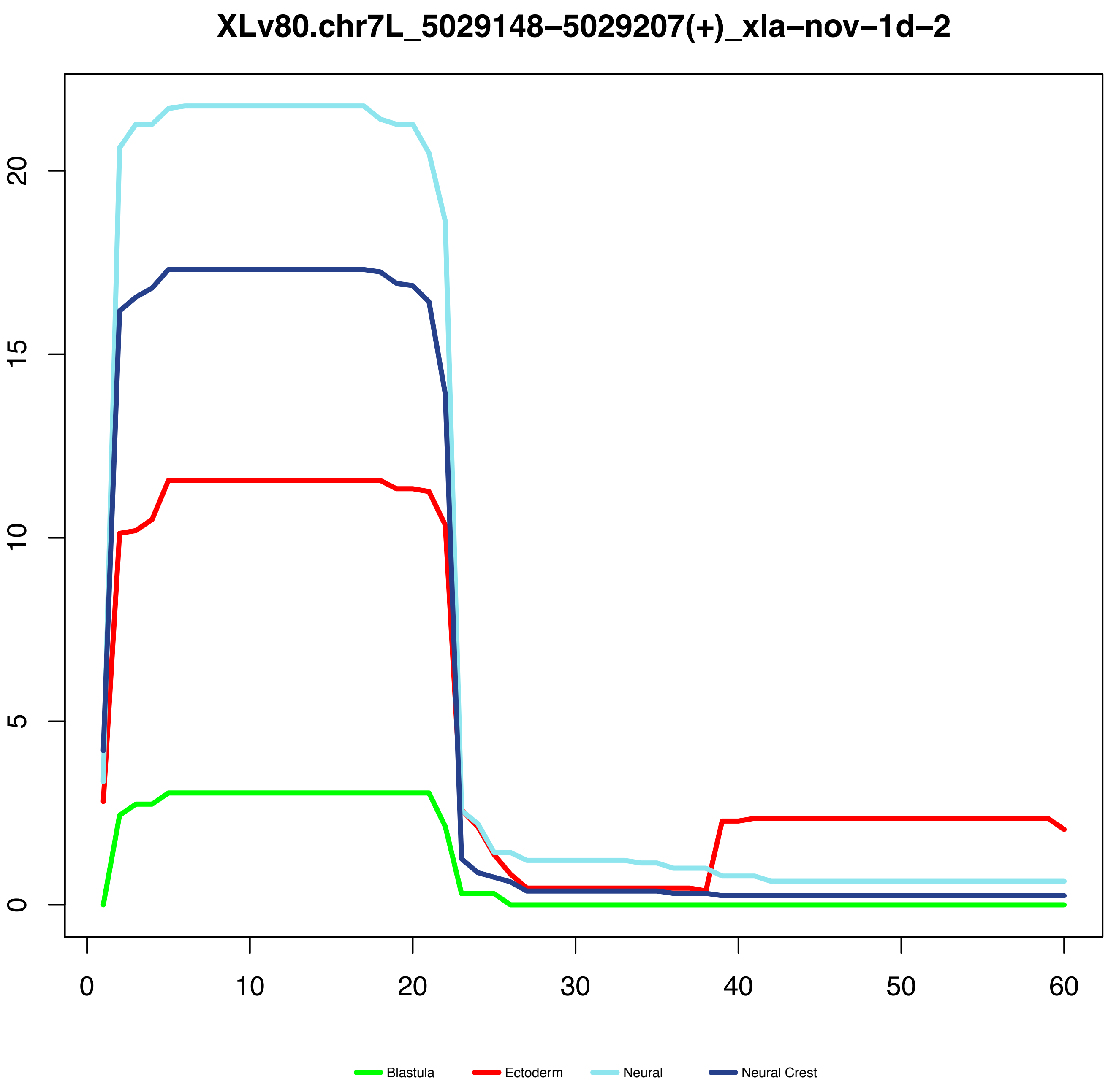

XLv80.chr7L_5029148−5029207(+)_xla−nov−1d−2
0
5
10
15
20
0
10
20
30
40
50
60
Blastula
Ectoderm
Neural
Neural Crest

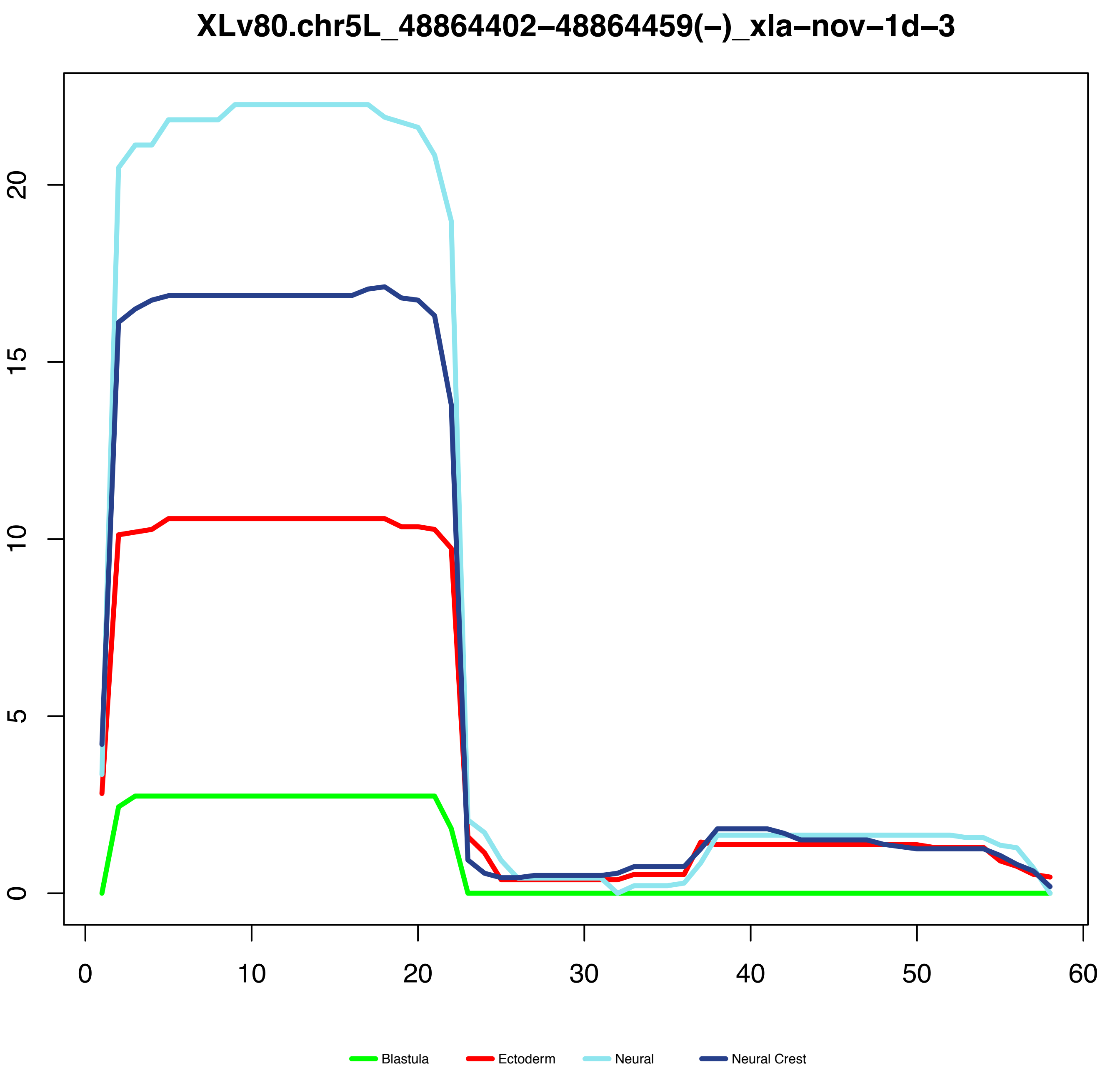

XLv80.chr5L_48864402–48864459(–)_xla–nov–1d–3
0
5
10
15
20
0
10
20
30
40
50
60
Blastula
Ectoderm
Neural
Neural Crest

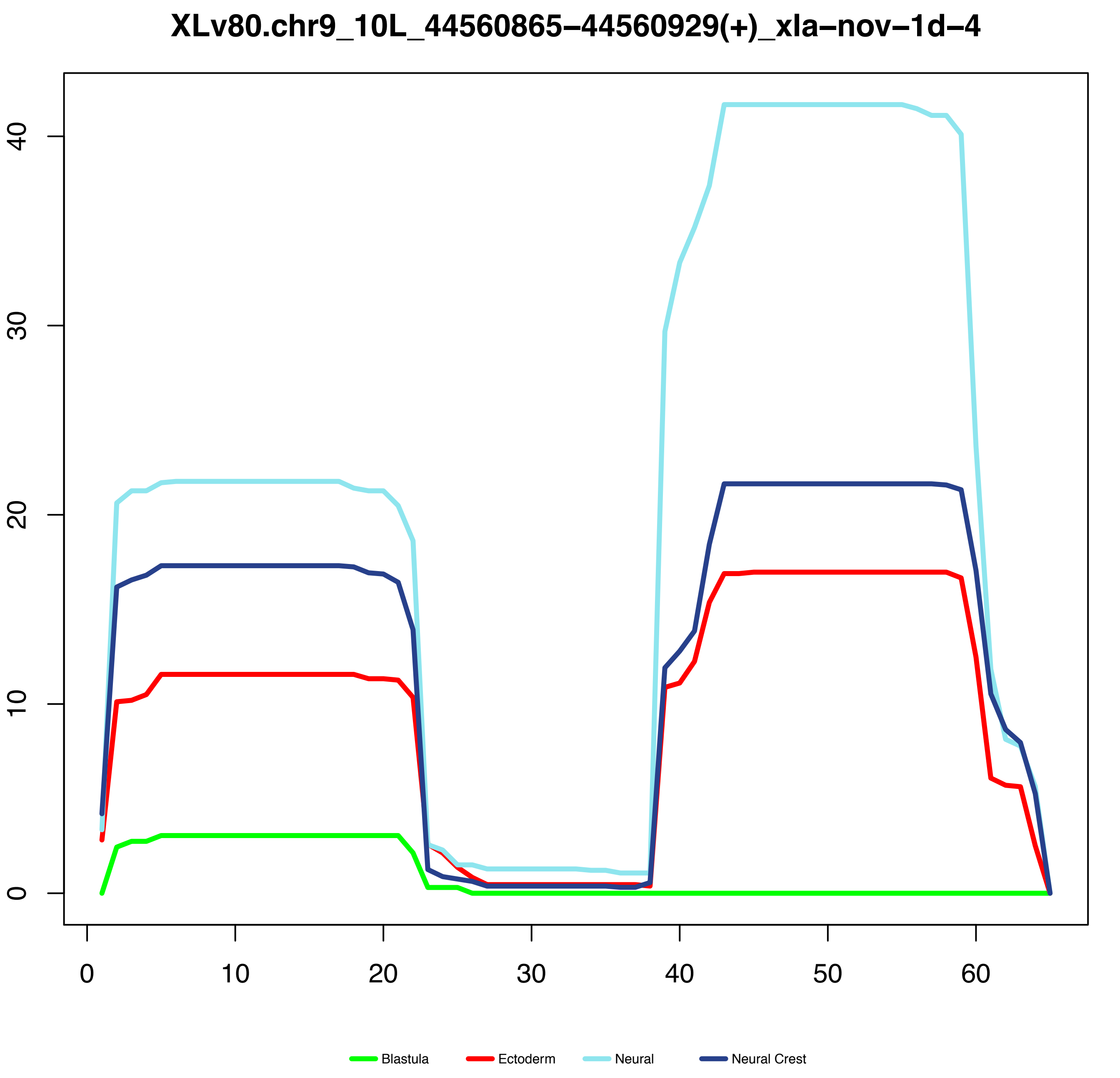
XLv80.chr9_10L_44560865−44560929(+)_xla−nov−1d−4
0
10
20
30
40
0
10
20
30
40
50
60
Blastula
Ectoderm
Neural
Neural Crest

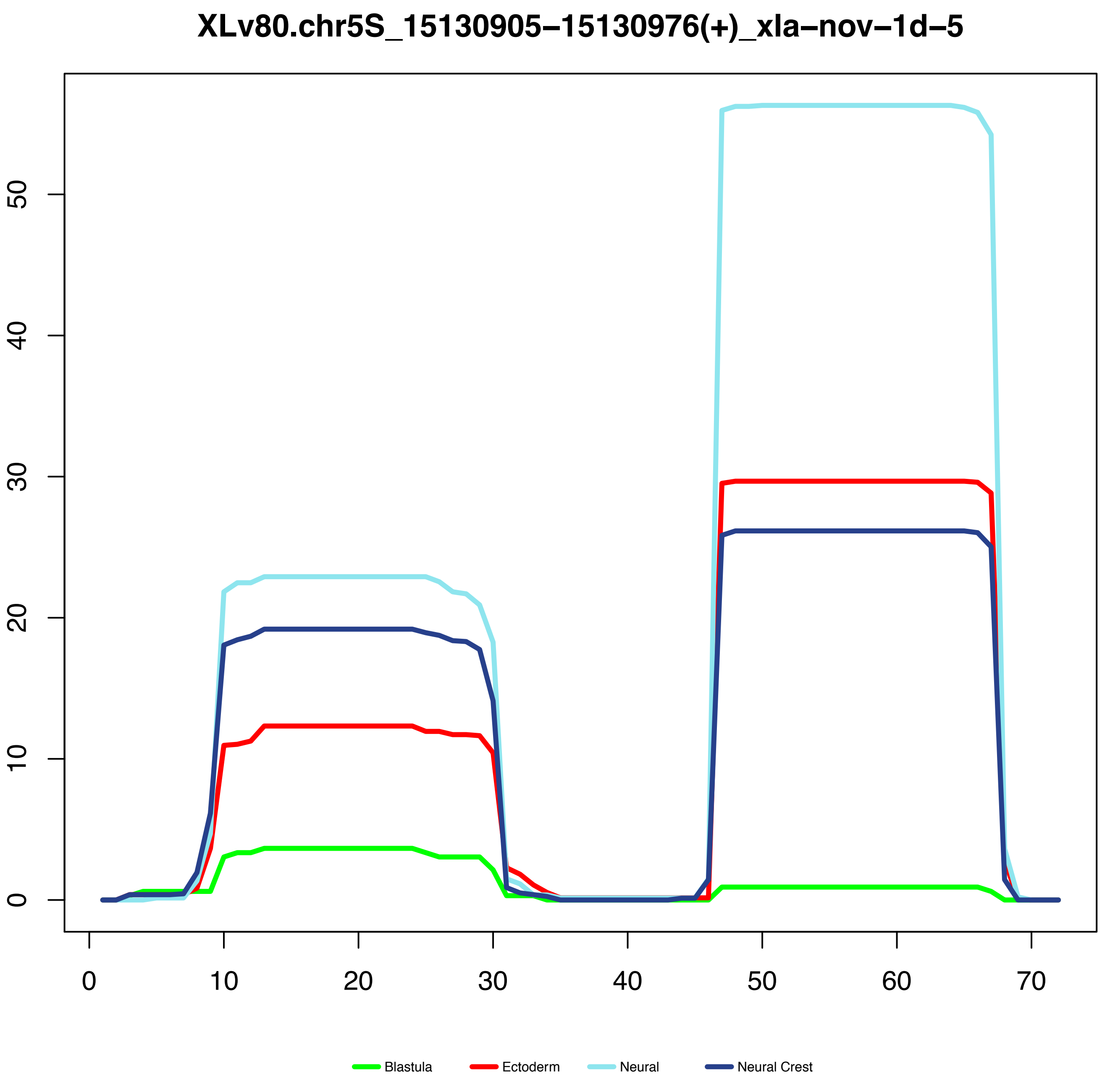
XLv80.chr5S_15130905–15130976(+)_xla–nov–1d–5
0
10
20
30
40
50
0
10
20
30
40
50
60
70
Blastula
Ectoderm
Neural
Neural Crest

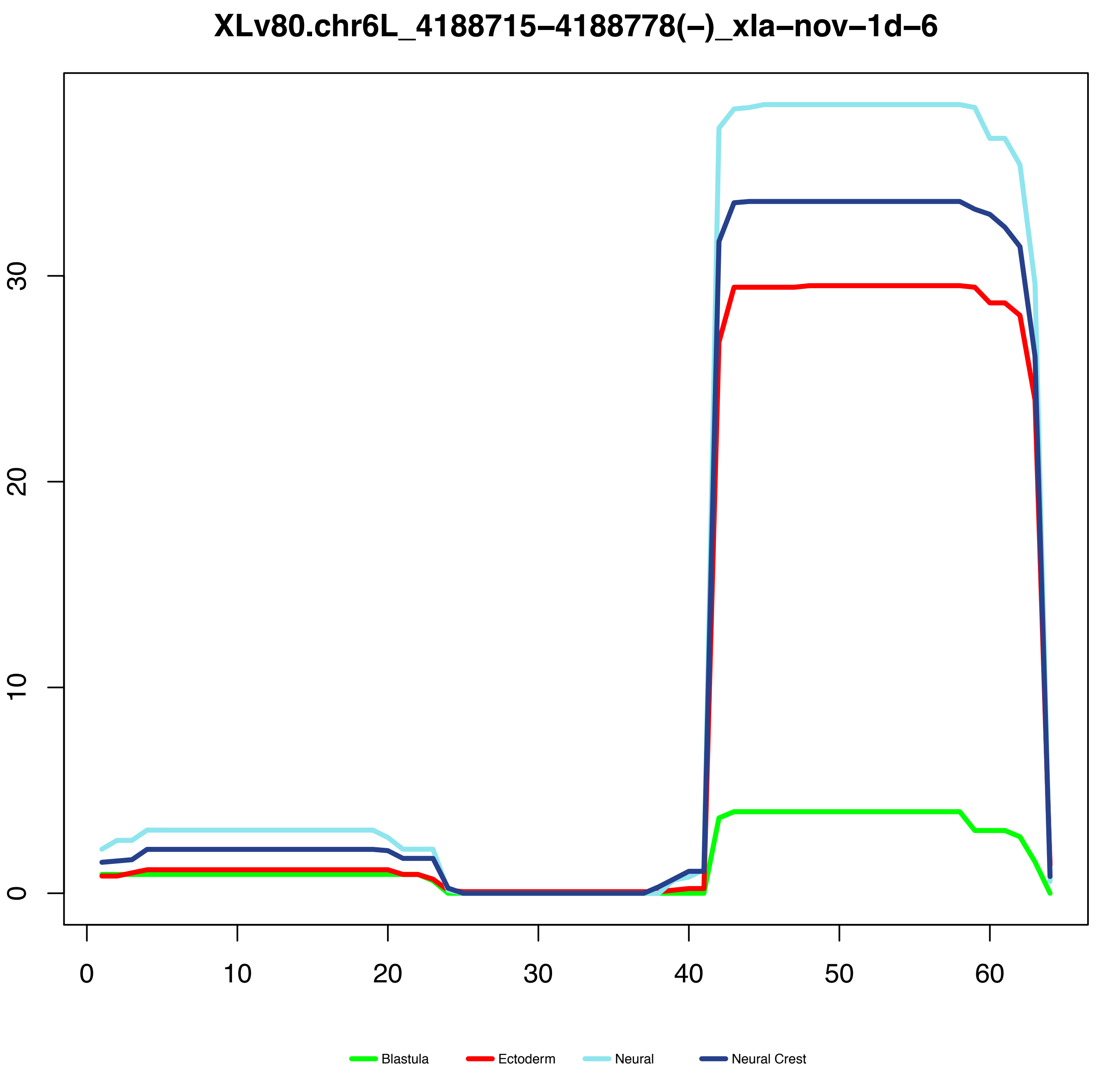

XLv80.chr6L_4188715−4188778(−)_xla−nov−1d−6
0
10
20
30
0
10
20
30
40
50
60
Blastula
Ectoderm
Neural
Neural Crest

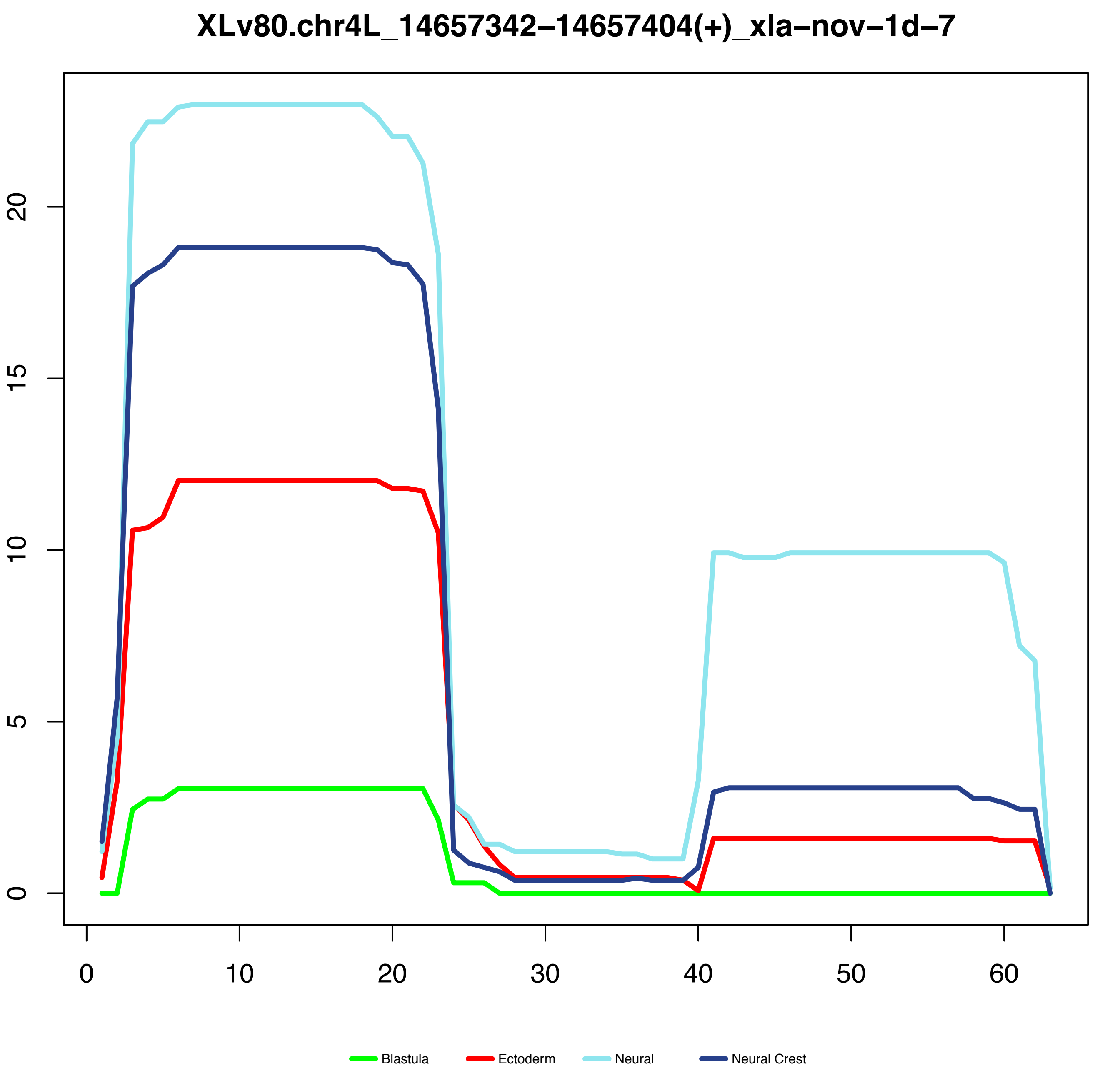

XLv80.chr4L_14657342−14657404(+)_xla−nov−1d−7
0
5
10
15
20
0
10
20
30
40
50
60
Blastula
Ectoderm
Neural
Neural Crest

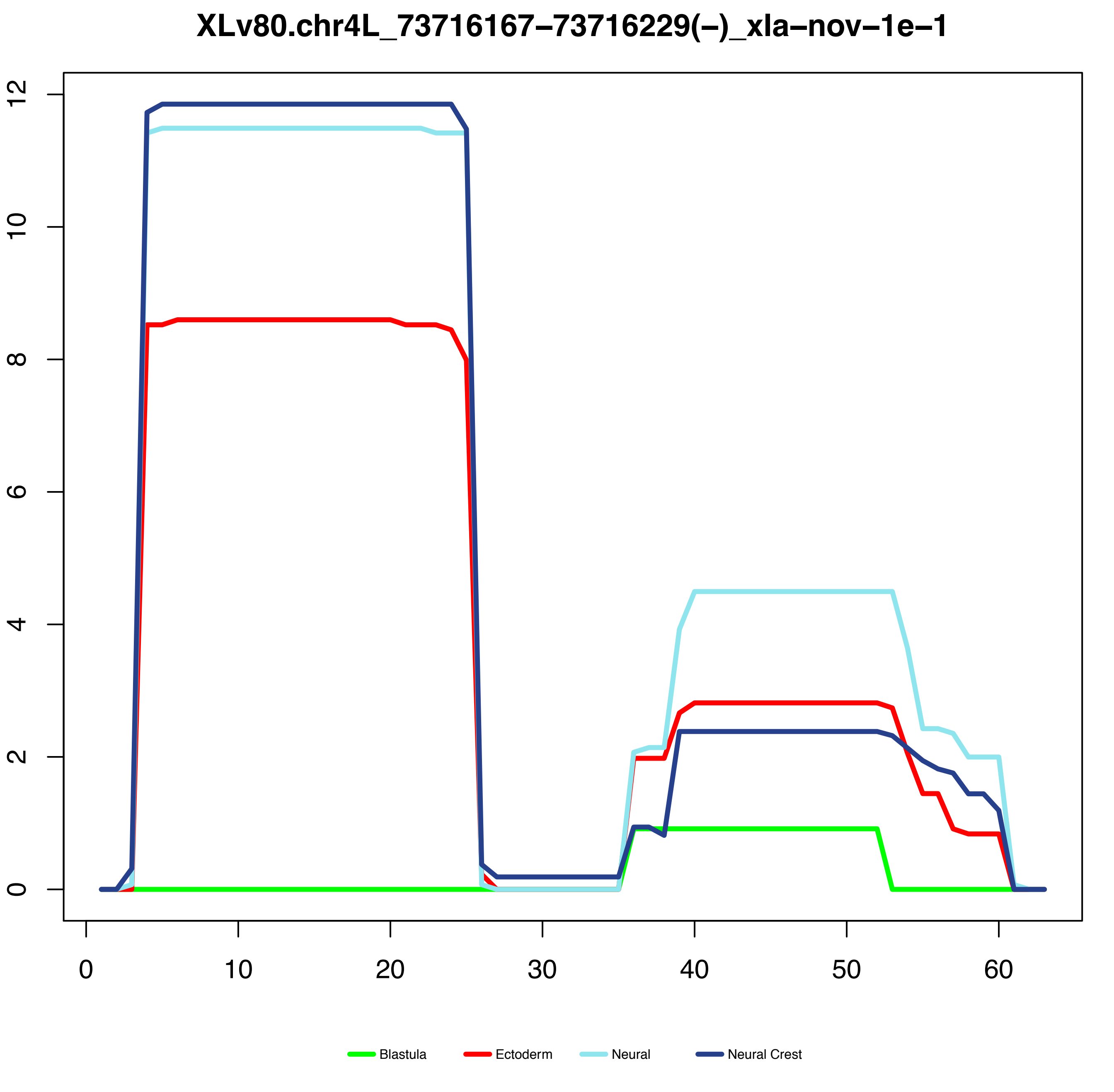
XLv80.chr4L_73716167−73716229(−)_xla−nov−1e−1
0
2
4
6
8
10
12
0
10
20
30
40
50
60
Blastula
Ectoderm
Neural
Neural Crest

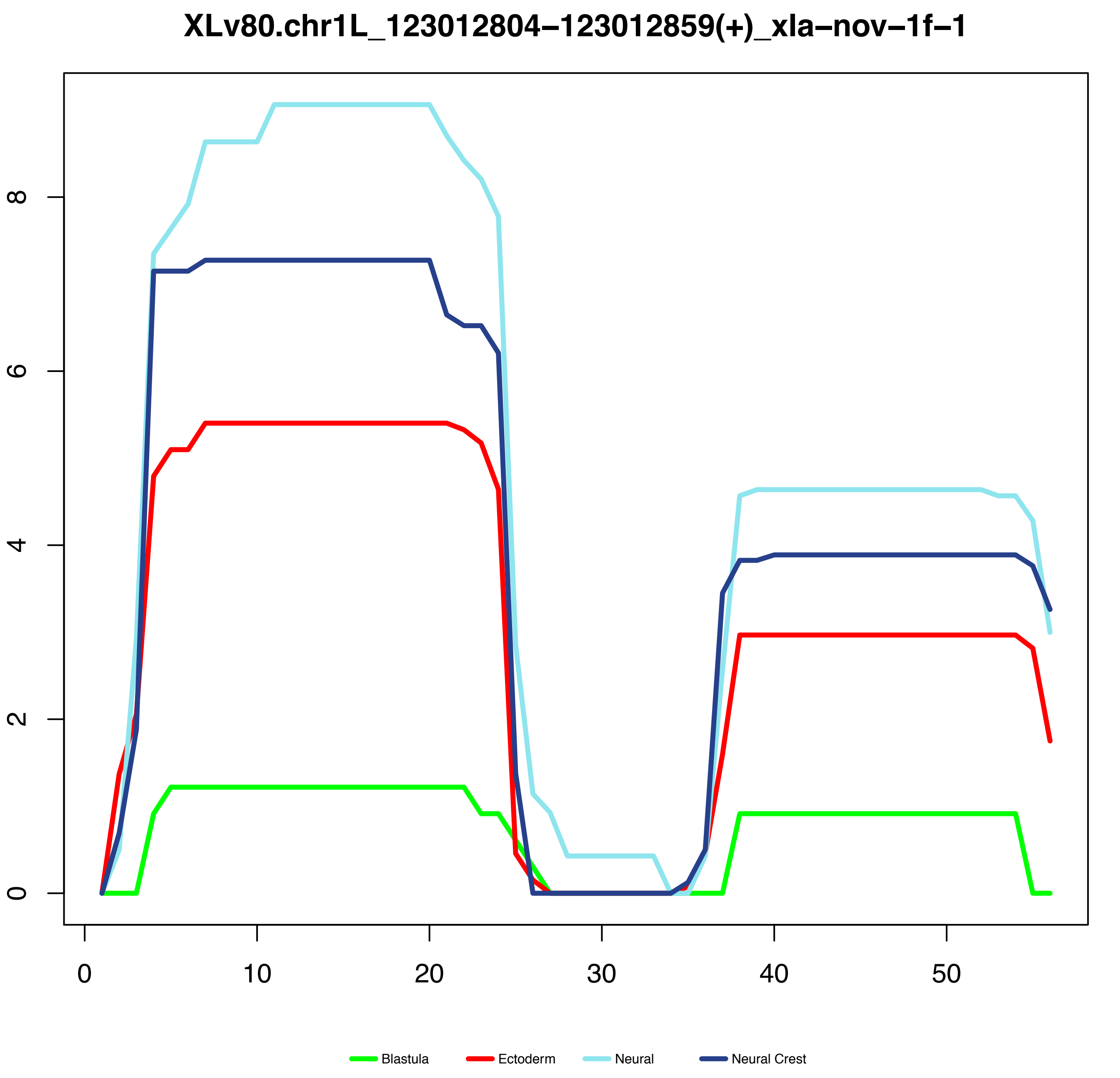

XLv80.chr1L_123012804−123012859(+)_xla−nov−1f−1
0
2
4
6
8
0
10
20
30
40
50
Blastula
Ectoderm
Neural
Neural Crest

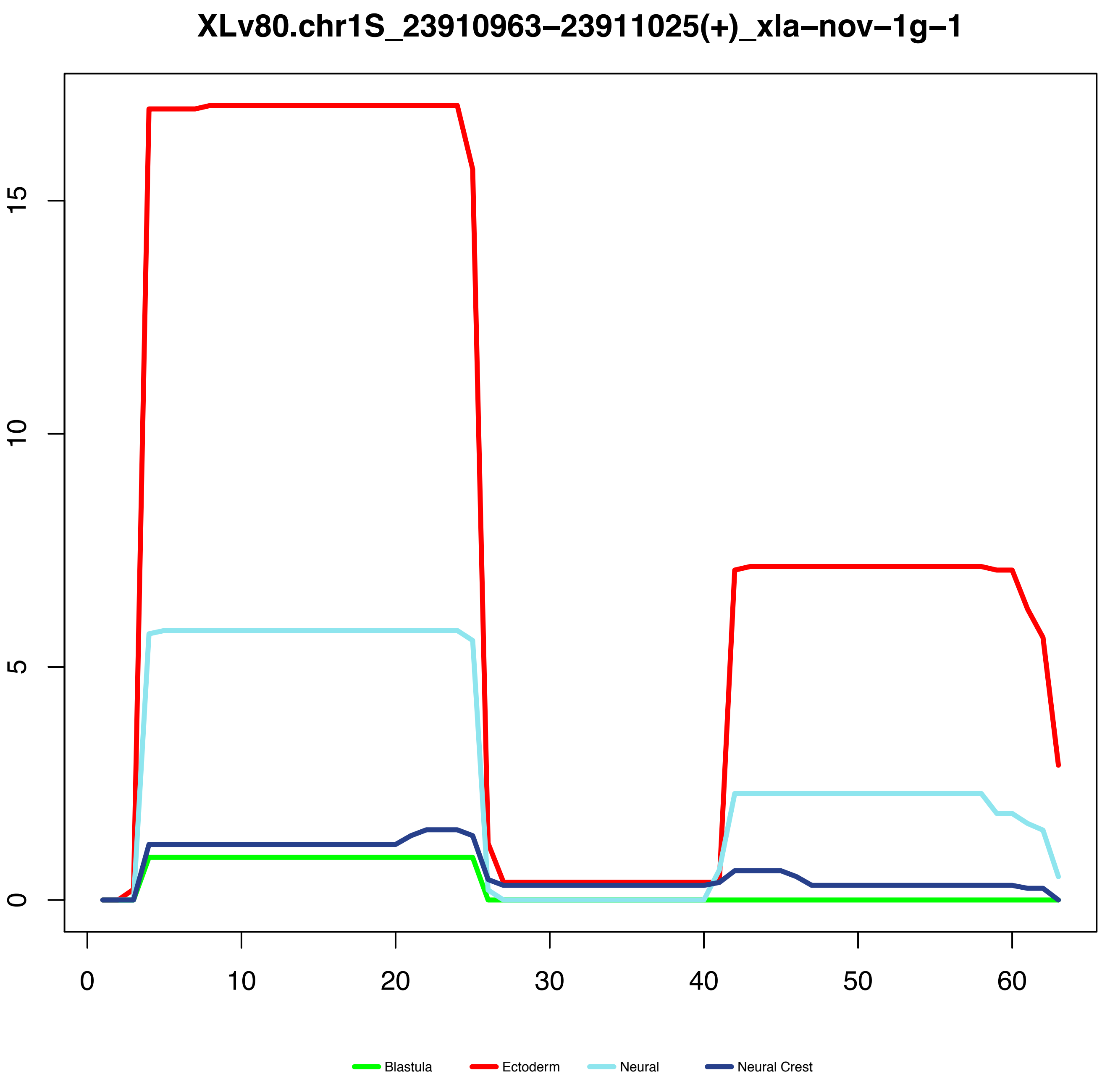
XLv80.chr1S_23910963−23911025(+)_xla−nov−1g−1
0
5
10
15
0
10
20
30
40
50
60
Blastula
Ectoderm
Neural
Neural Crest

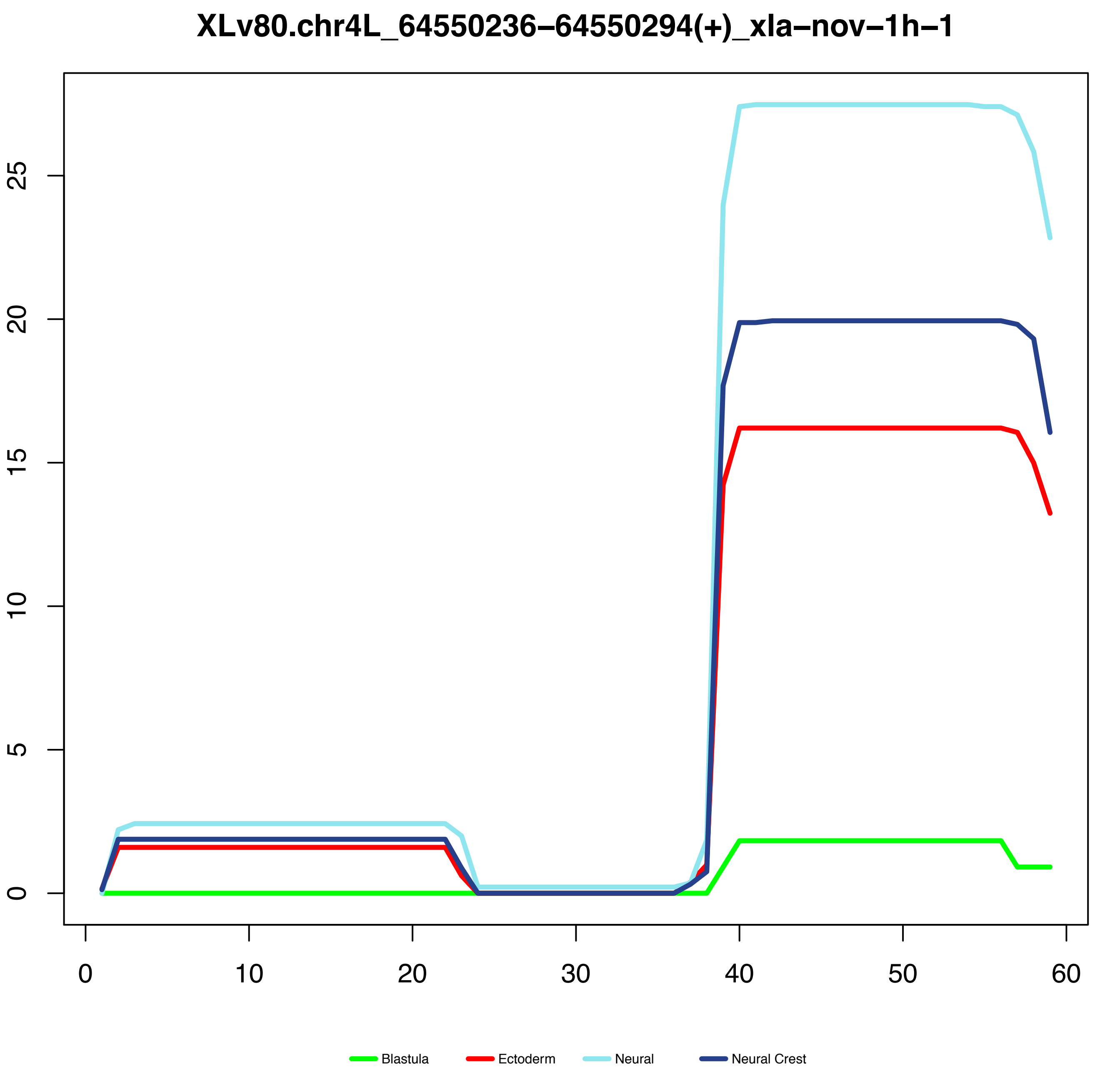
XLv80.chr4L_64550236−64550294(+)_xla−nov−1h−1
0
5
10
15
20
25
0
10
20
30
40
50
60
Blastula
Ectoderm
Neural
Neural Crest

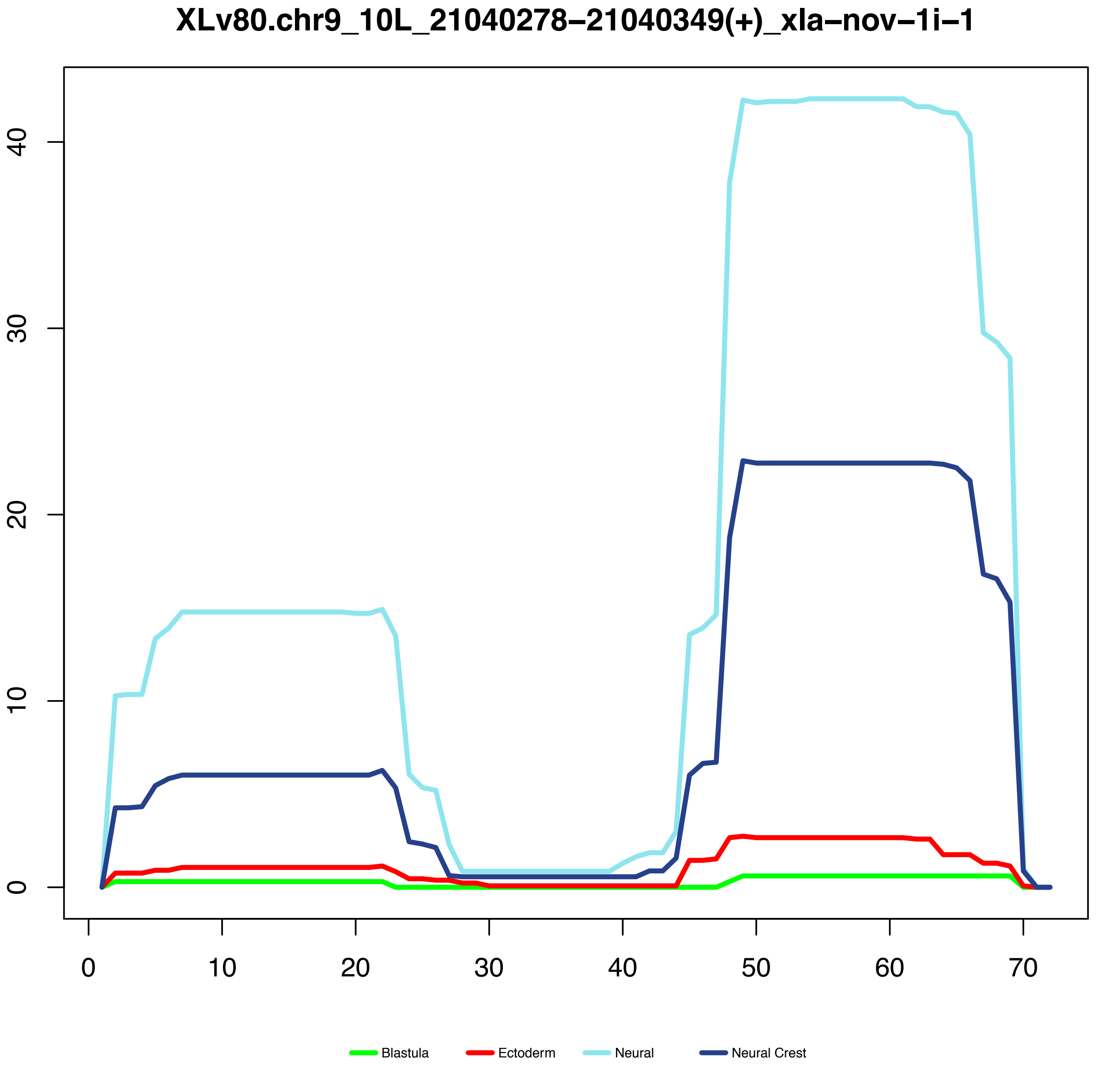
XLv80.chr9_10L_21040278−21040349(+)_xla−nov−1i−1
0
10
20
30
40
0
10
20
30
40
50
60
70
Blastula
Ectoderm
Neural
Neural Crest

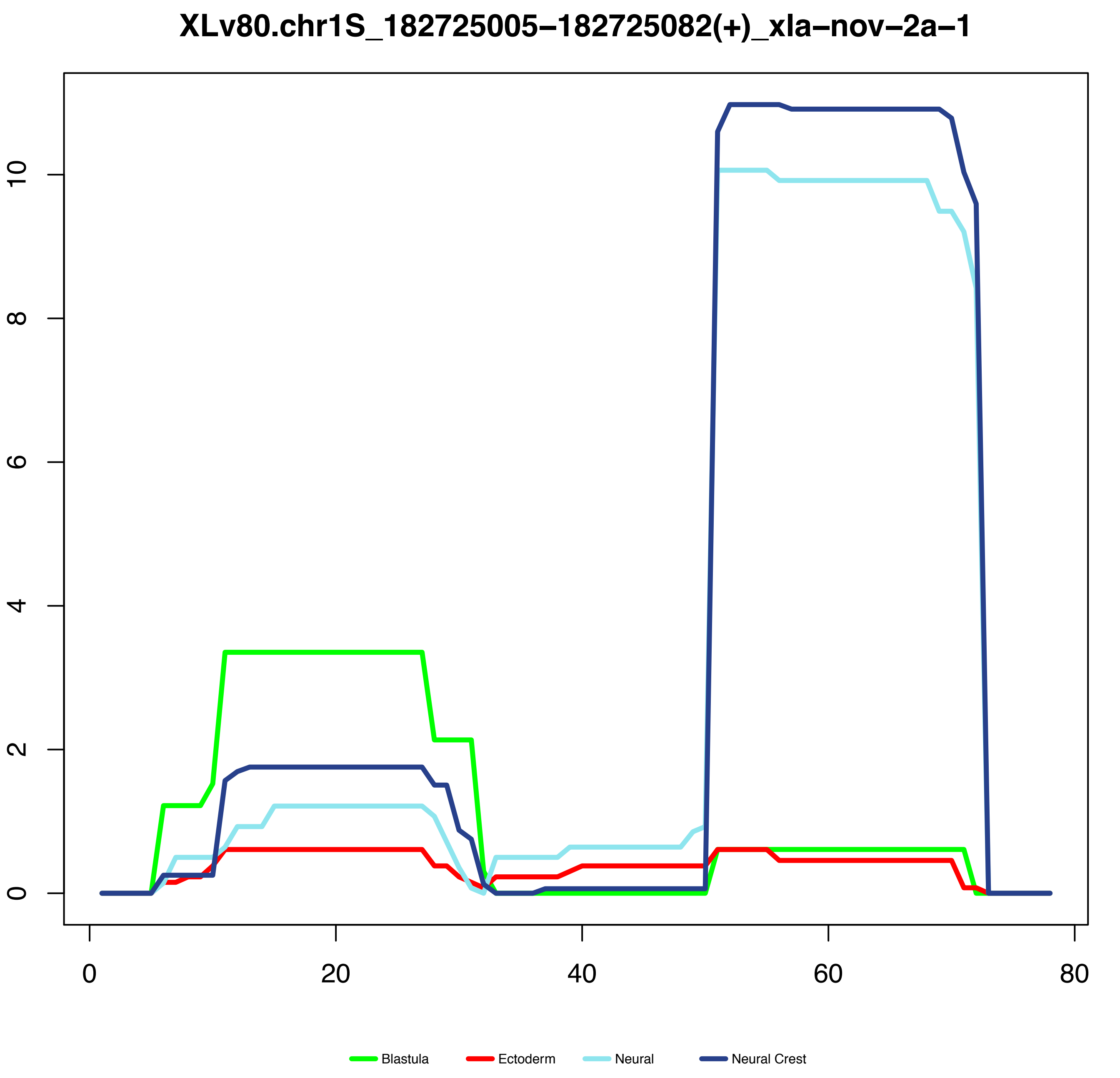

XLv80.chr1S_182725005−182725082(+)_xla−nov−2a−1
0
2
4
6
8
10
0
20
40
60
80
Blastula
Ectoderm
Neural
Neural Crest

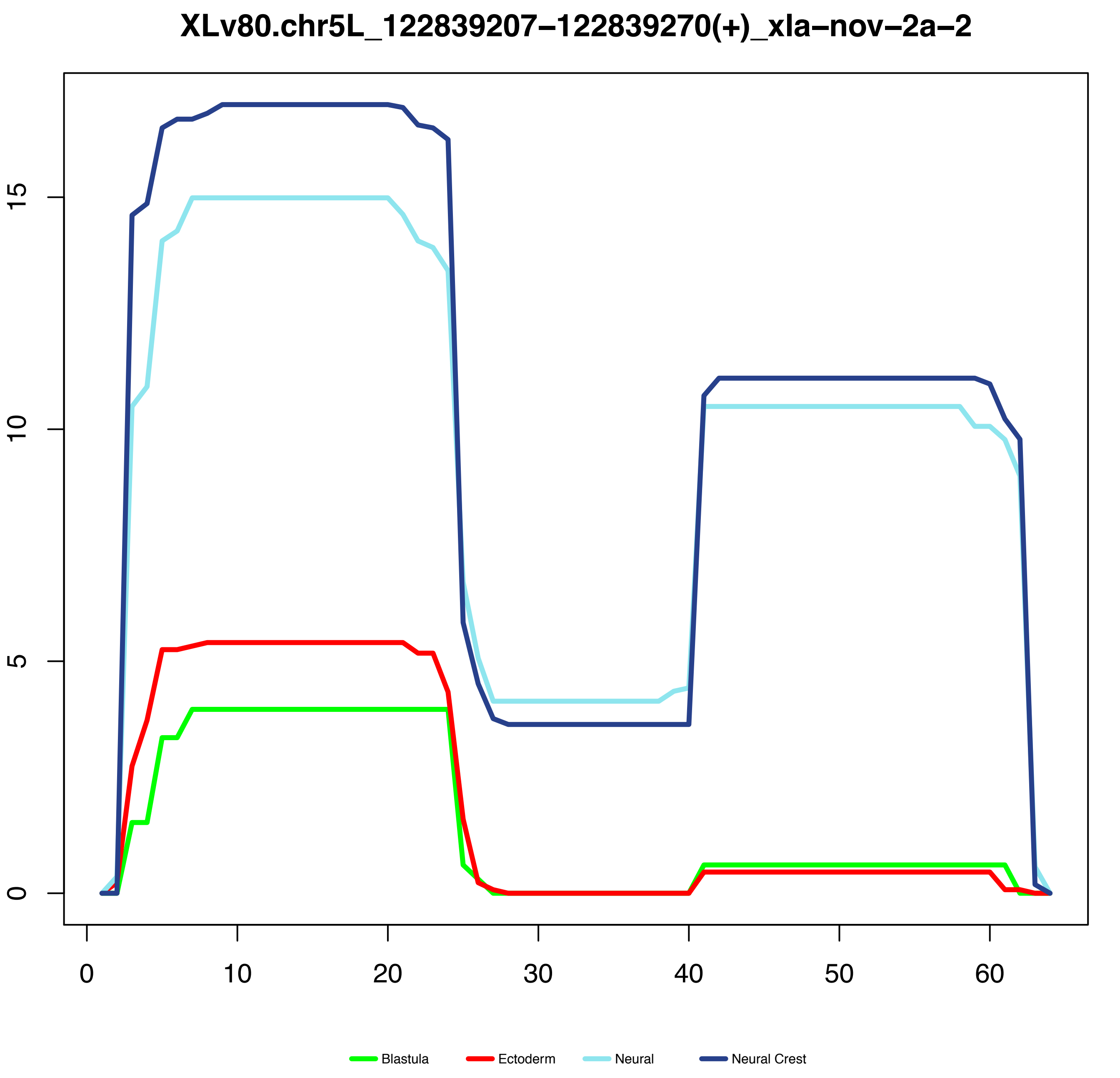
XLv80.chr5L_122839207−122839270(+)_xla−nov−2a−2
0
5
10
15
0
10
20
30
40
50
60
Blastula
Ectoderm
Neural
Neural Crest

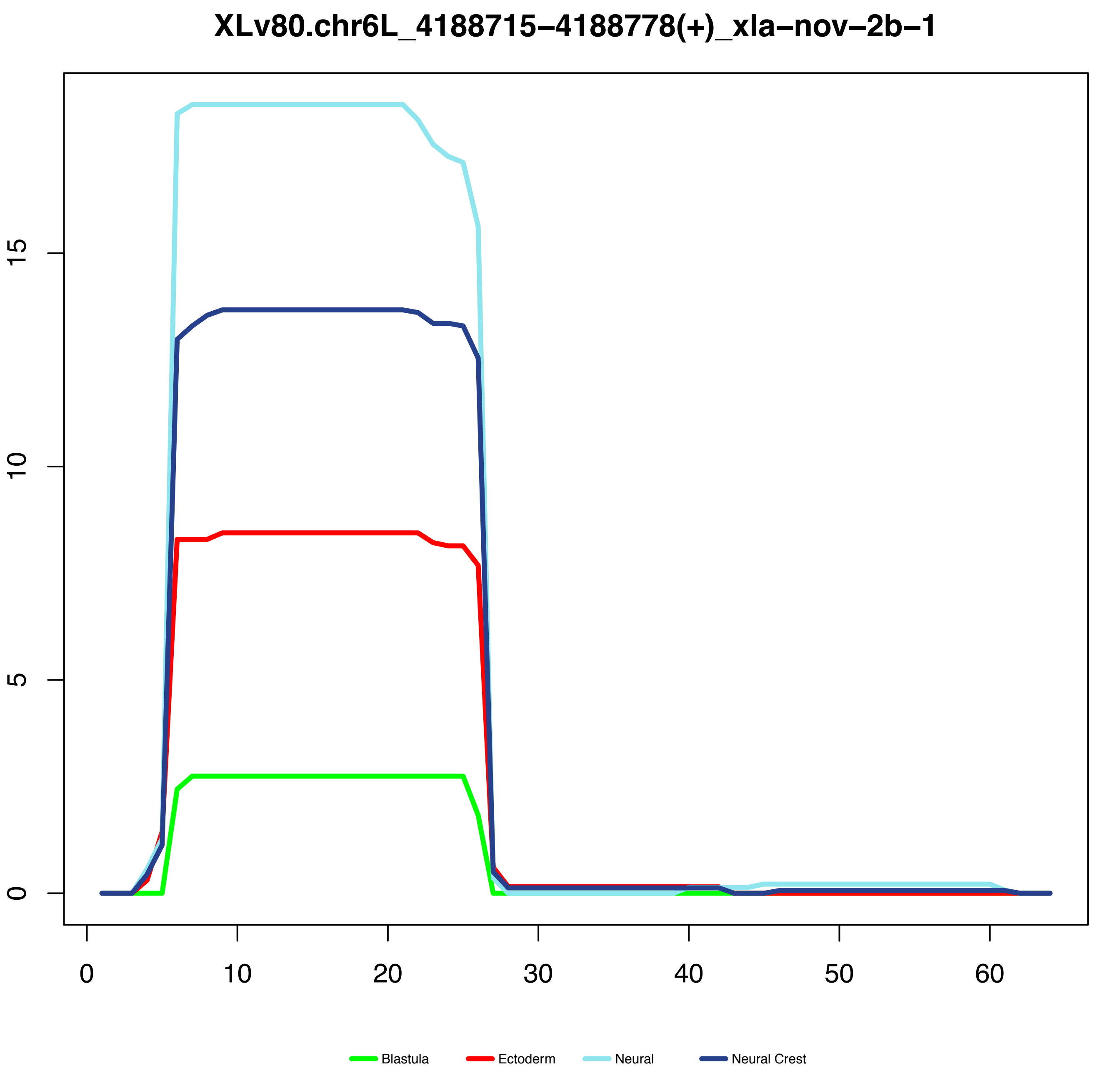
XLv80.chr6L_4188715−4188778(+)_xla−nov−2b−1
0
5
10
15
0
10
20
30
40
50
60
Blastula
Ectoderm
Neural
Neural Crest

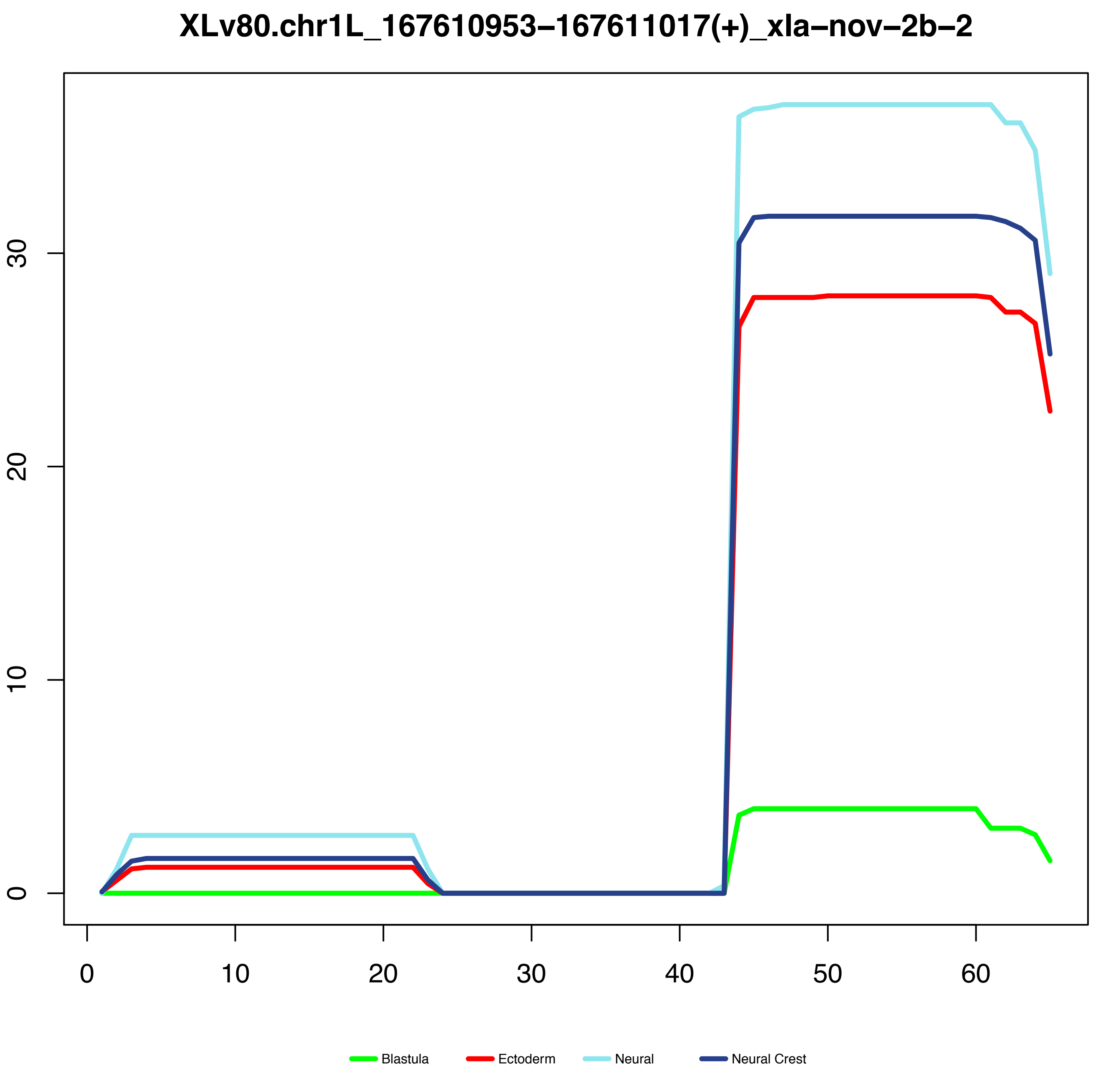
XLv80.chr1L_167610953−167611017(+)_xla−nov−2b−2
0
10
20
30
0
10
20
30
40
50
60
Blastula
Ectoderm
Neural
Neural Crest

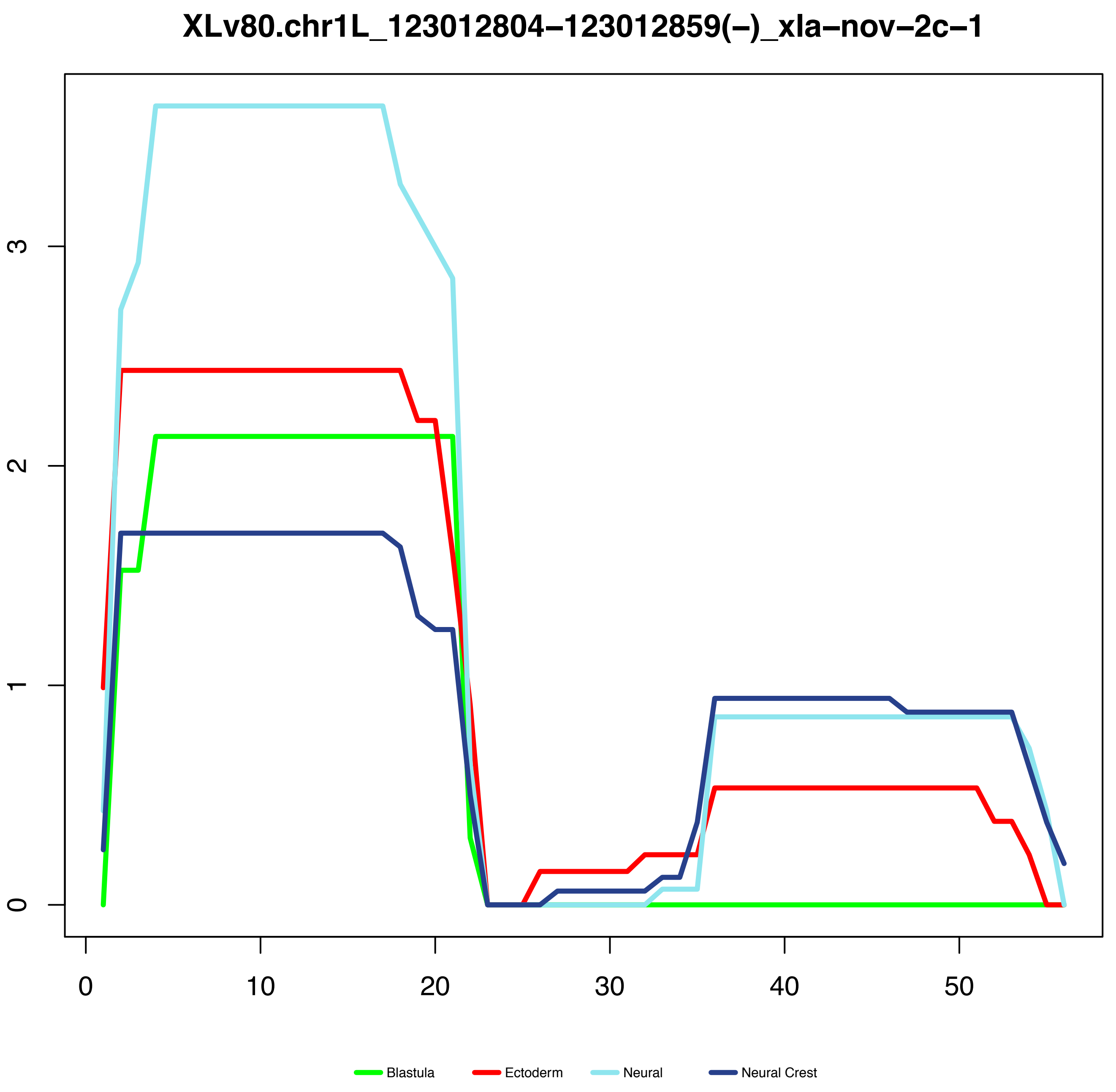

XLv80.chr1L_123012804–123012859(–)_xla–nov–2c–1
0
1
2
3
0
10
20
30
40
50
Blastula
Ectoderm
Neural
Neural Crest

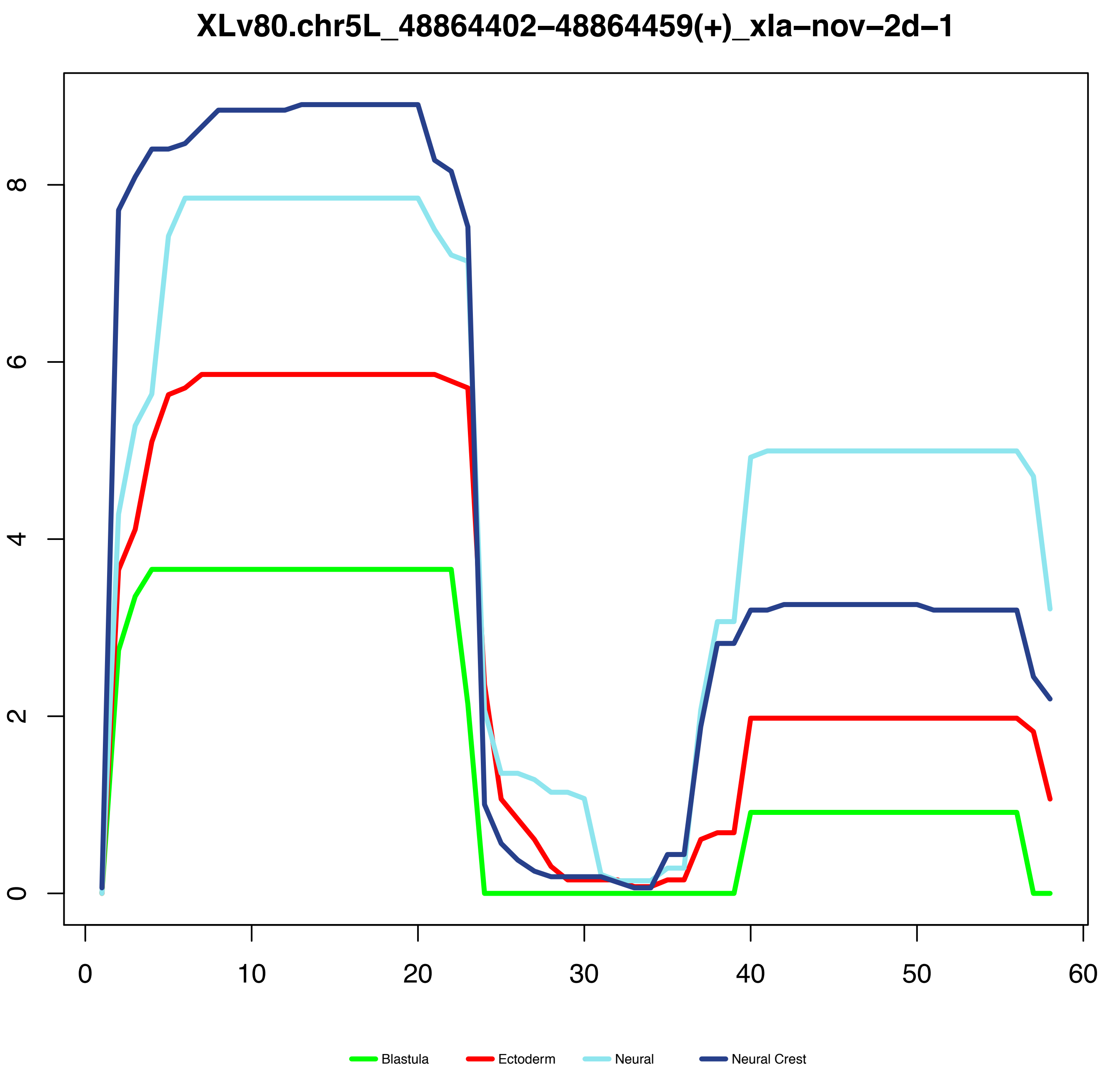
XLv80.chr5L_48864402−48864459(+)_xla−nov−2d−1
0
2
4
6
8
0
10
20
30
40
50
60
Blastula
Ectoderm
Neural
Neural Crest

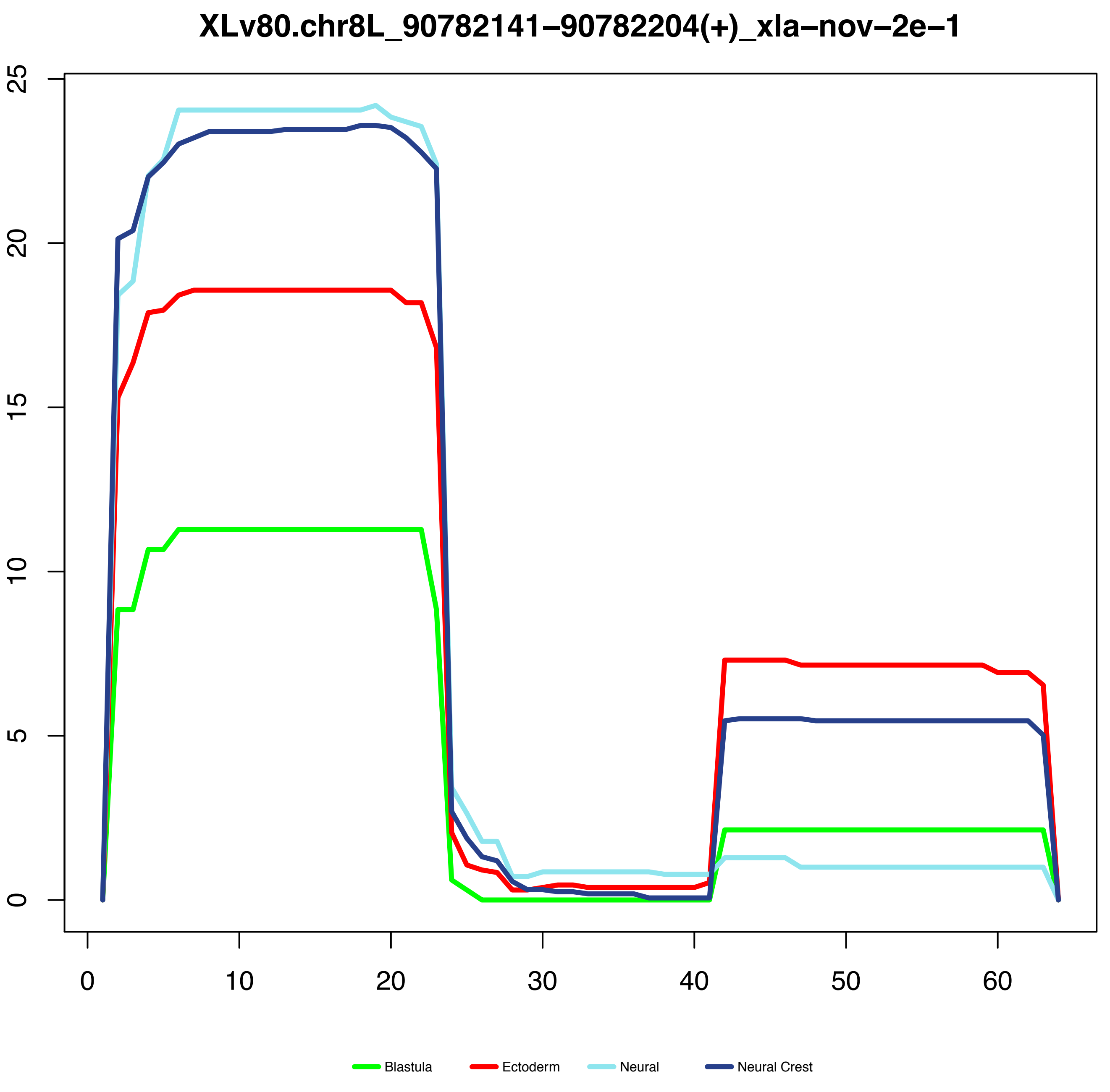

XLv80.chr8L_90782141−90782204(+)_xla−nov−2e−1
0
5
10
15
20
25
0
10
20
30
40
50
60
Blastula
Ectoderm
Neural
Neural Crest

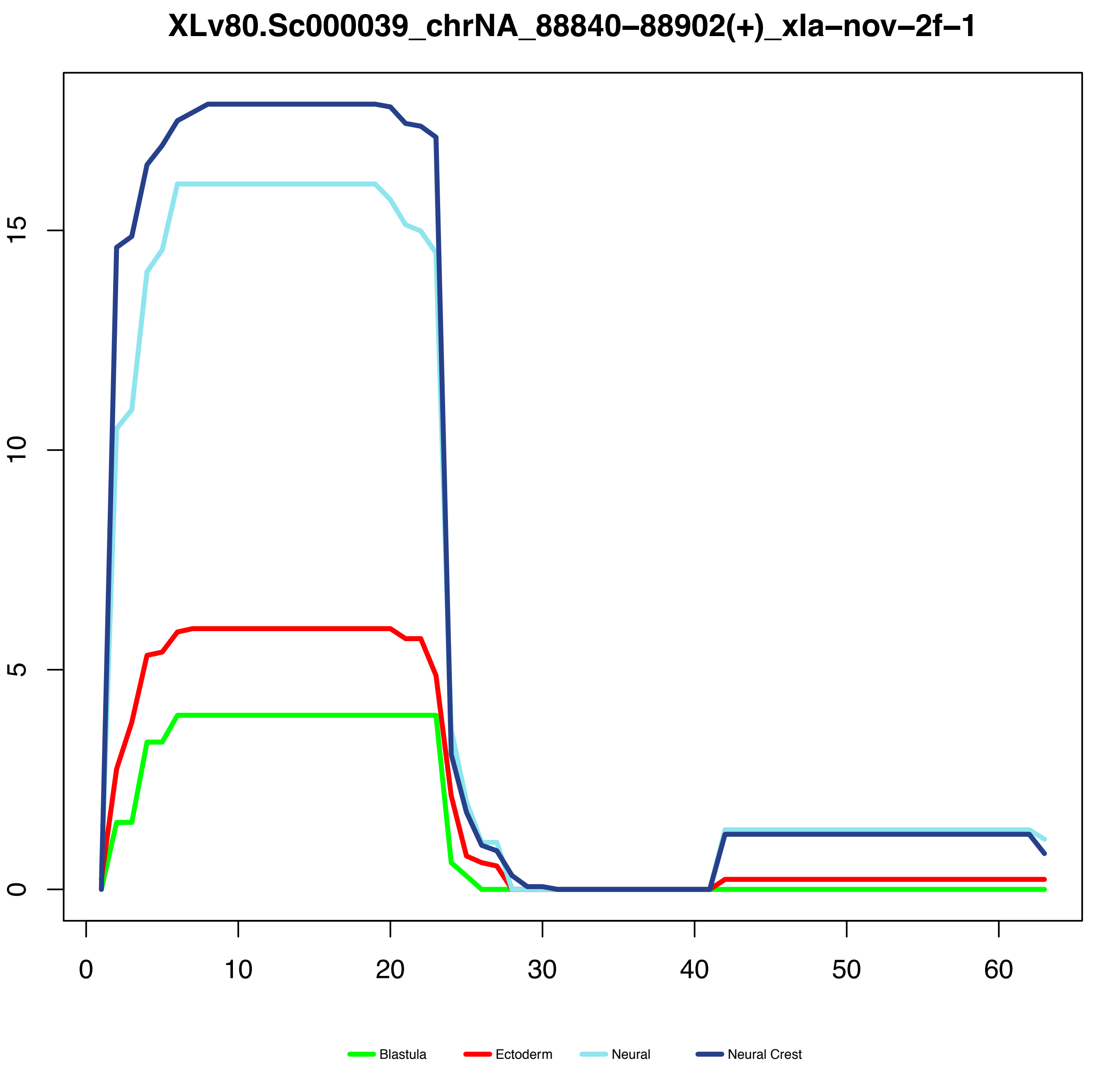
XLv80.Sc000039_chrNA_88840−88902(+)_xla−nov−2f−1
0
5
10
15
0
10
20
30
40
50
60
Blastula
Ectoderm
Neural
Neural Crest

XLv80.Sc000022_chr6L_6625831−6625892(+)_xla−nov−2g−1

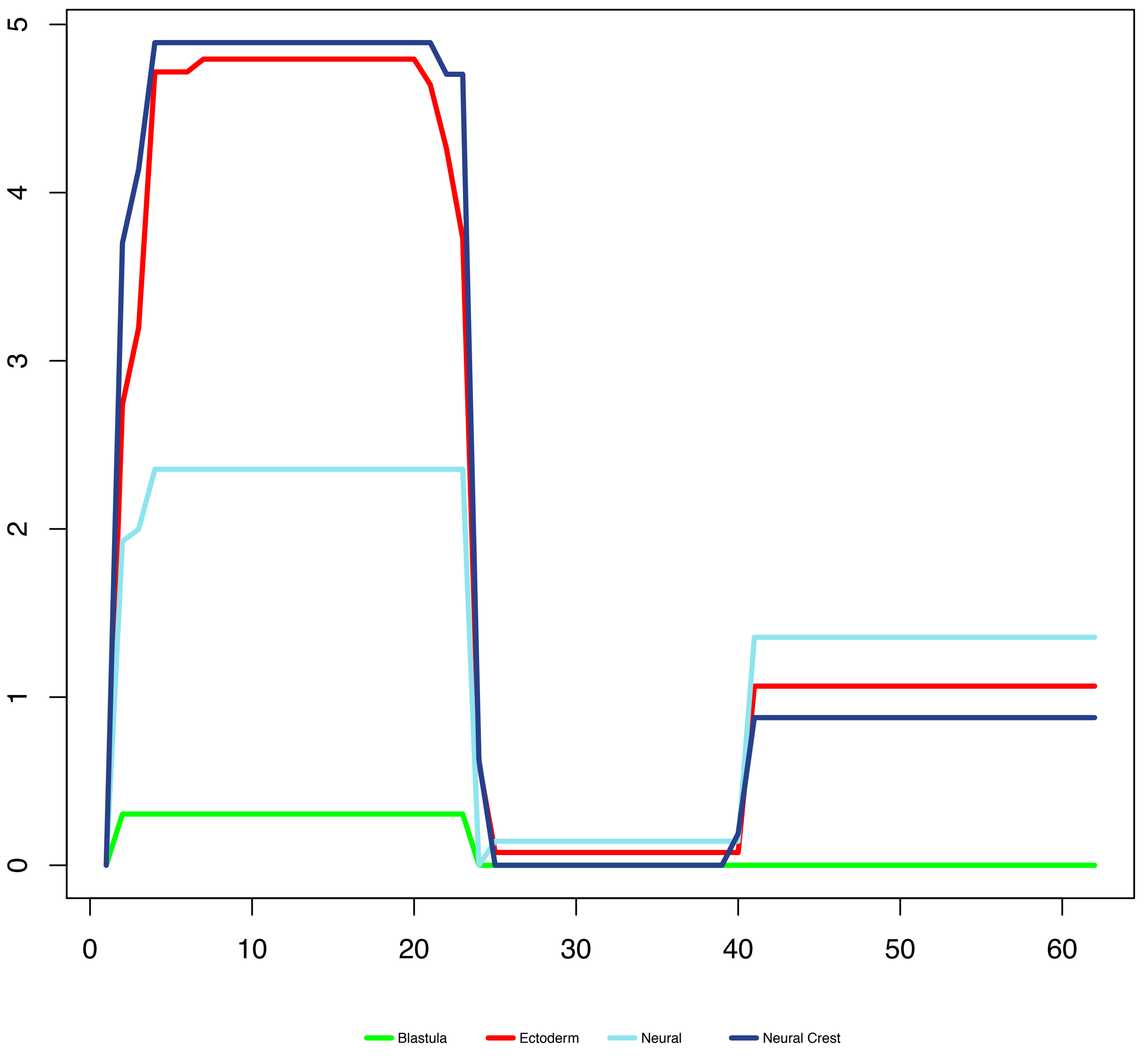

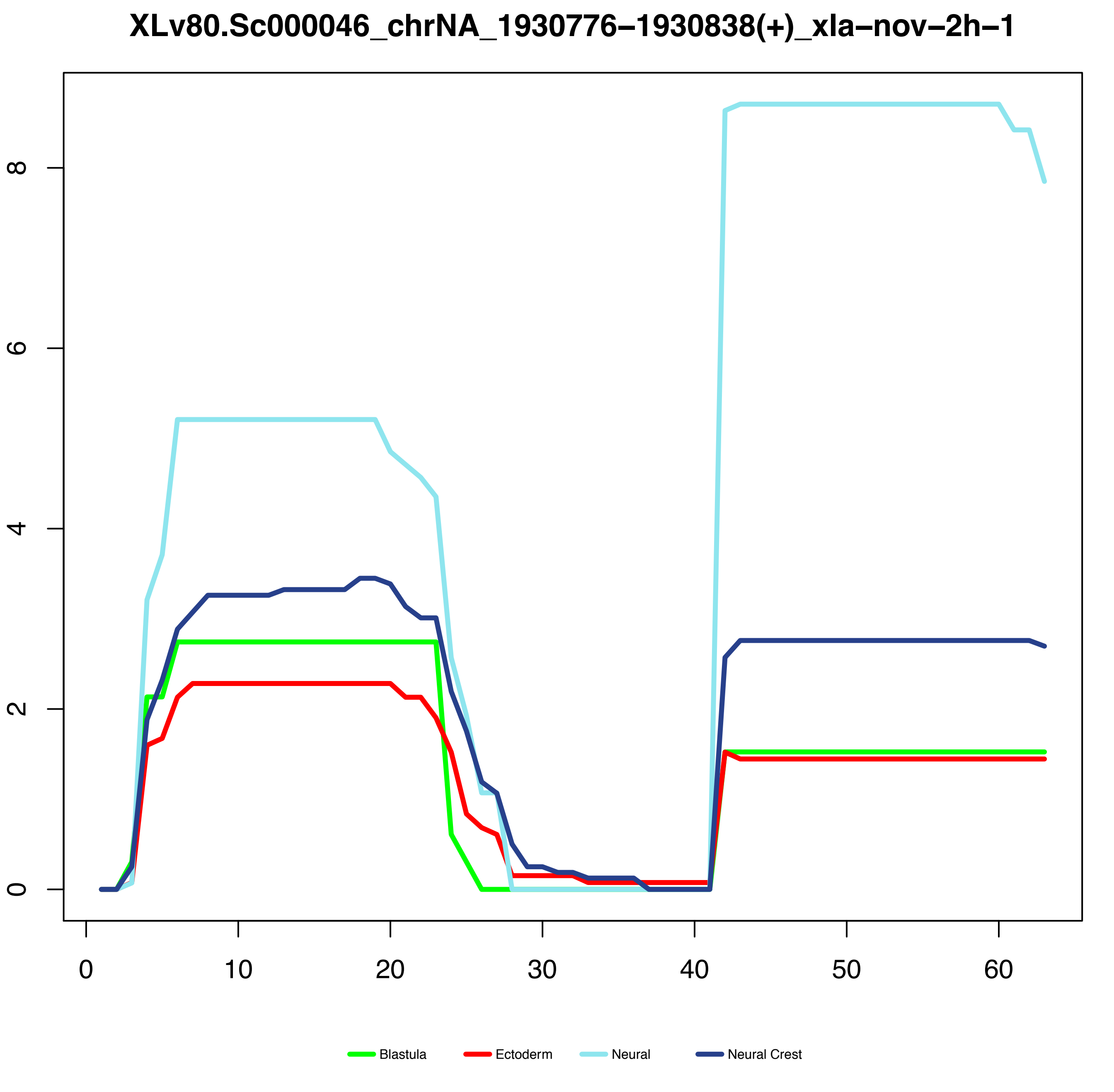
XLv80.Sc000046_chrNA_1930776–1930838(+)_xla–nov–2h–1
0
2
4
6
8
0
10
20
30
40
50
60
Blastula
Ectoderm
Neural
Neural Crest

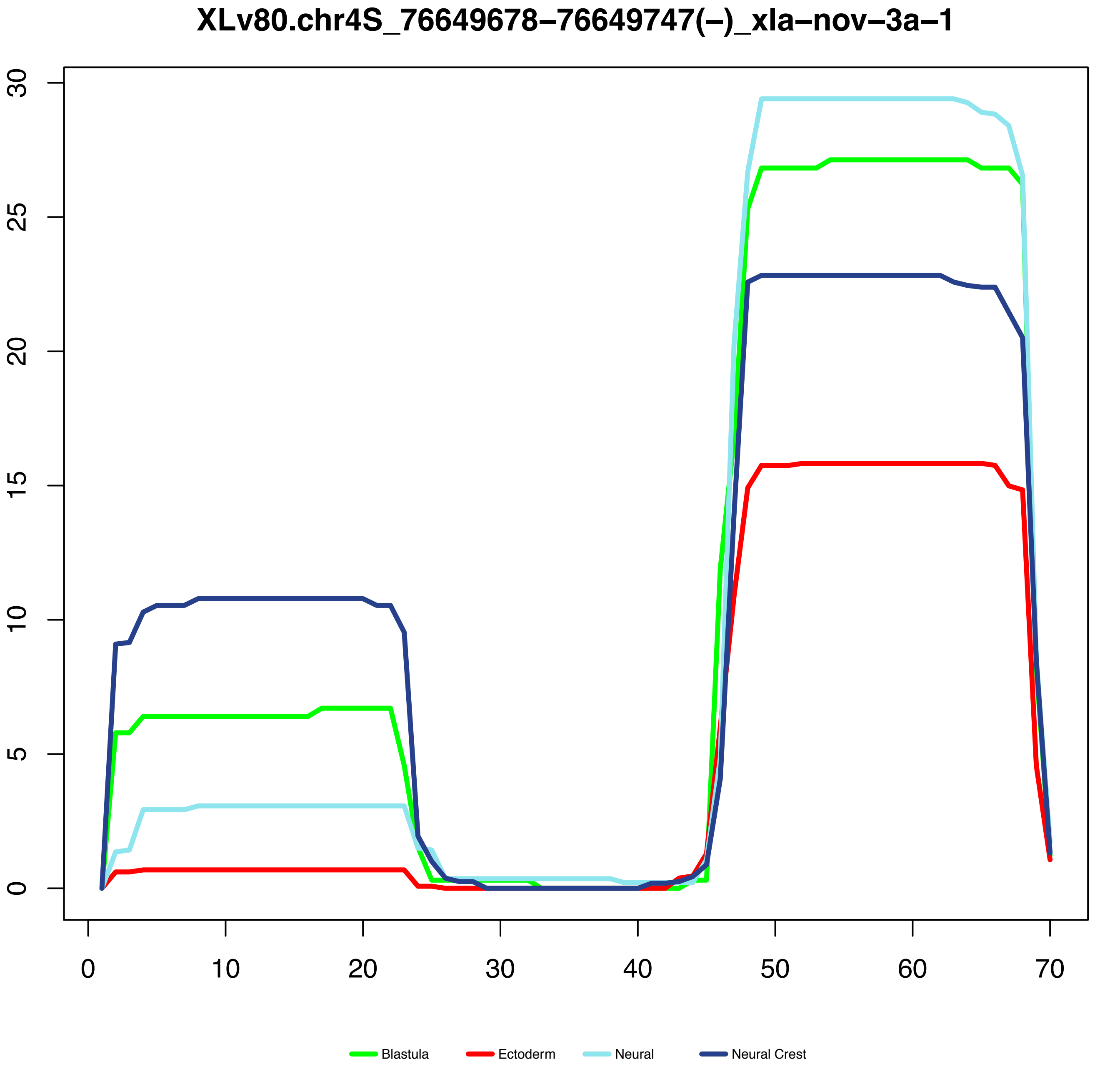

XLv80.chr4S_76649678−76649747(−)_xla−nov−3a−1
0
5
10
15
20
25
30
0
10
20
30
40
50
60
70
Blastula
Ectoderm
Neural
Neural Crest

XLv80.chr5S_74682246−74682319(+)_xla−nov−3b−1

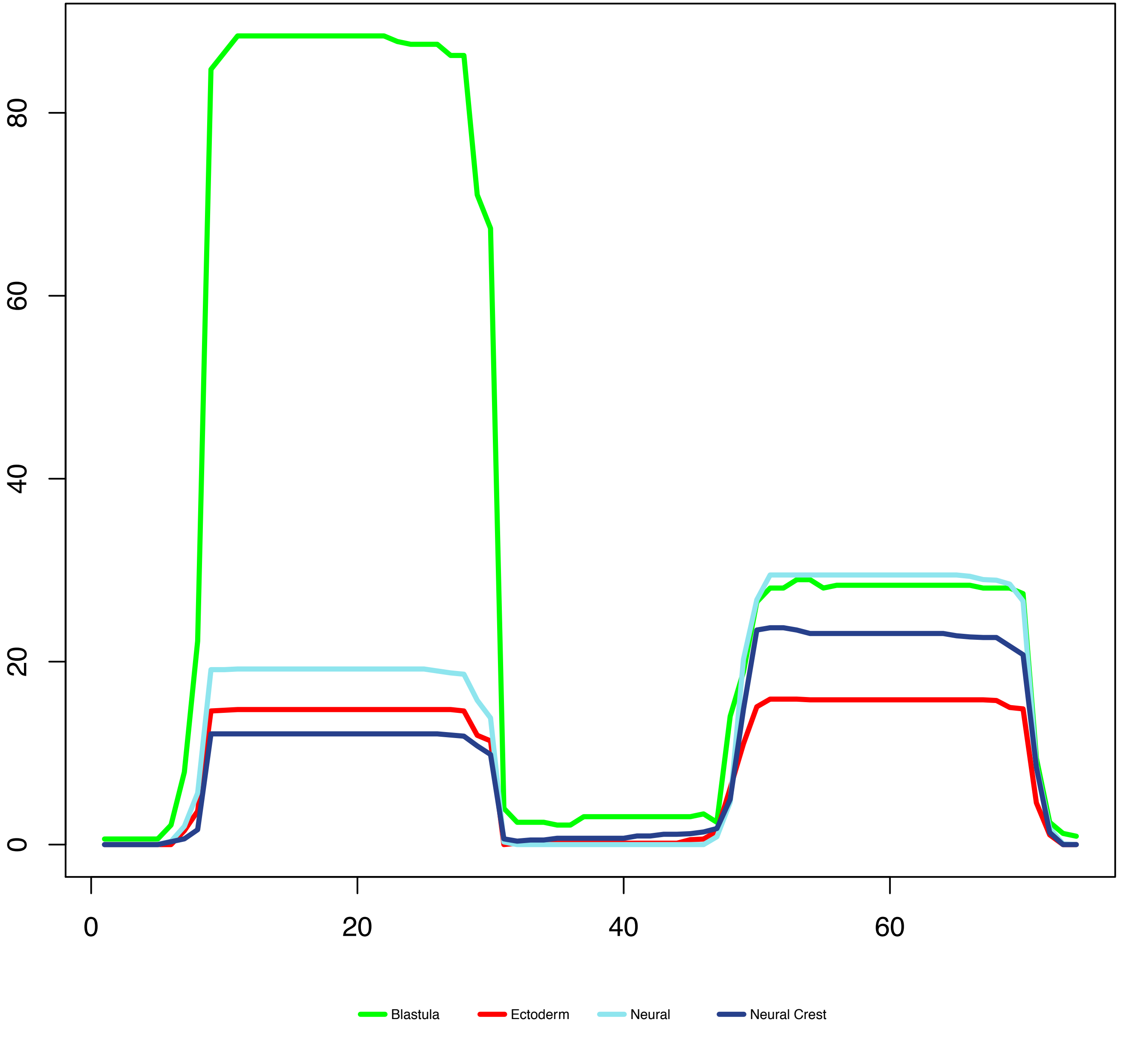

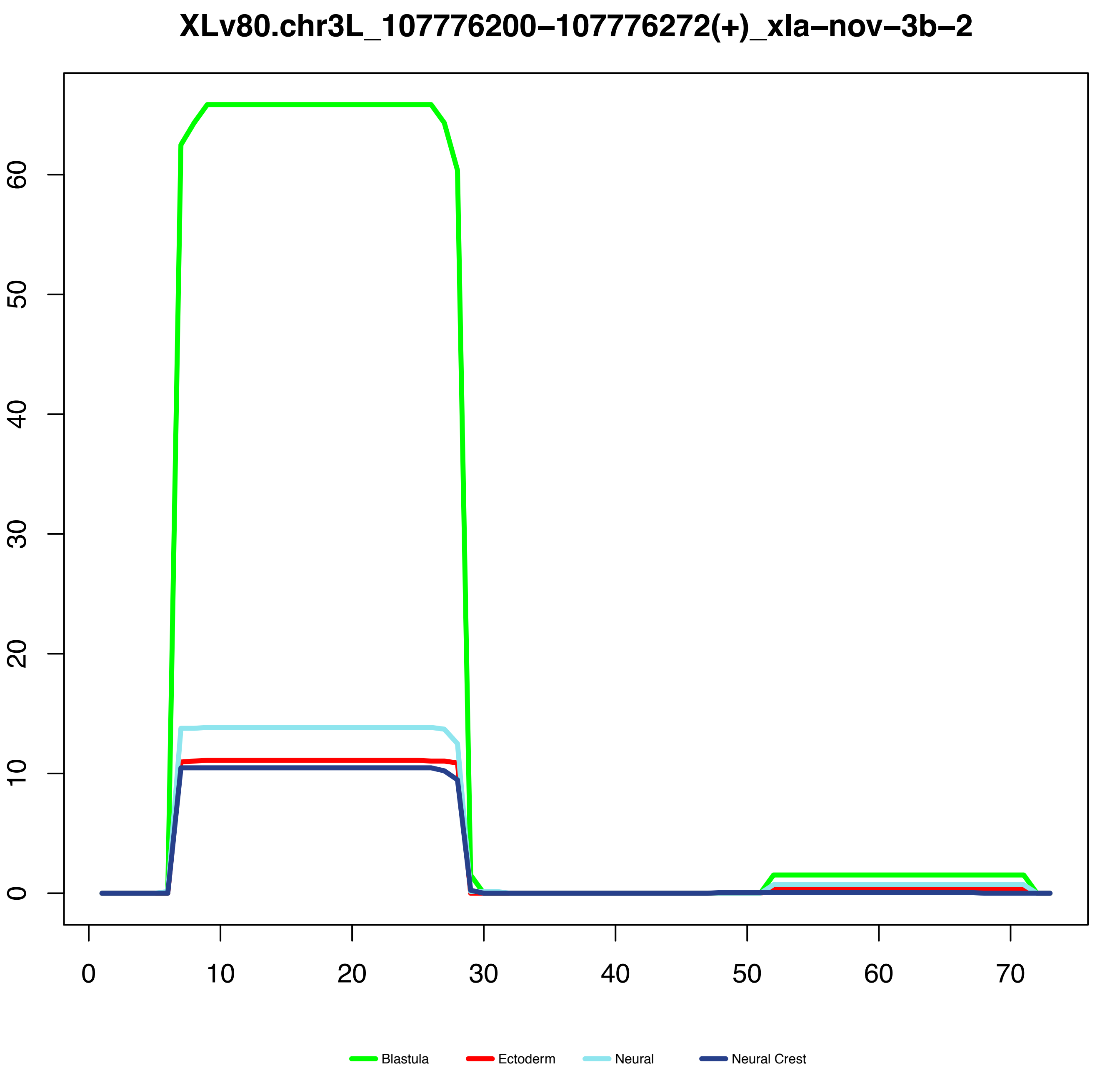

XLv80.chr3L_107776200−107776272(+)_xla−nov−3b−2
0
10
20
30
40
50
60
0
10
20
30
40
50
60
70
Blastula
Ectoderm
Neural
Neural Crest

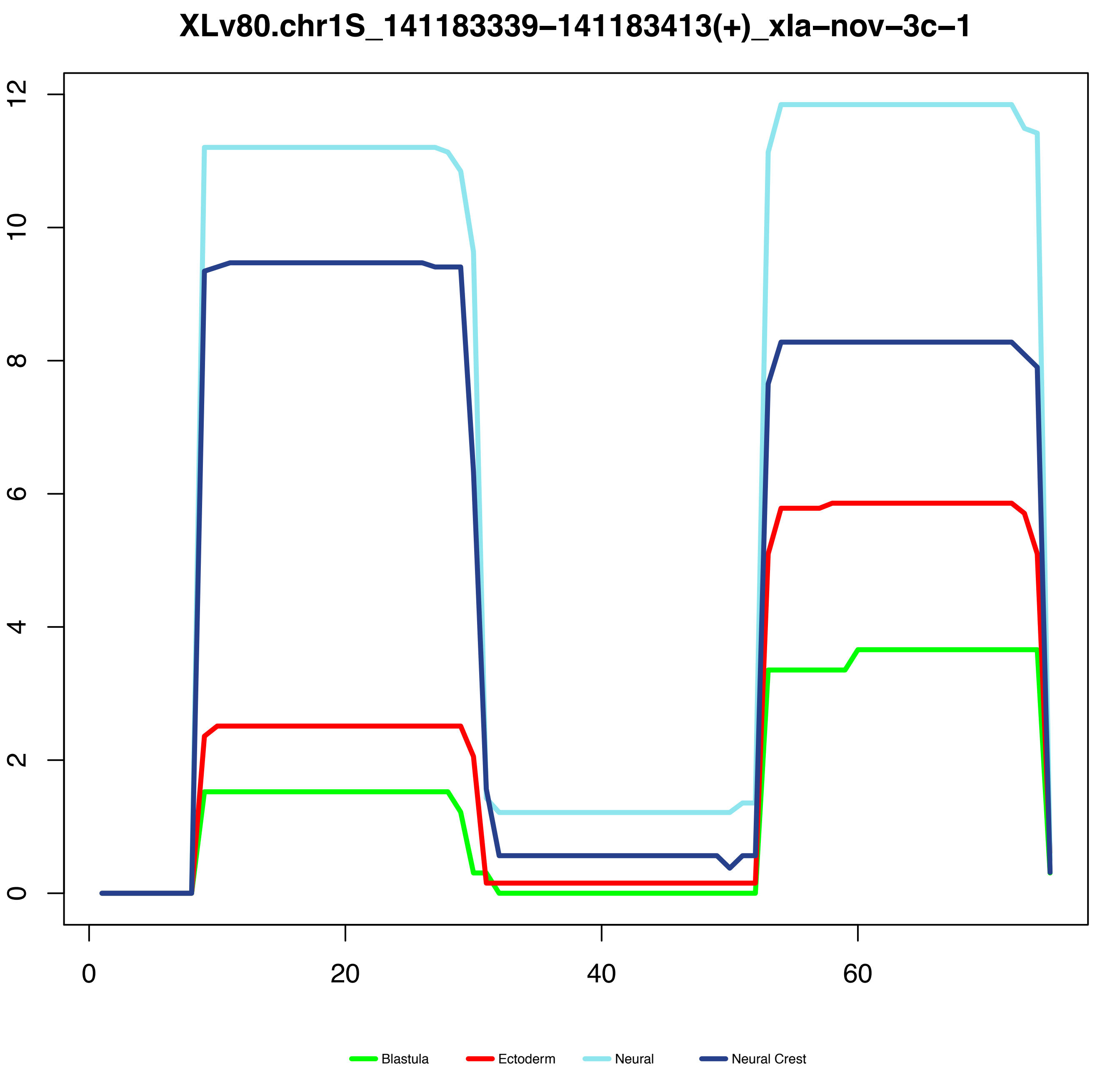

XLv80.chr1S_141183339−141183413(+)_xla−nov−3c−1
0
2
4
6
8
10
12
0
20
40
60
Blastula
Ectoderm
Neural
Neural Crest

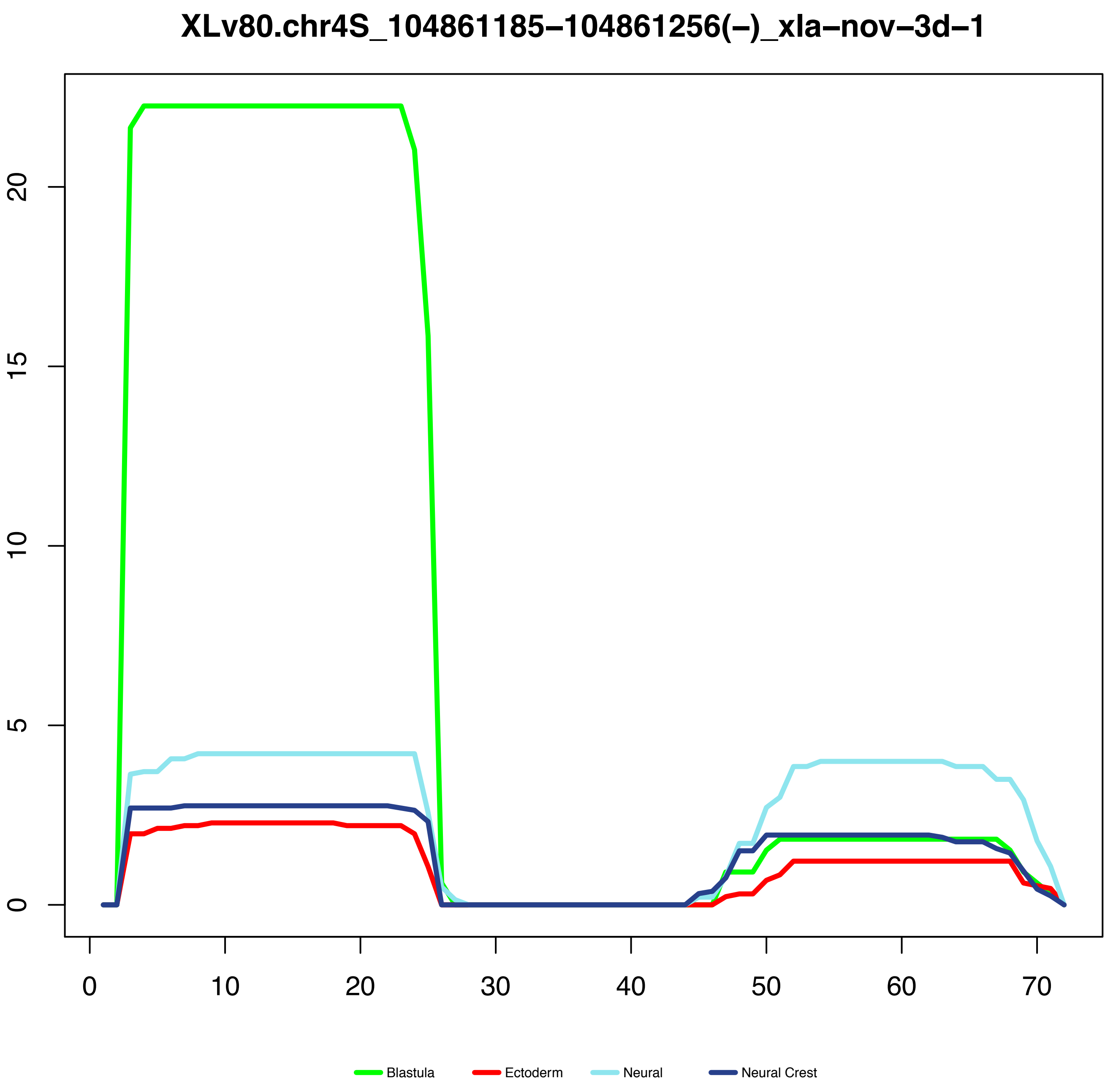

XLv80.chr4S_104861185−104861256(−)_xla−nov−3d−1
0
5
10
15
20
0
10
20
30
40
50
60
70
Blastula
Ectoderm
Neural
Neural Crest

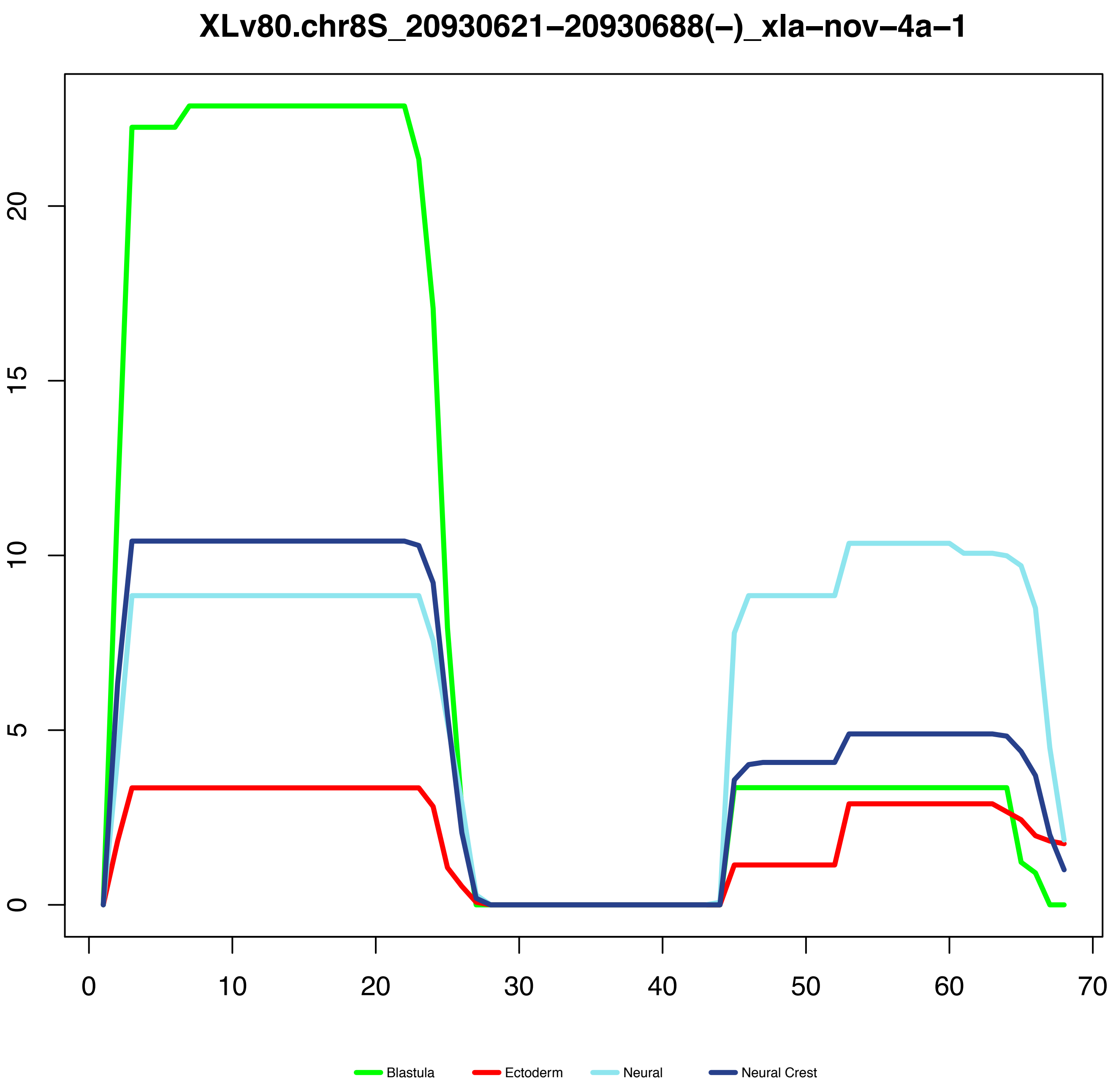

XLv80.chr8S_20930621−20930688(−)_xla−nov−4a−1
0
5
10
15
20
0
10
20
30
40
50
60
70
Blastula
Ectoderm
Neural
Neural Crest

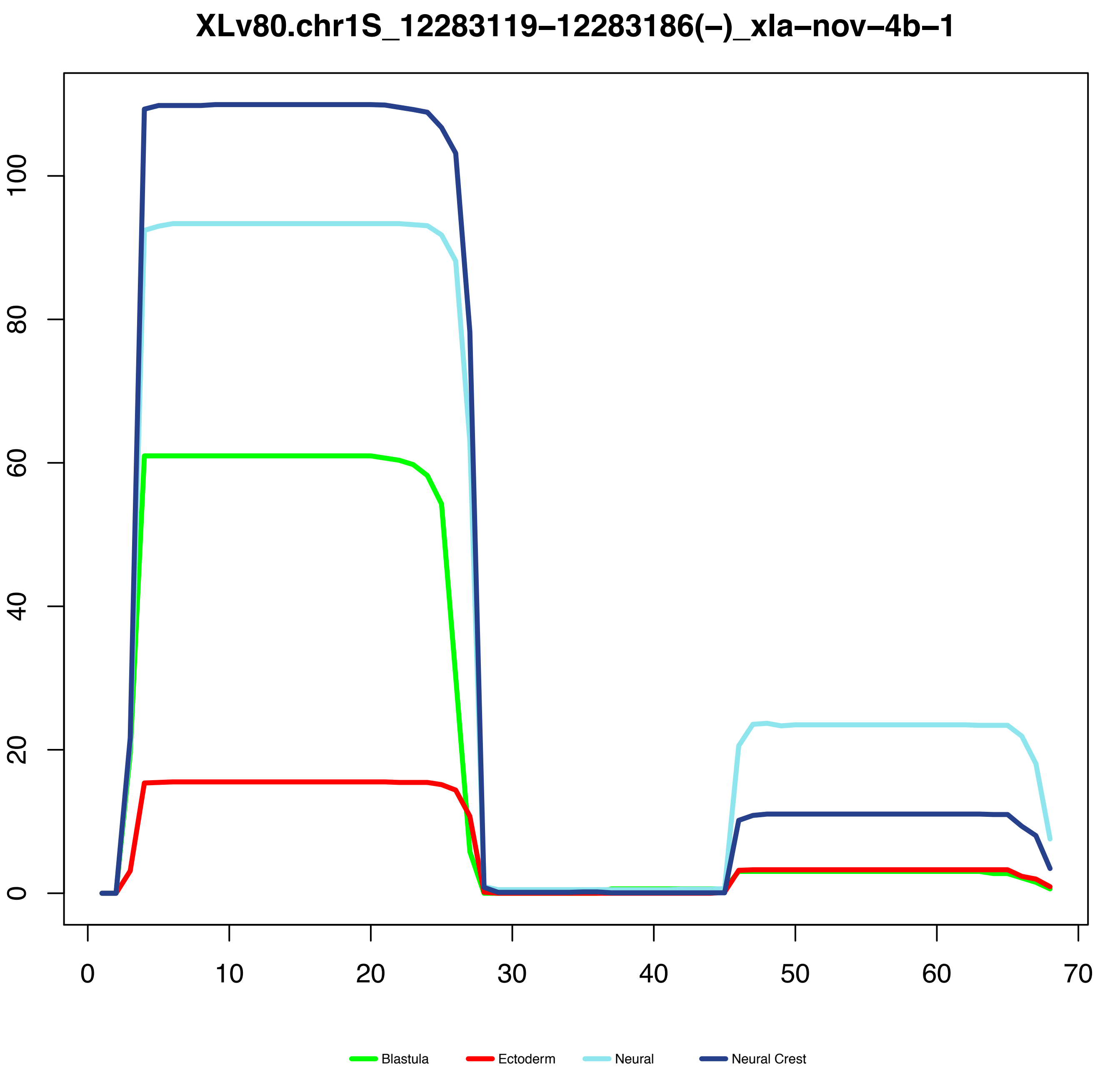
XLv80.chr1S_12283119−12283186(−)_xla−nov−4b−1
0
20
40
60
80
100
0
10
20
30
40
50
60
70
Blastula
Ectoderm
Neural
Neural Crest

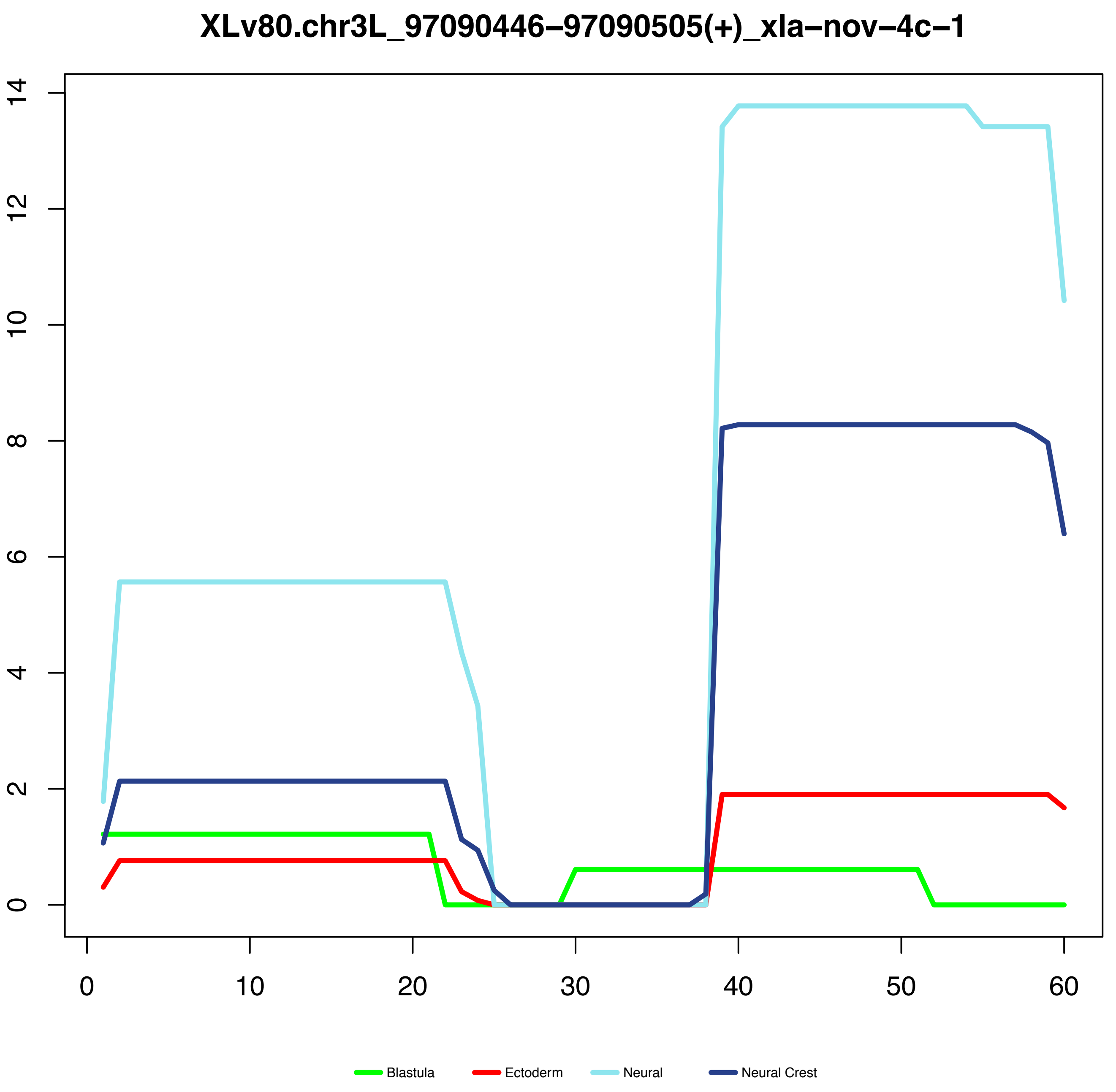

XLv80.chr3L_97090446−97090505(+)_xla−nov−4c−1
0
2
4
6
8
10
12
14
0
10
20
30
40
50
60
Blastula
Ectoderm
Neural
Neural Crest

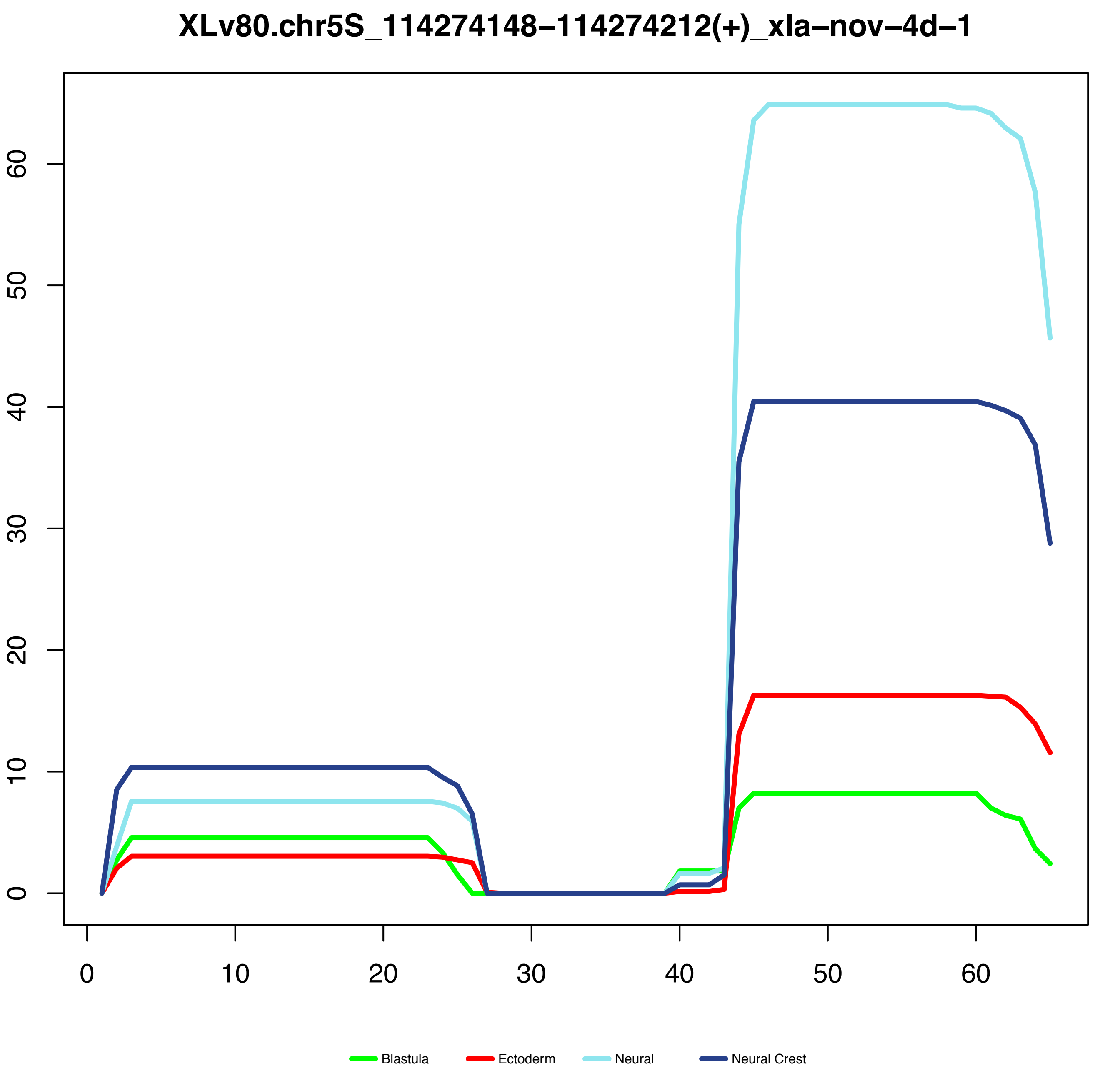
XLv80.chr5S_114274148−114274212(+)_xla−nov−4d−1
0
10
20
30
40
50
60
0
10
20
30
40
50
60
Blastula
Ectoderm
Neural
Neural Crest

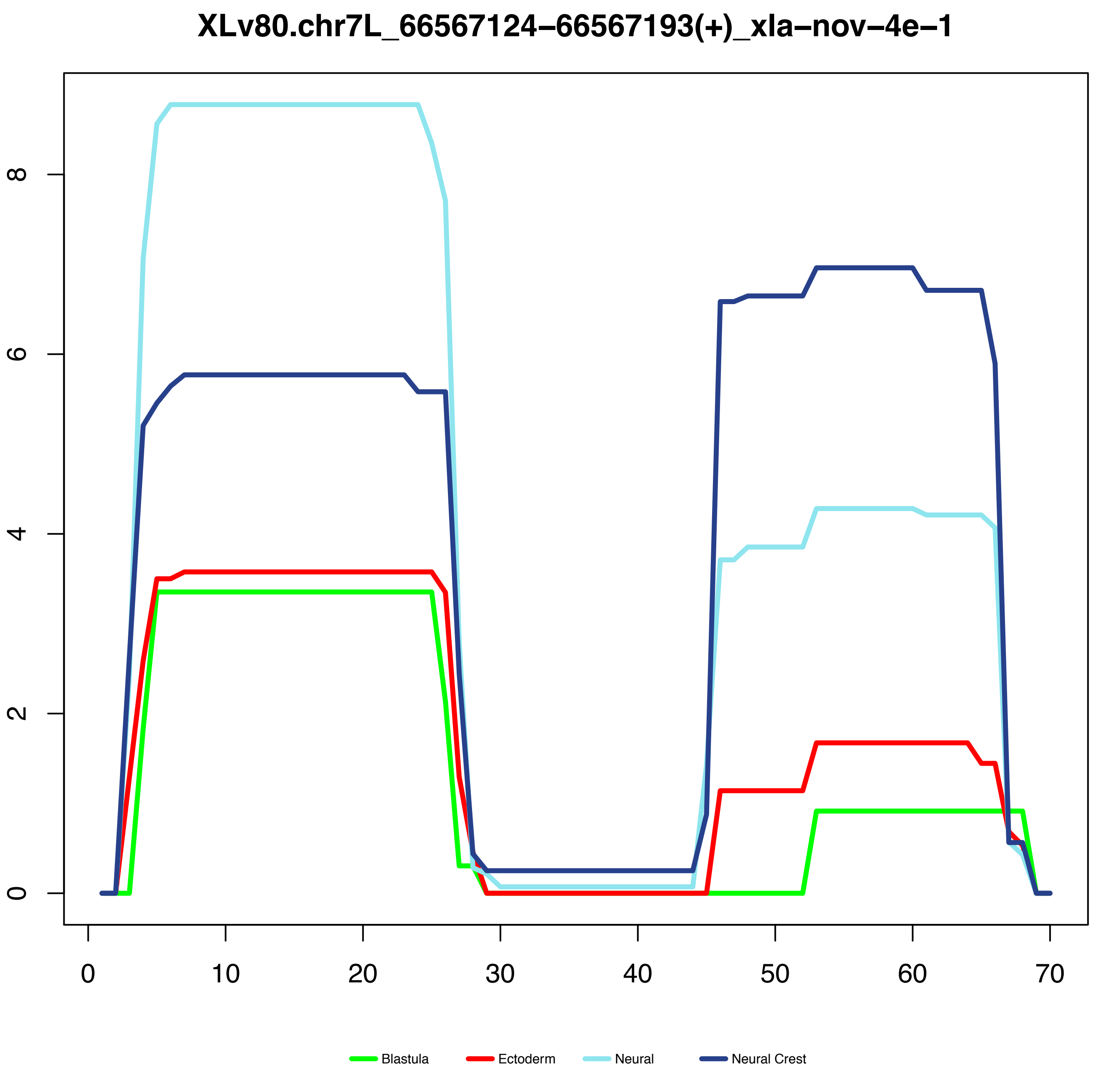

XLv80.chr7L_66567124−66567193(+)_xla−nov−4e−1
0
2
4
6
8
0
10
20
30
40
50
60
70
Blastula
Ectoderm
Neural
Neural Crest

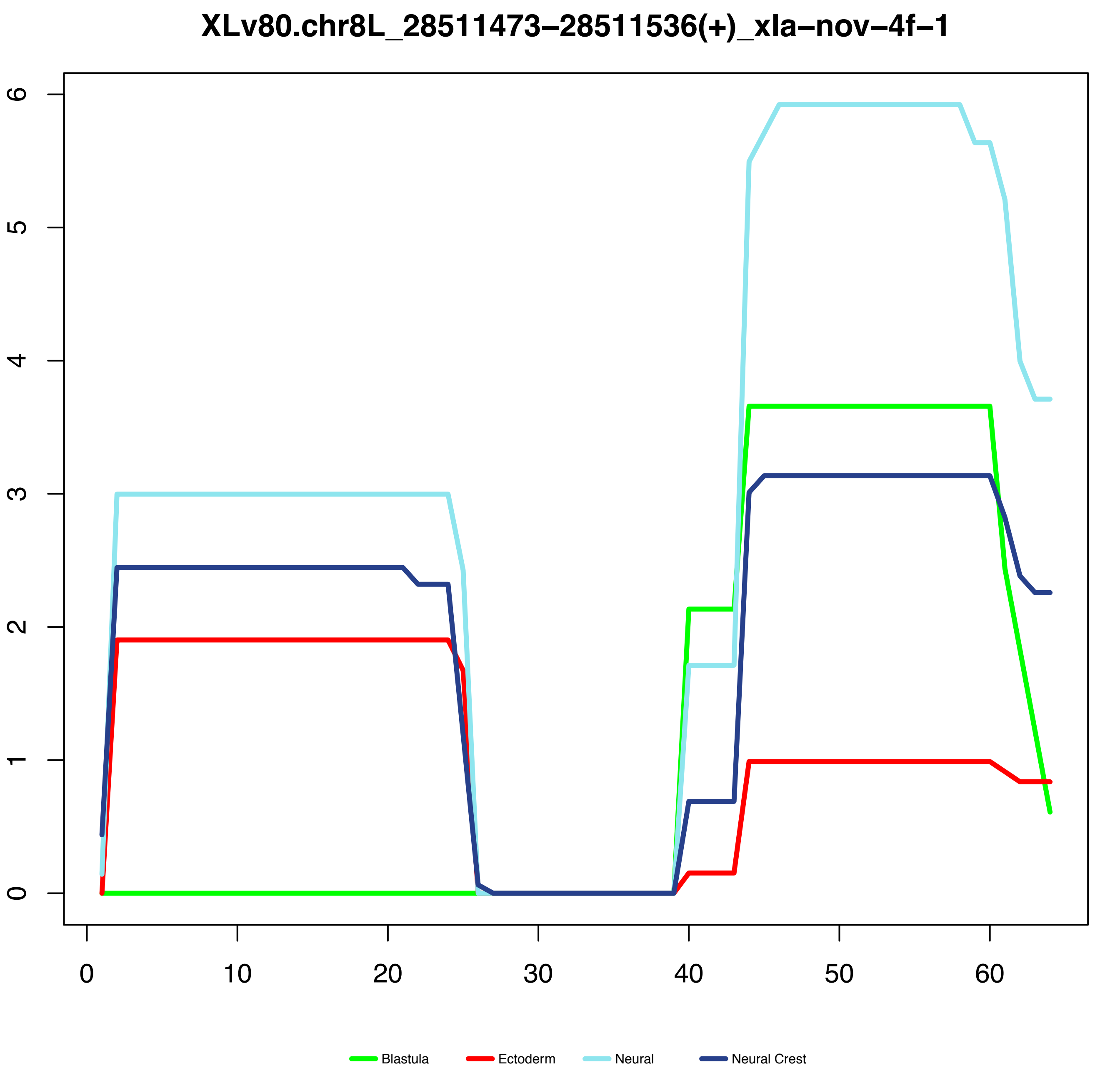
XLv80.chr8L_28511473−28511536(+)_xla−nov−4f−1
0
1
2
3
4
5
6
0
10
20
30
40
50
60
Blastula
Ectoderm
Neural
Neural Crest

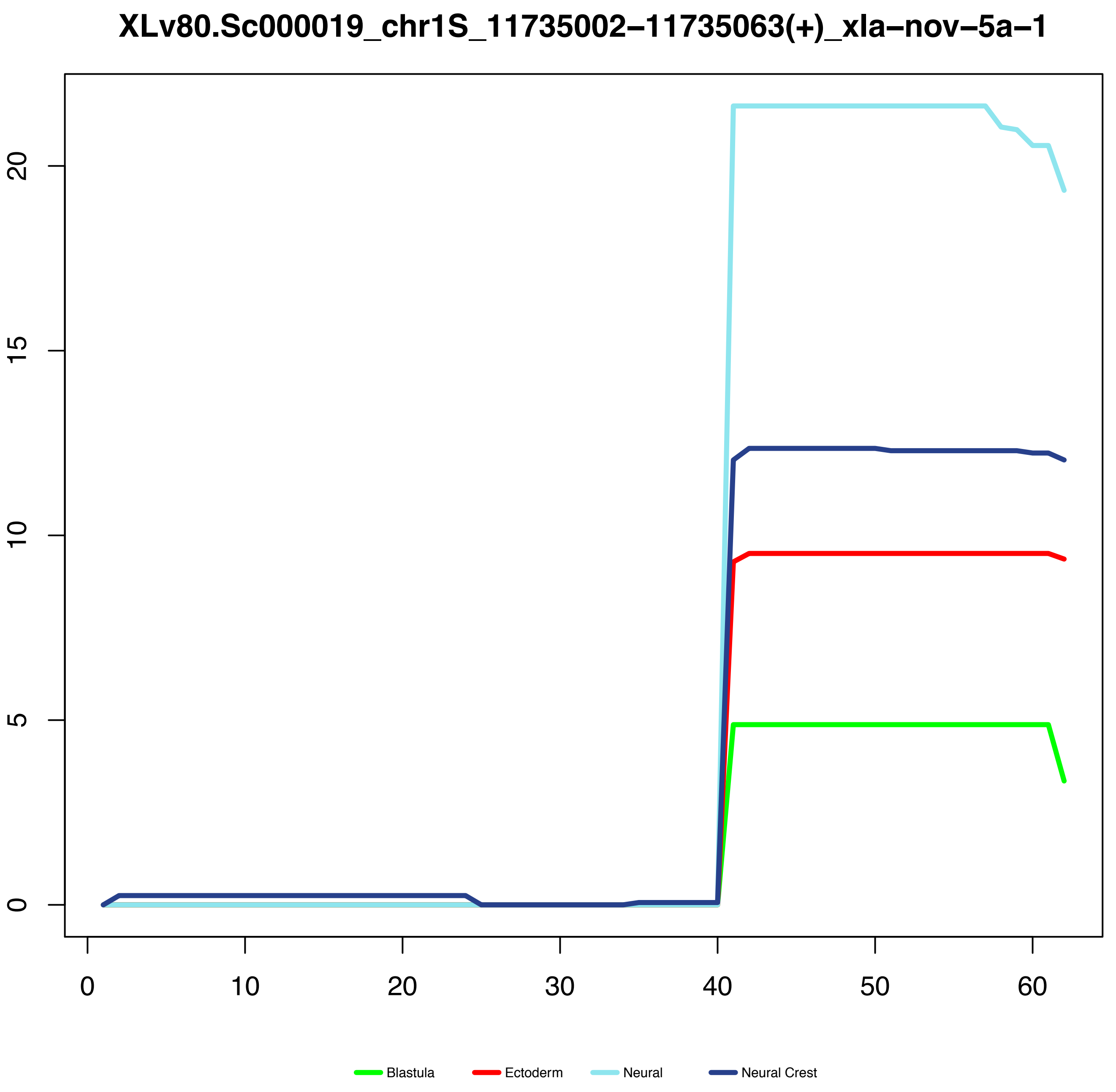

XLv80.Sc000019_chr1S_11735002−11735063(+)_xla−nov−5a−1
0
5
10
15
20
0
10
20
30
40
50
60
Blastula
Ectoderm
Neural
Neural Crest

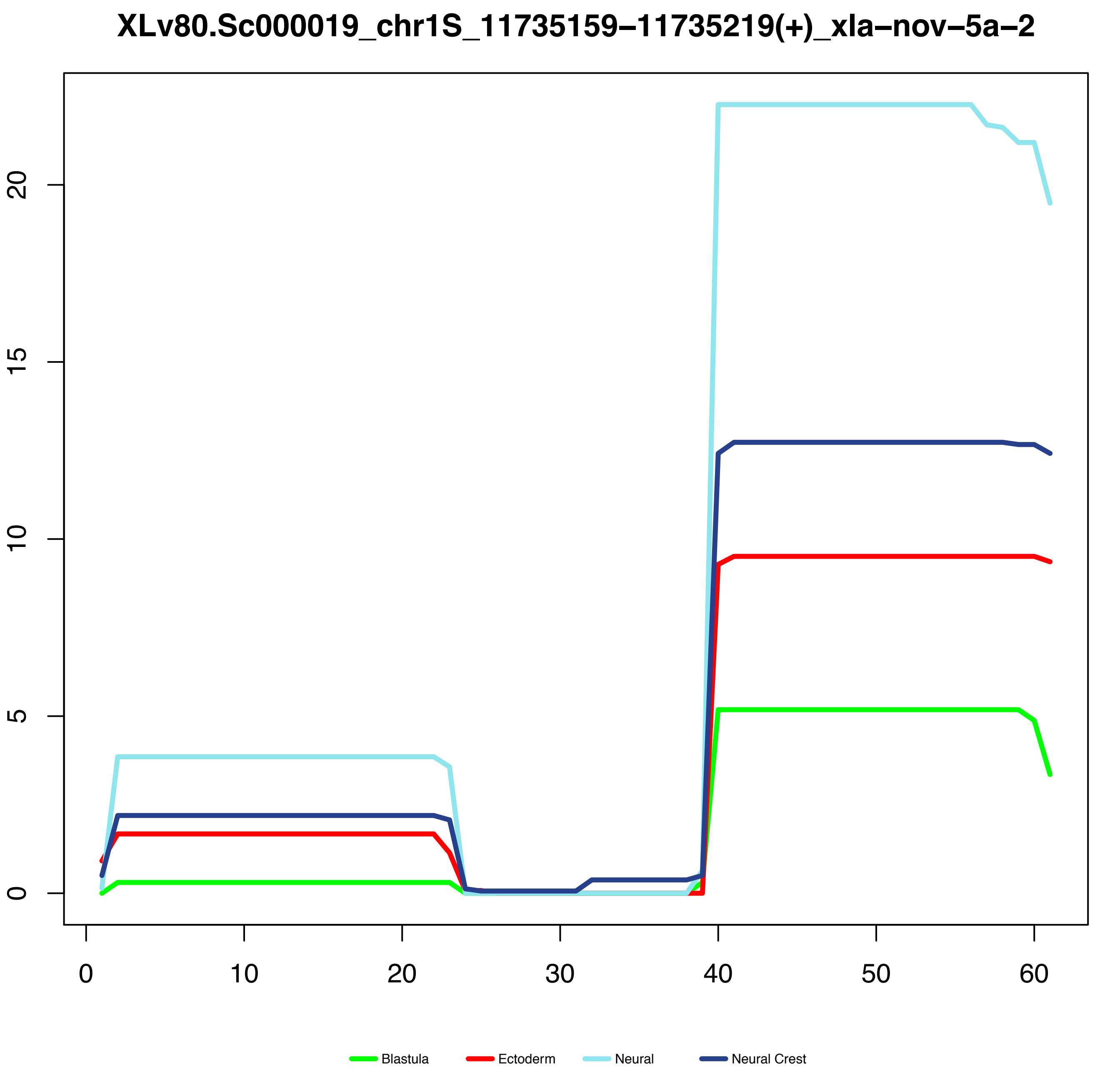

XLv80.Sc000019_chr1S_11735159−11735219(+)_xla−nov−5a−2
0
5
10
15
20
0
10
20
30
40
50
60
Blastula
Ectoderm
Neural
Neural Crest

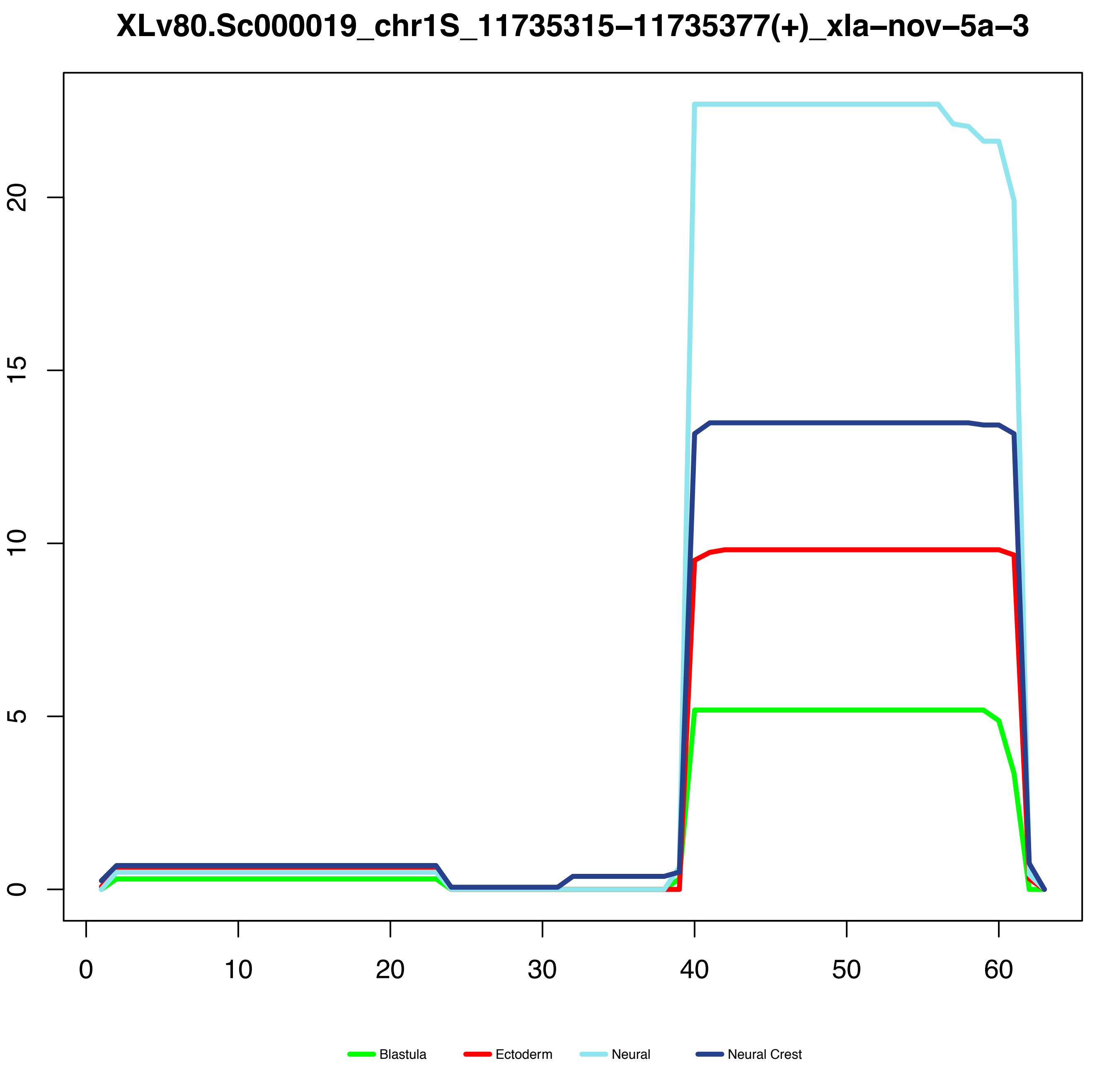
XLv80.Sc000019_chr1S_11735315−11735377(+)_xla−nov−5a−3
0
5
10
15
20
0
10
20
30
40
50
60
Blastula
Ectoderm
Neural
Neural Crest

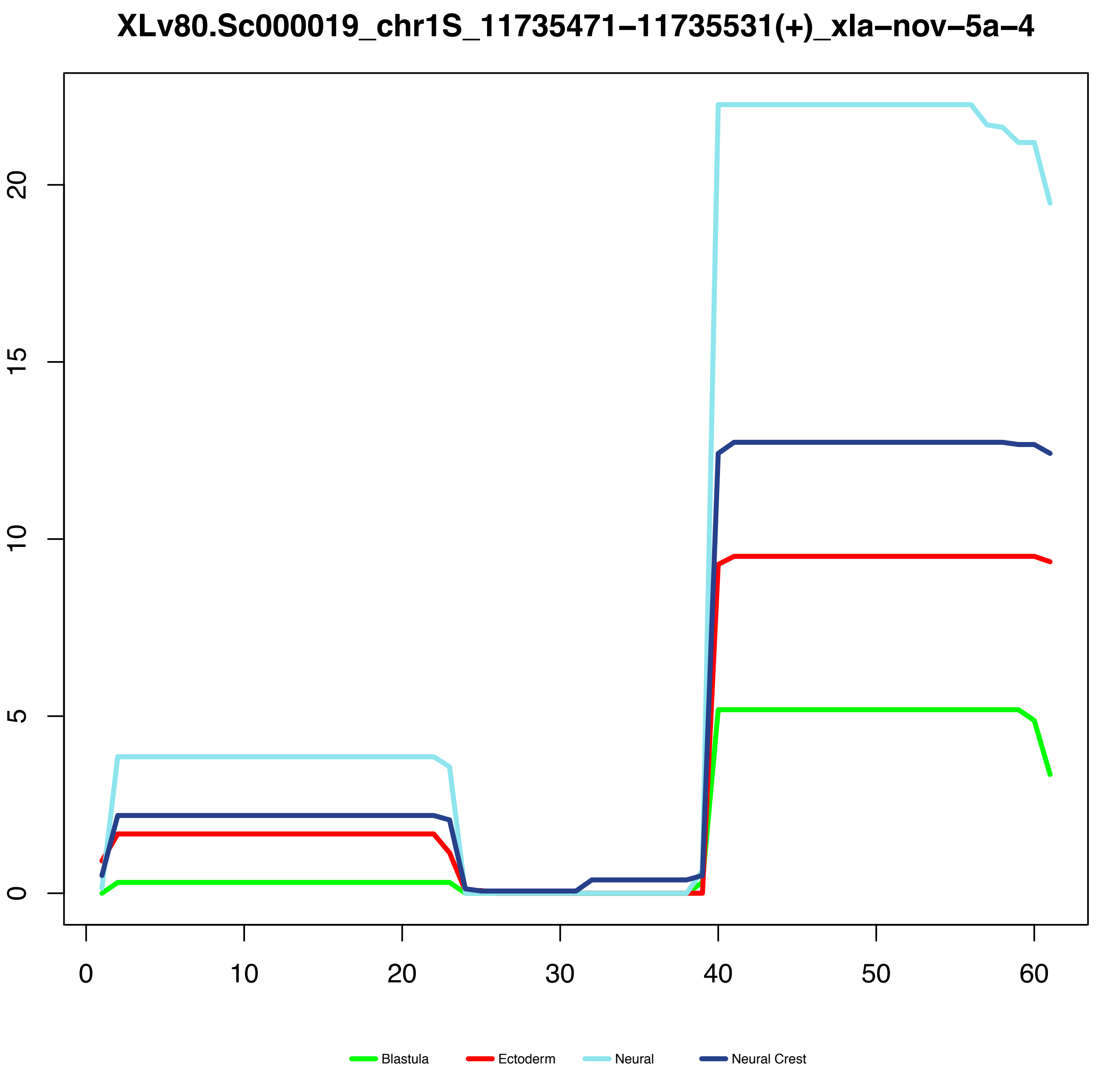
XLv80.Sc000019_chr1S_11735471−11735531(+)_xla−nov−5a−4
0
5
10
15
20
0
10
20
30
40
50
60
Blastula
Ectoderm
Neural
Neural Crest

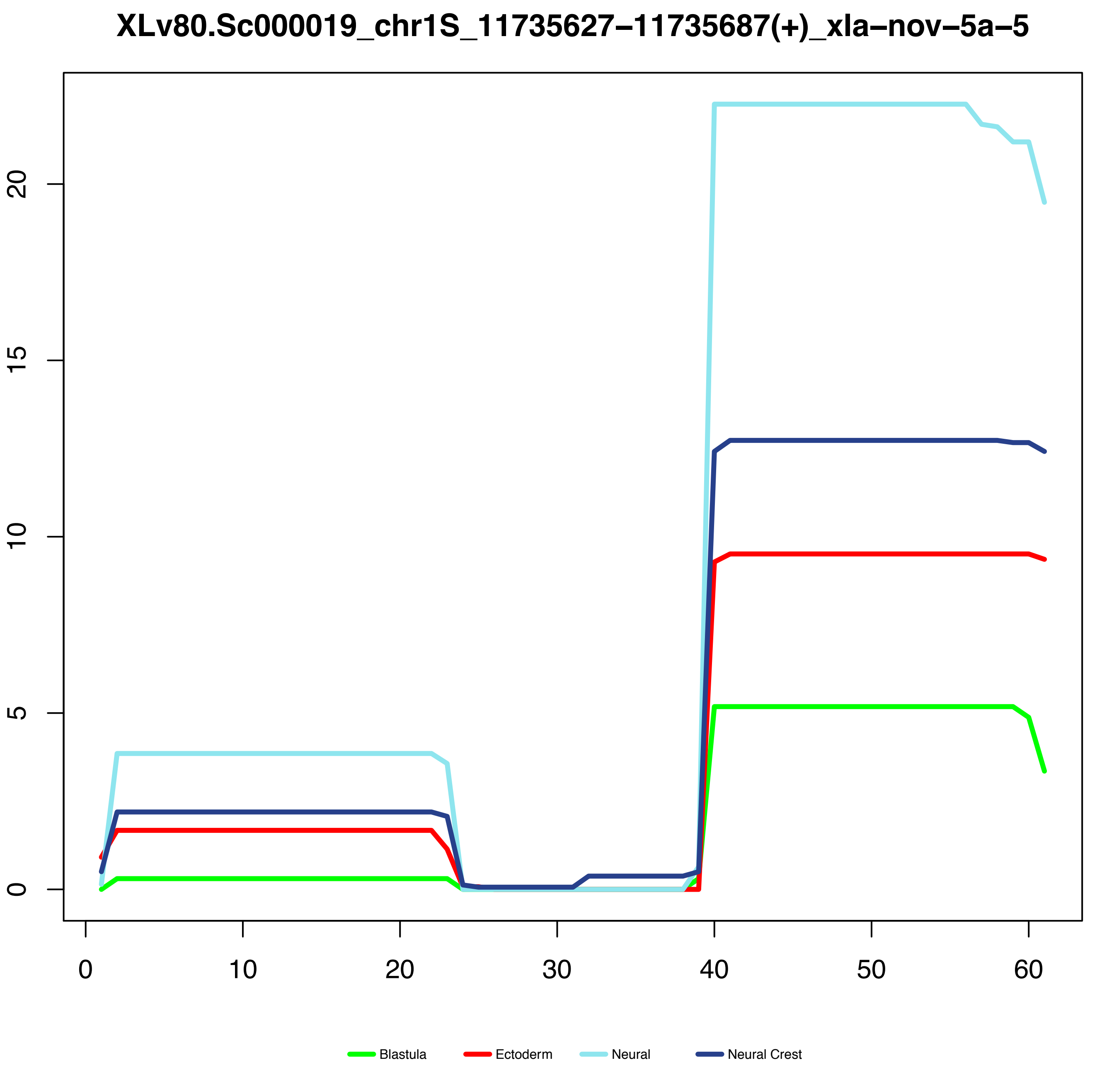
XLv80.Sc000019_chr1S_11735627−11735687(+)_xla−nov−5a−5
0
5
10
15
20
0
10
20
30
40
50
60
Blastula
Ectoderm
Neural
Neural Crest

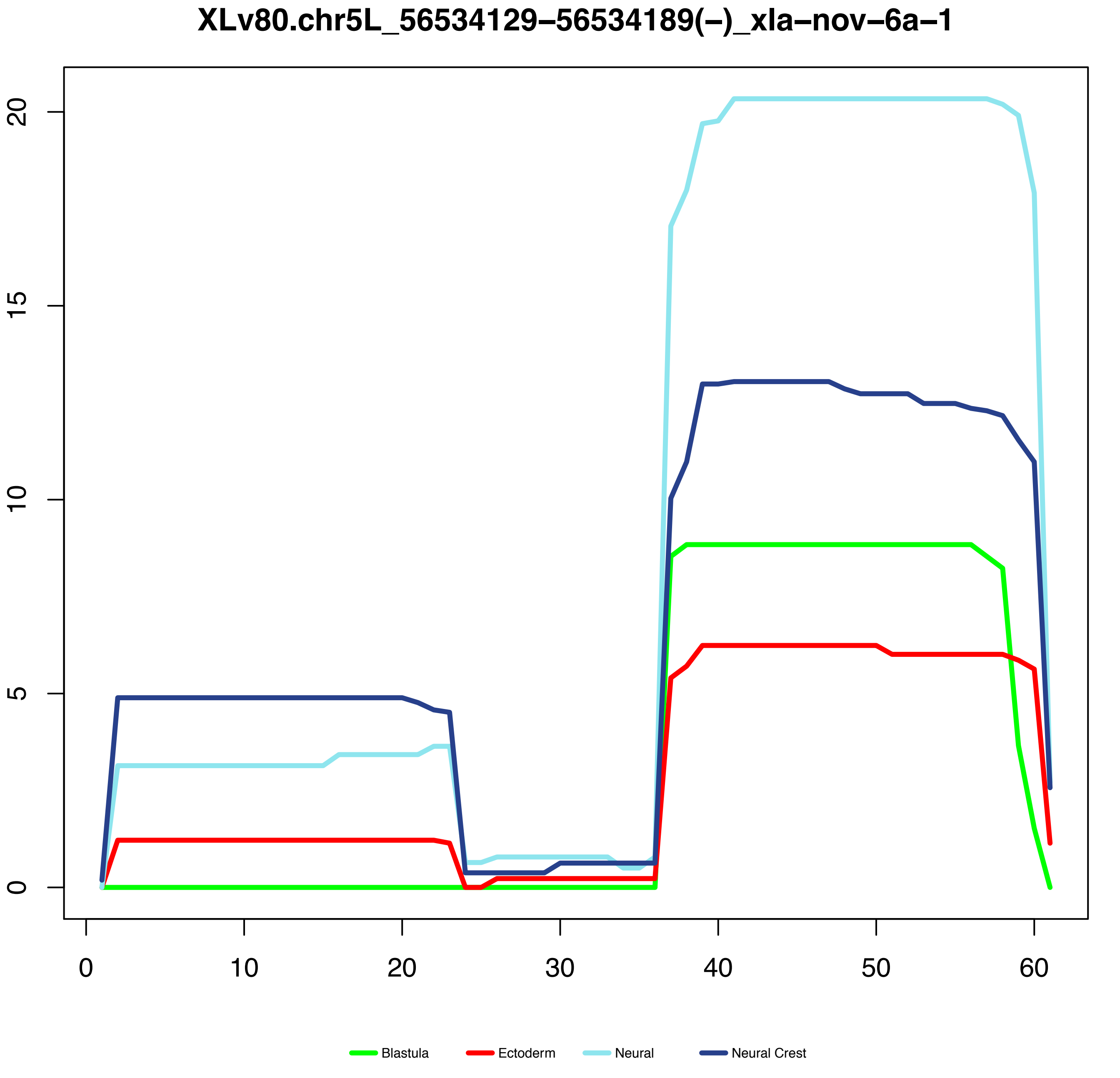

XLv80.chr5L_56534129–56534189(–)_xla–nov–6a–1
0
5
10
15
20
0
10
20
30
40
50
60
Blastula
Ectoderm
Neural
Neural Crest

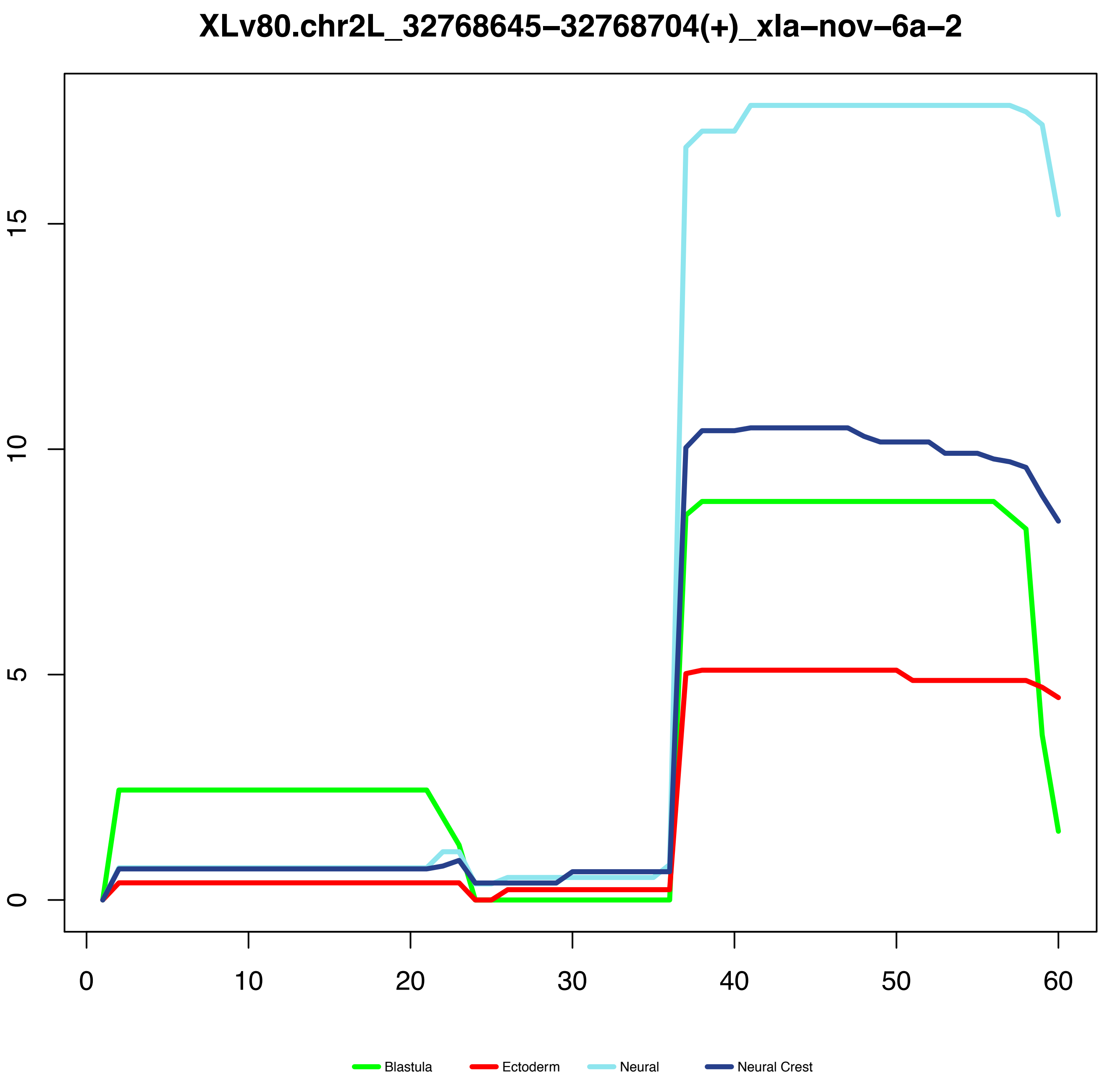
XLv80.chr2L_32768645−32768704(+)_xla−nov−6a−2
0
5
10
15
0
10
20
30
40
50
60
Blastula
Ectoderm
Neural
Neural Crest

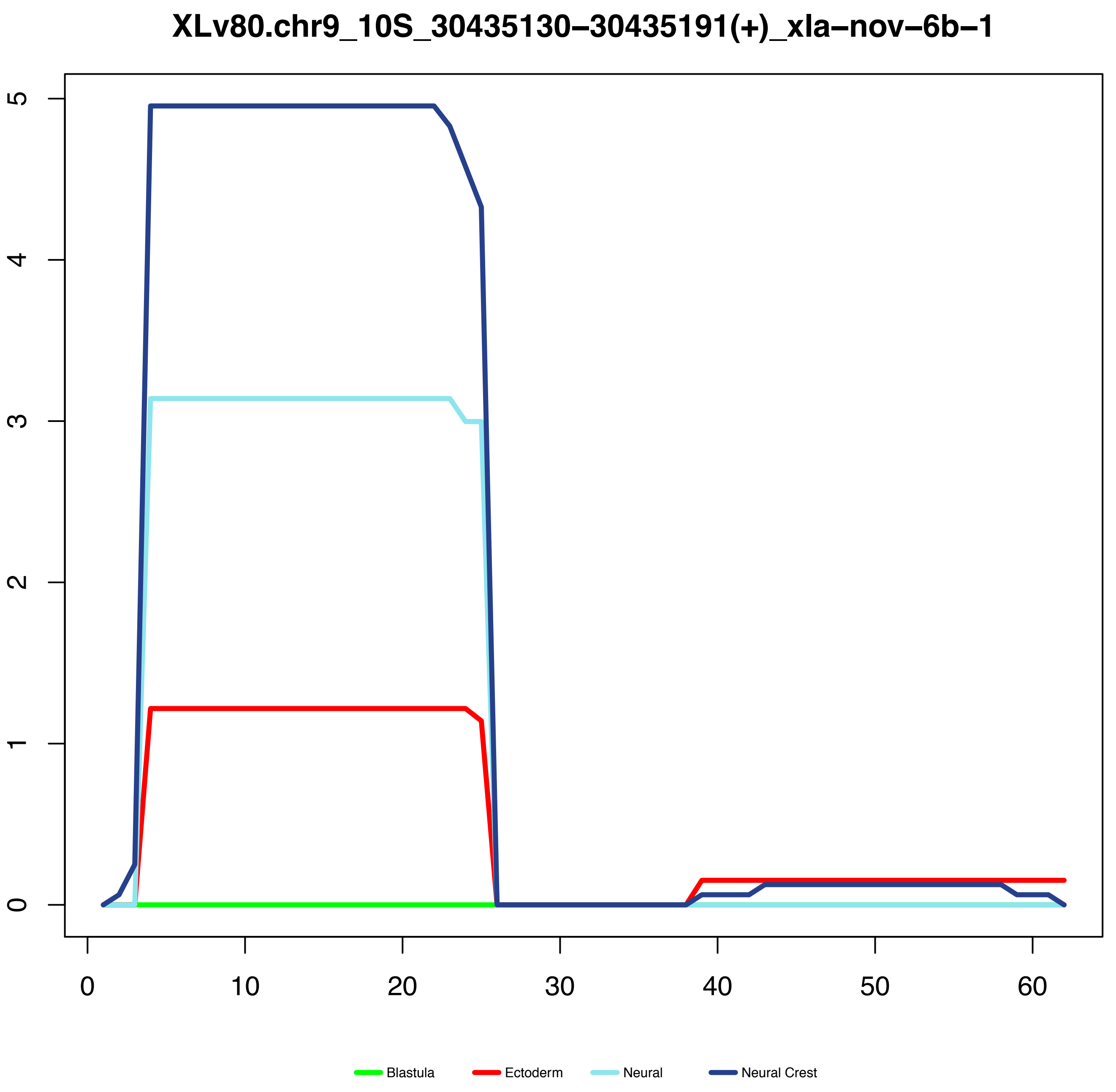

XLv80.chr9_10S_30435130−30435191(+)_xla−nov−6b−1
0
1
2
3
4
5
0
10
20
30
40
50
60
Blastula
Ectoderm
Neural
Neural Crest

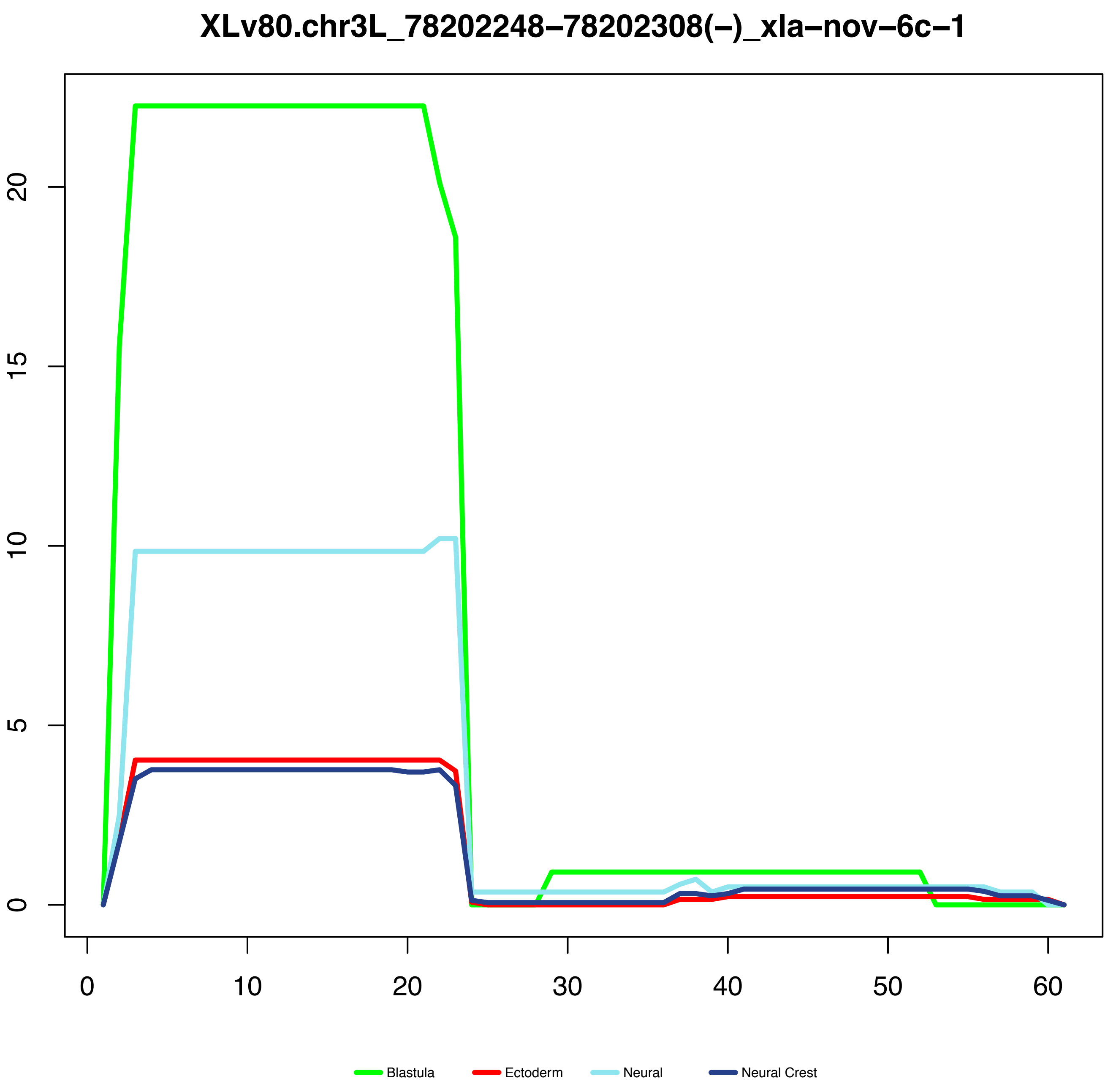
XLv80.chr3L_78202248−78202308(−)_xla−nov−6c−1
0
5
10
15
20
0
10
20
30
40
50
60
Blastula
Ectoderm
Neural
Neural Crest

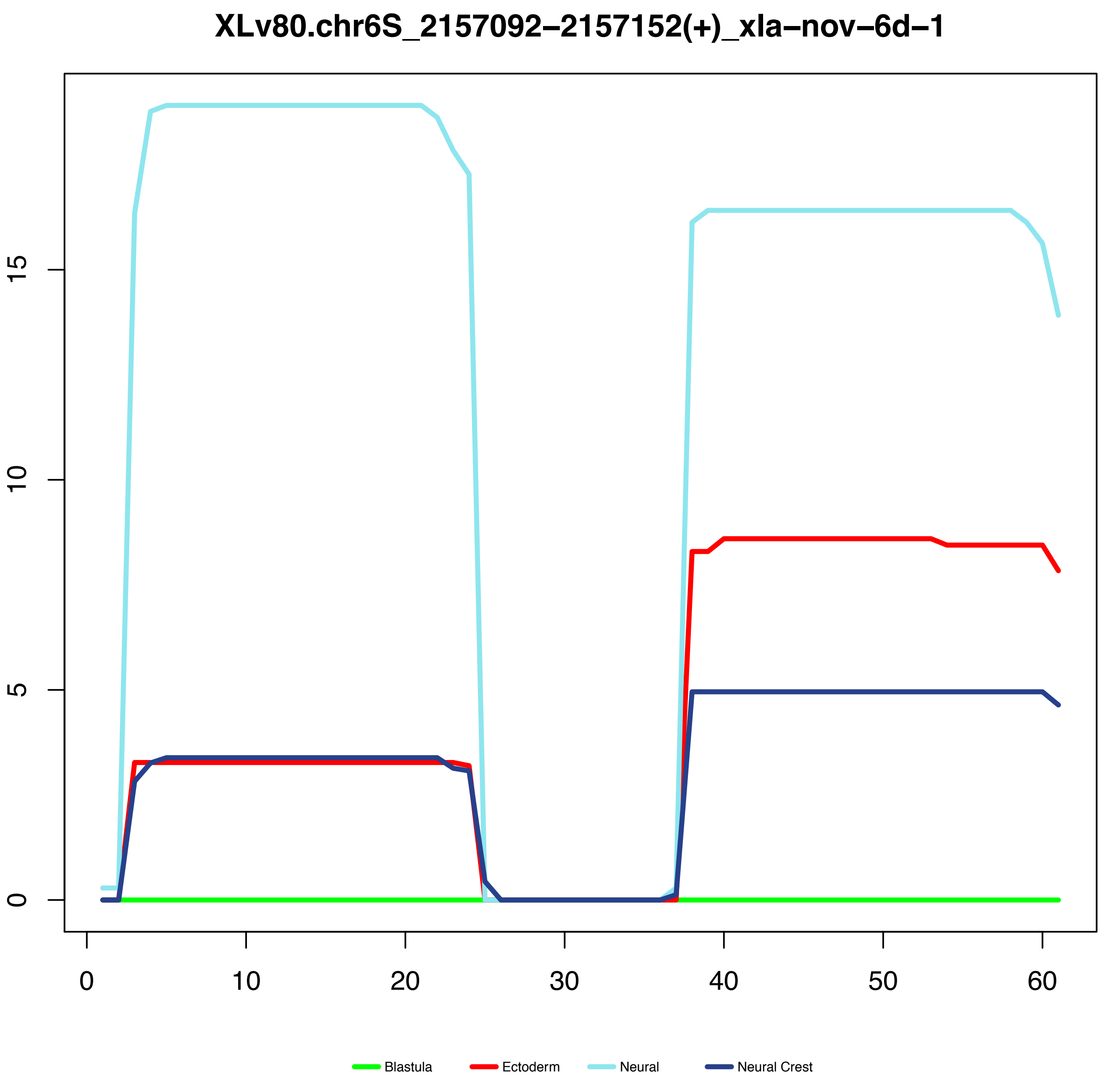
XLv80.chr6S_2157092−2157152(+)_xla−nov−6d−1
0
5
10
15
0
10
20
30
40
50
60
Blastula
Ectoderm
Neural
Neural Crest

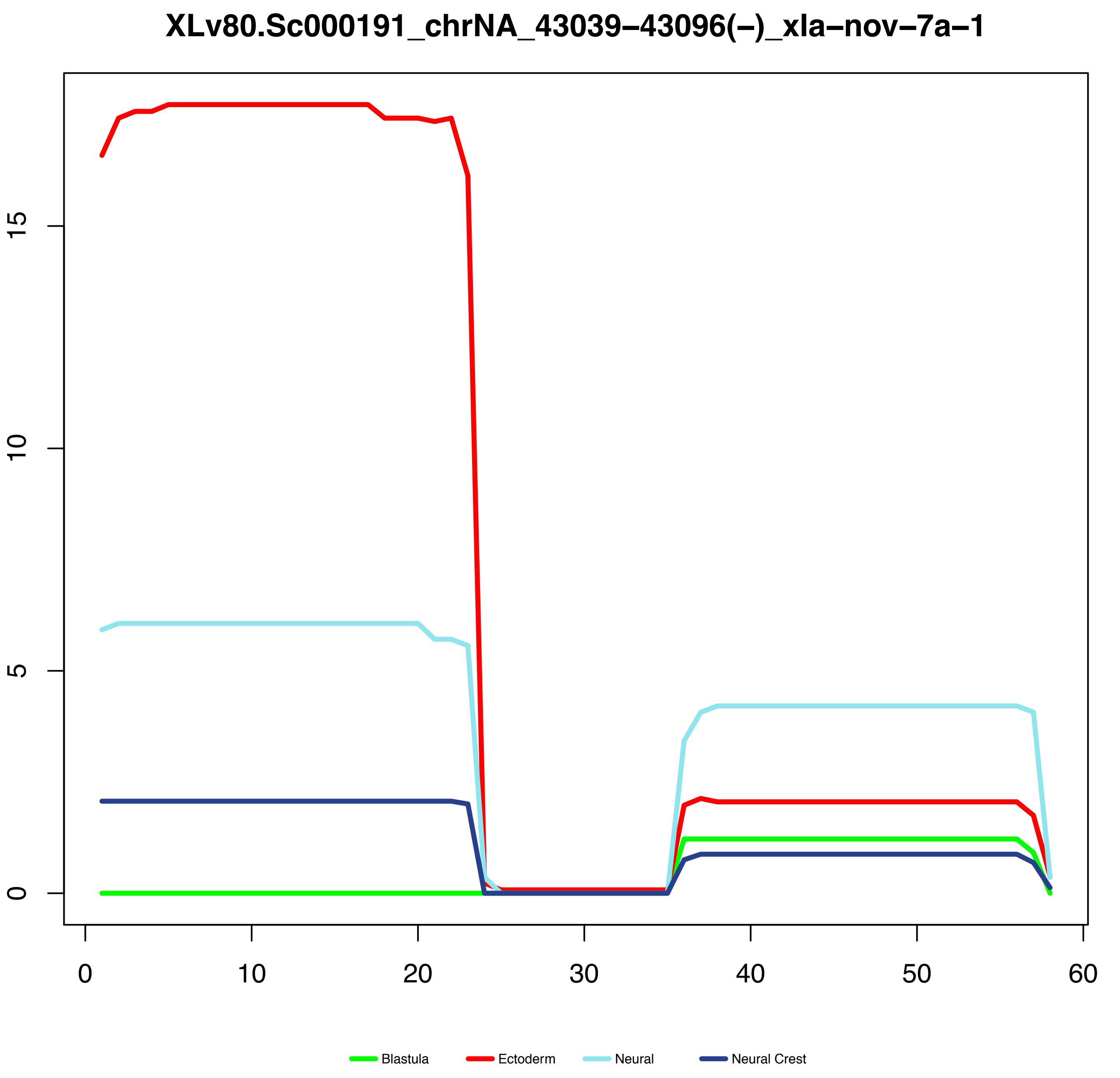
XLv80.Sc000191_chrNA_43039−43096(−)_xla−nov−7a−1
0
5
10
15
0
10
20
30
40
50
60
Blastula
Ectoderm
Neural
Neural Crest

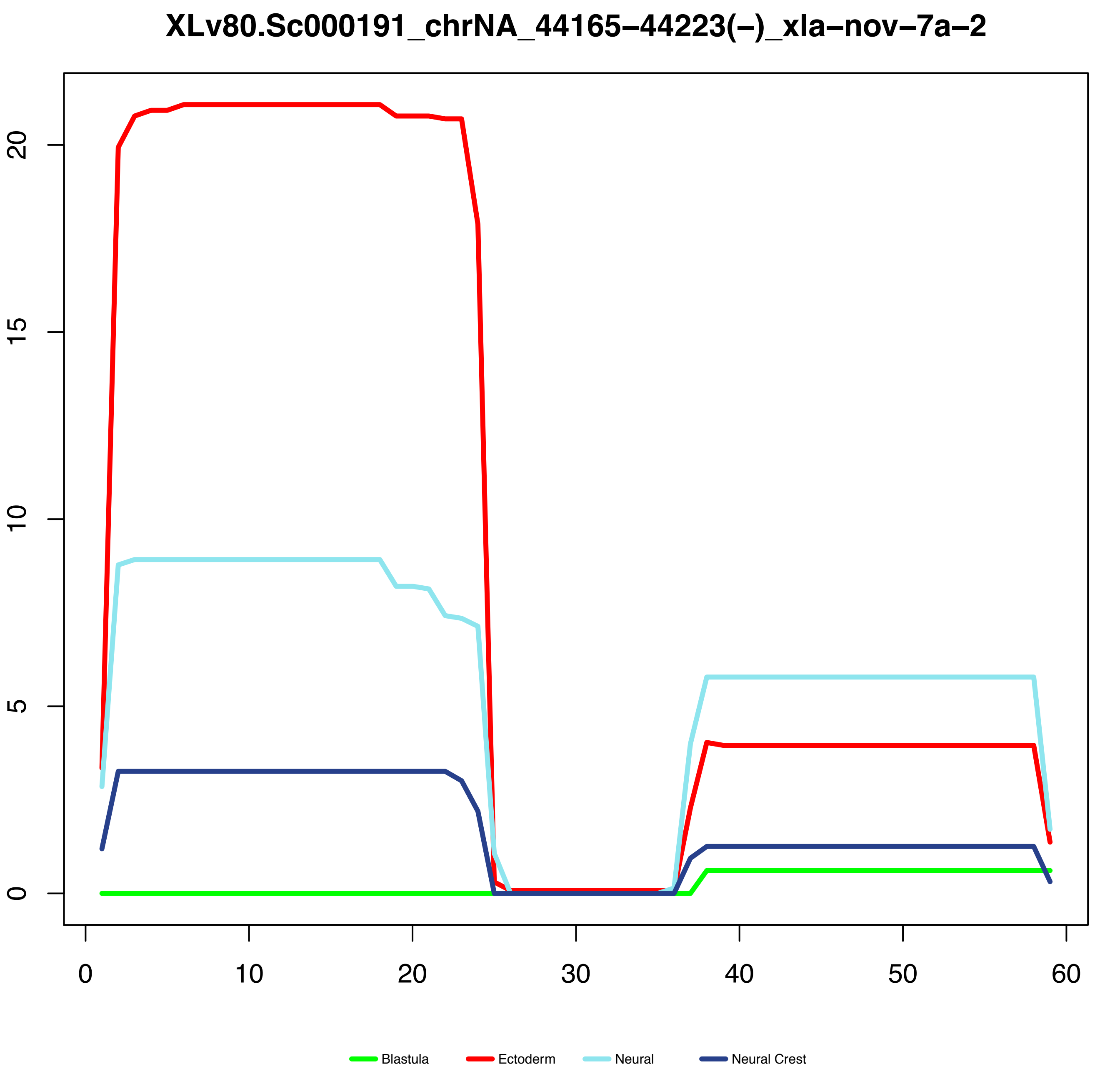
XLv80.Sc000191_chrNA_44165−44223(−)_xla−nov−7a−2
0
5
10
15
20
0
10
20
30
40
50
60
Blastula
Ectoderm
Neural
Neural Crest

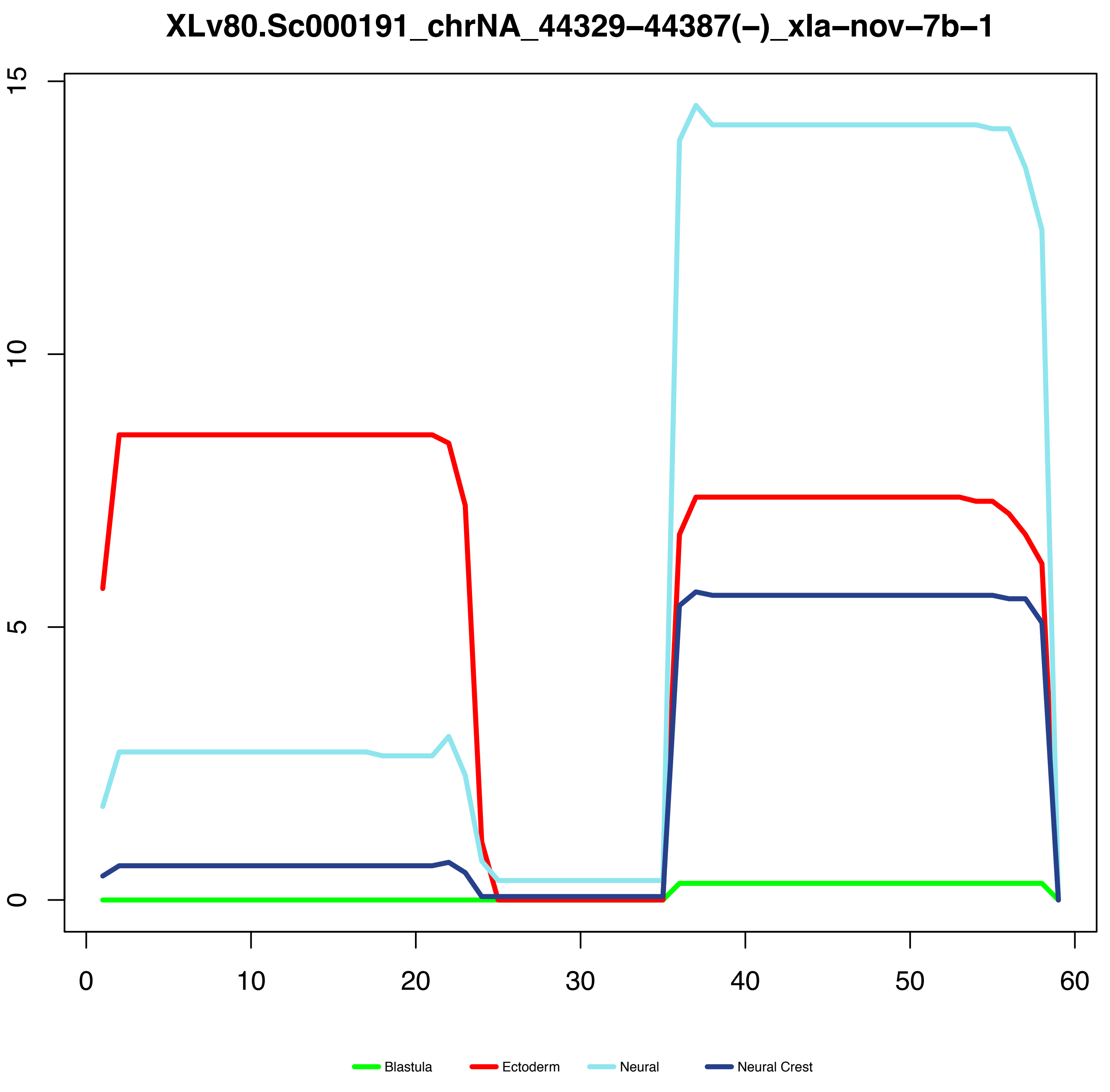

XLv80.Sc000191_chrNA_44329−44387(−)_xla−nov−7b−1
0
5
10
15
0
10
20
30
40
50
60
Blastula
Ectoderm
Neural
Neural Crest

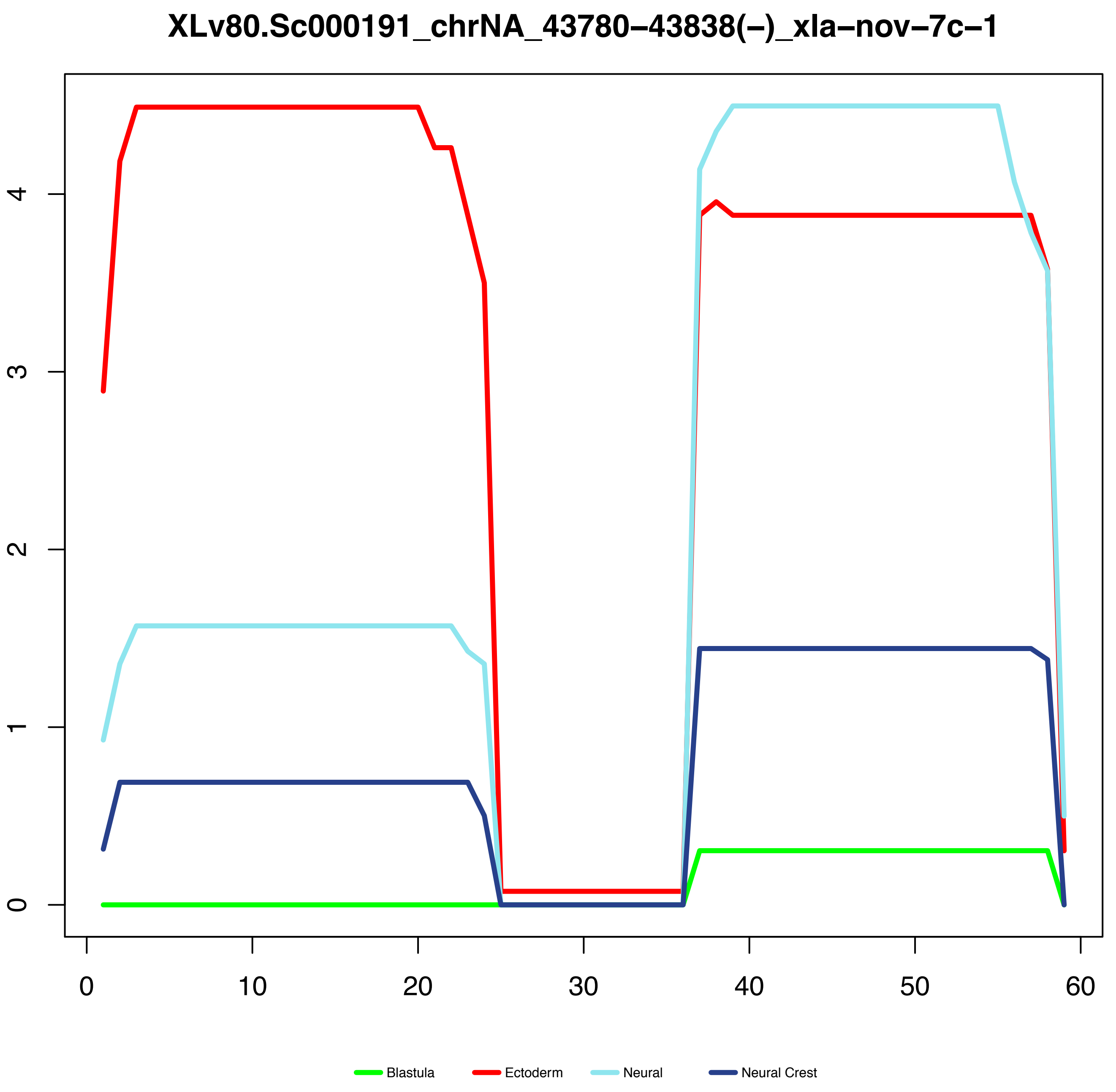
XLv80.Sc000191_chrNA_43780−43838(−)_xla−nov−7c−1
0
1
2
3
4
0
10
20
30
40
50
60
Blastula
Ectoderm
Neural
Neural Crest

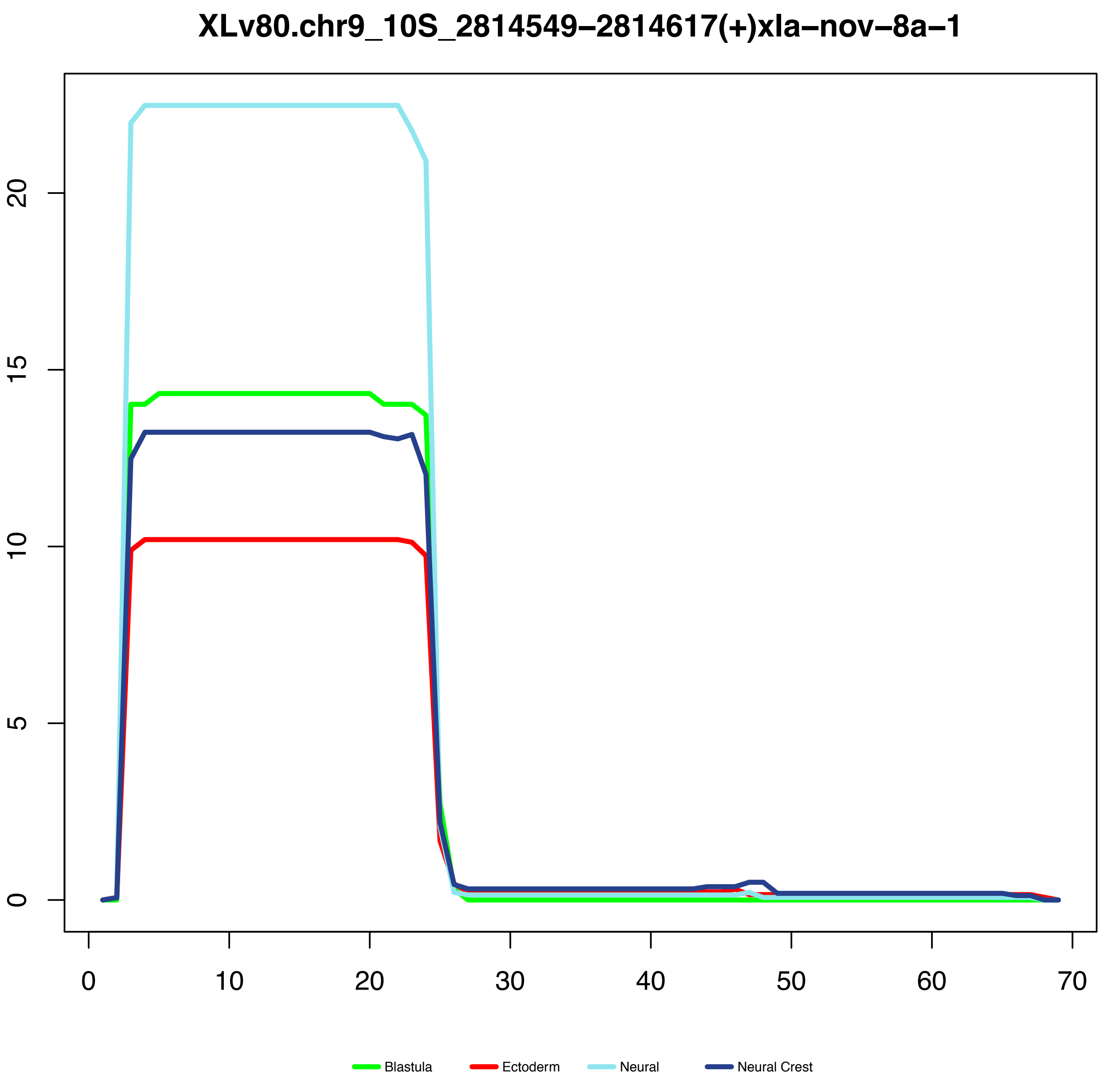
XLv80.chr9_10S_2814549−2814617(+)xla−nov−8a−1
0
5
10
15
20
0
10
20
30
40
50
60
70
Blastula
Ectoderm
Neural
Neural Crest

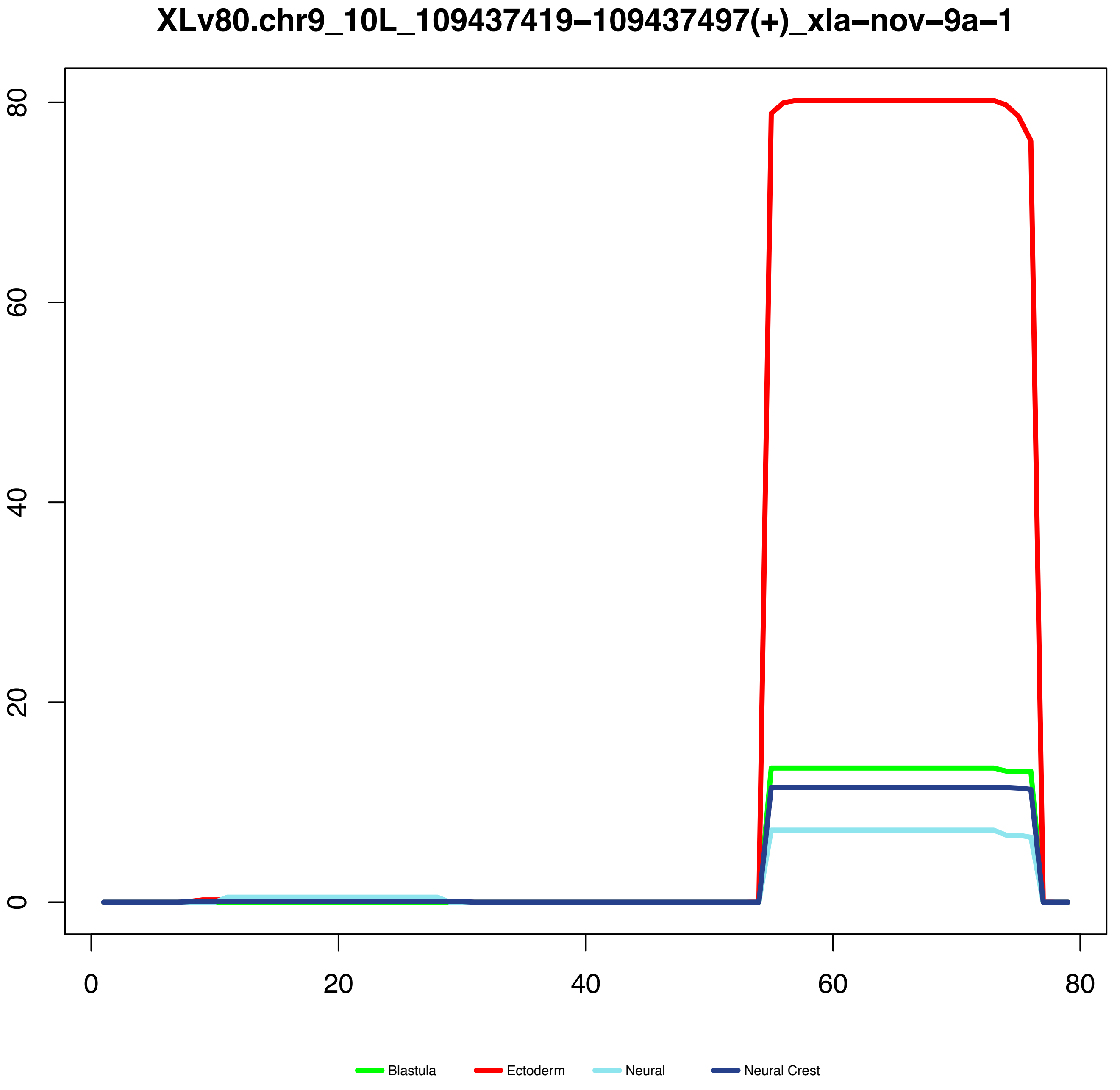

XLv80.chr9_10L_109437419−109437497(+)_xla−nov−9a−1
0
20
40
60
80
0
20
40
60
80
Blastula
Ectoderm
Neural
Neural Crest

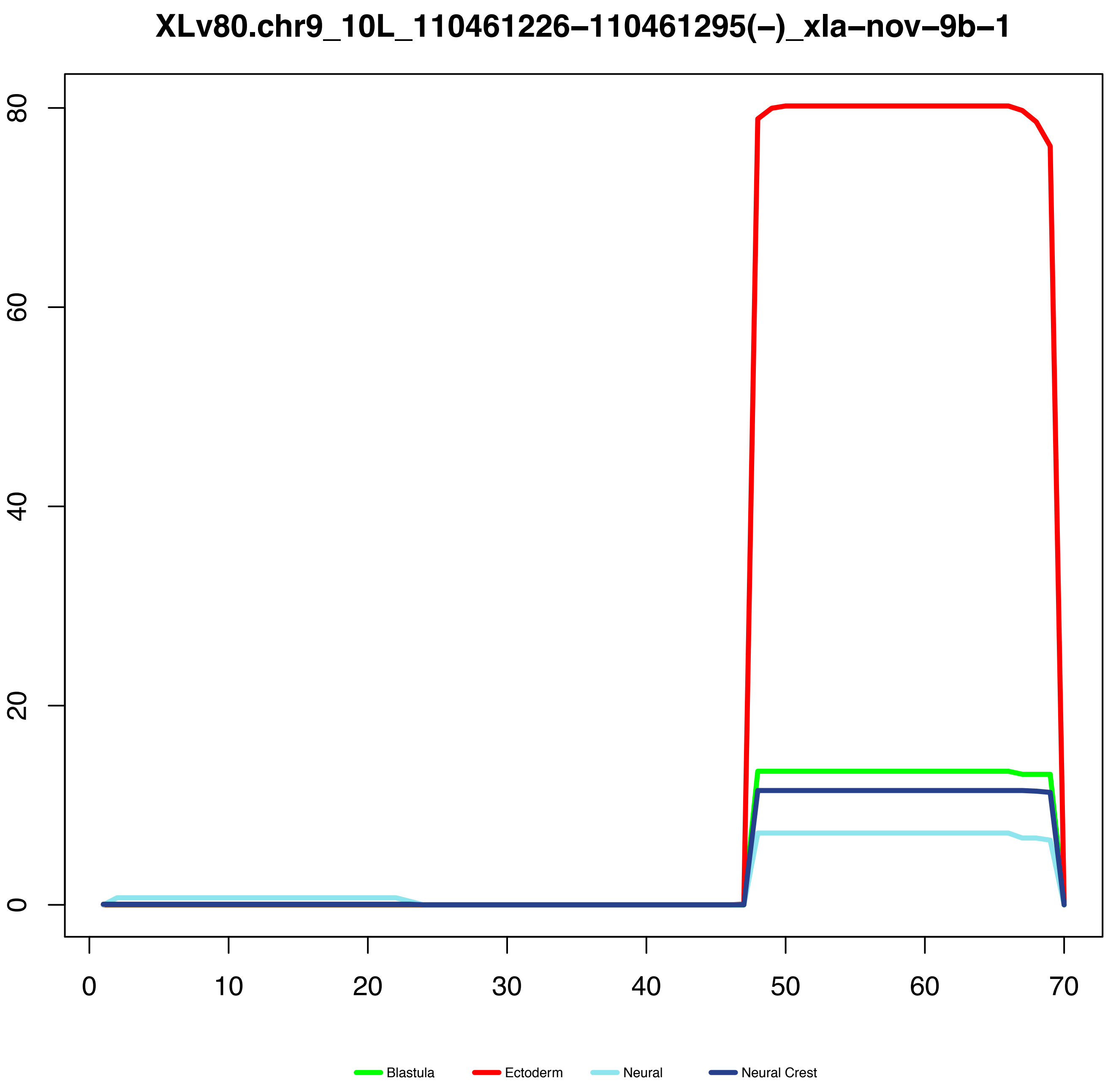
XLv80.chr9_10L_110461226−110461295(−)_xla−nov−9b−1
0
20
40
60
80
0
10
20
30
40
50
60
70
Blastula
Ectoderm
Neural
Neural Crest

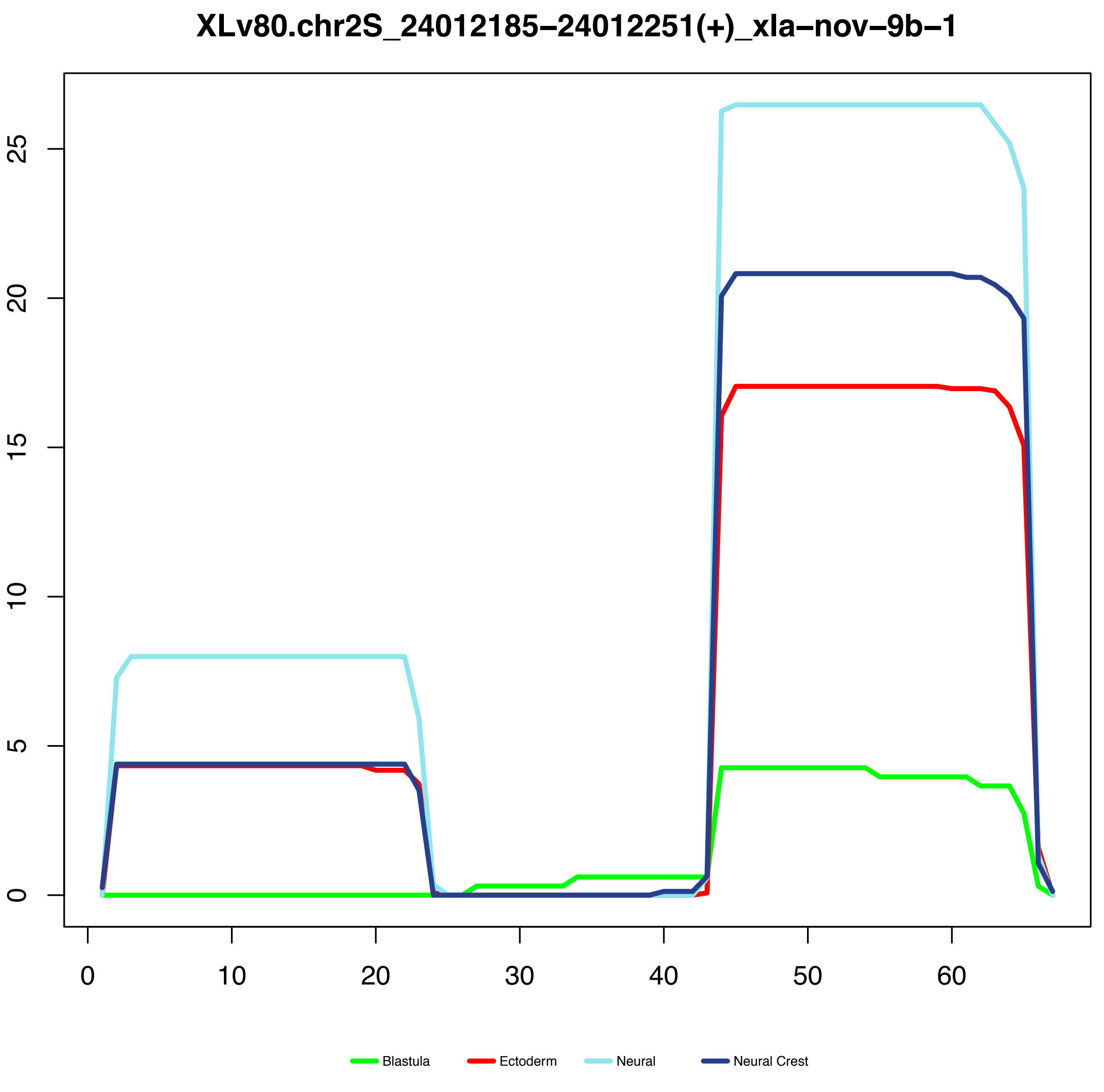
XLv80.chr2S_24012185−24012251(+)_xla−nov−9b−1
0
5
10
15
20
25
0
10
20
30
40
50
60
Blastula
Ectoderm
Neural
Neural Crest

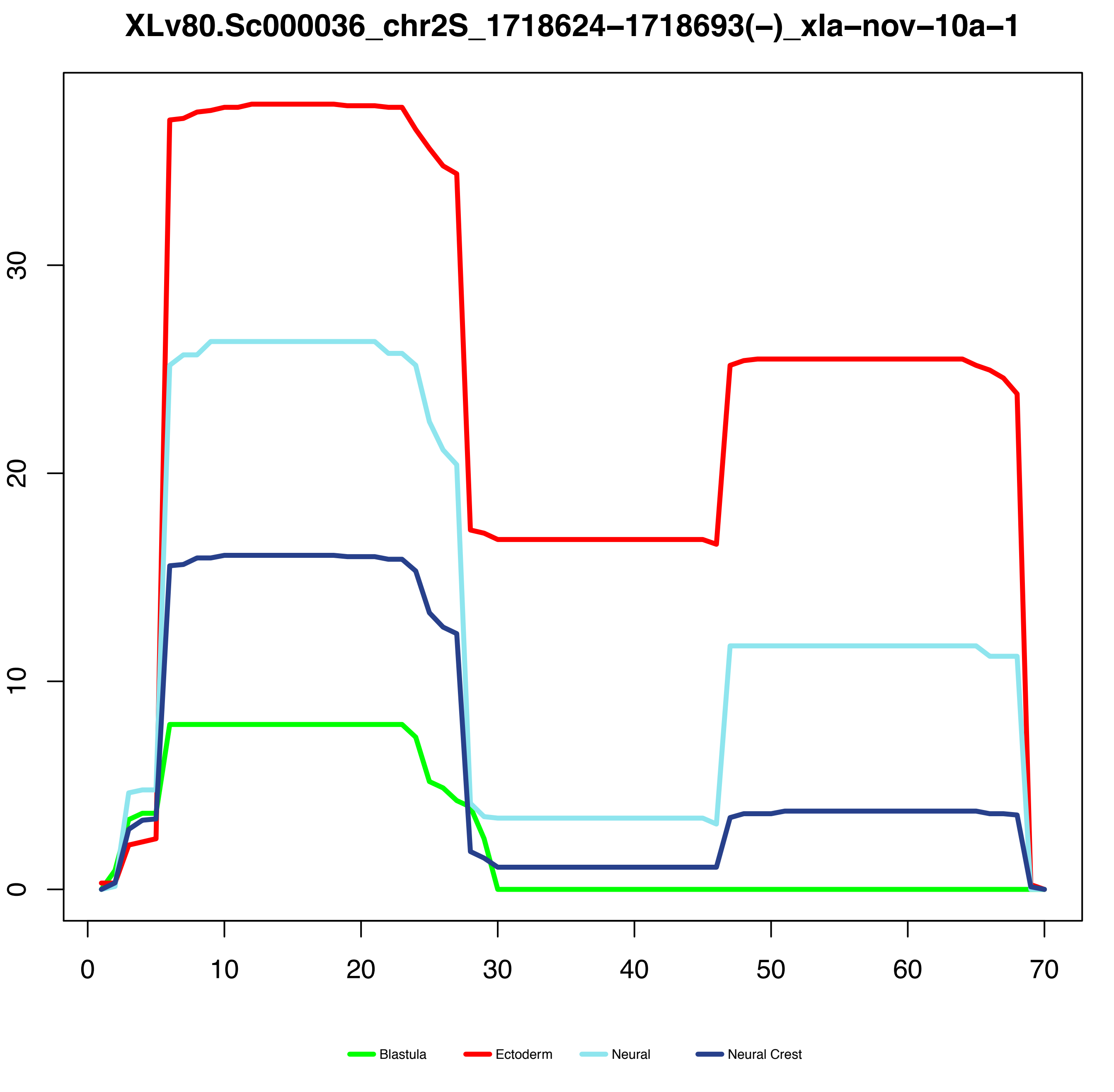
XLv80.Sc000036_chr2S_1718624−1718693(−)_xla−nov−10a−1
0
10
20
30
0
10
20
30
40
50
60
70
Blastula
Ectoderm
Neural
Neural Crest

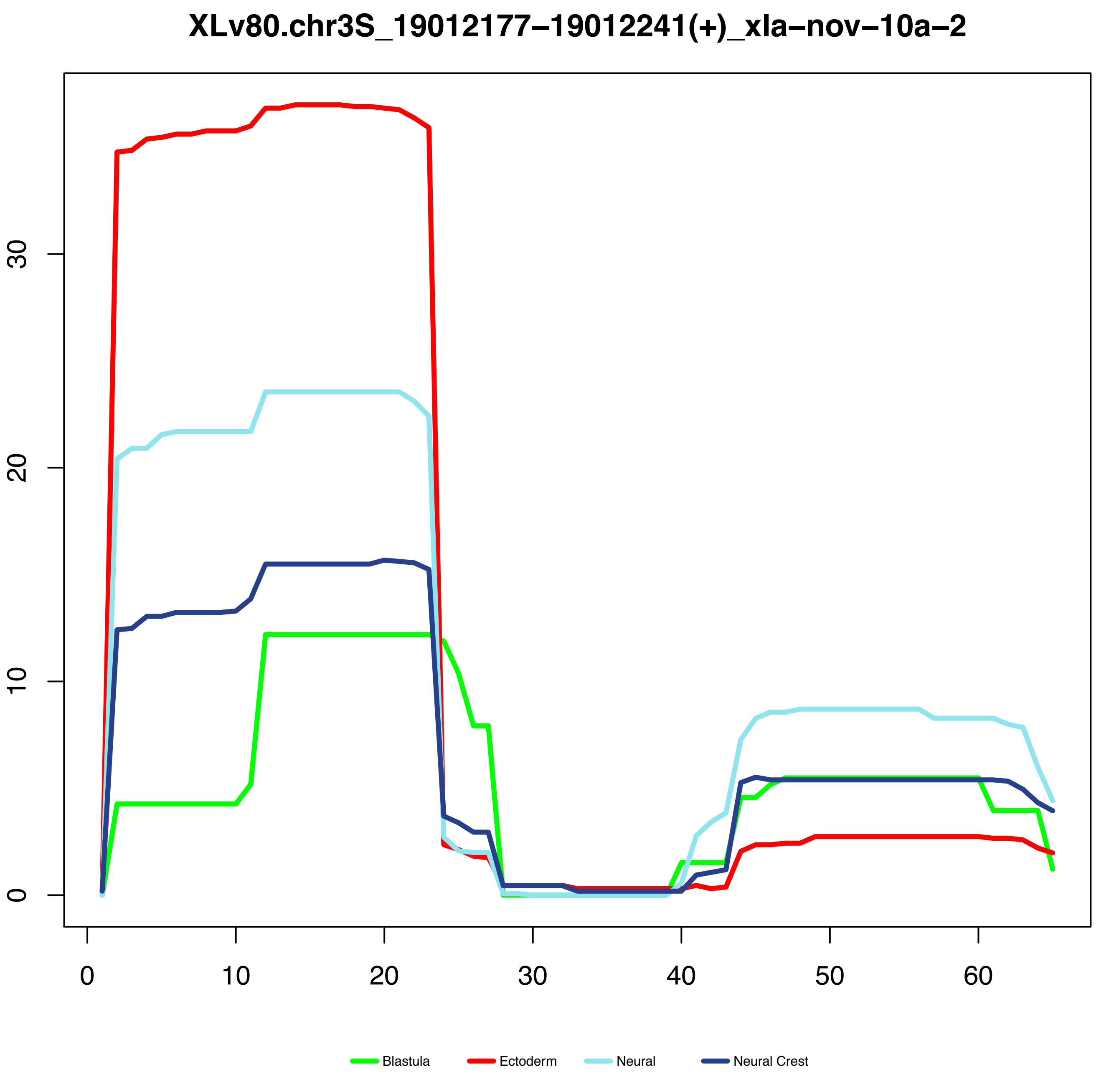
XLv80.chr3S_19012177−19012241(+)_xla−nov−10a−2
0
10
20
30
0
10
20
30
40
50
60
Blastula
Ectoderm
Neural
Neural Crest

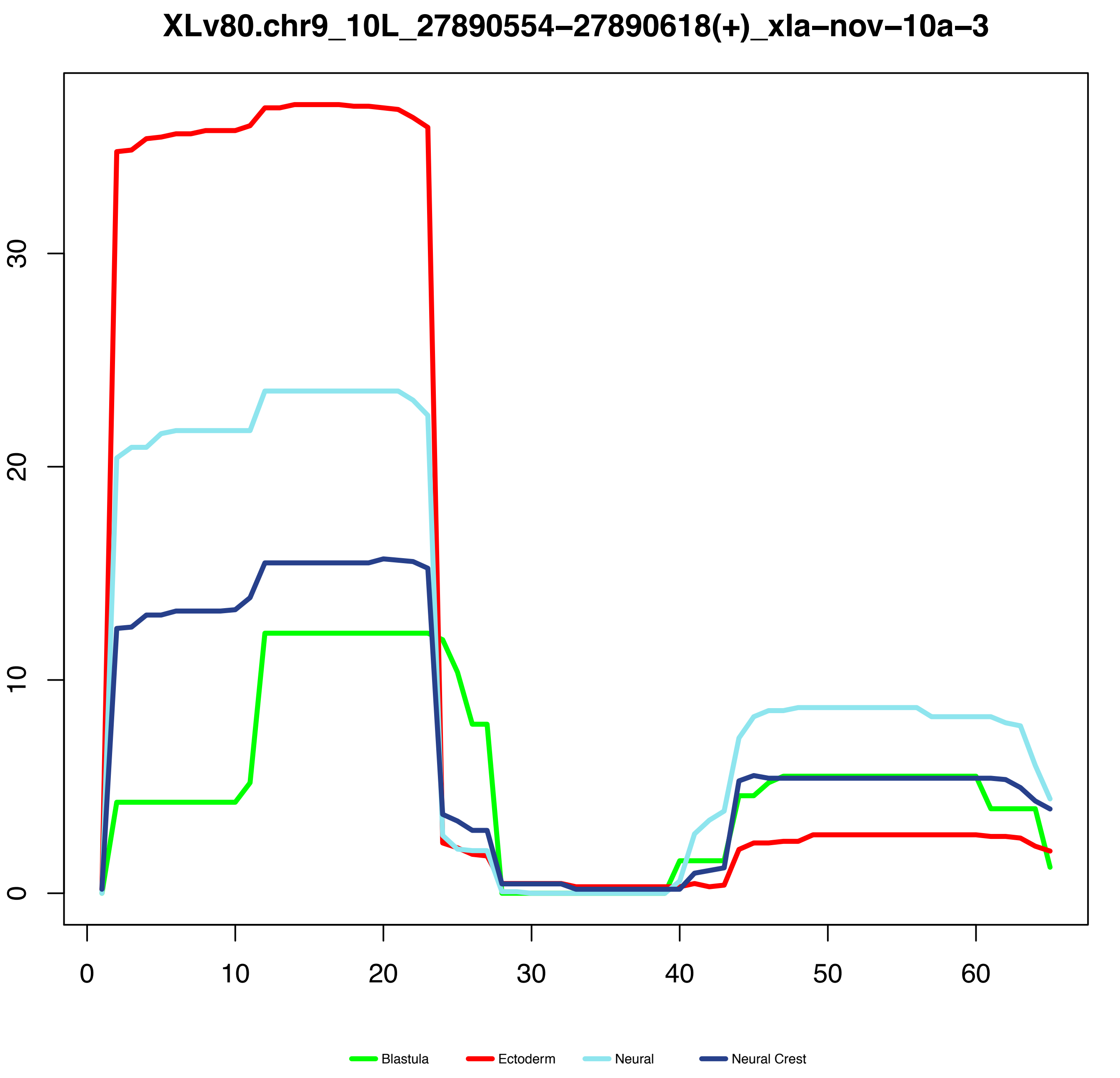
XLv80.chr9_10L_27890554−27890618(+)_xla−nov−10a−3
0
10
20
30
0
10
20
30
40
50
60
Blastula
Ectoderm
Neural
Neural Crest

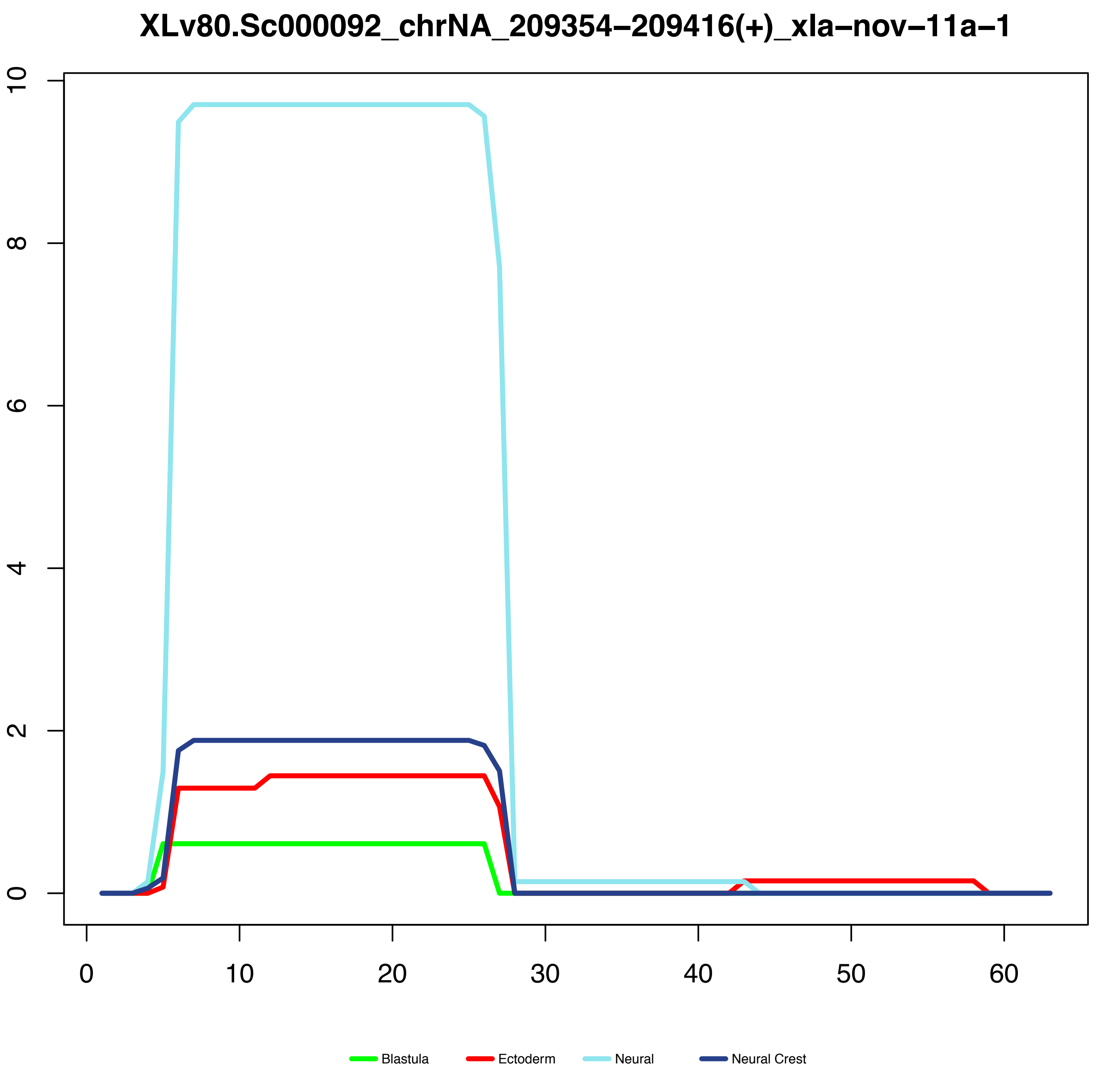
XLv80.Sc000092_chrNA_209354−209416(+)_xla−nov−11a−1
0
2
4
6
8
10
0
10
20
30
40
50
60
Blastula
Ectoderm
Neural
Neural Crest

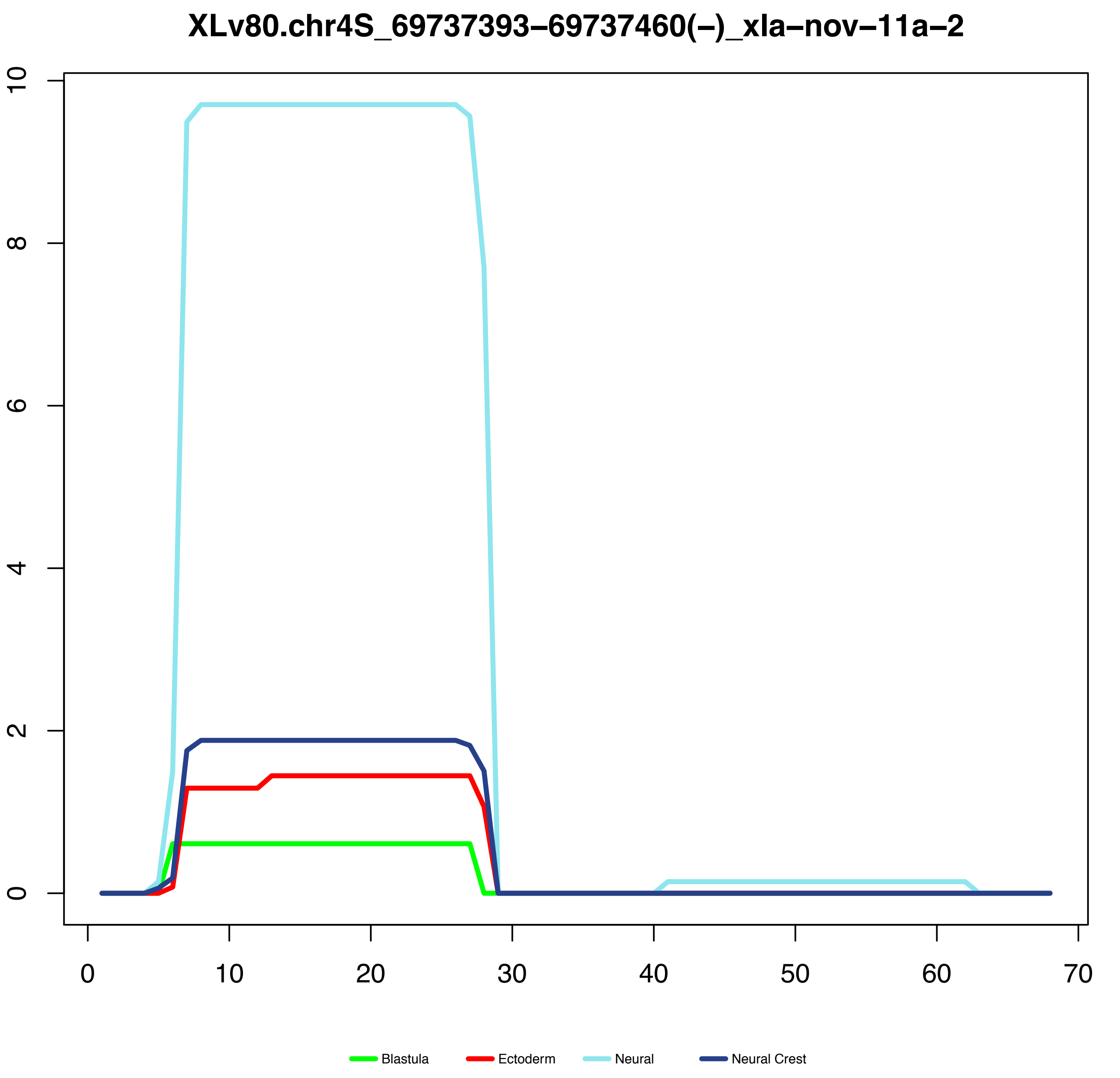
XLv80.chr4S_69737393–69737460(–)_xla–nov–11a–2
0
2
4
6
8
10
0
10
20
30
40
50
60
70
Blastula
Ectoderm
Neural
Neural Crest

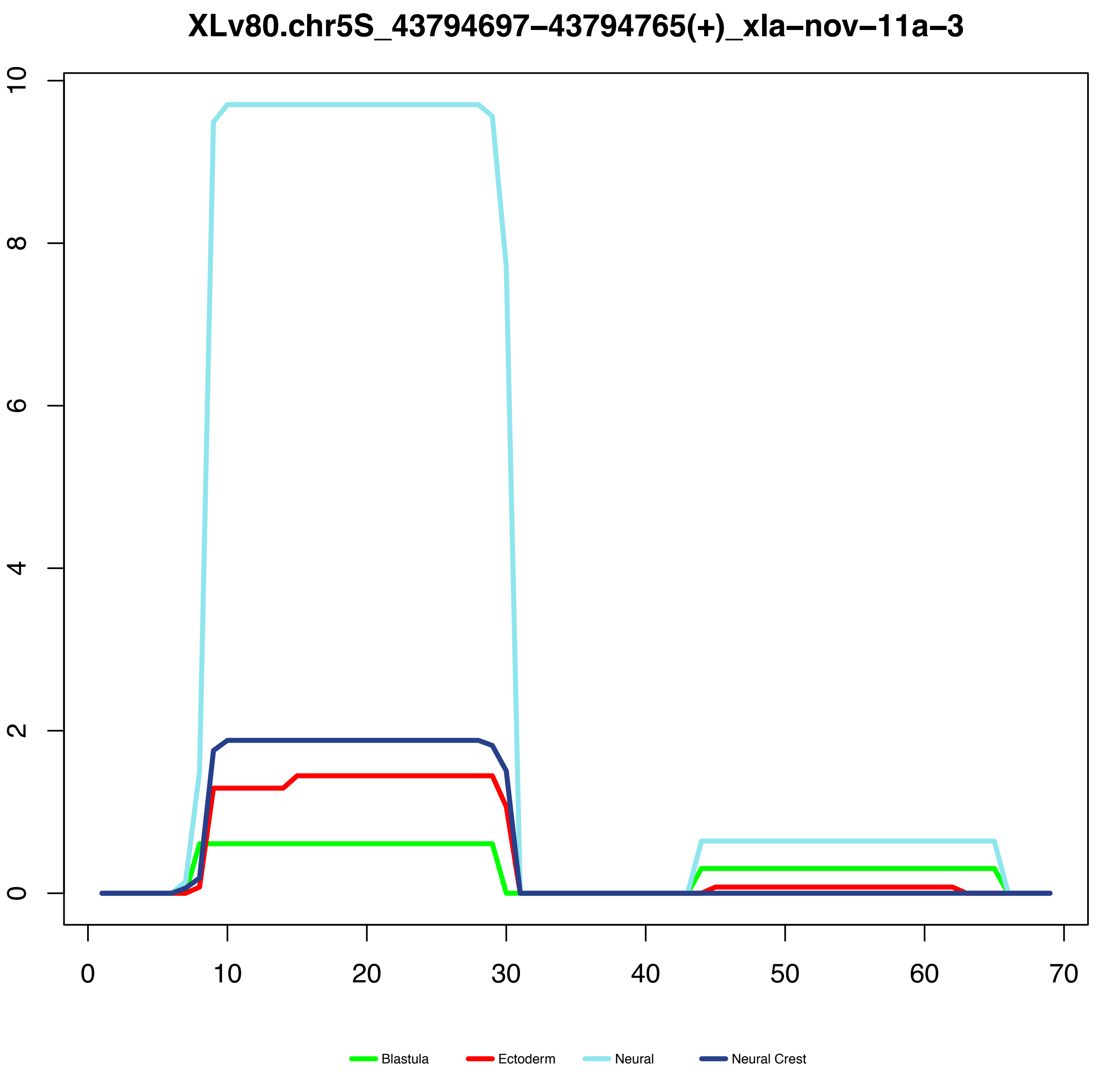
XLv80.chr5S_43794697−43794765(+)_xla−nov−11a−3
0
2
4
6
8
10
0
10
20
30
40
50
60
70
Blastula
Ectoderm
Neural
Neural Crest

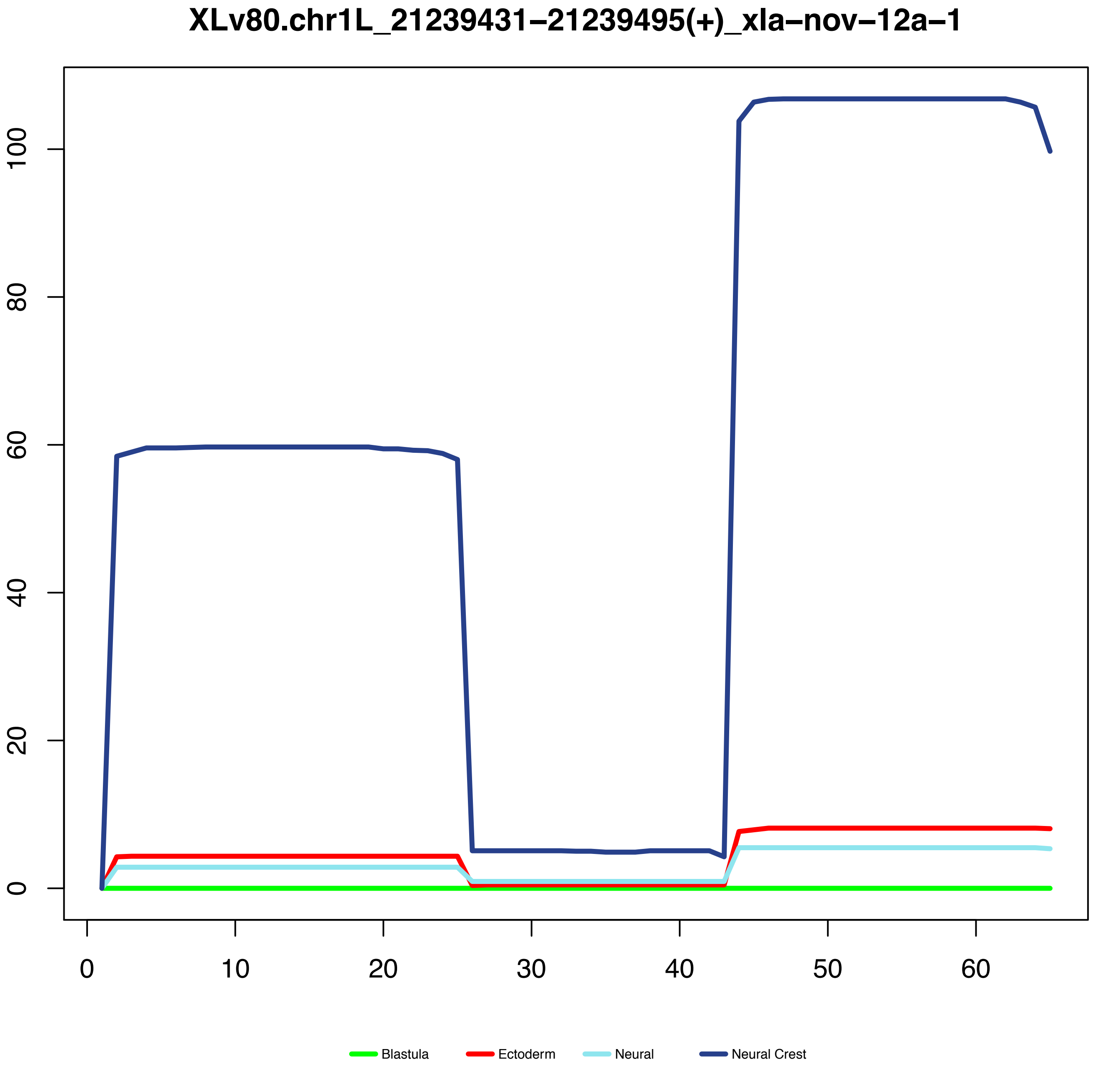
XLv80.chr1L_21239431−21239495(+)_xla−nov−12a−1
0
20
40
60
80
100
0
10
20
30
40
50
60
Blastula
Ectoderm
Neural
Neural Crest

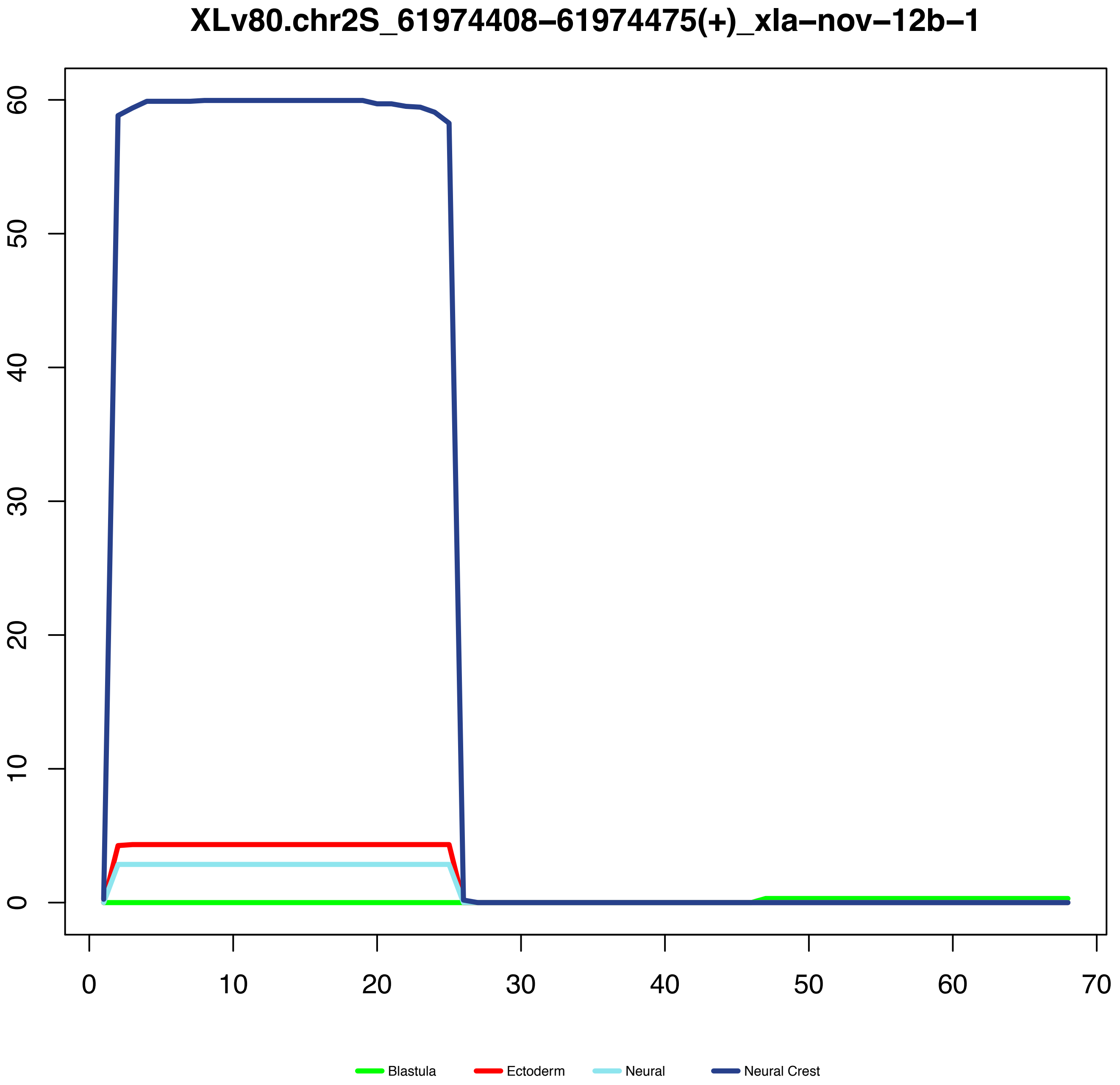

XLv80.chr2S_61974408−61974475(+)_xla−nov−12b−1
0
10
20
30
40
50
60
0
10
20
30
40
50
60
70
Blastula
Ectoderm
Neural
Neural Crest

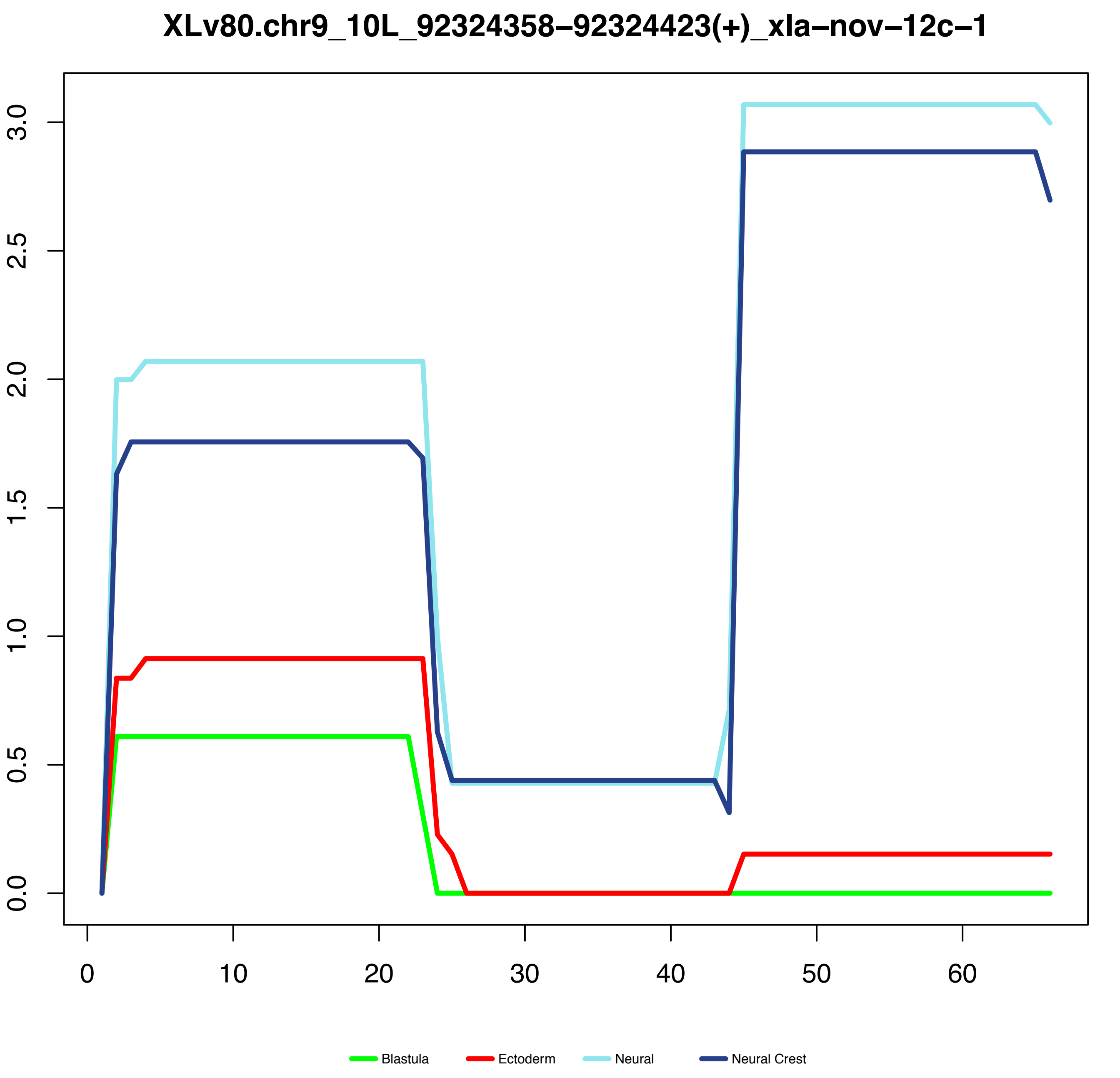
XLv80.chr9_10L_92324358−92324423(+)_xla−nov−12c−1
0.0
0.5
1.0
1.5
2.0
2.5
3.0
0
10
20
30
40
50
60
Blastula
Ectoderm
Neural
Neural Crest

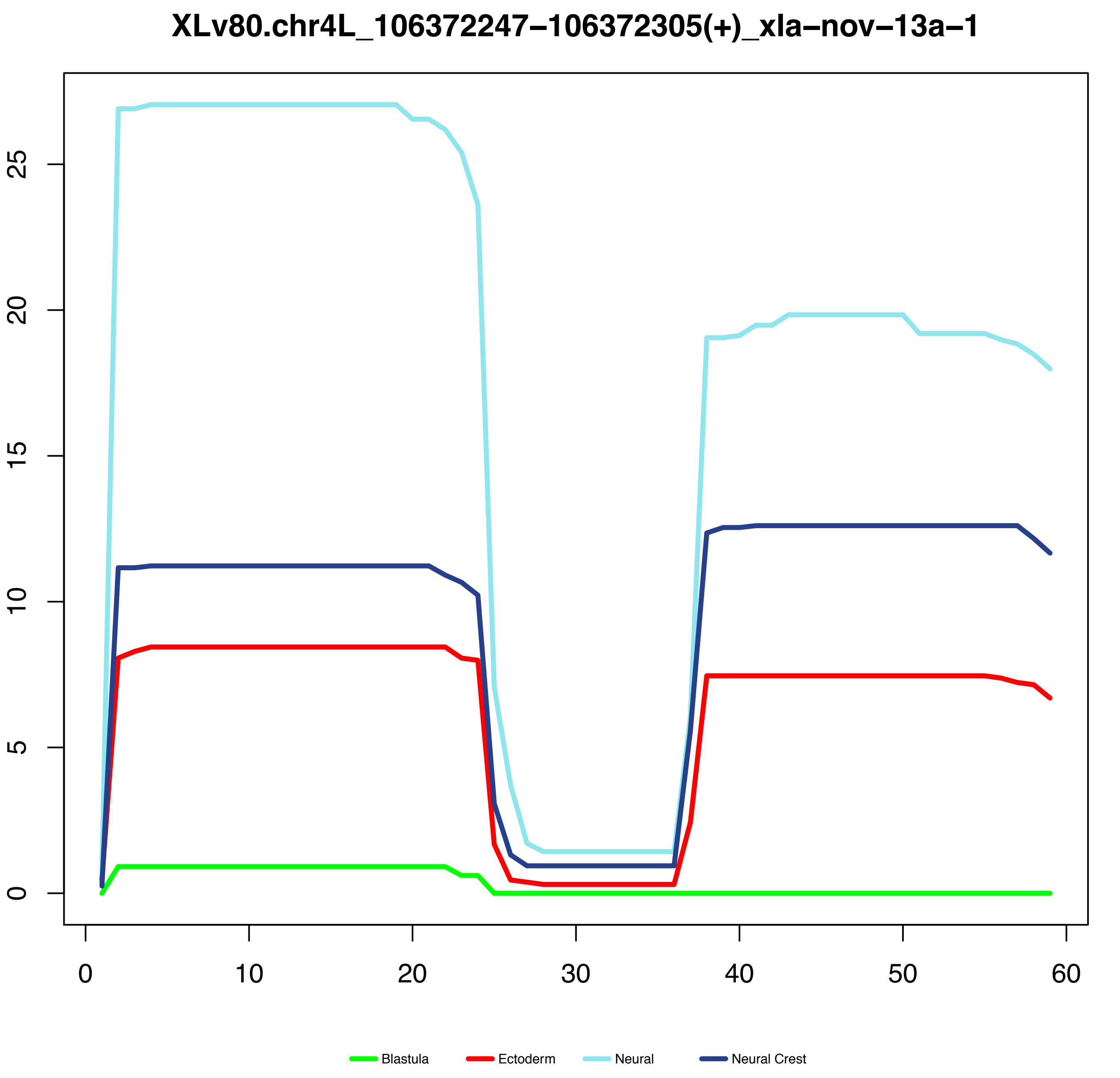

XLv80.chr4L_106372247−106372305(+)_xla−nov−13a−1
0
5
10
15
20
25
0
10
20
30
40
50
60
Blastula
Ectoderm
Neural
Neural Crest

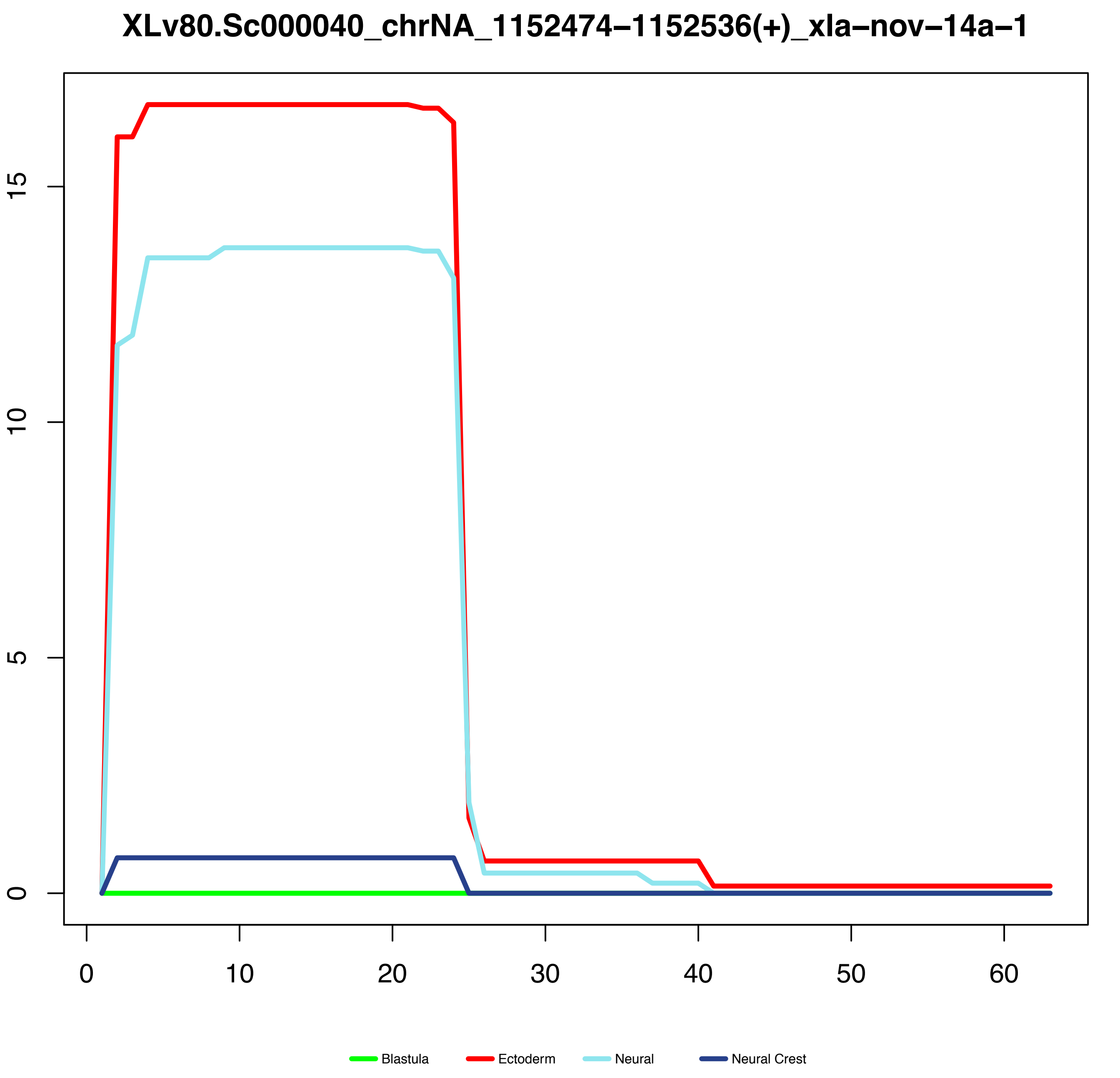
XLv80.Sc000040_chrNA_1152474−1152536(+)_xla−nov−14a−1
0
5
10
15
0
10
20
30
40
50
60
Blastula
Ectoderm
Neural
Neural Crest

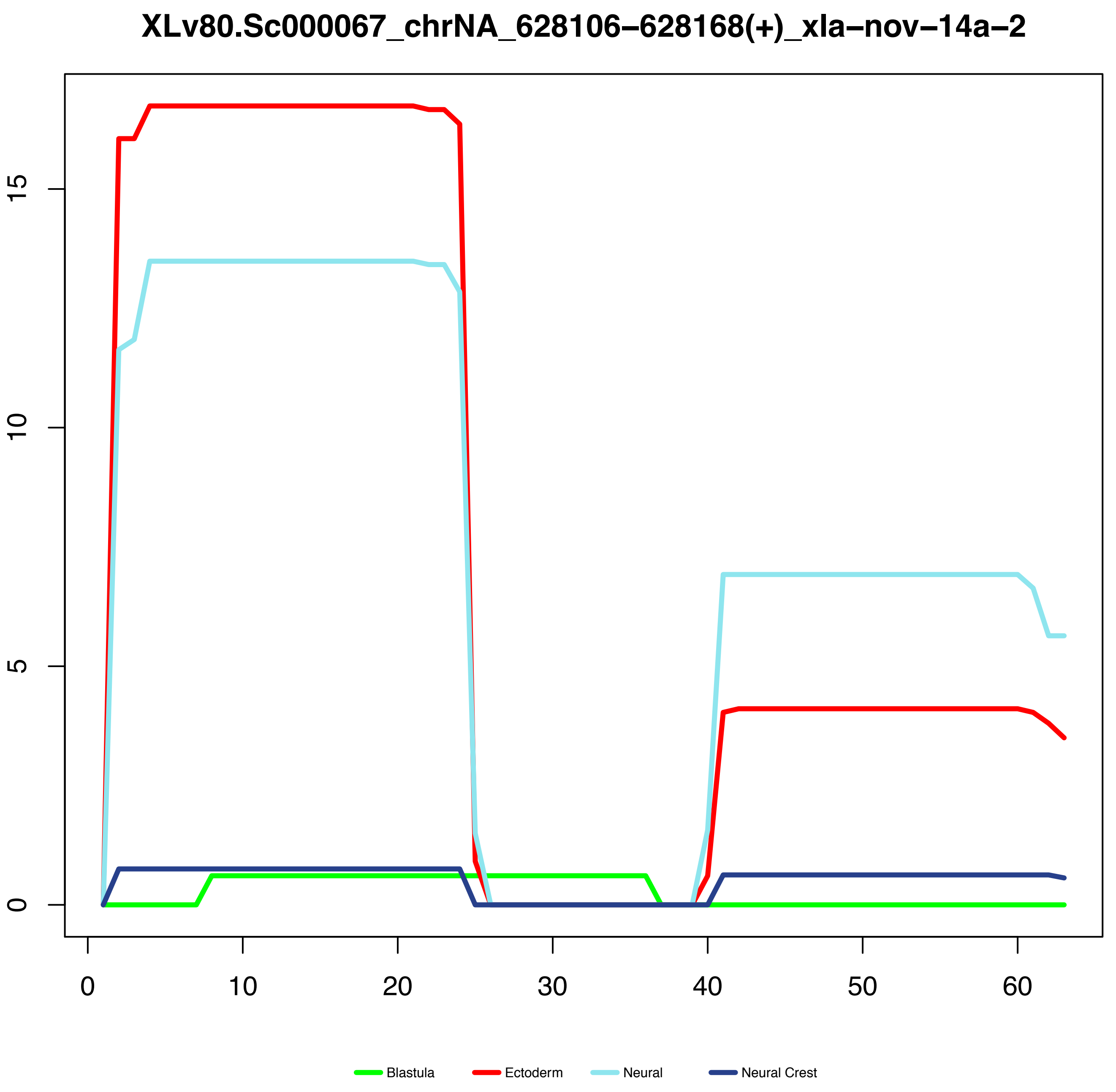

XLv80.Sc000067_chrNA_628106−628168(+)_xla−nov−14a−2
0
5
10
15
0
10
20
30
40
50
60
Blastula
Ectoderm
Neural
Neural Crest

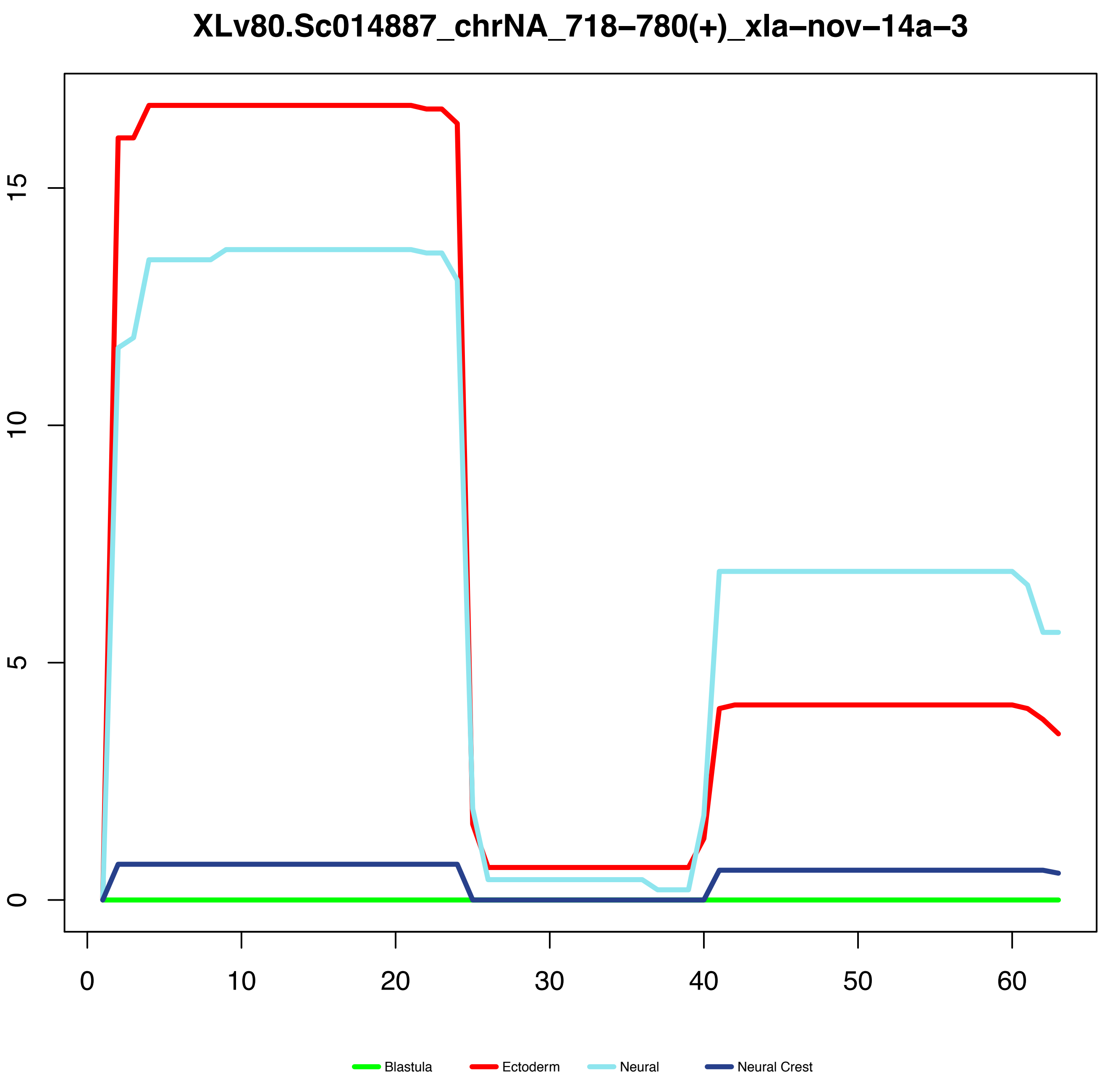
XLv80.Sc014887_chrNA_718−780(+)_xla−nov−14a−3
0
5
10
15
0
10
20
30
40
50
60
Blastula
Ectoderm
Neural
Neural Crest

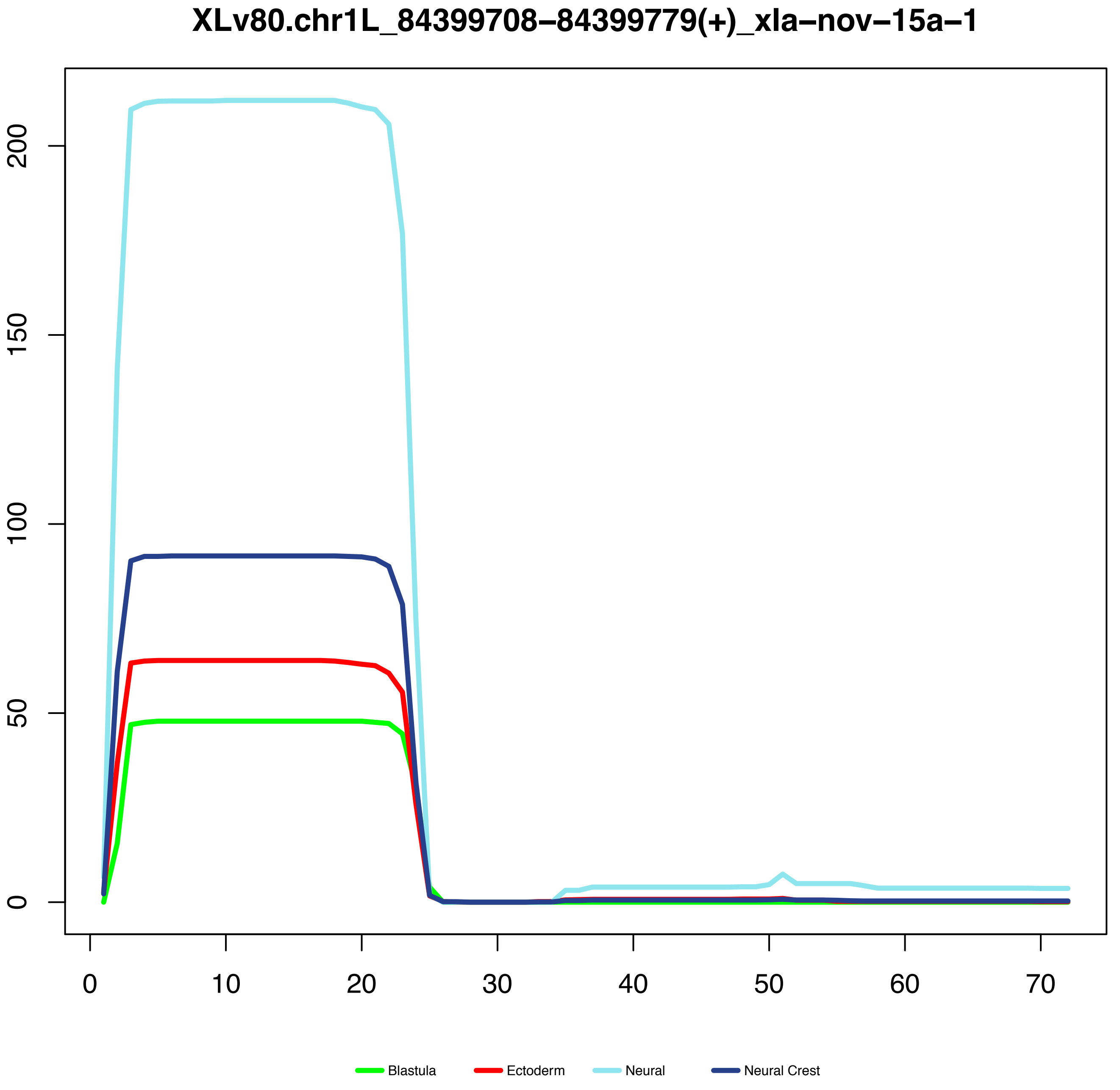
XLv80.chr1L_84399708−84399779(+)_xla−nov−15a−1
0
50
100
150
200
0
10
20
30
40
50
60
70
Blastula
Ectoderm
Neural
Neural Crest

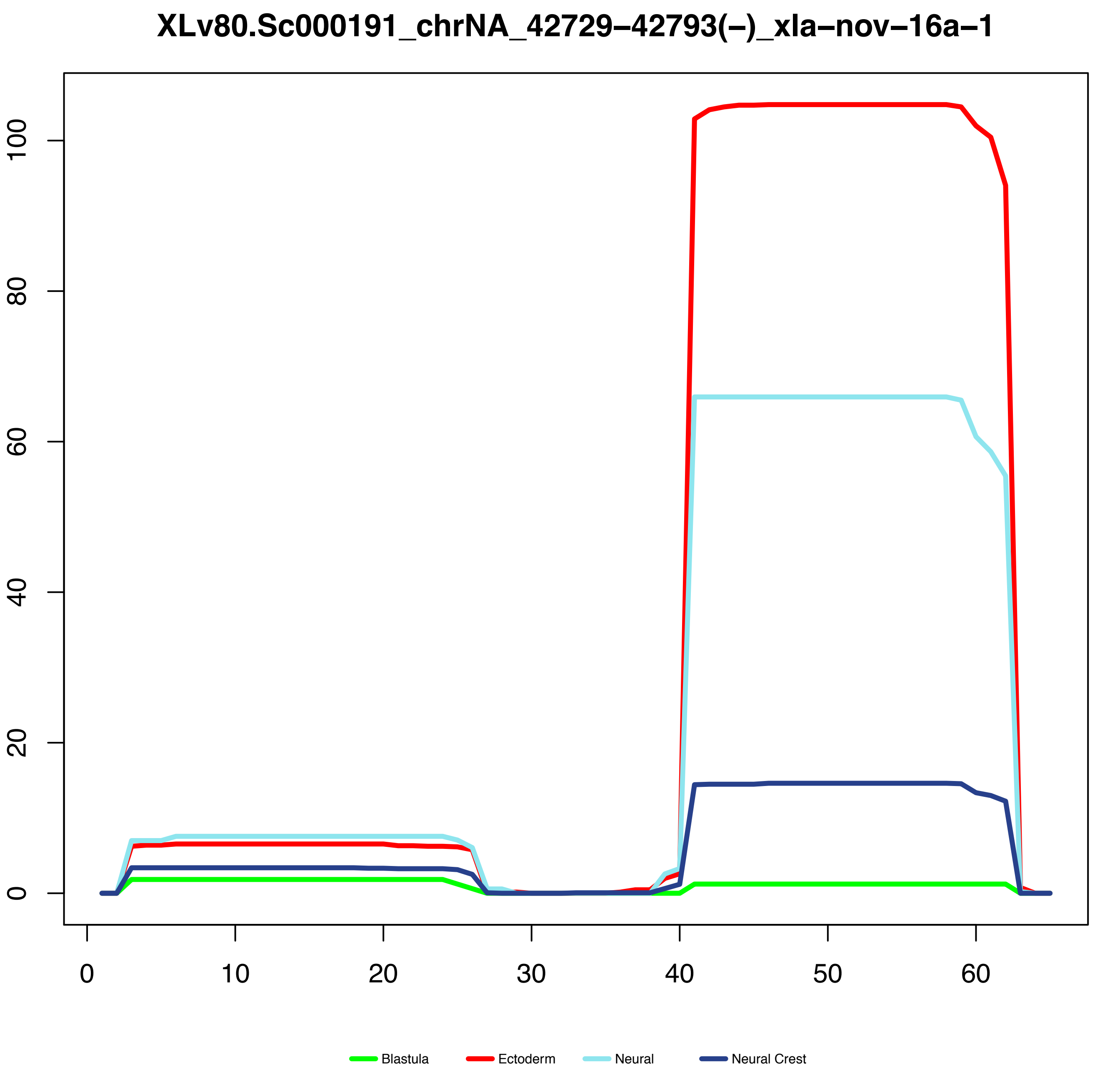

XLv80.Sc000191_chrNA_42729−42793(−)_xla−nov−16a−1
0
20
40
60
80
100
0
10
20
30
40
50
60
Blastula
Ectoderm
Neural
Neural Crest

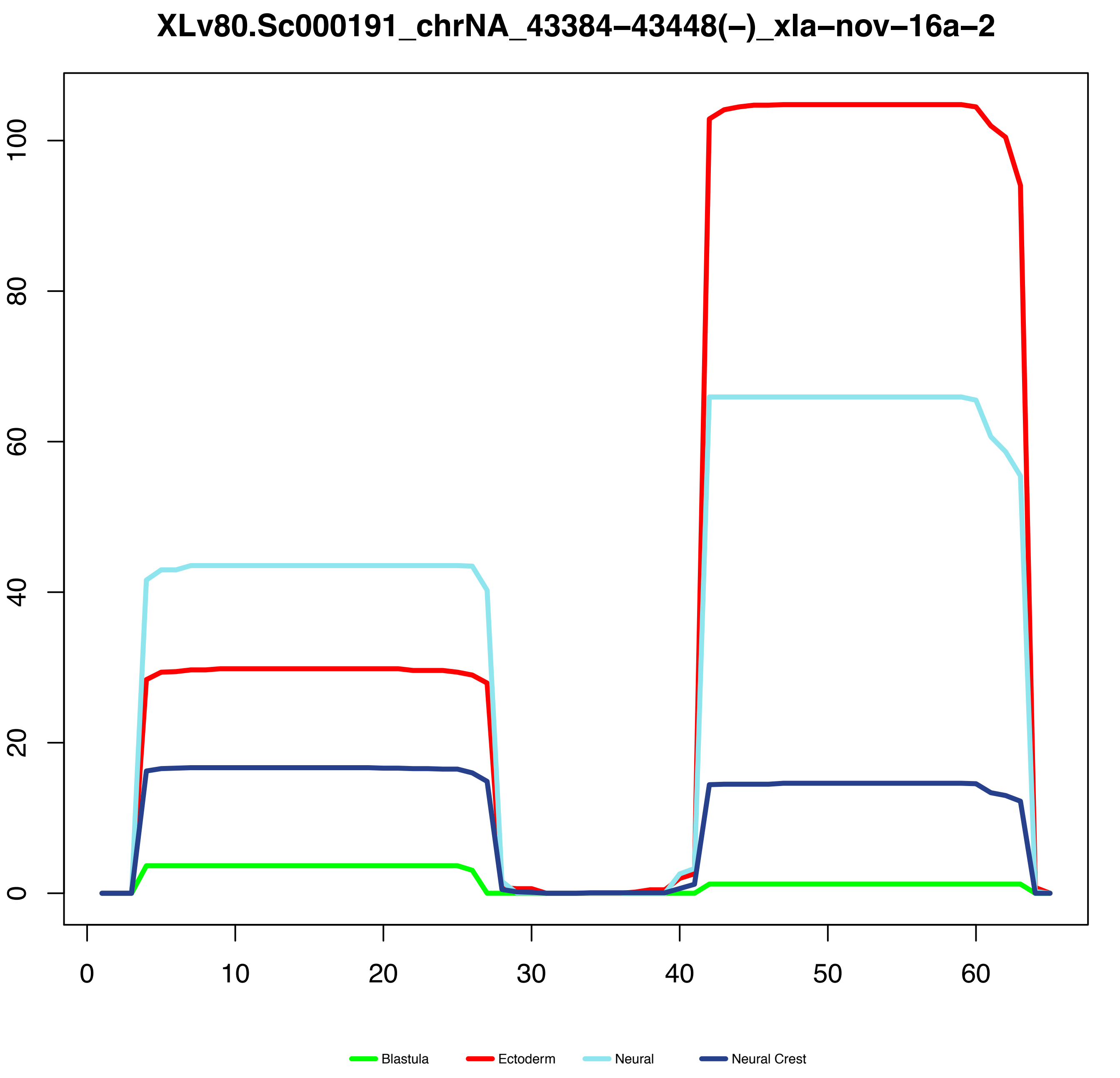
XLv80.Sc000191_chrNA_43384−43448(−)_xla−nov−16a−2
0
20
40
60
80
100
0
10
20
30
40
50
60
Blastula
Ectoderm
Neural
Neural Crest

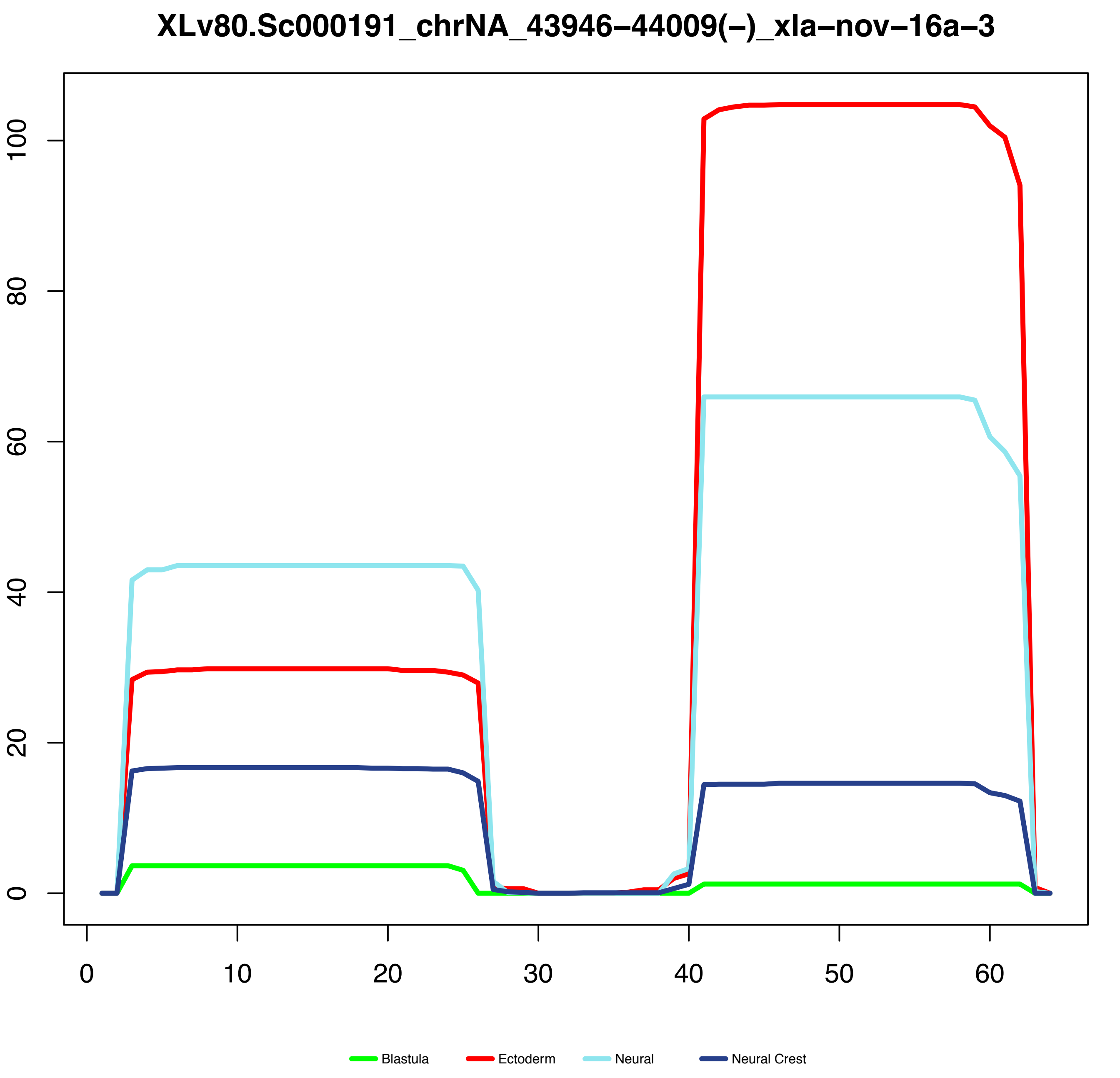

XLv80.Sc000191_chrNA_43946−44009(−)_xla−nov−16a−3
0
20
40
60
80
100
0
10
20
30
40
50
60
Blastula
Ectoderm
Neural
Neural Crest

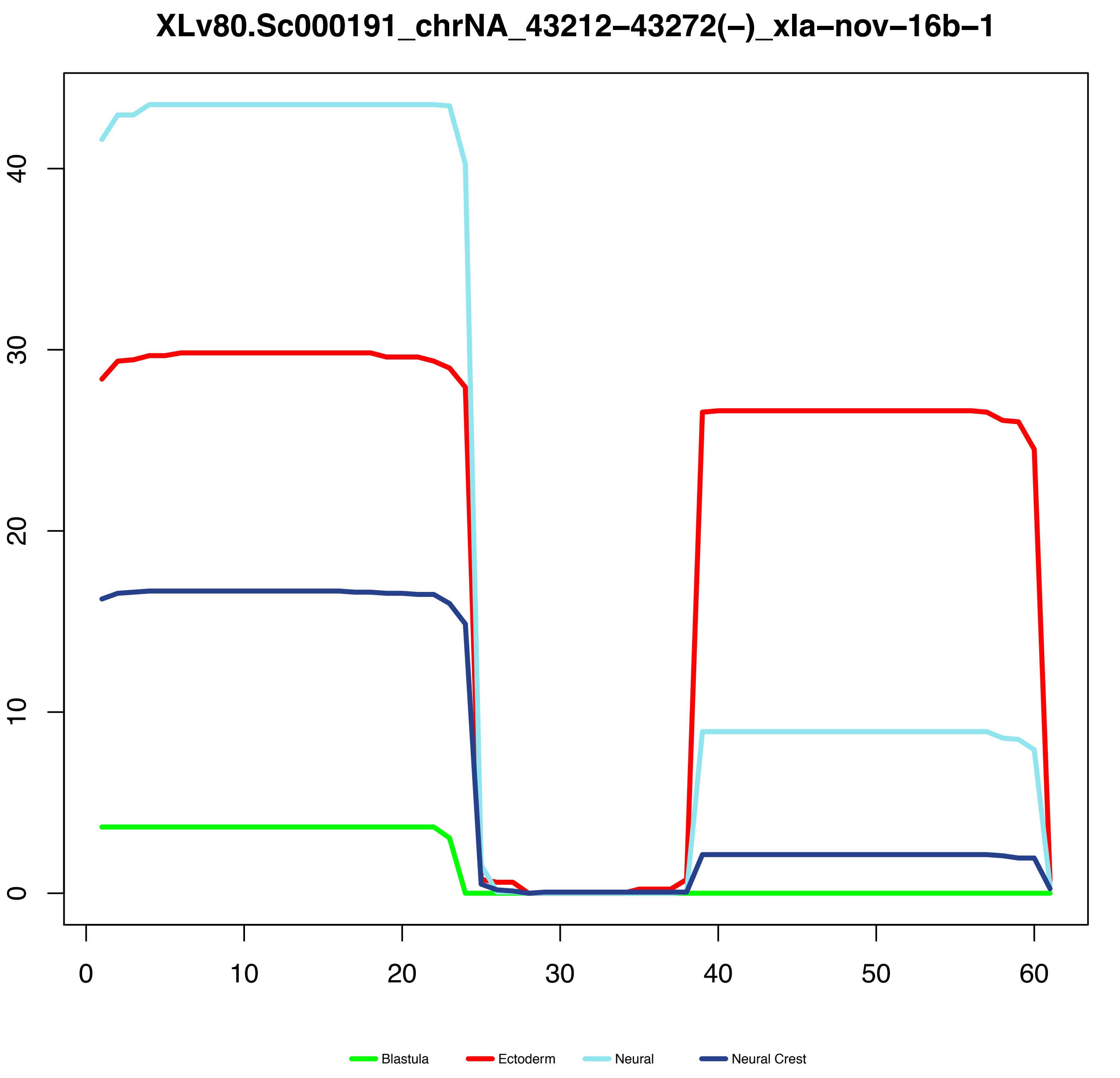

XLv80.Sc000191_chrNA_43212−43272(−)_xla−nov−16b−1
0
10
20
30
40
50
60
Blastula
Ectoderm
Neural
Neural Crest

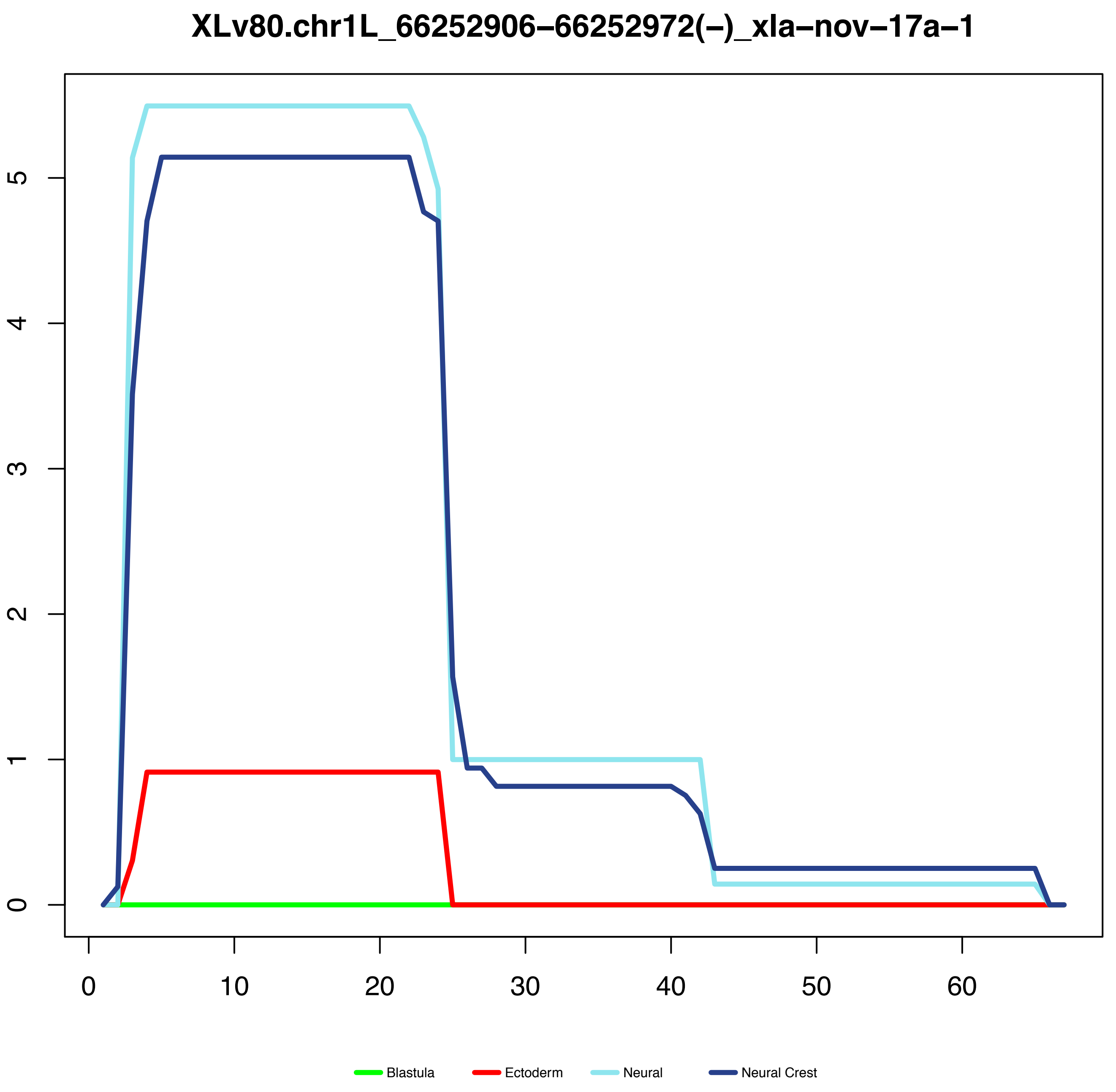
XLv80.chr1L_66252906−66252972(−)_xla−nov−17a−1
0
1
2
3
4
5
0
10
20
30
40
50
60
Blastula
Ectoderm
Neural
Neural Crest

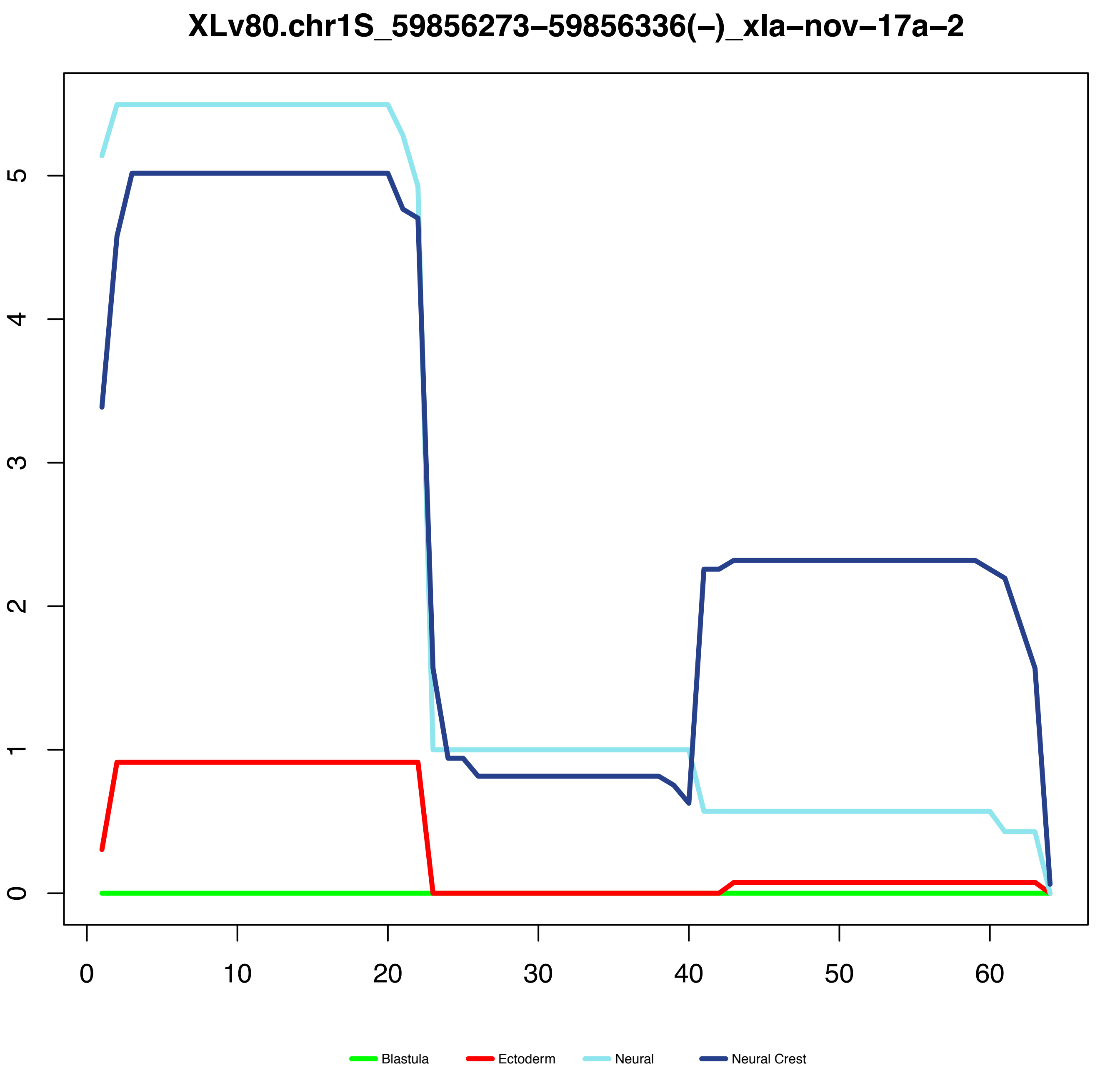
XLv80.chr1S_59856273−59856336(−)_xla−nov−17a−2
0
1
2
3
4
5
0
10
20
30
40
50
60
Blastula
Ectoderm
Neural
Neural Crest

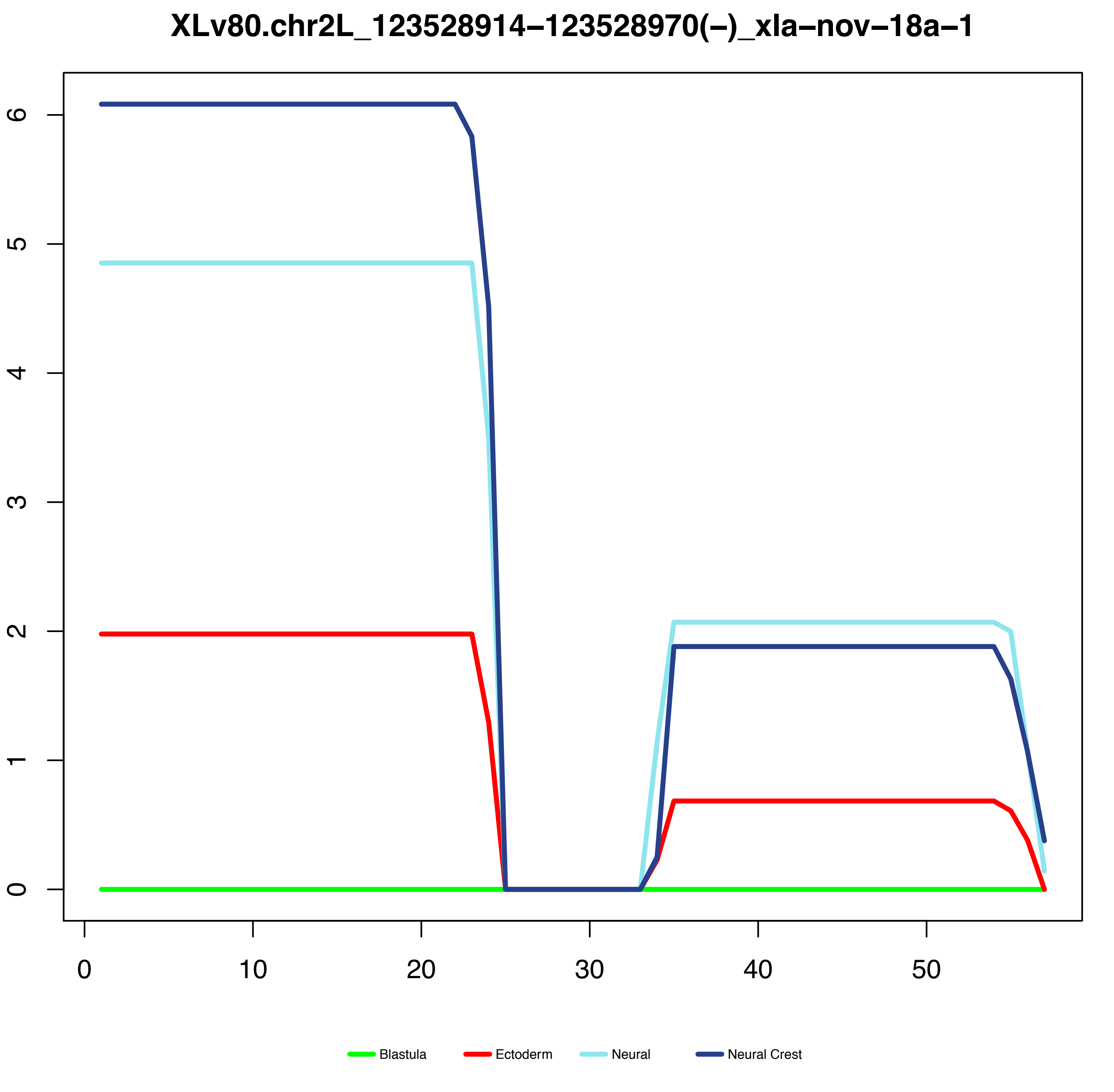
XLv80.chr2L_123528914−123528970(−)_xla−nov−18a−1
0
1
2
3
4
5
6
0
10
20
30
40
50
Blastula
Ectoderm
Neural
Neural Crest

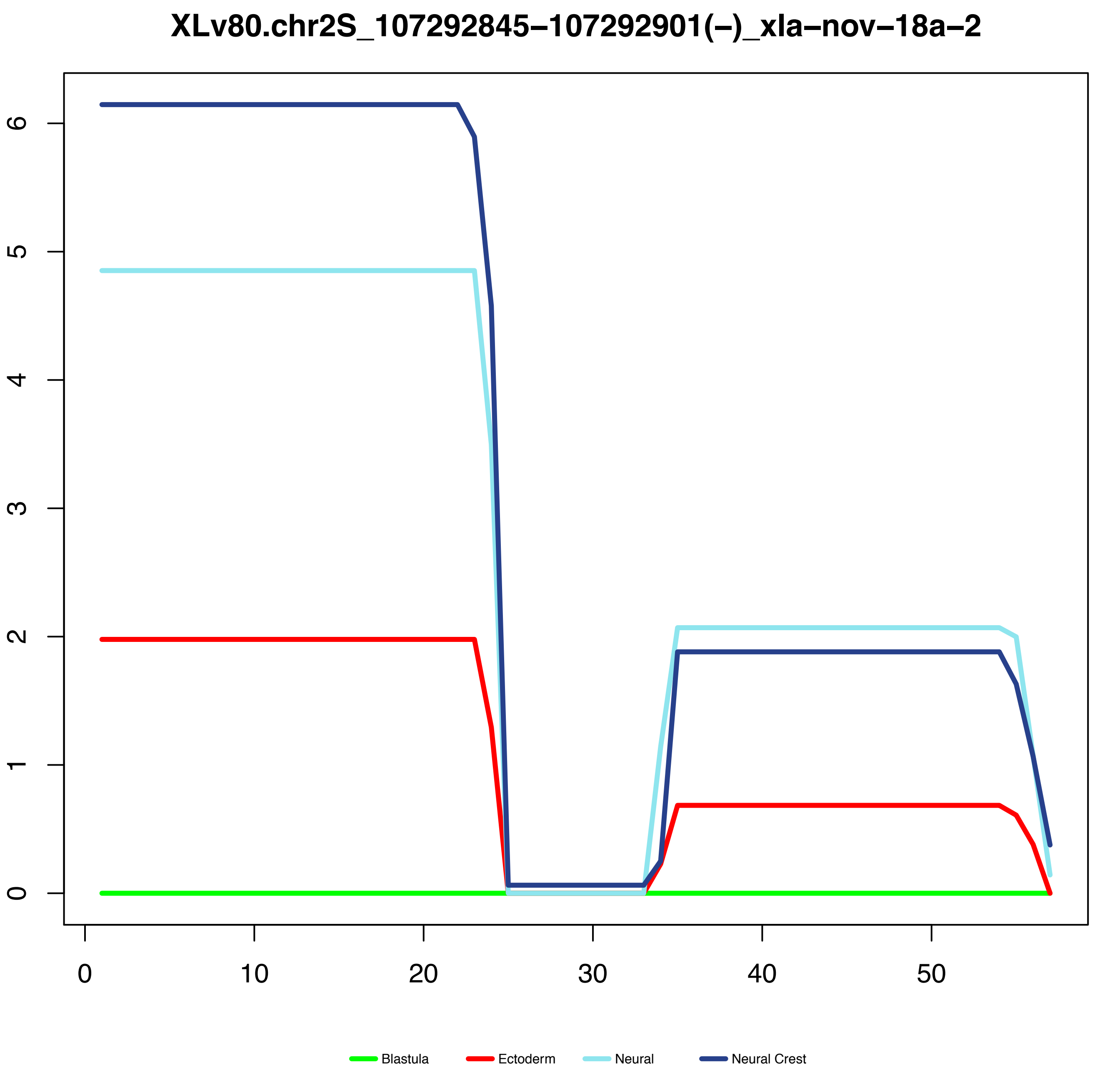

XLv80.chr2S_107292845–107292901(–)_xla–nov–18a–2
0
1
2
3
4
5
6
0
10
20
30
40
50
Blastula
Ectoderm
Neural
Neural Crest

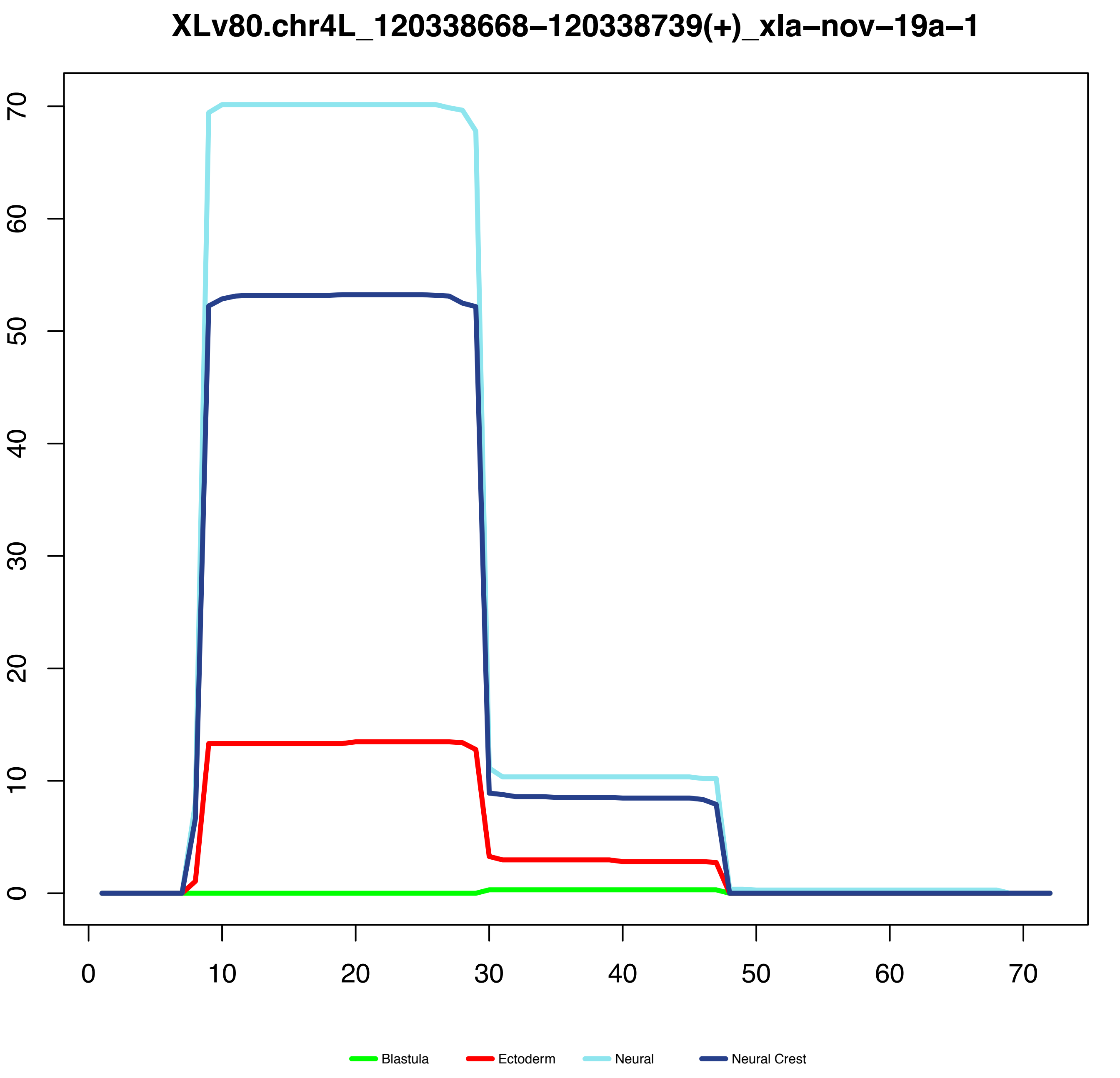

XLv80.chr4L_120338668−120338739(+)_xla−nov−19a−1
0
10
20
30
40
50
60
70
Blastula
Ectoderm
Neural
Neural Crest

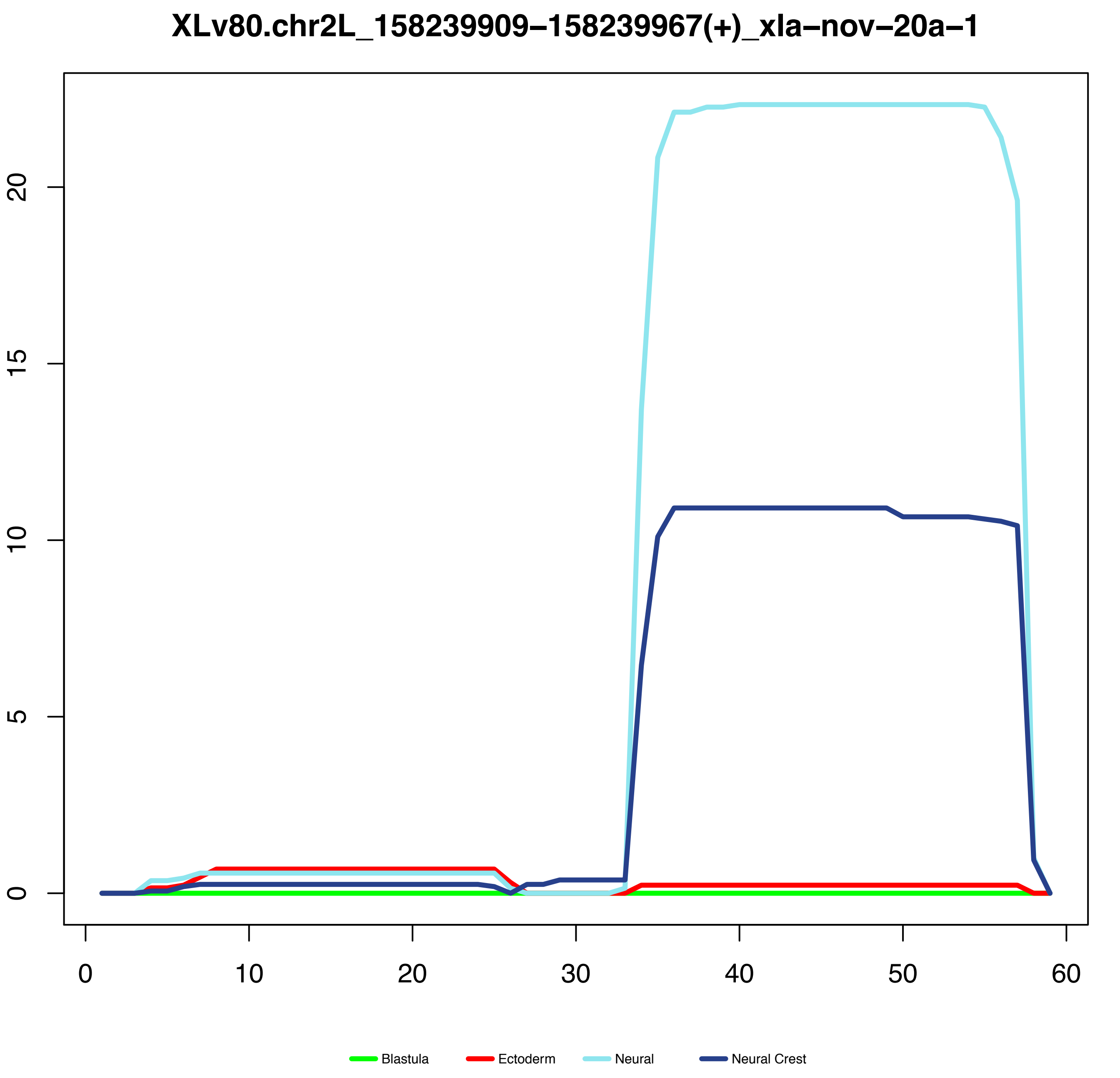
XLv80.chr2L_158239909−158239967(+)_xla−nov−20a−1
0
5
10
15
20
0
10
20
30
40
50
60
Blastula
Ectoderm
Neural
Neural Crest

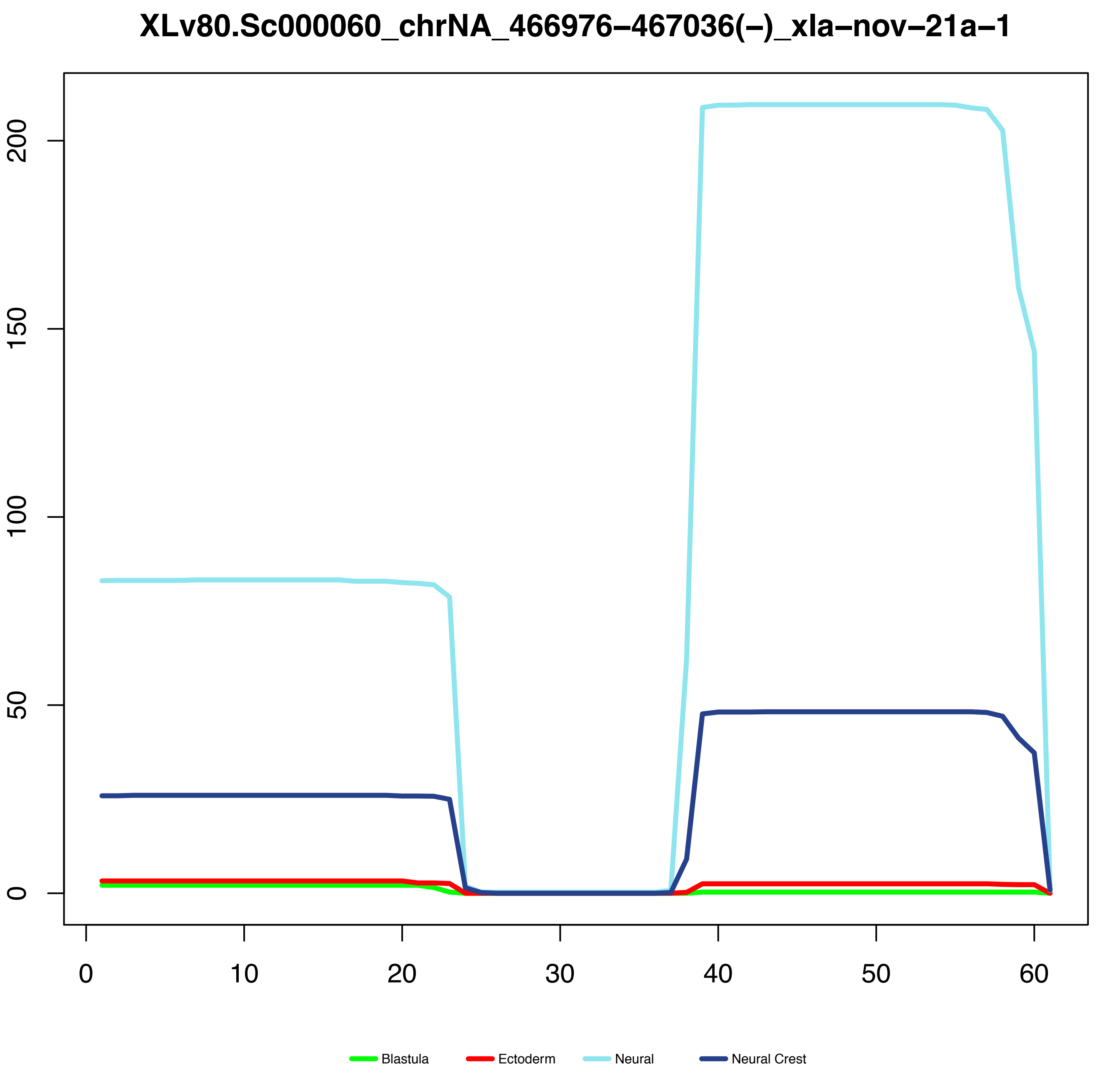
XLv80.Sc000060_chrNA_466976–467036(–)_xla–nov–21a–1
0
50
100
150
200
0
10
20
30
40
50
60
Blastula
Ectoderm
Neural
Neural Crest

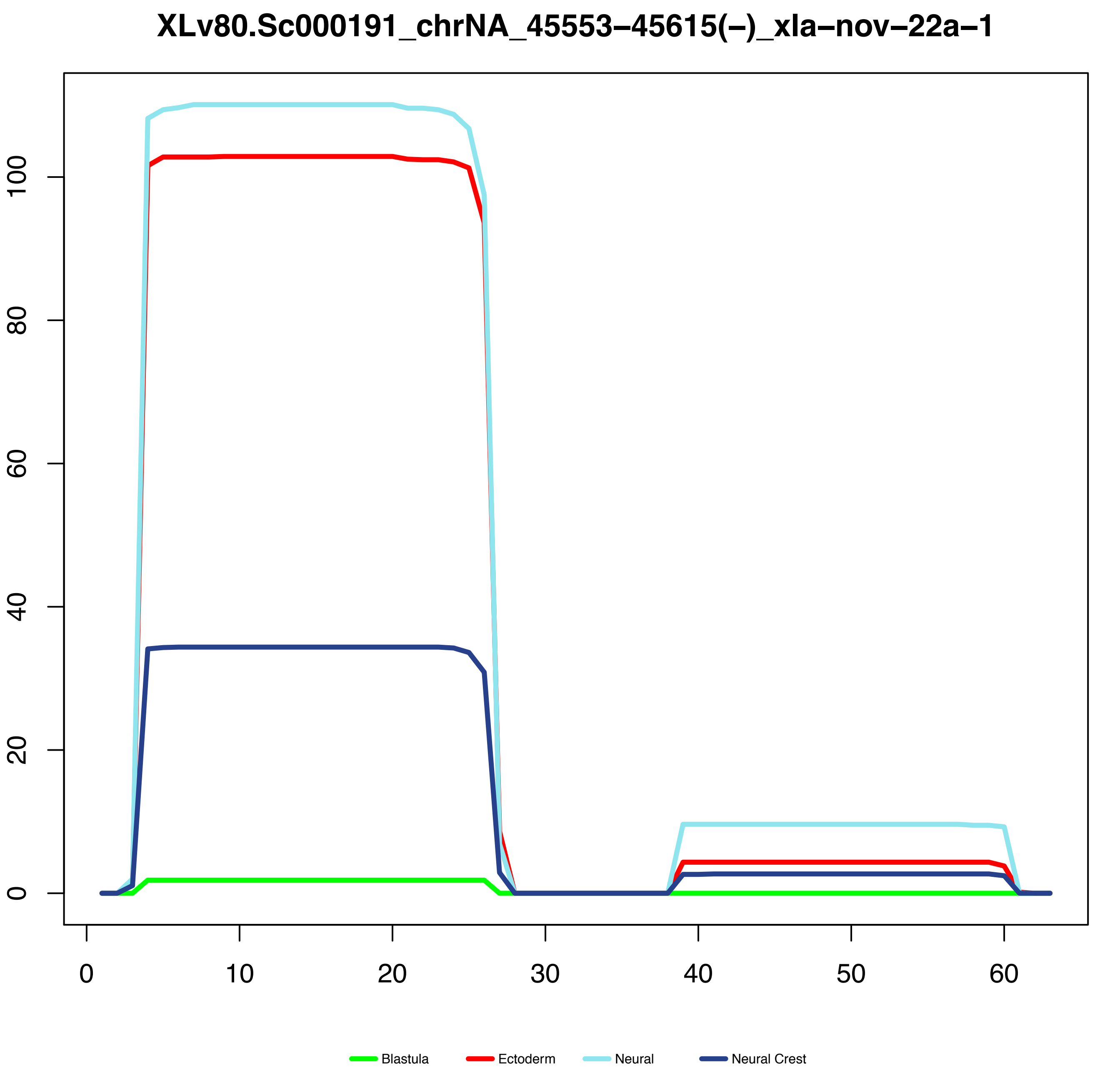

XLv80.Sc000191_chrNA_45553−45615(−)_xla−nov−22a−1
0
20
40
60
80
100
0
10
20
30
40
50
60
Blastula
Ectoderm
Neural
Neural Crest

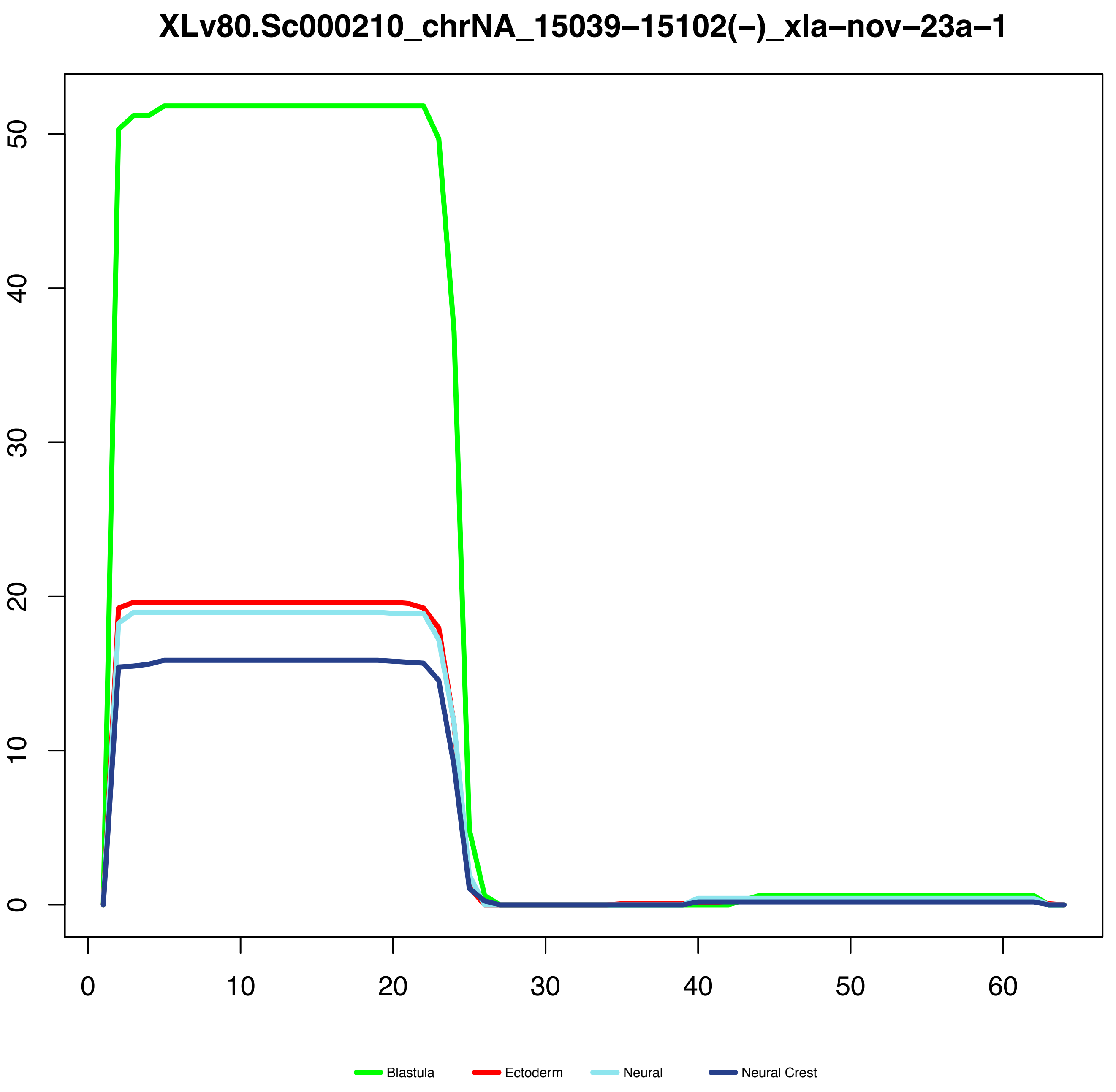
XLv80.Sc000210_chrNA_15039−15102(−)_xla−nov−23a−1
0
10
20
30
40
50
60
Blastula
Ectoderm
Neural
Neural Crest

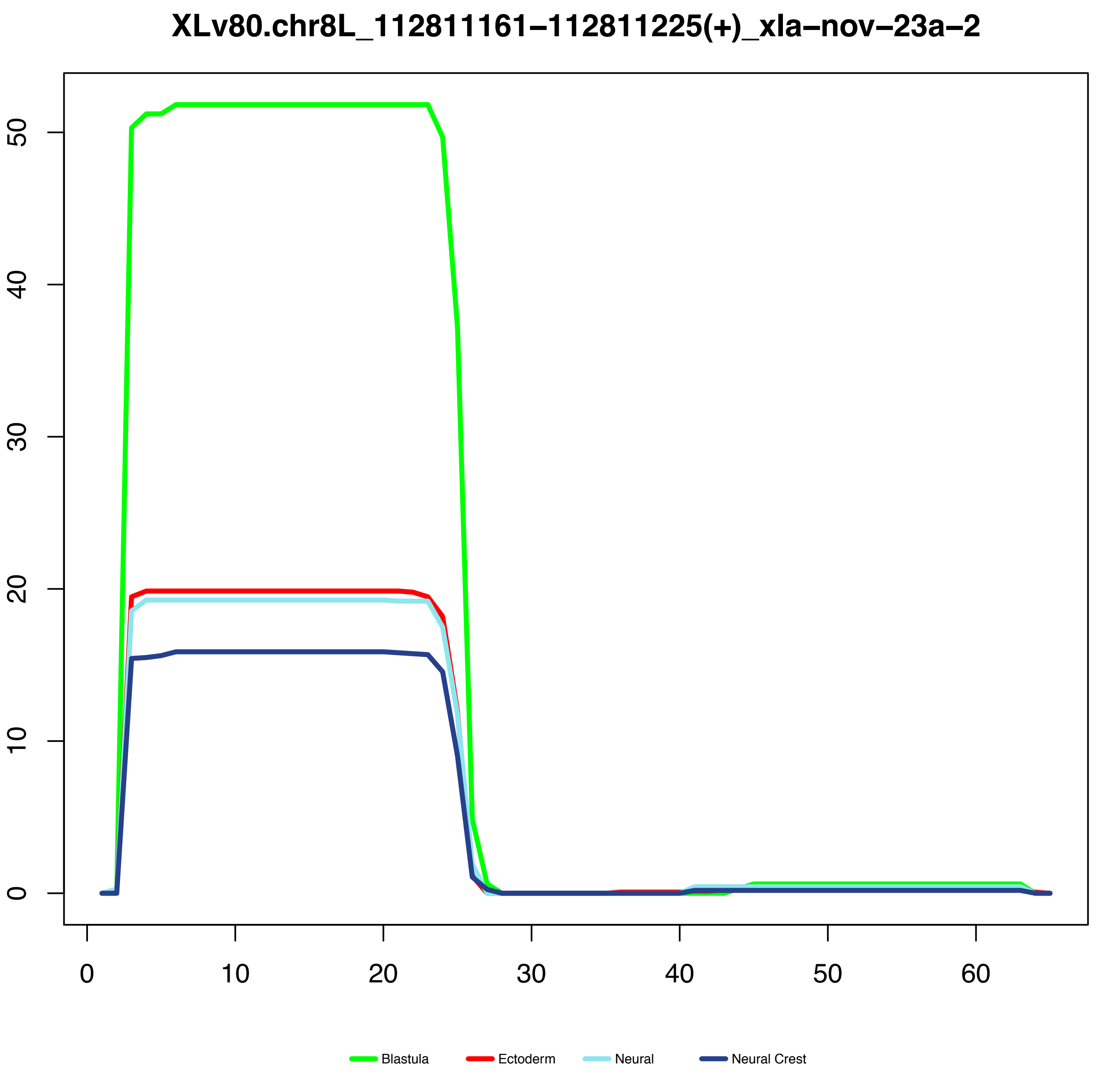
XLv80.chr8L_112811161−112811225(+)_xla−nov−23a−2
0
10
20
30
40
50
0
10
20
30
40
50
60
Blastula
Ectoderm
Neural
Neural Crest

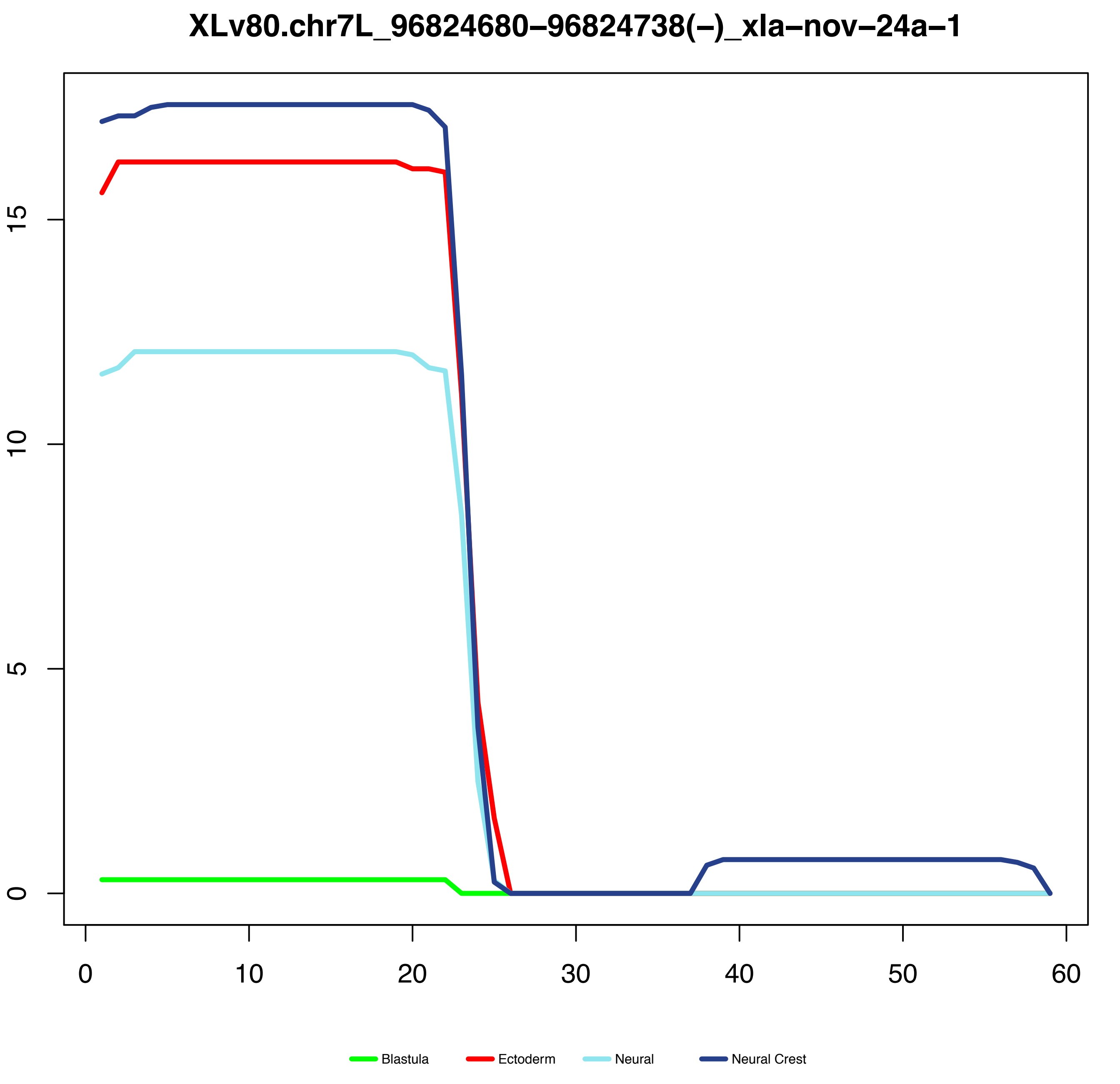
XLv80.chr7L_96824680−96824738(−)_xla−nov−24a−1
0
5
10
15
0
10
20
30
40
50
60
Blastula
Ectoderm
Neural
Neural Crest

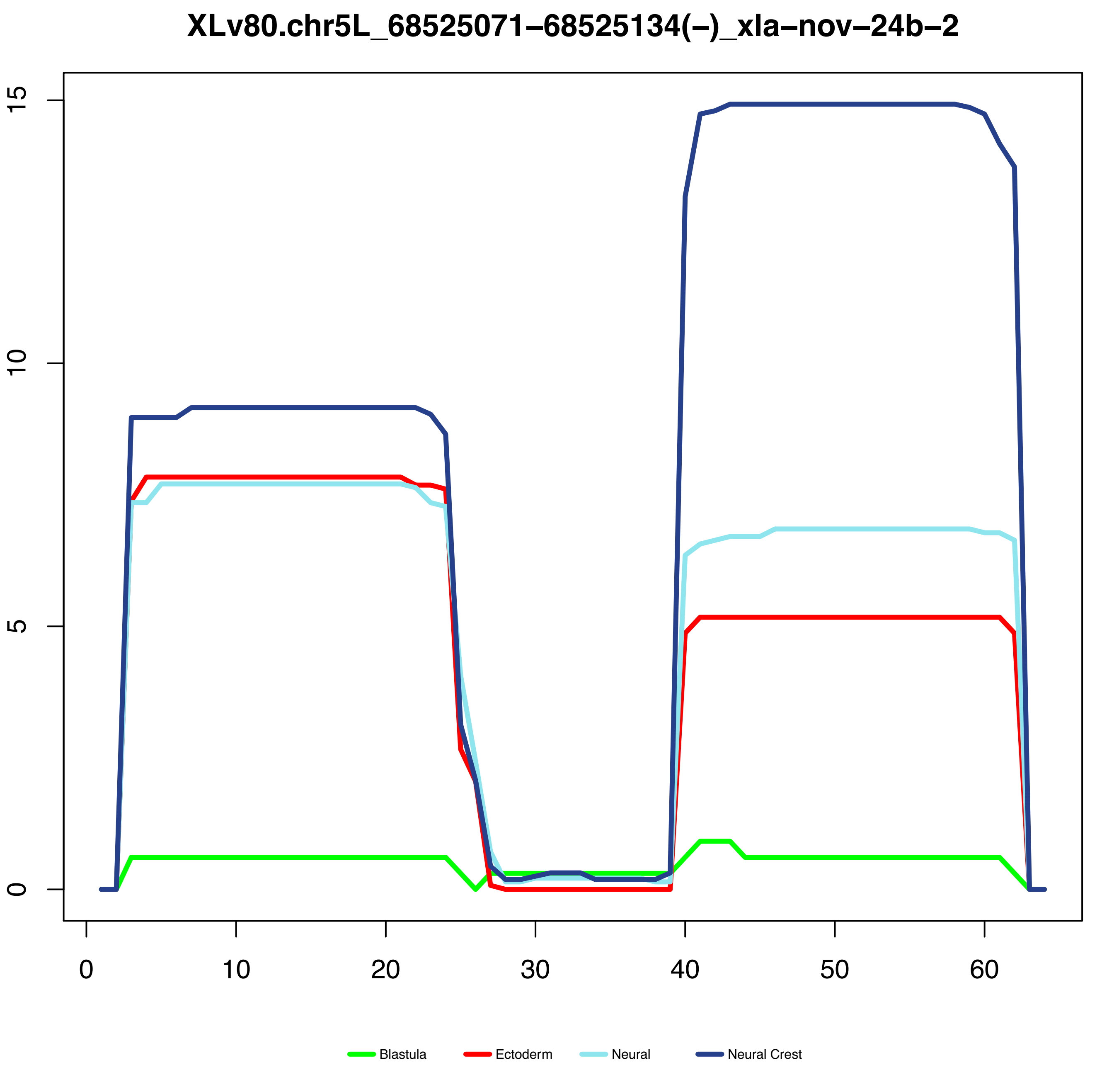
XLv80.chr5L_68525071−68525134(−)_xla−nov−24b−2
0
5
10
15
0
10
20
30
40
50
60
Blastula
Ectoderm
Neural
Neural Crest

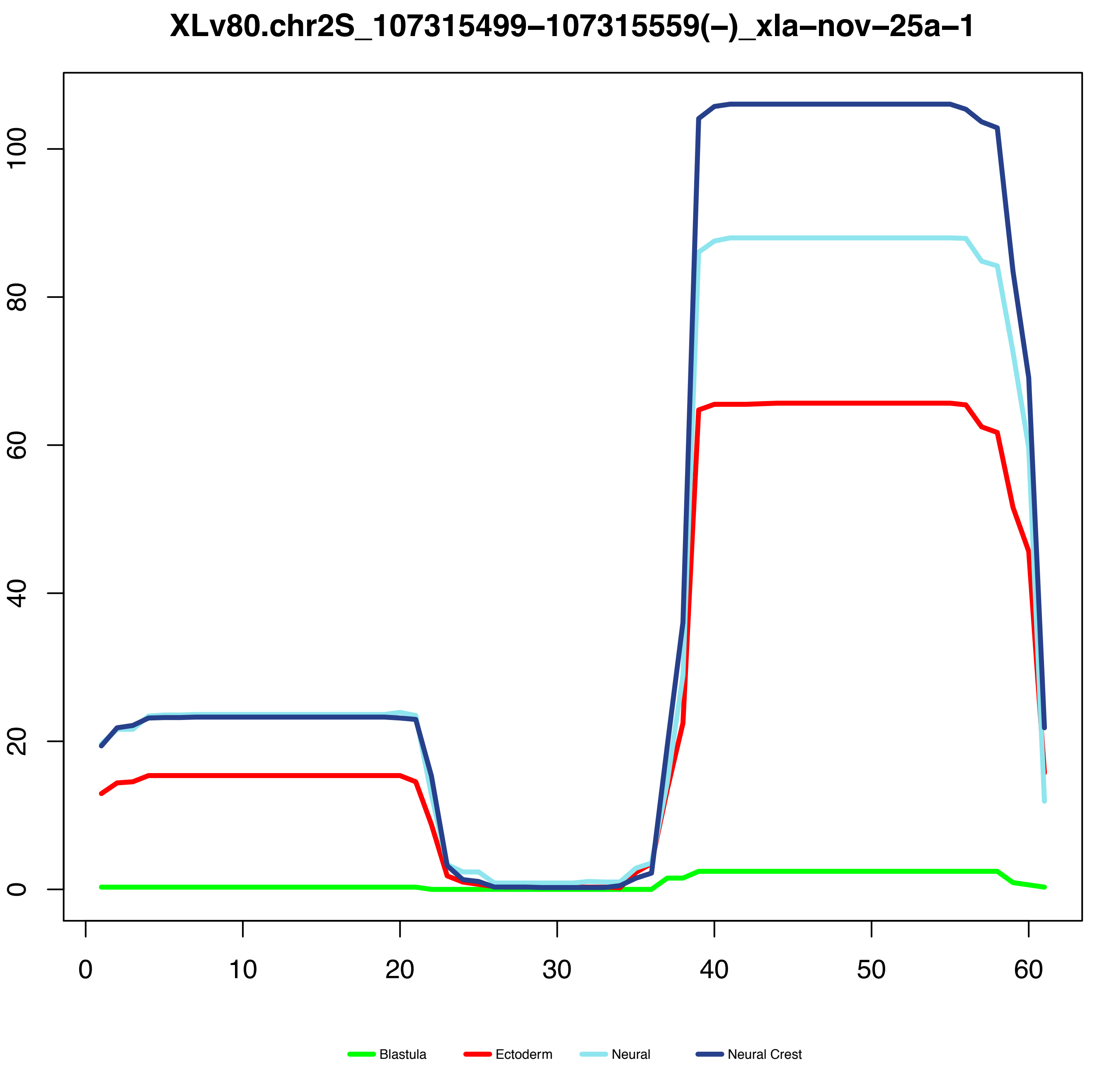

XLv80.chr2S_107315499−107315559(−)_xla−nov−25a−1
0
20
40
60
80
100
0
10
20
30
40
50
60
Blastula
Ectoderm
Neural
Neural Crest

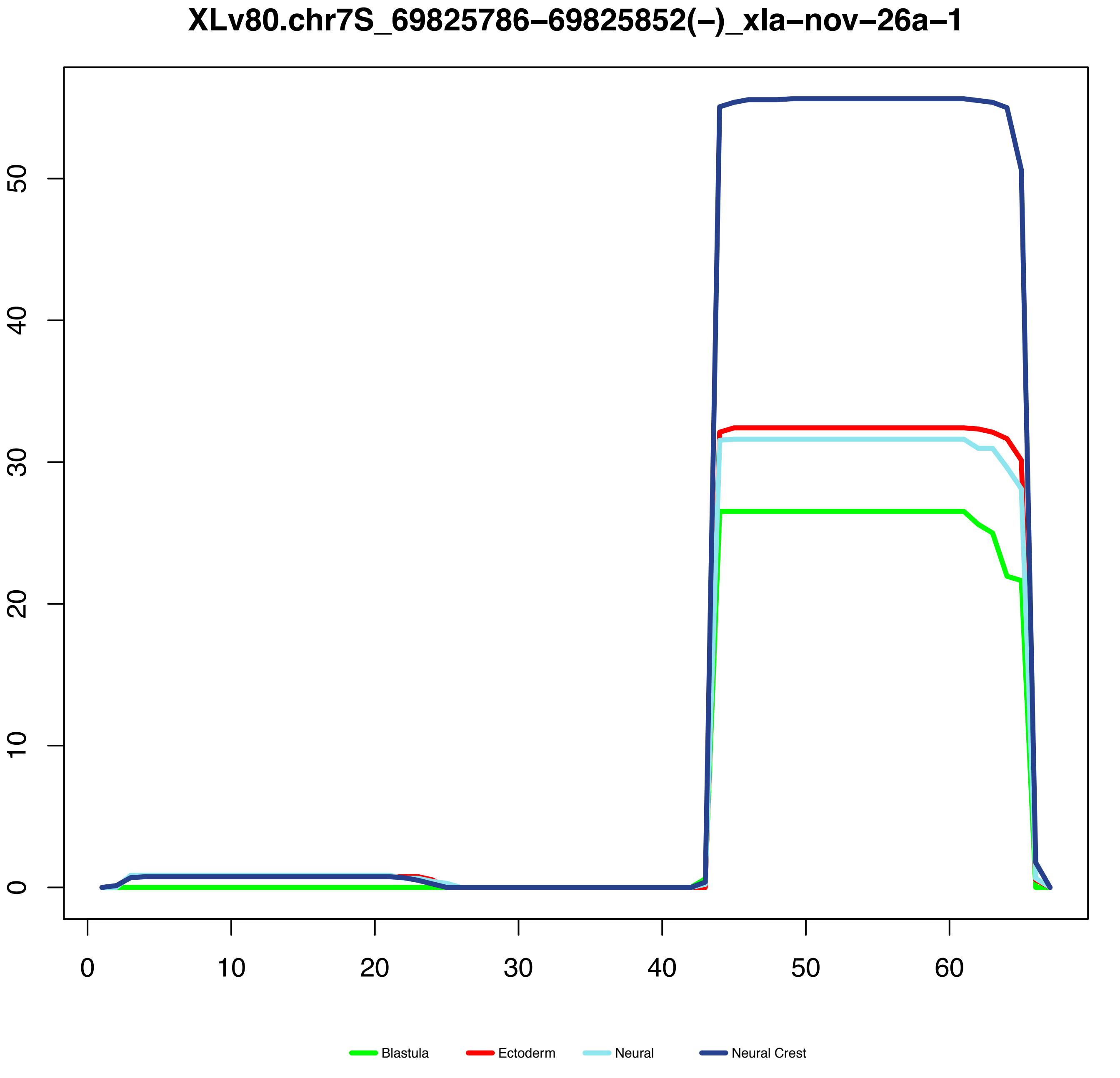
XLv80.chr7S_69825786−69825852(−)_xla−nov−26a−1
0
10
20
30
40
50
0
10
20
30
40
50
60
Blastula
Ectoderm
Neural
Neural Crest

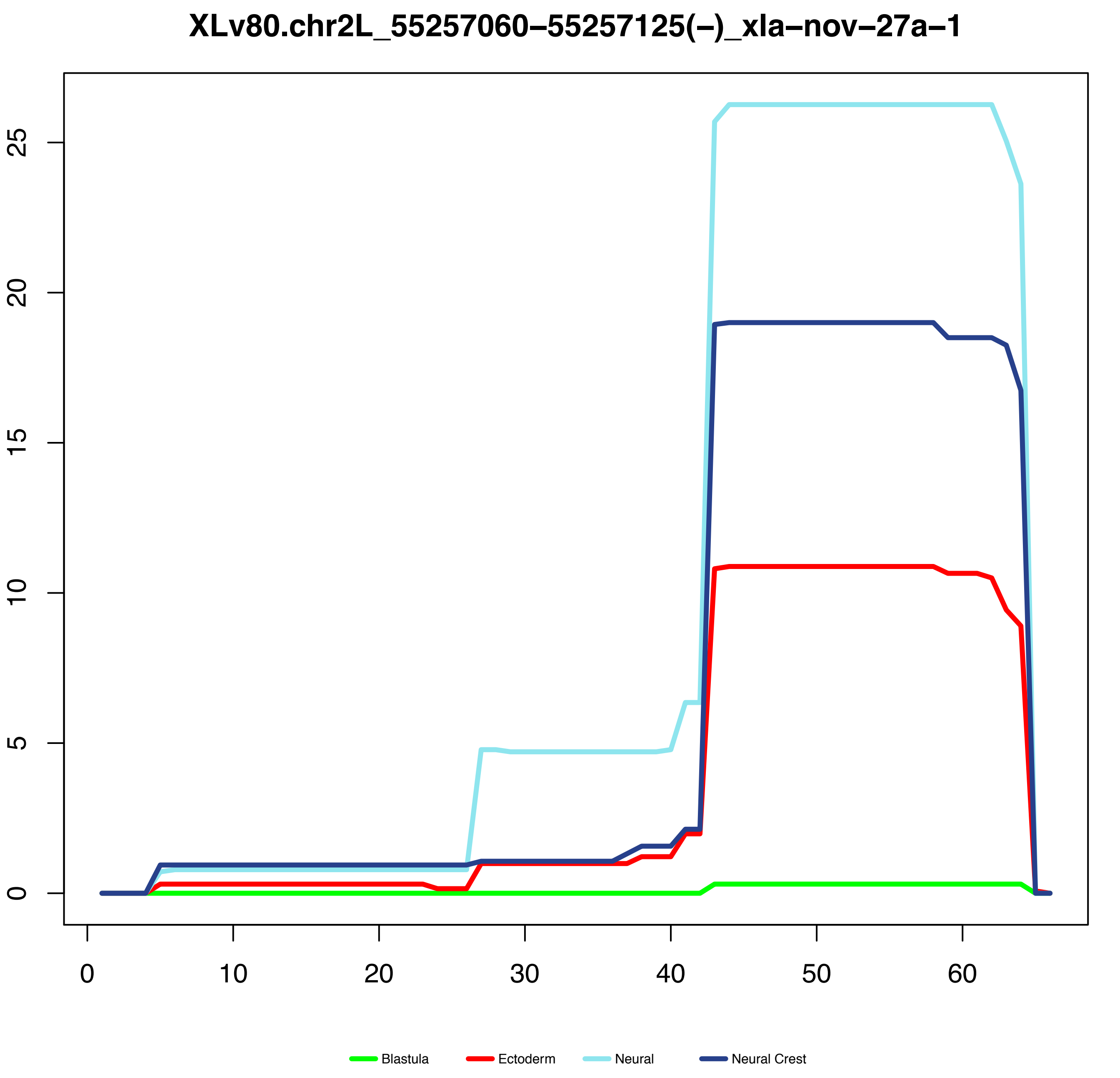
XLv80.chr2L_55257060−55257125(−)_xla−nov−27a−1
0
5
10
15
20
25
0
10
20
30
40
50
60
Blastula
Ectoderm
Neural
Neural Crest

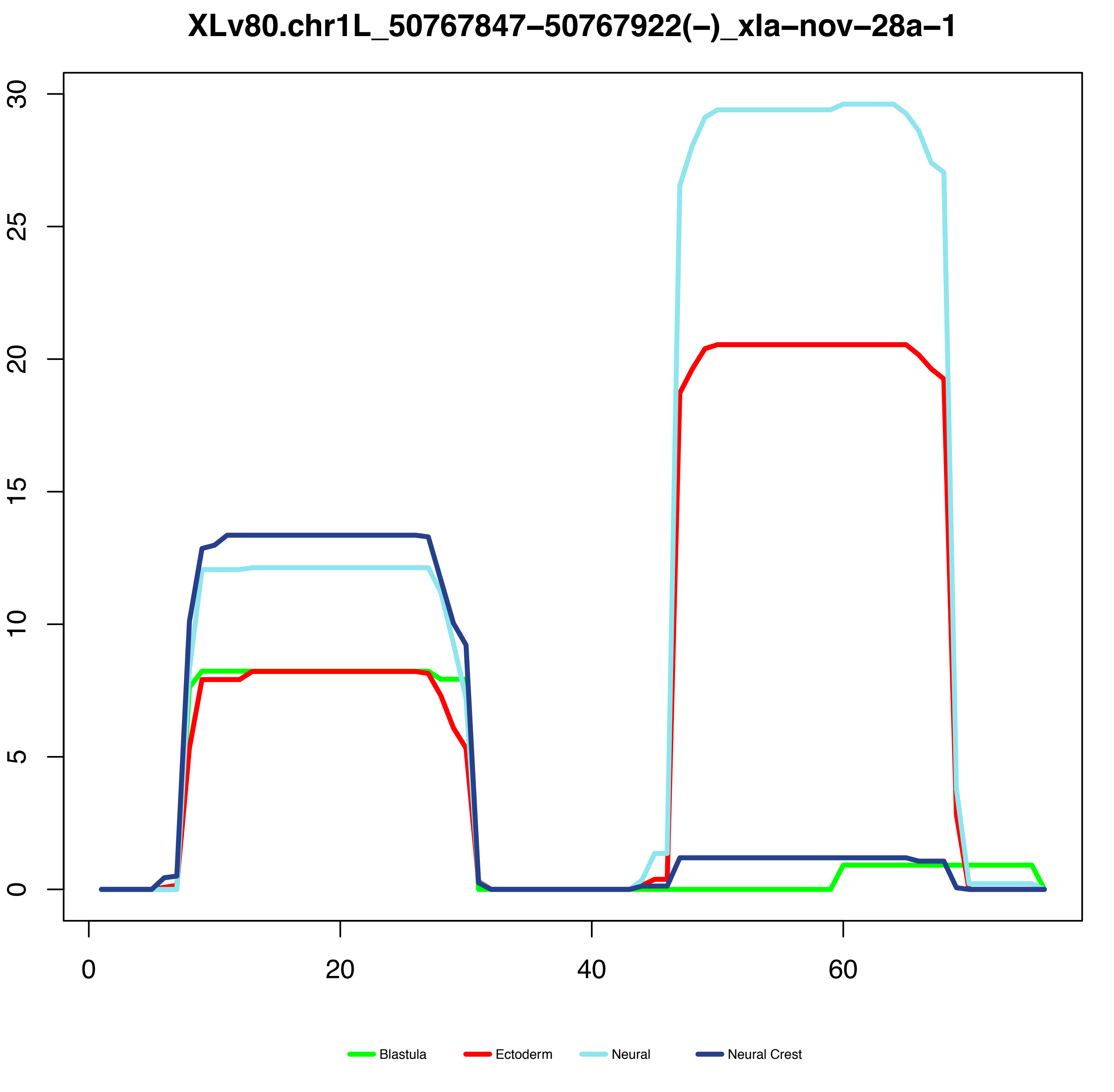
XLv80.chr1L_50767847–50767922(–)_xla–nov–28a–1
0
5
10
15
20
25
30
0
20
40
60
Blastula
Ectoderm
Neural
Neural Crest

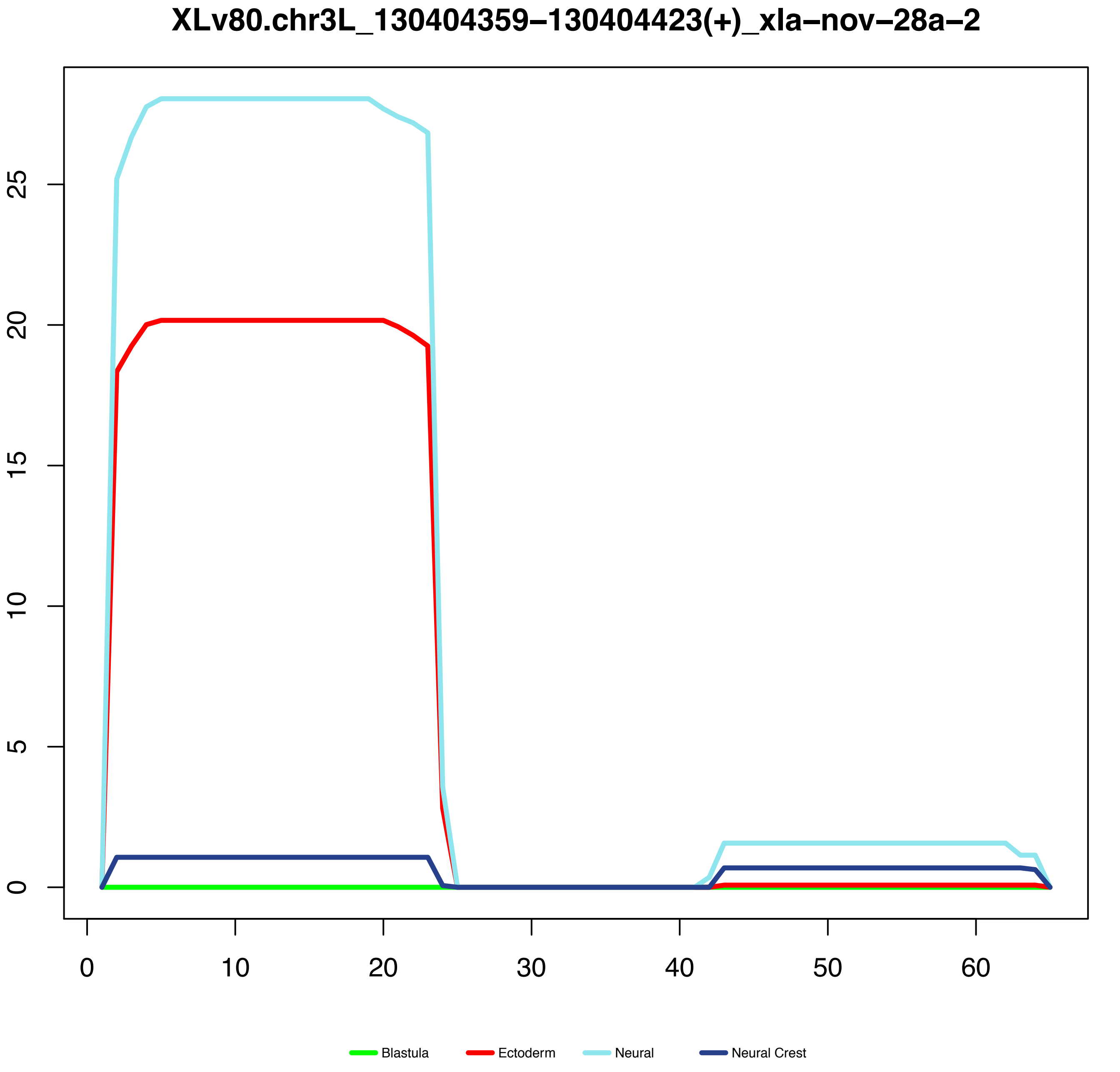
XLv80.chr3L_130404359−130404423(+)_xla−nov−28a−2
0
5
10
15
20
25
0
10
20
30
40
50
60
Blastula
Ectoderm
Neural
Neural Crest

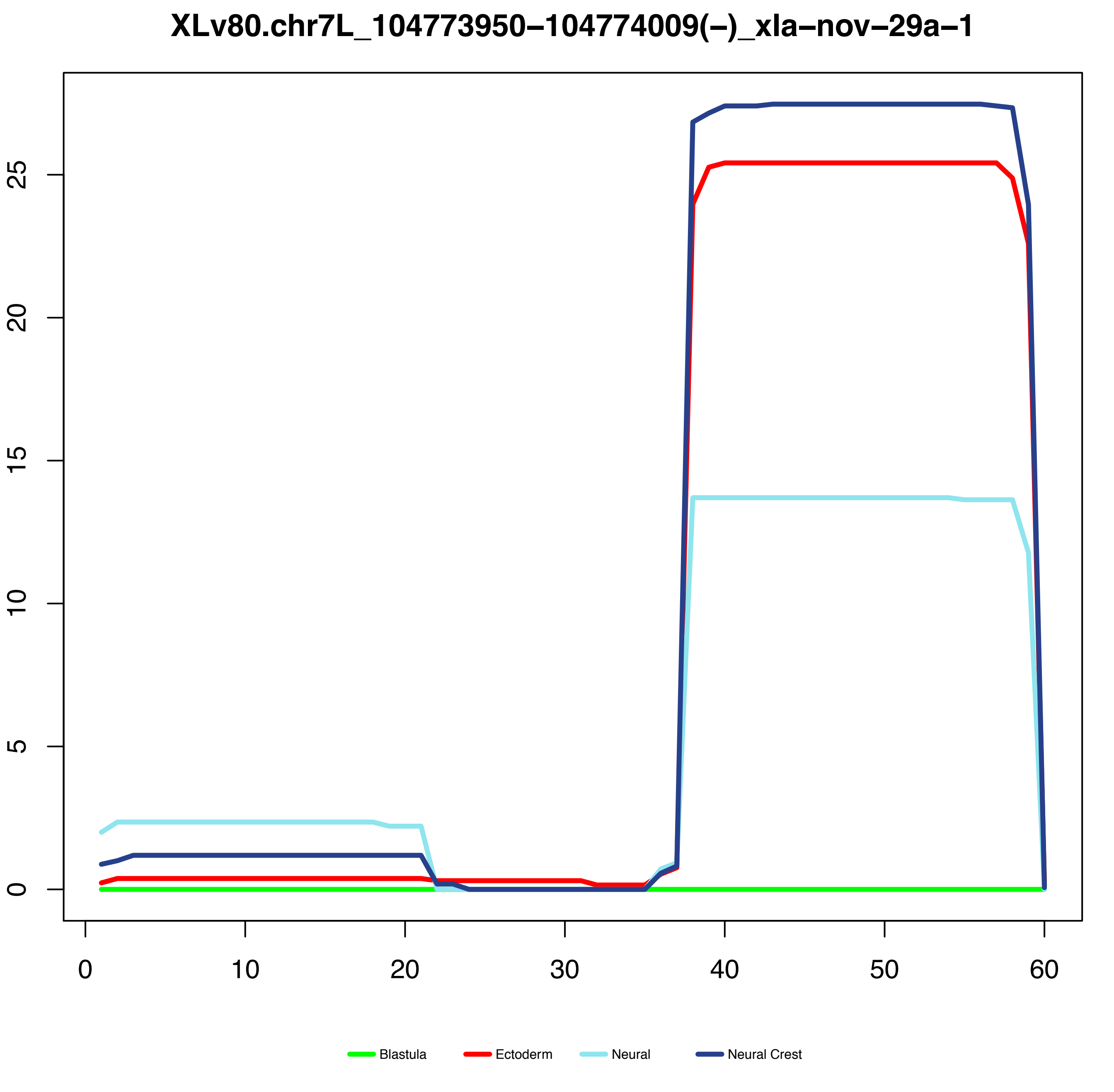

XLv80.chr7L_104773950−104774009(−)_xla−nov−29a−1
0
5
10
15
20
25
0
10
20
30
40
50
60
Blastula
Ectoderm
Neural
Neural Crest

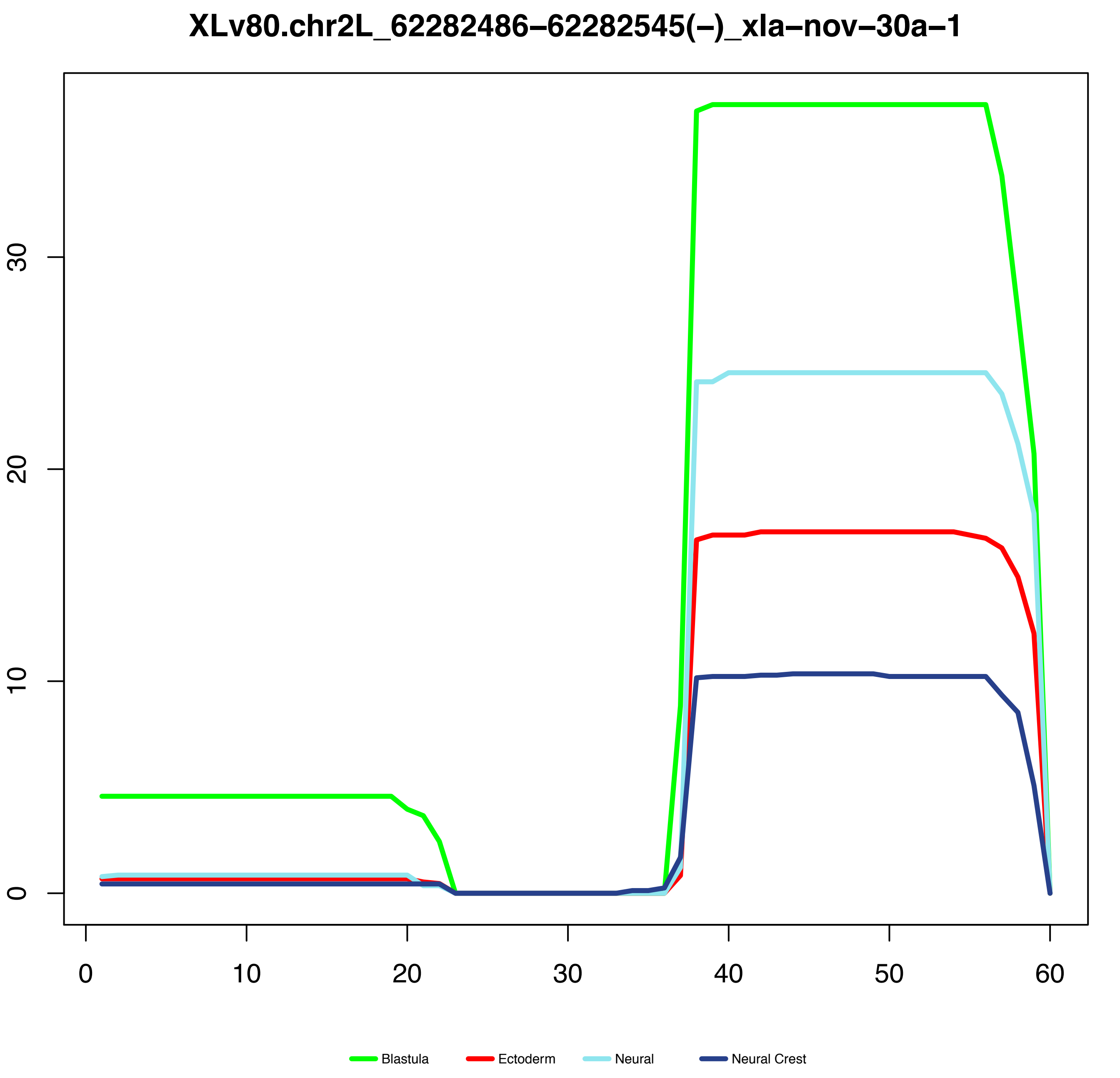
XLv80.chr2L_62282486−62282545(−)_xla−nov−30a−1
0
10
20
30
0
10
20
30
40
50
60
Blastula
Ectoderm
Neural
Neural Crest

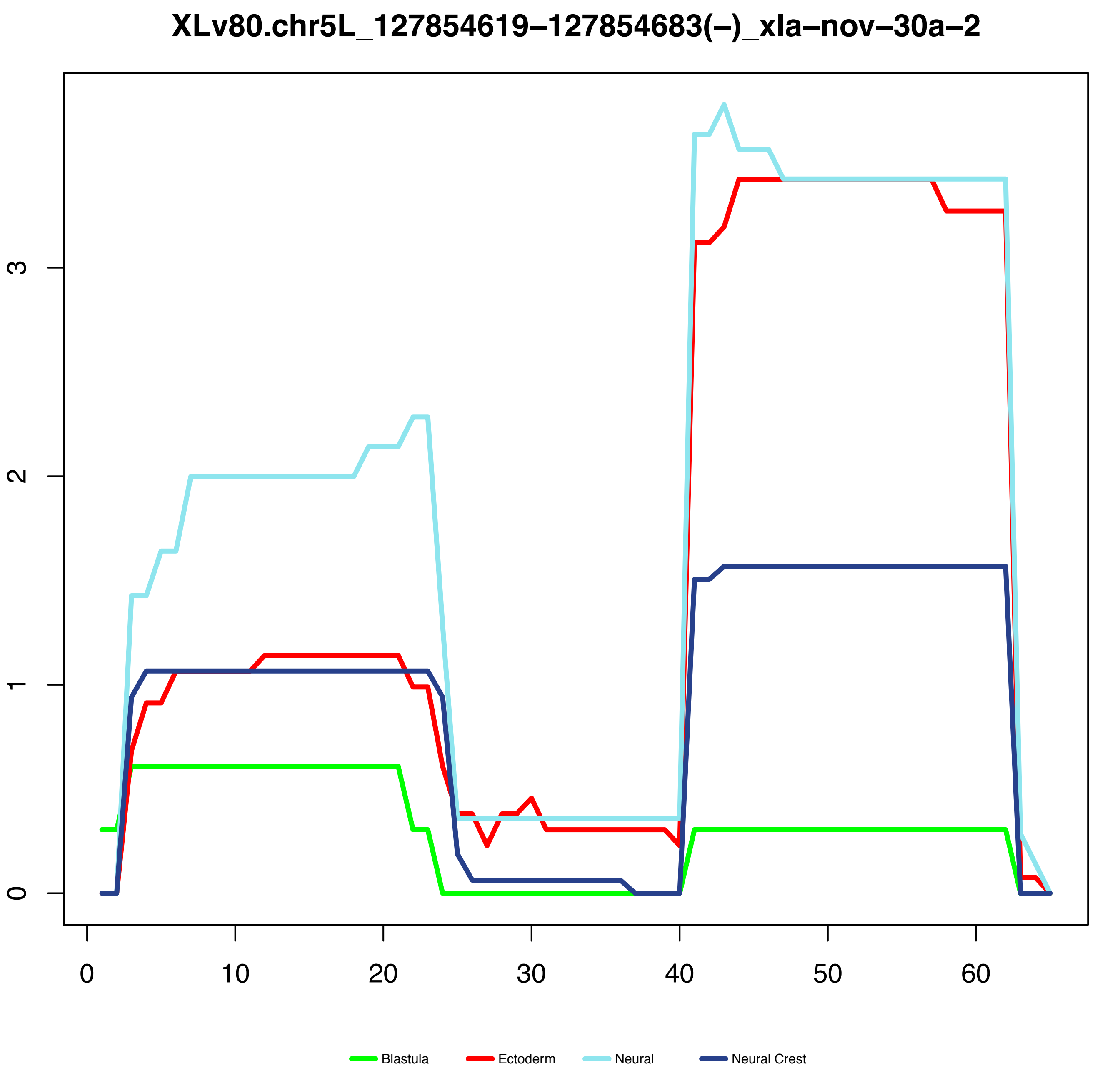
XLv80.chr5L_127854619−127854683(−)_xla−nov−30a−2
0
1
2
3
0
10
20
30
40
50
60
Blastula
Ectoderm
Neural
Neural Crest

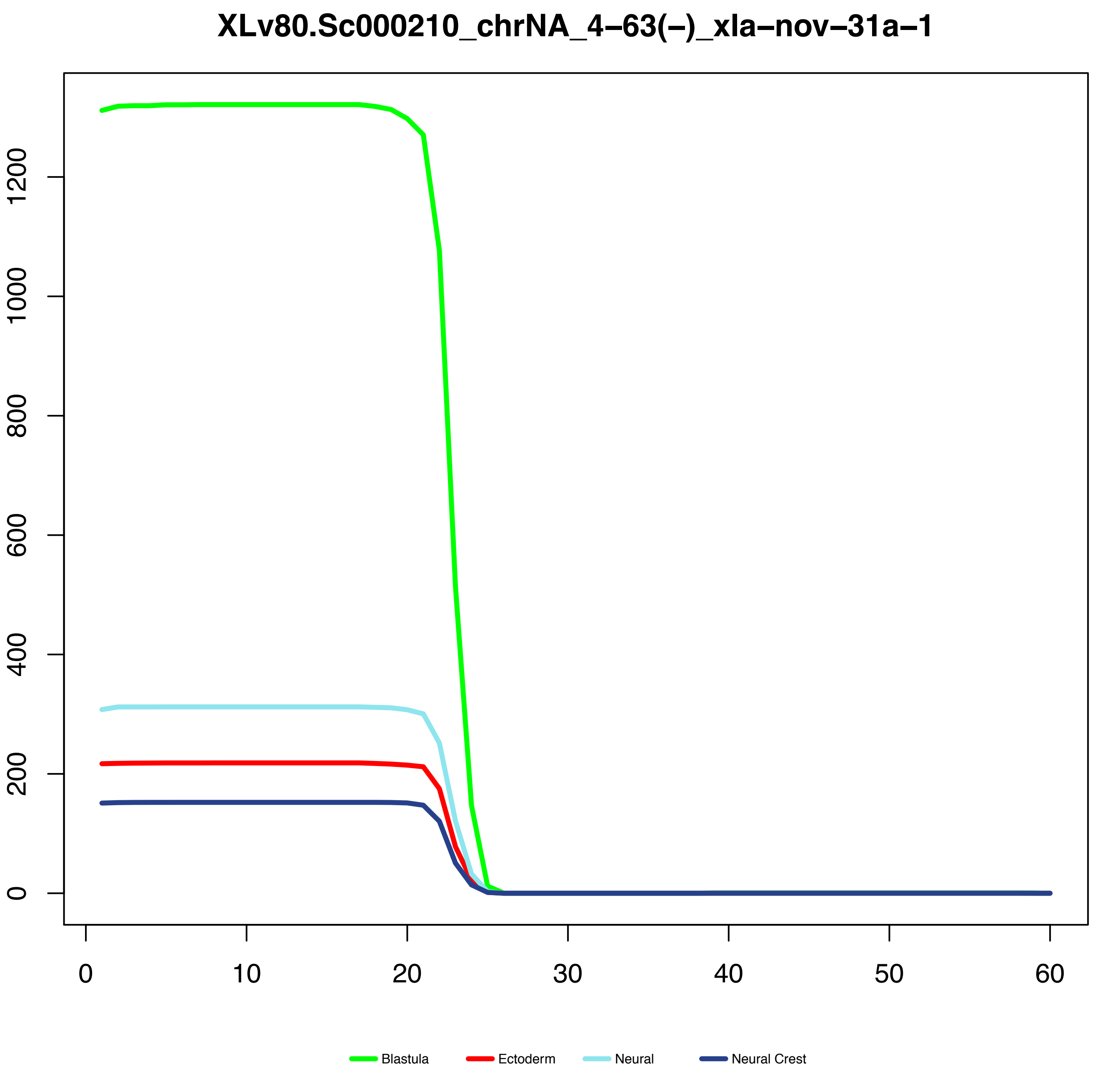

XLv80.Sc000210_chrNA_4−63(−)_xla−nov−31a−1
0
200
400
600
800
1000
1200
0
10
20
30
40
50
60
Blastula
Ectoderm
Neural
Neural Crest

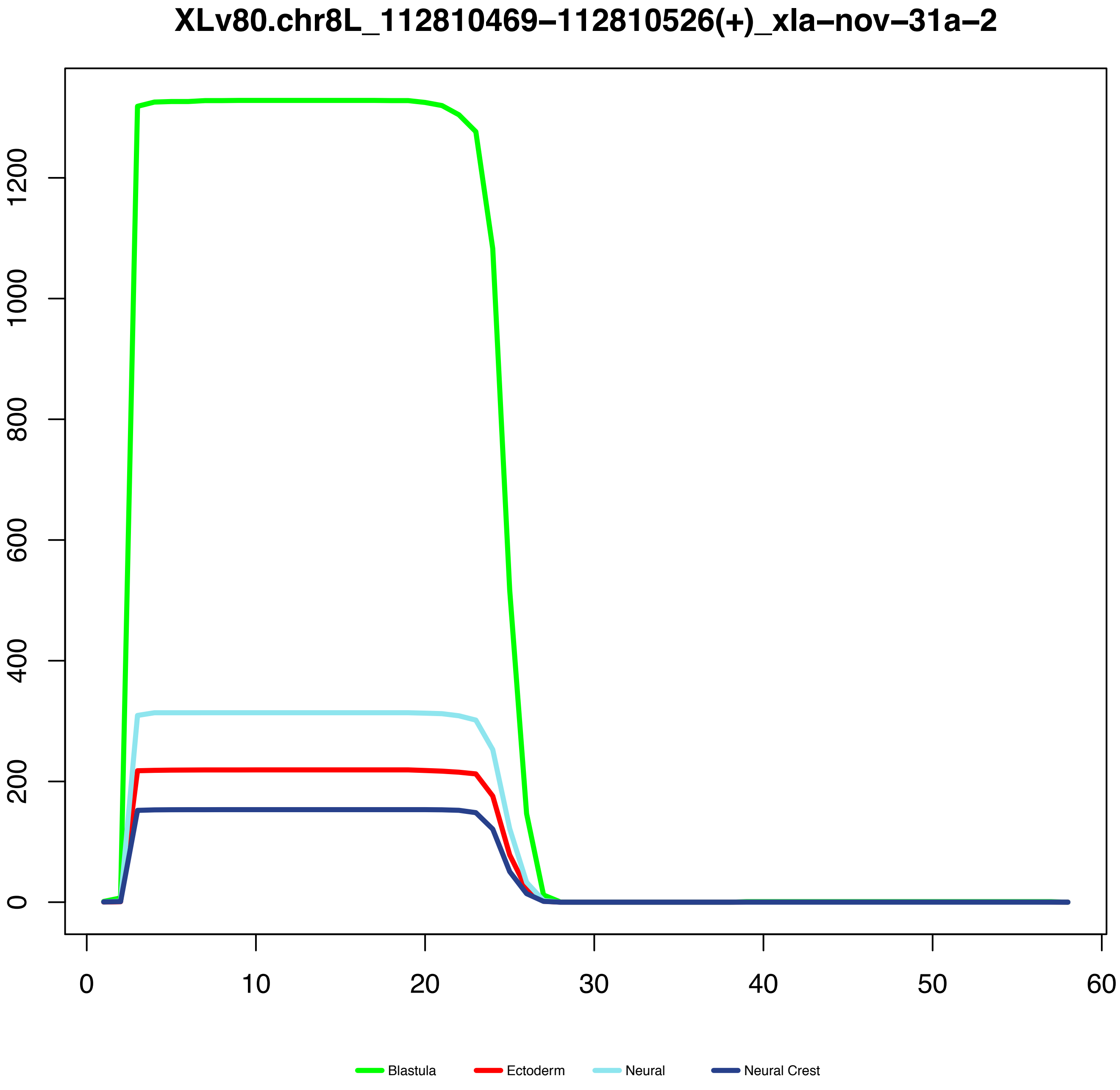
XLv80.chr8L_112810469−112810526(+)_xla−nov−31a−2
0
200
400
600
800
1000
1200
0
10
20
30
40
50
60
Blastula
Ectoderm
Neural
Neural Crest

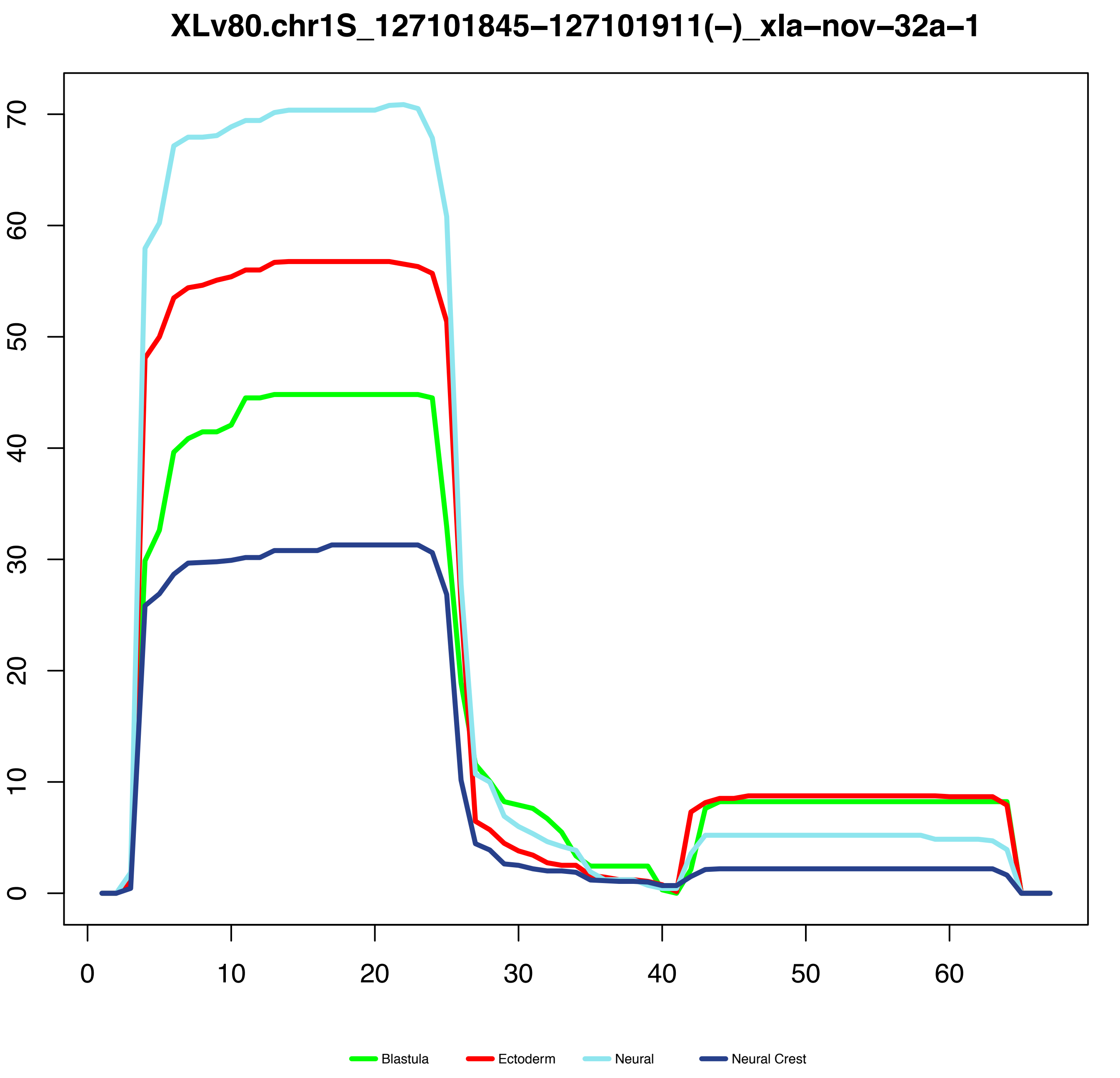
XLv80.chr1S_127101845−127101911(−)_xla−nov−32a−1
0
10
20
30
40
50
60
70
0
10
20
30
40
50
60
Blastula
Ectoderm
Neural
Neural Crest

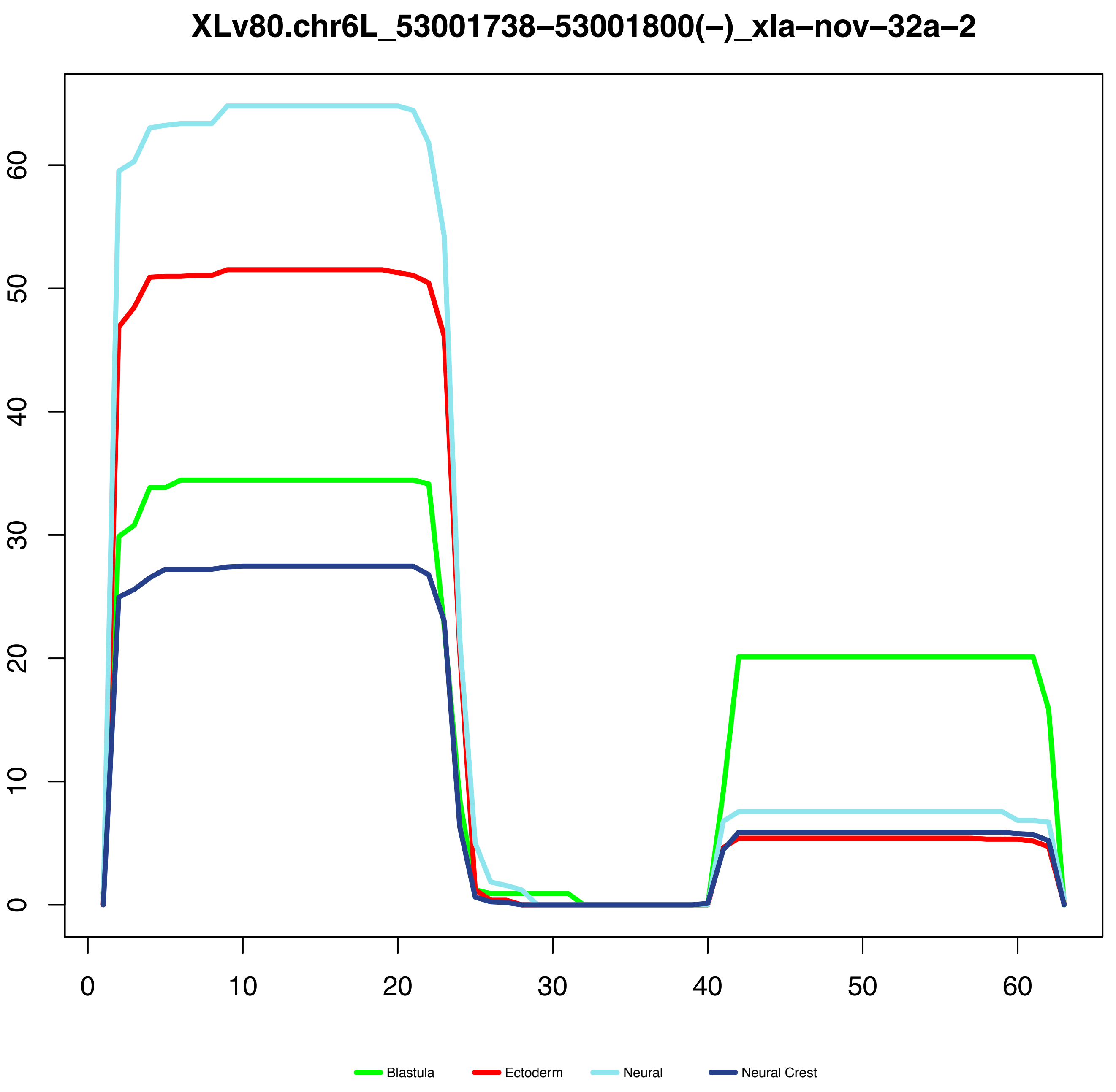
XLv80.chr6L_53001738−53001800(−)_xla−nov−32a−2
0
10
20
30
40
50
60
0
10
20
30
40
50
60
Blastula
Ectoderm
Neural
Neural Crest

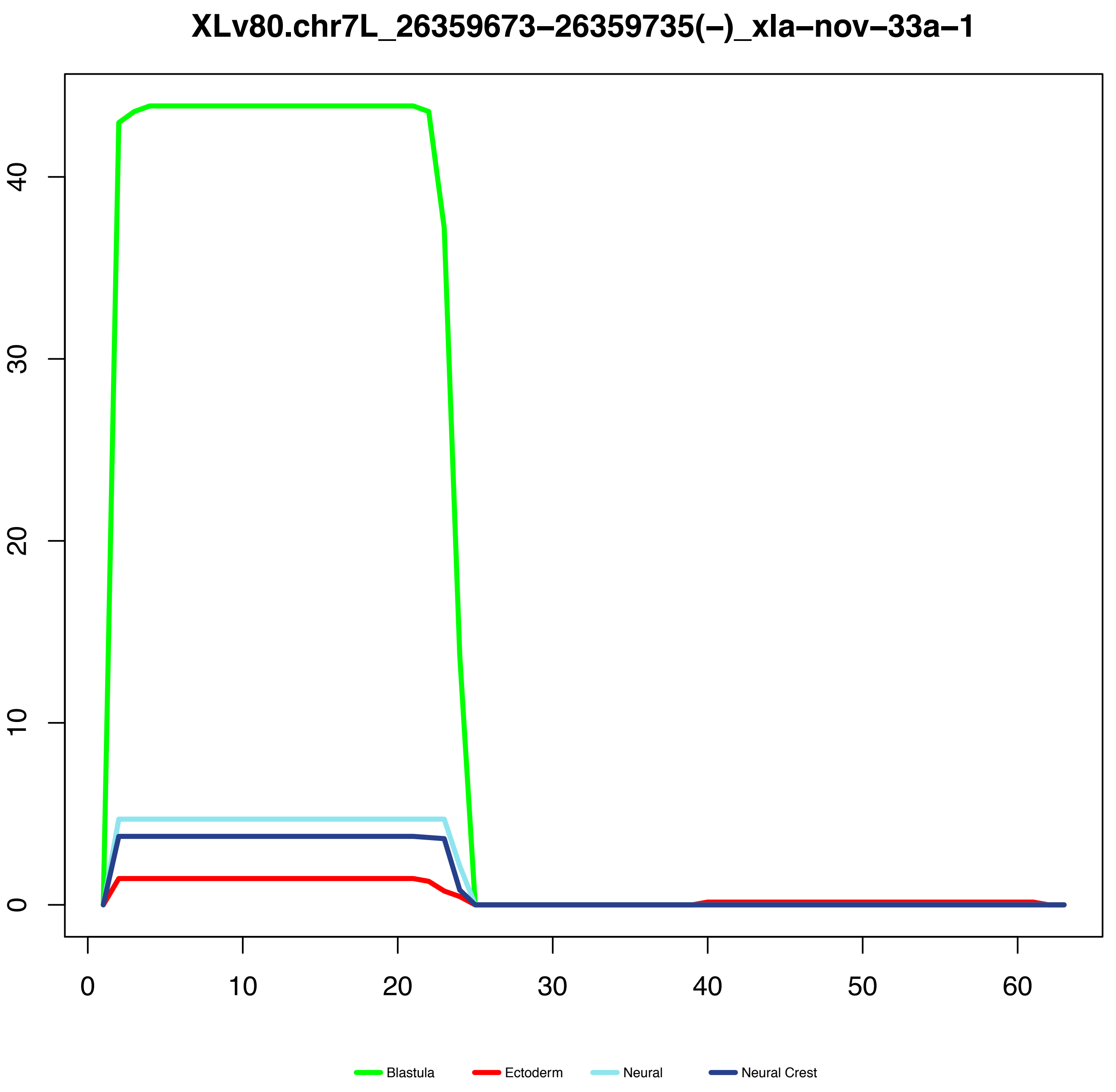
XLv80.chr7L_26359673–26359735(–)_xla–nov–33a–1
0
10
20
30
40
0
10
20
30
40
50
60
Blastula
Ectoderm
Neural
Neural Crest

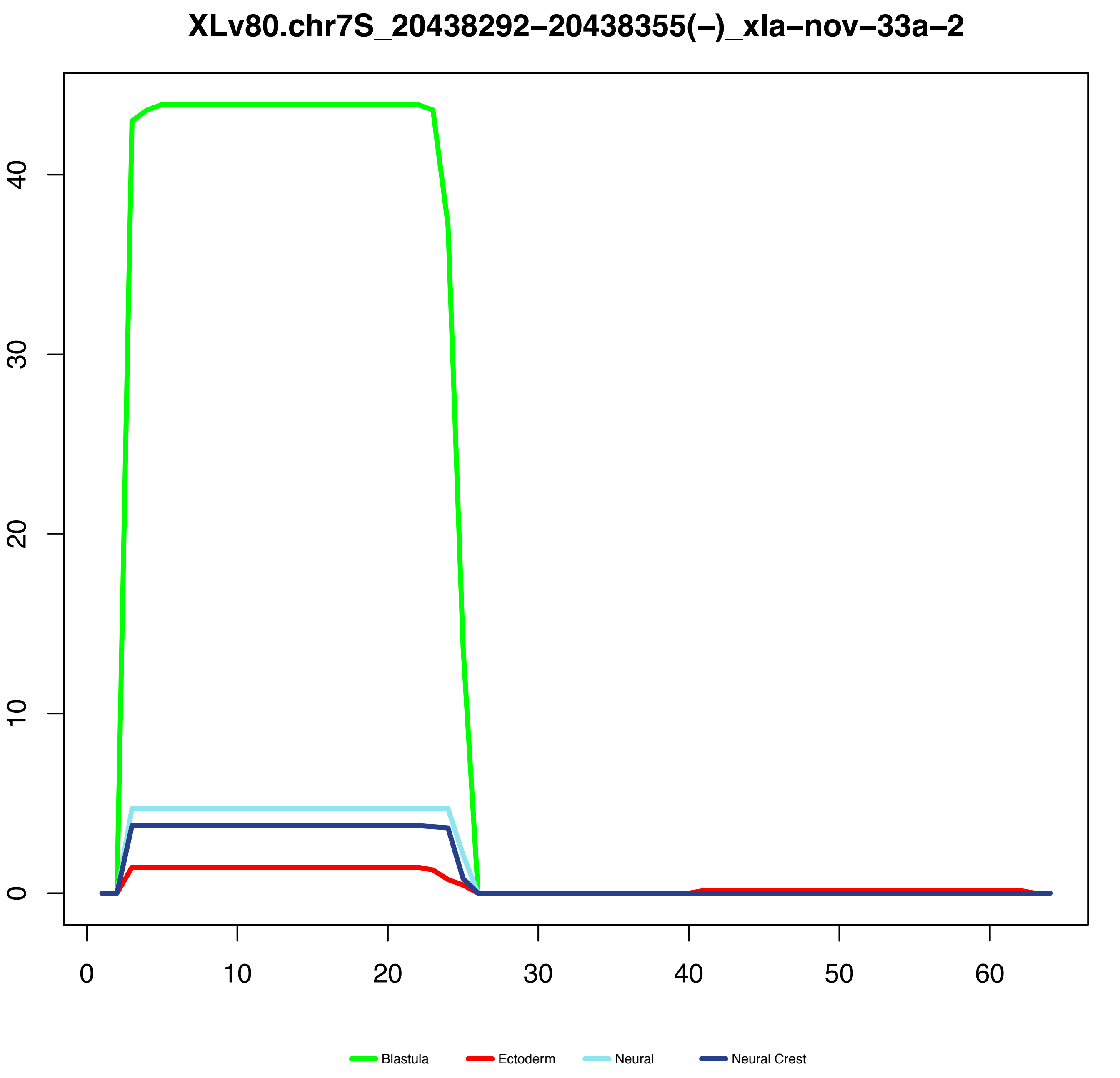
XLv80.chr7S_20438292−20438355(−)_xla−nov−33a−2
0
10
20
30
40
0
10
20
30
40
50
60
Blastula
Ectoderm
Neural
Neural Crest

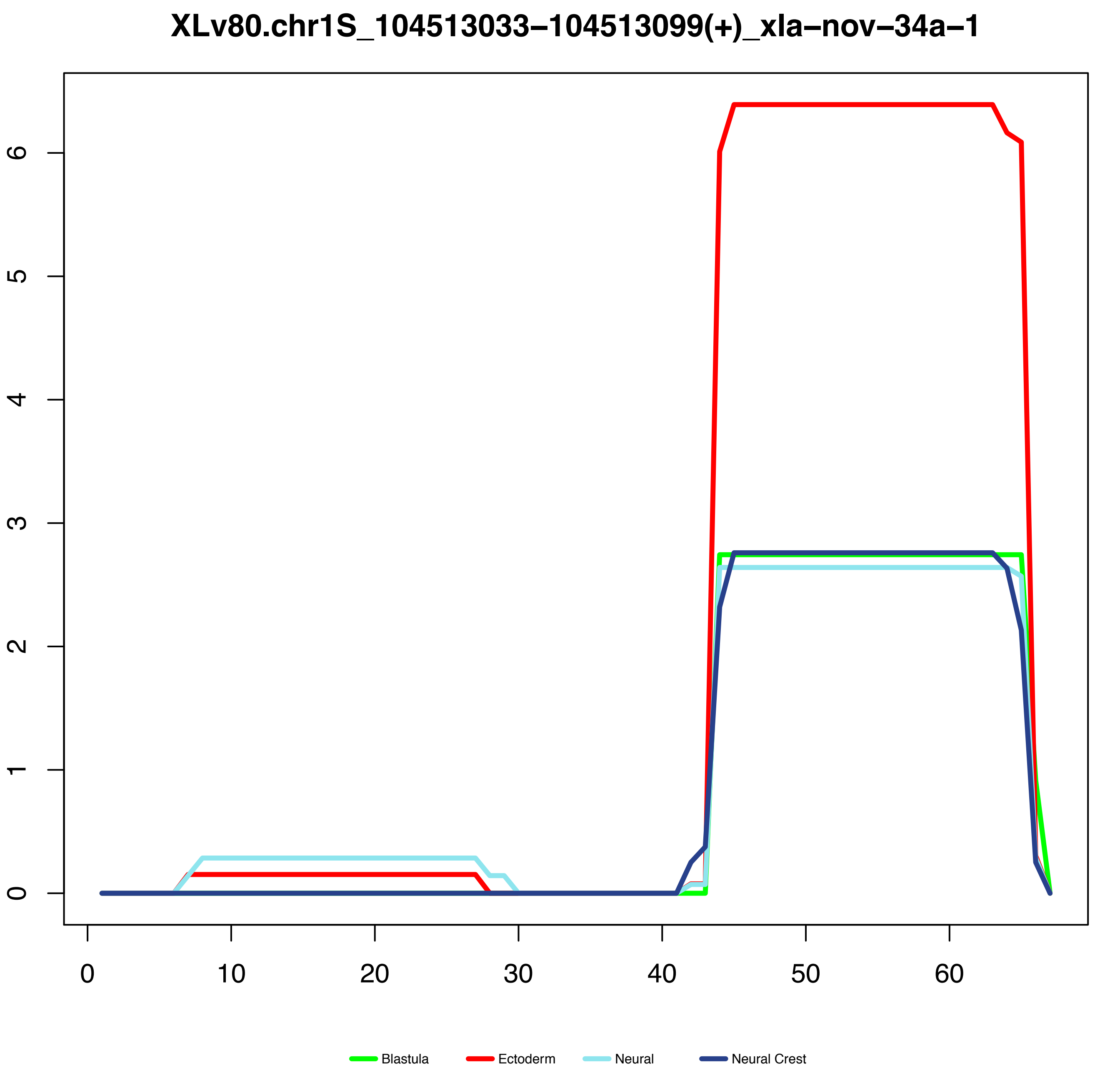
XLv80.chr1S_104513033−104513099(+)_xla−nov−34a−1
0
1
2
3
4
5
6
0
10
20
30
40
50
60
Blastula
Ectoderm
Neural
Neural Crest

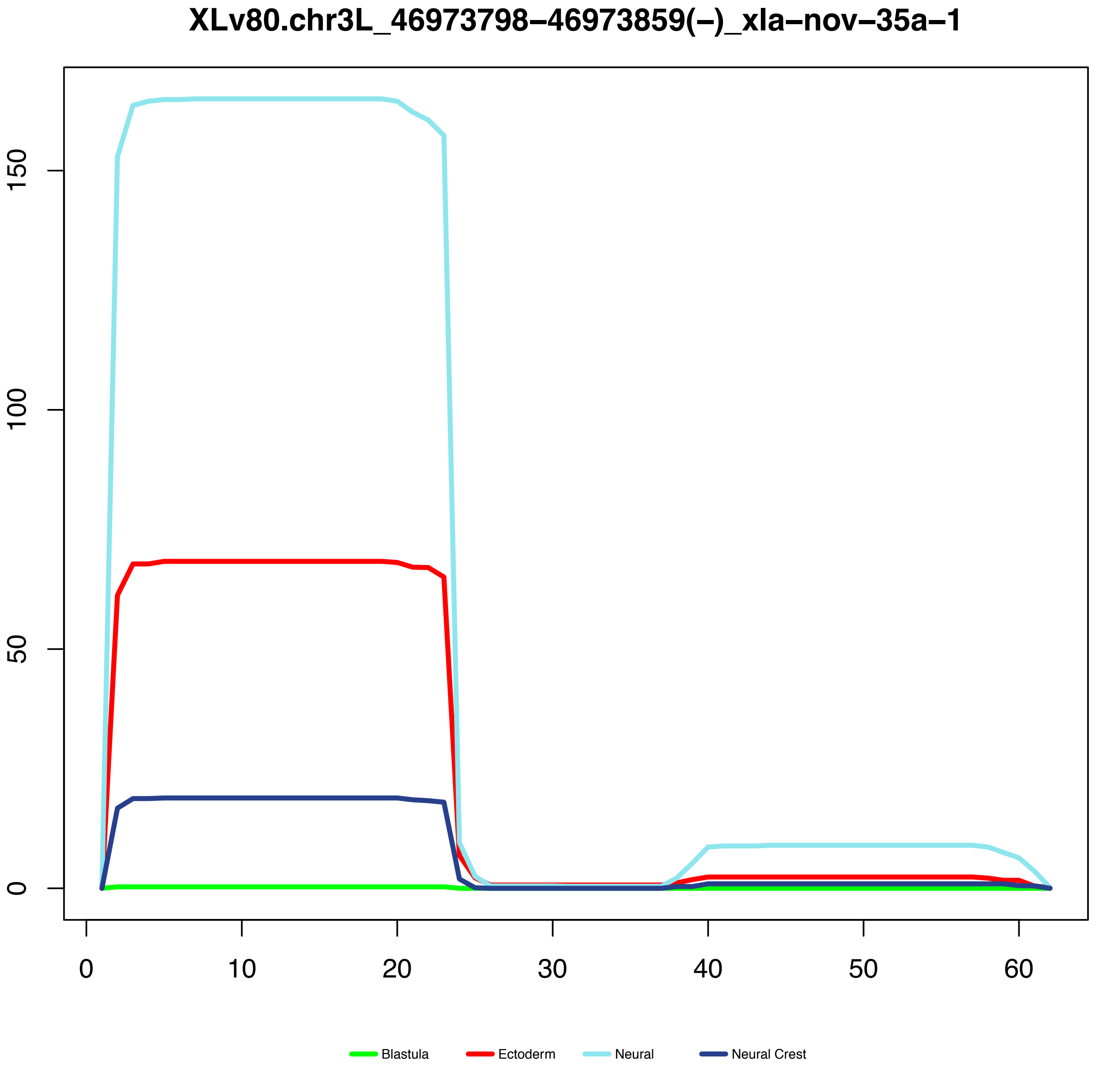

XLv80.chr3L_46973798−46973859(−)_xla−nov−35a−1
0
50
100
150
0
10
20
30
40
50
60
Blastula
Ectoderm
Neural
Neural Crest

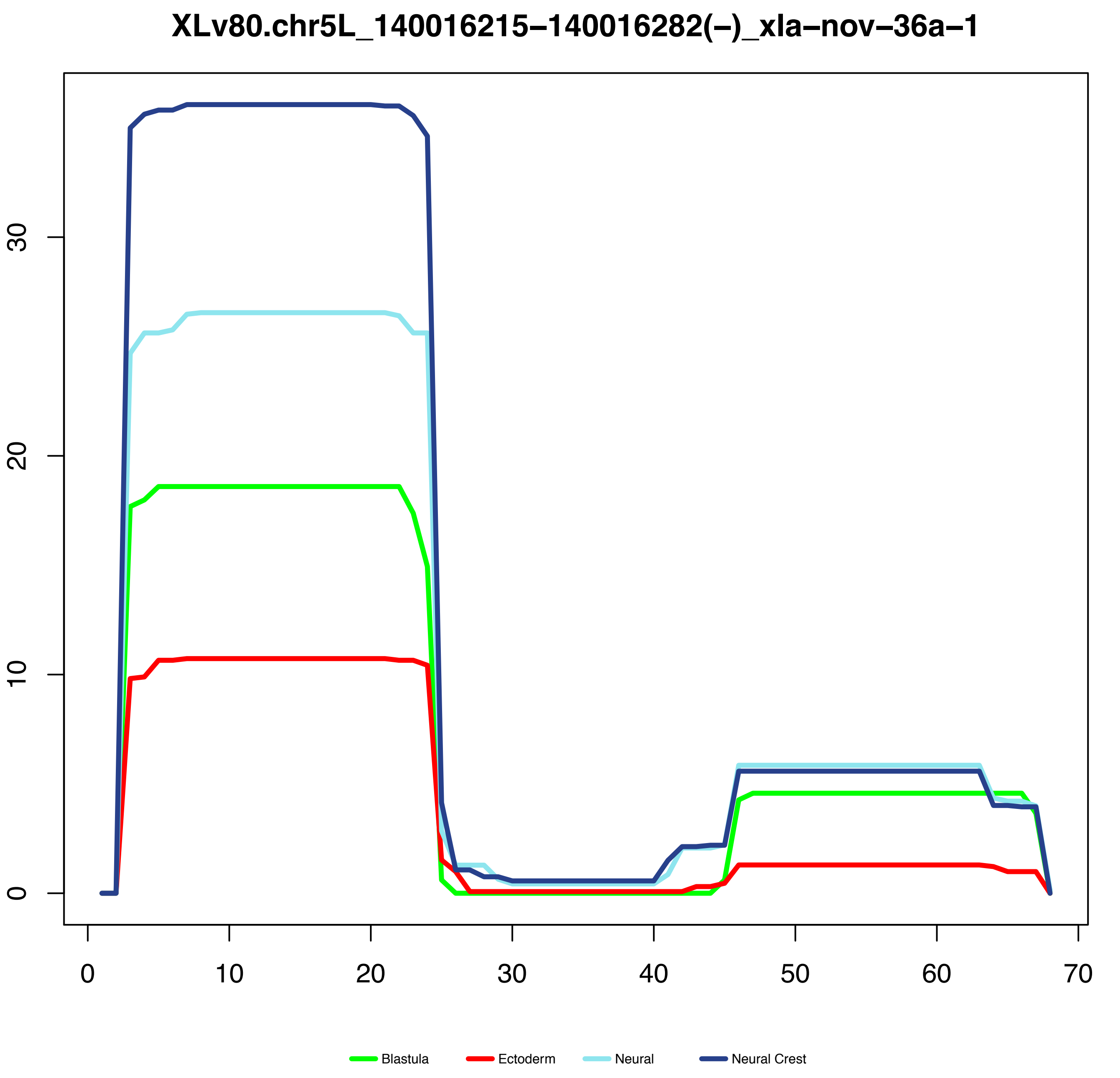
XLv80.chr5L_140016215−140016282(−)_xla−nov−36a−1
0
10
20
30
0
10
20
30
40
50
60
70
Blastula
Ectoderm
Neural
Neural Crest

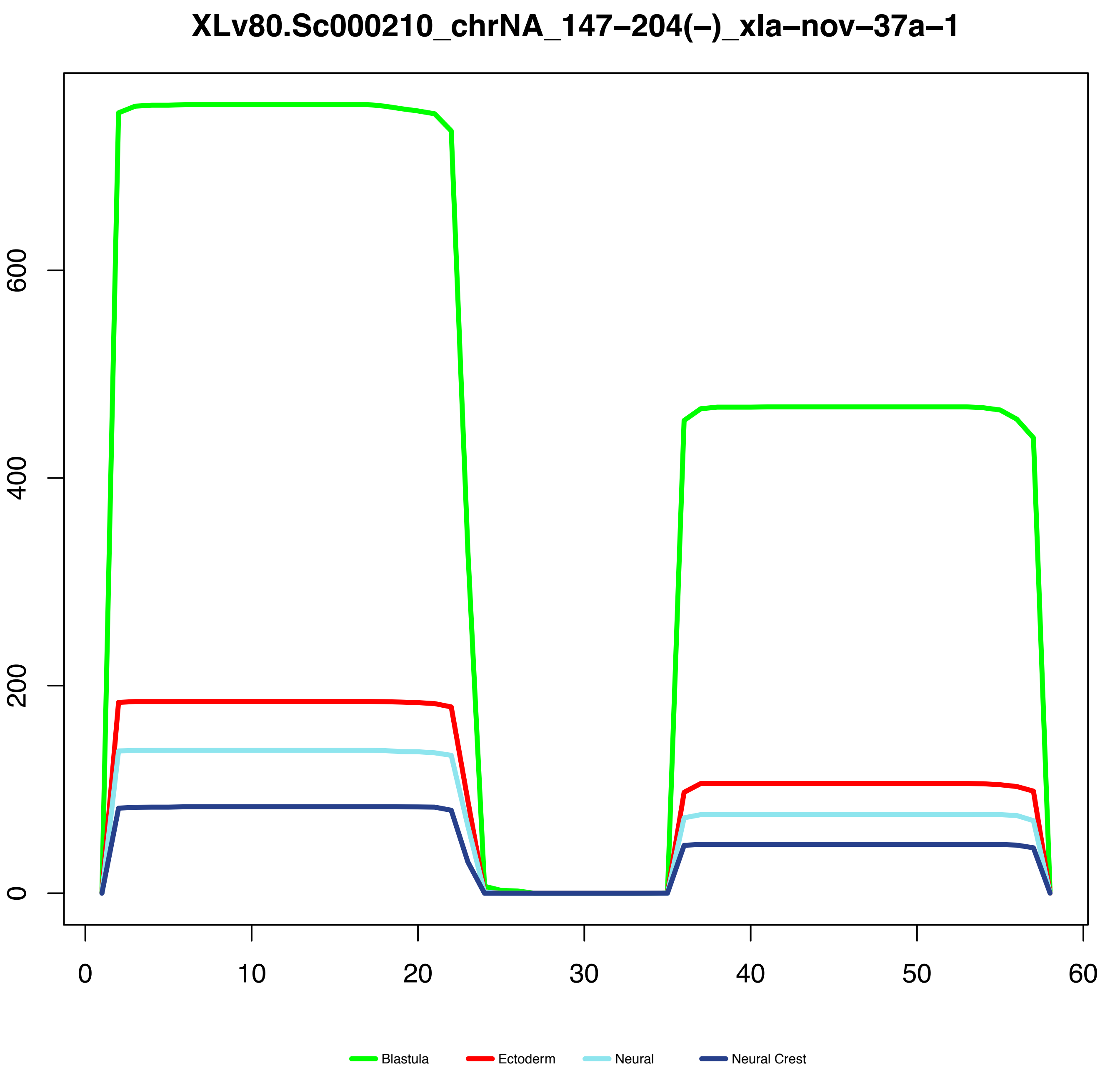
XLv80.Sc000210_chrNA_147−204(−)_xla−nov−37a−1
0
200
400
600
0
10
20
30
40
50
60
Blastula
Ectoderm
Neural
Neural Crest

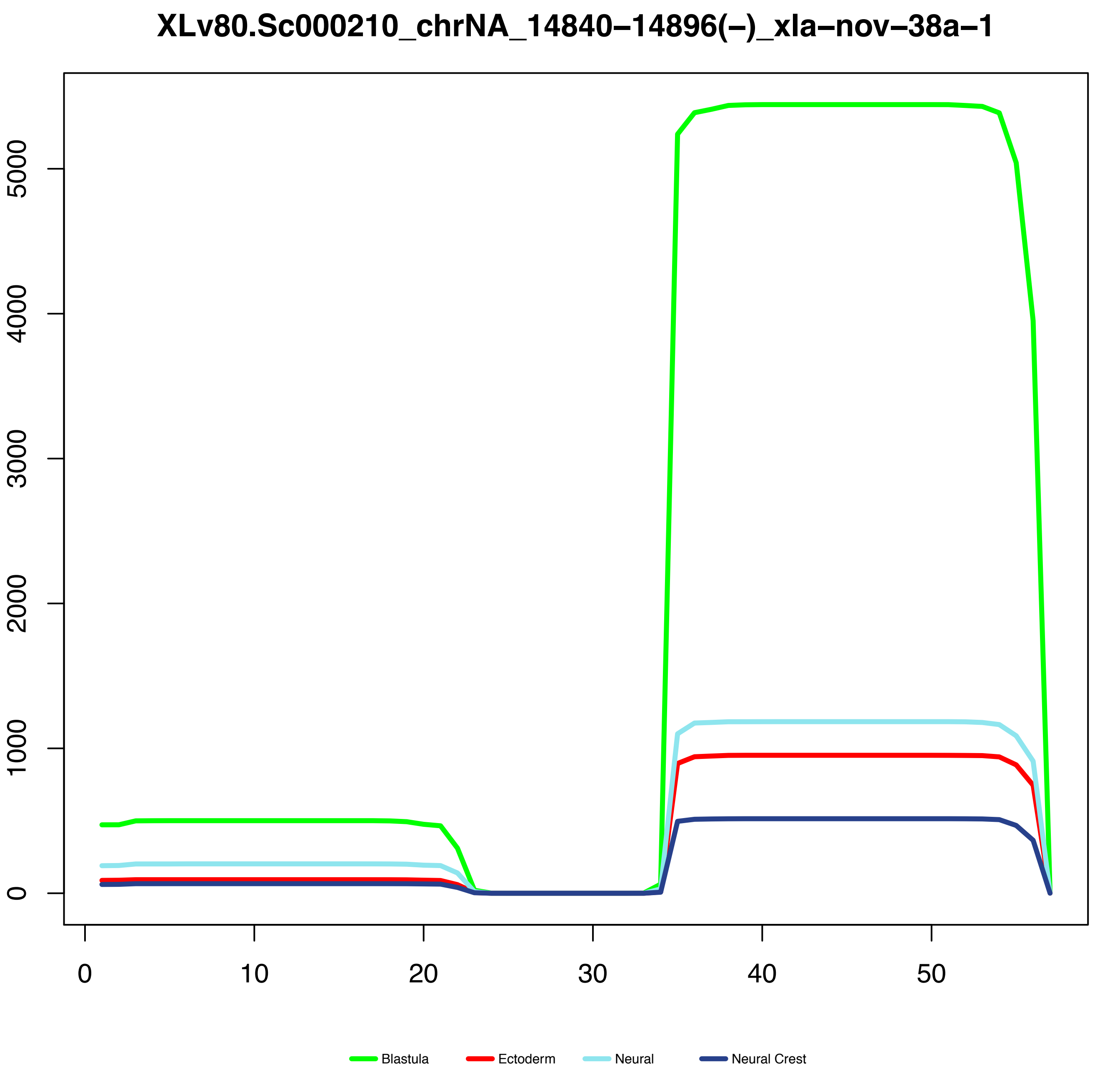

XLv80.Sc000210_chrNA_14840−14896(−)_xla−nov−38a−1
0
1000
2000
3000
4000
5000
0
10
20
30
40
50
Blastula
Ectoderm
Neural
Neural Crest

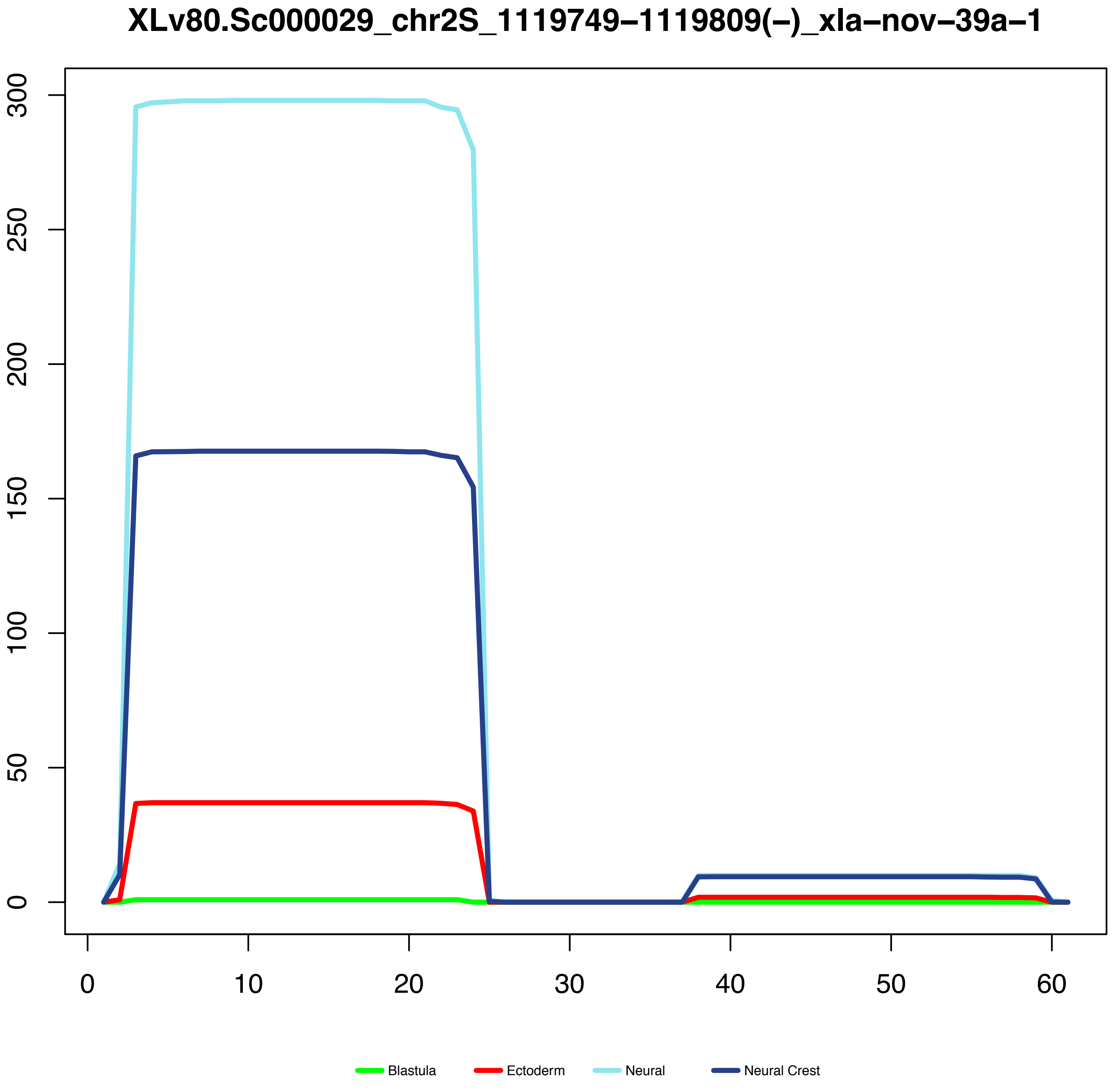
XLv80.Sc000029_chr2S_1119749−1119809(−)_xla−nov−39a−1
0
50
100
150
200
250
300
0
10
20
30
40
50
60
Blastula
Ectoderm
Neural
Neural Crest

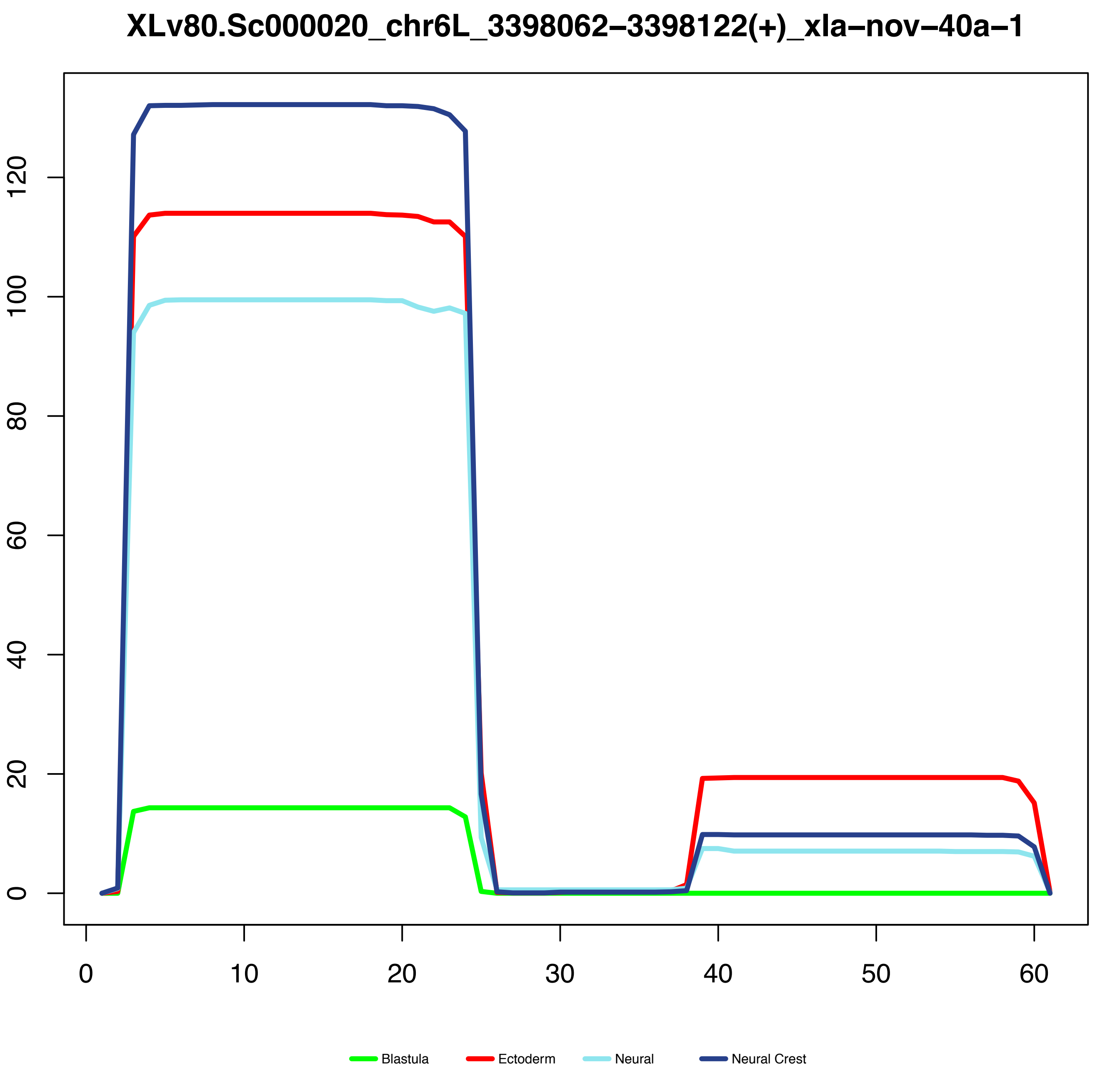

XLv80.Sc000020_chr6L_3398062−3398122(+)_xla−nov−40a−1
0
20
40
60
80
100
120
0
10
20
30
40
50
60
Blastula
Ectoderm
Neural
Neural Crest

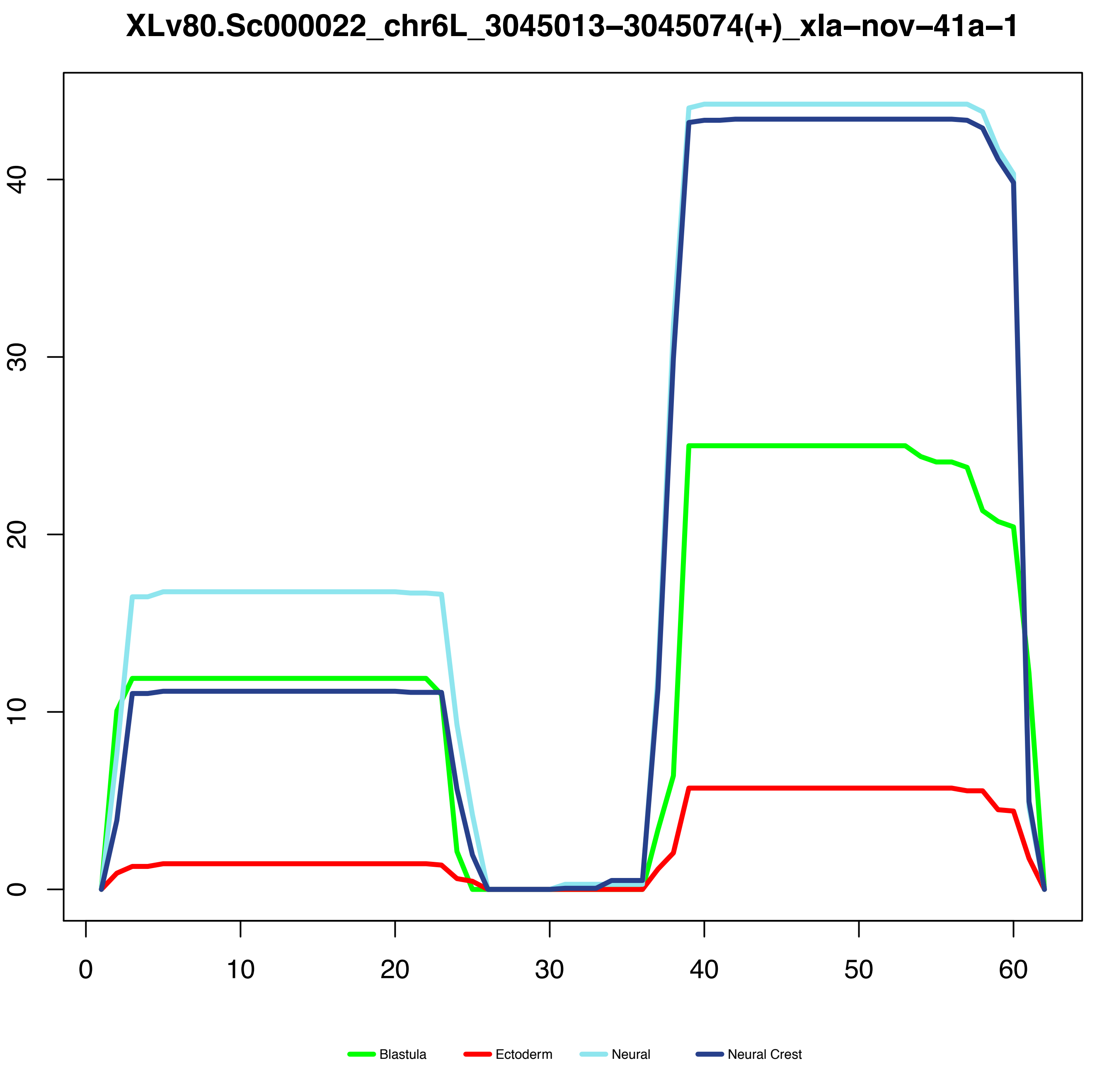
XLv80.Sc000022_chr6L_3045013−3045074(+)_xla−nov−41a−1
0
10
20
30
40
0
10
20
30
40
50
60
Blastula
Ectoderm
Neural
Neural Crest

XLv80.Sc000059_chr2L_875418−875481(+)_xla−nov−42a−1

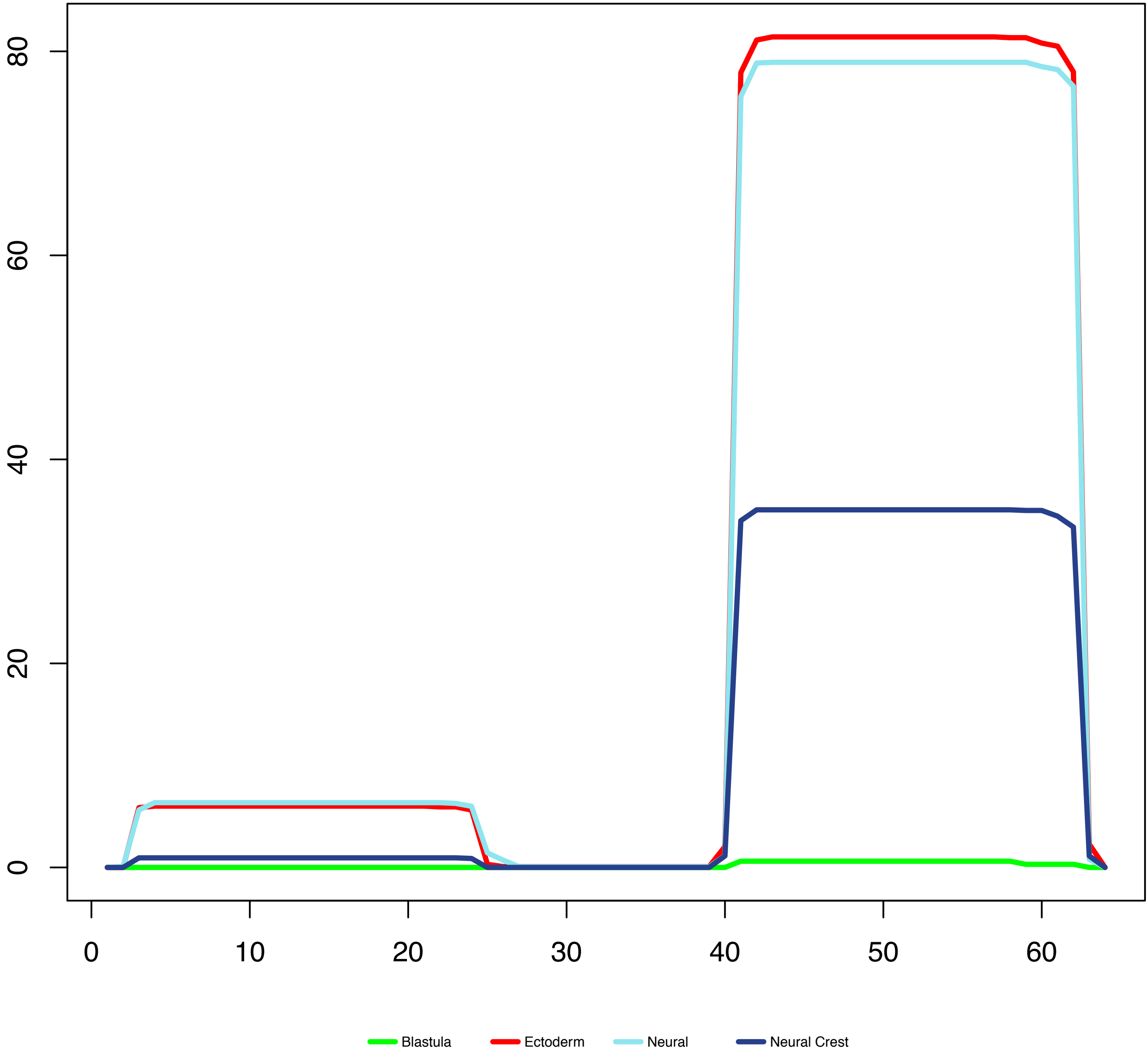

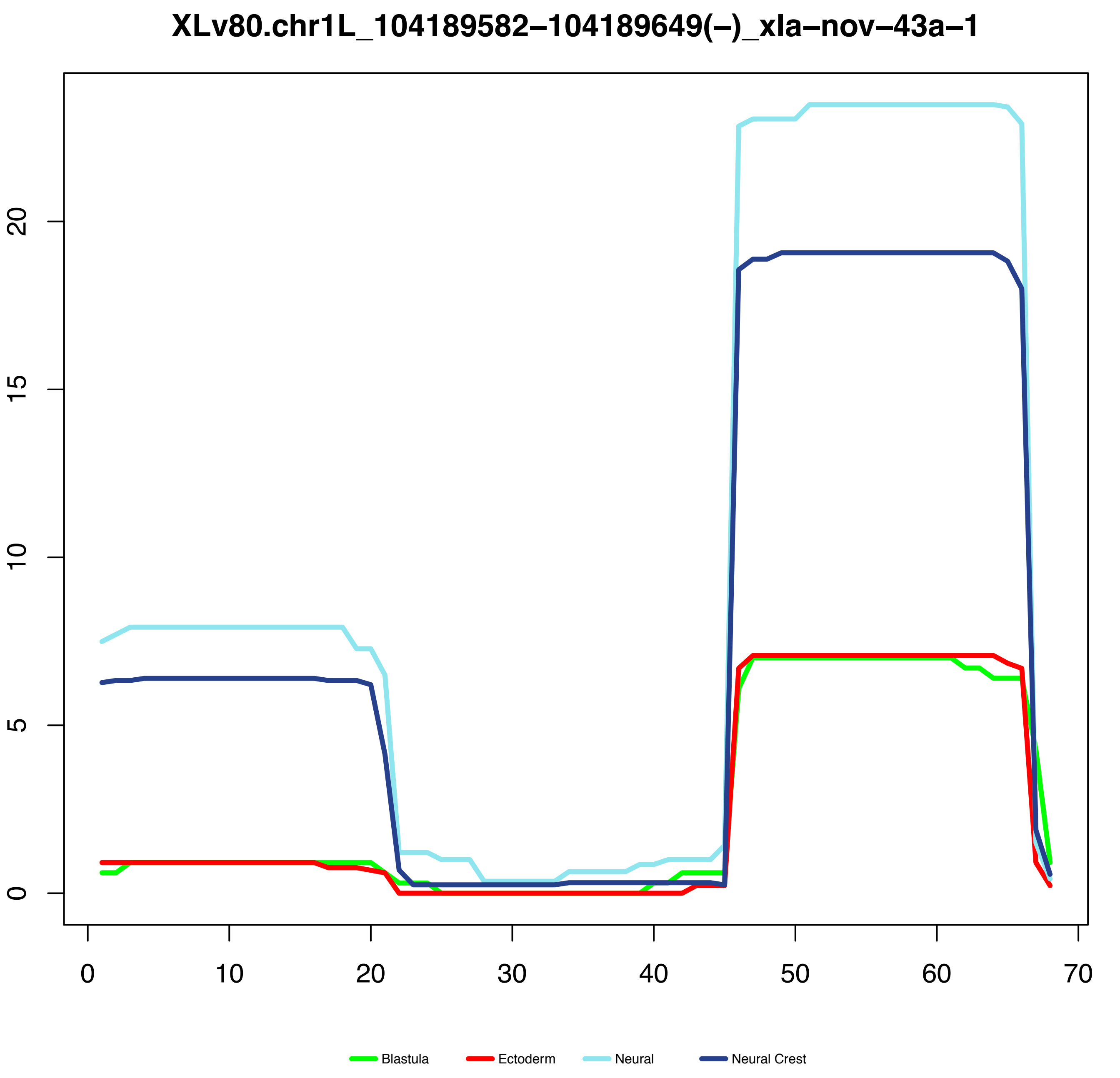
XLv80.chr1L_104189582−104189649(−)_xla−nov−43a−1
0
5
10
15
20
0
10
20
30
40
50
60
70
Blastula
Ectoderm
Neural
Neural Crest

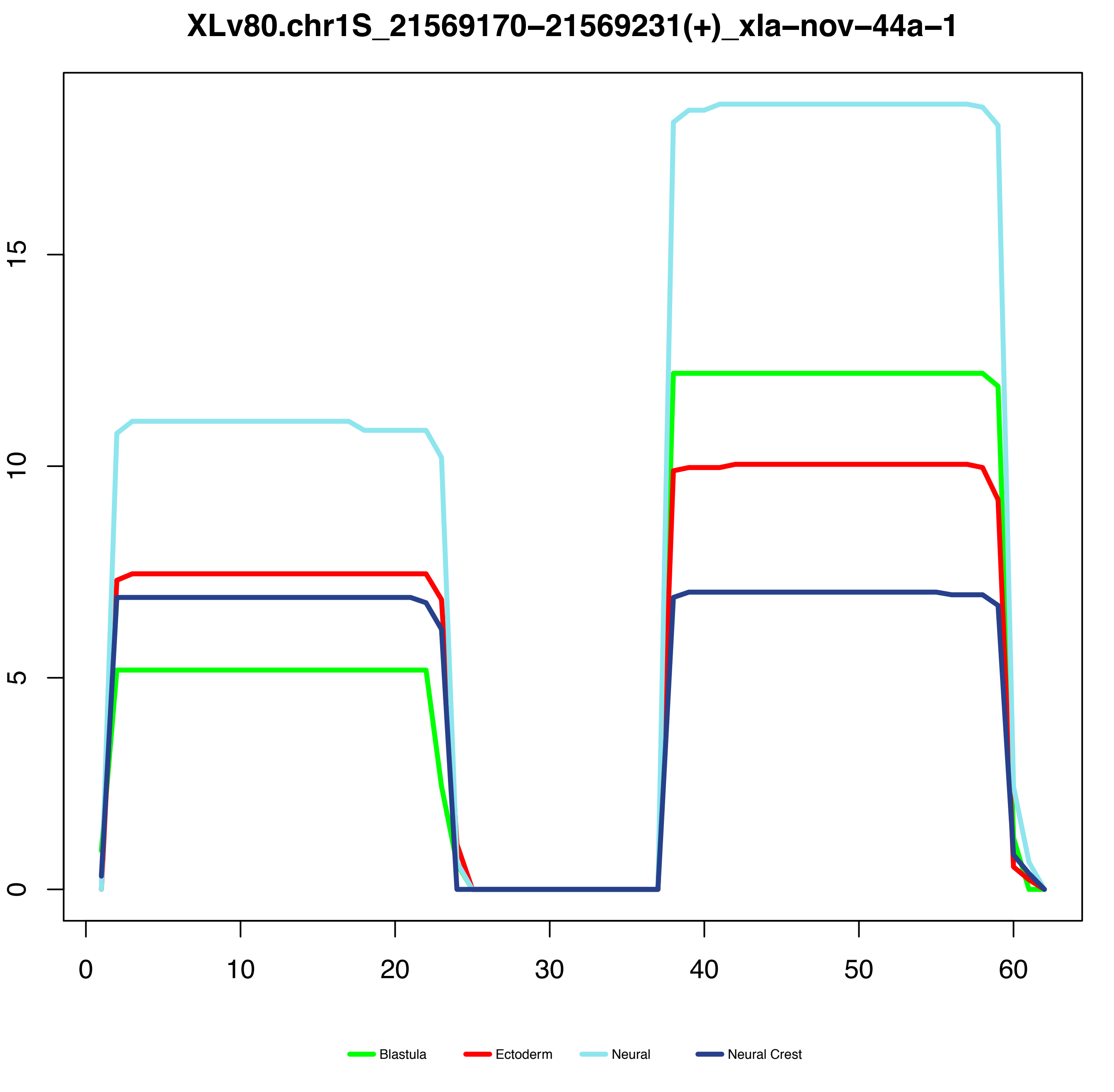

XLv80.chr1S_21569170−21569231(+)_xla−nov−44a−1
0
5
10
15
0
10
20
30
40
50
60
Blastula
Ectoderm
Neural
Neural Crest

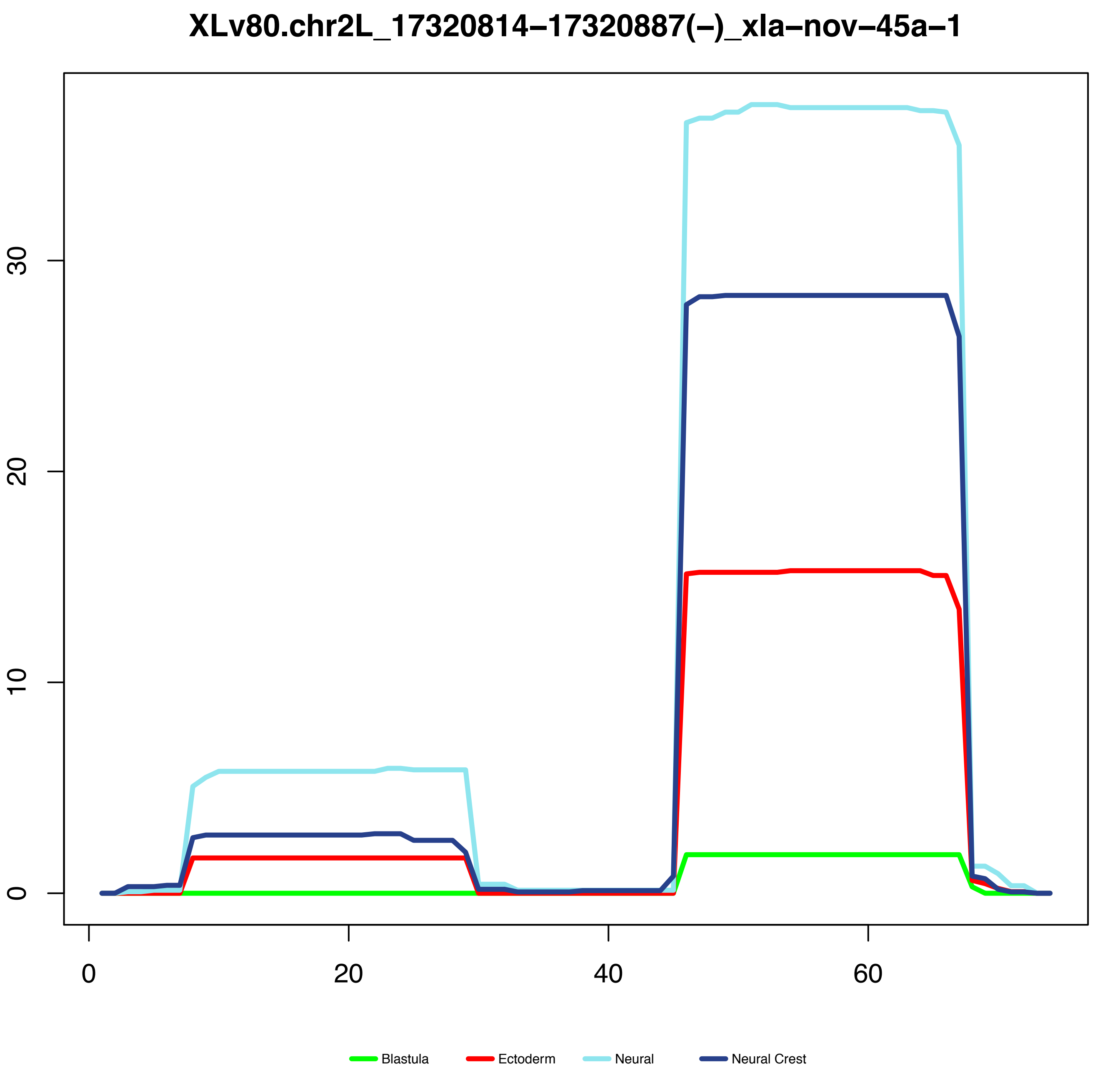

XLv80.chr2L_17320814−17320887(−)_xla−nov−45a−1
0
10
20
30
0
20
40
60
Blastula
Ectoderm
Neural
Neural Crest

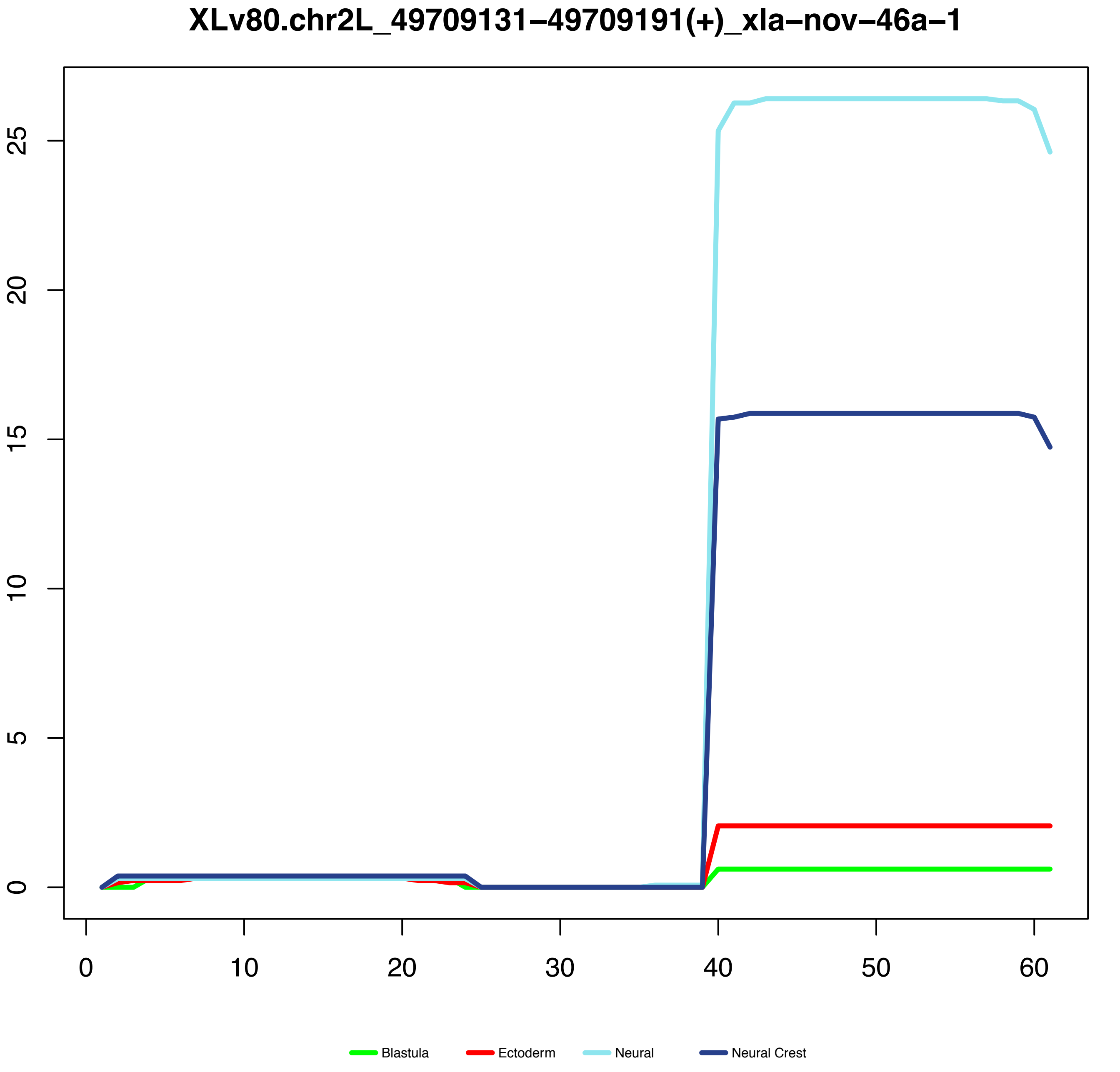

XLv80.chr2L_49709131−49709191(+)_xla−nov−46a−1
0
5
10
15
20
25
0
10
20
30
40
50
60
Blastula
Ectoderm
Neural
Neural Crest

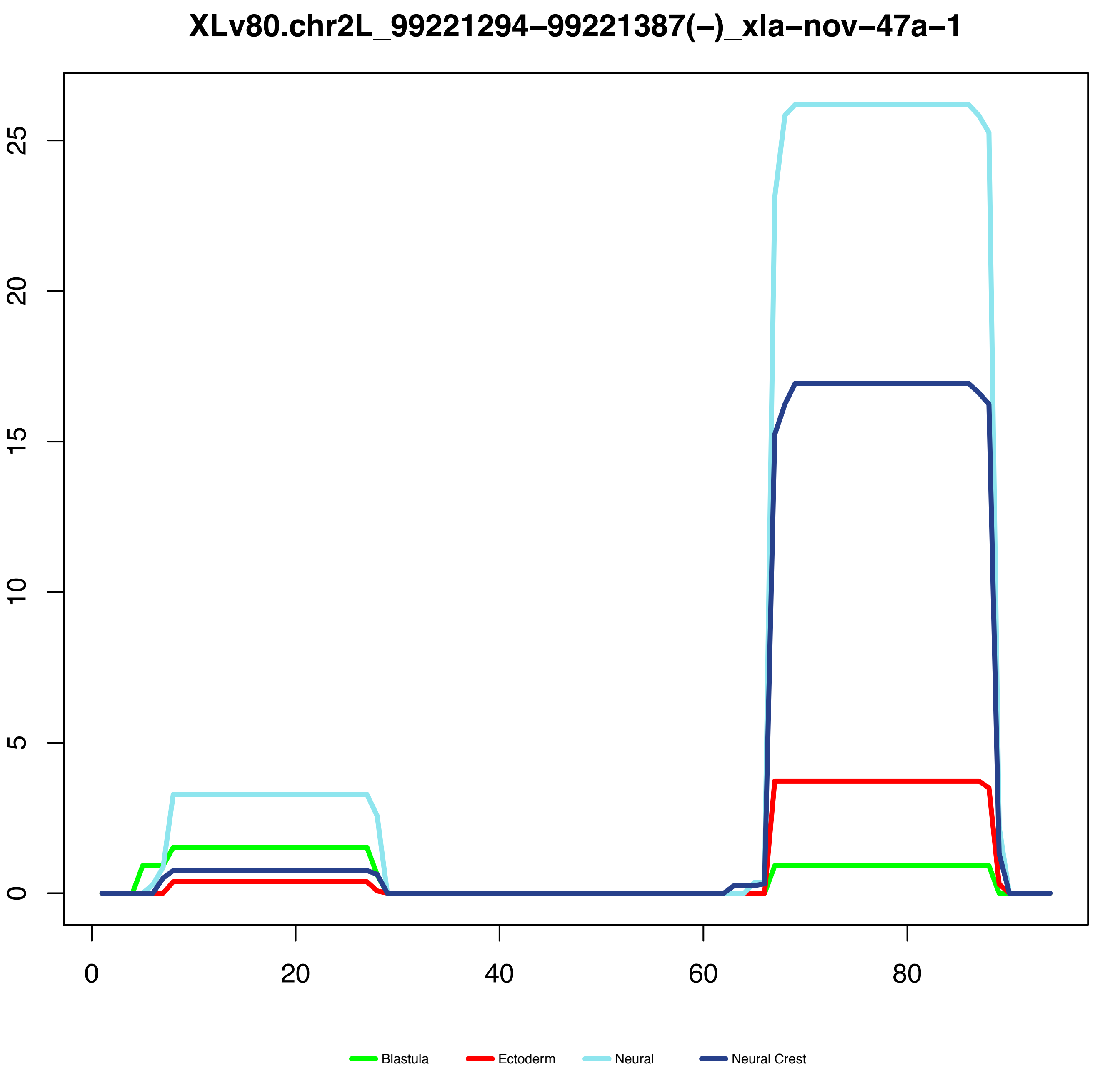
XLv80.chr2L_99221294−99221387(−)_xla−nov−47a−1
0
5
10
15
20
25
0
20
40
60
80
Blastula
Ectoderm
Neural
Neural Crest

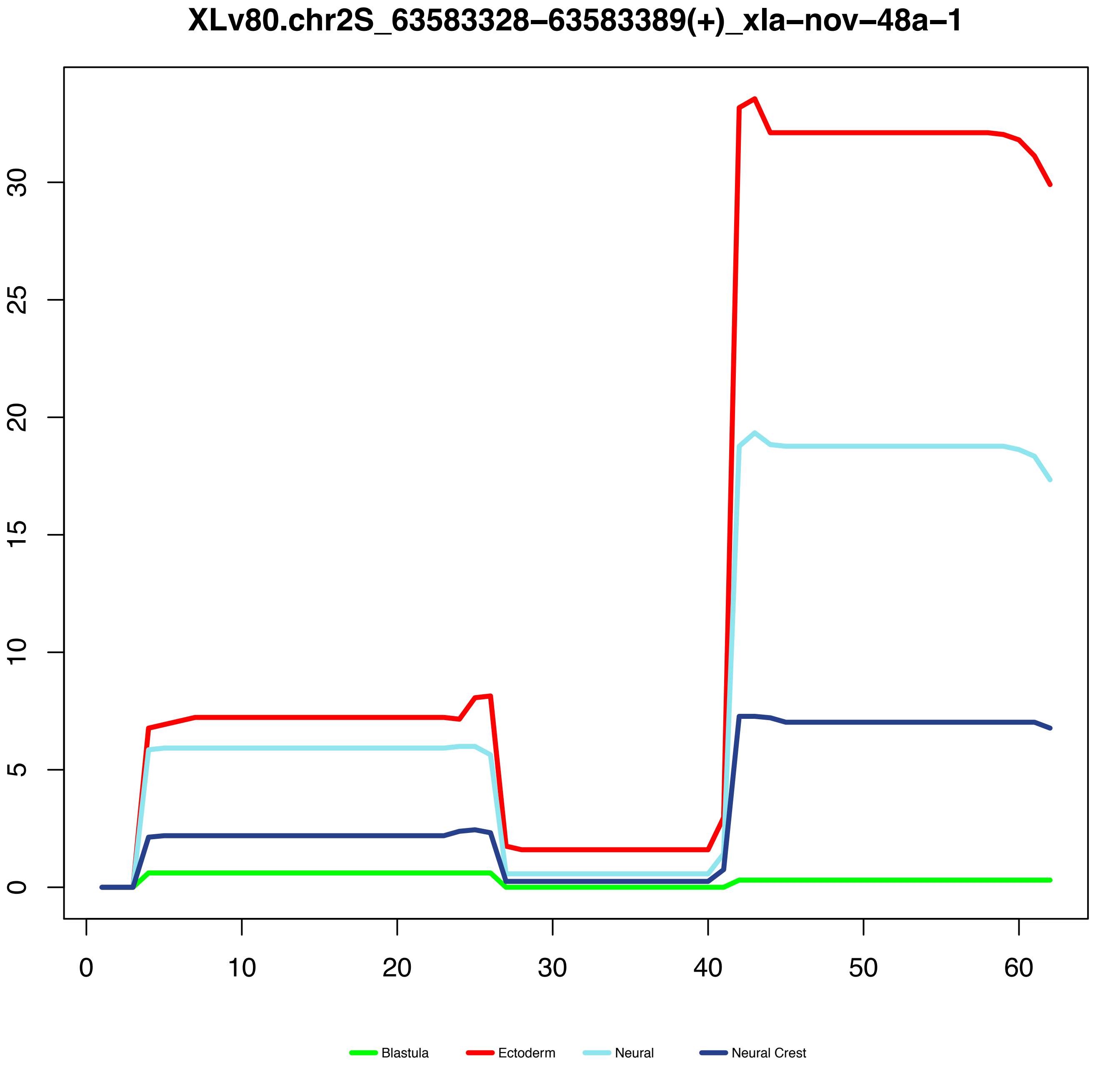
XLv80.chr2S_63583328−63583389(+)_xla−nov−48a−1
0
5
10
15
20
25
30
0
10
20
30
40
50
60
Blastula
Ectoderm
Neural
Neural Crest

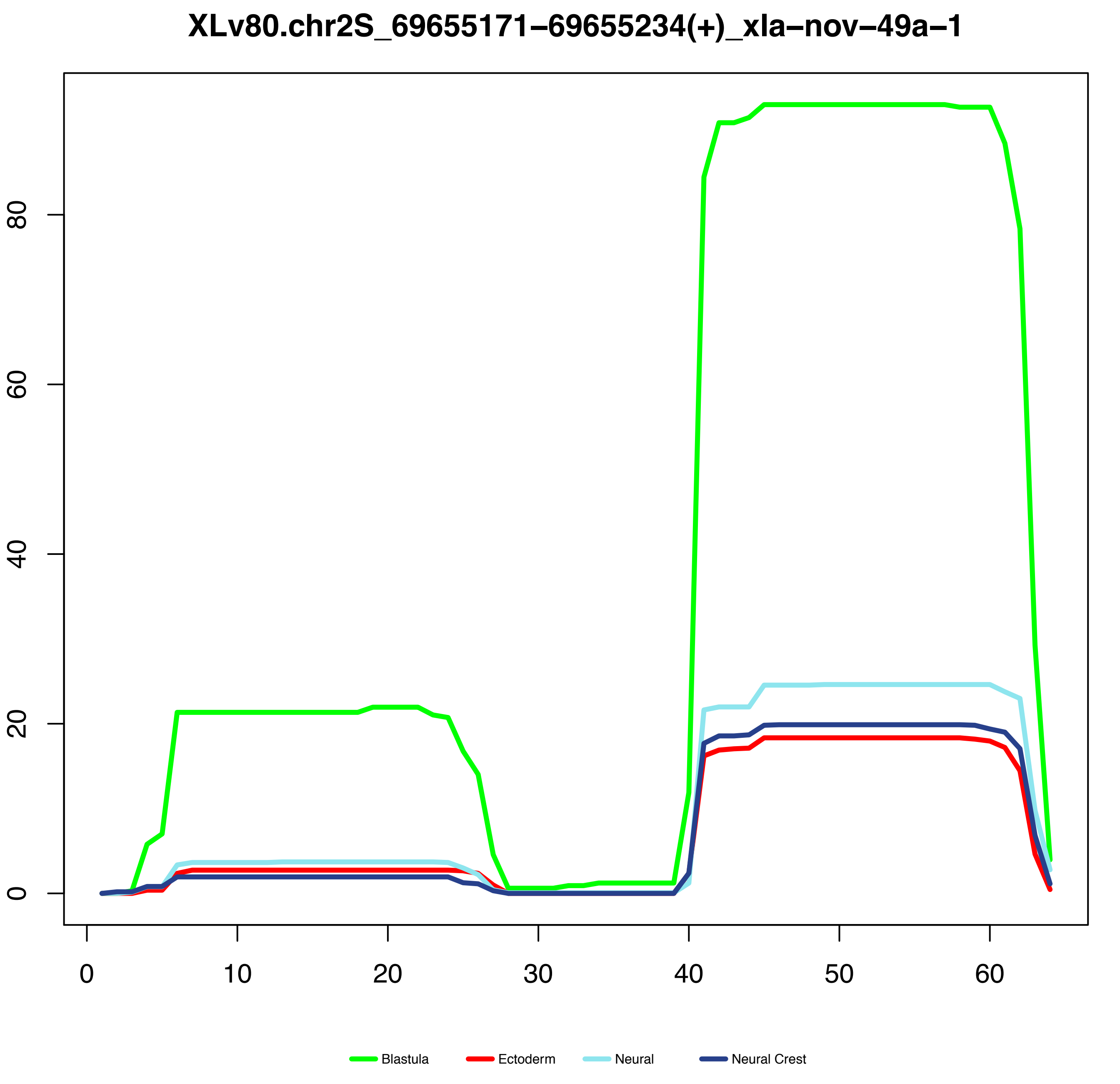

XLv80.chr2S_69655171−69655234(+)_xla−nov−49a−1
0
20
40
60
80
0
10
20
30
40
50
60
Blastula
Ectoderm
Neural
Neural Crest

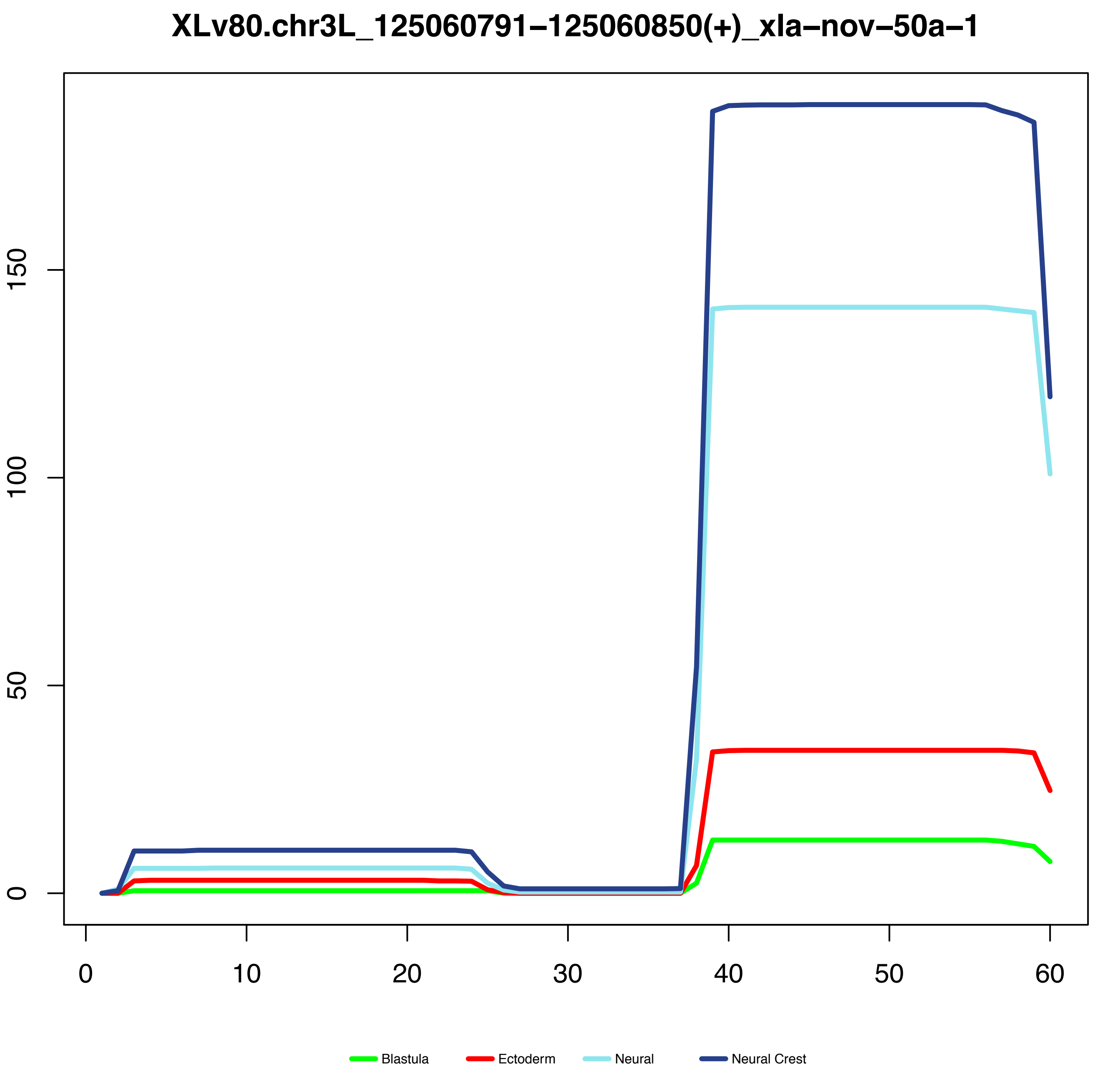
XLv80.chr3L_125060791−125060850(+)_xla−nov−50a−1
0
50
100
150
0
10
20
30
40
50
60
Blastula
Ectoderm
Neural
Neural Crest

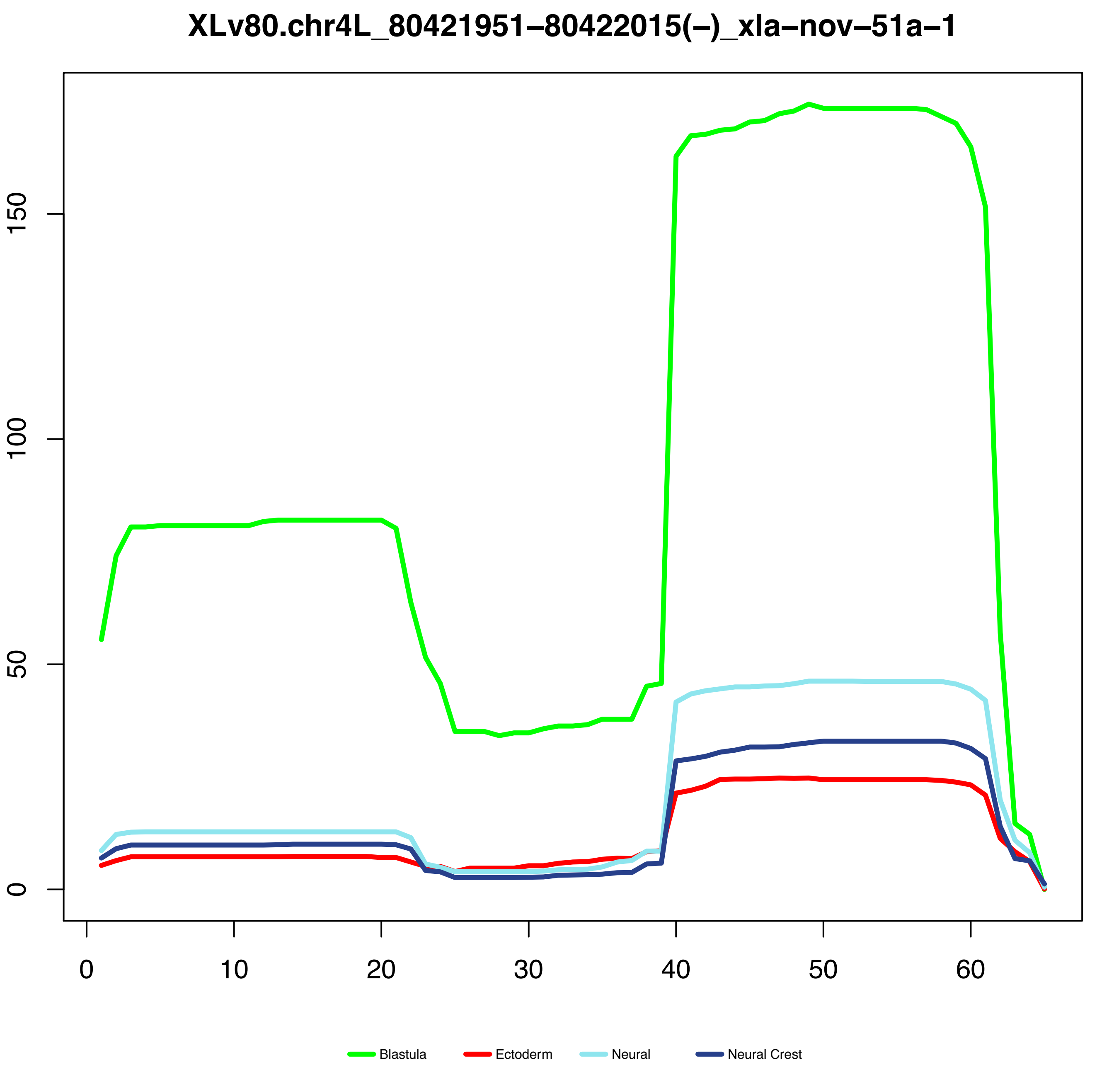
XLv80.chr4L_80421951−80422015(−)_xla−nov−51a−1
0
50
100
150
0
10
20
30
40
50
60
Blastula
Ectoderm
Neural
Neural Crest

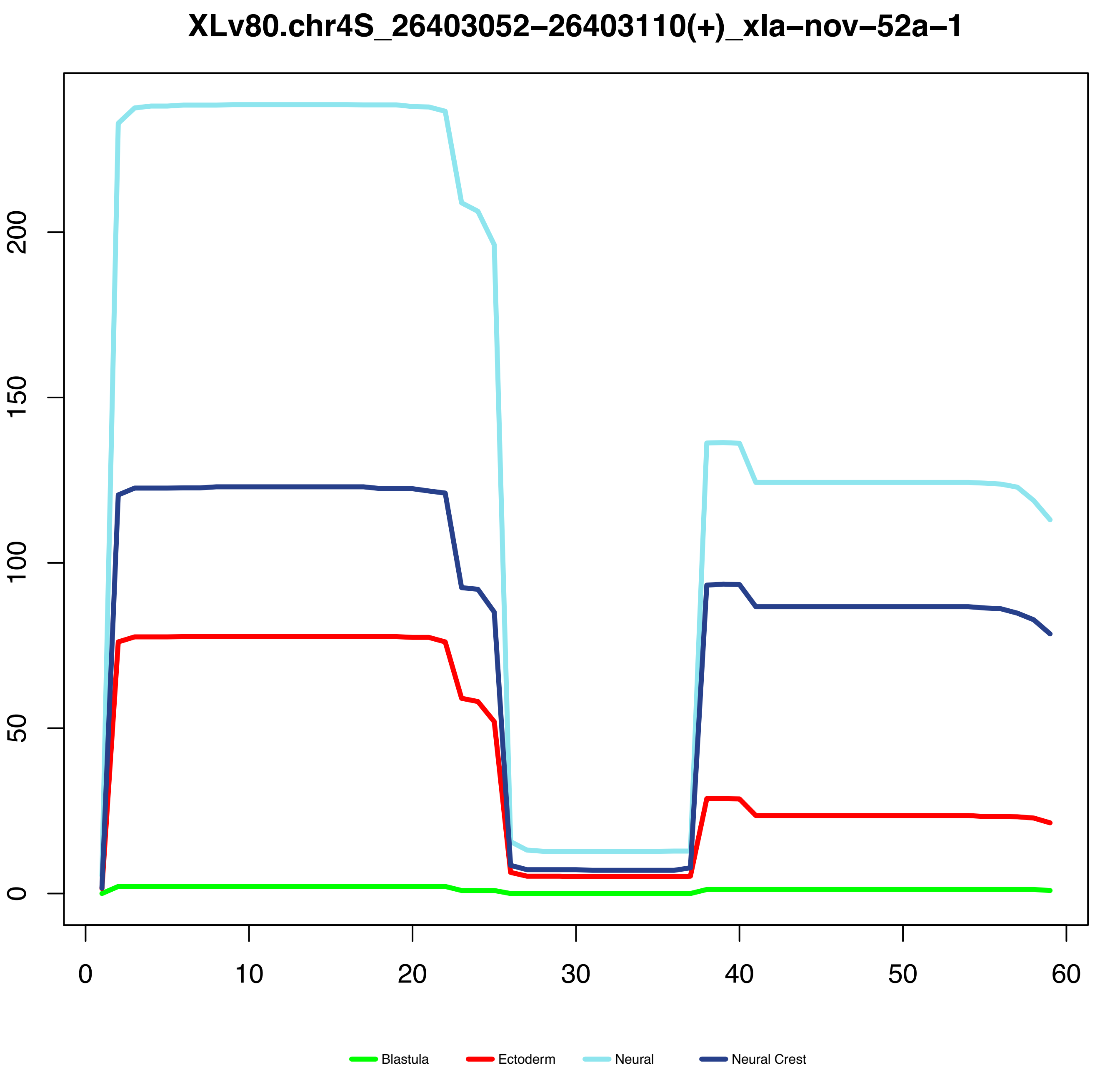
XLv80.chr4S_26403052−26403110(+)_xla−nov−52a−1
0
50
100
150
200
0
10
20
30
40
50
60
Blastula
Ectoderm
Neural
Neural Crest

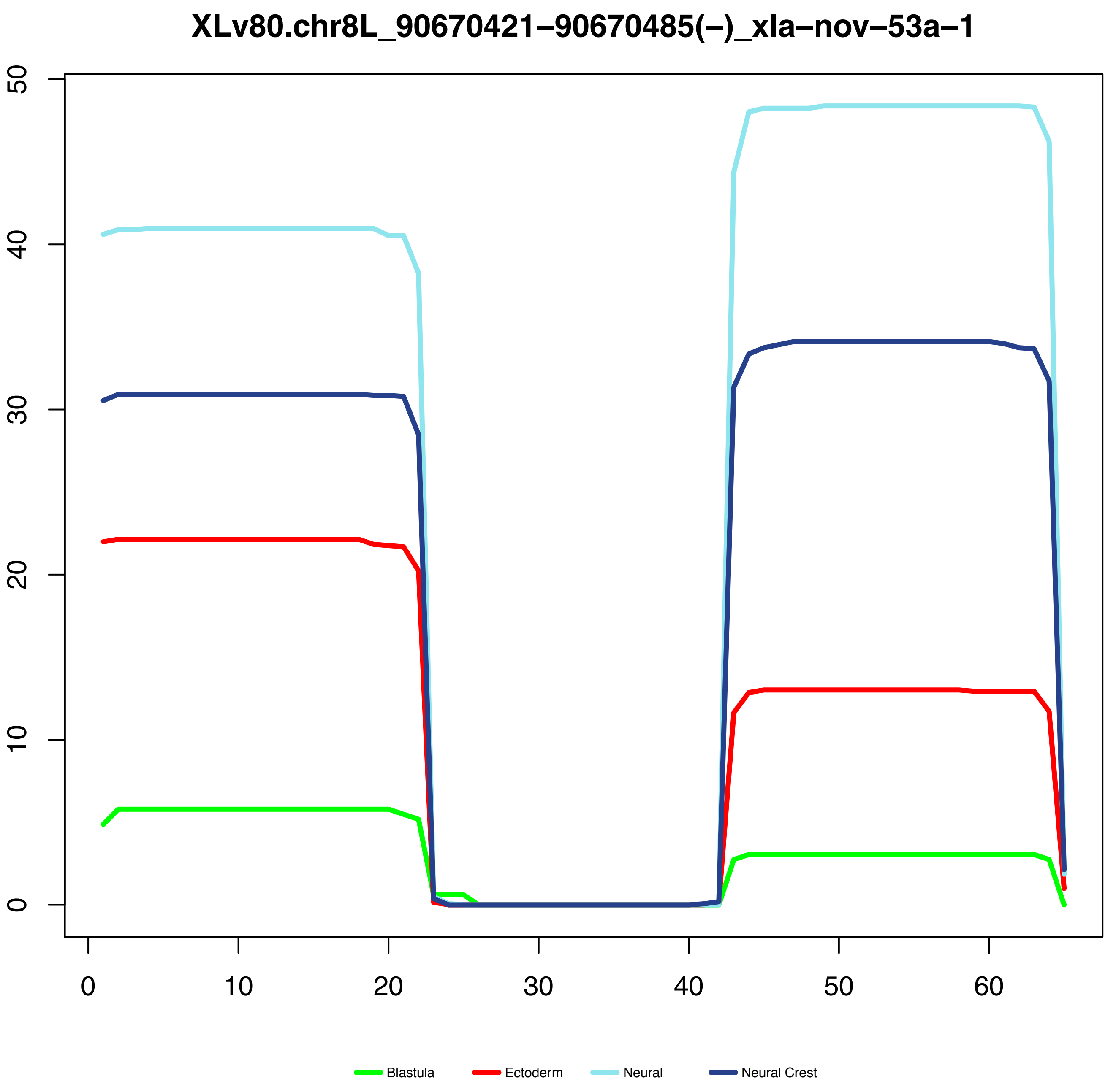
XLv80.chr8L_90670421−90670485(−)_xla−nov−53a−1
0
10
20
30
40
50
0
10
20
30
40
50
60
Blastula
Ectoderm
Neural
Neural Crest

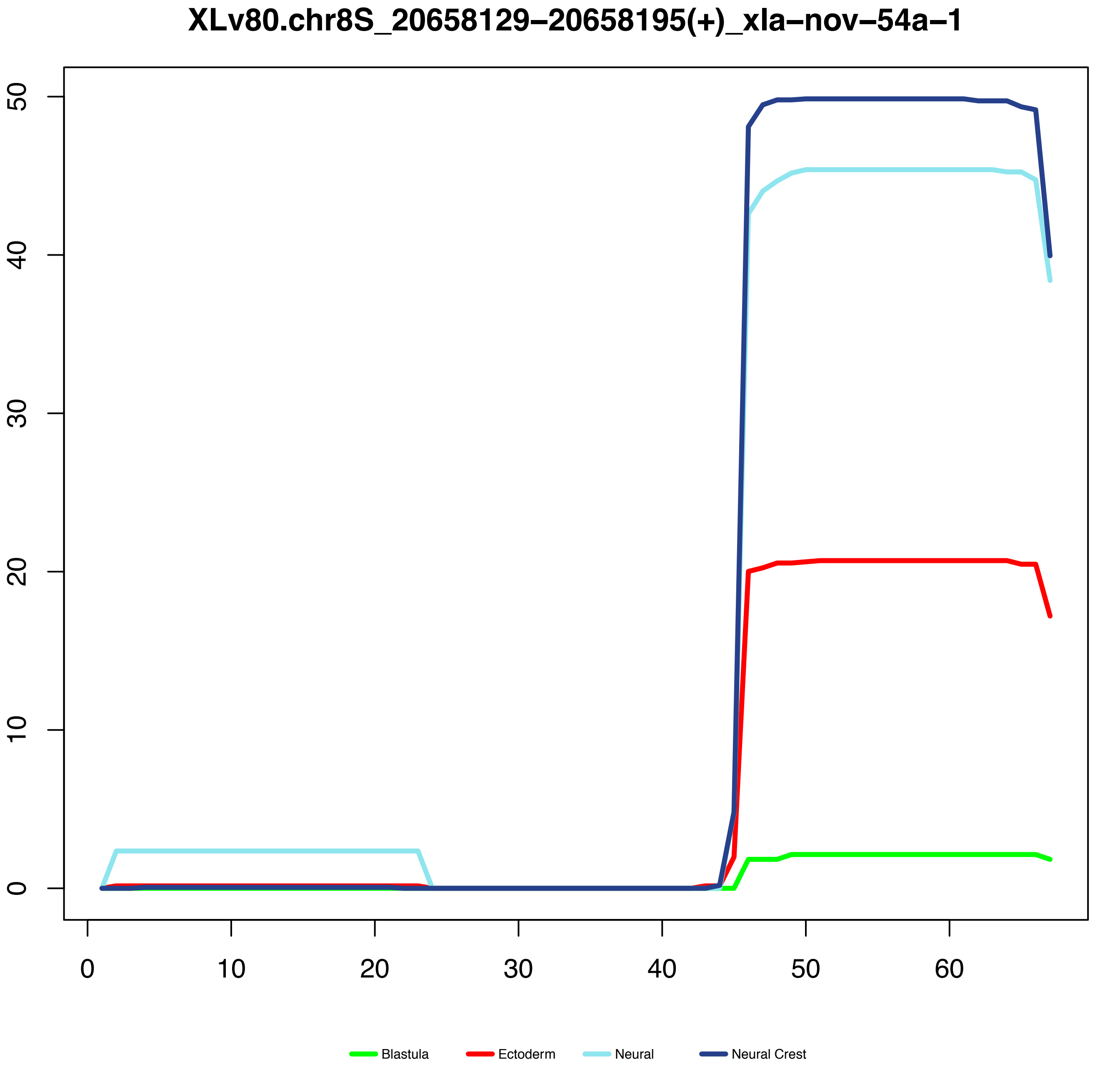

XLv80.chr8S_20658129−20658195(+)_xla−nov−54a−1
0
10
20
30
40
50
0
10
20
30
40
50
60
Blastula
Ectoderm
Neural
Neural Crest

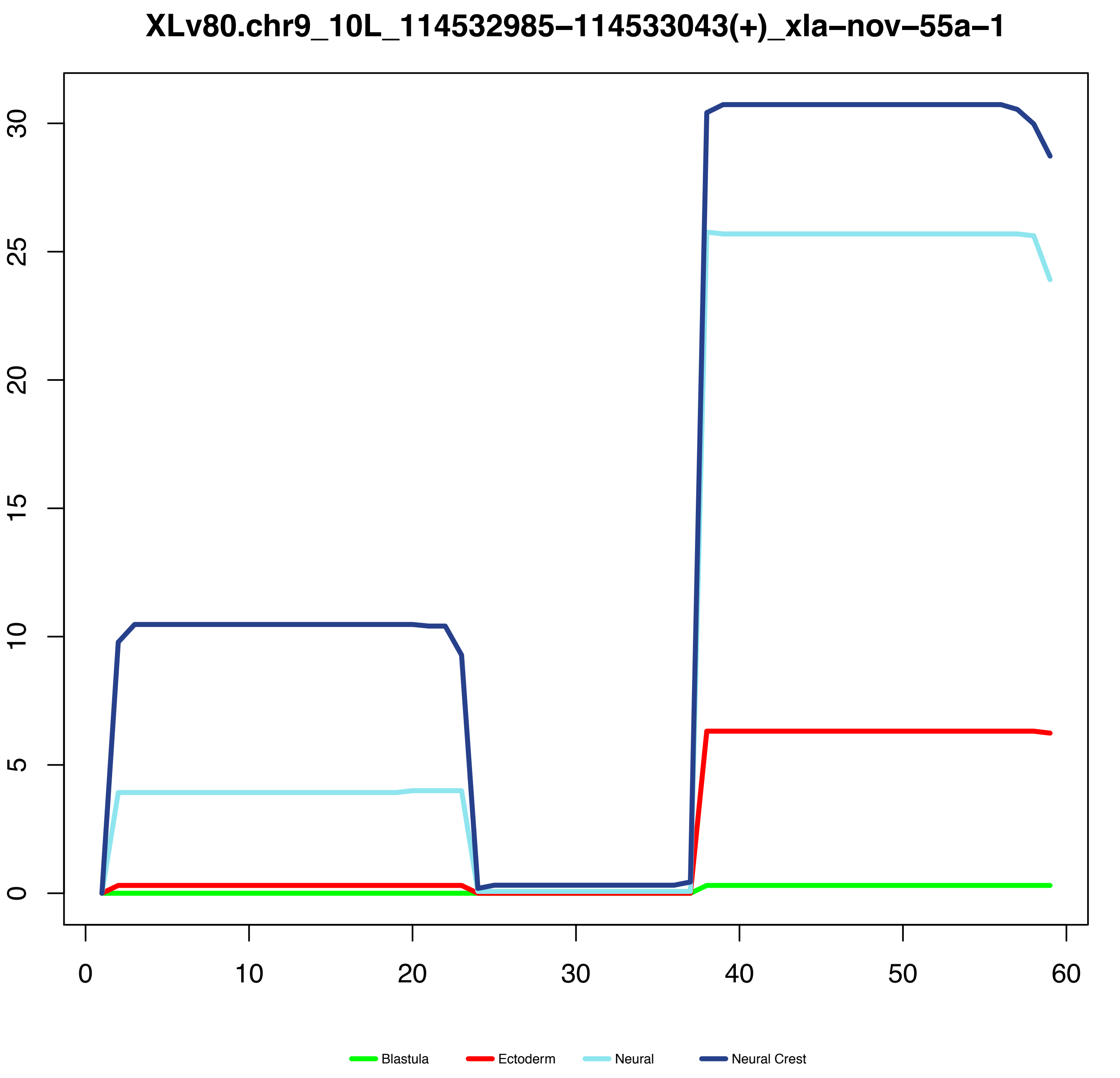

XLv80.chr9_10L_114532985−114533043(+)_xla−nov−55a−1
0
5
10
15
20
25
30
0
10
20
30
40
50
60
Blastula
Ectoderm
Neural
Neural Crest

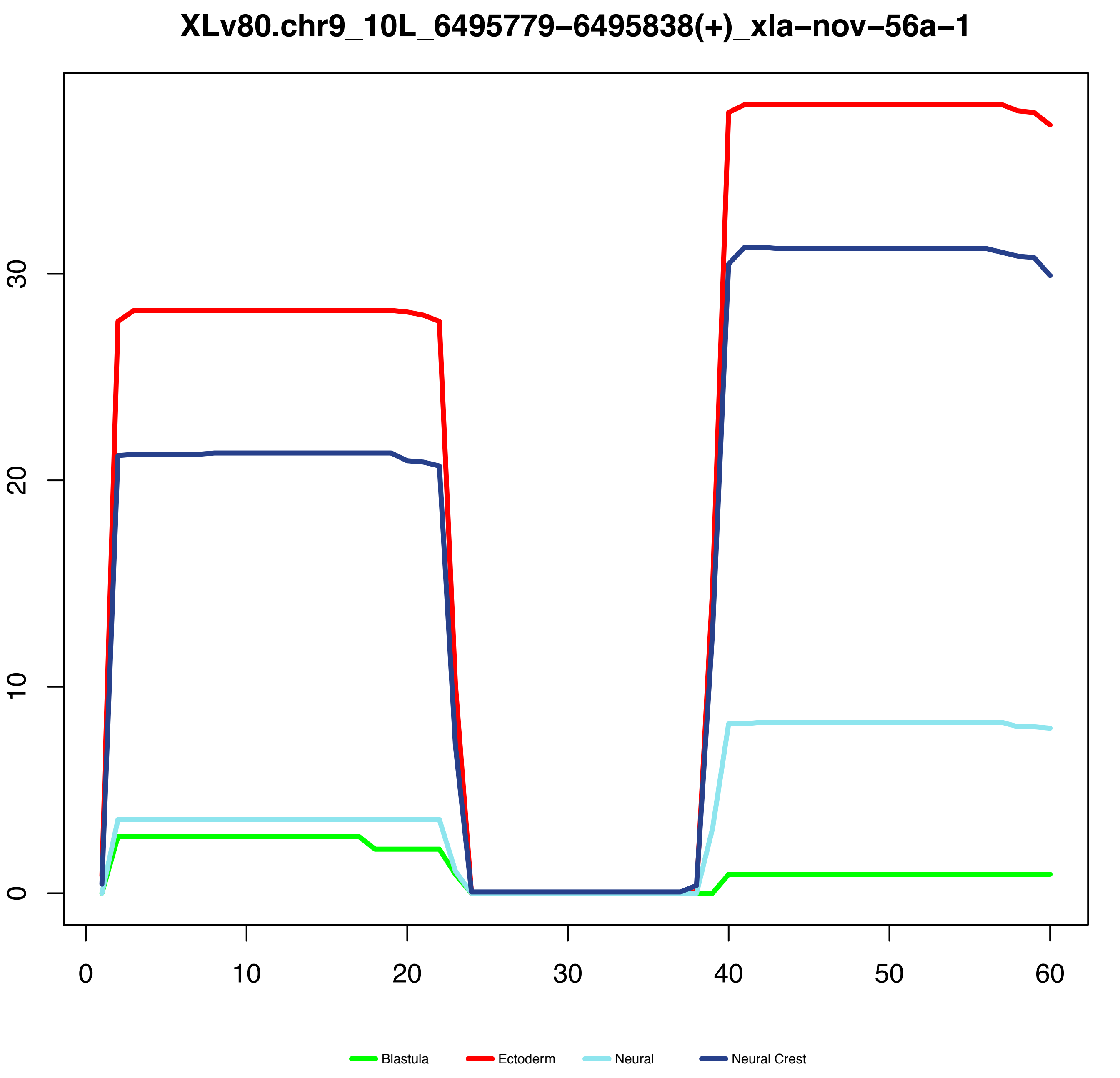
XLv80.chr9_10L_6495779−6495838(+)_xla−nov−56a−1
0
10
20
30
0
10
20
30
40
50
60
Blastula
Ectoderm
Neural
Neural Crest

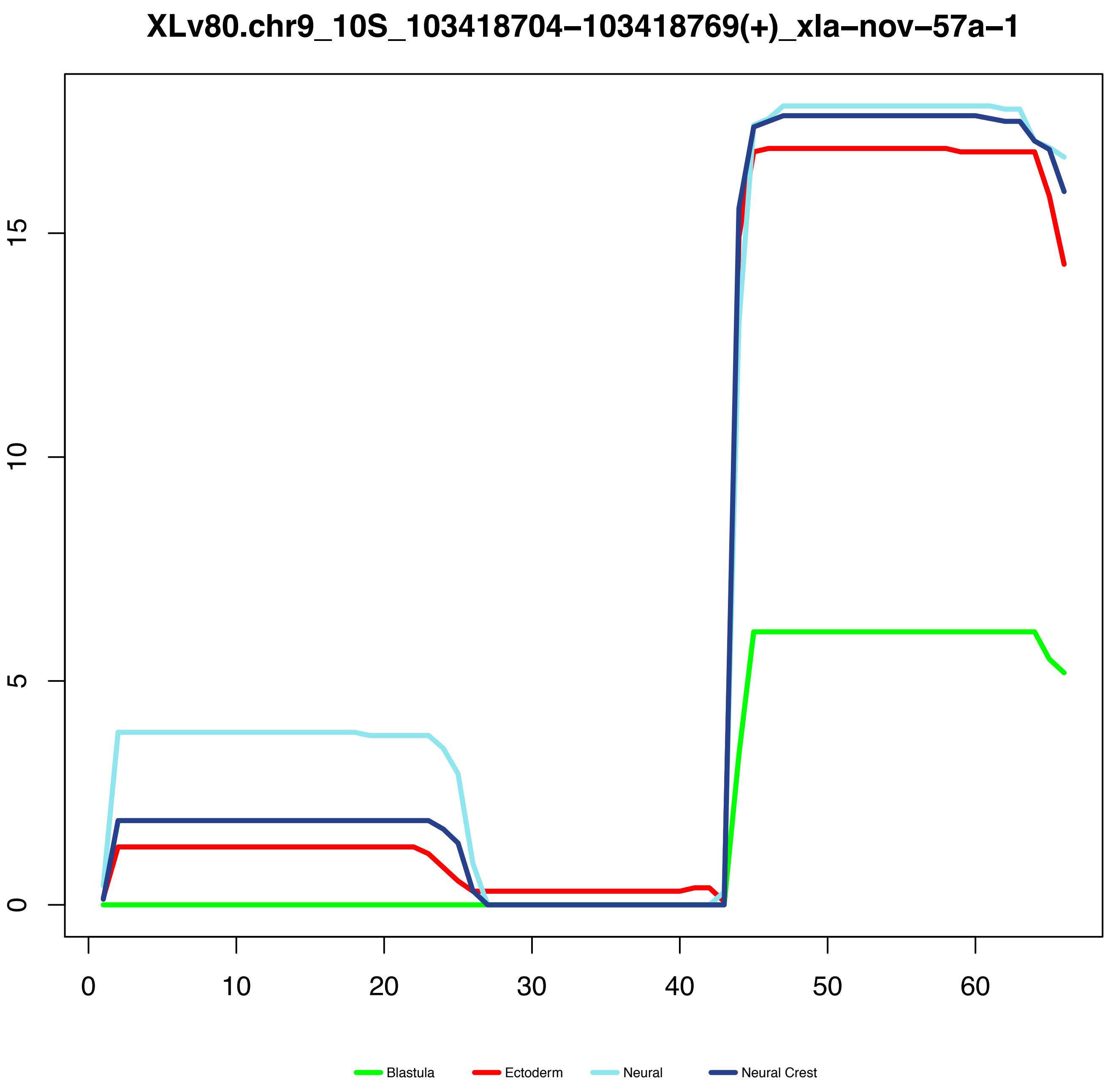
XLv80.chr9_10S_103418704−103418769(+)_xla−nov−57a−1
0
5
10
15
0
10
20
30
40
50
60
Blastula
Ectoderm
Neural
Neural Crest

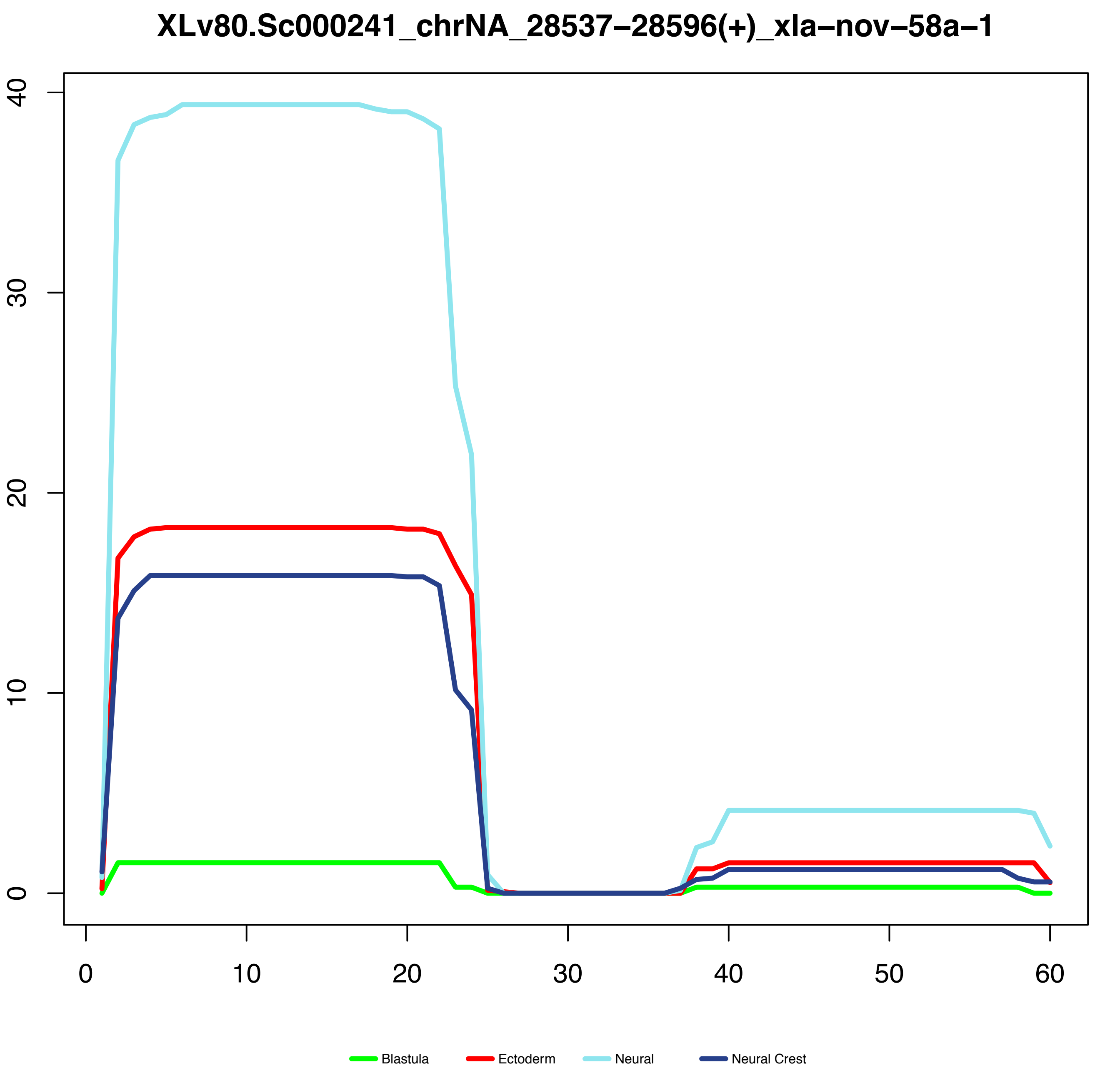
XLv80.Sc000241_chrNA_28537−28596(+)_xla−nov−58a−1
0
10
20
30
40
0
10
20
30
40
50
60
Blastula
Ectoderm
Neural
Neural Crest

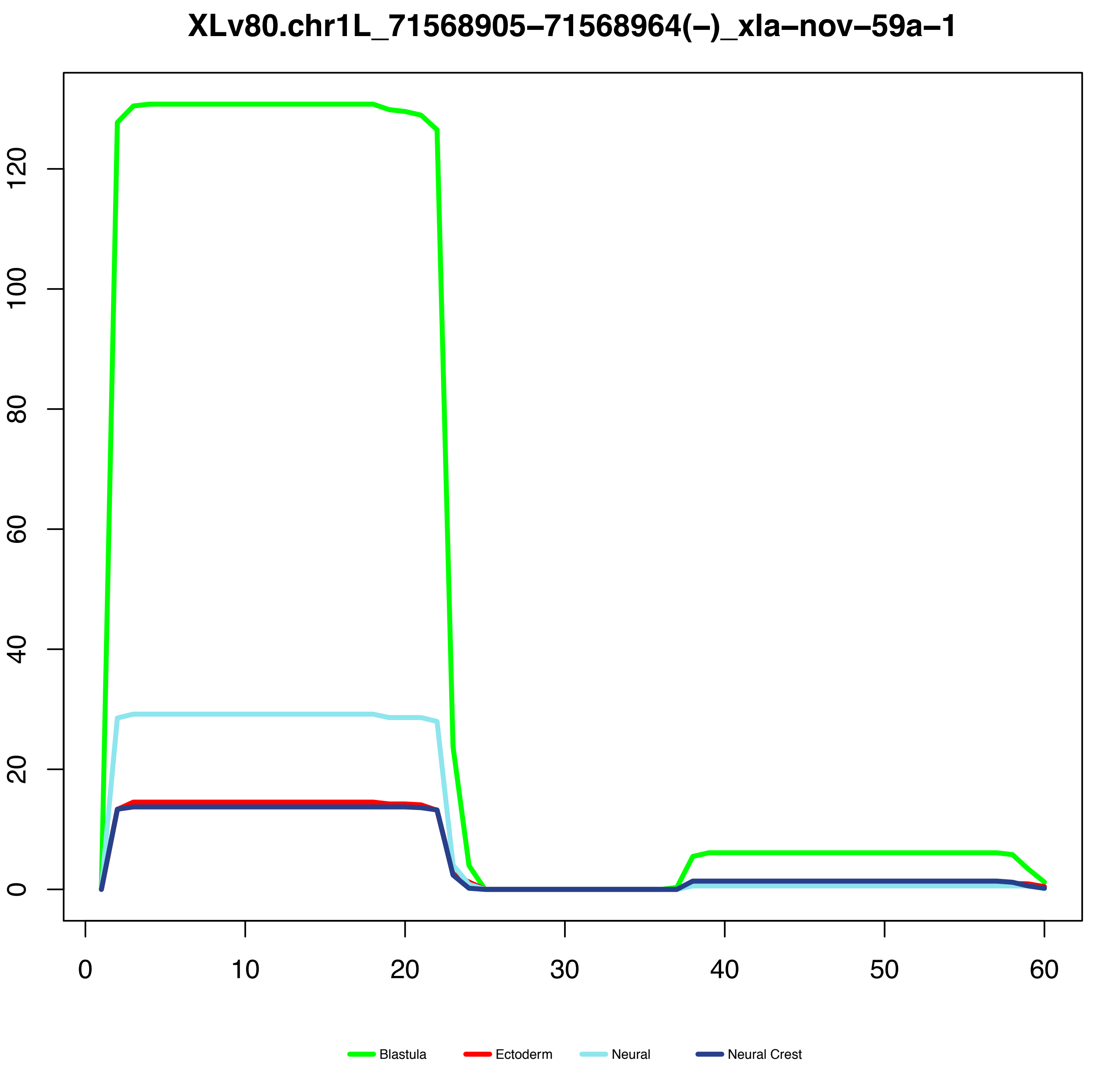
XLv80.chr1L_71568905−71568964(−)_xla−nov−59a−1
0
20
40
60
80
100
120
0
10
20
30
40
50
60
Blastula
Ectoderm
Neural
Neural Crest

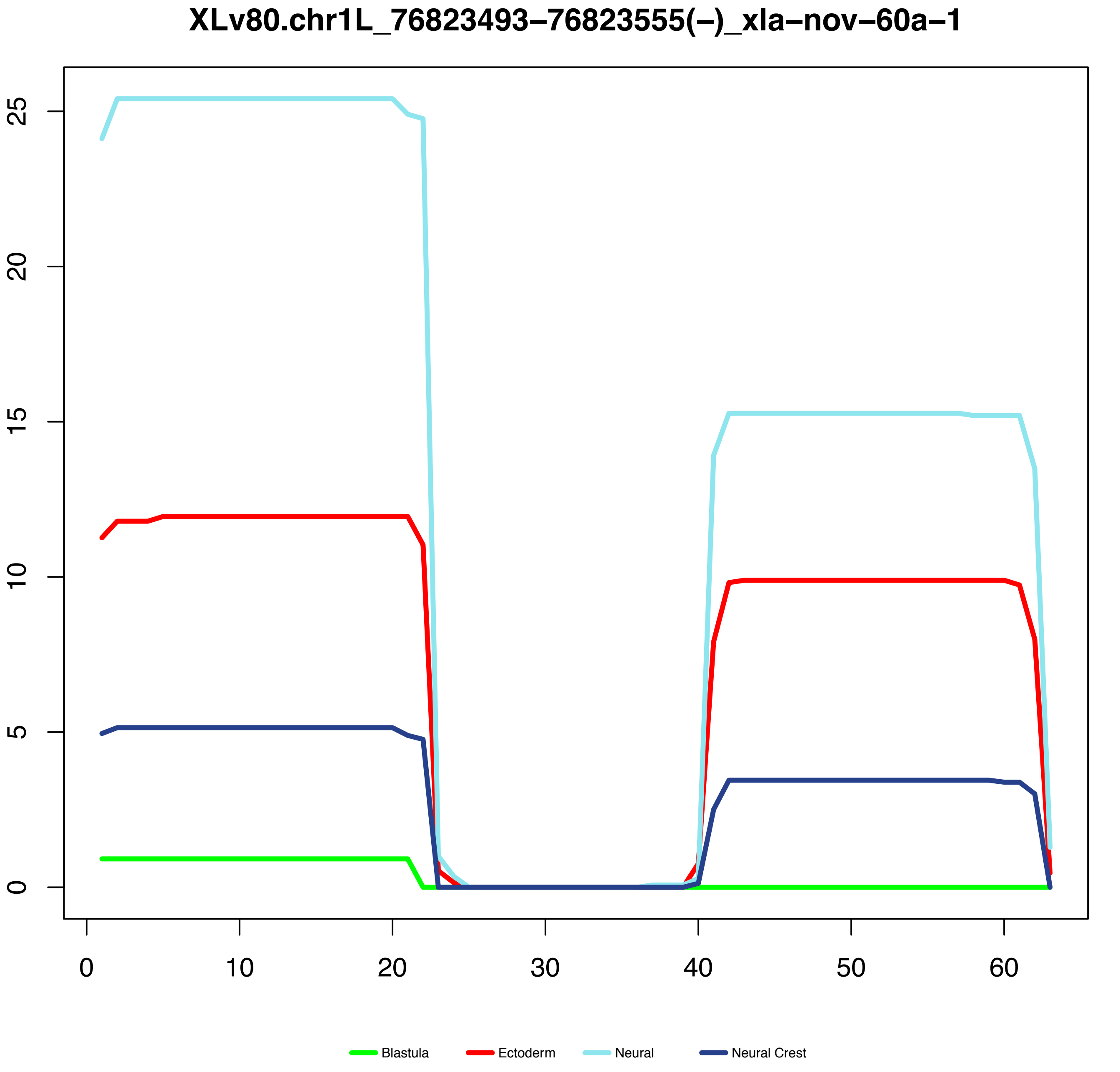
XLv80.chr1L_76823493−76823555(−)_xla−nov−60a−1
0
5
10
15
20
25
0
10
20
30
40
50
60
Blastula
Ectoderm
Neural
Neural Crest

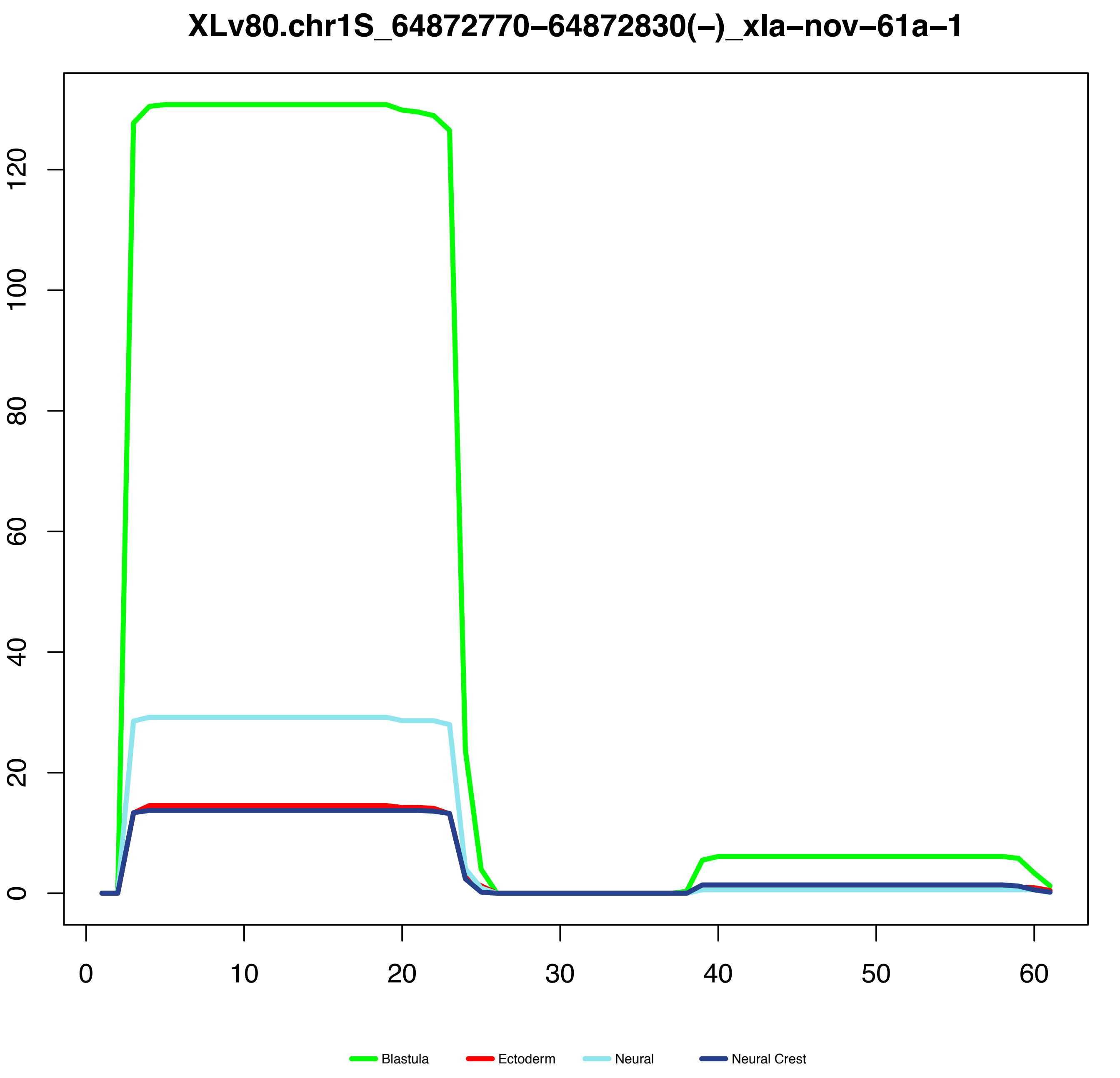
XLv80.chr1S_64872770–64872830(–)_xla–nov–61a–1
0
20
40
60
80
100
120
0
10
20
30
40
50
60
Blastula
Ectoderm
Neural
Neural Crest

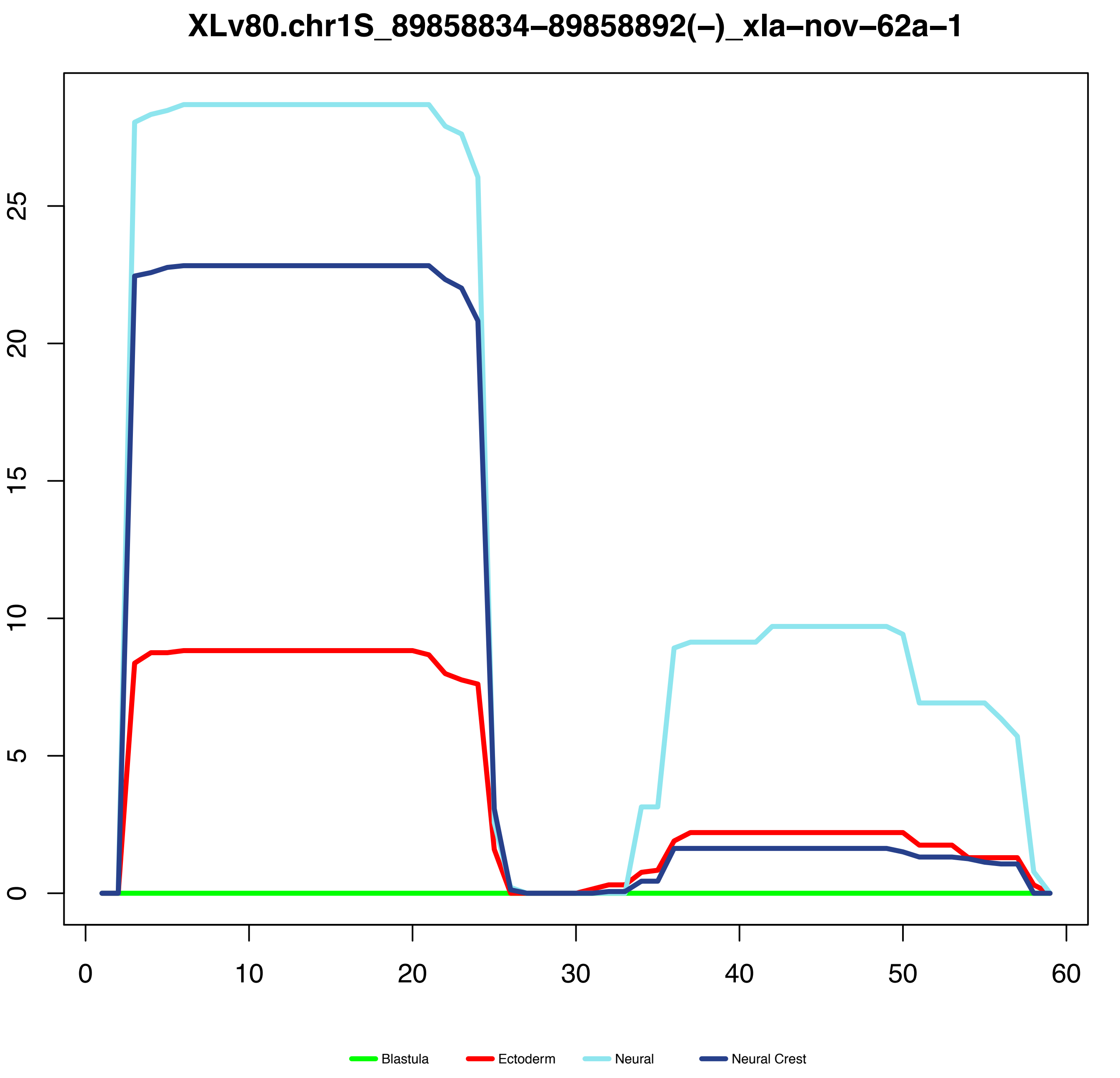

XLv80.chr1S_89858834−89858892(−)_xla−nov−62a−1
0
5
10
15
20
25
0
10
20
30
40
50
60
Blastula
Ectoderm
Neural
Neural Crest

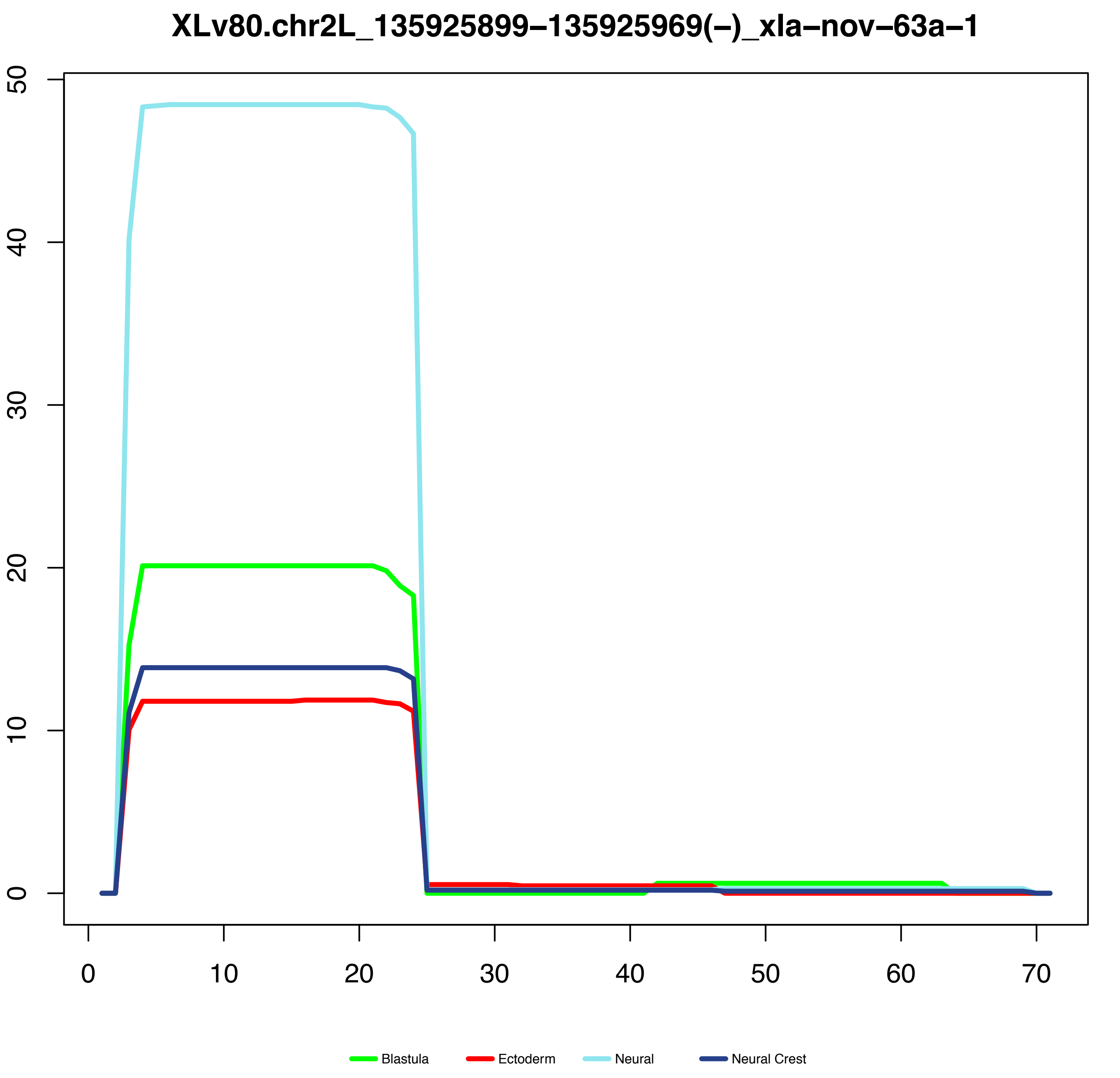

XLv80.chr2L_135925899−135925969(−)_xla−nov−63a−1
0
10
20
30
40
50
0
10
20
30
40
50
60
70
Blastula
Ectoderm
Neural
Neural Crest

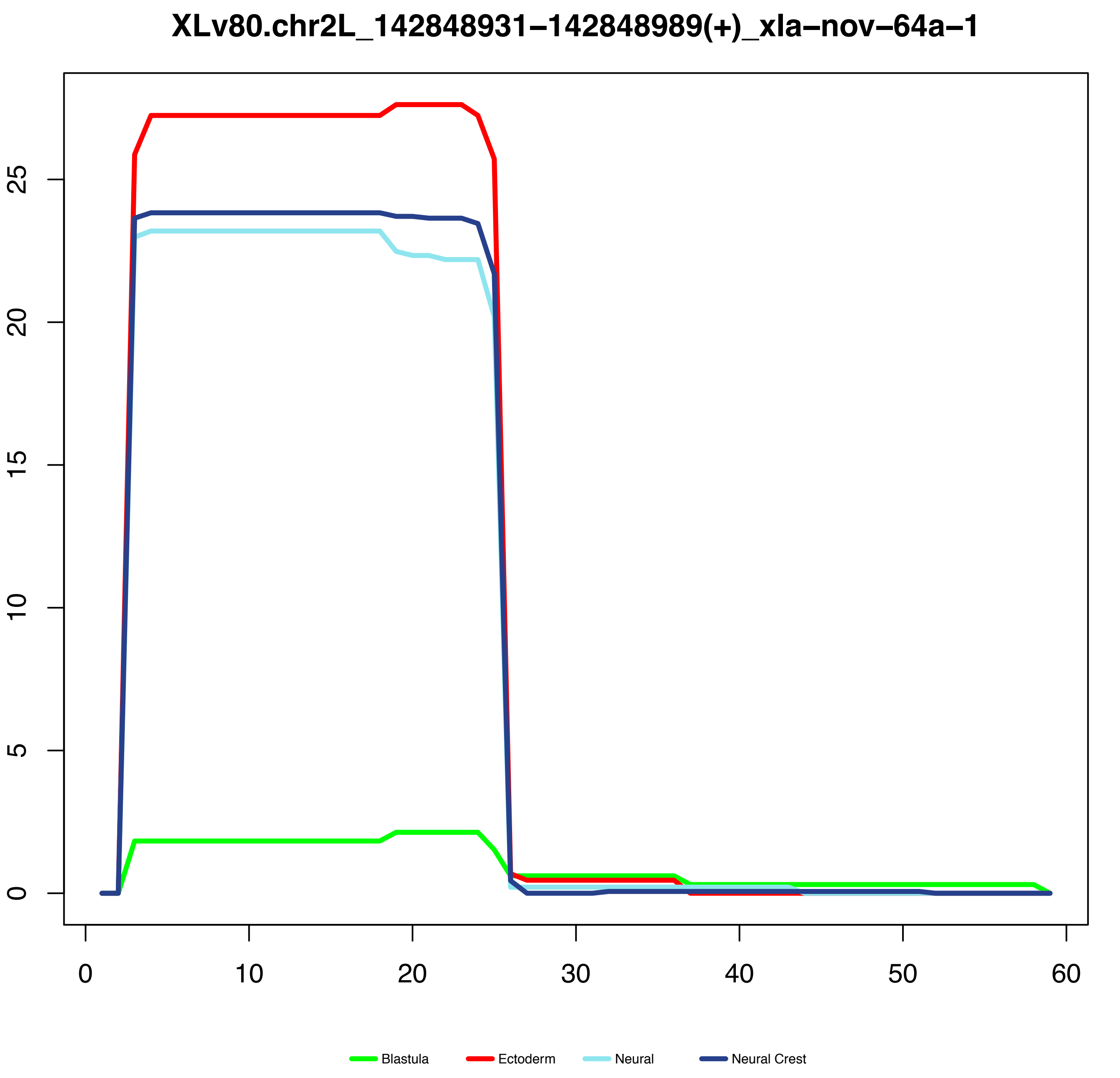
XLv80.chr2L_142848931−142848989(+)_xla−nov−64a−1
0
5
10
15
20
25
0
10
20
30
40
50
60
Blastula
Ectoderm
Neural
Neural Crest

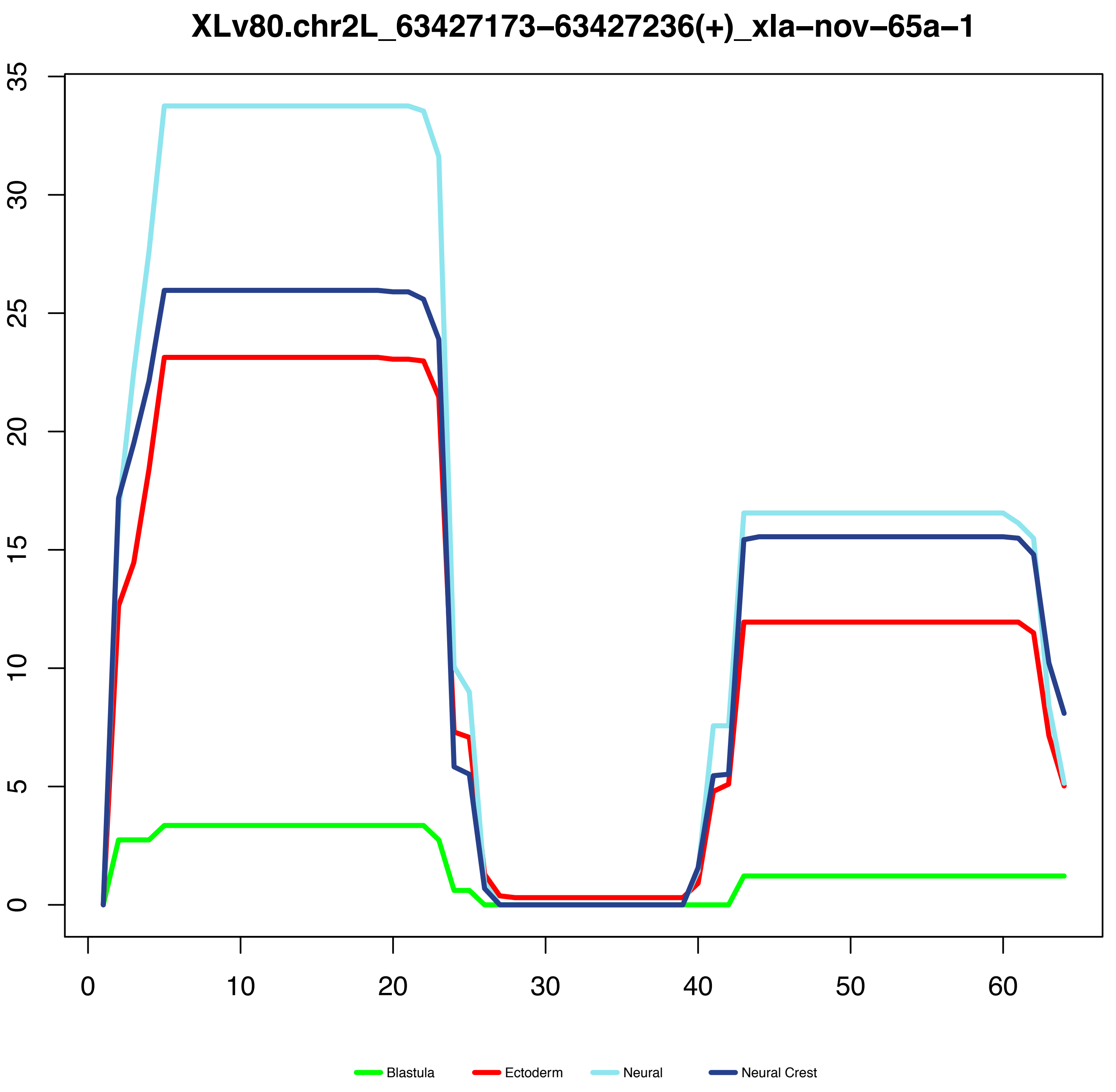
XLv80.chr2L_63427173−63427236(+)_xla−nov−65a−1
0
5
10
15
20
25
30
35
0
10
20
30
40
50
60
Blastula
Ectoderm
Neural
Neural Crest

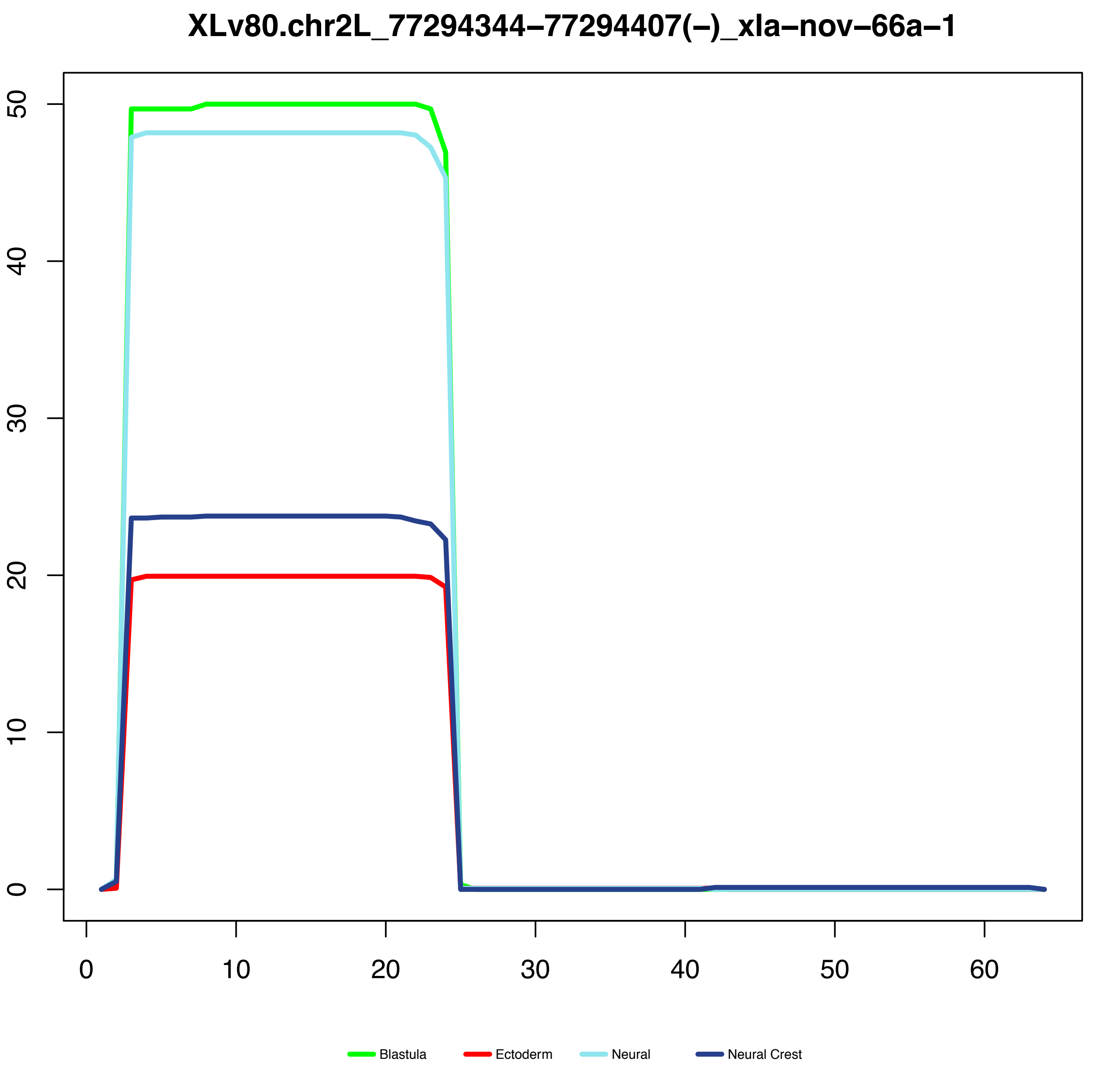

XLv80.chr2L_77294344−77294407(−)_xla−nov−66a−1
0
10
20
30
40
50
0
10
20
30
40
50
60
Blastula
Ectoderm
Neural
Neural Crest

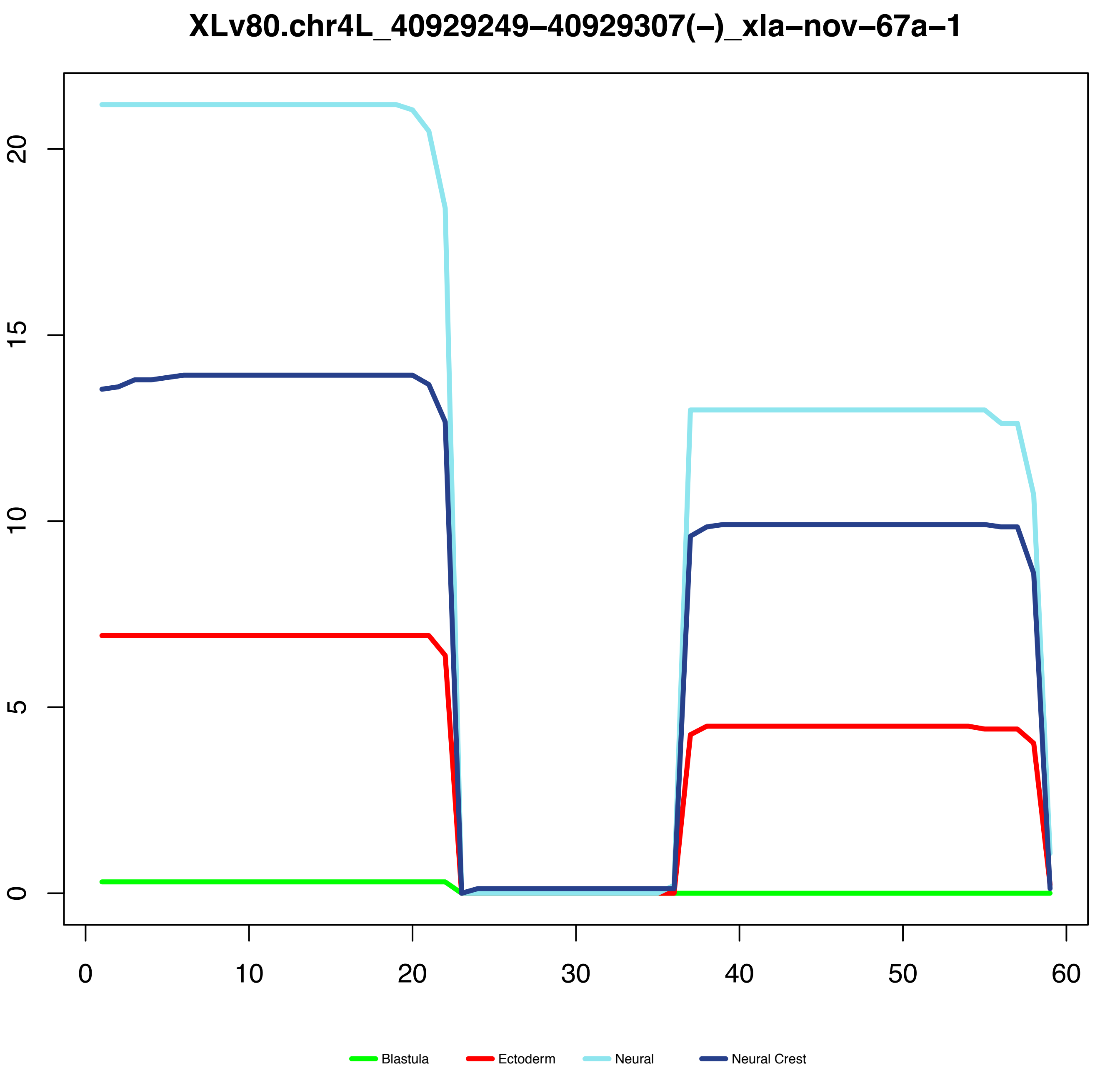
XLv80.chr4L_40929249−40929307(−)_xla−nov−67a−1
0
5
10
15
20
0
10
20
30
40
50
60
Blastula
Ectoderm
Neural
Neural Crest

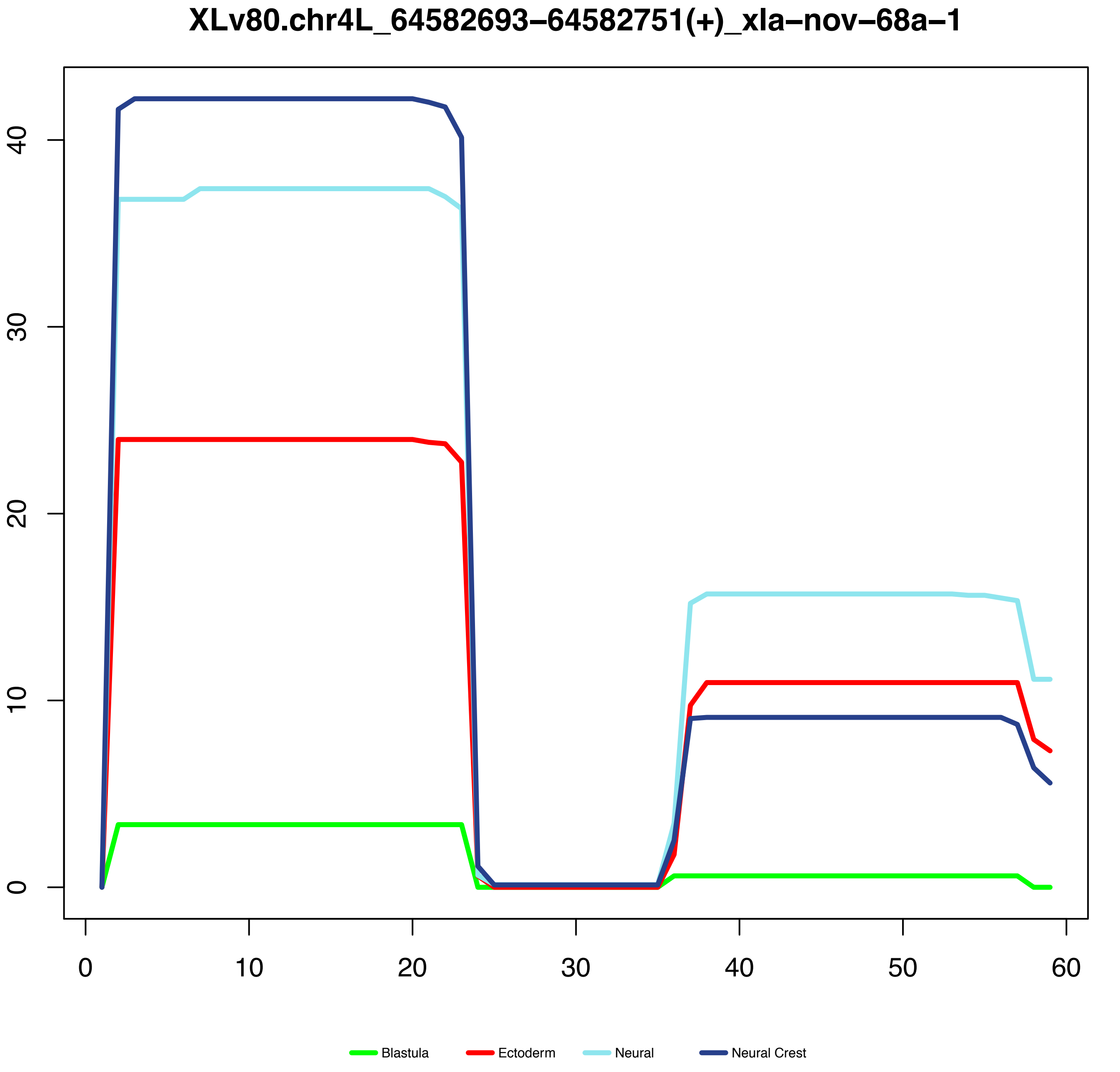
XLv80.chr4L_64582693−64582751(+)_xla−nov−68a−1
0
10
20
30
40
0
10
20
30
40
50
60
Blastula
Ectoderm
Neural
Neural Crest

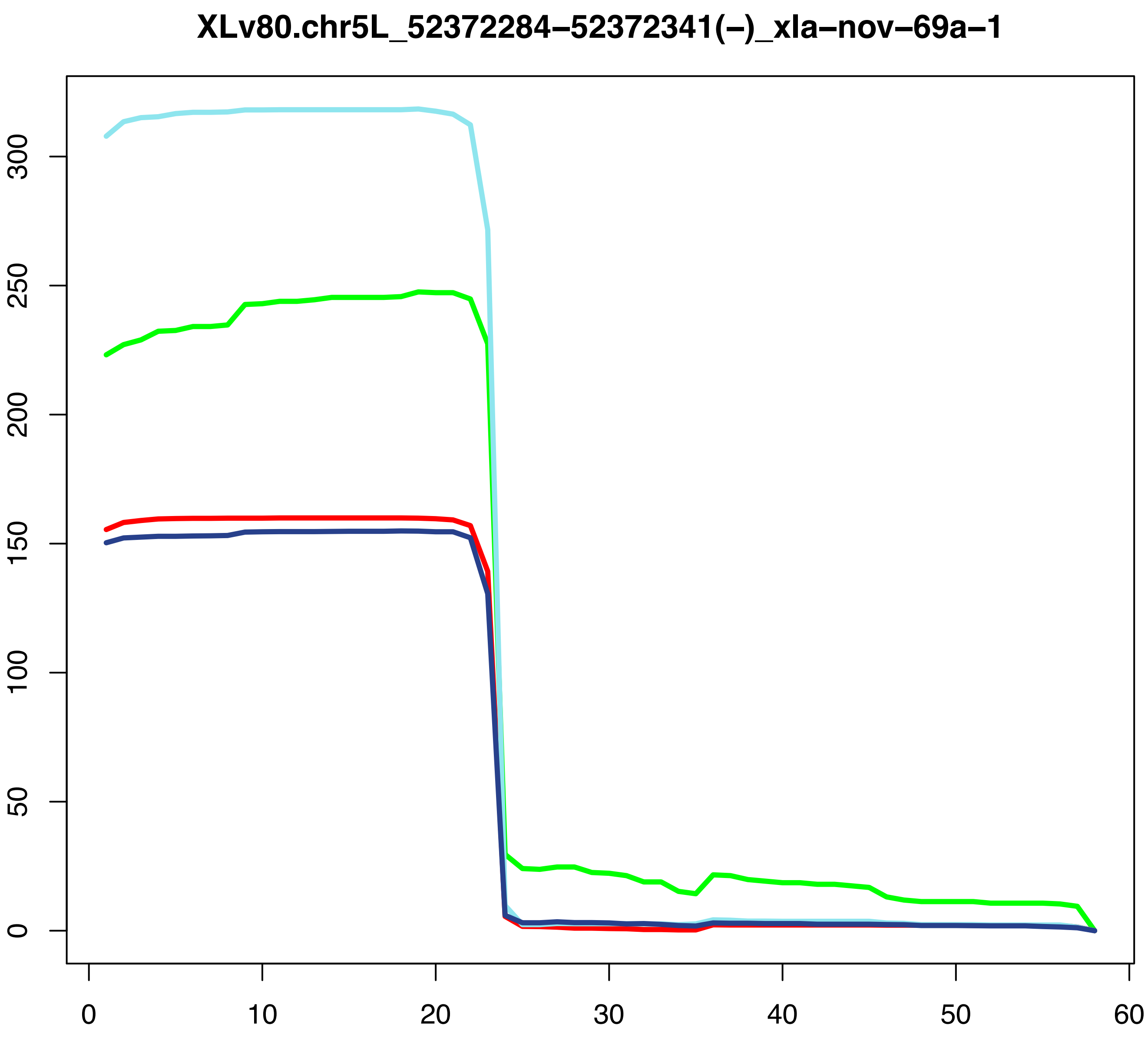
XLv80.chr5L_52372284–52372341(–)_xla–nov–69a–1
0
50
100
150
200
250
300
0
10
20
30
40
50
60
