# Supplementary material for: microRNAs associated with early neural crest development in Xenopus laevis
Source: BMC Genomics. 2018 Jan 18;19:59. doi: 10.1186/s12864-018-4436-0 (PMC5774138; doi:10.1186/s12864-018-4436-0)
Supplement: Supplementary file 7 — List of sequenced miRNAs that were not previously annotated in Xenopus laevis. (DOCX 48 kb) [file 12864_2018_4436_MOESM7_ESM.docx]

| **Additional file 7: Table S3: List of sequenced miRNAs that were not previously annotated in *Xenopus laevis***   \|  \| **miRNA** \| \| --- \| --- \| \| 1 \| miR-21 \| \| 2 \| miR-32 \| \| 3 \| miR-141 \| \| 4 \| miR-352 \| \| 5 \| miR-454 \| \| 6 \| miR-456 \| \| 7 \| miR-458 \| \| 8 \| miR-460 \| \| 9 \| miR-802 \| \| 10 \| miR-1329 \| \| 11 \| miR-1388 \| \| 12 \| miR-1662 \| \| 13 \| miR-1788 \| \| 14 \| miR-2985 \| \| 15 \| miR-3618 \| |
| --- | --- | --- | --- | --- | --- | --- | --- | --- | --- | --- | --- | --- | --- | --- | --- | --- | --- | --- | --- | --- | --- | --- | --- | --- | --- | --- | --- | --- | --- | --- | --- | --- |
