# Supplementary figures and images for: microRNAs associated with early neural crest development in Xenopus laevis
Source: BMC Genomics. 2018 Jan 18;19:59. doi: 10.1186/s12864-018-4436-0 (PMC5774138; doi:10.1186/s12864-018-4436-0)

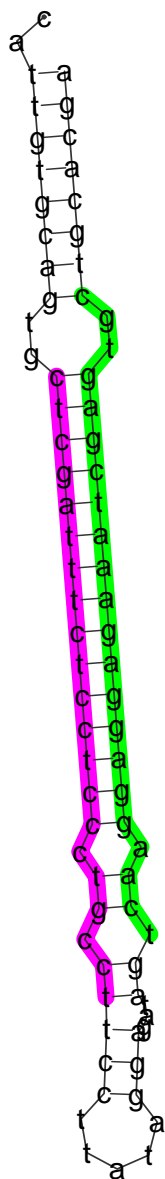

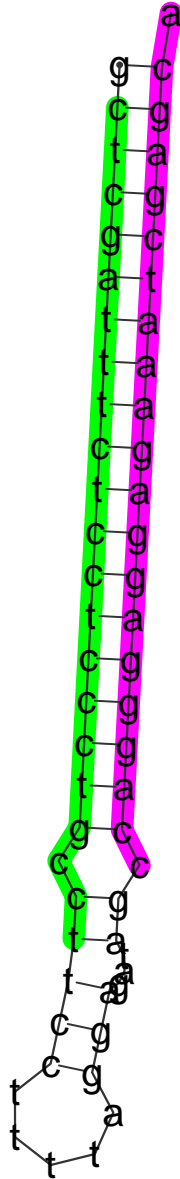

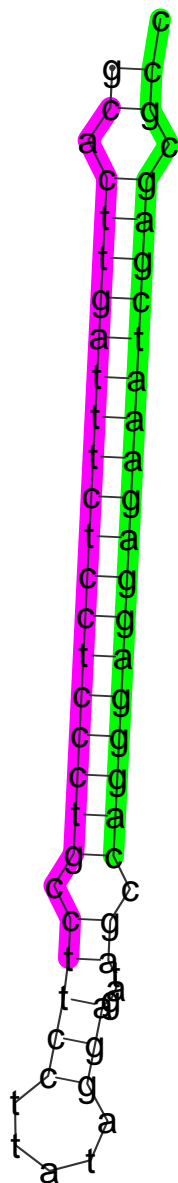

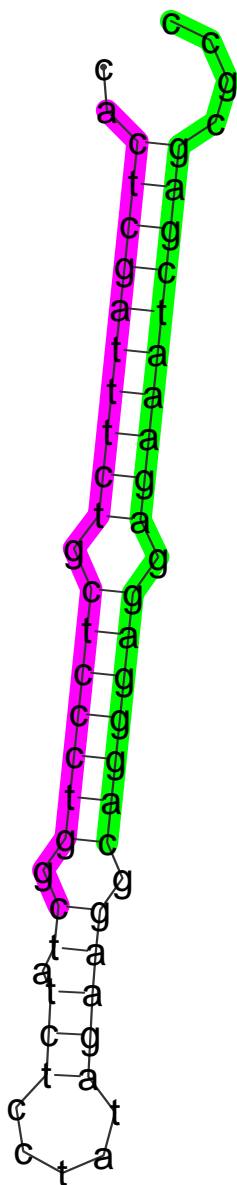

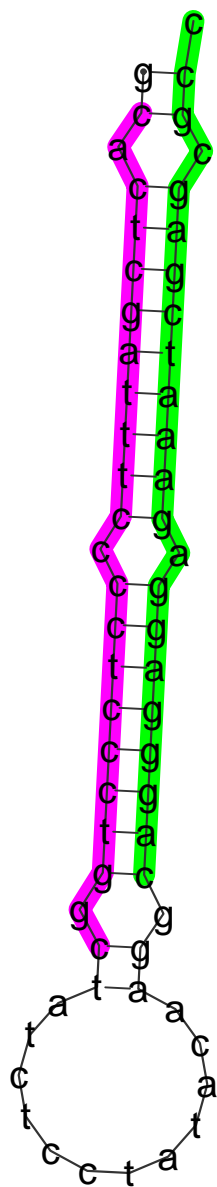

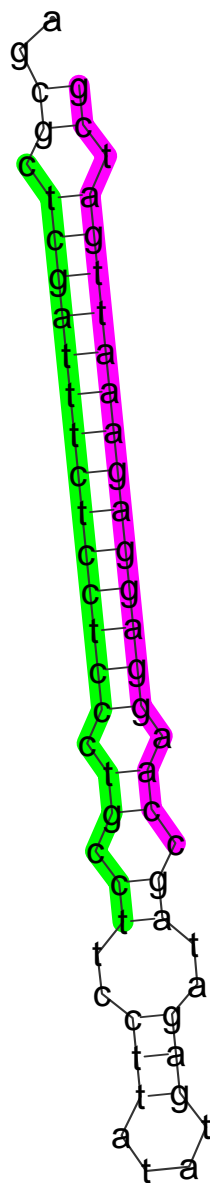

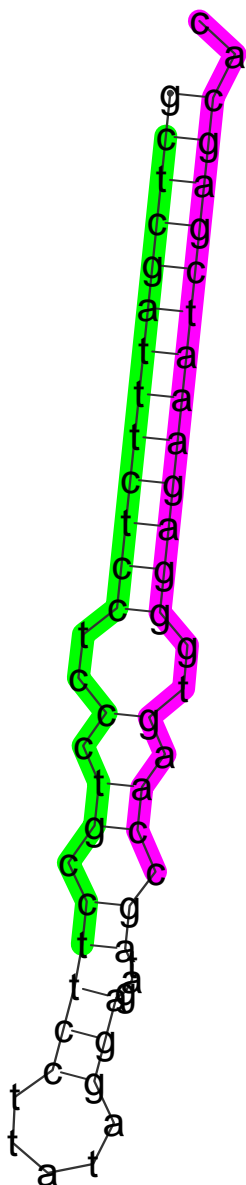

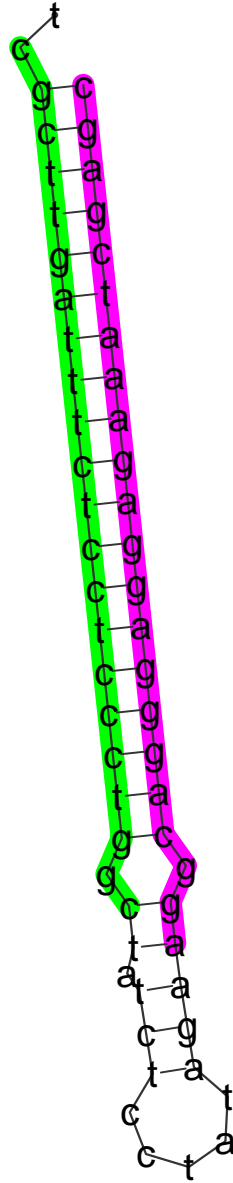

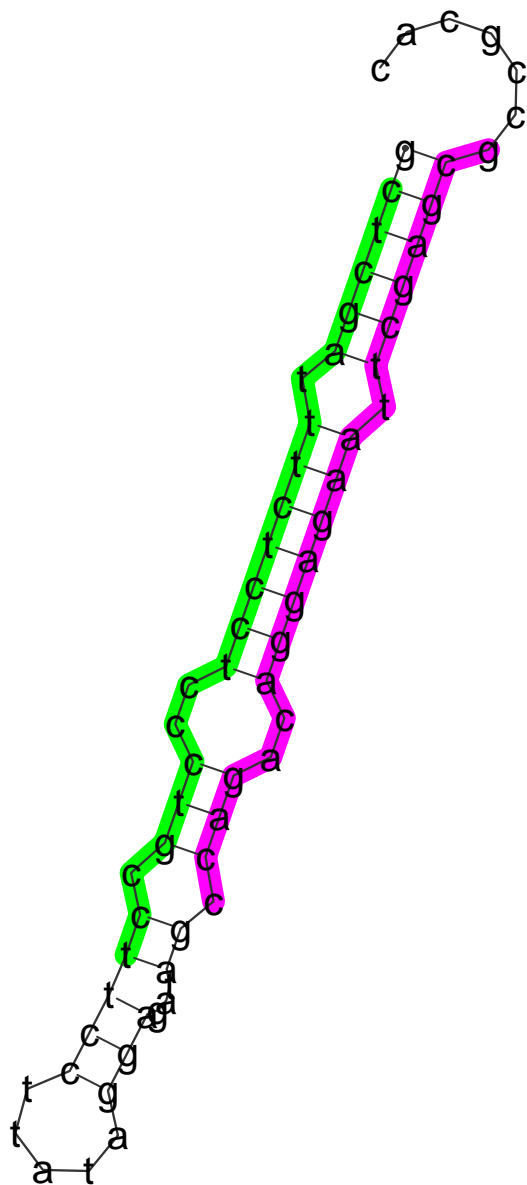

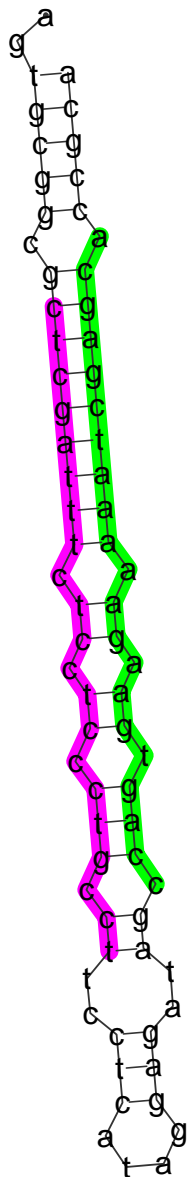

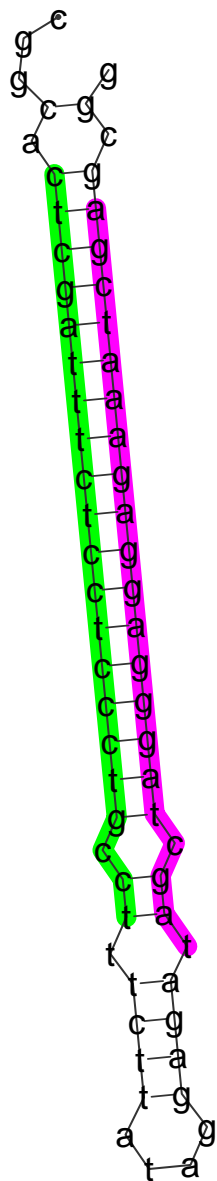

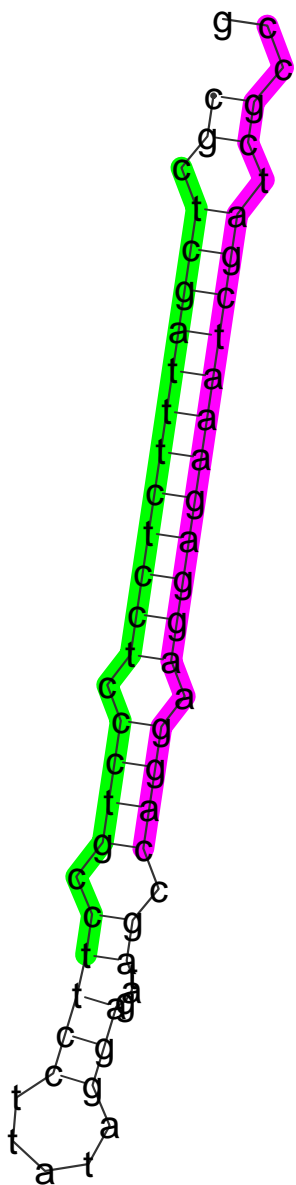

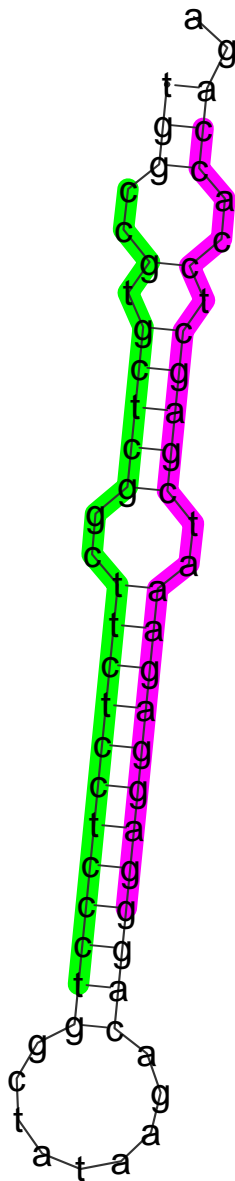

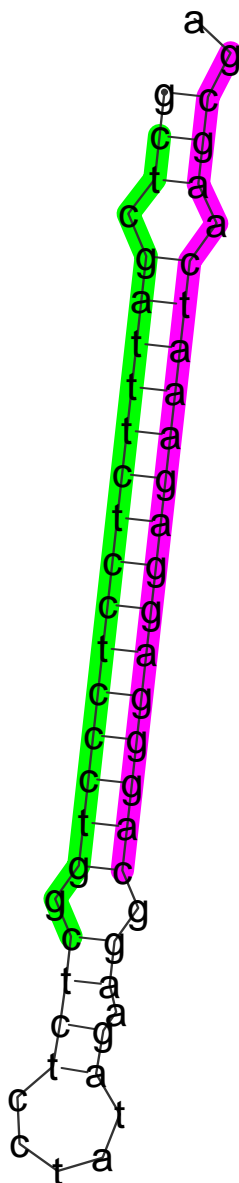

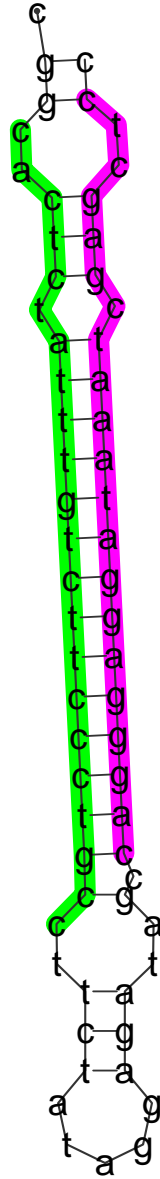

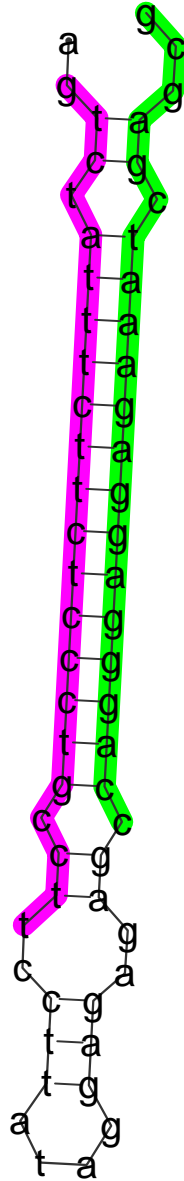

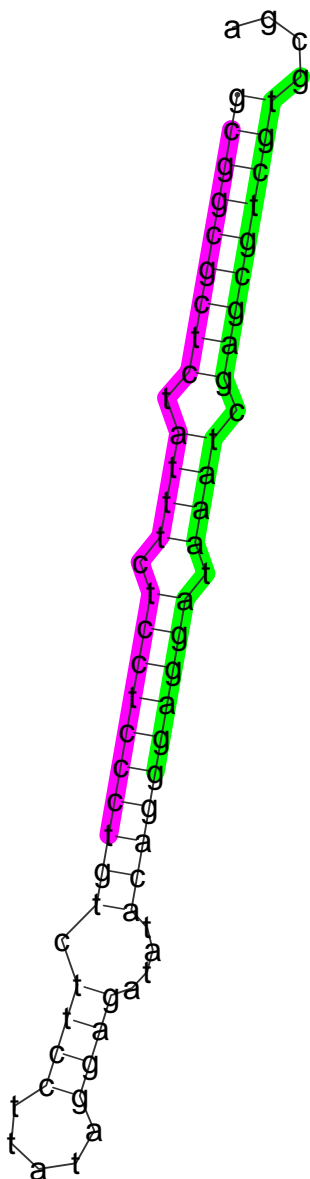

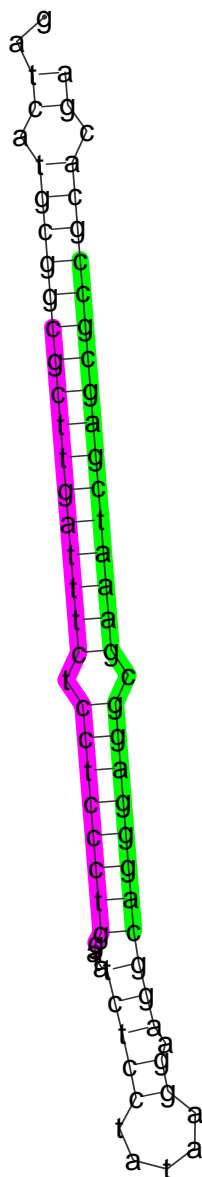

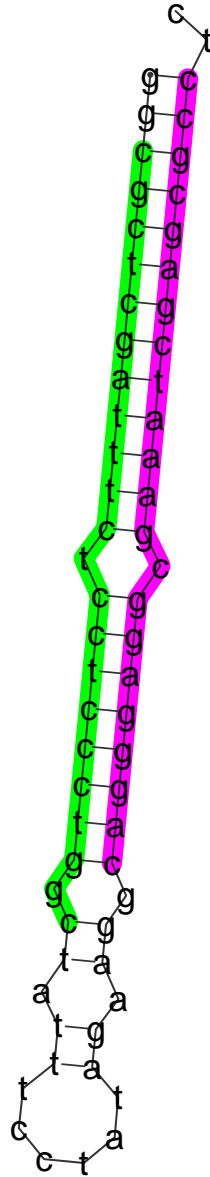

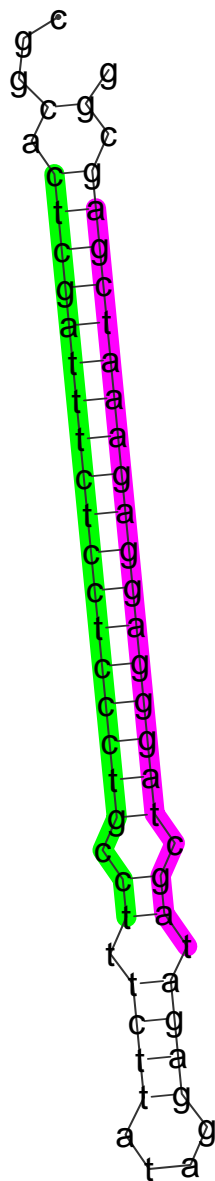

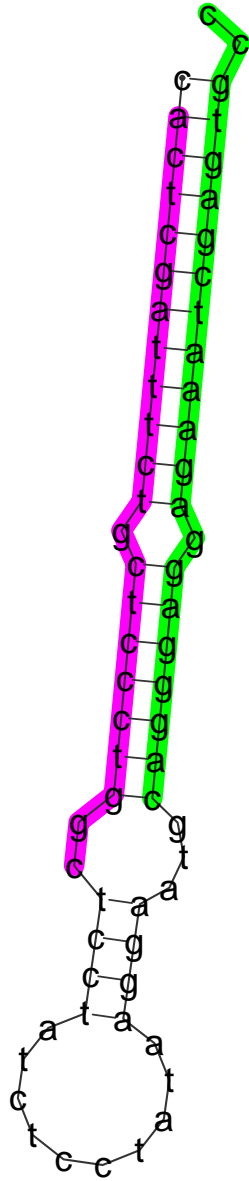

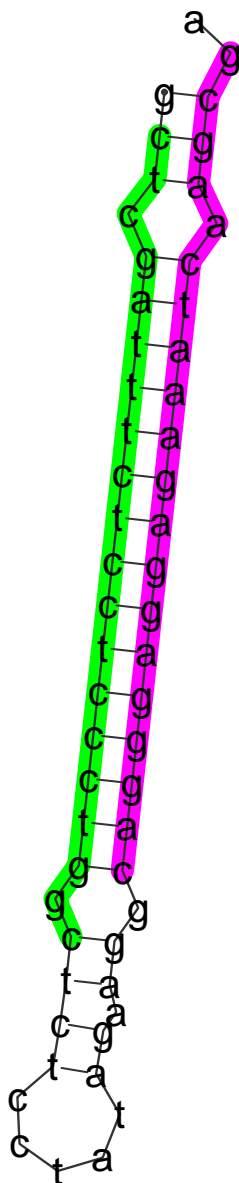

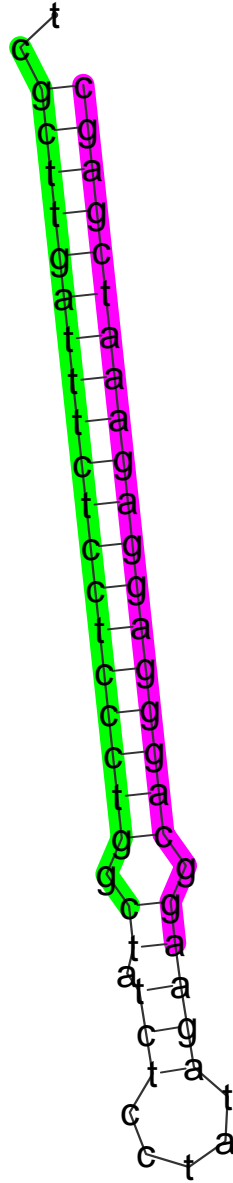

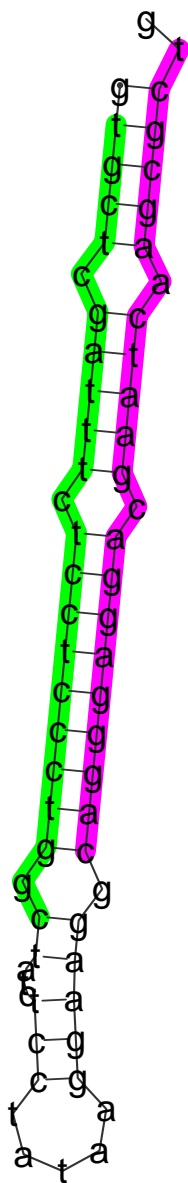

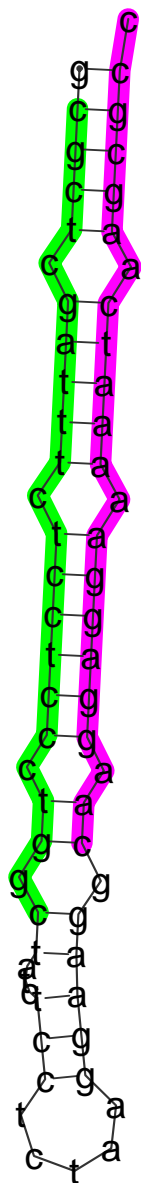

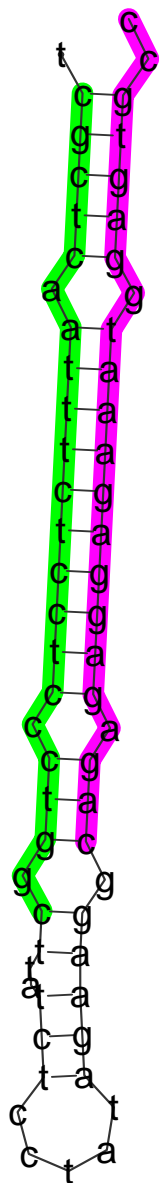

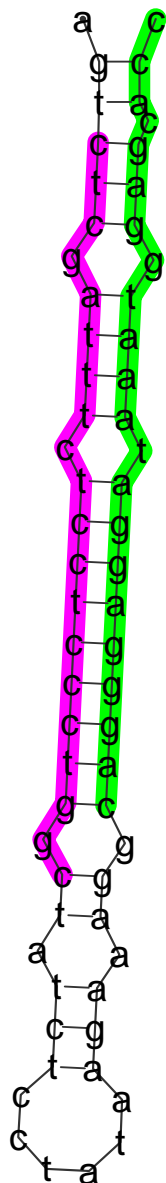

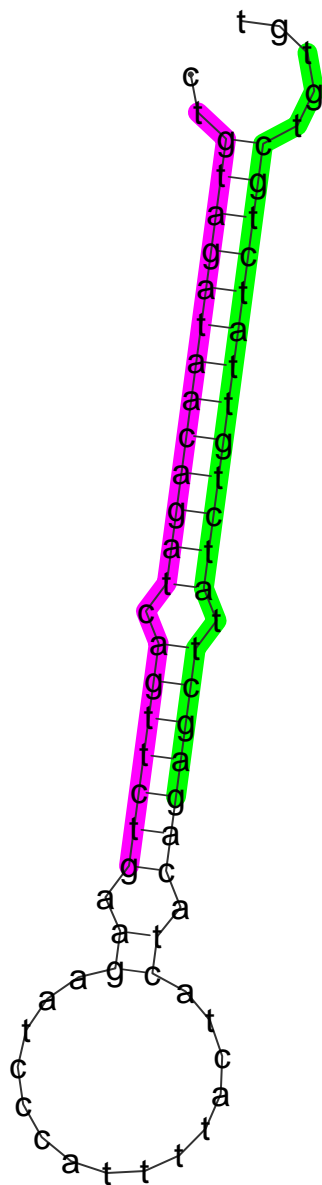

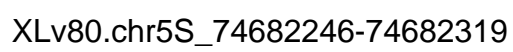

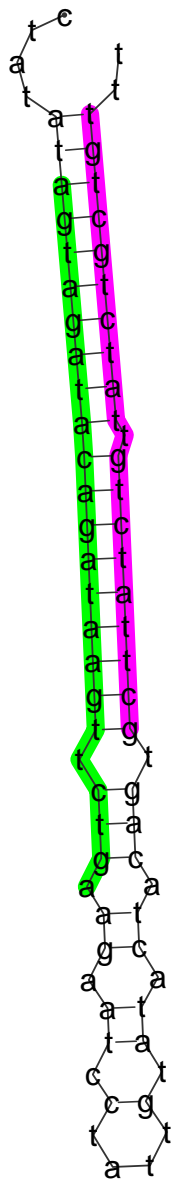

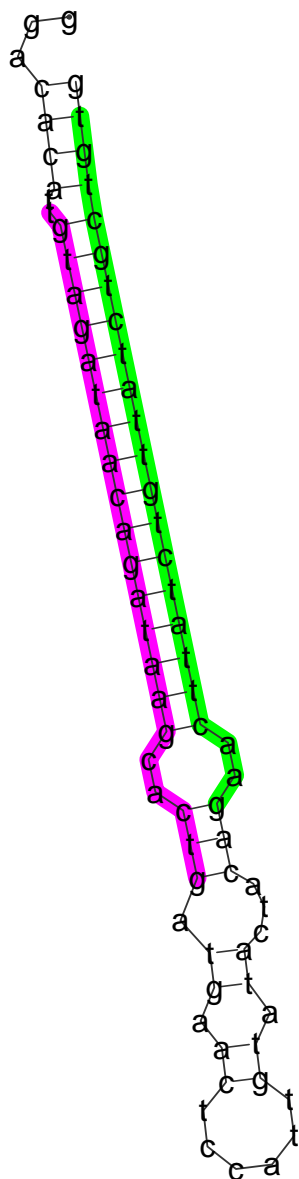

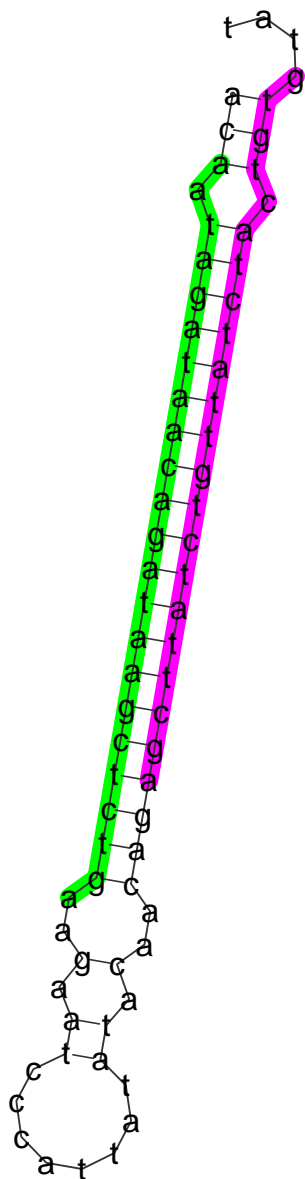

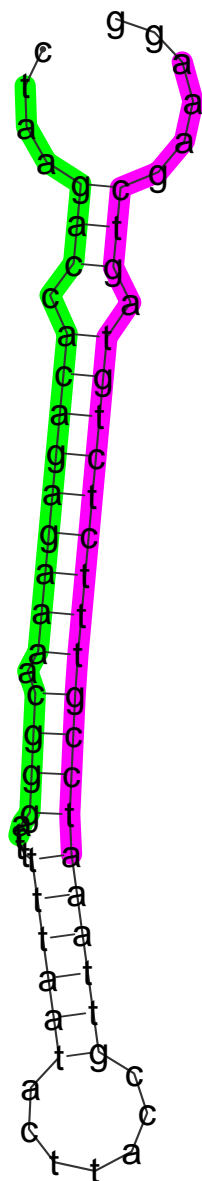

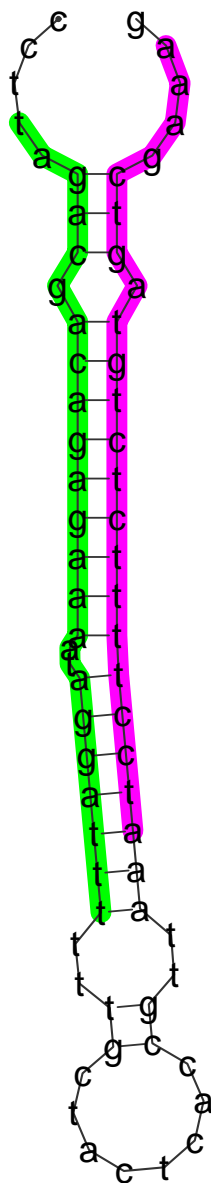

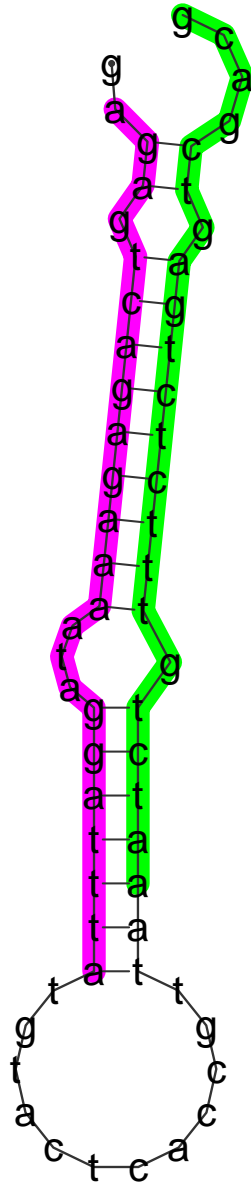

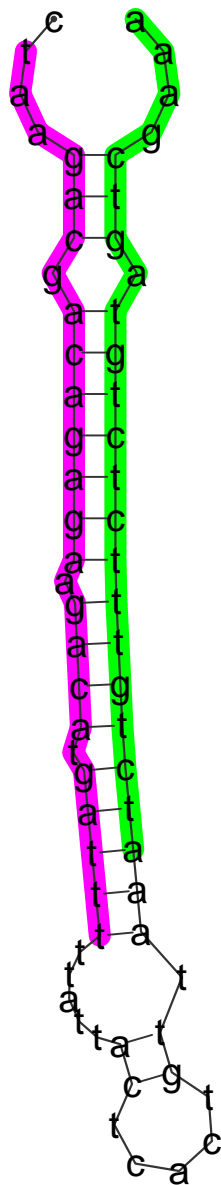

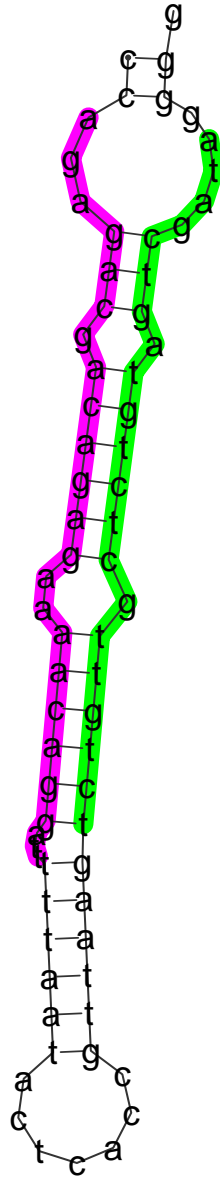

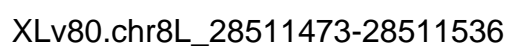

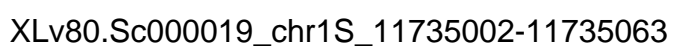

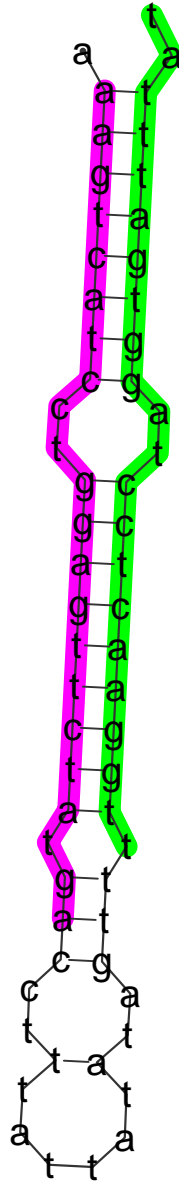

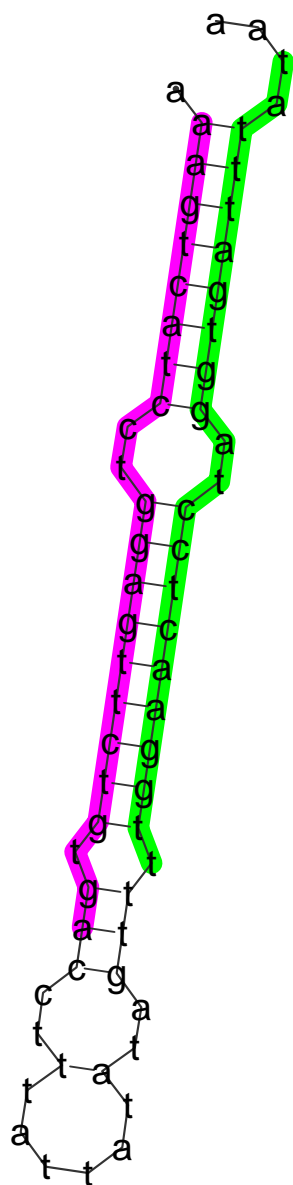

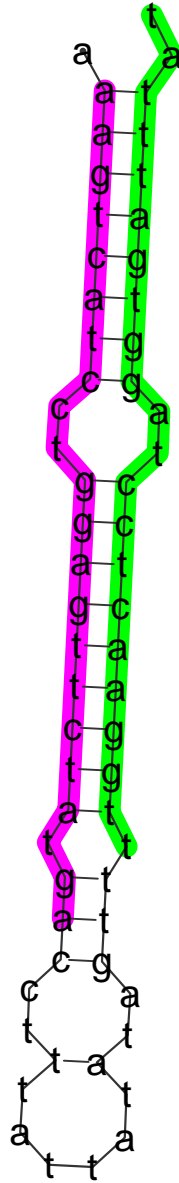

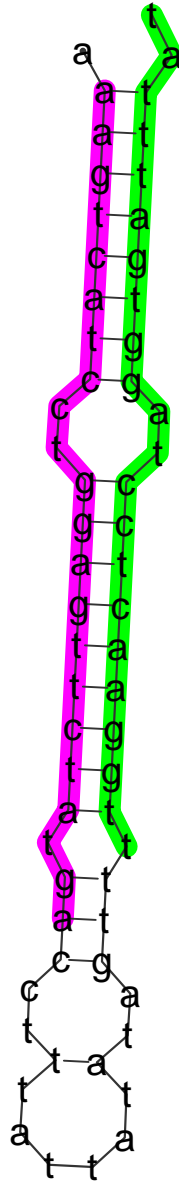

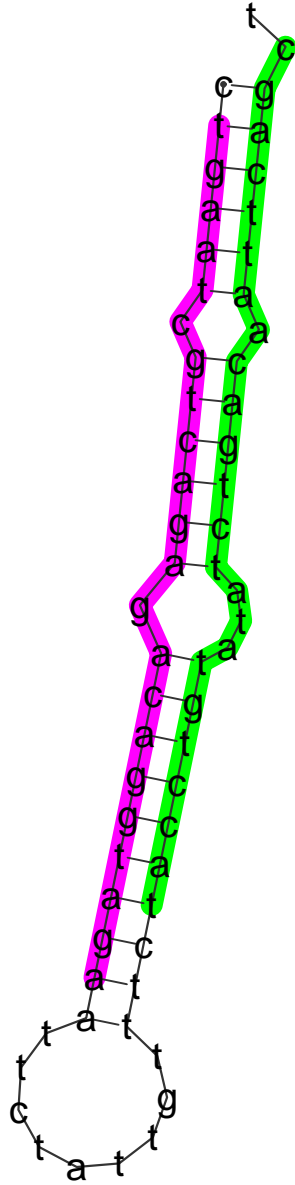

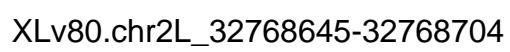

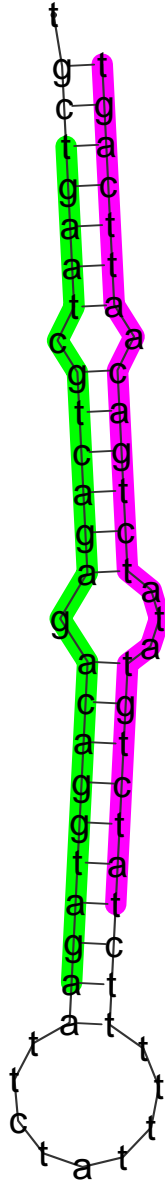

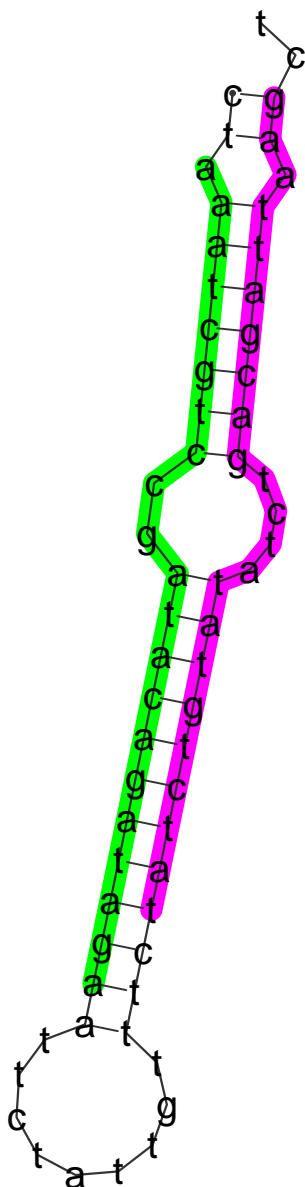

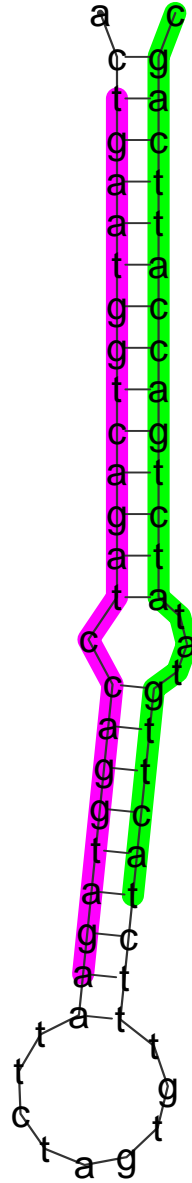

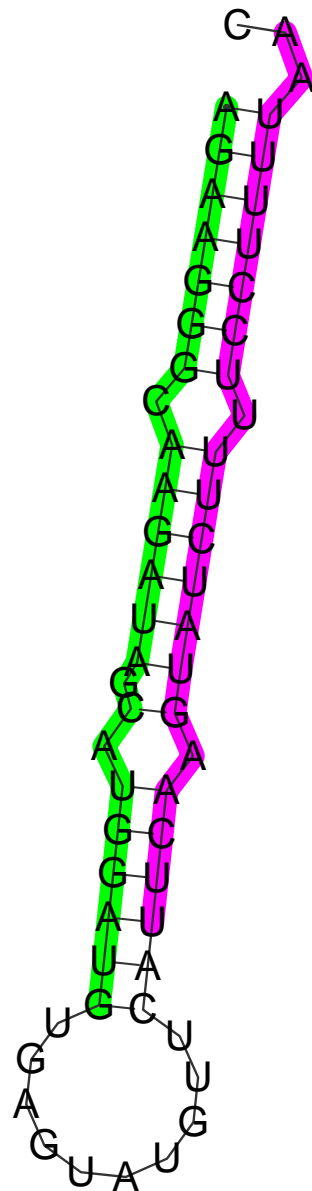

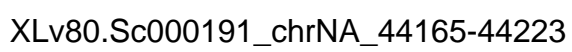

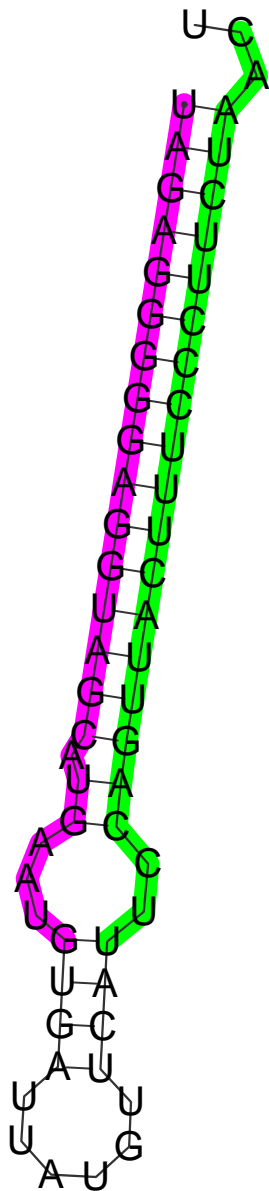

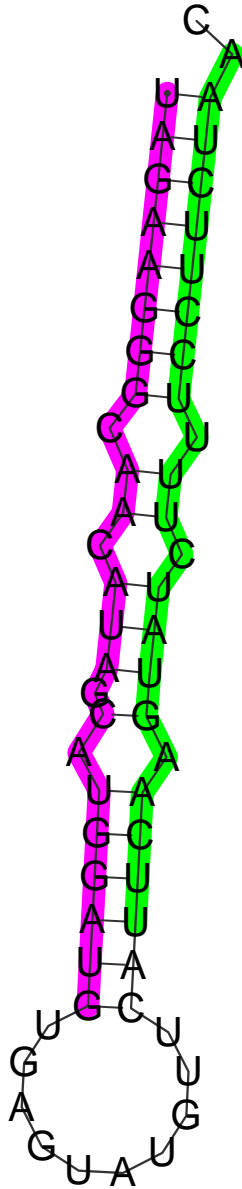

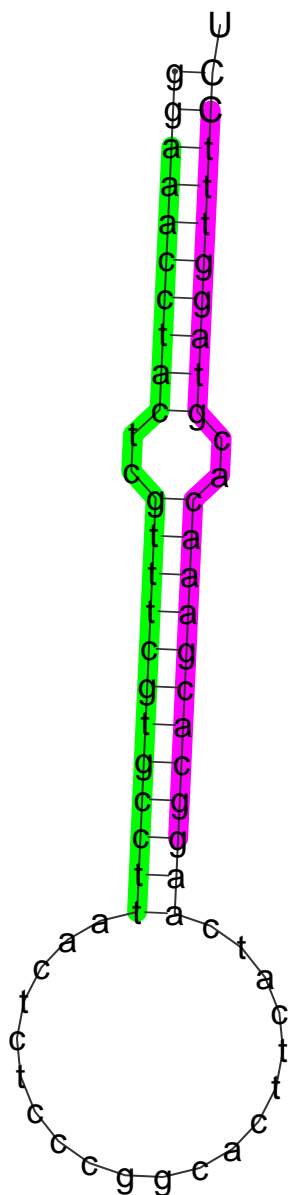

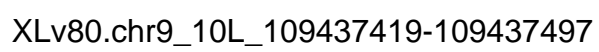

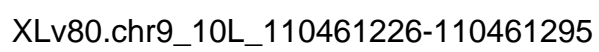

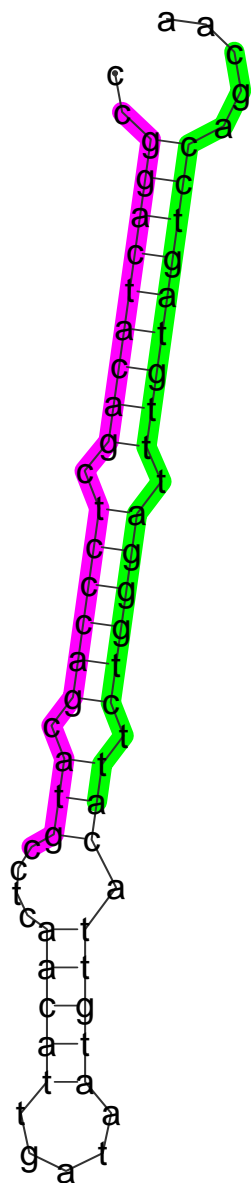

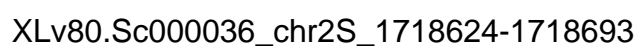

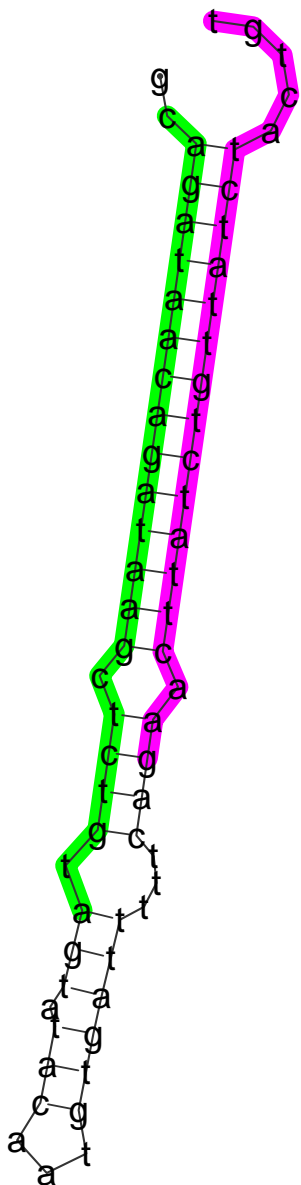

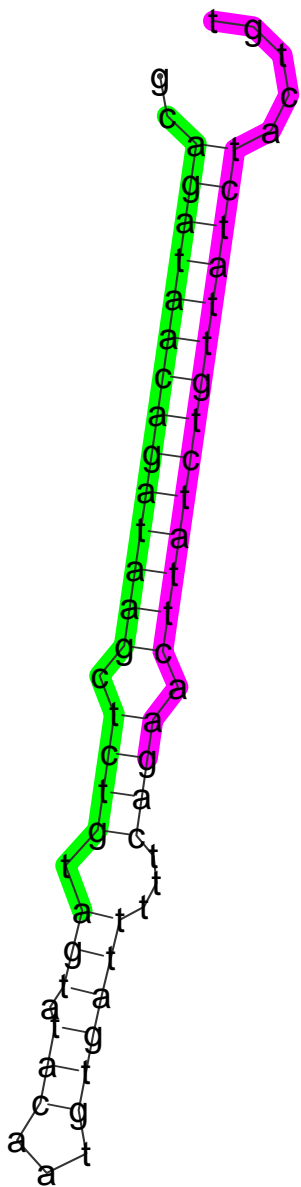

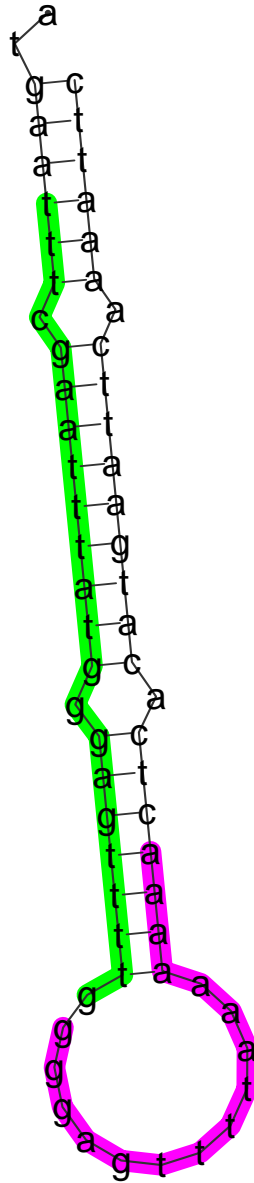

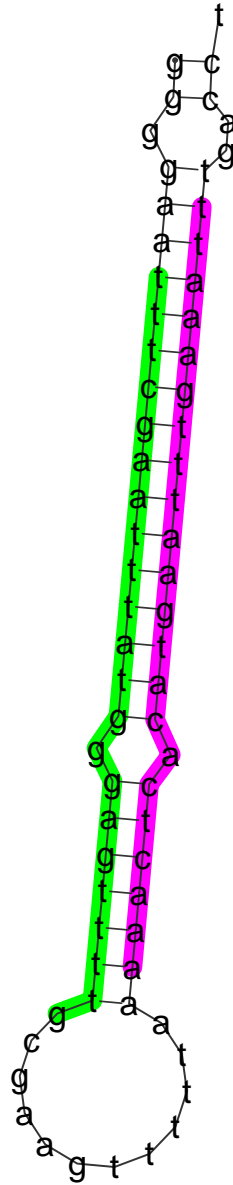

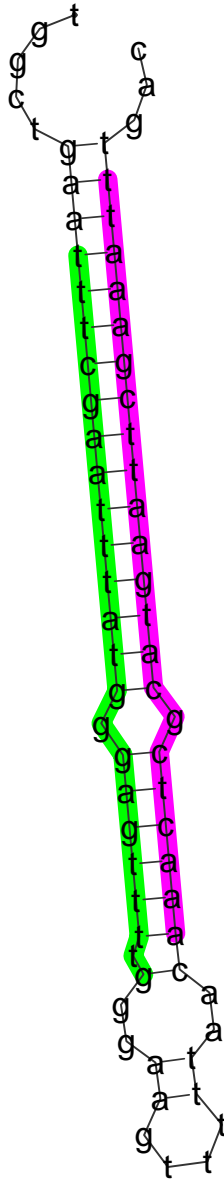

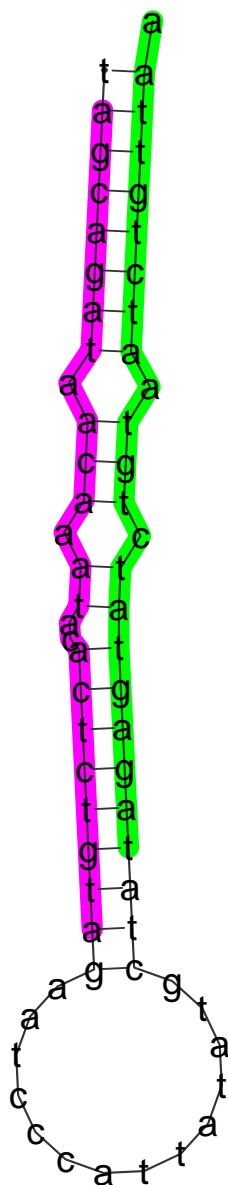

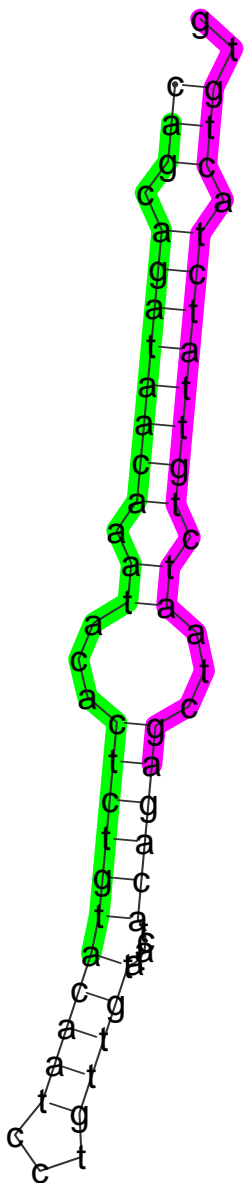

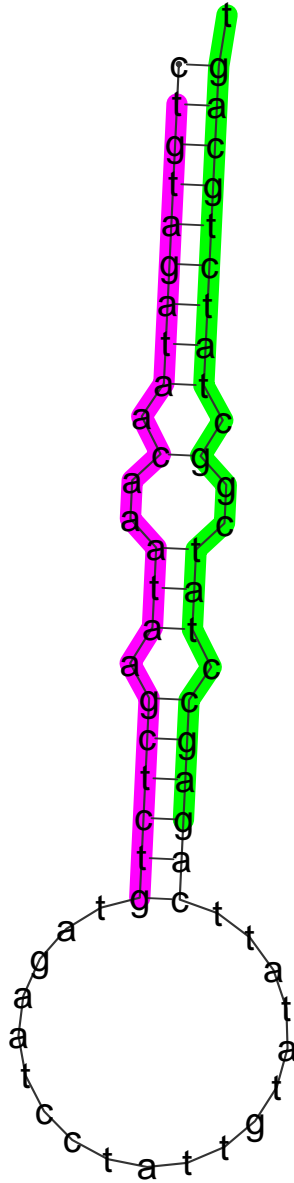

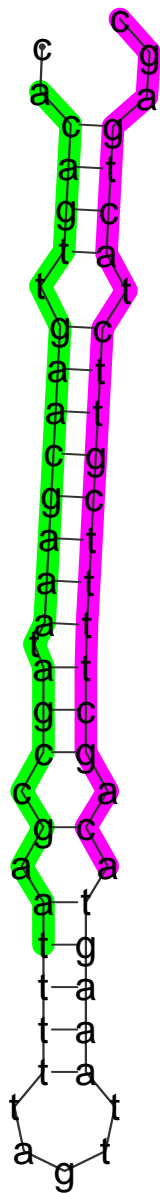

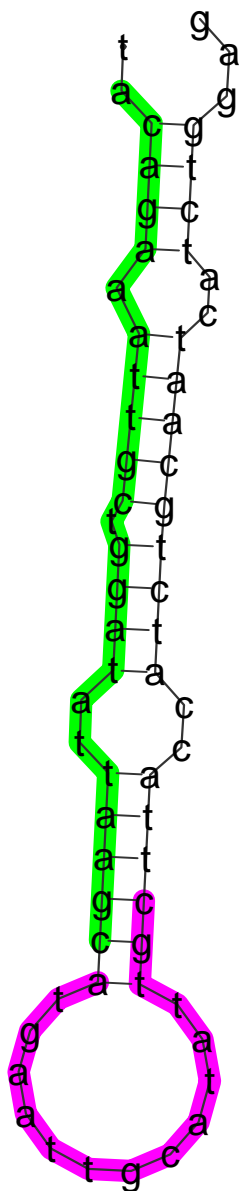

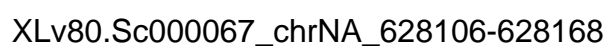

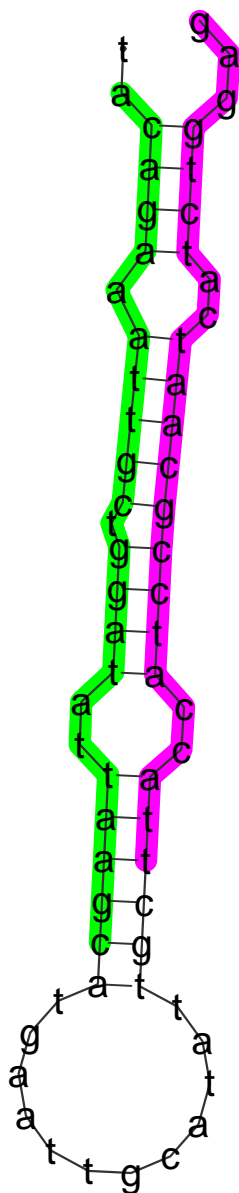

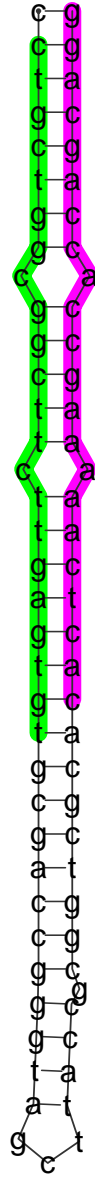

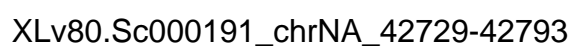

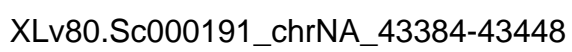

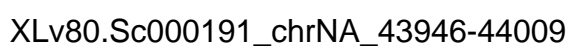

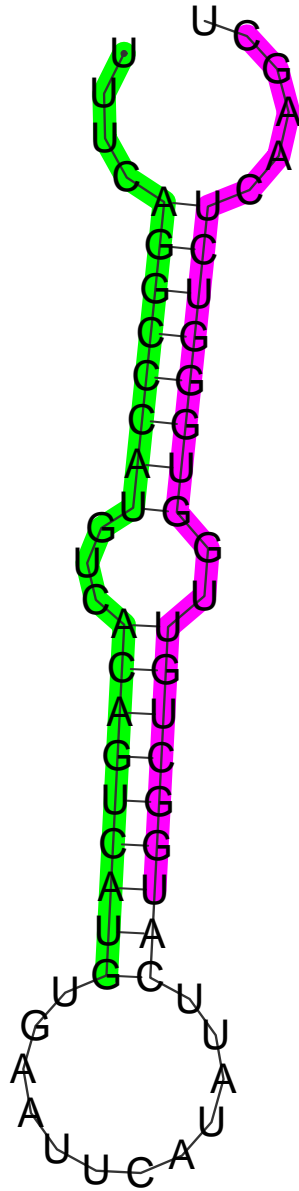

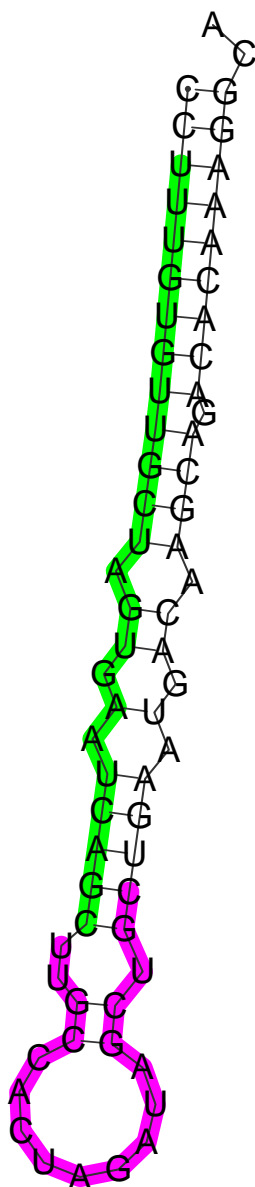

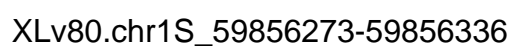



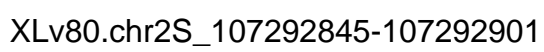

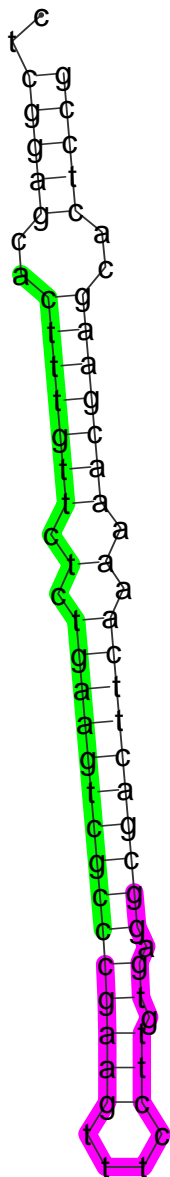

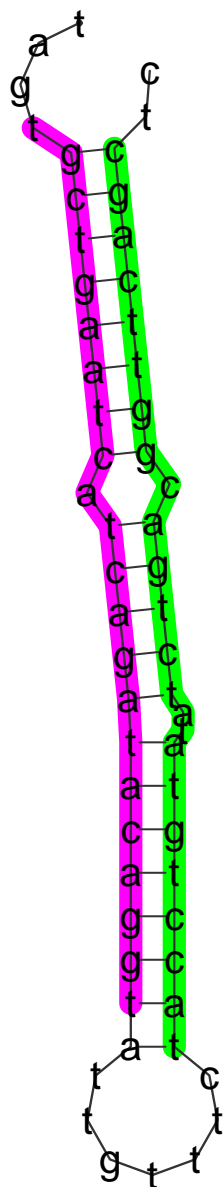

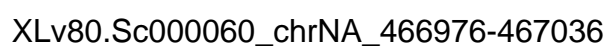

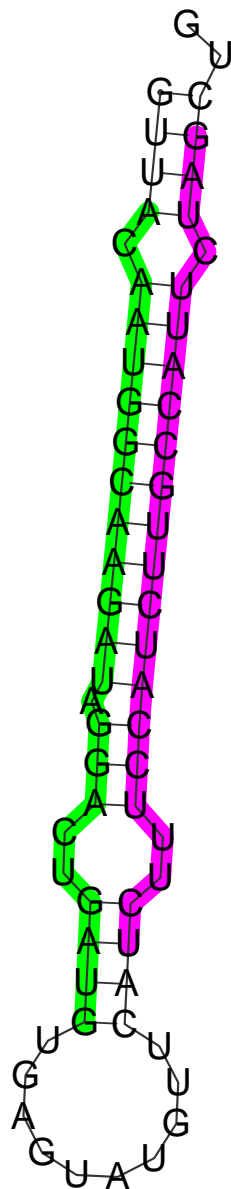

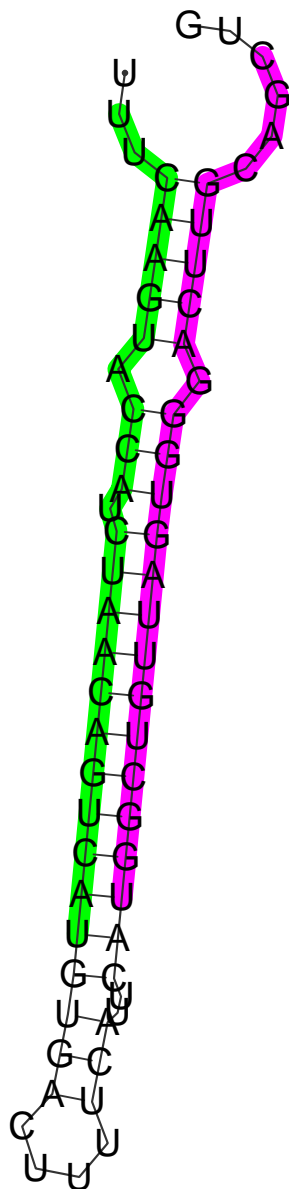

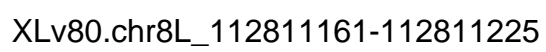

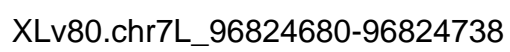

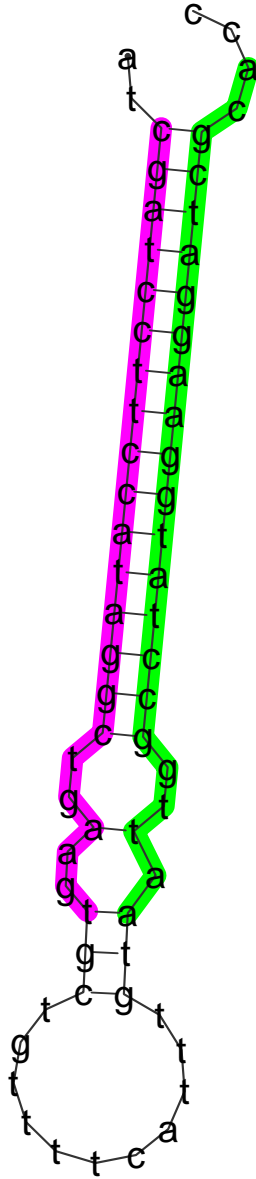

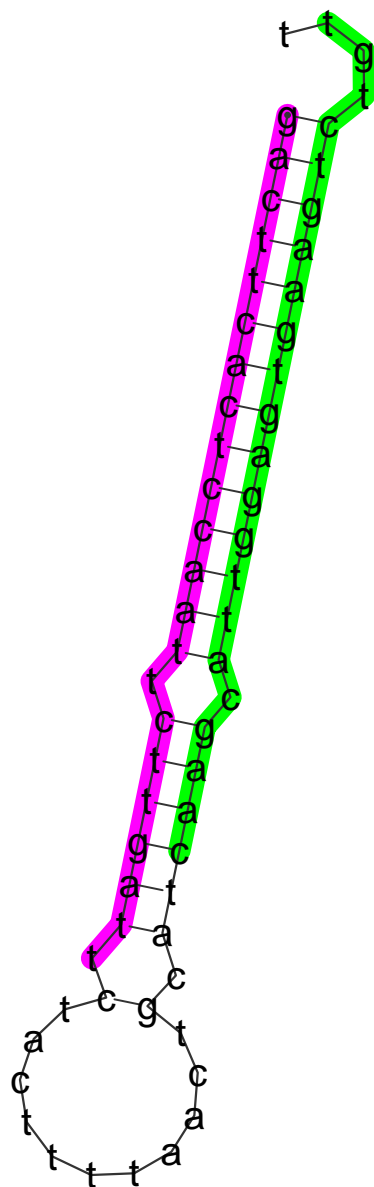

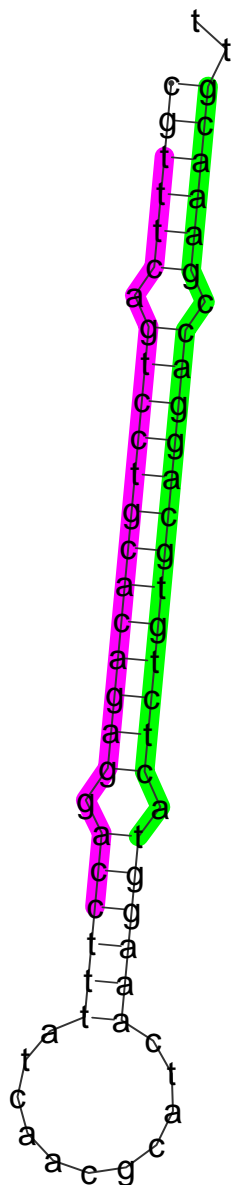

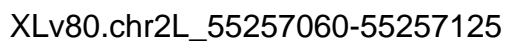

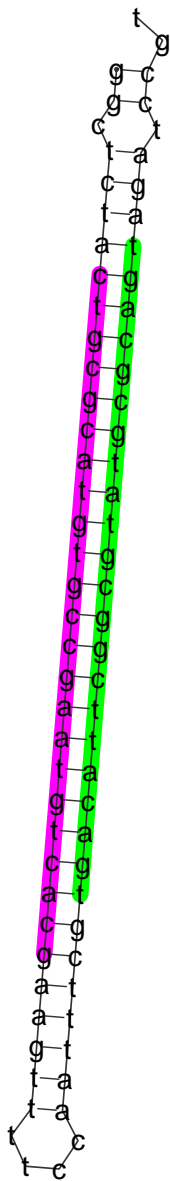

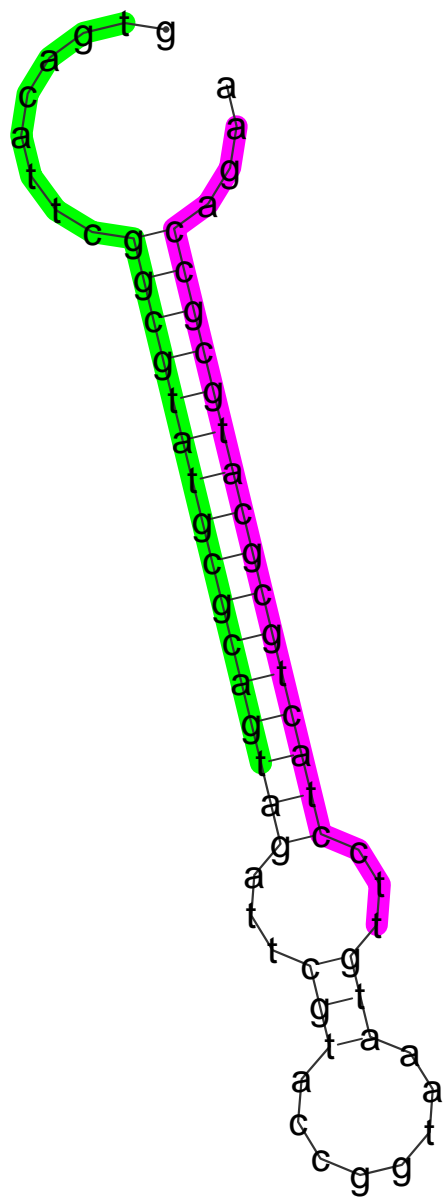

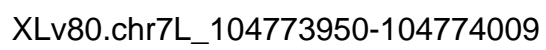

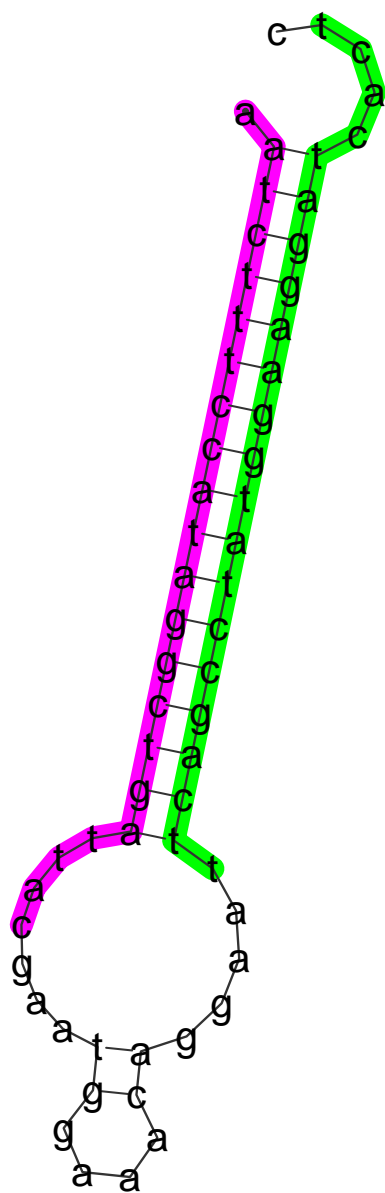

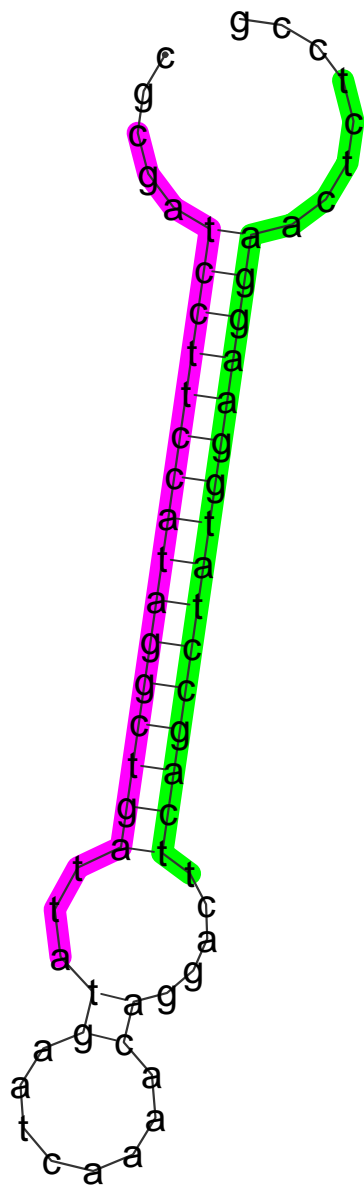

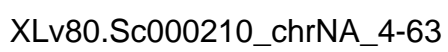

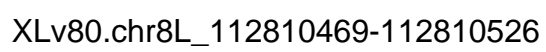

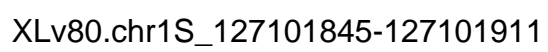

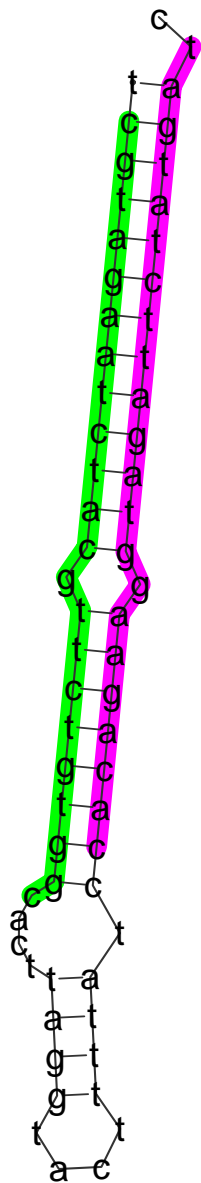

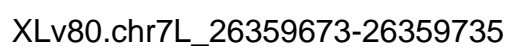

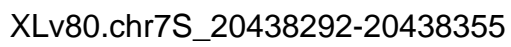

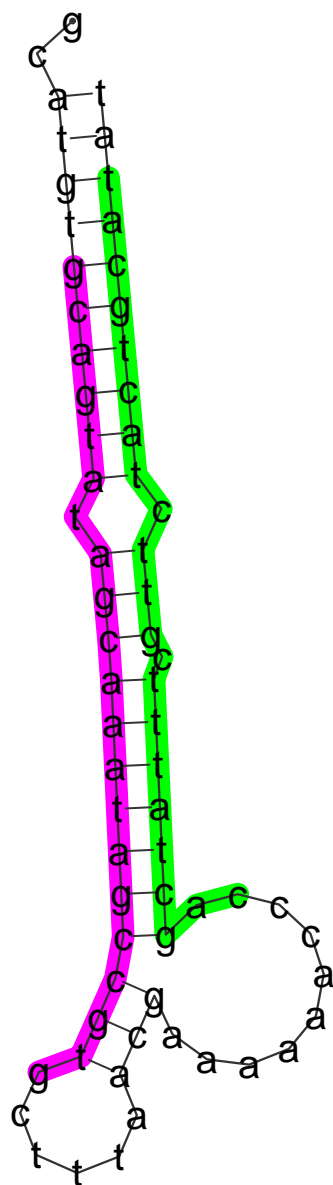

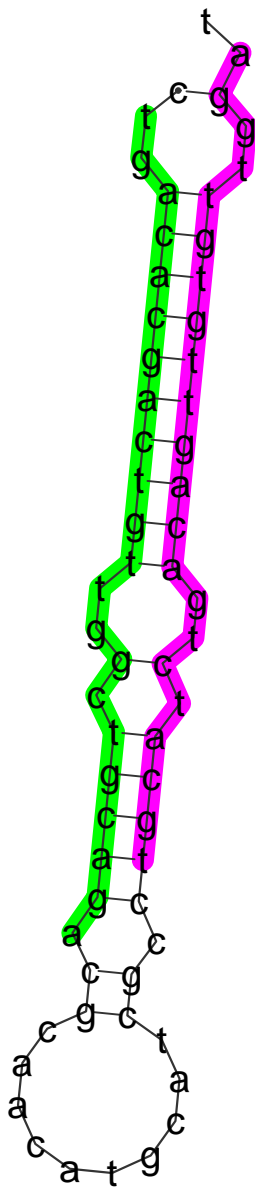

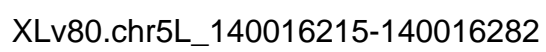

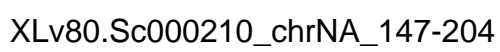

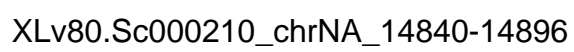

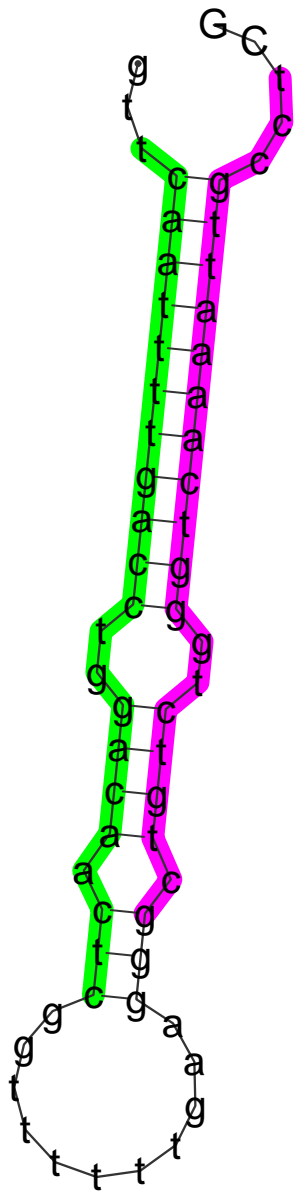

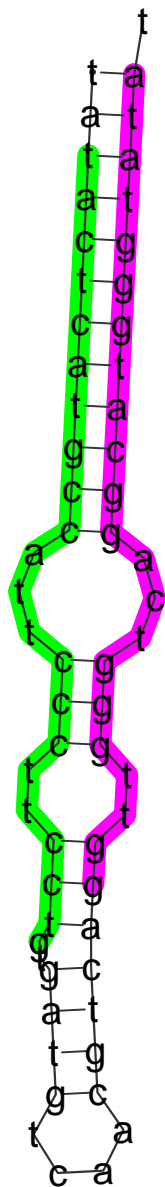

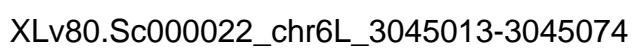

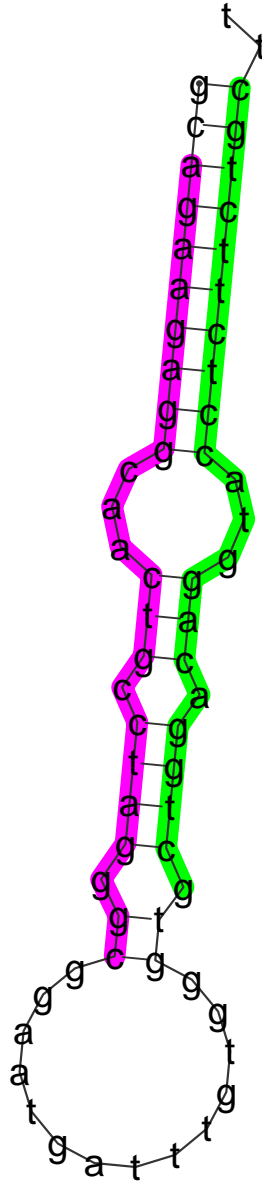

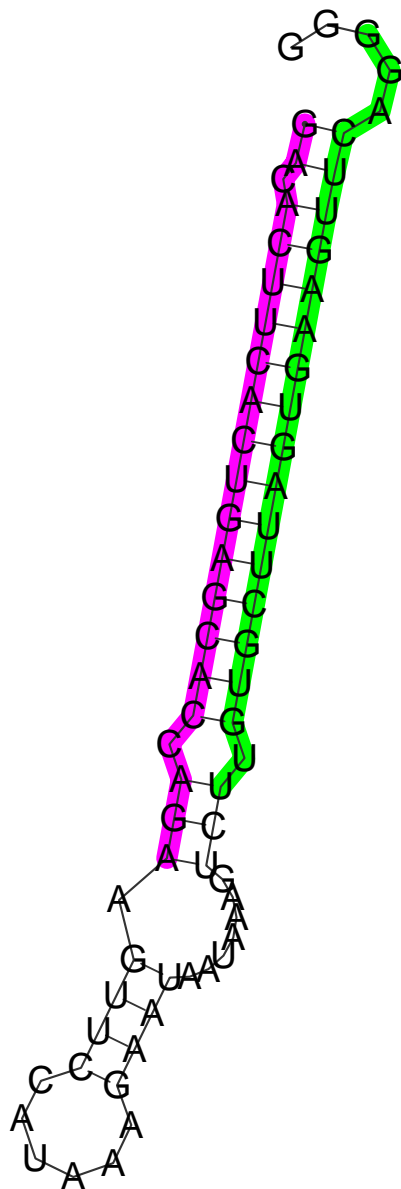

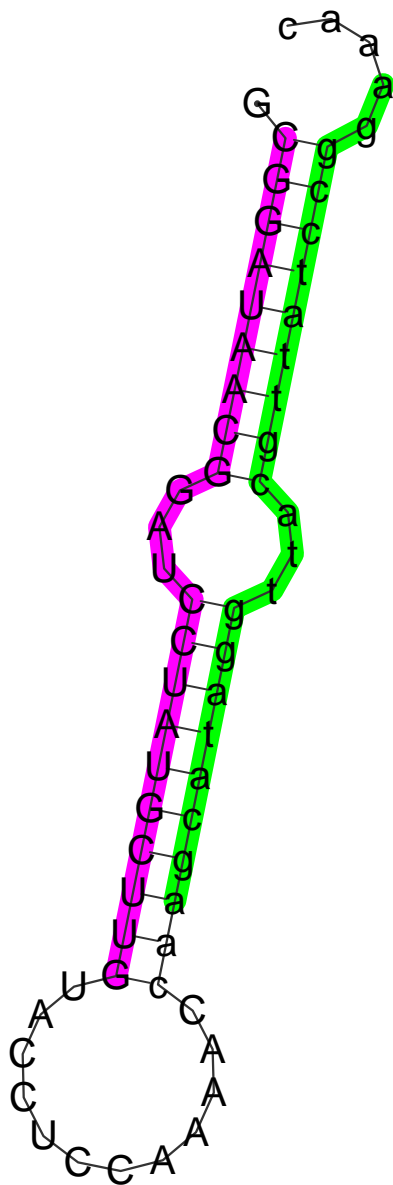

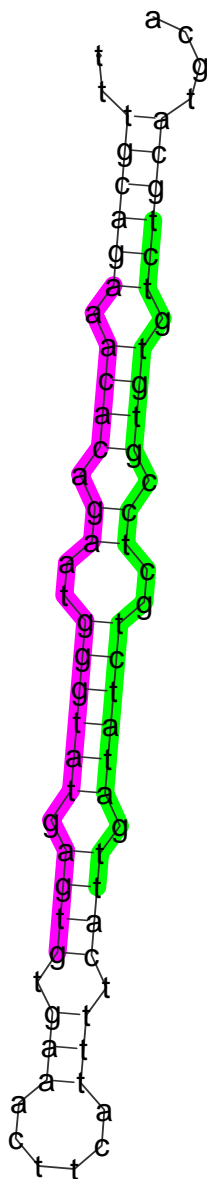

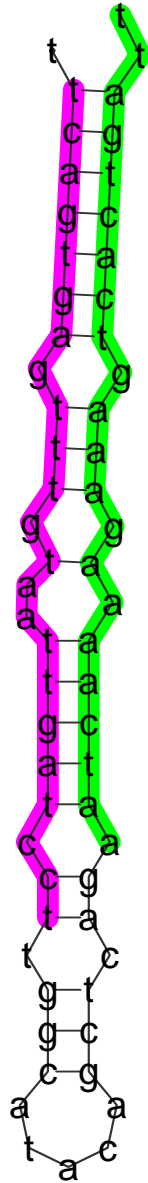

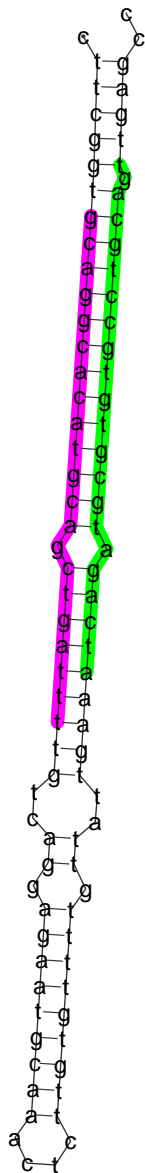

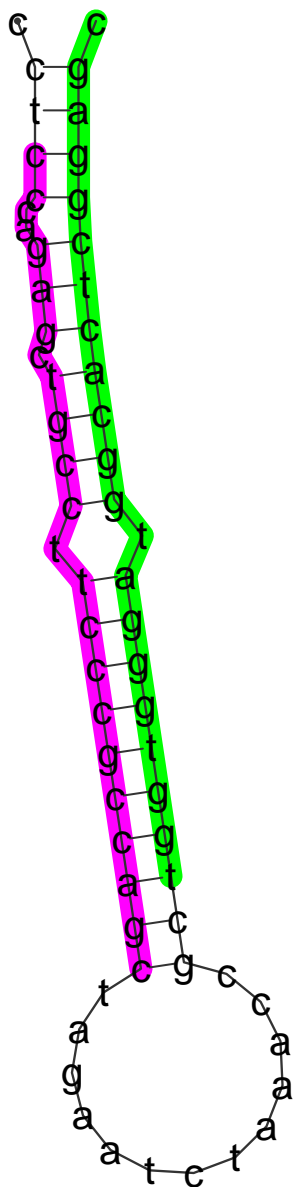

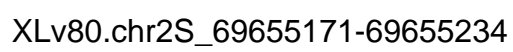

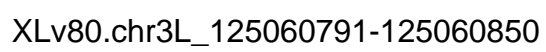

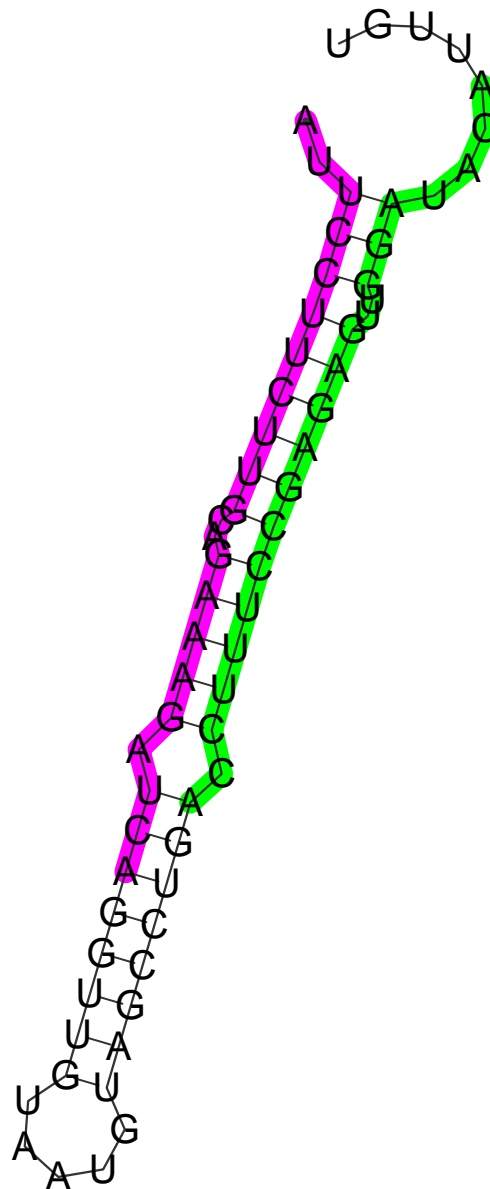

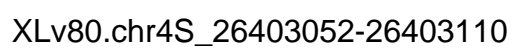

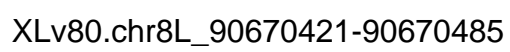

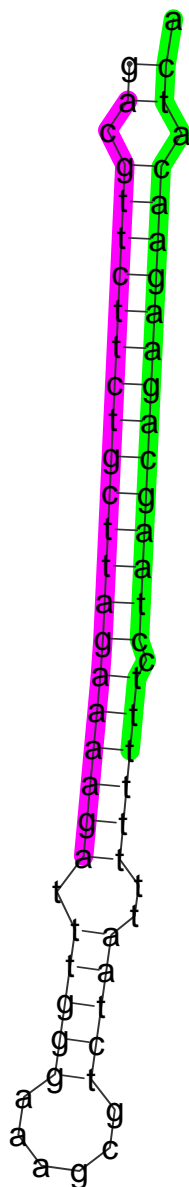

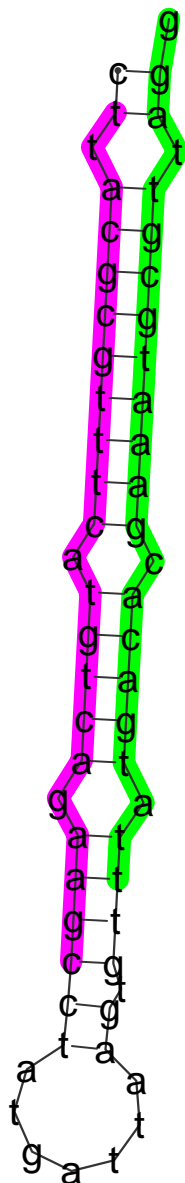

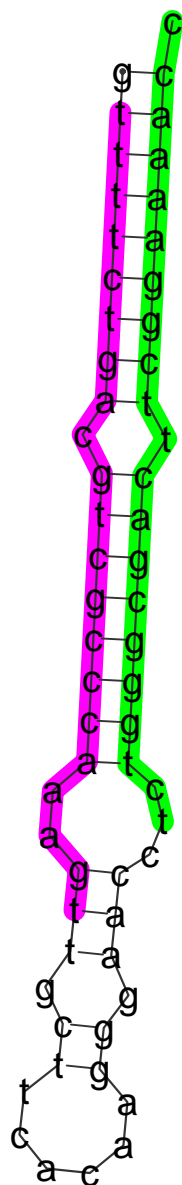

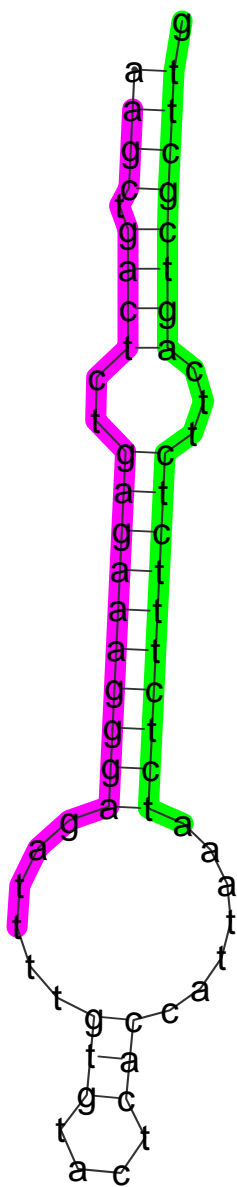

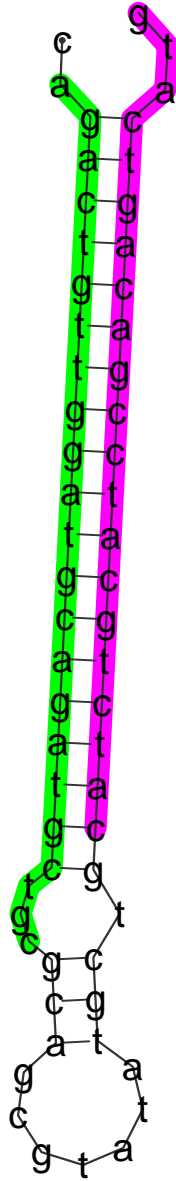

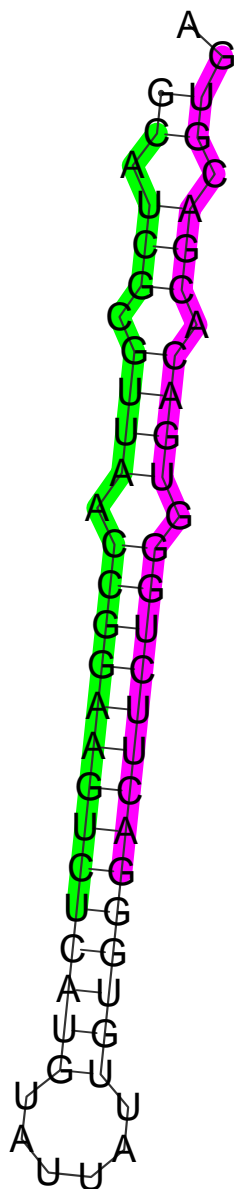

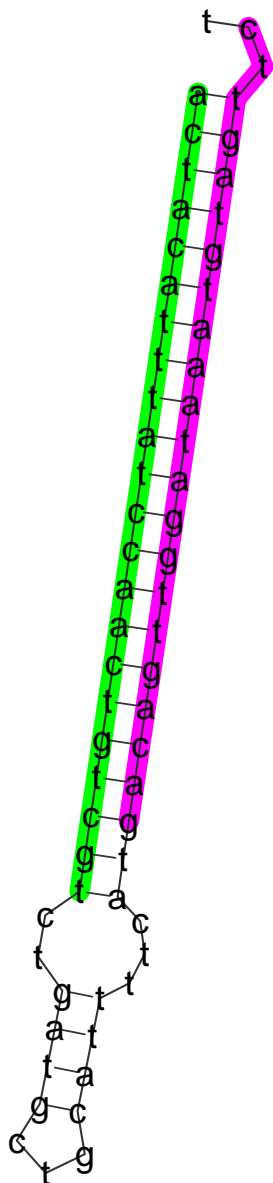

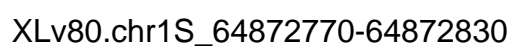

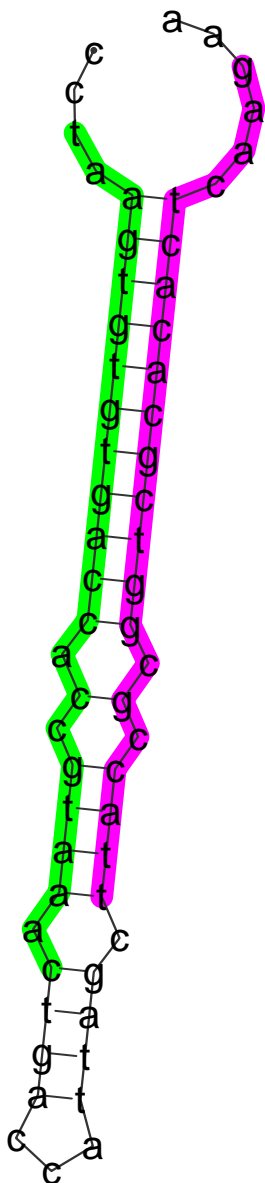

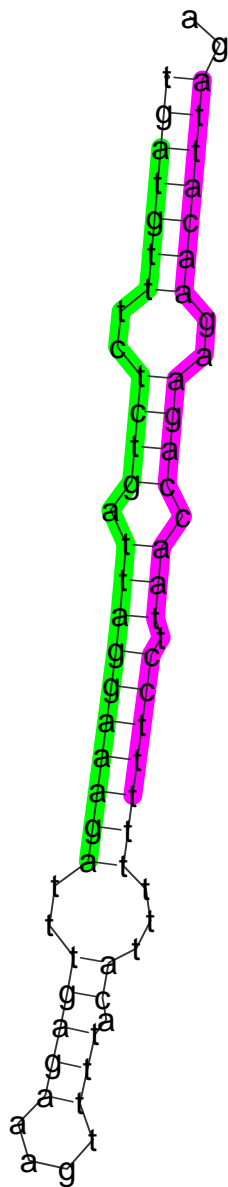

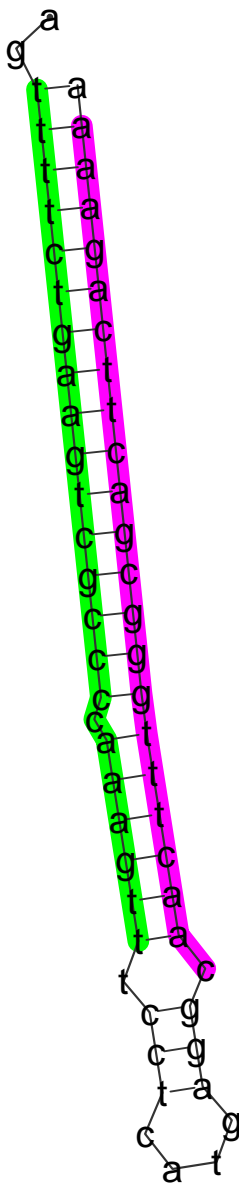

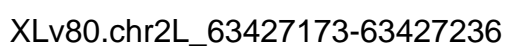

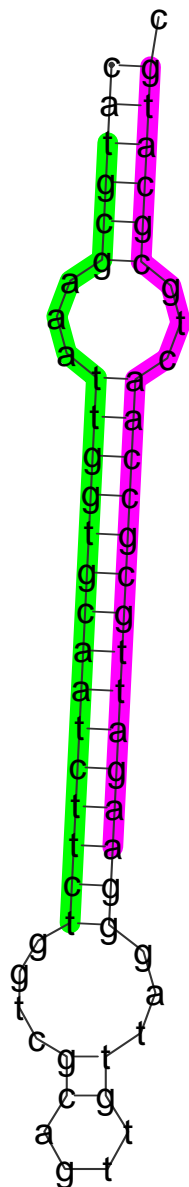

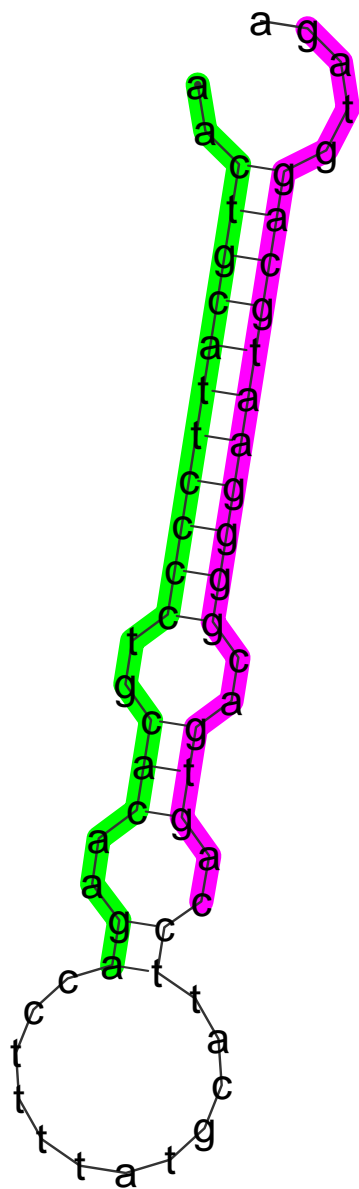

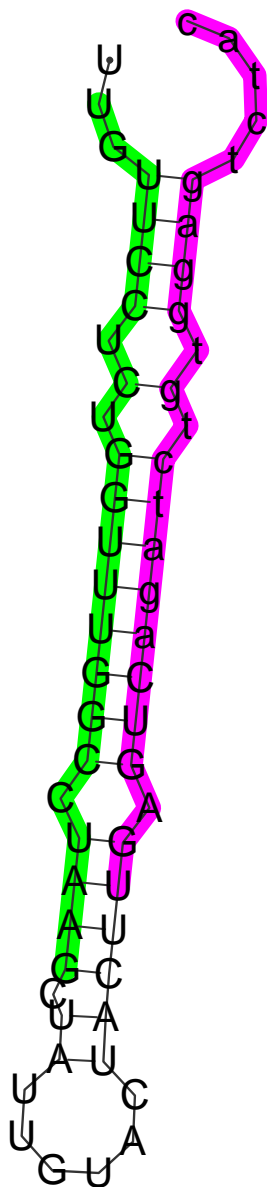

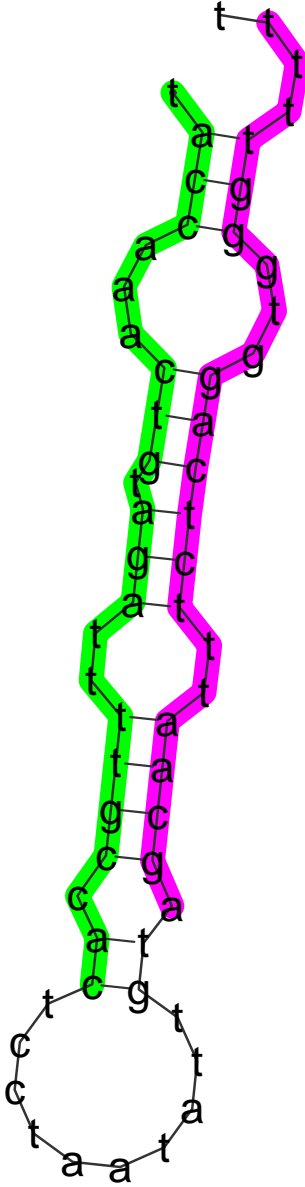

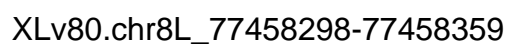

Supplement: Supplementary file 8 — Hairpin structures of all novel miRNAs. (PDF 248 kb) [file 12864_2018_4436_MOESM8_ESM.pdf]
