# Supplementary material for: microRNAs associated with early neural crest development in Xenopus laevis
Source: BMC Genomics. 2018 Jan 18;19:59. doi: 10.1186/s12864-018-4436-0 (PMC5774138; doi:10.1186/s12864-018-4436-0)
Supplement: Supplementary file 9 — qPCR validation of small RNA sequencing. NC miRNAs identified by sRNA sequencing were validated using qPCR. The same RNA was used to make both the sRNA libraries and for qPCR. (A) Abundance plots of miRNAs; miR-219, miR-196a, miR-302 and nov-12a-1 following sRNA sequencing on blastula and ectoderm animal cap tissue and animal caps induced to form NC and neural tissue (B) qPCR validation of the miRNAs identified from the sRNA sequencing in the same order as A. One way ANOVA with Tukey post-test statistical analyses were performed on the results of each qPCR. For significance, we considered P > 0.01*; P > 0.001**; P > 0.0001*** and P > 0.0001****. (DOCX 186 kb) [file 12864_2018_4436_MOESM9_ESM.docx]

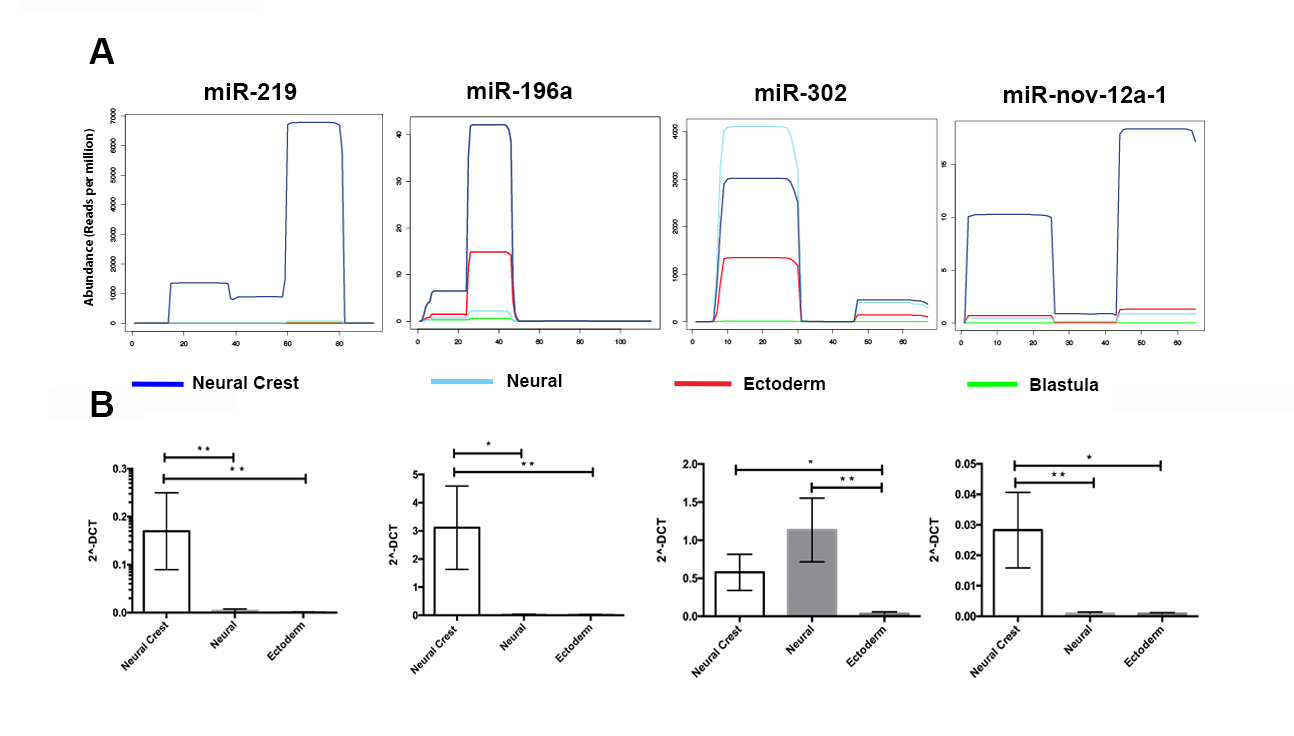


**Additional file 9: FIgure S6: qPCR validation of small RNA sequencing.** NC miRNAs identified by sRNA sequencing were validated using qPCR. The same RNA was used to make both the sRNA libraries and for qPCR. (**A**) Abundance plots of miRNAs; miR-219, miR-196a, miR-302 and nov-12a-1 following sRNA sequencing on blastula and ectoderm animal cap tissue and animal caps induced to form NC and neural tissue (**B**) qPCR validation of the miRNAs identified from the sRNA sequencing in the same order as **A**. One way ANOVA with Tukey post-test statistical analyses were performed on the results of each qPCR. For significance, we considered P>0.01*; P>0.001**; P>0.0001*** and P>0.0001****.
